# Supplementary material for: The Molecular Epidemiology of Hepatitis B Virus and Its Resistance-Associated Mutations in the Polymerase Gene in the Americas
Source: Microorganisms. 2025 Aug 16;13(8):1913. doi: 10.3390/microorganisms13081913 (PMC12388563; doi:10.3390/microorganisms13081913)
Supplement: Supplementary file 1 [file microorganisms-13-01913-s001.zip › Figure S1 - Report Subgenotyping (Confirming Reported).pdf]

Phylogenetic Analysis of Sequences

Confirmatory Trees

GENOTYPE AND SUBTYPE REPORTED

DOMINICAN REPUBLIC

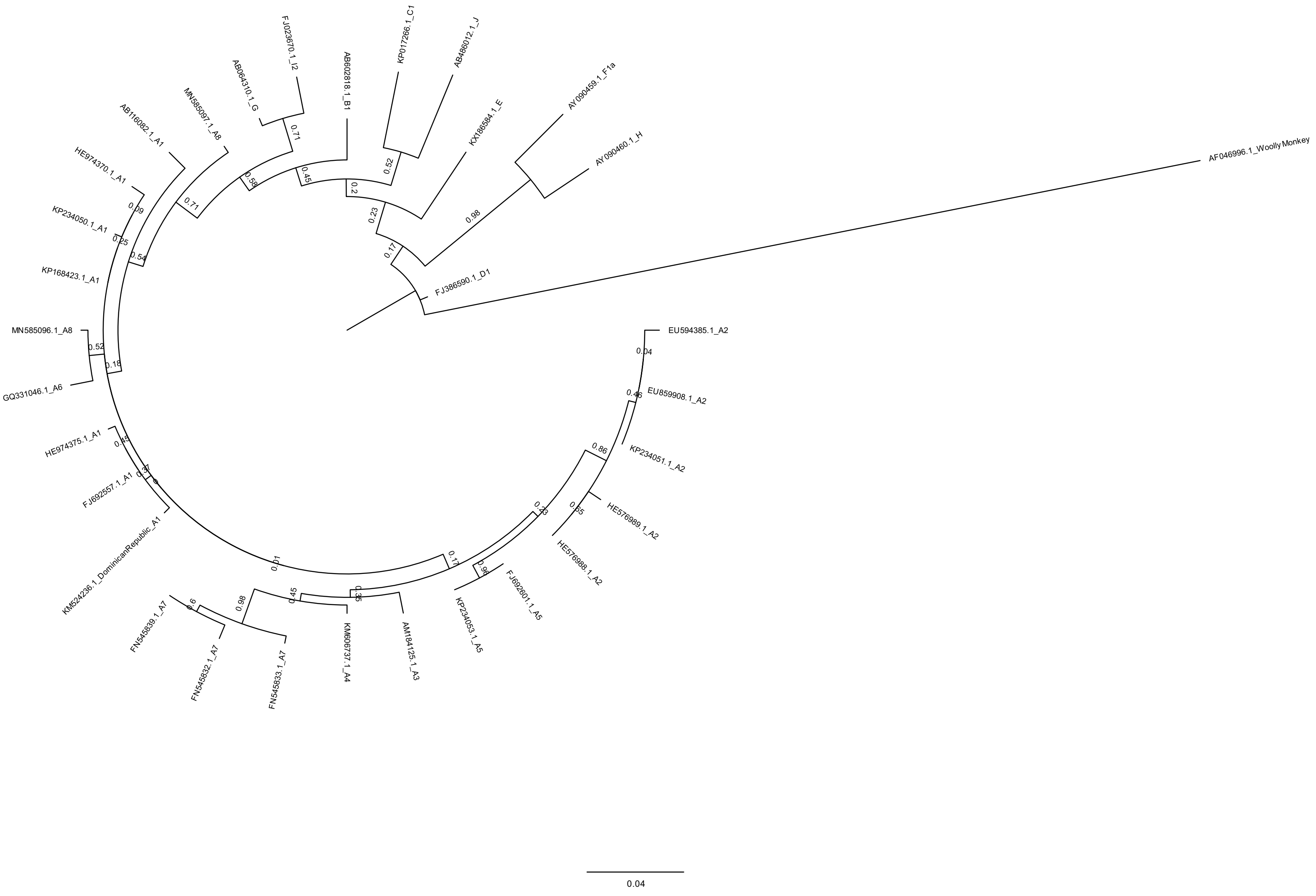

Tree 1. The evolutionary history was inferred by using the Maximum Likelihood method and Tamura-Nei model. The percentage of replicate trees in which the associated taxa clustered together in the bootstrap test (1000 replicates) are shown next to the branches. Initial tree(s) for the heuristic search were obtained automatically by applying Neighbor-Join and BioNJ algorithms to a matrix of pairwise distances estimated using the Tamura-Nei model, and then selecting the topology with superior log likelihood value. A discrete Gamma distribution was used to model evolutionary rate differences among sites (5 categories (+G, parameter = 0.1582)). The tree is drawn to scale, with branch lengths measured in the number of substitutions per site. The analysis involved 32 nucleotide sequences, of which 31 were used as marker sequences to determine the genotype of 1 sequence. All positions containing gaps and missing data were eliminated. There was a total of 402 positions in the final dataset. Evolutionary analyses were conducted in MEGA X.

ID	GENOTYPE	SUBTYPE	COUNTRY	TREE	ALIGNMENT[1]	BASE PAIRS
KM524236	A	A1	Dominican Republic	1	417-835	413

[1]Alignment to complete genome reference sequence VHB NC_003977.2

EL SALVADOR

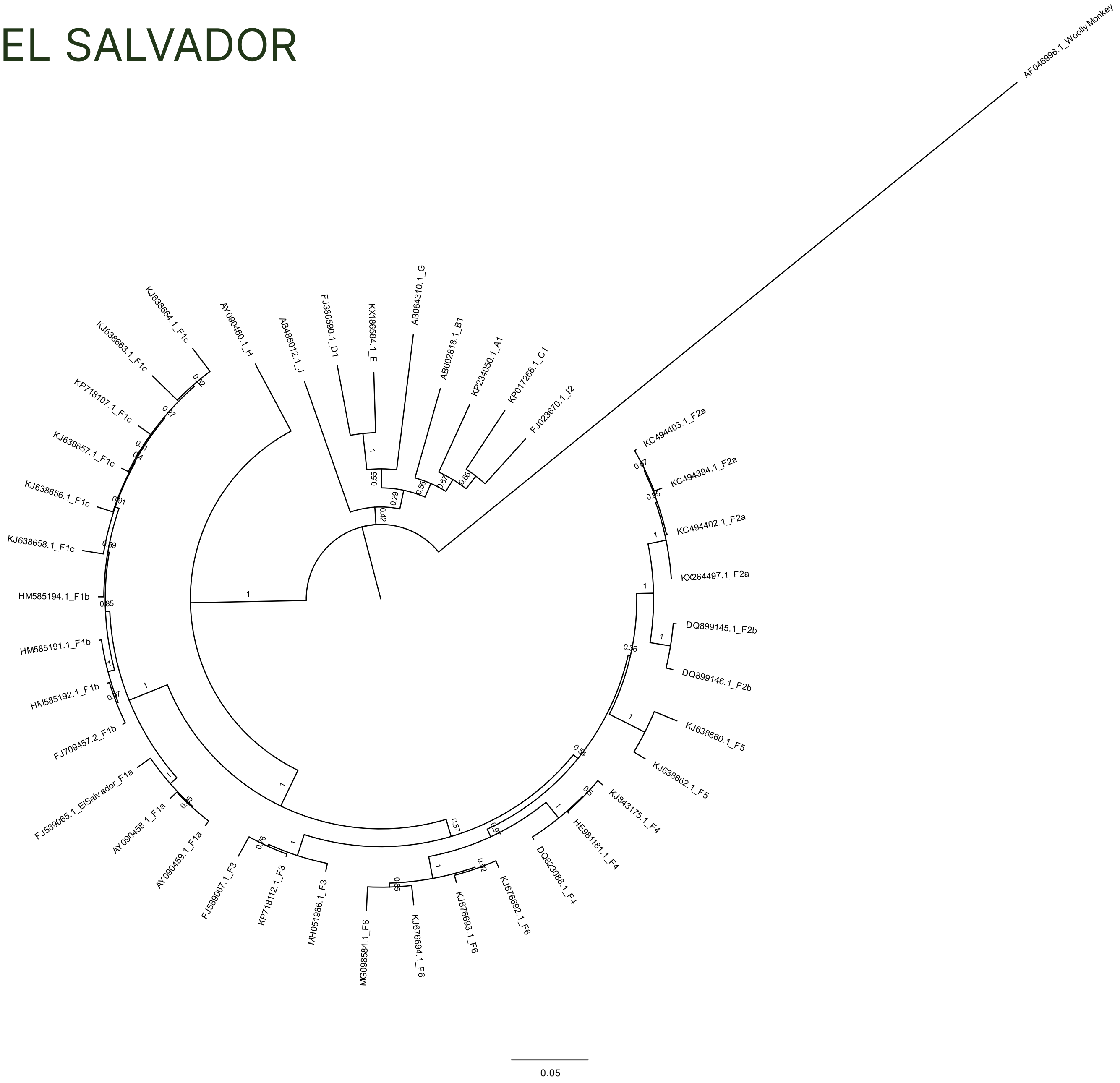

Tree 1. The evolutionary history was inferred by using the Maximum Likelihood method and Tamura-Nei model. The percentage of replicate trees in which the associated taxa clustered together in the bootstrap test (1000 replicates) are shown next to the branches. Initial tree(s) for the heuristic search were obtained automatically by applying Neighbor-Join and BioNJ algorithms to a matrix of pairwise distances estimated using the Tamura-Nei model, and then selecting the topology with superior log likelihood value. A discrete Gamma distribution was used to model evolutionary rate differences among sites (5 categories (+*G*, parameter = 0.2622)). The tree is drawn to scale, with branch lengths measured in the number of substitutions per site. The analysis involved 41 nucleotide sequences, of which 40 were used as marker sequences to determine the genotype of 1 sequence. All positions containing gaps and missing data were eliminated. There was a total of 3021 positions in the final dataset. Evolutionary analyses were conducted in MEGA X.

ID	GENOTYPE	SUBTYPE	COUNTRY	TREE	ALIGNMENT[1]	BASE PAIRS
FJ589065	F	F1a	El Salvador	1	Complete Genome	3174

[1]Alignment to complete genome reference sequence VHB NC_003977.2

NICARAGUA

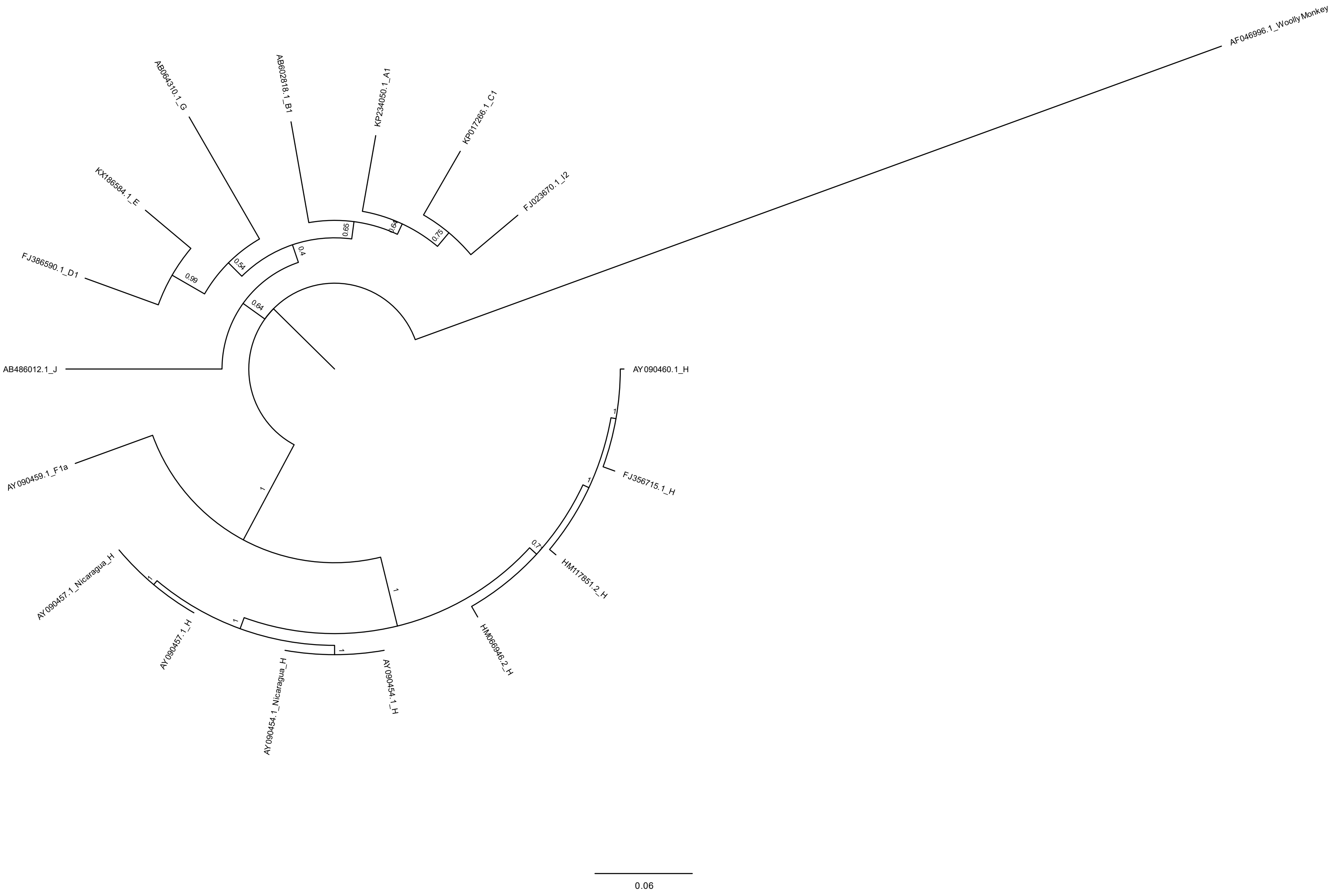

Tree 1. The evolutionary history was inferred by using the Maximum Likelihood method and Tamura-Nei model. The percentage of replicate trees in which the associated taxa clustered together in the bootstrap test (1000 replicates) are shown next to the branches. Initial tree(s) for the heuristic search were obtained automatically by applying Neighbor-Join and BioNJ algorithms to a matrix of pairwise distances estimated using the Tamura-Nei model, and then selecting the topology with superior log likelihood value. A discrete Gamma distribution was used to model evolutionary rate differences among sites (5 categories (+*G*, parameter = 0.2362)). The tree is drawn to scale, with branch lengths measured in the number of substitutions per site. The analysis involved 18 nucleotide sequences, of which 16 were used as marker sequences to determine the genotype of 2 sequences. All positions containing gaps and missing data were eliminated. There was a total of 3167 positions in the final dataset. Evolutionary analyses were conducted in MEGA X.

ID	GENOTYPE	SUBTYPE	COUNTRY	TREE	ALIGNMENT[1]	BASE PAIRS
AY090454	H	H	Nicaragua	1	Complete Genome	3215
AY090457	H	H	Nicaragua	1	Complete Genome	3215

[1]Alignment to complete genome reference sequence VHB NC_003977.2

MARTINIQUE

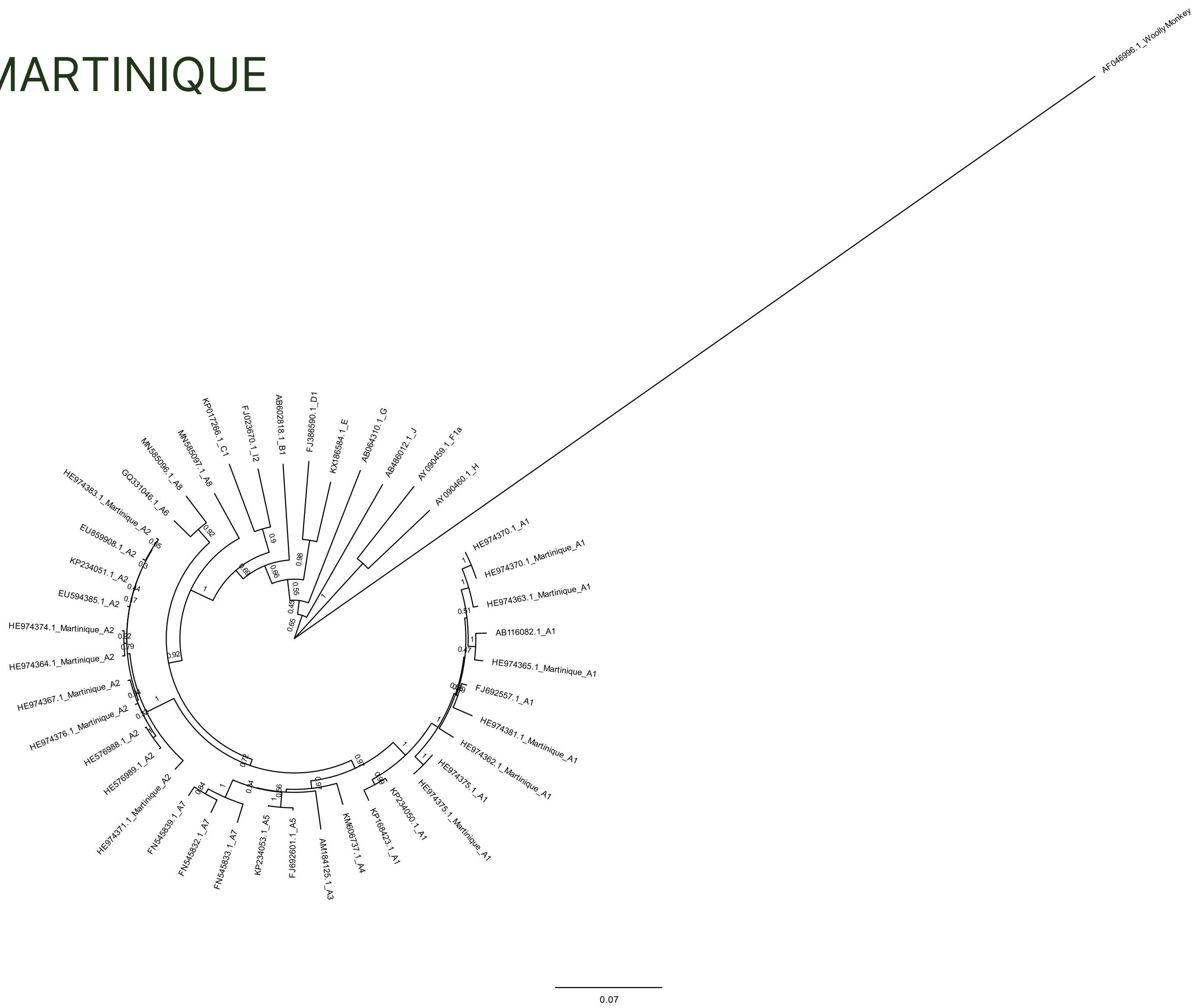

Tree 1. The evolutionary history was inferred by using the Maximum Likelihood method and Tamura-Nei model. The percentage of replicate trees in which the associated taxa clustered together in the bootstrap test (1000 replicates) are shown next to the branches. Initial tree(s) for the heuristic search were obtained automatically by applying Neighbor-Join and BioNJ algorithms to a matrix of pairwise distances estimated using the Tamura-Nei model, and then selecting the topology with superior log likelihood value. A discrete Gamma distribution was used to model evolutionary rate differences among sites (5 categories (+G, parameter = 0.2186)). The tree is drawn to scale, with branch lengths measured in the number of substitutions per site. The analysis involved 43 nucleotide sequences, of which 31 were used as marker sequences to determine the genotype of 12 sequences. All positions containing gaps and missing data were eliminated. There was a total of 3014 positions in the final dataset. Evolutionary analyses were conducted in MEGA X.

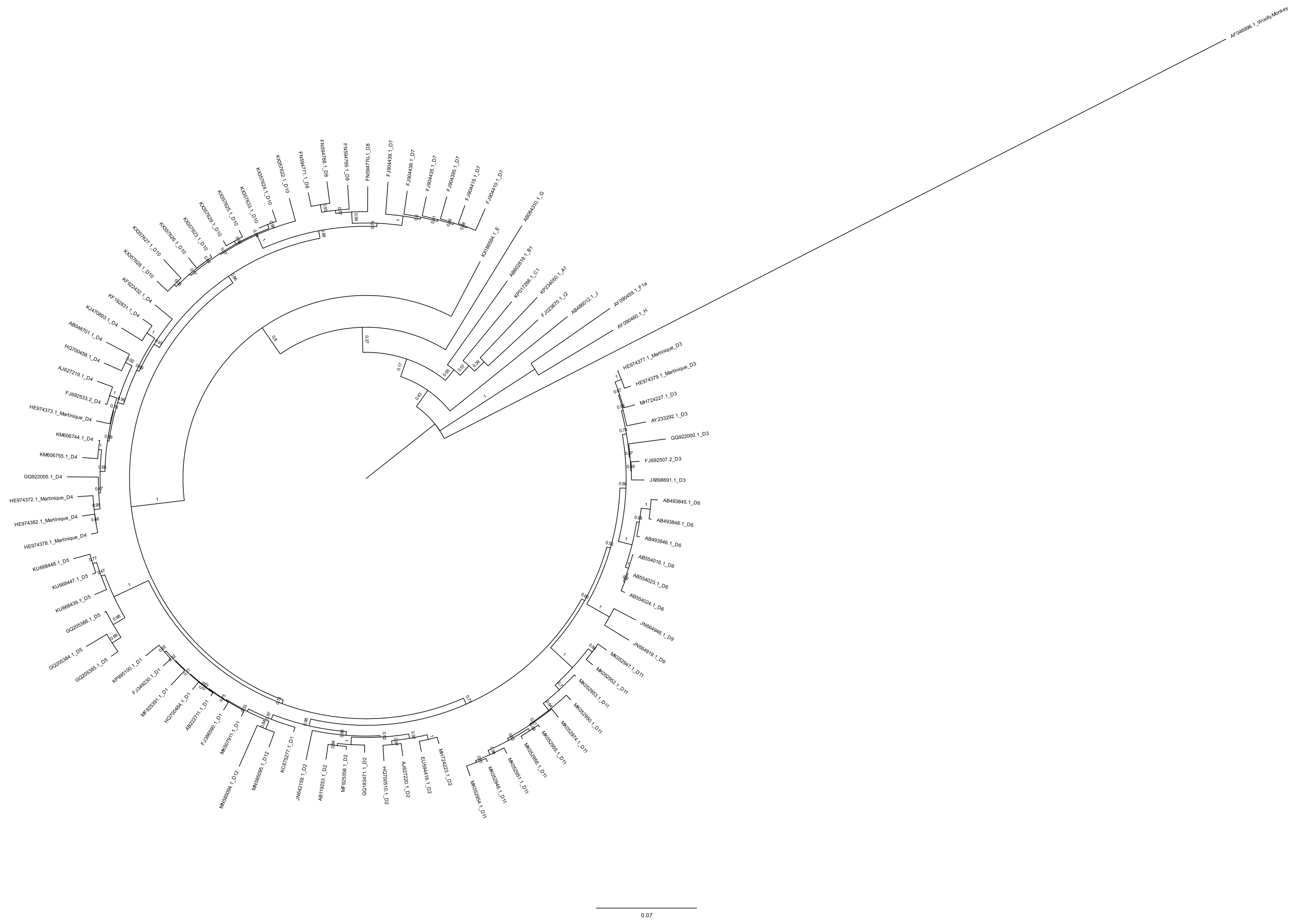

Tree 2. The evolutionary history was inferred by using the Maximum Likelihood method and Tamura-Nei model. The percentage of replicate trees in which the associated taxa clustered together in the bootstrap test (1000 replicates) are shown next to the branches. Initial tree(s) for the heuristic search were obtained automatically by applying Neighbor-Join and BioNJ algorithms to a matrix of pairwise distances estimated using the Tamura-Nei model, and then selecting the topology with superior log likelihood value. A discrete Gamma distribution was used to model evolutionary rate differences among sites (5 categories (+*G*, parameter = 0.2751)). The tree is drawn to scale, with branch lengths measured in the number of substitutions per site. The analysis involved 92 nucleotide sequences, of which 86 were used as marker sequences to determine the genotype of 6 sequences. All positions containing gaps and missing data were eliminated. There was a total of 3028 positions in the final dataset. Evolutionary analyses were conducted in MEGA X.

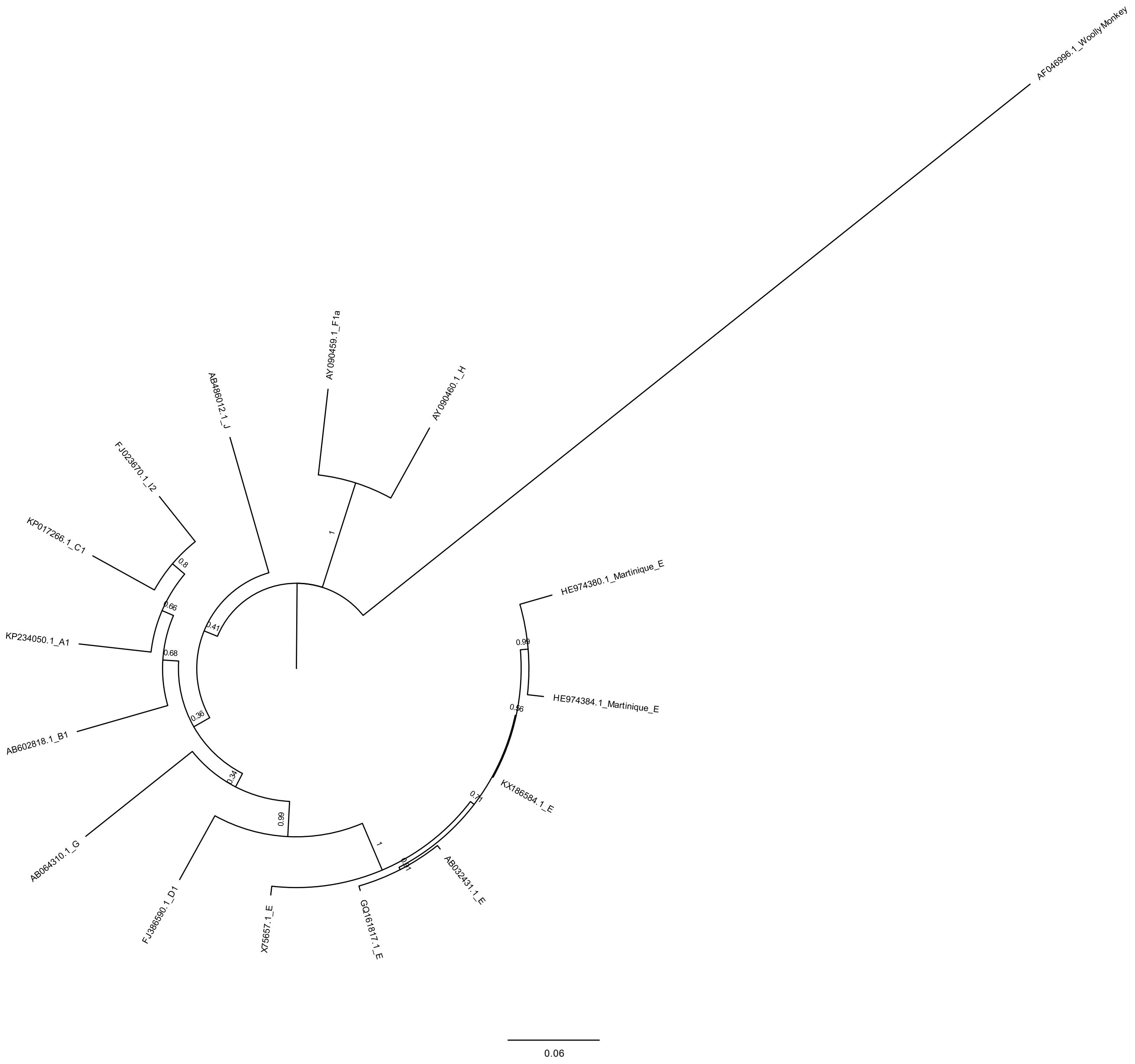

Tree 3. The evolutionary history was inferred by using the Maximum Likelihood method and Tamura-Nei model. The percentage of replicate trees in which the associated taxa clustered together in the bootstrap test (1000 replicates) are shown next to the branches. Initial tree(s) for the heuristic search were obtained automatically by applying Neighbor-Join and BioNJ algorithms to a matrix of pairwise distances estimated using the Tamura-Nei model, and then selecting the topology with superior log likelihood value. A discrete Gamma distribution was used to model evolutionary rate differences among sites (5 categories (+*G*, parameter = 0.2286)). The tree is drawn to scale, with branch lengths measured in the number of substitutions per site. The analysis involved 16 nucleotide sequences, of which 14 were used as marker sequences to determine the genotype of 2 sequences. All positions containing gaps and missing data were eliminated. There was a total of 3144 positions in the final dataset. Evolutionary analyses were conducted in MEGA X.

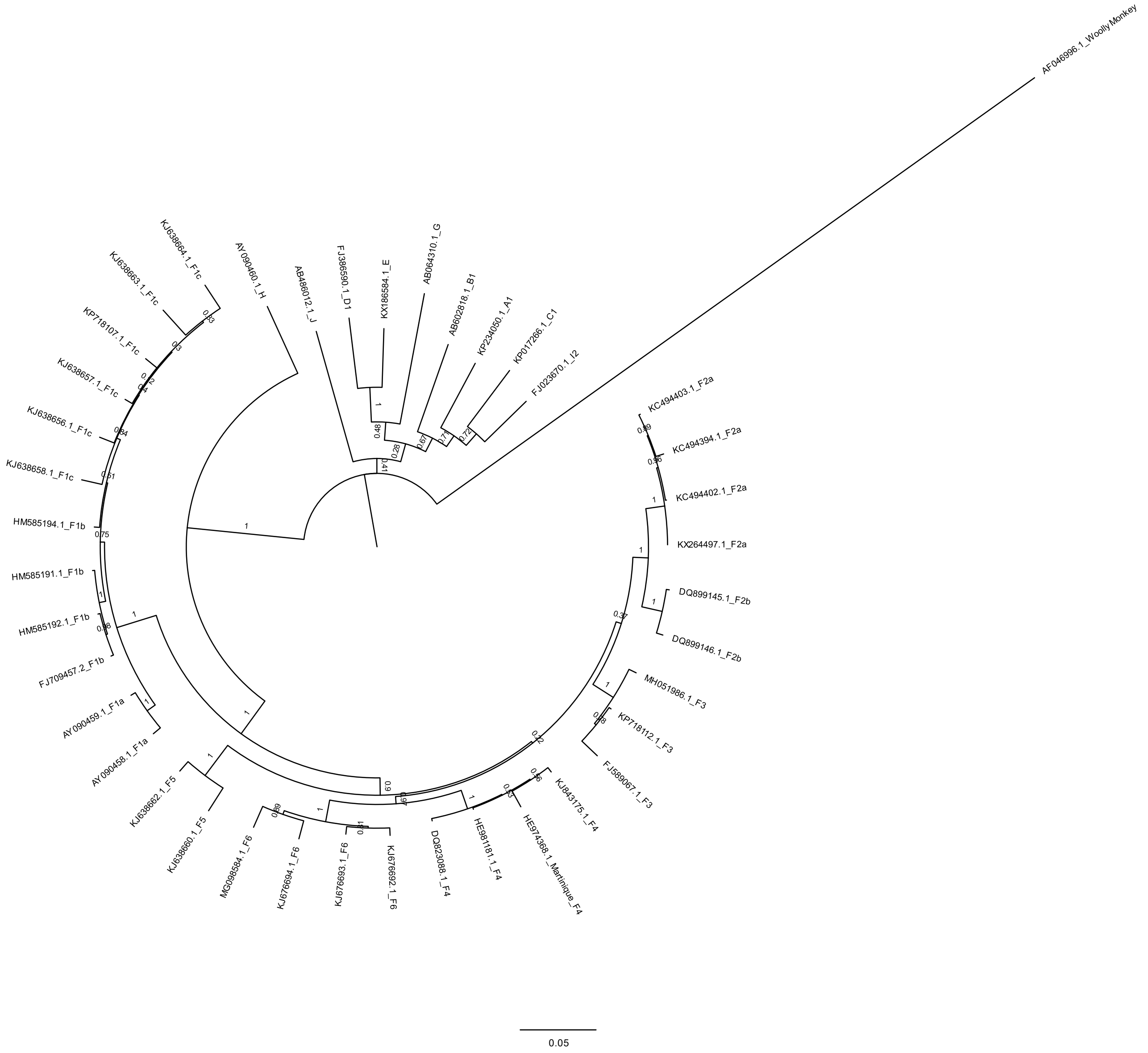

Tree 4. The evolutionary history was inferred by using the Maximum Likelihood method and Tamura-Nei model. The percentage of replicate trees in which the associated taxa clustered together in the bootstrap test (1000 replicates) are shown next to the branches. Initial tree(s) for the heuristic search were obtained automatically by applying Neighbor-Join and BioNJ algorithms to a matrix of pairwise distances estimated using the Tamura-Nei model, and then selecting the topology with superior log likelihood value. A discrete Gamma distribution was used to model evolutionary rate differences among sites (5 categories (+G, parameter = 0.2641)). The tree is drawn to scale, with branch lengths measured in the number of substitutions per site. The analysis involved 41 nucleotide sequences, of which 40 were used as marker sequences to determine the genotype of 1 sequence. All positions containing gaps and missing data were eliminated. There was a total of 3064 positions in the final dataset. Evolutionary analyses were conducted in MEGA X.

ID	GENOTYPE	SUBTYPE	COUNTRY	TREE	ALIGNMENT[1]	BASE PAIRS
HE974362	A	A1	Martinique	1	Complete Genome	3221
HE974363	A	A1	Martinique	1	Complete Genome	3213
HE974364	A	A2	Martinique	1	Complete Genome	3221

HE974365	A	A1	Martinique	1	Complete Genome	3221
HE974367	A	A2	Martinique	1	Complete Genome	3221
HE974370	A	A1	Martinique	1	Complete Genome	3221
HE974371	A	A2	Martinique	1	Complete Genome	3221
HE974374	A	A2	Martinique	1	Complete Genome	3221
HE974375	A	A1	Martinique	1	Complete Genome	3218
HE974376	A	A2	Martinique	1	Complete Genome	3221
HE974381	A	A1	Martinique	1	Complete Genome	3221
HE974383	A	A2	Martinique	1	Complete Genome	3221
HE974372	D	D4	Martinique	2	Complete Genome	3182
HE974373	D	D4	Martinique	2	Complete Genome	3182
HE974377	D	D3	Martinique	2	Complete Genome	3182
HE974378	D	D4	Martinique	2	Complete Genome	3182
HE974379	D	D3	Martinique	2	Complete Genome	3179
HE974382	D	D4	Martinique	2	Complete Genome	3182
HE974380	E	E	Martinique	3	Complete Genome	3212
HE974384	E	E	Martinique	3	Complete Genome	3212
HE974368	F	F4	Martinique	4	Complete Genome	3215

[1]Alignment to complete genome reference sequence VHB NC_003977.2

PANAMA

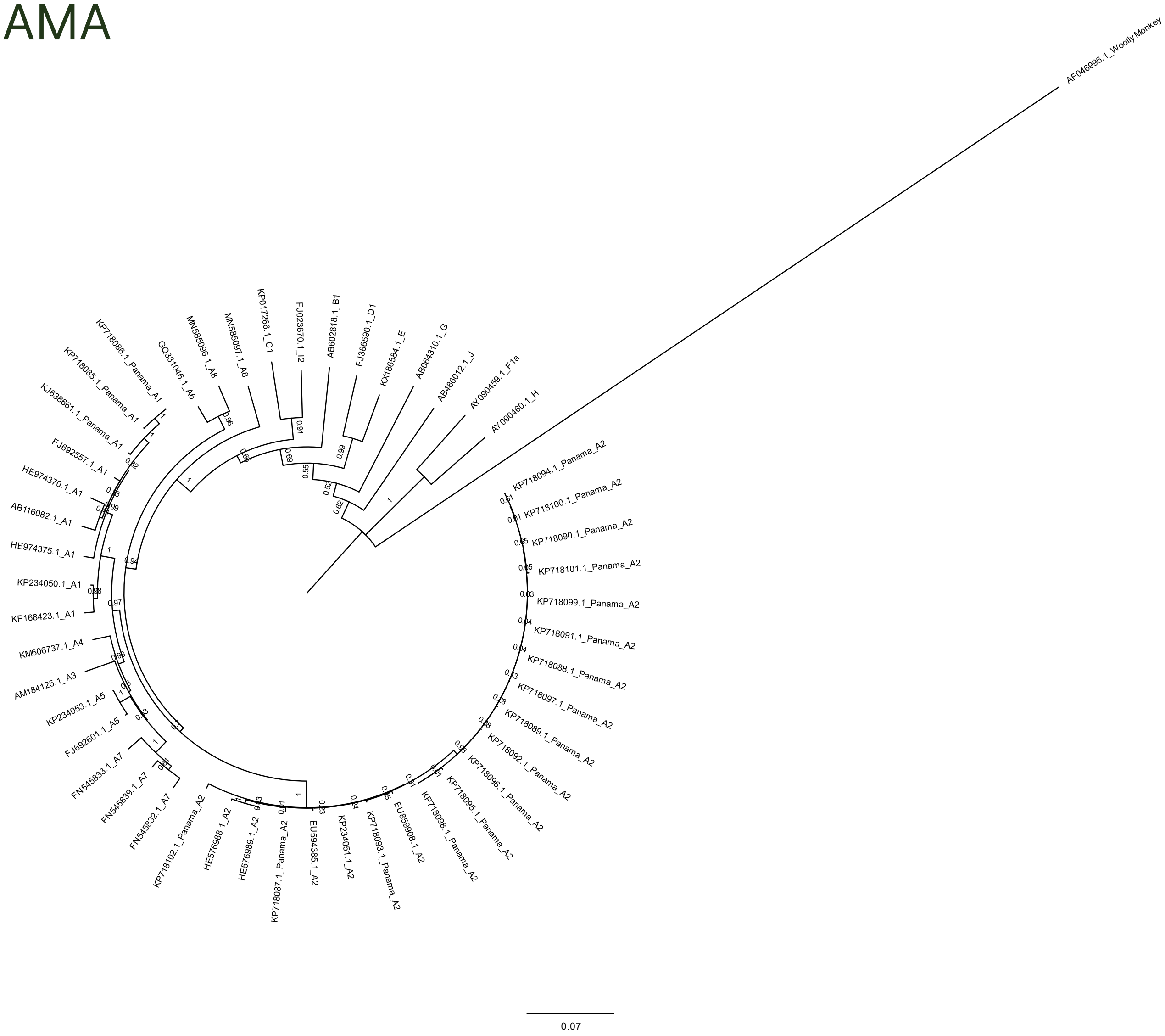

Tree 1. The evolutionary history was inferred by using the Maximum Likelihood method and Tamura-Nei model. The percentage of replicate trees in which the associated taxa clustered together in the bootstrap test (1000 replicates) are shown next to the branches. Initial tree(s) for the heuristic search were obtained automatically by applying Neighbor-Join and BioNJ algorithms to a matrix of pairwise distances estimated using the Tamura-Nei model, and then selecting the topology with superior log likelihood value. A discrete Gamma distribution was used to model evolutionary rate differences among sites (5 categories (+*G*, parameter = 0.2148)). The tree is drawn to scale, with branch lengths measured in the number of substitutions per site. The analysis involved 50 nucleotide sequences, of which 31 were used as marker sequences to determine the genotype of 19 sequences. All positions containing gaps and missing data were eliminated. There was a total of 2991 positions in the final dataset. Evolutionary analyses were conducted in MEGA X.

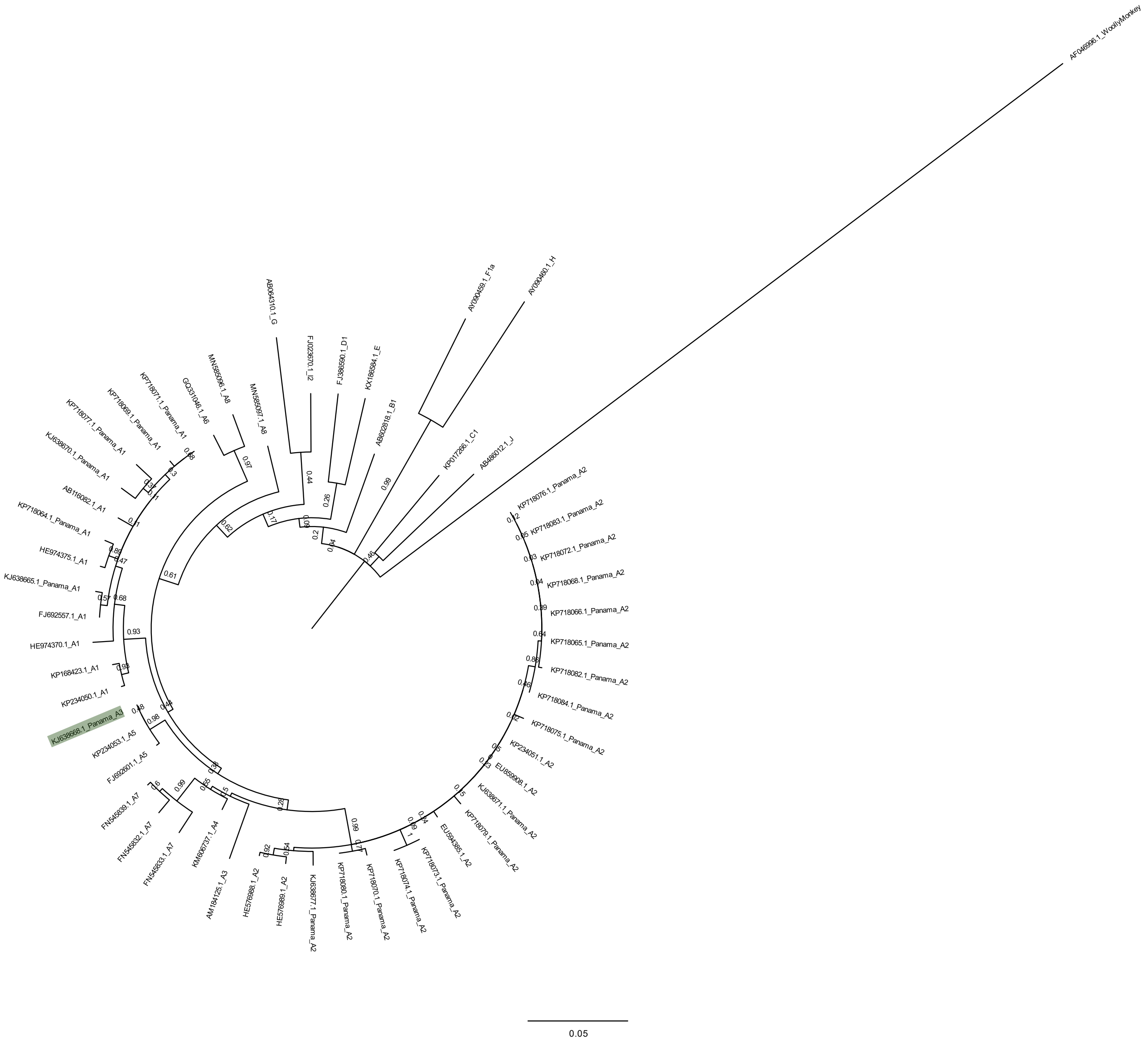

Tree 2. The evolutionary history was inferred by using the Maximum Likelihood method and Tamura-Nei model. The percentage of replicate trees in which the associated taxa clustered together in the bootstrap test (1000 replicates) are shown next to the branches. Initial tree(s) for the heuristic search were obtained automatically by applying Neighbor-Join and BioNJ algorithms to a matrix of pairwise distances estimated using the Tamura-Nei model, and then selecting the topology with superior log likelihood value. A discrete Gamma distribution was used to model evolutionary rate differences among sites (5 categories (+G, parameter = 0.1791)). The tree is drawn to scale, with branch lengths measured in the number of substitutions per site. The analysis involved 54 nucleotide sequences, of which 31 were used as marker sequences to determine the genotype of 23 sequences. Sequence KJ638668 changed from subtype A3 to A5; the authors in the original article grouped it with reference sequences belonging to A5, erroneously labeled as A3. All positions containing gaps and missing data were eliminated. There was a total of 739 positions in the final dataset. Evolutionary analyses were conducted in MEGA X.

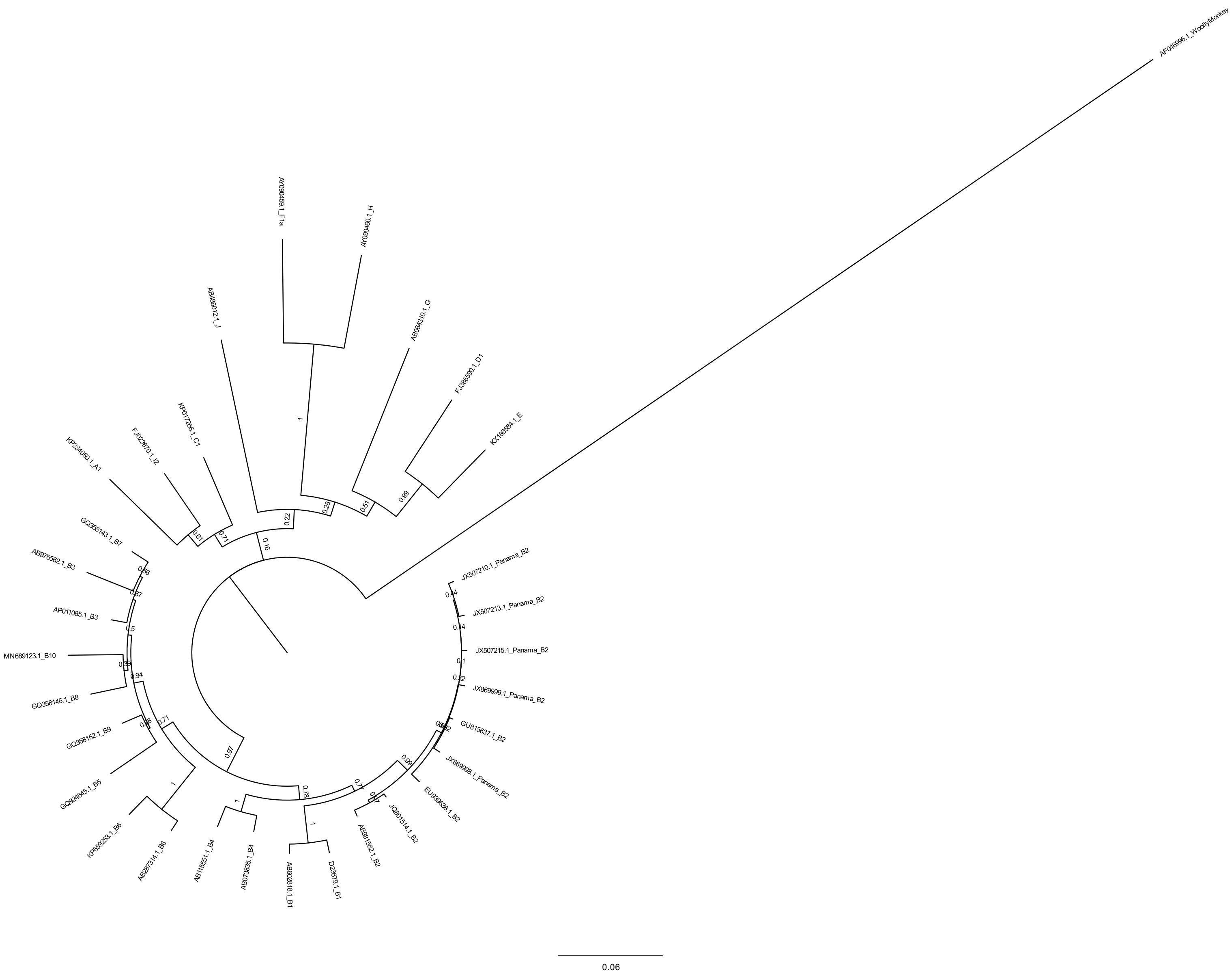

Tree 3. The evolutionary history was inferred by using the Maximum Likelihood method and Tamura-Nei model. The percentage of replicate trees in which the associated taxa clustered together in the bootstrap test (1000 replicates) are shown next to the branches. Initial tree(s) for the heuristic search were obtained automatically by applying Neighbor-Join and BioNJ algorithms to a matrix of pairwise distances estimated using the Tamura-Nei model, and then selecting the topology with superior log likelihood value. A discrete Gamma distribution was used to model evolutionary rate differences among sites (5 categories (+G, parameter = 0.2556)). The tree is drawn to scale, with branch lengths measured in the number of substitutions per site. The analysis involved 32 nucleotide sequences, of which 27 were used as marker sequences to determine the genotype of 5 sequences. All positions containing gaps and missing data were eliminated. There was a total of 3120 positions in the final dataset. Evolutionary analyses were conducted in MEGA X.

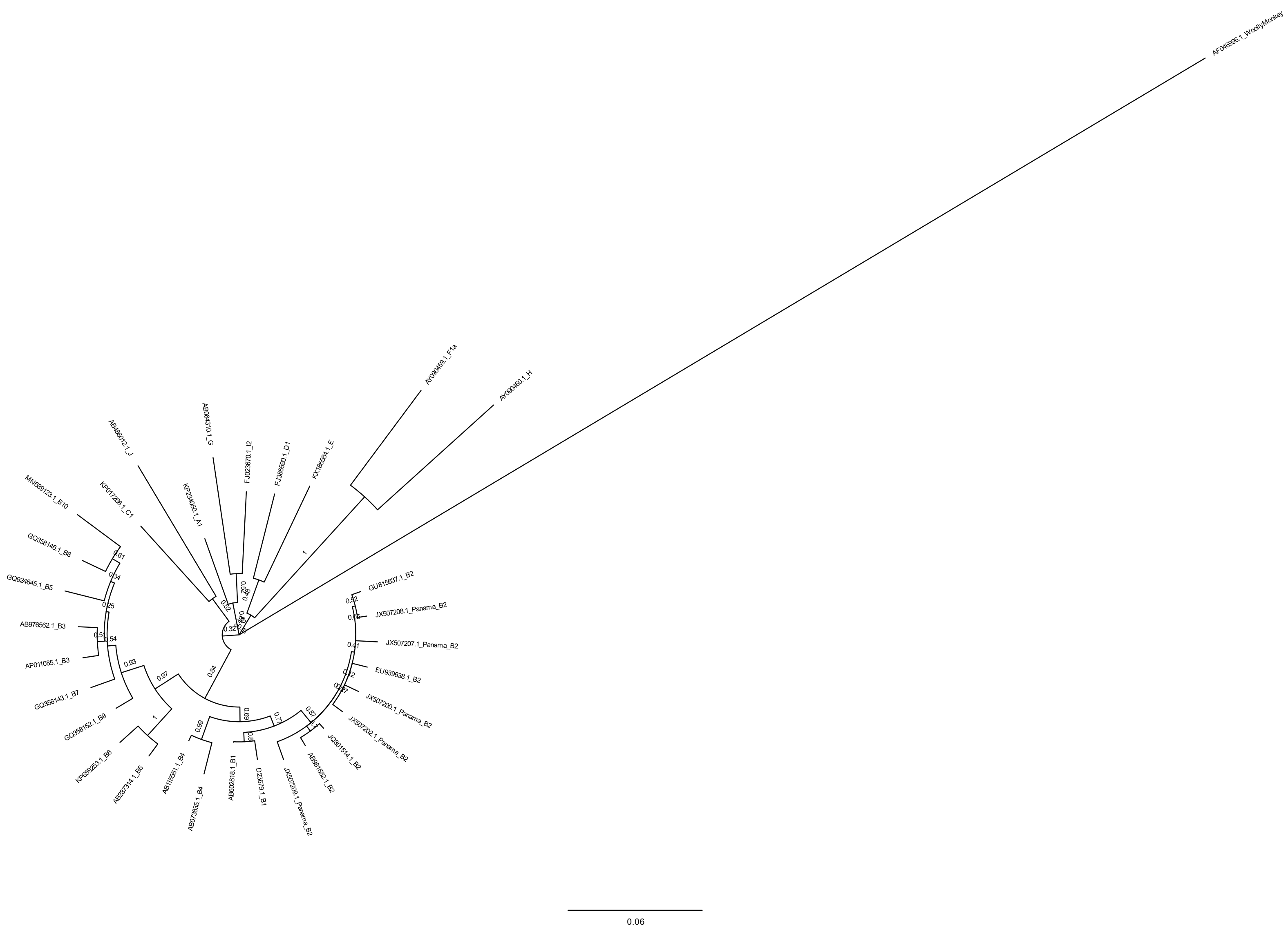

Tree 4. The evolutionary history was inferred by using the Maximum Likelihood method and Tamura-Nei model. The percentage of replicate trees in which the associated taxa clustered together in the bootstrap test (1000 replicates) are shown next to the branches. Initial tree(s) for the heuristic search were obtained automatically by applying Neighbor-Join and BioNJ algorithms to a matrix of pairwise distances estimated using the Tamura-Nei model, and then selecting the topology with superior log likelihood value. A discrete Gamma distribution was used to model evolutionary rate differences among sites (5 categories (+*G*, parameter = 0.1742)). The tree is drawn to scale, with branch lengths measured in the number of substitutions per site. The analysis involved 32 nucleotide sequences, of which 27 were used as marker sequences to determine the genotype of 5 sequences. All positions containing gaps and missing data were eliminated. There was a total of 841 positions in the final dataset. Evolutionary analyses were conducted in MEGA X.

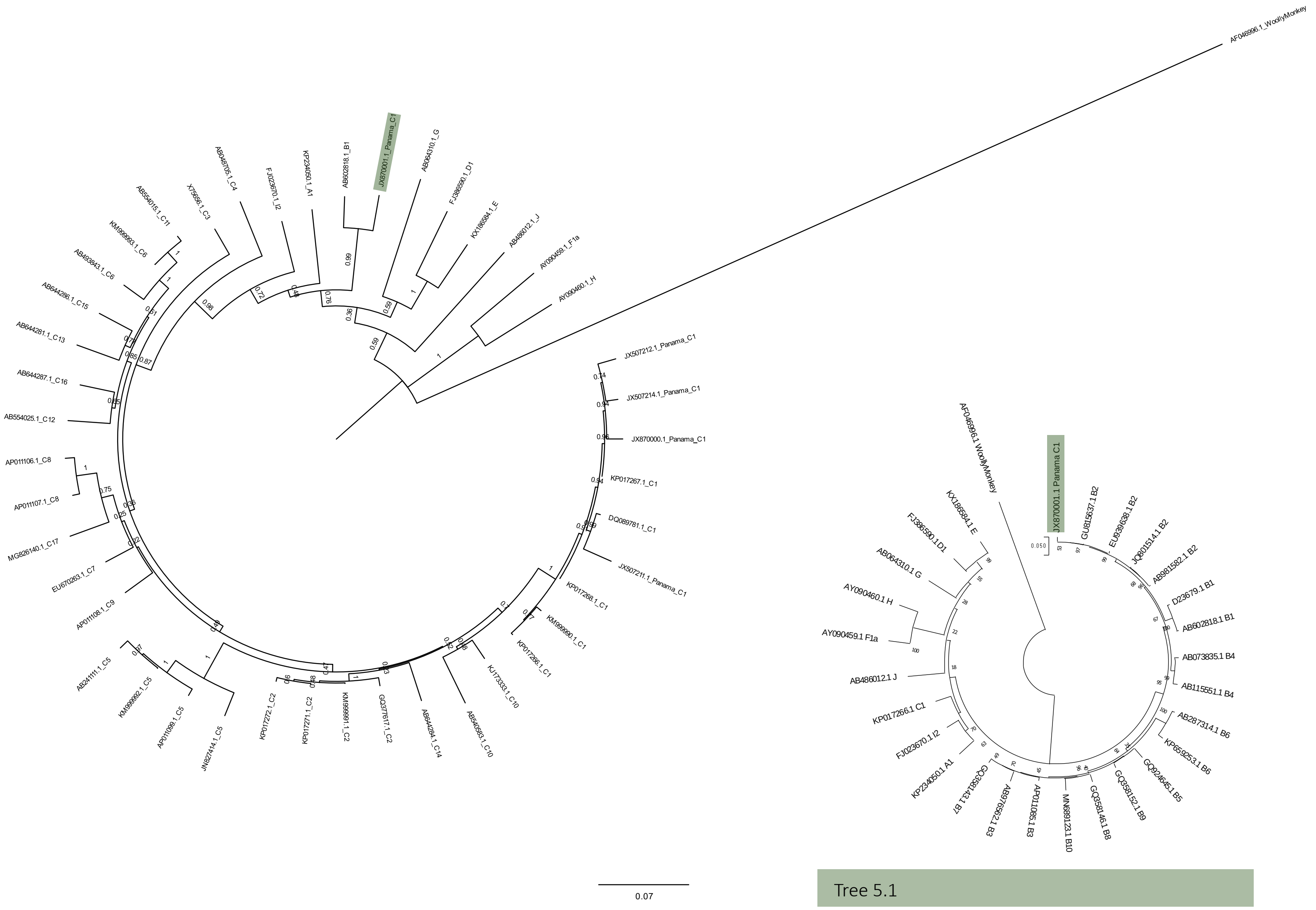

Tree 5.1

Tree 5. The evolutionary history was inferred by using the Maximum Likelihood method and Tamura-Nei model. The percentage of replicate trees in which the associated taxa clustered together in the bootstrap test (1000 replicates) are shown next to the branches. Initial tree(s) for the heuristic search were obtained automatically by applying Neighbor-Join and BioNJ algorithms to a matrix of pairwise distances estimated using the Tamura-Nei model, and then selecting the topology with superior log likelihood value. A discrete Gamma distribution was used to model evolutionary rate differences among sites (5 categories (+G, parameter = 0.2310)). The tree is drawn to scale, with branch lengths measured in the number of substitutions per site. The analysis involved 45 nucleotide sequences, of which 40 were used as marker sequences to determine the genotype of 5 sequences. Sequence JX870001 was grouped with outgroup genotype B, for which a new phylogenetic tree was produced under the same parameters (Tree 5.1) in which it was classified under subtype B2. The article linked to said sequence does not provide labeled trees nor specific tables but does report B2 sequences as well as the reported C1 (probable labeling mistake). All positions containing gaps and missing data were eliminated. There was a total of 3119 positions in the final dataset. Evolutionary analyses were conducted in MEGA X.

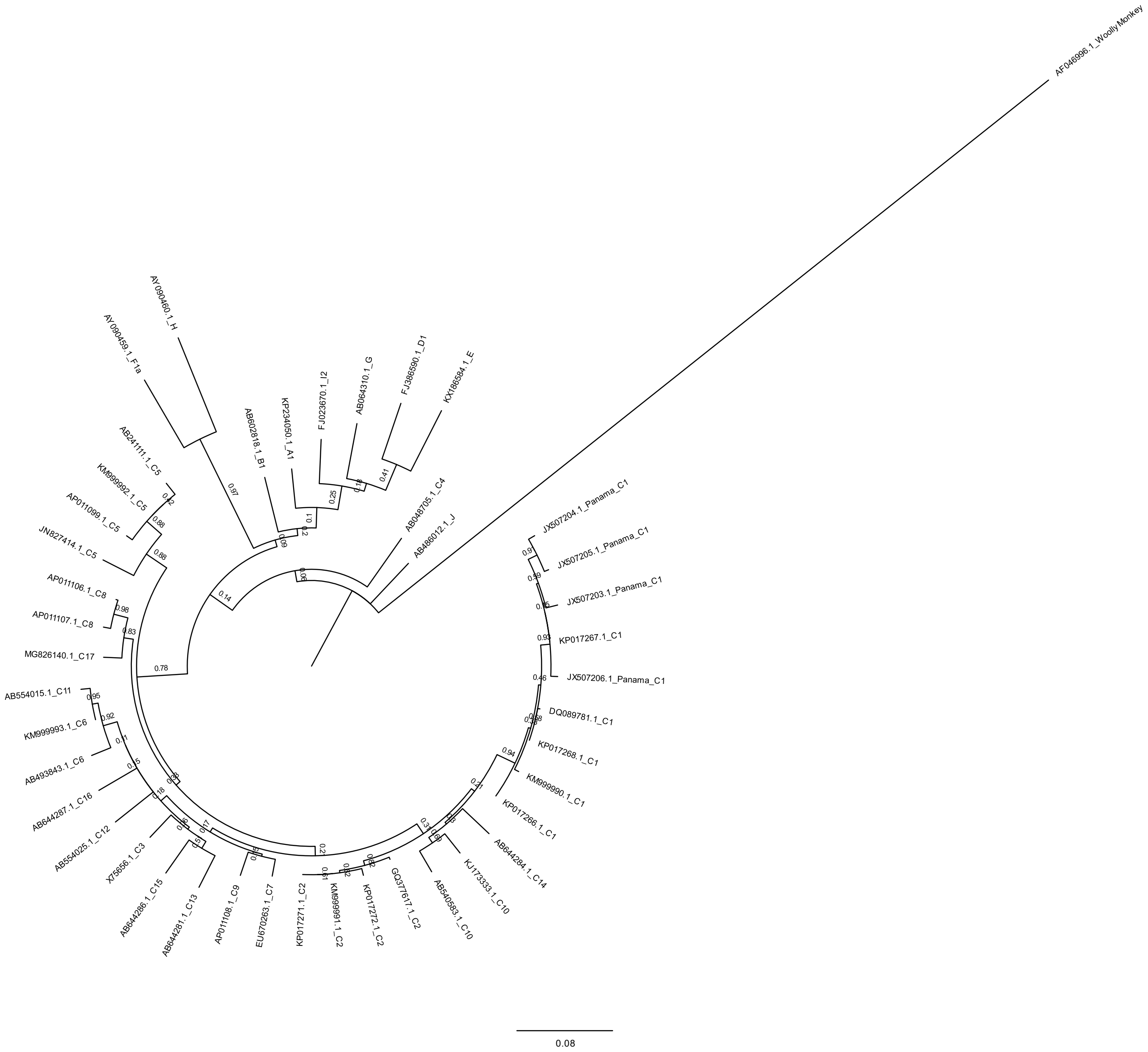

Tree 6. The evolutionary history was inferred by using the Maximum Likelihood method and Tamura-Nei model. The percentage of replicate trees in which the associated taxa clustered together in the bootstrap test (1000 replicates) are shown next to the branches. Initial tree(s) for the heuristic search were obtained automatically by applying Neighbor-Join and BioNJ algorithms to a matrix of pairwise distances estimated using the Tamura-Nei model, and then selecting the topology with superior log likelihood value. A discrete Gamma distribution was used to model evolutionary rate differences among sites (5 categories (+*G*, parameter = 0.1580)). The tree is drawn to scale, with branch lengths measured in the number of substitutions per site. The analysis involved 44 nucleotide sequences, of which 40 were used as marker sequences to determine the genotype of 4 sequences. All positions containing gaps and missing data were eliminated. There was a total of 719 positions in the final dataset. Evolutionary analyses were conducted in MEGA X.

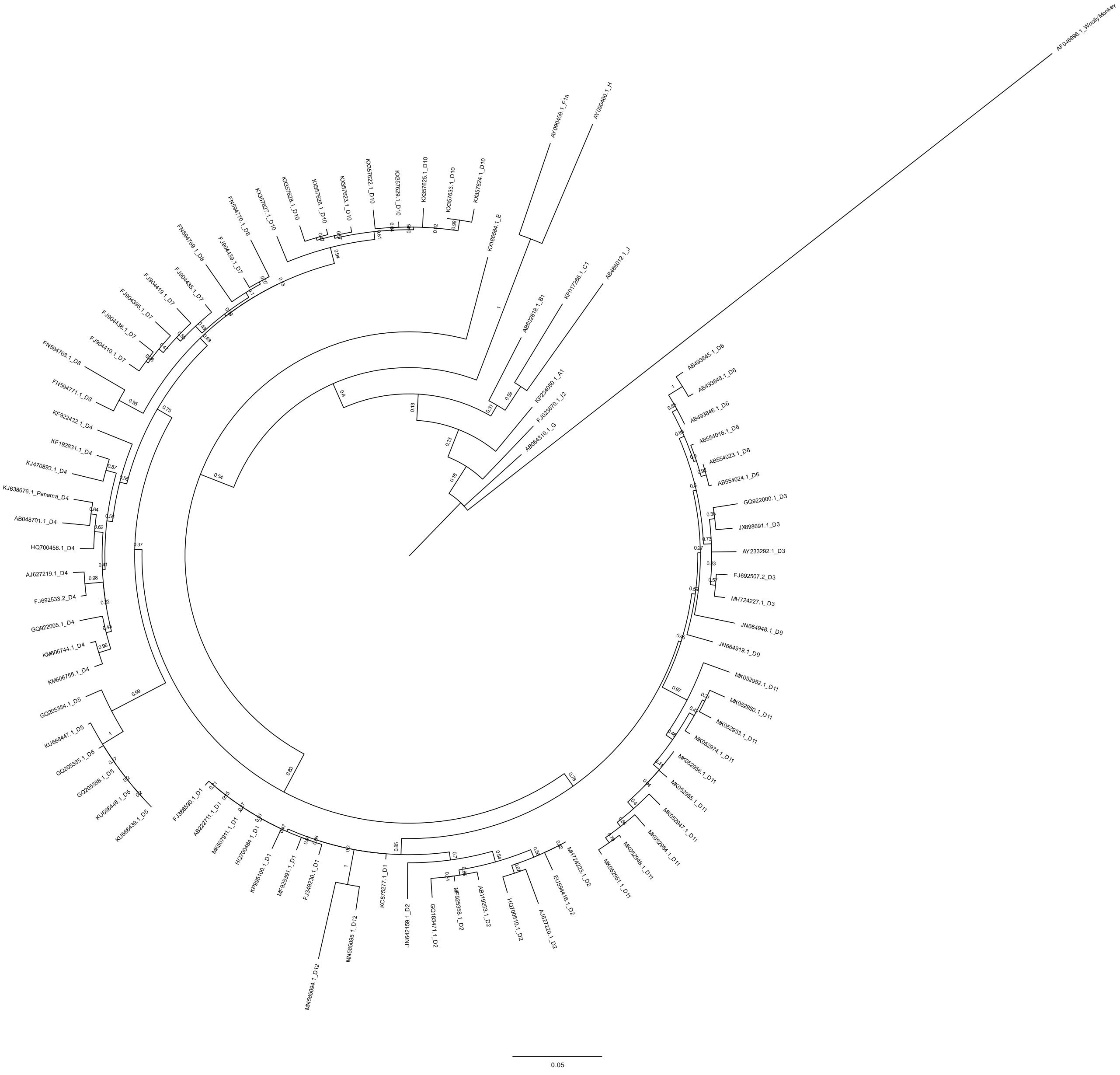

Tree 7. The evolutionary history was inferred by using the Maximum Likelihood method and Tamura-Nei model. The percentage of replicate trees in which the associated taxa clustered together in the bootstrap test (1000 replicates) are shown next to the branches. Initial tree(s) for the heuristic search were obtained automatically by applying Neighbor-Join and BioNJ algorithms to a matrix of pairwise distances estimated using the Tamura-Nei model, and then selecting the topology with superior log likelihood value. A discrete Gamma distribution was used to model evolutionary rate differences among sites (5 categories (+*G*, parameter = 0.2494)). The tree is drawn to scale, with branch lengths measured in the number of substitutions per site. The analysis involved 87 nucleotide sequences, of which 86 were used as marker sequences to determine the genotype of 1 sequence. All positions containing gaps and missing data were eliminated. There was a total of 840 positions in the final dataset. Evolutionary analyses were conducted in MEGA X.

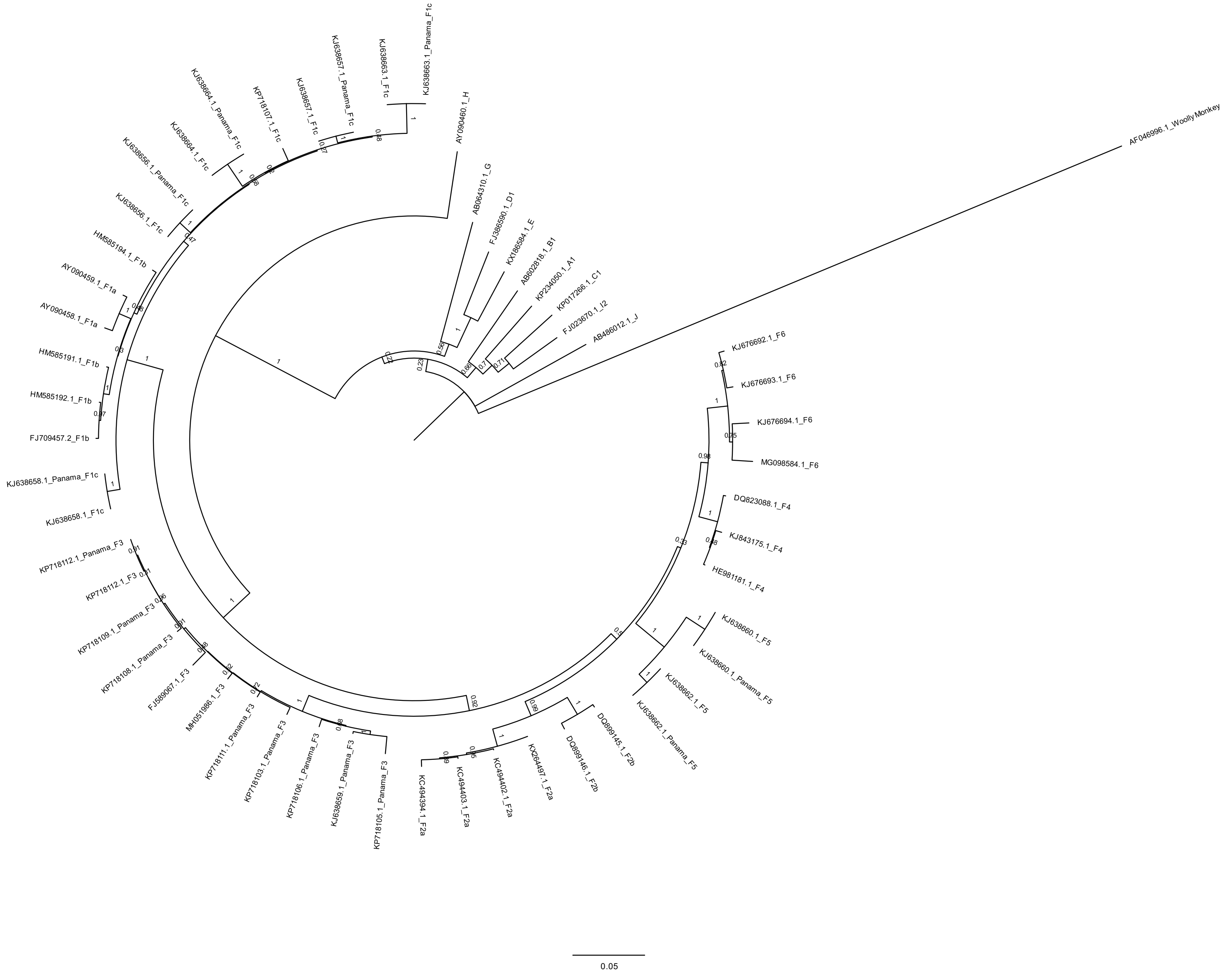

Tree 8. The evolutionary history was inferred by using the Maximum Likelihood method and Tamura-Nei model. The percentage of replicate trees in which the associated taxa clustered together in the bootstrap test (1000 replicates) are shown next to the branches. Initial tree(s) for the heuristic search were obtained automatically by applying Neighbor-Join and BioNJ algorithms to a matrix of pairwise distances estimated using the Tamura-Nei model, and then selecting the topology with superior log likelihood value. A discrete Gamma distribution was used to model evolutionary rate differences among sites (5 categories (+G, parameter = 0.2663)). The tree is drawn to scale, with branch lengths measured in the number of substitutions per site. The analysis involved 55 nucleotide sequences, of which 40 were used as marker sequences to determine the genotype of 15 sequences. All positions containing gaps and missing data were eliminated. There was a total of 3032 positions in the final dataset. Evolutionary analyses were conducted in MEGA X.

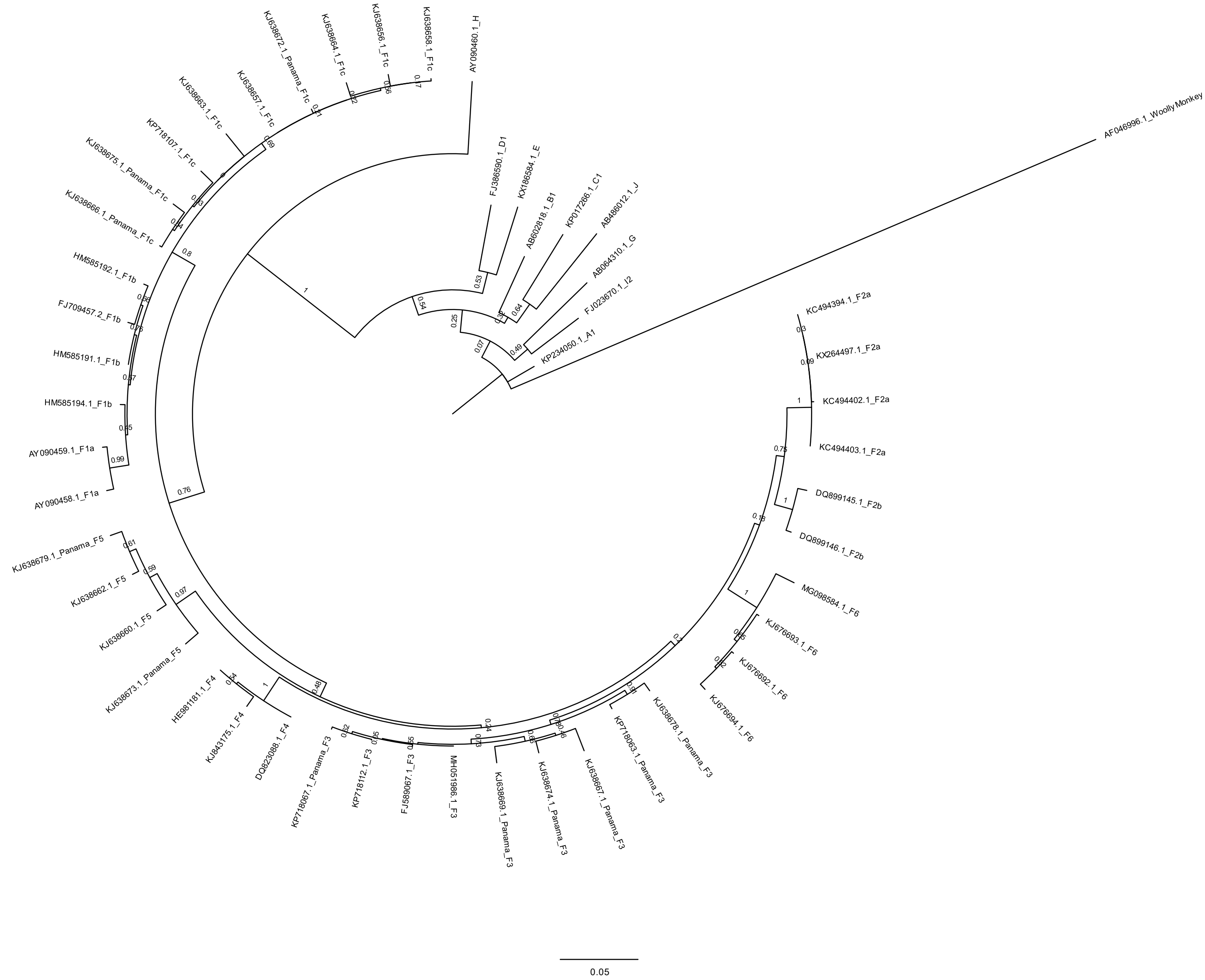

Tree 9. The evolutionary history was inferred by using the Maximum Likelihood method and Tamura-Nei model. The percentage of replicate trees in which the associated taxa clustered together in the bootstrap test (1000 replicates) are shown next to the branches. Initial tree(s) for the heuristic search were obtained automatically by applying Neighbor-Join and BioNJ algorithms to a matrix of pairwise distances estimated using the Tamura-Nei model, and then selecting the topology with superior log likelihood value. A discrete Gamma distribution was used to model evolutionary rate differences among sites (5 categories (+*G*, parameter = 0.2103)). The tree is drawn to scale, with branch lengths measured in the number of substitutions per site. The analysis involved 51 nucleotide sequences, of which 40 were used as marker sequences to determine the genotype of 11 sequences. All positions containing gaps and missing data were eliminated. There was a total of 805 positions in the final dataset. Evolutionary analyses were conducted in MEGA X.

ID	GENOTYPE	SUBTYPE	COUNTRY	TREE	ALIGNMENT[1]	BASE PAIRS
KP718085	A	A1	Panama	1	Complete Genome	3221
KP718086	A	A1	Panama	1	Complete Genome	3221
KP718087	A	A2	Panama	1	Complete Genome	3221
KP718088	A	A2	Panama	1	Complete Genome	3221
KP718089	A	A2	Panama	1	Complete Genome	3221

KP718090	A	A2	Panama	1	Complete Genome	3221
KP718091	A	A2	Panama	1	Complete Genome	3221
KP718092	A	A2	Panama	1	Complete Genome	3221
KP718093	A	A2	Panama	1	Complete Genome	3221
KP718094	A	A2	Panama	1	Complete Genome	3221
KP718095	A	A2	Panama	1	Complete Genome	3221
KP718096	A	A2	Panama	1	Complete Genome	3221
KP718097	A	A2	Panama	1	Complete Genome	3221
KP718098	A	A2	Panama	1	Complete Genome	3221
KP718099	A	A2	Panama	1	Complete Genome	3221
KP718100	A	A2	Panama	1	Complete Genome	3221
KP718101	A	A2	Panama	1	Complete Genome	3221
KP718102	A	A2	Panama	1	Complete Genome	3221
KJ638661	A	A1	Panama	1	Complete Genome	3221
KJ638665	A	A1	Panama	2	276-1049	774
KJ638668	A	A5	Panama	2	276-1115	840
KJ638670	A	A1	Panama	2	276-1122	847
KJ638671	A	A2	Panama	2	276-1123	848
KJ638677	A	A2	Panama	2	276-1123	848
KP718064	A	A1	Panama	2	246-1109	864
KP718065	A	A2	Panama	2	246-1123	878
KP718066	A	A2	Panama	2	252-1123	872
KP718068	A	A2	Panama	2	246-1122	877
KP718069	A	A1	Panama	2	246-1123	878
KP718070	A	A2	Panama	2	246-1123	878
KP718071	A	A1	Panama	2	246-1123	878
KP718072	A	A2	Panama	2	246-1114	869
KP718073	A	A2	Panama	2	246-1111	866
KP718074	A	A2	Panama	2	246-1111	866
KP718075	A	A2	Panama	2	249-1110	862
KP718076	A	A2	Panama	2	261-1106	846
KP718077	A	A1	Panama	2	249-1107	859
KP718079	A	A2	Panama	2	258-1108	851
KP718080	A	A2	Panama	2	261-1108	848
KP718082	A	A2	Panama	2	246-1123	878
KP718083	A	A2	Panama	2	246-1123	878
KP718084	A	A2	Panama	2	246-1123	878
JX507210	B	B2	Panama	3	Complete Genome	3215
JX507213	B	B2	Panama	3	Complete Genome	3215
JX507215	B	B2	Panama	3	Complete Genome	3215
JX869998	B	B2	Panama	3	Complete Genome	3215
JX869999	B	B2	Panama	3	Complete Genome	3215
JX507200	B	B2	Panama	4	94-1122	1029
JX507202	B	B2	Panama	4	84-1110	1027
JX507207	B	B2	Panama	4	247-1112	866
JX507208	B	B2	Panama	4	139-1110	972

JX507209	B	B2	Panama	4	200-1121	922
JX507211	C	C1	Panama	5	Complete Genome	3215
JX507212	C	C1	Panama	5	Complete Genome	3215
JX507214	C	C1	Panama	5	Complete Genome	3215
JX870000	C	C1	Panama	5	Complete Genome	3215
JX870001	B	B2	Panama	5	Complete Genome	3215
JX507203	C	C1	Panama	6	257-1110	854
JX507204	C	C1	Panama	6	79-989	911
JX507205	C	C1	Panama	6	145-1036	892
JX507206	C	C1	Panama	6	248-1114	867
KJ638676	D	D4	Panama	7	276-1123	848
KJ638656	F	F1c	Panama	8	Complete Genome	3215
KJ638657	F	F1c	Panama	8	Complete Genome	3215
KJ638658	F	F1c	Panama	8	Complete Genome	3215
KJ638659	F	F3	Panama	8	Complete Genome	3215
KJ638660	F	F5	Panama	8	Complete Genome	3215
KJ638662	F	F5	Panama	8	Complete Genome	3215
KJ638663	F	F1c	Panama	8	Complete Genome	3215
KJ638664	F	F1c	Panama	8	Complete Genome	3212
KP718103	F	F3	Panama	8	Complete Genome	3215
KP718105	F	F3	Panama	8	Complete Genome	3215
KP718106	F	F3	Panama	8	Complete Genome	3215
KP718108	F	F3	Panama	8	Complete Genome	3215
KP718109	F	F3	Panama	8	Complete Genome	3215
KP718111	F	F3	Panama	8	Complete Genome	3215
KP718112	F	F3	Panama	8	Complete Genome	3215
KJ638666	F	F1c	Panama	9	276-1122	847
KJ638667	F	F3	Panama	9	276-1123	848
KJ638669	F	F3	Panama	9	276-1122	847
KJ638672	F	F1c	Panama	9	276-1123	848
KJ638673	F	F5	Panama	9	276-1124	849
KJ638674	F	F3	Panama	9	276-1123	848
KJ638675	F	F1c	Panama	9	276-1123	848
KJ638678	F	F3	Panama	9	276-1123	848
KJ638679	F	F5	Panama	9	276-1123	848
KP718063	F	F3	Panama	9	246-1122	877
KP718067	F	F3	Panama	9	246-1110	865

[1]Alignment to complete genome reference sequence VHB NC_003977.2

MEXICO

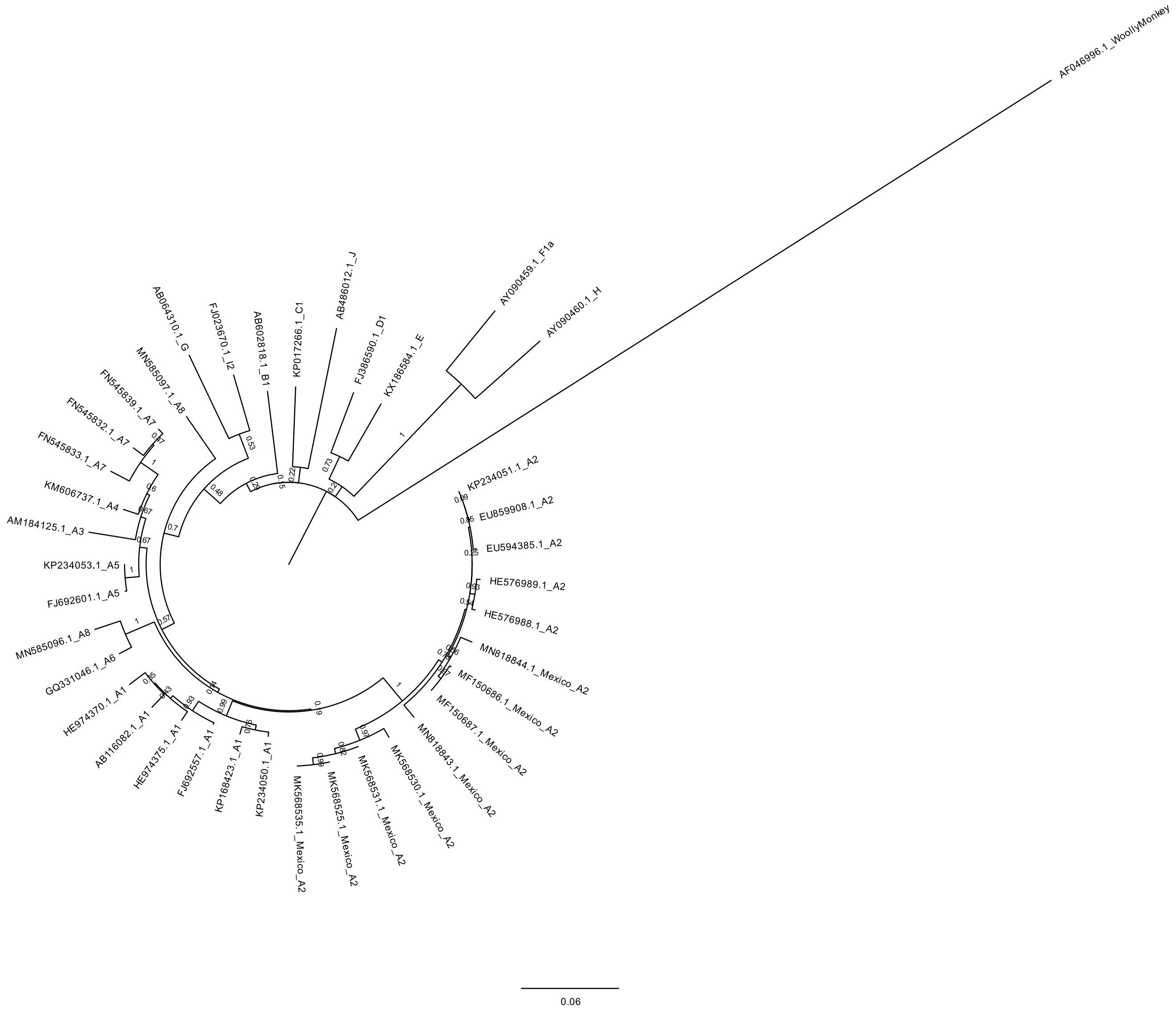

Tree 1. The evolutionary history was inferred by using the Maximum Likelihood method and Tamura-Nei model. The percentage of replicate trees in which the associated taxa clustered together in the bootstrap test (1000 replicates) are shown next to the branches. Initial tree(s) for the heuristic search were obtained automatically by applying Neighbor-Join and BioNJ algorithms to a matrix of pairwise distances estimated using the Tamura-Nei model, and then selecting the topology with superior log likelihood value. A discrete Gamma distribution was used to model evolutionary rate differences among sites (5 categories (+*G*, parameter = 0.1842)). The tree is drawn to scale, with branch lengths measured in the number of substitutions per site. The analysis involved 39 nucleotide sequences, of which 31 were used as marker sequences to determine the genotype of 8 sequences. All positions containing gaps and missing data were eliminated. There was a total of 1156 positions in the final dataset. Evolutionary analyses were conducted in MEGA X.

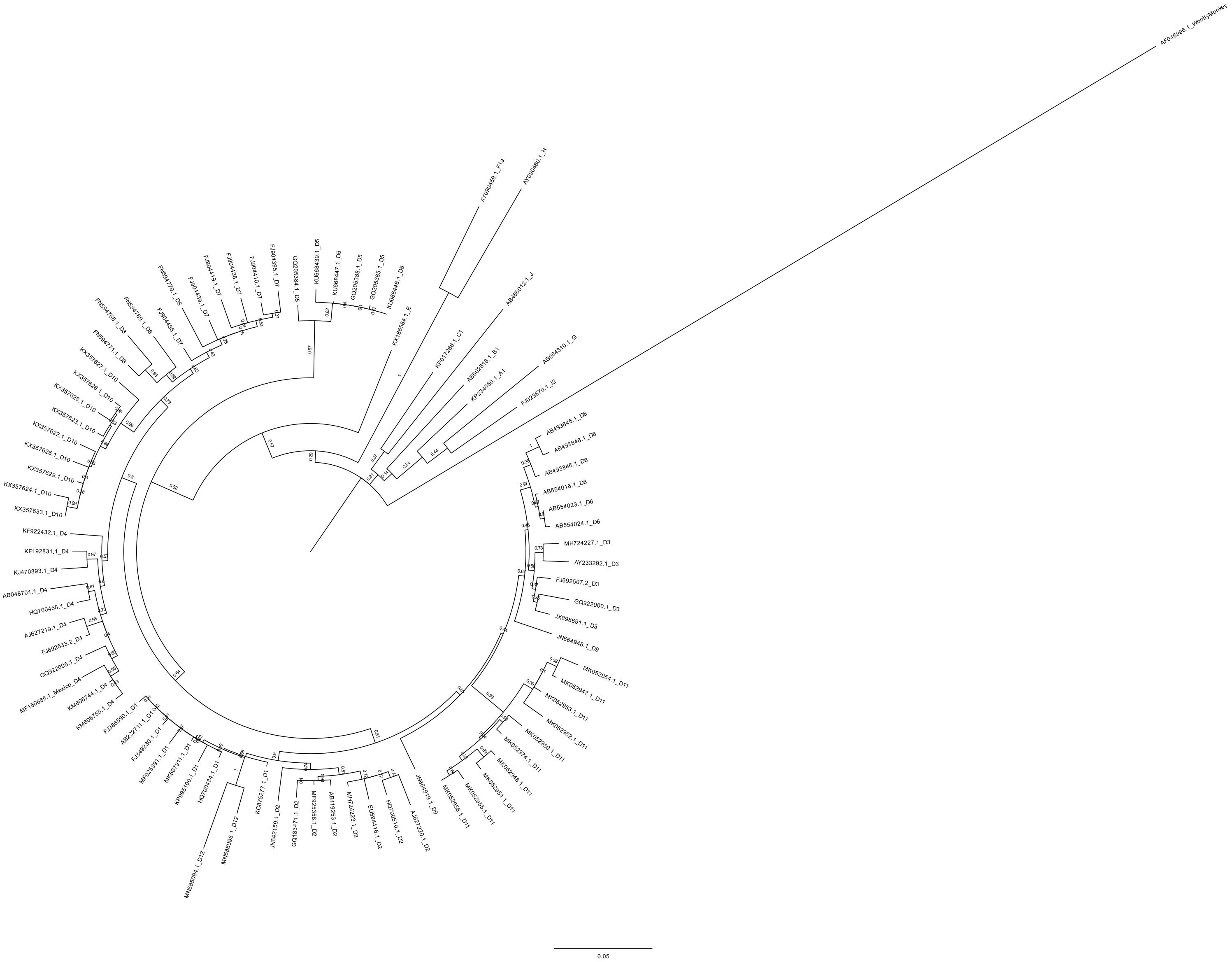

Tree 2. The evolutionary history was inferred by using the Maximum Likelihood method and Tamura-Nei model. The percentage of replicate trees in which the associated taxa clustered together in the bootstrap test (1000 replicates) are shown next to the branches. Initial tree(s) for the heuristic search were obtained automatically by applying Neighbor-Join and BioNJ algorithms to a matrix of pairwise distances estimated using the Tamura-Nei model, and then selecting the topology with superior log likelihood value. A discrete Gamma distribution was used to model evolutionary rate differences among sites (5 categories (+*G*, parameter = 0.2448)). The tree is drawn to scale, with branch lengths measured in the number of substitutions per site. The analysis involved 87 nucleotide sequences, of which 86 were used as marker sequences to determine the genotype of 1 sequence. All positions containing gaps and missing data were eliminated. There was a total of 1194 positions in the final dataset. Evolutionary analyses were conducted in MEGA X.

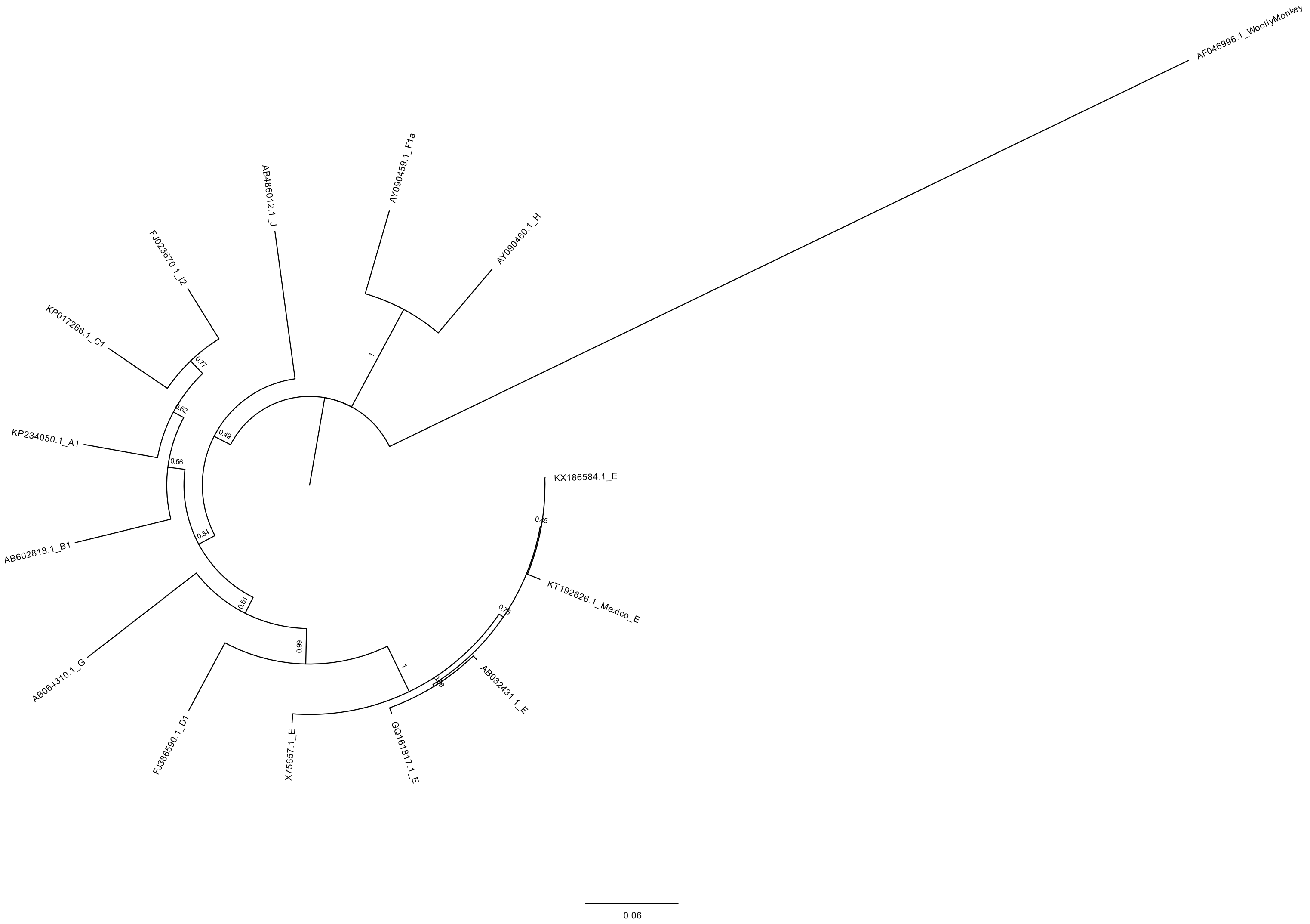

Tree 3. The evolutionary history was inferred by using the Maximum Likelihood method and Tamura-Nei model. The percentage of replicate trees in which the associated taxa clustered together in the bootstrap test (1000 replicates) are shown next to the branches. Initial tree(s) for the heuristic search were obtained automatically by applying Neighbor-Join and BioNJ algorithms to a matrix of pairwise distances estimated using the Tamura-Nei model, and then selecting the topology with superior log likelihood value. A discrete Gamma distribution was used to model evolutionary rate differences among sites (5 categories (+*G*, parameter = 0.2249)). The tree is drawn to scale, with branch lengths measured in the number of substitutions per site. The analysis involved 15 nucleotide sequences, of which 14 were used as marker sequences to determine the genotype of 1 sequence. All positions containing gaps and missing data were eliminated. There was a total of 3171 positions in the final dataset. Evolutionary analyses were conducted in MEGA X.

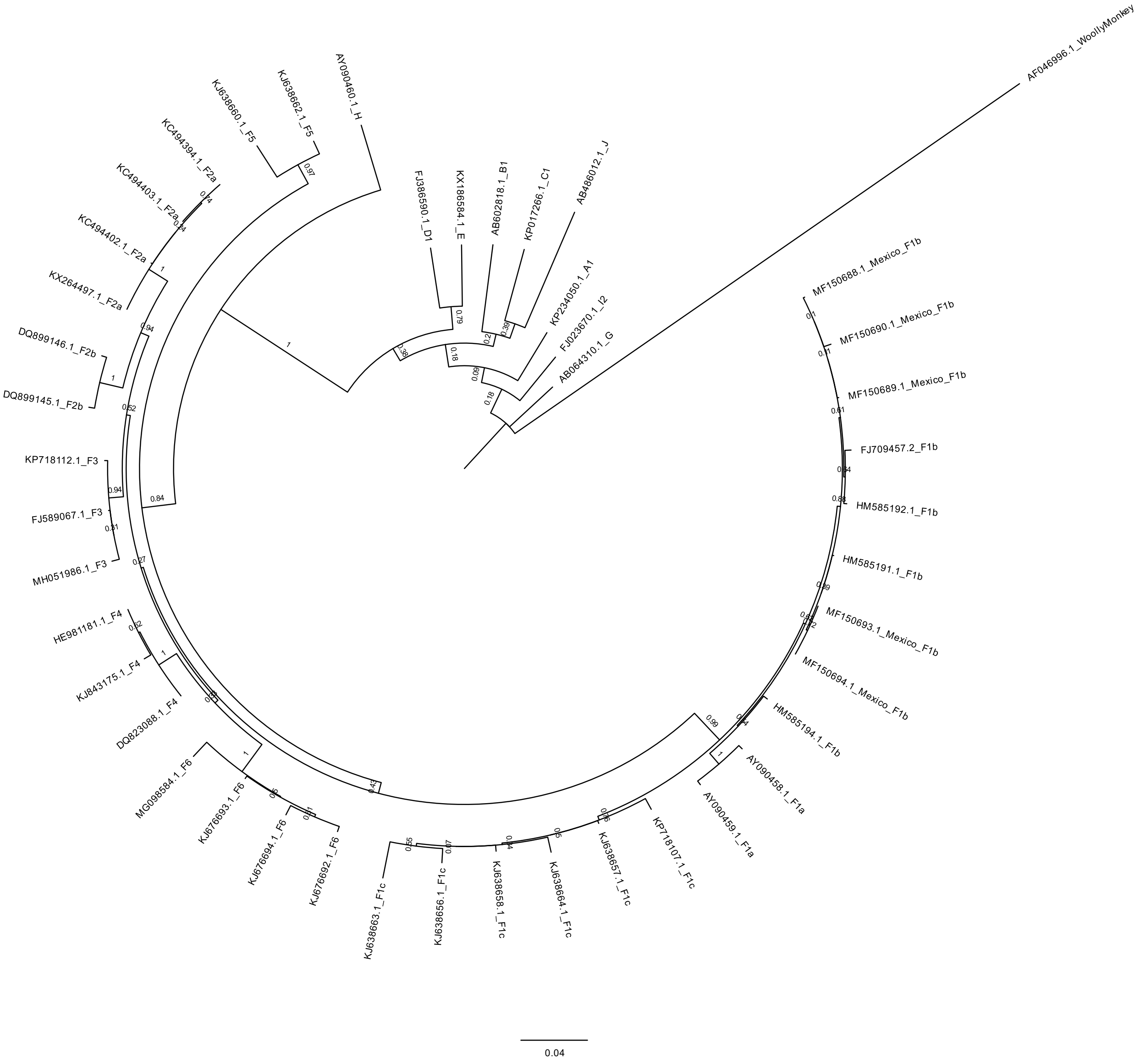

Tree 4. The evolutionary history was inferred by using the Maximum Likelihood method and Tamura-Nei model. The percentage of replicate trees in which the associated taxa clustered together in the bootstrap test (1000 replicates) are shown next to the branches. Initial tree(s) for the heuristic search were obtained automatically by applying Neighbor-Join and BioNJ algorithms to a matrix of pairwise distances estimated using the Tamura-Nei model, and then selecting the topology with superior log likelihood value. A discrete Gamma distribution was used to model evolutionary rate differences among sites (5 categories (+*G*, parameter = 0.2168)). The tree is drawn to scale, with branch lengths measured in the number of substitutions per site. The analysis involved 45 nucleotide sequences, of which 40 were used as marker sequences to determine the genotype of 5 sequences. All positions containing gaps and missing data were eliminated. There was a total of 1208 positions in the final dataset. Evolutionary analyses were conducted in MEGA X.

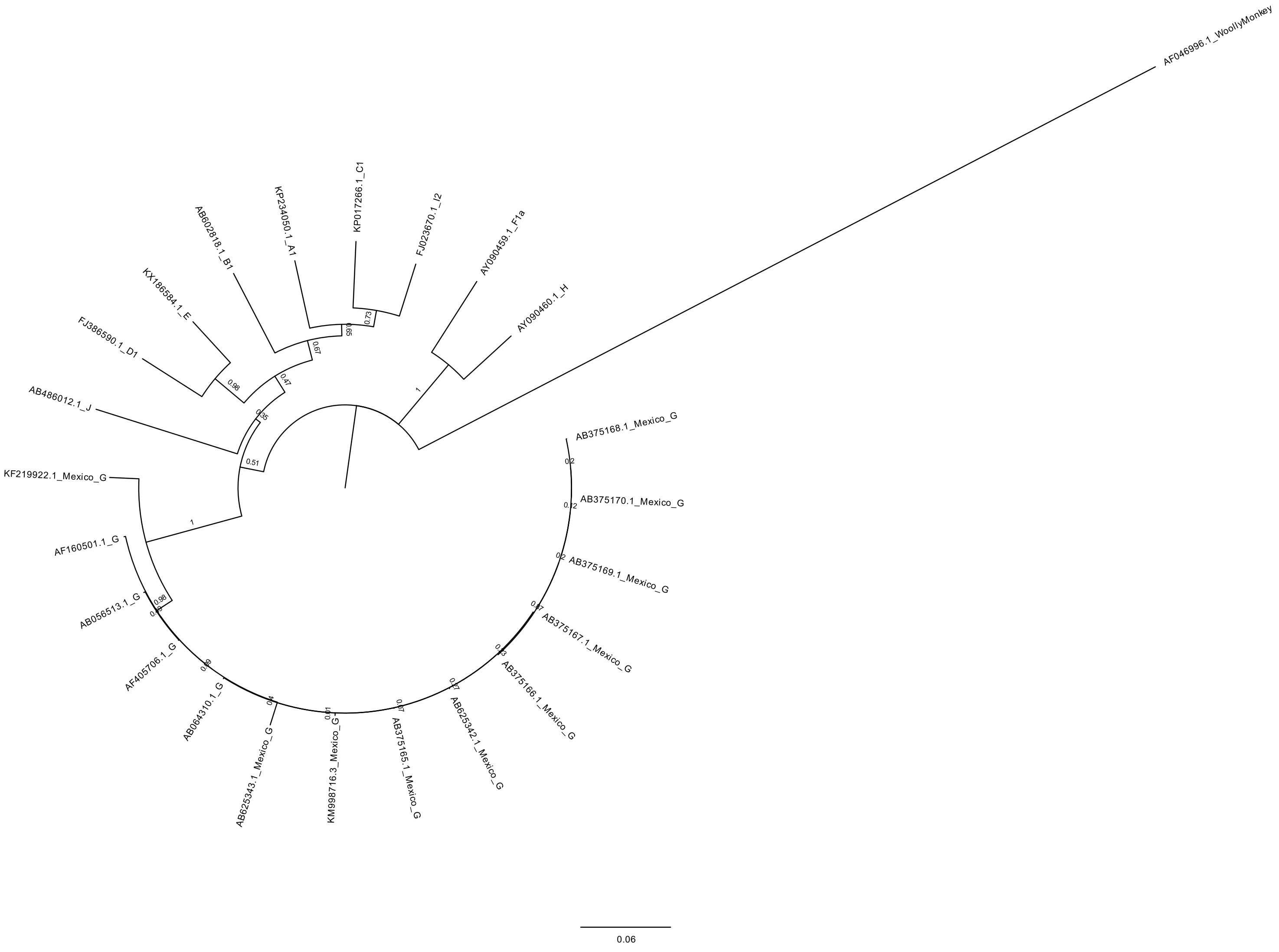

Tree 5. The evolutionary history was inferred by using the Maximum Likelihood method and Tamura-Nei model. The percentage of replicate trees in which the associated taxa clustered together in the bootstrap test (1000 replicates) are shown next to the branches. Initial tree(s) for the heuristic search were obtained automatically by applying Neighbor-Join and BioNJ algorithms to a matrix of pairwise distances estimated using the Tamura-Nei model, and then selecting the topology with superior log likelihood value. A discrete Gamma distribution was used to model evolutionary rate differences among sites (5 categories (+*G*, parameter = 0.2111)). The tree is drawn to scale, with branch lengths measured in the number of substitutions per site. The analysis involved 24 nucleotide sequences, of which 14 were used as marker sequences to determine the genotype of 10 sequences. All positions containing gaps and missing data were eliminated. There was a total of 3038 positions in the final dataset. Evolutionary analyses were conducted in MEGA X.

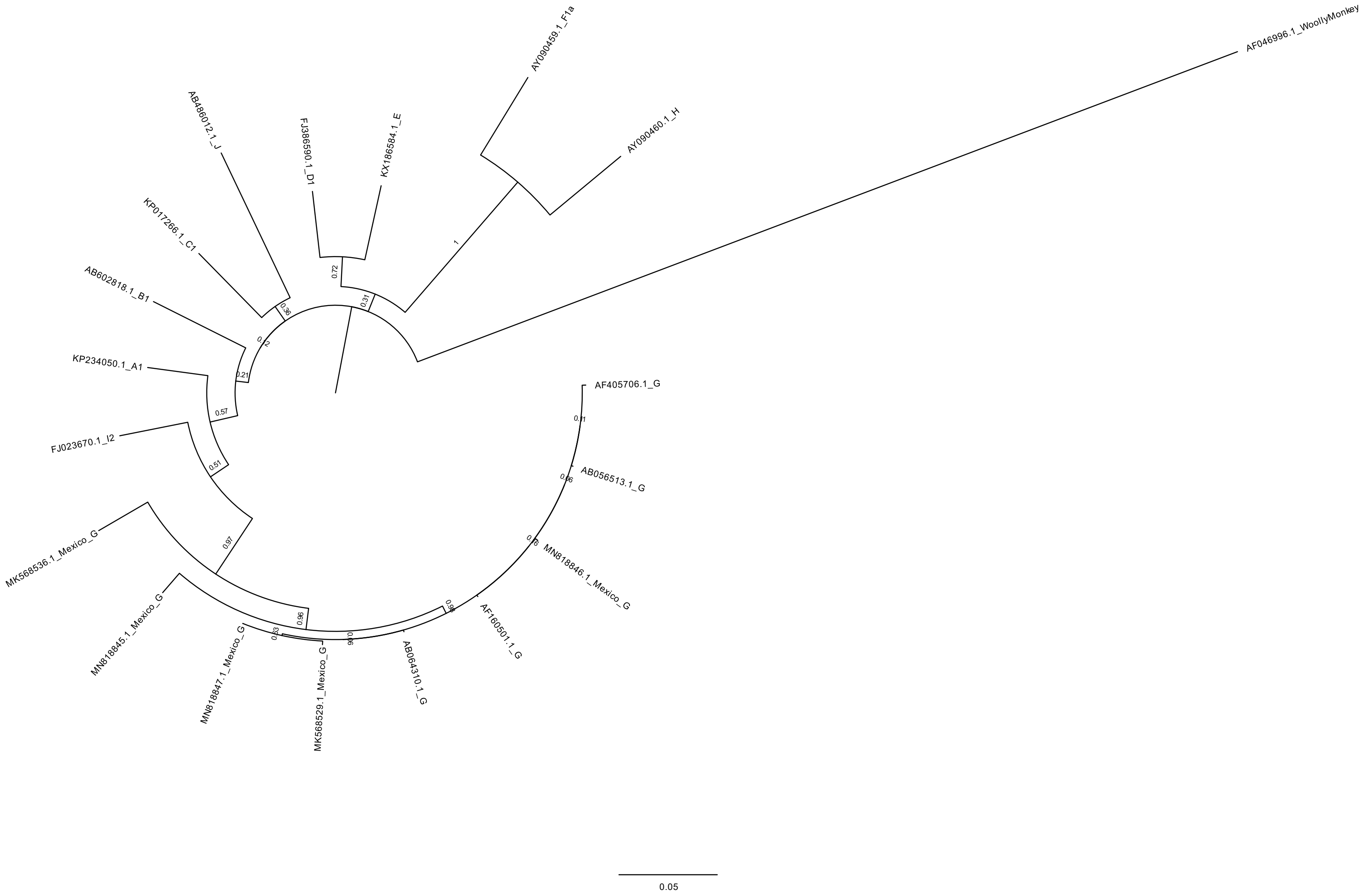

Tree 6. The evolutionary history was inferred by using the Maximum Likelihood method and Tamura-Nei model. The percentage of replicate trees in which the associated taxa clustered together in the bootstrap test (1000 replicates) are shown next to the branches. Initial tree(s) for the heuristic search were obtained automatically by applying Neighbor-Join and BioNJ algorithms to a matrix of pairwise distances estimated using the Tamura-Nei model, and then selecting the topology with superior log likelihood value. A discrete Gamma distribution was used to model evolutionary rate differences among sites (5 categories (+*G*, parameter = 0.1928)). The tree is drawn to scale, with branch lengths measured in the number of substitutions per site. The analysis involved 19 nucleotide sequences, of which 14 were used as marker sequences to determine the genotype of 5 sequences. All positions containing gaps and missing data were eliminated. There was a total of 1146 positions in the final dataset. Evolutionary analyses were conducted in MEGA X.

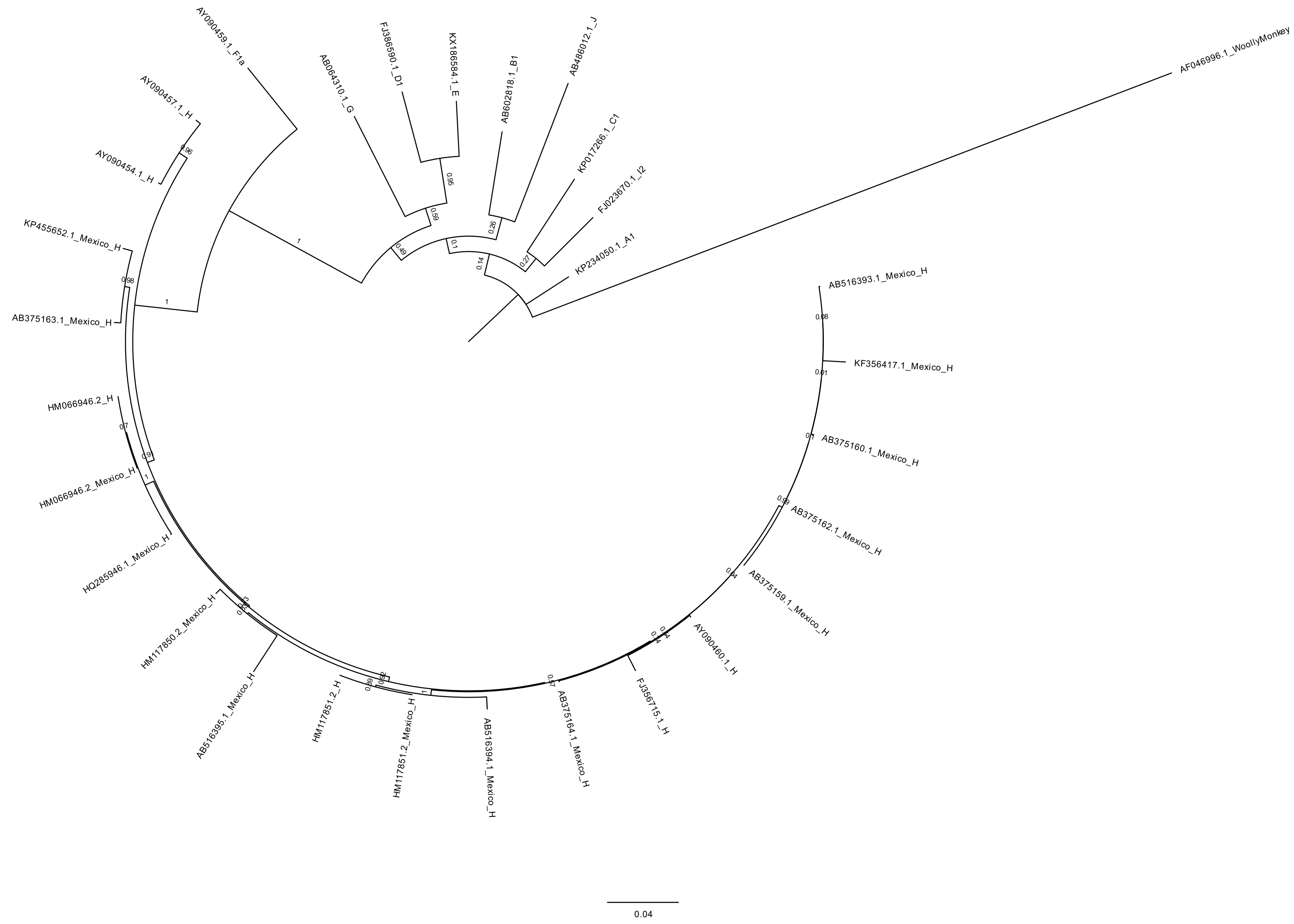

Tree 7. The evolutionary history was inferred by using the Maximum Likelihood method and Tamura-Nei model. The percentage of replicate trees in which the associated taxa clustered together in the bootstrap test (1000 replicates) are shown next to the branches. Initial tree(s) for the heuristic search were obtained automatically by applying Neighbor-Join and BioNJ algorithms to a matrix of pairwise distances estimated using the Tamura-Nei model, and then selecting the topology with superior log likelihood value. A discrete Gamma distribution was used to model evolutionary rate differences among sites (5 categories (+*G*, parameter = 0.2745)). The tree is drawn to scale, with branch lengths measured in the number of substitutions per site. The analysis involved 30 nucleotide sequences, of which 16 were used as marker sequences to determine the genotype of 14 sequences. All positions containing gaps and missing data were eliminated. There was a total of 1960 positions in the final dataset. Evolutionary analyses were conducted in MEGA X.

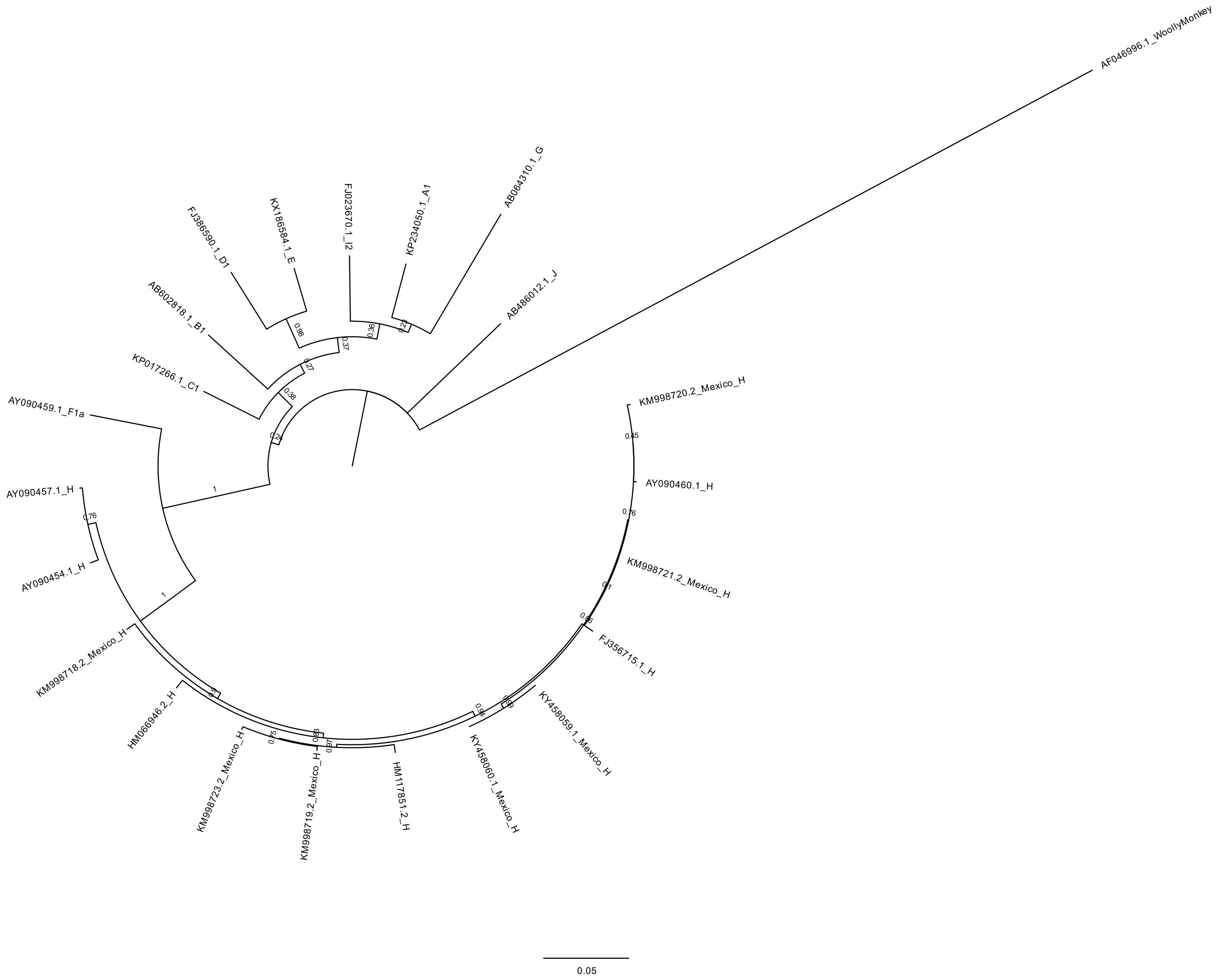

Tree 8. The evolutionary history was inferred by using the Maximum Likelihood method and Tamura-Nei model. The percentage of replicate trees in which the associated taxa clustered together in the bootstrap test (1000 replicates) are shown next to the branches. Initial tree(s) for the heuristic search were obtained automatically by applying Neighbor-Join and BioNJ algorithms to a matrix of pairwise distances estimated using the Tamura-Nei model, and then selecting the topology with superior log likelihood value. A discrete Gamma distribution was used to model evolutionary rate differences among sites (5 categories (+*G*, parameter = 0.2047)). The tree is drawn to scale, with branch lengths measured in the number of substitutions per site. The analysis involved 23 nucleotide sequences, of which 16 were used as marker sequences to determine the genotype of 7 sequences. All positions containing gaps and missing data were eliminated. There was a total of 1835 positions in the final dataset. Evolutionary analyses were conducted in MEGA X.

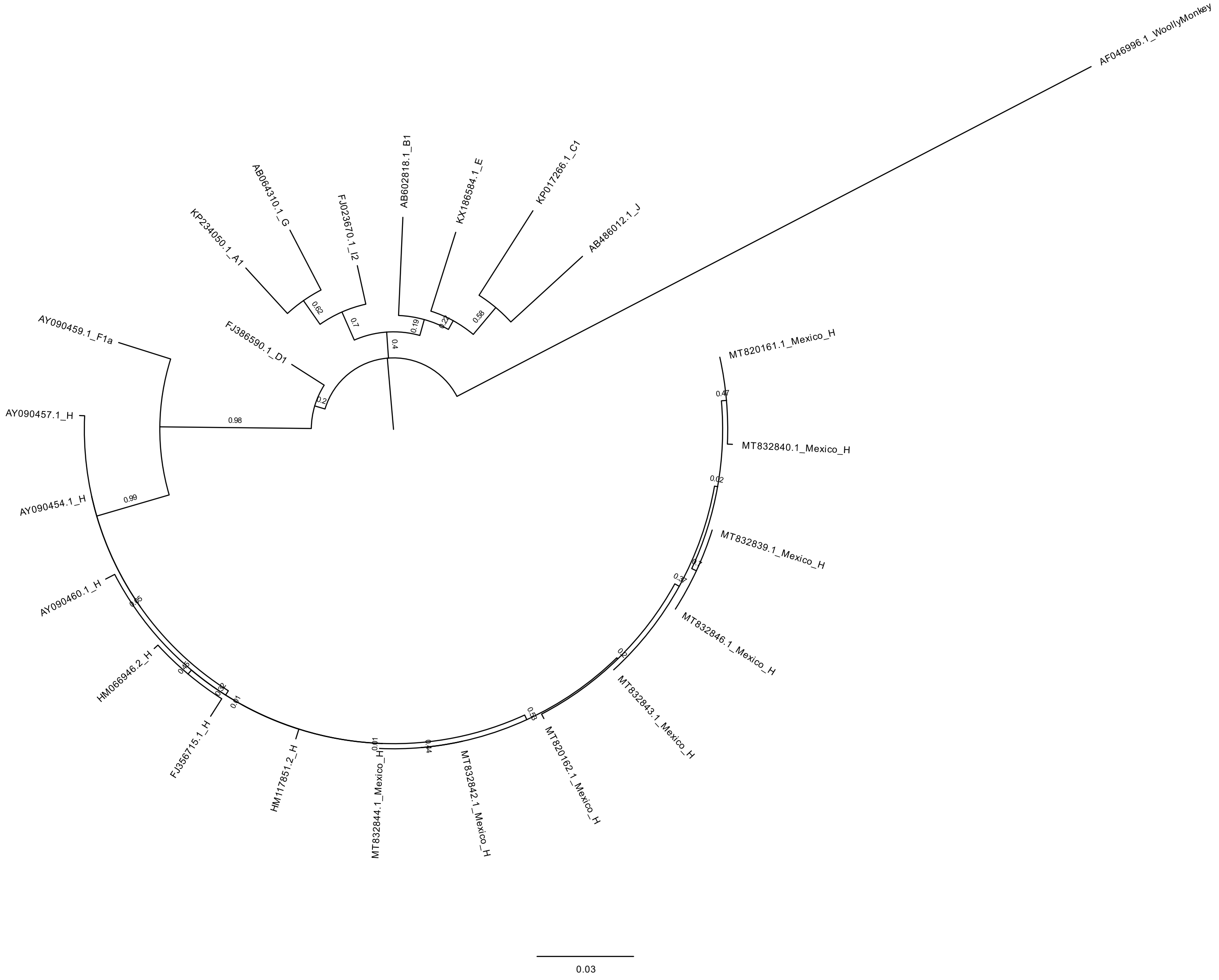

Tree 9. The evolutionary history was inferred by using the Maximum Likelihood method and Tamura-Nei model. The percentage of replicate trees in which the associated taxa clustered together in the bootstrap test (1000 replicates) are shown next to the branches. Initial tree(s) for the heuristic search were obtained automatically by applying Neighbor-Join and BioNJ algorithms to a matrix of pairwise distances estimated using the Tamura-Nei model, and then selecting the topology with superior log likelihood value. A discrete Gamma distribution was used to model evolutionary rate differences among sites (5 categories (+*G*, parameter = 0.1358)). The tree is drawn to scale, with branch lengths measured in the number of substitutions per site. The analysis involved 24 nucleotide sequences, of which 16 were used as marker sequences to determine the genotype of 8 sequences. All positions containing gaps and missing data were eliminated. There was a total of 681 positions in the final dataset. Evolutionary analyses were conducted in MEGA X.

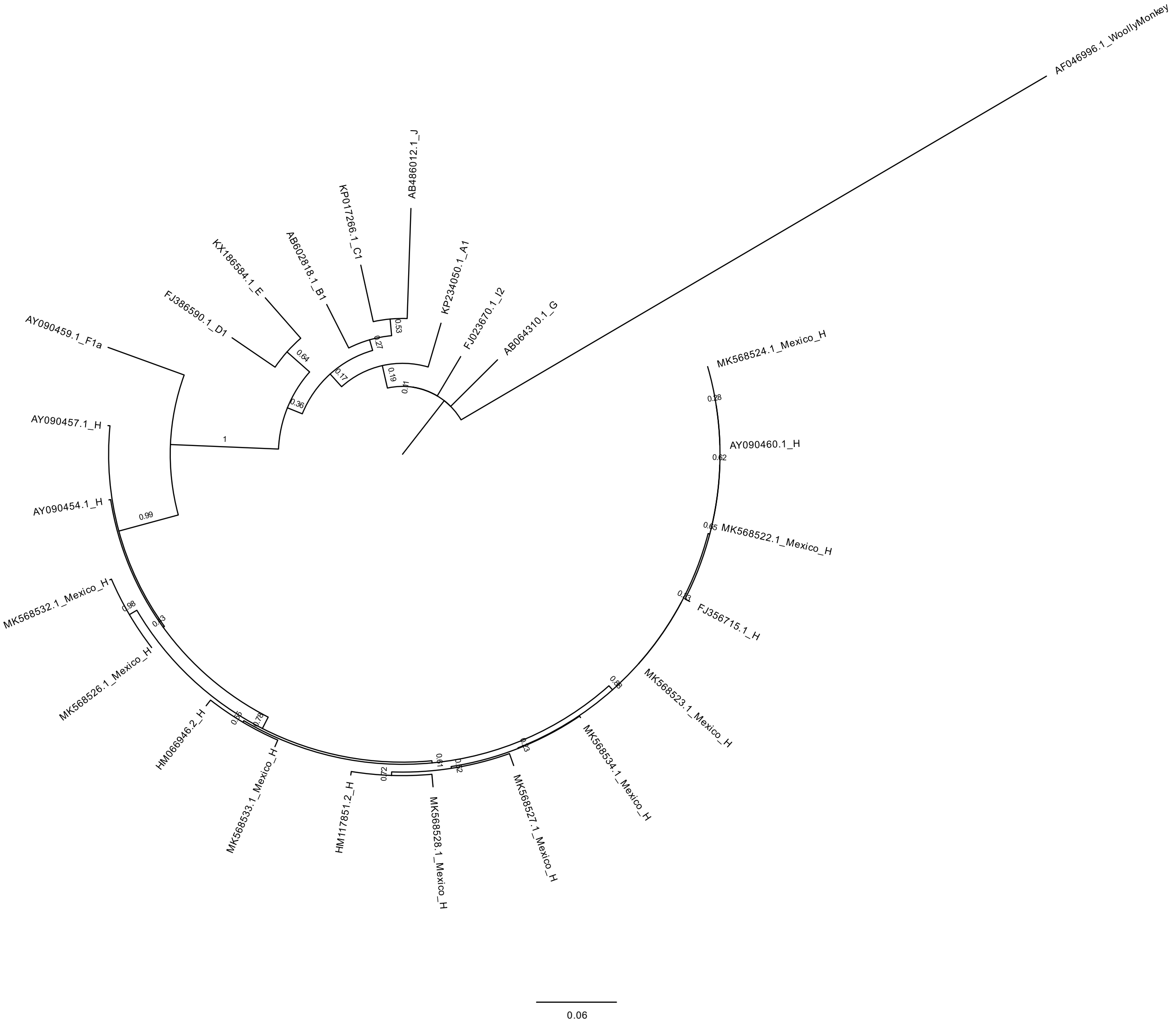

Tree 10. The evolutionary history was inferred by using the Maximum Likelihood method and Tamura-Nei model. The percentage of replicate trees in which the associated taxa clustered together in the bootstrap test (1000 replicates) are shown next to the branches. Initial tree(s) for the heuristic search were obtained automatically by applying Neighbor-Join and BioNJ algorithms to a matrix of pairwise distances estimated using the Tamura-Nei model, and then selecting the topology with superior log likelihood value. A discrete Gamma distribution was used to model evolutionary rate differences among sites (5 categories (+*G*, parameter = 0.1870)). The tree is drawn to scale, with branch lengths measured in the number of substitutions per site. The analysis involved 25 nucleotide sequences, of which 16 were used as marker sequences to determine the genotype of 9 sequences. All positions containing gaps and missing data were eliminated. There was a total of 823 positions in the final dataset. Evolutionary analyses were conducted in MEGA X.

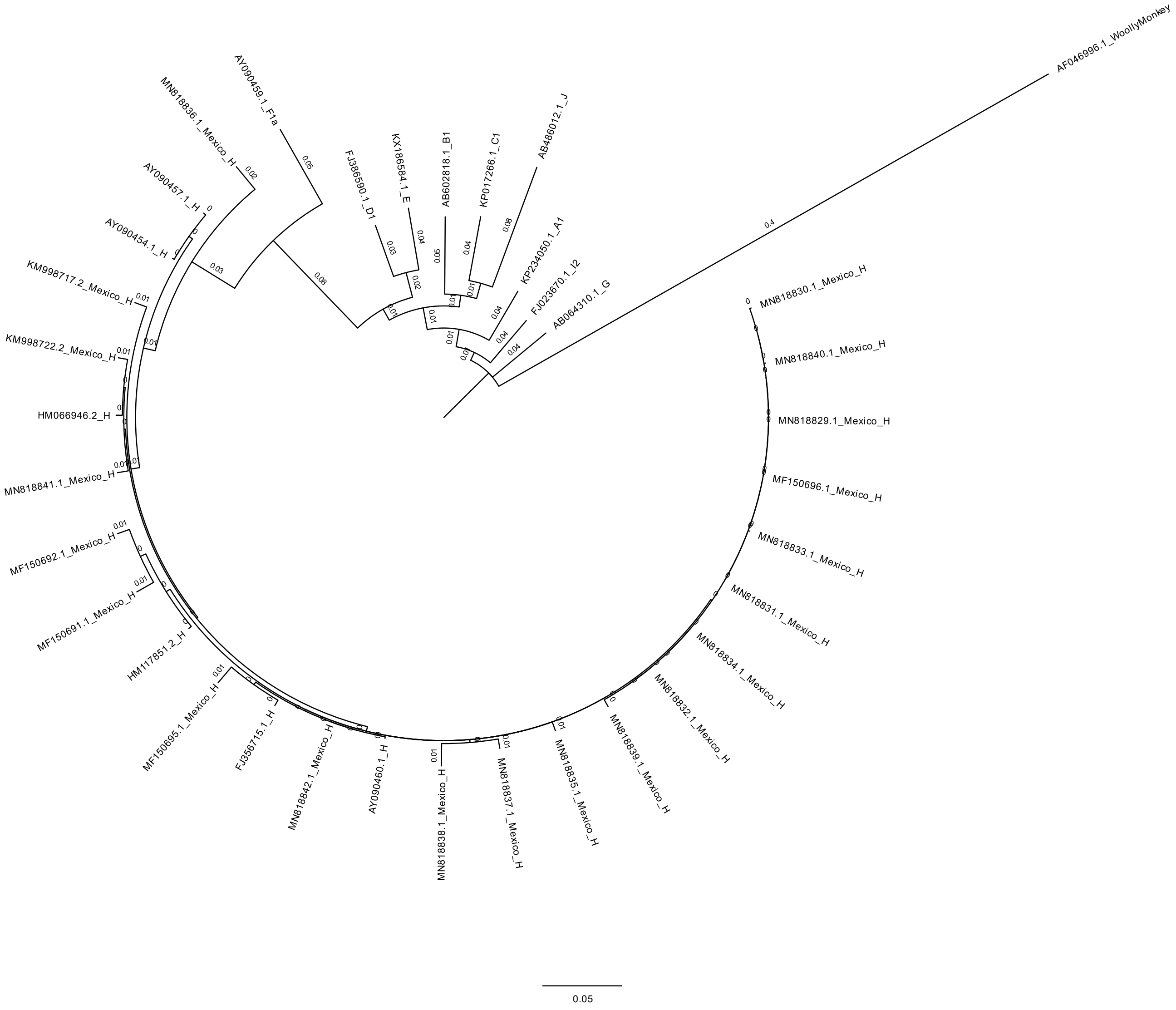

Tree 11. The evolutionary history was inferred by using the Maximum Likelihood method and Tamura-Nei model. The percentage of replicate trees in which the associated taxa clustered together in the bootstrap test (1000 replicates) are shown next to the branches. Initial tree(s) for the heuristic search were obtained automatically by applying Neighbor-Join and BioNJ algorithms to a matrix of pairwise distances estimated using the Tamura-Nei model, and then selecting the topology with superior log likelihood value. A discrete Gamma distribution was used to model evolutionary rate differences among sites (5 categories (+*G*, parameter = 0.2157)). The tree is drawn to scale, with branch lengths measured in the number of substitutions per site. The analysis involved 36 nucleotide sequences, of which 16 were used as marker sequences to determine the genotype of 20 sequences. All positions containing gaps and missing data were eliminated. There was a total of 1097 positions in the final dataset. Evolutionary analyses were conducted in MEGA X.

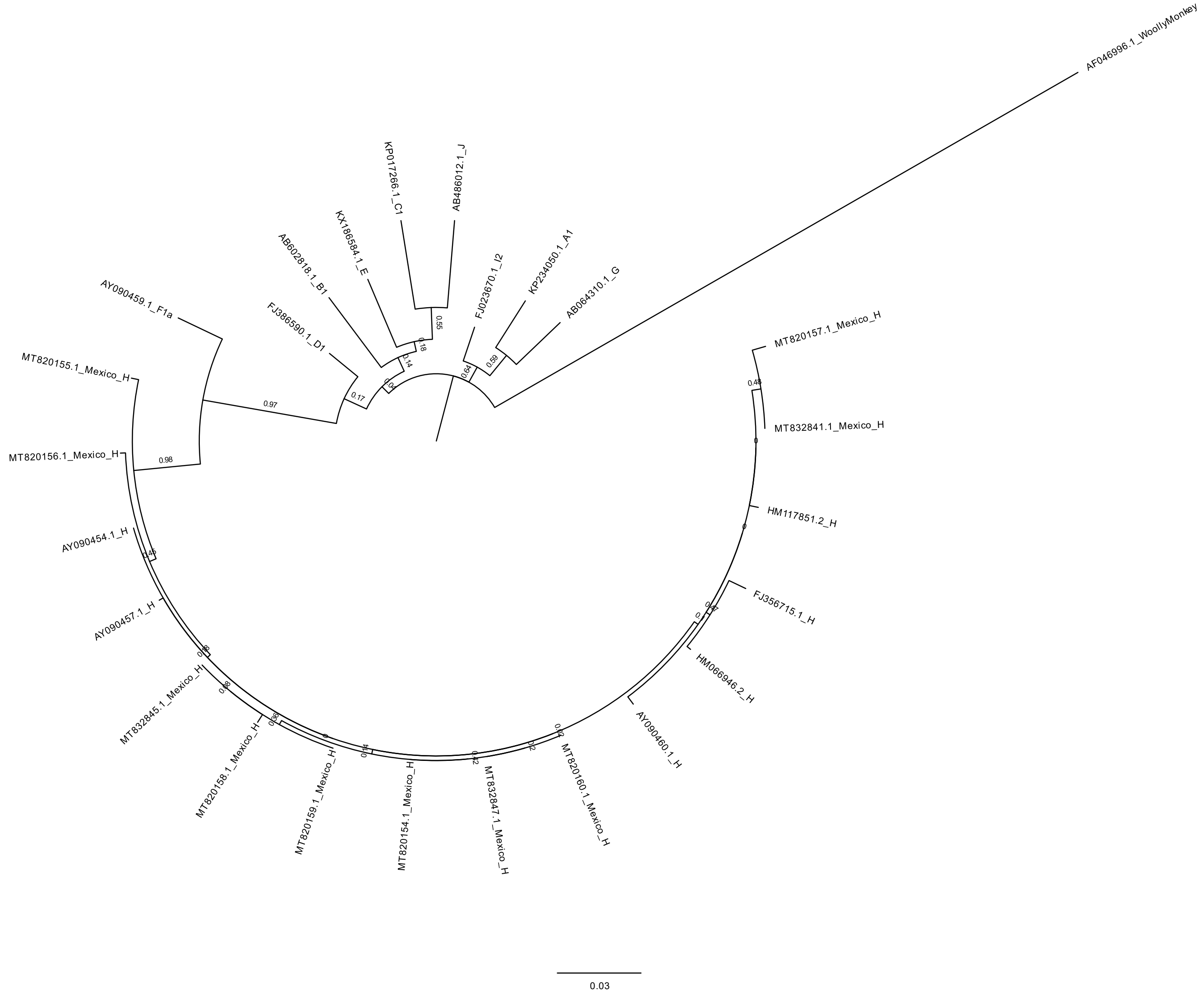

Tree 12. The evolutionary history was inferred by using the Maximum Likelihood method and Tamura-Nei model. The percentage of replicate trees in which the associated taxa clustered together in the bootstrap test (1000 replicates) are shown next to the branches. Initial tree(s) for the heuristic search were obtained automatically by applying Neighbor-Join and BioNJ algorithms to a matrix of pairwise distances estimated using the Tamura-Nei model, and then selecting the topology with superior log likelihood value. A discrete Gamma distribution was used to model evolutionary rate differences among sites (5 categories (+*G*, parameter = 0.1364)). The tree is drawn to scale, with branch lengths measured in the number of substitutions per site. The analysis involved 26 nucleotide sequences, of which 16 were used as marker sequences to determine the genotype of 10 sequences. All positions containing gaps and missing data were eliminated. There was a total of 678 positions in the final dataset. Evolutionary analyses were conducted in MEGA X.

ID	GENOTYPE	SUBTYPE	COUNTRY	TREE	ALIGNMENT[1]	BASE PAIRS
MF150686	A	A2	Mexico	1	21-1251	1231
MF150687	A	A2	Mexico	1	21-1251	1231
MK568525	A	A2	Mexico	1	1-3182	1304
MK568530	A	A2	Mexico	1	1-3182	1304
MK568531	A	A2	Mexico	1	1-3182	1251
MK568535	A	A2	Mexico	1	1-3182	1304
MN818843	A	A2	Mexico	1	21-1251	1231
MN818844	A	A2	Mexico	1	21-1251	1231
MF150685	D	D4	Mexico	2	21-1251	1231

KT192626	E	E	Mexico	3	Complete Genome	3212
MF150688	F	F1b	Mexico	4	21-1251	1231
MF150689	F	F1b	Mexico	4	21-1251	1231
MF150690	F	F1b	Mexico	4	21-1251	1231
MF150693	F	F1b	Mexico	4	21-1251	1231
MF150694	F	F1b	Mexico	4	21-1251	1231
AB375165	G	G	Mexico	5	Complete Genome	3248
AB375166	G	G	Mexico	5	Complete Genome	3248
AB375167	G	G	Mexico	5	Complete Genome	3248
AB375168	G	G	Mexico	5	Complete Genome	3248
AB375169	G	G	Mexico	5	Complete Genome	3248
AB375170	G	G	Mexico	5	Complete Genome	3248
AB625342	G	G	Mexico	5	Complete Genome	3248
AB625343	G	G	Mexico	5	Complete Genome	3179
KF219922	G	G	Mexico	5	Complete Genome	3248
KM998716	G	G	Mexico	5	Complete Genome	3248
MK568529	G	G	Mexico	6	3-1245	1197
MK568536	G	G	Mexico	6	33-1285	1253
MN818845	G	G	Mexico	6	21-1251	1231
MN818846	G	G	Mexico	6	21-1251	1231
MN818847	G	G	Mexico	6	21-1251	1231
AB375159	H	H	Mexico	7	Complete Genome	3215
AB375160	H	H	Mexico	7	Complete Genome	3215
AB375162	H	H	Mexico	7	Complete Genome	3215
AB375163	H	H	Mexico	7	Complete Genome	3215
AB375164	H	H	Mexico	7	Complete Genome	3215
AB516393	H	H	Mexico	7	Complete Genome	3215
AB516394	H	H	Mexico	7	Complete Genome	3215
HM066946	H	H	Mexico	7	Complete Genome	3215
HM117850	H	H	Mexico	7	Complete Genome	3215
HM117851	H	H	Mexico	7	Complete Genome	3215
HQ285946	H	H	Mexico	7	Complete Genome	3215
KF356417	H	H	Mexico	7	Complete Genome	3187
KP455652	H	H	Mexico	7	Complete Genome	2809
AB516395	H	H	Mexico	7	Complete Genome	3215
KM998718	H	H	Mexico	8	78-1922	1845
KM998719	H	H	Mexico	8	78-1925	1848
KM998720	H	H	Mexico	8	78-1927	1850
KM998721	H	H	Mexico	8	79-1927	1849
KM998723	H	H	Mexico	8	78-1922	1845
KY458059	H	H	Mexico	8	78-1922	1845
KY458060	H	H	Mexico	8	78-1924	1847
MT820161	H	H	Mexico	9	157-837	681
MT820162	H	H	Mexico	9	157-837	681
MT832839	H	H	Mexico	9	157-837	681
MT832840	H	H	Mexico	9	157-837	681

MT832842	H	H	Mexico	9	157-837	681
MT832843	H	H	Mexico	9	157-837	681
MT832844	H	H	Mexico	9	157-837	681
MT832846	H	H	Mexico	9	157-837	681
MK568522	H	H	Mexico	10	1-3182	1304
MK568523	H	H	Mexico	10	1-3182	1304
MK568524	H	H	Mexico	10	1-3182	1304
MK568526	H	H	Mexico	10	1-3182	1304
MK568527	H	H	Mexico	10	1-3182	1304
MK568528	H	H	Mexico	10	414-1285	872
MK568532	H	H	Mexico	10	1-3182	1304
MK568533	H	H	Mexico	10	1-3182	1257
MK568534	H	H	Mexico	10	1-3182	1304
MF150691	H	H	Mexico	11	21-1251	1231
MF150692	H	H	Mexico	11	21-1251	1231
MF150695	H	H	Mexico	11	21-1251	1231
MF150696	H	H	Mexico	11	21-1251	1231
MN818829	H	H	Mexico	11	21-1251	1231
MN818830	H	H	Mexico	11	21-1251	1231
MN818831	H	H	Mexico	11	21-1251	1231
MN818832	H	H	Mexico	11	21-1251	1231
MN818833	H	H	Mexico	11	21-1251	1231
MN818834	H	H	Mexico	11	21-1251	1231
MN818835	H	H	Mexico	11	21-1251	1231
MN818836	H	H	Mexico	11	21-1251	1231
MN818837	H	H	Mexico	11	21-1251	1231
MN818838	H	H	Mexico	11	21-1251	1231
MN818839	H	H	Mexico	11	21-1251	1231
MN818840	H	H	Mexico	11	21-1251	1231
MN818841	H	H	Mexico	11	21-1251	1231
MN818842	H	H	Mexico	11	21-1251	1231
KM998717	H	H	Mexico	11	78-1177	1100
KM998722	H	H	Mexico	11	78-1177	1100
MT820154	H	H	Mexico	12	157-837	681
MT820155	H	H	Mexico	12	157-837	681
MT820156	H	H	Mexico	12	157-837	681
MT820157	H	H	Mexico	12	157-837	681
MT820158	H	H	Mexico	12	157-837	681
MT820159	H	H	Mexico	12	157-837	681
MT820160	H	H	Mexico	12	157-837	681
MT832841	H	H	Mexico	12	157-837	681
MT832845	H	H	Mexico	12	157-837	681
MT832847	H	H	Mexico	12	157-837	681

[1]Alignment to complete genome reference sequence VHB NC_003977.2

HAITI

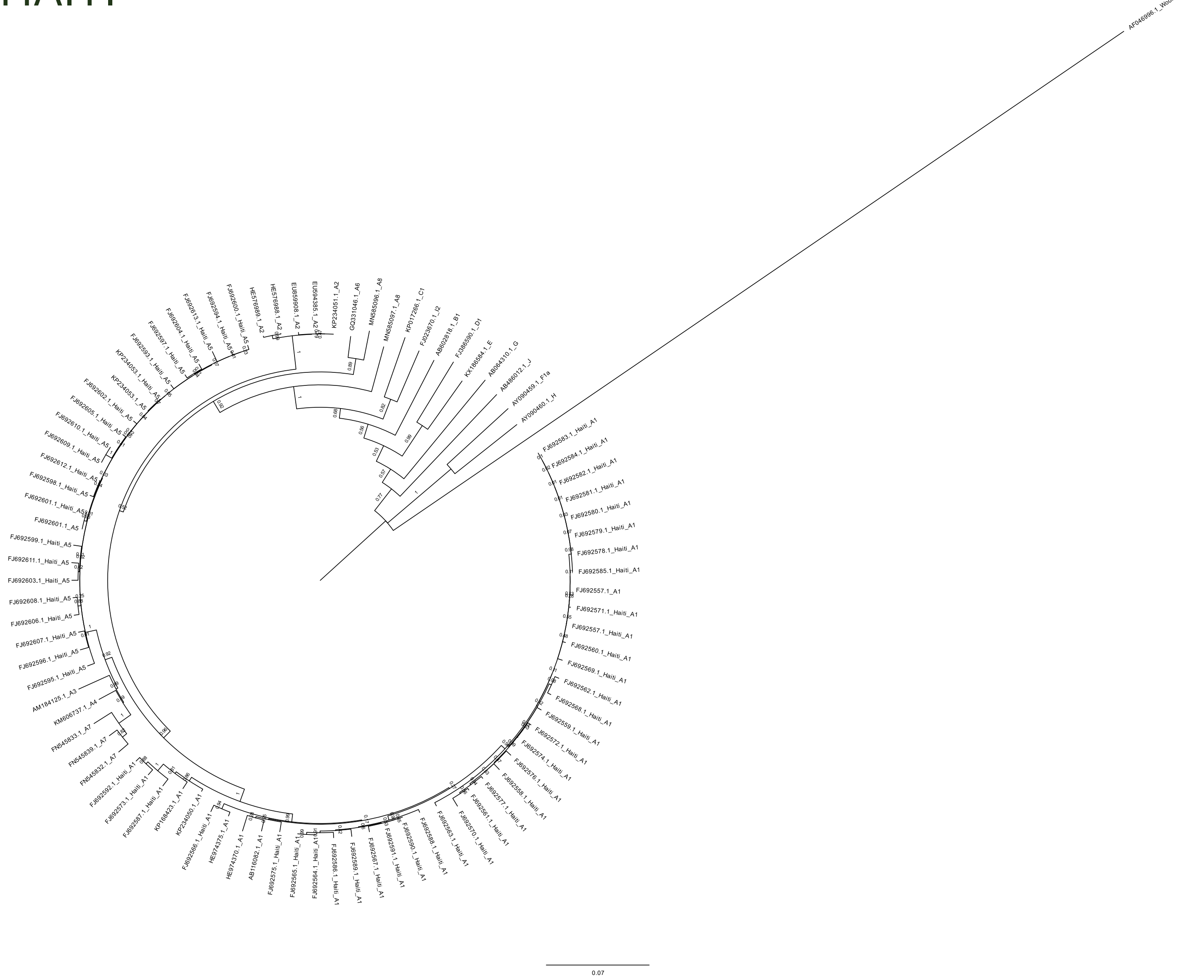

Tree 1. The evolutionary history was inferred by using the Maximum Likelihood method and Tamura-Nei model. The percentage of replicate trees in which the associated taxa clustered together in the bootstrap test (1000 replicates) are shown next to the branches. Initial tree(s) for the heuristic search were obtained automatically by applying Neighbor-Join and BioNJ algorithms to a matrix of pairwise distances estimated using the Tamura-Nei model, and then selecting the topology with superior log likelihood value. A discrete Gamma distribution was used to model evolutionary rate differences among sites (5 categories (+*G*, parameter = 0.2176)). The tree is drawn to scale, with branch lengths measured in the number of substitutions per site. The analysis involved 89 nucleotide sequences, of which 31 were used as marker sequences to determine the genotype of 58 sequences. All positions containing gaps and missing data were eliminated. There was a total of 2791 positions in the final dataset. Evolutionary analyses were conducted in MEGA X.

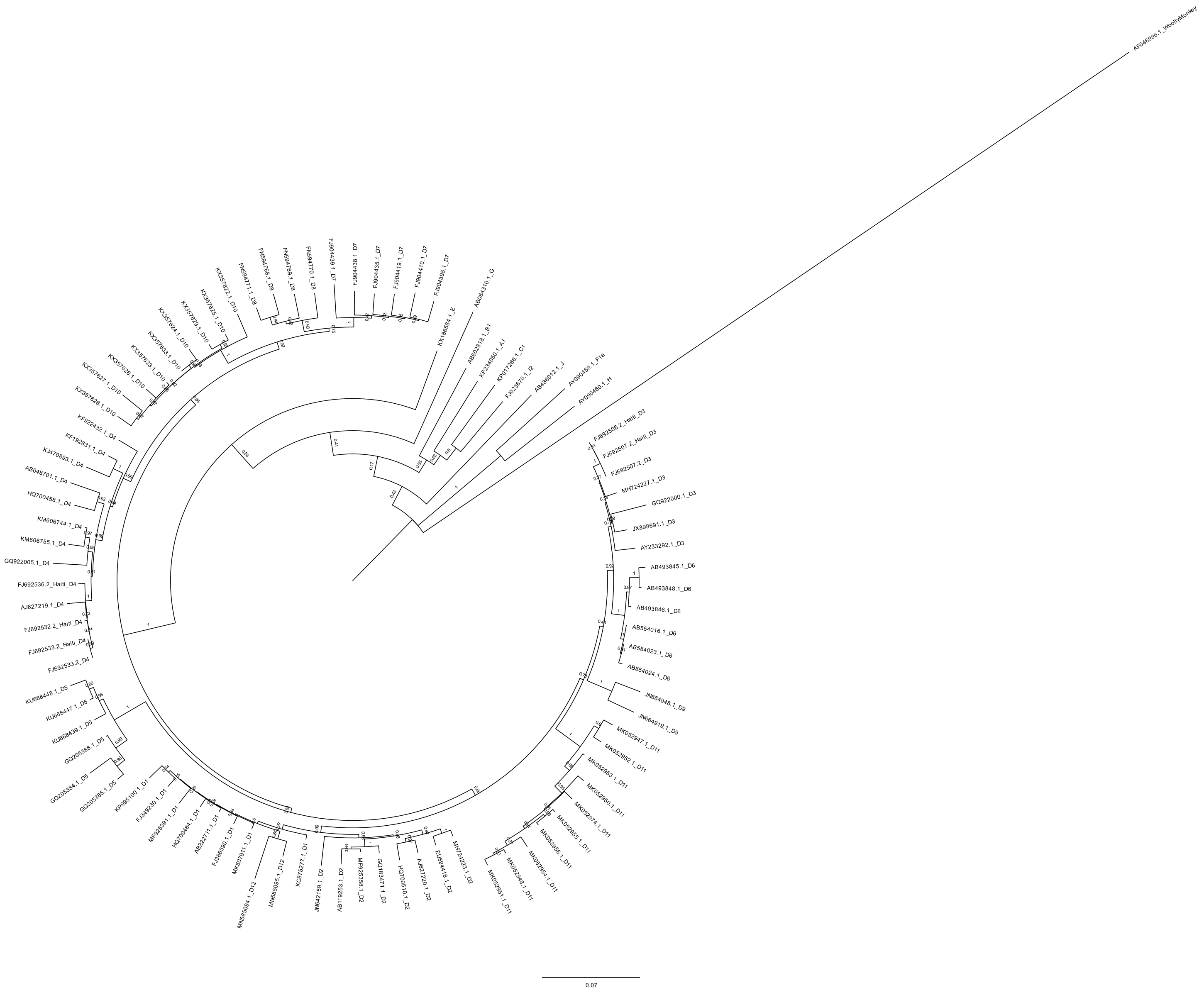

Tree 2. The evolutionary history was inferred by using the Maximum Likelihood method and Tamura-Nei model. The percentage of replicate trees in which the associated taxa clustered together in the bootstrap test (1000 replicates) are shown next to the branches. Initial tree(s) for the heuristic search were obtained automatically by applying Neighbor-Join and BioNJ algorithms to a matrix of pairwise distances estimated using the Tamura-Nei model, and then selecting the topology with superior log likelihood value. A discrete Gamma distribution was used to model evolutionary rate differences among sites (5 categories (+*G*, parameter = 0.2767)). The tree is drawn to scale, with branch lengths measured in the number of substitutions per site. The analysis involved 91 nucleotide sequences, of which 86 were used as marker sequences to determine the genotype of 5 sequences. All positions containing gaps and missing data were eliminated. There was a total of 3086 positions in the final dataset. Evolutionary analyses were conducted in MEGA X.

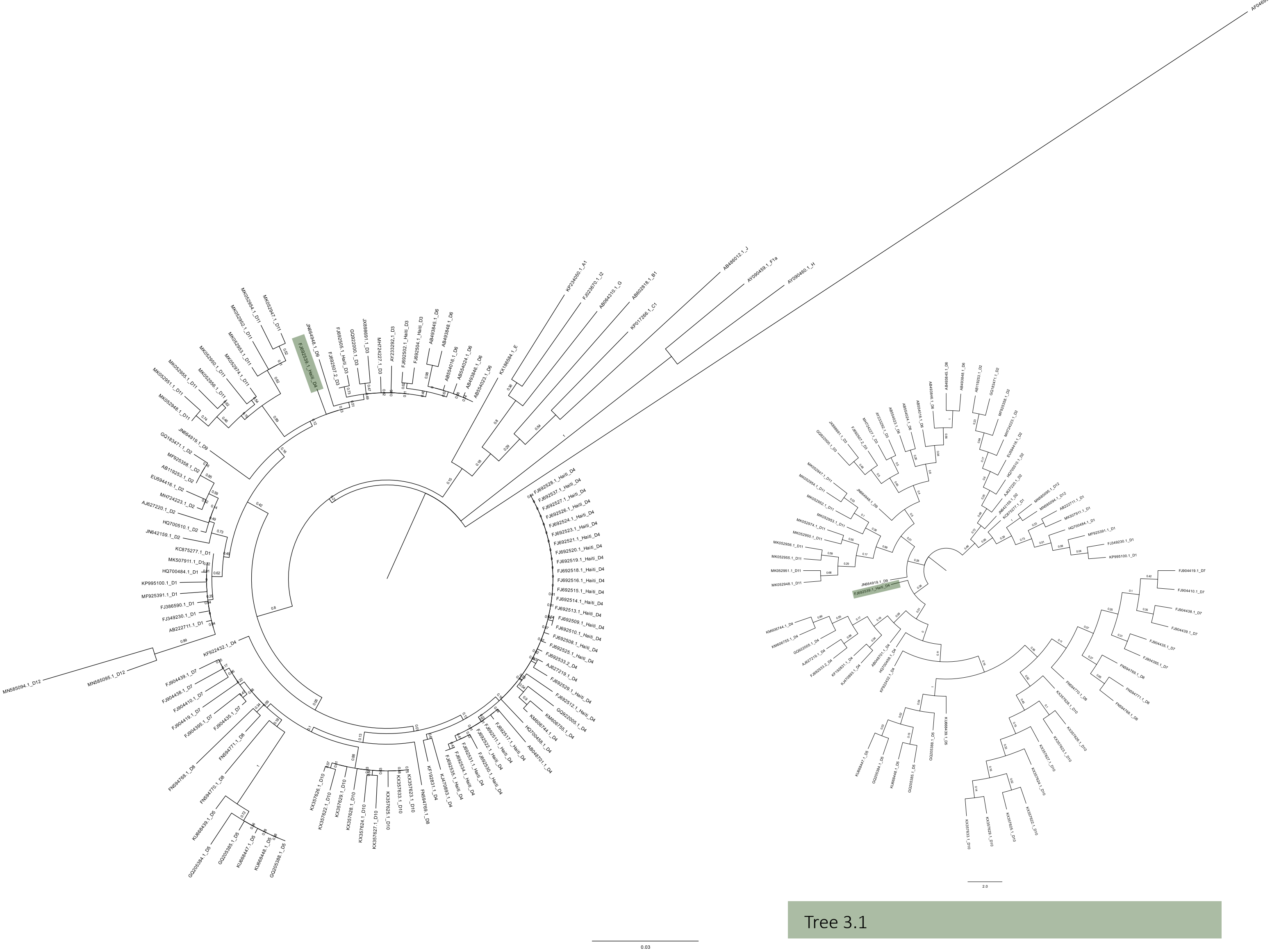

Tree 3. The evolutionary history was inferred by using the Maximum Likelihood method and Tamura-Nei model. The percentage of replicate trees in which the associated taxa clustered together in the bootstrap test (1000 replicates) are shown next to the branches. Initial tree(s) for the heuristic search were obtained automatically by applying Neighbor-Join and BioNJ algorithms to a matrix of pairwise distances estimated using the Tamura-Nei model, and then selecting the topology with superior log likelihood value. A discrete Gamma distribution was used to model evolutionary rate differences among sites (5 categories (+*G*, parameter = 0.2635)). The tree is drawn to scale, with branch lengths measured in the number of substitutions per site. The analysis involved 117 nucleotide sequences, of which 86 were used as marker sequences to determine the genotype of 31 sequences. Although sequence FJ692539 showed an uncertain placement in the initial tree, upon further analysis (Tree 3.1) it was determined that it was subtype D4. All positions containing gaps and missing data were eliminated. There was a total of 810 positions in the final dataset. Evolutionary analyses were conducted in MEGA X.

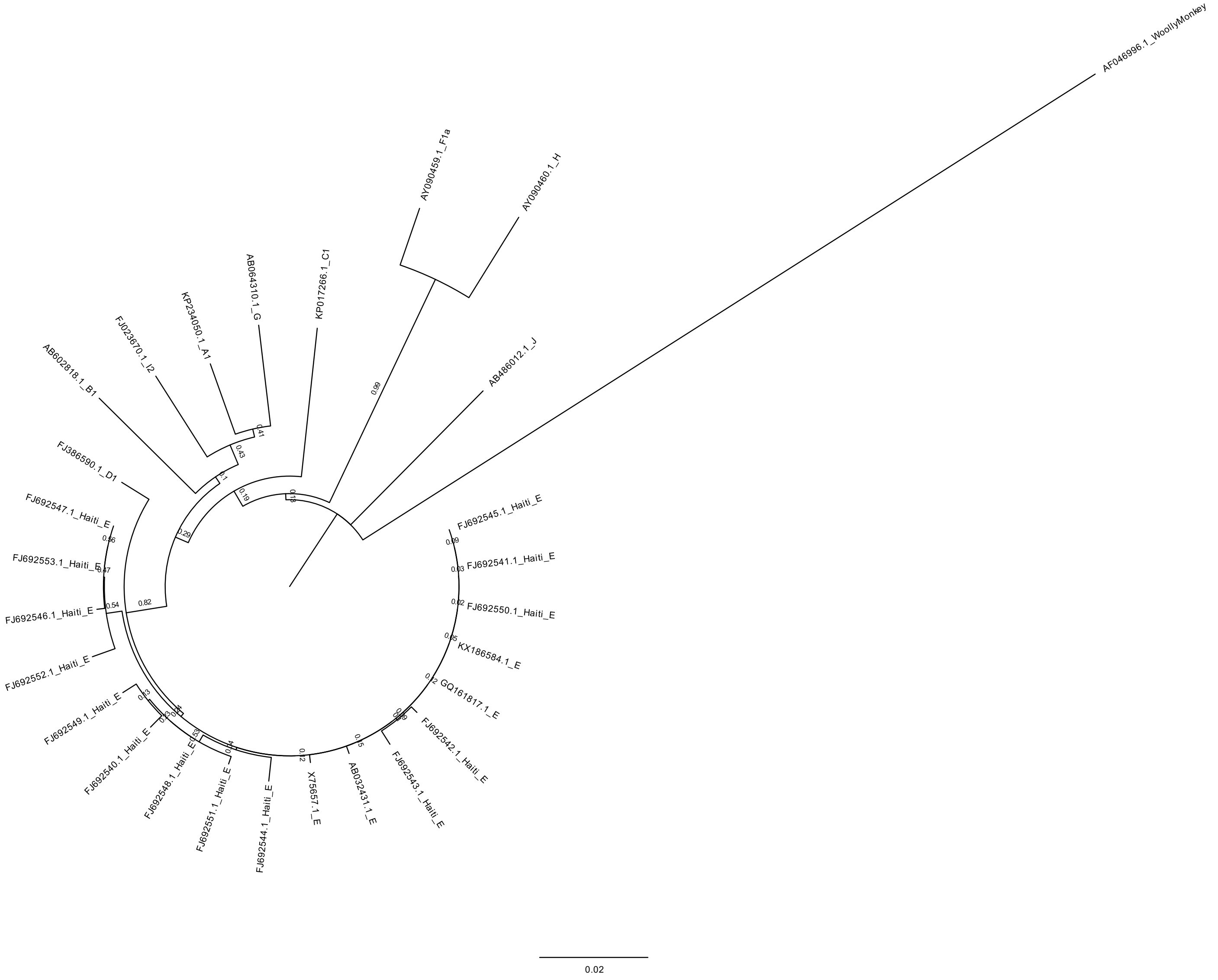

Tree 4. The evolutionary history was inferred by using the Maximum Likelihood method and Tamura-Nei model. The percentage of replicate trees in which the associated taxa clustered together in the bootstrap test (1000 replicates) are shown next to the branches. Initial tree(s) for the heuristic search were obtained automatically by applying Neighbor-Join and BioNJ algorithms to a matrix of pairwise distances estimated using the Tamura-Nei model, and then selecting the topology with superior log likelihood value. A discrete Gamma distribution was used to model evolutionary rate differences among sites (5 categories (+*G*, parameter = 0.1166)). The tree is drawn to scale, with branch lengths measured in the number of substitutions per site. The analysis involved 28 nucleotide sequences, of which 14 were used as marker sequences to determine the genotype of 14 sequences. All positions containing gaps and missing data were eliminated. There was a total of 701 positions in the final dataset. Evolutionary analyses were conducted in MEGA X.

ID	GENOTYPE	SUBTYPE	COUNTRY	TREE	ALIGNMENT[1]	BASE PAIRS
FJ692557	A	A1	Haiti	1	Complete Genome	3221
FJ692558	A	A1	Haiti	1	Complete Genome	3221
FJ692559	A	A1	Haiti	1	Complete Genome	3221
FJ692560	A	A1	Haiti	1	Complete Genome	3220
FJ692561	A	A1	Haiti	1	Complete Genome	3221
FJ692562	A	A1	Haiti	1	Complete Genome	3221

FJ692563	A	A1	Haiti	1	Complete Genome	3221
FJ692564	A	A1	Haiti	1	Complete Genome	3221
FJ692565	A	A1	Haiti	1	Complete Genome	3221
FJ692566	A	A1	Haiti	1	Complete Genome	3221
FJ692567	A	A1	Haiti	1	Complete Genome	3221
FJ692568	A	A1	Haiti	1	Complete Genome	3213
FJ692569	A	A1	Haiti	1	Complete Genome	3222
FJ692570	A	A1	Haiti	1	Complete Genome	3117
FJ692571	A	A1	Haiti	1	Complete Genome	3221
FJ692572	A	A1	Haiti	1	Complete Genome	3221
FJ692573	A	A1	Haiti	1	Complete Genome	3168
FJ692574	A	A1	Haiti	1	Complete Genome	3221
FJ692575	A	A1	Haiti	1	Complete Genome	3221
FJ692576	A	A1	Haiti	1	Complete Genome	3221
FJ692577	A	A1	Haiti	1	Complete Genome	3221
FJ692578	A	A1	Haiti	1	Complete Genome	3221
FJ692579	A	A1	Haiti	1	Complete Genome	3221
FJ692580	A	A1	Haiti	1	Complete Genome	3221
FJ692581	A	A1	Haiti	1	Complete Genome	3221
FJ692582	A	A1	Haiti	1	Complete Genome	3221
FJ692583	A	A1	Haiti	1	Complete Genome	3221
FJ692584	A	A1	Haiti	1	Complete Genome	3221
FJ692585	A	A1	Haiti	1	Complete Genome	3221
FJ692586	A	A1	Haiti	1	Complete Genome	3168
FJ692587	A	A1	Haiti	1	Complete Genome	3203
FJ692588	A	A1	Haiti	1	Complete Genome	3224
FJ692589	A	A1	Haiti	1	Complete Genome	3213
FJ692590	A	A1	Haiti	1	Complete Genome	3221
FJ692591	A	A1	Haiti	1	Complete Genome	3221
FJ692592	A	A1	Haiti	1	Complete Genome	3221
FJ692593	A	A5	Haiti	1	Complete Genome	3215
FJ692594	A	A5	Haiti	1	Complete Genome	3226
FJ692595	A	A5	Haiti	1	Complete Genome	3221
FJ692596	A	A5	Haiti	1	Complete Genome	3221
FJ692597	A	A5	Haiti	1	Complete Genome	3221
FJ692598	A	A5	Haiti	1	Complete Genome	3221
FJ692599	A	A5	Haiti	1	Complete Genome	3221
FJ692600	A	A5	Haiti	1	Complete Genome	3221
FJ692601	A	A5	Haiti	1	Complete Genome	3221
FJ692602	A	A5	Haiti	1	Complete Genome	3221
FJ692603	A	A5	Haiti	1	Complete Genome	3221
FJ692604	A	A5	Haiti	1	Complete Genome	3221
FJ692605	A	A5	Haiti	1	Complete Genome	3221
FJ692606	A	A5	Haiti	1	Complete Genome	3221
FJ692607	A	A5	Haiti	1	Complete Genome	3221
FJ692608	A	A5	Haiti	1	Complete Genome	3221

FJ692609	A	A5	Haiti	1	Complete Genome	3221
FJ692610	A	A5	Haiti	1	Complete Genome	3221
FJ692611	A	A5	Haiti	1	Complete Genome	3221
FJ692612	A	A5	Haiti	1	Complete Genome	3221
FJ692613	A	A5	Haiti	1	Complete Genome	3221
KP234053	A	A5	Haiti	1	Complete Genome	3221
FJ692532	D	D4	Haiti	2	Complete Genome	3182
FJ692533	D	D4	Haiti	2	Complete Genome	3182
FJ692536	D	D4	Haiti	2	Complete Genome	3182
FJ692506	D	D3	Haiti	2	Complete Genome	3182
FJ692507	D	D3	Haiti	2	Complete Genome	3182
FJ692508	D	D4	Haiti	3	70-910	841
FJ692509	D	D4	Haiti	3	70-910	841
FJ692510	D	D4	Haiti	3	70-910	841
FJ692511	D	D4	Haiti	3	70-910	841
FJ692512	D	D4	Haiti	3	70-910	841
FJ692513	D	D4	Haiti	3	70-910	841
FJ692514	D	D4	Haiti	3	70-910	841
FJ692515	D	D4	Haiti	3	70-910	841
FJ692516	D	D4	Haiti	3	70-910	841
FJ692517	D	D4	Haiti	3	70-910	841
FJ692518	D	D4	Haiti	3	70-910	841
FJ692519	D	D4	Haiti	3	70-910	841
FJ692520	D	D4	Haiti	3	70-910	841
FJ692521	D	D4	Haiti	3	70-910	841
FJ692522	D	D4	Haiti	3	70-910	841
FJ692523	D	D4	Haiti	3	70-910	841
FJ692524	D	D4	Haiti	3	70-910	841
FJ692525	D	D4	Haiti	3	70-910	841
FJ692526	D	D4	Haiti	3	70-910	841
FJ692527	D	D4	Haiti	3	70-910	841
FJ692528	D	D4	Haiti	3	70-910	841
FJ692529	D	D4	Haiti	3	70-910	841
FJ692530	D	D4	Haiti	3	70-906	837
FJ692531	D	D4	Haiti	3	70-910	841
FJ692534	D	D4	Haiti	3	70-910	841
FJ692535	D	D4	Haiti	3	70-910	841
FJ692537	D	D4	Haiti	3	70-910	841
FJ692539	D	D4	Haiti	3	70-910	841
FJ692502	D	D3	Haiti	3	70-910	841
FJ692504	D	D3	Haiti	3	70-910	841
FJ692505	D	D3	Haiti	3	70-910	841
FJ692540	E	E	Haiti	4	70-910	841
FJ692541	E	E	Haiti	4	70-910	841
FJ692542	E	E	Haiti	4	71-910	840
FJ692543	E	E	Haiti	4	70-880	811

FJ692544	E	E	Haiti	4	70-862	793
FJ692545	E	E	Haiti	4	70-910	841
FJ692546	E	E	Haiti	4	70-910	841
FJ692547	E	E	Haiti	4	70-910	841
FJ692548	E	E	Haiti	4	70-910	841
FJ692549	E	E	Haiti	4	70-910	840
FJ692550	E	E	Haiti	4	70-910	841
FJ692551	E	E	Haiti	4	70-910	841
FJ692552	E	E	Haiti	4	76-910	835
FJ692553	E	E	Haiti	4	106-910	805

[1]Alignment to complete genome reference sequence VHB NC_003977.2

CUBA

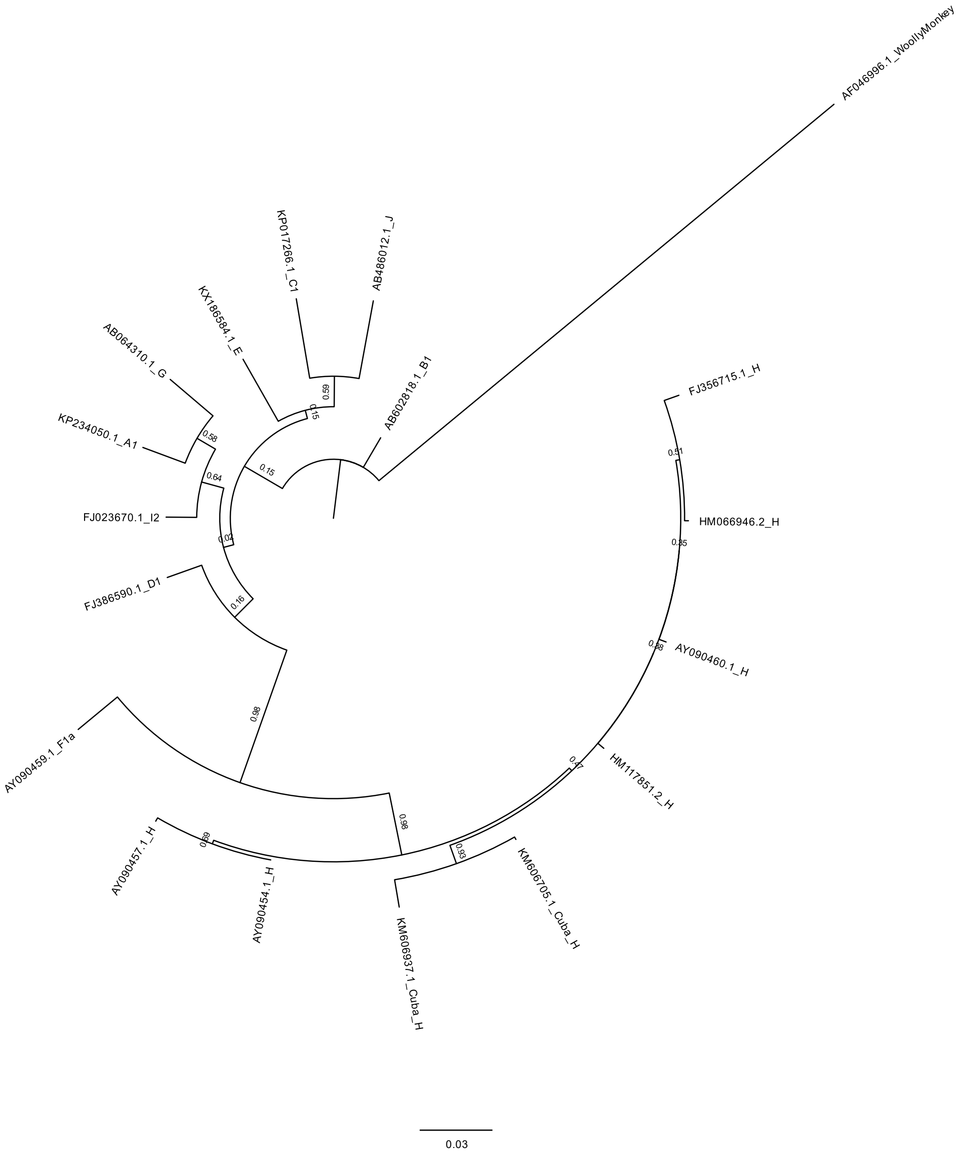

Tree 1. The evolutionary history was inferred by using the Maximum Likelihood method and Tamura-Nei model. The percentage of replicate trees in which the associated taxa clustered together in the bootstrap test (1000 replicates) are shown next to the branches. Initial tree(s) for the heuristic search were obtained automatically by applying Neighbor-Join and BioNJ algorithms to a matrix of pairwise distances estimated using the Tamura-Nei model, and then selecting the topology with superior log likelihood value. A discrete Gamma distribution was used to model evolutionary rate differences among sites (5 categories (+*G*, parameter = 0.1449)). The tree is drawn to scale, with branch lengths measured in the number of substitutions per site. The analysis involved 18 nucleotide sequences, of which 16 were used as marker sequences to determine the genotype of 2 sequences. All positions containing gaps and missing data were eliminated. There was a total of 691 positions in the final dataset. Evolutionary analyses were conducted in MEGA X.

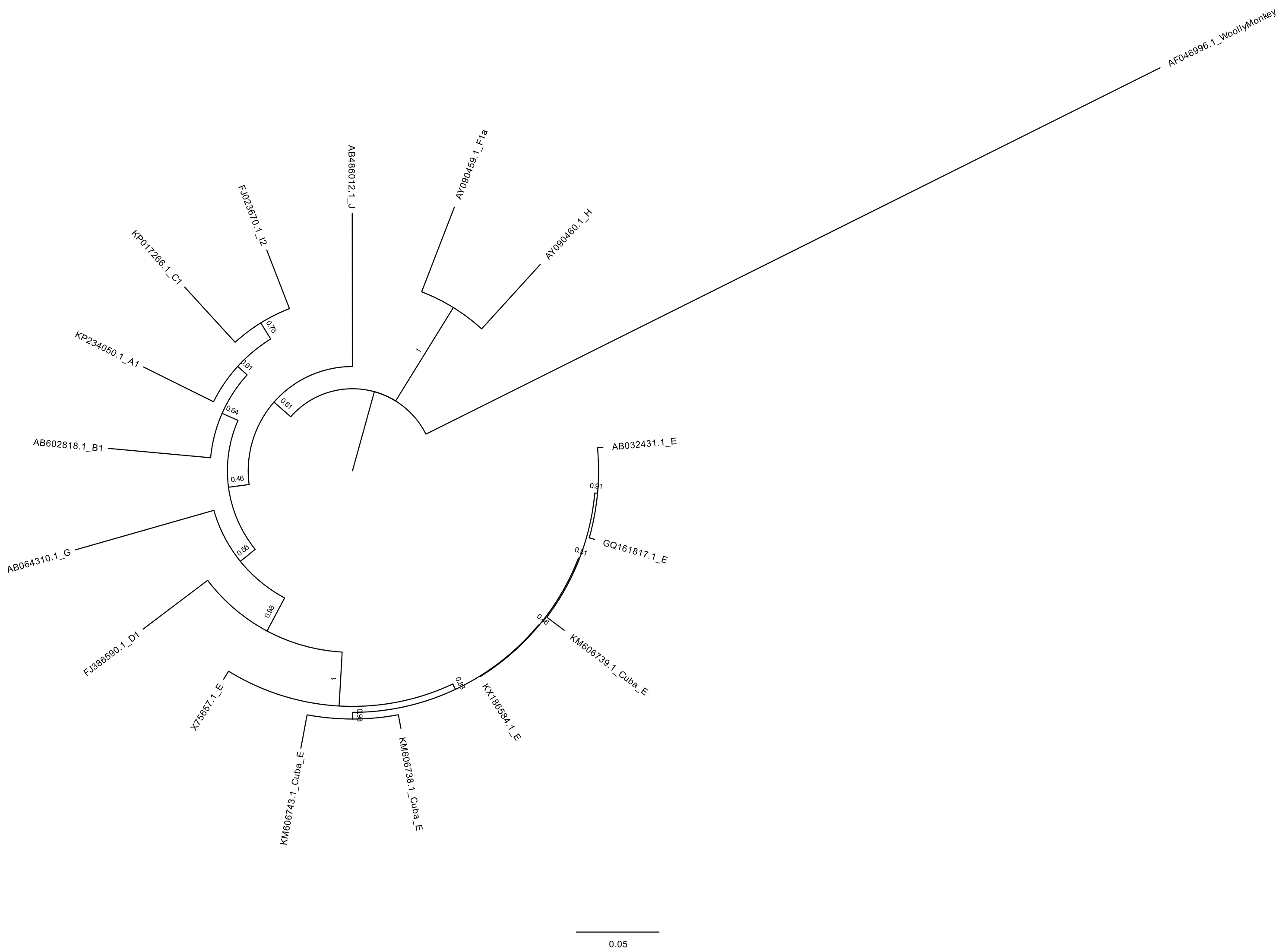

Tree 2. The evolutionary history was inferred by using the Maximum Likelihood method and Tamura-Nei model. The percentage of replicate trees in which the associated taxa clustered together in the bootstrap test (1000 replicates) are shown next to the branches. Initial tree(s) for the heuristic search were obtained automatically by applying Neighbor-Join and BioNJ algorithms to a matrix of pairwise distances estimated using the Tamura-Nei model, and then selecting the topology with superior log likelihood value. A discrete Gamma distribution was used to model evolutionary rate differences among sites (5 categories (+*G*, parameter = 0.2521)). The tree is drawn to scale, with branch lengths measured in the number of substitutions per site. The analysis involved 17 nucleotide sequences, of which 14 were used as marker sequences to determine the genotype of 3 sequences. All positions containing gaps and missing data were eliminated. There was a total of 3168 positions in the final dataset. Evolutionary analyses were conducted in MEGA X.

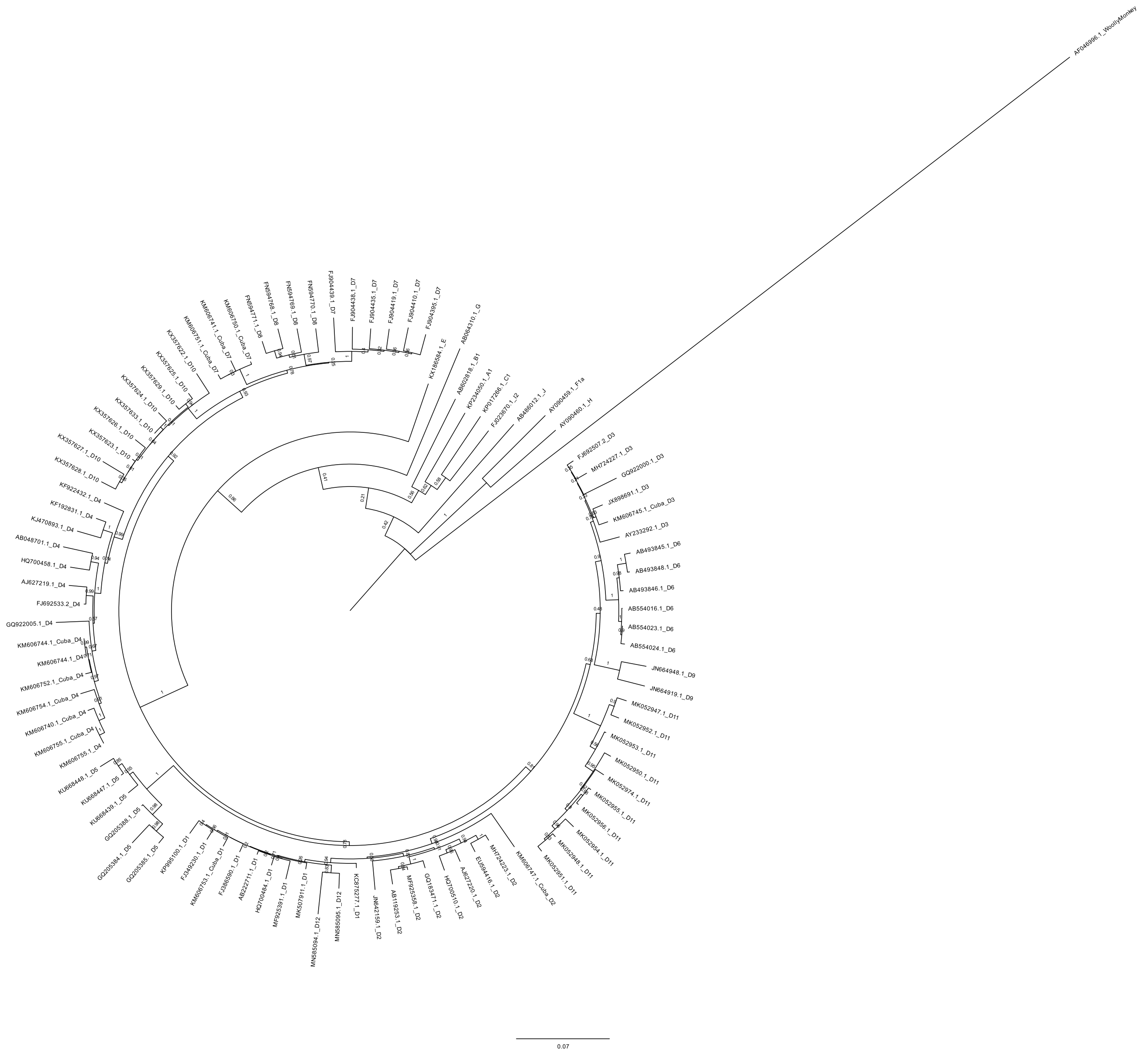

Tree 3. The evolutionary history was inferred by using the Maximum Likelihood method and Tamura-Nei model. The percentage of replicate trees in which the associated taxa clustered together in the bootstrap test (1000 replicates) are shown next to the branches. Initial tree(s) for the heuristic search were obtained automatically by applying Neighbor-Join and BioNJ algorithms to a matrix of pairwise distances estimated using the Tamura-Nei model, and then selecting the topology with superior log likelihood value. A discrete Gamma distribution was used to model evolutionary rate differences among sites (5 categories (+*G*, parameter = 0.2801)). The tree is drawn to scale, with branch lengths measured in the number of substitutions per site. The analysis involved 97 nucleotide sequences, of which 86 were used as marker sequences to determine the genotype of 11 sequences. All positions containing gaps and missing data were eliminated. There was a total of 3082 positions in the final dataset. Evolutionary analyses were conducted in MEGA X.

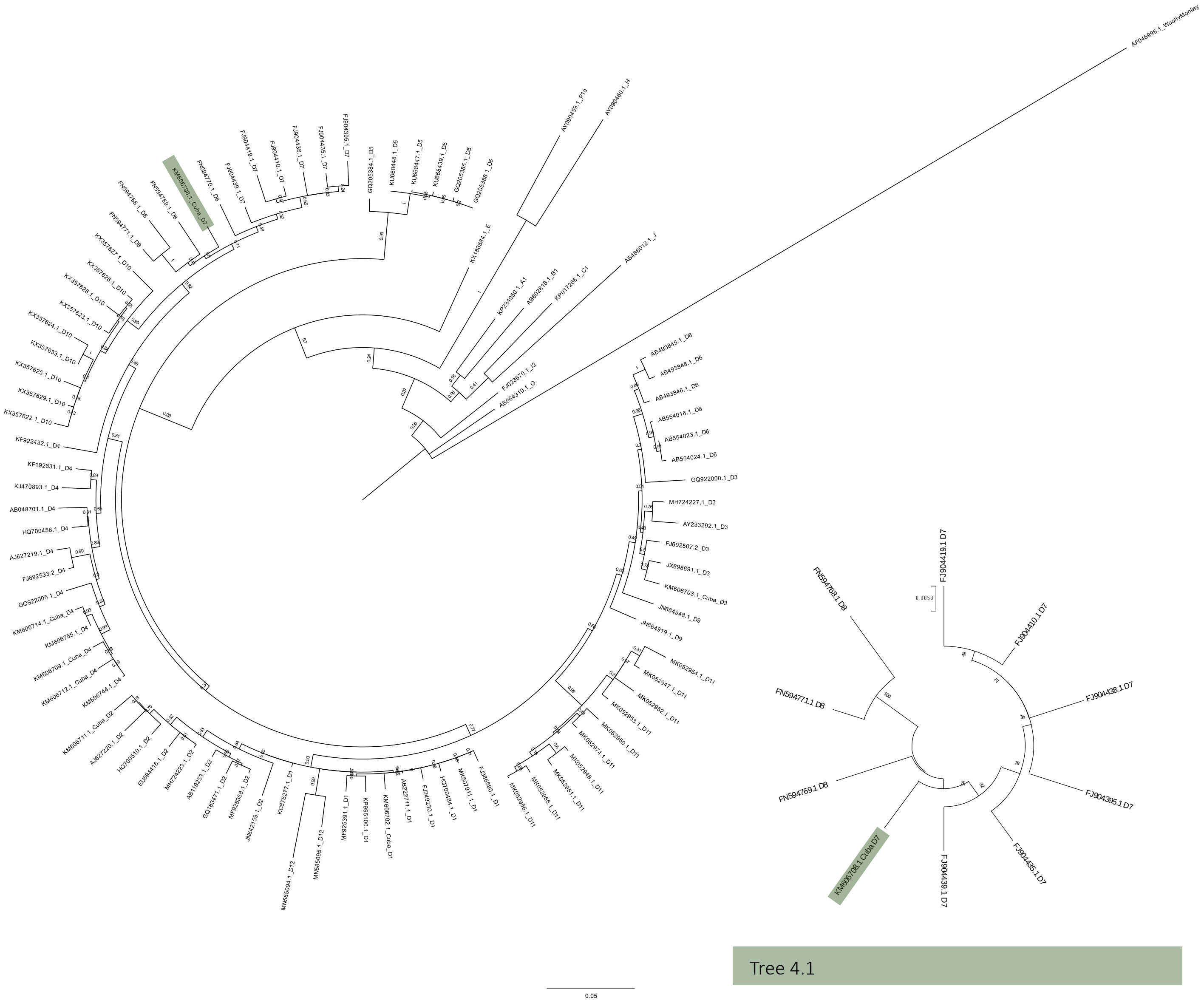

Tree 4. The evolutionary history was inferred by using the Maximum Likelihood method and Tamura-Nei model. The percentage of replicate trees in which the associated taxa clustered together in the bootstrap test (1000 replicates) are shown next to the branches. Initial tree(s) for the heuristic search were obtained automatically by applying Neighbor-Join and BioNJ algorithms to a matrix of pairwise distances estimated using the Tamura-Nei model, and then selecting the topology with superior log likelihood value. A discrete Gamma distribution was used to model evolutionary rate differences among sites (5 categories (+*G*, parameter = 0.2274)). The tree is drawn to scale, with branch lengths measured in the number of substitutions per site. The analysis involved 93 nucleotide sequences, of which 86 were used as marker sequences to determine the genotype of 7 sequences. Although sequence KM606708 showed an uncertain placement in the initial tree, upon further analysis (Tree 4.1) it was determined that it was subtype D7. All positions containing gaps and missing data were eliminated. There was a total of 1235 positions in the final dataset. Evolutionary analyses were conducted in MEGA X.

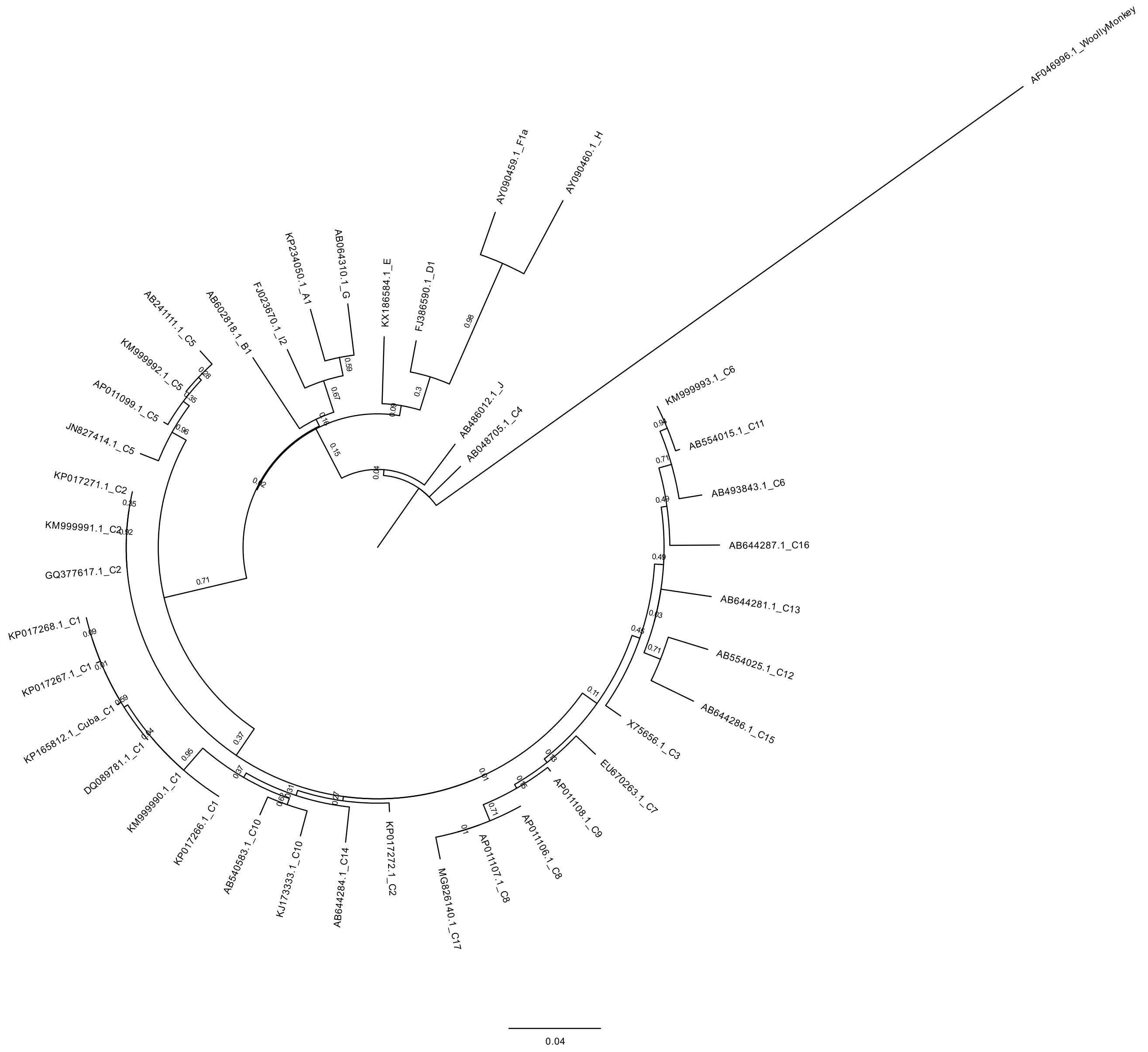

Tree 5. The evolutionary history was inferred by using the Maximum Likelihood method and Tamura-Nei model. The percentage of replicate trees in which the associated taxa clustered together in the bootstrap test (1000 replicates) are shown next to the branches. Initial tree(s) for the heuristic search were obtained automatically by applying Neighbor-Join and BioNJ algorithms to a matrix of pairwise distances estimated using the Tamura-Nei model, and then selecting the topology with superior log likelihood value. A discrete Gamma distribution was used to model evolutionary rate differences among sites (5 categories (+*G*, parameter = 0.1348)). The tree is drawn to scale, with branch lengths measured in the number of substitutions per site. The analysis involved 41 nucleotide sequences, of which 40 were used as marker sequences to determine the genotype of 1 sequence. All positions containing gaps and missing data were eliminated. There was a total of 678 positions in the final dataset. Evolutionary analyses were conducted in MEGA X.

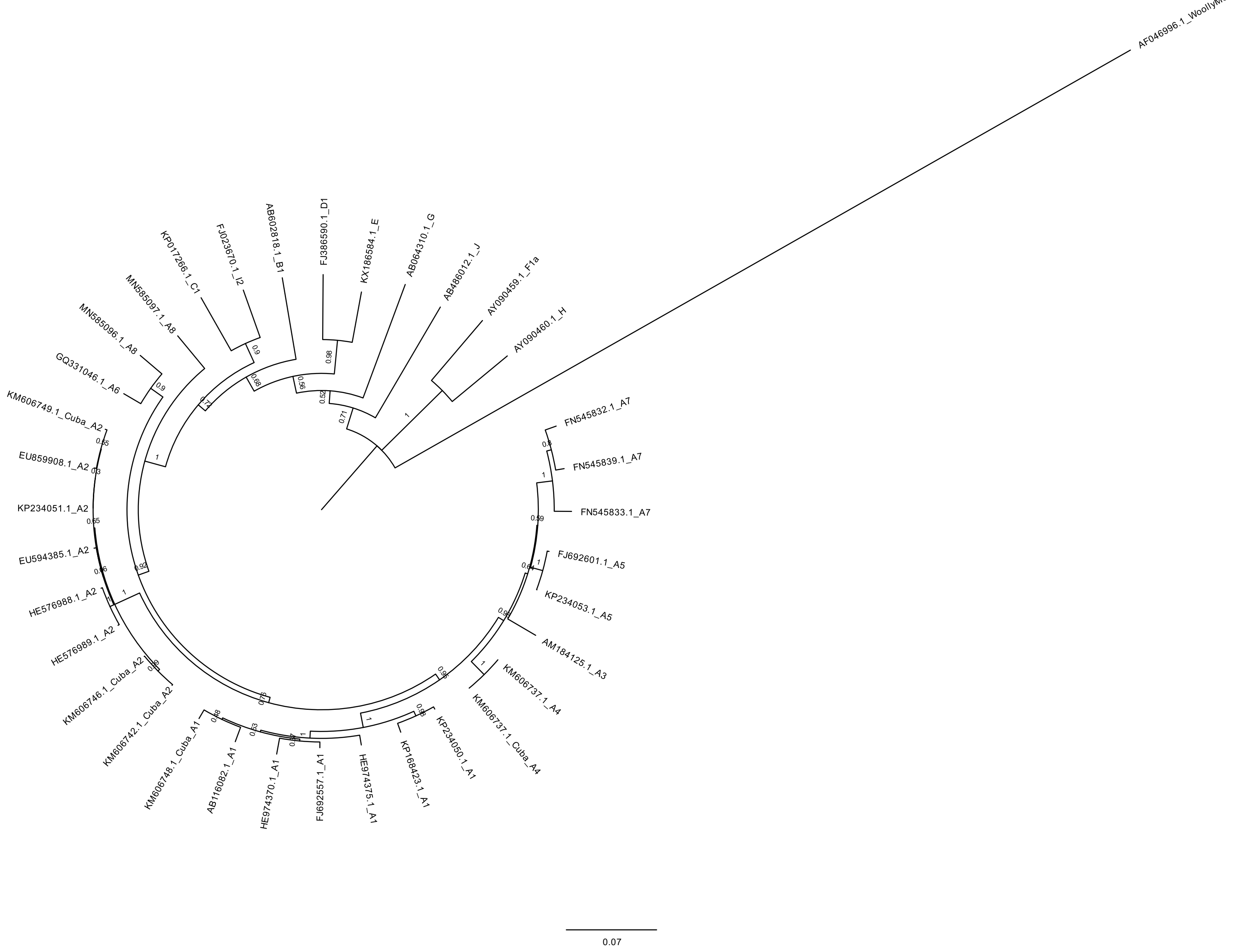

Tree 6. The evolutionary history was inferred by using the Maximum Likelihood method and Tamura-Nei model. The percentage of replicate trees in which the associated taxa clustered together in the bootstrap test (1000 replicates) are shown next to the branches. Initial tree(s) for the heuristic search were obtained automatically by applying Neighbor-Join and BioNJ algorithms to a matrix of pairwise distances estimated using the Tamura-Nei model, and then selecting the topology with superior log likelihood value. A discrete Gamma distribution was used to model evolutionary rate differences among sites (5 categories (+*G*, parameter = 0.2141)). The tree is drawn to scale, with branch lengths measured in the number of substitutions per site. The analysis involved 36 nucleotide sequences, of which 31 were used as marker sequences to determine the genotype of 5 sequences. All positions containing gaps and missing data were eliminated. There was a total of 3030 positions in the final dataset. Evolutionary analyses were conducted in MEGA X.

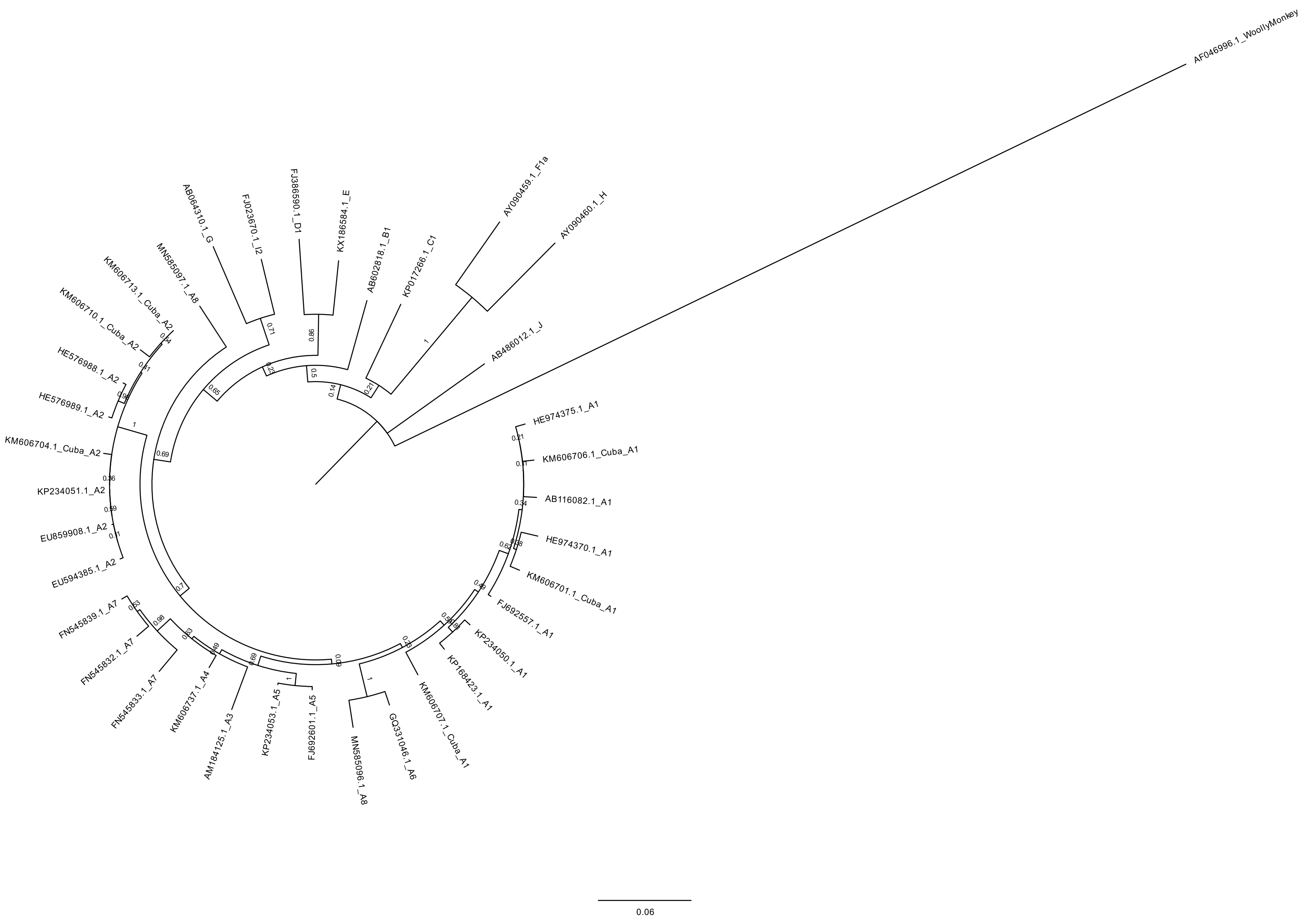

Tree 7. The evolutionary history was inferred by using the Maximum Likelihood method and Tamura-Nei model. The percentage of replicate trees in which the associated taxa clustered together in the bootstrap test (1000 replicates) are shown next to the branches. Initial tree(s) for the heuristic search were obtained automatically by applying Neighbor-Join and BioNJ algorithms to a matrix of pairwise distances estimated using the Tamura-Nei model, and then selecting the topology with superior log likelihood value. A discrete Gamma distribution was used to model evolutionary rate differences among sites (5 categories (+*G*, parameter = 0.1737)). The tree is drawn to scale, with branch lengths measured in the number of substitutions per site. The analysis involved 37 nucleotide sequences, of which 31 were used as marker sequences to determine the genotype of 6 sequences. All positions containing gaps and missing data were eliminated. There was a total of 1222 positions in the final dataset. Evolutionary analyses were conducted in MEGA X.

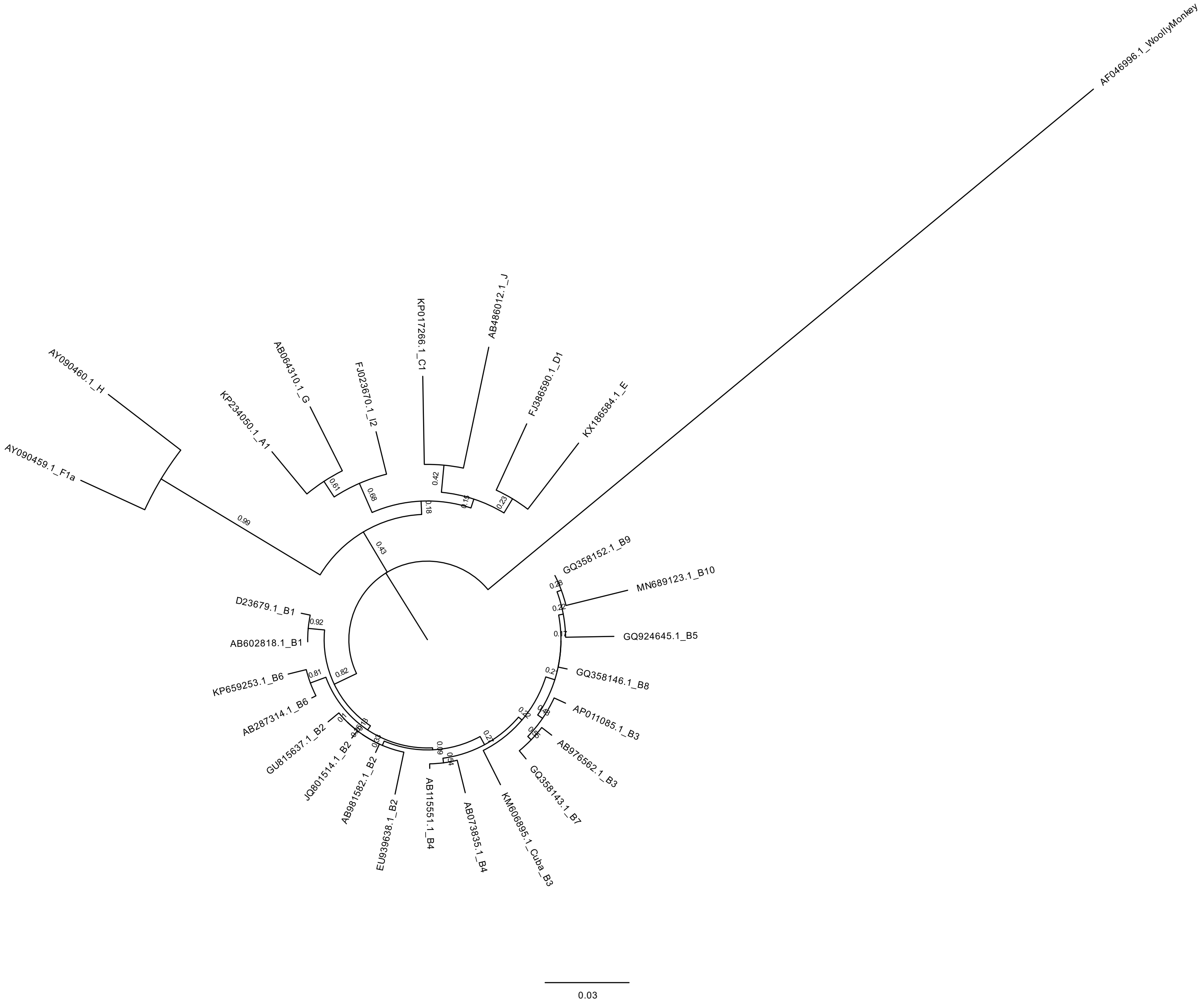

Tree 8. The evolutionary history was inferred by using the Maximum Likelihood method and Tamura-Nei model. The percentage of replicate trees in which the associated taxa clustered together in the bootstrap test (1000 replicates) are shown next to the branches. Initial tree(s) for the heuristic search were obtained automatically by applying Neighbor-Join and BioNJ algorithms to a matrix of pairwise distances estimated using the Tamura-Nei model, and then selecting the topology with superior log likelihood value. A discrete Gamma distribution was used to model evolutionary rate differences among sites (5 categories (+G, parameter = 0.1546)). The tree is drawn to scale, with branch lengths measured in the number of substitutions per site. The analysis involved 28 nucleotide sequences, of which 27 were used as marker sequences to determine the genotype of 1 sequence, which, after further analysis, could not be classified conclusively. All positions containing gaps and missing data were eliminated. There was a total of 698 positions in the final dataset. Evolutionary analyses were conducted in MEGA X.

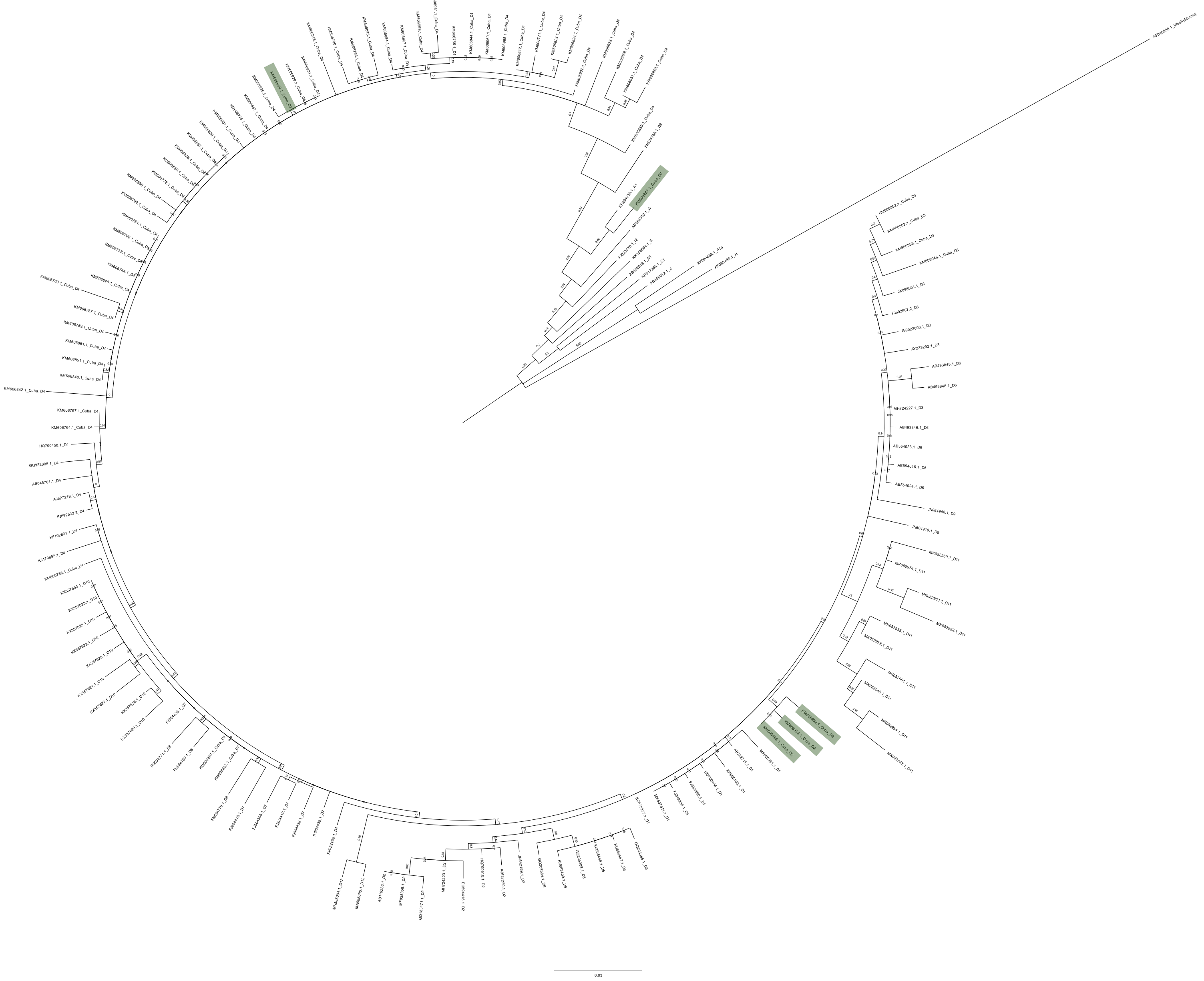

Tree 9. The evolutionary history was inferred by using the Maximum Likelihood method and Tamura-Nei model. The percentage of replicate trees in which the associated taxa clustered together in the bootstrap test (1000 replicates) are shown next to the branches. Initial tree(s) for the heuristic search were obtained automatically by applying Neighbor-Join and BioNJ algorithms to a matrix of pairwise distances estimated using the Tamura-Nei model, and then selecting the topology with superior log likelihood value. A discrete Gamma distribution was used to model evolutionary rate differences among sites (5 categories (+G, parameter = 0.2299)). The tree is drawn to scale, with branch lengths measured in the number of substitutions per site. The analysis involved 145 nucleotide sequences, of which 86 were used as marker sequences to determine the genotype of 59 sequences. Those sequences that exhibited unusual alignment were further analyzed in subsequent trees leading to a change in subtype of four sequences and a change in genotype of one (KM606887). All positions containing gaps and missing data were eliminated. There was a total of 664 positions in the final dataset. Evolutionary analyses were conducted in MEGA X.

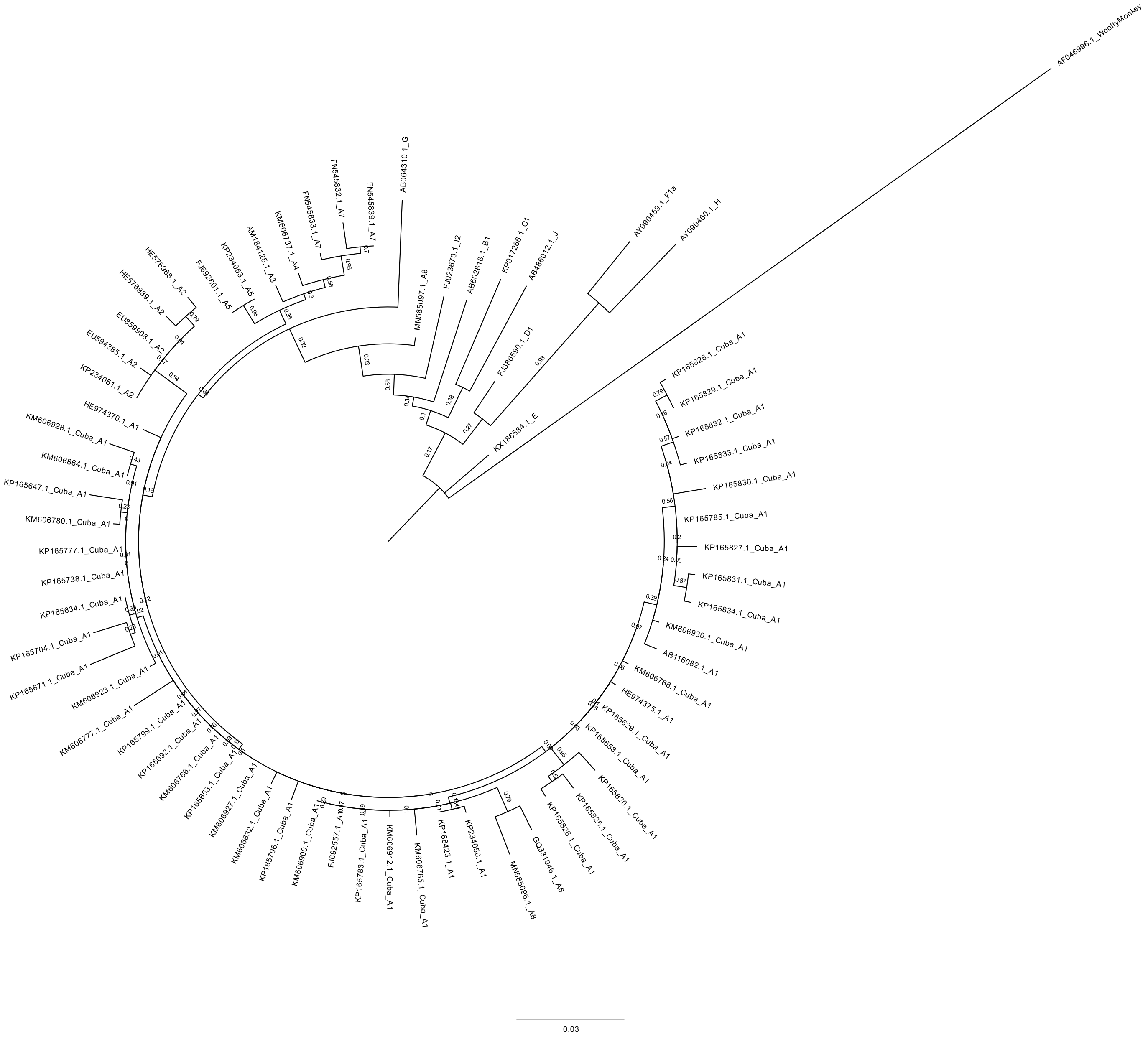

Tree 10. The evolutionary history was inferred by using the Maximum Likelihood method and Tamura-Nei model. The percentage of replicate trees in which the associated taxa clustered together in the bootstrap test (1000 replicates) are shown next to the branches. Initial tree(s) for the heuristic search were obtained automatically by applying Neighbor-Join and BioNJ algorithms to a matrix of pairwise distances estimated using the Tamura-Nei model, and then selecting the topology with superior log likelihood value. A discrete Gamma distribution was used to model evolutionary rate differences among sites (5 categories (+G, parameter = 0.1694)). The tree is drawn to scale, with branch lengths measured in the number of substitutions per site. The analysis involved 69 nucleotide sequences, of which 31 were used as marker sequences to determine the genotype of 38 sequences. All positions containing gaps and missing data were eliminated. There was a total of 657 positions in the final dataset. Evolutionary analyses were conducted in MEGA X.

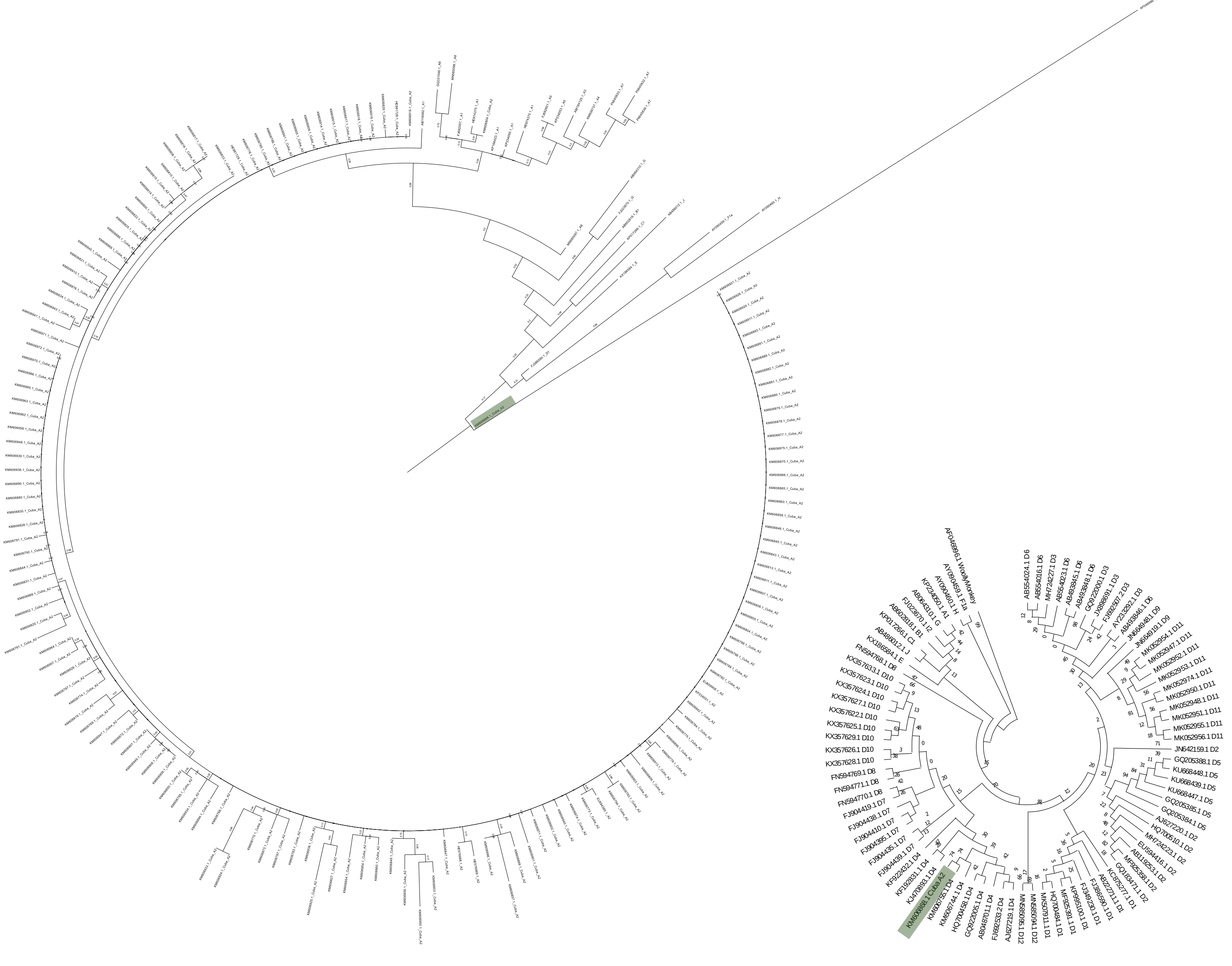

Tree 11.1

Tree 11. The evolutionary history was inferred by using the Maximum Likelihood method and Tamura-Nei model. The percentage of replicate trees in which the associated taxa clustered together in the bootstrap test (1000 replicates) are shown next to the branches. Initial tree(s) for the heuristic search were obtained automatically by applying Neighbor-Join and BioNJ algorithms to a matrix of pairwise distances estimated using the Tamura-Nei model, and then selecting the topology with superior log likelihood value. A discrete Gamma distribution was used to model evolutionary rate differences among sites (5 categories (+G, parameter = 0.2058)). The tree is drawn to scale, with branch lengths measured in the number of substitutions per site. The analysis involved 176 nucleotide sequences, of which 31 were used as marker sequences to determine the genotype of 145 sequences. Sequence KM606868 was reclassified to genotype D and subtype 4. All positions containing gaps and missing data were eliminated. There was a total of 511 positions in the final dataset. Evolutionary analyses were conducted in MEGA X.

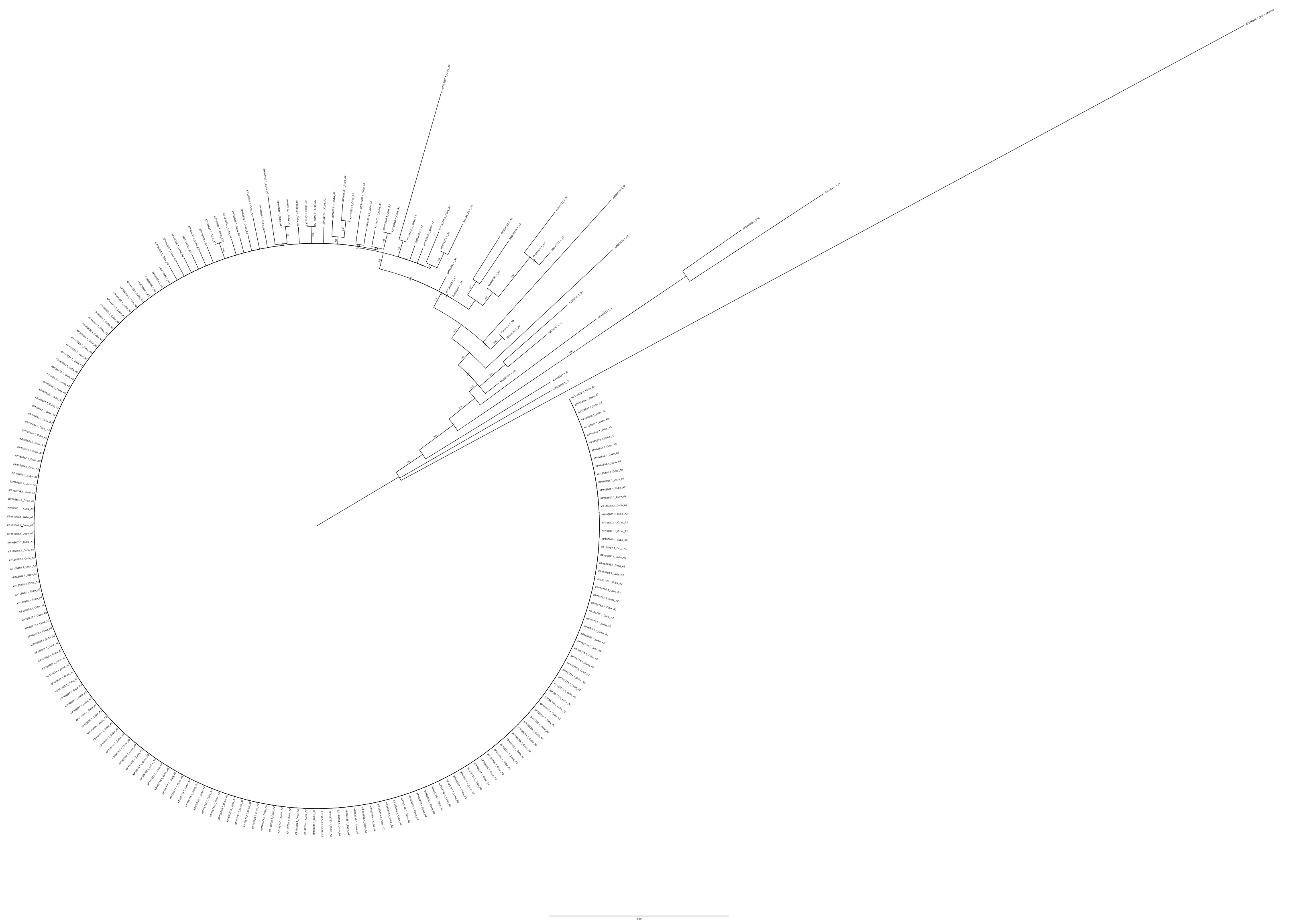

Tree 12. The evolutionary history was inferred by using the Maximum Likelihood method and Tamura-Nei model. The percentage of replicate trees in which the associated taxa clustered together in the bootstrap test (1000 replicates) are shown next to the branches. Initial tree(s) for the heuristic search were obtained automatically by applying Neighbor-Join and BioNJ algorithms to a matrix of pairwise distances estimated using the Tamura-Nei model, and then selecting the topology with superior log likelihood value. A discrete Gamma distribution was used to model evolutionary rate differences among sites (5 categories (+G, parameter = 0.1946)). The tree is drawn to scale, with branch lengths measured in the number of substitutions per site. The analysis involved 221 nucleotide sequences, of which 31 were used as marker sequences to determine the genotype of 190 sequences. Additional phylogenetic analysis was done for sequences that needed additional clarification. All positions containing gaps and missing data were eliminated. There was a total of 573 positions in the final dataset. Evolutionary analyses were conducted in MEGA X.

ID	GENOTYPE	SUBTYPE	COUNTRY	TREE	ALIGNMENT[1]	BASE PAIRS
KM606705	H	H	Cuba	1	155-1399	1245
KM606937	H	H	Cuba	1	132-845	714
KM606738	E	E	Cuba	2	Complete Genome	3212
KM606739	E	E	Cuba	2	Complete Genome	3212
KM606743	E	E	Cuba	2	Complete Genome	3211
KM606740	D	D4	Cuba	3	Complete Genome	3183
KM606741	D	D7	Cuba	3	Complete Genome	3182
KM606744	D	D4	Cuba	3	Complete Genome	3182
KM606745	D	D3/D6	Cuba	3	Complete Genome	3182
KM606747	D	D2	Cuba	3	Complete Genome	3174
KM606750	D	D7	Cuba	3	Complete Genome	3182
KM606751	D	D7	Cuba	3	Complete Genome	3182
KM606752	D	D4	Cuba	3	Complete Genome	3182
KM606753	D	D1	Cuba	3	Complete Genome	3182
KM606754	D	D4	Cuba	3	Complete Genome	3182
KM606755	D	D4	Cuba	3	Complete Genome	3182
KM606702	D	D1	Cuba	4	155-1399	1245
KM606703	D	D3/D6	Cuba	4	155-1399	1245
KM606708	D	D7	Cuba	4	155-1399	1245
KM606709	D	D4	Cuba	4	155-1399	1245
KM606711	D	D2	Cuba	4	155-1399	1245
KM606712	D	D4	Cuba	4	155-1399	1245
KM606714	D	D4	Cuba	4	155-1399	1245
KP165812	C	C1	Cuba	5	157-837	618
KM606737	A	A4	Cuba	6	Complete Genome	3221
KM606742	A	A2	Cuba	6	Complete Genome	3221
KM606746	A	A2	Cuba	6	Complete Genome	3221
KM606748	A	A1	Cuba	6	Complete Genome	3221
KM606749	A	A2	Cuba	6	Complete Genome	3221
KM606701	A	A1	Cuba	7	155-1399	1245
KM606704	A	A2	Cuba	7	155-1399	1245
KM606706	A	A1	Cuba	7	155-1399	1245
KM606707	A	A1	Cuba	7	155-1399	1245
KM606710	A	A2	Cuba	7	155-1399	1245
KM606713	A	A2	Cuba	7	155-1399	1245
KM606895	B	NA	Cuba	8	132-845	714
KM606756	D	D4	Cuba	9	132-845	714
KM606757	D	D4	Cuba	9	132-845	714
KM606758	D	D4	Cuba	9	154-846	693
KM606759	D	D4	Cuba	9	132-845	714
KM606760	D	D4	Cuba	9	132-846	715
KM606761	D	D4	Cuba	9	132-845	714
KM606762	D	D4	Cuba	9	132-846	715
KM606763	D	D4	Cuba	9	132-846	715

KM606764	D	D4	Cuba	9	132-846	715
KM606767	D	D4	Cuba	9	132-846	715
KM606771	D	D4	Cuba	9	132-846	715
KM606772	D	D4	Cuba	9	132-846	715
KM606776	D	D4	Cuba	9	132-846	715
KM606790	D	D4	Cuba	9	132-846	715
KM606796	D	D4	Cuba	9	132-846	715
KM606801	D	D4	Cuba	9	145-845	701
KM606812	D	D4	Cuba	9	152-845	694
KM606818	D	D4	Cuba	9	147-845	699
KM606823	D	D4	Cuba	9	152-845	694
KM606824	D	D4	Cuba	9	147-845	700
KM606835	D	D4	Cuba	9	147-845	699
KM606836	D	D4	Cuba	9	158-845	688
KM606837	D	D4	Cuba	9	147-845	699
KM606838	D	D4	Cuba	9	147-845	699
KM606839	D	D4	Cuba	9	147-845	699
KM606840	D	D4	Cuba	9	147-845	699
KM606842	D	D4	Cuba	9	147-845	699
KM606848	D	D4	Cuba	9	132-845	714
KM606851	D	D4	Cuba	9	132-845	714
KM606852	D	D3/D6	Cuba	9	132-845	714
KM606853	D	D1	Cuba	9	132-845	714
KM606855	D	D3/D6	Cuba	9	132-845	714
KM606859	D	D4	Cuba	9	132-845	714
KM606861	D	D4	Cuba	9	132-845	714
KM606862	D	D3/D6	Cuba	9	132-845	714
KM606867	D	D4	Cuba	9	132-845	714
KM606883	D	D4	Cuba	9	132-845	714
KM606884	D	D4	Cuba	9	132-845	714
KM606887	A	A2	Cuba	9	132-845	714
KM606892	D	D7	Cuba	9	132-845	714
KM606897	D	D7	Cuba	9	132-845	714
KM606898	D	D1	Cuba	9	132-845	714
KM606902	D	D4	Cuba	9	132-845	714
KM606922	D	D4	Cuba	9	132-848	717
KM606929	D	D4	Cuba	9	153-824	672
KM606931	D	D4	Cuba	9	132-845	714
KM606932	D	D2	Cuba	9	132-845	714
KM606935	D	D4	Cuba	9	132-845	714
KM606944	D	D4	Cuba	9	132-845	714
KM606946	D	D3/D6	Cuba	9	132-845	714
KM606951	D	D4	Cuba	9	132-845	714
KM606953	D	D4	Cuba	9	132-845	714
KM606955	D	D4	Cuba	9	132-845	714
KM606958	D	D4	Cuba	9	132-845	714

KM606959	D	D4	Cuba	9	132-845	714
KM606960	D	D4	Cuba	9	132-845	714
KM606961	D	D4	Cuba	9	132-845	714
KM606967	D	D4	Cuba	9	132-845	714
KM606968	D	D4	Cuba	9	132-845	714
KM606765	A	A1	Cuba	10	132-846	715
KM606766	A	A1	Cuba	10	132-846	715
KM606777	A	A1	Cuba	10	132-846	715
KM606780	A	A1	Cuba	10	132-846	715
KM606788	A	A1	Cuba	10	132-845	714
KM606832	A	A1	Cuba	10	147-845	699
KM606864	A	A1	Cuba	10	132-845	714
KM606900	A	A1	Cuba	10	132-845	714
KM606912	A	A1	Cuba	10	132-845	714
KM606923	A	A1	Cuba	10	132-845	714
KM606927	A	A1	Cuba	10	132-845	714
KM606928	A	A1	Cuba	10	132-845	714
KM606930	A	A1	Cuba	10	132-845	714
KP165629	A	A1	Cuba	10	157-837	681
KP165634	A	A1	Cuba	10	157-837	681
KP165647	A	A1	Cuba	10	157-837	681
KP165653	A	A1	Cuba	10	157-837	681
KP165658	A	A1	Cuba	10	157-837	681
KP165671	A	A1	Cuba	10	157-837	681
KP165692	A	A1	Cuba	10	157-837	681
KP165704	A	A1	Cuba	10	157-837	681
KP165706	A	A1	Cuba	10	157-837	681
KP165738	A	A1	Cuba	10	157-837	681
KP165777	A	A1	Cuba	10	157-837	681
KP165783	A	A1	Cuba	10	157-837	681
KP165785	A	A1	Cuba	10	157-837	681
KP165799	A	A1	Cuba	10	157-837	681
KP165820	A	A1	Cuba	10	157-837	681
KP165825	A	A1	Cuba	10	157-837	681
KP165826	A	A1	Cuba	10	157-837	681
KP165827	A	A1	Cuba	10	157-837	681
KP165828	A	A1	Cuba	10	157-837	681
KP165829	A	A1	Cuba	10	157-837	681
KP165830	A	A1	Cuba	10	157-837	681
KP165831	A	A1	Cuba	10	157-837	681
KP165832	A	A1	Cuba	10	157-837	681
KP165833	A	A1	Cuba	10	157-837	681
KP165834	A	A1	Cuba	10	157-837	681
HE981728	A	A2	Cuba	11	157-837	681
HE981729	A	A2	Cuba	11	157-837	681
KM606768	A	A2	Cuba	11	132-846	715

KM606769	A	A2	Cuba	11	132-846	715
KM606770	A	A2	Cuba	11	132-846	715
KM606773	A	A2	Cuba	11	132-846	715
KM606774	A	A2	Cuba	11	132-846	715
KM606775	A	A2	Cuba	11	132-846	715
KM606778	A	A2	Cuba	11	132-846	715
KM606779	A	A2	Cuba	11	132-846	715
KM606781	A	A2	Cuba	11	132-846	715
KM606782	A	A2	Cuba	11	132-846	715
KM606783	A	A2	Cuba	11	132-846	715
KM606784	A	A2	Cuba	11	132-846	715
KM606785	A	A2	Cuba	11	132-845	714
KM606786	A	A2	Cuba	11	132-846	715
KM606787	A	A2	Cuba	11	132-846	715
KM606789	A	A2	Cuba	11	132-846	715
KM606791	A	A2	Cuba	11	132-846	715
KM606792	A	A2	Cuba	11	132-846	715
KM606793	A	A2	Cuba	11	132-846	715
KM606794	A	A2	Cuba	11	132-846	715
KM606795	A	A2	Cuba	11	132-845	714
KM606797	A	A2	Cuba	11	132-846	715
KM606798	A	A2	Cuba	11	132-845	714
KM606799	A	A2	Cuba	11	318-842	525
KM606800	A	A2	Cuba	11	132-846	715
KM606802	A	A2	Cuba	11	144-845	702
KM606803	A	A2	Cuba	11	132-846	715
KM606804	A	A2	Cuba	11	153-845	693
KM606805	A	A2	Cuba	11	153-845	693
KM606806	A	A2	Cuba	11	152-845	694
KM606807	A	A2	Cuba	11	153-845	693
KM606808	A	A2	Cuba	11	153-845	693
KM606809	A	A2	Cuba	11	153-845	693
KM606810	A	A2	Cuba	11	153-846	694
KM606811	A	A2	Cuba	11	145-846	702
KM606813	A	A2	Cuba	11	147-845	699
KM606814	A	A2	Cuba	11	147-845	699
KM606815	A	A2	Cuba	11	147-845	699
KM606816	A	A2	Cuba	11	147-845	699
KM606817	A	A2	Cuba	11	147-845	699
KM606819	A	A2	Cuba	11	147-845	699
KM606820	A	A2	Cuba	11	147-845	699
KM606821	A	A2	Cuba	11	147-845	699
KM606822	A	A2	Cuba	11	147-845	699
KM606825	A	A2	Cuba	11	153-845	693
KM606826	A	A2	Cuba	11	147-845	699
KM606827	A	A2	Cuba	11	147-845	699

KM606828	A	A2	Cuba	11	153-845	693
KM606829	A	A2	Cuba	11	202-845	644
KM606830	A	A2	Cuba	11	147-845	699
KM606831	A	A2	Cuba	11	147-845	699
KM606833	A	A2	Cuba	11	147-845	699
KM606834	A	A2	Cuba	11	147-845	699
KM606841	A	A2	Cuba	11	147-845	699
KM606843	A	A2	Cuba	11	155-845	691
KM606844	A	A2	Cuba	11	132-845	714
KM606845	A	A2	Cuba	11	132-845	714
KM606846	A	A2	Cuba	11	132-845	714
KM606847	A	A2	Cuba	11	132-845	714
KM606849	A	A2	Cuba	11	132-845	714
KM606850	A	A2	Cuba	11	132-845	714
KM606854	A	A2	Cuba	11	132-845	714
KM606856	A	A2	Cuba	11	132-845	714
KM606857	A	A2	Cuba	11	132-845	714
KM606858	A	A2	Cuba	11	132-845	714
KM606860	A	A2	Cuba	11	132-845	714
KM606863	A	A2	Cuba	11	132-845	714
KM606865	A	A2	Cuba	11	132-845	714
KM606866	A	A2	Cuba	11	132-845	714
KM606868	D	D4	Cuba	11	132-845	714
KM606869	A	A2	Cuba	11	132-845	714
KM606870	A	A2	Cuba	11	132-845	714
KM606871	A	A2	Cuba	11	132-845	714
KM606872	A	A2	Cuba	11	132-845	714
KM606873	A	A2	Cuba	11	132-845	714
KM606874	A	A2	Cuba	11	132-845	714
KM606875	A	A2	Cuba	11	132-845	714
KM606876	A	A2	Cuba	11	132-845	714
KM606877	A	A2	Cuba	11	132-845	714
KM606878	A	A2	Cuba	11	132-845	714
KM606879	A	A2	Cuba	11	132-845	714
KM606880	A	A2	Cuba	11	132-845	714
KM606881	A	A2	Cuba	11	132-845	714
KM606882	A	A2	Cuba	11	132-845	714
KM606885	A	A2	Cuba	11	132-845	714
KM606886	A	A2	Cuba	11	132-845	714
KM606888	A	A2	Cuba	11	132-845	714
KM606889	A	A2	Cuba	11	132-845	714
KM606890	A	A2	Cuba	11	132-845	714
KM606891	A	A2	Cuba	11	132-845	714
KM606893	A	A2	Cuba	11	132-845	714
KM606894	A	A2	Cuba	11	132-845	714
KM606896	A	A2	Cuba	11	132-845	714

KM606899	A	A2	Cuba	11	132-845	714
KM606901	A	A2	Cuba	11	132-845	714
KM606903	A	A2	Cuba	11	132-845	714
KM606904	A	A2	Cuba	11	132-845	714
KM606905	A	A2	Cuba	11	132-845	714
KM606906	A	A2	Cuba	11	132-845	714
KM606907	A	A2	Cuba	11	132-845	714
KM606908	A	A2	Cuba	11	132-845	714
KM606909	A	A2	Cuba	11	132-845	714
KM606910	A	A2	Cuba	11	132-845	714
KM606911	A	A2	Cuba	11	132-845	714
KM606913	A	A2	Cuba	11	132-845	714
KM606914	A	A2	Cuba	11	132-845	714
KM606915	A	A2	Cuba	11	132-845	714
KM606916	A	A2	Cuba	11	132-845	714
KM606917	A	A2	Cuba	11	132-845	714
KM606918	A	A2	Cuba	11	132-845	714
KM606919	A	A2	Cuba	11	132-845	714
KM606920	A	A2	Cuba	11	132-845	714
KM606921	A	A2	Cuba	11	132-845	714
KM606924	A	A2	Cuba	11	132-845	714
KM606925	A	A2	Cuba	11	132-845	714
KM606926	A	A2	Cuba	11	132-845	714
KM606933	A	A2	Cuba	11	132-845	714
KM606934	A	A2	Cuba	11	132-845	714
KM606936	A	A2	Cuba	11	132-845	714
KM606938	A	A2	Cuba	11	132-845	714
KM606939	A	A2	Cuba	11	132-845	714
KM606940	A	A2	Cuba	11	132-845	714
KM606941	A	A2	Cuba	11	132-845	714
KM606942	A	A2	Cuba	11	132-845	714
KM606943	A	A2	Cuba	11	132-845	714
KM606945	A	A2	Cuba	11	132-845	714
KM606947	A	A2	Cuba	11	132-845	714
KM606948	A	A2	Cuba	11	132-845	714
KM606949	A	A2	Cuba	11	132-845	714
KM606950	A	A2	Cuba	11	132-845	714
KM606952	A	A2	Cuba	11	132-845	714
KM606954	A	A2	Cuba	11	132-845	714
KM606956	A	A2	Cuba	11	132-845	714
KM606957	A	A2	Cuba	11	132-845	714
KM606962	A	A2	Cuba	11	132-845	714
KM606963	A	A2	Cuba	11	132-845	714
KM606964	A	A2	Cuba	11	132-845	714
KM606965	A	A2	Cuba	11	132-845	714
KM606966	A	A2	Cuba	11	132-845	714

KM606969	A	A2	Cuba	11	132-845	714
KM606970	A	A2	Cuba	11	132-844	713
KM606971	A	A2	Cuba	11	132-845	714
KM606972	A	A2	Cuba	11	132-845	714
KP144200	A	A2	Cuba	12	157-837	681
KP144201	A	A2	Cuba	12	157-837	681
KP144202	A	A2	Cuba	12	157-837	681
KP144203	A	A2	Cuba	12	157-837	681
KP144204	A	A2	Cuba	12	157-837	681
KP144205	A	A2	Cuba	12	157-837	681
KP144206	A	A2	Cuba	12	157-837	681
KP144207	A	A2	Cuba	12	157-837	681
KP144208	A	A2	Cuba	12	157-837	681
KP144209	A	A2	Cuba	12	157-837	681
KP144210	A	A2	Cuba	12	157-837	681
KP165620	A	A2	Cuba	12	157-837	681
KP165621	A	A2	Cuba	12	157-837	681
KP165623	A	A2	Cuba	12	157-837	681
KP165624	A	A2	Cuba	12	157-837	681
KP165625	A	A2	Cuba	12	157-837	681
KP165626	A	A2	Cuba	12	157-837	681
KP165627	A	A2	Cuba	12	157-837	681
KP165628	A	A2	Cuba	12	157-837	681
KP165630	A	A2	Cuba	12	157-837	681
KP165631	A	A2	Cuba	12	157-837	681
KP165632	A	A2	Cuba	12	157-837	681
KP165633	A	A2	Cuba	12	157-837	681
KP165635	A	A2	Cuba	12	157-837	681
KP165637	A	A2	Cuba	12	157-837	681
KP165638	A	A2	Cuba	12	157-837	681
KP165639	A	A2	Cuba	12	157-837	681
KP165640	A	A2	Cuba	12	157-837	681
KP165641	A	A2	Cuba	12	157-837	681
KP165642	A	A2	Cuba	12	157-837	681
KP165643	A	A2	Cuba	12	157-837	681
KP165644	A	A2	Cuba	12	157-837	681
KP165645	A	A2	Cuba	12	157-837	681
KP165646	A	A2	Cuba	12	157-837	681
KP165648	A	A2	Cuba	12	157-837	681
KP165649	A	A2	Cuba	12	157-837	681
KP165650	A	A2	Cuba	12	157-837	681
KP165651	A	A2	Cuba	12	157-837	681
KP165652	A	A2	Cuba	12	157-837	681
KP165654	A	A2	Cuba	12	157-837	681
KP165655	A	A2	Cuba	12	157-837	681
KP165656	A	A2	Cuba	12	157-837	681

KP165657	A	A2	Cuba	12	157-837	681
KP165659	A	A2	Cuba	12	157-837	681
KP165660	A	A2	Cuba	12	157-837	681
KP165661	A	A2	Cuba	12	157-837	681
KP165662	A	A2	Cuba	12	157-837	681
KP165663	A	A2	Cuba	12	157-837	681
KP165664	A	A2	Cuba	12	157-837	681
KP165665	A	A2	Cuba	12	157-837	681
KP165666	A	A2	Cuba	12	157-837	681
KP165667	A	A2	Cuba	12	157-837	681
KP165668	A	A2	Cuba	12	157-837	681
KP165669	A	A2	Cuba	12	157-837	681
KP165670	A	A2	Cuba	12	157-837	681
KP165672	A	A2	Cuba	12	157-837	681
KP165673	A	A2	Cuba	12	157-837	681
KP165674	A	A2	Cuba	12	157-837	681
KP165675	A	A2	Cuba	12	157-837	681
KP165676	A	A2	Cuba	12	157-837	681
KP165677	A	A2	Cuba	12	157-837	681
KP165678	A	A2	Cuba	12	157-837	681
KP165679	A	A2	Cuba	12	157-837	681
KP165680	A	A2	Cuba	12	157-837	681
KP165681	A	A2	Cuba	12	157-837	681
KP165682	A	A2	Cuba	12	157-837	681
KP165683	A	A2	Cuba	12	157-837	681
KP165684	A	A2	Cuba	12	157-837	681
KP165685	A	A2	Cuba	12	157-837	681
KP165687	A	A2	Cuba	12	157-837	681
KP165688	A	A2	Cuba	12	157-837	681
KP165690	A	A2	Cuba	12	157-837	681
KP165691	A	A2	Cuba	12	157-837	681
KP165693	A	A2	Cuba	12	157-837	681
KP165694	A	A2	Cuba	12	157-837	681
KP165695	A	A2	Cuba	12	157-837	681
KP165696	A	A2	Cuba	12	157-837	681
KP165697	A	A2	Cuba	12	157-837	681
KP165698	A	A2	Cuba	12	157-837	681
KP165699	A	A2	Cuba	12	157-837	681
KP165700	A	A2	Cuba	12	157-837	681
KP165701	A	A2	Cuba	12	157-837	681
KP165702	A	A2	Cuba	12	157-837	681
KP165703	A	A2	Cuba	12	157-837	681
KP165705	A	A2	Cuba	12	157-837	681
KP165707	A	A2	Cuba	12	157-837	681
KP165708	A	A2	Cuba	12	157-837	681
KP165709	A	A2	Cuba	12	157-837	681

KP165710	A	A2	Cuba	12	157-837	681
KP165711	A	A2	Cuba	12	157-837	681
KP165712	A	A2	Cuba	12	157-837	681
KP165714	A	A2	Cuba	12	157-837	681
KP165715	A	A2	Cuba	12	157-837	681
KP165716	A	A2	Cuba	12	157-837	681
KP165717	A	A2	Cuba	12	157-837	681
KP165718	A	A2	Cuba	12	157-837	681
KP165719	A	A2	Cuba	12	157-837	681
KP165720	A	A2	Cuba	12	157-837	681
KP165721	A	A2	Cuba	12	157-837	681
KP165722	A	A2	Cuba	12	157-837	681
KP165723	A	A2	Cuba	12	157-837	681
KP165724	A	A2	Cuba	12	157-837	681
KP165726	A	A2	Cuba	12	157-837	681
KP165727	A	A2	Cuba	12	157-837	681
KP165728	A	A2	Cuba	12	157-837	681
KP165729	A	A2	Cuba	12	157-837	681
KP165730	A	A2	Cuba	12	157-837	681
KP165731	A	A2	Cuba	12	157-837	681
KP165732	A	A2	Cuba	12	157-837	681
KP165733	A	A2	Cuba	12	157-837	681
KP165734	A	A2	Cuba	12	157-837	681
KP165735	A	A2	Cuba	12	157-837	681
KP165736	A	A2	Cuba	12	157-837	681
KP165737	A	A2	Cuba	12	157-837	681
KP165739	A	A2	Cuba	12	157-837	681
KP165740	A	A2	Cuba	12	157-837	681
KP165741	A	A2	Cuba	12	157-837	681
KP165742	A	A2	Cuba	12	157-837	681
KP165744	A	A2	Cuba	12	157-837	681
KP165745	A	A2	Cuba	12	157-837	681
KP165747	A	A2	Cuba	12	157-837	681
KP165748	A	A2	Cuba	12	157-837	681
KP165749	A	A2	Cuba	12	157-837	681
KP165750	A	A2	Cuba	12	157-837	681
KP165751	A	A2	Cuba	12	157-837	681
KP165752	A	A2	Cuba	12	157-837	681
KP165753	A	A2	Cuba	12	157-837	681
KP165754	A	A2	Cuba	12	157-837	681
KP165755	A	A2	Cuba	12	157-837	681
KP165756	A	A2	Cuba	12	157-837	681
KP165757	A	A2	Cuba	12	157-837	681
KP165758	A	A2	Cuba	12	157-837	681
KP165759	A	A2	Cuba	12	157-837	681
KP165760	A	A2	Cuba	12	157-837	681

KP165761	A	A2	Cuba	12	157-837	681
KP165762	A	A2	Cuba	12	157-837	681
KP165763	A	A2	Cuba	12	157-837	681
KP165764	A	A2	Cuba	12	157-837	681
KP165765	A	A2	Cuba	12	157-837	681
KP165766	A	A2	Cuba	12	157-837	681
KP165767	A	A2	Cuba	12	157-837	681
KP165768	A	A2	Cuba	12	157-837	681
KP165770	A	A2	Cuba	12	157-837	681
KP165771	A	A2	Cuba	12	157-837	681
KP165772	A	A2	Cuba	12	157-837	681
KP165773	A	A2	Cuba	12	157-837	681
KP165774	A	A2	Cuba	12	157-837	681
KP165775	A	A2	Cuba	12	157-837	681
KP165776	A	A2	Cuba	12	157-837	681
KP165778	A	A2	Cuba	12	157-837	681
KP165779	A	A2	Cuba	12	157-837	681
KP165780	A	A2	Cuba	12	157-837	681
KP165781	A	A2	Cuba	12	157-837	681
KP165782	A	A2	Cuba	12	157-837	681
KP165784	A	A2	Cuba	12	157-837	681
KP165786	A	A2	Cuba	12	157-837	681
KP165788	A	A2	Cuba	12	157-837	681
KP165789	A	A2	Cuba	12	157-837	681
KP165790	A	A2	Cuba	12	157-837	681
KP165791	A	A2	Cuba	12	157-837	681
KP165793	A	A2	Cuba	12	157-837	681
KP165794	A	A2	Cuba	12	157-837	681
KP165795	A	A2	Cuba	12	157-837	681
KP165796	A	A2	Cuba	12	157-837	681
KP165797	A	A2	Cuba	12	157-837	681
KP165800	A	A2	Cuba	12	157-837	681
KP165801	A	A2	Cuba	12	157-837	681
KP165802	A	A2	Cuba	12	157-837	681
KP165803	A	A2	Cuba	12	157-837	681
KP165804	A	A2	Cuba	12	157-837	681
KP165805	A	A2	Cuba	12	157-837	681
KP165806	A	A2	Cuba	12	157-837	681
KP165807	A	A2	Cuba	12	157-837	681
KP165808	A	A2	Cuba	12	157-837	681
KP165809	A	A2	Cuba	12	157-837	681
KP165810	A	A2	Cuba	12	157-837	681
KP165811	A	A2	Cuba	12	157-837	681
KP165813	A	A2	Cuba	12	157-837	681
KP165814	A	A2	Cuba	12	157-837	681
KP165815	A	A2	Cuba	12	157-837	681

KP165817	A	A2	Cuba	12	157-837	681
KP165818	A	A2	Cuba	12	157-837	681
KP165819	A	A2	Cuba	12	157-837	681
KP165821	A	A2	Cuba	12	157-837	681
KP165822	A	A2	Cuba	12	157-837	681
KP165823	A	A2	Cuba	12	157-837	681
KP165824	A	A2	Cuba	12	157-837	681
KP165835	A	A2	Cuba	12	157-837	681
KP165836	A	A2	Cuba	12	157-837	681
KP165837	A	A2	Cuba	12	157-837	681

[1]Alignment to complete genome reference sequence VHB NC_003977.2

ALASKA

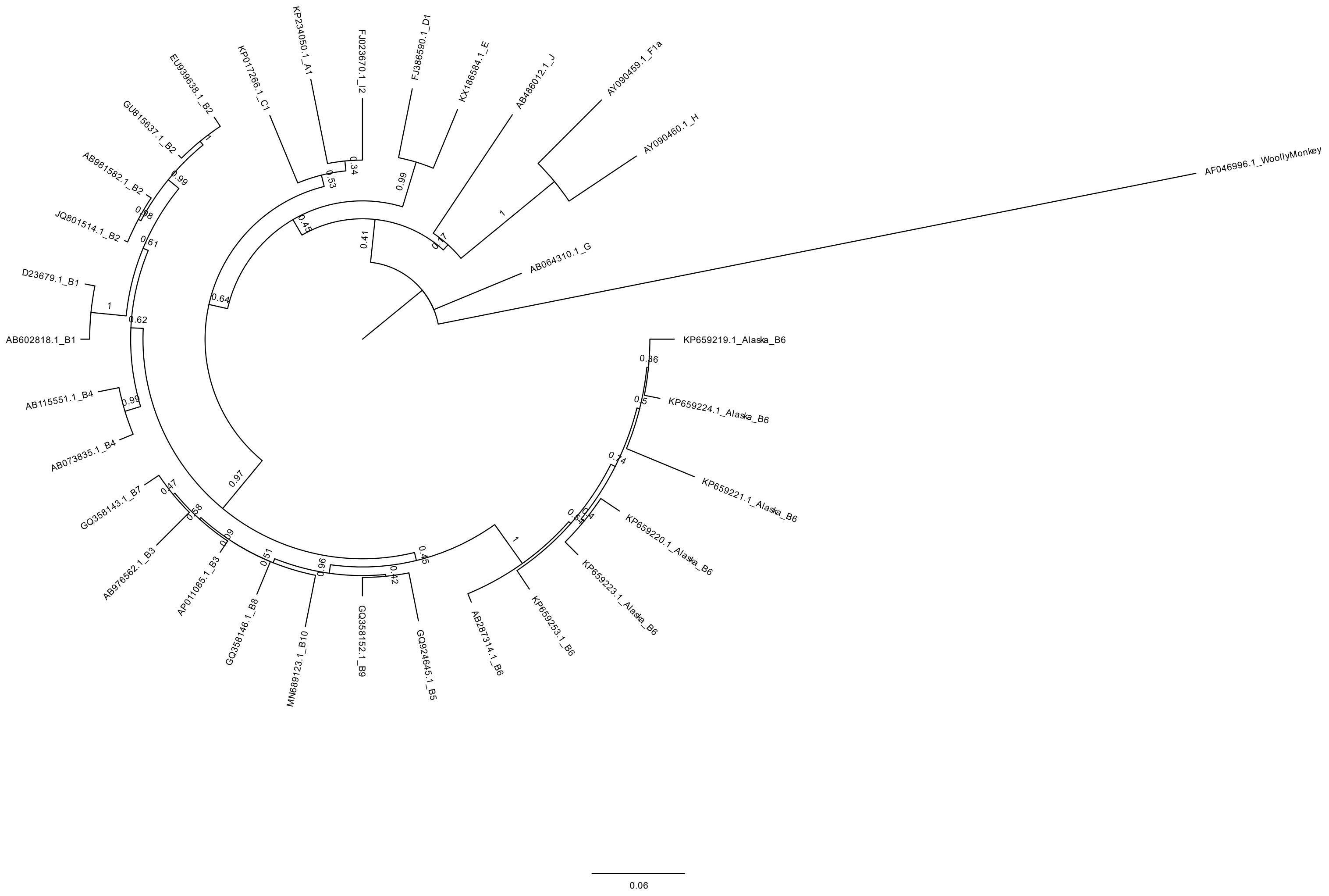

Tree 1. The evolutionary history was inferred by using the Maximum Likelihood method and Tamura-Nei model. The percentage of replicate trees in which the associated taxa clustered together in the bootstrap test (1000 replicates) are shown next to the branches. Initial tree(s) for the heuristic search were obtained automatically by applying Neighbor-Join and BioNJ algorithms to a matrix of pairwise distances estimated using the Tamura-Nei model, and then selecting the topology with superior log likelihood value. A discrete Gamma distribution was used to model evolutionary rate differences among sites (5 categories (+G, parameter = 0.2640)). The tree is drawn to scale, with branch lengths measured in the number of substitutions per site. The analysis involved 32 nucleotide sequences, of which 27 were used as marker sequences to determine the genotype of 5 sequences. All positions containing gaps and missing data were eliminated. There was a total of 2951 positions in the final dataset. Evolutionary analyses were conducted in MEGA X.

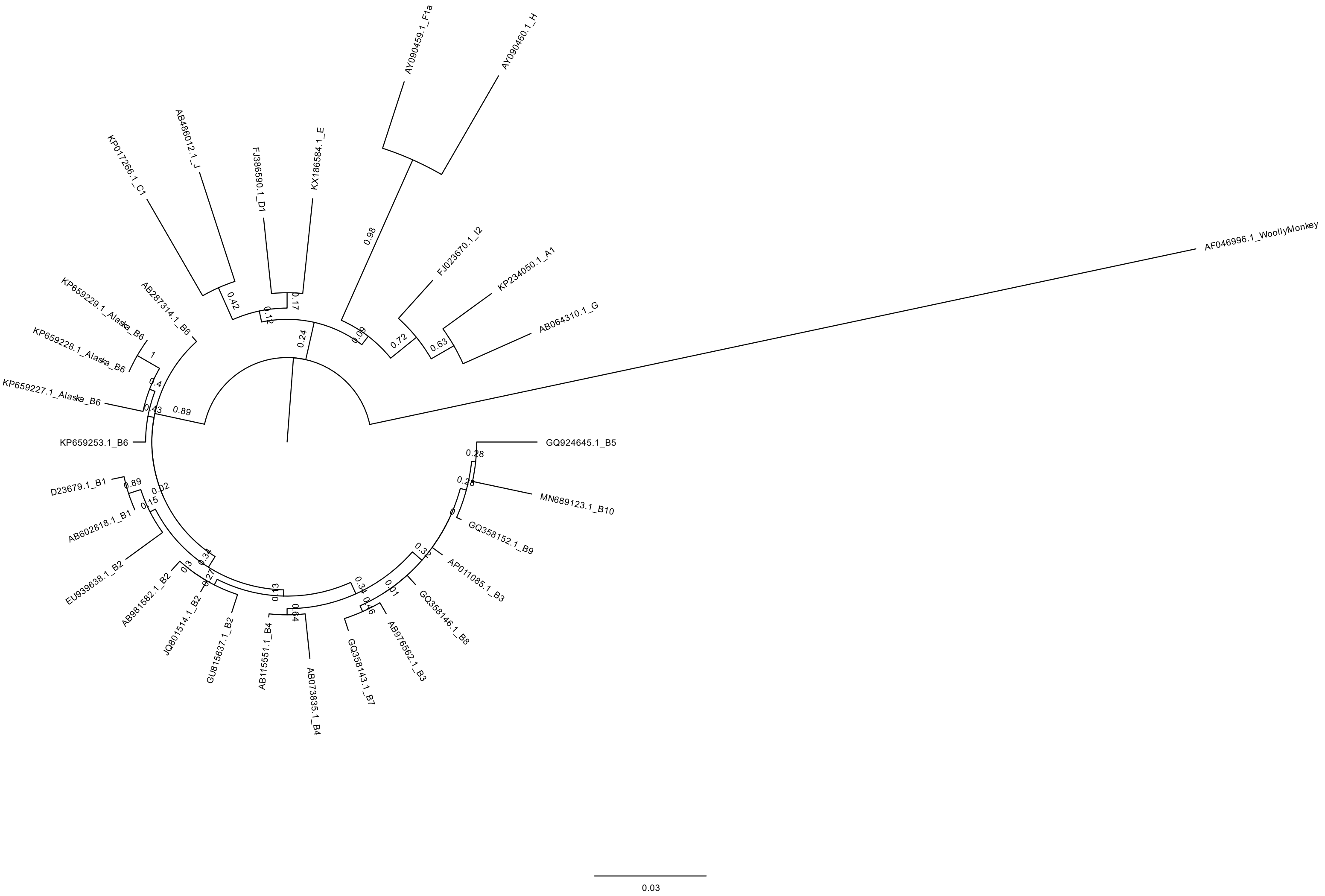

Tree 2. The evolutionary history was inferred by using the Maximum Likelihood method and Tamura-Nei model. The percentage of replicate trees in which the associated taxa clustered together in the bootstrap test (1000 replicates) are shown next to the branches. Initial tree(s) for the heuristic search were obtained automatically by applying Neighbor-Join and BioNJ algorithms to a matrix of pairwise distances estimated using the Tamura-Nei model, and then selecting the topology with superior log likelihood value. A discrete Gamma distribution was used to model evolutionary rate differences among sites (5 categories (+*G*, parameter = 0.1550)). The tree is drawn to scale, with branch lengths measured in the number of substitutions per site. The analysis involved 30 nucleotide sequences, of which 27 were used as marker sequences to determine the genotype of 3 sequences. All positions containing gaps and missing data were eliminated. There was a total of 664 positions in the final dataset. Evolutionary analyses were conducted in MEGA X.

ID	GENOTYPE	SUBTYPE	COUNTRY	TREE	ALIGNMENT[1]	BASE PAIRS
KP659219	B	B6	Alaska	1	Complete Genome	3215
KP659220	B	B6	Alaska	1	Complete Genome	3215

KP659221	B	B6	Alaska	1	Complete Genome	3038
KP659223	B	B6	Alaska	1	Complete Genome	3215
KP659224	B	B6	Alaska	1	Complete Genome	3215
KP659227	B	B6	Alaska	2	157-837	681
KP659228	B	B6	Alaska	2	157-837	681
KP659229	B	B6	Alaska	2	157-837	681

[1]Alignment to complete genome reference sequence VHB NC_003977.2

GREENLAND

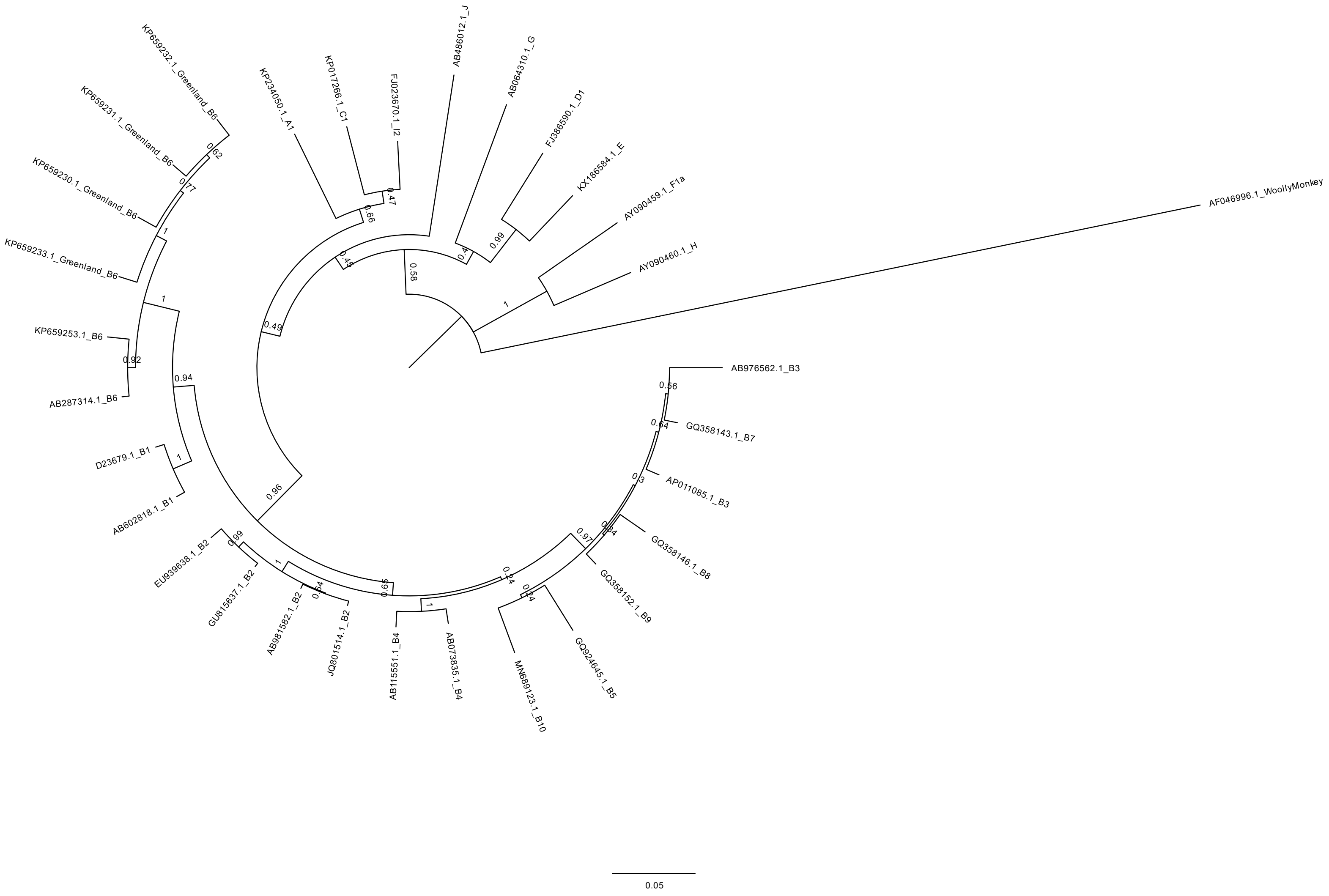

Tree 1. The evolutionary history was inferred by using the Maximum Likelihood method and Tamura-Nei model. The percentage of replicate trees in which the associated taxa clustered together in the bootstrap test (1000 replicates) are shown next to the branches. Initial tree(s) for the heuristic search were obtained automatically by applying Neighbor-Join and BioNJ algorithms to a matrix of pairwise distances estimated using the Tamura-Nei model, and then selecting the topology with superior log likelihood value. A discrete Gamma distribution was used to model evolutionary rate differences among sites (5 categories (+*G*, parameter = 0.2719)). The tree is drawn to scale, with branch lengths measured in the number of substitutions per site. The analysis involved 31 nucleotide sequences, of which 27 were used as marker sequences to determine the genotype of 4 sequences. All positions containing gaps and missing data were eliminated. There was a total of 2125 positions in the final dataset. Evolutionary analyses were conducted in MEGA X.

ID	GENOTYPE	SUBTYPE	COUNTRY	TREE	ALIGNMENT[1]	BASE PAIRS
KP659230	B	B6	Greenland	1	1-3182	3215
KP659231	B	B6	Greenland	1	1-3182	3215
KP659232	B	B6	Greenland	1	1-3182	3215
KP659233	B	B6	Greenland	1	1-3182	3215

[1]Alignment to complete genome reference sequence VHB NC_003977.2

CANADA

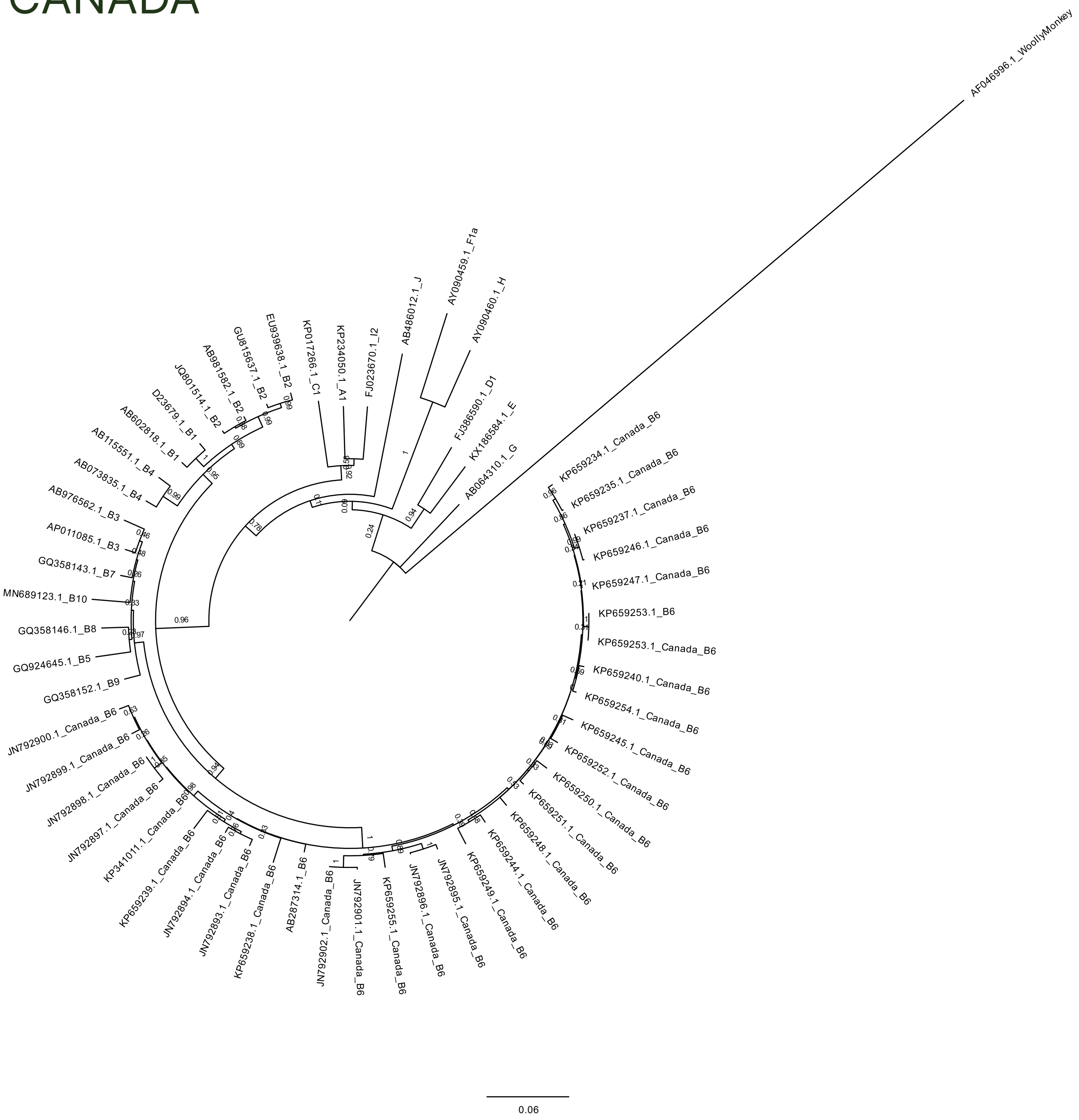

Tree 1. The evolutionary history was inferred by using the Maximum Likelihood method and Tamura-Nei model. The percentage of replicate trees in which the associated taxa clustered together in the bootstrap test (1000 replicates) are shown next to the branches. Initial tree(s) for the heuristic search were obtained automatically by applying Neighbor-Join and BioNJ algorithms to a matrix of pairwise distances estimated using the Tamura-Nei model, and then selecting the topology with superior log likelihood value. A discrete Gamma distribution was used to model evolutionary rate differences among sites (5 categories (+*G*, parameter = 0.2703)). The tree is drawn to scale, with branch lengths measured in the number of substitutions per site. The analysis involved 56 nucleotide sequences, of which 27 were used as marker sequences to determine the genotype of 29 sequences. All positions containing gaps and missing data were eliminated. There was a total of 2469 positions in the final dataset. Evolutionary analyses were conducted in MEGA X.

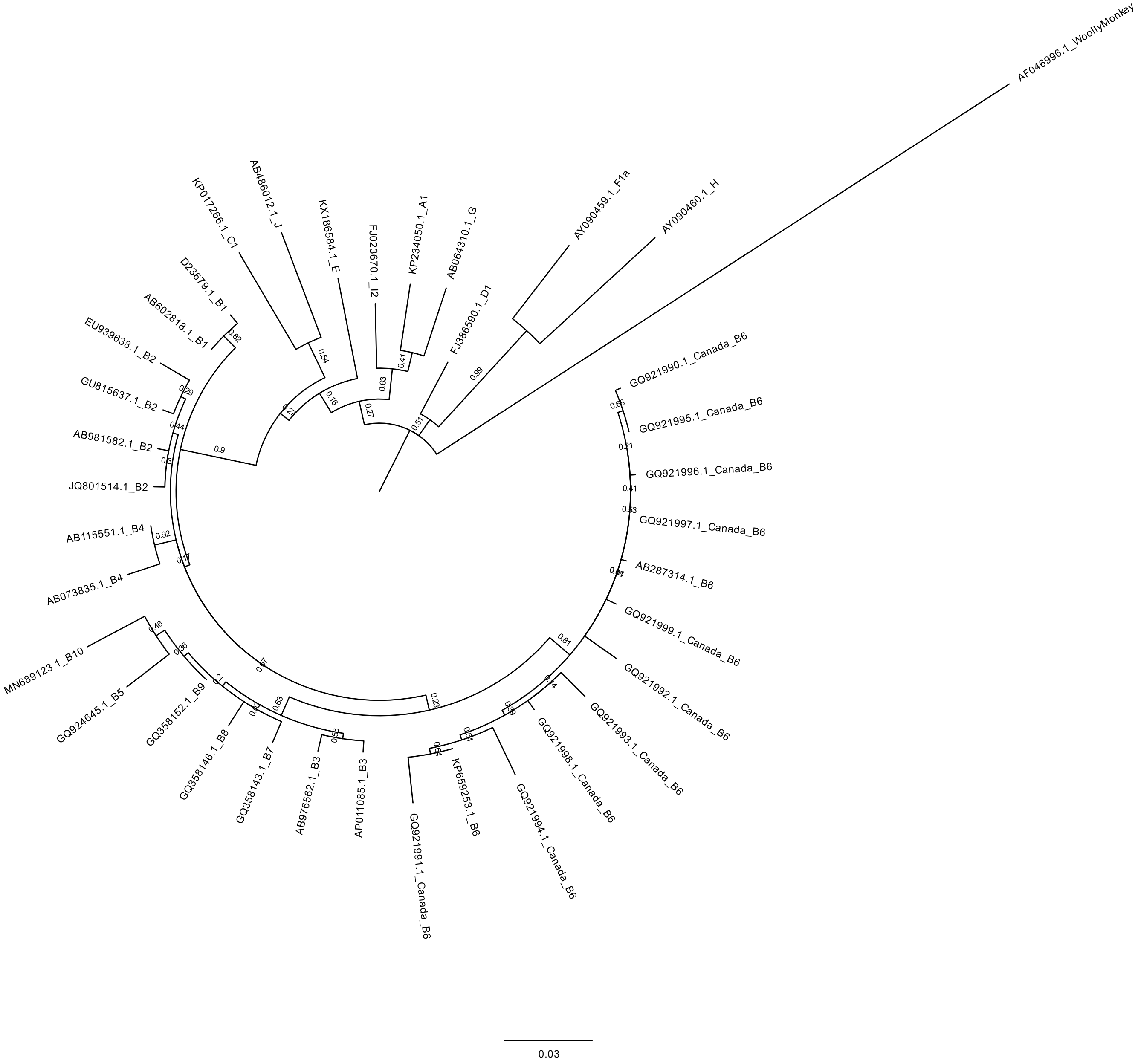

Tree 2. The evolutionary history was inferred by using the Maximum Likelihood method and Tamura-Nei model. The percentage of replicate trees in which the associated taxa clustered together in the bootstrap test (1000 replicates) are shown next to the branches. Initial tree(s) for the heuristic search were obtained automatically by applying Neighbor-Join and BioNJ algorithms to a matrix of pairwise distances estimated using the Tamura-Nei model, and then selecting the topology with superior log likelihood value. A discrete Gamma distribution was used to model evolutionary rate differences among sites (5 categories (+G, parameter = 0.1898)). The tree is drawn to scale, with branch lengths measured in the number of substitutions per site. The analysis involved 37 nucleotide sequences, of which 27 were used as marker sequences to determine the genotype of 10 sequences. All positions containing gaps and missing data were eliminated. There was a total of 607 positions in the final dataset. Evolutionary analyses were conducted in MEGA X.

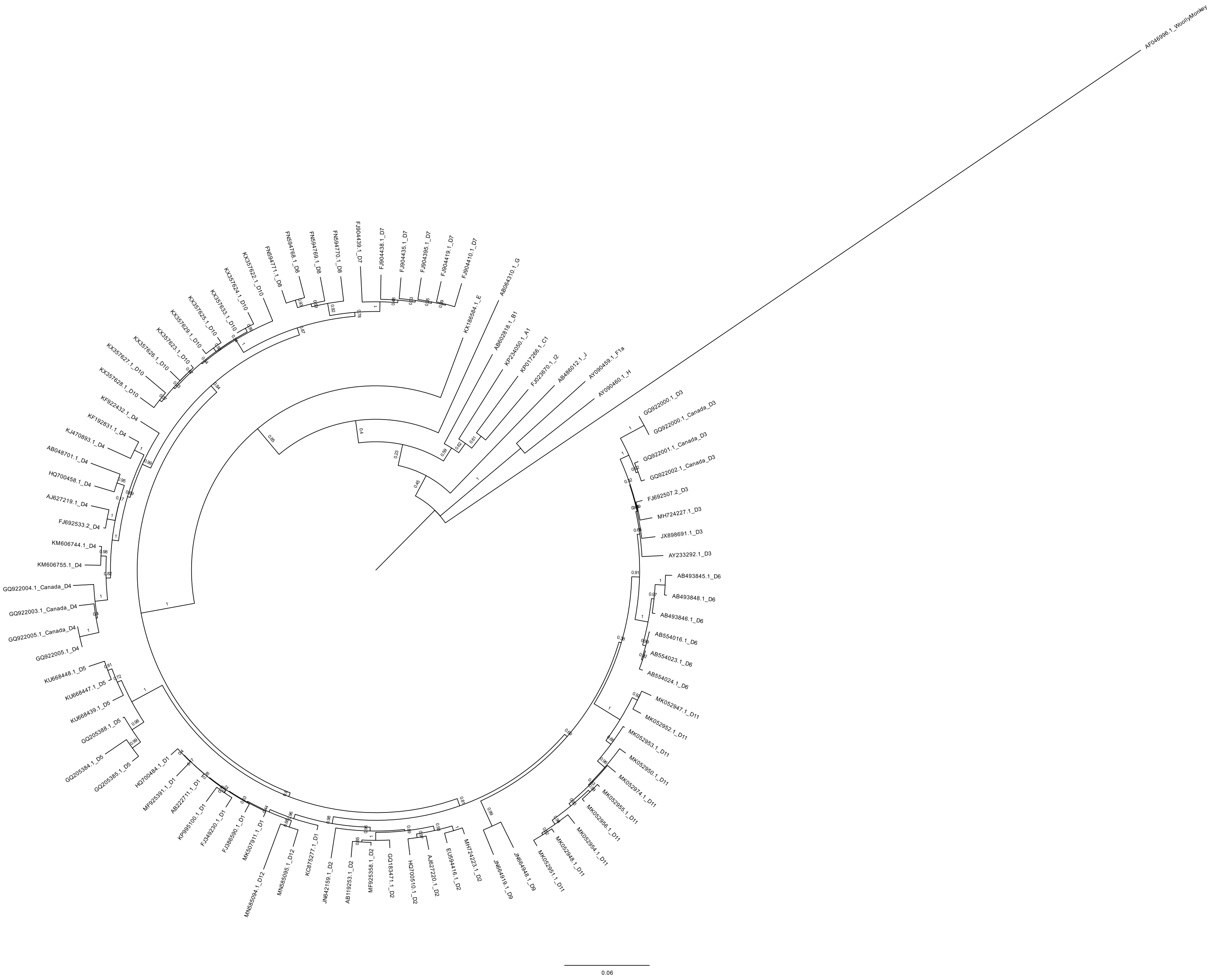

Tree 3. The evolutionary history was inferred by using the Maximum Likelihood method and Tamura-Nei model. The percentage of replicate trees in which the associated taxa clustered together in the bootstrap test (1000 replicates) are shown next to the branches. Initial tree(s) for the heuristic search were obtained automatically by applying Neighbor-Join and BioNJ algorithms to a matrix of pairwise distances estimated using the Tamura-Nei model, and then selecting the topology with superior log likelihood value. A discrete Gamma distribution was used to model evolutionary rate differences among sites (5 categories (+*G*, parameter = 0.2782)). The tree is drawn to scale, with branch lengths measured in the number of substitutions per site. The analysis involved 92 nucleotide sequences, of which 86 were used as marker sequences to determine the genotype of 6 sequences. All positions containing gaps and missing data were eliminated. There was a total of 3054 positions in the final dataset. Evolutionary analyses were conducted in MEGA X.

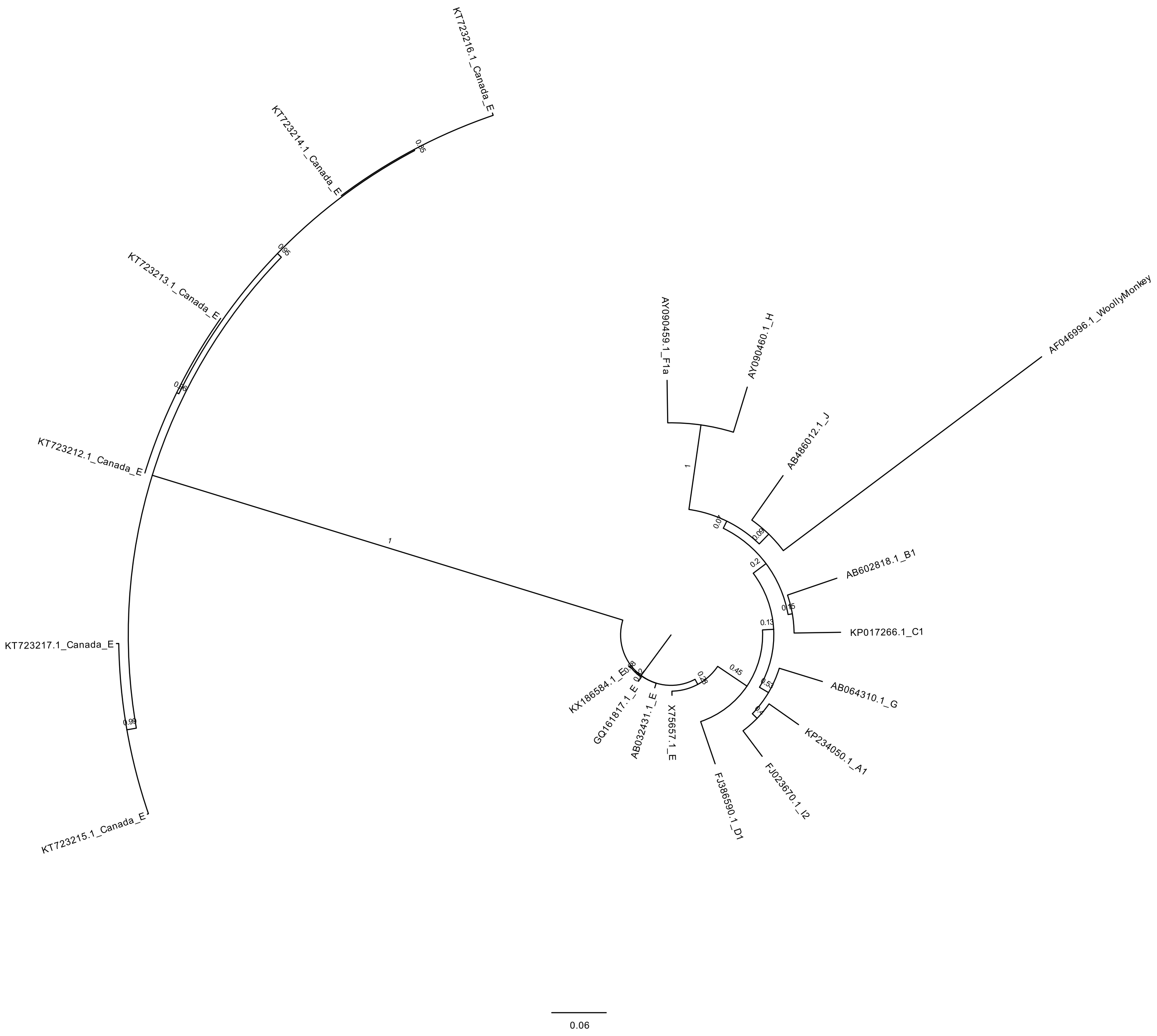

Tree 4. The evolutionary history was inferred by using the Maximum Likelihood method and Tamura-Nei model. The percentage of replicate trees in which the associated taxa clustered together in the bootstrap test (1000 replicates) are shown next to the branches. Initial tree(s) for the heuristic search were obtained automatically by applying Neighbor-Join and BioNJ algorithms to a matrix of pairwise distances estimated using the Tamura-Nei model, and then selecting the topology with superior log likelihood value. A discrete Gamma distribution was used to model evolutionary rate differences among sites (5 categories (+*G*, parameter = 0.3458)). The tree is drawn to scale, with branch lengths measured in the number of substitutions per site. The analysis involved 20 nucleotide sequences, of which 14 were used as marker sequences to determine the genotype of 6 sequences. All positions containing gaps and missing data were eliminated. There was a total of 840 positions in the final dataset. Evolutionary analyses were conducted in MEGA X.

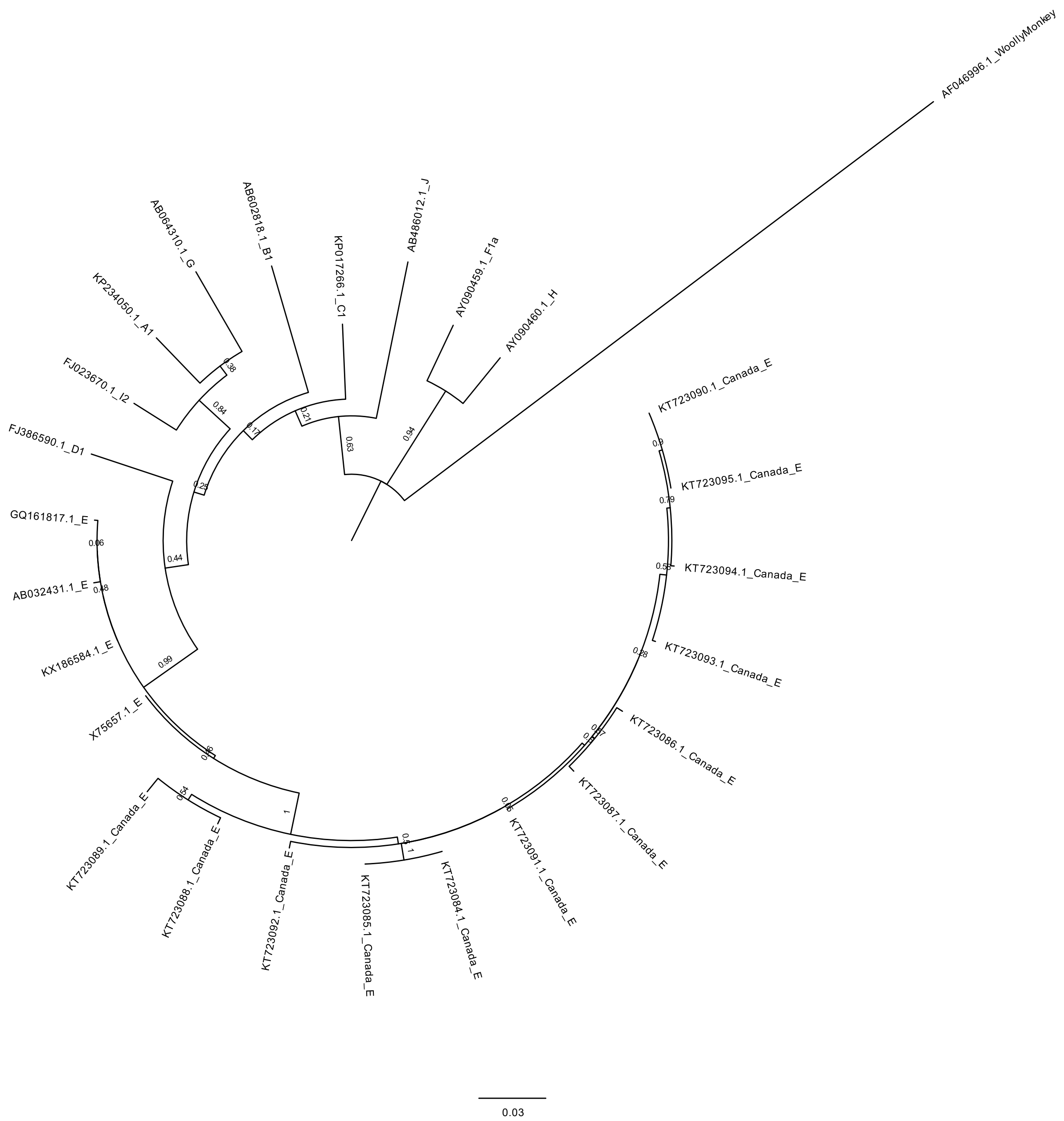

Tree 5. The evolutionary history was inferred by using the Maximum Likelihood method and Tamura-Nei model. The percentage of replicate trees in which the associated taxa clustered together in the bootstrap test (1000 replicates) are shown next to the branches. Initial tree(s) for the heuristic search were obtained automatically by applying Neighbor-Join and BioNJ algorithms to a matrix of pairwise distances estimated using the Tamura-Nei model, and then selecting the topology with superior log likelihood value. A discrete Gamma distribution was used to model evolutionary rate differences among sites (5 categories (+*G*, parameter = 0.2829)). The tree is drawn to scale, with branch lengths measured in the number of substitutions per site. The analysis involved 26 nucleotide sequences, of which 14 were used as marker sequences to determine the genotype of 12 sequences. All positions containing gaps and missing data were eliminated. There was a total of 662 positions in the final dataset. Evolutionary analyses were conducted in MEGA X.

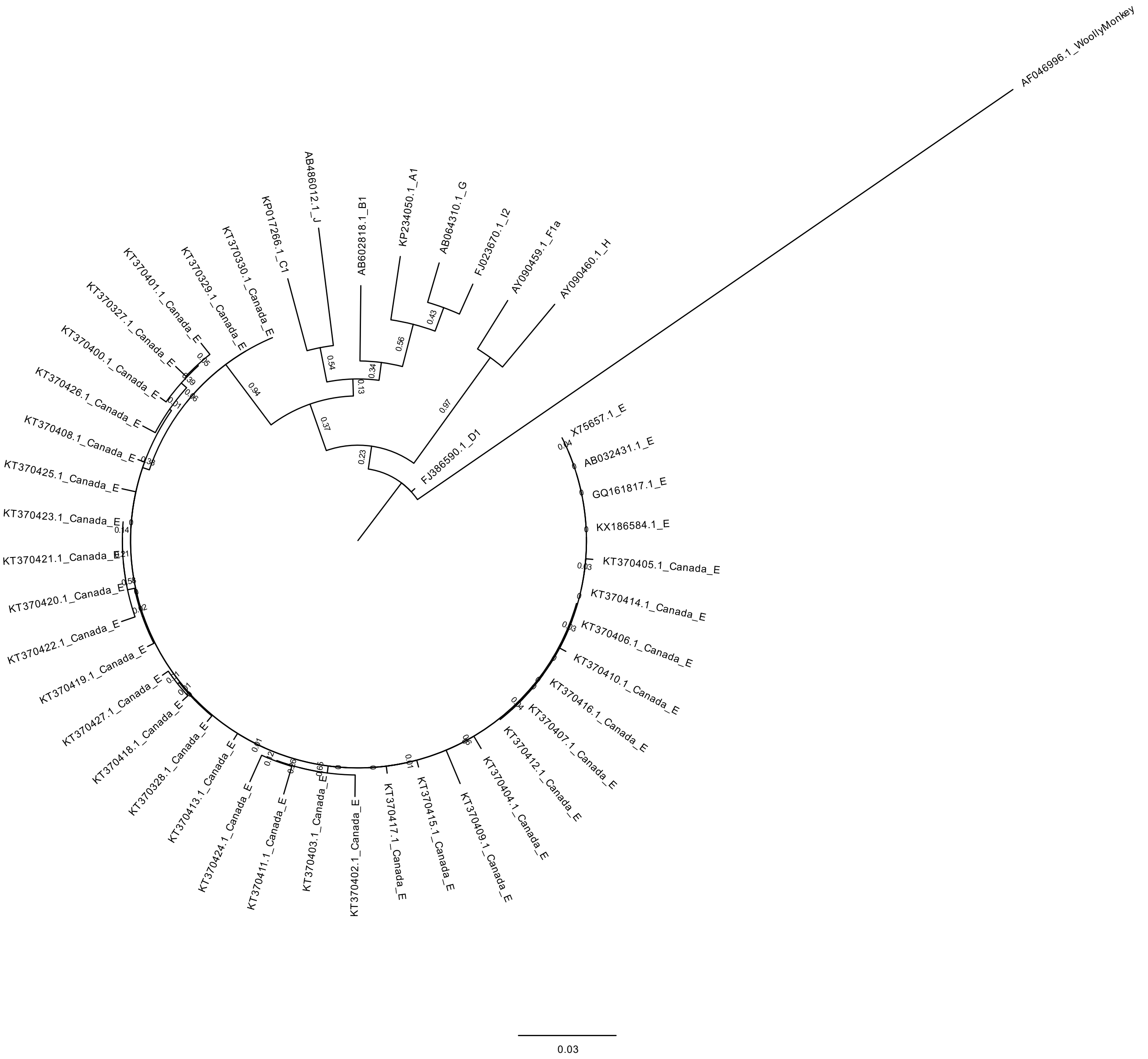

Tree 6. The evolutionary history was inferred by using the Maximum Likelihood method and Tamura-Nei model. The percentage of replicate trees in which the associated taxa clustered together in the bootstrap test (1000 replicates) are shown next to the branches. Initial tree(s) for the heuristic search were obtained automatically by applying Neighbor-Join and BioNJ algorithms to a matrix of pairwise distances estimated using the Tamura-Nei model, and then selecting the topology with superior log likelihood value. A discrete Gamma distribution was used to model evolutionary rate differences among sites (5 categories (+*G*, parameter = 0.1665)). The tree is drawn to scale, with branch lengths measured in the number of substitutions per site. The analysis involved 46 nucleotide sequences, of which 14 were used as marker sequences to determine the genotype of 32 sequences. All positions containing gaps and missing data were eliminated. There was a total of 504 positions in the final dataset. Evolutionary analyses were conducted in MEGA X.

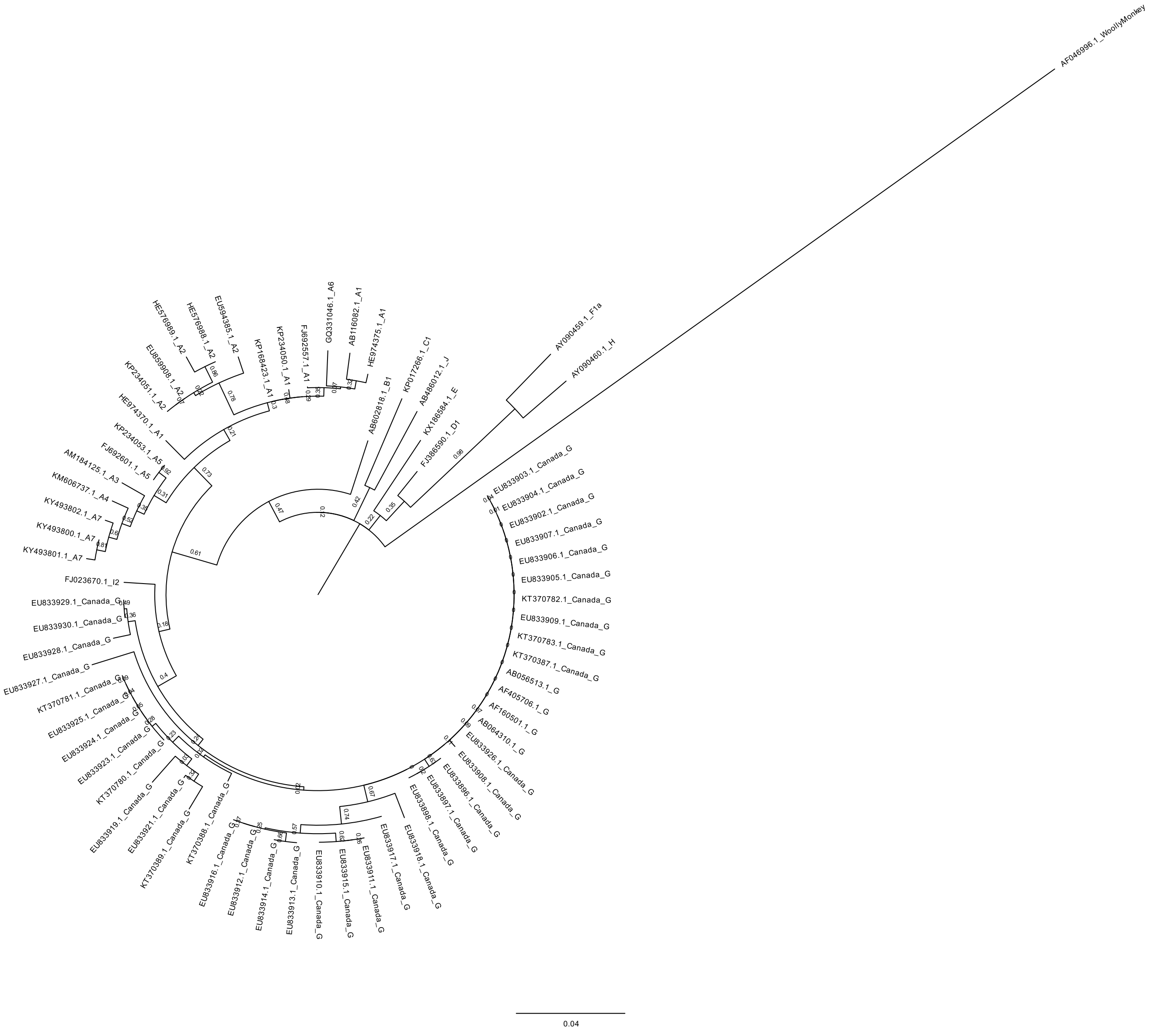

Tree 7. The evolutionary history was inferred by using the Maximum Likelihood method and Tamura-Nei model. The percentage of replicate trees in which the associated taxa clustered together in the bootstrap test (1000 replicates) are shown next to the branches. Initial tree(s) for the heuristic search were obtained automatically by applying Neighbor-Join and BioNJ algorithms to a matrix of pairwise distances estimated using the Tamura-Nei model, and then selecting the topology with superior log likelihood value. A discrete Gamma distribution was used to model evolutionary rate differences among sites (5 categories (+*G*, parameter = 0.1838)). The tree is drawn to scale, with branch lengths measured in the number of substitutions per site. The analysis involved 69 nucleotide sequences, of which 32 were used as marker sequences to determine the genotype of 37 sequences. All positions containing gaps and missing data were eliminated. There was a total of 392 positions in the final dataset. Evolutionary analyses were conducted in MEGA X.

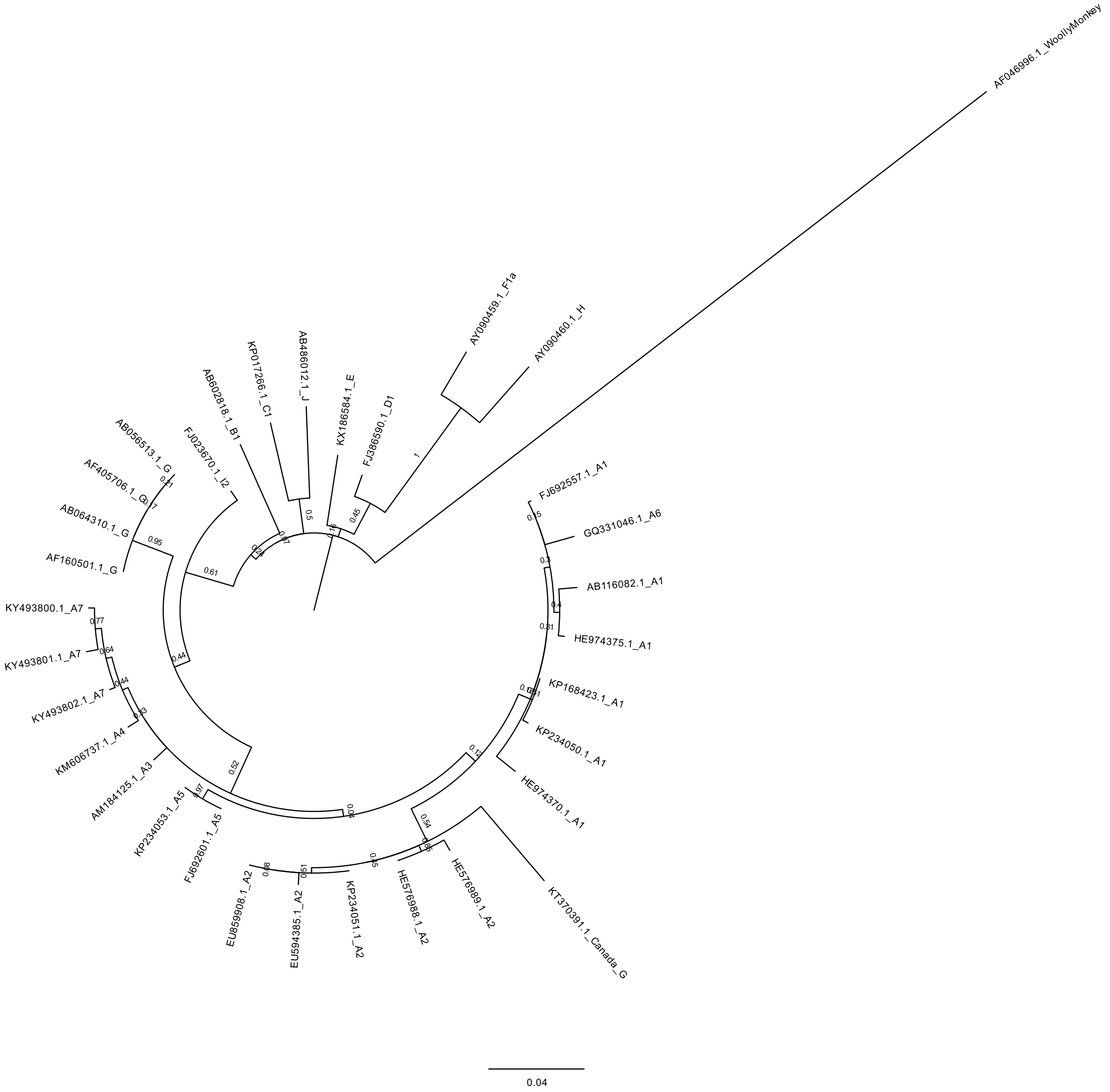

Tree 8. The evolutionary history was inferred by using the Maximum Likelihood method and Tamura-Nei model. The percentage of replicate trees in which the associated taxa clustered together in the bootstrap test (1000 replicates) are shown next to the branches. Initial tree(s) for the heuristic search were obtained automatically by applying Neighbor-Join and BioNJ algorithms to a matrix of pairwise distances estimated using the Tamura-Nei model, and then selecting the topology with superior log likelihood value. A discrete Gamma distribution was used to model evolutionary rate differences among sites (5 categories (+*G*, parameter = 0.1553)). The tree is drawn to scale, with branch lengths measured in the number of substitutions per site. The analysis involved 33 nucleotide sequences, of which 32 were used as marker sequences to determine the genotype of 1 sequence. Although originally labeled as genotype G, the sequence was classified under genotype A2 on our trees. All positions containing gaps and missing data were eliminated. There was a total of 509 positions in the final dataset. Evolutionary analyses were conducted in MEGA X.

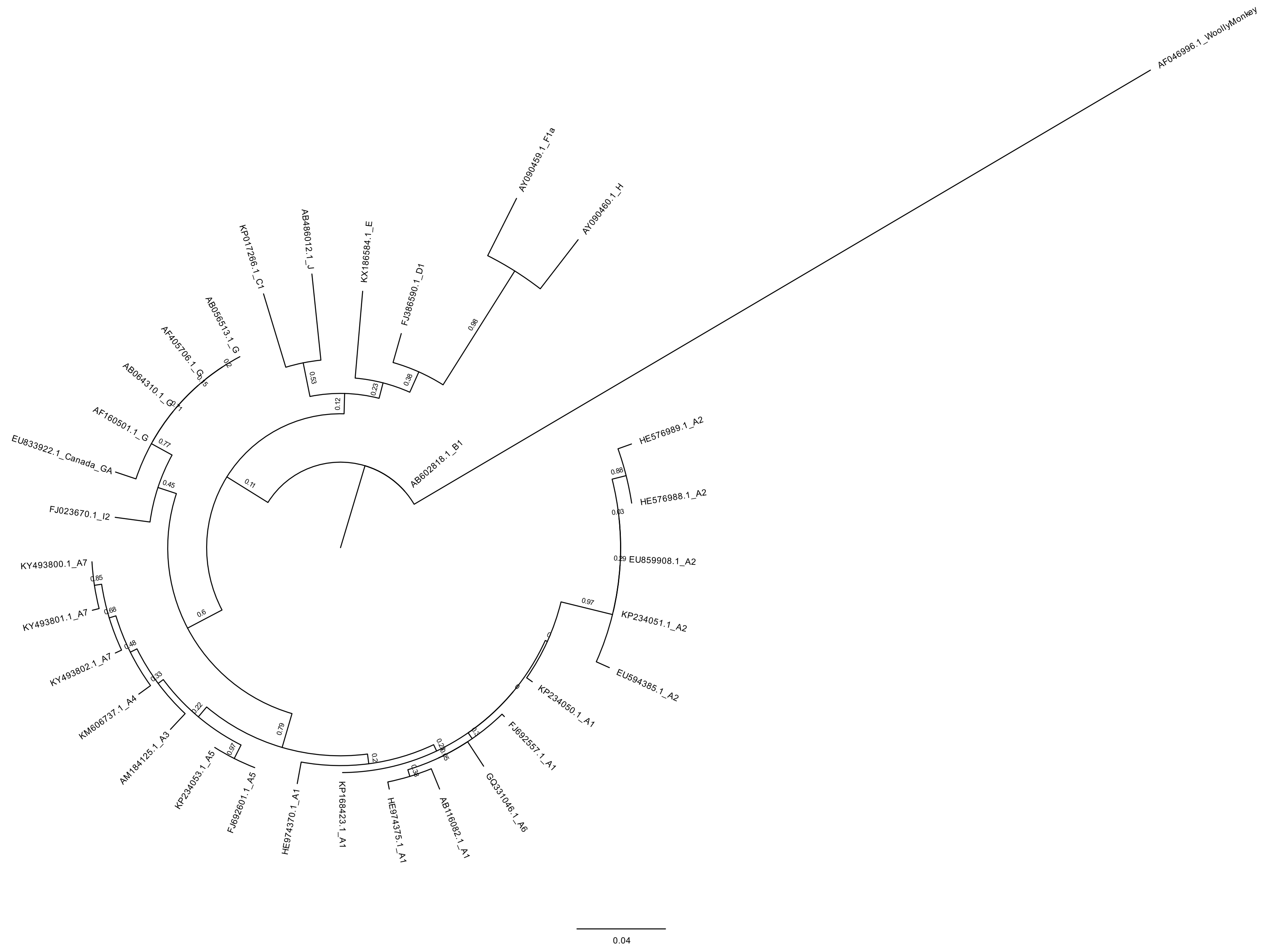

Tree 9. The evolutionary history was inferred by using the Maximum Likelihood method and Tamura-Nei model. The percentage of replicate trees in which the associated taxa clustered together in the bootstrap test (1000 replicates) are shown next to the branches. Initial tree(s) for the heuristic search were obtained automatically by applying Neighbor-Join and BioNJ algorithms to a matrix of pairwise distances estimated using the Tamura-Nei model, and then selecting the topology with superior log likelihood value. A discrete Gamma distribution was used to model evolutionary rate differences among sites (5 categories (+*G*, parameter = 0.1561)). The tree is drawn to scale, with branch lengths measured in the number of substitutions per site. The analysis involved 33 nucleotide sequences, of which 32 were used as marker sequences to determine the genotype of 1 sequence. Originally labeled as a natural G/A recombinant, the tree we created corresponds with this conclusion. All positions containing gaps and missing data were eliminated. There was a total of 401 positions in the final dataset. Evolutionary analyses were conducted in MEGA X.

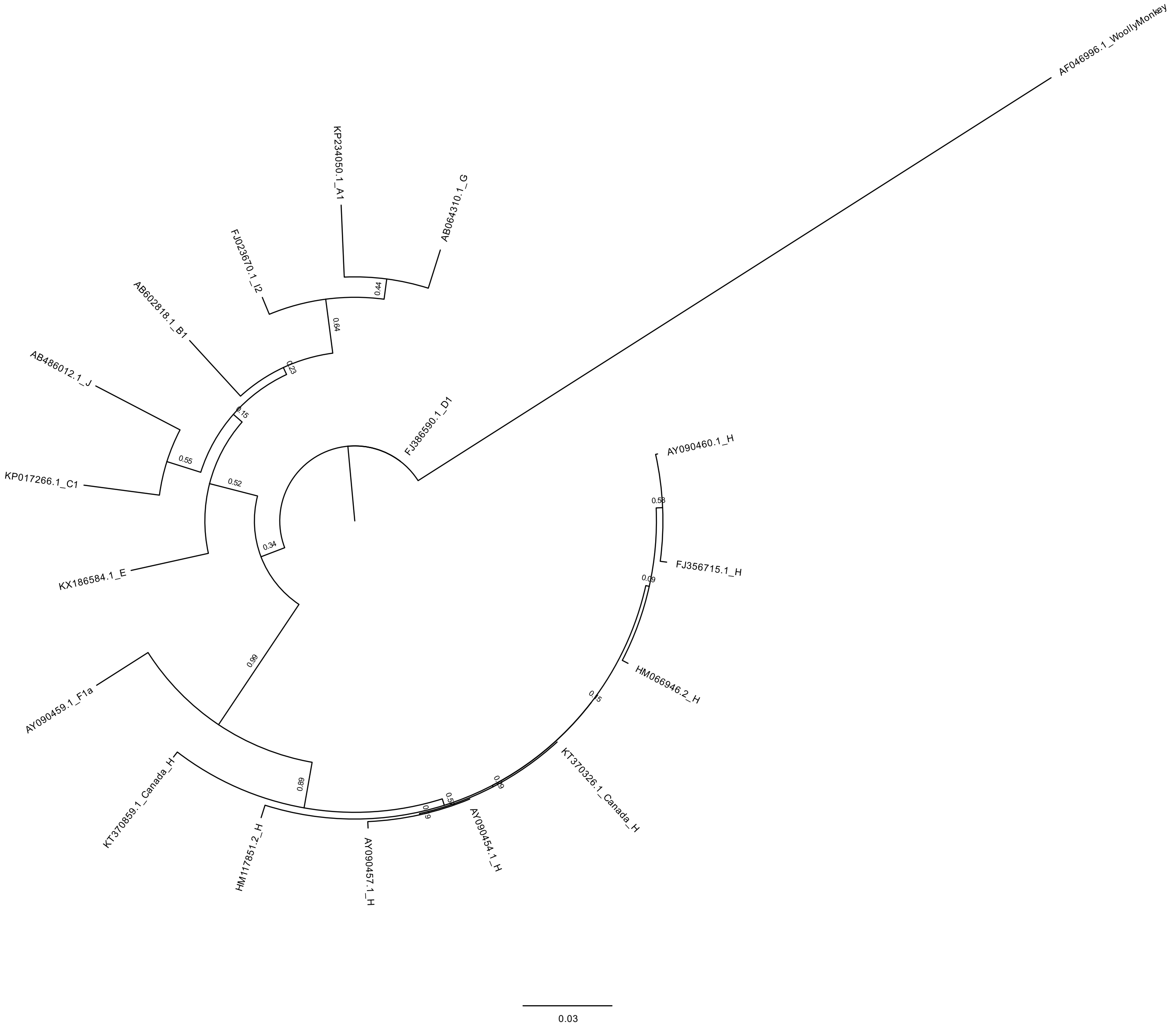

Tree 9. The evolutionary history was inferred by using the Maximum Likelihood method and Tamura-Nei model. The percentage of replicate trees in which the associated taxa clustered together in the bootstrap test (1000 replicates) are shown next to the branches. Initial tree(s) for the heuristic search were obtained automatically by applying Neighbor-Join and BioNJ algorithms to a matrix of pairwise distances estimated using the Tamura-Nei model, and then selecting the topology with superior log likelihood value. A discrete Gamma distribution was used to model evolutionary rate differences among sites (5 categories (+*G*, parameter = 0.1300)). The tree is drawn to scale, with branch lengths measured in the number of substitutions per site. The analysis involved 18 nucleotide sequences, of which 16 were used as marker sequences to determine the genotype of 2 sequences. All positions containing gaps and missing data were eliminated. There was a total of 499 positions in the final dataset. Evolutionary analyses were conducted in MEGA X.

ID	GENOTYPE	SUBTYPE	COUNTRY	TREE	ALIGNMENT[1]	BASE PAIRS
JN792893	B	B6	Canada	1	Complete Genome	3215
JN792894	B	B6	Canada	1	Complete Genome	3215

JN792895	B	B6	Canada	1	Complete Genome	3215
JN792896	B	B6	Canada	1	Complete Genome	3215
JN792897	B	B6	Canada	1	Complete Genome	3215
JN792898	B	B6	Canada	1	Complete Genome	3215
JN792899	B	B6	Canada	1	Complete Genome	3215
JN792900	B	B6	Canada	1	Complete Genome	3215
JN792901	B	B6	Canada	1	Complete Genome	3215
JN792902	B	B6	Canada	1	Complete Genome	3215
KP341011	B	B6	Canada	1	Complete Genome	3215
KP659234	B	B6	Canada	1	Complete Genome	3215
KP659235	B	B6	Canada	1	Complete Genome	3215
KP659237	B	B6	Canada	1	Complete Genome	3215
KP659238	B	B6	Canada	1	Complete Genome	3215
KP659239	B	B6	Canada	1	Complete Genome	3215
KP659240	B	B6	Canada	1	Complete Genome	3215
KP659244	B	B6	Canada	1	Complete Genome	3215
KP659245	B	B6	Canada	1	Complete Genome	3215
KP659246	B	B6	Canada	1	Complete Genome	3215
KP659247	B	B6	Canada	1	Complete Genome	3215
KP659248	B	B6	Canada	1	Complete Genome	3215
KP659249	B	B6	Canada	1	Complete Genome	3215
KP659250	B	B6	Canada	1	Complete Genome	3215
KP659251	B	B6	Canada	1	Complete Genome	3215
KP659252	B	B6	Canada	1	Complete Genome	3215
KP659253	B	B6	Canada	1	Complete Genome	3215
KP659254	B	B6	Canada	1	Complete Genome	3215
KP659255	B	B6	Canada	1	Complete Genome	3215
GQ921990	B	B6	Canada	2	266-888	623
GQ921991	B	B6	Canada	2	266-888	623
GQ921992	B	B6	Canada	2	266-888	623
GQ921993	B	B6	Canada	2	266-888	623
GQ921994	B	B6	Canada	2	266-888	623
GQ921995	B	B6	Canada	2	266-888	623
GQ921996	B	B6	Canada	2	266-888	623
GQ921997	B	B6	Canada	2	266-888	623
GQ921998	B	B6	Canada	2	266-888	623
GQ921999	B	B6	Canada	2	266-888	623
GQ922000	D	D3	Canada	3	Complete Genome	3182
GQ922001	D	D3	Canada	3	Complete Genome	3182
GQ922002	D	D3	Canada	3	Complete Genome	3182
GQ922003	D	D4	Canada	3	Complete Genome	3182

GQ922004	D	D4	Canada	3	Complete Genome	3182
GQ922005	D	D4	Canada	3	Complete Genome	3182
KT723212	E	E	Canada	4	1-3182	849
KT723213	E	E	Canada	4	1-3182	961
KT723214	E	E	Canada	4	1-3182	961
KT723215	E	E	Canada	4	1-3182	964
KT723216	E	E	Canada	4	1-3182	961
KT723217	E	E	Canada	4	1-3182	964
KT723084	E	E	Canada	5	1-3182	665
KT723085	E	E	Canada	5	1-3182	665
KT723086	E	E	Canada	5	1-3182	665
KT723087	E	E	Canada	5	1-3182	665
KT723088	E	E	Canada	5	1-3182	662
KT723089	E	E	Canada	5	1-3182	662
KT723090	E	E	Canada	5	1-3182	665
KT723091	E	E	Canada	5	1-3182	665
KT723092	E	E	Canada	5	1-3182	665
KT723093	E	E	Canada	5	1-3182	665
KT723094	E	E	Canada	5	1-3182	665
KT723095	E	E	Canada	5	1-3182	665
KT370327	E	E	Canada	6	313-831	519
KT370328	E	E	Canada	6	313-831	519
KT370329	E	E	Canada	6	313-831	519
KT370330	E	E	Canada	6	313-831	519
KT370400	E	E	Canada	6	313-831	519
KT370401	E	E	Canada	6	313-831	519
KT370402	E	E	Canada	6	313-828	516
KT370403	E	E	Canada	6	313-831	519
KT370404	E	E	Canada	6	313-831	519
KT370405	E	E	Canada	6	313-831	519
KT370406	E	E	Canada	6	313-831	519
KT370407	E	E	Canada	6	313-831	519
KT370408	E	E	Canada	6	313-831	519
KT370409	E	E	Canada	6	313-831	519
KT370410	E	E	Canada	6	313-831	519
KT370411	E	E	Canada	6	313-831	519
KT370412	E	E	Canada	6	313-831	519
KT370413	E	E	Canada	6	313-831	519
KT370414	E	E	Canada	6	313-831	519
KT370415	E	E	Canada	6	313-831	519
KT370416	E	E	Canada	6	313-831	519

KT370417	E	E	Canada	6	313-831	519
KT370418	E	E	Canada	6	313-831	519
KT370419	E	E	Canada	6	313-831	519
KT370420	E	E	Canada	6	313-831	519
KT370421	E	E	Canada	6	313-831	519
KT370422	E	E	Canada	6	313-831	519
KT370423	E	E	Canada	6	313-831	519
KT370424	E	E	Canada	6	313-831	519
KT370425	E	E	Canada	6	313-831	519
KT370426	E	E	Canada	6	313-831	519
KT370427	E	E	Canada	6	313-831	519
EU833896	G	G	Canada	7	417-827	411
EU833897	G	G	Canada	7	417-827	411
EU833898	G	G	Canada	7	417-827	411
EU833902	G	G	Canada	7	417-827	411
EU833903	G	G	Canada	7	417-827	411
EU833904	G	G	Canada	7	417-827	411
EU833905	G	G	Canada	7	417-827	411
EU833906	G	G	Canada	7	417-827	411
EU833907	G	G	Canada	7	417-827	411
EU833908	G	G	Canada	7	417-827	411
EU833909	G	G	Canada	7	417-827	411
EU833910	G	G	Canada	7	417-827	411
EU833911	G	G	Canada	7	417-827	411
EU833912	G	G	Canada	7	417-827	411
EU833913	G	G	Canada	7	417-827	411
EU833914	G	G	Canada	7	417-827	411
EU833915	G	G	Canada	7	417-827	411
EU833916	G	G	Canada	7	417-827	411
EU833917	G	G	Canada	7	417-827	411
EU833918	G	G	Canada	7	417-827	411
EU833919	G	G	Canada	7	417-827	411
EU833921	G	G	Canada	7	417-827	411
EU833923	G	G	Canada	7	417-827	411
EU833924	G	G	Canada	7	417-827	411
EU833925	G	G	Canada	7	417-827	411
EU833926	G	G	Canada	7	417-827	411
EU833927	G	G	Canada	7	417-827	411
EU833928	G	G	Canada	7	417-827	411
EU833929	G	G	Canada	7	417-827	411
EU833930	G	G	Canada	7	417-827	411

KT370387	G	G	Canada	7	313-831	519
KT370388	G	G	Canada	7	313-831	519
KT370389	G	G	Canada	7	313-831	519
KT370780	G	G	Canada	7	313-831	519
KT370781	G	G	Canada	7	313-831	519
KT370782	G	G	Canada	7	313-831	519
KT370783	G	G	Canada	7	313-831	519
KT370391	A	A2	Canada	8	313-831	519
EU833922	G/A	G/A	Canada	9	417-827	411
KT370326	H	H	Canada	10	313-831	519
KT370859	H	H	Canada	10	330-831	502

[1]Alignment to complete genome reference sequence VHB NC_003977.2

UNITED STATES OF AMERICA

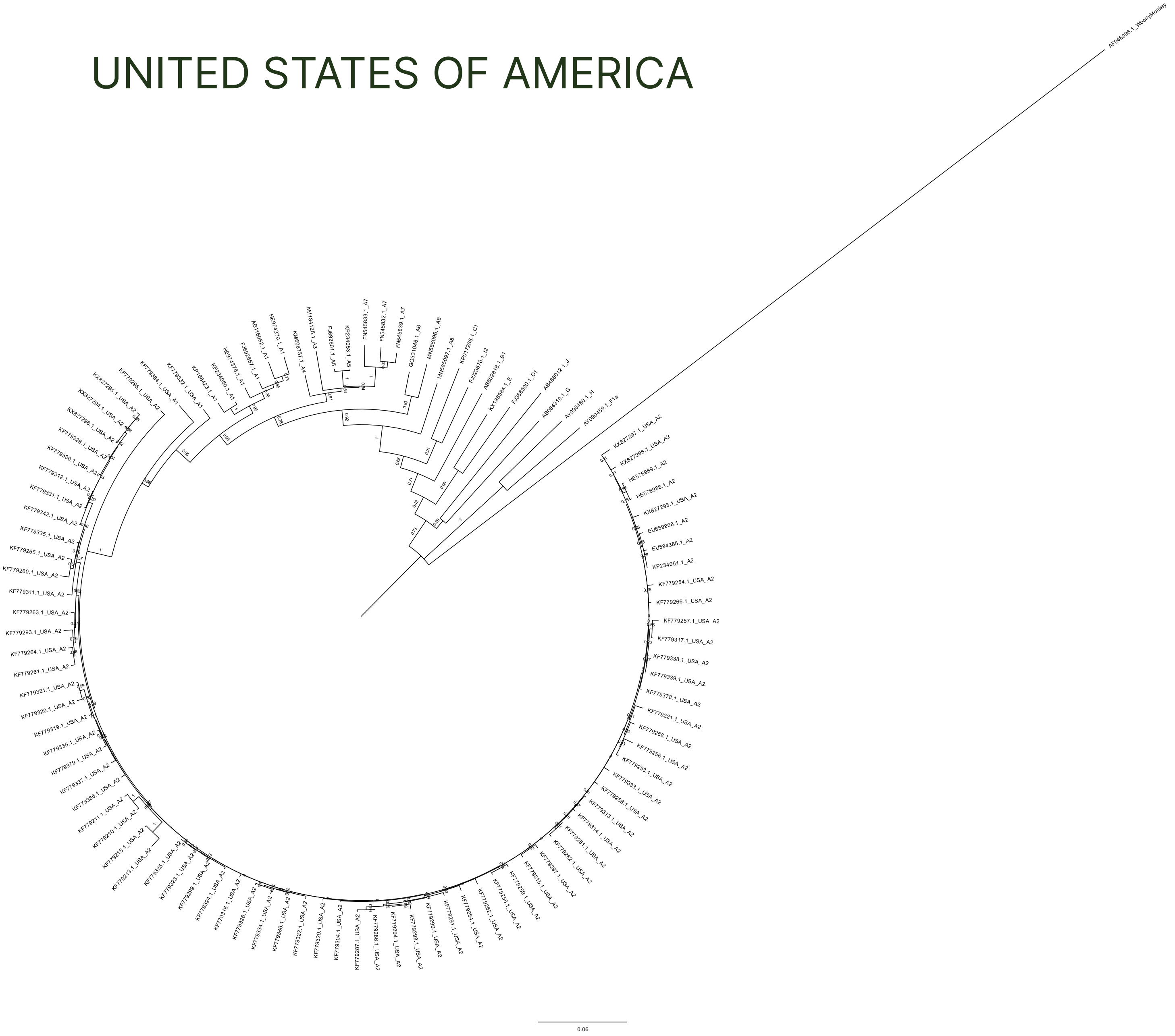

Tree 1. The evolutionary history was inferred by using the Maximum Likelihood method and Tamura-Nei model. The percentage of replicate trees in which the associated taxa clustered together in the bootstrap test (1000 replicates) are shown next to the branches. Initial tree(s) for the heuristic search were obtained automatically by applying Neighbor-Join and BioNJ algorithms to a matrix of pairwise distances estimated using the Tamura-Nei model, and then selecting the topology with superior log likelihood value. A discrete Gamma distribution was used to model evolutionary rate differences among sites (5 categories (+*G*, parameter = 0.2456)). The tree is drawn to scale, with branch lengths measured in the number of substitutions per site. The analysis involved 104 nucleotide sequences, of which 31 were used as marker sequences to determine the genotype of 73 sequences. All positions containing gaps and missing data were eliminated. There was a total of 2955 positions in the final dataset. Evolutionary analyses were conducted in MEGA X.

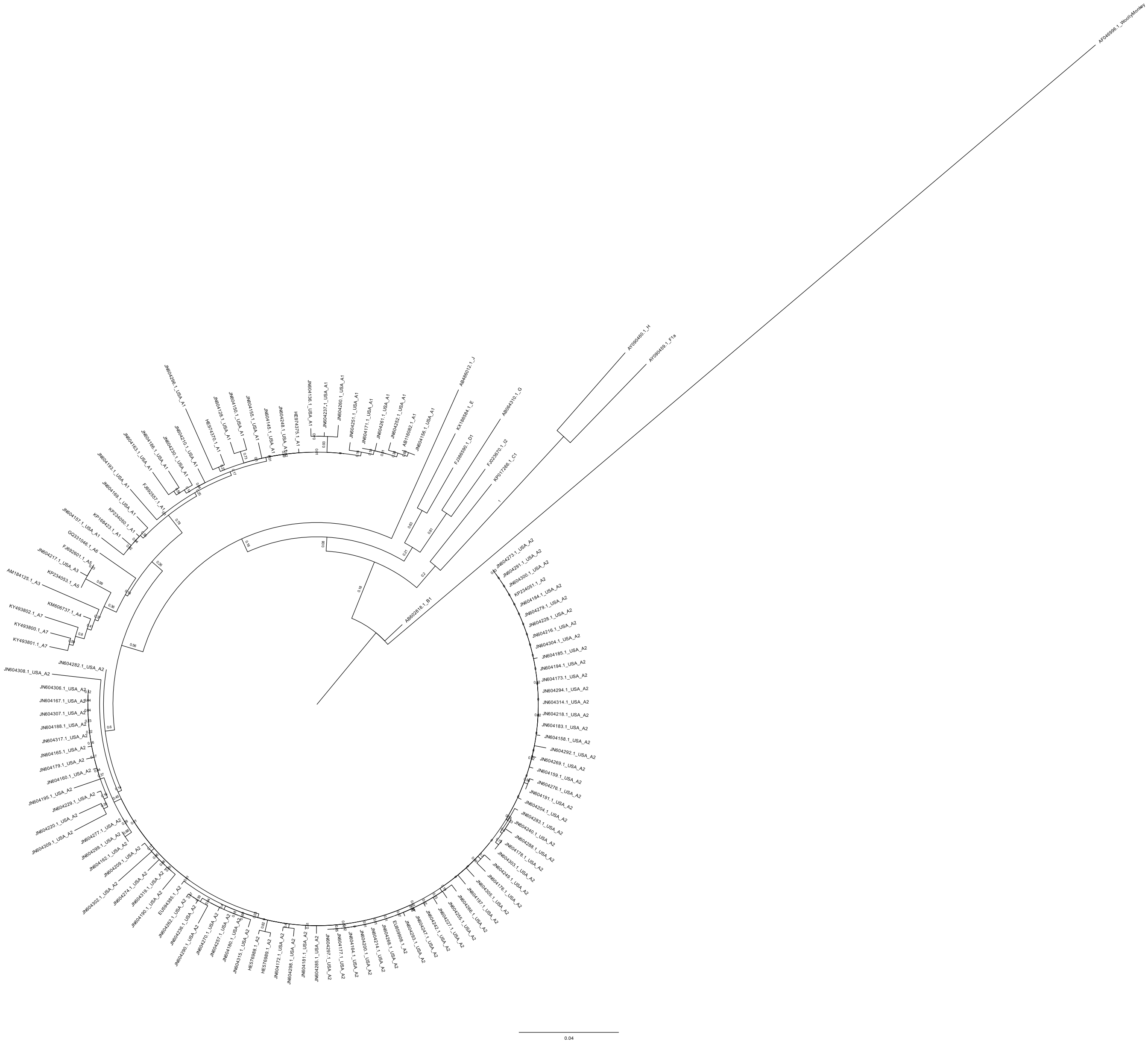

Tree 2. The evolutionary history was inferred by using the Maximum Likelihood method and Tamura-Nei model. The percentage of replicate trees in which the associated taxa clustered together in the bootstrap test (1000 replicates) are shown next to the branches. Initial tree(s) for the heuristic search were obtained automatically by applying Neighbor-Join and BioNJ algorithms to a matrix of pairwise distances estimated using the Tamura-Nei model, and then selecting the topology with superior log likelihood value. A discrete Gamma distribution was used to model evolutionary rate differences among sites (5 categories (+*G*, parameter = 0.1822)). The tree is drawn to scale, with branch lengths measured in the number of substitutions per site. The analysis involved 127 nucleotide sequences, of which 29 were used as marker sequences to determine the genotype of 98 sequences. All positions containing gaps and missing data were eliminated. There was a total of 829 positions in the final dataset. Evolutionary analyses were conducted in MEGA X.

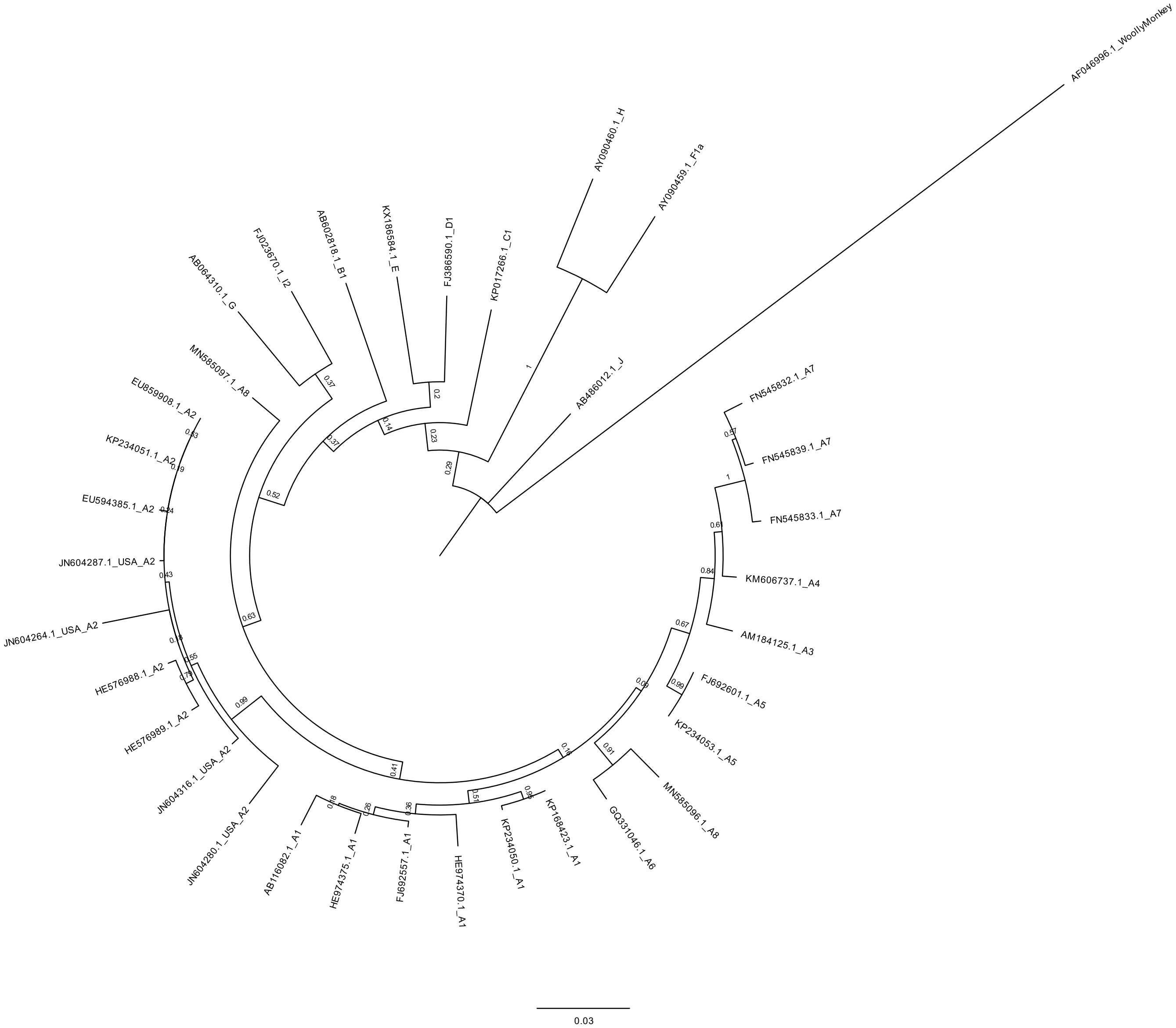

Tree 3. The evolutionary history was inferred by using the Maximum Likelihood method and Tamura-Nei model. The percentage of replicate trees in which the associated taxa clustered together in the bootstrap test (1000 replicates) are shown next to the branches. Initial tree(s) for the heuristic search were obtained automatically by applying Neighbor-Join and BioNJ algorithms to a matrix of pairwise distances estimated using the Tamura-Nei model, and then selecting the topology with superior log likelihood value. A discrete Gamma distribution was used to model evolutionary rate differences among sites (5 categories (+*G*, parameter = 0.1705)). The tree is drawn to scale, with branch lengths measured in the number of substitutions per site. The analysis involved 35 nucleotide sequences, of which 31 were used as marker sequences to determine the genotype of 4 sequences. All positions containing gaps and missing data were eliminated. There was a total of 828 positions in the final dataset. Evolutionary analyses were conducted in MEGA X.

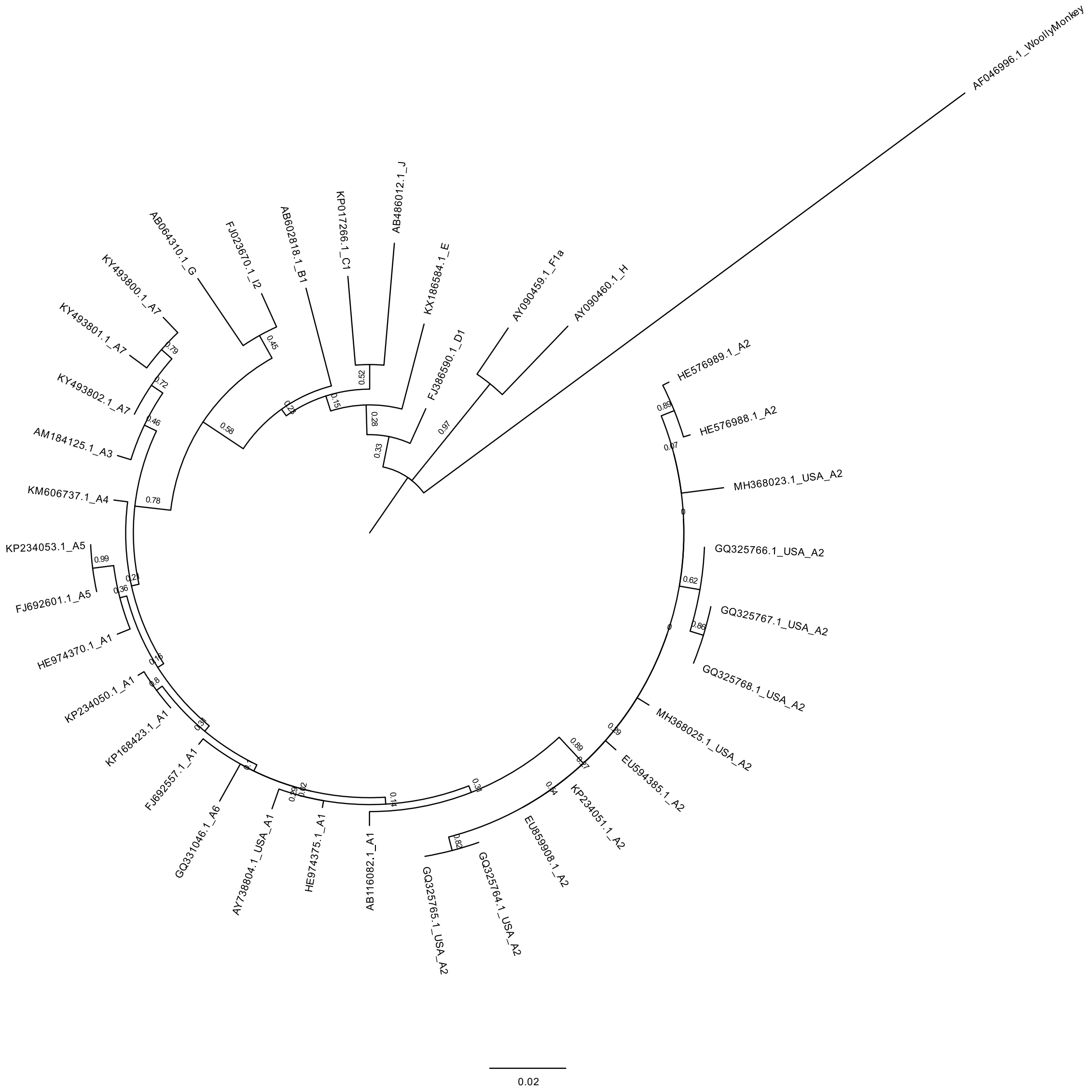

Tree 4. The evolutionary history was inferred by using the Maximum Likelihood method and Tamura-Nei model. The percentage of replicate trees in which the associated taxa clustered together in the bootstrap test (1000 replicates) are shown next to the branches. Initial tree(s) for the heuristic search were obtained automatically by applying Neighbor-Join and BioNJ algorithms to a matrix of pairwise distances estimated using the Tamura-Nei model, and then selecting the topology with superior log likelihood value. A discrete Gamma distribution was used to model evolutionary rate differences among sites (5 categories (+*G*, parameter = 0.1629)). The tree is drawn to scale, with branch lengths measured in the number of substitutions per site. The analysis involved 37 nucleotide sequences, of which 29 were used as marker sequences to determine the genotype of 8 sequences. All positions containing gaps and missing data were eliminated. There was a total of 602 positions in the final dataset. Evolutionary analyses were conducted in MEGA X.

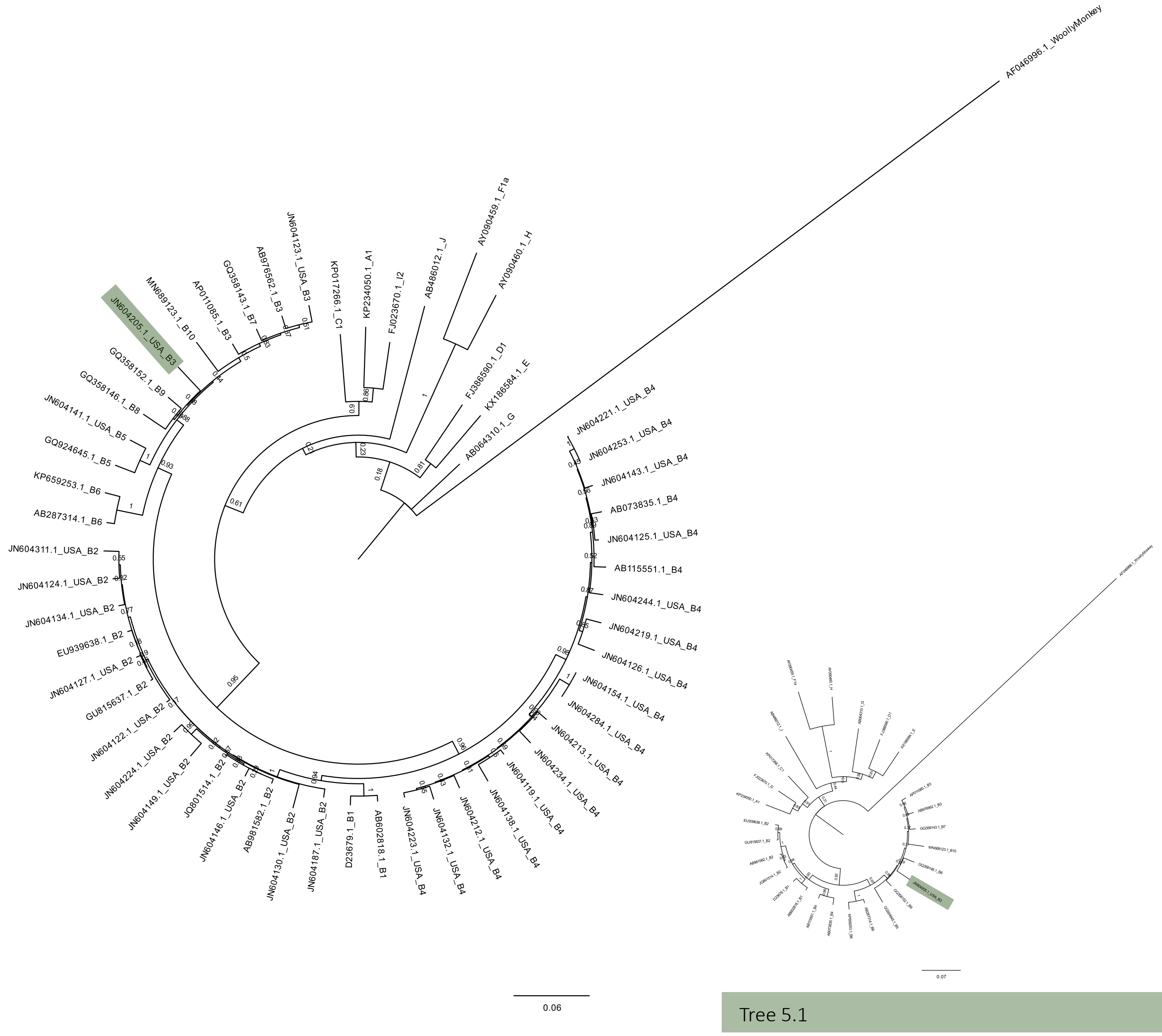

Tree 5.1

Tree 5. The evolutionary history was inferred by using the Maximum Likelihood method and Tamura-Nei model. The percentage of replicate trees in which the associated taxa clustered together in the bootstrap test (1000 replicates) are shown next to the branches. Initial tree(s) for the heuristic search were obtained automatically by applying Neighbor-Join and BioNJ algorithms to a matrix of pairwise distances estimated using the Tamura-Nei model, and then selecting the topology with superior log likelihood value. A discrete Gamma distribution was used to model evolutionary rate differences among sites (5 categories (+*G*, parameter = 0.2697)). The tree is drawn to scale, with branch lengths measured in the number of substitutions per site. The analysis involved 56 nucleotide sequences, of which 27 were used as marker sequences to determine the genotype of 29 sequences. Sequence JN604205 was categorized differently in our tree, upon further analysis in a separate tree (Tree 5.1) it was determined that it is subgenotype B8. All positions containing gaps and missing data were eliminated. There was a total of 1873 positions in the final dataset. Evolutionary analyses were conducted in MEGA X.

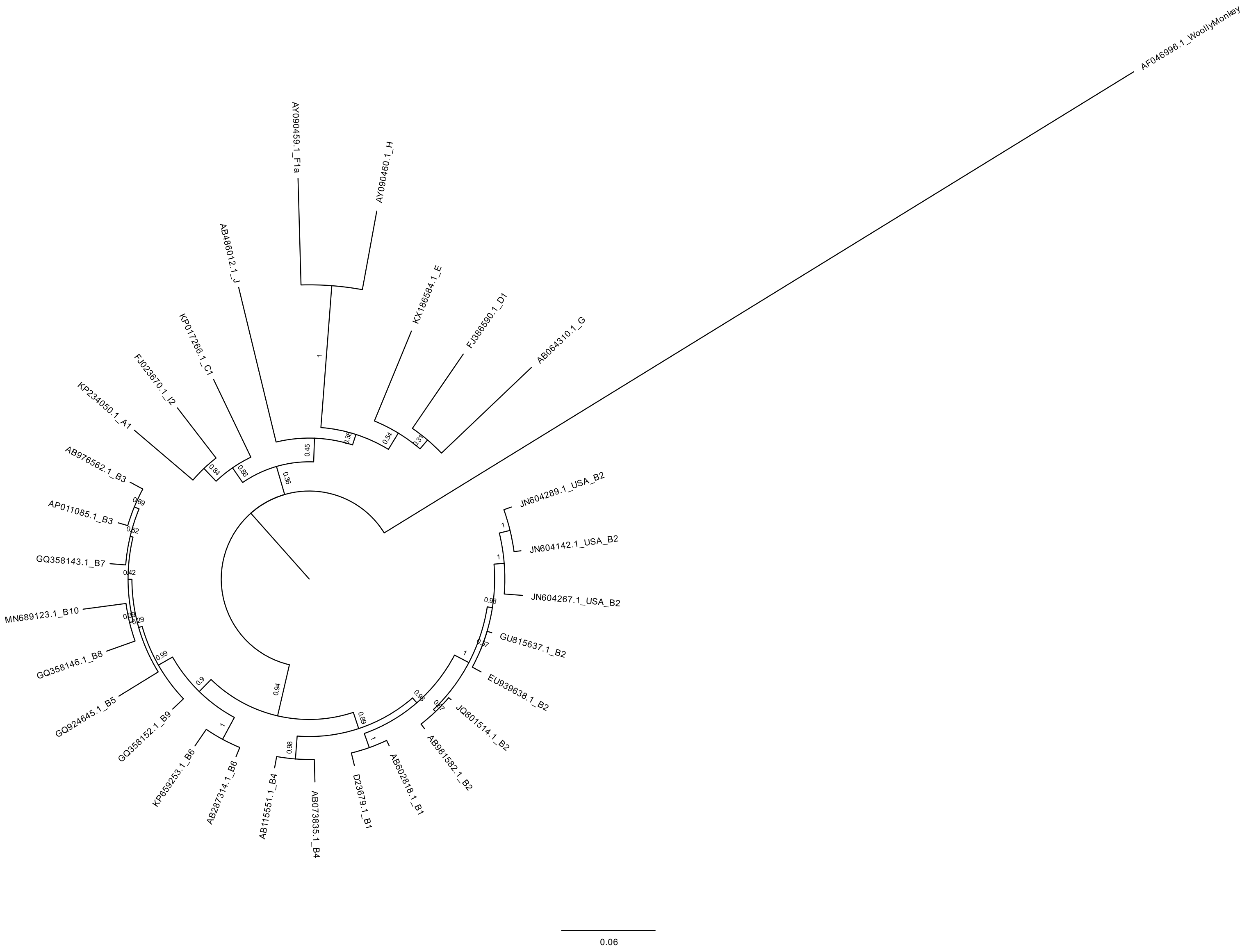

Tree 6. The evolutionary history was inferred by using the Maximum Likelihood method and Tamura-Nei model. The percentage of replicate trees in which the associated taxa clustered together in the bootstrap test (1000 replicates) are shown next to the branches. Initial tree(s) for the heuristic search were obtained automatically by applying Neighbor-Join and BioNJ algorithms to a matrix of pairwise distances estimated using the Tamura-Nei model, and then selecting the topology with superior log likelihood value. A discrete Gamma distribution was used to model evolutionary rate differences among sites (5 categories (+*G*, parameter = 0.2513)). The tree is drawn to scale, with branch lengths measured in the number of substitutions per site. The analysis involved 30 nucleotide sequences, of which 27 were used as marker sequences to determine the genotype of 3 sequences. All positions containing gaps and missing data were eliminated. There was a total of 1687 positions in the final dataset. Evolutionary analyses were conducted in MEGA X.

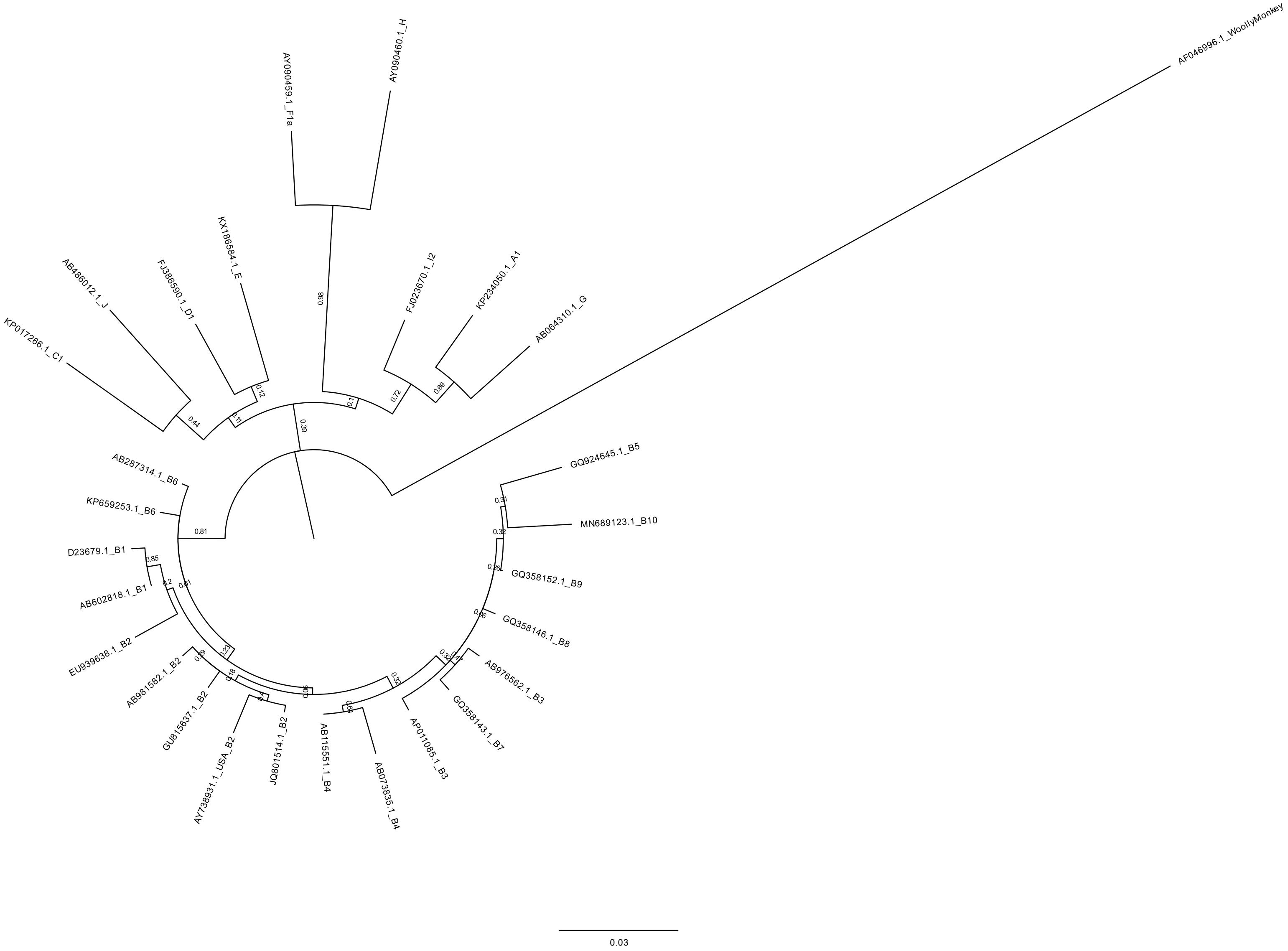

Tree 7. The evolutionary history was inferred by using the Maximum Likelihood method and Tamura-Nei model. The percentage of replicate trees in which the associated taxa clustered together in the bootstrap test (1000 replicates) are shown next to the branches. Initial tree(s) for the heuristic search were obtained automatically by applying Neighbor-Join and BioNJ algorithms to a matrix of pairwise distances estimated using the Tamura-Nei model, and then selecting the topology with superior log likelihood value. A discrete Gamma distribution was used to model evolutionary rate differences among sites (5 categories (+*G*, parameter = 0.1499)). The tree is drawn to scale, with branch lengths measured in the number of substitutions per site. The analysis involved 28 nucleotide sequences, of which 27 were used as marker sequences to determine the genotype of 1 sequence. All positions containing gaps and missing data were eliminated. There was a total of 665 positions in the final dataset. Evolutionary analyses were conducted in MEGA X.

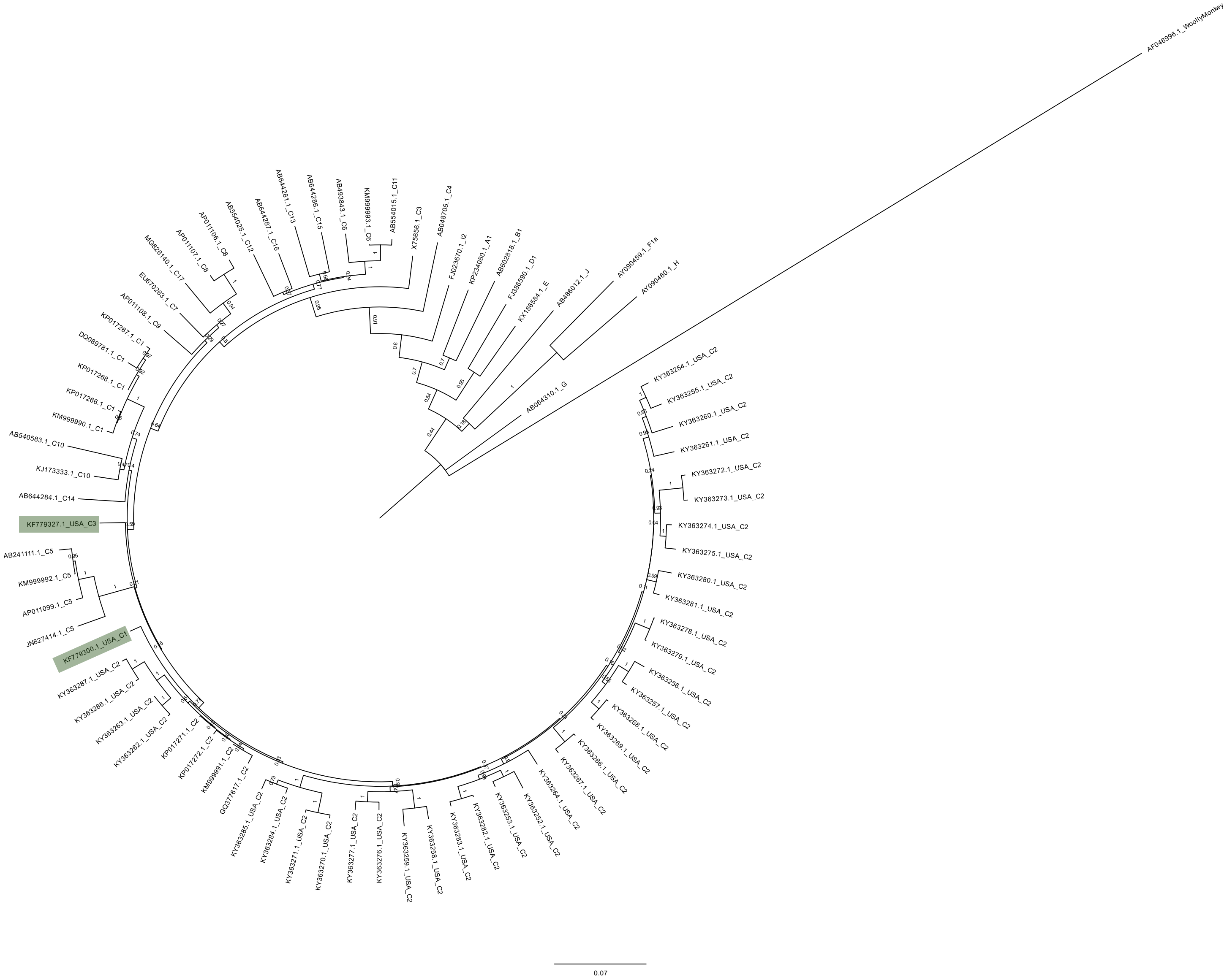

Tree 8. The evolutionary history was inferred by using the Maximum Likelihood method and Tamura-Nei model. The percentage of replicate trees in which the associated taxa clustered together in the bootstrap test (1000 replicates) are shown next to the branches. Initial tree(s) for the heuristic search were obtained automatically by applying Neighbor-Join and BioNJ algorithms to a matrix of pairwise distances estimated using the Tamura-Nei model, and then selecting the topology with superior log likelihood value. A discrete Gamma distribution was used to model evolutionary rate differences among sites (5 categories (+*G*, parameter = 0.2545)). The tree is drawn to scale, with branch lengths measured in the number of substitutions per site. The analysis involved 77 nucleotide sequences, of which 40 were used as marker sequences to determine the genotype of 37 sequences. Sequences KF779300 and KF779327 exhibited irregular grouping. Additional phylogenetic trees were made, and it was concluded that both were subgenotype C2. All positions containing gaps and missing data were eliminated. There was a total of 2634 positions in the final dataset. Evolutionary analyses were conducted in MEGA X.

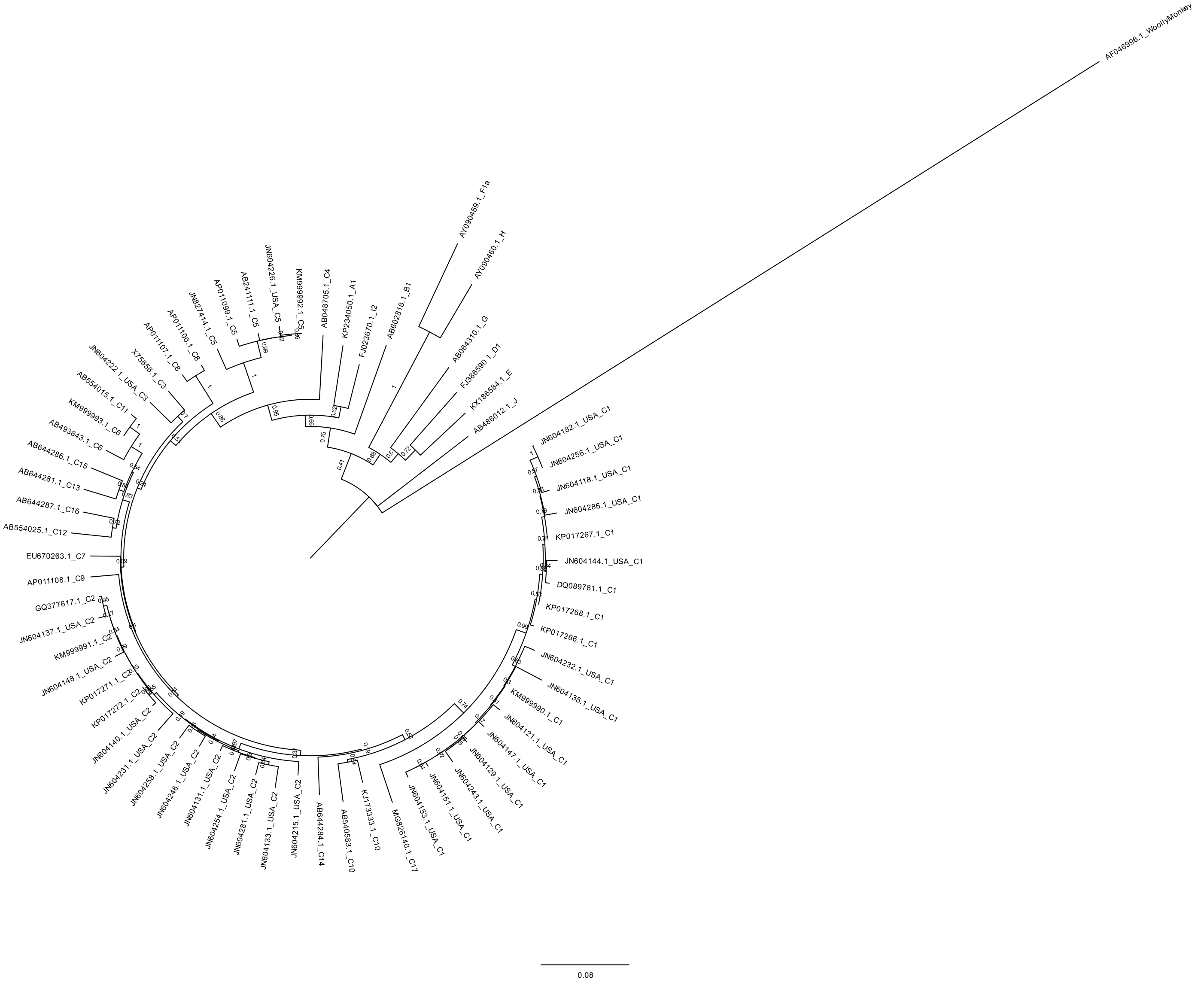

Tree 9. The evolutionary history was inferred by using the Maximum Likelihood method and Tamura-Nei model. The percentage of replicate trees in which the associated taxa clustered together in the bootstrap test (1000 replicates) are shown next to the branches. Initial tree(s) for the heuristic search were obtained automatically by applying Neighbor-Join and BioNJ algorithms to a matrix of pairwise distances estimated using the Tamura-Nei model, and then selecting the topology with superior log likelihood value. A discrete Gamma distribution was used to model evolutionary rate differences among sites (5 categories (+*G*, parameter = 0.2417)). The tree is drawn to scale, with branch lengths measured in the number of substitutions per site. The analysis involved 66 nucleotide sequences, of which 40 were used as marker sequences to determine the genotype of 26 sequences. All positions containing gaps and missing data were eliminated. There was a total of 1911 positions in the final dataset. Evolutionary analyses were conducted in MEGA X.

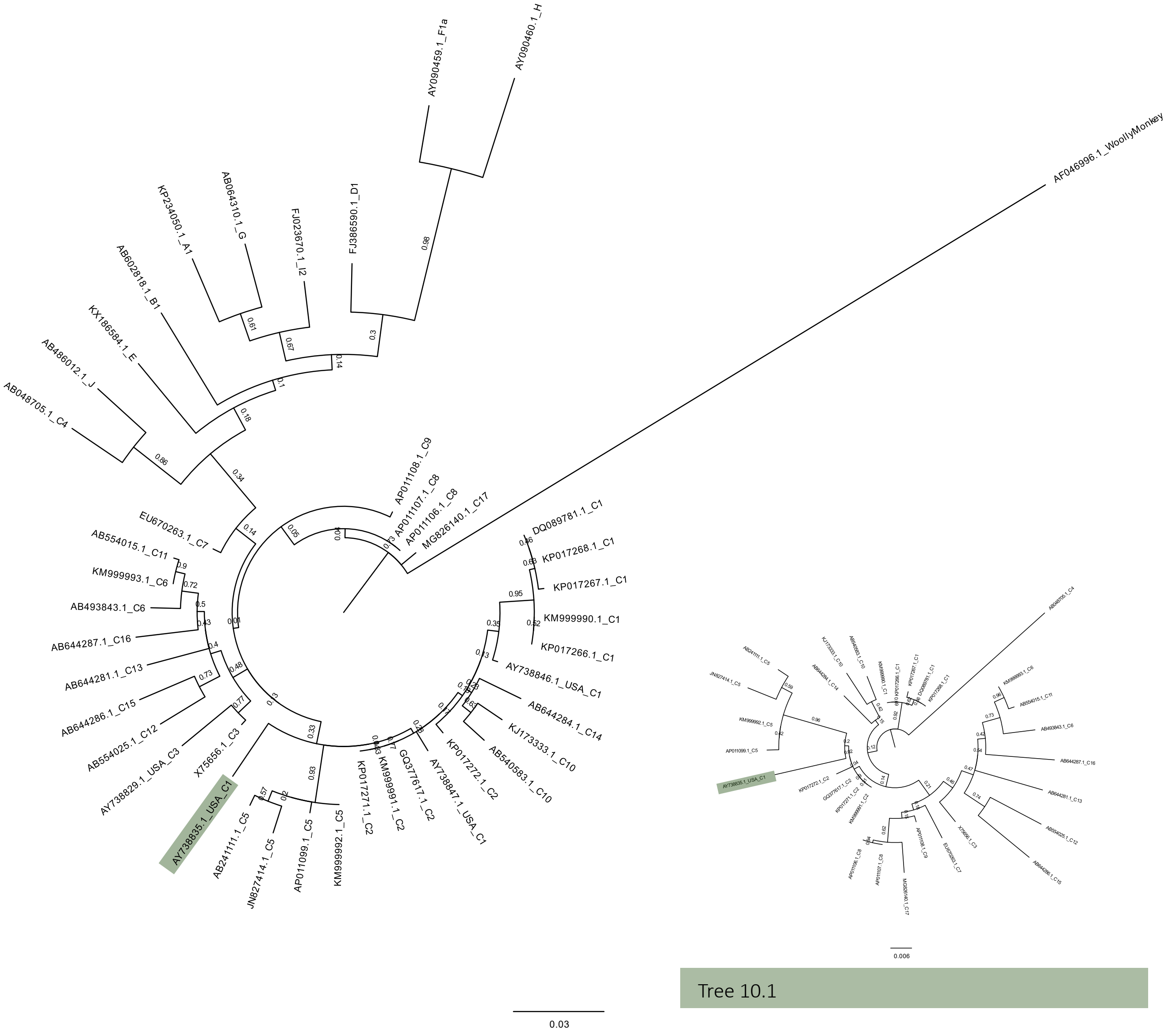

Tree 10. The evolutionary history was inferred by using the Maximum Likelihood method and Tamura-Nei model. The percentage of replicate trees in which the associated taxa clustered together in the bootstrap test (1000 replicates) are shown next to the branches. Initial tree(s) for the heuristic search were obtained automatically by applying Neighbor-Join and BioNJ algorithms to a matrix of pairwise distances estimated using the Tamura-Nei model, and then selecting the topology with superior log likelihood value. A discrete Gamma distribution was used to model evolutionary rate differences among sites (5 categories (+*G*, parameter = 0.1451)). The tree is drawn to scale, with branch lengths measured in the number of substitutions per site. The analysis involved 44 nucleotide sequences, of which 40 were used as marker sequences to determine the genotype of 4 sequences. Sequence AY738847 grouped with subgenotype C2 and was therefore recategorized. Sequence AY738835 was put in an additional phylogenetic analysis (Tree 10.1), and it was concluded that its genotype was C5. All positions containing gaps and missing data were eliminated. There was a total of 678 positions in the final dataset. Evolutionary analyses were conducted in MEGA X.

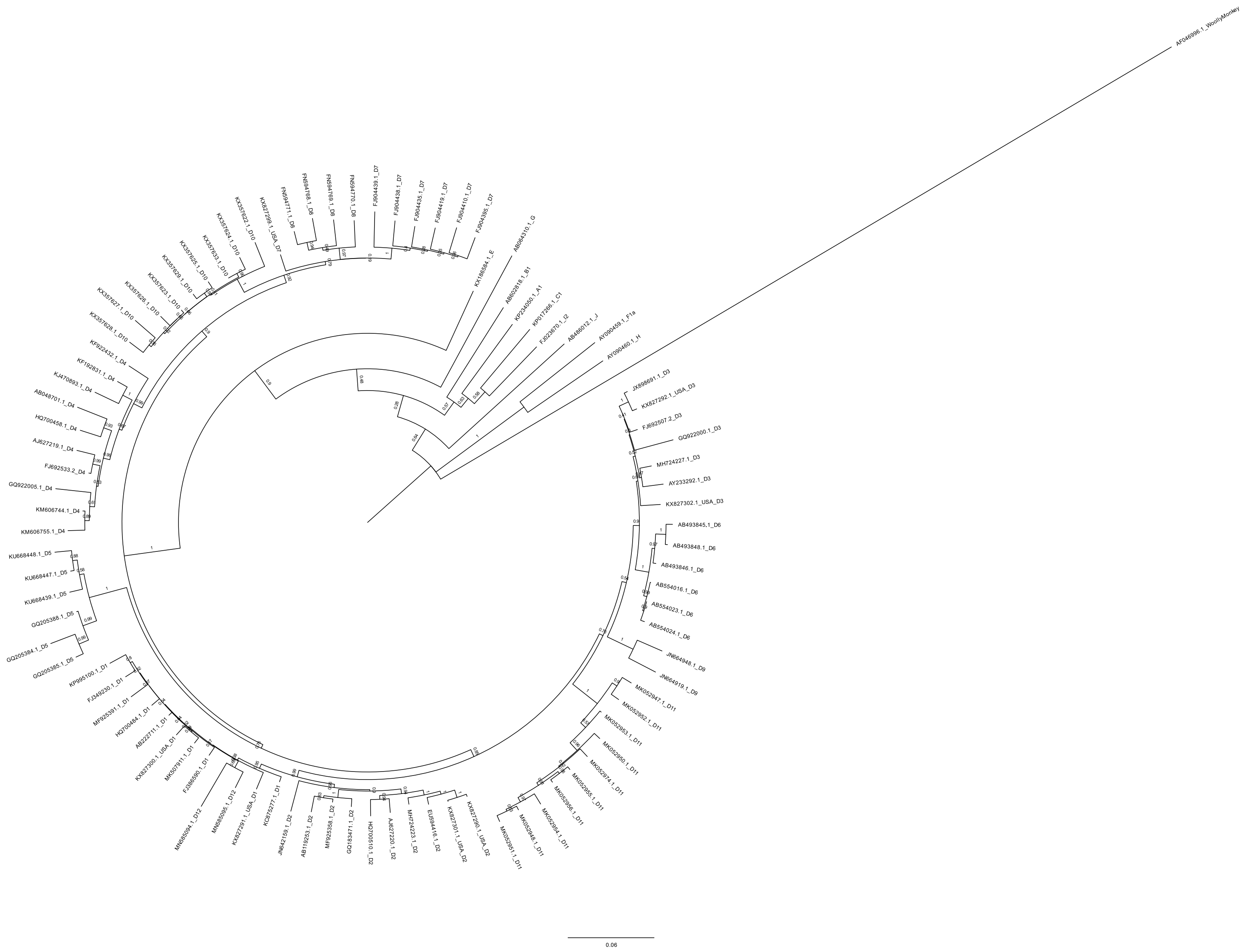

Tree 11. The evolutionary history was inferred by using the Maximum Likelihood method and Tamura-Nei model. The percentage of replicate trees in which the associated taxa clustered together in the bootstrap test (1000 replicates) are shown next to the branches. Initial tree(s) for the heuristic search were obtained automatically by applying Neighbor-Join and BioNJ algorithms to a matrix of pairwise distances estimated using the Tamura-Nei model, and then selecting the topology with superior log likelihood value. A discrete Gamma distribution was used to model evolutionary rate differences among sites (5 categories (+G, parameter = 0.2818)). The tree is drawn to scale, with branch lengths measured in the number of substitutions per site. The analysis involved 93 nucleotide sequences, of which 86 were used as marker sequences to determine the genotype of 7 sequences. All positions containing gaps and missing data were eliminated. There was a total of 3084 positions in the final dataset. Evolutionary analyses were conducted in MEGA X.

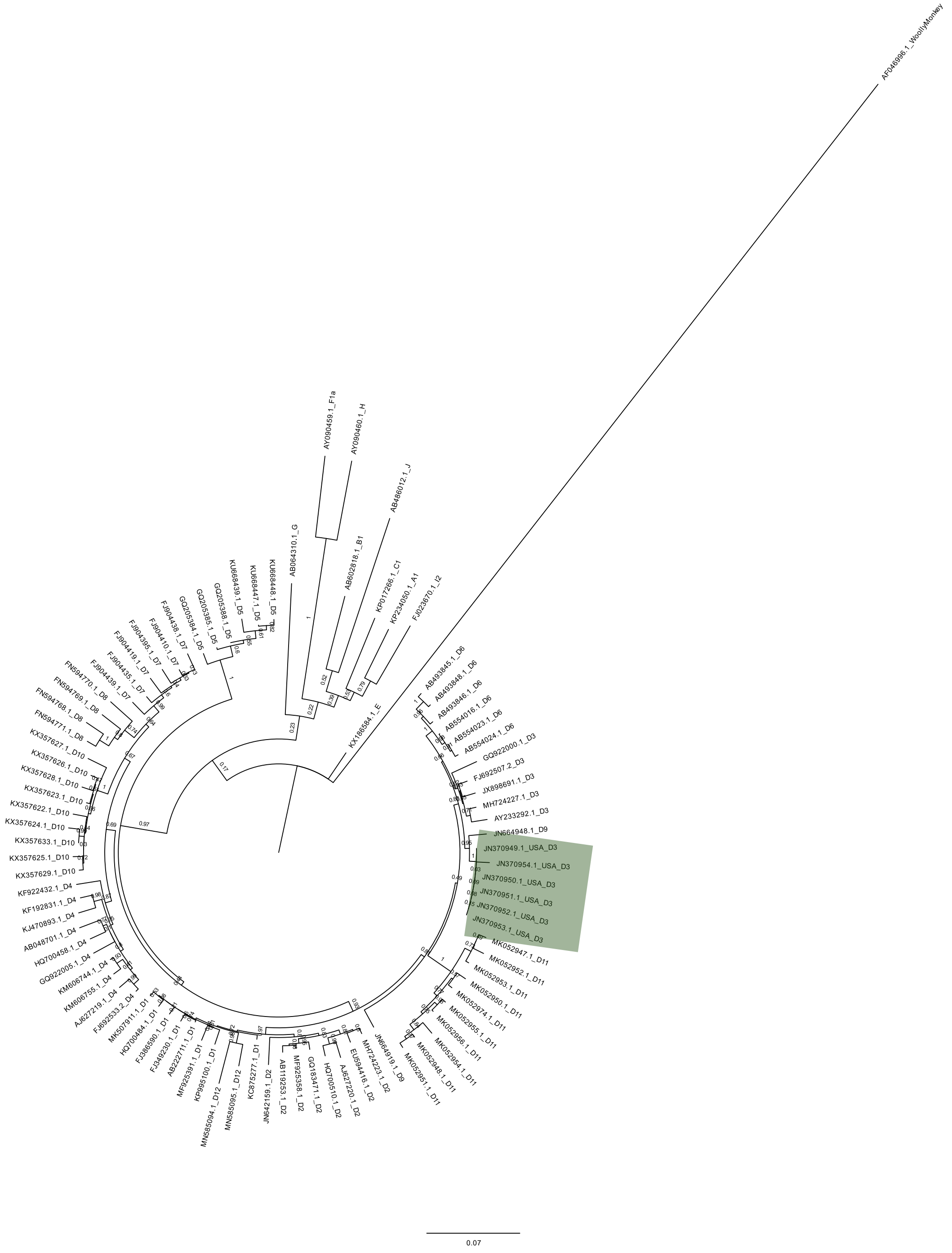

Tree 12. The evolutionary history was inferred by using the Maximum Likelihood method and Tamura-Nei model. The percentage of replicate trees in which the associated taxa clustered together in the bootstrap test (1000 replicates) are shown next to the branches. Initial tree(s) for the heuristic search were obtained automatically by applying Neighbor-Join and BioNJ algorithms to a matrix of pairwise distances estimated using the Tamura-Nei model, and then selecting the topology with superior log likelihood value. A discrete Gamma distribution was used to model evolutionary rate differences among sites (5 categories (+G, parameter = 0.2652)). The tree is drawn to scale, with branch lengths measured in the number of substitutions per site. The analysis involved 92 nucleotide sequences, of which 86 were used as marker sequences to determine the genotype of 6 sequences. All 6 sequences analyzed grouped more adequately with subgenotype D9 in further phylogenetic trees and were therefore recategorized. All positions containing gaps and missing data were eliminated. There was a total of 1980 positions in the final dataset. Evolutionary analyses were conducted in MEGA X.

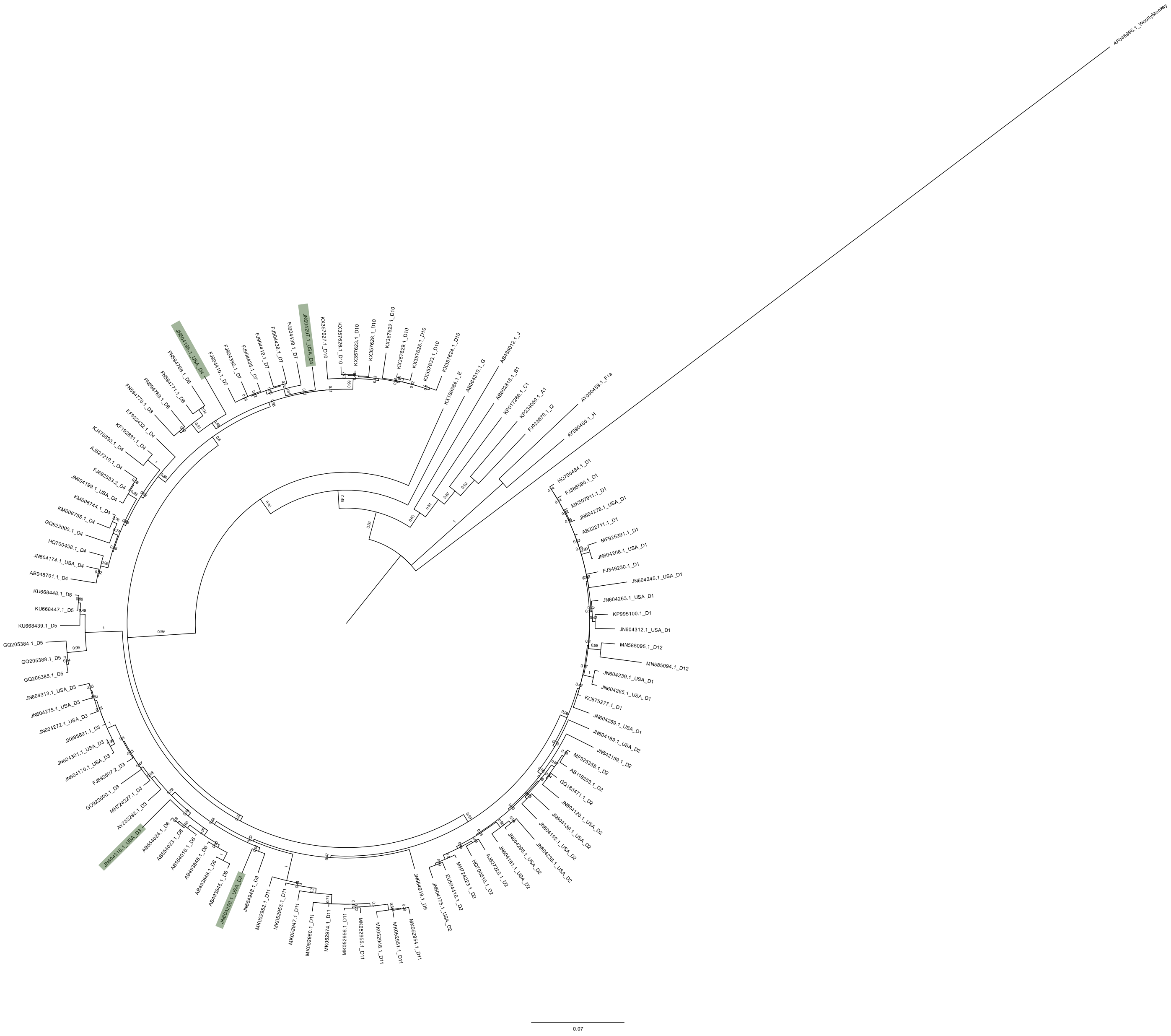

Tree 13. The evolutionary history was inferred by using the Maximum Likelihood method and Tamura-Nei model. The percentage of replicate trees in which the associated taxa clustered together in the bootstrap test (1000 replicates) are shown next to the branches. Initial tree(s) for the heuristic search were obtained automatically by applying Neighbor-Join and BioNJ algorithms to a matrix of pairwise distances estimated using the Tamura-Nei model, and then selecting the topology with superior log likelihood value. A discrete Gamma distribution was used to model evolutionary rate differences among sites (5 categories (+G, parameter = 0.2964)). The tree is drawn to scale, with branch lengths measured in the number of substitutions per site. The analysis involved 113 nucleotide sequences, of which 86 were used as marker sequences to determine the genotype of 27 sequences. Four (JN604196, JN604207, JN604250, JN604318) of the sequences analyzed were recategorized due to their grouping in our phylogenetic tree. All positions containing gaps and missing data were eliminated. There was a total of 1739 positions in the final dataset. Evolutionary analyses were conducted in MEGA X.

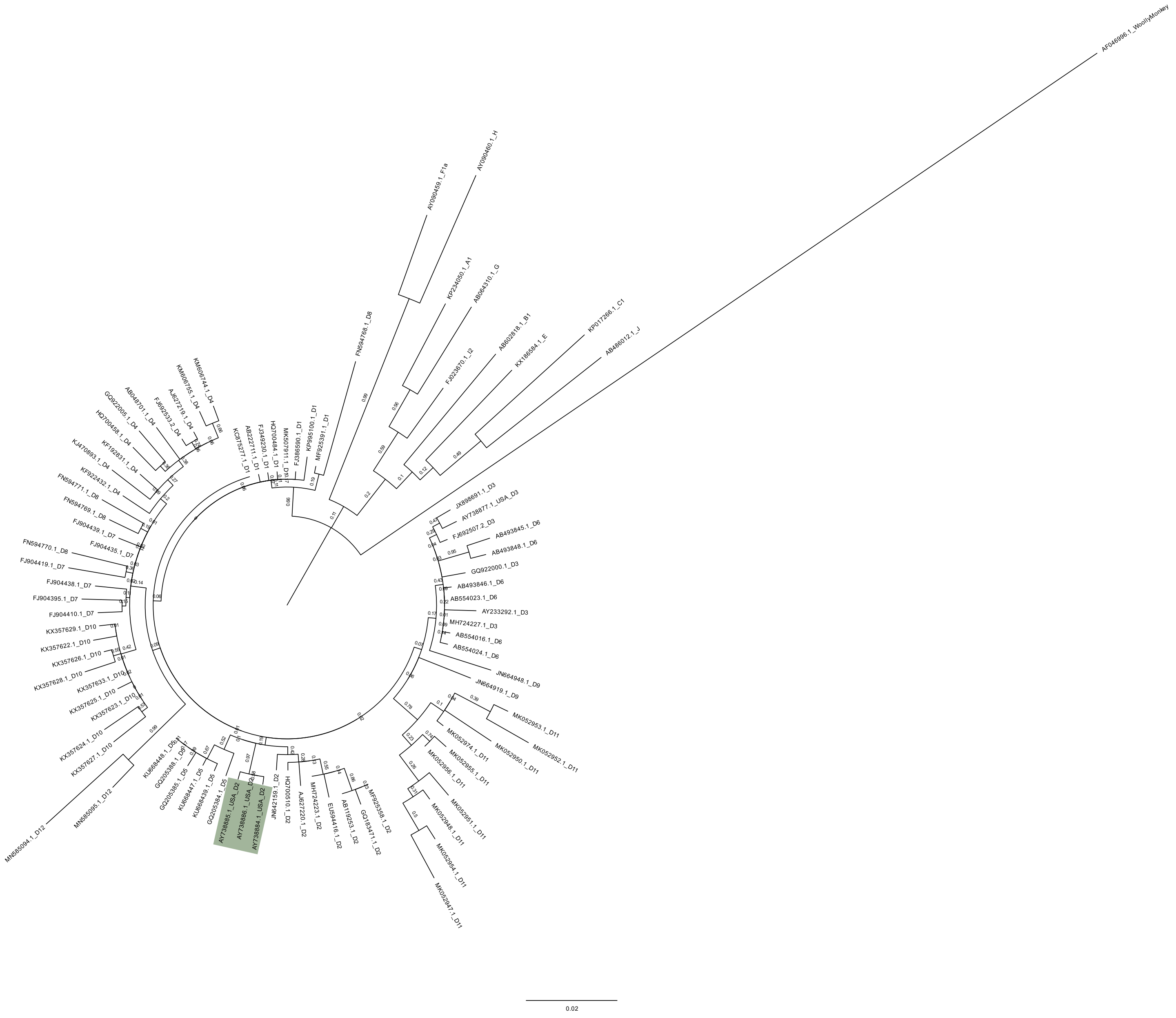

Tree 14. The evolutionary history was inferred by using the Maximum Likelihood method and Tamura-Nei model. The percentage of replicate trees in which the associated taxa clustered together in the bootstrap test (1000 replicates) are shown next to the branches. Initial tree(s) for the heuristic search were obtained automatically by applying Neighbor-Join and BioNJ algorithms to a matrix of pairwise distances estimated using the Tamura-Nei model, and then selecting the topology with superior log likelihood value. A discrete Gamma distribution was used to model evolutionary rate differences among sites (5 categories (+G, parameter = 0.2491)). The tree is drawn to scale, with branch lengths measured in the number of substitutions per site. The analysis involved 90 nucleotide sequences, of which 86 were used as marker sequences to determine the genotype of 4 sequences. Three sequences (AY738884, AY738885, AY738886) originally categorized as D2 were recategorized as D5. All positions containing gaps and missing data were eliminated. There was a total of 678 positions in the final dataset. Evolutionary analyses were conducted in MEGA X.

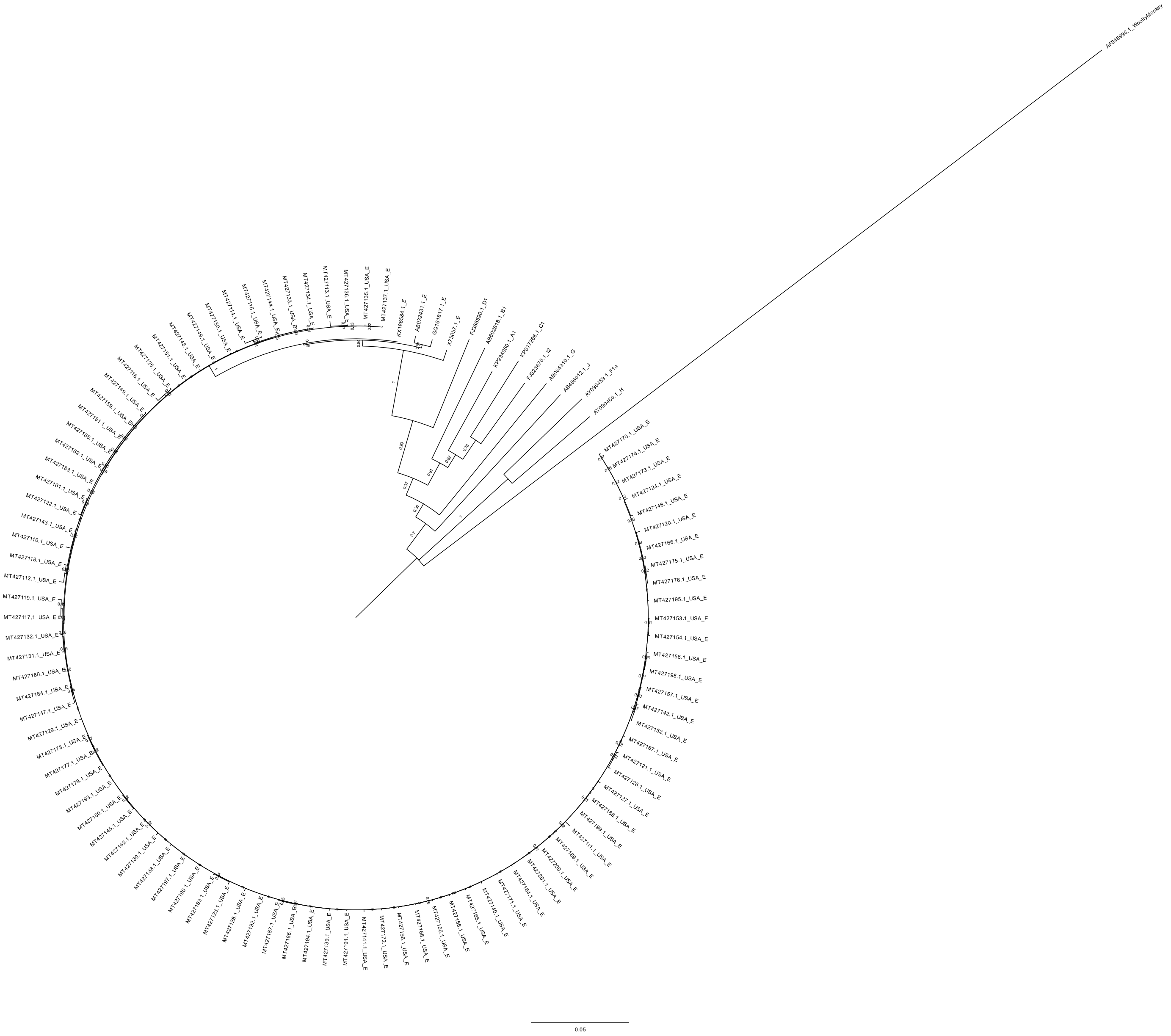

Tree 15. The evolutionary history was inferred by using the Maximum Likelihood method and Tamura-Nei model. The percentage of replicate trees in which the associated taxa clustered together in the bootstrap test (1000 replicates) are shown next to the branches. Initial tree(s) for the heuristic search were obtained automatically by applying Neighbor-Join and BioNJ algorithms to a matrix of pairwise distances estimated using the Tamura-Nei model, and then selecting the topology with superior log likelihood value. A discrete Gamma distribution was used to model evolutionary rate differences among sites (5 categories (+G, parameter = 0.2915)). The tree is drawn to scale, with branch lengths measured in the number of substitutions per site. The analysis involved 106 nucleotide sequences, of which 14 were used as marker sequences to determine the genotype of 92 sequences. All positions containing gaps and missing data were eliminated. There was a total of 3106 positions in the final dataset. Evolutionary analyses were conducted in MEGA X.

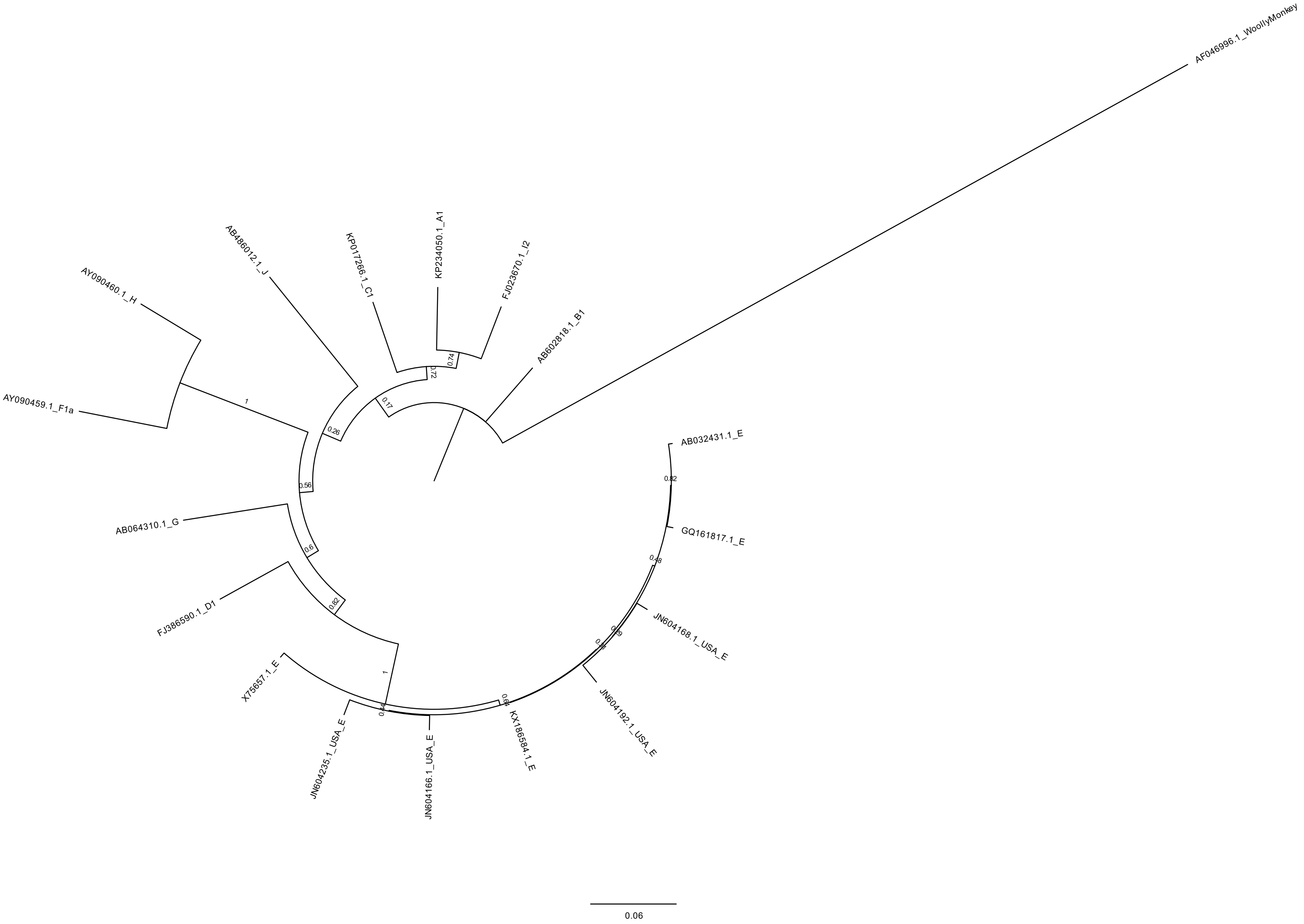

Tree 16. The evolutionary history was inferred by using the Maximum Likelihood method and Tamura-Nei model. The percentage of replicate trees in which the associated taxa clustered together in the bootstrap test (1000 replicates) are shown next to the branches. Initial tree(s) for the heuristic search were obtained automatically by applying Neighbor-Join and BioNJ algorithms to a matrix of pairwise distances estimated using the Tamura-Nei model, and then selecting the topology with superior log likelihood value. A discrete Gamma distribution was used to model evolutionary rate differences among sites (5 categories (+G, parameter = 0.2518)). The tree is drawn to scale, with branch lengths measured in the number of substitutions per site. The analysis involved 18 nucleotide sequences, of which 14 were used as marker sequences to determine the genotype of 4 sequences. All positions containing gaps and missing data were eliminated. There was a total of 1924 positions in the final dataset. Evolutionary analyses were conducted in MEGA X.

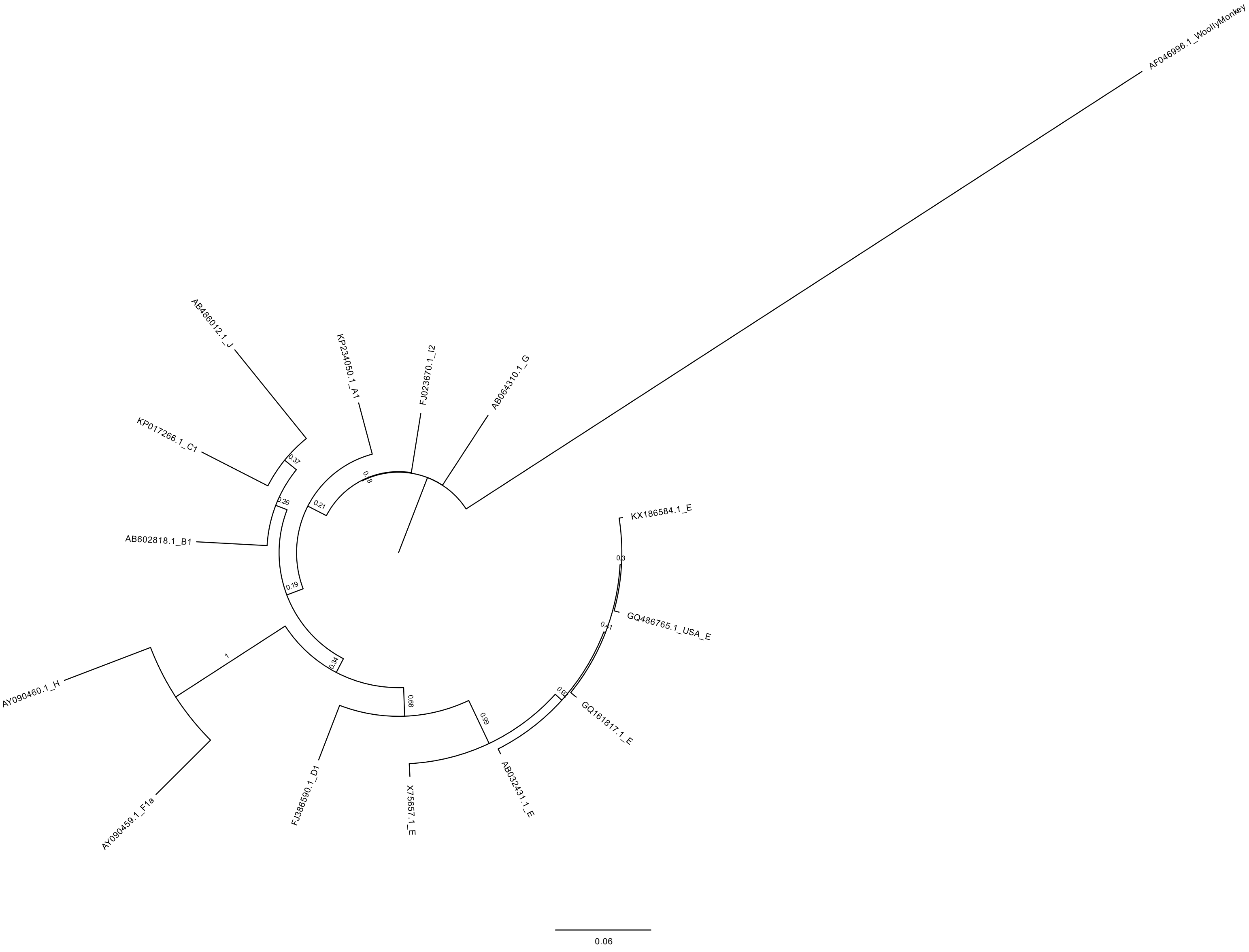

Tree 17. The evolutionary history was inferred by using the Maximum Likelihood method and Tamura-Nei model. The percentage of replicate trees in which the associated taxa clustered together in the bootstrap test (1000 replicates) are shown next to the branches. Initial tree(s) for the heuristic search were obtained automatically by applying Neighbor-Join and BioNJ algorithms to a matrix of pairwise distances estimated using the Tamura-Nei model, and then selecting the topology with superior log likelihood value. A discrete Gamma distribution was used to model evolutionary rate differences among sites (5 categories). The tree is drawn to scale, with branch lengths measured in the number of substitutions per site. The analysis involved 15 nucleotide sequences, of which 14 were used as marker sequences to determine the genotype of 1 sequence. All positions containing gaps and missing data were eliminated. There was a total of 1025 positions in the final dataset. Evolutionary analyses were conducted in MEGA X.

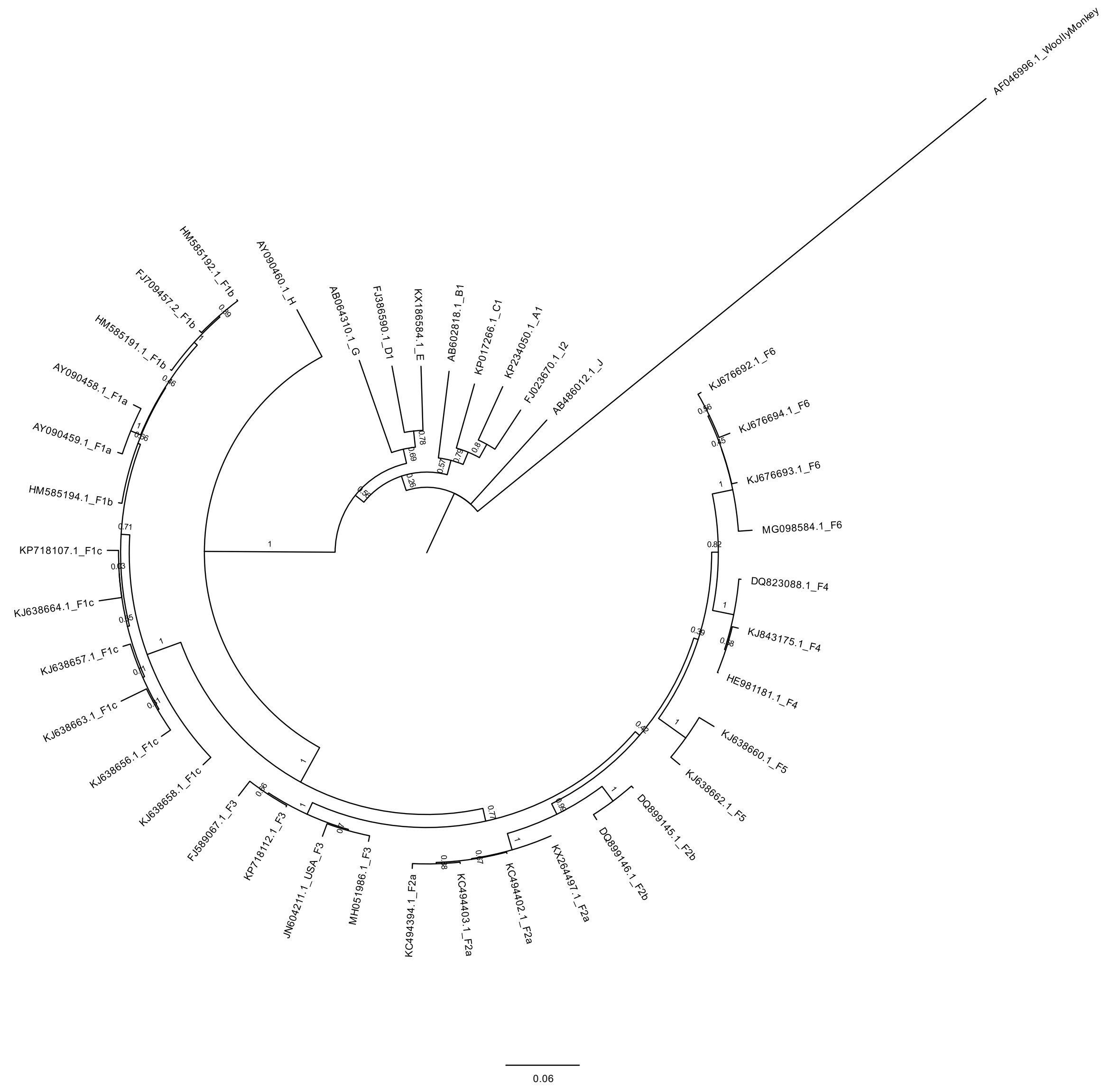

Tree 18. The evolutionary history was inferred by using the Maximum Likelihood method and Tamura-Nei model. The percentage of replicate trees in which the associated taxa clustered together in the bootstrap test (1000 replicates) are shown next to the branches. Initial tree(s) for the heuristic search were obtained automatically by applying Neighbor-Join and BioNJ algorithms to a matrix of pairwise distances estimated using the Tamura-Nei model, and then selecting the topology with superior log likelihood value. A discrete Gamma distribution was used to model evolutionary rate differences among sites (5 categories (+G, parameter = 0.2816)). The tree is drawn to scale, with branch lengths measured in the number of substitutions per site. The analysis involved 41 nucleotide sequences, of which 40 were used as marker sequences to determine the genotype of 1 sequence. All positions containing gaps and missing data were eliminated. There was a total of 1929 positions in the final dataset. Evolutionary analyses were conducted in MEGA X.

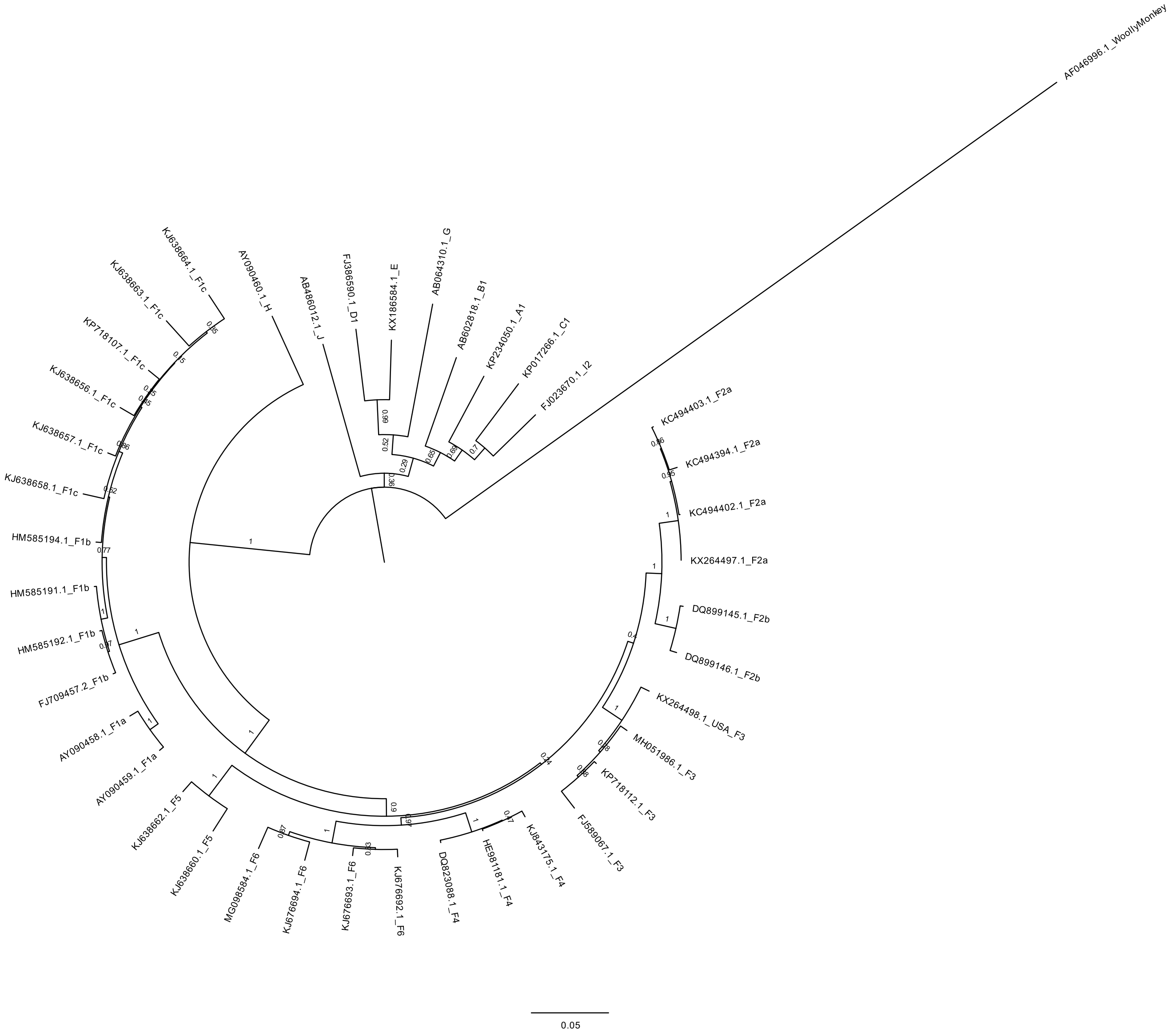

Tree 19. The evolutionary history was inferred by using the Maximum Likelihood method and Tamura-Nei model. The percentage of replicate trees in which the associated taxa clustered together in the bootstrap test (1000 replicates) are shown next to the branches. Initial tree(s) for the heuristic search were obtained automatically by applying Neighbor-Join and BioNJ algorithms to a matrix of pairwise distances estimated using the Tamura-Nei model, and then selecting the topology with superior log likelihood value. A discrete Gamma distribution was used to model evolutionary rate differences among sites (5 categories (+G, parameter = 0.2632)). The tree is drawn to scale, with branch lengths measured in the number of substitutions per site. The analysis involved 41 nucleotide sequences, of which 40 were used as marker sequences to determine the genotype of 1 sequence. All positions containing gaps and missing data were eliminated. There was a total of 3065 positions in the final dataset. Evolutionary analyses were conducted in MEGA X.

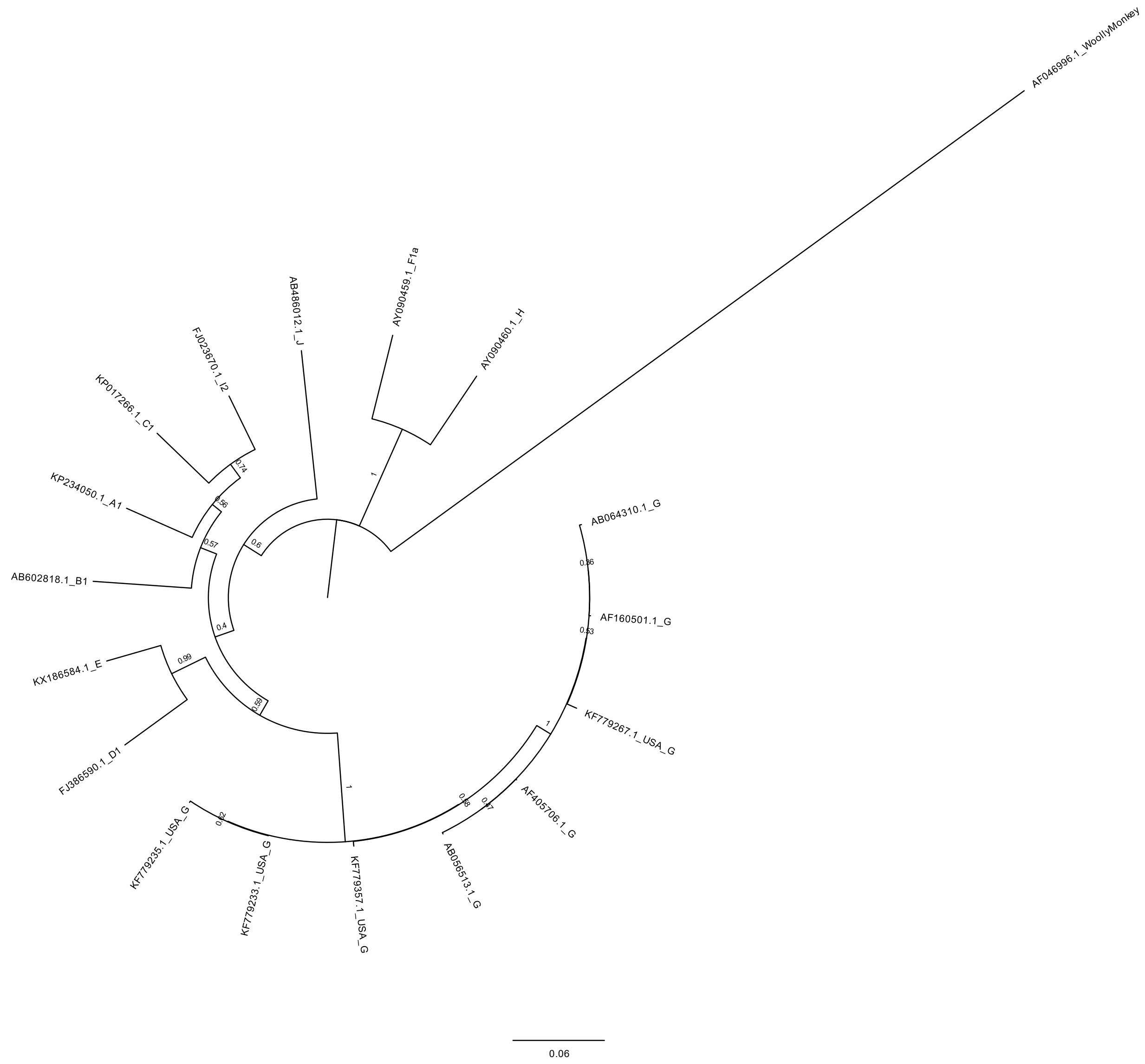

Tree 20. The evolutionary history was inferred by using the Maximum Likelihood method and Tamura-Nei model. The percentage of replicate trees in which the associated taxa clustered together in the bootstrap test (1000 replicates) are shown next to the branches. Initial tree(s) for the heuristic search were obtained automatically by applying Neighbor-Join and BioNJ algorithms to a matrix of pairwise distances estimated using the Tamura-Nei model, and then selecting the topology with superior log likelihood value. A discrete Gamma distribution was used to model evolutionary rate differences among sites (5 categories (+G, parameter = 0.2489)). The tree is drawn to scale, with branch lengths measured in the number of substitutions per site. The analysis involved 18 nucleotide sequences, of which 14 were used as marker sequences to determine the genotype of 4 sequences. All positions containing gaps and missing data were eliminated. There was a total of 3072 positions in the final dataset. Evolutionary analyses were conducted in MEGA X.

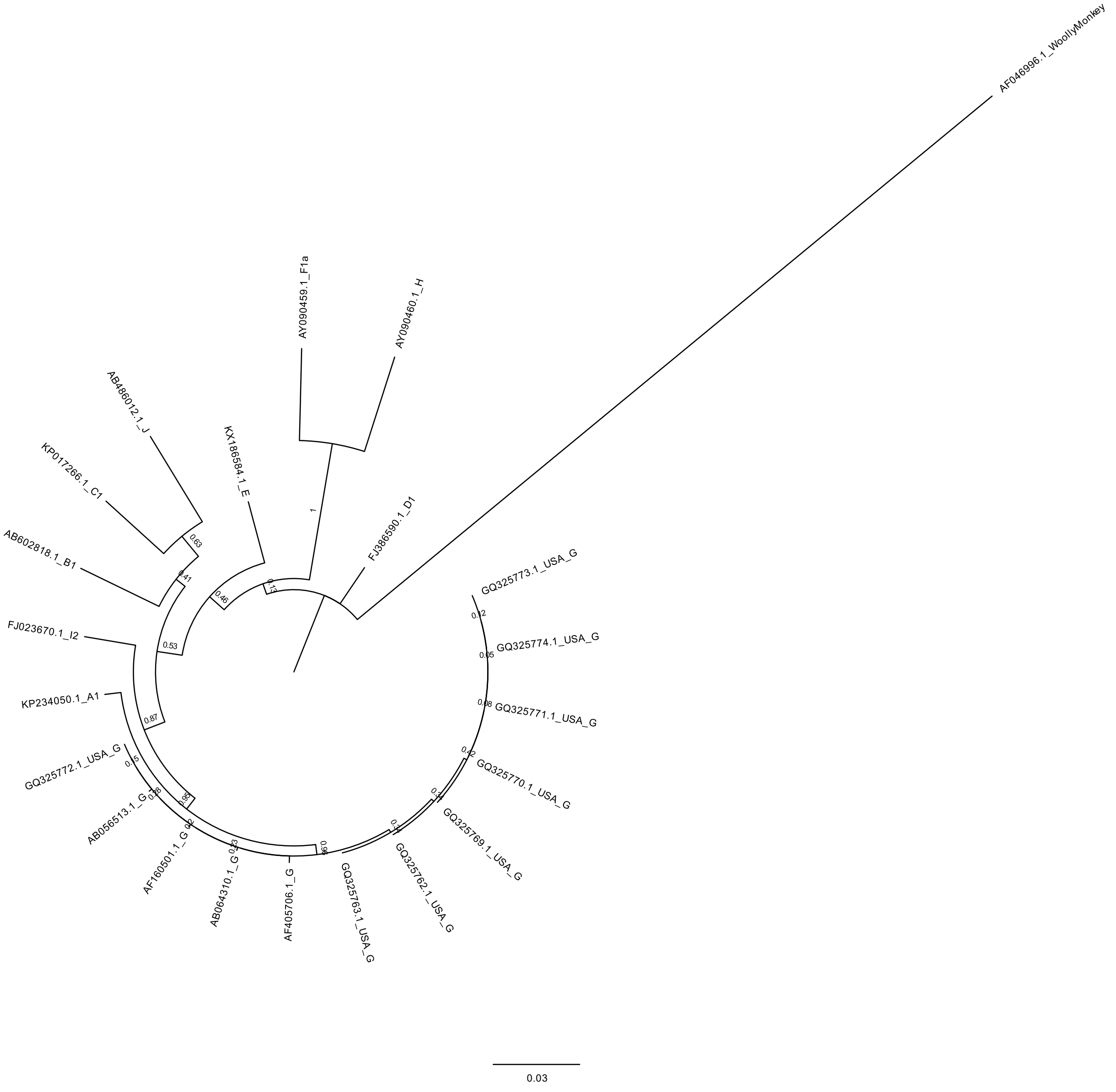

Tree 21. The evolutionary history was inferred by using the Maximum Likelihood method and Tamura-Nei model. The percentage of replicate trees in which the associated taxa clustered together in the bootstrap test (1000 replicates) are shown next to the branches. Initial tree(s) for the heuristic search were obtained automatically by applying Neighbor-Join and BioNJ algorithms to a matrix of pairwise distances estimated using the Tamura-Nei model, and then selecting the topology with superior log likelihood value. A discrete Gamma distribution was used to model evolutionary rate differences among sites (5 categories (+G, parameter = 0.1505)). The tree is drawn to scale, with branch lengths measured in the number of substitutions per site. The analysis involved 22 nucleotide sequences, of which 14 were used as marker sequences to determine the genotype of 8 sequences. All positions containing gaps and missing data were eliminated. There was a total of 944 positions in the final dataset. Evolutionary analyses were conducted in MEGA X.

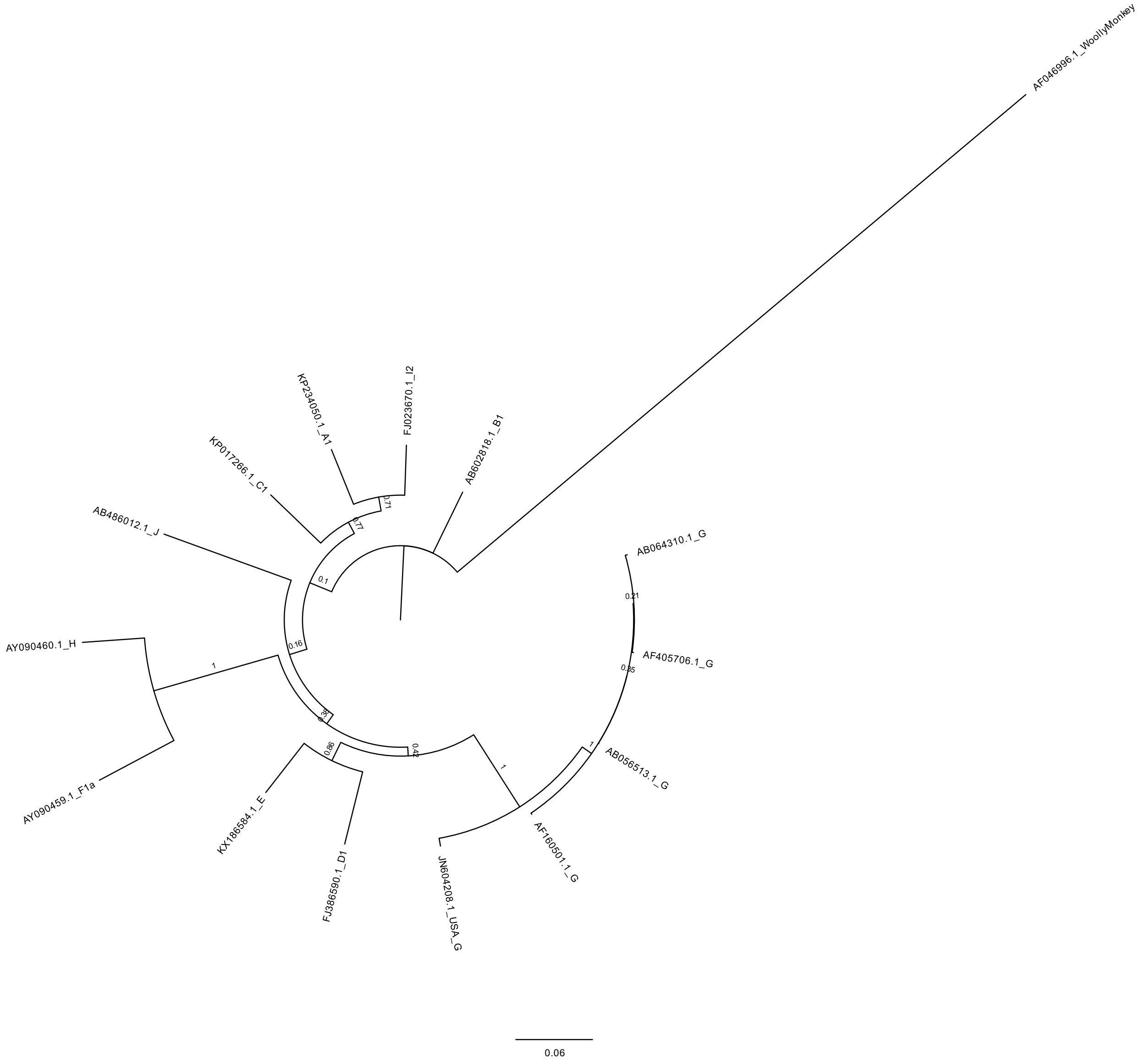

Tree 22. The evolutionary history was inferred by using the Maximum Likelihood method and Tamura-Nei model. The percentage of replicate trees in which the associated taxa clustered together in the bootstrap test (1000 replicates) are shown next to the branches. Initial tree(s) for the heuristic search were obtained automatically by applying Neighbor-Join and BioNJ algorithms to a matrix of pairwise distances estimated using the Tamura-Nei model, and then selecting the topology with superior log likelihood value. A discrete Gamma distribution was used to model evolutionary rate differences among sites (5 categories (+G, parameter = 0.2499)). The tree is drawn to scale, with branch lengths measured in the number of substitutions per site. The analysis involved 15 nucleotide sequences, of which 14 were used as marker sequences to determine the genotype of 1 sequence. All positions containing gaps and missing data were eliminated. There was a total of 1975 positions in the final dataset. Evolutionary analyses were conducted in MEGA X.

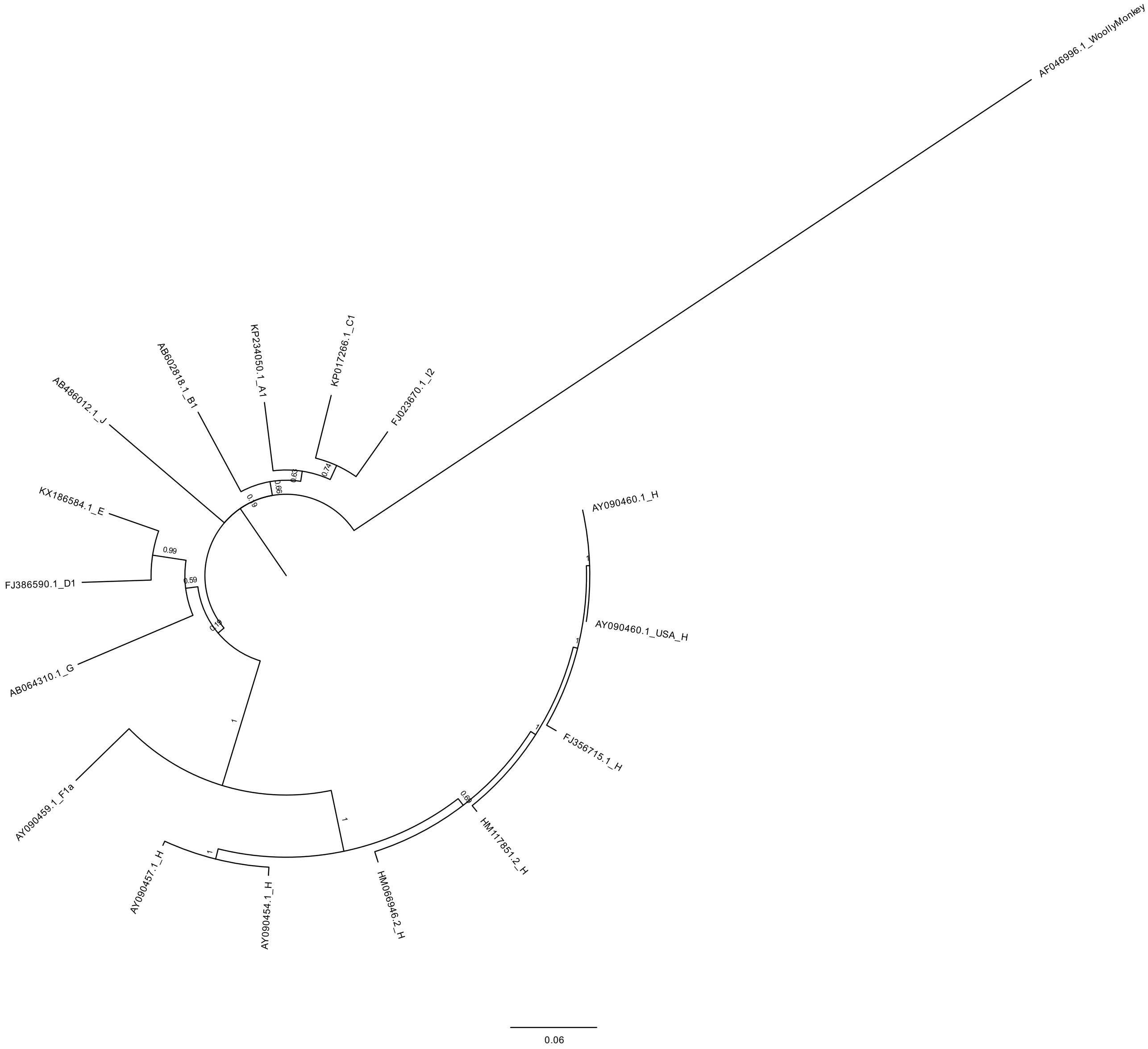

Tree 23. The evolutionary history was inferred by using the Maximum Likelihood method and Tamura-Nei model. The percentage of replicate trees in which the associated taxa clustered together in the bootstrap test (1000 replicates) are shown next to the branches. Initial tree(s) for the heuristic search were obtained automatically by applying Neighbor-Join and BioNJ algorithms to a matrix of pairwise distances estimated using the Tamura-Nei model, and then selecting the topology with superior log likelihood value. A discrete Gamma distribution was used to model evolutionary rate differences among sites (5 categories (+G, parameter = 0.2301)). The tree is drawn to scale, with branch lengths measured in the number of substitutions per site. The analysis involved 17 nucleotide sequences, of which 16 were used as marker sequences to determine the genotype of 1 sequence. All positions containing gaps and missing data were eliminated. There was a total of 3167 positions in the final dataset. Evolutionary analyses were conducted in MEGA X.

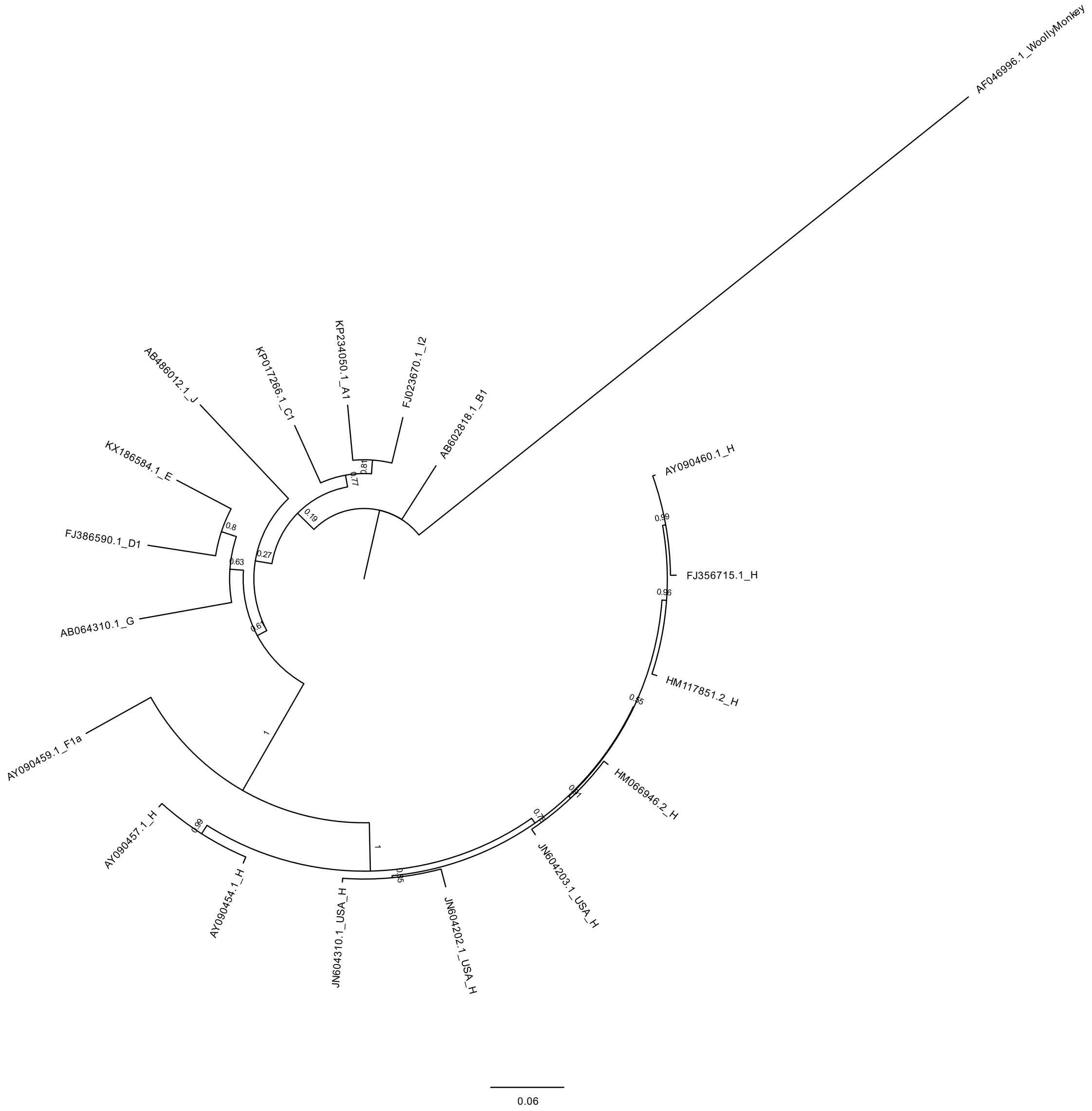

Tree 24. The evolutionary history was inferred by using the Maximum Likelihood method and Tamura-Nei model. The percentage of replicate trees in which the associated taxa clustered together in the bootstrap test (1000 replicates) are shown next to the branches. Initial tree(s) for the heuristic search were obtained automatically by applying Neighbor-Join and BioNJ algorithms to a matrix of pairwise distances estimated using the Tamura-Nei model, and then selecting the topology with superior log likelihood value. A discrete Gamma distribution was used to model evolutionary rate differences among sites (5 categories (+G, parameter = 0.2541)). The tree is drawn to scale, with branch lengths measured in the number of substitutions per site. The analysis involved 19 nucleotide sequences, of which 16 were used as marker sequences to determine the genotype of 3 sequences. All positions containing gaps and missing data were eliminated. There was a total of 1975 positions in the final dataset. Evolutionary analyses were conducted in MEGA X.

ID	GENOTYPE	SUBTYPE	COUNTRY	TREE	ALIGNMENT[1]	BASE PAIRS
KF779210	A	A2	USA	1	Complete Genome	3165
KF779211	A	A2	USA	1	Complete Genome	3165

KF779213	A	A2	USA	1	Complete Genome	3165
KF779215	A	A2	USA	1	Complete Genome	3165
KF779221	A	A2	USA	1	Complete Genome	3165
KF779251	A	A2	USA	1	Complete Genome	3165
KF779252	A	A2	USA	1	Complete Genome	3165
KF779253	A	A2	USA	1	Complete Genome	3165
KF779254	A	A2	USA	1	Complete Genome	3165
KF779255	A	A2	USA	1	Complete Genome	3165
KF779256	A	A2	USA	1	Complete Genome	3165
KF779257	A	A2	USA	1	Complete Genome	3165
KF779258	A	A2	USA	1	Complete Genome	3165
KF779259	A	A2	USA	1	Complete Genome	3165
KF779260	A	A2	USA	1	Complete Genome	3165
KF779261	A	A2	USA	1	Complete Genome	3165
KF779262	A	A2	USA	1	Complete Genome	3165
KF779263	A	A2	USA	1	Complete Genome	3165
KF779264	A	A2	USA	1	Complete Genome	3165
KF779265	A	A2	USA	1	Complete Genome	3165
KF779266	A	A2	USA	1	Complete Genome	3165
KF779268	A	A2	USA	1	Complete Genome	3165
KF779284	A	A2	USA	1	Complete Genome	3165
KF779286	A	A2	USA	1	Complete Genome	3165
KF779287	A	A2	USA	1	Complete Genome	3165
KF779290	A	A2	USA	1	Complete Genome	3165
KF779291	A	A2	USA	1	Complete Genome	3165
KF779293	A	A2	USA	1	Complete Genome	3165
KF779294	A	A2	USA	1	Complete Genome	3165
KF779295	A	A2	USA	1	Complete Genome	3165
KF779297	A	A2	USA	1	Complete Genome	3165
KF779298	A	A2	USA	1	Complete Genome	3165
KF779299	A	A2	USA	1	Complete Genome	3165
KF779304	A	A2	USA	1	Complete Genome	3165
KF779311	A	A2	USA	1	Complete Genome	3165
KF779312	A	A2	USA	1	Complete Genome	3165
KF779313	A	A2	USA	1	Complete Genome	3165
KF779314	A	A2	USA	1	Complete Genome	3165
KF779315	A	A2	USA	1	Complete Genome	3165
KF779316	A	A2	USA	1	Complete Genome	3165
KF779317	A	A2	USA	1	Complete Genome	3165
KF779319	A	A2	USA	1	Complete Genome	3165
KF779320	A	A2	USA	1	Complete Genome	3165

KF779321	A	A2	USA	1	Complete Genome	3165
KF779322	A	A2	USA	1	Complete Genome	3165
KF779323	A	A2	USA	1	Complete Genome	3165
KF779324	A	A2	USA	1	Complete Genome	3165
KF779325	A	A2	USA	1	Complete Genome	3165
KF779326	A	A2	USA	1	Complete Genome	3165
KF779328	A	A2	USA	1	Complete Genome	3165
KF779329	A	A2	USA	1	Complete Genome	3165
KF779330	A	A2	USA	1	Complete Genome	3165
KF779331	A	A2	USA	1	Complete Genome	3165
KF779332	A	A1	USA	1	Complete Genome	3165
KF779333	A	A2	USA	1	Complete Genome	3165
KF779334	A	A2	USA	1	Complete Genome	3165
KF779335	A	A2	USA	1	Complete Genome	3165
KF779336	A	A2	USA	1	Complete Genome	3165
KF779337	A	A2	USA	1	Complete Genome	3165
KF779338	A	A2	USA	1	Complete Genome	3165
KF779339	A	A2	USA	1	Complete Genome	3165
KF779342	A	A2	USA	1	Complete Genome	3165
KF779378	A	A2	USA	1	Complete Genome	3165
KF779379	A	A2	USA	1	Complete Genome	3165
KF779384	A	A1	USA	1	Complete Genome	3165
KF779385	A	A2	USA	1	Complete Genome	3165
KF779386	A	A2	USA	1	Complete Genome	3165
KX827293	A	A2	USA	1	Complete Genome	3221
KX827294	A	A2	USA	1	Complete Genome	3221
KX827295	A	A2	USA	1	Complete Genome	3221
KX827296	A	A2	USA	1	Complete Genome	3221
KX827297	A	A2	USA	1	Complete Genome	3221
KX827298	A	A2	USA	1	Complete Genome	3221
JN604128	A	A1	USA	2	1-3182	1965
JN604136	A	A1	USA	2	1-3182	2015
JN604145	A	A1	USA	2	1-3182	2015
JN604150	A	A1	USA	2	1-3182	2015
JN604155	A	A1	USA	2	1-3182	2014
JN604156	A	A1	USA	2	1-3182	2015
JN604157	A	A1	USA	2	1-3182	2015
JN604158	A	A2	USA	2	1-3182	2015
JN604159	A	A2	USA	2	1-3182	1997
JN604160	A	A2	USA	2	1-3182	2015
JN604162	A	A2	USA	2	1-3182	2015

JN604163	A	A1	USA	2	1-3182	2015
JN604164	A	A2	USA	2	1-3182	2034
JN604165	A	A2	USA	2	1-3182	2015
JN604167	A	A2	USA	2	1-3182	2015
JN604169	A	A1	USA	2	1-3182	2015
JN604171	A	A1	USA	2	1-3182	2015
JN604172	A	A2	USA	2	1-3182	2015
JN604173	A	A2	USA	2	1-3182	2015
JN604176	A	A2	USA	2	1-3182	2015
JN604177	A	A2	USA	2	1-3182	2016
JN604178	A	A2	USA	2	1-3182	2015
JN604179	A	A2	USA	2	1-3182	2012
JN604180	A	A2	USA	2	1-3182	2015
JN604181	A	A2	USA	2	1-3182	2015
JN604183	A	A2	USA	2	1-3182	2005
JN604184	A	A2	USA	2	1-3182	2015
JN604185	A	A2	USA	2	1-3182	2015
JN604186	A	A1	USA	2	1-3182	2015
JN604188	A	A2	USA	2	1-3182	2015
JN604190	A	A2	USA	2	1-3182	2015
JN604191	A	A2	USA	2	1-3182	2015
JN604193	A	A1	USA	2	1-3182	2015
JN604194	A	A2	USA	2	1-3182	1968
JN604195	A	A2	USA	2	1-3182	2015
JN604197	A	A2	USA	2	1-3182	2015
JN604200	A	A2	USA	2	1-3182	2015
JN604204	A	A2	USA	2	1-3182	2015
JN604209	A	A2	USA	2	1-3182	2015
JN604210	A	A1	USA	2	1-3182	2017
JN604214	A	A2	USA	2	1-3182	2015
JN604216	A	A2	USA	2	1-3182	2015
JN604217	A	A3	USA	2	1-3182	2015
JN604218	A	A2	USA	2	1-3182	2015
JN604220	A	A2	USA	2	1-3182	2015
JN604227	A	A2	USA	2	1-3182	2015
JN604228	A	A2	USA	2	1-3182	2015
JN604229	A	A2	USA	2	1-3182	2015
JN604230	A	A1	USA	2	1-3182	2015
JN604236	A	A2	USA	2	1-3182	2015
JN604237	A	A1	USA	2	1-3182	2015
JN604240	A	A2	USA	2	1-3182	2015

JN604242	A	A2	USA	2	1-3182	2015
JN604247	A	A2	USA	2	1-3182	2015
JN604248	A	A1	USA	2	1-3182	2016
JN604249	A	A2	USA	2	1-3182	2015
JN604251	A	A1	USA	2	1-3182	2015
JN604252	A	A1	USA	2	1-3182	2015
JN604255	A	A2	USA	2	24-3172	1965
JN604257	A	A2	USA	2	1-3182	2015
JN604260	A	A1	USA	2	1-3182	2015
JN604261	A	A1	USA	2	1-3182	2015
JN604262	A	A2	USA	2	1-3182	2015
JN604266	A	A2	USA	2	1-3182	2015
JN604268	A	A2	USA	2	1-3182	2015
JN604269	A	A2	USA	2	1-3182	2015
JN604270	A	A2	USA	2	1-3182	2015
JN604273	A	A2	USA	2	1-3182	2015
JN604274	A	A2	USA	2	1-3182	2015
JN604276	A	A2	USA	2	1-3182	2015
JN604277	A	A2	USA	2	1-3182	2015
JN604279	A	A2	USA	2	1-3182	2015
JN604282	A	A2	USA	2	1-3182	2015
JN604283	A	A2	USA	2	1-3182	2015
JN604285	A	A2	USA	2	1-3182	2015
JN604288	A	A2	USA	2	1-3182	2015
JN604290	A	A2	USA	2	1-3182	2015
JN604291	A	A2	USA	2	1-3182	2015
JN604292	A	A2	USA	2	1-3182	2015
JN604293	A	A2	USA	2	1-3182	2015
JN604294	A	A2	USA	2	1-3182	2015
JN604296	A	A1	USA	2	1-3182	2015
JN604297	A	A2	USA	2	1-3182	2015
JN604298	A	A2	USA	2	1-3182	2015
JN604299	A	A2	USA	2	1-3182	2015
JN604300	A	A2	USA	2	1-3182	2015
JN604302	A	A2	USA	2	1-3182	2015
JN604303	A	A2	USA	2	1-3182	2015
JN604304	A	A2	USA	2	1-3182	2015
JN604305	A	A2	USA	2	1-3182	2015
JN604306	A	A2	USA	2	1-3182	2015
JN604307	A	A2	USA	2	1-3182	1966
JN604308	A	A2	USA	2	1-3182	2012

JN604309	A	A2	USA	2	1-3182	2015
JN604314	A	A2	USA	2	1-3182	2015
JN604315	A	A2	USA	2	1-3182	2015
JN604317	A	A2	USA	2	1-3182	2015
JN604319	A	A2	USA	2	1-3182	2015
JN604264	A	A2	USA	3	56-2868	1625
JN604280	A	A2	USA	3	49-2924	1709
JN604287	A	A2	USA	3	1-3182	1269
JN604316	A	A2	USA	3	1-3182	1874
AY738804	A	A1	USA	4	157-837	681
GQ325764	A	A2	USA	4	132-1163	1032
GQ325765	A	A2	USA	4	132-1163	1032
GQ325766	A	A2	USA	4	132-1163	1032
GQ325767	A	A2	USA	4	132-1163	1032
GQ325768	A	A2	USA	4	132-1163	1032
MH368023	A	A2	USA	4	207-1187	981
MH368025	A	A2	USA	4	207-1187	981
JN604119	B	B4	USA	5	1-3182	2015
JN604122	B	B2	USA	5	1-3182	2015
JN604123	B	B3	USA	5	1-3182	2015
JN604124	B	B2	USA	5	1-3182	2015
JN604125	B	B4	USA	5	1-3182	2015
JN604126	B	B4	USA	5	1-3182	1992
JN604127	B	B2	USA	5	1-3182	2015
JN604130	B	B2	USA	5	1-3182	2016
JN604132	B	B4	USA	5	1-3182	2015
JN604134	B	B2	USA	5	1-3182	2015
JN604138	B	B4	USA	5	1-3182	2015
JN604141	B	B5	USA	5	1-3182	2015
JN604143	B	B4	USA	5	1-3182	2015
JN604146	B	B2	USA	5	1-3182	2015
JN604149	B	B2	USA	5	1-3182	2015
JN604154	B	B4	USA	5	1-3182	2015
JN604187	B	B2	USA	5	1-3182	2015
JN604205	B	B8	USA	5	1-3182	2016
JN604212	B	B4	USA	5	1-3182	2015
JN604213	B	B4	USA	5	1-3182	2015
JN604219	B	B4	USA	5	1-3182	2016
JN604221	B	B4	USA	5	1-3182	2015
JN604223	B	B4	USA	5	1-3182	2015
JN604224	B	B2	USA	5	1-3182	2013

JN604234	B	B4	USA	5	1-3182	2015
JN604244	B	B4	USA	5	1-3182	2015
JN604253	B	B4	USA	5	1-3182	2015
JN604284	B	B4	USA	5	1-3182	2015
JN604311	B	B2	USA	5	1-3182	2015
JN604142	B	B2	USA	6	4-3057	1886
JN604267	B	B2	USA	6	4-3057	1763
JN604289	B	B2	USA	6	4-3057	1875
AY738931	B	B2	USA	7	157-837	681
KF779300	C	C2	USA	8	1-3182	3162
KF779327	C	C2	USA	8	1-3182	3162
KY363252	C	C2	USA	8	1-3182	3215
KY363253	C	C2	USA	8	1-3182	3215
KY363254	C	C2	USA	8	1-3182	3215
KY363255	C	C2	USA	8	1-3182	3175
KY363256	C	C2	USA	8	1-3182	3206
KY363257	C	C2	USA	8	1-3182	3182
KY363258	C	C2	USA	8	1-3182	3215
KY363259	C	C2	USA	8	1-3182	3115
KY363260	C	C2	USA	8	1-3182	3215
KY363261	C	C2	USA	8	1-3182	3101
KY363262	C	C2	USA	8	55-3176	3155
KY363263	C	C2	USA	8	1-3182	3215
KY363264	C	C2	USA	8	1-3182	3215
KY363266	C	C2	USA	8	35-3174	3155
KY363267	C	C2	USA	8	35-3174	3155
KY363268	C	C2	USA	8	1-3182	3197
KY363269	C	C2	USA	8	1-3182	3196
KY363270	C	C2	USA	8	1-3182	3119
KY363271	C	C2	USA	8	1-3182	3119
KY363272	C	C2	USA	8	1-3182	3167
KY363273	C	C2	USA	8	1-3182	3167
KY363274	C	C2	USA	8	1-3182	3215
KY363275	C	C2	USA	8	1-3182	3215
KY363276	C	C2	USA	8	1-3182	3200
KY363277	C	C2	USA	8	1-3182	3089
KY363278	C	C2	USA	8	1-3182	3215
KY363279	C	C2	USA	8	1-3182	3215
KY363280	C	C2	USA	8	1-3182	3173
KY363281	C	C2	USA	8	1-3182	3215
KY363282	C	C2	USA	8	1-3182	3215

KY363283	C	C2	USA	8	1-3182	3215
KY363284	C	C2	USA	8	1-3182	3215
KY363285	C	C2	USA	8	61-3098	3053
KY363286	C	C2	USA	8	1-3182	3215
KY363287	C	C2	USA	8	1-3182	3215
JN604118	C	C1	USA	9	1-3182	2015
JN604121	C	C1	USA	9	1-3182	2014
JN604129	C	C1	USA	9	1-3182	2109
JN604131	C	C2	USA	9	1-3182	2015
JN604133	C	C2	USA	9	1-3182	2018
JN604135	C	C1	USA	9	1-3182	2005
JN604137	C	C2	USA	9	1-3182	2014
JN604140	C	C2	USA	9	1-3182	2015
JN604144	C	C1	USA	9	1-3182	2015
JN604147	C	C1	USA	9	1-3182	2015
JN604148	C	C2	USA	9	1-3182	2015
JN604151	C	C1	USA	9	1-3182	2015
JN604153	C	C1	USA	9	1-3182	2020
JN604182	C	C1	USA	9	1-3182	2015
JN604215	C	C2	USA	9	1-3182	2015
JN604222	C	C3	USA	9	1-3182	2015
JN604226	C	C5	USA	9	1-3182	2015
JN604231	C	C2	USA	9	1-3182	2015
JN604232	C	C1	USA	9	1-3182	2015
JN604243	C	C1	USA	9	1-3182	2015
JN604246	C	C2	USA	9	1-3182	2015
JN604254	C	C2	USA	9	1-3182	2015
JN604256	C	C1	USA	9	1-3182	2015
JN604258	C	C2	USA	9	1-3182	2015
JN604281	C	C2	USA	9	1-3182	2015
JN604286	C	C1	USA	9	1-3182	2015
AY738829	C	C3	USA	10	157-837	681
AY738835	C	C5	USA	10	157-837	681
AY738846	C	C1	USA	10	157-837	681
AY738847	C	C2	USA	10	157-837	681
KX827290	D	D2	USA	11	Complete Genome	3182
KX827291	D	D1	USA	11	Complete Genome	3182
KX827292	D	D3	USA	11	Complete Genome	3182
KX827299	D	D7	USA	11	Complete Genome	3182
KX827300	D	D1	USA	11	Complete Genome	3182
KX827301	D	D2	USA	11	Complete Genome	3182

KX827302	D	D3	USA	11	Complete Genome	3182
JN370949	D	D9	USA	12	1-3182	2053
JN370950	D	D9	USA	12	1-3182	2053
JN370951	D	D9	USA	12	1-3182	2053
JN370952	D	D9	USA	12	1-3182	2053
JN370953	D	D9	USA	12	1-3182	2053
JN370954	D	D9	USA	12	1-3182	2053
JN604120	D	D2	USA	13	1-3182	1982
JN604139	D	D2	USA	13	1-3182	1982
JN604152	D	D2	USA	13	1-3182	1982
JN604161	D	D2	USA	13	1-3182	1982
JN604170	D	D3	USA	13	1-3182	1982
JN604174	D	D4	USA	13	1-3182	1982
JN604175	D	D2	USA	13	1-3182	1982
JN604189	D	D2	USA	13	1-3182	1982
JN604196	D	D8	USA	13	1-3182	1964
JN604199	D	D4	USA	13	1-3182	1984
JN604206	D	D1	USA	13	1-3182	1982
JN604207	D	D7	USA	13	1-3182	1982
JN604238	D	D2	USA	13	1-3182	1982
JN604239	D	D1	USA	13	1-3182	1915
JN604245	D	D1	USA	13	1-3182	1982
JN604250	D	D9	USA	13	1-3182	1982
JN604259	D	D1	USA	13	1-3182	1982
JN604263	D	D1	USA	13	1-3182	1982
JN604265	D	D1	USA	13	1-3182	1982
JN604272	D	D3	USA	13	1-3182	1982
JN604275	D	D3	USA	13	1-3182	1982
JN604278	D	D1	USA	13	1-3182	1953
JN604295	D	D2	USA	13	1-3182	1971
JN604301	D	D3	USA	13	1-3182	1982
JN604312	D	D1	USA	13	1-3182	1980
JN604313	D	D3	USA	13	1-3182	1982
JN604318	D	D6	USA	13	1-3182	1982
AY738877	D	D3	USA	14	157-837	681
AY738884	D	D5	USA	14	157-837	681
AY738885	D	D5	USA	14	157-837	681
AY738886	D	D5	USA	14	157-837	681
MT427110	E	E	USA	15	Complete Genome	3212
MT427111	E	E	USA	15	Complete Genome	3212
MT427112	E	E	USA	15	Complete Genome	3212

MT427113	E	E	USA	15	Complete Genome	3212
MT427114	E	E	USA	15	Complete Genome	3212
MT427115	E	E	USA	15	Complete Genome	3212
MT427116	E	E	USA	15	Complete Genome	3212
MT427117	E	E	USA	15	Complete Genome	3212
MT427118	E	E	USA	15	Complete Genome	3212
MT427119	E	E	USA	15	Complete Genome	3212
MT427120	E	E	USA	15	Complete Genome	3212
MT427121	E	E	USA	15	Complete Genome	3212
MT427122	E	E	USA	15	Complete Genome	3212
MT427123	E	E	USA	15	Complete Genome	3212
MT427124	E	E	USA	15	Complete Genome	3212
MT427125	E	E	USA	15	Complete Genome	3212
MT427126	E	E	USA	15	Complete Genome	3212
MT427127	E	E	USA	15	Complete Genome	3212
MT427128	E	E	USA	15	Complete Genome	3212
MT427129	E	E	USA	15	Complete Genome	3212
MT427130	E	E	USA	15	Complete Genome	3212
MT427131	E	E	USA	15	Complete Genome	3212
MT427132	E	E	USA	15	Complete Genome	3212
MT427133	E	E	USA	15	Complete Genome	3212
MT427134	E	E	USA	15	Complete Genome	3212
MT427135	E	E	USA	15	Complete Genome	3212
MT427136	E	E	USA	15	Complete Genome	3212
MT427137	E	E	USA	15	Complete Genome	3212
MT427138	E	E	USA	15	Complete Genome	3212
MT427139	E	E	USA	15	Complete Genome	3212
MT427140	E	E	USA	15	Complete Genome	3212
MT427141	E	E	USA	15	Complete Genome	3212
MT427142	E	E	USA	15	Complete Genome	3212
MT427143	E	E	USA	15	Complete Genome	3212
MT427144	E	E	USA	15	Complete Genome	3212
MT427145	E	E	USA	15	Complete Genome	3212
MT427146	E	E	USA	15	Complete Genome	3212
MT427147	E	E	USA	15	Complete Genome	3212
MT427148	E	E	USA	15	Complete Genome	3212
MT427149	E	E	USA	15	Complete Genome	3212
MT427150	E	E	USA	15	Complete Genome	3212
MT427151	E	E	USA	15	Complete Genome	3212
MT427152	E	E	USA	15	Complete Genome	3212
MT427153	E	E	USA	15	Complete Genome	3212

MT427154	E	E	USA	15	Complete Genome	3212
MT427155	E	E	USA	15	Complete Genome	3212
MT427156	E	E	USA	15	Complete Genome	3212
MT427157	E	E	USA	15	Complete Genome	3212
MT427158	E	E	USA	15	Complete Genome	3212
MT427159	E	E	USA	15	Complete Genome	3212
MT427160	E	E	USA	15	Complete Genome	3212
MT427161	E	E	USA	15	Complete Genome	3212
MT427162	E	E	USA	15	Complete Genome	3212
MT427163	E	E	USA	15	Complete Genome	3212
MT427164	E	E	USA	15	Complete Genome	3212
MT427165	E	E	USA	15	Complete Genome	3212
MT427166	E	E	USA	15	Complete Genome	3212
MT427167	E	E	USA	15	Complete Genome	3212
MT427168	E	E	USA	15	Complete Genome	3212
MT427169	E	E	USA	15	Complete Genome	3212
MT427170	E	E	USA	15	Complete Genome	3212
MT427171	E	E	USA	15	Complete Genome	3212
MT427172	E	E	USA	15	Complete Genome	3212
MT427173	E	E	USA	15	Complete Genome	3212
MT427174	E	E	USA	15	Complete Genome	3212
MT427175	E	E	USA	15	Complete Genome	3212
MT427176	E	E	USA	15	Complete Genome	3212
MT427177	E	E	USA	15	Complete Genome	3212
MT427178	E	E	USA	15	Complete Genome	3212
MT427179	E	E	USA	15	Complete Genome	3212
MT427180	E	E	USA	15	Complete Genome	3212
MT427181	E	E	USA	15	Complete Genome	3212
MT427182	E	E	USA	15	Complete Genome	3212
MT427183	E	E	USA	15	Complete Genome	3212
MT427184	E	E	USA	15	Complete Genome	3212
MT427185	E	E	USA	15	Complete Genome	3212
MT427186	E	E	USA	15	Complete Genome	3212
MT427187	E	E	USA	15	Complete Genome	3212
MT427188	E	E	USA	15	Complete Genome	3212
MT427189	E	E	USA	15	Complete Genome	3212
MT427190	E	E	USA	15	Complete Genome	3212
MT427191	E	E	USA	15	Complete Genome	3212
MT427192	E	E	USA	15	Complete Genome	3212
MT427193	E	E	USA	15	Complete Genome	3212
MT427194	E	E	USA	15	Complete Genome	3212

MT427195	E	E	USA	15	Complete Genome	3212
MT427196	E	E	USA	15	Complete Genome	3212
MT427197	E	E	USA	15	Complete Genome	3212
MT427198	E	E	USA	15	Complete Genome	3212
MT427199	E	E	USA	15	Complete Genome	3212
MT427200	E	E	USA	15	Complete Genome	3212
MT427201	E	E	USA	15	Complete Genome	3212
JN604166	E	E	USA	16	1-3182	2012
JN604168	E	E	USA	16	1-3182	2012
JN604192	E	E	USA	16	1-3182	1988
JN604235	E	E	USA	16	1-3182	2007
GQ486765	E	E	USA	17	132-1163	1032
JN604211	F	F3	USA	18	1-3182	2015
KX264498	F	F3	USA	19	Complete Genome	3215
KF779233	G	G	USA	20	Complete Genome	3156
KF779235	G	G	USA	20	Complete Genome	3156
KF779267	G	G	USA	20	Complete Genome	3156
KF779357	G	G	USA	20	Complete Genome	3156
GQ325762	G	G	USA	21	132-1163	1032
GQ325763	G	G	USA	21	132-1163	1032
GQ325769	G	G	USA	21	132-1163	1032
GQ325770	G	G	USA	21	132-1163	1032
GQ325771	G	G	USA	21	132-1163	1032
GQ325772	G	G	USA	21	132-1163	1032
GQ325773	G	G	USA	21	132-1163	1032
GQ325774	G	G	USA	21	132-1163	1032
JN604208	G	G	USA	22	1-3182	2012
AY090460	H	H	USA	23	Complete Genome	3215
JN604202	H	H	USA	24	1-3182	2015
JN604203	H	H	USA	24	1-3182	2015
JN604310	H	H	USA	24	1-3182	2015

[1]Alignment to complete genome reference sequence VHB NC_003977.2

BRAZIL

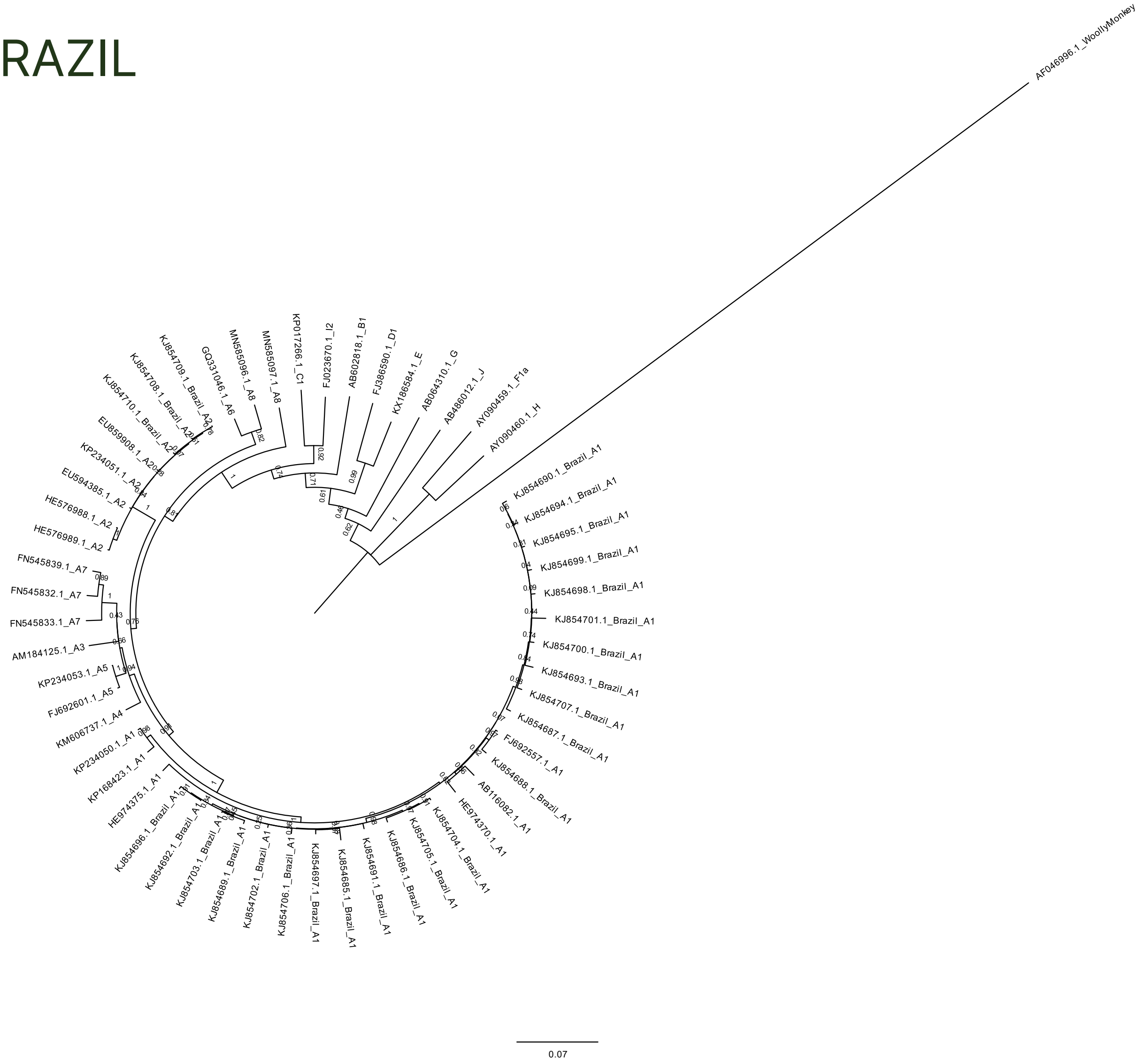

Tree 1. The evolutionary history was inferred by using the Maximum Likelihood method and Tamura-Nei model. The percentage of replicate trees in which the associated taxa clustered together in the bootstrap test (1000 replicates) are shown next to the branches. Initial tree(s) for the heuristic search were obtained automatically by applying Neighbor-Join and BioNJ algorithms to a matrix of pairwise distances estimated using the Tamura-Nei model, and then selecting the topology with superior log likelihood value. A discrete Gamma distribution was used to model evolutionary rate differences among sites (5 categories (+*G*, parameter = 0.2230)). The tree is drawn to scale, with branch lengths measured in the number of substitutions per site. The analysis involved 57 nucleotide sequences, of which 31 were used as marker sequences to determine the genotype of 26 sequences. All positions containing gaps and missing data were eliminated. There was a total of 2846 positions in the final dataset. Evolutionary analyses were conducted in MEGA X.

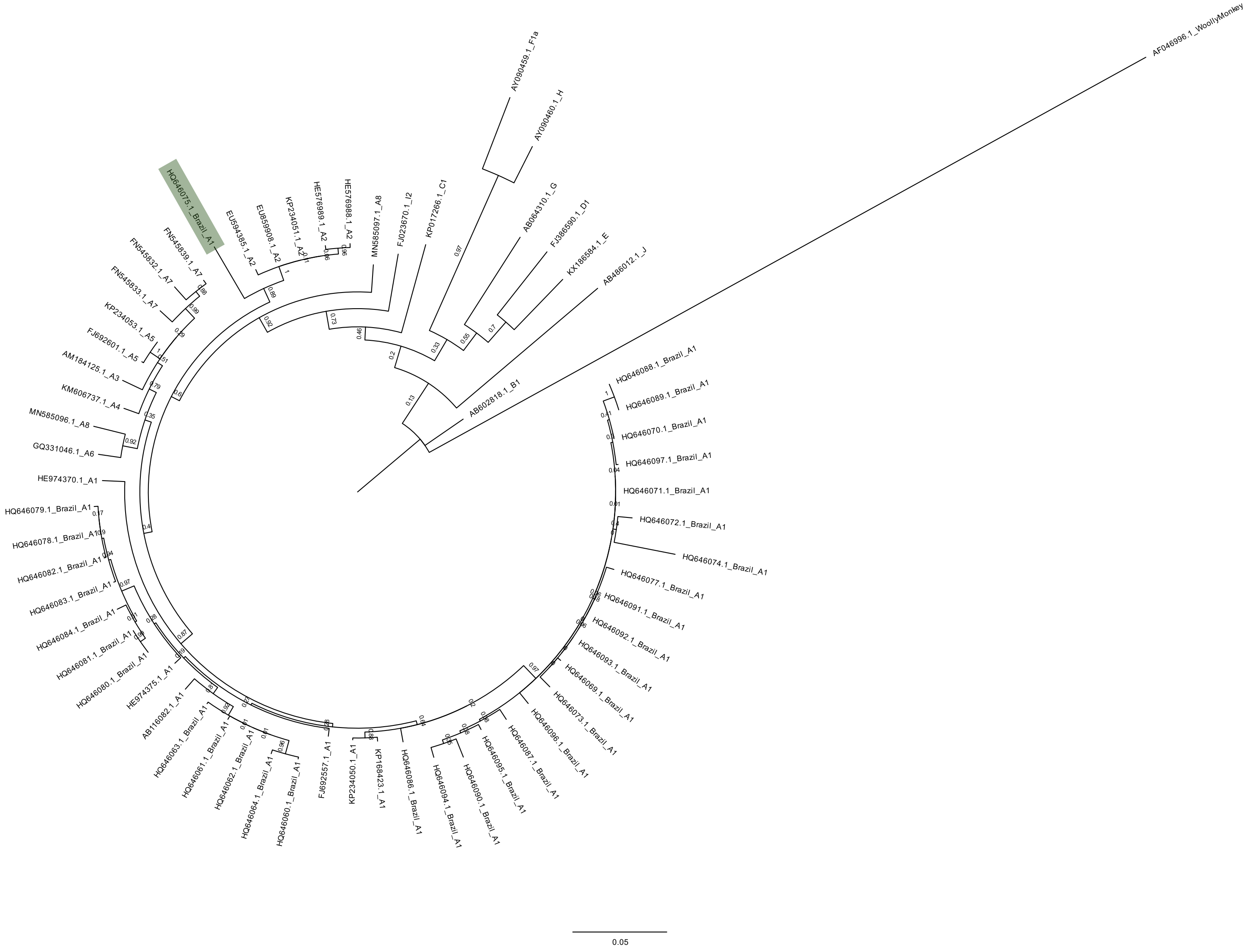

Tree 2. The evolutionary history was inferred by using the Maximum Likelihood method and Tamura-Nei model. The percentage of replicate trees in which the associated taxa clustered together in the bootstrap test (1000 replicates) are shown next to the branches. Initial tree(s) for the heuristic search were obtained automatically by applying Neighbor-Join and BioNJ algorithms to a matrix of pairwise distances estimated using the Tamura-Nei model, and then selecting the topology with superior log likelihood value. A discrete Gamma distribution was used to model evolutionary rate differences among sites (5 categories (+*G*, parameter = 0.2340)). The tree is drawn to scale, with branch lengths measured in the number of substitutions per site. The analysis involved 63 nucleotide sequences, of which 31 were used as marker sequences to determine the genotype of 32 sequences. Sequence HQ646075 was recategorized to subgenotype A2. All positions containing gaps and missing data were eliminated. There was a total of 1057 positions in the final dataset. Evolutionary analyses were conducted in MEGA X.

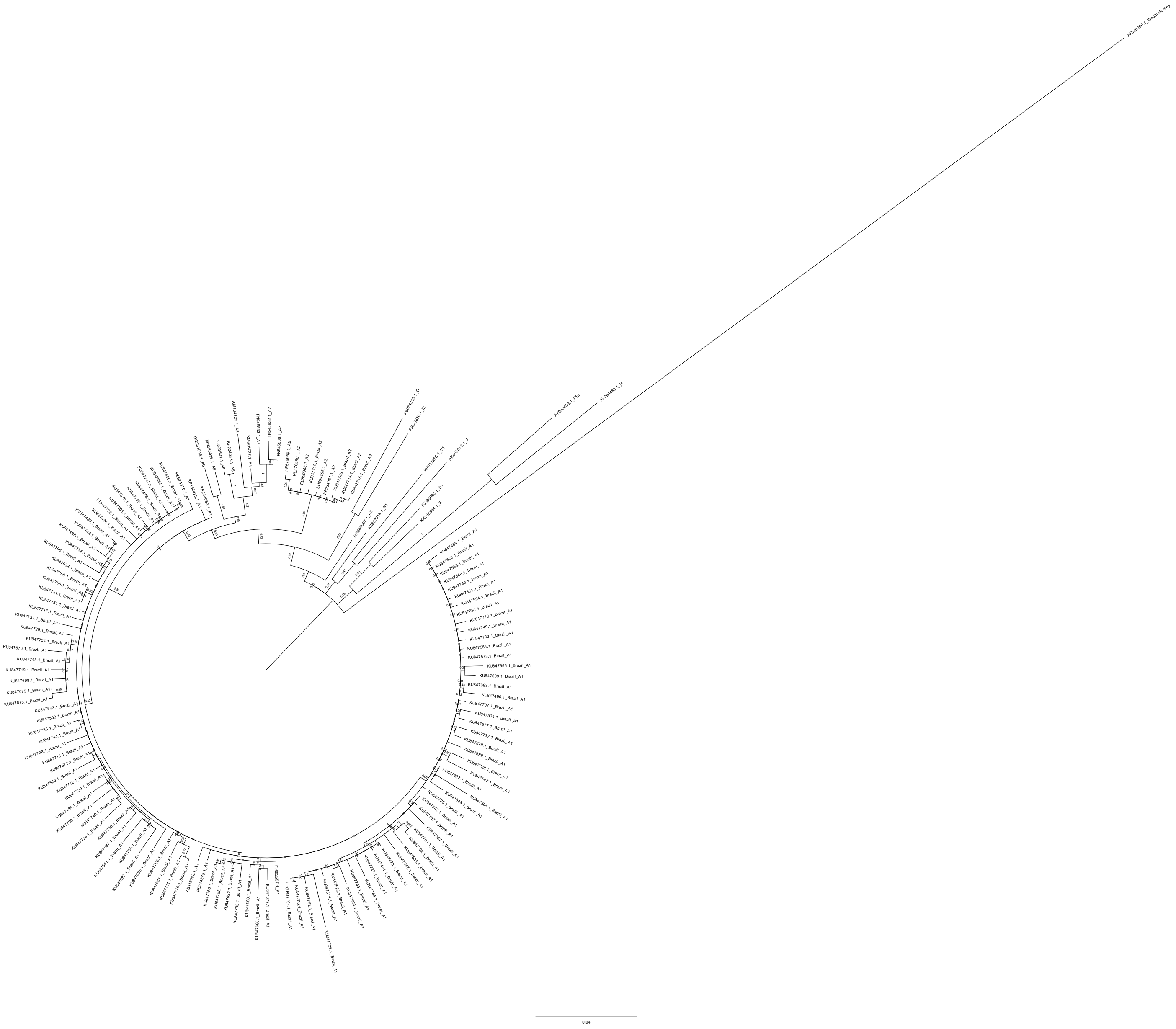

Tree 3. The evolutionary history was inferred by using the Maximum Likelihood method and Tamura-Nei model. The percentage of replicate trees in which the associated taxa clustered together in the bootstrap test (1000 replicates) are shown next to the branches. Initial tree(s) for the heuristic search were obtained automatically by applying Neighbor-Join and BioNJ algorithms to a matrix of pairwise distances estimated using the Tamura-Nei model, and then selecting the topology with superior log likelihood value. A discrete Gamma distribution was used to model evolutionary rate differences among sites (5 categories (+*G*, parameter = 0.1977)). The tree is drawn to scale, with branch lengths measured in the number of substitutions per site. The analysis involved 143 nucleotide sequences, of which 31 were used as marker sequences to determine the genotype of 112 sequences. All positions containing gaps and missing data were eliminated. There was a total of 876 positions in the final dataset. Evolutionary analyses were conducted in MEGA X.

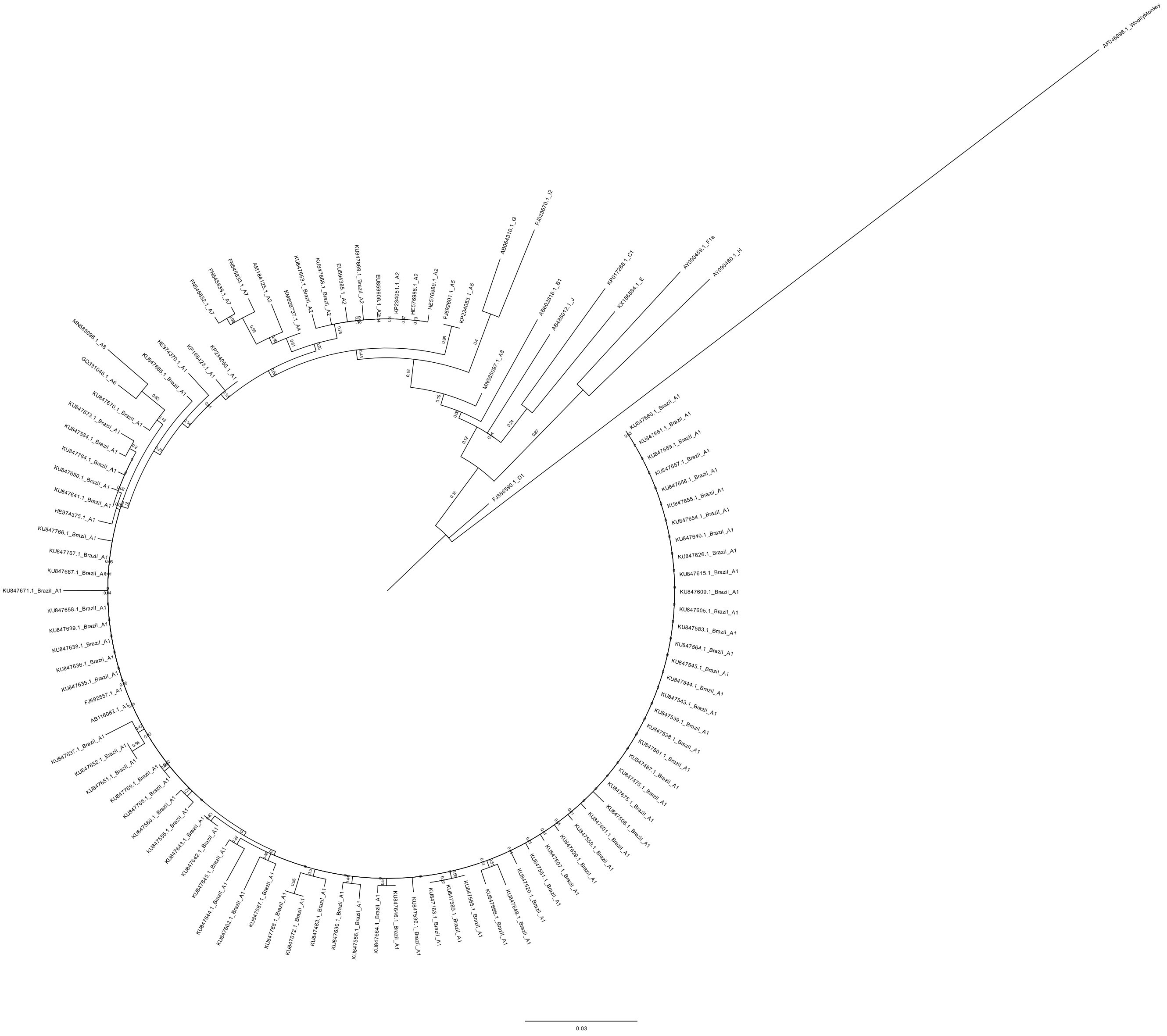

Tree 4. The evolutionary history was inferred by using the Maximum Likelihood method and Tamura-Nei model. The percentage of replicate trees in which the associated taxa clustered together in the bootstrap test (1000 replicates) are shown next to the branches. Initial tree(s) for the heuristic search were obtained automatically by applying Neighbor-Join and BioNJ algorithms to a matrix of pairwise distances estimated using the Tamura-Nei model, and then selecting the topology with superior log likelihood value. A discrete Gamma distribution was used to model evolutionary rate differences among sites (5 categories (+*G*, parameter = 0.1691)). The tree is drawn to scale, with branch lengths measured in the number of substitutions per site. The analysis involved 106 nucleotide sequences, of which 31 were used as marker sequences to determine the genotype of 75 sequences. All positions containing gaps and missing data were eliminated. There was a total of 635 positions in the final dataset. Evolutionary analyses were conducted in MEGA X.

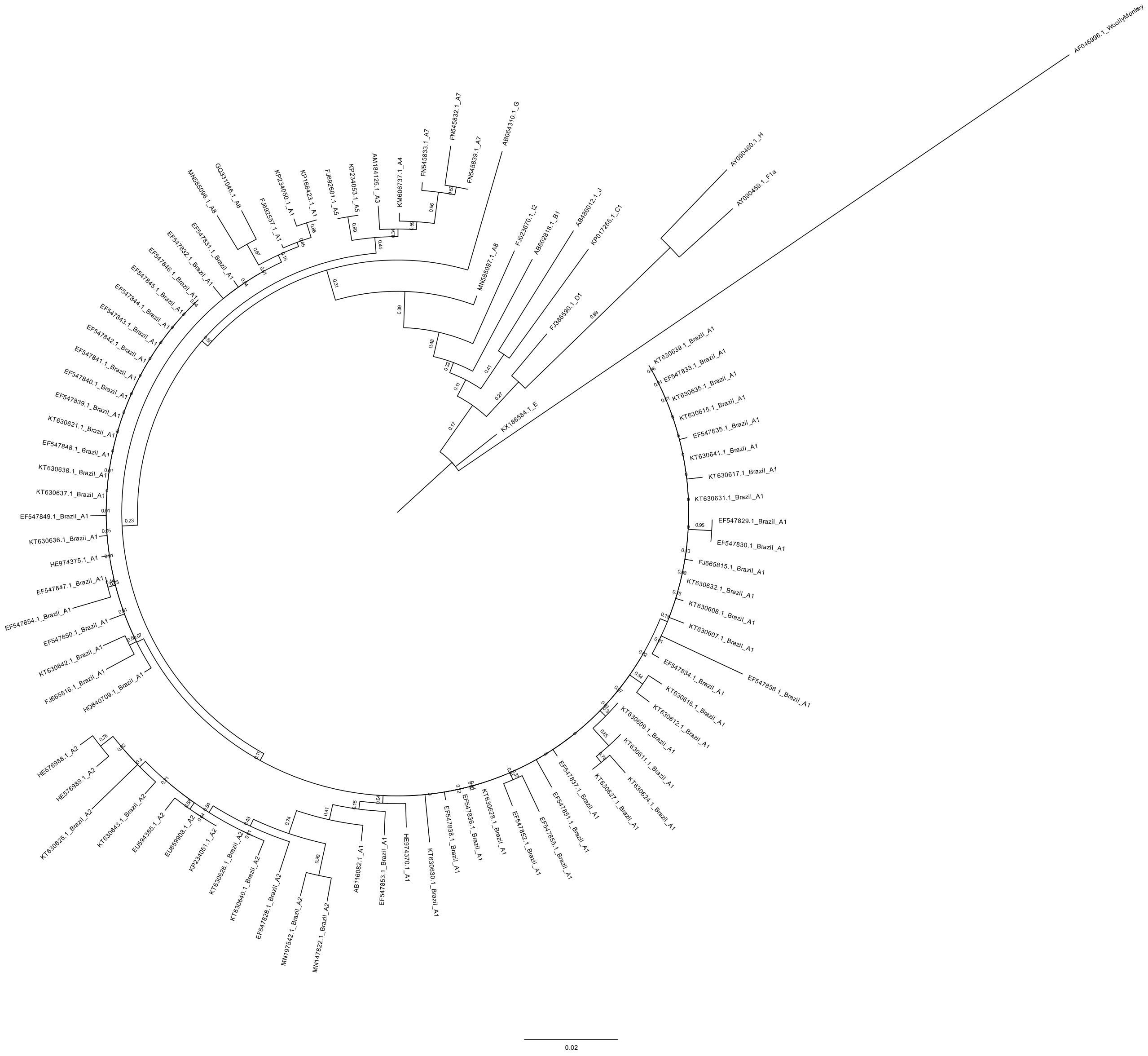

Tree 5. The evolutionary history was inferred by using the Maximum Likelihood method and Tamura-Nei model. The percentage of replicate trees in which the associated taxa clustered together in the bootstrap test (1000 replicates) are shown next to the branches. Initial tree(s) for the heuristic search were obtained automatically by applying Neighbor-Join and BioNJ algorithms to a matrix of pairwise distances estimated using the Tamura-Nei model, and then selecting the topology with superior log likelihood value. A discrete Gamma distribution was used to model evolutionary rate differences among sites (5 categories (+*G*, parameter = 0.2394)). The tree is drawn to scale, with branch lengths measured in the number of substitutions per site. The analysis involved 91 nucleotide sequences, of which 31 were used as marker sequences to determine the genotype of 60 sequences. All positions containing gaps and missing data were eliminated. There was a total of 657 positions in the final dataset. Evolutionary analyses were conducted in MEGA X.

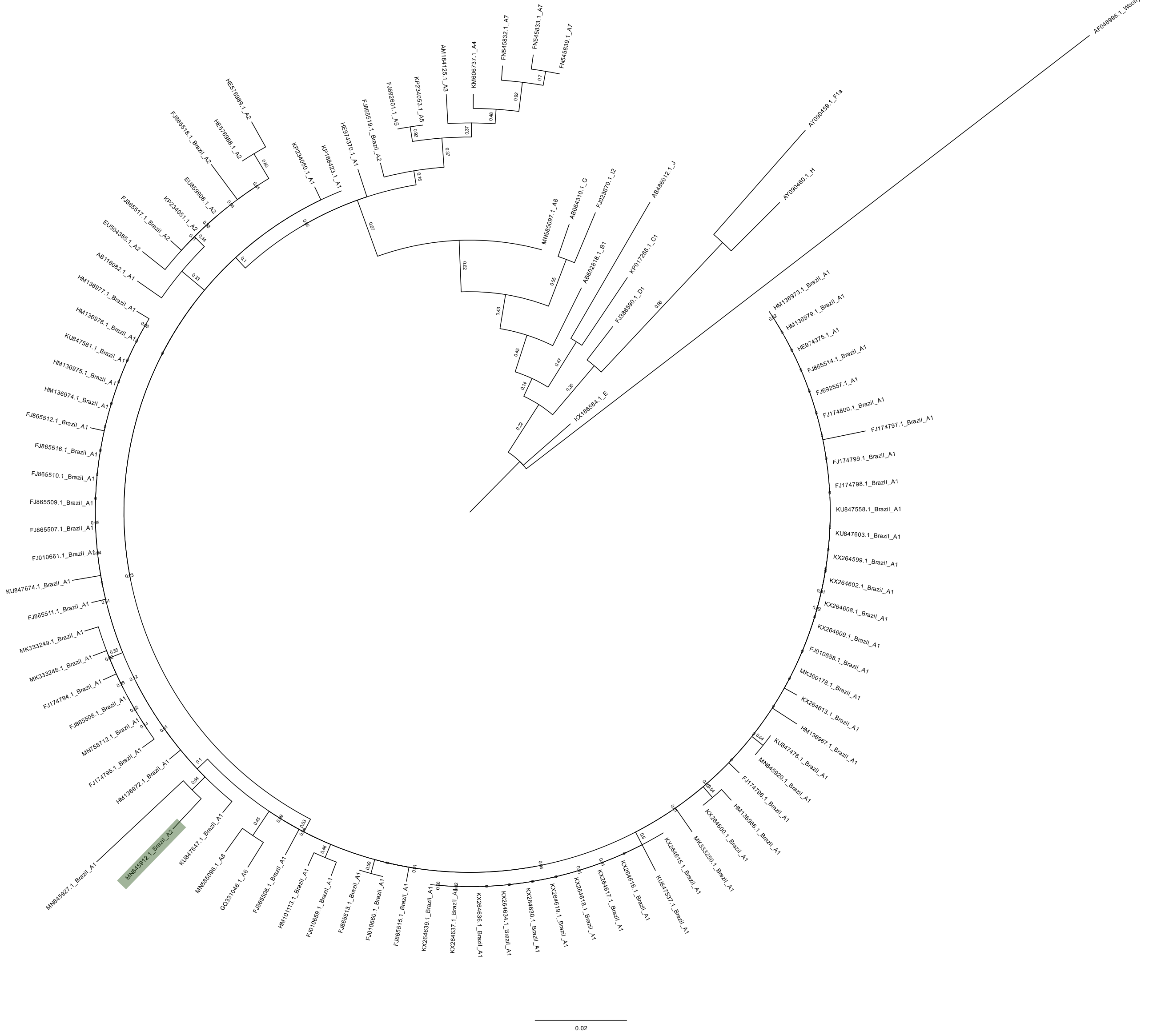

Tree 6. The evolutionary history was inferred by using the Maximum Likelihood method and Tamura-Nei model. The percentage of replicate trees in which the associated taxa clustered together in the bootstrap test (1000 replicates) are shown next to the branches. Initial tree(s) for the heuristic search were obtained automatically by applying Neighbor-Join and BioNJ algorithms to a matrix of pairwise distances estimated using the Tamura-Nei model, and then selecting the topology with superior log likelihood value. A discrete Gamma distribution was used to model evolutionary rate differences among sites (5 categories (+*G*, parameter = 0.2018)). The tree is drawn to scale, with branch lengths measured in the number of substitutions per site. The analysis involved 97 nucleotide sequences, of which 31 were used as marker sequences to determine the genotype of 66 sequences. Sequence MN845912 was recategorized to subgenotype A1. All positions containing gaps and missing data were eliminated. There was a total of 364 positions in the final dataset. Evolutionary analyses were conducted in MEGA X.

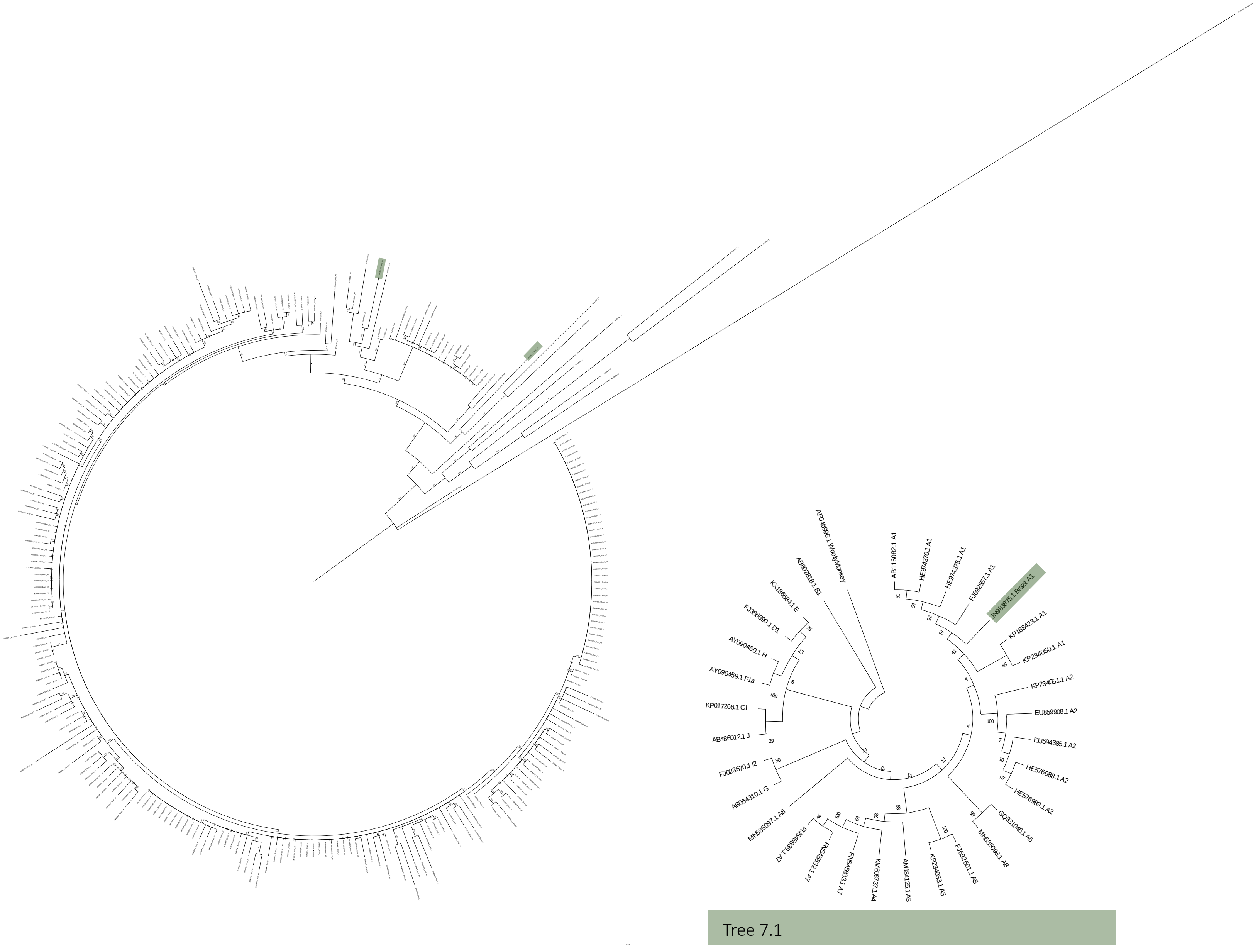

Tree 7.1

Tree 7. The evolutionary history was inferred by using the Maximum Likelihood method and Tamura-Nei model. The percentage of replicate trees in which the associated taxa clustered together in the bootstrap test (1000 replicates) are shown next to the branches. Initial tree(s) for the heuristic search were obtained automatically by applying Neighbor-Join and BioNJ algorithms to a matrix of pairwise distances estimated using the Tamura-Nei model, and then selecting the topology with superior log likelihood value. A discrete Gamma distribution was used to model evolutionary rate differences among sites (5 categories (+*G*, parameter = 0.2191)). The tree is drawn to scale, with branch lengths measured in the number of substitutions per site. The analysis involved 272 nucleotide sequences, of which 31 were used as marker sequences to determine the genotype of 241 sequences. Sequence MN845912 was recategorized to subgenotype A1. Sequence JN983875 demonstrated uncertain grouping and was further analyzed in an additional phylogenetic tree (Tree 7.1) which confirmed it’s genotype as A1. All positions containing gaps and missing data were eliminated. There was a total of 932 positions in the final dataset. Evolutionary analyses were conducted in MEGA X.

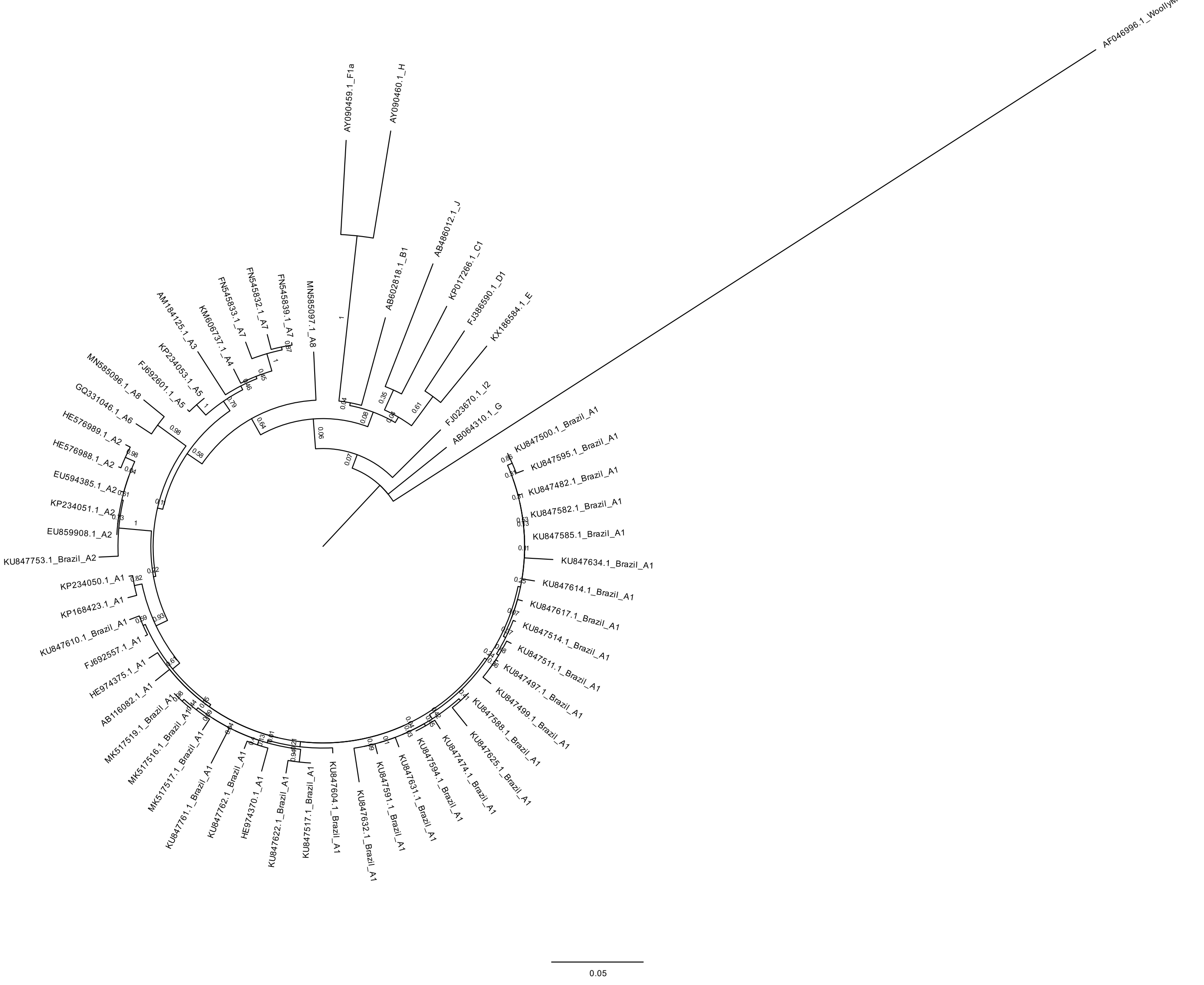

Tree 8. The evolutionary history was inferred by using the Maximum Likelihood method and Tamura-Nei model. The percentage of replicate trees in which the associated taxa clustered together in the bootstrap test (1000 replicates) are shown next to the branches. Initial tree(s) for the heuristic search were obtained automatically by applying Neighbor-Join and BioNJ algorithms to a matrix of pairwise distances estimated using the Tamura-Nei model, and then selecting the topology with superior log likelihood value. A discrete Gamma distribution was used to model evolutionary rate differences among sites (5 categories (+*G*, parameter = 0.1825)). The tree is drawn to scale, with branch lengths measured in the number of substitutions per site. The analysis involved 60 nucleotide sequences, of which 31 were used as marker sequences to determine the genotype of 29 sequences. All positions containing gaps and missing data were eliminated. There was a total of 938 positions in the final dataset. Evolutionary analyses were conducted in MEGA X.

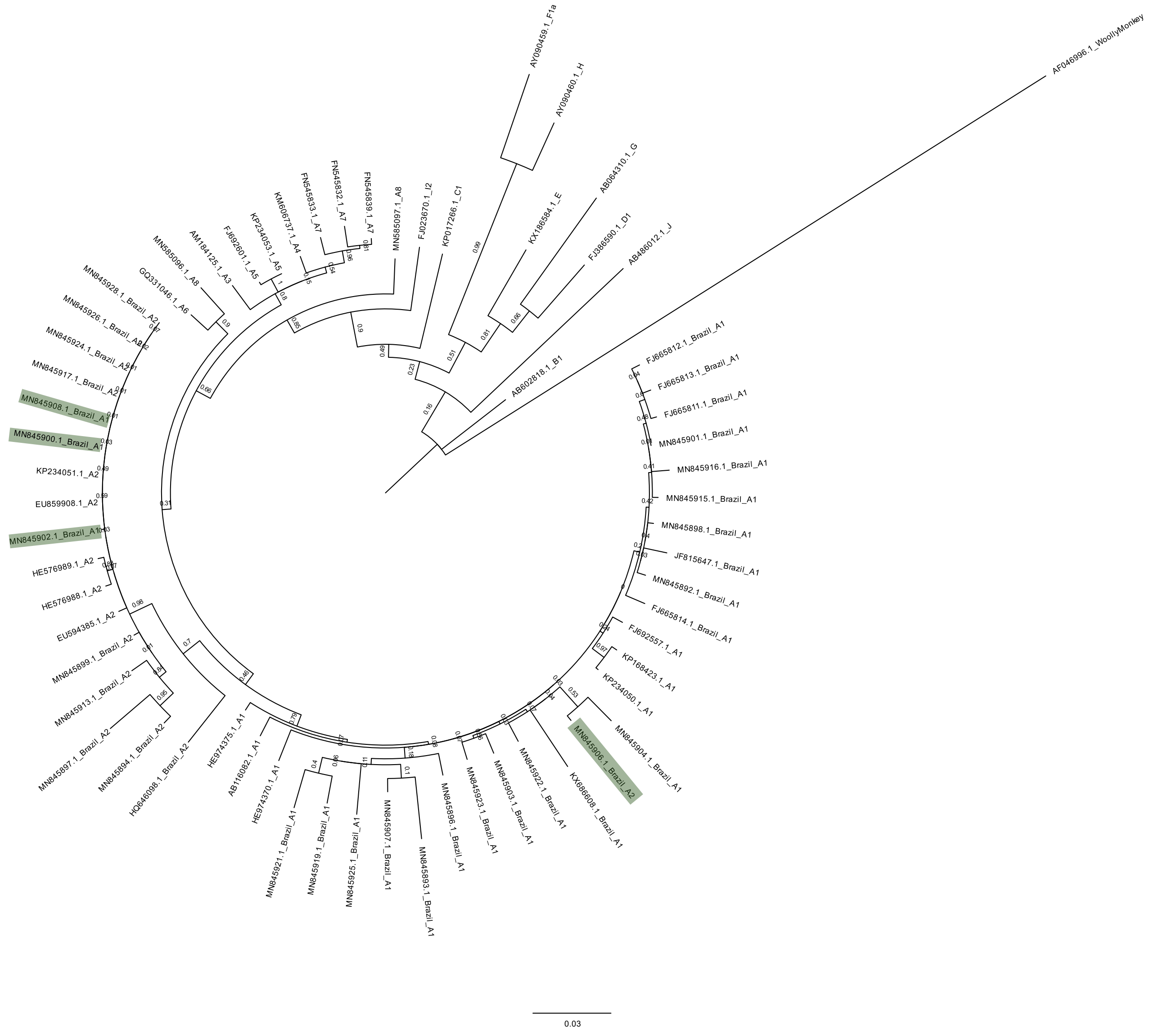

Tree 9. The evolutionary history was inferred by using the Maximum Likelihood method and Tamura-Nei model. The percentage of replicate trees in which the associated taxa clustered together in the bootstrap test (1000 replicates) are shown next to the branches. Initial tree(s) for the heuristic search were obtained automatically by applying Neighbor-Join and BioNJ algorithms to a matrix of pairwise distances estimated using the Tamura-Nei model, and then selecting the topology with superior log likelihood value. A discrete Gamma distribution was used to model evolutionary rate differences among sites (5 categories (+*G*, parameter = 0.2862)). The tree is drawn to scale, with branch lengths measured in the number of substitutions per site. The analysis involved 65 nucleotide sequences, of which 31 were used as marker sequences to determine the genotype of 34 sequences. Sequences MN845900, MN845902, MN845906. and MN845908 were recategorized. All positions containing gaps and missing data were eliminated. There was a total of 895 positions in the final dataset. Evolutionary analyses were conducted in MEGA X.

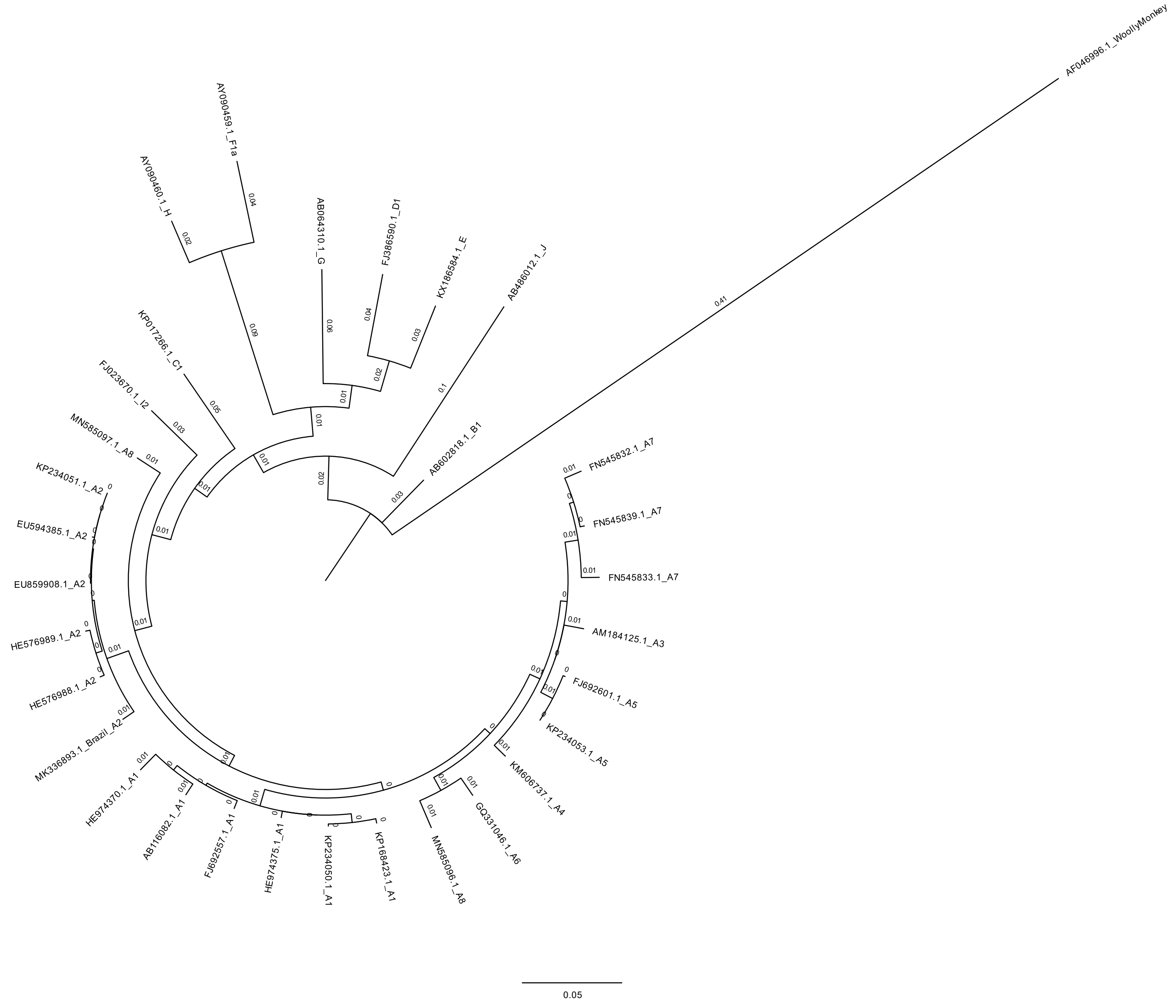

Tree 10. The evolutionary history was inferred by using the Maximum Likelihood method and Tamura-Nei model. The percentage of replicate trees in which the associated taxa clustered together in the bootstrap test (1000 replicates) are shown next to the branches. Initial tree(s) for the heuristic search were obtained automatically by applying Neighbor-Join and BioNJ algorithms to a matrix of pairwise distances estimated using the Tamura-Nei model, and then selecting the topology with superior log likelihood value. A discrete Gamma distribution was used to model evolutionary rate differences among sites (5 categories (+*G*, parameter = 0.2345)). The tree is drawn to scale, with branch lengths measured in the number of substitutions per site. The analysis involved 32 nucleotide sequences, of which 31 were used as marker sequences to determine the genotype of 1 sequence. All positions containing gaps and missing data were eliminated. There was a total of 931 positions in the final dataset. Evolutionary analyses were conducted in MEGA X.

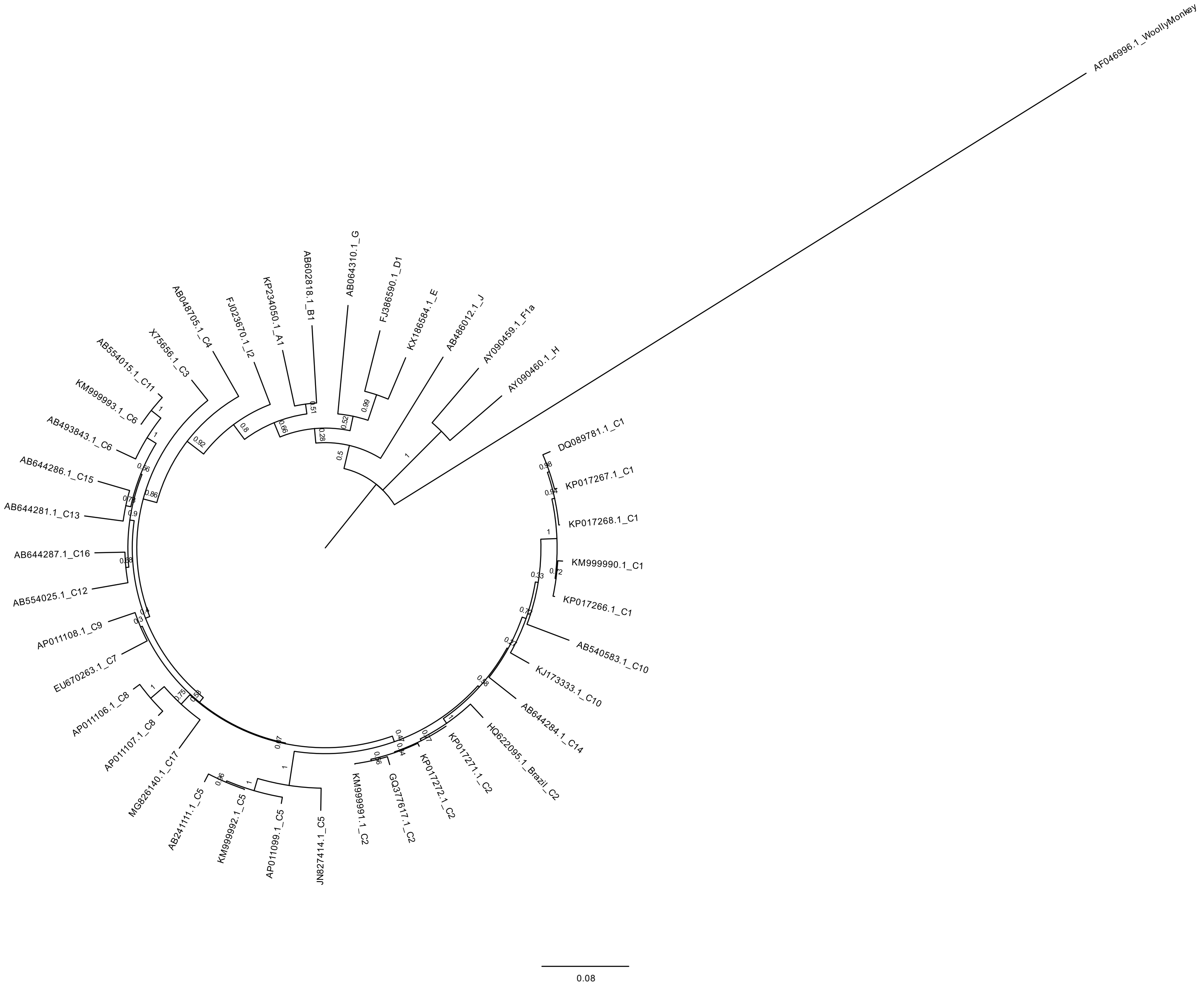

Tree 11. The evolutionary history was inferred by using the Maximum Likelihood method and Tamura-Nei model. The percentage of replicate trees in which the associated taxa clustered together in the bootstrap test (1000 replicates) are shown next to the branches. Initial tree(s) for the heuristic search were obtained automatically by applying Neighbor-Join and BioNJ algorithms to a matrix of pairwise distances estimated using the Tamura-Nei model, and then selecting the topology with superior log likelihood value. A discrete Gamma distribution was used to model evolutionary rate differences among sites (5 categories (+*G*, parameter = 0.2247)). The tree is drawn to scale, with branch lengths measured in the number of substitutions per site. The analysis involved 41 nucleotide sequences, of which 40 were used as marker sequences to determine the genotype of 1 sequence. All positions containing gaps and missing data were eliminated. There was a total of 3140 positions in the final dataset. Evolutionary analyses were conducted in MEGA X.

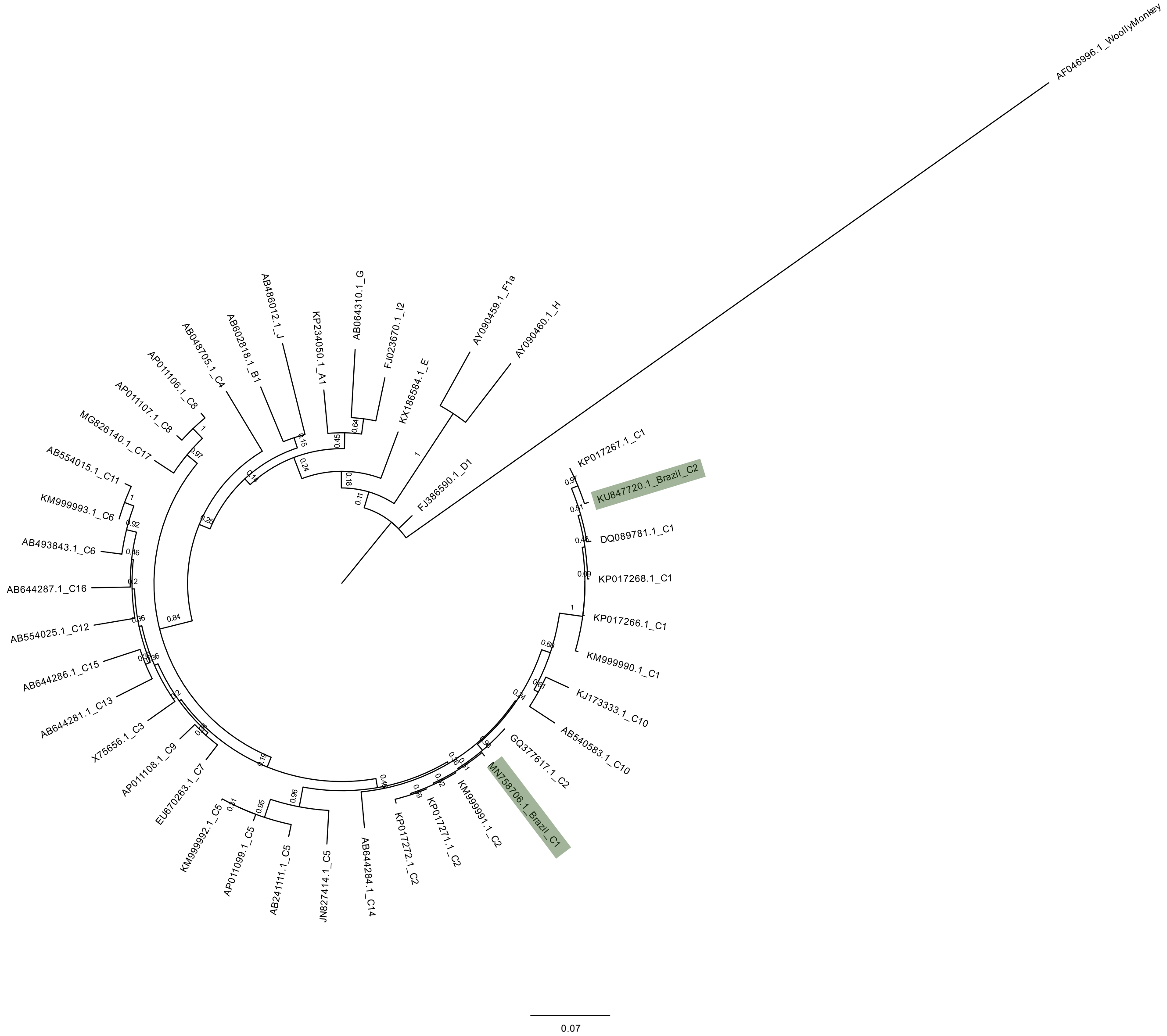

Tree 12. The evolutionary history was inferred by using the Maximum Likelihood method and Tamura-Nei model. The percentage of replicate trees in which the associated taxa clustered together in the bootstrap test (1000 replicates) are shown next to the branches. Initial tree(s) for the heuristic search were obtained automatically by applying Neighbor-Join and BioNJ algorithms to a matrix of pairwise distances estimated using the Tamura-Nei model, and then selecting the topology with superior log likelihood value. A discrete Gamma distribution was used to model evolutionary rate differences among sites (5 categories (+*G*, parameter = 0.1666)). The tree is drawn to scale, with branch lengths measured in the number of substitutions per site. The analysis involved 42 nucleotide sequences, of which 40 were used as marker sequences to determine the genotype of 2 sequences. Both sequences analyzed were recategorized. All positions containing gaps and missing data were eliminated. There was a total of 1025 positions in the final dataset. Evolutionary analyses were conducted in MEGA X.

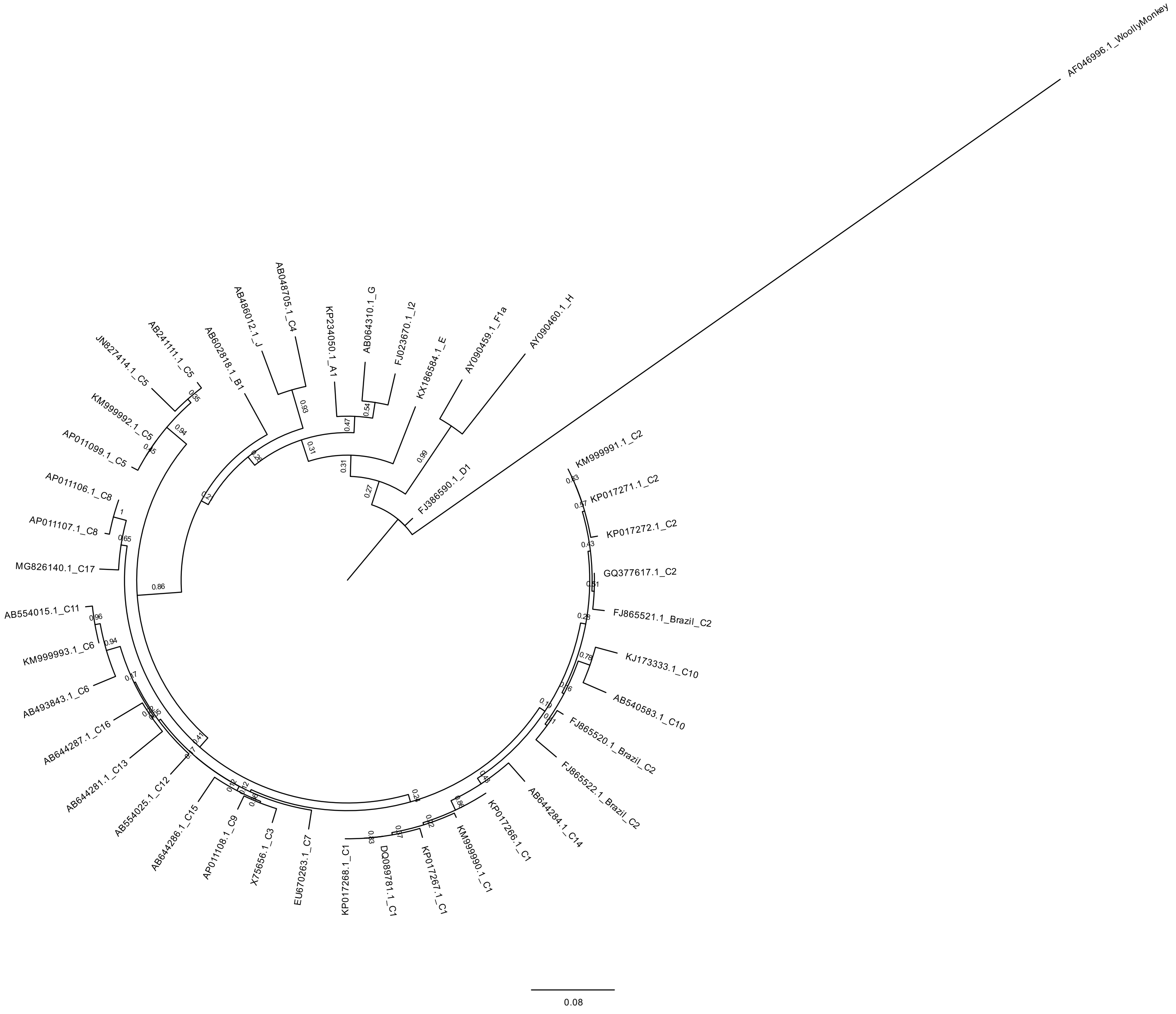

Tree 13. The evolutionary history was inferred by using the Maximum Likelihood method and Tamura-Nei model. The percentage of replicate trees in which the associated taxa clustered together in the bootstrap test (1000 replicates) are shown next to the branches. Initial tree(s) for the heuristic search were obtained automatically by applying Neighbor-Join and BioNJ algorithms to a matrix of pairwise distances estimated using the Tamura-Nei model, and then selecting the topology with superior log likelihood value. A discrete Gamma distribution was used to model evolutionary rate differences among sites (5 categories (+*G*, parameter = 0.1565)). The tree is drawn to scale, with branch lengths measured in the number of substitutions per site. The analysis involved 43 nucleotide sequences, of which 40 were used as marker sequences to determine the genotype of 3 sequences. All positions containing gaps and missing data were eliminated. There was a total of 626 positions in the final dataset. Evolutionary analyses were conducted in MEGA X.

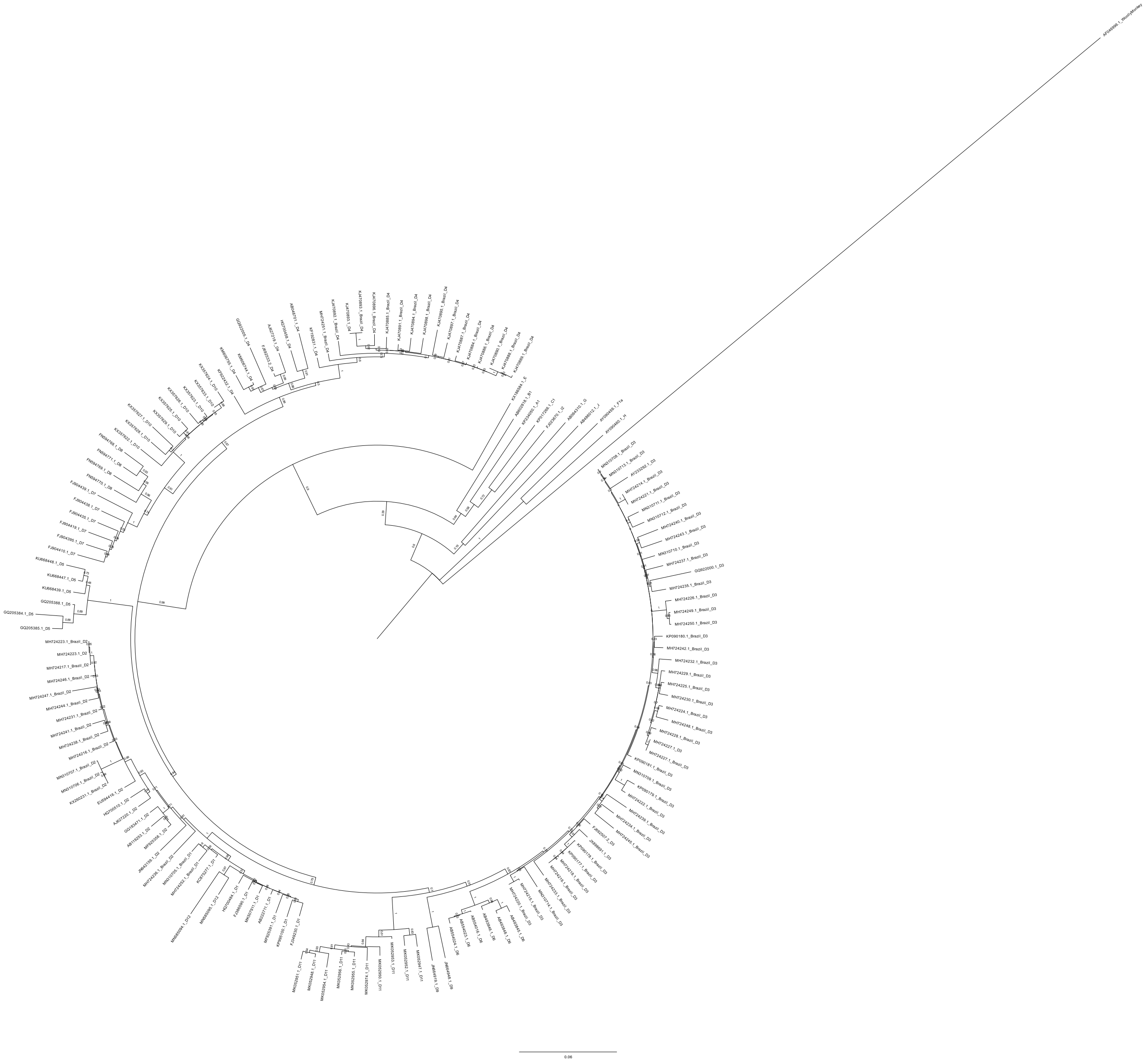

Tree 14. The evolutionary history was inferred by using the Maximum Likelihood method and Tamura-Nei model. The percentage of replicate trees in which the associated taxa clustered together in the bootstrap test (1000 replicates) are shown next to the branches. Initial tree(s) for the heuristic search were obtained automatically by applying Neighbor-Join and BioNJ algorithms to a matrix of pairwise distances estimated using the Tamura-Nei model, and then selecting the topology with superior log likelihood value. A discrete Gamma distribution was used to model evolutionary rate differences among sites (5 categories (+*G*, parameter = 0.2912)). The tree is drawn to scale, with branch lengths measured in the number of substitutions per site. The analysis involved 156 nucleotide sequences, of which 86 were used as marker sequences to determine the genotype of 70 sequences. All positions containing gaps and missing data were eliminated. There was a total of 2889 positions in the final dataset. Evolutionary analyses were conducted in MEGA X.

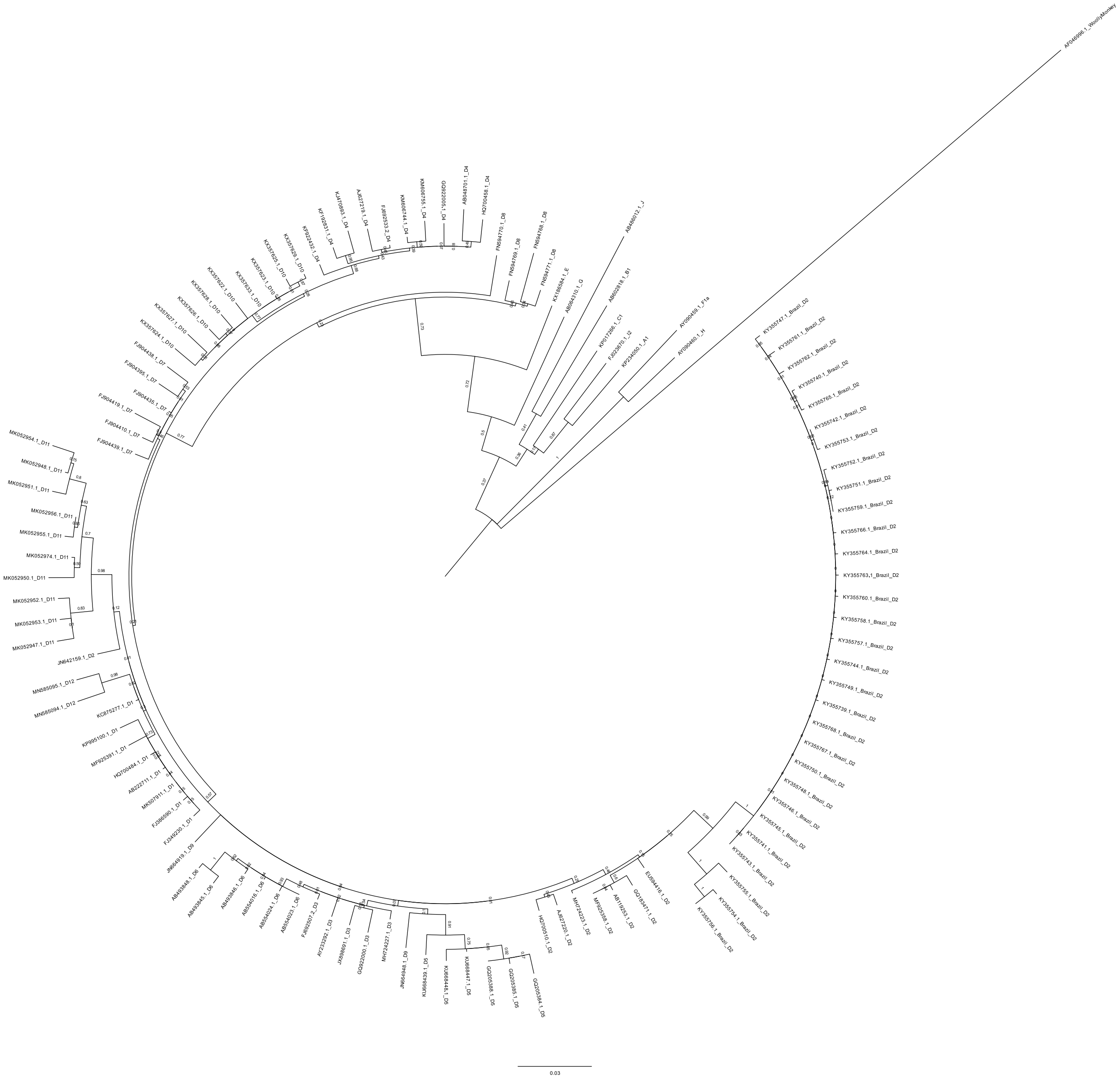

Tree 15. The evolutionary history was inferred by using the Maximum Likelihood method and Tamura-Nei model. The percentage of replicate trees in which the associated taxa clustered together in the bootstrap test (1000 replicates) are shown next to the branches. Initial tree(s) for the heuristic search were obtained automatically by applying Neighbor-Join and BioNJ algorithms to a matrix of pairwise distances estimated using the Tamura-Nei model, and then selecting the topology with superior log likelihood value. A discrete Gamma distribution was used to model evolutionary rate differences among sites (5 categories (+*G*, parameter = 0.3280)). The tree is drawn to scale, with branch lengths measured in the number of substitutions per site. The analysis involved 116 nucleotide sequences, of which 86 were used as marker sequences to determine the genotype of 30 sequences. All positions containing gaps and missing data were eliminated. There was a total of 1004 positions in the final dataset. Evolutionary analyses were conducted in MEGA X.

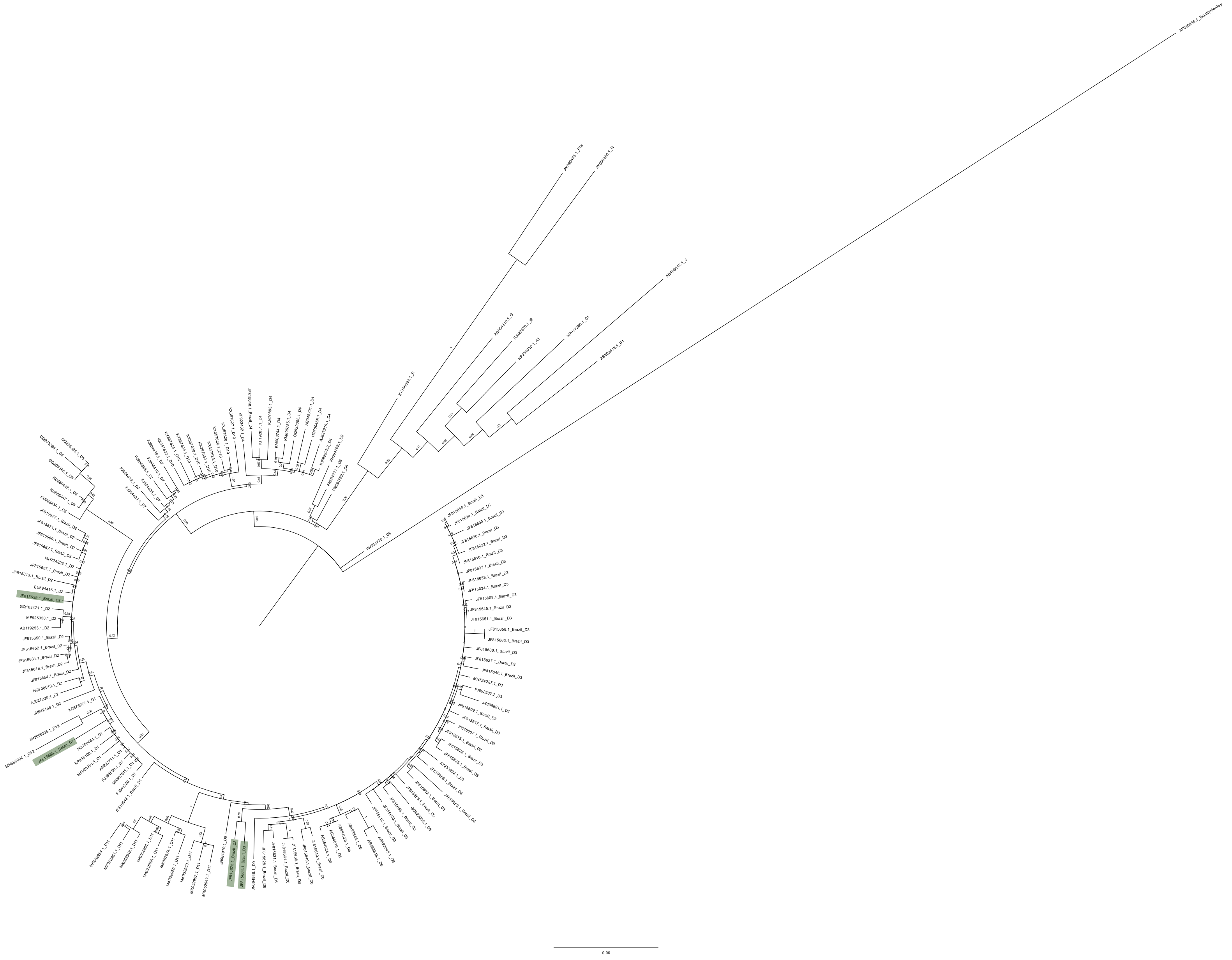

Tree 16. The evolutionary history was inferred by using the Maximum Likelihood method and Tamura-Nei model. The percentage of replicate trees in which the associated taxa clustered together in the bootstrap test (1000 replicates) are shown next to the branches. Initial tree(s) for the heuristic search were obtained automatically by applying Neighbor-Join and BioNJ algorithms to a matrix of pairwise distances estimated using the Tamura-Nei model, and then selecting the topology with superior log likelihood value. A discrete Gamma distribution was used to model evolutionary rate differences among sites (5 categories (+*G*, parameter = 0.2725)). The tree is drawn to scale, with branch lengths measured in the number of substitutions per site. The analysis involved 139 nucleotide sequences, of which 86 were used as marker sequences to determine the genotype of 53 sequences. Sequences JF815636, JF815639, JF815664, and JF815675 were reclassified in accordance to our results in posterior phylogenetic trees. All positions containing gaps and missing data were eliminated. There was a total of 1134 positions in the final dataset. Evolutionary analyses were conducted in MEGA X.

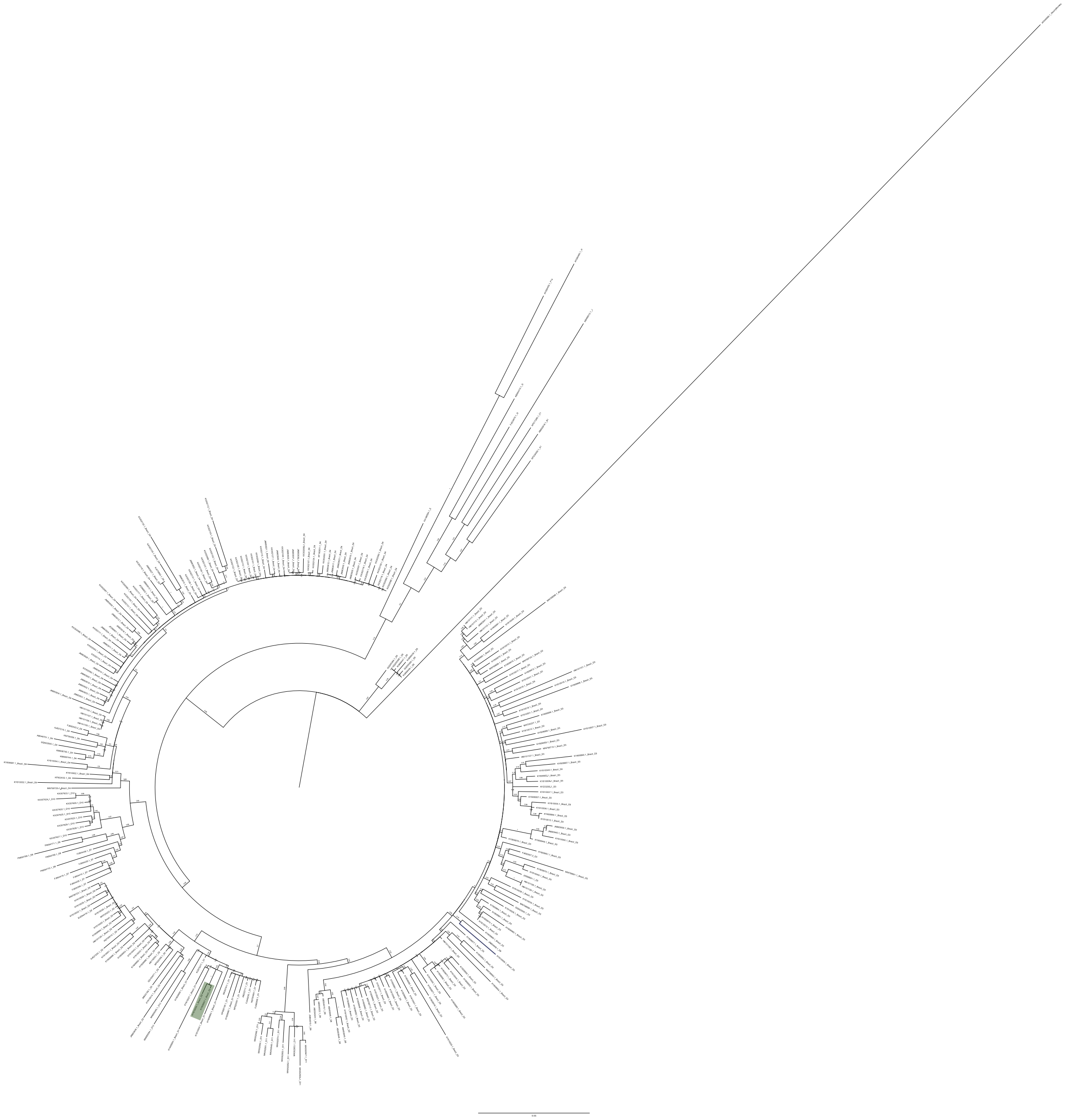

Tree 17. The evolutionary history was inferred by using the Maximum Likelihood method and Tamura-Nei model. The percentage of replicate trees in which the associated taxa clustered together in the bootstrap test (1000 replicates) are shown next to the branches. Initial tree(s) for the heuristic search were obtained automatically by applying Neighbor-Join and BioNJ algorithms to a matrix of pairwise distances estimated using the Tamura-Nei model, and then selecting the topology with superior log likelihood value. A discrete Gamma distribution was used to model evolutionary rate differences among sites. The tree is drawn to scale, with branch lengths measured in the number of substitutions per site. The analysis involved 284 nucleotide sequences, of which 86 were used as marker sequences to determine the genotype of 198 sequences. Sequences KY809947 and KY810030 were reclassified to subgenotype D1. All positions containing gaps and missing data were eliminated. Evolutionary analyses were conducted in MEGA X.

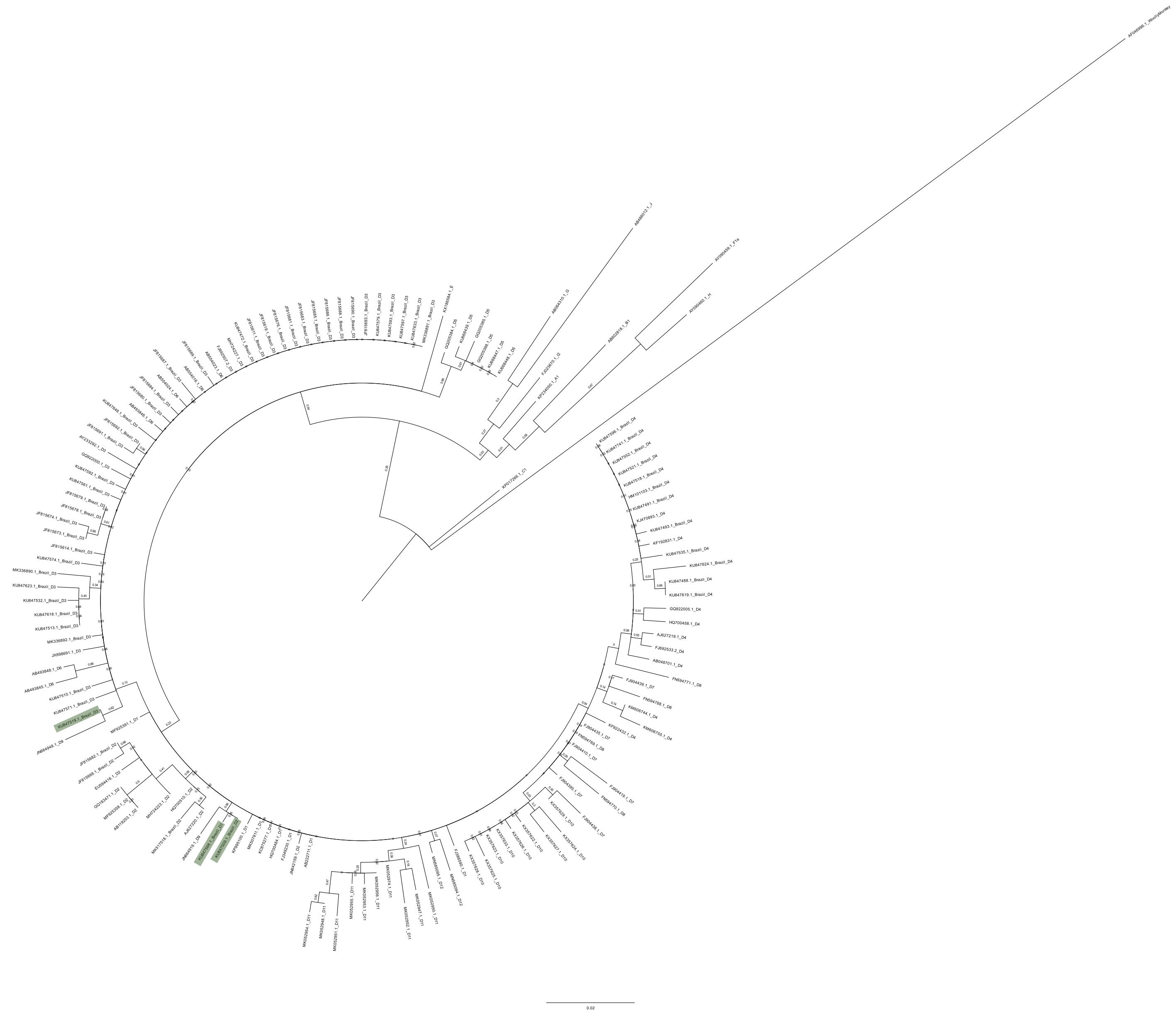

Tree 18. The evolutionary history was inferred by using the Maximum Likelihood method and Tamura-Nei model. The percentage of replicate trees in which the associated taxa clustered together in the bootstrap test (1000 replicates) are shown next to the branches. Initial tree(s) for the heuristic search were obtained automatically by applying Neighbor-Join and BioNJ algorithms to a matrix of pairwise distances estimated using the Tamura-Nei model, and then selecting the topology with superior log likelihood value. A discrete Gamma distribution was used to model evolutionary rate differences among sites (5 categories (+*G*, parameter = 0.1987)). The tree is drawn to scale, with branch lengths measured in the number of substitutions per site. The analysis involved 143 nucleotide sequences, of which 86 were used as marker sequences to determine the genotype of 57 sequences. Sequences KU847509, KU847519, and KU847568 were recategorized as subgenotype D9. All positions containing gaps and missing data were eliminated. There was a total of 504 positions in the final dataset. Evolutionary analyses were conducted in MEGA X.

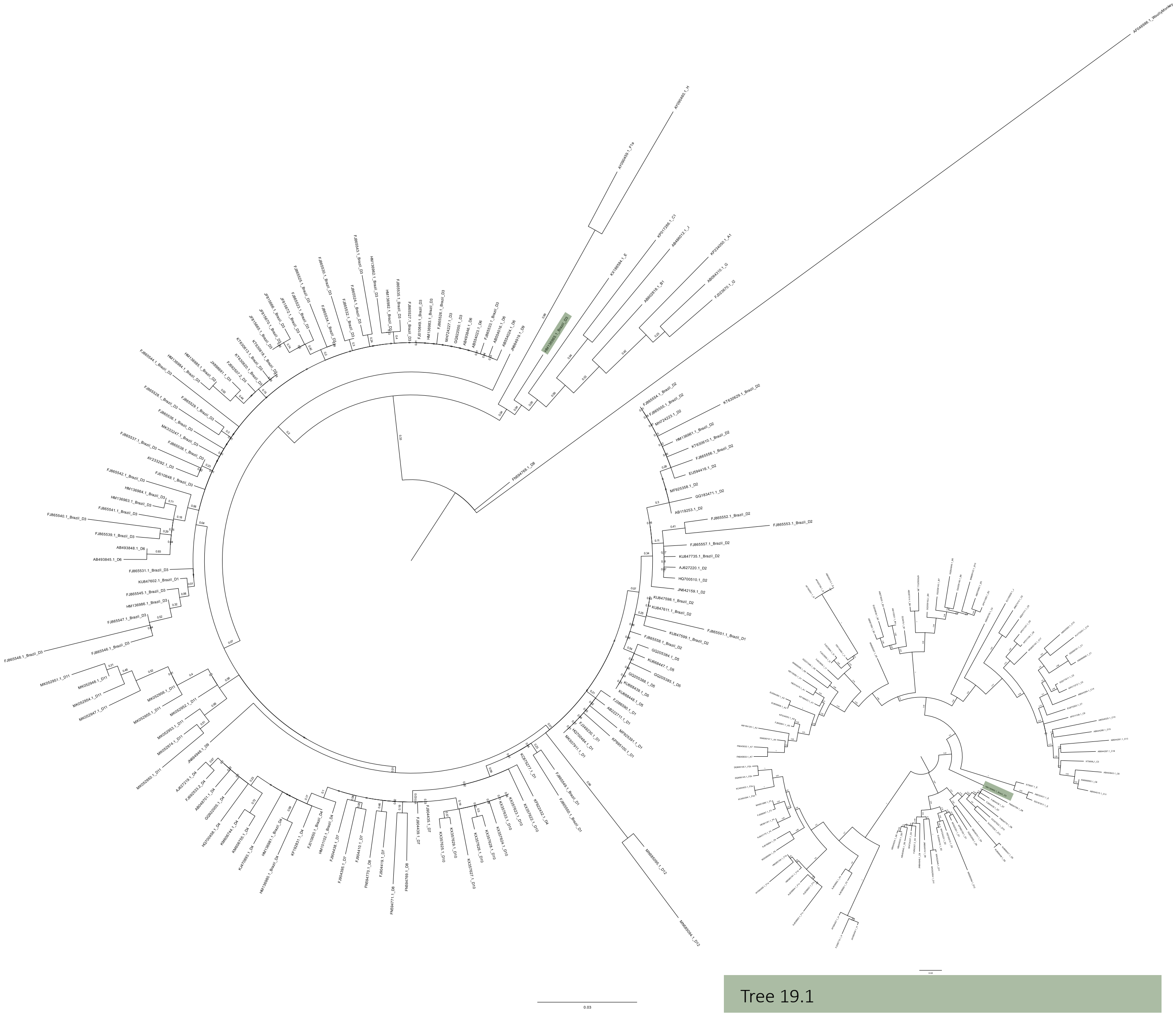

Tree 19.1

Tree 19. The evolutionary history was inferred by using the Maximum Likelihood method and Tamura-Nei model. The percentage of replicate trees in which the associated taxa clustered together in the bootstrap test (1000 replicates) are shown next to the branches. Initial tree(s) for the heuristic search were obtained automatically by applying Neighbor-Join and BioNJ algorithms to a matrix of pairwise distances estimated using the Tamura-Nei model, and then selecting the topology with superior log likelihood value. A discrete Gamma distribution was used to model evolutionary rate differences among sites (5 categories (+*G*, parameter = 0.2894)). The tree is drawn to scale, with branch lengths measured in the number of substitutions per site. The analysis involved 153 nucleotide sequences, of which 86 were used as marker sequences to determine the genotype of 67 sequences. 11 sequences were recategorized. Despite being previously classified as genotype D, sequence HM136965 retained an unclear alignment with any genotype in further analysis (Tree 19.1) and we replaced its classification as NA. All positions containing gaps and missing data were eliminated. There was a total of 405 positions in the final dataset. Evolutionary analyses were conducted in MEGA X.

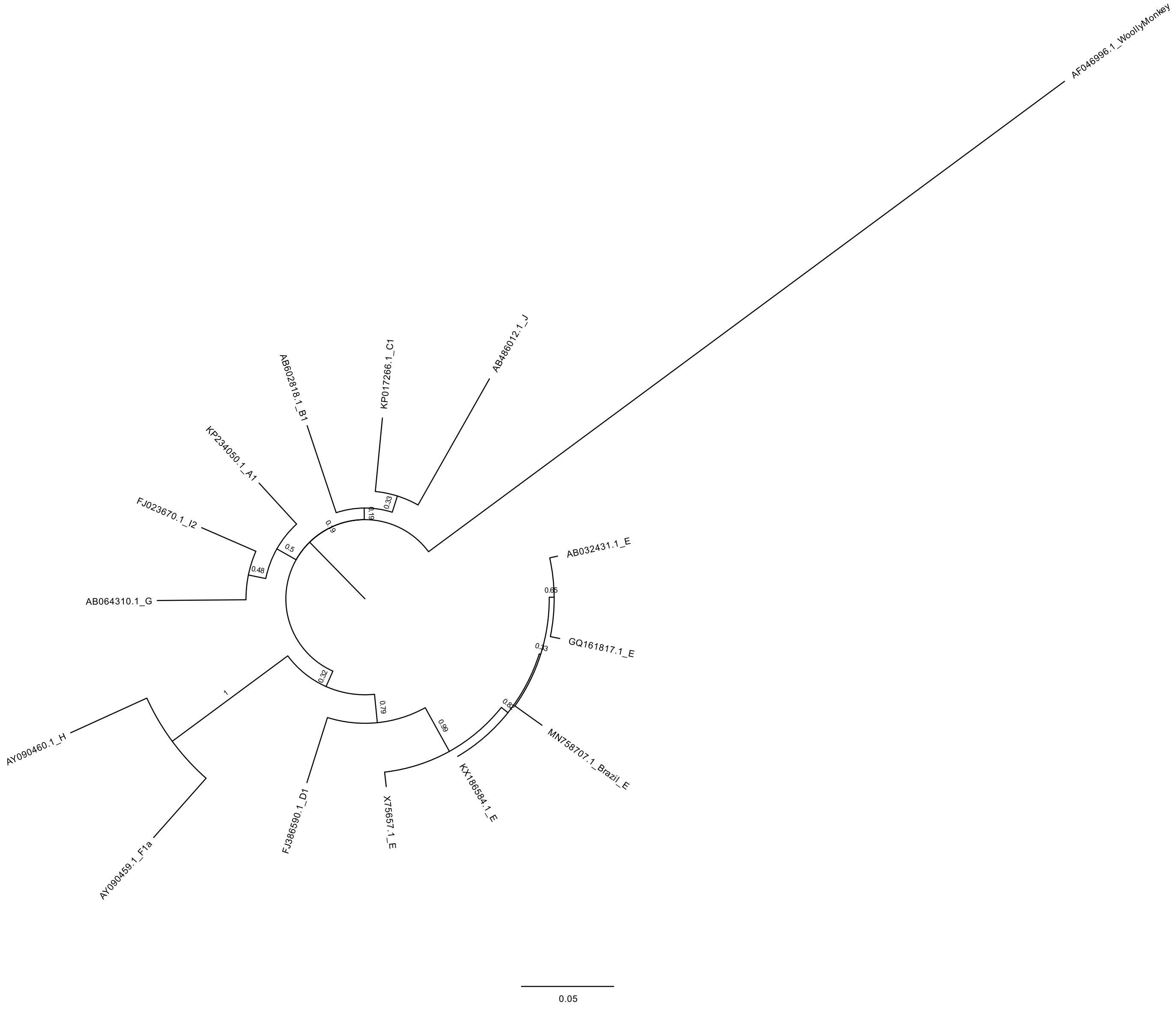

Tree 20. The evolutionary history was inferred by using the Maximum Likelihood method and Tamura-Nei model. The percentage of replicate trees in which the associated taxa clustered together in the bootstrap test (1000 replicates) are shown next to the branches. Initial tree(s) for the heuristic search were obtained automatically by applying Neighbor-Join and BioNJ algorithms to a matrix of pairwise distances estimated using the Tamura-Nei model, and then selecting the topology with superior log likelihood value. A discrete Gamma distribution was used to model evolutionary rate differences among sites (5 categories (+*G*, parameter = 0.1866)). The tree is drawn to scale, with branch lengths measured in the number of substitutions per site. The analysis involved 15 nucleotide sequences, of which 14 were used as marker sequences to determine the genotype of 1 sequence. All positions containing gaps and missing data were eliminated. There was a total of 1239 positions in the final dataset. Evolutionary analyses were conducted in MEGA X.

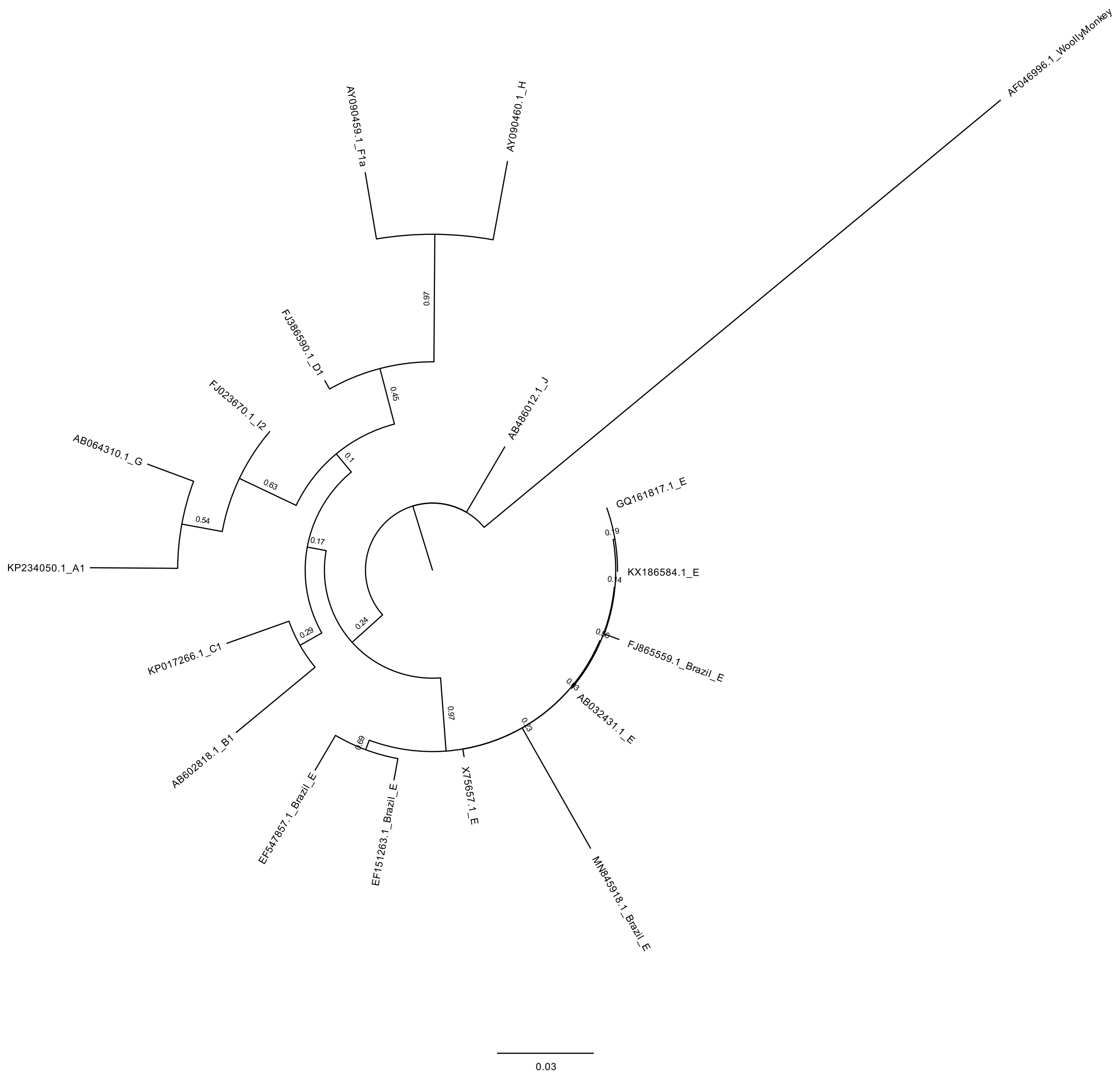

Tree 21. The evolutionary history was inferred by using the Maximum Likelihood method and Tamura-Nei model. The percentage of replicate trees in which the associated taxa clustered together in the bootstrap test (1000 replicates) are shown next to the branches. Initial tree(s) for the heuristic search were obtained automatically by applying Neighbor-Join and BioNJ algorithms to a matrix of pairwise distances estimated using the Tamura-Nei model, and then selecting the topology with superior log likelihood value. A discrete Gamma distribution was used to model evolutionary rate differences among sites (5 categories (+*G*, parameter = 0.1588)). The tree is drawn to scale, with branch lengths measured in the number of substitutions per site. The analysis involved 18 nucleotide sequences, of which 14 were used as marker sequences to determine the genotype of 4 sequences. All positions containing gaps and missing data were eliminated. There was a total of 448 positions in the final dataset. Evolutionary analyses were conducted in MEGA X.

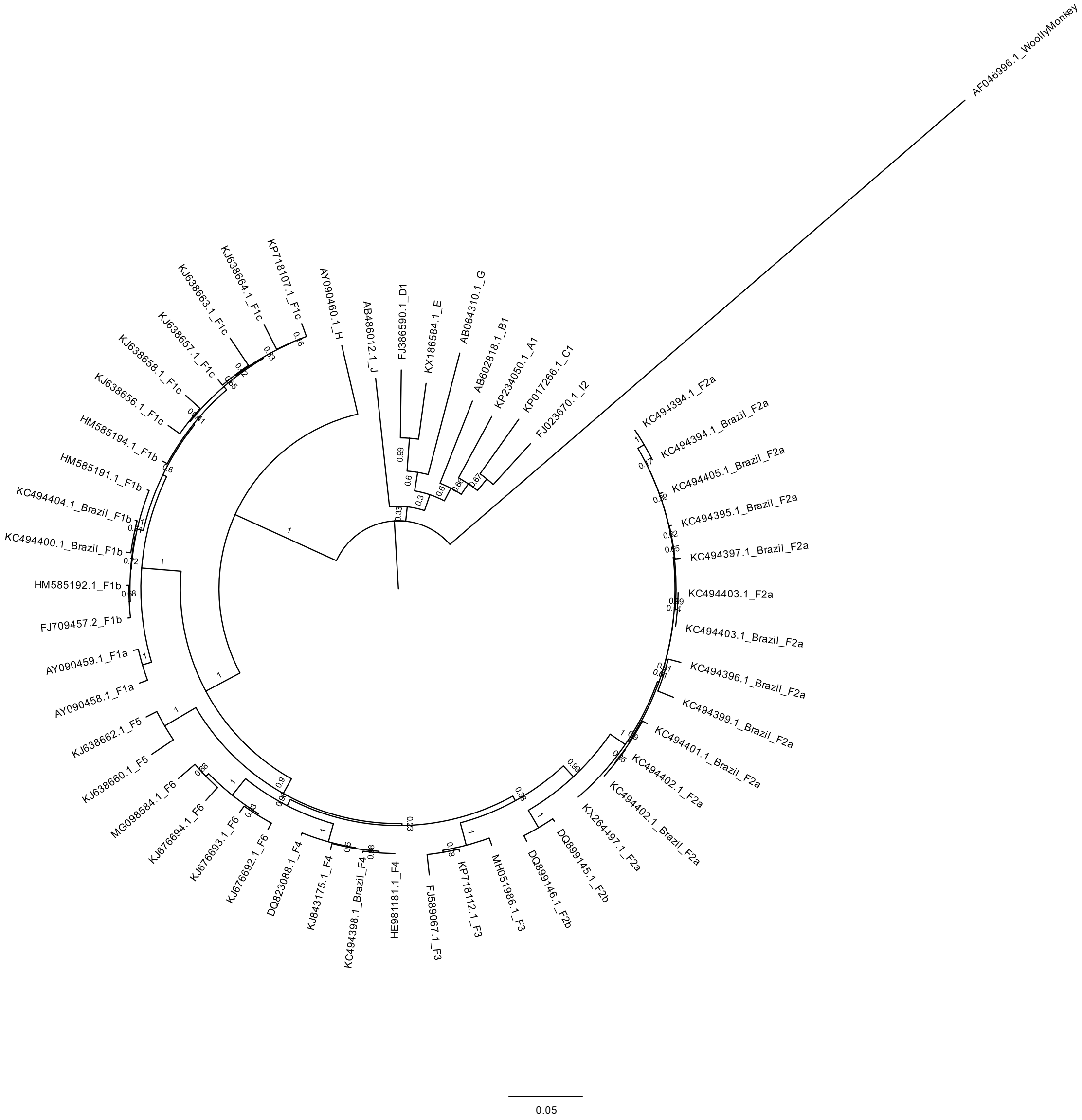

Tree 22. The evolutionary history was inferred by using the Maximum Likelihood method and Tamura-Nei model. The percentage of replicate trees in which the associated taxa clustered together in the bootstrap test (1000 replicates) are shown next to the branches. Initial tree(s) for the heuristic search were obtained automatically by applying Neighbor-Join and BioNJ algorithms to a matrix of pairwise distances estimated using the Tamura-Nei model, and then selecting the topology with superior log likelihood value. A discrete Gamma distribution was used to model evolutionary rate differences among sites (5 categories (+*G*, parameter = 0.2730)). The tree is drawn to scale, with branch lengths measured in the number of substitutions per site. The analysis involved 52 nucleotide sequences, of which 40 were used as marker sequences to determine the genotype of 12 sequences. All positions containing gaps and missing data were eliminated. There was a total of 3064 positions in the final dataset. Evolutionary analyses were conducted in MEGA X.

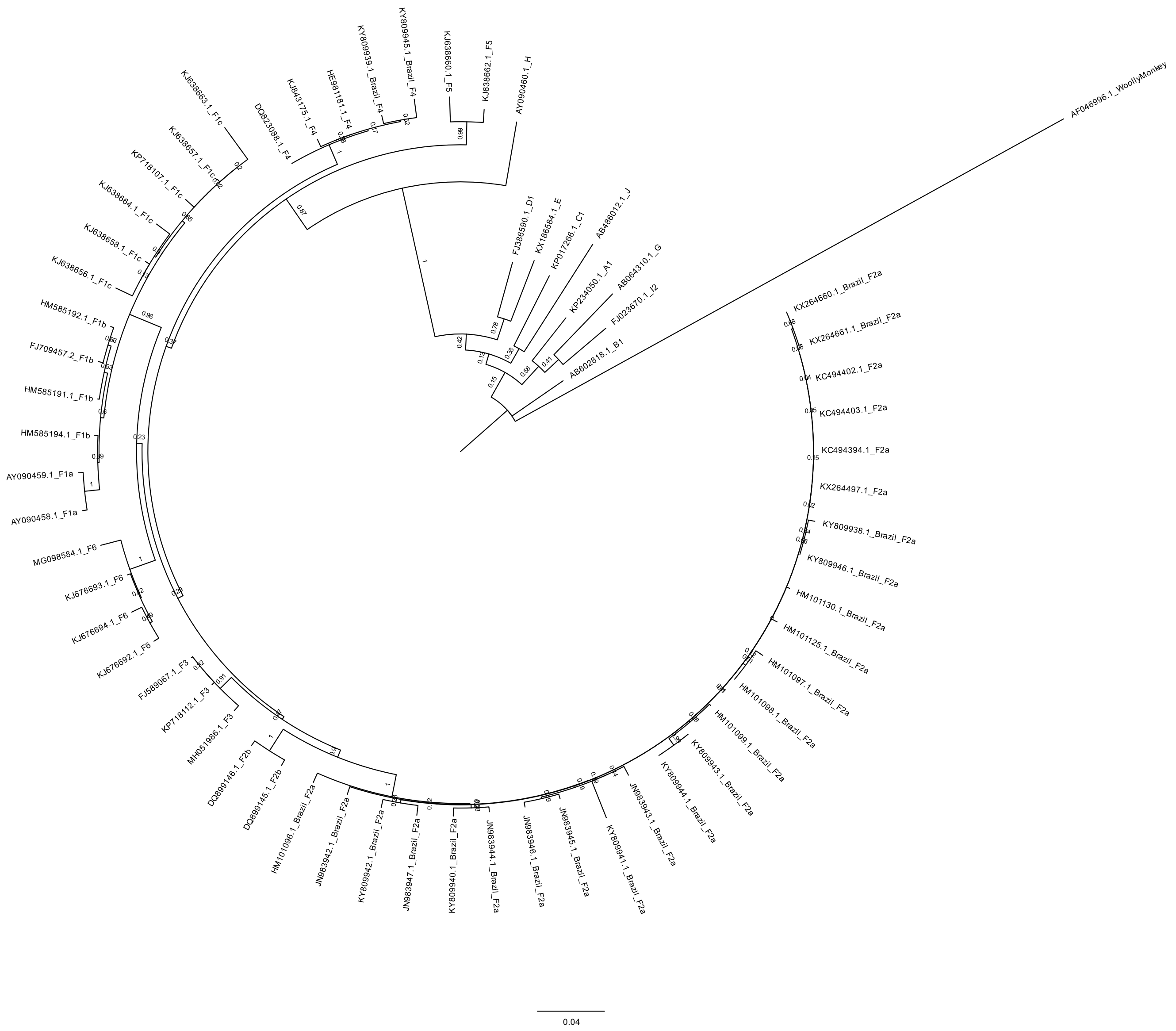

Tree 23. The evolutionary history was inferred by using the Maximum Likelihood method and Tamura-Nei model. The percentage of replicate trees in which the associated taxa clustered together in the bootstrap test (1000 replicates) are shown next to the branches. Initial tree(s) for the heuristic search were obtained automatically by applying Neighbor-Join and BioNJ algorithms to a matrix of pairwise distances estimated using the Tamura-Nei model, and then selecting the topology with superior log likelihood value. A discrete Gamma distribution was used to model evolutionary rate differences among sites (5 categories (+*G*, parameter = 0.2233)). The tree is drawn to scale, with branch lengths measured in the number of substitutions per site. The analysis involved 63 nucleotide sequences, of which 40 were used as marker sequences to determine the genotype of 23 sequences. All positions containing gaps and missing data were eliminated. There was a total of 1123 positions in the final dataset. Evolutionary analyses were conducted in MEGA X.

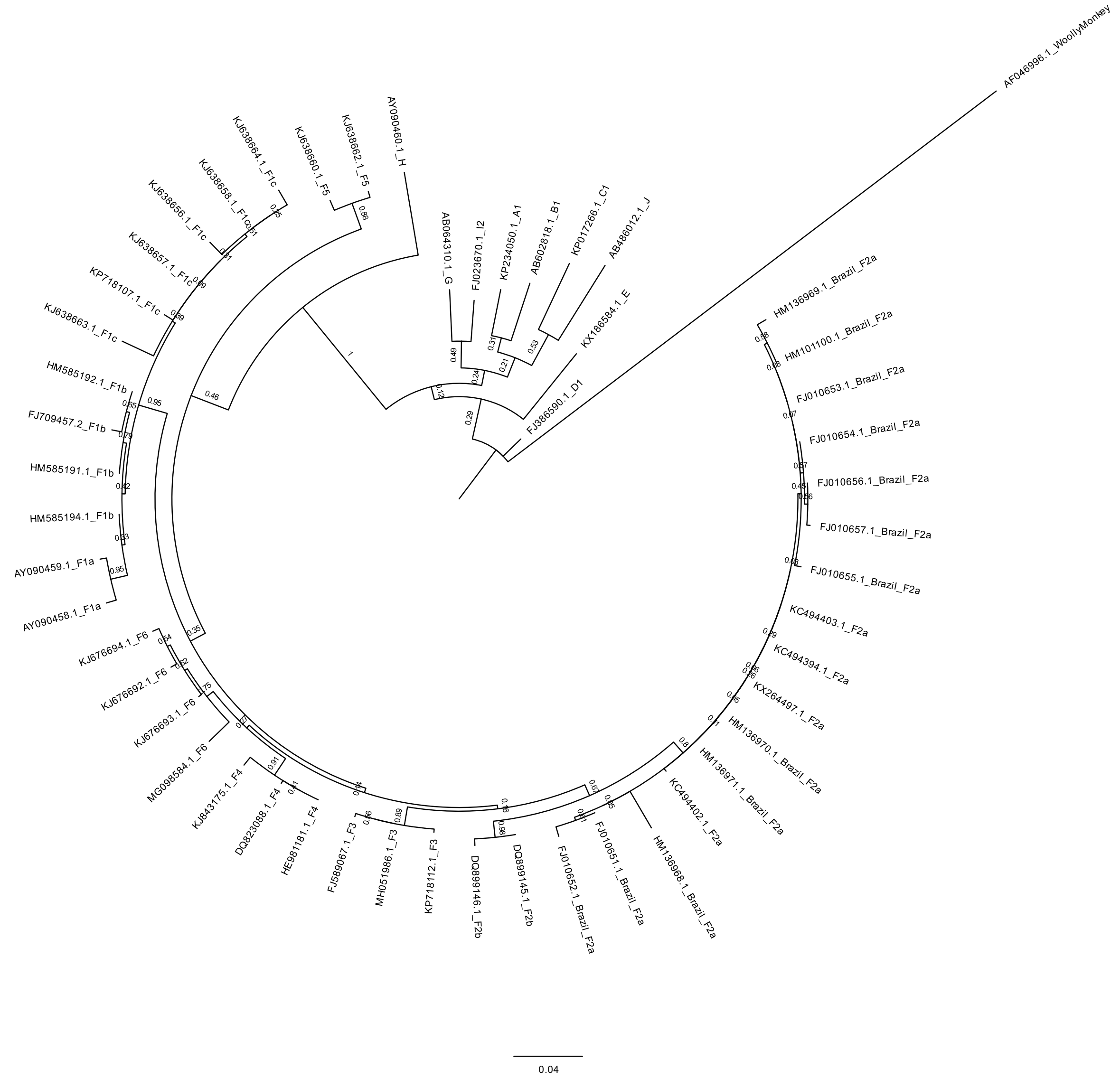

Tree 24. The evolutionary history was inferred by using the Maximum Likelihood method and Tamura-Nei model. The percentage of replicate trees in which the associated taxa clustered together in the bootstrap test (1000 replicates) are shown next to the branches. Initial tree(s) for the heuristic search were obtained automatically by applying Neighbor-Join and BioNJ algorithms to a matrix of pairwise distances estimated using the Tamura-Nei model, and then selecting the topology with superior log likelihood value. A discrete Gamma distribution was used to model evolutionary rate differences among sites (5 categories (+*G*, parameter = 0.1695)). The tree is drawn to scale, with branch lengths measured in the number of substitutions per site. The analysis involved 52 nucleotide sequences, of which 40 were used as marker sequences to determine the genotype of 12 sequences. All positions containing gaps and missing data were eliminated. There was a total of 616 positions in the final dataset. Evolutionary analyses were conducted in MEGA X.

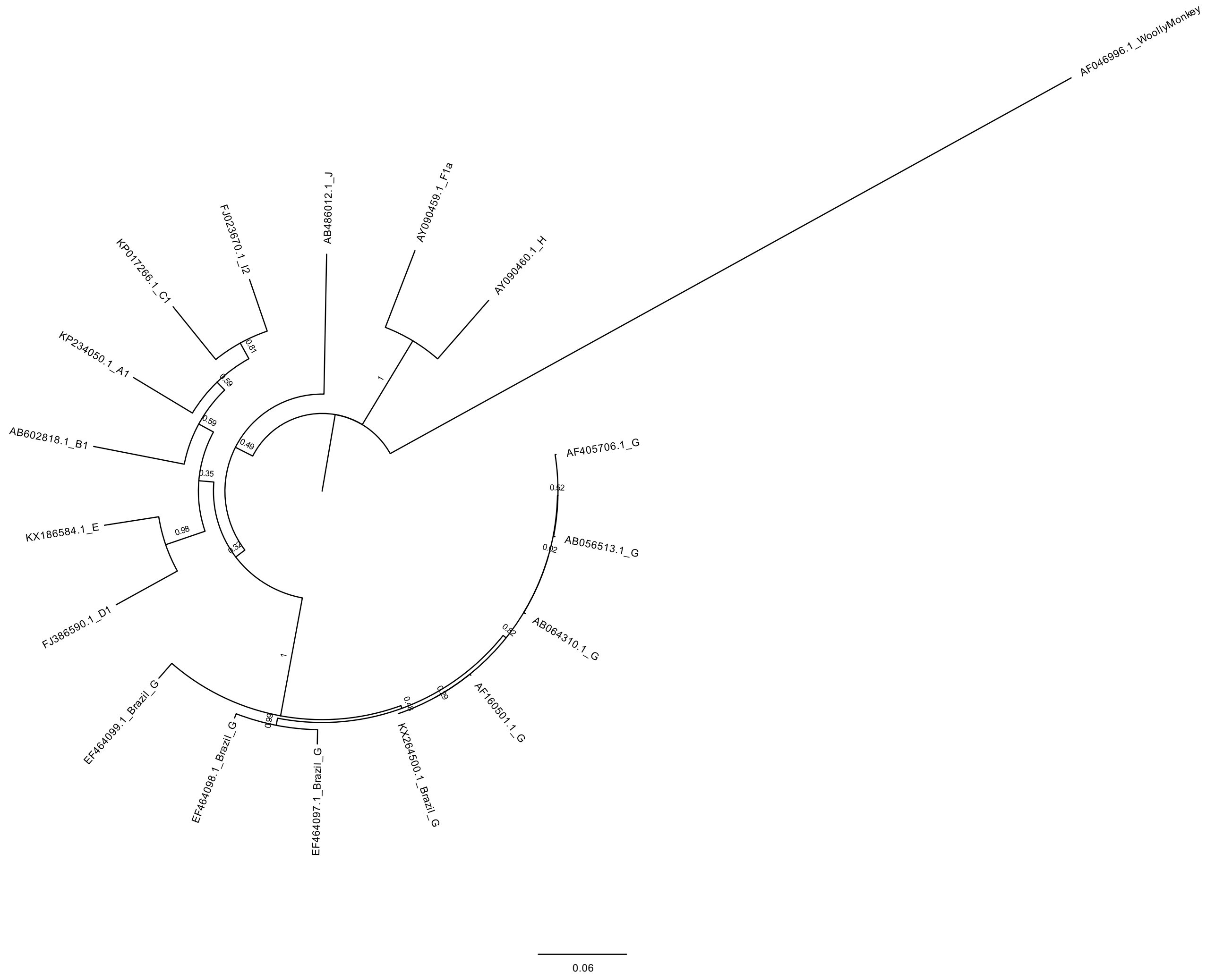

Tree 25. The evolutionary history was inferred by using the Maximum Likelihood method and Tamura-Nei model. The percentage of replicate trees in which the associated taxa clustered together in the bootstrap test (1000 replicates) are shown next to the branches. Initial tree(s) for the heuristic search were obtained automatically by applying Neighbor-Join and BioNJ algorithms to a matrix of pairwise distances estimated using the Tamura-Nei model, and then selecting the topology with superior log likelihood value. A discrete Gamma distribution was used to model evolutionary rate differences among sites (5 categories (+*G*, parameter = 0.2406)). The tree is drawn to scale, with branch lengths measured in the number of substitutions per site. The analysis involved 18 nucleotide sequences, of which 14 were used as marker sequences to determine the genotype of 4 sequences. All positions containing gaps and missing data were eliminated. There was a total of 3169 positions in the final dataset. Evolutionary analyses were conducted in MEGA X.

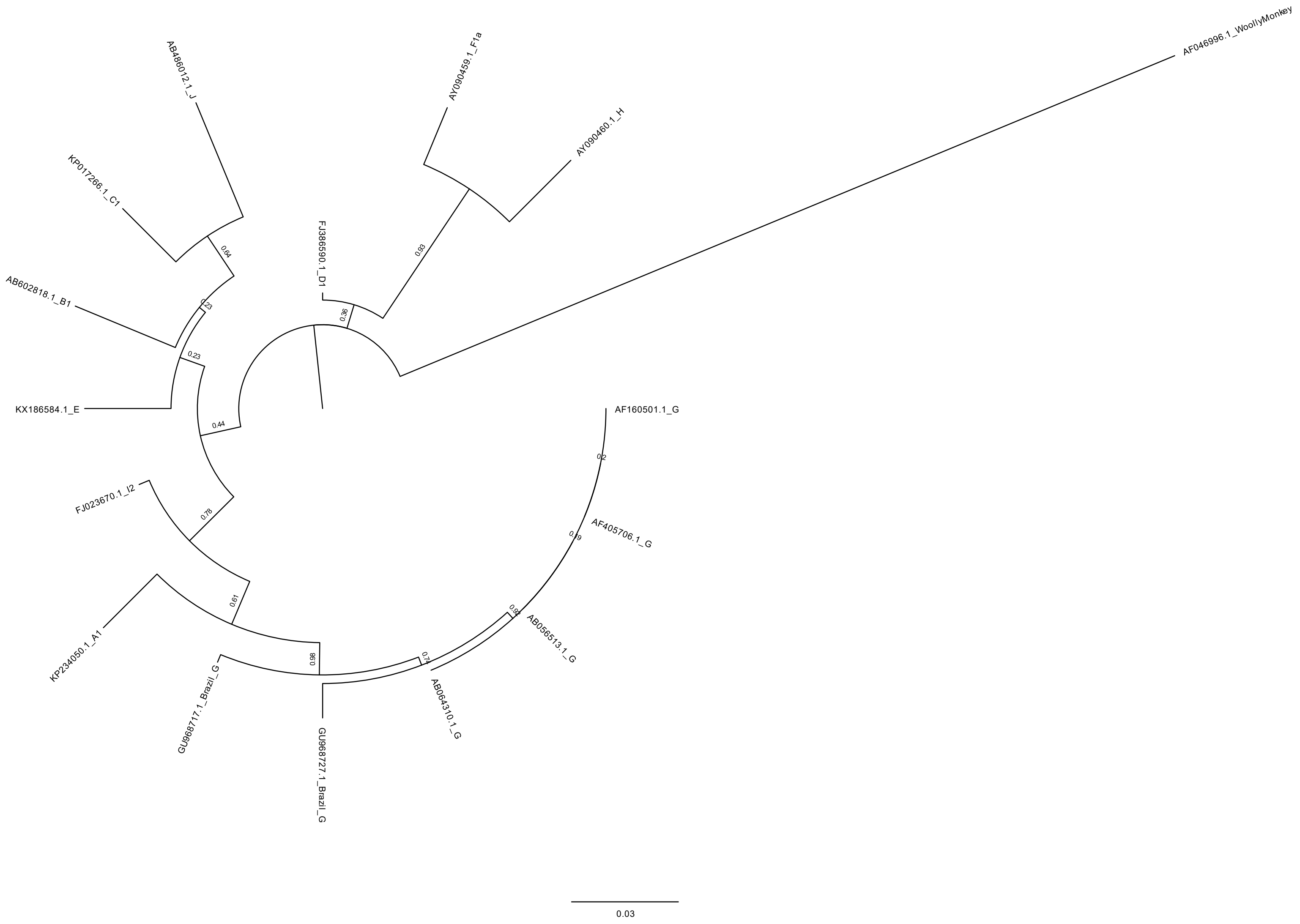

Tree 26. The evolutionary history was inferred by using the Maximum Likelihood method and Tamura-Nei model. The percentage of replicate trees in which the associated taxa clustered together in the bootstrap test (1000 replicates) are shown next to the branches. Initial tree(s) for the heuristic search were obtained automatically by applying Neighbor-Join and BioNJ algorithms to a matrix of pairwise distances estimated using the Tamura-Nei model, and then selecting the topology with superior log likelihood value. A discrete Gamma distribution was used to model evolutionary rate differences among sites (5 categories (+*G*, parameter = 0.1128)). The tree is drawn to scale, with branch lengths measured in the number of substitutions per site. The analysis involved 16 nucleotide sequences, of which 14 were used as marker sequences to determine the genotype of 2 sequences. All positions containing gaps and missing data were eliminated. There was a total of 474 positions in the final dataset. Evolutionary analyses were conducted in MEGA X.

ID	GENOTYPE	SUBTYPE	COUNTRY	TREE	ALIGNMENT[1]	BASE PAIRS
KJ854685	A	A1	Brazil	1	Complete Genome	3221
KJ854686	A	A1	Brazil	1	Complete Genome	3221

KJ854687	A	A1	Brazil	1	Complete Genome	3221
KJ854688	A	A1	Brazil	1	Complete Genome	3221
KJ854689	A	A1	Brazil	1	Complete Genome	3221
KJ854690	A	A1	Brazil	1	Complete Genome	3221
KJ854691	A	A1	Brazil	1	Complete Genome	3221
KJ854692	A	A1	Brazil	1	Complete Genome	3221
KJ854693	A	A1	Brazil	1	Complete Genome	3221
KJ854694	A	A1	Brazil	1	Complete Genome	3221
KJ854695	A	A1	Brazil	1	Complete Genome	3221
KJ854696	A	A1	Brazil	1	Complete Genome	3221
KJ854697	A	A1	Brazil	1	Complete Genome	3221
KJ854698	A	A1	Brazil	1	Complete Genome	3221
KJ854699	A	A1	Brazil	1	Complete Genome	3221
KJ854700	A	A1	Brazil	1	Complete Genome	3221
KJ854701	A	A1	Brazil	1	Complete Genome	3221
KJ854702	A	A1	Brazil	1	Complete Genome	3221
KJ854703	A	A1	Brazil	1	Complete Genome	3221
KJ854704	A	A1	Brazil	1	Complete Genome	3221
KJ854705	A	A1	Brazil	1	Complete Genome	3221
KJ854706	A	A1	Brazil	1	Complete Genome	3221
KJ854707	A	A1	Brazil	1	Complete Genome	3221
KJ854708	A	A2	Brazil	1	Complete Genome	3221
KJ854709	A	A2	Brazil	1	Complete Genome	3221
KJ854710	A	A2	Brazil	1	Complete Genome	3221
HQ646060	A	A1	Brazil	2	1-3182	1178
HQ646061	A	A1	Brazil	2	1-3182	1178
HQ646062	A	A1	Brazil	2	1-3182	1178
HQ646063	A	A1	Brazil	2	1-3182	1178
HQ646064	A	A1	Brazil	2	1-3182	1179
HQ646069	A	A1	Brazil	2	1-3182	1178
HQ646070	A	A1	Brazil	2	1-3182	1178
HQ646071	A	A1	Brazil	2	1-3182	1178
HQ646072	A	A1	Brazil	2	1-3182	1178
HQ646073	A	A1	Brazil	2	1-3182	1178
HQ646074	A	A1	Brazil	2	1-3182	1178
HQ646075	A	A2	Brazil	2	1-3182	1178
HQ646077	A	A1	Brazil	2	1-3182	1178
HQ646078	A	A1	Brazil	2	1-3182	1178
HQ646079	A	A1	Brazil	2	1-3182	1178
HQ646080	A	A1	Brazil	2	1-3182	1178
HQ646081	A	A1	Brazil	2	1-3182	1178

HQ646082	A	A1	Brazil	2	1-3182	1178
HQ646083	A	A1	Brazil	2	1-3182	1178
HQ646084	A	A1	Brazil	2	1-3182	1178
HQ646086	A	A1	Brazil	2	1-3182	1178
HQ646087	A	A1	Brazil	2	1-3182	1178
HQ646088	A	A1	Brazil	2	1-3182	1178
HQ646089	A	A1	Brazil	2	1-3182	1178
HQ646090	A	A1	Brazil	2	1-3182	1178
HQ646091	A	A1	Brazil	2	1-3182	1178
HQ646092	A	A1	Brazil	2	1-3182	1178
HQ646093	A	A1	Brazil	2	1-3182	1178
HQ646094	A	A1	Brazil	2	1-3182	1178
HQ646095	A	A1	Brazil	2	1-3182	1178
HQ646096	A	A1	Brazil	2	1-3182	1178
HQ646097	A	A1	Brazil	2	1-3182	1178
KU847473	A	A1	Brazil	3	132-1163	1032
KU847478	A	A1	Brazil	3	132-1163	1032
KU847481	A	A1	Brazil	3	132-1163	1032
KU847484	A	A1	Brazil	3	132-1163	1032
KU847486	A	A1	Brazil	3	132-1163	1032
KU847489	A	A1	Brazil	3	132-1163	1032
KU847490	A	A1	Brazil	3	132-1163	1032
KU847494	A	A1	Brazil	3	132-1163	1032
KU847495	A	A1	Brazil	3	132-1163	1032
KU847503	A	A1	Brazil	3	132-1163	1032
KU847504	A	A1	Brazil	3	132-1163	1032
KU847505	A	A1	Brazil	3	132-1163	1032
KU847508	A	A1	Brazil	3	132-1163	1032
KU847523	A	A1	Brazil	3	132-1163	1032
KU847526	A	A1	Brazil	3	132-1163	1032
KU847527	A	A1	Brazil	3	132-1163	1032
KU847529	A	A1	Brazil	3	132-1163	1032
KU847531	A	A1	Brazil	3	132-1163	1032
KU847533	A	A1	Brazil	3	132-1163	1032
KU847534	A	A1	Brazil	3	132-1163	1032
KU847541	A	A1	Brazil	3	132-1163	1032
KU847542	A	A1	Brazil	3	132-1163	1032
KU847546	A	A1	Brazil	3	132-1163	1032
KU847547	A	A1	Brazil	3	132-1163	1032
KU847548	A	A1	Brazil	3	132-1163	1032
KU847553	A	A1	Brazil	3	132-1163	1032

KU847554	A	A1	Brazil	3	132-1163	1032
KU847557	A	A1	Brazil	3	132-1163	1032
KU847563	A	A1	Brazil	3	132-1163	1032
KU847567	A	A1	Brazil	3	132-1163	1032
KU847570	A	A1	Brazil	3	132-1163	1032
KU847572	A	A1	Brazil	3	132-1163	1032
KU847573	A	A1	Brazil	3	132-1163	1032
KU847575	A	A1	Brazil	3	132-1163	1032
KU847577	A	A1	Brazil	3	132-1163	1032
KU847578	A	A1	Brazil	3	132-1163	1032
KU847676	A	A1	Brazil	3	132-1163	1032
KU847677	A	A1	Brazil	3	132-1163	1032
KU847678	A	A1	Brazil	3	132-1163	1032
KU847679	A	A1	Brazil	3	132-1163	1032
KU847680	A	A1	Brazil	3	132-1163	1032
KU847681	A	A1	Brazil	3	132-1163	1032
KU847682	A	A1	Brazil	3	132-1163	1032
KU847683	A	A1	Brazil	3	132-1163	1032
KU847684	A	A1	Brazil	3	132-1163	1032
KU847685	A	A1	Brazil	3	132-1163	1032
KU847687	A	A1	Brazil	3	132-1163	1032
KU847688	A	A1	Brazil	3	132-1163	1032
KU847690	A	A1	Brazil	3	132-1163	1032
KU847691	A	A1	Brazil	3	132-1163	1032
KU847692	A	A1	Brazil	3	132-1163	1032
KU847693	A	A1	Brazil	3	132-1163	1032
KU847695	A	A1	Brazil	3	132-1163	1032
KU847696	A	A1	Brazil	3	132-1163	1032
KU847697	A	A1	Brazil	3	132-1163	1032
KU847698	A	A1	Brazil	3	132-1163	1032
KU847699	A	A1	Brazil	3	132-1163	1032
KU847700	A	A1	Brazil	3	132-1163	1032
KU847701	A	A1	Brazil	3	132-1163	1032
KU847702	A	A1	Brazil	3	132-1163	1032
KU847703	A	A1	Brazil	3	132-1163	1032
KU847704	A	A1	Brazil	3	132-1163	1032
KU847705	A	A1	Brazil	3	132-1163	1032
KU847706	A	A1	Brazil	3	132-1163	1032
KU847707	A	A1	Brazil	3	132-1163	1032
KU847708	A	A1	Brazil	3	132-1163	1032
KU847709	A	A1	Brazil	3	132-1163	1032

KU847710	A	A1	Brazil	3	132-1163	1032
KU847711	A	A1	Brazil	3	132-1163	1032
KU847712	A	A1	Brazil	3	132-1163	1032
KU847713	A	A1	Brazil	3	132-1163	1032
KU847714	A	A2	Brazil	3	132-1163	1032
KU847715	A	A2	Brazil	3	132-1163	1032
KU847716	A	A1	Brazil	3	132-1163	1032
KU847717	A	A1	Brazil	3	132-1163	1032
KU847718	A	A2	Brazil	3	132-1163	1032
KU847719	A	A1	Brazil	3	132-1163	1032
KU847721	A	A1	Brazil	3	132-1163	1032
KU847722	A	A1	Brazil	3	132-1163	1032
KU847724	A	A1	Brazil	3	132-1163	1032
KU847725	A	A1	Brazil	3	132-1163	1032
KU847726	A	A1	Brazil	3	132-1163	1032
KU847727	A	A1	Brazil	3	132-1163	1032
KU847729	A	A1	Brazil	3	132-1163	1032
KU847730	A	A1	Brazil	3	132-1163	1032
KU847731	A	A1	Brazil	3	132-1163	1032
KU847732	A	A1	Brazil	3	132-1163	1032
KU847733	A	A1	Brazil	3	132-1163	1032
KU847734	A	A1	Brazil	3	132-1163	1032
KU847736	A	A1	Brazil	3	132-1163	1032
KU847737	A	A1	Brazil	3	132-1163	1032
KU847738	A	A1	Brazil	3	132-1163	1032
KU847739	A	A1	Brazil	3	132-1163	1032
KU847740	A	A1	Brazil	3	132-1163	1032
KU847742	A	A1	Brazil	3	132-1163	1032
KU847743	A	A1	Brazil	3	132-1163	1032
KU847744	A	A1	Brazil	3	132-1163	1032
KU847745	A	A1	Brazil	3	138-1163	1026
KU847746	A	A2	Brazil	3	132-1163	1032
KU847747	A	A1	Brazil	3	132-1163	1032
KU847748	A	A1	Brazil	3	132-1163	1032
KU847749	A	A1	Brazil	3	132-1163	1032
KU847750	A	A1	Brazil	3	132-1163	1032
KU847751	A	A1	Brazil	3	132-1163	1032
KU847752	A	A1	Brazil	3	132-1163	1032
KU847754	A	A1	Brazil	3	132-1163	1032
KU847755	A	A1	Brazil	3	132-1163	1032
KU847756	A	A1	Brazil	3	132-1163	1032

KU847757	A	A1	Brazil	3	132-1163	1032
KU847758	A	A1	Brazil	3	132-1163	1032
KU847759	A	A1	Brazil	3	132-1163	1032
KU847760	A	A1	Brazil	3	132-1163	1032
KU847475	A	A1	Brazil	4	132-932	801
KU847483	A	A1	Brazil	4	132-932	801
KU847487	A	A1	Brazil	4	132-932	801
KU847501	A	A1	Brazil	4	132-932	801
KU847506	A	A1	Brazil	4	132-932	801
KU847520	A	A1	Brazil	4	132-932	801
KU847530	A	A1	Brazil	4	132-932	801
KU847538	A	A1	Brazil	4	132-932	801
KU847539	A	A1	Brazil	4	132-932	801
KU847543	A	A1	Brazil	4	132-932	801
KU847544	A	A1	Brazil	4	132-932	801
KU847545	A	A1	Brazil	4	138-932	795
KU847551	A	A1	Brazil	4	132-932	801
KU847555	A	A1	Brazil	4	132-929	798
KU847556	A	A1	Brazil	4	132-932	801
KU847559	A	A1	Brazil	4	144-929	786
KU847560	A	A1	Brazil	4	132-932	801
KU847564	A	A1	Brazil	4	132-932	801
KU847565	A	A1	Brazil	4	132-929	798
KU847583	A	A1	Brazil	4	132-932	801
KU847584	A	A1	Brazil	4	132-932	801
KU847587	A	A1	Brazil	4	132-932	801
KU847589	A	A1	Brazil	4	132-932	801
KU847601	A	A1	Brazil	4	132-929	798
KU847605	A	A1	Brazil	4	132-932	801
KU847607	A	A1	Brazil	4	132-932	801
KU847609	A	A1	Brazil	4	132-932	801
KU847615	A	A1	Brazil	4	132-932	801
KU847626	A	A1	Brazil	4	132-932	801
KU847629	A	A1	Brazil	4	132-932	801
KU847630	A	A1	Brazil	4	132-932	801
KU847635	A	A1	Brazil	4	162-932	771
KU847636	A	A1	Brazil	4	156-929	774
KU847637	A	A1	Brazil	4	153-932	780
KU847638	A	A1	Brazil	4	153-932	780
KU847639	A	A1	Brazil	4	132-932	801
KU847640	A	A1	Brazil	4	132-932	801

KU847641	A	A1	Brazil	4	132-932	801
KU847642	A	A1	Brazil	4	138-932	795
KU847643	A	A1	Brazil	4	132-932	801
KU847644	A	A1	Brazil	4	159-932	774
KU847645	A	A1	Brazil	4	132-935	804
KU847646	A	A1	Brazil	4	132-932	801
KU847649	A	A1	Brazil	4	132-932	801
KU847650	A	A1	Brazil	4	162-932	771
KU847651	A	A1	Brazil	4	132-932	801
KU847652	A	A1	Brazil	4	132-932	801
KU847654	A	A1	Brazil	4	132-932	801
KU847655	A	A1	Brazil	4	156-932	777
KU847656	A	A1	Brazil	4	132-929	798
KU847657	A	A1	Brazil	4	132-932	801
KU847658	A	A1	Brazil	4	156-932	777
KU847659	A	A1	Brazil	4	132-932	801
KU847660	A	A1	Brazil	4	132-929	798
KU847661	A	A1	Brazil	4	132-932	801
KU847662	A	A1	Brazil	4	132-929	798
KU847663	A	A2	Brazil	4	132-932	801
KU847664	A	A1	Brazil	4	144-932	789
KU847665	A	A1	Brazil	4	162-932	771
KU847666	A	A1	Brazil	4	132-932	801
KU847667	A	A1	Brazil	4	132-932	801
KU847668	A	A2	Brazil	4	132-932	801
KU847669	A	A2	Brazil	4	132-935	804
KU847670	A	A1	Brazil	4	132-929	798
KU847671	A	A1	Brazil	4	132-932	801
KU847672	A	A1	Brazil	4	132-932	801
KU847673	A	A1	Brazil	4	132-932	801
KU847675	A	A1	Brazil	4	132-932	801
KU847763	A	A1	Brazil	4	132-935	804
KU847764	A	A1	Brazil	4	132-932	801
KU847765	A	A1	Brazil	4	132-929	798
KU847766	A	A1	Brazil	4	132-929	798
KU847767	A	A1	Brazil	4	132-932	801
KU847768	A	A1	Brazil	4	132-932	801
KU847769	A	A1	Brazil	4	132-932	801
EF547828	A	A2	Brazil	5	157-837	681
EF547829	A	A1	Brazil	5	157-837	681
EF547830	A	A1	Brazil	5	157-837	681

EF547831	A	A1	Brazil	5	157-837	681
EF547832	A	A1	Brazil	5	157-837	681
EF547833	A	A1	Brazil	5	157-837	681
EF547834	A	A1	Brazil	5	157-837	681
EF547835	A	A1	Brazil	5	157-837	681
EF547836	A	A1	Brazil	5	157-837	681
EF547837	A	A1	Brazil	5	157-837	681
EF547838	A	A1	Brazil	5	157-837	681
EF547839	A	A1	Brazil	5	157-837	681
EF547840	A	A1	Brazil	5	157-837	681
EF547841	A	A1	Brazil	5	157-837	681
EF547842	A	A1	Brazil	5	157-837	681
EF547843	A	A1	Brazil	5	157-837	681
EF547844	A	A1	Brazil	5	157-837	681
EF547845	A	A1	Brazil	5	157-837	681
EF547846	A	A1	Brazil	5	157-837	681
EF547847	A	A1	Brazil	5	157-837	681
EF547848	A	A1	Brazil	5	157-837	681
EF547849	A	A1	Brazil	5	157-837	681
EF547850	A	A1	Brazil	5	157-837	681
EF547851	A	A1	Brazil	5	157-837	681
EF547852	A	A1	Brazil	5	157-837	681
EF547853	A	A1	Brazil	5	157-837	681
EF547854	A	A1	Brazil	5	157-837	681
EF547855	A	A1	Brazil	5	157-837	681
EF547856	A	A1	Brazil	5	157-837	681
FJ665815	A	A1	Brazil	5	157-834	678
FJ665816	A	A1	Brazil	5	157-834	678
HQ840709	A	A1	Brazil	5	157-837	681
KT630607	A	A1	Brazil	5	157-837	681
KT630608	A	A1	Brazil	5	157-837	681
KT630609	A	A1	Brazil	5	157-837	681
KT630611	A	A1	Brazil	5	157-837	681
KT630612	A	A1	Brazil	5	157-837	681
KT630615	A	A1	Brazil	5	157-837	681
KT630616	A	A1	Brazil	5	157-837	681
KT630617	A	A1	Brazil	5	157-837	681
KT630621	A	A1	Brazil	5	157-837	681
KT630624	A	A1	Brazil	5	157-837	681
KT630625	A	A2	Brazil	5	157-837	681
KT630626	A	A2	Brazil	5	157-837	681

KT630627	A	A1	Brazil	5	157-837	681
KT630628	A	A1	Brazil	5	157-837	681
KT630630	A	A1	Brazil	5	157-837	681
KT630631	A	A1	Brazil	5	157-837	681
KT630632	A	A1	Brazil	5	157-837	681
KT630635	A	A1	Brazil	5	157-837	681
KT630636	A	A1	Brazil	5	157-837	681
KT630637	A	A1	Brazil	5	157-837	681
KT630638	A	A1	Brazil	5	157-837	681
KT630639	A	A1	Brazil	5	157-837	681
KT630640	A	A2	Brazil	5	157-837	681
KT630641	A	A1	Brazil	5	157-837	681
KT630642	A	A1	Brazil	5	157-837	681
KT630643	A	A2	Brazil	5	157-837	681
MN147822	A	A2	Brazil	5	157-837	681
MN197542	A	A2	Brazil	5	157-832	676
FJ010658	A	A1	Brazil	6	310-936	627
FJ010659	A	A1	Brazil	6	310-936	627
FJ010660	A	A1	Brazil	6	310-936	627
FJ010661	A	A1	Brazil	6	310-936	627
FJ174794	A	A1	Brazil	6	157-815	659
FJ174795	A	A1	Brazil	6	157-815	659
FJ174796	A	A1	Brazil	6	157-815	659
FJ174797	A	A1	Brazil	6	157-815	659
FJ174798	A	A1	Brazil	6	157-815	659
FJ174799	A	A1	Brazil	6	157-815	659
FJ174800	A	A1	Brazil	6	157-815	659
FJ865506	A	A1	Brazil	6	322-950	629
FJ865507	A	A1	Brazil	6	322-950	629
FJ865508	A	A1	Brazil	6	322-950	629
FJ865509	A	A1	Brazil	6	322-950	629
FJ865510	A	A1	Brazil	6	322-950	629
FJ865511	A	A1	Brazil	6	322-950	629
FJ865512	A	A1	Brazil	6	322-950	629
FJ865513	A	A1	Brazil	6	322-950	629
FJ865514	A	A1	Brazil	6	322-950	629
FJ865515	A	A1	Brazil	6	322-950	629
FJ865516	A	A1	Brazil	6	322-950	629
FJ865517	A	A2	Brazil	6	322-950	629
FJ865518	A	A2	Brazil	6	322-950	629
FJ865519	A	A2	Brazil	6	322-950	629

HM101113	A	A1	Brazil	6	3-948	946
HM136966	A	A1	Brazil	6	299-982	984
HM136967	A	A1	Brazil	6	276-943	668
HM136972	A	A1	Brazil	6	275-982	708
HM136973	A	A1	Brazil	6	276-982	707
HM136974	A	A1	Brazil	6	275-977	703
HM136975	A	A1	Brazil	6	275-982	708
HM136976	A	A1	Brazil	6	275-982	708
HM136977	A	A1	Brazil	6	275-982	708
HM136979	A	A1	Brazil	6	275-979	705
KU847476	A	A1	Brazil	6	183-932	750
KU847537	A	A1	Brazil	6	240-1163	924
KU847558	A	A1	Brazil	6	228-1163	936
KU847581	A	A1	Brazil	6	135-812	678
KU847603	A	A1	Brazil	6	240-1163	924
KU847647	A	A1	Brazil	6	153-836	684
KU847674	A	A1	Brazil	6	393-836	444
KX264599	A	A1	Brazil	6	274-976	700
KX264600	A	A1	Brazil	6	275-1181	702
KX264602	A	A1	Brazil	6	270-973	703
KX264608	A	A1	Brazil	6	269-982	714
KX264609	A	A1	Brazil	6	275-973	907
KX264613	A	A1	Brazil	6	272-982	711
KX264615	A	A1	Brazil	6	275-976	702
KX264616	A	A1	Brazil	6	275-973	699
KX264617	A	A1	Brazil	6	274-973	700
KX264618	A	A1	Brazil	6	275-973	699
KX264619	A	A1	Brazil	6	274-980	707
KX264630	A	A1	Brazil	6	277-973	697
KX264634	A	A1	Brazil	6	272-976	705
KX264636	A	A1	Brazil	6	277-973	697
KX264637	A	A1	Brazil	6	270-978	709
KX264639	A	A1	Brazil	6	272-973	702
MK333248	A	A1	Brazil	6	133-781	649
MK333249	A	A1	Brazil	6	137-844	708
MK333250	A	A1	Brazil	6	159-848	690
MK360178	A	A1	Brazil	6	157-837	681
MN758712	A	A1	Brazil	6	273-1274	1002
MN845912	A	A1	Brazil	6	70-779	709
MN845920	A	A1	Brazil	6	27-926	856
MN845927	A	A1	Brazil	6	140-780	641

HM101114	A	A1	Brazil	7	71-1243	1173
HM101115	A	A1	Brazil	7	5-1279	1277
HM101116	A	A1	Brazil	7	5-1279	1277
HM101117	A	A1	Brazil	7	5-1243	1239
HM101118	A	A1	Brazil	7	5-1279	1277
HM101119	A	A1	Brazil	7	5-1274	1272
HM101120	A	A1	Brazil	7	5-1278	1276
HM101121	A	A1	Brazil	7	5-1278	1276
HM101122	A	A1	Brazil	7	5-1276	1272
HM101123	A	A1	Brazil	7	87-1279	1193
HM101124	A	A1	Brazil	7	5-1279	1277
HM101129	A	A1	Brazil	7	5-1278	1276
HM772994	A	A1	Brazil	7	5-1280	1278
HM772995	A	A1	Brazil	7	5-1280	1278
HM772996	A	A1	Brazil	7	5-1280	1278
HM772997	A	A1	Brazil	7	5-1280	1278
JF298903	A	A1	Brazil	7	6-1269	1269
JN983831	A	A1	Brazil	7	6-1274	1269
JN983832	A	A1	Brazil	7	6-1274	1269
JN983833	A	A1	Brazil	7	54-1274	1221
JN983834	A	A1	Brazil	7	6-1274	1269
JN983835	A	A1	Brazil	7	6-1274	1269
JN983836	A	A1	Brazil	7	6-1274	1269
JN983837	A	A1	Brazil	7	6-1274	1269
JN983838	A	A1	Brazil	7	6-1274	1269
JN983839	A	A1	Brazil	7	6-1274	1269
JN983840	A	A1	Brazil	7	6-1274	1269
JN983841	A	A1	Brazil	7	6-1274	1269
JN983842	A	A1	Brazil	7	60-1274	1215
JN983843	A	A1	Brazil	7	6-1274	1269
JN983844	A	A1	Brazil	7	6-1274	1269
JN983845	A	A1	Brazil	7	6-1274	1269
JN983846	A	A1	Brazil	7	6-1274	1269
JN983847	A	A1	Brazil	7	6-1274	1269
JN983848	A	A1	Brazil	7	6-1274	1269
JN983849	A	A1	Brazil	7	6-1274	1269
JN983850	A	A1	Brazil	7	6-1274	1269
JN983851	A	A1	Brazil	7	6-1274	1269
JN983852	A	A1	Brazil	7	60-1274	1215
JN983853	A	A1	Brazil	7	47-1274	1220
JN983854	A	A1	Brazil	7	6-1274	1269

JN983855	A	A1	Brazil	7	6-1274	1269
JN983856	A	A1	Brazil	7	24-1242	1202
JN983857	A	A1	Brazil	7	6-1274	1269
JN983858	A	A1	Brazil	7	6-1274	1269
JN983859	A	A1	Brazil	7	6-1274	1269
JN983860	A	A1	Brazil	7	6-1274	1269
JN983861	A	A1	Brazil	7	6-1274	1269
JN983862	A	A1	Brazil	7	6-1274	1269
JN983863	A	A1	Brazil	7	6-1274	1269
JN983864	A	A1	Brazil	7	6-1276	1269
JN983865	A	A1	Brazil	7	26-1274	1249
JN983866	A	A1	Brazil	7	60-1274	1215
JN983867	A	A1	Brazil	7	57-1274	1218
JN983868	A	A1	Brazil	7	6-1274	1269
JN983869	A	A1	Brazil	7	6-1274	1269
JN983870	A	A1	Brazil	7	6-1274	1269
JN983871	A	A1	Brazil	7	6-1274	1269
JN983872	A	A1	Brazil	7	6-1274	1269
JN983873	A	A1	Brazil	7	63-1274	1212
JN983874	A	A1	Brazil	7	6-1276	1269
JN983875	A	A1	Brazil	7	6-1274	1269
JN983876	A	A1	Brazil	7	6-1276	1269
JN983877	A	A1	Brazil	7	57-1274	1218
JN983878	A	A1	Brazil	7	6-1274	1269
JN983879	A	A1	Brazil	7	51-1274	1224
JN983880	A	A1	Brazil	7	6-1274	1269
JN983881	A	A1	Brazil	7	6-1274	1269
JN983882	A	A1	Brazil	7	6-1274	1269
JN983883	A	A1	Brazil	7	6-1274	1269
JN983884	A	A1	Brazil	7	6-1274	1269
JN983885	A	A1	Brazil	7	6-1274	1269
JN983886	A	A1	Brazil	7	35-1274	1240
JN983887	A	A1	Brazil	7	6-1274	1269
JN983888	A	A1	Brazil	7	6-1274	1269
JN983889	A	A1	Brazil	7	6-1274	1269
JN983890	A	A1	Brazil	7	6-1274	1269
JN983891	A	A1	Brazil	7	6-1274	1269
JN983892	A	A1	Brazil	7	6-1274	1269
JN983893	A	A1	Brazil	7	6-1274	1269
JN983894	A	A1	Brazil	7	6-1274	1269
JN983895	A	A1	Brazil	7	57-1274	1218

JN983896	A	A1	Brazil	7	48-1274	1227
JN983897	A	A1	Brazil	7	6-1274	1269
JN983898	A	A1	Brazil	7	6-1274	1269
JN983899	A	A1	Brazil	7	6-1274	1269
JN983900	A	A1	Brazil	7	6-1274	1269
JN983901	A	A1	Brazil	7	6-1274	1257
JN983902	A	A1	Brazil	7	6-1274	1269
JN983903	A	A1	Brazil	7	6-1274	1269
JN983904	A	A1	Brazil	7	57-1274	1218
JN983905	A	A1	Brazil	7	59-1274	1216
JN983907	A	A1	Brazil	7	6-1274	1269
JN983908	A	A1	Brazil	7	6-1274	1269
JN983909	A	A1	Brazil	7	44-1274	1231
KX264594	A	A1	Brazil	7	22-1246	1225
KX264595	A	A1	Brazil	7	39-1219	1181
KX264596	A	A1	Brazil	7	39-1239	1201
KX264597	A	A1	Brazil	7	22-1239	1218
KX264598	A	A1	Brazil	7	35-1247	1213
KX264601	A	A1	Brazil	7	22-1234	1213
KX264603	A	A1	Brazil	7	27-1238	1212
KX264604	A	A1	Brazil	7	39-1234	1196
KX264605	A	A1	Brazil	7	23-1246	1224
KX264606	A	A1	Brazil	7	22-1239	1218
KX264607	A	A1	Brazil	7	39-1239	1201
KX264610	A	A1	Brazil	7	23-1240	1217
KX264611	A	A1	Brazil	7	22-1234	1213
KX264612	A	A1	Brazil	7	39-1246	1208
KX264614	A	A1	Brazil	7	37-1239	1203
KX264620	A	A1	Brazil	7	35-1237	1203
KX264621	A	A1	Brazil	7	35-1239	1205
KX264622	A	A1	Brazil	7	22-1239	1218
KX264623	A	A1	Brazil	7	40-1246	1207
KX264624	A	A1	Brazil	7	35-1239	1205
KX264625	A	A1	Brazil	7	55-1212	1158
KX264626	A	A1	Brazil	7	5-1292	1290
KX264627	A	A1	Brazil	7	22-1245	1224
KX264628	A	A1	Brazil	7	40-1249	1210
KX264629	A	A1	Brazil	7	52-1236	1185
KX264631	A	A1	Brazil	7	45-1238	1194
KX264632	A	A1	Brazil	7	39-1237	1199
KX264633	A	A1	Brazil	7	35-1245	1211

KX264635	A	A1	Brazil	7	39-1245	1207
KX264638	A	A1	Brazil	7	22-1247	1226
KX264640	A	A1	Brazil	7	23-1237	1215
KX264641	A	A1	Brazil	7	22-1240	1219
KX264642	A	A1	Brazil	7	23-1239	1217
KX264643	A	A1	Brazil	7	12-1239	1228
KX264644	A	A1	Brazil	7	24-1237	1214
KX264645	A	A1	Brazil	7	23-1237	1215
KX264646	A	A1	Brazil	7	39-1239	1201
KX264647	A	A1	Brazil	7	35-1235	1201
KX264648	A	A1	Brazil	7	31-1250	1220
KX264649	A	A1	Brazil	7	21-1239	1219
KX264650	A	A1	Brazil	7	34-1247	1214
KX264651	A	A1	Brazil	7	38-1235	1198
KX264652	A	A1	Brazil	7	45-1239	1195
KX264653	A	A1	Brazil	7	24-1239	1216
KX264654	A	A1	Brazil	7	23-1236	1214
KX264655	A	A1	Brazil	7	38-1237	1200
KX264656	A	A1	Brazil	7	24-1245	1222
KX264657	A	A1	Brazil	7	23-1241	1219
KX264658	A	A1	Brazil	7	12-1277	1266
KX264659	A	A1	Brazil	7	22-1235	1214
KX302095	A	A1	Brazil	7	5-1273	1271
KX302105	A	A1	Brazil	7	57-1273	1217
KX302106	A	A1	Brazil	7	5-1275	1271
KX302113	A	A1	Brazil	7	5-1273	1270
KX302129	A	A1	Brazil	7	53-1222	1170
KX302130	A	A1	Brazil	7	5-1273	1271
KX302133	A	A1	Brazil	7	5-1273	1271
KX302134	A	A1	Brazil	7	59-1273	1215
KY809880	A	A1	Brazil	7	5-1274	1270
KY809881	A	A1	Brazil	7	5-1274	1270
KY809882	A	A1	Brazil	7	5-1274	1270
KY809883	A	A1	Brazil	7	5-1274	1270
KY809884	A	A2	Brazil	7	5-1274	1270
KY809885	A	A1	Brazil	7	5-1276	1270
KY809886	A	A1	Brazil	7	5-1276	1270
KY809887	A	A1	Brazil	7	5-1274	1270
KY809888	A	A1	Brazil	7	5-1276	1270
KY809889	A	A1	Brazil	7	5-1274	1270
KY809890	A	A1	Brazil	7	5-1274	1270

KY809891	A	A2	Brazil	7	5-1274	1270
KY809892	A	A2	Brazil	7	5-1274	1270
KY809893	A	A1	Brazil	7	56-1274	1219
KY809894	A	A1	Brazil	7	5-1274	1270
KY809895	A	A2	Brazil	7	5-1274	1270
KY809896	A	A1	Brazil	7	5-1274	1270
KY809897	A	A2	Brazil	7	5-1274	1270
KY809898	A	A1	Brazil	7	5-1274	1270
KY809899	A	A1	Brazil	7	5-1274	1270
KY809900	A	A1	Brazil	7	5-1274	1270
KY809901	A	A1	Brazil	7	56-1274	1219
KY809902	A	A2	Brazil	7	5-1274	1270
KY809903	A	A1	Brazil	7	58-1274	1217
KY809904	A	A1	Brazil	7	22-1274	1226
KY809905	A	A1	Brazil	7	5-1274	1270
KY809906	A	A1	Brazil	7	5-1274	1270
KY809907	A	A1	Brazil	7	5-1274	1270
KY809908	A	A1	Brazil	7	52-1274	1223
KY809909	A	A1	Brazil	7	5-1276	1270
KY809910	A	A1	Brazil	7	5-1274	1270
KY809911	A	A2	Brazil	7	5-1274	1270
KY809912	A	A1	Brazil	7	5-1274	1270
KY809913	A	A1	Brazil	7	58-1274	1217
KY809914	A	A1	Brazil	7	5-1274	1270
KY809915	A	A1	Brazil	7	5-1276	1270
KY809916	A	A1	Brazil	7	5-1274	1270
KY809917	A	A1	Brazil	7	5-1274	1270
KY809918	A	A1	Brazil	7	5-1238	1234
KY809919	A	A1	Brazil	7	5-1276	1270
KY809920	A	A1	Brazil	7	5-1274	1270
KY809921	A	A1	Brazil	7	58-1274	1217
KY809922	A	A1	Brazil	7	5-1274	1270
KY809923	A	A2	Brazil	7	5-1274	1270
KY809924	A	A1	Brazil	7	5-1274	1270
KY809925	A	A2	Brazil	7	5-1274	1267
KY809926	A	A1	Brazil	7	5-1274	1270
KY809927	A	A1	Brazil	7	5-1276	1270
KY809928	A	A1	Brazil	7	5-1274	1270
KY809929	A	A2	Brazil	7	5-1275	1270
KY809930	A	A1	Brazil	7	5-1274	1270
KY809931	A	A1	Brazil	7	5-1276	1270

KY809932	A	A1	Brazil	7	5-1274	1270
KY809933	A	A1	Brazil	7	5-1274	1270
KY809934	A	A1	Brazil	7	5-1276	1270
KY809935	A	A1	Brazil	7	5-1274	1270
KY809936	A	A1	Brazil	7	5-1274	1270
KY809937	A	A1	Brazil	7	5-1274	1270
MN758680	A	A1	Brazil	7	60-1274	1215
MN758681	A	A2	Brazil	7	33-1277	1245
MN758682	A	A1	Brazil	7	57-1274	1218
MN758683	A	A2	Brazil	7	24-1286	1263
MN758684	A	A1	Brazil	7	5-1277	1284
MN758685	A	A1	Brazil	7	36-1242	1207
MN758687	A	A1	Brazil	7	90-1277	1188
MN758689	A	A1	Brazil	7	72-1277	1206
MN758690	A	A1	Brazil	7	33-1286	1254
MN758692	A	A1	Brazil	7	57-1277	1221
MN758694	A	A1	Brazil	7	190-1286	1098
MN758695	A	A1	Brazil	7	84-1283	1200
MN758696	A	A2	Brazil	7	42-1277	1236
MN758697	A	A1	Brazil	7	45-1277	1233
MN758699	A	A1	Brazil	7	5-1277	1284
MN758700	A	A1	Brazil	7	84-1274	1191
MN758701	A	A1	Brazil	7	5-1277	1278
MN758702	A	A1	Brazil	7	63-1286	1224
MN758703	A	A1	Brazil	7	57-1277	1221
MN758705	A	A1	Brazil	7	30-1259	1230
MN758709	A	A1	Brazil	7	81-1274	1194
MN758710	A	A4	Brazil	7	66-1286	1221
MN758711	A	A1	Brazil	7	45-1273	1229
MN758714	A	A1	Brazil	7	51-1273	1223
MN758716	A	A1	Brazil	7	5-1279	1278
MN758717	A	A1	Brazil	7	42-1277	1236
MN758718	A	A1	Brazil	7	87-1238	1152
MN758719	A	A1	Brazil	7	63-1286	1224
MN758721	A	A1	Brazil	7	36-1283	1248
MN758724	A	A1	Brazil	7	30-1288	1257
KU847474	A	A1	Brazil	8	132-1163	1032
KU847482	A	A1	Brazil	8	132-1163	1032
KU847497	A	A1	Brazil	8	132-1163	1032
KU847499	A	A1	Brazil	8	132-1163	1032
KU847500	A	A1	Brazil	8	132-1163	1032

KU847511	A	A1	Brazil	8	132-1163	1032
KU847514	A	A1	Brazil	8	132-1163	1032
KU847517	A	A1	Brazil	8	132-1163	1032
KU847582	A	A1	Brazil	8	132-1163	1032
KU847585	A	A1	Brazil	8	132-1163	1032
KU847588	A	A1	Brazil	8	132-1163	1032
KU847591	A	A1	Brazil	8	132-1163	1032
KU847594	A	A1	Brazil	8	132-1163	1032
KU847595	A	A1	Brazil	8	132-1163	1032
KU847604	A	A1	Brazil	8	132-1163	1032
KU847610	A	A1	Brazil	8	132-1163	1032
KU847614	A	A1	Brazil	8	132-1163	1032
KU847617	A	A1	Brazil	8	132-1163	1032
KU847622	A	A1	Brazil	8	132-1163	1032
KU847625	A	A1	Brazil	8	132-1163	1032
KU847631	A	A1	Brazil	8	132-1163	1032
KU847632	A	A1	Brazil	8	138-1163	1026
KU847634	A	A1	Brazil	8	132-1163	1032
KU847753	A	A2	Brazil	8	132-1163	1032
KU847761	A	A1	Brazil	8	132-1163	1032
KU847762	A	A1	Brazil	8	132-1163	1032
MK517516	A	A1	Brazil	8	1-3182	1271
MK517517	A	A1	Brazil	8	1-3182	1271
MK517519	A	A1	Brazil	8	1-3182	1271
FJ665811	A	A1	Brazil	9	1-3182	1200
FJ665812	A	A1	Brazil	9	1-3182	1200
FJ665813	A	A1	Brazil	9	1-3182	1200
FJ665814	A	A1	Brazil	9	1-3182	1200
HQ646098	A	A2	Brazil	9	1-3182	1178
JF815647	A	A1	Brazil	9	1-3127	1389
KX686608	A	A1	Brazil	9	1-3182	1203
MN845892	A	A1	Brazil	9	1-3182	1118
MN845893	A	A1	Brazil	9	1-3182	1114
MN845894	A	A2	Brazil	9	1-3182	1124
MN845896	A	A1	Brazil	9	1-3182	1112
MN845897	A	A2	Brazil	9	1-3182	1112
MN845898	A	A1	Brazil	9	1-3182	1122
MN845899	A	A2	Brazil	9	1-3182	1126
MN845900	A	A1	Brazil	9	1-3182	1115
MN845901	A	A1	Brazil	9	1-3182	1122
MN845902	A	A1	Brazil	9	1-3182	1118

MN845903	A	A1	Brazil	9	1-3182	1111
MN845904	A	A1	Brazil	9	1-3182	1114
MN845906	A	A1	Brazil	9	1-3182	1122
MN845907	A	A1	Brazil	9	1-3182	1113
MN845908	A	A1	Brazil	9	1-3182	1122
MN845913	A	A2	Brazil	9	1-3182	1113
MN845915	A	A1	Brazil	9	1-3182	1109
MN845916	A	A1	Brazil	9	1-3182	1119
MN845917	A	A2	Brazil	9	1-3182	1124
MN845919	A	A1	Brazil	9	1-3182	1120
MN845921	A	A1	Brazil	9	1-3182	1107
MN845922	A	A1	Brazil	9	1-3182	1118
MN845923	A	A1	Brazil	9	1-3182	1104
MN845924	A	A2	Brazil	9	1-3182	1114
MN845925	A	A1	Brazil	9	1-3182	1089
MN845926	A	A2	Brazil	9	1-3182	1118
MN845928	A	A2	Brazil	9	1-3182	1121
MK336893	A	A2	Brazil	10	1-3182	1019
HQ622095	C	C2	Brazil	11	Complete Genome	3215
KU847720	C	C1	Brazil	12	132-1163	1032
MN758706	C	C2	Brazil	12	1-3182	1197
FJ865520	C	C2	Brazil	13	322-950	629
FJ865521	C	C2	Brazil	13	322-950	629
FJ865522	C	C2	Brazil	13	322-950	629
KJ470884	D	D4	Brazil	14	Complete Genome	3182
KJ470885	D	D4	Brazil	14	Complete Genome	3179
KJ470886	D	D4	Brazil	14	Complete Genome	3182
KJ470887	D	D4	Brazil	14	Complete Genome	3182
KJ470888	D	D4	Brazil	14	Complete Genome	3182
KJ470889	D	D4	Brazil	14	Complete Genome	3182
KJ470890	D	D4	Brazil	14	Complete Genome	3182
KJ470891	D	D4	Brazil	14	Complete Genome	3182
KJ470892	D	D4	Brazil	14	Complete Genome	3167
KJ470893	D	D4	Brazil	14	Complete Genome	3182
KJ470894	D	D4	Brazil	14	Complete Genome	3182
KJ470895	D	D4	Brazil	14	Complete Genome	3182
KJ470896	D	D4	Brazil	14	Complete Genome	3182
KJ470897	D	D4	Brazil	14	Complete Genome	3182
KJ470898	D	D4	Brazil	14	Complete Genome	3182
KP090177	D	D3	Brazil	14	Complete Genome	3182
KP090178	D	D3	Brazil	14	Complete Genome	3182

KP090179	D	D3	Brazil	14	Complete Genome	3182
KP090180	D	D3	Brazil	14	Complete Genome	3182
KP090181	D	D3	Brazil	14	Complete Genome	3182
KX260231	D	D2	Brazil	14	Complete Genome	3062
MH724214	D	D3	Brazil	14	Complete Genome	3182
MH724215	D	D3	Brazil	14	Complete Genome	3182
MH724216	D	D2	Brazil	14	Complete Genome	3182
MH724217	D	D2	Brazil	14	Complete Genome	3182
MH724218	D	D3	Brazil	14	Complete Genome	3182
MH724219	D	D3	Brazil	14	Complete Genome	3182
MH724220	D	D3	Brazil	14	Complete Genome	3182
MH724221	D	D3	Brazil	14	Complete Genome	3182
MH724222	D	D3	Brazil	14	Complete Genome	3182
MH724223	D	D2	Brazil	14	Complete Genome	3182
MH724224	D	D3	Brazil	14	Complete Genome	3182
MH724225	D	D3	Brazil	14	Complete Genome	3182
MH724226	D	D3	Brazil	14	Complete Genome	3182
MH724227	D	D3	Brazil	14	Complete Genome	3182
MH724228	D	D3	Brazil	14	Complete Genome	3182
MH724229	D	D3	Brazil	14	Complete Genome	3182
MH724230	D	D3	Brazil	14	Complete Genome	3182
MH724231	D	D2	Brazil	14	Complete Genome	3182
MH724232	D	D3	Brazil	14	Complete Genome	3182
MH724233	D	D3	Brazil	14	Complete Genome	3182
MH724234	D	D3	Brazil	14	Complete Genome	3182
MH724235	D	D3	Brazil	14	Complete Genome	3182
MH724236	D	D2	Brazil	14	Complete Genome	3182
MH724237	D	D3	Brazil	14	Complete Genome	3182
MH724238	D	D2	Brazil	14	Complete Genome	3182
MH724239	D	D3	Brazil	14	Complete Genome	3182
MH724240	D	D3	Brazil	14	Complete Genome	3182
MH724241	D	D2	Brazil	14	Complete Genome	3182
MH724242	D	D3	Brazil	14	Complete Genome	3182
MH724243	D	D3	Brazil	14	Complete Genome	3182
MH724244	D	D2	Brazil	14	Complete Genome	3182
MH724245	D	D3	Brazil	14	Complete Genome	3182
MH724246	D	D2	Brazil	14	Complete Genome	3182
MH724247	D	D2	Brazil	14	Complete Genome	3182
MH724248	D	D3	Brazil	14	Complete Genome	3182
MH724249	D	D3	Brazil	14	Complete Genome	3182
MH724250	D	D3	Brazil	14	Complete Genome	3182

MH724251	D	D4	Brazil	14	Complete Genome	3182
MH724252	D	D1	Brazil	14	Complete Genome	3182
MN310705	D	D1	Brazil	14	Complete Genome	3182
MN310706	D	D2	Brazil	14	Complete Genome	3182
MN310707	D	D2	Brazil	14	Complete Genome	3182
MN310708	D	D3	Brazil	14	Complete Genome	3182
MN310709	D	D3	Brazil	14	Complete Genome	3182
MN310710	D	D3	Brazil	14	Complete Genome	3182
MN310711	D	D3	Brazil	14	Complete Genome	3182
MN310712	D	D3	Brazil	14	Complete Genome	3182
MN310713	D	D3	Brazil	14	Complete Genome	3182
MN310714	D	D3	Brazil	14	Complete Genome	3182
KY355739	D	D2	Brazil	15	1-3182	1050
KY355740	D	D2	Brazil	15	1-3182	1050
KY355741	D	D2	Brazil	15	1-3182	1050
KY355742	D	D2	Brazil	15	1-3182	1050
KY355743	D	D2	Brazil	15	1-3182	1050
KY355744	D	D2	Brazil	15	1-3182	1050
KY355745	D	D2	Brazil	15	1-3182	1050
KY355746	D	D2	Brazil	15	1-3182	1050
KY355747	D	D2	Brazil	15	1-3182	1050
KY355748	D	D2	Brazil	15	1-3182	1050
KY355749	D	D2	Brazil	15	1-3182	1050
KY355750	D	D2	Brazil	15	1-3182	1050
KY355751	D	D2	Brazil	15	1-3182	1050
KY355752	D	D2	Brazil	15	1-3182	1050
KY355753	D	D2	Brazil	15	1-3182	1050
KY355754	D	D2	Brazil	15	1-3182	1137
KY355755	D	D2	Brazil	15	1-3182	1137
KY355756	D	D2	Brazil	15	1-3182	1137
KY355757	D	D2	Brazil	15	1-3182	1050
KY355758	D	D2	Brazil	15	1-3182	1050
KY355759	D	D2	Brazil	15	1-3182	1050
KY355760	D	D2	Brazil	15	1-3182	1050
KY355761	D	D2	Brazil	15	1-3182	1050
KY355762	D	D2	Brazil	15	1-3182	1050
KY355763	D	D2	Brazil	15	1-3182	1050
KY355764	D	D2	Brazil	15	1-3182	1050
KY355765	D	D2	Brazil	15	1-3182	1050
KY355766	D	D2	Brazil	15	1-3182	1050
KY355767	D	D2	Brazil	15	1-3182	1050

KY355768	D	D2	Brazil	15	1-3182	1050
JF815606	D	D6	Brazil	16	1-3182	1431
JF815607	D	D3	Brazil	16	1-3182	1425
JF815608	D	D3	Brazil	16	1-3182	1437
JF815609	D	D3	Brazil	16	1-3182	1416
JF815610	D	D3	Brazil	16	1-3182	1437
JF815612	D	D3	Brazil	16	1-3182	1431
JF815613	D	D2	Brazil	16	1-3182	1428
JF815615	D	D3	Brazil	16	1-3182	1446
JF815616	D	D3	Brazil	16	1-3182	1496
JF815617	D	D3	Brazil	16	1-3182	1416
JF815618	D	D2	Brazil	16	1-3182	1413
JF815620	D	D3	Brazil	16	1-3182	1368
JF815621	D	D6	Brazil	16	1-3182	1414
JF815624	D	D3	Brazil	16	1-3182	1437
JF815625	D	D3	Brazil	16	1-3182	1437
JF815626	D	D3	Brazil	16	1-3182	1425
JF815627	D	D3	Brazil	16	1-3182	1437
JF815628	D	D6	Brazil	16	1-3182	1425
JF815630	D	D3	Brazil	16	1-3182	1440
JF815631	D	D2	Brazil	16	1-3182	1422
JF815632	D	D3	Brazil	16	1-3182	1434
JF815633	D	D3	Brazil	16	1-3182	1425
JF815634	D	D3	Brazil	16	1-3182	1440
JF815635	D	D3	Brazil	16	1-3182	1437
JF815636	D	D12	Brazil	16	1-3182	1437
JF815637	D	D3	Brazil	16	1-3182	1425
JF815639	D	D2	Brazil	16	1-3182	1320
JF815640	D	D6	Brazil	16	1-3182	1425
JF815642	D	D1	Brazil	16	1-3182	1356
JF815645	D	D3	Brazil	16	1-3182	1437
JF815646	D	D3	Brazil	16	1-3182	1425
JF815648	D	D4	Brazil	16	1-3182	1425
JF815649	D	D6	Brazil	16	1-3182	1437
JF815650	D	D2	Brazil	16	1-3182	1416
JF815651	D	D3	Brazil	16	1-3182	1425
JF815652	D	D2	Brazil	16	1-3182	1353
JF815653	D	D3	Brazil	16	1-3182	1314
JF815654	D	D2	Brazil	16	1-3182	1416
JF815655	D	D3	Brazil	16	1-3182	1425
JF815656	D	D3	Brazil	16	1-3182	1425

JF815657	D	D2	Brazil	16	1-3182	1425
JF815658	D	D3	Brazil	16	1-3182	1425
JF815659	D	D3	Brazil	16	1-3182	1425
JF815660	D	D3	Brazil	16	1-3182	1383
JF815661	D	D6	Brazil	16	1-3182	1374
JF815662	D	D3	Brazil	16	1-3182	1428
JF815663	D	D3	Brazil	16	1-3182	1425
JF815664	D	D9	Brazil	16	1-3182	1367
JF815667	D	D2	Brazil	16	1-3182	1367
JF815669	D	D2	Brazil	16	1-3182	1367
JF815671	D	D2	Brazil	16	1-3182	1367
JF815675	D	D9	Brazil	16	1-3182	1356
JF815677	D	D2	Brazil	16	1-3182	1367
HM101101	D	D3	Brazil	17	3-1280	1278
HM101104	D	D4	Brazil	17	3-1280	1278
HM101105	D	D4	Brazil	17	6-1267	1262
HM101106	D	D4	Brazil	17	3-1272	1270
HM101107	D	D4	Brazil	17	3-1252	1250
HM101108	D	D3	Brazil	17	3-1279	1277
HM101109	D	D3	Brazil	17	3-1279	1277
HM101110	D	D3	Brazil	17	3-1272	1270
HM101111	D	D3	Brazil	17	27-1276	1250
HM101112	D	D3	Brazil	17	3-1279	1277
HM101126	D	D2	Brazil	17	3-1279	1277
HM101127	D	D3	Brazil	17	3-1279	1277
HM101128	D	D3	Brazil	17	11-1280	1270
JF298901	D	D4	Brazil	17	6-1274	1269
JF298902	D	D4	Brazil	17	6-1274	1248
JN983910	D	D4	Brazil	17	6-1274	1269
JN983911	D	D4	Brazil	17	6-1274	1269
JN983912	D	D4	Brazil	17	6-1274	1248
JN983913	D	D4	Brazil	17	6-1274	1269
JN983914	D	D4	Brazil	17	6-1274	1269
JN983915	D	D4	Brazil	17	6-1274	1269
JN983916	D	D4	Brazil	17	6-1274	1269
JN983917	D	D4	Brazil	17	6-1274	1269
JN983918	D	D4	Brazil	17	56-1274	1219
JN983919	D	D4	Brazil	17	6-1274	1269
JN983920	D	D4	Brazil	17	6-1274	1269
JN983921	D	D4	Brazil	17	6-1274	1269
JN983922	D	D4	Brazil	17	6-1274	1269

JN983923	D	D4	Brazil	17	6-1274	1269
JN983924	D	D4	Brazil	17	56-1274	1219
JN983925	D	D4	Brazil	17	6-1274	1269
JN983926	D	D4	Brazil	17	6-1274	1269
JN983927	D	D4	Brazil	17	6-1274	1269
JN983928	D	D4	Brazil	17	6-1274	1269
JN983929	D	D4	Brazil	17	6-1274	1269
JN983930	D	D4	Brazil	17	6-1274	1269
JN983931	D	D4	Brazil	17	6-1274	1266
JN983932	D	D4	Brazil	17	6-1274	1269
JN983933	D	D4	Brazil	17	6-1274	1269
JN983934	D	D4	Brazil	17	6-1274	1269
JN983935	D	D4	Brazil	17	6-1274	1269
JN983936	D	D4	Brazil	17	6-1274	1269
JN983937	D	D4	Brazil	17	6-1274	1269
JN983938	D	D2	Brazil	17	58-1274	1217
JN983939	D	D3	Brazil	17	6-1274	1269
JN983940	D	D3	Brazil	17	6-1274	1269
JN983941	D	D3	Brazil	17	6-1274	1269
KX302085	D	D4	Brazil	17	3-1273	1270
KX302086	D	D4	Brazil	17	3-1273	1271
KX302087	D	D4	Brazil	17	3-1273	1271
KX302088	D	D4	Brazil	17	3-1273	1271
KX302089	D	D4	Brazil	17	3-1273	1271
KX302090	D	D4	Brazil	17	51-1273	1223
KX302091	D	D4	Brazil	17	3-1273	1271
KX302092	D	D4	Brazil	17	3-1273	1271
KX302093	D	D4	Brazil	17	8-1273	1266
KX302094	D	D4	Brazil	17	3-1273	1271
KX302096	D	D4	Brazil	17	3-1273	1271
KX302097	D	D4	Brazil	17	3-1273	1271
KX302098	D	D4	Brazil	17	3-1273	1271
KX302099	D	D4	Brazil	17	3-1273	1271
KX302100	D	D4	Brazil	17	3-1273	1271
KX302101	D	D4	Brazil	17	3-1270	1268
KX302102	D	D4	Brazil	17	58-1273	1216
KX302103	D	D4	Brazil	17	3-1273	1271
KX302104	D	D4	Brazil	17	3-1273	1271
KX302107	D	D4	Brazil	17	3-1273	1271
KX302108	D	D4	Brazil	17	40-1273	1234
KX302109	D	D4	Brazil	17	23-1255	1202

KX302110	D	D4	Brazil	17	3-1273	1271
KX302111	D	D4	Brazil	17	3-1273	1271
KX302112	D	D4	Brazil	17	3-1273	1271
KX302114	D	D4	Brazil	17	3-1273	1271
KX302115	D	D4	Brazil	17	3-1273	1271
KX302116	D	D4	Brazil	17	3-1273	1271
KX302117	D	D4	Brazil	17	3-1273	1271
KX302118	D	D4	Brazil	17	3-1273	1271
KX302119	D	D4	Brazil	17	3-1273	1271
KX302120	D	D4	Brazil	17	3-1273	1271
KX302121	D	D4	Brazil	17	3-1273	1271
KX302122	D	D4	Brazil	17	3-1273	1271
KX302123	D	D4	Brazil	17	3-1273	1271
KX302124	D	D4	Brazil	17	3-1273	1271
KX302125	D	D4	Brazil	17	3-1273	1271
KX302126	D	D4	Brazil	17	3-1273	1271
KX302127	D	D4	Brazil	17	3-1273	1271
KX302128	D	D4	Brazil	17	58-1273	1216
KX302131	D	D4	Brazil	17	3-1273	1271
KX302132	D	D4	Brazil	17	3-1273	1271
KY809947	D	D1	Brazil	17	5-1274	1270
KY809948	D	D3	Brazil	17	5-1274	1270
KY809949	D	D3	Brazil	17	5-1274	1270
KY809950	D	D3	Brazil	17	5-1274	1270
KY809951	D	D3	Brazil	17	5-1274	1270
KY809952	D	D3	Brazil	17	5-1274	1270
KY809953	D	D2	Brazil	17	5-1274	1270
KY809954	D	D3	Brazil	17	5-1274	1270
KY809955	D	D3	Brazil	17	5-1274	1270
KY809956	D	D1	Brazil	17	5-1274	1270
KY809957	D	D3	Brazil	17	5-1274	1270
KY809958	D	D3	Brazil	17	5-1274	1270
KY809959	D	D3	Brazil	17	5-1274	1270
KY809960	D	D3	Brazil	17	5-1274	1270
KY809961	D	D3	Brazil	17	5-1274	1270
KY809962	D	D3	Brazil	17	5-1274	1270
KY809963	D	D3	Brazil	17	5-1274	1270
KY809964	D	D3	Brazil	17	5-1274	1270
KY809965	D	D3	Brazil	17	5-1274	1270
KY809966	D	D3	Brazil	17	5-1274	1270
KY809967	D	D3	Brazil	17	5-1274	1270

KY809968	D	D3	Brazil	17	5-1274	1270
KY809969	D	D3	Brazil	17	35-1274	1231
KY809970	D	D3	Brazil	17	5-1274	1270
KY809971	D	D3	Brazil	17	5-1274	1270
KY809972	D	D3	Brazil	17	5-1274	1270
KY809973	D	D3	Brazil	17	5-1274	1270
KY809974	D	D3	Brazil	17	5-1274	1270
KY809975	D	D3	Brazil	17	5-1274	1270
KY809976	D	D3	Brazil	17	5-1274	1270
KY809977	D	D3	Brazil	17	5-1274	1270
KY809978	D	D3	Brazil	17	5-1274	1270
KY809979	D	D3	Brazil	17	5-1274	1270
KY809980	D	D3	Brazil	17	5-1274	1270
KY809981	D	D3	Brazil	17	5-1274	1270
KY809982	D	D3	Brazil	17	5-1274	1270
KY809983	D	D1	Brazil	17	5-1274	1270
KY809984	D	D3	Brazil	17	5-1274	1270
KY809985	D	D3	Brazil	17	5-1274	1270
KY809986	D	D3	Brazil	17	5-1274	1270
KY809987	D	D4	Brazil	17	5-1274	1270
KY809988	D	D2	Brazil	17	5-1274	1270
KY809989	D	D3	Brazil	17	5-1274	1270
KY809990	D	D3	Brazil	17	5-1274	1270
KY809991	D	D3	Brazil	17	5-1274	1270
KY809992	D	D2	Brazil	17	5-1274	1270
KY809993	D	D1	Brazil	17	5-1274	1270
KY809994	D	D2	Brazil	17	5-1274	1270
KY809995	D	D3	Brazil	17	5-1274	1270
KY809996	D	D3	Brazil	17	5-1274	1270
KY809997	D	D1	Brazil	17	5-1274	1270
KY809998	D	D2	Brazil	17	5-1274	1270
KY809999	D	D3	Brazil	17	5-1274	1270
KY810000	D	D3	Brazil	17	5-1274	1270
KY810001	D	D2	Brazil	17	5-1274	1270
KY810002	D	D4	Brazil	17	5-1274	1270
KY810003	D	D3	Brazil	17	5-1274	1270
KY810004	D	D4	Brazil	17	5-1274	1270
KY810005	D	D3	Brazil	17	5-1274	1270
KY810006	D	D3	Brazil	17	5-1274	1270
KY810007	D	D3	Brazil	17	5-1274	1270
KY810008	D	D3	Brazil	17	5-1274	1270

KY810009	D	D3	Brazil	17	5-1274	1270
KY810010	D	D3	Brazil	17	5-1274	1270
KY810011	D	D3	Brazil	17	5-1274	1270
KY810012	D	D3	Brazil	17	5-1274	1270
KY810013	D	D3	Brazil	17	5-1274	1270
KY810014	D	D2	Brazil	17	5-1274	1270
KY810015	D	D3	Brazil	17	5-1274	1270
KY810016	D	D3	Brazil	17	5-1274	1270
KY810017	D	D3	Brazil	17	5-1274	1270
KY810018	D	D3	Brazil	17	5-1274	1270
KY810019	D	D3	Brazil	17	5-1274	1270
KY810020	D	D2	Brazil	17	5-1274	1270
KY810021	D	D3	Brazil	17	5-1274	1270
KY810022	D	D2	Brazil	17	5-1274	1270
KY810023	D	D3	Brazil	17	5-1274	1270
KY810024	D	D1	Brazil	17	5-1274	1270
KY810025	D	D3	Brazil	17	5-1274	1270
KY810026	D	D2	Brazil	17	5-1274	1270
KY810027	D	D1	Brazil	17	5-1274	1270
KY810028	D	D3	Brazil	17	5-1274	1270
KY810029	D	D2	Brazil	17	5-1274	1270
KY810030	D	D1	Brazil	17	5-1274	1270
KY810031	D	D3	Brazil	17	5-1274	1270
KY810032	D	D4	Brazil	17	5-1274	1270
KY810033	D	D2	Brazil	17	5-1274	1270
KY810034	D	D3	Brazil	17	5-1274	1270
KY810035	D	D2	Brazil	17	5-1274	1270
KY810036	D	D3	Brazil	17	5-1274	1270
KY810037	D	D3	Brazil	17	5-1274	1270
KY810038	D	D3	Brazil	17	5-1274	1270
KY810039	D	D3	Brazil	17	5-1274	1270
KY810040	D	D3	Brazil	17	5-1274	1249
KY810041	D	D3	Brazil	17	5-1274	1270
KY810042	D	D3	Brazil	17	5-1274	1270
KY810043	D	D2	Brazil	17	5-1274	1270
KY810044	D	D3	Brazil	17	5-1274	1270
KY810045	D	D3	Brazil	17	5-1274	1270
MN758686	D	D3	Brazil	17	33-1274	1242
MN758688	D	D3	Brazil	17	42-1277	1236
MN758691	D	D3	Brazil	17	48-1283	1236
MN758693	D	D3	Brazil	17	39-1283	1245

MN758698	D	D3	Brazil	17	3-1286	1284
MN758713	D	D3	Brazil	17	3-1280	1278
MN758715	D	D3	Brazil	17	42-1283	1242
MN758720	D	D4	Brazil	17	30-1241	1212
MN758722	D	D3	Brazil	17	6-1277	1272
MN758723	D	D2	Brazil	17	30-1274	1245
HM101103	D	D4	Brazil	18	47-1037	991
JF815611	D	D3	Brazil	18	1-3182	1167
JF815614	D	D3	Brazil	18	1-3182	1077
JF815619	D	D3	Brazil	18	1-3182	1167
JF815668	D	D2	Brazil	18	1-3182	1047
JF815673	D	D3/D6	Brazil	18	1-3182	1140
JF815674	D	D3/D6	Brazil	18	1-3182	1047
JF815676	D	D3/D6	Brazil	18	1-3182	1140
JF815678	D	D3/D6	Brazil	18	1-3182	1131
JF815679	D	D3/D6	Brazil	18	1-3182	1047
JF815680	D	D3/D6	Brazil	18	1-3182	987
JF815681	D	D3/D6	Brazil	18	1-3182	996
JF815682	D	D2	Brazil	18	1-3182	996
JF815683	D	D3/D6	Brazil	18	1-3182	996
JF815684	D	D3/D6	Brazil	18	1-3182	995
JF815685	D	D3/D6	Brazil	18	1-3182	996
JF815686	D	D3/D6	Brazil	18	1-3182	996
JF815687	D	D3/D6	Brazil	18	1-3182	996
JF815688	D	D3/D6	Brazil	18	1-3182	945
JF815689	D	D3/D6	Brazil	18	1-3182	996
JF815690	D	D3/D6	Brazil	18	1-3182	978
JF815691	D	D3/D6	Brazil	18	1-3182	945
JF815692	D	D3/D6	Brazil	18	1-3182	945
JF815693	D	D3/D6	Brazil	18	1-3182	948
KU847472	D	D3	Brazil	18	132-929	798
KU847488	D	D4	Brazil	18	132-1163	1032
KU847491	D	D4	Brazil	18	132-1163	1032
KU847493	D	D4	Brazil	18	132-932	801
KU847509	D	D9	Brazil	18	132-932	801
KU847510	D	D3	Brazil	18	132-1163	1032
KU847513	D	D3	Brazil	18	132-932	801
KU847518	D	D4	Brazil	18	132-932	801
KU847519	D	D9	Brazil	18	132-1163	1032
KU847521	D	D4	Brazil	18	132-1163	1032
KU847532	D	D3	Brazil	18	132-1163	1032

KU847535	D	D4	Brazil	18	132-1163	1032
KU847552	D	D4	Brazil	18	132-1163	1032
KU847561	D	D3	Brazil	18	132-1163	1032
KU847568	D	D9	Brazil	18	132-932	801
KU847571	D	D3	Brazil	18	132-932	801
KU847574	D	D3	Brazil	18	132-929	798
KU847579	D	D3	Brazil	18	132-932	801
KU847592	D	D3	Brazil	18	132-1163	1032
KU847593	D	D3	Brazil	18	132-1163	1032
KU847596	D	D4	Brazil	18	132-932	801
KU847597	D	D3	Brazil	18	132-932	801
KU847618	D	D3	Brazil	18	132-1163	1032
KU847619	D	D4	Brazil	18	132-1163	1032
KU847623	D	D3	Brazil	18	132-1163	1032
KU847624	D	D4	Brazil	18	132-1163	1032
KU847633	D	D3	Brazil	18	132-1163	1032
KU847648	D	D3	Brazil	18	132-926	795
KU847741	D	D4	Brazil	18	132-1163	1032
MK336890	D	D3	Brazil	18	1-3182	1019
MK336891	D	D3	Brazil	18	1-3182	1019
MK336892	D	D3	Brazil	18	1-3182	1019
MK517518	D	D2	Brazil	18	1-3182	1271
FJ010648	D	D3	Brazil	19	322-936	627
FJ010649	D	D3/D6	Brazil	19	322-936	627
FJ010650	D	D4	Brazil	19	322-936	627
FJ865523	D	D3	Brazil	19	322-950	629
FJ865524	D	D3	Brazil	19	322-950	629
FJ865525	D	D3	Brazil	19	322-950	629
FJ865526	D	D3/D6	Brazil	19	322-950	629
FJ865527	D	D3/D6	Brazil	19	322-950	629
FJ865528	D	D3	Brazil	19	322-950	629
FJ865529	D	D3	Brazil	19	322-950	629
FJ865530	D	D3	Brazil	19	322-950	629
FJ865531	D	D3	Brazil	19	322-950	629
FJ865532	D	D3	Brazil	19	322-950	629
FJ865533	D	D3/D6	Brazil	19	322-950	629
FJ865534	D	D3	Brazil	19	322-950	629
FJ865535	D	D3	Brazil	19	322-950	629
FJ865536	D	D3	Brazil	19	322-950	629
FJ865537	D	D3	Brazil	19	322-950	629
FJ865538	D	D3	Brazil	19	322-950	629

FJ865539	D	D3/D6	Brazil	19	322-950	629
FJ865540	D	D3/D6	Brazil	19	322-950	629
FJ865541	D	D3	Brazil	19	322-950	629
FJ865542	D	D3	Brazil	19	322-950	629
FJ865543	D	D3	Brazil	19	322-950	629
FJ865544	D	D3	Brazil	19	322-950	629
FJ865545	D	D3	Brazil	19	322-950	629
FJ865546	D	D3	Brazil	19	322-950	629
FJ865547	D	D3	Brazil	19	322-950	629
FJ865548	D	D3	Brazil	19	322-950	629
FJ865549	D	D1	Brazil	19	322-950	629
FJ865550	D	D1	Brazil	19	322-950	629
FJ865551	D	D5	Brazil	19	322-950	629
FJ865552	D	D2	Brazil	19	322-950	629
FJ865553	D	D2	Brazil	19	322-950	629
FJ865554	D	D2	Brazil	19	322-950	629
FJ865555	D	D2	Brazil	19	322-950	629
FJ865556	D	D2	Brazil	19	322-950	629
FJ865557	D	D2	Brazil	19	322-950	629
FJ865558	D	D5	Brazil	19	322-950	629
HM101102	D	D4	Brazil	19	360-1243	884
HM136961	D	D2	Brazil	19	299-962	664
HM136962	D	D3	Brazil	19	275-979	705
HM136963	D	D3	Brazil	19	299-982	684
HM136964	D	D3	Brazil	19	275-982	708
HM136965	D	D3	Brazil	19	275-968	694
HM136980	D	D4	Brazil	19	275-982	708
HM136981	D	D4	Brazil	19	275-982	708
HM136982	D	D3	Brazil	19	299-982	684
HM136983	D	D3/D6	Brazil	19	275-982	708
HM136984	D	D3	Brazil	19	294-980	686
HM136985	D	D3	Brazil	19	275-858	584
HM136986	D	D3	Brazil	19	425-956	532
JF815665	D	D3/D6	Brazil	19	1-3182	1047
JF815666	D	D3/D6	Brazil	19	1-3182	1047
JF815670	D	D3/D6	Brazil	19	1-3182	1047
JF815672	D	D3/D6	Brazil	19	1-3182	1047
KT630610	D	D2	Brazil	19	157-837	681
KT630613	D	D3	Brazil	19	157-837	681
KT630618	D	D3	Brazil	19	157-837	681
KT630620	D	D3	Brazil	19	157-837	681

KT630629	D	D2	Brazil	19	157-837	681
KU847598	D	D2	Brazil	19	132-1163	1032
KU847599	D	D2	Brazil	19	132-1163	1032
KU847602	D	D9	Brazil	19	132-1163	1032
KU847611	D	D2	Brazil	19	132-1163	1032
KU847735	D	D2	Brazil	19	132-1163	1032
MK333247	D	D3	Brazil	19	179-842	664
MN758707	E	E	Brazil	20	33-1277	1245
EF151263	E	E	Brazil	21	295-830	536
EF547857	E	E	Brazil	21	157-837	681
FJ865559	E	E	Brazil	21	322-950	629
MN845918	E	E	Brazil	21	1-3182	882
KC494394	F	F2a	Brazil	22	Complete Genome	3215
KC494395	F	F2a	Brazil	22	Complete Genome	3215
KC494396	F	F2a	Brazil	22	Complete Genome	3215
KC494397	F	F2a	Brazil	22	Complete Genome	3215
KC494398	F	F4	Brazil	22	Complete Genome	3215
KC494399	F	F2a	Brazil	22	Complete Genome	3215
KC494400	F	F1b	Brazil	22	Complete Genome	3215
KC494401	F	F2a	Brazil	22	Complete Genome	3215
KC494402	F	F2a	Brazil	22	Complete Genome	3215
KC494403	F	F2a	Brazil	22	Complete Genome	3215
KC494404	F	F1b	Brazil	22	Complete Genome	3215
KC494405	F	F2a	Brazil	22	Complete Genome	3215
HM101096	F	F2a	Brazil	23	6-1267	1265
HM101097	F	F2a	Brazil	23	6-1275	1273
HM101098	F	F2a	Brazil	23	6-1280	1278
HM101099	F	F2a	Brazil	23	17-1280	1263
HM101125	F	F2a	Brazil	23	6-1278	1276
HM101130	F	F2a	Brazil	23	6-1277	1275
JN983942	F	F2a	Brazil	23	9-1274	1269
JN983943	F	F2a	Brazil	23	9-1274	1269
JN983944	F	F2a	Brazil	23	9-1274	1269
JN983945	F	F2a	Brazil	23	9-1274	1269
JN983946	F	F2a	Brazil	23	9-1274	1269
JN983947	F	F2a	Brazil	23	9-1274	1269
KX264660	F	F2a	Brazil	23	22-1239	1218
KX264661	F	F2a	Brazil	23	39-1190	1152
KY809938	F	F2a	Brazil	23	8-1274	1270
KY809939	F	F4	Brazil	23	8-1274	1270
KY809940	F	F2a	Brazil	23	8-1274	1270

KY809941	F	F2a	Brazil	23	8-1274	1237
KY809942	F	F2a	Brazil	23	8-1274	1270
KY809943	F	F2a	Brazil	23	8-1274	1270
KY809944	F	F2a	Brazil	23	8-1274	1270
KY809945	F	F4	Brazil	23	8-1274	1270
KY809946	F	F2a	Brazil	23	8-1274	1270
FJ010651	F	F2a	Brazil	24	310-936	627
FJ010652	F	F2a	Brazil	24	310-936	627
FJ010653	F	F2a	Brazil	24	310-936	627
FJ010654	F	F2a	Brazil	24	310-936	627
FJ010655	F	F2a	Brazil	24	310-936	627
FJ010656	F	F2a	Brazil	24	310-936	627
FJ010657	F	F2a	Brazil	24	310-936	627
HM101100	F	F2a	Brazil	24	274-1280	1007
HM136968	F	F2a	Brazil	24	294-982	689
HM136969	F	F2a	Brazil	24	275-982	708
HM136970	F	F2a	Brazil	24	275-982	708
HM136971	F	F2a	Brazil	24	275-982	708
EF464097	G	G	Brazil	25	Complete Genome	3248
EF464098	G	G	Brazil	25	Complete Genome	3248
EF464099	G	G	Brazil	25	Complete Genome	3248
KX264500	G	G	Brazil	25	Complete Genome	3248
GU968717	G	G	Brazil	26	312-789	478
GU968727	G	G	Brazil	26	312-789	478

[1]Alignment to complete genome reference sequence VHB NC_003977.2

GUYANA

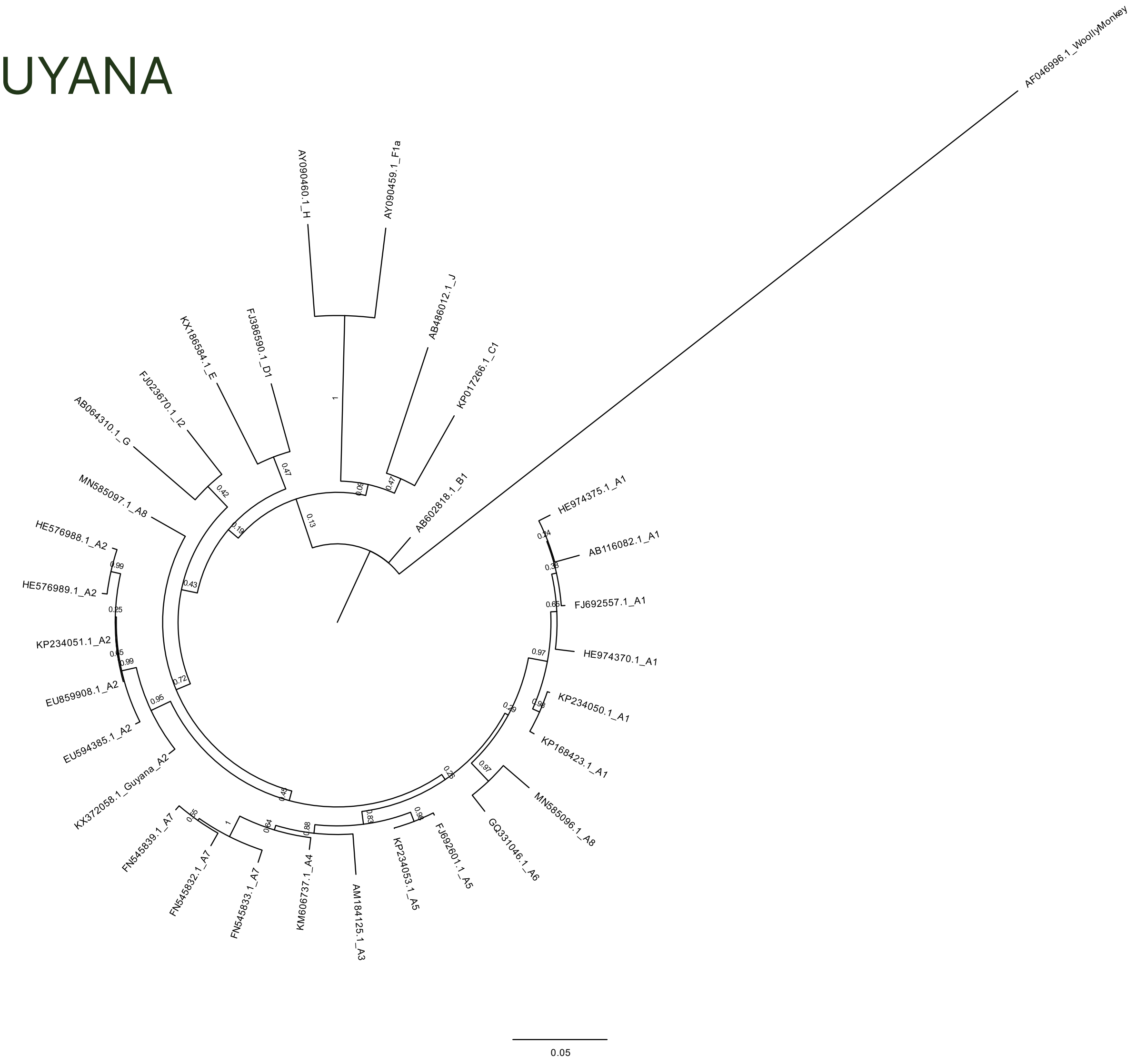

Tree 1. The evolutionary history was inferred by using the Maximum Likelihood method and Tamura-Nei model. The percentage of replicate trees in which the associated taxa clustered together in the bootstrap test (1000 replicates) are shown next to the branches. Initial tree(s) for the heuristic search were obtained automatically by applying Neighbor-Join and BioNJ algorithms to a matrix of pairwise distances estimated using the Tamura-Nei model, and then selecting the topology with superior log likelihood value. A discrete Gamma distribution was used to model evolutionary rate differences among sites (5 categories (+G, parameter = 0.1642)). The tree is drawn to scale, with branch lengths measured in the number of substitutions per site. The analysis involved 32 nucleotide sequences, of which 31 were used as marker sequences to determine the genotype of 1 sequence. All positions containing gaps and missing data were eliminated. There was a total of 898 positions in the final dataset. Evolutionary analyses were conducted in MEGA X.

ID	GENOTYPE	SUBTYPE	COUNTRY	TREE	ALIGNMENT[1]	BASE PAIRS
KX372058	A	A2	Guyana	1	6-971	966

[1]Alignment to complete genome reference sequence VHB NC_003977.2

PARAGUAY

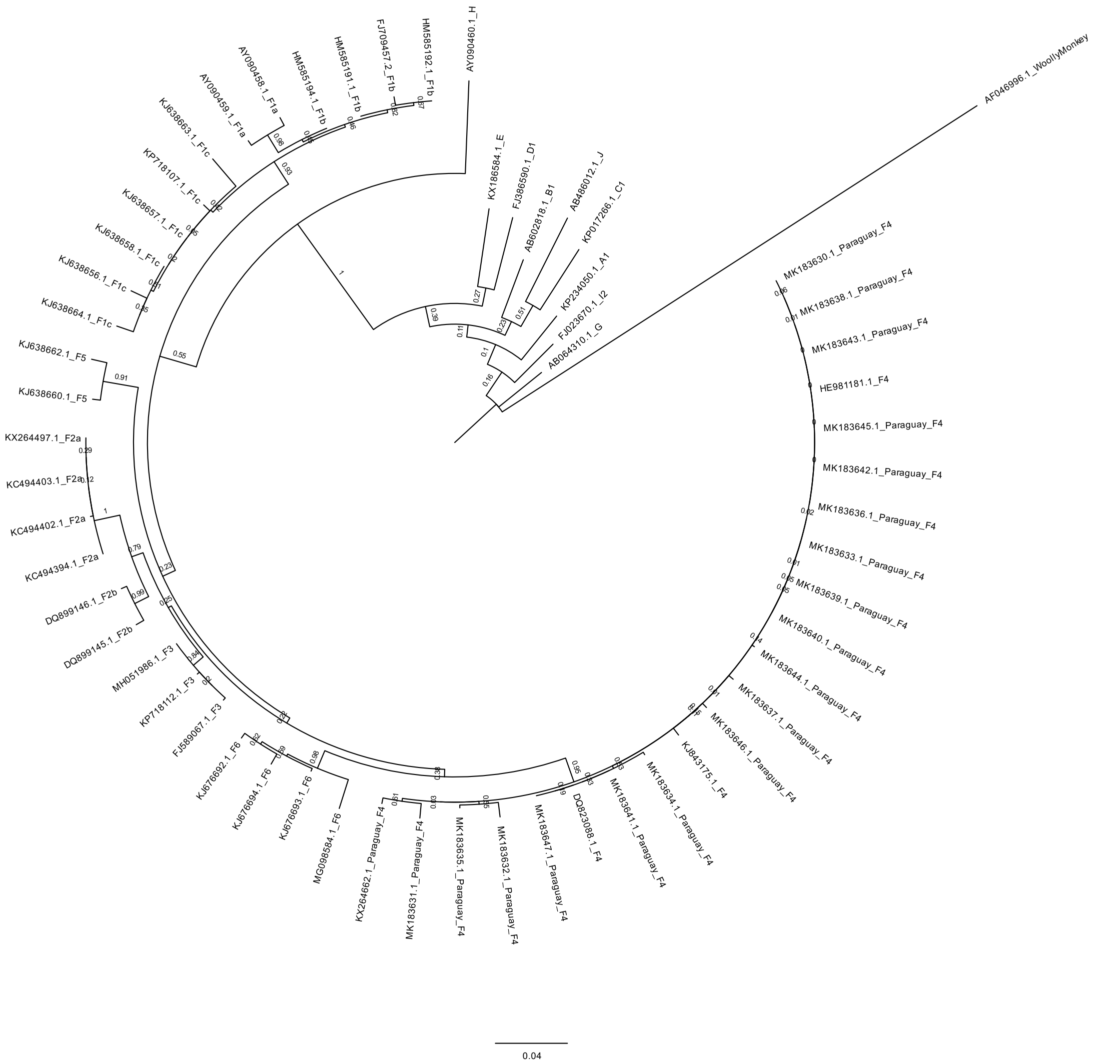

Tree 1. The evolutionary history was inferred by using the Maximum Likelihood method and Tamura-Nei model. The percentage of replicate trees in which the associated taxa clustered together in the bootstrap test (1000 replicates) are shown next to the branches. Initial tree(s) for the heuristic search were obtained automatically by applying Neighbor-Join and BioNJ algorithms to a matrix of pairwise distances estimated using the Tamura-Nei model, and then selecting the topology with superior log likelihood value. A discrete Gamma distribution was used to model evolutionary rate differences among sites (5 categories (+G, parameter = 0.1909)). The tree is drawn to scale, with branch lengths measured in the number of substitutions per site. The analysis involved 59 nucleotide sequences, of which 40 were used as marker sequences to determine the genotype of 19 sequences. All positions containing gaps and missing data were eliminated. There was a total of 736 positions in the final dataset. Evolutionary analyses were conducted in MEGA X.

ID	GENOTYPE	SUBTYPE	COUNTRY	TREE	ALIGNMENT[1]	BASE PAIRS
KX264662	F	F4	Paraguay	1	46-1220	1175
MK183635	F	F4	Paraguay	1	246-2942	2730

MK183634	F	F4	Paraguay	1	Complete Genome	3214
MK183642	F	F4	Paraguay	1	Complete Genome	3214
MK183630	F	F4	Paraguay	1	Complete Genome	3215
MK183631	F	F4	Paraguay	1	Complete Genome	3215
MK183632	F	F4	Paraguay	1	Complete Genome	3215
MK183633	F	F4	Paraguay	1	Complete Genome	3215
MK183636	F	F4	Paraguay	1	Complete Genome	3215
MK183637	F	F4	Paraguay	1	Complete Genome	3215
MK183638	F	F4	Paraguay	1	Complete Genome	3215
MK183639	F	F4	Paraguay	1	Complete Genome	3215
MK183640	F	F4	Paraguay	1	Complete Genome	3215
MK183641	F	F4	Paraguay	1	Complete Genome	3215
MK183643	F	F4	Paraguay	1	Complete Genome	3215
MK183644	F	F4	Paraguay	1	Complete Genome	3215
MK183645	F	F4	Paraguay	1	Complete Genome	3215
MK183647	F	F4	Paraguay	1	Complete Genome	3215
MK183646	F	F4	Paraguay	1	Complete Genome	3216

[1]Alignment to complete genome reference sequence VHB NC_003977.2

SURINAME

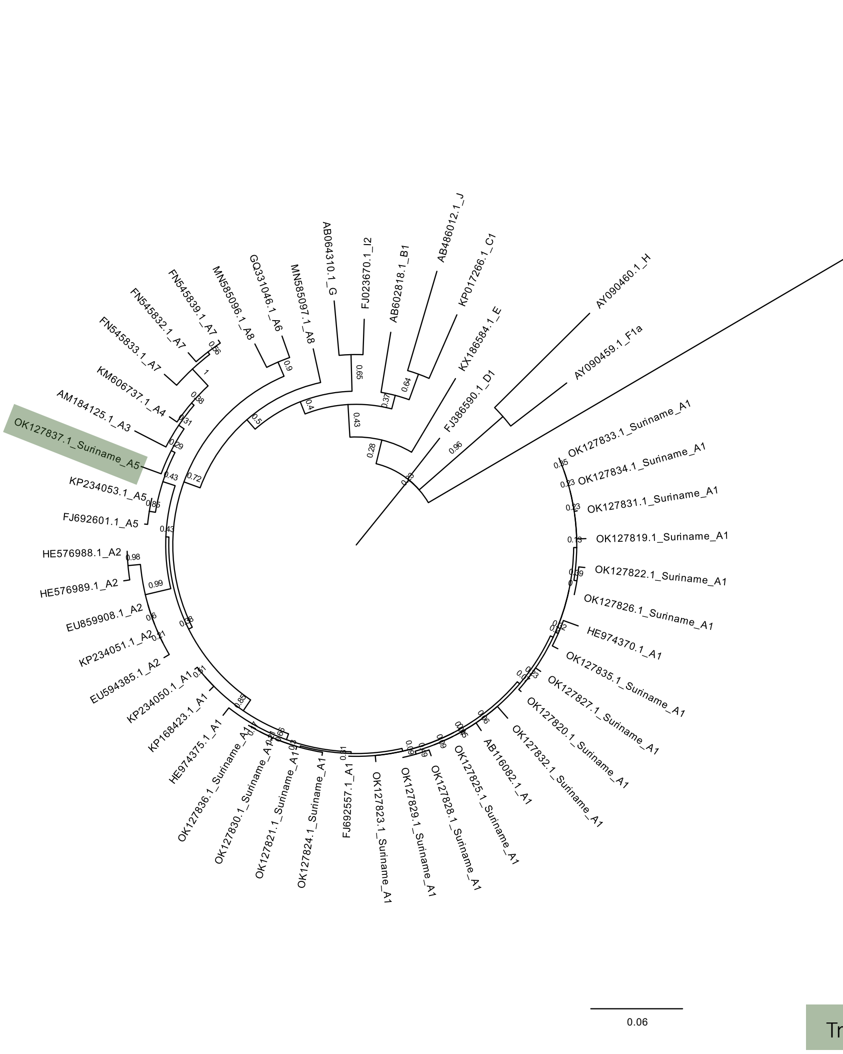

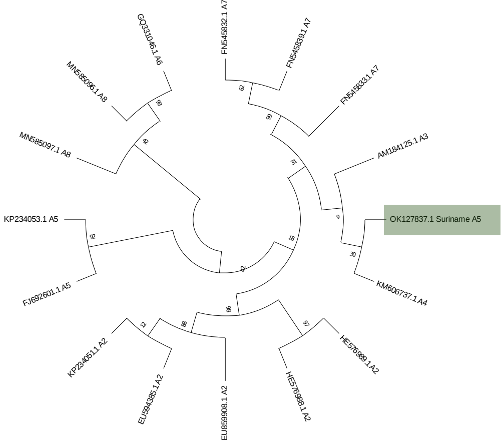

Tree 1.1

Tree 1. The evolutionary history was inferred by using the Maximum Likelihood method and Tamura-Nei model. The percentage of replicate trees in which the associated taxa clustered together in the bootstrap test (1000 replicates) are shown next to the branches. Initial tree(s) for the heuristic search were obtained automatically by applying Neighbor-Join and BioNJ algorithms to a matrix of pairwise distances estimated using the Tamura-Nei model, and then selecting the topology with superior log likelihood value. A discrete Gamma distribution was used to model evolutionary rate differences among sites (5 categories (+G, parameter = 0.1608)). The tree is drawn to scale, with branch lengths measured in the number of substitutions per site. The analysis involved 50 nucleotide sequences, of which 31 were used as marker sequences to determine the genotype of 19 sequences. Sequence OK127837 was further analyzed (Tree 1.1) and reclassified to subgenotype A4. All positions containing gaps and missing data were eliminated. There was a total of 629 positions in the final dataset. Evolutionary analyses were conducted in MEGA X.

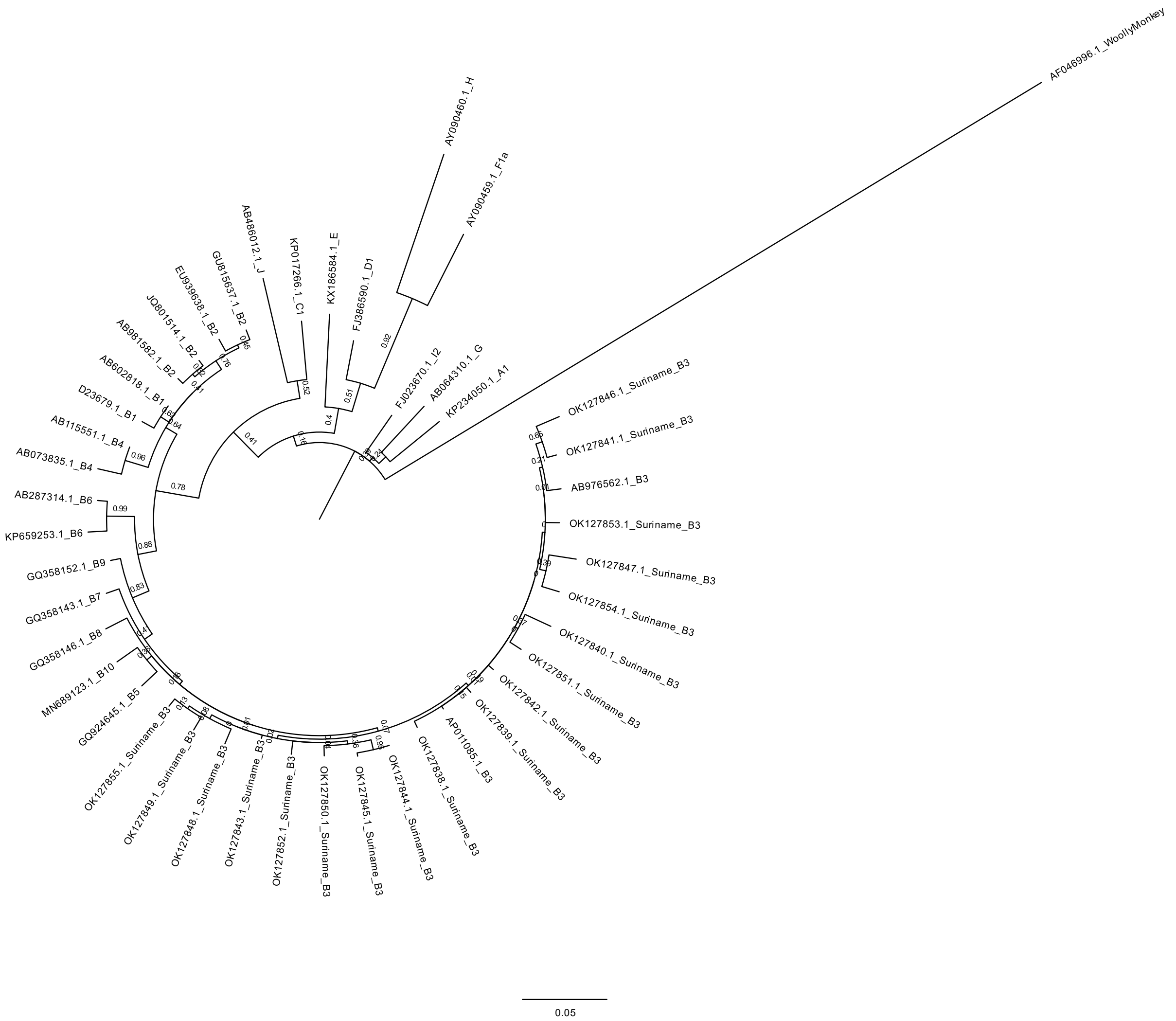

Tree 2. The evolutionary history was inferred by using the Maximum Likelihood method and Tamura-Nei model. The percentage of replicate trees in which the associated taxa clustered together in the bootstrap test (1000 replicates) are shown next to the branches. Initial tree(s) for the heuristic search were obtained automatically by applying Neighbor-Join and BioNJ algorithms to a matrix of pairwise distances estimated using the Tamura-Nei model, and then selecting the topology with superior log likelihood value. A discrete Gamma distribution was used to model evolutionary rate differences among sites (5 categories (+G, parameter = 0.1639)). The tree is drawn to scale, with branch lengths measured in the number of substitutions per site. The analysis involved 45 nucleotide sequences, of which 27 were used as marker sequences to determine the genotype of 18 sequences. All positions containing gaps and missing data were eliminated. There was a total of 616 positions in the final dataset. Evolutionary analyses were conducted in MEGA X.

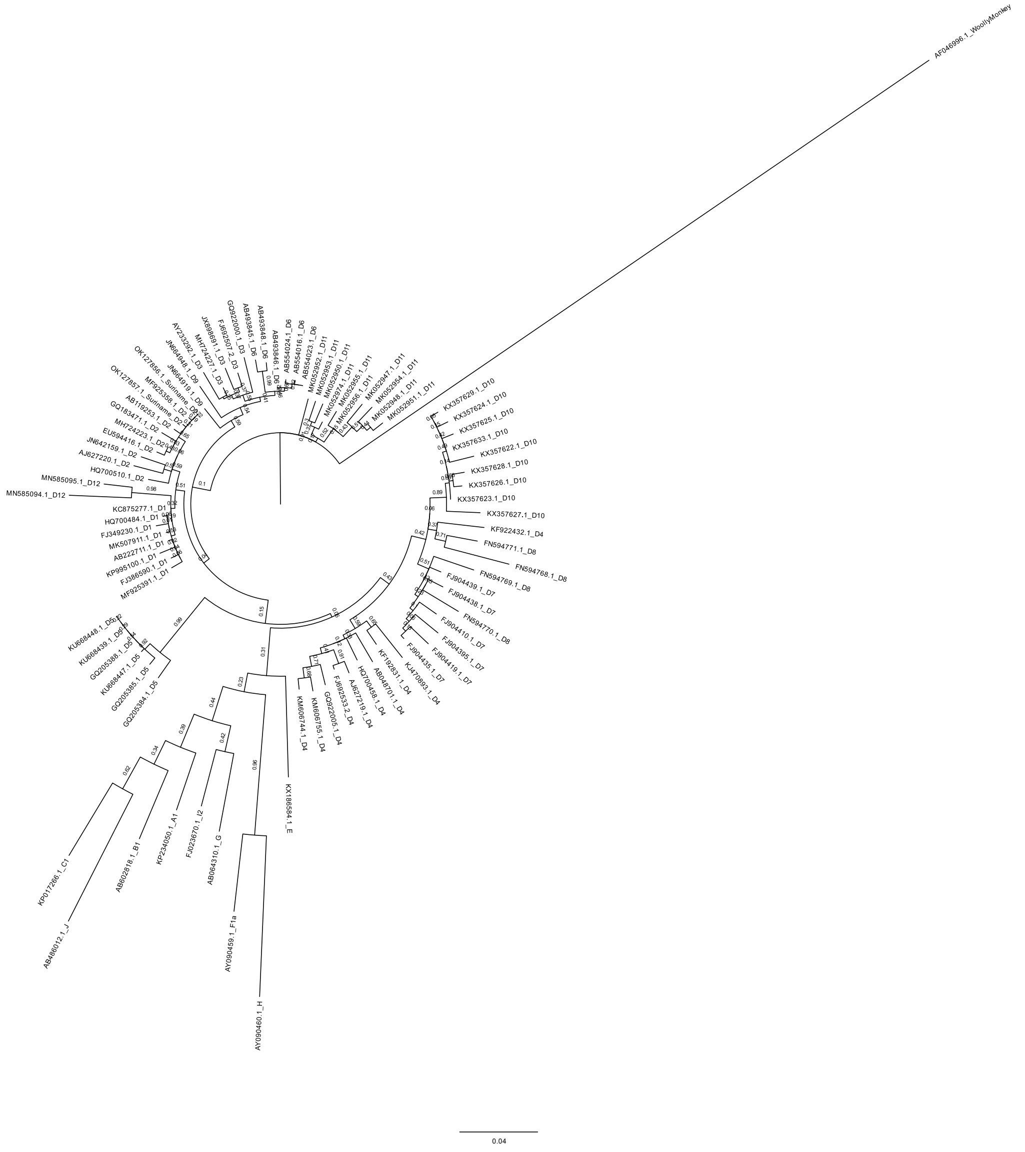

Tree 3. The evolutionary history was inferred by using the Maximum Likelihood method and Tamura-Nei model. The percentage of replicate trees in which the associated taxa clustered together in the bootstrap test (1000 replicates) are shown next to the branches. Initial tree(s) for the heuristic search were obtained automatically by applying Neighbor-Join and BioNJ algorithms to a matrix of pairwise distances estimated using the Tamura-Nei model, and then selecting the topology with superior log likelihood value. A discrete Gamma distribution was used to model evolutionary rate differences among sites (5 categories (+G, parameter = 0.2390)). The tree is drawn to scale, with branch lengths measured in the number of substitutions per site. The analysis involved 88 nucleotide sequences, of which 86 were used as marker sequences to determine the genotype of 2 sequences. All positions containing gaps and missing data were eliminated. There was a total of 635 positions in the final dataset. Evolutionary analyses were conducted in MEGA X.

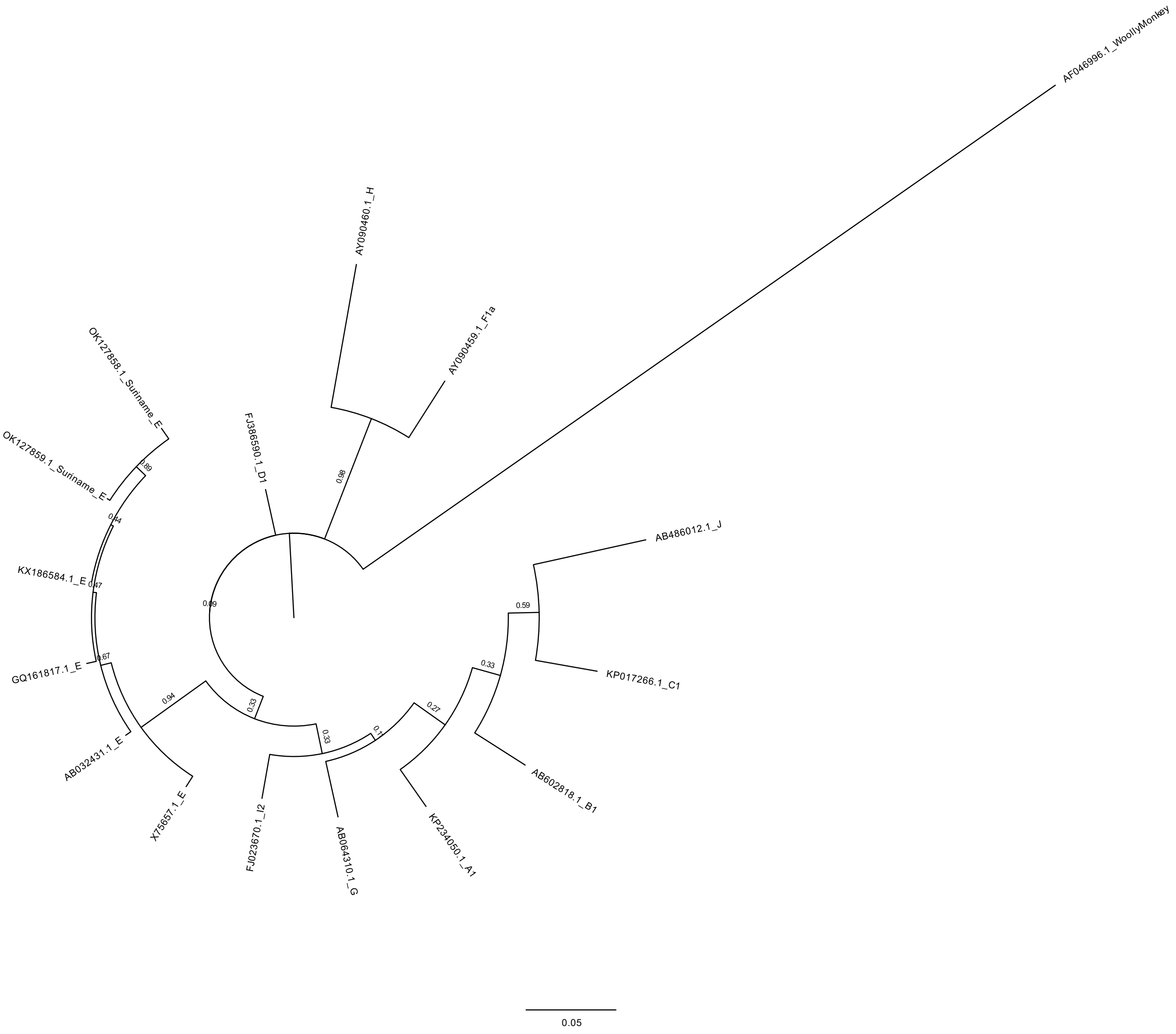

Tree 4. The evolutionary history was inferred by using the Maximum Likelihood method and Tamura-Nei model. The percentage of replicate trees in which the associated taxa clustered together in the bootstrap test (1000 replicates) are shown next to the branches. Initial tree(s) for the heuristic search were obtained automatically by applying Neighbor-Join and BioNJ algorithms to a matrix of pairwise distances estimated using the Tamura-Nei model, and then selecting the topology with superior log likelihood value. A discrete Gamma distribution was used to model evolutionary rate differences among sites (5 categories (+G, parameter = 0.1397)). The tree is drawn to scale, with branch lengths measured in the number of substitutions per site. The analysis involved 16 nucleotide sequences, of which 14 were used as marker sequences to determine the genotype of 2 sequences. All positions containing gaps and missing data were eliminated. There was a total of 643 positions in the final dataset. Evolutionary analyses were conducted in MEGA X.

ID	GENOTYPE	SUBTYPE	COUNTRY	TREE	ALIGNMENT[1]	BASE PAIRS
OK127819	A	A1	Suriname	1	318-969	652
OK127820	A	A1	Suriname	1	318-969	652
OK127821	A	A1	Suriname	1	318-969	652

OK127822	A	A1	Suriname	1	318-969	652
OK127823	A	A1	Suriname	1	318-969	652
OK127824	A	A1	Suriname	1	318-969	652
OK127825	A	A1	Suriname	1	318-969	652
OK127826	A	A1	Suriname	1	318-969	652
OK127827	A	A1	Suriname	1	318-969	652
OK127828	A	A1	Suriname	1	318-969	652
OK127829	A	A1	Suriname	1	318-969	652
OK127830	A	A1	Suriname	1	318-969	652
OK127831	A	A1	Suriname	1	318-969	652
OK127832	A	A1	Suriname	1	318-969	652
OK127833	A	A1	Suriname	1	318-969	652
OK127834	A	A1	Suriname	1	318-969	652
OK127835	A	A1	Suriname	1	318-969	652
OK127836	A	A1	Suriname	1	318-969	652
OK127837	A	A4	Suriname	1	318-969	652
OK127838	B	B3	Suriname	2	318-969	652
OK127839	B	B3	Suriname	2	318-969	652
OK127840	B	B3	Suriname	2	318-969	652
OK127841	B	B3	Suriname	2	318-969	652
OK127842	B	B3	Suriname	2	318-969	652
OK127843	B	B3	Suriname	2	318-969	652
OK127844	B	B3	Suriname	2	318-969	652
OK127845	B	B3	Suriname	2	318-969	652
OK127846	B	B3	Suriname	2	318-969	652
OK127847	B	B3	Suriname	2	318-969	652
OK127848	B	B3	Suriname	2	318-969	652
OK127849	B	B3	Suriname	2	318-969	652
OK127850	B	B3	Suriname	2	318-969	652
OK127851	B	B3	Suriname	2	318-969	652
OK127852	B	B3	Suriname	2	318-969	652
OK127853	B	B3	Suriname	2	318-969	652
OK127854	B	B3	Suriname	2	318-969	652
OK127855	B	B3	Suriname	2	318-969	652
OK127856	D	D2	Suriname	3	318-969	652
OK127857	D	D2	Suriname	3	318-969	652
OK127858	E	E	Suriname	4	318-969	652
OK127859	E	E	Suriname	4	318-969	652

[1]Alignment to complete genome reference sequence VHB NC_003977.2

VENEZUELA

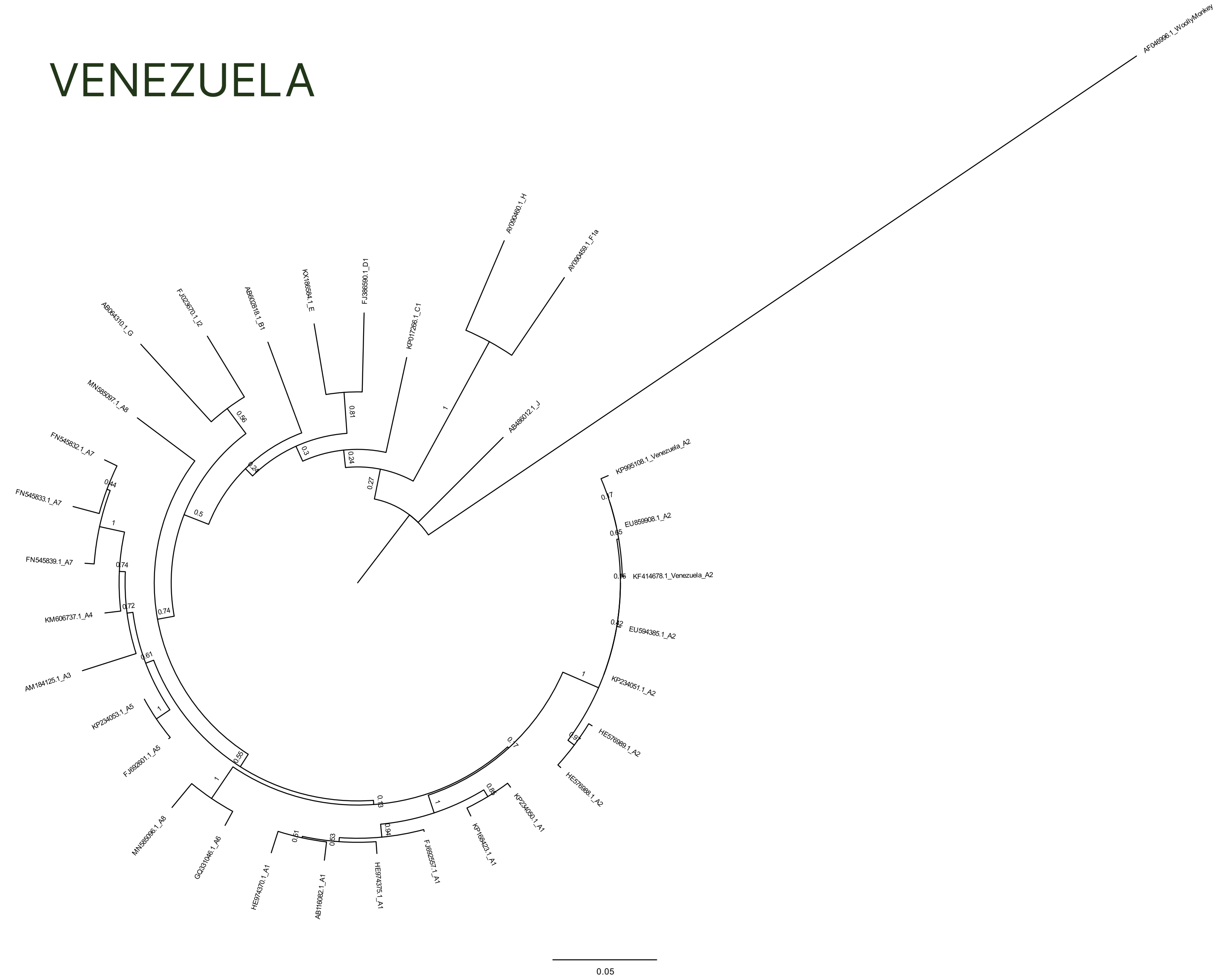

Tree 1. The evolutionary history was inferred by using the Maximum Likelihood method and Tamura-Nei model. The percentage of replicate trees in which the associated taxa clustered together in the bootstrap test (1000 replicates) are shown next to the branches. Initial tree(s) for the heuristic search were obtained automatically by applying Neighbor-Join and BioNJ algorithms to a matrix of pairwise distances estimated using the Tamura-Nei model, and then selecting the topology with superior log likelihood value. A discrete Gamma distribution was used to model evolutionary rate differences among sites (5 categories (+G, parameter = 0.1816)). The tree is drawn to scale, with branch lengths measured in the number of substitutions per site. The analysis involved 33 nucleotide sequences, of which 31 were used as marker sequences to determine the genotype of 2 sequences. All positions containing gaps and missing data were eliminated. There was a total of 1238 positions in the final dataset. Evolutionary analyses were conducted in MEGA X.

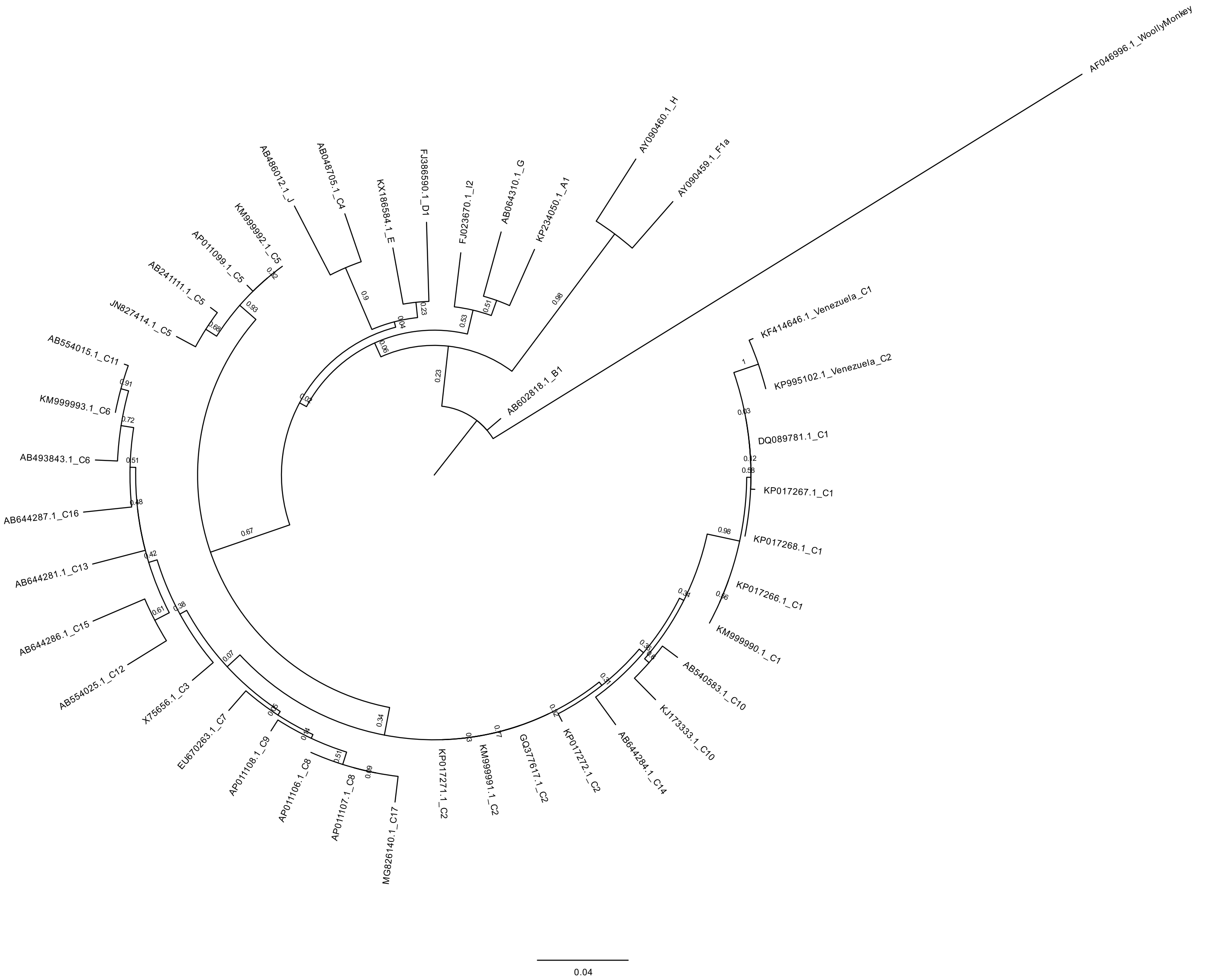

Tree 2. The evolutionary history was inferred by using the Maximum Likelihood method and Tamura-Nei model. The percentage of replicate trees in which the associated taxa clustered together in the bootstrap test (1000 replicates) are shown next to the branches. Initial tree(s) for the heuristic search were obtained automatically by applying Neighbor-Join and BioNJ algorithms to a matrix of pairwise distances estimated using the Tamura-Nei model, and then selecting the topology with superior log likelihood value. A discrete Gamma distribution was used to model evolutionary rate differences among sites (5 categories (+G, parameter = 0.1460)). The tree is drawn to scale, with branch lengths measured in the number of substitutions per site. The analysis involved 42 nucleotide sequences, of which 40 were used as marker sequences to determine the genotype of 2 sequences. All positions containing gaps and missing data were eliminated. There was a total of 703 positions in the final dataset. Evolutionary analyses were conducted in MEGA X.

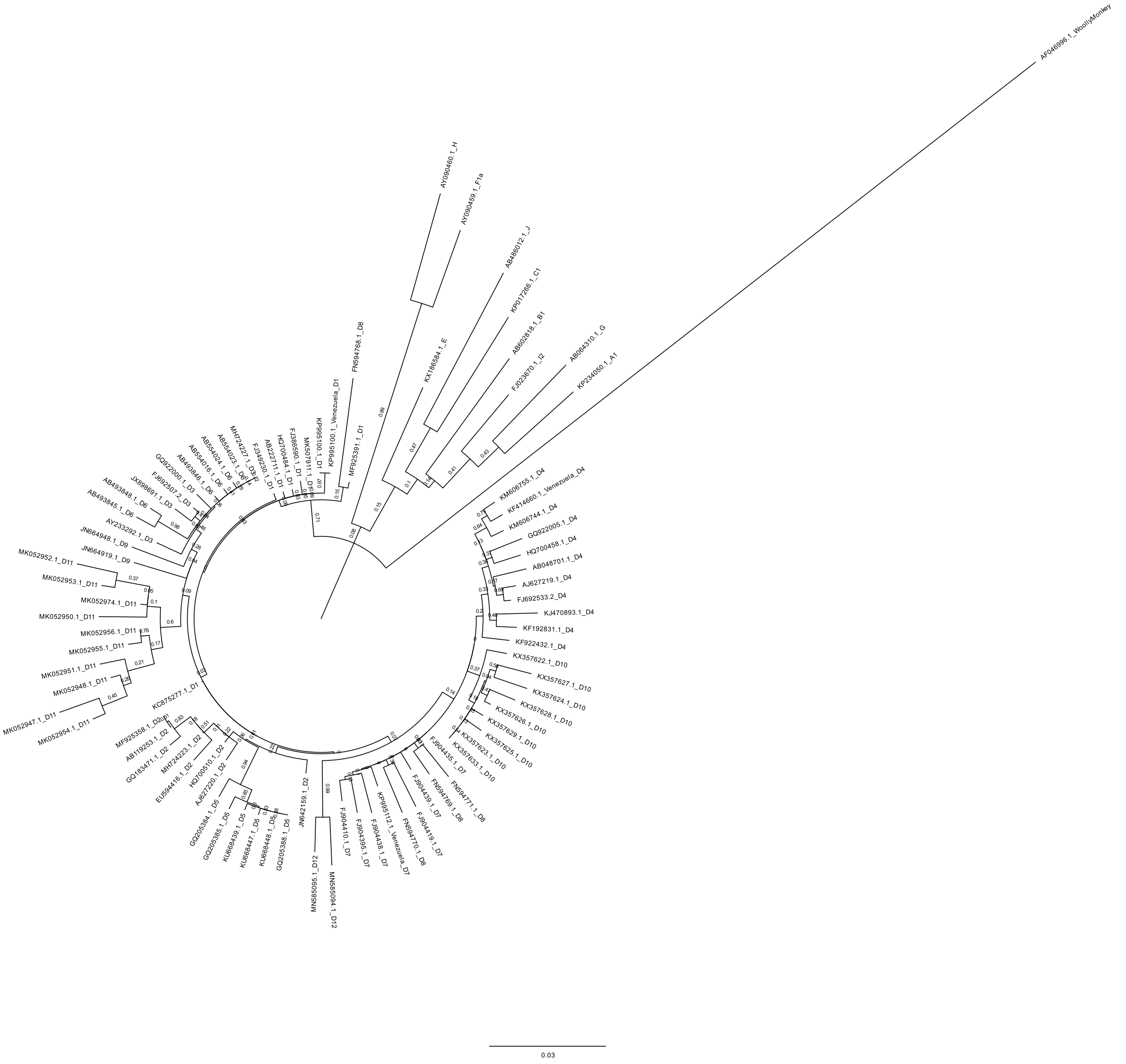

Tree 3. The evolutionary history was inferred by using the Maximum Likelihood method and Tamura-Nei model. The percentage of replicate trees in which the associated taxa clustered together in the bootstrap test (1000 replicates) are shown next to the branches. Initial tree(s) for the heuristic search were obtained automatically by applying Neighbor-Join and BioNJ algorithms to a matrix of pairwise distances estimated using the Tamura-Nei model, and then selecting the topology with superior log likelihood value. A discrete Gamma distribution was used to model evolutionary rate differences among sites (5 categories (+G, parameter = 0.2382)). The tree is drawn to scale, with branch lengths measured in the number of substitutions per site. The analysis involved 89 nucleotide sequences, of which 86 were used as marker sequences to determine the genotype of 3 sequences. All positions containing gaps and missing data were eliminated. There was a total of 697 positions in the final dataset. Evolutionary analyses were conducted in MEGA X.

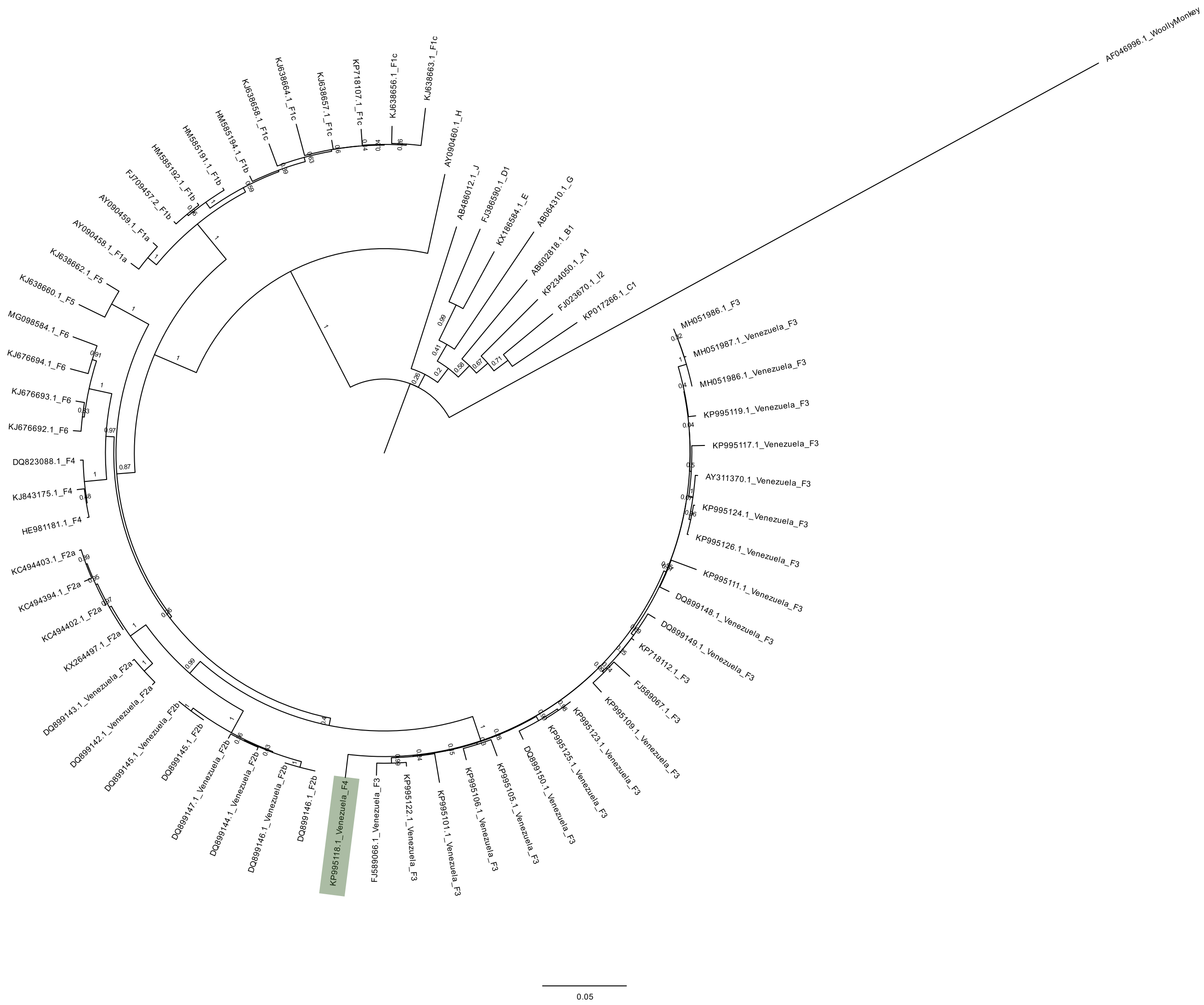

Tree 4. The evolutionary history was inferred by using the Maximum Likelihood method and Tamura-Nei model. The percentage of replicate trees in which the associated taxa clustered together in the bootstrap test (1000 replicates) are shown next to the branches. Initial tree(s) for the heuristic search were obtained automatically by applying Neighbor-Join and BioNJ algorithms to a matrix of pairwise distances estimated using the Tamura-Nei model, and then selecting the topology with superior log likelihood value. A discrete Gamma distribution was used to model evolutionary rate differences among sites (5 categories (+G, parameter = 0.2736)). The tree is drawn to scale, with branch lengths measured in the number of substitutions per site. The analysis involved 66 nucleotide sequences, of which 40 were used as marker sequences to determine the genotype of 26 sequences. Sequence KP995118 was reclassified to subgenotype F3. All positions containing gaps and missing data were eliminated. There was a total of 3038 positions in the final dataset. Evolutionary analyses were conducted in MEGA X.

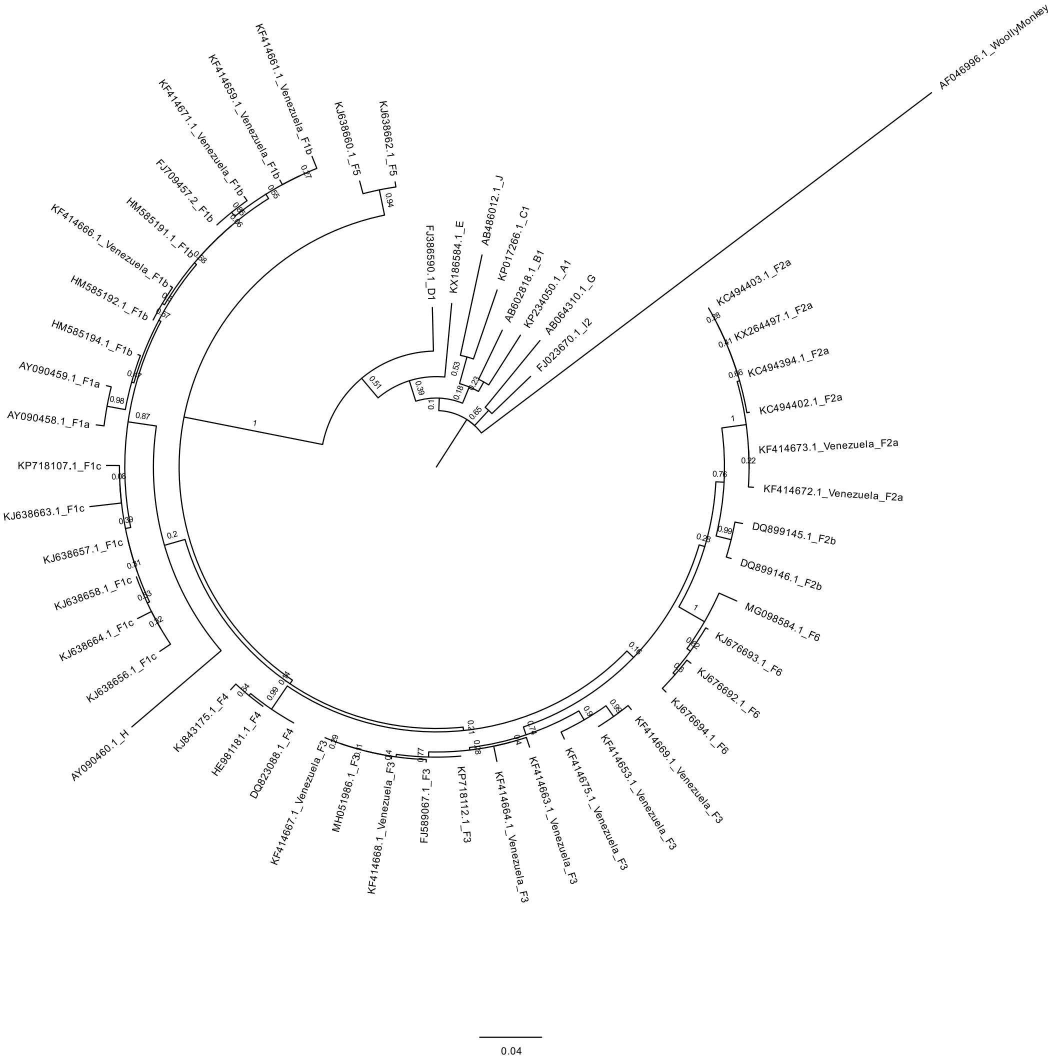

Tree 5. The evolutionary history was inferred by using the Maximum Likelihood method and Tamura-Nei model. The percentage of replicate trees in which the associated taxa clustered together in the bootstrap test (1000 replicates) are shown next to the branches. Initial tree(s) for the heuristic search were obtained automatically by applying Neighbor-Join and BioNJ algorithms to a matrix of pairwise distances estimated using the Tamura-Nei model, and then selecting the topology with superior log likelihood value. A discrete Gamma distribution was used to model evolutionary rate differences among sites (5 categories (+G, parameter = 0.1898)). The tree is drawn to scale, with branch lengths measured in the number of substitutions per site. The analysis involved 53 nucleotide sequences, of which 40 were used as marker sequences to determine the genotype of 13 sequences. All positions containing gaps and missing data were eliminated. There was a total of 707 positions in the final dataset. Evolutionary analyses were conducted in MEGA X.

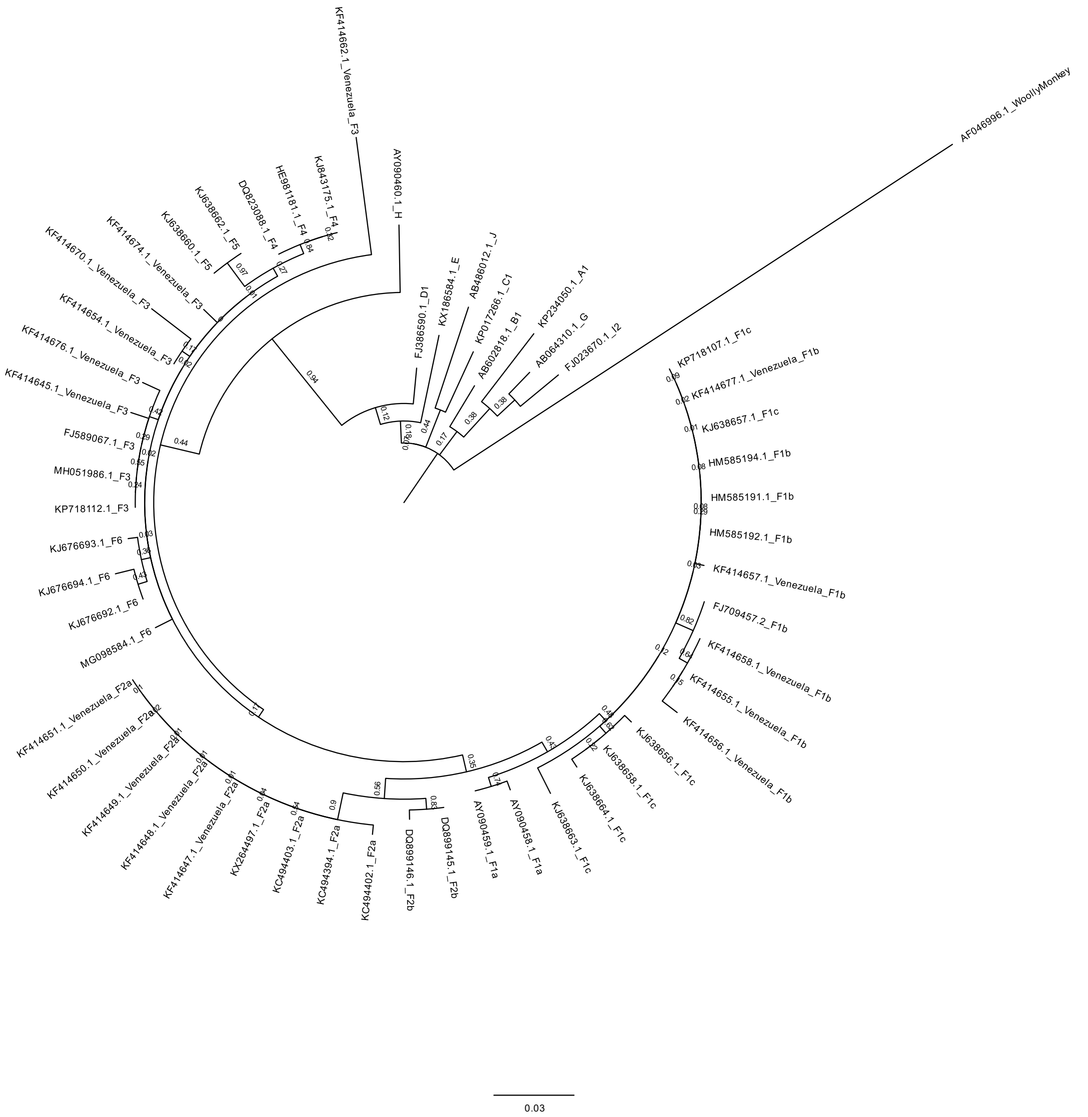

Tree 6. The evolutionary history was inferred by using the Maximum Likelihood method and Tamura-Nei model. The percentage of replicate trees in which the associated taxa clustered together in the bootstrap test (1000 replicates) are shown next to the branches. Initial tree(s) for the heuristic search were obtained automatically by applying Neighbor-Join and BioNJ algorithms to a matrix of pairwise distances estimated using the Tamura-Nei model, and then selecting the topology with superior log likelihood value. A discrete Gamma distribution was used to model evolutionary rate differences among sites (5 categories (+G, parameter = 0.1645)). The tree is drawn to scale, with branch lengths measured in the number of substitutions per site. The analysis involved 56 nucleotide sequences, of which 40 were used as marker sequences to determine the genotype of 16 sequences. Certain sequences in the analysis aligned differently than what originally labeled; additional analyses were done and concluded that no changes would need to be made. All positions containing gaps and missing data were eliminated. There was a total of 330 positions in the final dataset. Evolutionary analyses were conducted in MEGA X.

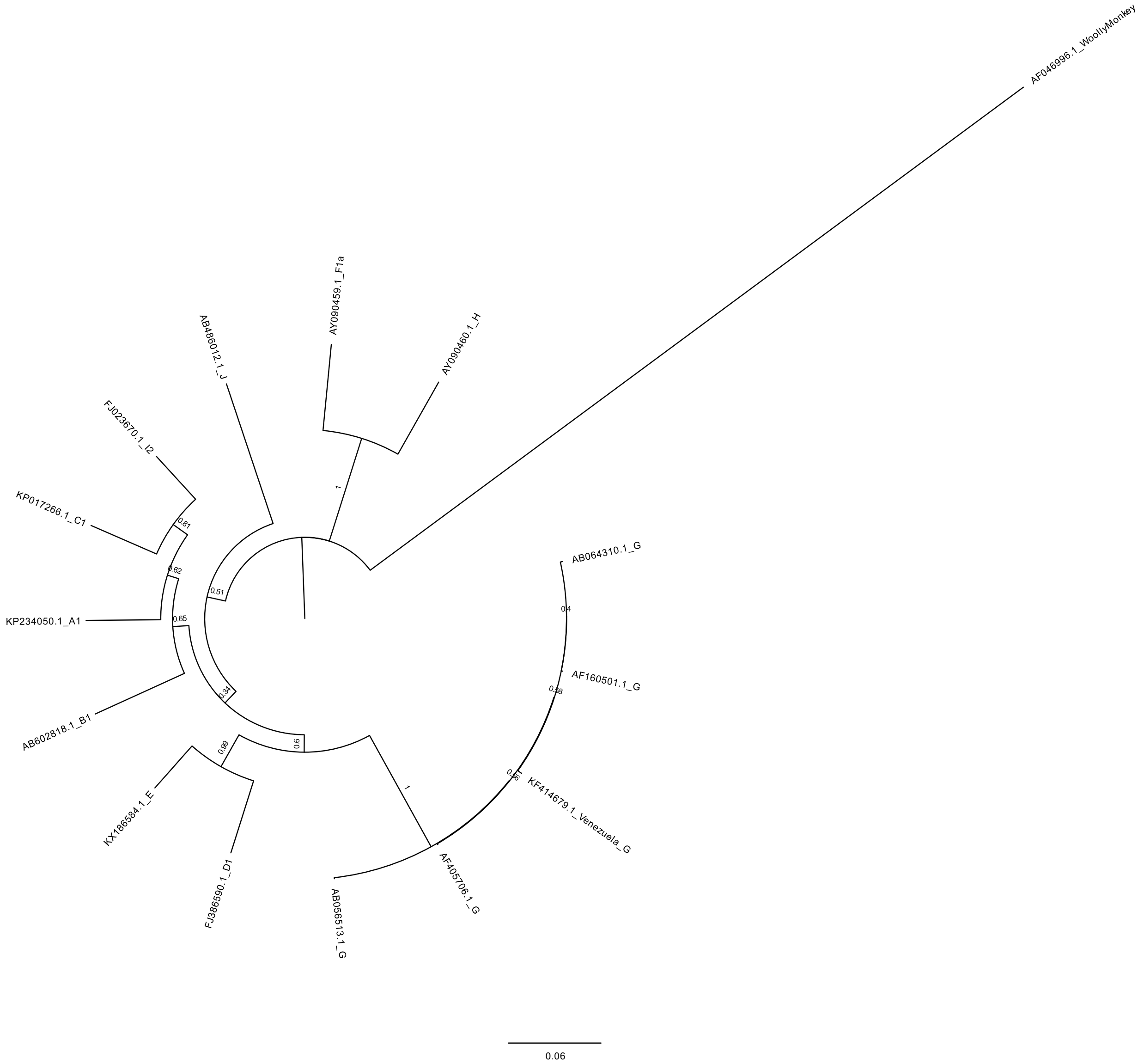

Tree 7. The evolutionary history was inferred by using the Maximum Likelihood method and Tamura-Nei model. The percentage of replicate trees in which the associated taxa clustered together in the bootstrap test (1000 replicates) are shown next to the branches. Initial tree(s) for the heuristic search were obtained automatically by applying Neighbor-Join and BioNJ algorithms to a matrix of pairwise distances estimated using the Tamura-Nei model, and then selecting the topology with superior log likelihood value. A discrete Gamma distribution was used to model evolutionary rate differences among sites (5 categories (+G, parameter = 0.2268)). The tree is drawn to scale, with branch lengths measured in the number of substitutions per site. The analysis involved 15 nucleotide sequences, of which 14 were used as marker sequences to determine the genotype of 1 sequence. All positions containing gaps and missing data were eliminated. There was a total of 3160 positions in the final dataset. Evolutionary analyses were conducted in MEGA X.

ID	GENOTYPE	SUBTYPE	COUNTRY	TREE	ALIGNMENT[1]	BASE PAIRS
KF414678	A	A2	Venezuela	1	31-1318	1288
KP995108	A	A2	Venezuela	1	Complete Genome	3221
KF414646	C	C1	Venezuela	2	130-838	709

KP995102	C	C1	Venezuela	2	Complete Genome	3216
KP995100	D	D1	Venezuela	3	Complete Genome	3182
KF414660	D	D4	Venezuela	3	132-845	714
KP995112	D	D7	Venezuela	3	Complete Genome	3182
KP995122	F	F3	Venezuela	4	Complete Genome	3214
DQ899142	F	F2a?	Venezuela	4	Complete Genome	3215
DQ899143	F	F2a?	Venezuela	4	Complete Genome	3215
DQ899144	F	F2b?	Venezuela	4	Complete Genome	3215
DQ899145	F	F2b?	Venezuela	4	Complete Genome	3215
DQ899146	F	F2b?	Venezuela	4	Complete Genome	3215
DQ899147	F	F2b?	Venezuela	4	Complete Genome	3215
AY311370	F	F3	Venezuela	4	Complete Genome	3215
DQ899148	F	F3	Venezuela	4	Complete Genome	3215
DQ899149	F	F3	Venezuela	4	Complete Genome	3215
DQ899150	F	F3	Venezuela	4	Complete Genome	3215
FJ589066	F	F3	Venezuela	4	Complete Genome	3215
KP995101	F	F3	Venezuela	4	Complete Genome	3215
KP995105	F	F3	Venezuela	4	Complete Genome	3215
KP995106	F	F3	Venezuela	4	Complete Genome	3215
KP995109	F	F3	Venezuela	4	Complete Genome	3215
KP995111	F	F3	Venezuela	4	Complete Genome	3215
KP995117	F	F3	Venezuela	4	Complete Genome	3215
KP995119	F	F3	Venezuela	4	Complete Genome	3215
KP995123	F	F3	Venezuela	4	Complete Genome	3215
KP995124	F	F3	Venezuela	4	Complete Genome	3215
KP995125	F	F3	Venezuela	4	Complete Genome	3215
KP995126	F	F3	Venezuela	4	Complete Genome	3215
MH051986	F	F3	Venezuela	4	Complete Genome	3215
MH051987	F	F3	Venezuela	4	Complete Genome	3215
KP995118	F	F3	Venezuela	4	Complete Genome	3215
KF414653	F	F3	Venezuela	5	217-1465	1249
KF414659	F	F1b	Venezuela	5	26-1192	1167
KF414661	F	F1b	Venezuela	5	99-1402	1304
KF414663	F	F3	Venezuela	5	217-1087	871
KF414664	F	F3	Venezuela	5	99-1061	963
KF414666	F	F1b	Venezuela	5	48-1481	1434
KF414667	F	F3	Venezuela	5	217-1506	1290
KF414668	F	F3	Venezuela	5	337-1510	1174
KF414669	F	F3	Venezuela	5	145-1083	939
KF414671	F	F1b	Venezuela	5	98-1113	1016
KF414672	F	F2a	Venezuela	5	100-1113	1014

KF414673	F	F2a	Venezuela	5	107-1060	954
KF414675	F	F3	Venezuela	5	145-1113	969
KF414645	F	F3	Venezuela	6	424-761	338
KF414647	F	F2a	Venezuela	6	165-829	665
KF414648	F	F2a	Venezuela	6	167-814	648
KF414649	F	F2a	Venezuela	6	131-844	714
KF414650	F	F2a	Venezuela	6	131-844	714
KF414651	F	F2a	Venezuela	6	168-843	676
KF414654	F	F3	Venezuela	6	134-844	711
KF414655	F	F1b	Venezuela	6	134-838	705
KF414656	F	F1b	Venezuela	6	134-846	713
KF414657	F	F1b	Venezuela	6	130-846	717
KF414658	F	F1b	Venezuela	6	134-842	709
KF414662	F	F3	Venezuela	6	131-846	716
KF414670	F	F3	Venezuela	6	131-840	710
KF414674	F	F3	Venezuela	6	164-847	684
KF414676	F	F3	Venezuela	6	132-846	715
KF414677	F	F1b	Venezuela	6	130-846	717
KF414679	G	G	Venezuela	7	Complete Genome	3248

[1]Alignment to complete genome reference sequence VHB NC_003977.2

PERU

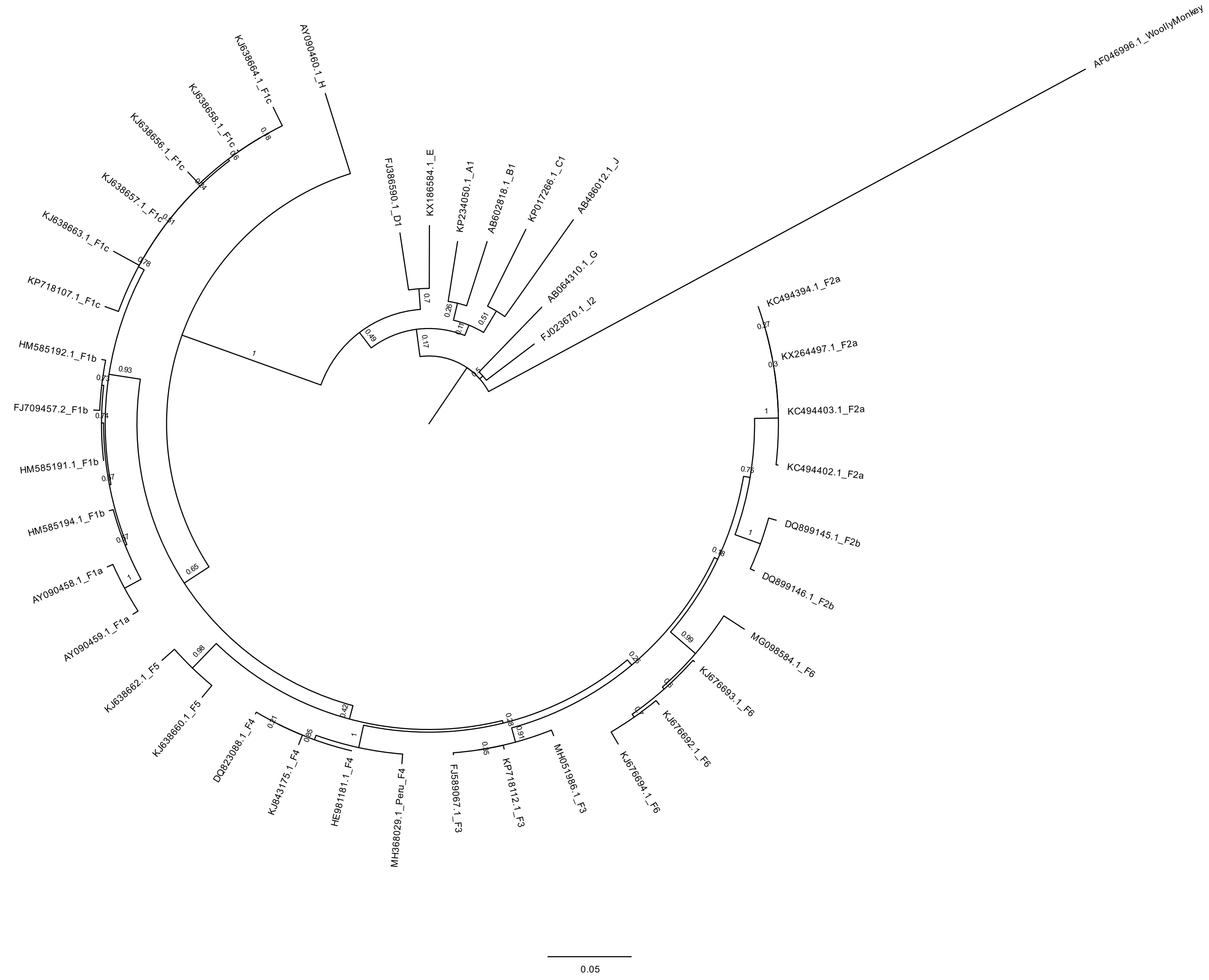

Tree 1. The evolutionary history was inferred by using the Maximum Likelihood method and Tamura-Nei model. The percentage of replicate trees in which the associated taxa clustered together in the bootstrap test (1000 replicates) are shown next to the branches. Initial tree(s) for the heuristic search were obtained automatically by applying Neighbor-Join and BioNJ algorithms to a matrix of pairwise distances estimated using the Tamura-Nei model, and then selecting the topology with superior log likelihood value. A discrete Gamma distribution was used to model evolutionary rate differences among sites (5 categories (+G, parameter = 0.1938)). The tree is drawn to scale, with branch lengths measured in the number of substitutions per site. The analysis involved 41 nucleotide sequences, of which 40 were used as marker sequences to determine the genotype of 1 sequence. All positions containing gaps and missing data were eliminated. There was a total of 961 positions in the final dataset. Evolutionary analyses were conducted in MEGA X.

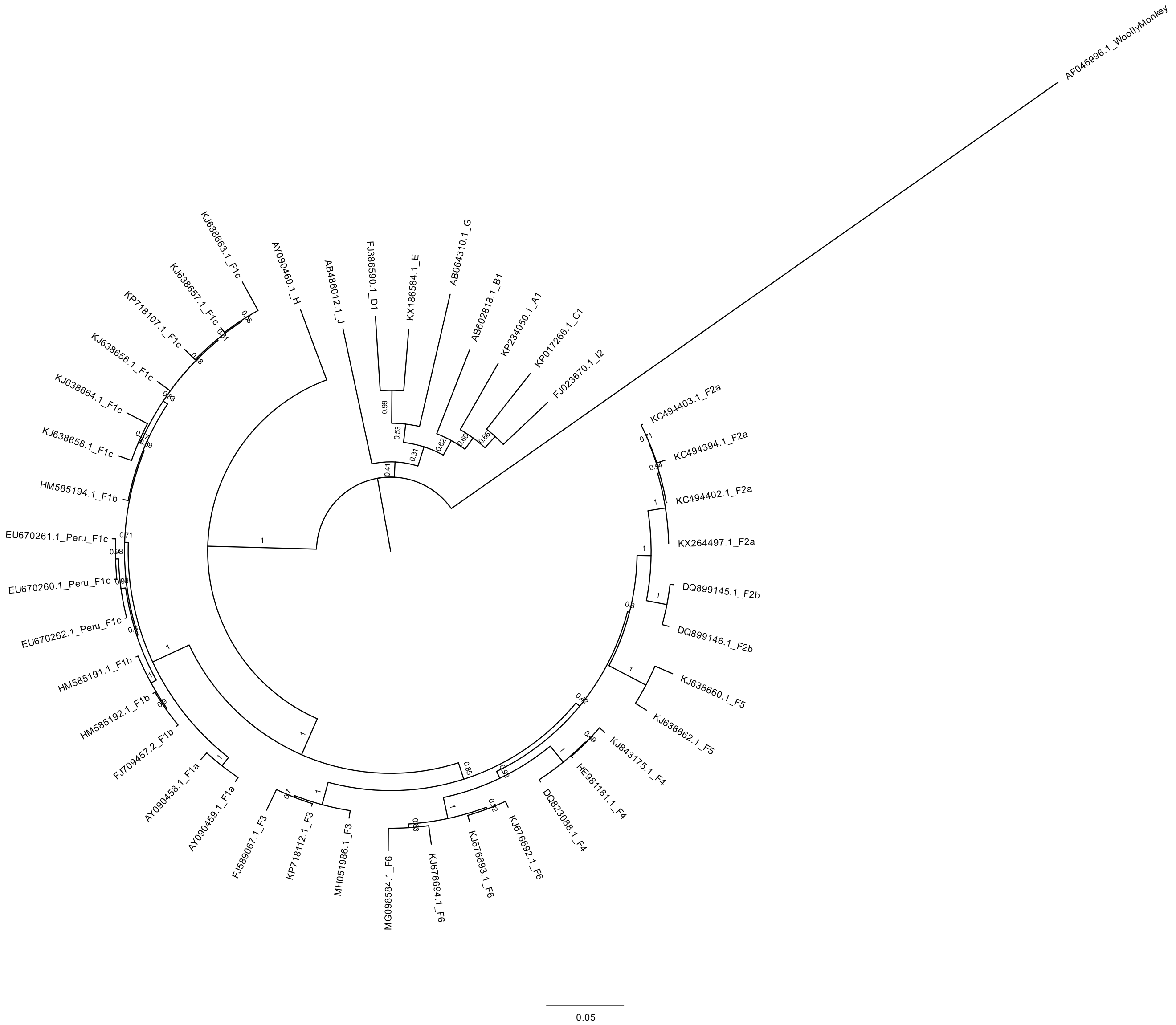

Tree 2. The evolutionary history was inferred by using the Maximum Likelihood method and Tamura-Nei model. The percentage of replicate trees in which the associated taxa clustered together in the bootstrap test (1000 replicates) are shown next to the branches. Initial tree(s) for the heuristic search were obtained automatically by applying Neighbor-Join and BioNJ algorithms to a matrix of pairwise distances estimated using the Tamura-Nei model, and then selecting the topology with superior log likelihood value. A discrete Gamma distribution was used to model evolutionary rate differences among sites (5 categories (+G, parameter = 0.2542)). The tree is drawn to scale, with branch lengths measured in the number of substitutions per site. The analysis involved 43 nucleotide sequences, of which 40 were used as marker sequences to determine the genotype of 3 sequences. The three sequences analyzed were reclassified as subgenotype F1b. All positions containing gaps and missing data were eliminated. There was a total of 2873 positions in the final dataset. Evolutionary analyses were conducted in MEGA X.

ID	GENOTYPE	SUBTYPE	COUNTRY	TREE	ALIGNMENT[1]	BASE PAIRS
MH368029	F	F4	Peru	1	207-1187	981
EU670260	F	F1b	Peru	2	Complete Genome	3034
EU670261	F	F1b	Peru	2	Complete Genome	3135

EU670262	F	F1b	Peru	2	Complete Genome	3215

[1]Alignment to complete genome reference sequence VHB NC_003977.2

COLOMBIA

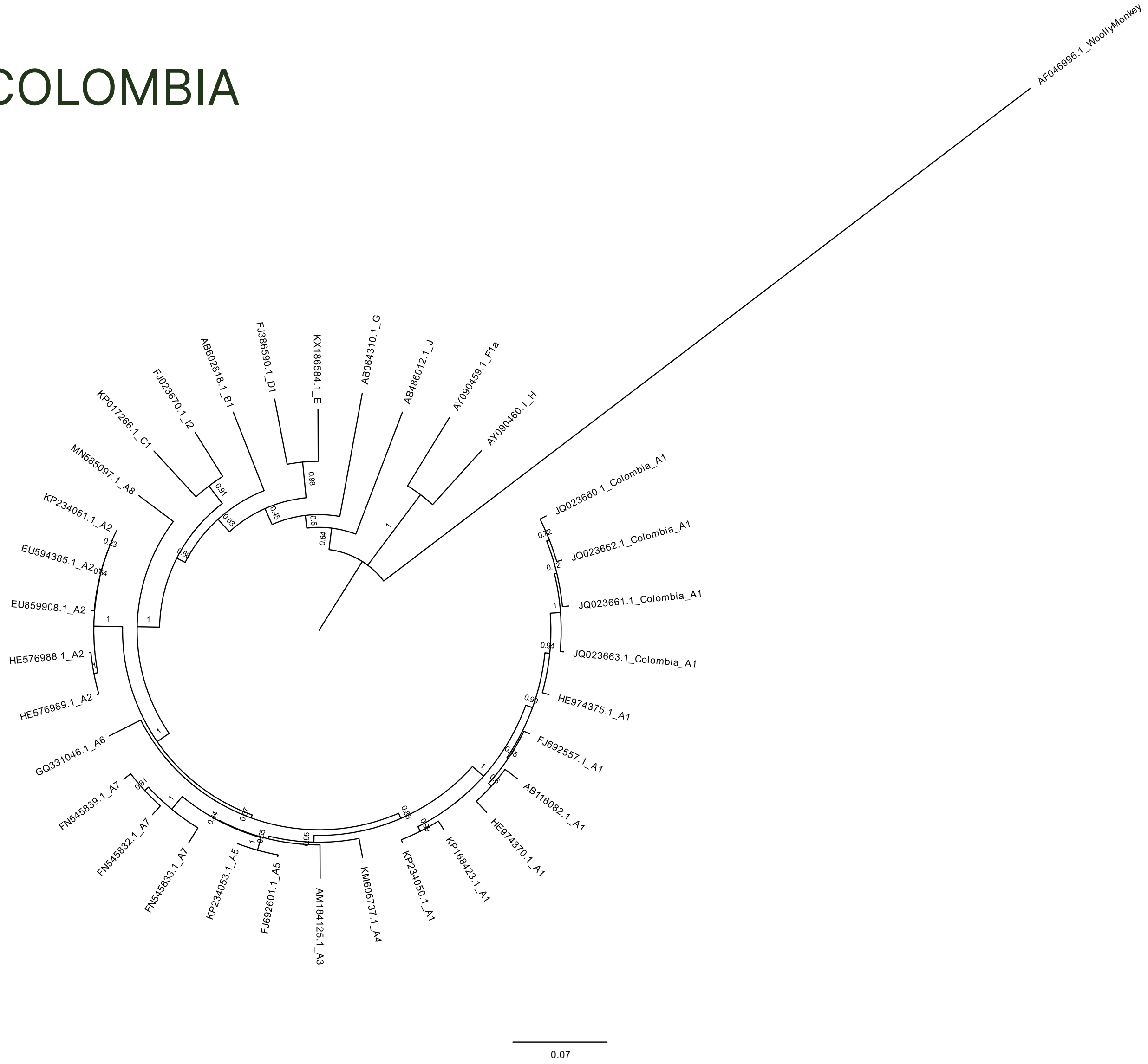

Tree 1. The evolutionary history was inferred by using the Maximum Likelihood method and Tamura-Nei model. The percentage of replicate trees in which the associated taxa clustered together in the bootstrap test (1000 replicates) are shown next to the branches. Initial tree(s) for the heuristic search were obtained automatically by applying Neighbor-Join and BioNJ algorithms to a matrix of pairwise distances estimated using the Tamura-Nei model, and then selecting the topology with superior log likelihood value. A discrete Gamma distribution was used to model evolutionary rate differences among sites (5 categories (+G, parameter = 0.2253)). The tree is drawn to scale, with branch lengths measured in the number of substitutions per site. The analysis involved 34 nucleotide sequences, of which 30 were used as marker sequences to determine the genotype of 4 sequences. All positions containing gaps and missing data were eliminated. There was a total of 3031 positions in the final dataset. Evolutionary analyses were conducted in MEGA X.

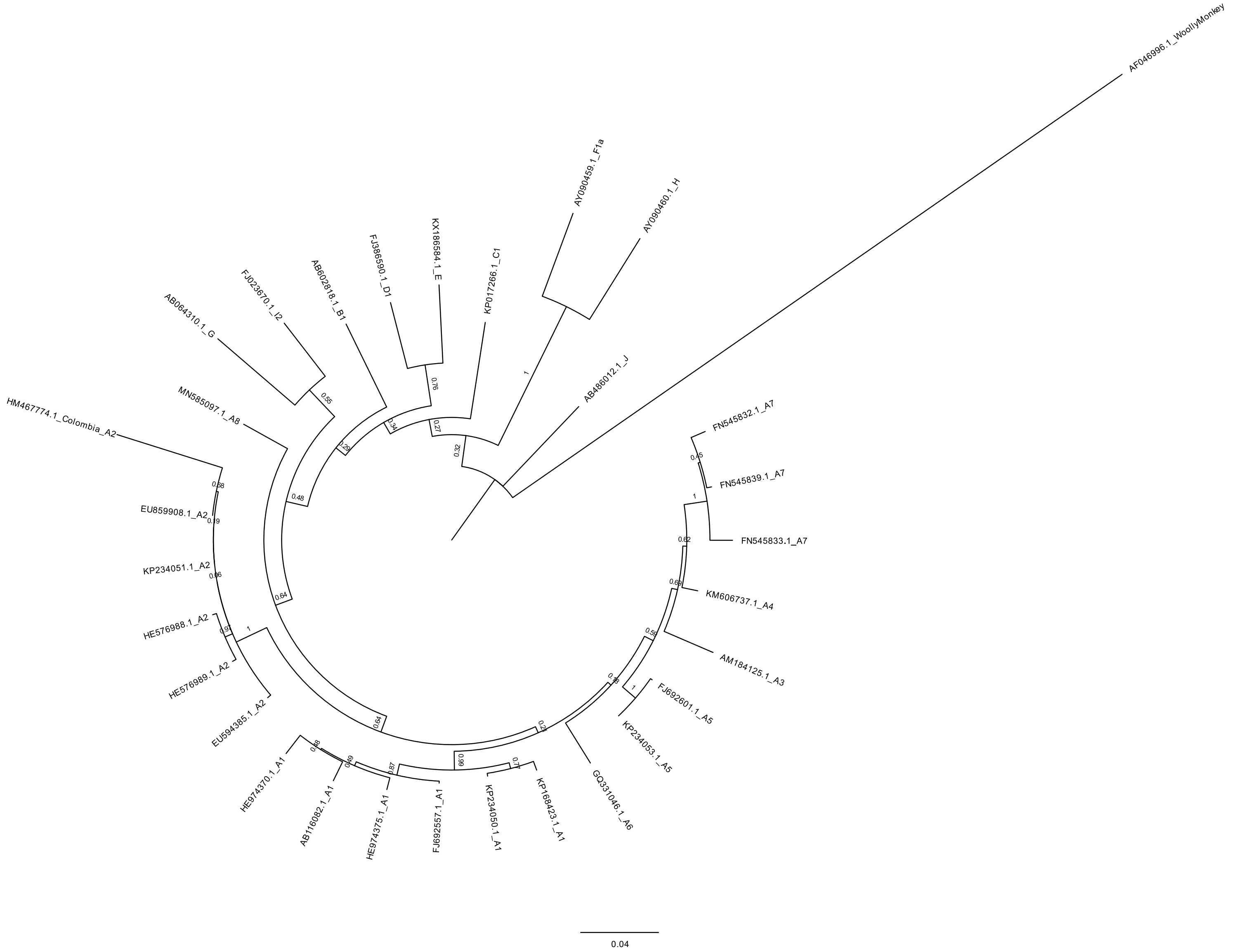

Tree 2. The evolutionary history was inferred by using the Maximum Likelihood method and Tamura-Nei model. The percentage of replicate trees in which the associated taxa clustered together in the bootstrap test (1000 replicates) are shown next to the branches. Initial tree(s) for the heuristic search were obtained automatically by applying Neighbor-Join and BioNJ algorithms to a matrix of pairwise distances estimated using the Tamura-Nei model, and then selecting the topology with superior log likelihood value. A discrete Gamma distribution was used to model evolutionary rate differences among sites (5 categories (+G, parameter = 0.2228)). The tree is drawn to scale, with branch lengths measured in the number of substitutions per site. The analysis involved 31 nucleotide sequences, of which 30 were used as marker sequences to determine the genotype of 1 sequence. All positions containing gaps and missing data were eliminated. There was a total of 1118 positions in the final dataset. Evolutionary analyses were conducted in MEGA X.

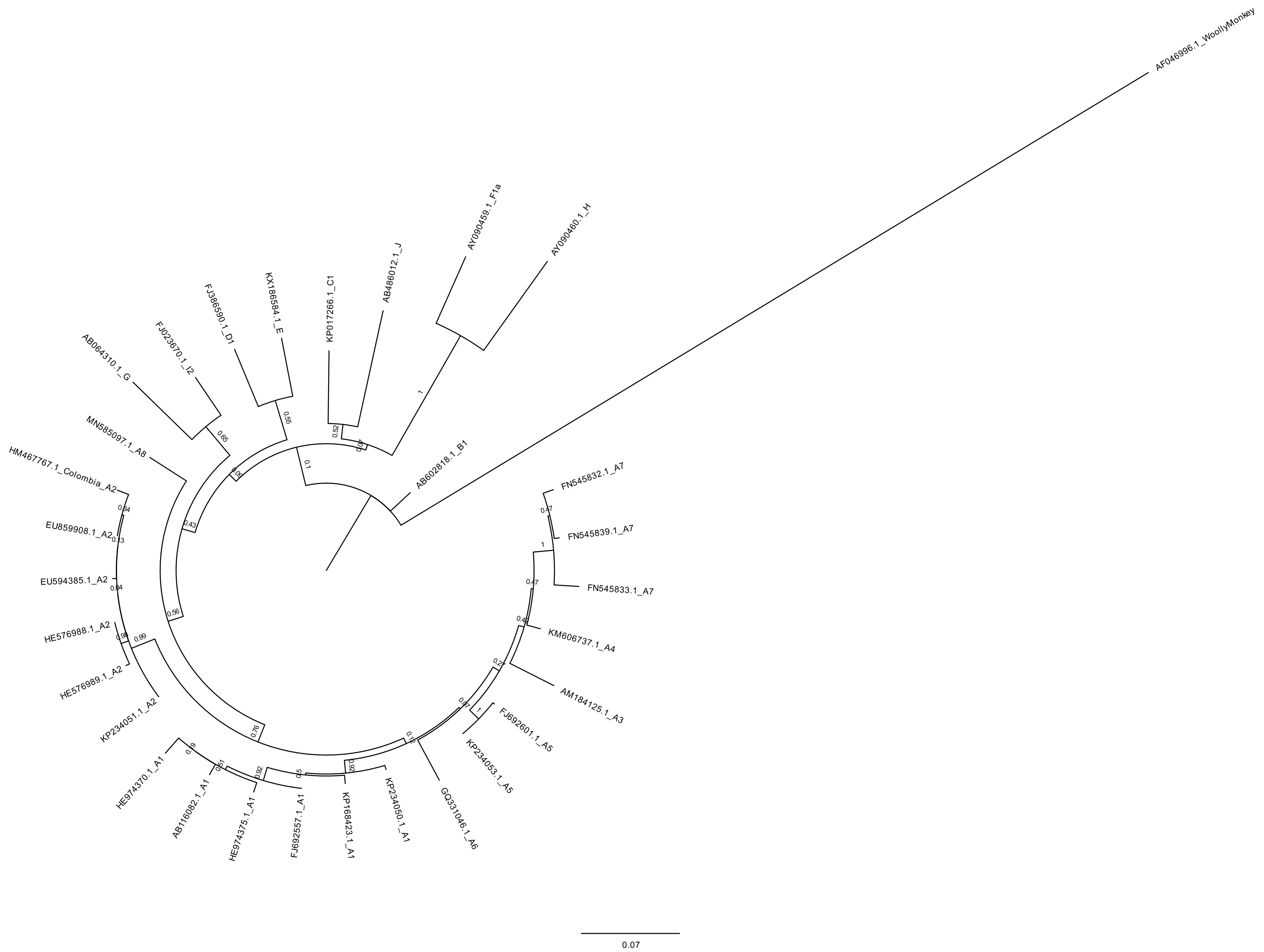

Tree 3. The evolutionary history was inferred by using the Maximum Likelihood method and Tamura-Nei model. The percentage of replicate trees in which the associated taxa clustered together in the bootstrap test (1000 replicates) are shown next to the branches. Initial tree(s) for the heuristic search were obtained automatically by applying Neighbor-Join and BioNJ algorithms to a matrix of pairwise distances estimated using the Tamura-Nei model, and then selecting the topology with superior log likelihood value. A discrete Gamma distribution was used to model evolutionary rate differences among sites (5 categories (+G, parameter = 0.1767)). The tree is drawn to scale, with branch lengths measured in the number of substitutions per site. The analysis involved 31 nucleotide sequences, of which 30 were used as marker sequences to determine the genotype of 1 sequence. All positions containing gaps and missing data were eliminated. There was a total of 900 positions in the final dataset. Evolutionary analyses were conducted in MEGA X.

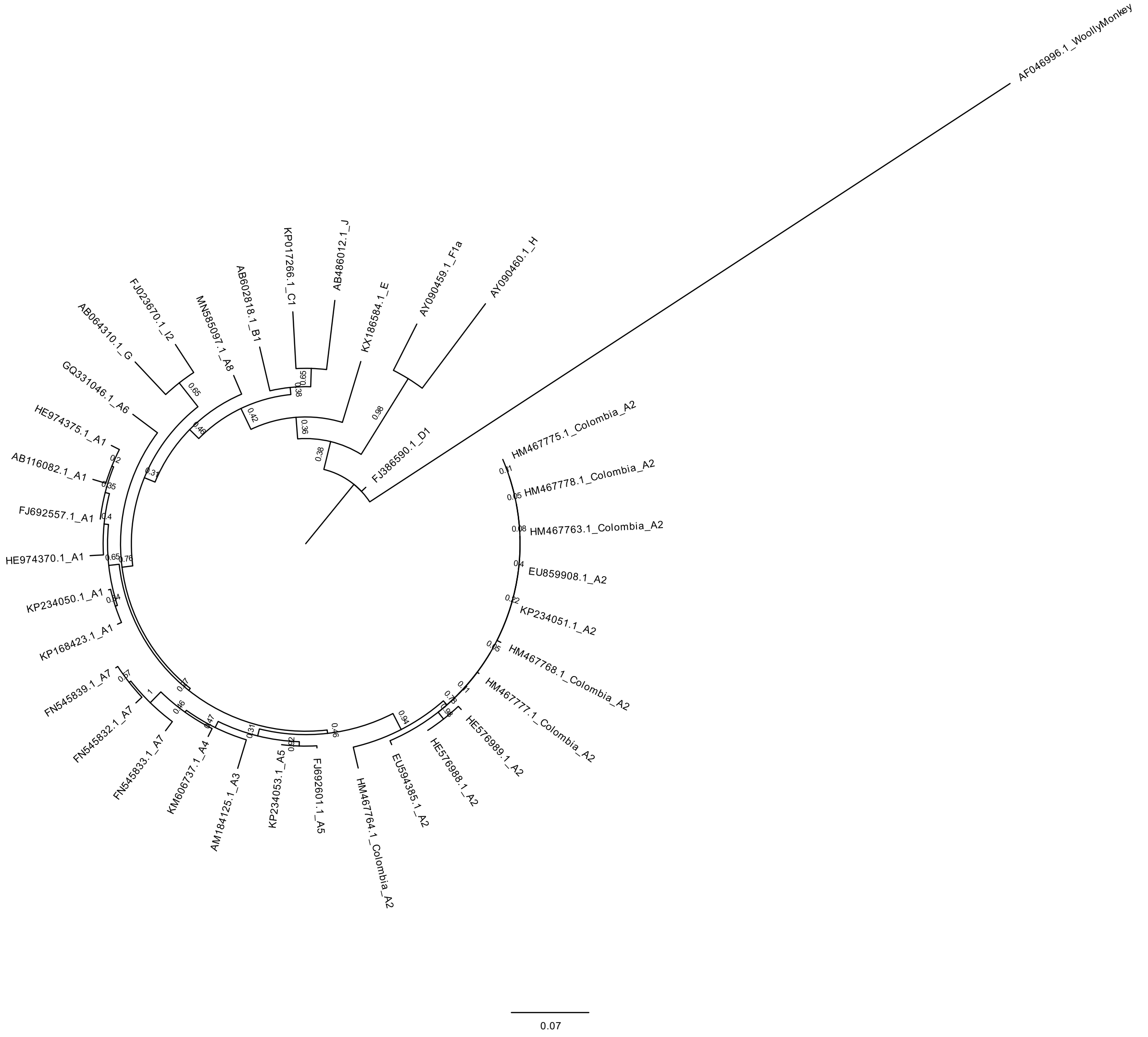

Tree 4. The evolutionary history was inferred by using the Maximum Likelihood method and Tamura-Nei model. The percentage of replicate trees in which the associated taxa clustered together in the bootstrap test (1000 replicates) are shown next to the branches. Initial tree(s) for the heuristic search were obtained automatically by applying Neighbor-Join and BioNJ algorithms to a matrix of pairwise distances estimated using the Tamura-Nei model, and then selecting the topology with superior log likelihood value. A discrete Gamma distribution was used to model evolutionary rate differences among sites (5 categories (+G, parameter = 0.1546)). The tree is drawn to scale, with branch lengths measured in the number of substitutions per site. The analysis involved 36 nucleotide sequences, of which 30 were used as marker sequences to determine the genotype of 6 sequences. All positions containing gaps and missing data were eliminated. There was a total of 659 positions in the final dataset. Evolutionary analyses were conducted in MEGA X.

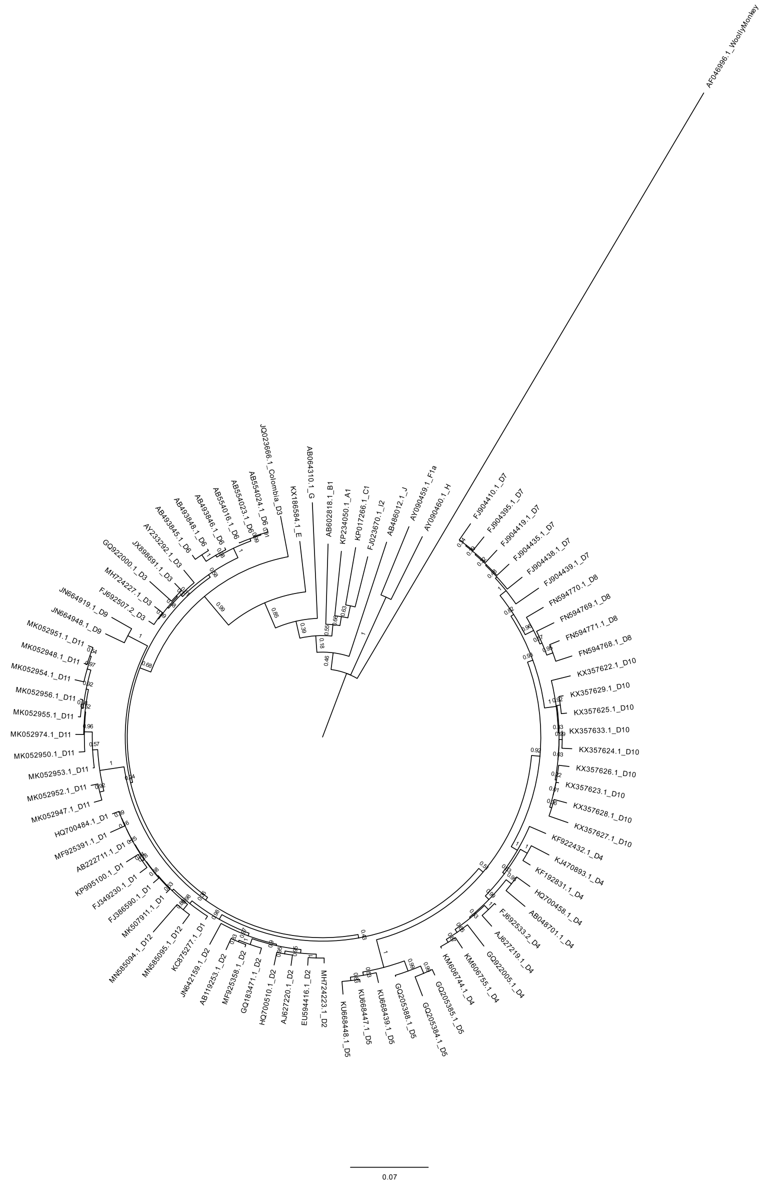

Tree 5. The evolutionary history was inferred by using the Maximum Likelihood method and Tamura-Nei model. The percentage of replicate trees in which the associated taxa clustered together in the bootstrap test (1000 replicates) are shown next to the branches. Initial tree(s) for the heuristic search were obtained automatically by applying Neighbor-Join and BioNJ algorithms to a matrix of pairwise distances estimated using the Tamura-Nei model, and then selecting the topology with superior log likelihood value. A discrete Gamma distribution was used to model evolutionary rate differences among sites (5 categories (+G, parameter = 0.2762)). The tree is drawn to scale, with branch lengths measured in the number of substitutions per site. The analysis involved 87 nucleotide sequences, of which 86 were used as marker sequences to determine the genotype of 1 sequence. After an additional phylogenetic tree was run, it was concluded that its subgenotype would remain as D3. All positions containing gaps and missing data were eliminated. There was a total of 3088 positions in the final dataset. Evolutionary analyses were conducted in MEGA X.

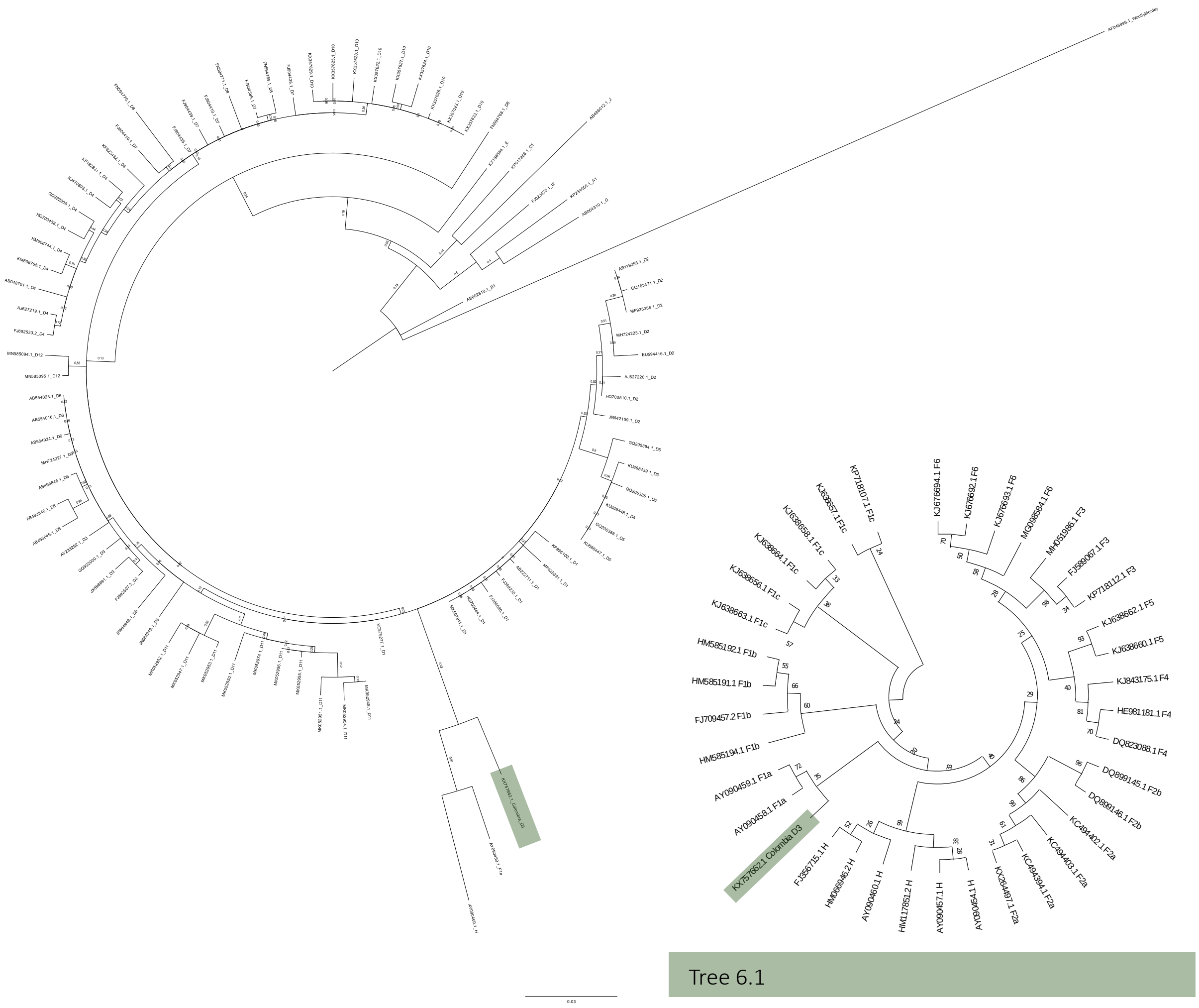

Tree 6. The evolutionary history was inferred by using the Maximum Likelihood method and Tamura-Nei model. The percentage of replicate trees in which the associated taxa clustered together in the bootstrap test (1000 replicates) are shown next to the branches. Initial tree(s) for the heuristic search were obtained automatically by applying Neighbor-Join and BioNJ algorithms to a matrix of pairwise distances estimated using the Tamura-Nei model, and then selecting the topology with superior log likelihood value. A discrete Gamma distribution was used to model evolutionary rate differences among sites (5 categories (+G, parameter = 0.2183)). The tree is drawn to scale, with branch lengths measured in the number of substitutions per site. The analysis involved 87 nucleotide sequences, of which 86 were used as marker sequences to determine the genotype of 1 sequence. Sequence KX757662 was reported as genotype D3, but upon further inspection (Tree 6.1) we reclassified it as F1a. All positions containing gaps and missing data were eliminated. There was a total of 618 positions in the final dataset. Evolutionary analyses were conducted in MEGA X.

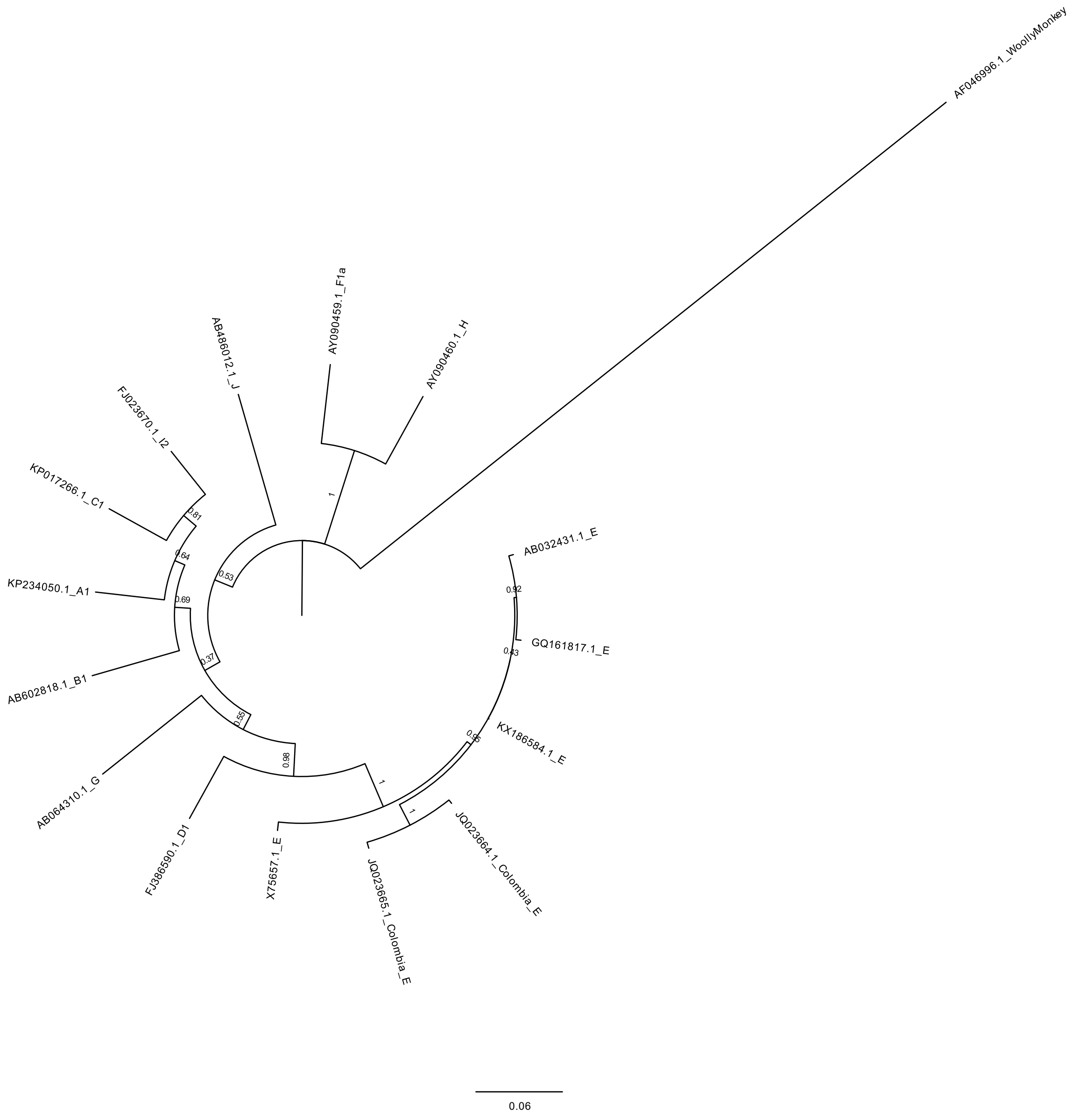

Tree 7. The evolutionary history was inferred by using the Maximum Likelihood method and Tamura-Nei model. The percentage of replicate trees in which the associated taxa clustered together in the bootstrap test (1000 replicates) are shown next to the branches. Initial tree(s) for the heuristic search were obtained automatically by applying Neighbor-Join and BioNJ algorithms to a matrix of pairwise distances estimated using the Tamura-Nei model, and then selecting the topology with superior log likelihood value. A discrete Gamma distribution was used to model evolutionary rate differences among sites (5 categories (+G, parameter = 0.2453)). The tree is drawn to scale, with branch lengths measured in the number of substitutions per site. The analysis involved 16 nucleotide sequences, of which 14 were used as marker sequences to determine the genotype of 2 sequences. All positions containing gaps and missing data were eliminated. There was a total of 3170 positions in the final dataset. Evolutionary analyses were conducted in MEGA X.

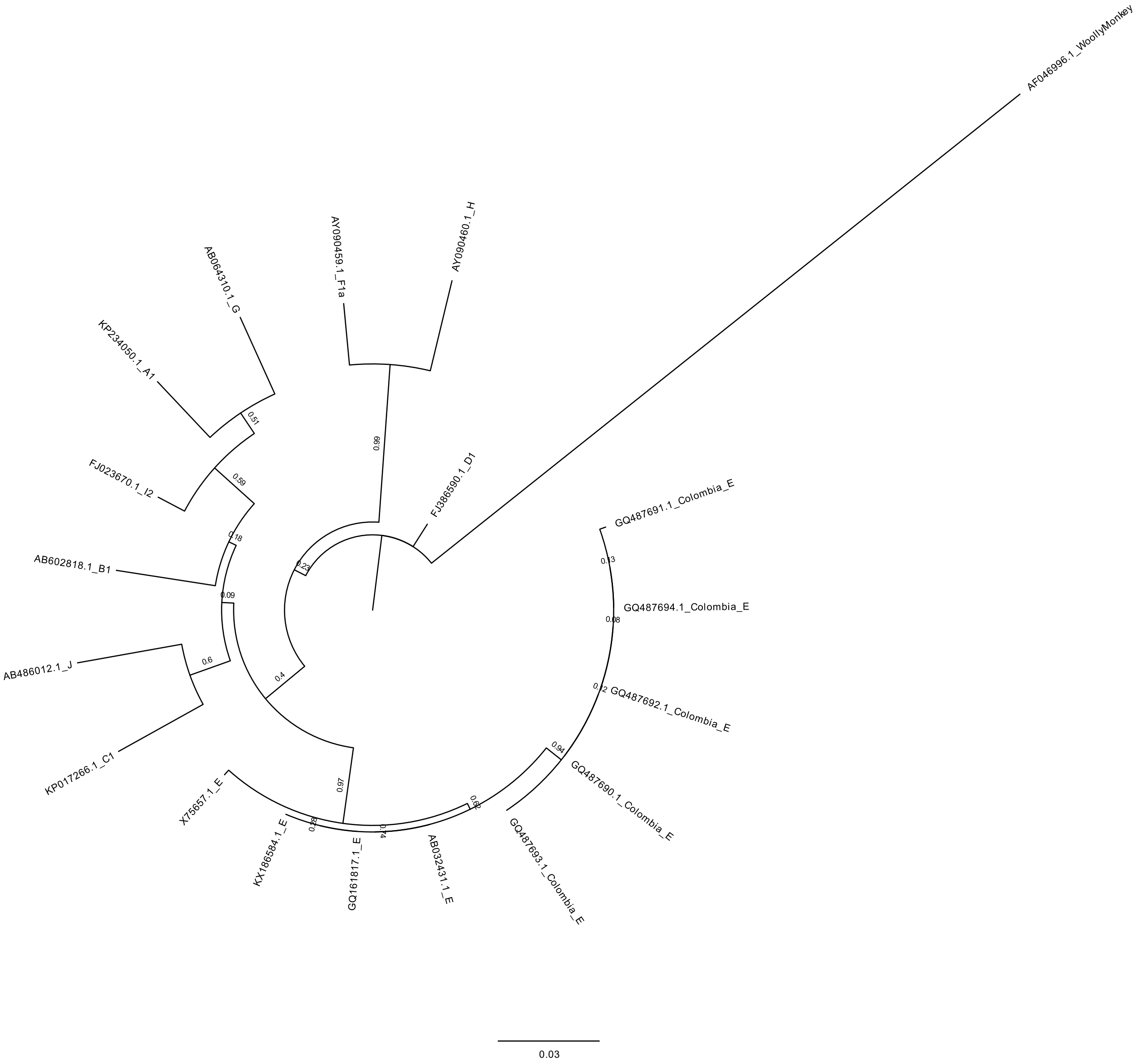

Tree 8. The evolutionary history was inferred by using the Maximum Likelihood method and Tamura-Nei model. The percentage of replicate trees in which the associated taxa clustered together in the bootstrap test (1000 replicates) are shown next to the branches. Initial tree(s) for the heuristic search were obtained automatically by applying Neighbor-Join and BioNJ algorithms to a matrix of pairwise distances estimated using the Tamura-Nei model, and then selecting the topology with superior log likelihood value. A discrete Gamma distribution was used to model evolutionary rate differences among sites (5 categories (+G, parameter = 0.1320)). The tree is drawn to scale, with branch lengths measured in the number of substitutions per site. The analysis involved 19 nucleotide sequences, of which 14 were used as marker sequences to determine the genotype of 5 sequences. All positions containing gaps and missing data were eliminated. There was a total of 584 positions in the final dataset. Evolutionary analyses were conducted in MEGA X.

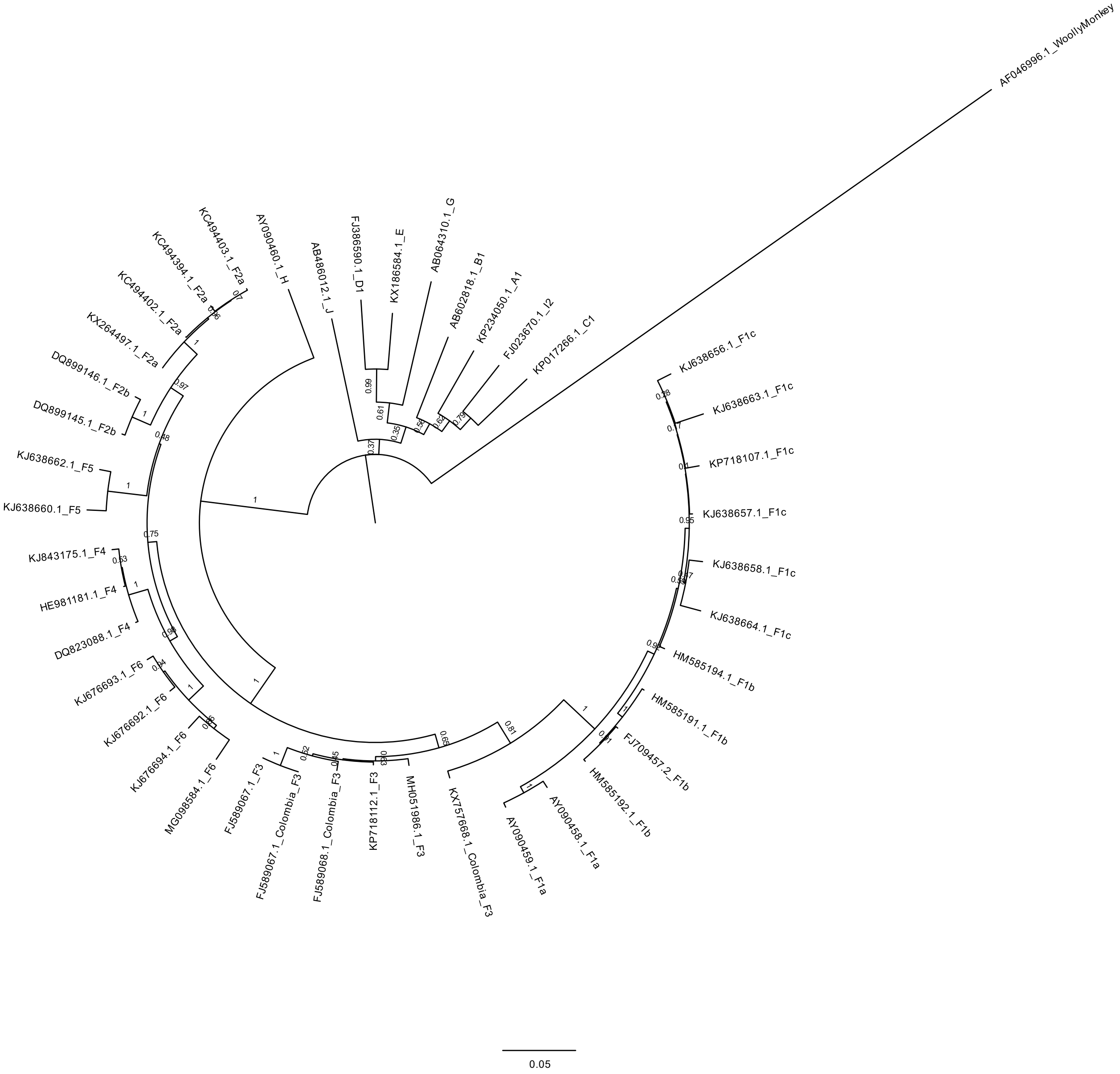

Tree 9. The evolutionary history was inferred by using the Maximum Likelihood method and Tamura-Nei model. The percentage of replicate trees in which the associated taxa clustered together in the bootstrap test (1000 replicates) are shown next to the branches. Initial tree(s) for the heuristic search were obtained automatically by applying Neighbor-Join and BioNJ algorithms to a matrix of pairwise distances estimated using the Tamura-Nei model, and then selecting the topology with superior log likelihood value. A discrete Gamma distribution was used to model evolutionary rate differences among sites (5 categories (+G, parameter = 0.2400)). The tree is drawn to scale, with branch lengths measured in the number of substitutions per site. The analysis involved 43 nucleotide sequences, of which 40 were used as marker sequences to determine the genotype of 3 sequences. All positions containing gaps and missing data were eliminated. There was a total of 2865 positions in the final dataset. Evolutionary analyses were conducted in MEGA X.

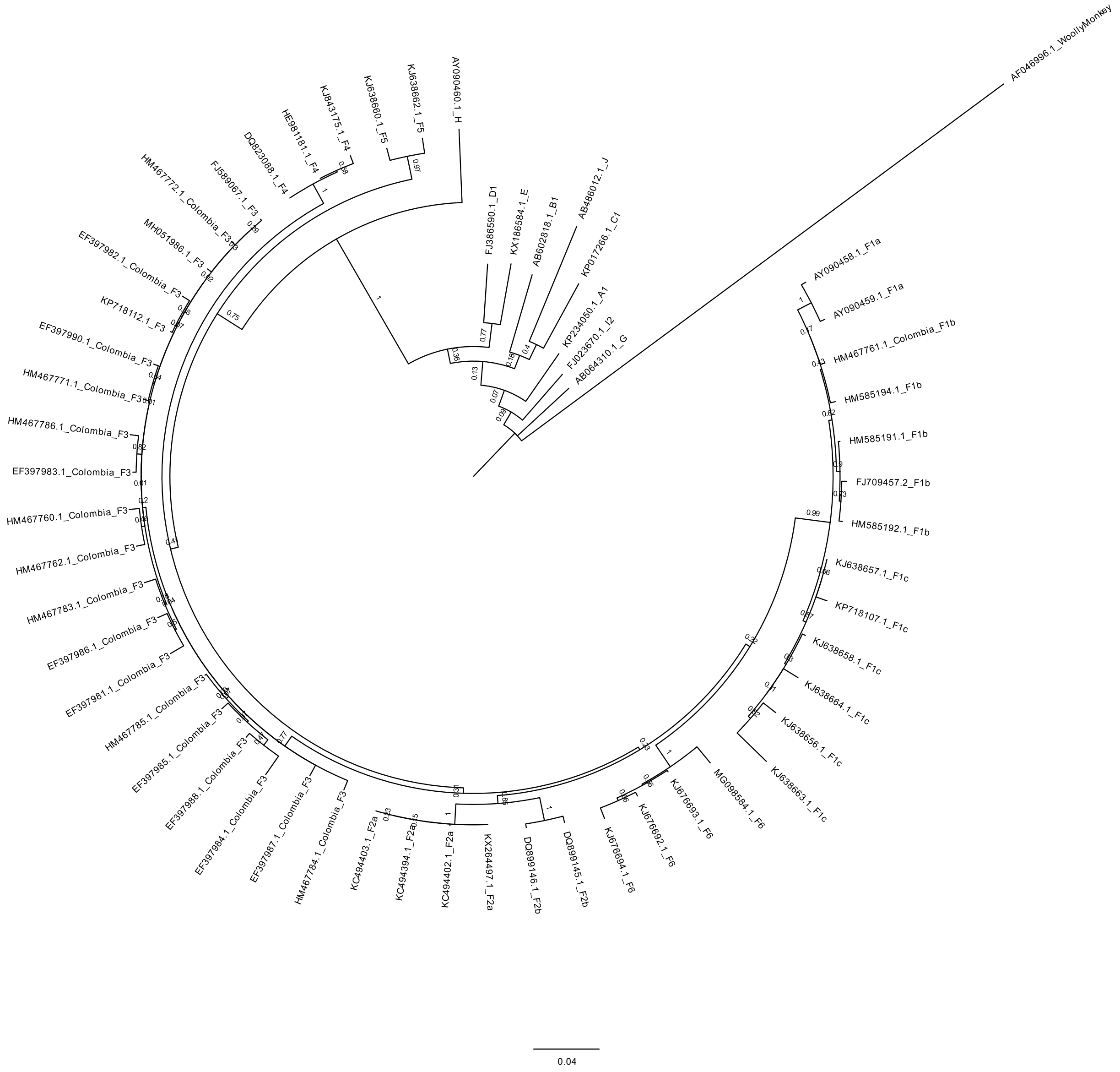

Tree 10. The evolutionary history was inferred by using the Maximum Likelihood method and Tamura-Nei model. The percentage of replicate trees in which the associated taxa clustered together in the bootstrap test (1000 replicates) are shown next to the branches. Initial tree(s) for the heuristic search were obtained automatically by applying Neighbor-Join and BioNJ algorithms to a matrix of pairwise distances estimated using the Tamura-Nei model, and then selecting the topology with superior log likelihood value. A discrete Gamma distribution was used to model evolutionary rate differences among sites (5 categories (+G, parameter = 0.2122)). The tree is drawn to scale, with branch lengths measured in the number of substitutions per site. The analysis involved 58 nucleotide sequences, of which 40 were used as marker sequences to determine the genotype of 18 sequences. All positions containing gaps and missing data were eliminated. There was a total of 1125 positions in the final dataset. Evolutionary analyses were conducted in MEGA X.

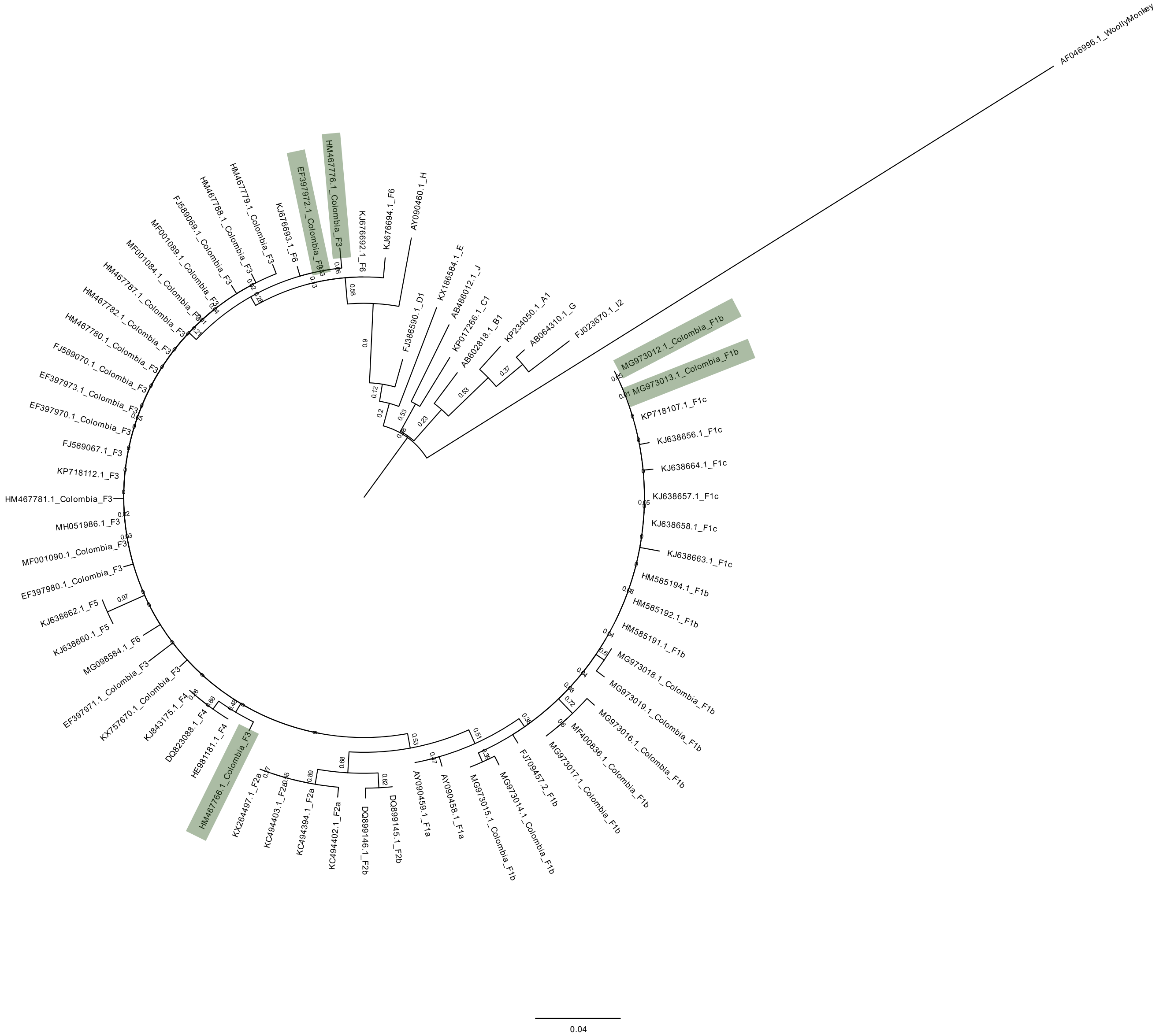

Tree 11. The evolutionary history was inferred by using the Maximum Likelihood method and Tamura-Nei model. The percentage of replicate trees in which the associated taxa clustered together in the bootstrap test (1000 replicates) are shown next to the branches. Initial tree(s) for the heuristic search were obtained automatically by applying Neighbor-Join and BioNJ algorithms to a matrix of pairwise distances estimated using the Tamura-Nei model, and then selecting the topology with superior log likelihood value. A discrete Gamma distribution was used to model evolutionary rate differences among sites (5 categories (+G, parameter = 0.2256)). The tree is drawn to scale, with branch lengths measured in the number of substitutions per site. The analysis involved 68 nucleotide sequences, of which 40 were used as marker sequences to determine the genotype of 28 sequences. Seven sequences in the initial analysis showed an irregular position in the phylogenetic tree, and upon further analysis, five of these were reclassified into different subgenotypes. All positions containing gaps and missing data were eliminated. There was a total of 241 positions in the final dataset. Evolutionary analyses were conducted in MEGA X.

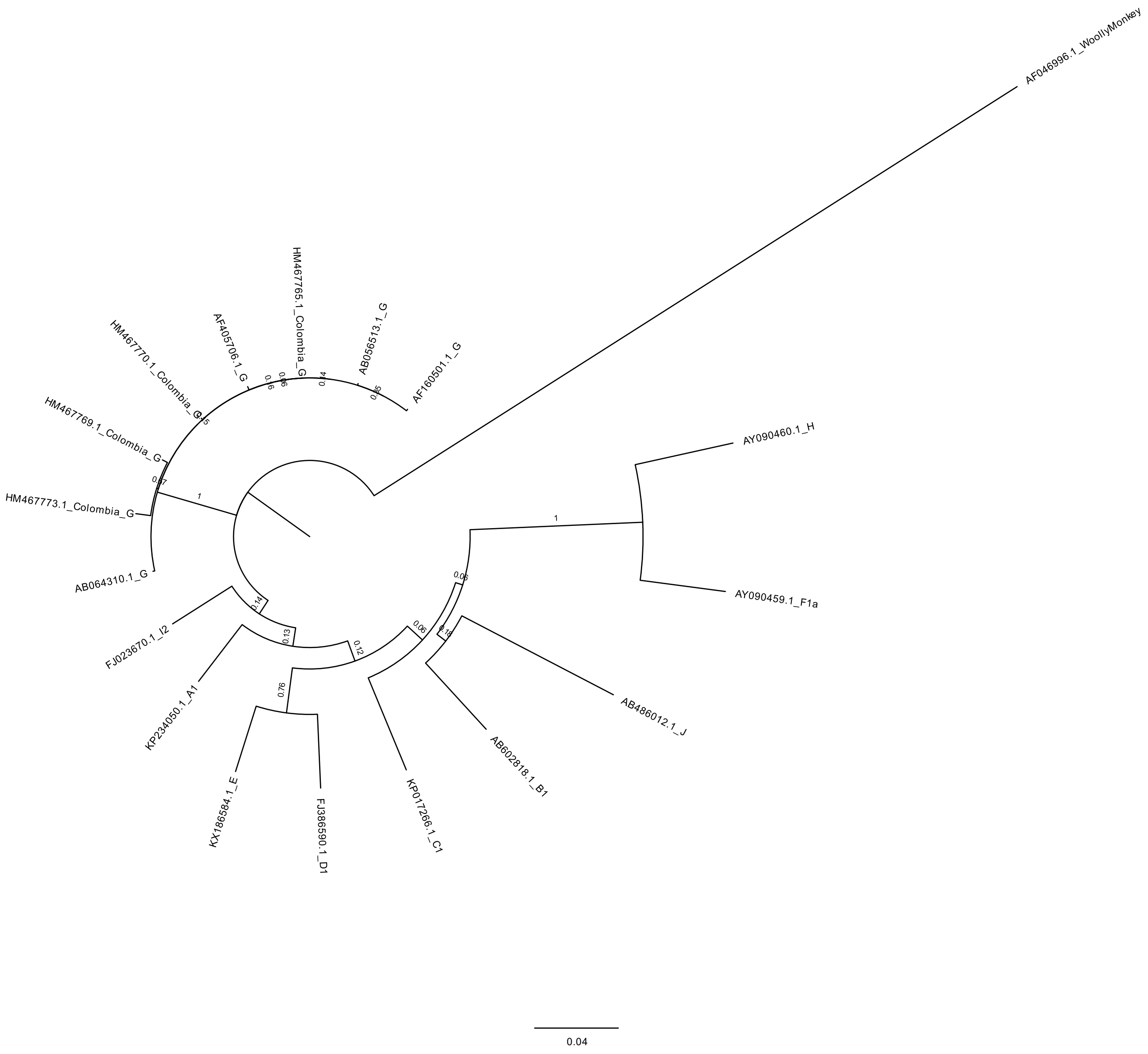

Tree 12. The evolutionary history was inferred by using the Maximum Likelihood method and Tamura-Nei model. The percentage of replicate trees in which the associated taxa clustered together in the bootstrap test (1000 replicates) are shown next to the branches. Initial tree(s) for the heuristic search were obtained automatically by applying Neighbor-Join and BioNJ algorithms to a matrix of pairwise distances estimated using the Tamura-Nei model, and then selecting the topology with superior log likelihood value. A discrete Gamma distribution was used to model evolutionary rate differences among sites (5 categories (+G, parameter = 0.1819)). The tree is drawn to scale, with branch lengths measured in the number of substitutions per site. The analysis involved 18 nucleotide sequences, of which 14 were used as marker sequences to determine the genotype of 4 sequences. All positions containing gaps and missing data were eliminated. There was a total of 1193 positions in the final dataset. Evolutionary analyses were conducted in MEGA X.

ID	GENOTYPE	SUBTYPE	COUNTRY	TREE	ALIGNMENT[1]	BASE PAIRS
JQ023660	A	A1	Colombia	1	Complete Genome	3221
JQ023661	A	A1	Colombia	1	Complete Genome	3221

JQ023662	A	A1	Colombia	1	Complete Genome	3221
JQ023663	A	A1	Colombia	1	Complete Genome	3221
HM467774	A	A2	Colombia	2	36-1242	1159
HM467767	A	A2	Colombia	3	315-1231	916
HM467763	A	A2	Colombia	4	301-992	629
HM467768	A	A2	Colombia	4	301-979	669
HM467764	A	A2	Colombia	4	311-979	679
HM467777	A	A2	Colombia	4	293-983	683
HM467778	A	A2	Colombia	4	300-982	686
HM467775	A	A2	Colombia	4	307-992	691
JQ023666	D	D3	Colombia	5	Complete Genome	3180
KX757662	F	F1a	Colombia	6	125-750	620
JQ023664	E	E	Colombia	7	Complete Genome	3211
JQ023665	E	E	Colombia	7	Complete Genome	3211
GQ487690	E	E	Colombia	8	252-835	584
GQ487691	E	E	Colombia	8	252-835	584
GQ487692	E	E	Colombia	8	252-835	584
GQ487693	E	E	Colombia	8	252-835	584
GQ487694	E	E	Colombia	8	252-835	584
FJ589068	F	F3	Colombia	9	Complete Genome	3131
KX757668	F	F3	Colombia	9	Complete Genome	3197
FJ589067	F	F3	Colombia	9	Complete Genome	3215
EF397981	F	F3	Colombia	10	95-1401	1307
EF397982	F	F3	Colombia	10	95-1915	1821
EF397983	F	F3	Colombia	10	95-1399	1305
EF397984	F	F3	Colombia	10	95-1408	1314
EF397985	F	F3	Colombia	10	95-1409	1315
EF397986	F	F3	Colombia	10	95-1409	1315
EF397987	F	F3	Colombia	10	3-1409	1407
EF397988	F	F3	Colombia	10	3-2376	2375
EF397990	F	F3	Colombia	10	3-1409	1407
HM467760	F	F3	Colombia	10	3-1271	1269
HM467761	F	F1b	Colombia	10	3-1274	1271
HM467762	F	F3	Colombia	10	3-1273	1271
HM467771	F	F3	Colombia	10	3-1280	1278
HM467772	F	F3	Colombia	10	3-1280	1278
HM467783	F	F3	Colombia	10	3-1241	1239
HM467784	F	F3	Colombia	10	3-1278	1276
HM467785	F	F3	Colombia	10	3-1272	1270
HM467786	F	F3	Colombia	10	3-1271	1269
EF397970	F	F3	Colombia	11	157-753	597

EF397971	F	F3	Colombia	11	157-753	597
EF397972	F	F6	Colombia	11	157-753	597
EF397973	F	F3	Colombia	11	157-753	597
EF397980	F	F3	Colombia	11	157-753	597
FJ589069	F	F3	Colombia	11	424-762	340
FJ589070	F	F3	Colombia	11	157-837	681
HM467766	F	F4	Colombia	11	308-992	685
HM467776	F	F6	Colombia	11	307-1008	702
HM467779	F	F3	Colombia	11	307-1006	700
HM467780	F	F3	Colombia	11	307-979	673
HM467781	F	F3	Colombia	11	307-979	673
HM467782	F	F3	Colombia	11	307-979	673
HM467787	F	F3	Colombia	11	340-979	640
HM467788	F	F3	Colombia	11	340-1241	902
KX757670	F	F3	Colombia	11	421-741	321
MF001084	F	F3	Colombia	11	224-768	545
MF001089	F	F3	Colombia	11	224-768	545
MF001090	F	F3	Colombia	11	224-768	545
MF400836	F	F1b	Colombia	11	463-754	292
MG973012	F	F1c	Colombia	11	463-789	327
MG973013	F	F1c	Colombia	11	463-789	327
MG973014	F	F1b	Colombia	11	463-789	327
MG973015	F	F1b	Colombia	11	463-789	327
MG973016	F	F1b	Colombia	11	463-789	327
MG973017	F	F1b	Colombia	11	432-709	278
MG973018	F	F1b	Colombia	11	463-789	327
MG973019	F	F1b	Colombia	11	463-789	327
HM467765	G	G	Colombia	12	3-1239	1237
HM467769	G	G	Colombia	12	44-1275	1232
HM467770	G	G	Colombia	12	3-1278	1276
HM467773	G	G	Colombia	12	40-1242	1203

[1]Alignment to complete genome reference sequence VHB NC_003977.2

BOLIVIA

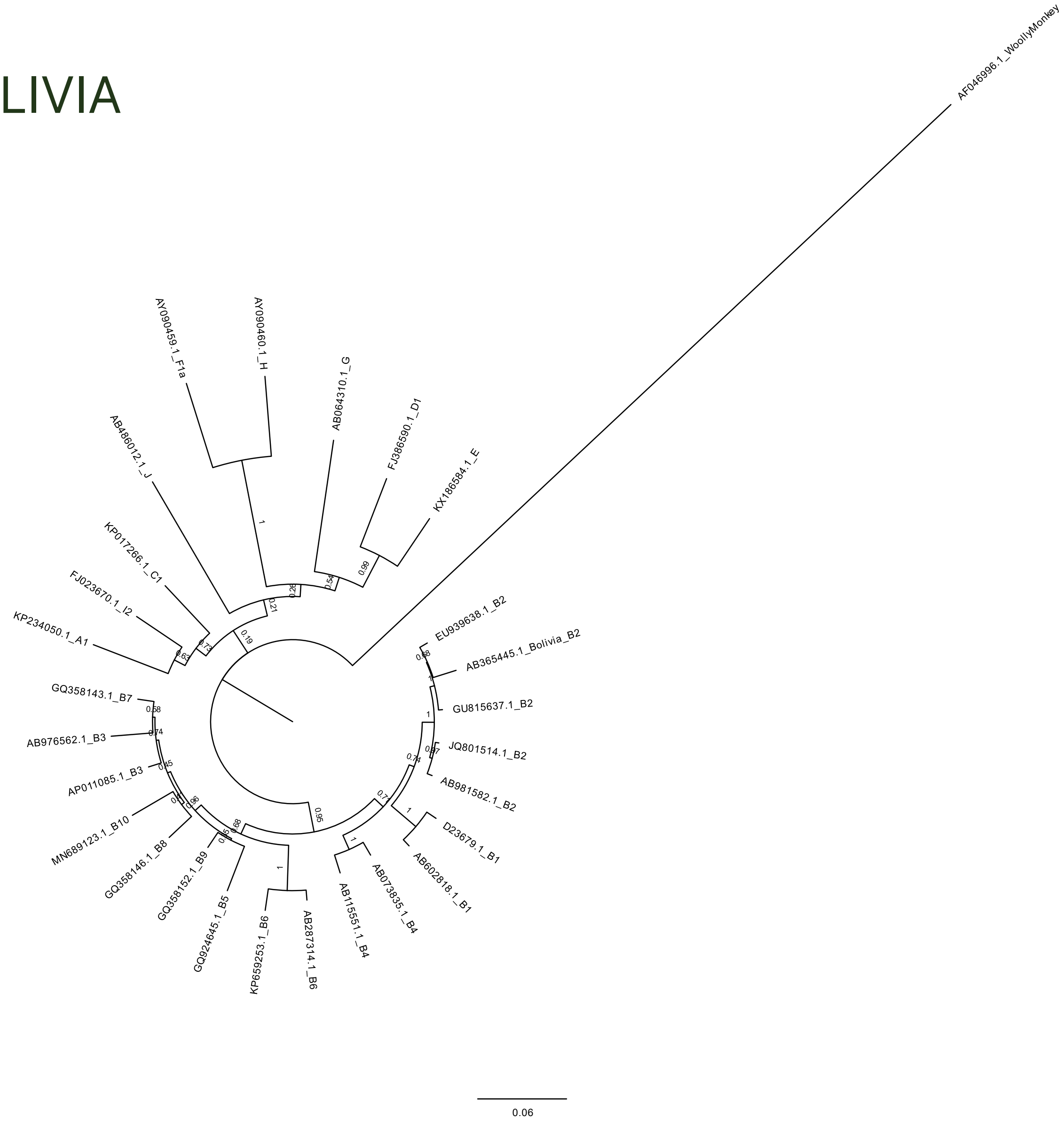

Tree 1. The evolutionary history was inferred by using the Maximum Likelihood method and Tamura-Nei model. The percentage of replicate trees in which the associated taxa clustered together in the bootstrap test (1000 replicates) are shown next to the branches. Initial tree(s) for the heuristic search were obtained automatically by applying Neighbor-Join and BioNJ algorithms to a matrix of pairwise distances estimated using the Tamura-Nei model, and then selecting the topology with superior log likelihood value. A discrete Gamma distribution was used to model evolutionary rate differences among sites (5 categories (+G, parameter = 0.2570)). The tree is drawn to scale, with branch lengths measured in the number of substitutions per site. The analysis involved 28 nucleotide sequences, of which 27 were used as marker sequences to determine the genotype of 1 sequence. All positions containing gaps and missing data were eliminated. There was a total of 3126 positions in the final dataset. Evolutionary analyses were conducted in MEGA X.

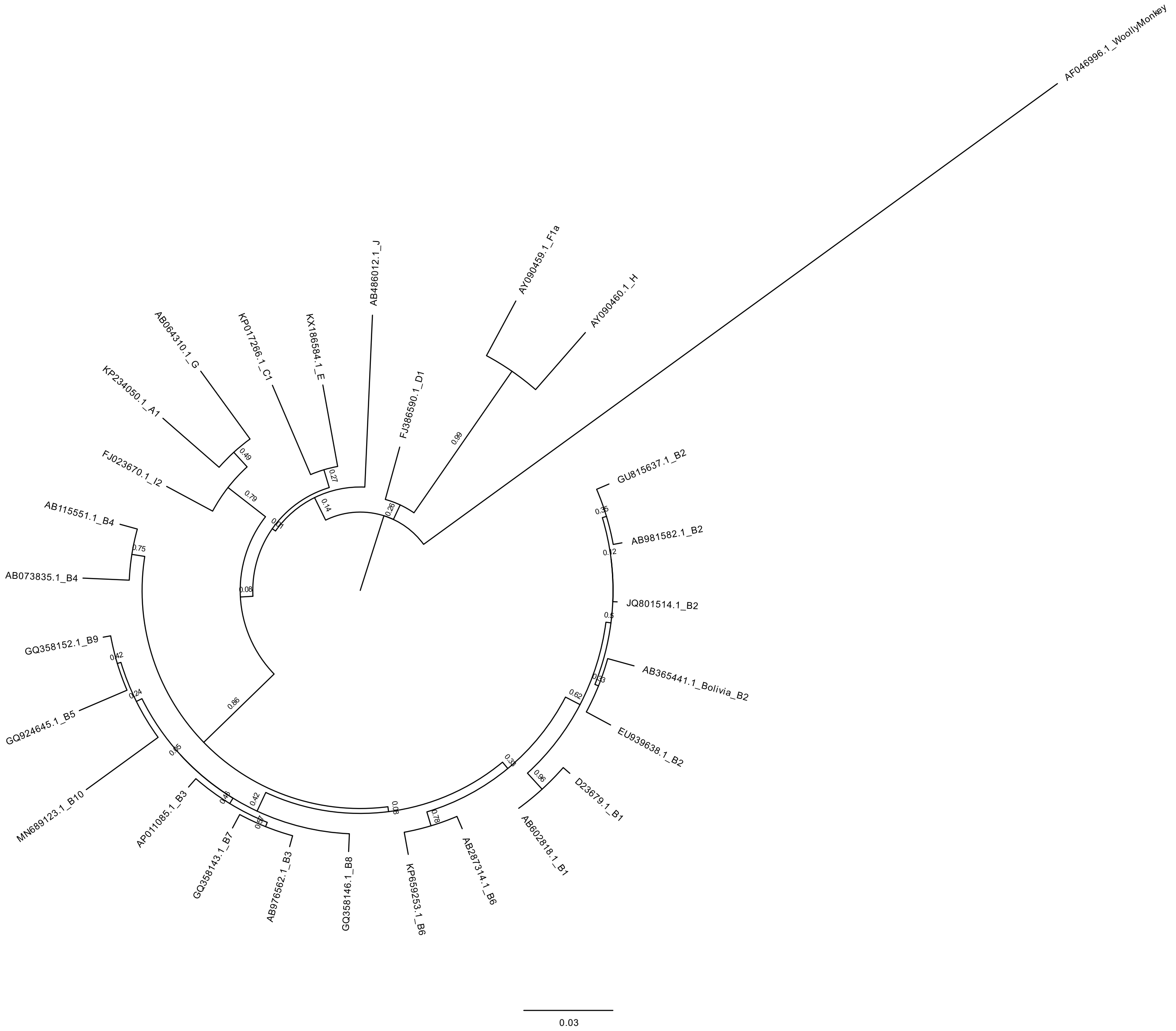

Tree 2. The evolutionary history was inferred by using the Maximum Likelihood method and Tamura-Nei model. The percentage of replicate trees in which the associated taxa clustered together in the bootstrap test (1000 replicates) are shown next to the branches. Initial tree(s) for the heuristic search were obtained automatically by applying Neighbor-Join and BioNJ algorithms to a matrix of pairwise distances estimated using the Tamura-Nei model, and then selecting the topology with superior log likelihood value. A discrete Gamma distribution was used to model evolutionary rate differences among sites (5 categories (+G, parameter = 0.1733)). The tree is drawn to scale, with branch lengths measured in the number of substitutions per site. The analysis involved 28 nucleotide sequences, of which 27 were used as marker sequences to determine the genotype of 1 sequence. All positions containing gaps and missing data were eliminated. There was a total of 746 positions in the final dataset. Evolutionary analyses were conducted in MEGA X.

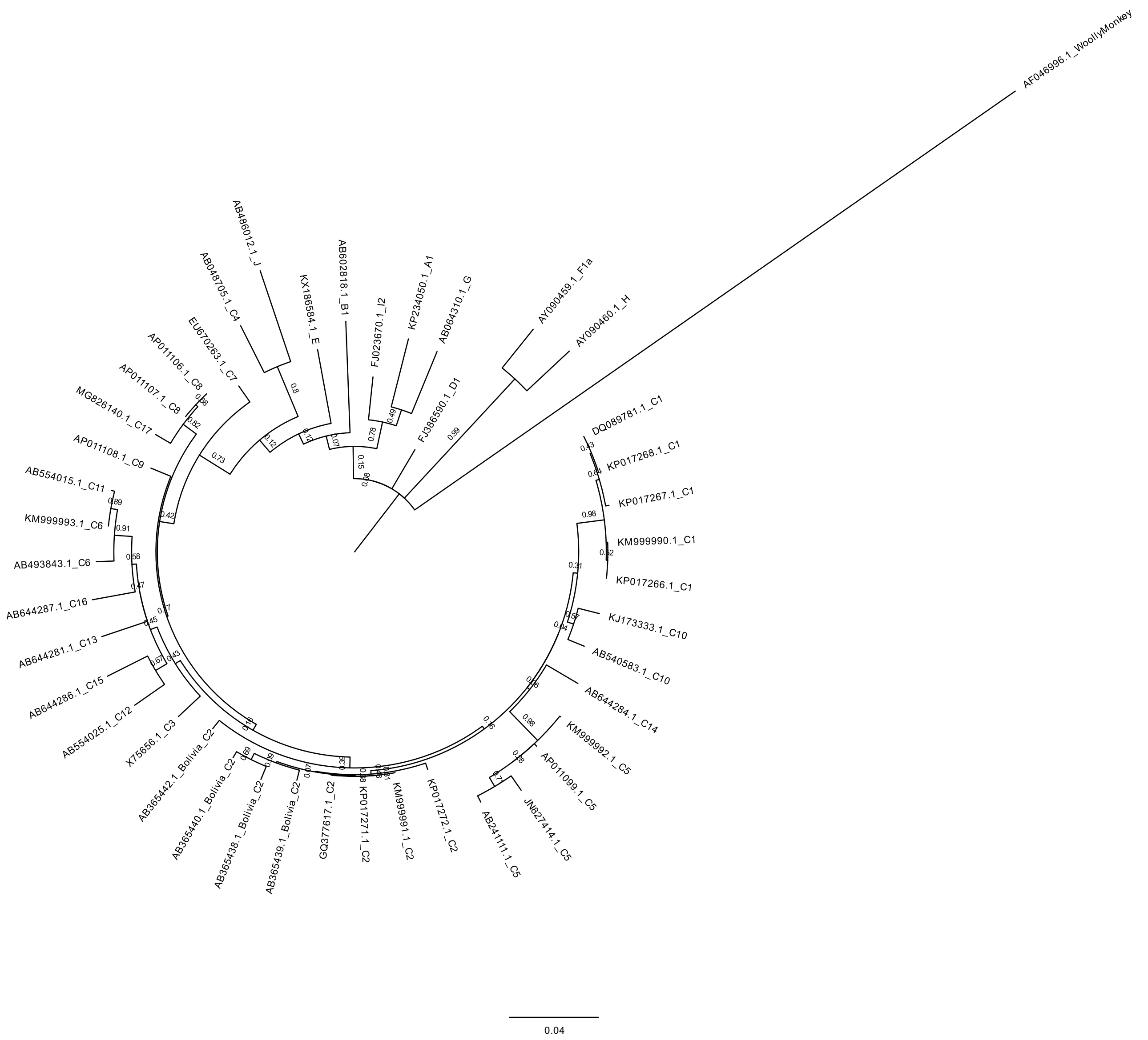

Tree 3. The evolutionary history was inferred by using the Maximum Likelihood method and Tamura-Nei model. The percentage of replicate trees in which the associated taxa clustered together in the bootstrap test (1000 replicates) are shown next to the branches. Initial tree(s) for the heuristic search were obtained automatically by applying Neighbor-Join and BioNJ algorithms to a matrix of pairwise distances estimated using the Tamura-Nei model, and then selecting the topology with superior log likelihood value. A discrete Gamma distribution was used to model evolutionary rate differences among sites (5 categories (+G, parameter = 0.1555)). The tree is drawn to scale, with branch lengths measured in the number of substitutions per site. The analysis involved 44 nucleotide sequences, of which 40 were used as marker sequences to determine the genotype of 4 sequences. All positions containing gaps and missing data were eliminated. There was a total of 759 positions in the final dataset. Evolutionary analyses were conducted in MEGA X.

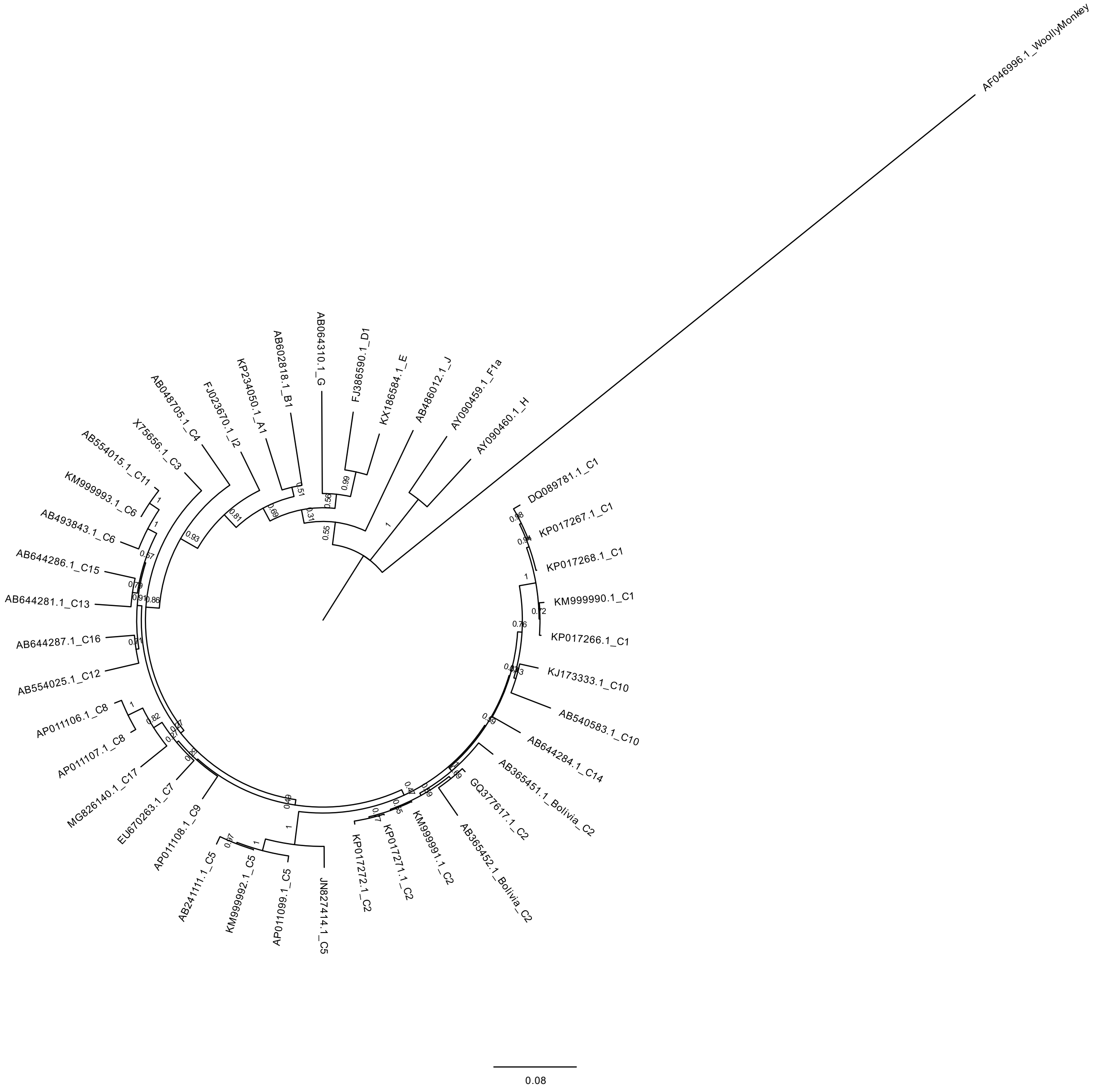

Tree 4. The evolutionary history was inferred by using the Maximum Likelihood method and Tamura-Nei model. The percentage of replicate trees in which the associated taxa clustered together in the bootstrap test (1000 replicates) are shown next to the branches. Initial tree(s) for the heuristic search were obtained automatically by applying Neighbor-Join and BioNJ algorithms to a matrix of pairwise distances estimated using the Tamura-Nei model, and then selecting the topology with superior log likelihood value. A discrete Gamma distribution was used to model evolutionary rate differences among sites (5 categories (+G, parameter = 0.2266)). The tree is drawn to scale, with branch lengths measured in the number of substitutions per site. The analysis involved 42 nucleotide sequences, of which 40 were used as marker sequences to determine the genotype of 2 sequences. All positions containing gaps and missing data were eliminated. There was a total of 3138 positions in the final dataset. Evolutionary analyses were conducted in MEGA X.

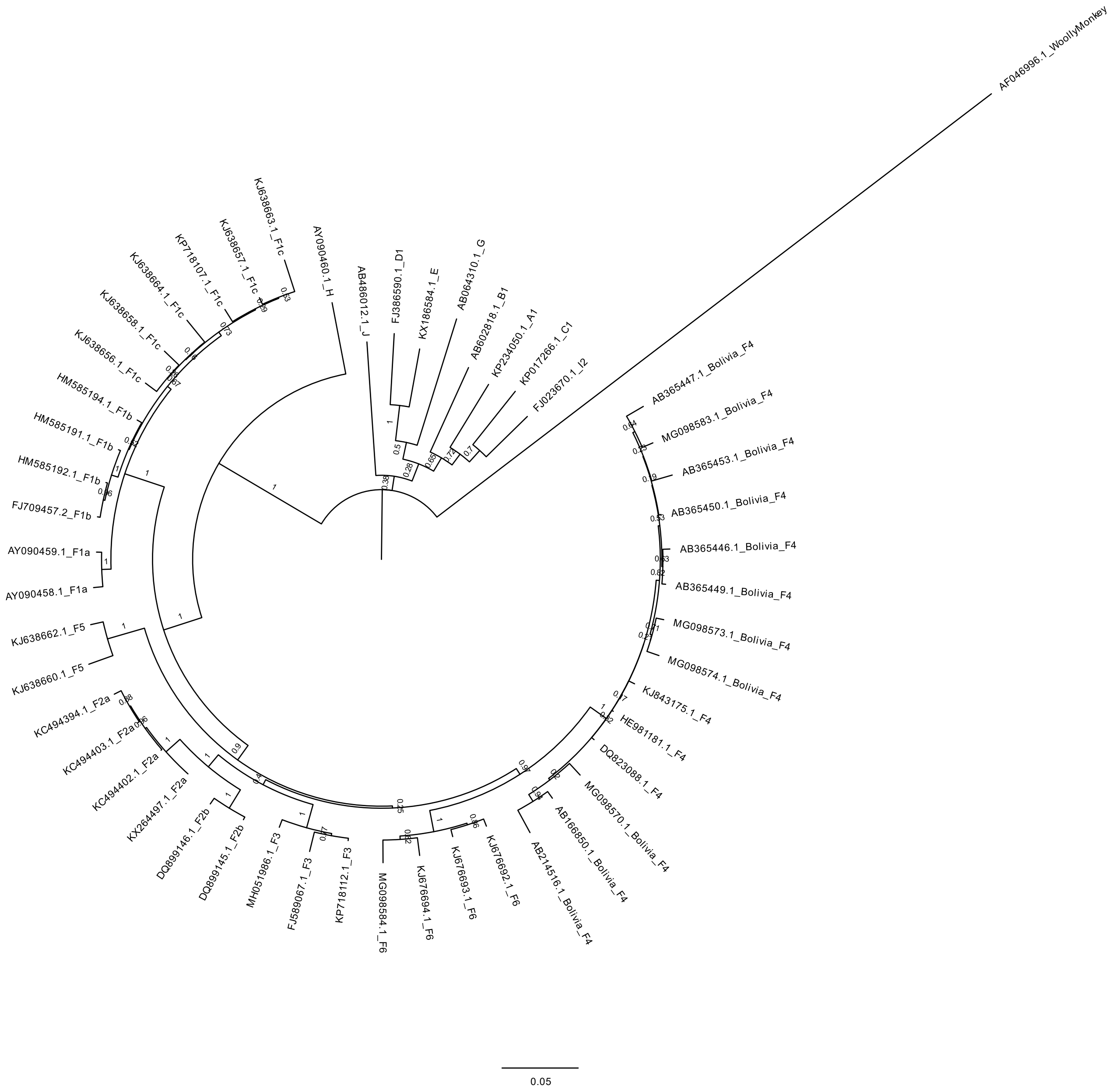

Tree 5. The evolutionary history was inferred by using the Maximum Likelihood method and Tamura-Nei model. The percentage of replicate trees in which the associated taxa clustered together in the bootstrap test (1000 replicates) are shown next to the branches. Initial tree(s) for the heuristic search were obtained automatically by applying Neighbor-Join and BioNJ algorithms to a matrix of pairwise distances estimated using the Tamura-Nei model, and then selecting the topology with superior log likelihood value. A discrete Gamma distribution was used to model evolutionary rate differences among sites (5 categories (+G, parameter = 0.2809)). The tree is drawn to scale, with branch lengths measured in the number of substitutions per site. The analysis involved 51 nucleotide sequences, of which 40 were used as marker sequences to determine the genotype of 11 sequences. All positions containing gaps and missing data were eliminated. There was a total of 3025 positions in the final dataset. Evolutionary analyses were conducted in MEGA X.

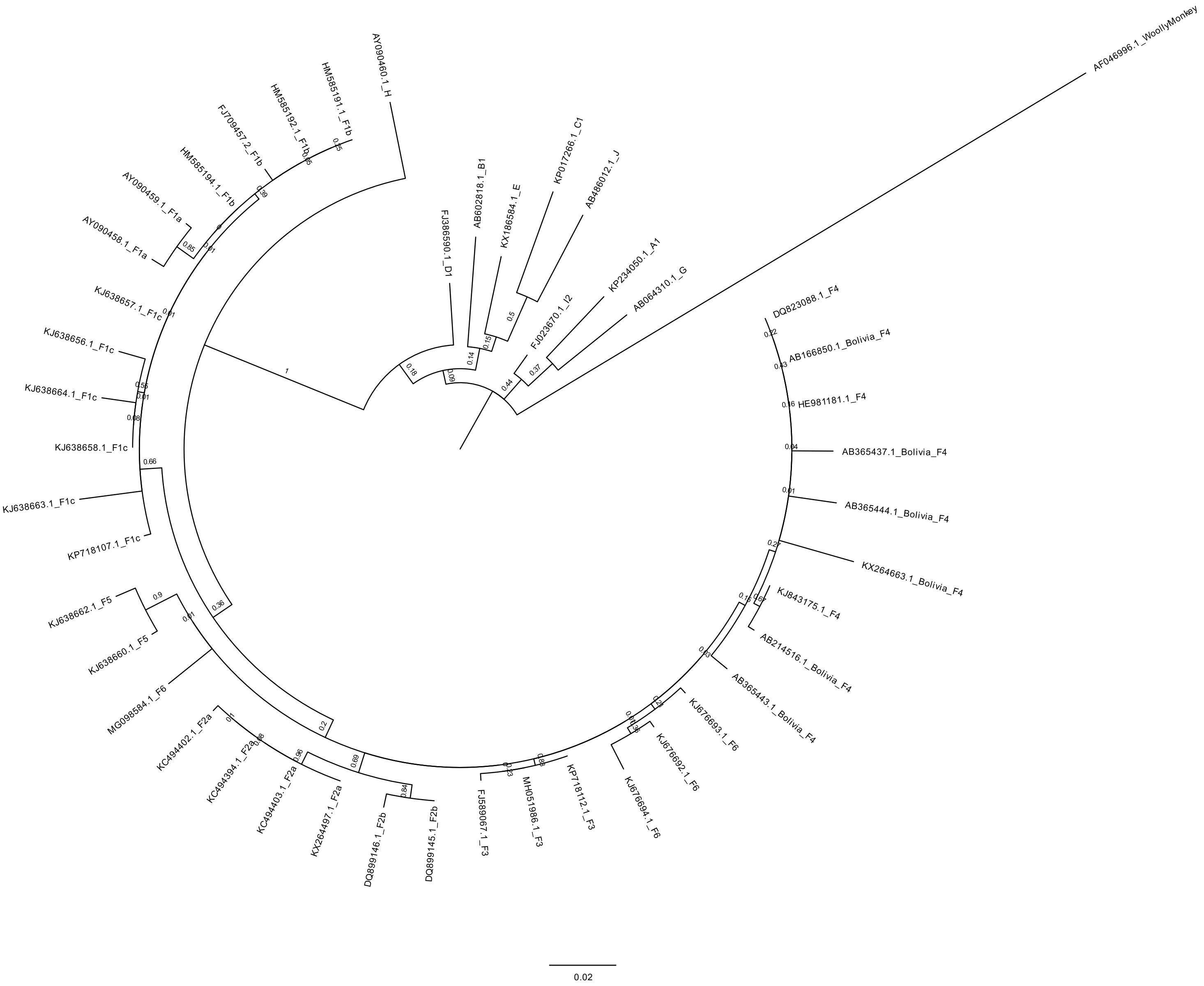

Tree 6. The evolutionary history was inferred by using the Maximum Likelihood method and Tamura-Nei model. The percentage of replicate trees in which the associated taxa clustered together in the bootstrap test (1000 replicates) are shown next to the branches. Initial tree(s) for the heuristic search were obtained automatically by applying Neighbor-Join and BioNJ algorithms to a matrix of pairwise distances estimated using the Tamura-Nei model, and then selecting the topology with superior log likelihood value. A discrete Gamma distribution was used to model evolutionary rate differences among sites (5 categories (+G, parameter = 0.2214)). The tree is drawn to scale, with branch lengths measured in the number of substitutions per site. The analysis involved 46 nucleotide sequences, of which 40 were used as marker sequences to determine the genotype of 6 sequences. All positions containing gaps and missing data were eliminated. There was a total of 554 positions in the final dataset. Evolutionary analyses were conducted in MEGA X.

ID	GENOTYPE	SUBTYPE	COUNTRY	TREE	ALIGNMENT[1]	BASE PAIRS
AB365445	B	B2	Bolivia	1	Complete Genome	3215
AB365441	B	B2	Bolivia	2	73-834	762

AB365438	C	C2	Bolivia	3	73-834	762
AB365439	C	C2	Bolivia	3	73-834	762
AB365440	C	C2	Bolivia	3	73-834	762
AB365442	C	C2	Bolivia	3	73-834	762
AB365451	C	C2	Bolivia	4	Complete Genome	3215
AB365452	C	C2	Bolivia	4	Complete Genome	3216
AB365447	F	F4	Bolivia	5	Complete Genome	3214
AB365446	F	F4	Bolivia	5	Complete Genome	3215
AB365449	F	F4	Bolivia	5	Complete Genome	3215
AB365450	F	F4	Bolivia	5	Complete Genome	3215
MG098570	F	F4	Bolivia	5	Complete Genome	3215
MG098573	F	F4	Bolivia	5	Complete Genome	3215
MG098574	F	F4	Bolivia	5	Complete Genome	3215
MG098583	F	F4	Bolivia	5	Complete Genome	3215
AB365453	F	F4	Bolivia	5	Complete Genome	3227
AB166850	F	F4	Bolivia	6	Complete Genome	3215
AB214516	F	F4	Bolivia	6	Complete Genome	3215
KX264663	F	F4	Bolivia	6	272-973	702
AB365437	F	F4	Bolivia	6	73-834	762
AB365443	F	F4	Bolivia	6	73-834	762
AB365444	F	F4	Bolivia	6	73-834	762

[1]Alignment to complete genome reference sequence VHB NC_003977.2

URUGUAY

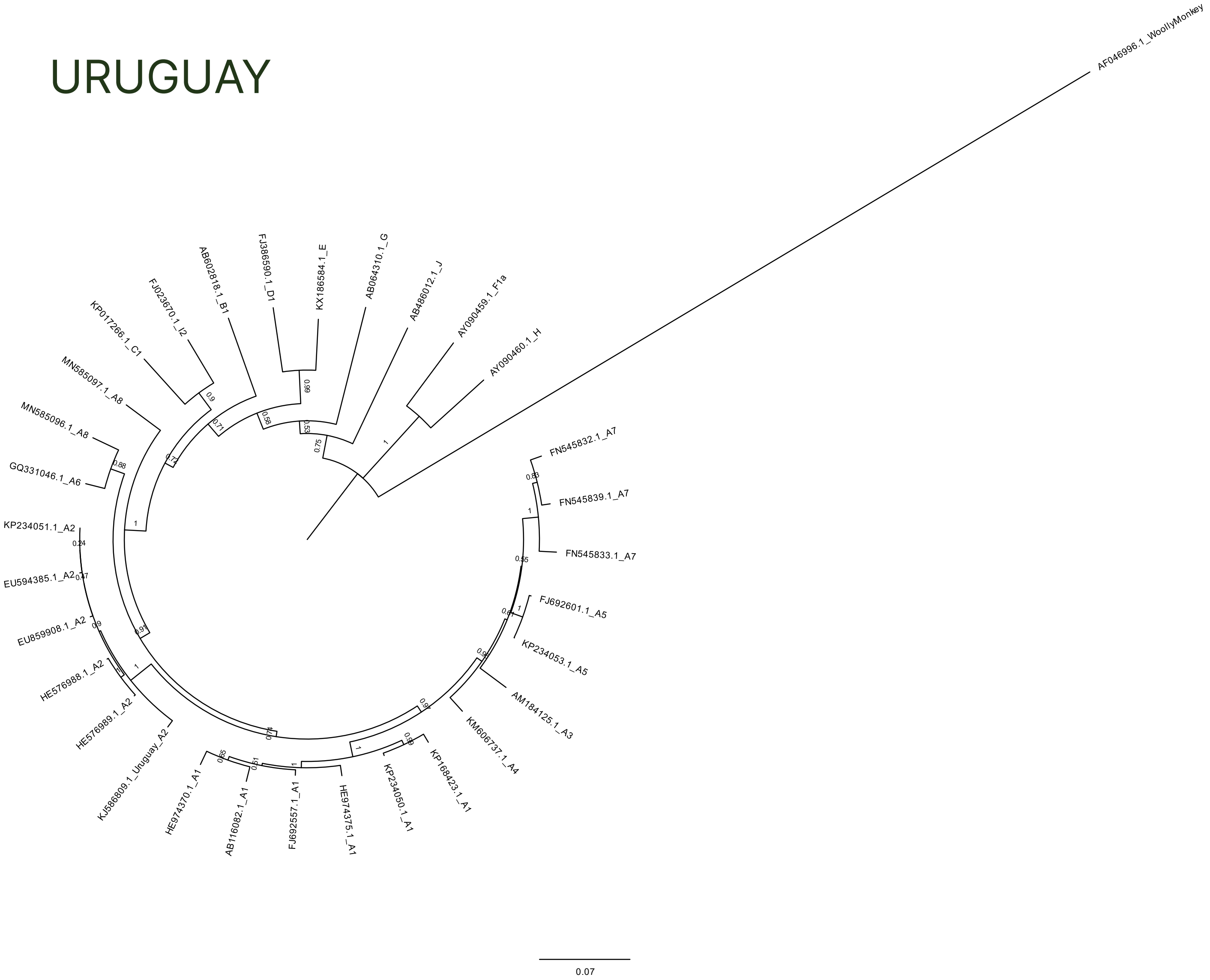

Tree 1. The evolutionary history was inferred by using the Maximum Likelihood method and Tamura-Nei model. The percentage of replicate trees in which the associated taxa clustered together in the bootstrap test (1000 replicates) are shown next to the branches. Initial tree(s) for the heuristic search were obtained automatically by applying Neighbor-Join and BioNJ algorithms to a matrix of pairwise distances estimated using the Tamura-Nei model, and then selecting the topology with superior log likelihood value. A discrete Gamma distribution was used to model evolutionary rate differences among sites (5 categories (+G, parameter = 0.2160)). The tree is drawn to scale, with branch lengths measured in the number of substitutions per site. The analysis involved 32 nucleotide sequences, of which 31 were used as marker sequences to determine the genotype of 1 sequence. All positions containing gaps and missing data were eliminated. There was a total of 3030 positions in the final dataset. Evolutionary analyses were conducted in MEGA X.

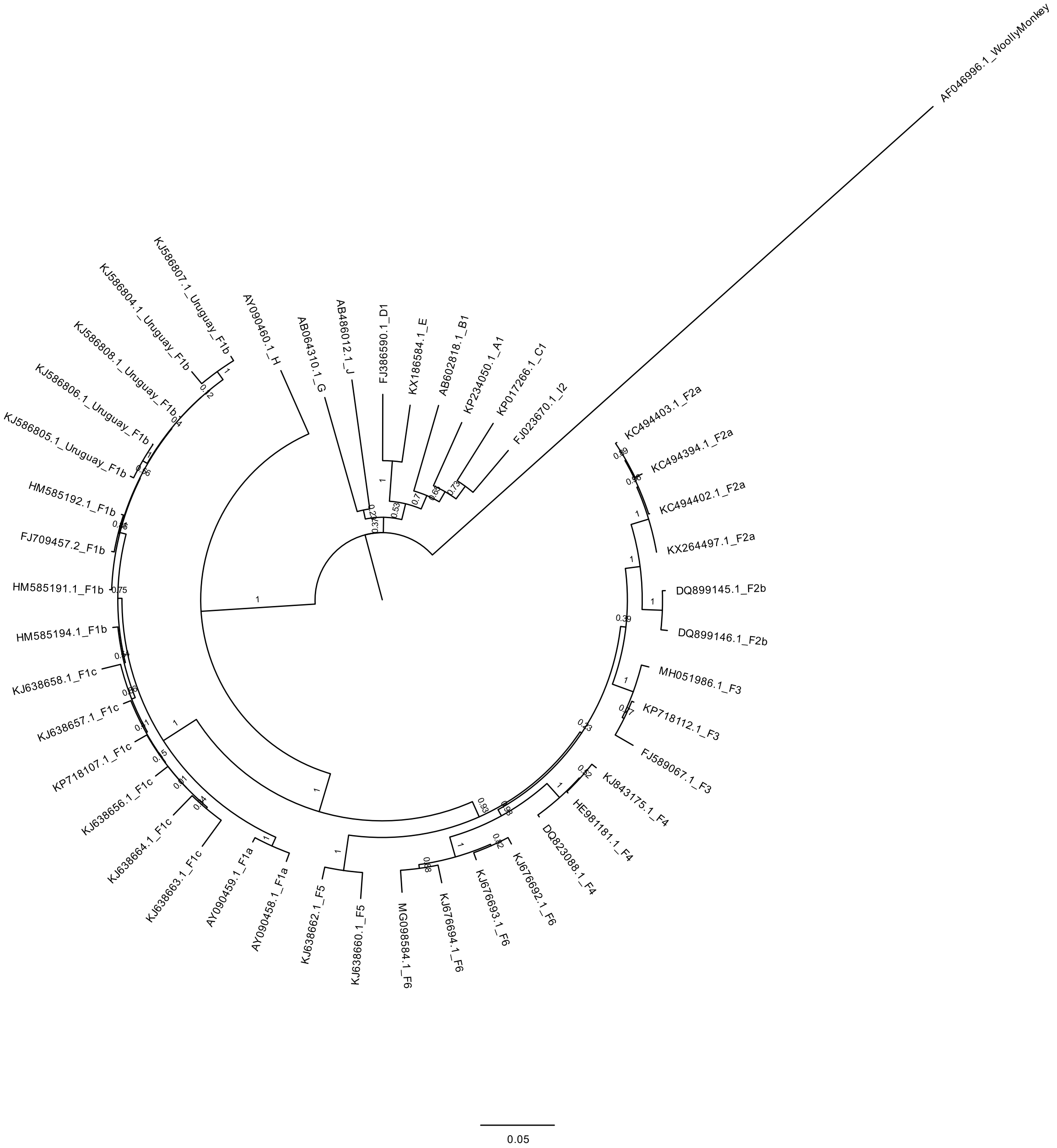

Tree 2. The evolutionary history was inferred by using the Maximum Likelihood method and Tamura-Nei model. The percentage of replicate trees in which the associated taxa clustered together in the bootstrap test (1000 replicates) are shown next to the branches. Initial tree(s) for the heuristic search were obtained automatically by applying Neighbor-Join and BioNJ algorithms to a matrix of pairwise distances estimated using the Tamura-Nei model, and then selecting the topology with superior log likelihood value. A discrete Gamma distribution was used to model evolutionary rate differences among sites (5 categories (+G, parameter = 0.2700)). The tree is drawn to scale, with branch lengths measured in the number of substitutions per site. The analysis involved 45 nucleotide sequences, of which 40 were used as marker sequences to determine the genotype of 5 sequences. All positions containing gaps and missing data were eliminated. There was a total of 3049 positions in the final dataset. Evolutionary analyses were conducted in MEGA X.

ID	GENOTYPE	SUBTYPE	COUNTRY	TREE	ALIGNMENT[1]	BASE PAIRS
KJ586809	A	A2	Uruguay	1	Complete Genome	3221

KJ586804	F	F1b	Uruguay	2	Complete Genome	3215
KJ586805	F	F1b	Uruguay	2	Complete Genome	3182
KJ586806	F	F1b	Uruguay	2	Complete Genome	3182
KJ586807	F	F1b	Uruguay	2	Complete Genome	3215
KJ586808	F	F1b	Uruguay	2	Complete Genome	3215

[1]Alignment to complete genome reference sequence VHB NC_003977.2

CHILE

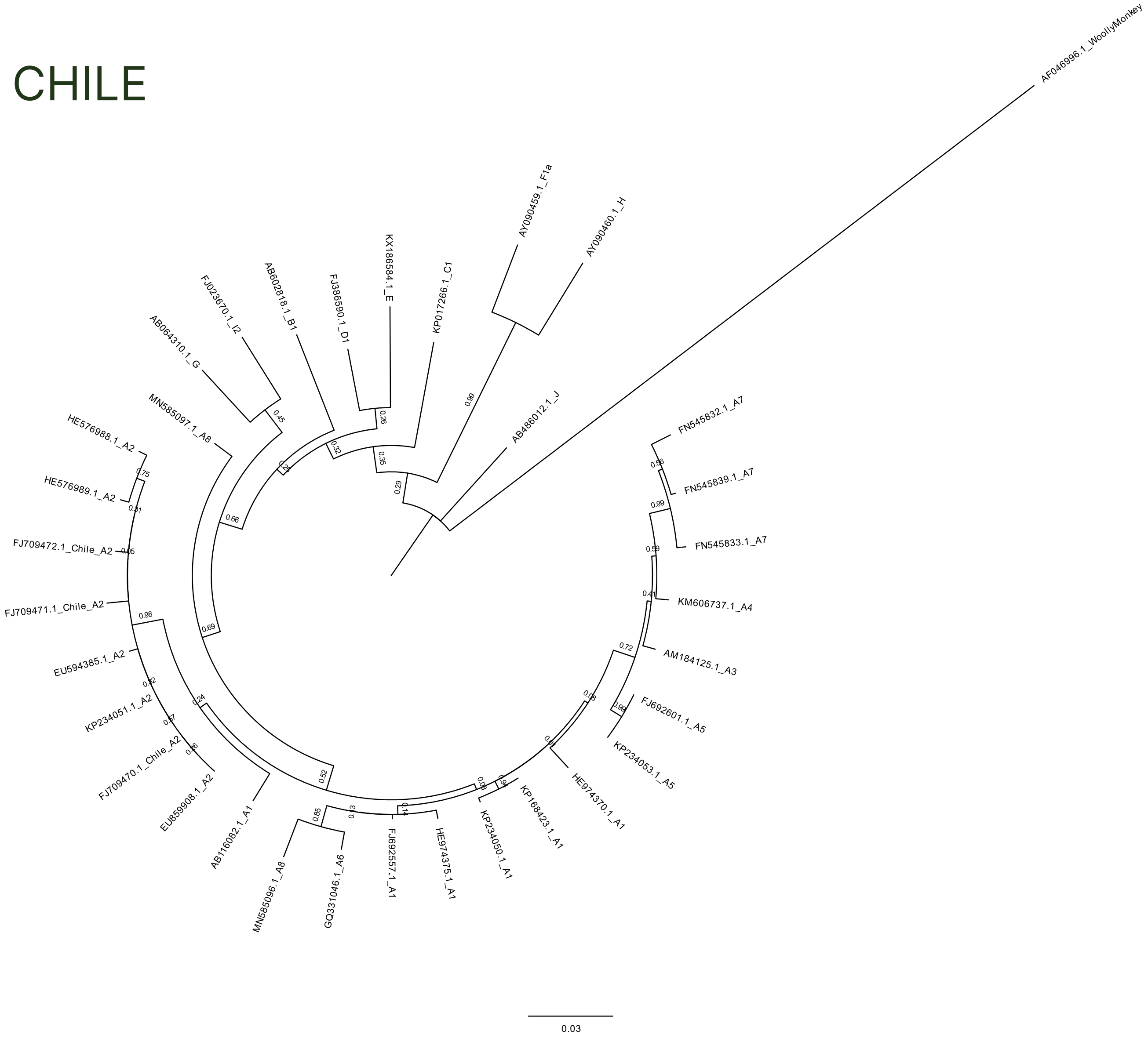

Tree 1. The evolutionary history was inferred by using the Maximum Likelihood method and Tamura-Nei model. The percentage of replicate trees in which the associated taxa clustered together in the bootstrap test (1000 replicates) are shown next to the branches. Initial tree(s) for the heuristic search were obtained automatically by applying Neighbor-Join and BioNJ algorithms to a matrix of pairwise distances estimated using the Tamura-Nei model, and then selecting the topology with superior log likelihood value. A discrete Gamma distribution was used to model evolutionary rate differences among sites (5 categories (+G, parameter = 0.1638)). The tree is drawn to scale, with branch lengths measured in the number of substitutions per site. The analysis involved 34 nucleotide sequences, of which 31 were used as marker sequences to determine the genotype of 3 sequences. All positions containing gaps and missing data were eliminated. There was a total of 740 positions in the final dataset. Evolutionary analyses were conducted in MEGA X.

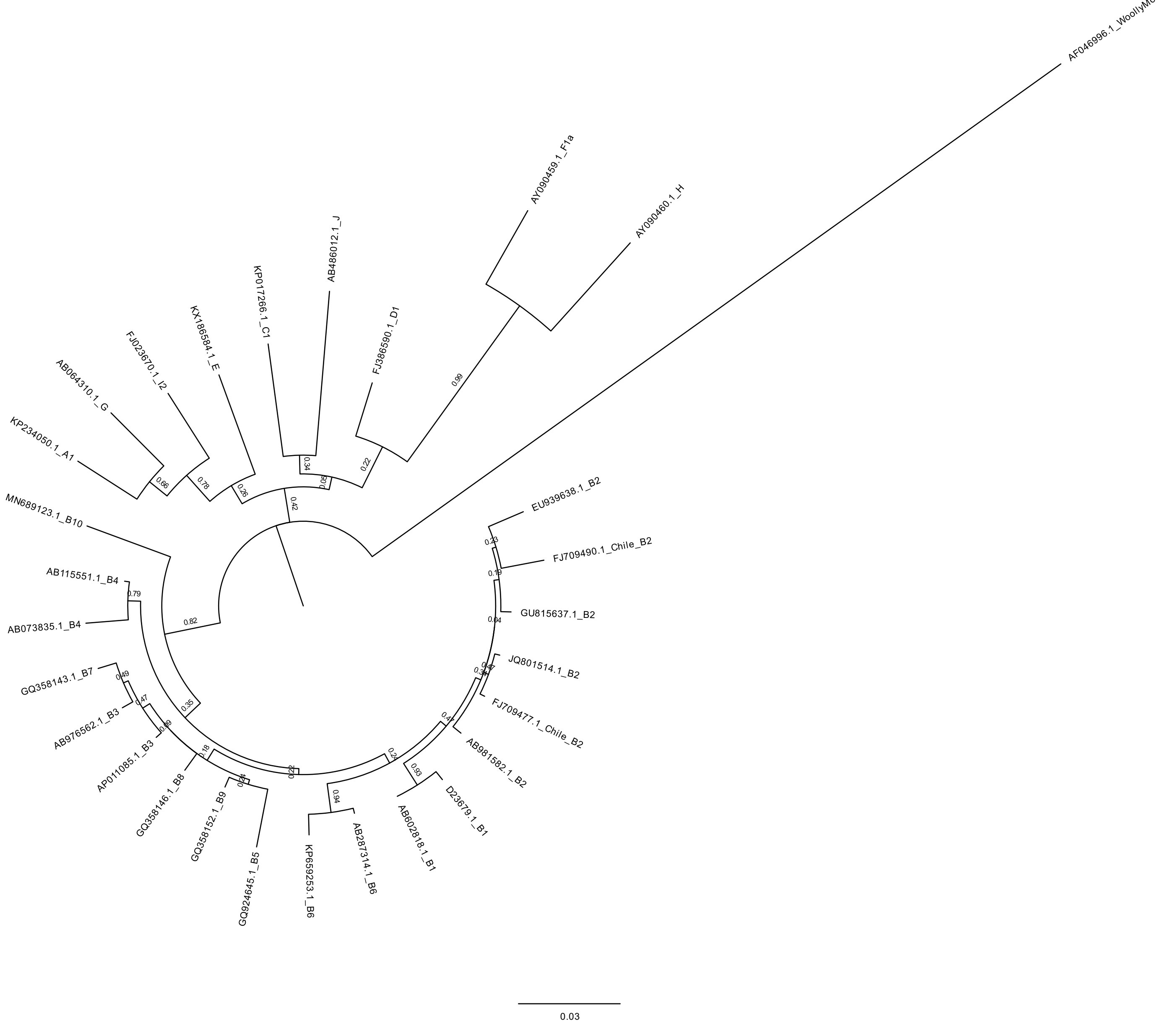

Tree 2. The evolutionary history was inferred by using the Maximum Likelihood method and Tamura-Nei model. The percentage of replicate trees in which the associated taxa clustered together in the bootstrap test (1000 replicates) are shown next to the branches. Initial tree(s) for the heuristic search were obtained automatically by applying Neighbor-Join and BioNJ algorithms to a matrix of pairwise distances estimated using the Tamura-Nei model, and then selecting the topology with superior log likelihood value. A discrete Gamma distribution was used to model evolutionary rate differences among sites (5 categories (+G, parameter = 0.1650)). The tree is drawn to scale, with branch lengths measured in the number of substitutions per site. The analysis involved 29 nucleotide sequences, of which 27 were used as marker sequences to determine the genotype of 2 sequences. All positions containing gaps and missing data were eliminated. There was a total of 736 positions in the final dataset. Evolutionary analyses were conducted in MEGA X.

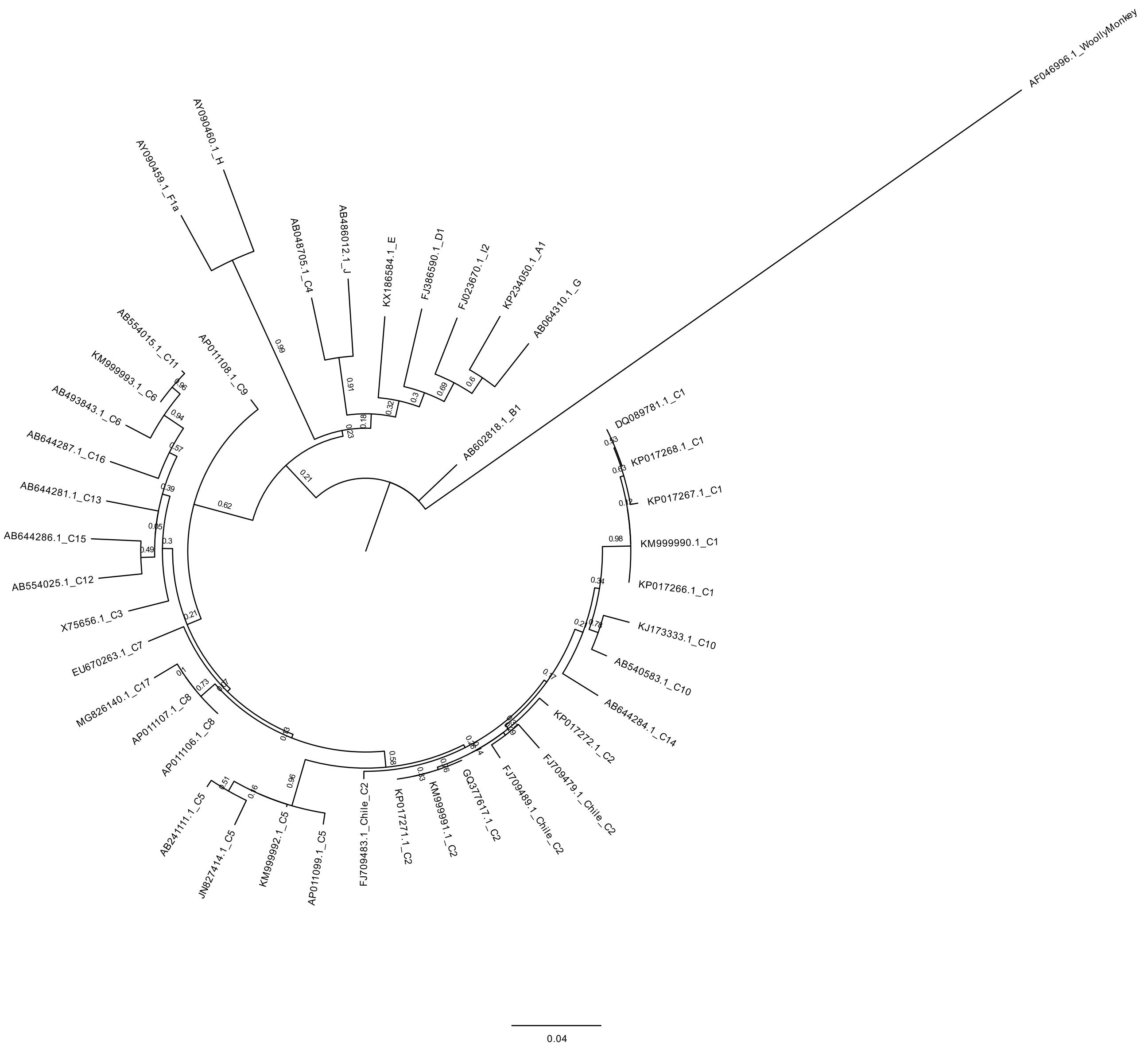

Tree 3. The evolutionary history was inferred by using the Maximum Likelihood method and Tamura-Nei model. The percentage of replicate trees in which the associated taxa clustered together in the bootstrap test (1000 replicates) are shown next to the branches. Initial tree(s) for the heuristic search were obtained automatically by applying Neighbor-Join and BioNJ algorithms to a matrix of pairwise distances estimated using the Tamura-Nei model, and then selecting the topology with superior log likelihood value. A discrete Gamma distribution was used to model evolutionary rate differences among sites (5 categories (+G, parameter = 0.1495)). The tree is drawn to scale, with branch lengths measured in the number of substitutions per site. The analysis involved 43 nucleotide sequences, of which 40 were used as marker sequences to determine the genotype of 3 sequences. All positions containing gaps and missing data were eliminated. There was a total of 749 positions in the final dataset. Evolutionary analyses were conducted in MEGA X.

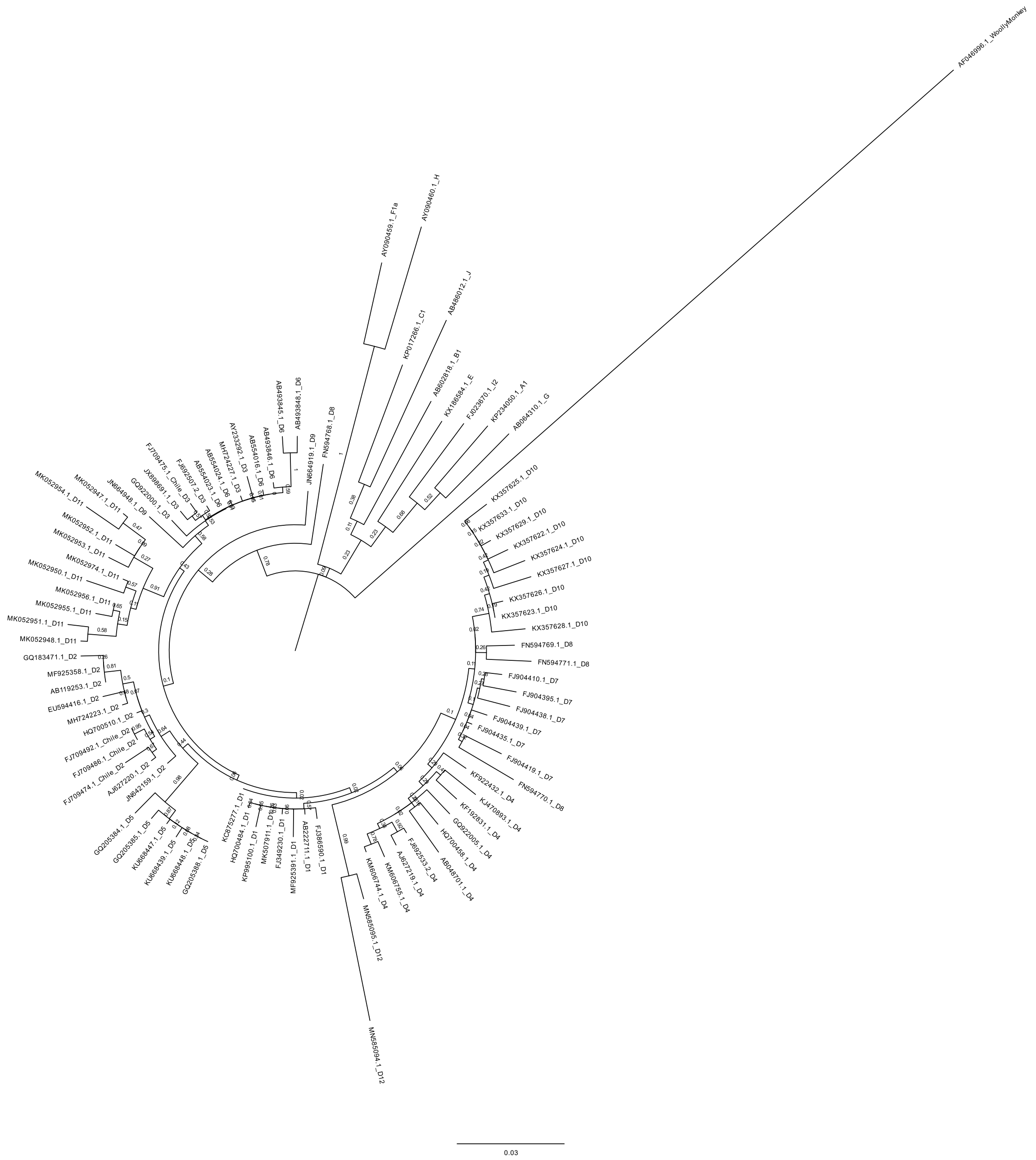

Tree 4. The evolutionary history was inferred by using the Maximum Likelihood method and Tamura-Nei model. The percentage of replicate trees in which the associated taxa clustered together in the bootstrap test (1000 replicates) are shown next to the branches. Initial tree(s) for the heuristic search were obtained automatically by applying Neighbor-Join and BioNJ algorithms to a matrix of pairwise distances estimated using the Tamura-Nei model, and then selecting the topology with superior log likelihood value. A discrete Gamma distribution was used to model evolutionary rate differences among sites (5 categories (+G, parameter = 0.2576)). The tree is drawn to scale, with branch lengths measured in the number of substitutions per site. The analysis involved 90 nucleotide sequences, of which 86 were used as marker sequences to determine the genotype of 4 sequences. All positions containing gaps and missing data were eliminated. There was a total of 749 positions in the final dataset. Evolutionary analyses were conducted in MEGA X.

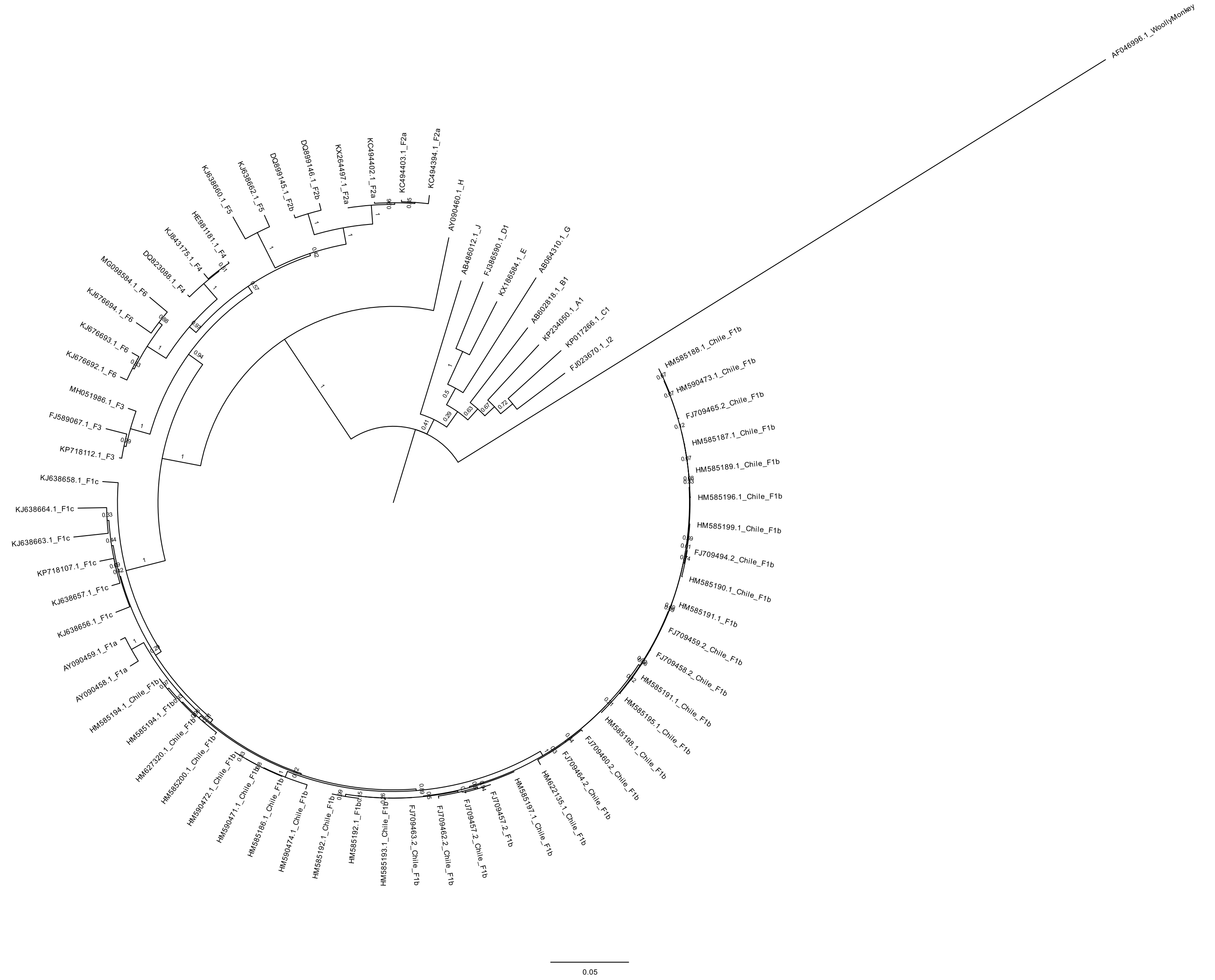

Tree 5. The evolutionary history was inferred by using the Maximum Likelihood method and Tamura-Nei model. The percentage of replicate trees in which the associated taxa clustered together in the bootstrap test (1000 replicates) are shown next to the branches. Initial tree(s) for the heuristic search were obtained automatically by applying Neighbor-Join and BioNJ algorithms to a matrix of pairwise distances estimated using the Tamura-Nei model, and then selecting the topology with superior log likelihood value. A discrete Gamma distribution was used to model evolutionary rate differences among sites (5 categories (+G, parameter = 0.2629)). The tree is drawn to scale, with branch lengths measured in the number of substitutions per site. The analysis involved 70 nucleotide sequences, of which 40 were used as marker sequences to determine the genotype of 30 sequences. All positions containing gaps and missing data were eliminated. There was a total of 3051 positions in the final dataset. Evolutionary analyses were conducted in MEGA X.

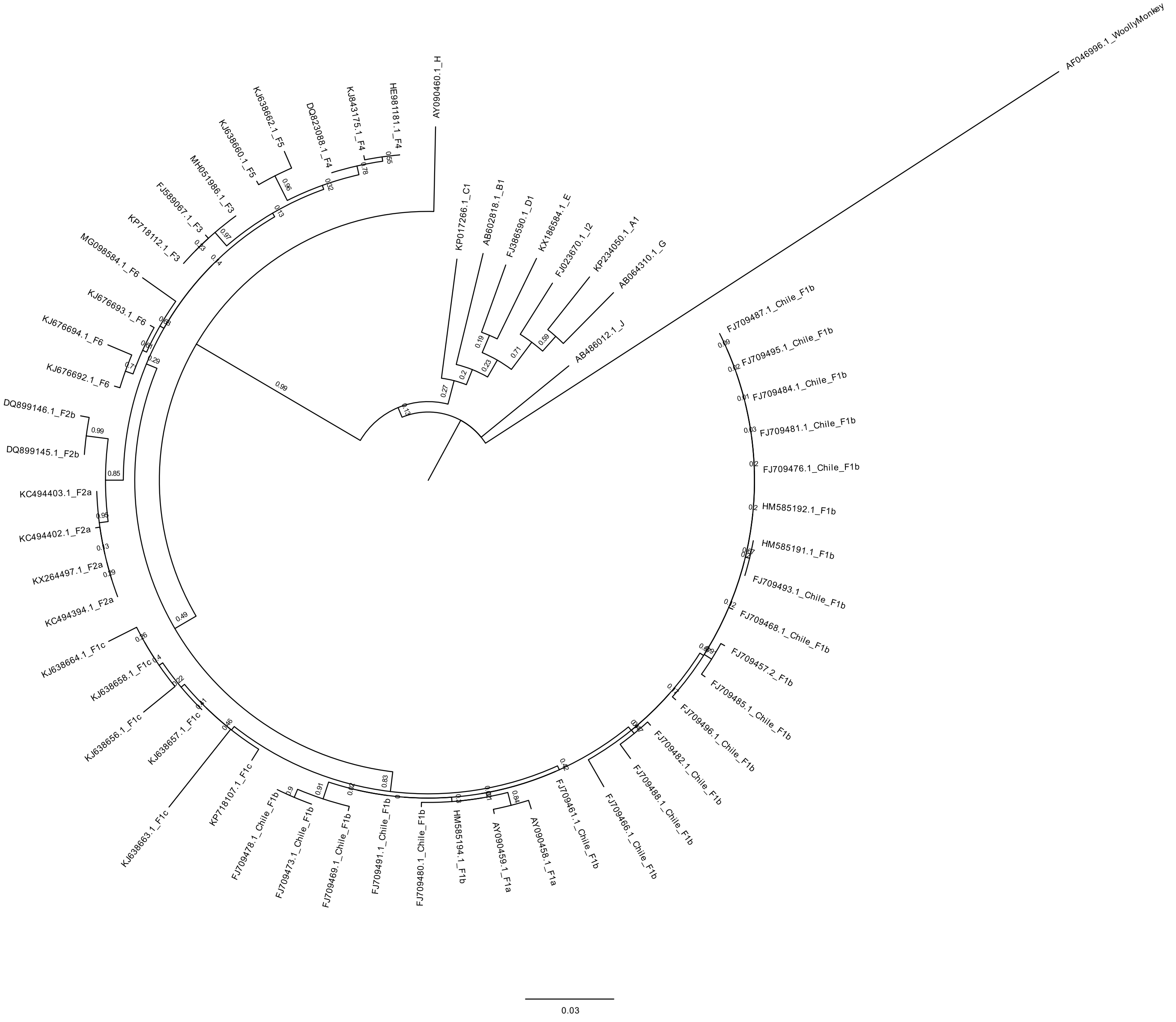

Tree 6. The evolutionary history was inferred by using the Maximum Likelihood method and Tamura-Nei model. The percentage of replicate trees in which the associated taxa clustered together in the bootstrap test (1000 replicates) are shown next to the branches. Initial tree(s) for the heuristic search were obtained automatically by applying Neighbor-Join and BioNJ algorithms to a matrix of pairwise distances estimated using the Tamura-Nei model, and then selecting the topology with superior log likelihood value. A discrete Gamma distribution was used to model evolutionary rate differences among sites (5 categories (+G, parameter = 0.1914)). The tree is drawn to scale, with branch lengths measured in the number of substitutions per site. The analysis involved 58 nucleotide sequences, of which 40 were used as marker sequences to determine the genotype of 18 sequences. Although certain sequences initially showed unclear alignment, posterior phylogenetic analysis supported their initial classification. All positions containing gaps and missing data were eliminated. There was a total of 739 positions in the final dataset. Evolutionary analyses were conducted in MEGA X.

ID	GENOTYPE	SUBTYPE	COUNTRY	TREE	ALIGNMENT[1]	BASE PAIRS
FJ709470	A	A2	Chile	1	106-858	753
FJ709471	A	A2	Chile	1	106-858	753
FJ709472	A	A2	Chile	1	106-858	753
FJ709477	B	B2	Chile	2	106-858	753
FJ709490	B	B2	Chile	2	106-858	753
FJ709479	C	C2	Chile	3	106-858	753
FJ709483	C	C2	Chile	3	106-858	753
FJ709489	C	C2	Chile	3	106-858	753
FJ709474	D	D2	Chile	4	106-858	753
FJ709475	D	D3	Chile	4	106-858	753
FJ709486	D	D2	Chile	4	106-858	753
FJ709492	D	D2	Chile	4	106-858	753
FJ709494	F	F1b	Chile	5	Complete Genome	3215
FJ709457	F	F1b	Chile	5	Complete Genome	3215
FJ709458	F	F1b	Chile	5	Complete Genome	3215
FJ709459	F	F1b	Chile	5	Complete Genome	3215
FJ709460	F	F1b	Chile	5	Complete Genome	3215
FJ709462	F	F1b	Chile	5	Complete Genome	3215
FJ709463	F	F1b	Chile	5	Complete Genome	3215
FJ709464	F	F1b	Chile	5	Complete Genome	3215
FJ709465	F	F1b	Chile	5	Complete Genome	3215
HM585186	F	F1b	Chile	5	Complete Genome	3215
HM585187	F	F1b	Chile	5	Complete Genome	3215
HM585188	F	F1b	Chile	5	Complete Genome	3215
HM585189	F	F1b	Chile	5	Complete Genome	3215
HM585190	F	F1b	Chile	5	Complete Genome	3215
HM585191	F	F1b	Chile	5	Complete Genome	3215
HM585192	F	F1b	Chile	5	Complete Genome	3215
HM585193	F	F1b	Chile	5	Complete Genome	3215
HM585194	F	F1b	Chile	5	Complete Genome	3215
HM585195	F	F1b	Chile	5	Complete Genome	3215
HM585196	F	F1b	Chile	5	Complete Genome	3215
HM585197	F	F1b	Chile	5	Complete Genome	3215
HM585198	F	F1b	Chile	5	Complete Genome	3215
HM585199	F	F1b	Chile	5	Complete Genome	3215
HM585200	F	F1b	Chile	5	Complete Genome	3215
HM590471	F	F1b	Chile	5	Complete Genome	3215
HM590472	F	F1b	Chile	5	Complete Genome	3215
HM590473	F	F1b	Chile	5	Complete Genome	3215

HM590474	F	F1b	Chile	5	Complete Genome	3215
HM622135	F	F1b	Chile	5	Complete Genome	3215
HM627320	F	F1b	Chile	5	Complete Genome	3215
FJ709461	F	F1b	Chile	6	106-858	753
FJ709466	F	F1b	Chile	6	106-858	753
FJ709468	F	F1b	Chile	6	106-858	753
FJ709469	F	F1b	Chile	6	106-858	753
FJ709473	F	F1b	Chile	6	106-858	753
FJ709476	F	F1b	Chile	6	106-858	753
FJ709478	F	F1b	Chile	6	106-858	753
FJ709480	F	F1b	Chile	6	106-858	753
FJ709481	F	F1b	Chile	6	106-858	753
FJ709482	F	F1b	Chile	6	106-858	753
FJ709484	F	F1b	Chile	6	106-858	753
FJ709485	F	F1b	Chile	6	106-858	753
FJ709487	F	F1b	Chile	6	106-858	753
FJ709488	F	F1b	Chile	6	106-858	753
FJ709491	F	F1b	Chile	6	106-858	753
FJ709493	F	F1b	Chile	6	106-858	753
FJ709495	F	F1b	Chile	6	106-858	753
FJ709496	F	F1b	Chile	6	106-858	753

[1]Alignment to complete genome reference sequence VHB NC_003977.2

ARGENTINA

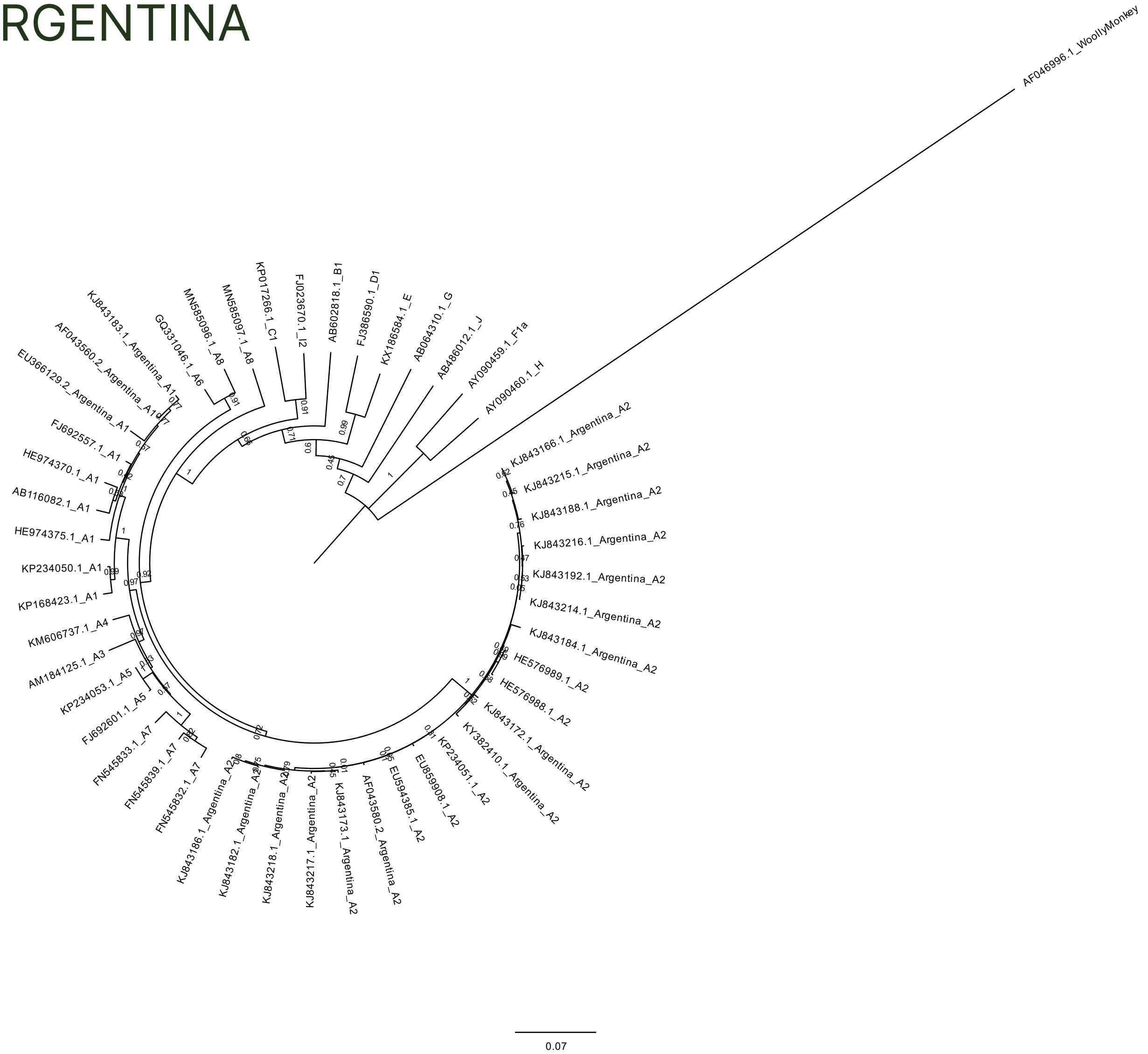

Tree 1. The evolutionary history was inferred by using the Maximum Likelihood method and Tamura-Nei model. The percentage of replicate trees in which the associated taxa clustered together in the bootstrap test (1000 replicates) are shown next to the branches. Initial tree(s) for the heuristic search were obtained automatically by applying Neighbor-Join and BioNJ algorithms to a matrix of pairwise distances estimated using the Tamura-Nei model, and then selecting the topology with superior log likelihood value. A discrete Gamma distribution was used to model evolutionary rate differences among sites (5 categories (+G, parameter = 0.2165)). The tree is drawn to scale, with branch lengths measured in the number of substitutions per site. The analysis involved 49 nucleotide sequences, of which 31 were used as marker sequences to determine the genotype of 18 sequences. All positions containing gaps and missing data were eliminated. There was a total of 3010 positions in the final dataset. Evolutionary analyses were conducted in MEGA X.

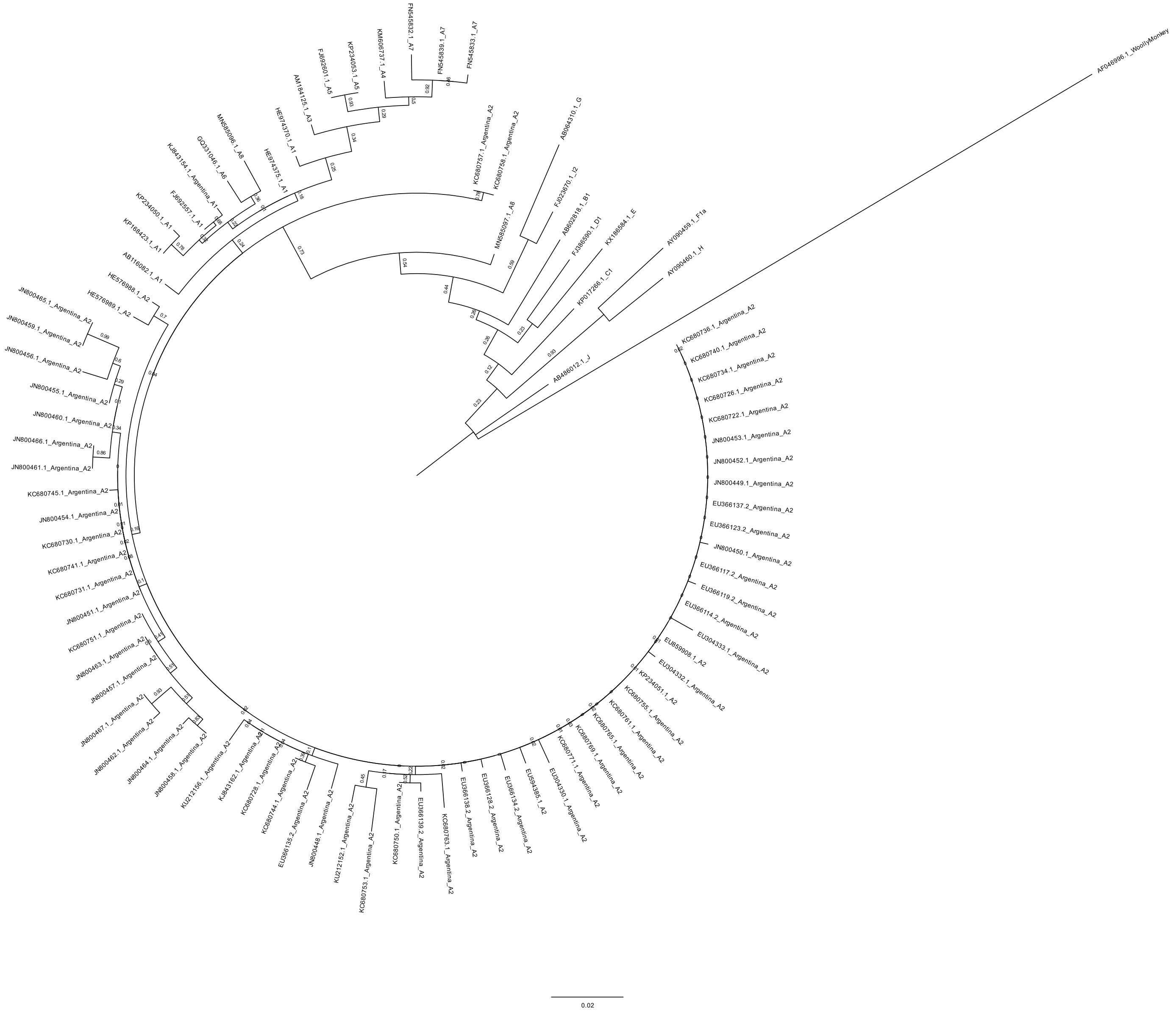

Tree 2. The evolutionary history was inferred by using the Maximum Likelihood method and Tamura-Nei model. The percentage of replicate trees in which the associated taxa clustered together in the bootstrap test (1000 replicates) are shown next to the branches. Initial tree(s) for the heuristic search were obtained automatically by applying Neighbor-Join and BioNJ algorithms to a matrix of pairwise distances estimated using the Tamura-Nei model, and then selecting the topology with superior log likelihood value. A discrete Gamma distribution was used to model evolutionary rate differences among sites (5 categories (+G, parameter = 0.2084)). The tree is drawn to scale, with branch lengths measured in the number of substitutions per site. The analysis involved 90 nucleotide sequences, of which 31 were used as marker sequences to determine the genotype of 59 sequences. Although some sequences showed uncertain placement, posterior analysis supported their initial classification. All positions containing gaps and missing data were eliminated. There was a total of 499 positions in the final dataset. Evolutionary analyses were conducted in MEGA X.

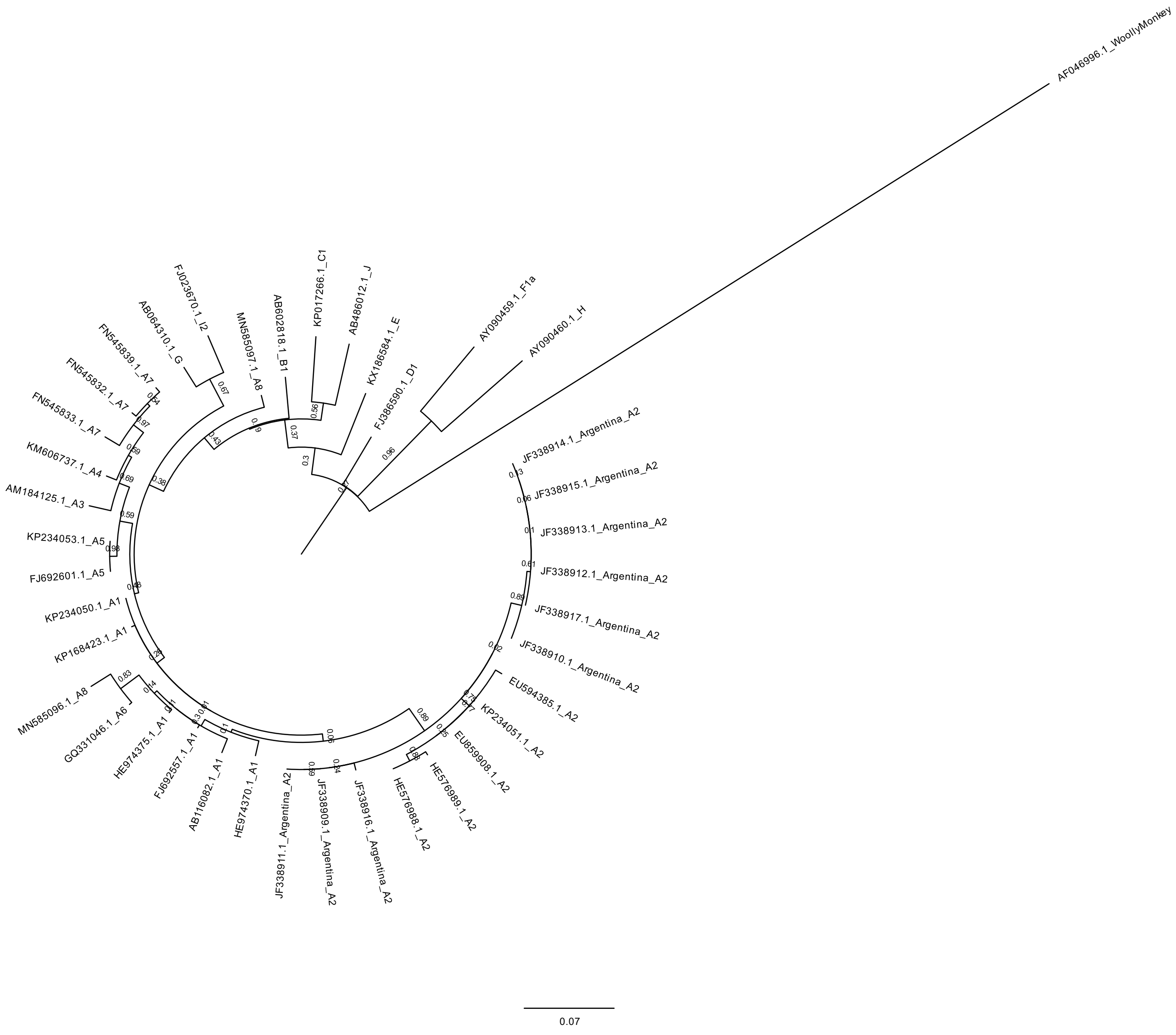

Tree 3. The evolutionary history was inferred by using the Maximum Likelihood method and Tamura-Nei model. The percentage of replicate trees in which the associated taxa clustered together in the bootstrap test (1000 replicates) are shown next to the branches. Initial tree(s) for the heuristic search were obtained automatically by applying Neighbor-Join and BioNJ algorithms to a matrix of pairwise distances estimated using the Tamura-Nei model, and then selecting the topology with superior log likelihood value. A discrete Gamma distribution was used to model evolutionary rate differences among sites (5 categories (+G, parameter = 0.1678)). The tree is drawn to scale, with branch lengths measured in the number of substitutions per site. The analysis involved 40 nucleotide sequences, of which 31 were used as marker sequences to determine the genotype of 9 sequences. All positions containing gaps and missing data were eliminated. There was a total of 445 positions in the final dataset. Evolutionary analyses were conducted in MEGA X.

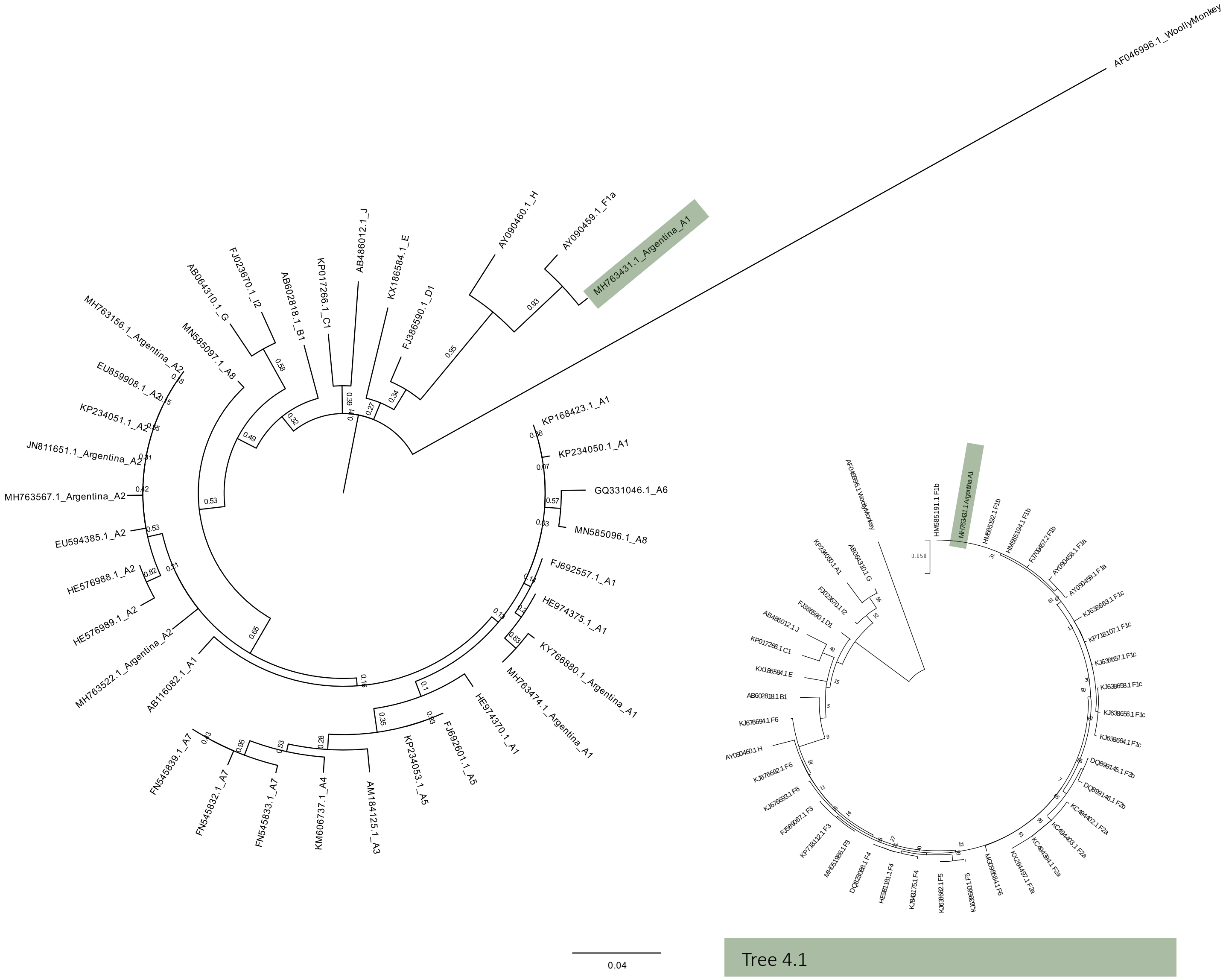

Tree 4.1

Tree 4. The evolutionary history was inferred by using the Maximum Likelihood method and Tamura-Nei model. The percentage of replicate trees in which the associated taxa clustered together in the bootstrap test (1000 replicates) are shown next to the branches. Initial tree(s) for the heuristic search were obtained automatically by applying Neighbor-Join and BioNJ algorithms to a matrix of pairwise distances estimated using the Tamura-Nei model, and then selecting the topology with superior log likelihood value. A discrete Gamma distribution was used to model evolutionary rate differences among sites (5 categories (+G, parameter = 0.1472)). The tree is drawn to scale, with branch lengths measured in the number of substitutions per site. The analysis involved 38 nucleotide sequences, of which 31 were used as marker sequences to determine the genotype of 7 sequences. Sequence MH763431 aligned with the F class outgroup in the initial phylogenetic tree, and upon further analysis (Tree 4.1) it was reclassified to F1b. All positions containing gaps and missing data were eliminated. There was a total of 385 positions in the final dataset. Evolutionary analyses were conducted in MEGA X.

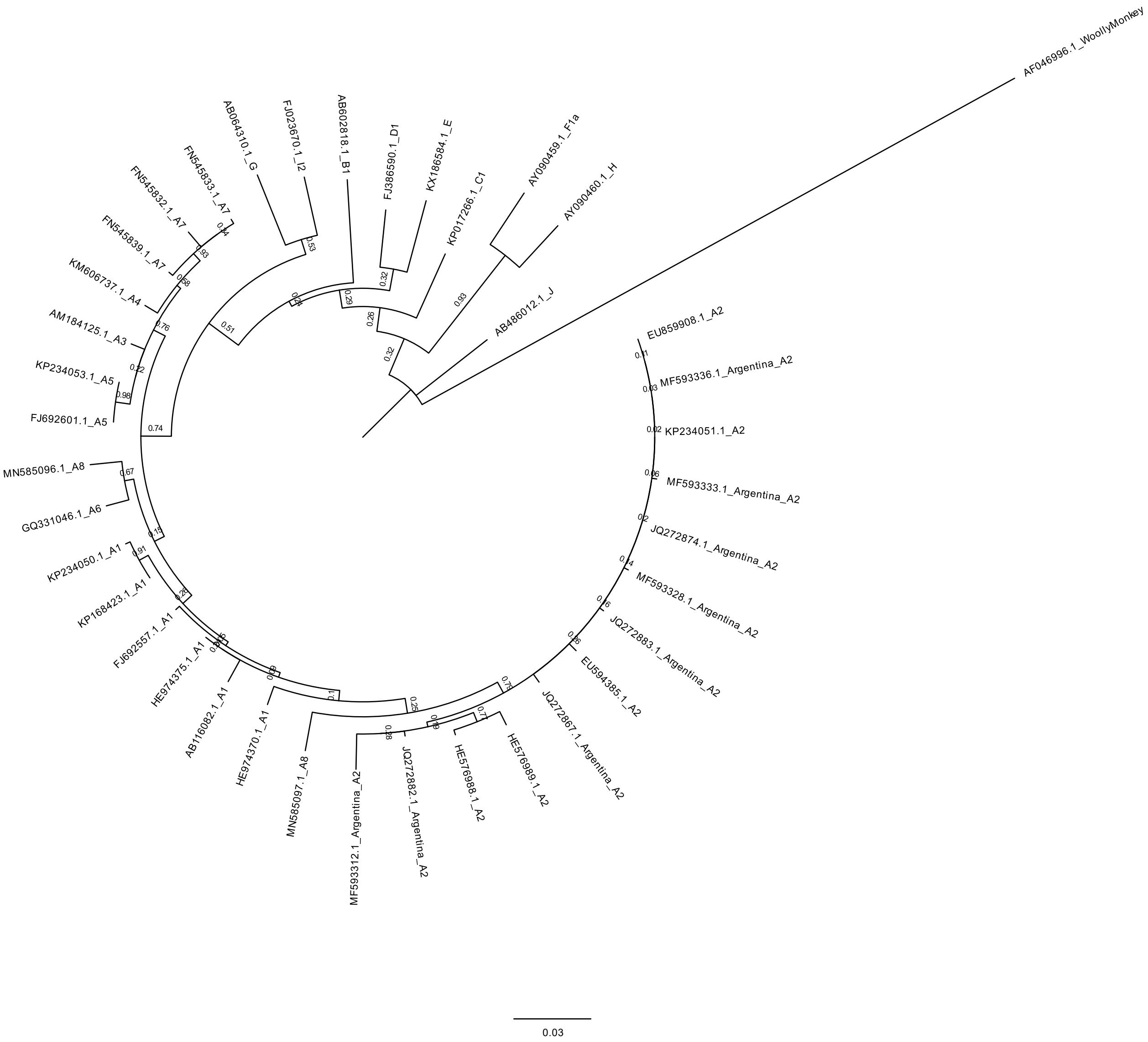

Tree 5. The evolutionary history was inferred by using the Maximum Likelihood method and Tamura-Nei model. The percentage of replicate trees in which the associated taxa clustered together in the bootstrap test (1000 replicates) are shown next to the branches. Initial tree(s) for the heuristic search were obtained automatically by applying Neighbor-Join and BioNJ algorithms to a matrix of pairwise distances estimated using the Tamura-Nei model, and then selecting the topology with superior log likelihood value. A discrete Gamma distribution was used to model evolutionary rate differences among sites (5 categories (+G, parameter = 0.1580)). The tree is drawn to scale, with branch lengths measured in the number of substitutions per site. The analysis involved 39 nucleotide sequences, of which 31 were used as marker sequences to determine the genotype of 8 sequences. All positions containing gaps and missing data were eliminated. There was a total of 622 positions in the final dataset. Evolutionary analyses were conducted in MEGA X.

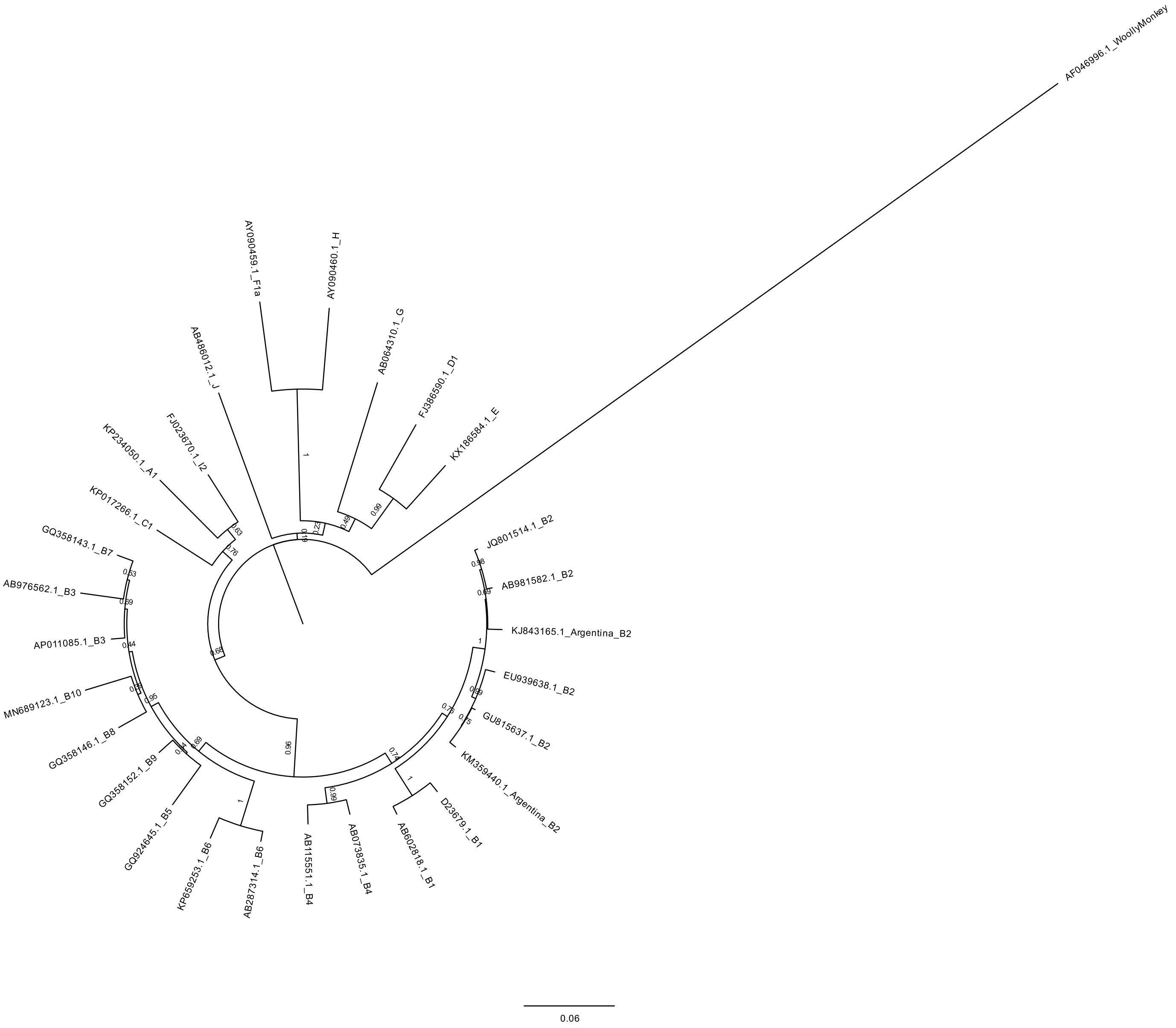

Tree 6. The evolutionary history was inferred by using the Maximum Likelihood method and Tamura-Nei model. The percentage of replicate trees in which the associated taxa clustered together in the bootstrap test (1000 replicates) are shown next to the branches. Initial tree(s) for the heuristic search were obtained automatically by applying Neighbor-Join and BioNJ algorithms to a matrix of pairwise distances estimated using the Tamura-Nei model, and then selecting the topology with superior log likelihood value. A discrete Gamma distribution was used to model evolutionary rate differences among sites (5 categories (+G, parameter = 0.2568)). The tree is drawn to scale, with branch lengths measured in the number of substitutions per site. The analysis involved 29 nucleotide sequences, of which 27 were used as marker sequences to determine the genotype of 2 sequences. All positions containing gaps and missing data were eliminated. There was a total of 3124 positions in the final dataset. Evolutionary analyses were conducted in MEGA X.

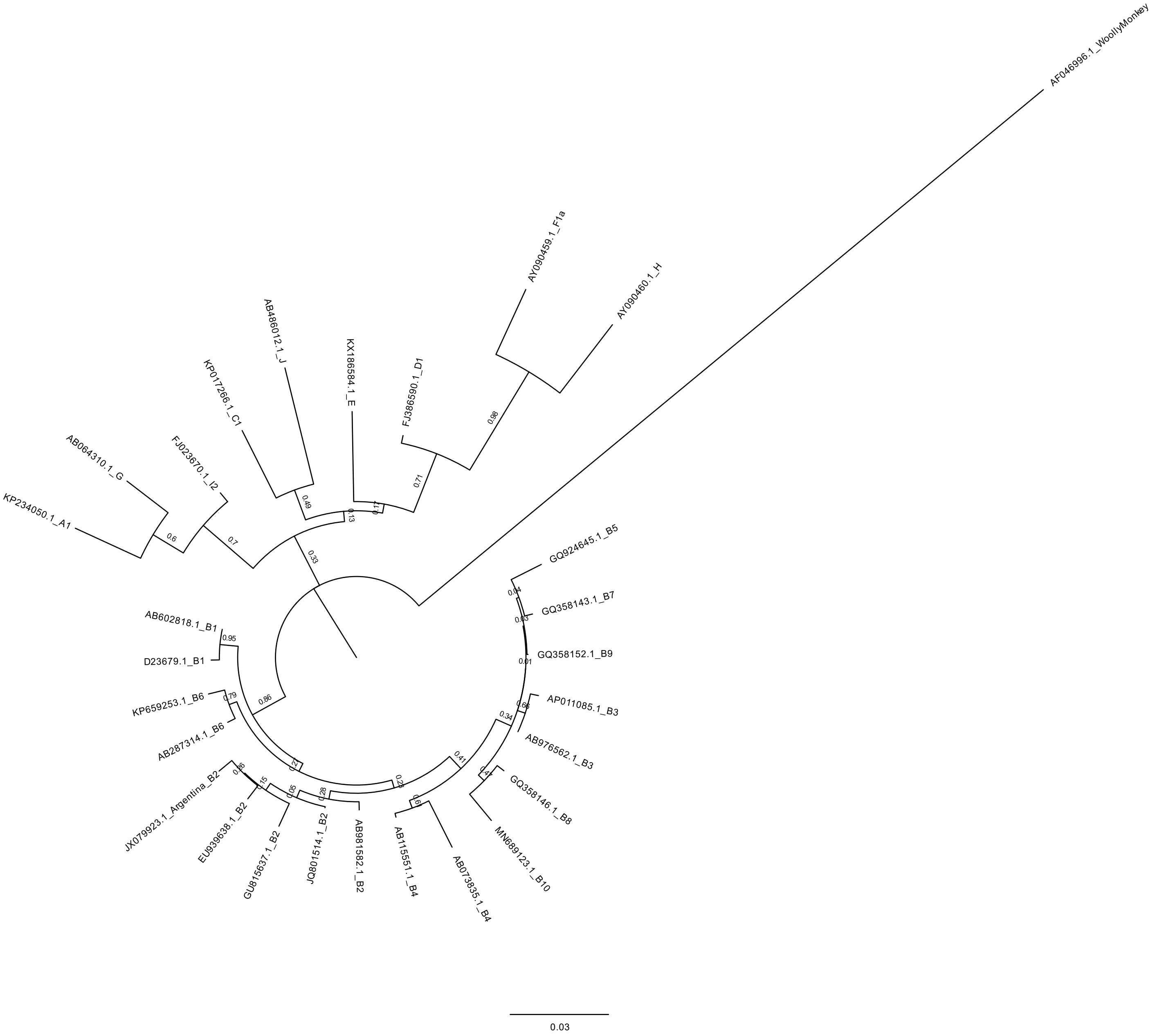

Tree 7. The evolutionary history was inferred by using the Maximum Likelihood method and Tamura-Nei model. The percentage of replicate trees in which the associated taxa clustered together in the bootstrap test (1000 replicates) are shown next to the branches. Initial tree(s) for the heuristic search were obtained automatically by applying Neighbor-Join and BioNJ algorithms to a matrix of pairwise distances estimated using the Tamura-Nei model, and then selecting the topology with superior log likelihood value. A discrete Gamma distribution was used to model evolutionary rate differences among sites (5 categories (+G, parameter = 0.1493)). The tree is drawn to scale, with branch lengths measured in the number of substitutions per site. The analysis involved 28 nucleotide sequences, of which 27 were used as marker sequences to determine the genotype of 1 sequence. All positions containing gaps and missing data were eliminated. There was a total of 466 positions in the final dataset. Evolutionary analyses were conducted in MEGA X.

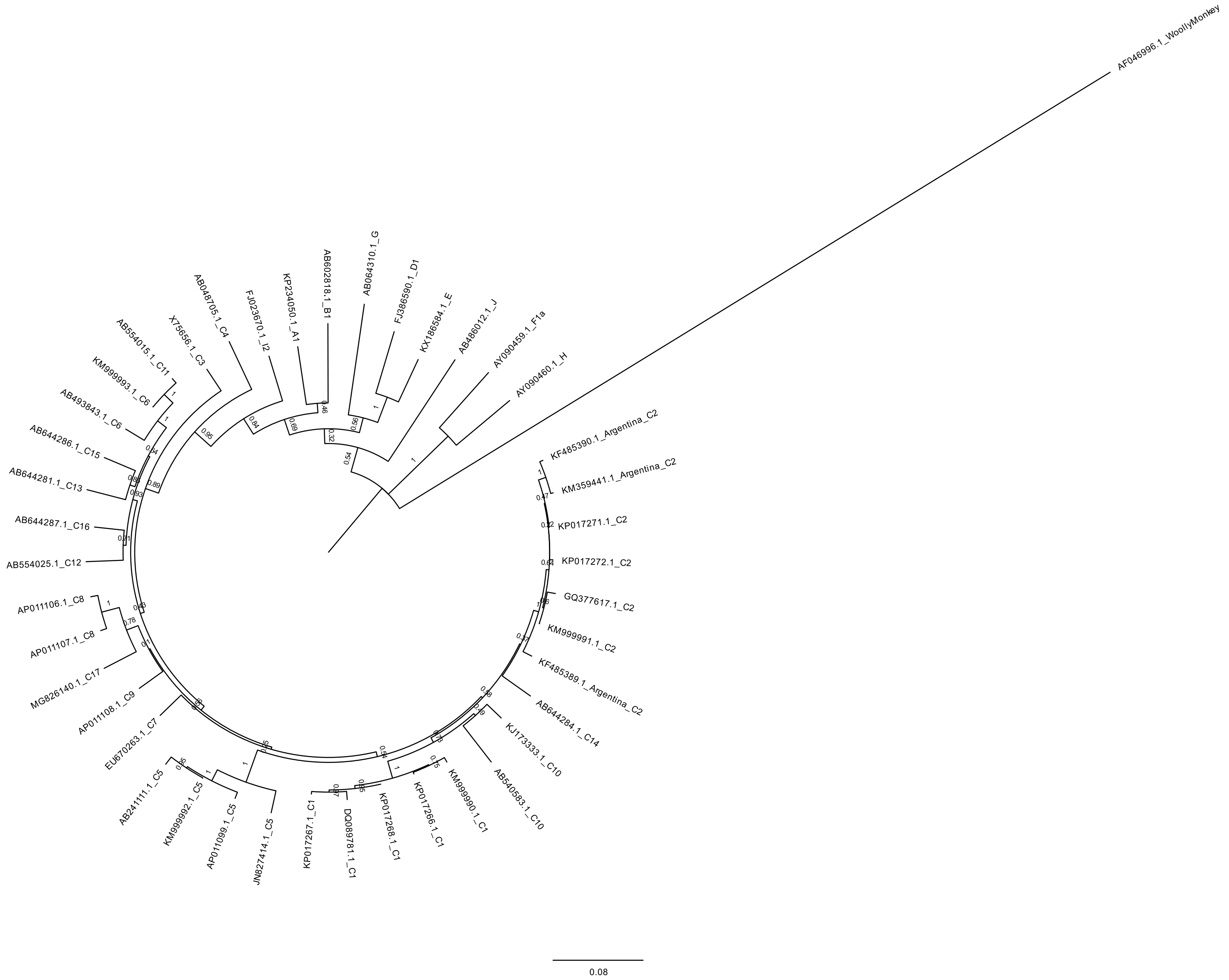

Tree 8. The evolutionary history was inferred by using the Maximum Likelihood method and Tamura-Nei model. The percentage of replicate trees in which the associated taxa clustered together in the bootstrap test (1000 replicates) are shown next to the branches. Initial tree(s) for the heuristic search were obtained automatically by applying Neighbor-Join and BioNJ algorithms to a matrix of pairwise distances estimated using the Tamura-Nei model, and then selecting the topology with superior log likelihood value. A discrete Gamma distribution was used to model evolutionary rate differences among sites (5 categories (+G, parameter = 0.2253)). The tree is drawn to scale, with branch lengths measured in the number of substitutions per site. The analysis involved 43 nucleotide sequences, of which 40 were used as marker sequences to determine the genotype of 3 sequences. All positions containing gaps and missing data were eliminated. There was a total of 3124 positions in the final dataset. Evolutionary analyses were conducted in MEGA X.

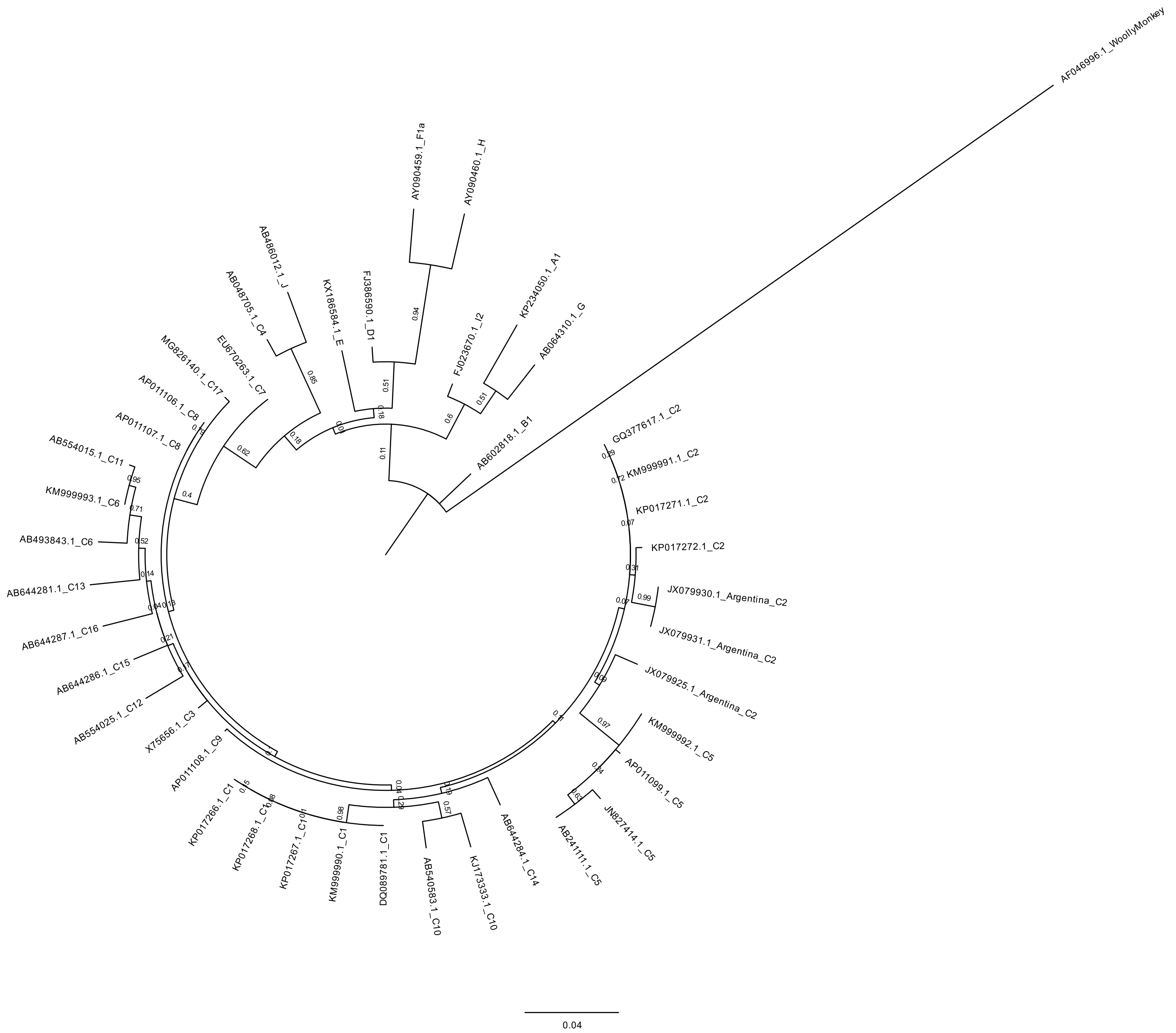

Tree 9. The evolutionary history was inferred by using the Maximum Likelihood method and Tamura-Nei model. The percentage of replicate trees in which the associated taxa clustered together in the bootstrap test (1000 replicates) are shown next to the branches. Initial tree(s) for the heuristic search were obtained automatically by applying Neighbor-Join and BioNJ algorithms to a matrix of pairwise distances estimated using the Tamura-Nei model, and then selecting the topology with superior log likelihood value. A discrete Gamma distribution was used to model evolutionary rate differences among sites (5 categories (+G, parameter = 0.1257)). The tree is drawn to scale, with branch lengths measured in the number of substitutions per site. The analysis involved 43 nucleotide sequences, of which 40 were used as marker sequences to determine the genotype of 3 sequences. All positions containing gaps and missing data were eliminated. There was a total of 485 positions in the final dataset. Evolutionary analyses were conducted in MEGA X.

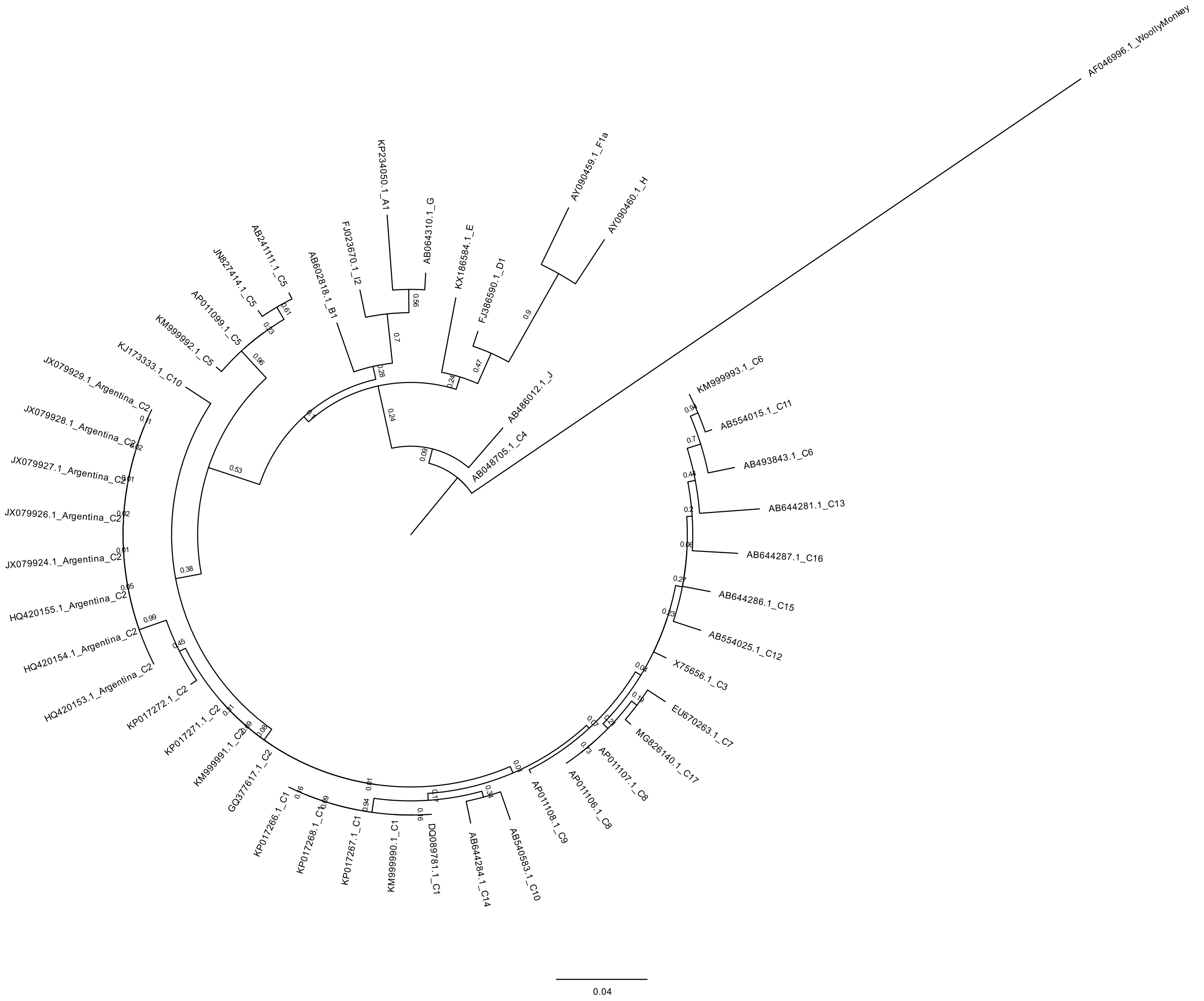

Tree 10. The evolutionary history was inferred by using the Maximum Likelihood method and Tamura-Nei model. The percentage of replicate trees in which the associated taxa clustered together in the bootstrap test (1000 replicates) are shown next to the branches. Initial tree(s) for the heuristic search were obtained automatically by applying Neighbor-Join and BioNJ algorithms to a matrix of pairwise distances estimated using the Tamura-Nei model, and then selecting the topology with superior log likelihood value. A discrete Gamma distribution was used to model evolutionary rate differences among sites (5 categories (+G, parameter = 0.1248)). The tree is drawn to scale, with branch lengths measured in the number of substitutions per site. The analysis involved 48 nucleotide sequences, of which 40 were used as marker sequences to determine the genotype of 8 sequences. All positions containing gaps and missing data were eliminated. There was a total of 415 positions in the final dataset. Evolutionary analyses were conducted in MEGA X.

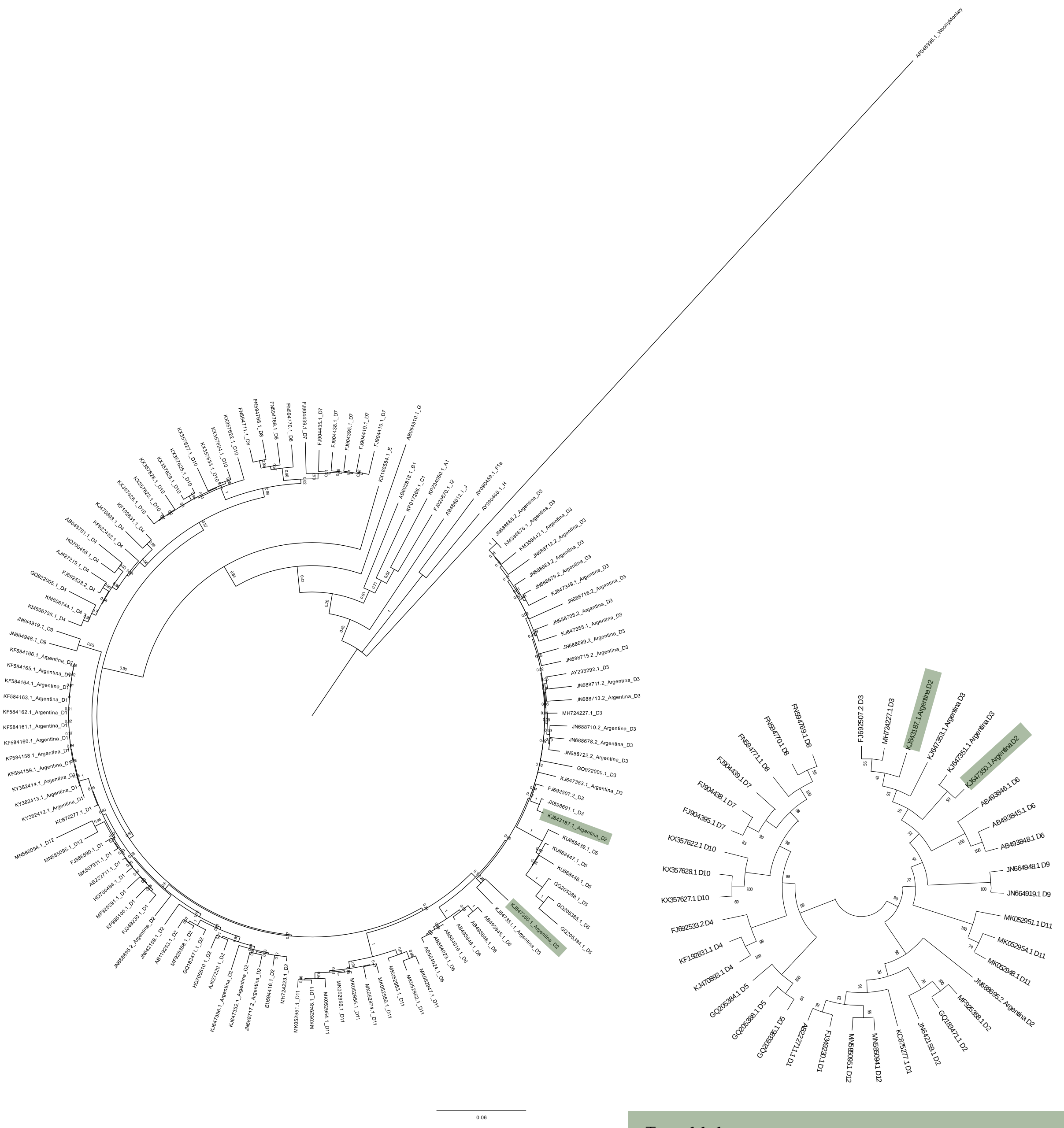

Tree 11.1

Tree 11. The evolutionary history was inferred by using the Maximum Likelihood method and Tamura-Nei model. The percentage of replicate trees in which the associated taxa clustered together in the bootstrap test (1000 replicates) are shown next to the branches. Initial tree(s) for the heuristic search were obtained automatically by applying Neighbor-Join and BioNJ algorithms to a matrix of pairwise distances estimated using the Tamura-Nei model, and then selecting the topology with superior log likelihood value. A discrete Gamma distribution was used to model evolutionary rate differences among sites (5 categories (+G, parameter = 0.2720)). The tree is drawn to scale, with branch lengths measured in the number of substitutions per site. The analysis involved 123 nucleotide sequences, of which 86 were used as marker sequences to determine the genotype of 37 sequences. Sequences KJ647350 and KJ843187 were reclassified as subgenotype D3. All positions containing gaps and missing data were eliminated. There was a total of 2808 positions in the final dataset. Evolutionary analyses were conducted in MEGA X.

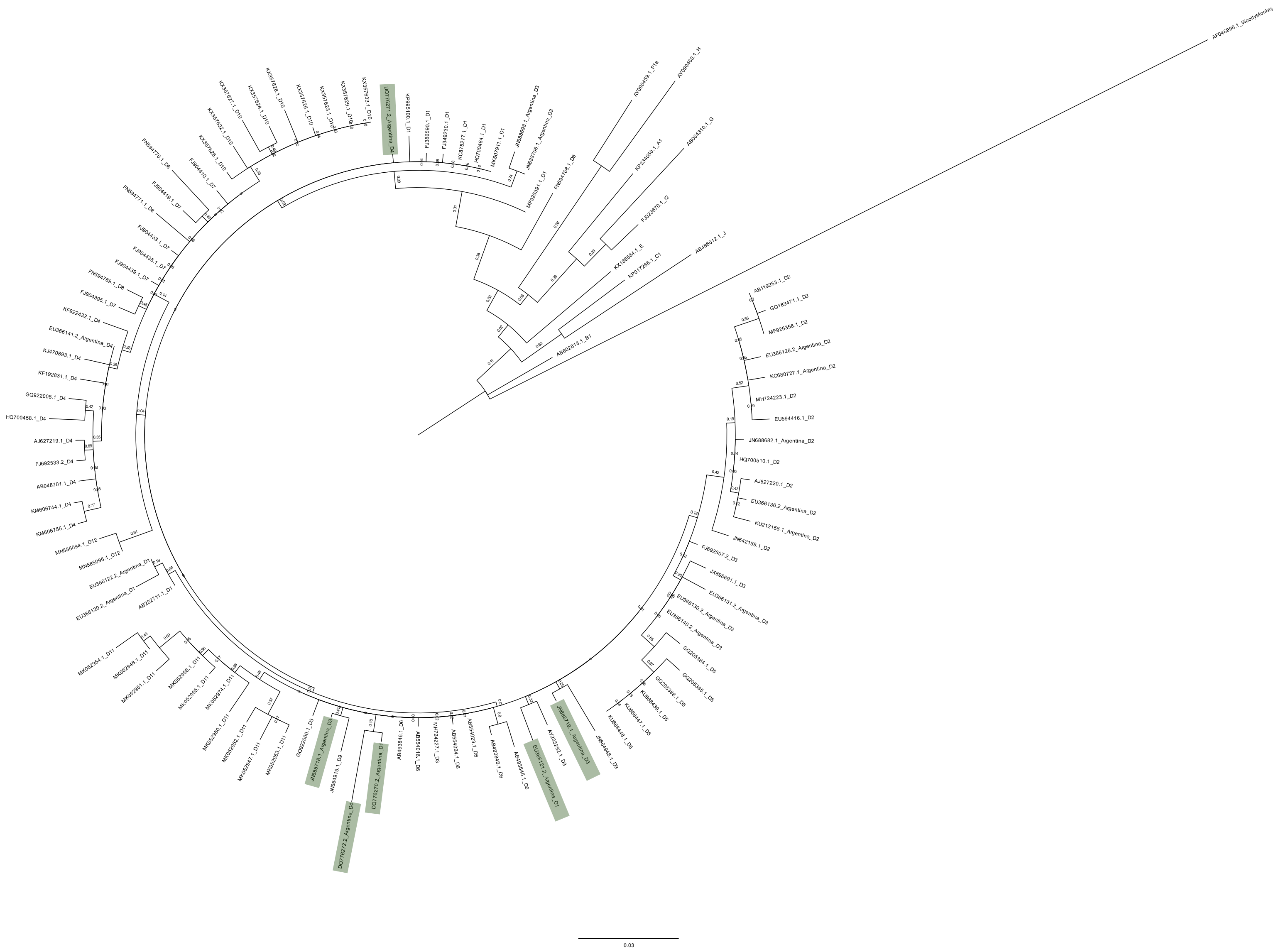

Tree 12. The evolutionary history was inferred by using the Maximum Likelihood method and Tamura-Nei model. The percentage of replicate trees in which the associated taxa clustered together in the bootstrap test (1000 replicates) are shown next to the branches. Initial tree(s) for the heuristic search were obtained automatically by applying Neighbor-Join and BioNJ algorithms to a matrix of pairwise distances estimated using the Tamura-Nei model, and then selecting the topology with superior log likelihood value. A discrete Gamma distribution was used to model evolutionary rate differences among sites (5 categories (+G, parameter = 0.2159)). The tree is drawn to scale, with branch lengths measured in the number of substitutions per site. The analysis involved 105 nucleotide sequences, of which 86 were used as marker sequences to determine the genotype of 19 sequences. Six sequences were reclassified due to posterior phylogenetic analysis. All positions containing gaps and missing data were eliminated. There was a total of 499 positions in the final dataset. Evolutionary analyses were conducted in MEGA X.

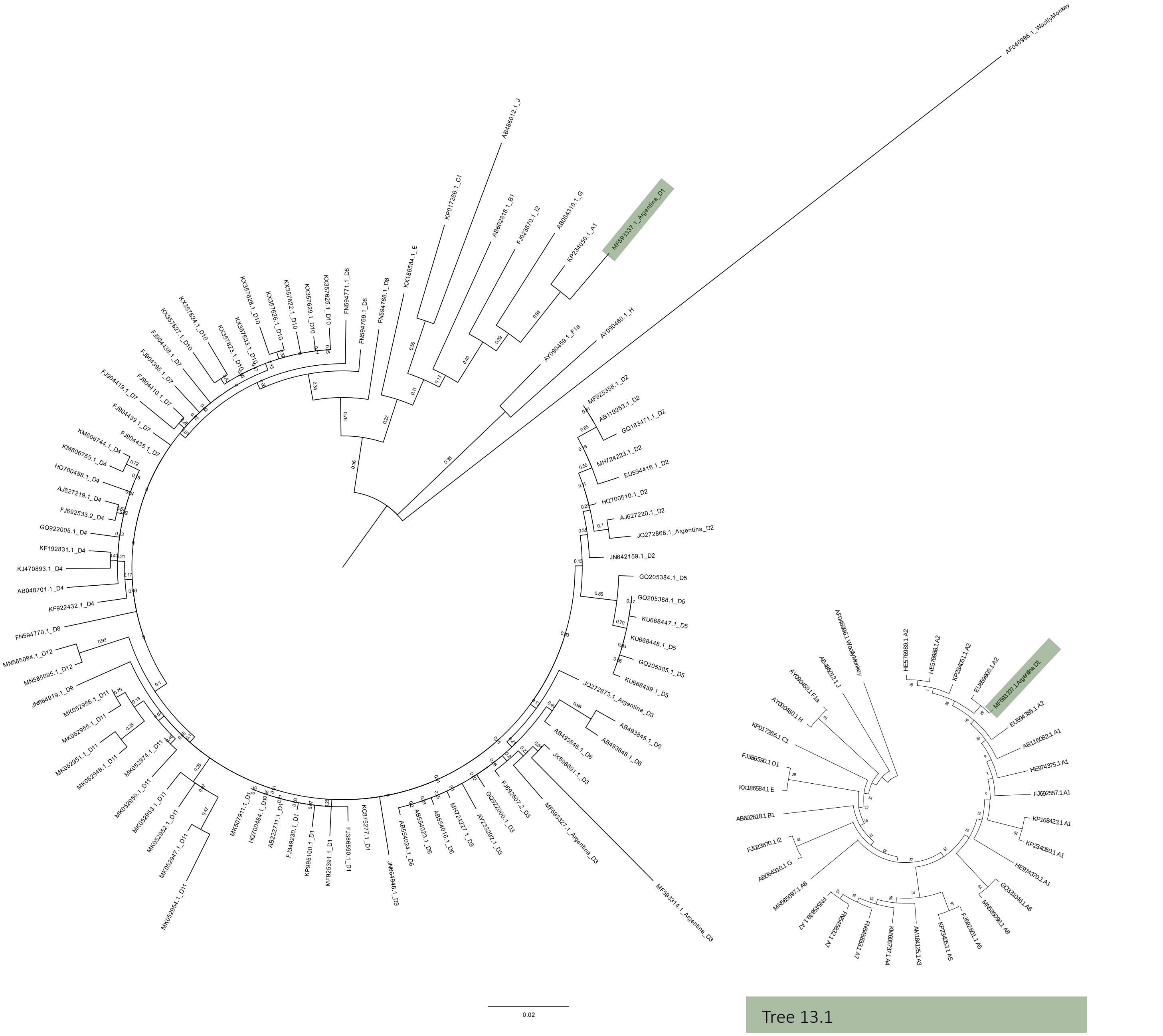

Tree 13.1

Tree 13. The evolutionary history was inferred by using the Maximum Likelihood method and Tamura-Nei model. The percentage of replicate trees in which the associated taxa clustered together in the bootstrap test (1000 replicates) are shown next to the branches. Initial tree(s) for the heuristic search were obtained automatically by applying Neighbor-Join and BioNJ algorithms to a matrix of pairwise distances estimated using the Tamura-Nei model, and then selecting the topology with superior log likelihood value. A discrete Gamma distribution was used to model evolutionary rate differences among sites (5 categories (+G, parameter = 0.2588)). The tree is drawn to scale, with branch lengths measured in the number of substitutions per site. The analysis involved 91 nucleotide sequences, of which 86 were used as marker sequences to determine the genotype of 5 sequences. Sequence MF593337 aligned with outgroup A, and upon further analysis (Tree 13.1) was reclassified as subgenotype A2. All positions containing gaps and missing data were eliminated. There was a total of 652 positions in the final dataset. Evolutionary analyses were conducted in MEGA X.

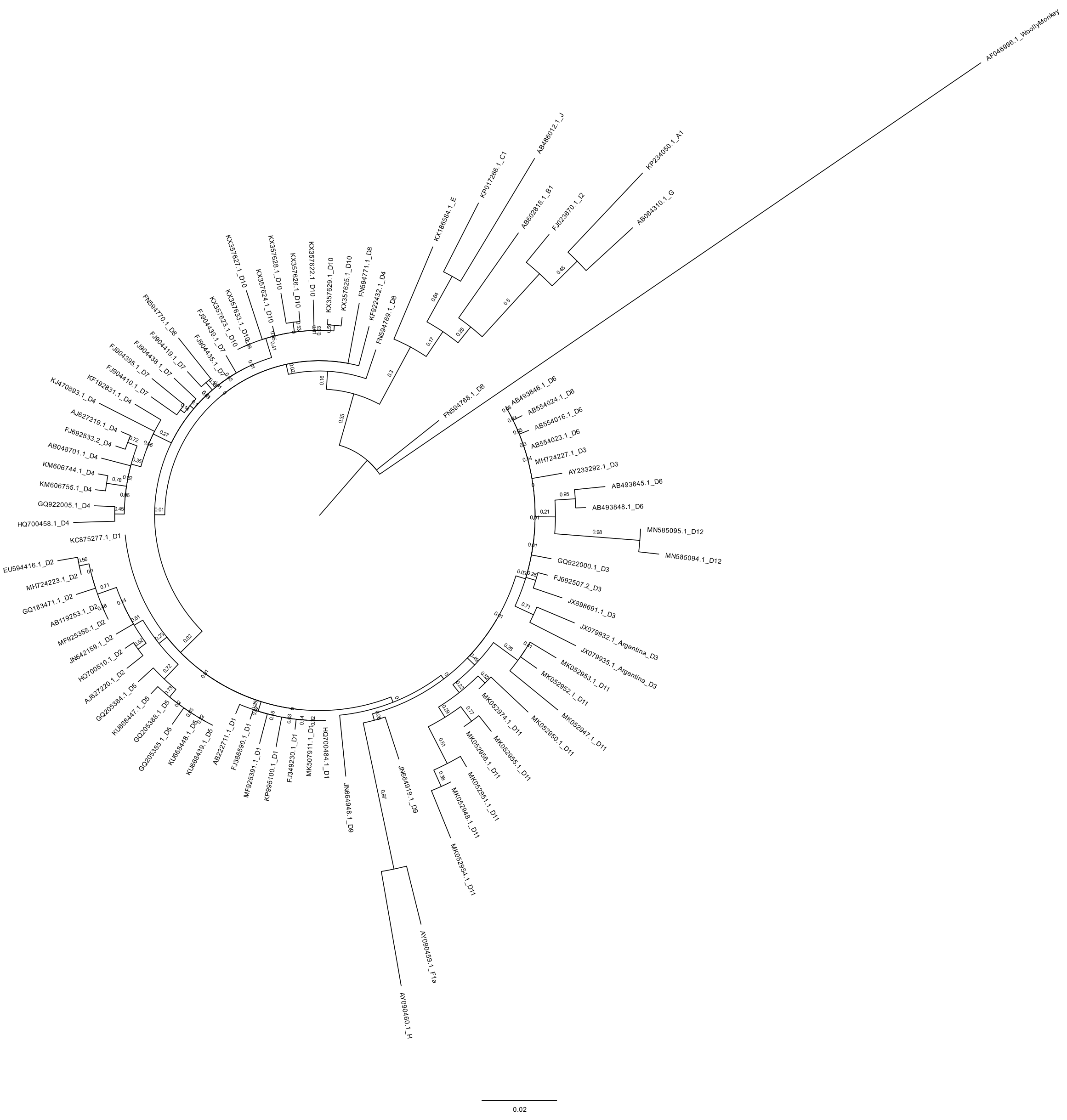

Tree 14. The evolutionary history was inferred by using the Maximum Likelihood method and Tamura-Nei model. The percentage of replicate trees in which the associated taxa clustered together in the bootstrap test (1000 replicates) are shown next to the branches. Initial tree(s) for the heuristic search were obtained automatically by applying Neighbor-Join and BioNJ algorithms to a matrix of pairwise distances estimated using the Tamura-Nei model, and then selecting the topology with superior log likelihood value. A discrete Gamma distribution was used to model evolutionary rate differences among sites (5 categories (+G, parameter = 0.2507)). The tree is drawn to scale, with branch lengths measured in the number of substitutions per site. The analysis involved 88 nucleotide sequences, of which 86 were used as marker sequences to determine the genotype of 2 sequences. All positions containing gaps and missing data were eliminated. There was a total of 460 positions in the final dataset. Evolutionary analyses were conducted in MEGA X.

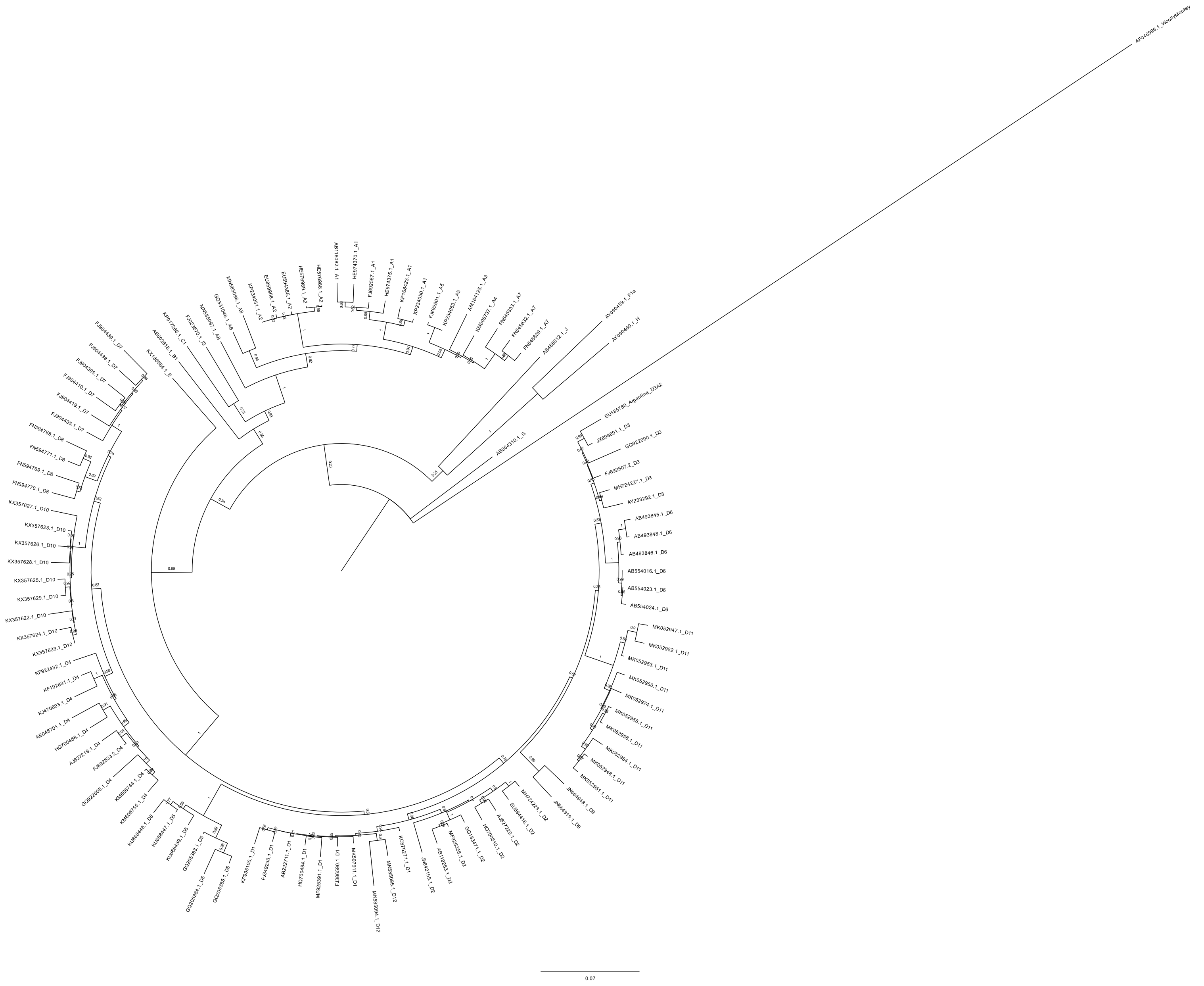

Tree 15. The evolutionary history was inferred by using the Maximum Likelihood method and Tamura-Nei model. The percentage of replicate trees in which the associated taxa clustered together in the bootstrap test (1000 replicates) are shown next to the branches. Initial tree(s) for the heuristic search were obtained automatically by applying Neighbor-Join and BioNJ algorithms to a matrix of pairwise distances estimated using the Tamura-Nei model, and then selecting the topology with superior log likelihood value. A discrete Gamma distribution was used to model evolutionary rate differences among sites (5 categories (+G, parameter = 0.2707)). The tree is drawn to scale, with branch lengths measured in the number of substitutions per site. The analysis involved 87 nucleotide sequences, of which 86 were used as marker sequences to determine the genotype of 1 sequence originally reported as a D3/A2 recombinant. All positions containing gaps and missing data were eliminated. There was a total of 2980 positions in the final dataset. Evolutionary analyses were conducted in MEGA X.

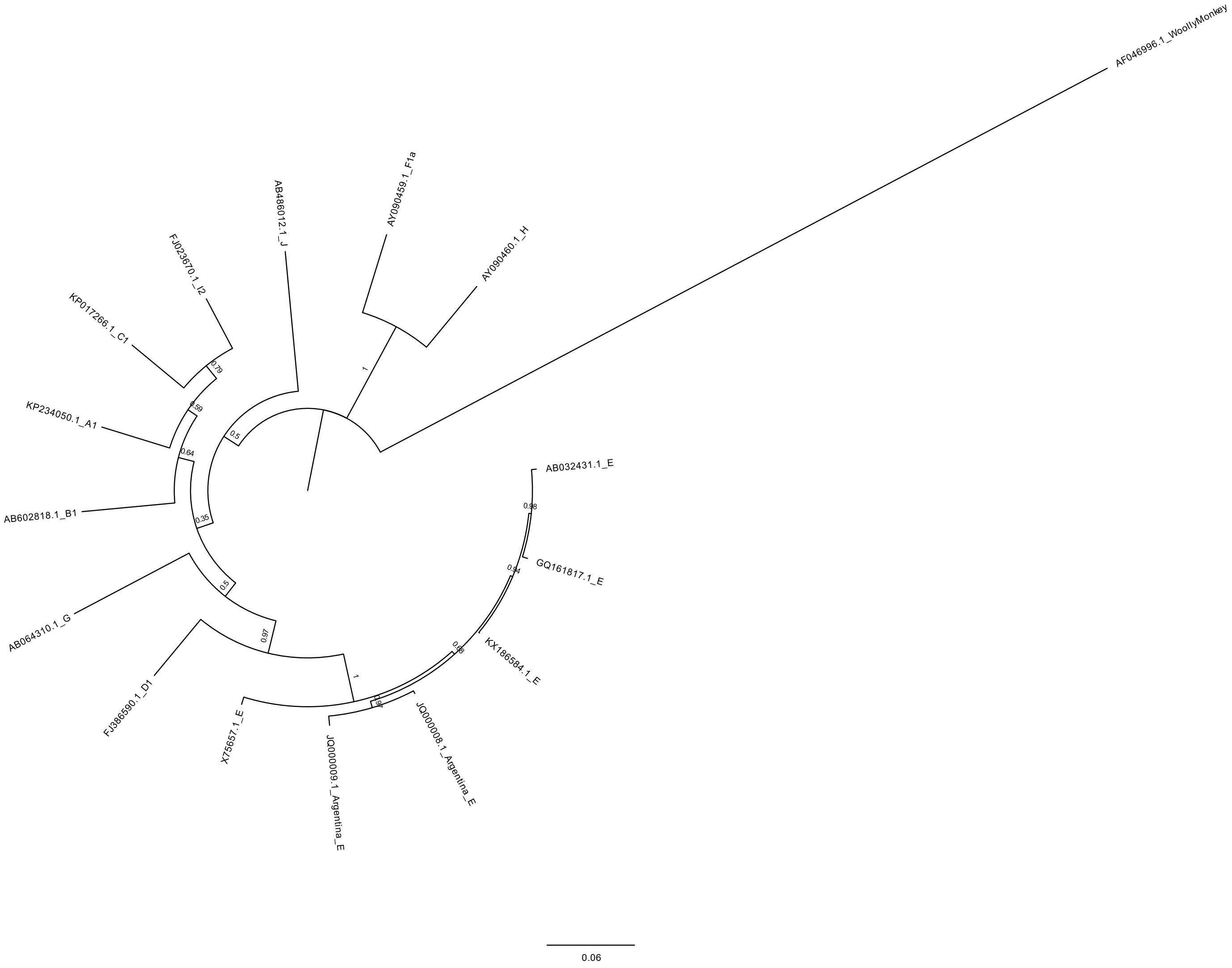

Tree 16. The evolutionary history was inferred by using the Maximum Likelihood method and Tamura-Nei model. The percentage of replicate trees in which the associated taxa clustered together in the bootstrap test (1000 replicates) are shown next to the branches. Initial tree(s) for the heuristic search were obtained automatically by applying Neighbor-Join and BioNJ algorithms to a matrix of pairwise distances estimated using the Tamura-Nei model, and then selecting the topology with superior log likelihood value. A discrete Gamma distribution was used to model evolutionary rate differences among sites (5 categories (+G, parameter = 0.2297)). The tree is drawn to scale, with branch lengths measured in the number of substitutions per site. The analysis involved 16 nucleotide sequences, of which 14 were used as marker sequences to determine the genotype of 2 sequences. All positions containing gaps and missing data were eliminated. There was a total of 3170 positions in the final dataset. Evolutionary analyses were conducted in MEGA X.

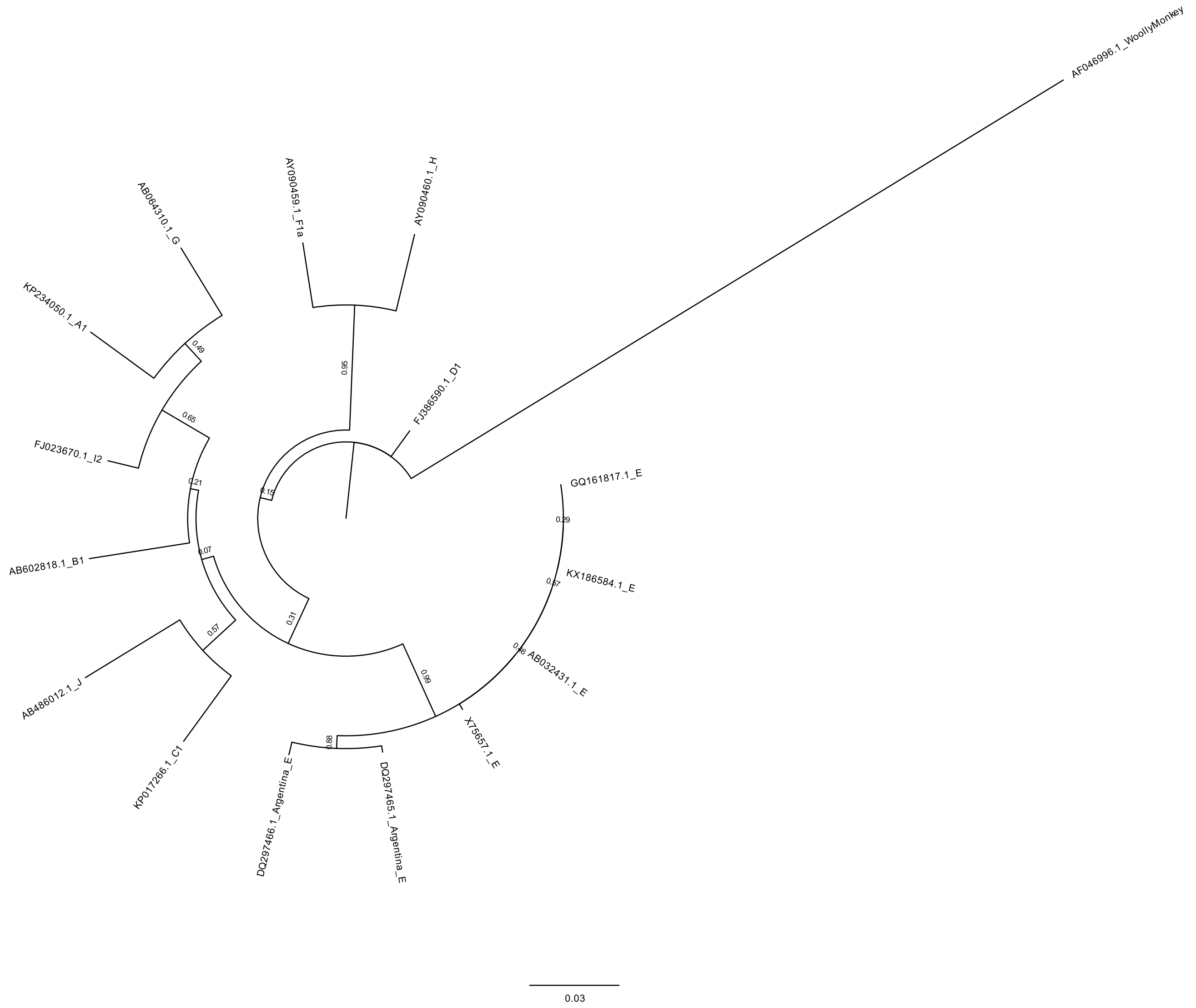

Tree 17. The evolutionary history was inferred by using the Maximum Likelihood method and Tamura-Nei model. The percentage of replicate trees in which the associated taxa clustered together in the bootstrap test (1000 replicates) are shown next to the branches. Initial tree(s) for the heuristic search were obtained automatically by applying Neighbor-Join and BioNJ algorithms to a matrix of pairwise distances estimated using the Tamura-Nei model, and then selecting the topology with superior log likelihood value. A discrete Gamma distribution was used to model evolutionary rate differences among sites (5 categories (+G, parameter = 0.1355)). The tree is drawn to scale, with branch lengths measured in the number of substitutions per site. The analysis involved 16 nucleotide sequences, of which 14 were used as marker sequences to determine the genotype of 2 sequences. All positions containing gaps and missing data were eliminated. There was a total of 513 positions in the final dataset. Evolutionary analyses were conducted in MEGA X.

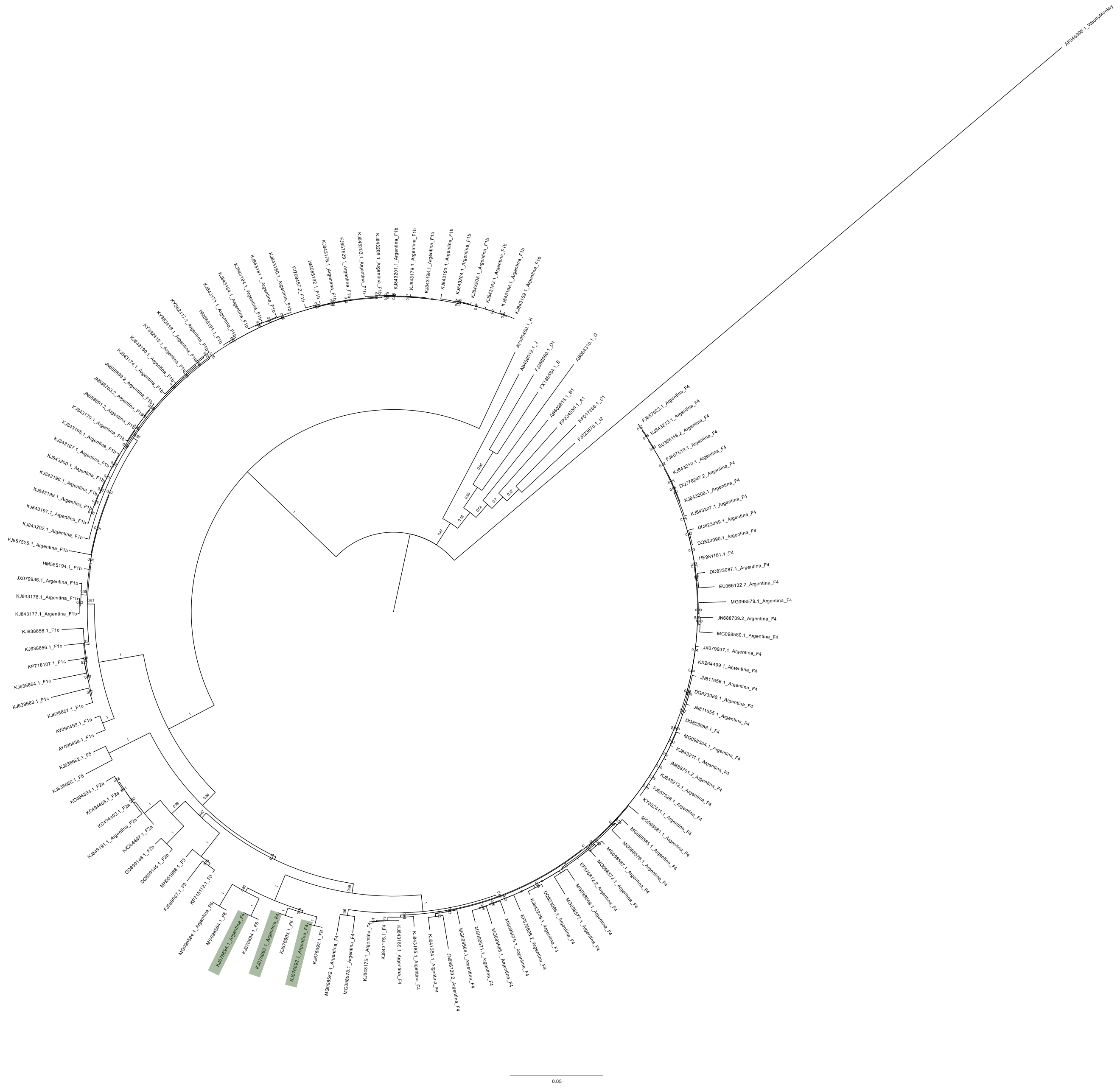

Tree 18. The evolutionary history was inferred by using the Maximum Likelihood method and Tamura-Nei model. The percentage of replicate trees in which the associated taxa clustered together in the bootstrap test (1000 replicates) are shown next to the branches. Initial tree(s) for the heuristic search were obtained automatically by applying Neighbor-Join and BioNJ algorithms to a matrix of pairwise distances estimated using the Tamura-Nei model, and then selecting the topology with superior log likelihood value. A discrete Gamma distribution was used to model evolutionary rate differences among sites (5 categories (+G, parameter = 0.2972)). The tree is drawn to scale, with branch lengths measured in the number of substitutions per site. The analysis involved 131 nucleotide sequences, of which 40 were used as marker sequences to determine the genotype of 91 sequences. Sequences KJ676692, KJ676693, and KJ676694 were all reclassified as subgenotype F6, as at the time they were originally propositioned as an F4 variant which would later become F6. All positions containing gaps and missing data were eliminated. There was a total of 2922 positions in the final dataset. Evolutionary analyses were conducted in MEGA X.

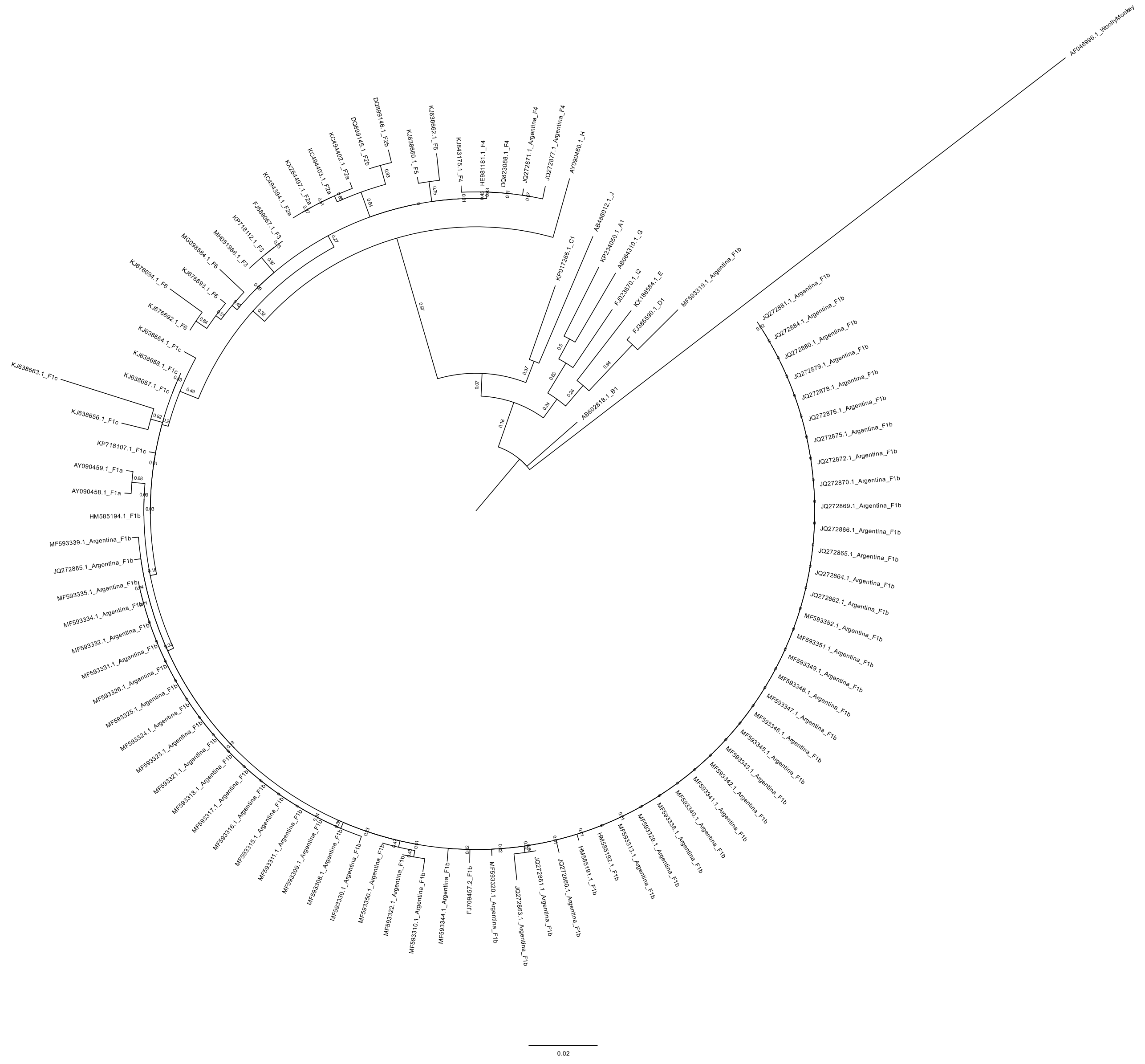

Tree 19. The evolutionary history was inferred by using the Maximum Likelihood method and Tamura-Nei model. The percentage of replicate trees in which the associated taxa clustered together in the bootstrap test (1000 replicates) are shown next to the branches. Initial tree(s) for the heuristic search were obtained automatically by applying Neighbor-Join and BioNJ algorithms to a matrix of pairwise distances estimated using the Tamura-Nei model, and then selecting the topology with superior log likelihood value. A discrete Gamma distribution was used to model evolutionary rate differences among sites (5 categories (+G, parameter = 0.1813)). The tree is drawn to scale, with branch lengths measured in the number of substitutions per site. The analysis involved 98 nucleotide sequences, of which 40 were used as marker sequences to determine the genotype of 58 sequences. All positions containing gaps and missing data were eliminated. There was a total of 585 positions in the final dataset. Evolutionary analyses were conducted in MEGA X.

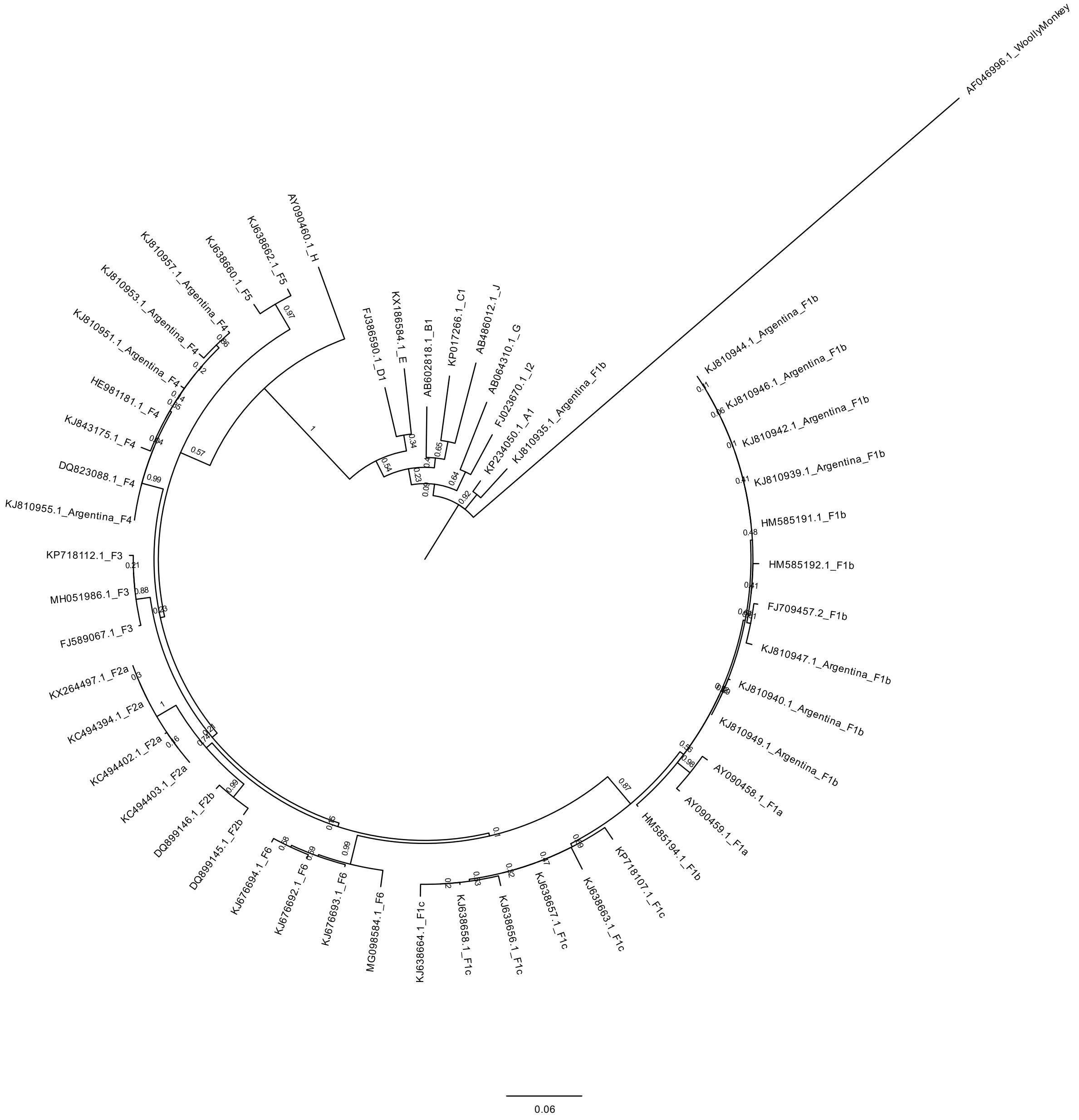

Tree 20. The evolutionary history was inferred by using the Maximum Likelihood method and Tamura-Nei model. The percentage of replicate trees in which the associated taxa clustered together in the bootstrap test (1000 replicates) are shown next to the branches. Initial tree(s) for the heuristic search were obtained automatically by applying Neighbor-Join and BioNJ algorithms to a matrix of pairwise distances estimated using the Tamura-Nei model, and then selecting the topology with superior log likelihood value. A discrete Gamma distribution was used to model evolutionary rate differences among sites (5 categories (+G, parameter = 0.1941)). The tree is drawn to scale, with branch lengths measured in the number of substitutions per site. The analysis involved 52 nucleotide sequences, of which 40 were used as marker sequences to determine the genotype of 12 sequences. All positions containing gaps and missing data were eliminated. There was a total of 773 positions in the final dataset. Evolutionary analyses were conducted in MEGA X.

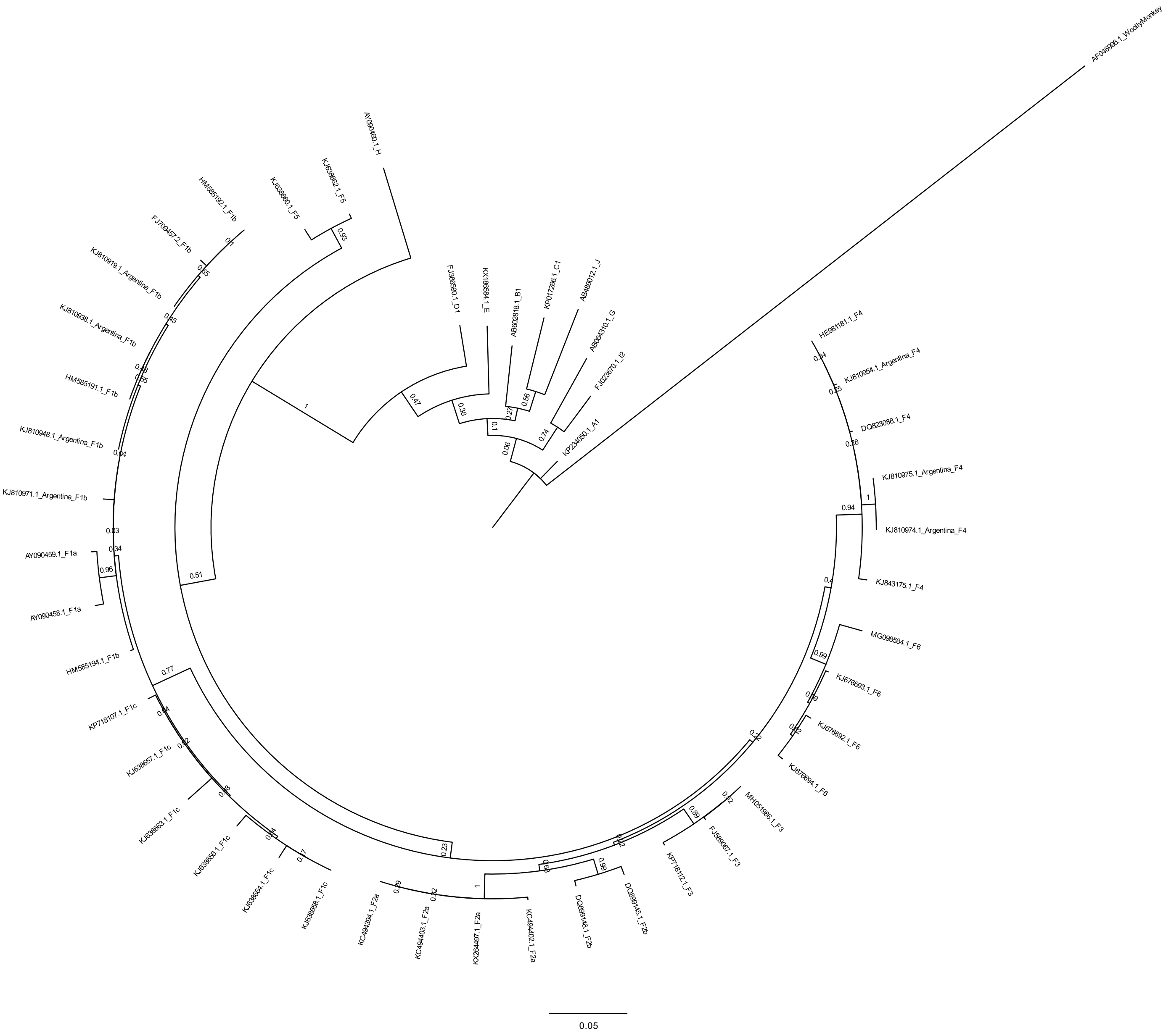

Tree 21. The evolutionary history was inferred by using the Maximum Likelihood method and Tamura-Nei model. The percentage of replicate trees in which the associated taxa clustered together in the bootstrap test (1000 replicates) are shown next to the branches. Initial tree(s) for the heuristic search were obtained automatically by applying Neighbor-Join and BioNJ algorithms to a matrix of pairwise distances estimated using the Tamura-Nei model, and then selecting the topology with superior log likelihood value. A discrete Gamma distribution was used to model evolutionary rate differences among sites (5 categories (+G, parameter = 0.1792)). The tree is drawn to scale, with branch lengths measured in the number of substitutions per site. The analysis involved 47 nucleotide sequences, of which 40 were used as marker sequences to determine the genotype of 7 sequences. All positions containing gaps and missing data were eliminated. There was a total of 668 positions in the final dataset. Evolutionary analyses were conducted in MEGA X.

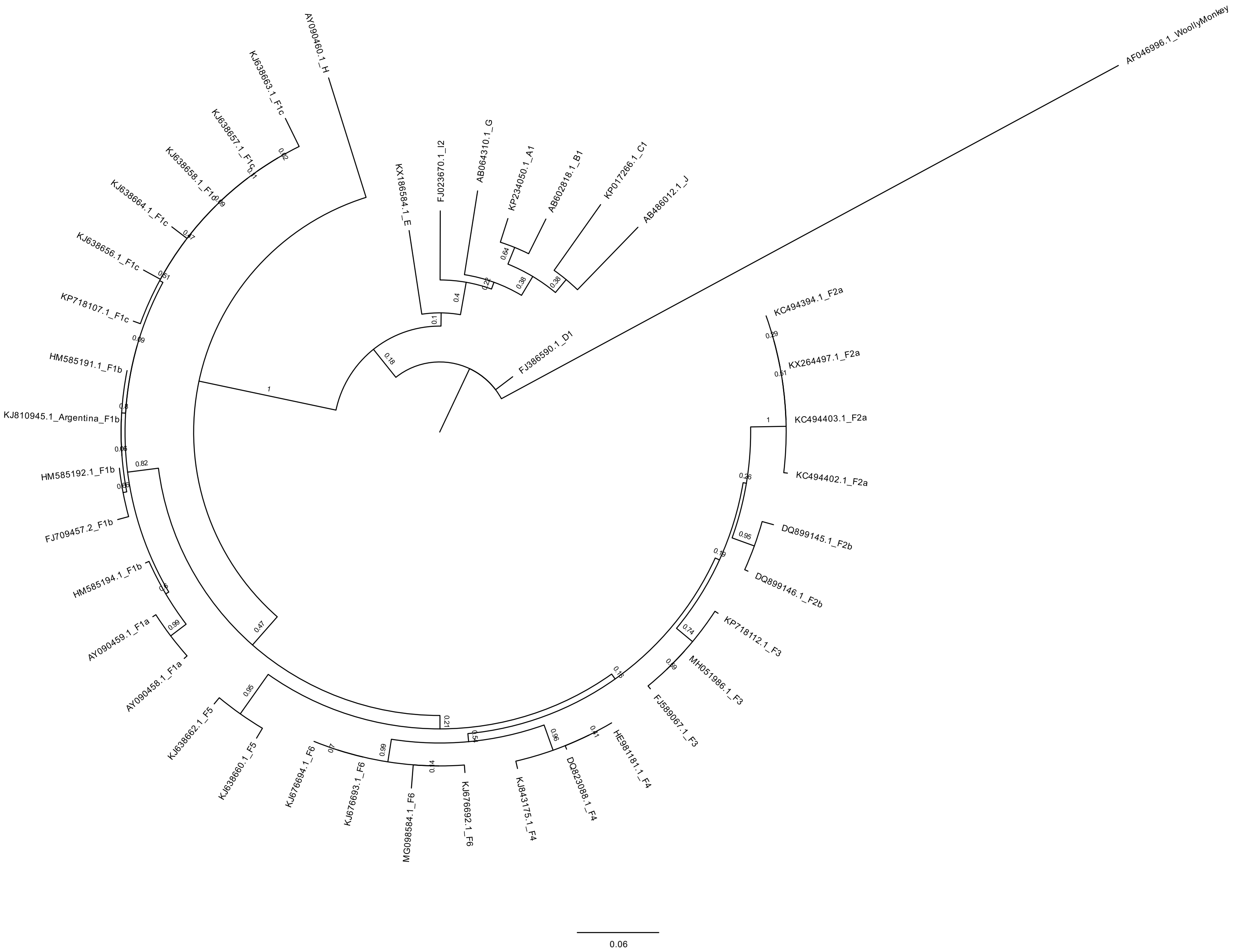

Tree 22. The evolutionary history was inferred by using the Maximum Likelihood method and Tamura-Nei model. The percentage of replicate trees in which the associated taxa clustered together in the bootstrap test (1000 replicates) are shown next to the branches. Initial tree(s) for the heuristic search were obtained automatically by applying Neighbor-Join and BioNJ algorithms to a matrix of pairwise distances estimated using the Tamura-Nei model, and then selecting the topology with superior log likelihood value. A discrete Gamma distribution was used to model evolutionary rate differences among sites (5 categories (+G, parameter = 0.1931)). The tree is drawn to scale, with branch lengths measured in the number of substitutions per site. The analysis involved 41 nucleotide sequences, of which 40 were used as marker sequences to determine the genotype of 1 sequence. All positions containing gaps and missing data were eliminated. There was a total of 420 positions in the final dataset. Evolutionary analyses were conducted in MEGA X.

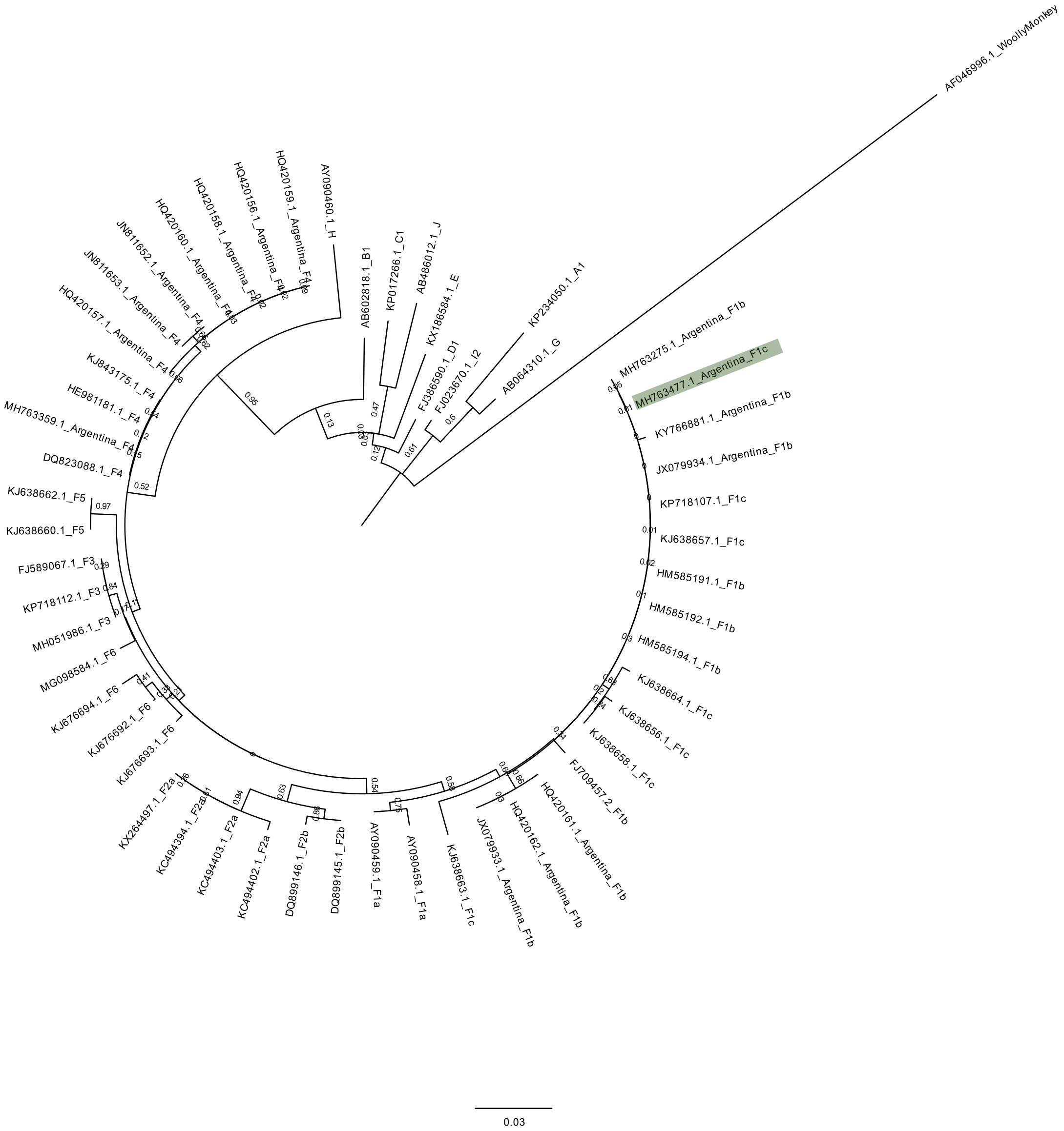

Tree 23. The evolutionary history was inferred by using the Maximum Likelihood method and Tamura-Nei model. The percentage of replicate trees in which the associated taxa clustered together in the bootstrap test (1000 replicates) are shown next to the branches. Initial tree(s) for the heuristic search were obtained automatically by applying Neighbor-Join and BioNJ algorithms to a matrix of pairwise distances estimated using the Tamura-Nei model, and then selecting the topology with superior log likelihood value. A discrete Gamma distribution was used to model evolutionary rate differences among sites (5 categories (+G, parameter = 0.1563)). The tree is drawn to scale, with branch lengths measured in the number of substitutions per site. The analysis involved 55 nucleotide sequences, of which 40 were used as marker sequences to determine the genotype of 15 sequences. Sequences of the F1 category exhibited uncertain positioning, for which they were inputted into further phylogenetic analysis resulting in the reclassification of sequence MH763477 as F1b. All positions containing gaps and missing data were eliminated. There was a total of 353 positions in the final dataset. Evolutionary analyses were conducted in MEGA X.

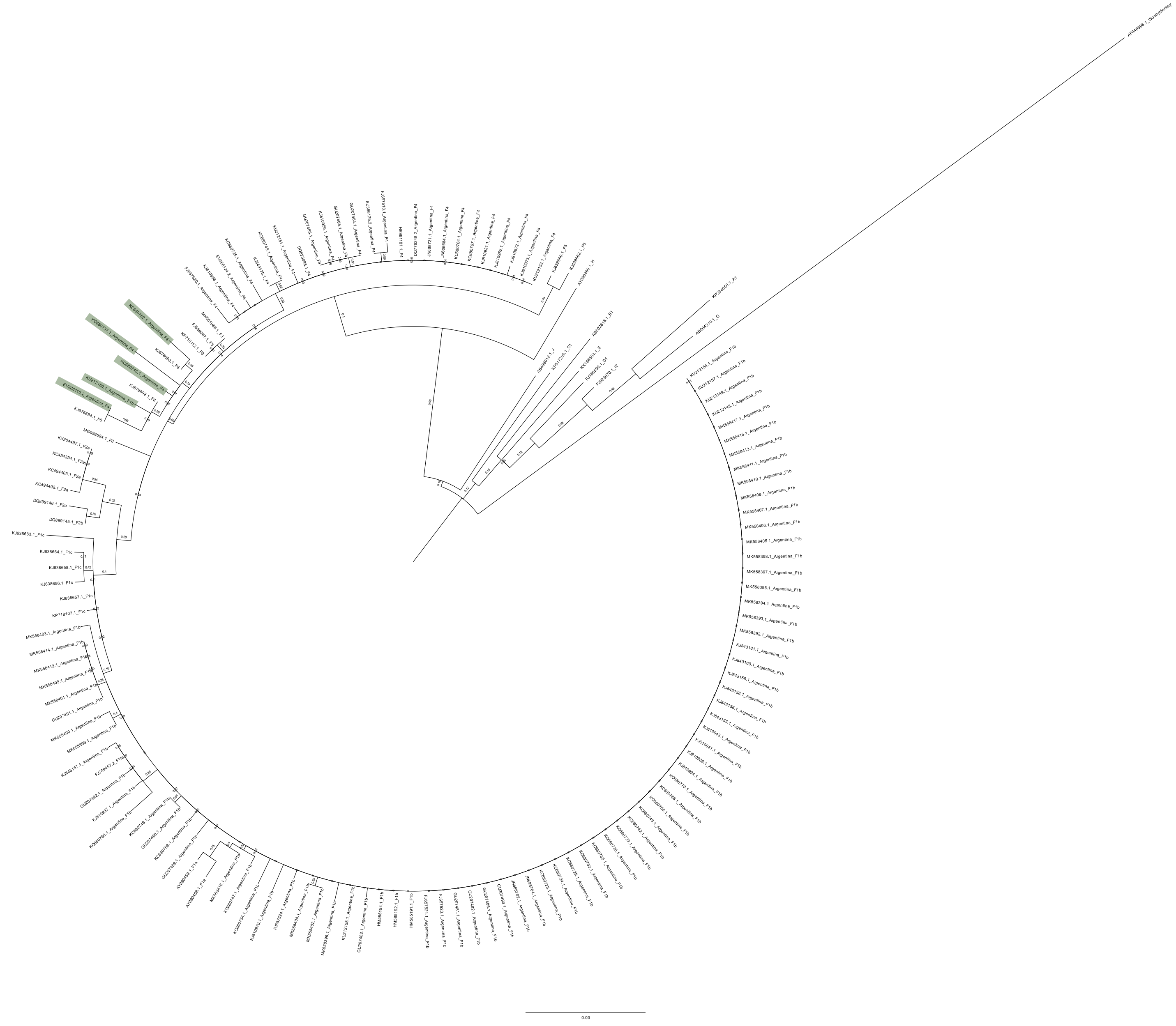

Tree 24. The evolutionary history was inferred by using the Maximum Likelihood method and Tamura-Nei model. The percentage of replicate trees in which the associated taxa clustered together in the bootstrap test (1000 replicates) are shown next to the branches. Initial tree(s) for the heuristic search were obtained automatically by applying Neighbor-Join and BioNJ algorithms to a matrix of pairwise distances estimated using the Tamura-Nei model, and then selecting the topology with superior log likelihood value. A discrete Gamma distribution was used to model evolutionary rate differences among sites (5 categories (+G, parameter = 0.1601)). The tree is drawn to scale, with branch lengths measured in the number of substitutions per site. The analysis involved 142 nucleotide sequences, of which 40 were used as marker sequences to determine the genotype of 102 sequences. Six sequences were reclassified as subgenotype F6. All positions containing gaps and missing data were eliminated. There was a total of 477 positions in the final dataset. Evolutionary analyses were conducted in MEGA X.

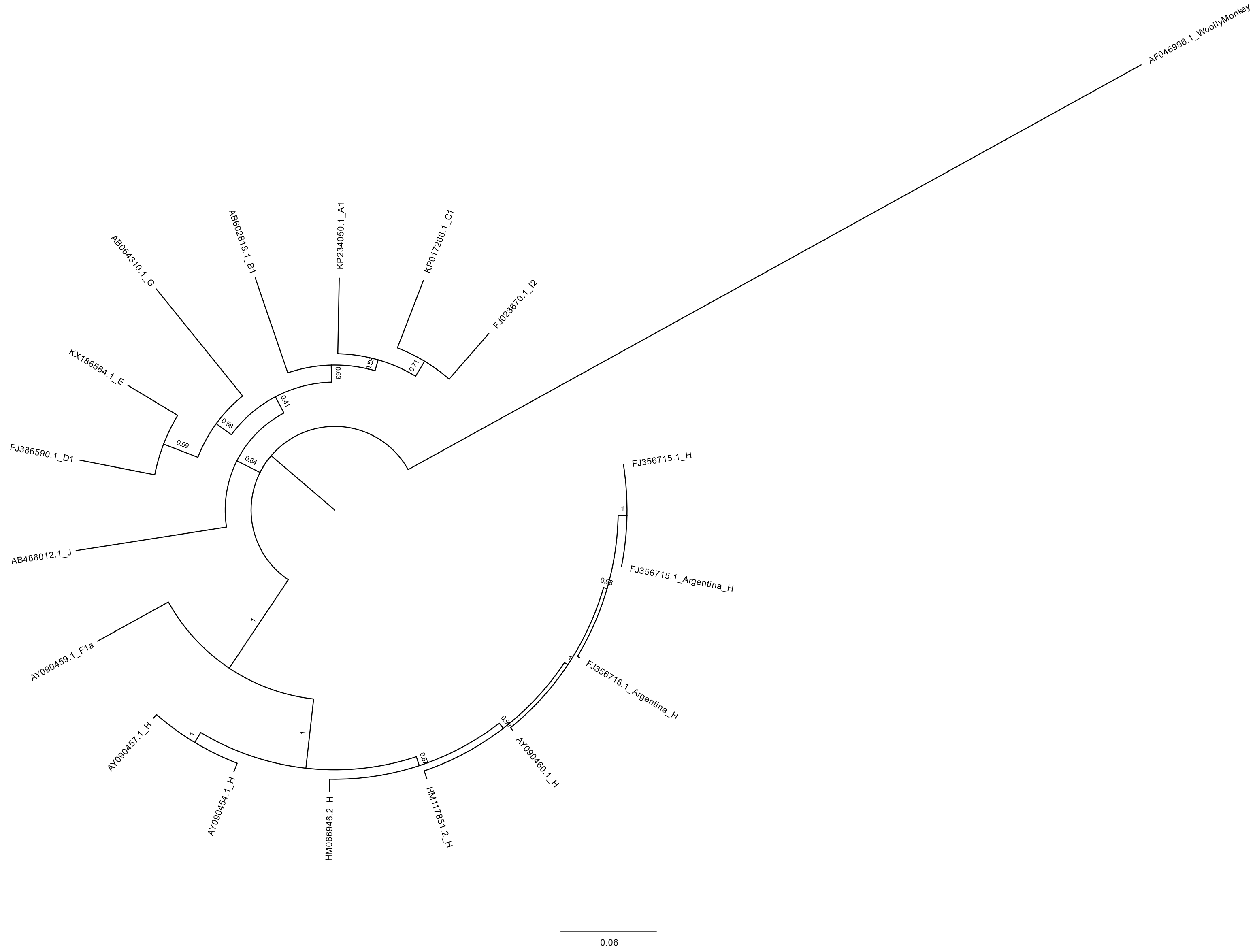

Tree 25. The evolutionary history was inferred by using the Maximum Likelihood method and Tamura-Nei model. The percentage of replicate trees in which the associated taxa clustered together in the bootstrap test (1000 replicates) are shown next to the branches. Initial tree(s) for the heuristic search were obtained automatically by applying Neighbor-Join and BioNJ algorithms to a matrix of pairwise distances estimated using the Tamura-Nei model, and then selecting the topology with superior log likelihood value. A discrete Gamma distribution was used to model evolutionary rate differences among sites (5 categories (+G, parameter = 0.2370)). The tree is drawn to scale, with branch lengths measured in the number of substitutions per site. The analysis involved 18 nucleotide sequences, of which 16 were used as marker sequences to determine the genotype of 2 sequences. All positions containing gaps and missing data were eliminated. There was a total of 3165 positions in the final dataset. Evolutionary analyses were conducted in MEGA X.

ID	GENOTYPE	SUBTYPE	COUNTRY	TREE	ALIGNMENT[1]	BASE PAIRS
AF043560	A	A1	Argentina	1	Complete Genome	3221
AF043580	A	A2	Argentina	1	Complete Genome	3221

EU366129	A	A1	Argentina	1	Complete Genome	3221
KJ843166	A	A2	Argentina	1	Complete Genome	3221
KJ843172	A	A2	Argentina	1	Complete Genome	3221
KJ843173	A	A2	Argentina	1	Complete Genome	3221
KJ843182	A	A2	Argentina	1	Complete Genome	3221
KJ843183	A	A1	Argentina	1	Complete Genome	3221
KJ843184	A	A2	Argentina	1	Complete Genome	3221
KJ843186	A	A2	Argentina	1	Complete Genome	3221
KJ843188	A	A2	Argentina	1	Complete Genome	3221
KJ843192	A	A2	Argentina	1	Complete Genome	3221
KJ843214	A	A2	Argentina	1	Complete Genome	3221
KJ843215	A	A2	Argentina	1	Complete Genome	3221
KJ843216	A	A2	Argentina	1	Complete Genome	3221
KJ843217	A	A2	Argentina	1	Complete Genome	3221
KJ843218	A	A2	Argentina	1	Complete Genome	3221
KY382410	A	A2	Argentina	1	Complete Genome	3221
EU304330	A	A2	Argentina	2	253-782	530
EU304332	A	A2	Argentina	2	253-782	530
EU304333	A	A2	Argentina	2	252-782	531
EU366114	A	A2	Argentina	2	244-768	525
EU366117	A	A2	Argentina	2	244-768	525
EU366119	A	A2	Argentina	2	244-768	525
EU366123	A	A2	Argentina	2	244-768	525
EU366128	A	A2	Argentina	2	244-768	525
EU366134	A	A2	Argentina	2	244-768	525
EU366135	A	A2	Argentina	2	244-768	525
EU366137	A	A2	Argentina	2	244-768	525
EU366138	A	A2	Argentina	2	244-768	525
EU366139	A	A2	Argentina	2	244-768	525
JN800448	A	A2	Argentina	2	205-788	584
JN800449	A	A2	Argentina	2	205-788	584
JN800450	A	A2	Argentina	2	205-788	584
JN800451	A	A2	Argentina	2	205-788	584
JN800452	A	A2	Argentina	2	205-788	584
JN800453	A	A2	Argentina	2	205-788	584
JN800454	A	A2	Argentina	2	205-788	584
JN800455	A	A2	Argentina	2	205-788	584
JN800456	A	A2	Argentina	2	205-788	584
JN800457	A	A2	Argentina	2	205-788	584
JN800458	A	A2	Argentina	2	205-788	584
JN800459	A	A2	Argentina	2	205-788	584

JN800460	A	A2	Argentina	2	205-788	584
JN800461	A	A2	Argentina	2	205-788	584
JN800462	A	A2	Argentina	2	205-788	584
JN800463	A	A2	Argentina	2	205-788	584
JN800464	A	A2	Argentina	2	205-788	584
JN800465	A	A2	Argentina	2	205-788	584
JN800466	A	A2	Argentina	2	205-788	584
JN800467	A	A2	Argentina	2	205-788	584
KC680722	A	A2	Argentina	2	244-768	525
KC680726	A	A2	Argentina	2	244-768	525
KC680728	A	A2	Argentina	2	244-768	525
KC680730	A	A2	Argentina	2	244-768	525
KC680731	A	A2	Argentina	2	244-768	525
KC680734	A	A2	Argentina	2	244-768	525
KC680736	A	A2	Argentina	2	244-768	525
KC680740	A	A2	Argentina	2	244-768	525
KC680741	A	A2	Argentina	2	244-768	525
KC680744	A	A2	Argentina	2	244-768	525
KC680745	A	A2	Argentina	2	244-768	525
KC680750	A	A2	Argentina	2	244-768	525
KC680751	A	A2	Argentina	2	244-768	525
KC680753	A	A2	Argentina	2	244-768	525
KC680755	A	A2	Argentina	2	244-768	525
KC680757	A	A2	Argentina	2	244-768	525
KC680758	A	A2	Argentina	2	244-768	525
KC680761	A	A2	Argentina	2	244-768	525
KC680763	A	A2	Argentina	2	244-768	525
KC680765	A	A2	Argentina	2	244-768	525
KC680769	A	A2	Argentina	2	244-768	525
KC680771	A	A2	Argentina	2	244-768	525
KJ843154	A	A1	Argentina	2	246-768	523
KJ843162	A	A2	Argentina	2	244-768	525
KU212152	A	A2	Argentina	2	244-768	525
KU212156	A	A2	Argentina	2	244-768	525
JF338909	A	A2	Argentina	3	468-925	458
JF338910	A	A2	Argentina	3	468-925	458
JF338911	A	A2	Argentina	3	468-925	458
JF338912	A	A2	Argentina	3	468-925	458
JF338913	A	A2	Argentina	3	468-925	458
JF338914	A	A2	Argentina	3	468-925	458
JF338915	A	A2	Argentina	3	468-925	458

JF338916	A	A2	Argentina	3	468-925	458
JF338917	A	A2	Argentina	3	468-925	458
JN811651	A	A2	Argentina	4	313-798	486
KY766880	A	A1	Argentina	4	342-780	439
MH763156	A	A2	Argentina	4	364-756	393
MH763431	F	F1b	Argentina	4	364-756	393
MH763474	A	A1	Argentina	4	364-756	393
MH763522	A	A2	Argentina	4	364-756	393
MH763567	A	A2	Argentina	4	364-756	393
JQ272867	A	A2	Argentina	5	78-793	716
JQ272874	A	A2	Argentina	5	78-793	716
JQ272882	A	A2	Argentina	5	78-793	716
JQ272883	A	A2	Argentina	5	78-793	716
MF593312	A	A2	Argentina	5	78-756	679
MF593328	A	A2	Argentina	5	117-793	677
MF593333	A	A2	Argentina	5	117-793	677
MF593336	A	A2	Argentina	5	117-793	677
KJ843165	B	B2	Argentina	6	Complete Genome	3215
KM359440	B	B2	Argentina	6	Complete Genome	3215
JX079923	B	B2	Argentina	7	313-789	477
KF485389	C	C2	Argentina	8	Complete Genome	3215
KF485390	C	C2	Argentina	8	Complete Genome	3215
KM359441	C	C2	Argentina	8	Complete Genome	3215
JX079925	C	C2	Argentina	9	290-785	496
JX079930	C	C2	Argentina	9	290-788	499
JX079931	C	C2	Argentina	9	298-790	493
HQ420153	C	C2	Argentina	10	370-788	419
HQ420154	C	C2	Argentina	10	331-791	461
HQ420155	C	C2	Argentina	10	319-788	470
JX079924	C	C2	Argentina	10	317-787	471
JX079926	C	C2	Argentina	10	345-787	443
JX079927	C	C2	Argentina	10	317-789	473
JX079928	C	C2	Argentina	10	347-789	443
JX079929	C	C2	Argentina	10	344-789	446
JN688722	D	D3	Argentina	11	Complete Genome	3098
KF584158	D	D1	Argentina	11	Complete Genome	3174
KY382413	D	D1	Argentina	11	Complete Genome	3174
JN688685	D	D3	Argentina	11	Complete Genome	3179
JN688715	D	D3	Argentina	11	Complete Genome	3179
KM386676	D	D3	Argentina	11	Complete Genome	3179
JN688678	D	D3	Argentina	11	Complete Genome	3182

JN688679	D	D3	Argentina	11	Complete Genome	3182
JN688683	D	D3	Argentina	11	Complete Genome	3182
JN688689	D	D3	Argentina	11	Complete Genome	3182
JN688695	D	D2	Argentina	11	Complete Genome	3182
JN688708	D	D3	Argentina	11	Complete Genome	3182
JN688710	D	D3	Argentina	11	Complete Genome	3182
JN688711	D	D3	Argentina	11	Complete Genome	3182
JN688712	D	D3	Argentina	11	Complete Genome	3182
JN688716	D	D3	Argentina	11	Complete Genome	3182
JN688717	D	D2	Argentina	11	Complete Genome	3182
KF584159	D	D1	Argentina	11	Complete Genome	3182
KF584160	D	D1	Argentina	11	Complete Genome	3182
KF584161	D	D1	Argentina	11	Complete Genome	3182
KF584162	D	D1	Argentina	11	Complete Genome	3182
KF584163	D	D1	Argentina	11	Complete Genome	3182
KF584164	D	D1	Argentina	11	Complete Genome	3182
KF584165	D	D1	Argentina	11	Complete Genome	3182
KF584166	D	D1	Argentina	11	Complete Genome	3182
KJ647349	D	D3	Argentina	11	Complete Genome	3182
KJ647350	D	D3	Argentina	11	Complete Genome	3182
KJ647351	D	D3	Argentina	11	Complete Genome	3182
KJ647352	D	D2	Argentina	11	Complete Genome	3182
KJ647353	D	D3	Argentina	11	Complete Genome	3182
KJ647355	D	D3	Argentina	11	Complete Genome	3182
KJ647356	D	D2	Argentina	11	Complete Genome	3182
KJ843187	D	D3	Argentina	11	Complete Genome	3182
KM359442	D	D3	Argentina	11	Complete Genome	3182
KY382412	D	D1	Argentina	11	Complete Genome	3182
KY382414	D	D1	Argentina	11	Complete Genome	3182
JN688713	D	D3	Argentina	11	Complete Genome	3185
DQ776270	D	D9	Argentina	12	262-768	507
DQ776271	D	NA	Argentina	12	244-768	525
DQ776272	D	D9	Argentina	12	262-768	507
EU366120	D	D1	Argentina	12	244-768	525
EU366121	D	D3	Argentina	12	244-768	525
EU366122	D	D1	Argentina	12	244-768	525
EU366126	D	D2	Argentina	12	244-768	525
EU366130	D	D3	Argentina	12	244-768	525
EU366131	D	D3	Argentina	12	245-768	524
EU366136	D	D2	Argentina	12	244-768	525
EU366140	D	D3	Argentina	12	244-768	525

EU366141	D	D4	Argentina	12	244-768	525
JN688682	D	D2	Argentina	12	217-771	555
JN688698	D	D3	Argentina	12	217-771	555
JN688706	D	D3	Argentina	12	217-771	555
JN688718	D	D9	Argentina	12	217-771	555
JN688719	D	D9	Argentina	12	217-771	555
KC680727	D	D2	Argentina	12	244-768	525
KU212155	D	D2	Argentina	12	244-768	525
MF593337	A	A2	Argentina	13	117-793	677
MF593314	D	D3	Argentina	13	78-793	707
JQ272868	D	D2	Argentina	13	78-793	716
JQ272873	D	D3	Argentina	13	78-793	716
MF593327	D	D3	Argentina	13	78-793	716
JX079932	D	D3/D6	Argentina	14	328-789	462
JX079935	D	D3	Argentina	14	326-789	464
EU185780	D/A	D3/A2	Argentina	15	Complete Genome	3182
JQ000008	E	E	Argentina	16	Complete Genome	3212
JQ000009	E	E	Argentina	16	Complete Genome	3212
DQ297465	E	E	Argentina	17	283-795	513
DQ297466	E	E	Argentina	17	283-795	513
DQ776247	F	F4	Argentina	18	Complete Genome	3215
DQ823086	F	F4	Argentina	18	Complete Genome	3215
DQ823087	F	F4	Argentina	18	Complete Genome	3215
DQ823088	F	F4	Argentina	18	Complete Genome	3215
DQ823089	F	F4	Argentina	18	Complete Genome	3215
DQ823090	F	F4	Argentina	18	Complete Genome	3215
EF576808	F	F4	Argentina	18	Complete Genome	3215
EF576812	F	F4	Argentina	18	Complete Genome	3215
EU366116	F	F4	Argentina	18	Complete Genome	3215
EU366132	F	F4	Argentina	18	Complete Genome	3215
FJ657519	F	F4	Argentina	18	Complete Genome	3215
FJ657522	F	F4	Argentina	18	Complete Genome	3215
FJ657525	F	F1b	Argentina	18	Complete Genome	3215
FJ657528	F	F4	Argentina	18	Complete Genome	3215
FJ657529	F	F1b	Argentina	18	Complete Genome	3215
JN688691	F	F1b	Argentina	18	Complete Genome	3215
JN688699	F	F1b	Argentina	18	Complete Genome	3215
JN688701	F	F4	Argentina	18	Complete Genome	3215
JN688703	F	F1b	Argentina	18	Complete Genome	3215
JN688709	F	F4	Argentina	18	Complete Genome	3215
JN688720	F	F4	Argentina	18	Complete Genome	3215

JN811655	F	F4	Argentina	18	Complete Genome	3215
JN811656	F	F4	Argentina	18	Complete Genome	3215
JX079936	F	F1b	Argentina	18	Complete Genome	3215
JX079937	F	F4	Argentina	18	Complete Genome	3215
KJ647354	F	F4	Argentina	18	Complete Genome	3215
KJ676692	F	F4	Argentina	18	Complete Genome	3216
KJ676693	F	F4	Argentina	18	Complete Genome	3216
KJ676694	F	F4	Argentina	18	Complete Genome	3215
KJ843163	F	F1b	Argentina	18	Complete Genome	3215
KJ843164	F	F1b	Argentina	18	Complete Genome	3215
KJ843167	F	F1b	Argentina	18	Complete Genome	3215
KJ843168	F	F1b	Argentina	18	Complete Genome	3203
KJ843169	F	F1b	Argentina	18	Complete Genome	3215
KJ843170	F	F1b	Argentina	18	Complete Genome	3215
KJ843171	F	F1b	Argentina	18	Complete Genome	3215
KJ843174	F	F1b	Argentina	18	Complete Genome	3215
KJ843175	F	F4	Argentina	18	Complete Genome	3215
KJ843176	F	F1b	Argentina	18	Complete Genome	3215
KJ843177	F	F1b	Argentina	18	Complete Genome	3215
KJ843178	F	F1b	Argentina	18	Complete Genome	3215
KJ843179	F	F1b	Argentina	18	Complete Genome	3215
KJ843180	F	F1b	Argentina	18	Complete Genome	3215
KJ843181	F	F1b	Argentina	18	Complete Genome	3215
KJ843185	F	F4	Argentina	18	Complete Genome	3215
KJ843189	F	F4	Argentina	18	Complete Genome	3215
KJ843190	F	F1b	Argentina	18	Complete Genome	3215
KJ843191	F	F2a	Argentina	18	Complete Genome	3215
KJ843193	F	F1b	Argentina	18	Complete Genome	3215
KJ843194	F	F1b	Argentina	18	Complete Genome	3215
KJ843195	F	F1b	Argentina	18	Complete Genome	3215
KJ843196	F	F1b	Argentina	18	Complete Genome	3215
KJ843197	F	F1b	Argentina	18	Complete Genome	3215
KJ843198	F	F1b	Argentina	18	Complete Genome	3215
KJ843199	F	F1b	Argentina	18	Complete Genome	3215
KJ843200	F	F1b	Argentina	18	Complete Genome	3215
KJ843201	F	F1b	Argentina	18	Complete Genome	3215
KJ843202	F	F1b	Argentina	18	Complete Genome	3215
KJ843203	F	F1b	Argentina	18	Complete Genome	3215
KJ843204	F	F1b	Argentina	18	Complete Genome	3215
KJ843205	F	F1b	Argentina	18	Complete Genome	3215
KJ843206	F	F1b	Argentina	18	Complete Genome	3215

KJ843207	F	F4	Argentina	18	Complete Genome	3215
KJ843208	F	F4	Argentina	18	Complete Genome	3215
KJ843209	F	F4	Argentina	18	Complete Genome	3215
KJ843210	F	F4	Argentina	18	Complete Genome	3215
KJ843211	F	F4	Argentina	18	Complete Genome	3215
KJ843212	F	F4	Argentina	18	Complete Genome	3215
KJ843213	F	F4	Argentina	18	Complete Genome	3215
KX264499	F	F4	Argentina	18	Complete Genome	3215
KY382411	F	F4	Argentina	18	Complete Genome	3215
KY382415	F	F1b	Argentina	18	Complete Genome	3215
KY382416	F	F1b	Argentina	18	Complete Genome	3215
KY382417	F	F1b	Argentina	18	Complete Genome	3215
MG098564	F	F4	Argentina	18	Complete Genome	3215
MG098565	F	F4	Argentina	18	Complete Genome	3215
MG098566	F	F4	Argentina	18	Complete Genome	3215
MG098567	F	F4	Argentina	18	Complete Genome	3214
MG098568	F	F4	Argentina	18	Complete Genome	3214
MG098569	F	F4	Argentina	18	Complete Genome	3215
MG098571	F	F4	Argentina	18	Complete Genome	3214
MG098572	F	F4	Argentina	18	Complete Genome	3214
MG098575	F	F4	Argentina	18	Complete Genome	3215
MG098576	F	F4	Argentina	18	Complete Genome	3216
MG098577	F	F4	Argentina	18	Complete Genome	3215
MG098578	F	F4	Argentina	18	Complete Genome	3215
MG098579	F	F4	Argentina	18	Complete Genome	3206
MG098580	F	F4	Argentina	18	Complete Genome	3215
MG098581	F	F4	Argentina	18	Complete Genome	3214
MG098582	F	F4	Argentina	18	Complete Genome	3215
MG098584	F	F6	Argentina	18	Complete Genome	3215
JQ272860	F	F1b	Argentina	19	78-793	716
JQ272861	F	F1b	Argentina	19	78-793	716
JQ272862	F	F1b	Argentina	19	78-793	716
JQ272863	F	F1b	Argentina	19	78-793	716
JQ272864	F	F1b	Argentina	19	78-793	716
JQ272865	F	F1b	Argentina	19	78-793	716
JQ272866	F	F1b	Argentina	19	78-793	716
JQ272869	F	F1b	Argentina	19	78-793	716
JQ272870	F	F1b	Argentina	19	78-793	716
JQ272871	F	F4	Argentina	19	78-793	716
JQ272872	F	F1b	Argentina	19	78-793	716
JQ272875	F	F1b	Argentina	19	78-793	716

JQ272876	F	F1b	Argentina	19	78-793	716
JQ272877	F	F4	Argentina	19	78-793	716
JQ272878	F	F1b	Argentina	19	78-793	716
JQ272879	F	F1b	Argentina	19	78-793	716
JQ272880	F	F1b	Argentina	19	78-793	716
JQ272881	F	F1b	Argentina	19	78-793	716
JQ272884	F	F1b	Argentina	19	78-793	716
JQ272885	F	F1b	Argentina	19	78-793	716
MF593308	F	F1b	Argentina	19	78-756	679
MF593309	F	F1b	Argentina	19	78-756	679
MF593310	F	F1b	Argentina	19	78-756	679
MF593311	F	F1b	Argentina	19	78-756	679
MF593313	F	F1b	Argentina	19	117-793	677
MF593315	F	F1b	Argentina	19	78-756	679
MF593316	F	F1b	Argentina	19	78-756	679
MF593317	F	F1b	Argentina	19	78-756	679
MF593318	F	F1b	Argentina	19	78-756	679
MF593319	F	F1b	Argentina	19	78-793	707
MF593320	F	F1b	Argentina	19	117-793	677
MF593321	F	F1b	Argentina	19	78-756	679
MF593322	F	F1b	Argentina	19	78-756	679
MF593323	F	F1b	Argentina	19	78-756	679
MF593324	F	F1b	Argentina	19	78-756	679
MF593325	F	F1b	Argentina	19	78-756	679
MF593326	F	F1b	Argentina	19	78-756	679
MF593329	F	F1b	Argentina	19	117-793	677
MF593330	F	F1b	Argentina	19	78-756	679
MF593331	F	F1b	Argentina	19	78-756	679
MF593332	F	F1b	Argentina	19	78-756	679
MF593334	F	F1b	Argentina	19	78-756	679
MF593335	F	F1b	Argentina	19	78-756	679
MF593338	F	F1b	Argentina	19	104-793	690
MF593339	F	F1b	Argentina	19	104-793	690
MF593340	F	F1b	Argentina	19	104-793	690
MF593341	F	F1b	Argentina	19	104-793	690
MF593342	F	F1b	Argentina	19	104-793	690
MF593343	F	F1b	Argentina	19	104-793	690
MF593344	F	F1b	Argentina	19	78-777	700
MF593345	F	F1b	Argentina	19	78-777	700
MF593346	F	F1b	Argentina	19	78-777	700
MF593347	F	F1b	Argentina	19	78-777	700

MF593348	F	F1b	Argentina	19	78-777	700
MF593349	F	F1b	Argentina	19	78-777	700
MF593350	F	F1b	Argentina	19	78-777	700
MF593351	F	F1b	Argentina	19	78-777	700
MF593352	F	F1b	Argentina	19	78-777	700
KJ810935	F	F1b	Argentina	20	314-1122	809
KJ810939	F	F1b	Argentina	20	314-1164	851
KJ810940	F	F1b	Argentina	20	256-1116	961
KJ810942	F	F1b	Argentina	20	314-1117	904
KJ810944	F	F1b	Argentina	20	314-1117	904
KJ810946	F	F1b	Argentina	20	256-1118	963
KJ810947	F	F1b	Argentina	20	256-1117	963
KJ810949	F	F1b	Argentina	20	310-1117	908
KJ810951	F	F4	Argentina	20	257-1117	961
KJ810953	F	F4	Argentina	20	277-1116	941
KJ810955	F	F4	Argentina	20	267-1116	951
KJ810957	F	F4	Argentina	20	267-1116	950
KJ810919	F	F1b	Argentina	21	267-1005	739
KJ810938	F	F1b	Argentina	21	256-1006	751
KJ810948	F	F1b	Argentina	21	312-1020	709
KJ810954	F	F4	Argentina	21	312-1007	696
KJ810971	F	F1b	Argentina	21	257-1004	748
KJ810974	F	F4	Argentina	21	267-1007	742
KJ810975	F	F4	Argentina	21	267-1007	742
KJ810945	F	F1b	Argentina	22	575-1006	432
HQ420156	F	F4	Argentina	23	323-790	468
HQ420157	F	F4	Argentina	23	370-790	421
HQ420158	F	F4	Argentina	23	319-790	472
HQ420159	F	F4	Argentina	23	319-791	473
HQ420160	F	F4	Argentina	23	324-771	448
HQ420161	F	F1b	Argentina	23	323-786	464
HQ420162	F	F1b	Argentina	23	319-795	477
JN811652	F	F4	Argentina	23	292-785	494
JN811653	F	F4	Argentina	23	393-785	393
JX079933	F	F1b	Argentina	23	350-788	439
JX079934	F	F1b	Argentina	23	399-786	388
KY766881	F	F1b	Argentina	23	333-779	447
MH763275	F	F1b	Argentina	23	364-784	421
MH763359	F	F4	Argentina	23	364-756	393
MH763477	F	F1b	Argentina	23	364-784	421
DQ776248	F	F4	Argentina	24	244-768	525

EU366115	F	F6	Argentina	24	244-768	525
EU366124	F	F4	Argentina	24	244-768	525
EU366125	F	F4	Argentina	24	244-768	525
FJ657518	F	F4	Argentina	24	244-768	525
FJ657520	F	F4	Argentina	24	244-768	525
FJ657521	F	F1b	Argentina	24	244-768	525
FJ657523	F	F1b	Argentina	24	244-768	525
FJ657524	F	F1b	Argentina	24	244-768	525
GU207481	F	F1b	Argentina	24	244-750	507
GU207482	F	F1b	Argentina	24	244-750	507
GU207483	F	F1b	Argentina	24	244-768	525
GU207484	F	F4	Argentina	24	244-768	525
GU207485	F	F4	Argentina	24	244-750	507
GU207486	F	F1b	Argentina	24	249-768	520
GU207488	F	F4	Argentina	24	244-750	507
GU207489	F	F1b	Argentina	24	244-768	525
GU207490	F	F1b	Argentina	24	244-768	525
GU207491	F	F1b	Argentina	24	244-768	525
GU207492	F	F1b	Argentina	24	244-768	525
GU207493	F	F1b	Argentina	24	244-768	525
JN688684	F	F4	Argentina	24	217-771	555
JN688702	F	F1b	Argentina	24	217-771	555
JN688704	F	F1b	Argentina	24	217-771	555
JN688721	F	F4	Argentina	24	217-771	555
KC680723	F	F1b	Argentina	24	244-768	525
KC680724	F	F1b	Argentina	24	244-768	525
KC680725	F	F4	Argentina	24	244-768	525
KC680729	F	F1b	Argentina	24	244-768	525
KC680732	F	F1b	Argentina	24	244-768	525
KC680735	F	F1b	Argentina	24	244-768	525
KC680737	F	F6	Argentina	24	244-768	525
KC680738	F	F1b	Argentina	24	244-768	525
KC680739	F	F1b	Argentina	24	244-768	525
KC680742	F	F1b	Argentina	24	244-768	525
KC680743	F	F1b	Argentina	24	244-768	525
KC680746	F	F6	Argentina	24	244-768	525
KC680747	F	F1b	Argentina	24	244-768	525
KC680748	F	F1b	Argentina	24	244-768	525
KC680749	F	F4	Argentina	24	244-768	525
KC680754	F	F1b	Argentina	24	244-768	525
KC680756	F	F1b	Argentina	24	244-768	525

KC680760	F	F1b	Argentina	24	244-768	525
KC680762	F	F6	Argentina	24	244-768	525
KC680764	F	F4	Argentina	24	244-768	525
KC680766	F	F1b	Argentina	24	244-768	525
KC680767	F	F4	Argentina	24	244-768	525
KC680768	F	F1b	Argentina	24	244-768	525
KC680770	F	F1b	Argentina	24	244-768	525
KJ810921	F	F4	Argentina	24	249-815	567
KJ810934	F	F1b	Argentina	24	252-794	543
KJ810936	F	F1b	Argentina	24	256-772	517
KJ810937	F	F1b	Argentina	24	256-774	519
KJ810941	F	F1b	Argentina	24	252-774	522
KJ810943	F	F1b	Argentina	24	252-774	522
KJ810952	F	F4	Argentina	24	256-794	539
KJ810956	F	F4	Argentina	24	245-751	507
KJ810958	F	F4	Argentina	24	256-773	518
KJ810970	F	F1b	Argentina	24	256-774	519
KJ810972	F	F4	Argentina	24	252-771	520
KJ810973	F	F4	Argentina	24	247-775	529
KJ843155	F	F1b	Argentina	24	246-768	523
KJ843156	F	F1b	Argentina	24	244-768	525
KJ843157	F	F1b	Argentina	24	249-768	520
KJ843158	F	F1b	Argentina	24	247-768	522
KJ843159	F	F1b	Argentina	24	246-768	523
KJ843160	F	F1b	Argentina	24	245-768	524
KJ843161	F	F1b	Argentina	24	246-768	523
KU212148	F	F1b	Argentina	24	244-768	525
KU212149	F	F6	Argentina	24	244-768	525
KU212150	F	F6	Argentina	24	244-768	525
KU212151	F	F4	Argentina	24	244-768	525
KU212153	F	F4	Argentina	24	244-768	525
KU212154	F	F1b	Argentina	24	244-768	525
KU212157	F	F1b	Argentina	24	244-768	525
KU212158	F	F1b	Argentina	24	244-768	525
MK558392	F	F1b	Argentina	24	256-877	622
MK558393	F	F1b	Argentina	24	256-877	622
MK558394	F	F1b	Argentina	24	256-877	622
MK558395	F	F1b	Argentina	24	256-877	622
MK558396	F	F1b	Argentina	24	256-877	622
MK558397	F	F1b	Argentina	24	256-877	622
MK558398	F	F1b	Argentina	24	256-877	622

MK558399	F	F1b	Argentina	24	256-877	622
MK558400	F	F1b	Argentina	24	256-877	622
MK558401	F	F1b	Argentina	24	256-877	622
MK558402	F	F1b	Argentina	24	256-877	622
MK558403	F	F1b	Argentina	24	256-877	622
MK558404	F	F1b	Argentina	24	256-877	622
MK558405	F	F1b	Argentina	24	256-877	622
MK558406	F	F1b	Argentina	24	256-877	622
MK558407	F	F1b	Argentina	24	256-877	622
MK558408	F	F1b	Argentina	24	256-877	622
MK558409	F	F1b	Argentina	24	256-877	622
MK558410	F	F1b	Argentina	24	256-877	622
MK558411	F	F1b	Argentina	24	256-877	622
MK558412	F	F1b	Argentina	24	256-877	622
MK558413	F	F1b	Argentina	24	256-877	622
MK558414	F	F1b	Argentina	24	256-877	622
MK558415	F	F1b	Argentina	24	256-877	622
MK558416	F	F1b	Argentina	24	256-877	622
MK558417	F	F1b	Argentina	24	256-877	622
FJ356715	H	H	Argentina	25	Complete Genome	3215
FJ356716	H	H	Argentina	25	Complete Genome	3215

[1]Alignment to complete genome reference sequence VHB NC_003977.2
