# Supplementary material for: The Molecular Epidemiology of Hepatitis B Virus and Its Resistance-Associated Mutations in the Polymerase Gene in the Americas
Source: Microorganisms. 2025 Aug 16;13(8):1913. doi: 10.3390/microorganisms13081913 (PMC12388563; doi:10.3390/microorganisms13081913)
Supplement: Supplementary file 1 [file microorganisms-13-01913-s001.zip › Figure S2 - Report Subgenotyping All.pdf]

Phylogenetic Analysis of Sequences

GENOTYPE AND SUBTYPE NOT PUBLICLY REPORTED

A – SUBGENOTYPE

ARGENTINA

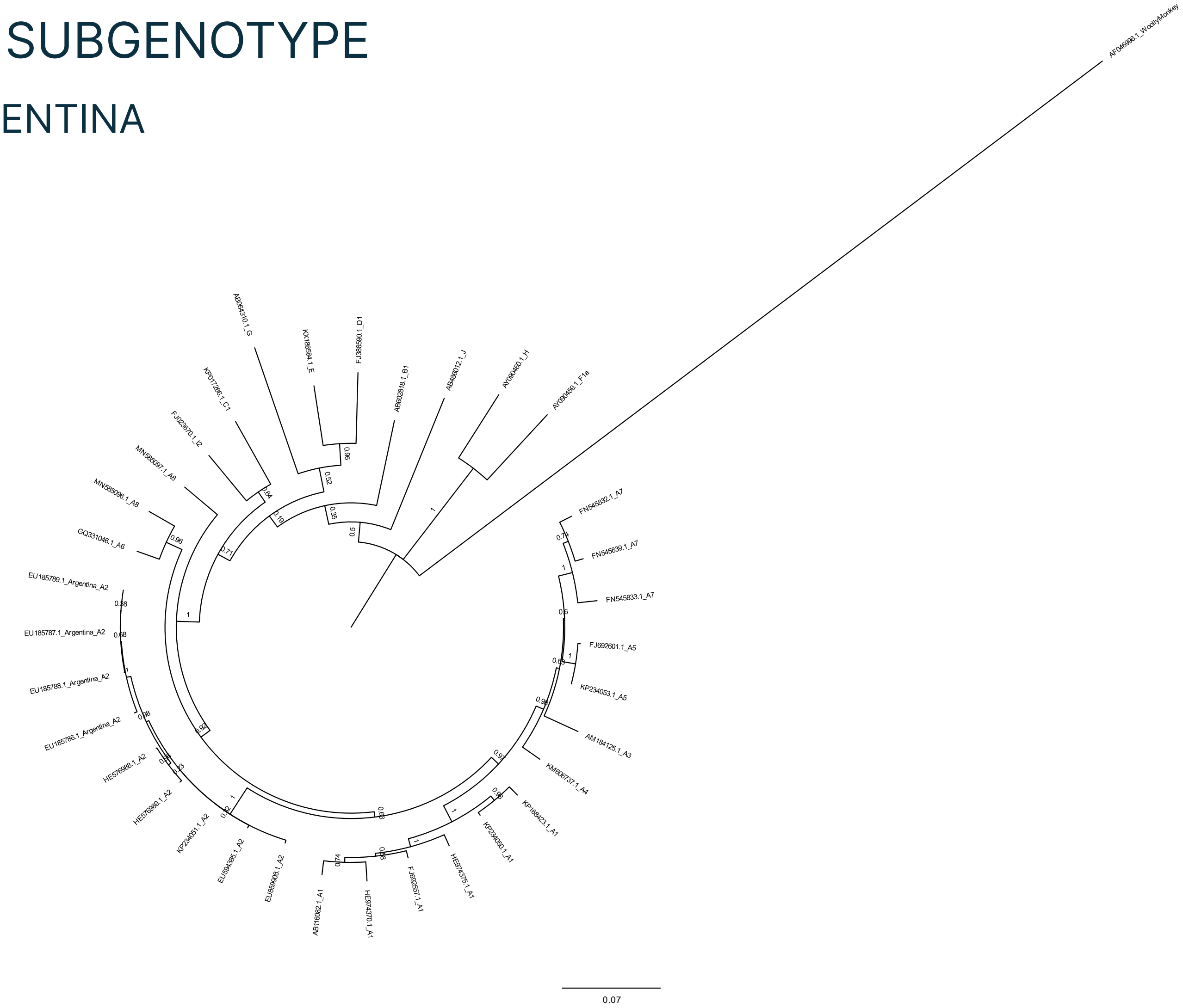

Tree 1. The evolutionary history was inferred by using the Maximum Likelihood method and Tamura-Nei model. The percentage of replicate trees in which the associated taxa clustered together in the bootstrap test (1000 replicates) are shown next to the branches. Initial tree(s) for the heuristic search were obtained automatically by applying Neighbor-Join and BioNJ algorithms to a matrix of pairwise distances estimated using the Tamura-Nei model, and then selecting the topology with superior log likelihood value. A discrete Gamma distribution was used to model evolutionary rate differences among sites (5 categories (+G, parameter = 0.2176)). The tree is drawn to scale, with branch lengths measured in the number of substitutions per site. The analysis involved 35 nucleotide sequences, of which 31 were used as marker sequences to determine the genotype of 4 sequences. All positions containing gaps and missing data were eliminated. There was a total of 2592 positions in the final dataset. Evolutionary analyses were conducted in MEGA X.

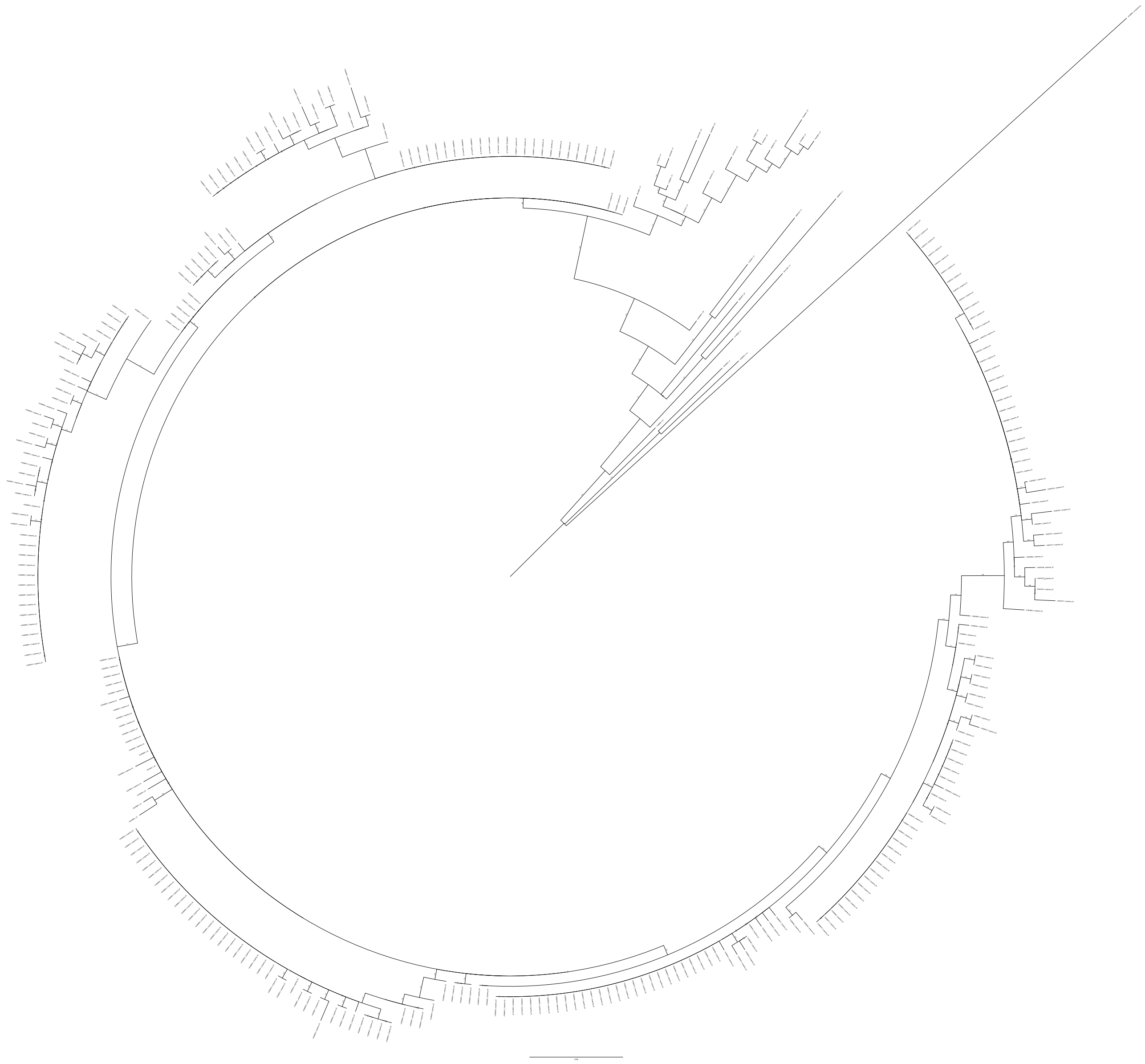

Tree 2. The evolutionary history was inferred by using the Maximum Likelihood method and Tamura-Nei model. The percentage of replicate trees in which the associated taxa clustered together in the bootstrap test (1000 replicates) are shown next to the branches. Initial tree(s) for the heuristic search were obtained automatically by applying Neighbor-Join and BioNJ algorithms to a matrix of pairwise distances estimated using the Tamura-Nei model, and then selecting the topology with superior log likelihood value. A discrete Gamma distribution was used to model evolutionary rate differences among sites (5 categories (+G, parameter = 0.1619)). The tree is drawn to scale, with branch lengths measured in the number of substitutions per site. The analysis involved 306 nucleotide sequences, of which 31 were used as marker sequences to determine the genotype of 275 sequences. All positions containing gaps and missing data were eliminated. There was a total of 557 positions in the final dataset. Evolutionary analyses were conducted in MEGA X.

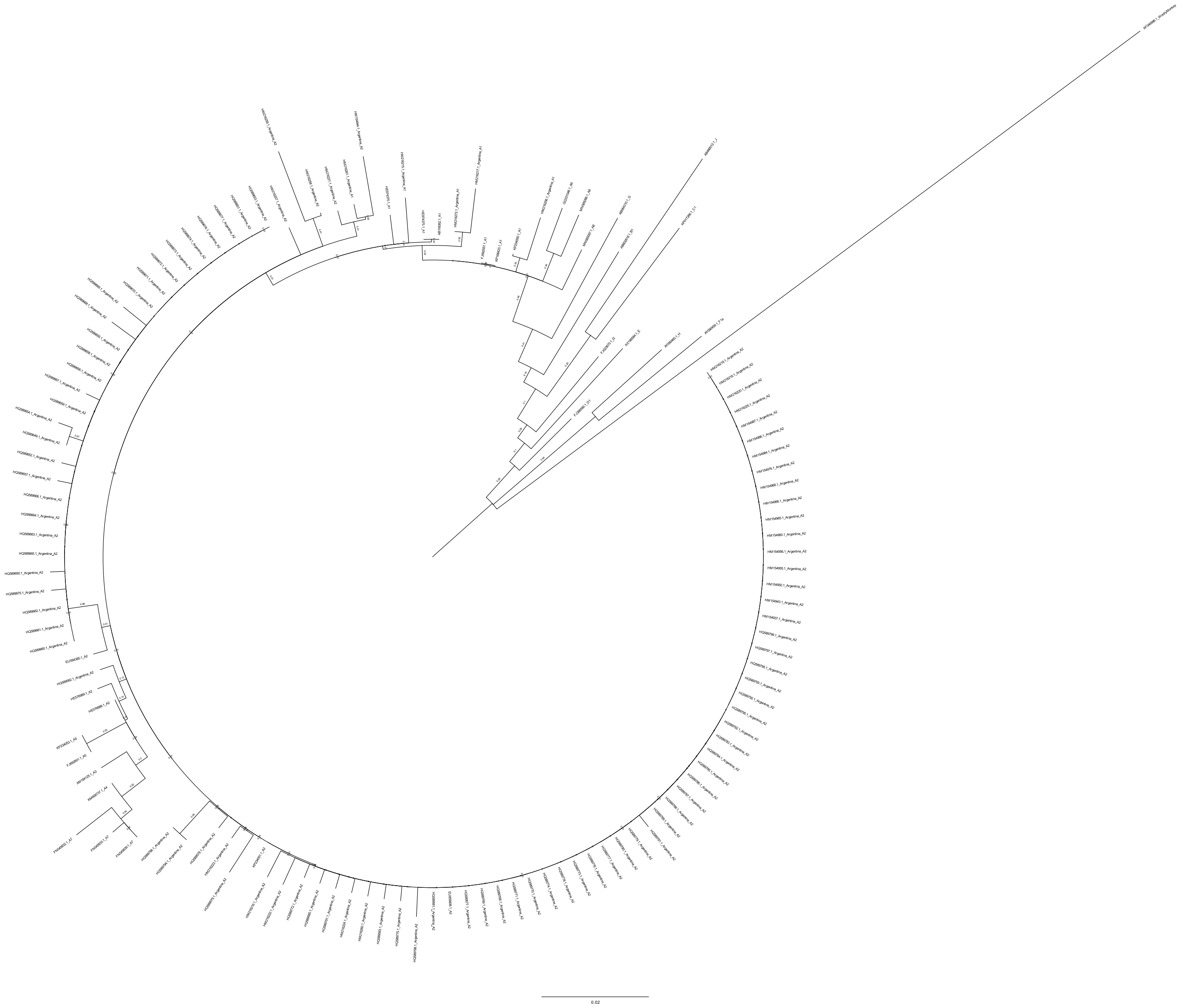

Tree 3. The evolutionary history was inferred by using the Maximum Likelihood method and Tamura-Nei model. The percentage of replicate trees in which the associated taxa clustered together in the bootstrap test (1000 replicates) are shown next to the branches. Initial tree(s) for the heuristic search were obtained automatically by applying Neighbor-Join and BioNJ algorithms to a matrix of pairwise distances estimated using the Tamura-Nei model, and then selecting the topology with superior log likelihood value. A discrete Gamma distribution was used to model evolutionary rate differences among sites (5 categories (+G, parameter = 0.2490)). The tree is drawn to scale, with branch lengths measured in the number of substitutions per site. The analysis involved 131 nucleotide sequences, of which 31 were used as marker sequences to determine the genotype of 100 sequences. All positions containing gaps and missing data were eliminated. There was a total of 433 positions in the final dataset. Evolutionary analyses were conducted in MEGA X.

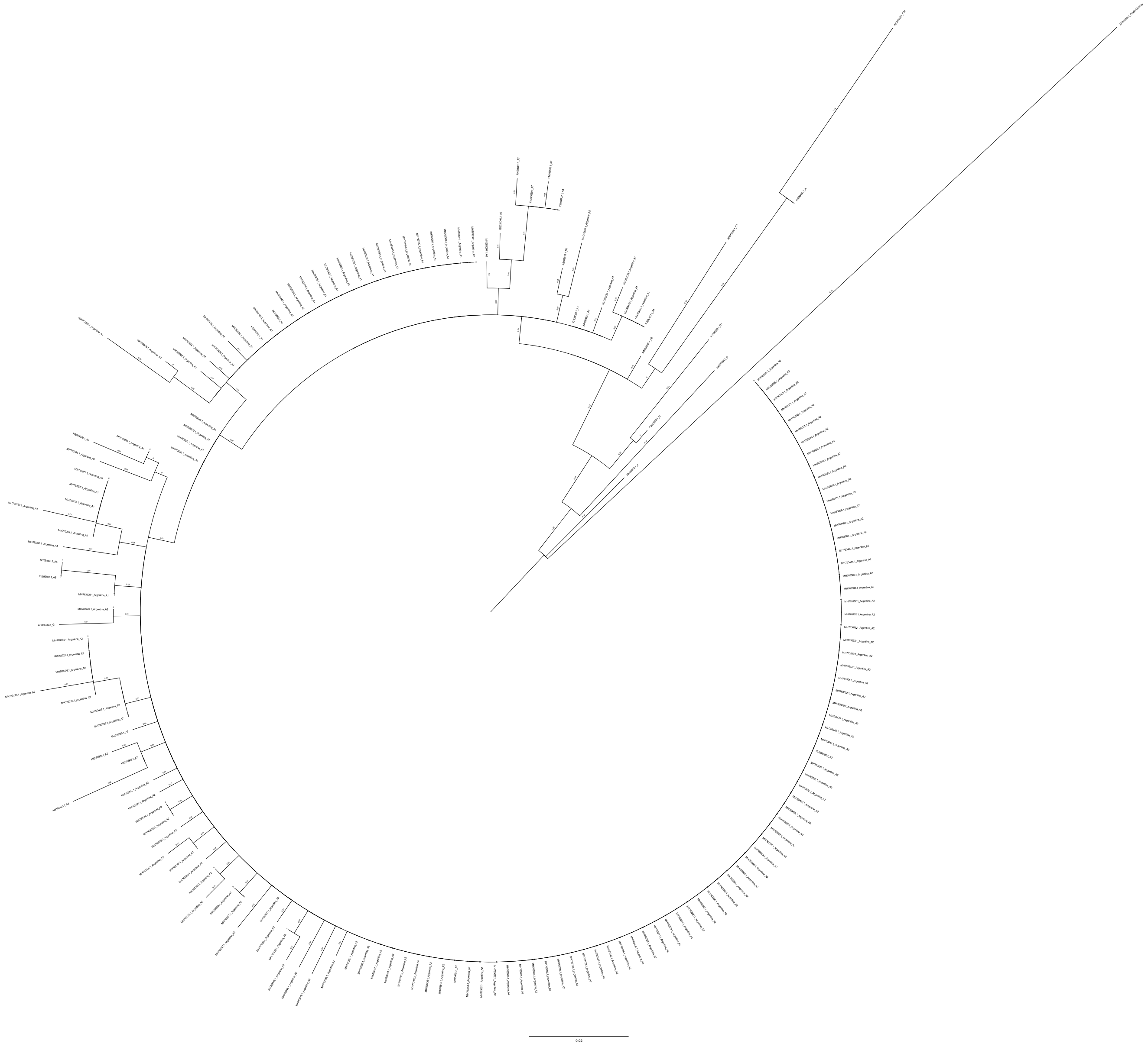

Tree 4. The evolutionary history was inferred by using the Maximum Likelihood method and Tamura-Nei model. The percentage of replicate trees in which the associated taxa clustered together in the bootstrap test (1000 replicates) are shown next to the branches. Initial tree(s) for the heuristic search were obtained automatically by applying Neighbor-Join and BioNJ algorithms to a matrix of pairwise distances estimated using the Tamura-Nei model, and then selecting the topology with superior log likelihood value. A discrete Gamma distribution was used to model evolutionary rate differences among sites (5 categories (+G, parameter = 0.1967)). The tree is drawn to scale, with branch lengths measured in the number of substitutions per site. The analysis involved 174 nucleotide sequences, of which 31 were used as marker sequences to determine the genotype of 143 sequences. All positions containing gaps and missing data were eliminated. There was a total of 222 positions in the final dataset. Evolutionary analyses were conducted in MEGA X.

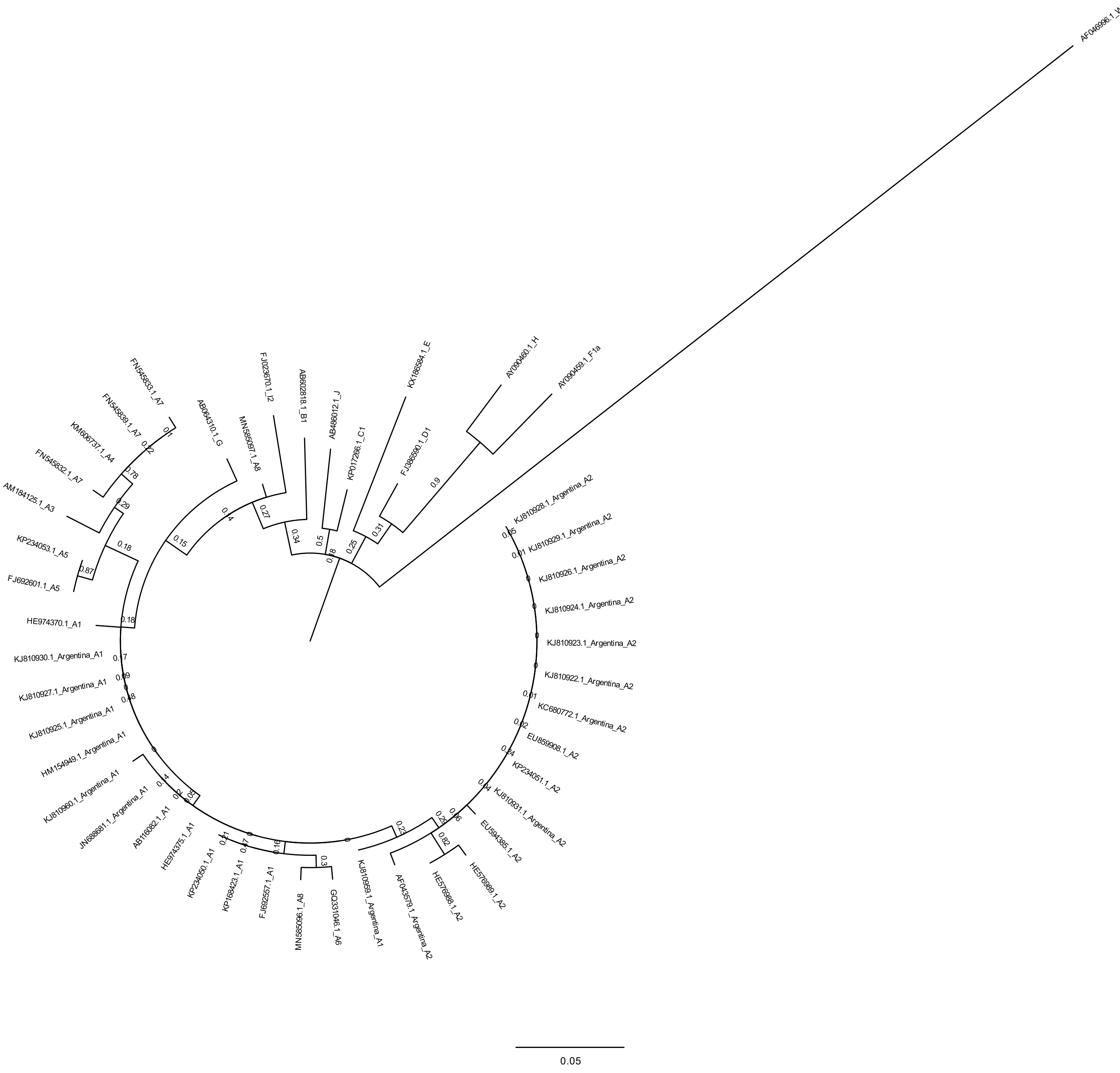

Tree 5. The evolutionary history was inferred by using the Maximum Likelihood method and Tamura-Nei model. The percentage of replicate trees in which the associated taxa clustered together in the bootstrap test (1000 replicates) are shown next to the branches. Initial tree(s) for the heuristic search were obtained automatically by applying Neighbor-Join and BioNJ algorithms to a matrix of pairwise distances estimated using the Tamura-Nei model, and then selecting the topology with superior log likelihood value. A discrete Gamma distribution was used to model evolutionary rate differences among sites (5 categories (+G, parameter = 0.1420)). The tree is drawn to scale, with branch lengths measured in the number of substitutions per site. The analysis involved 47 nucleotide sequences, of which 31 were used as marker sequences to determine the genotype of 16 sequences. All positions containing gaps and missing data were eliminated. There was a total of 230 positions in the final dataset. Evolutionary analyses were conducted in MEGA X.

ID	GENOTYPE	SUBTYPE	COUNTRY	TREE	ALIGNMENT[1]	BASE PAIRS
EU185786	A	A2	Argentina	1	Complete Genome	3221
EU185787	A	A2	Argentina	1	Complete Genome	3221
EU185788	A	A2	Argentina	1	Complete Genome	2802
EU185789	A	A2	Argentina	1	Complete Genome	3221
DQ249924	A	A2	Argentina	2	205-789	585
DQ249925	A	A2	Argentina	2	205-789	585
DQ249926	A	A2	Argentina	2	205-789	585
DQ249927	A	A2	Argentina	2	205-789	585
DQ249928	A	A2	Argentina	2	205-789	585
DQ249929	A	A2	Argentina	2	205-789	585
DQ249930	A	A2	Argentina	2	205-789	585
DQ249931	A	A2	Argentina	2	205-789	585
DQ249932	A	A2	Argentina	2	205-789	585
DQ249933	A	A2	Argentina	2	205-789	585
DQ249934	A	A2	Argentina	2	205-789	585
DQ249935	A	A2	Argentina	2	205-789	585
DQ249936	A	A2	Argentina	2	205-789	585
DQ249938	A	A2	Argentina	2	205-789	585
DQ249940	A	A2	Argentina	2	205-789	585
DQ249941	A	A2	Argentina	2	205-789	585
HQ589358	A	A2	Argentina	2	183-788	606
HQ589359	A	A2	Argentina	2	183-788	606
HQ589360	A	A2	Argentina	2	183-788	606
HQ589361	A	A2	Argentina	2	183-788	606
HQ589362	A	A2	Argentina	2	183-788	606
HQ589363	A	A2	Argentina	2	183-788	606
HQ589364	A	A2	Argentina	2	183-788	606
HQ589365	A	A2	Argentina	2	183-788	606
HQ589366	A	A2	Argentina	2	183-788	606
HQ589367	A	A2	Argentina	2	183-788	606
HQ589368	A	A2	Argentina	2	183-788	606
HQ589369	A	A2	Argentina	2	183-788	606
HQ589370	A	A2	Argentina	2	183-788	606
HQ589371	A	A2	Argentina	2	183-788	606
HQ589372	A	A2	Argentina	2	183-788	606
HQ589373	A	A2	Argentina	2	183-788	606
HQ589374	A	A2	Argentina	2	183-788	606
HQ589375	A	A2	Argentina	2	183-788	606
HQ589376	A	A2	Argentina	2	183-788	606
HQ589377	A	A2	Argentina	2	183-788	606
HQ589378	A	A2	Argentina	2	183-788	606
HQ589379	A	A2	Argentina	2	183-788	606
HQ589380	A	A2	Argentina	2	183-788	606
HQ589381	A	A2	Argentina	2	183-788	606

HQ589382	A	A2	Argentina	2	183-788	606
HQ589383	A	A2	Argentina	2	183-788	606
HQ589384	A	A2	Argentina	2	183-788	606
HQ589385	A	A2	Argentina	2	183-788	606
HQ589386	A	A2	Argentina	2	183-788	606
HQ589387	A	A2	Argentina	2	183-788	606
HQ589388	A	A2	Argentina	2	183-788	606
HQ589389	A	A2	Argentina	2	183-788	606
HQ589390	A	A2	Argentina	2	183-788	606
HQ589391	A	A2	Argentina	2	183-788	606
HQ589392	A	A2	Argentina	2	183-788	606
HQ589393	A	A2	Argentina	2	183-788	606
HQ589394	A	A2	Argentina	2	183-788	606
HQ589395	A	A2	Argentina	2	183-788	606
HQ589396	A	A2	Argentina	2	183-788	606
HQ589397	A	A2	Argentina	2	183-788	606
HQ589398	A	A2	Argentina	2	183-788	606
HQ589399	A	A2	Argentina	2	183-788	606
HQ589400	A	A2	Argentina	2	183-788	606
HQ589401	A	A2	Argentina	2	183-788	606
HQ589402	A	A2	Argentina	2	183-788	606
HQ589403	A	A2	Argentina	2	183-788	606
HQ589404	A	A2	Argentina	2	183-788	606
HQ589405	A	A2	Argentina	2	183-788	606
HQ589406	A	A2	Argentina	2	183-788	606
HQ589407	A	A2	Argentina	2	183-788	606
HQ589408	A	A2	Argentina	2	183-788	606
HQ589409	A	A2	Argentina	2	183-788	606
HQ589410	A	A2	Argentina	2	183-788	606
HQ589411	A	A2	Argentina	2	183-788	606
HQ589412	A	A2	Argentina	2	183-788	606
HQ589413	A	A2	Argentina	2	183-788	606
HQ589414	A	A2	Argentina	2	183-788	606
HQ589415	A	A2	Argentina	2	183-788	606
HQ589416	A	A2	Argentina	2	183-788	606
HQ589417	A	A2	Argentina	2	183-788	606
HQ589418	A	A2	Argentina	2	183-788	606
HQ589419	A	A2	Argentina	2	183-788	606
HQ589420	A	A2	Argentina	2	183-788	606
HQ589421	A	A2	Argentina	2	183-788	606
HQ589422	A	A2	Argentina	2	183-788	606
HQ589423	A	A2	Argentina	2	183-788	606
HQ589424	A	A2	Argentina	2	183-788	606
HQ589425	A	A2	Argentina	2	183-788	606
HQ589426	A	A2	Argentina	2	183-788	606
HQ589427	A	A2	Argentina	2	183-788	606

HQ589428	A	A2	Argentina	2	183-788	606
HQ589429	A	A2	Argentina	2	183-788	606
HQ589430	A	A2	Argentina	2	183-788	606
HQ589431	A	A2	Argentina	2	183-788	606
HQ589432	A	A2	Argentina	2	183-788	606
HQ589433	A	A2	Argentina	2	183-788	606
HQ589434	A	A2	Argentina	2	183-788	606
HQ589435	A	A2	Argentina	2	183-788	606
HQ589436	A	A2	Argentina	2	183-788	606
HQ589437	A	A2	Argentina	2	183-788	606
HQ589438	A	A2	Argentina	2	183-788	606
HQ589439	A	A2	Argentina	2	183-788	606
HQ589440	A	A2	Argentina	2	183-788	606
HQ589441	A	A2	Argentina	2	183-788	606
HQ589442	A	A2	Argentina	2	183-788	606
HQ589443	A	A2	Argentina	2	183-788	606
HQ589444	A	A2	Argentina	2	183-788	606
HQ589445	A	A2	Argentina	2	183-788	606
HQ589446	A	A2	Argentina	2	183-788	606
HQ589447	A	A2	Argentina	2	183-788	606
HQ589448	A	A2	Argentina	2	183-788	606
HQ589449	A	A2	Argentina	2	183-788	606
HQ589450	A	A2	Argentina	2	183-788	606
HQ589451	A	A2	Argentina	2	183-788	606
HQ589452	A	A2	Argentina	2	183-788	606
HQ589453	A	A2	Argentina	2	183-788	606
HQ589454	A	A2	Argentina	2	183-788	606
HQ589455	A	A2	Argentina	2	183-788	606
HQ589456	A	A2	Argentina	2	183-788	606
HQ589457	A	A2	Argentina	2	183-788	606
HQ589458	A	A2	Argentina	2	183-788	606
HQ589459	A	A2	Argentina	2	183-788	606
HQ589460	A	A2	Argentina	2	183-788	606
HQ589461	A	A2	Argentina	2	183-788	606
HQ589462	A	A2	Argentina	2	183-788	606
HQ589463	A	A2	Argentina	2	183-788	606
HQ589464	A	A2	Argentina	2	183-788	606
HQ589465	A	A2	Argentina	2	183-788	606
HQ589466	A	A2	Argentina	2	183-788	606
HQ589467	A	A2	Argentina	2	183-788	606
HQ589468	A	A2	Argentina	2	183-788	606
HQ589469	A	A2	Argentina	2	183-788	606
HQ589470	A	A2	Argentina	2	183-788	606
HQ589471	A	A2	Argentina	2	183-788	606
HQ589472	A	A2	Argentina	2	183-788	606
HQ589473	A	A2	Argentina	2	183-788	606

HQ589474	A	A2	Argentina	2	183-788	606
HQ589475	A	A2	Argentina	2	183-788	606
HQ589476	A	A2	Argentina	2	183-788	606
HQ589477	A	A2	Argentina	2	183-788	606
HQ589478	A	A2	Argentina	2	183-788	606
HQ589479	A	A2	Argentina	2	183-788	606
HQ589480	A	A2	Argentina	2	183-788	606
HQ589481	A	A2	Argentina	2	183-788	606
HQ589482	A	A2	Argentina	2	183-788	606
HQ589483	A	A2	Argentina	2	183-788	606
HQ589484	A	A2	Argentina	2	183-788	606
HQ589485	A	A2	Argentina	2	183-788	606
HQ589486	A	A2	Argentina	2	183-788	606
HQ589487	A	A2	Argentina	2	183-788	606
HQ589488	A	A2	Argentina	2	183-788	606
HQ589489	A	A2	Argentina	2	183-788	606
HQ589490	A	A2	Argentina	2	183-788	606
HQ589491	A	A2	Argentina	2	183-788	606
HQ589492	A	A2	Argentina	2	183-788	606
HQ589493	A	A2	Argentina	2	183-788	606
HQ589494	A	A2	Argentina	2	183-788	606
HQ589495	A	A2	Argentina	2	183-788	606
HQ589496	A	A2	Argentina	2	183-788	606
HQ589497	A	A2	Argentina	2	183-788	606
HQ589498	A	A2	Argentina	2	183-788	606
HQ589499	A	A2	Argentina	2	183-788	606
HQ589500	A	A2	Argentina	2	183-788	606
HQ589501	A	A2	Argentina	2	183-788	606
HQ589502	A	A2	Argentina	2	183-788	606
HQ589503	A	A2	Argentina	2	183-788	606
HQ589504	A	A2	Argentina	2	183-788	606
HQ589505	A	A2	Argentina	2	183-788	606
HQ589506	A	A2	Argentina	2	183-788	606
HQ589507	A	A2	Argentina	2	183-788	606
HQ589508	A	A2	Argentina	2	183-788	606
HQ589509	A	A2	Argentina	2	183-788	606
HQ589510	A	A2	Argentina	2	183-788	606
HQ589511	A	A2	Argentina	2	183-788	606
HQ589512	A	A2	Argentina	2	183-788	606
HQ589513	A	A2	Argentina	2	183-788	606
HQ589514	A	A2	Argentina	2	183-788	606
HQ589515	A	A2	Argentina	2	183-788	606
HQ589516	A	A2	Argentina	2	183-788	606
HQ589517	A	A2	Argentina	2	189-788	600
HQ589518	A	A2	Argentina	2	189-788	600
HQ589519	A	A2	Argentina	2	189-788	600

HQ589520	A	A2	Argentina	2	189-788	600
HQ589521	A	A2	Argentina	2	189-788	600
HQ589522	A	A2	Argentina	2	189-788	600
HQ589523	A	A2	Argentina	2	189-788	600
HQ589524	A	A2	Argentina	2	189-788	600
HQ589525	A	A2	Argentina	2	189-788	600
HQ589526	A	A2	Argentina	2	189-788	600
HQ589527	A	A2	Argentina	2	189-788	600
HQ589528	A	A2	Argentina	2	189-788	600
HQ589529	A	A2	Argentina	2	189-788	600
HQ589530	A	A2	Argentina	2	189-788	600
HQ589531	A	A2	Argentina	2	189-788	600
HQ589532	A	A2	Argentina	2	189-788	600
HQ589533	A	A2	Argentina	2	189-788	600
HQ589534	A	A2	Argentina	2	189-788	600
HQ589535	A	A2	Argentina	2	189-788	600
HQ589536	A	A2	Argentina	2	189-788	600
HQ589537	A	A2	Argentina	2	183-788	606
HQ589538	A	A2	Argentina	2	183-788	606
HQ589539	A	A2	Argentina	2	183-788	606
HQ589540	A	A2	Argentina	2	183-788	606
HQ589541	A	A2	Argentina	2	183-788	606
HQ589542	A	A2	Argentina	2	183-788	606
HQ589543	A	A2	Argentina	2	183-788	606
HQ589544	A	A2	Argentina	2	183-788	606
HQ589545	A	A2	Argentina	2	183-788	606
HQ589546	A	A2	Argentina	2	183-788	606
HQ589547	A	A2	Argentina	2	183-788	606
HQ589548	A	A2	Argentina	2	183-788	606
HQ589549	A	A2	Argentina	2	183-788	606
HQ589550	A	A2	Argentina	2	183-788	606
HQ589551	A	A2	Argentina	2	183-788	606
HQ589552	A	A2	Argentina	2	183-788	606
HQ589553	A	A2	Argentina	2	183-788	606
HQ589554	A	A2	Argentina	2	183-788	606
HQ589555	A	A2	Argentina	2	183-788	606
HQ589556	A	A2	Argentina	2	183-788	606
HQ589557	A	A2	Argentina	2	183-788	606
HQ589558	A	A2	Argentina	2	183-788	606
HQ589559	A	A2	Argentina	2	183-788	606
HQ589560	A	A2	Argentina	2	183-788	606
HQ589561	A	A2	Argentina	2	183-788	606
HQ589562	A	A2	Argentina	2	183-788	606
HQ589563	A	A2	Argentina	2	183-788	606
HQ589564	A	A2	Argentina	2	183-788	606
HQ589565	A	A2	Argentina	2	183-788	606

HQ589566	A	A2	Argentina	2	183-788	606
HQ589567	A	A2	Argentina	2	183-788	606
HQ589568	A	A2	Argentina	2	183-788	606
HQ589569	A	A2	Argentina	2	183-788	606
HQ589570	A	A2	Argentina	2	183-788	606
HQ589571	A	A2	Argentina	2	183-788	606
HQ589572	A	A2	Argentina	2	183-788	606
HQ589573	A	A2	Argentina	2	183-788	606
HQ589574	A	A2	Argentina	2	183-788	606
HQ589575	A	A2	Argentina	2	183-788	606
HQ589576	A	A2	Argentina	2	183-788	606
HQ603688	A	A2	Argentina	2	186-788	603
HQ603689	A	A2	Argentina	2	186-788	603
HQ603690	A	A2	Argentina	2	186-788	603
HQ603691	A	A2	Argentina	2	186-788	603
HQ603692	A	A2	Argentina	2	186-788	603
HQ603693	A	A2	Argentina	2	186-788	603
HQ603694	A	A2	Argentina	2	186-788	603
HQ603695	A	A2	Argentina	2	186-788	603
HQ603696	A	A2	Argentina	2	186-788	603
HQ603697	A	A2	Argentina	2	186-788	603
HQ603698	A	A2	Argentina	2	186-788	603
HQ603699	A	A2	Argentina	2	186-788	603
HQ603700	A	A2	Argentina	2	186-788	603
HQ603701	A	A2	Argentina	2	186-788	603
HQ603702	A	A2	Argentina	2	186-788	603
HQ603703	A	A2	Argentina	2	186-788	603
HQ603704	A	A2	Argentina	2	186-788	603
HQ603705	A	A2	Argentina	2	186-788	603
HQ603706	A	A2	Argentina	2	186-788	603
HQ603707	A	A2	Argentina	2	186-788	603
HQ603708	A	A2	Argentina	2	186-788	603
HQ603709	A	A2	Argentina	2	186-788	603
HQ603710	A	A2	Argentina	2	186-788	603
HQ603711	A	A2	Argentina	2	186-788	603
HQ603712	A	A2	Argentina	2	186-788	603
HQ603713	A	A2	Argentina	2	186-788	603
HQ603714	A	A2	Argentina	2	186-788	603
HQ603715	A	A2	Argentina	2	186-788	603
HQ603716	A	A2	Argentina	2	186-788	603
HQ603717	A	A2	Argentina	2	186-788	603
HQ603718	A	A2	Argentina	2	186-788	603
HQ603719	A	A2	Argentina	2	186-788	603
HQ603720	A	A2	Argentina	2	186-788	603
HQ603721	A	A2	Argentina	2	186-788	603
HQ603722	A	A2	Argentina	2	186-788	603

HQ603723	A	A2	Argentina	2	186-788	603
HQ603724	A	A2	Argentina	2	186-788	603
HQ603725	A	A2	Argentina	2	186-788	603
HQ603726	A	A2	Argentina	2	186-788	603
HQ603727	A	A2	Argentina	2	186-788	603
HM154937	A	A2	Argentina	3	244-744	501
HM154943	A	A2	Argentina	3	244-747	504
HM154944	A	A2	Argentina	3	244-747	504
HM154950	A	A2	Argentina	3	244-747	504
HM154955	A	A2	Argentina	3	244-747	504
HM154956	A	A2	Argentina	3	244-744	501
HM154960	A	A2	Argentina	3	244-741	498
HM154965	A	A2	Argentina	3	244-747	504
HM154966	A	A2	Argentina	3	244-747	504
HM154968	A	A2	Argentina	3	244-744	501
HM154976	A	A2	Argentina	3	244-747	504
HM154984	A	A2	Argentina	3	244-747	504
HM154986	A	A2	Argentina	3	244-747	504
HM154987	A	A2	Argentina	3	244-747	504
HM216215	A	A1	Argentina	3	259-766	508
HM216216	A	A2	Argentina	3	259-766	508
HM216217	A	A1	Argentina	3	259-766	508
HM216218	A	A2	Argentina	3	259-766	508
HM216219	A	A2	Argentina	3	259-766	508
HM216220	A	A2	Argentina	3	259-766	508
HM216221	A	A2	Argentina	3	259-766	508
HM216222	A	A2	Argentina	3	259-766	508
HM216223	A	A2	Argentina	3	259-766	508
HM216224	A	A2	Argentina	3	259-766	508
HM216225	A	A2	Argentina	3	259-766	508
HM216256	A	A1	Argentina	3	259-766	508
HM216257	A	A2	Argentina	3	259-766	508
HM216258	A	A2	Argentina	3	259-766	508
HM216259	A	A2	Argentina	3	259-766	508
HM216260	A	A2	Argentina	3	259-766	508
HM216261	A	A1	Argentina	3	259-766	508
HM216272	A	A1	Argentina	3	259-766	508
HQ589577	A	A2	Argentina	3	252-775	524
HQ589578	A	A2	Argentina	3	252-775	524
HQ589579	A	A2	Argentina	3	252-775	524
HQ589580	A	A2	Argentina	3	252-775	524
HQ589581	A	A2	Argentina	3	252-775	524
HQ589582	A	A2	Argentina	3	252-775	524
HQ589583	A	A2	Argentina	3	252-775	524
HQ589649	A	A2	Argentina	3	282-751	470
HQ589650	A	A2	Argentina	3	282-751	470

HQ589651	A	A2	Argentina	3	282-751	470
HQ589652	A	A2	Argentina	3	282-751	470
HQ589653	A	A2	Argentina	3	282-751	470
HQ589654	A	A2	Argentina	3	282-751	470
HQ589655	A	A2	Argentina	3	282-751	470
HQ589656	A	A2	Argentina	3	282-751	470
HQ589657	A	A2	Argentina	3	282-751	470
HQ589658	A	A2	Argentina	3	282-751	470
HQ589659	A	A2	Argentina	3	282-751	470
HQ589660	A	A2	Argentina	3	282-751	470
HQ589661	A	A2	Argentina	3	282-751	470
HQ589662	A	A2	Argentina	3	282-751	470
HQ589663	A	A2	Argentina	3	282-751	470
HQ589664	A	A2	Argentina	3	282-751	470
HQ589665	A	A2	Argentina	3	282-751	470
HQ589666	A	A2	Argentina	3	282-751	470
HQ589667	A	A2	Argentina	3	282-751	470
HQ589668	A	A2	Argentina	3	282-751	470
HQ589669	A	A2	Argentina	3	282-751	470
HQ589670	A	A2	Argentina	3	282-751	470
HQ589671	A	A2	Argentina	3	282-751	470
HQ589672	A	A2	Argentina	3	282-751	470
HQ589673	A	A2	Argentina	3	282-751	470
HQ589674	A	A2	Argentina	3	282-751	470
HQ589675	A	A2	Argentina	3	282-751	470
HQ589676	A	A2	Argentina	3	282-751	470
HQ589677	A	A2	Argentina	3	282-751	470
HQ589768	A	A2	Argentina	3	255-762	508
HQ589769	A	A2	Argentina	3	255-762	508
HQ589770	A	A2	Argentina	3	255-762	508
HQ589771	A	A2	Argentina	3	255-762	508
HQ589772	A	A2	Argentina	3	255-762	508
HQ589773	A	A2	Argentina	3	255-762	508
HQ589774	A	A2	Argentina	3	255-762	508
HQ589775	A	A2	Argentina	3	255-762	508
HQ589776	A	A2	Argentina	3	255-762	508
HQ589777	A	A2	Argentina	3	255-762	508
HQ589778	A	A2	Argentina	3	255-762	508
HQ589779	A	A2	Argentina	3	255-762	508
HQ589780	A	A2	Argentina	3	255-762	508
HQ589781	A	A2	Argentina	3	255-762	508
HQ589782	A	A2	Argentina	3	255-762	508
HQ589783	A	A2	Argentina	3	255-762	508
HQ589784	A	A2	Argentina	3	255-762	508
HQ589785	A	A2	Argentina	3	255-762	508
HQ589786	A	A2	Argentina	3	255-762	508

HQ589787	A	A2	Argentina	3	255-762	508
HQ589788	A	A2	Argentina	3	255-762	508
HQ589789	A	A2	Argentina	3	255-762	508
HQ589790	A	A2	Argentina	3	255-762	508
HQ589791	A	A2	Argentina	3	255-762	508
HQ589792	A	A2	Argentina	3	255-762	508
HQ589793	A	A2	Argentina	3	255-762	508
HQ589794	A	A2	Argentina	3	255-762	508
HQ589795	A	A2	Argentina	3	255-762	508
HQ589796	A	A2	Argentina	3	255-762	508
HQ589797	A	A2	Argentina	3	255-762	508
HQ589798	A	A2	Argentina	3	255-762	508
HQ589799	A	A2	Argentina	3	255-762	508
MH763042	A	A2	Argentina	4	364-756	393
MH763043	A	A1	Argentina	4	364-756	393
MH763049	A	A2	Argentina	4	364-756	393
MH763054	A	A2	Argentina	4	364-756	393
MH763057	A	A2	Argentina	4	364-756	393
MH763062	A	A1	Argentina	4	364-756	393
MH763065	A	A1	Argentina	4	364-756	393
MH763068	A	A2	Argentina	4	364-756	393
MH763072	A	A2	Argentina	4	364-756	393
MH763076	A	A2	Argentina	4	364-756	393
MH763077	A	A1	Argentina	4	364-756	393
MH763078	A	A2	Argentina	4	364-756	393
MH763080	A	A2	Argentina	4	364-756	393
MH763081	A	A2	Argentina	4	364-756	393
MH763082	A	A2	Argentina	4	364-756	393
MH763092	A	A2	Argentina	4	364-756	393
MH763093	A	A1	Argentina	4	364-756	393
MH763096	A	A2	Argentina	4	364-756	393
MH763100	A	A2	Argentina	4	364-756	393
MH763107	A	A2	Argentina	4	364-756	393
MH763118	A	A1	Argentina	4	364-756	393
MH763120	A	A1	Argentina	4	364-756	393
MH763121	A	A2	Argentina	4	364-756	393
MH763122	A	A2	Argentina	4	364-756	393
MH763123	A	A2	Argentina	4	364-756	393
MH763124	A	A1	Argentina	4	364-756	393
MH763131	A	A2	Argentina	4	364-756	393
MH763139	A	A2	Argentina	4	364-756	393
MH763140	A	A2	Argentina	4	364-756	393
MH763142	A	A2	Argentina	4	364-756	393
MH763144	A	A2	Argentina	4	364-756	393
MH763147	A	A2	Argentina	4	364-756	393
MH763152	A	A2	Argentina	4	364-756	393

MH763157	A	A2	Argentina	4	364-756	393
MH763159	A	A1	Argentina	4	364-756	393
MH763161	A	A2	Argentina	4	364-756	393
MH763164	A	A1	Argentina	4	364-756	393
MH763165	A	A2	Argentina	4	364-756	393
MH763179	A	A2	Argentina	4	364-756	393
MH763181	A	A1	Argentina	4	364-756	393
MH763182	A	A2	Argentina	4	364-756	393
MH763183	A	A2	Argentina	4	364-756	393
MH763186	A	A2	Argentina	4	364-756	393
MH763187	A	A1	Argentina	4	364-756	393
MH763196	A	A2	Argentina	4	364-756	393
MH763198	A	A1	Argentina	4	364-756	393
MH763210	A	A2	Argentina	4	364-756	393
MH763212	A	A2	Argentina	4	364-756	393
MH763215	A	A1	Argentina	4	364-756	393
MH763219	A	A2	Argentina	4	364-756	393
MH763220	A	A2	Argentina	4	364-756	393
MH763225	A	A2	Argentina	4	364-756	393
MH763226	A	A2	Argentina	4	364-756	393
MH763229	A	A2	Argentina	4	364-756	393
MH763230	A	A2	Argentina	4	364-756	393
MH763245	A	A1	Argentina	4	364-756	393
MH763246	A	A2	Argentina	4	364-756	393
MH763249	A	A2	Argentina	4	364-756	393
MH763250	A	A1	Argentina	4	364-756	393
MH763263	A	A1	Argentina	4	364-756	393
MH763272	A	A1	Argentina	4	364-756	393
MH763273	A	A2	Argentina	4	364-756	393
MH763274	A	A2	Argentina	4	364-756	393
MH763290	A	A2	Argentina	4	364-756	393
MH763292	A	A2	Argentina	4	364-756	393
MH763304	A	A2	Argentina	4	364-756	393
MH763306	A	A1	Argentina	4	364-756	393
MH763307	A	A1	Argentina	4	364-756	393
MH763310	A	A2	Argentina	4	364-756	393
MH763311	A	A2	Argentina	4	364-756	393
MH763318	A	A1	Argentina	4	364-756	393
MH763320	A	A1	Argentina	4	364-756	393
MH763321	A	A2	Argentina	4	364-756	393
MH763322	A	A2	Argentina	4	364-756	393
MH763326	A	A2	Argentina	4	364-756	393
MH763328	A	A1	Argentina	4	364-756	393
MH763330	A	A2	Argentina	4	364-756	393
MH763332	A	A2	Argentina	4	364-756	393
MH763338	A	A1	Argentina	4	364-756	393

MH763346	A	A2	Argentina	4	364-756	393
MH763347	A	A2	Argentina	4	364-756	393
MH763348	A	A2	Argentina	4	364-756	393
MH763353	A	A2	Argentina	4	364-756	393
MH763354	A	A2	Argentina	4	364-756	393
MH763362	A	A1	Argentina	4	364-756	393
MH763363	A	A2	Argentina	4	364-756	393
MH763367	A	A2	Argentina	4	364-756	393
MH763368	A	A2	Argentina	4	364-756	393
MH763369	A	A2	Argentina	4	364-756	393
MH763370	A	A1	Argentina	4	364-756	393
MH763371	A	A2	Argentina	4	364-756	393
MH763375	A	A1	Argentina	4	364-756	393
MH763376	A	A1	Argentina	4	364-756	393
MH763379	A	A2	Argentina	4	364-756	393
MH763388	A	A2	Argentina	4	364-756	393
MH763392	A	A1	Argentina	4	364-756	393
MH763404	A	A1	Argentina	4	364-756	393
MH763407	A	A2	Argentina	4	364-756	393
MH763408	A	A2	Argentina	4	364-756	393
MH763412	A	A2	Argentina	4	364-756	393
MH763415	A	A1	Argentina	4	364-756	393
MH763416	A	A2	Argentina	4	364-756	393
MH763420	A	A1	Argentina	4	364-756	393
MH763421	A	A1	Argentina	4	364-756	393
MH763422	A	A2	Argentina	4	364-756	393
MH763423	A	A1	Argentina	4	364-756	393
MH763427	A	A2	Argentina	4	364-756	393
MH763429	A	A1	Argentina	4	364-756	393
MH763432	A	A2	Argentina	4	364-756	393
MH763433	A	A1	Argentina	4	364-756	393
MH763435	A	A2	Argentina	4	364-756	393
MH763436	A	A2	Argentina	4	364-756	393
MH763437	A	A2	Argentina	4	364-756	393
MH763439	A	A1	Argentina	4	364-756	393
MH763441	A	A2	Argentina	4	364-756	393
MH763442	A	A1	Argentina	4	364-756	393
MH763444	A	A2	Argentina	4	364-756	393
MH763445	A	A2	Argentina	4	364-756	393
MH763451	A	A2	Argentina	4	364-756	393
MH763460	A	A2	Argentina	4	364-756	393
MH763461	A	A1	Argentina	4	364-756	393
MH763467	A	A2	Argentina	4	364-756	393
MH763475	A	A2	Argentina	4	364-756	393
MH763478	A	A2	Argentina	4	364-756	393
MH763482	A	A2	Argentina	4	364-756	393

MH763488	A	A2	Argentina	4	469-756	288
MH763490	A	A2	Argentina	4	364-756	393
MH763501	A	A8	Argentina	4	364-756	393
MH763502	A	A2	Argentina	4	364-756	393
MH763505	A	A2	Argentina	4	364-756	393
MH763510	A	A2	Argentina	4	364-756	393
MH763513	A	A2	Argentina	4	364-756	393
MH763519	A	A2	Argentina	4	364-756	393
MH763521	A	A2	Argentina	4	364-756	393
MH763526	A	A2	Argentina	4	364-756	393
MH763530	A	A2	Argentina	4	514-756	243
MH763533	A	A2	Argentina	4	364-756	393
MH763544	A	A1	Argentina	4	364-756	393
MH763546	A	A2	Argentina	4	364-756	393
MH763553	A	A2	Argentina	4	364-756	393
MH763554	A	A2	Argentina	4	364-756	393
MH763555	A	A2	Argentina	4	364-756	393
AF043579	A	A2	Argentina	5	497-752	256
HM154949	A	A1	Argentina	5	244-748	505
JN688681	A	A1	Argentina	5	217-771	555
KC680772	A	A2	Argentina	5	244-768	525
KJ810922	A	A2	Argentina	5	256-800	545
KJ810923	A	A2	Argentina	5	256-1161	906
KJ810924	A	A2	Argentina	5	361-1159	799
KJ810925	A	A1	Argentina	5	252-769	518
KJ810926	A	A2	Argentina	5	312-1217	906
KJ810927	A	A1	Argentina	5	311-1217	907
KJ810928	A	A2	Argentina	5	255-1218	964
KJ810929	A	A2	Argentina	5	313-1020	708
KJ810930	A	A1	Argentina	5	256-913	658
KJ810931	A	A2	Argentina	5	250-798	429
KJ810959	A	A2	Argentina	5	256-772	517
KJ810960	A	A1	Argentina	5	255-994	740
MH763563	A	A2	Argentina	4	364-756	393

[1]Alignment to complete genome reference sequence VHB NC_003977.2

BRAZIL

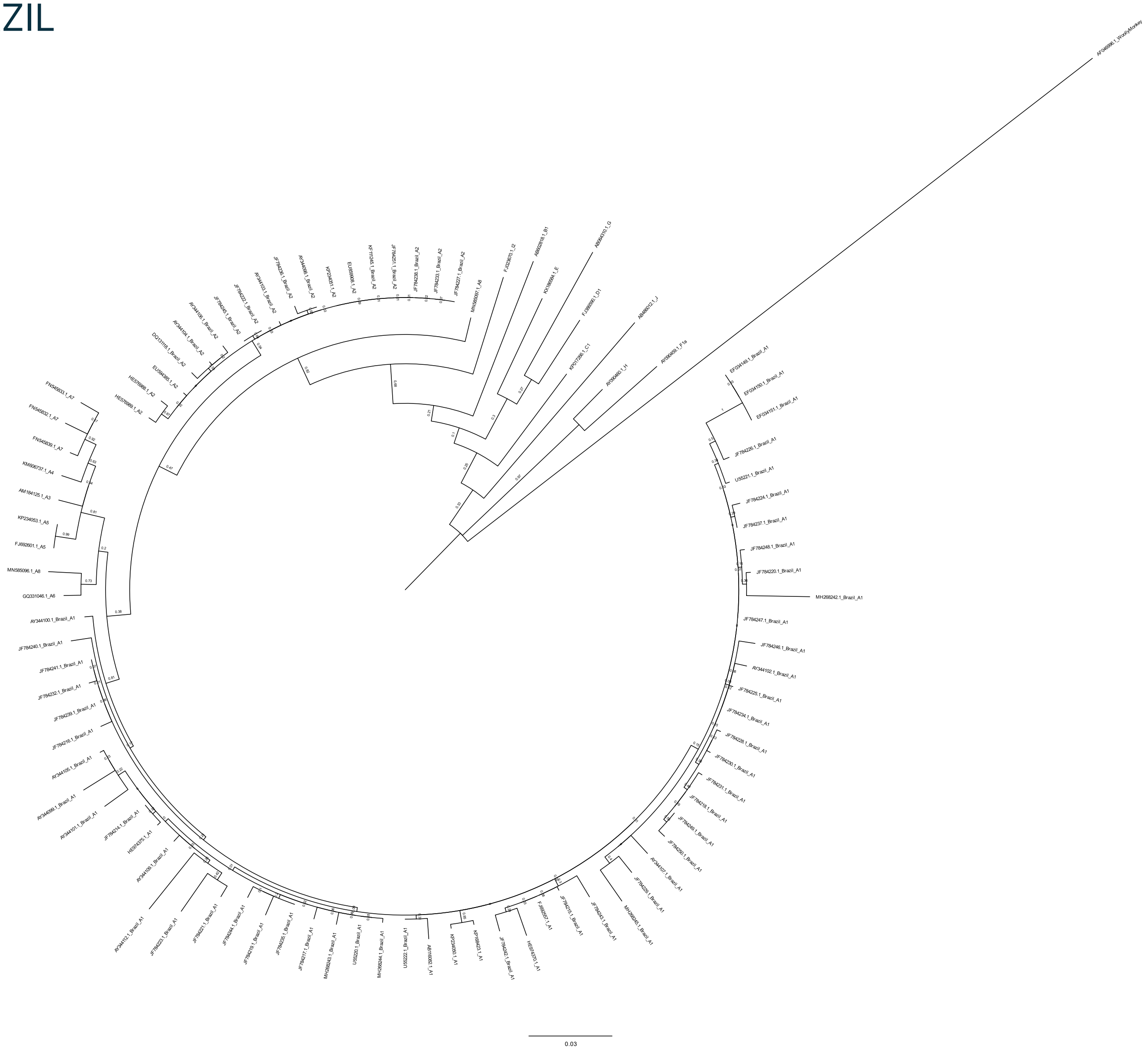

Tree 1. The evolutionary history was inferred by using the Maximum Likelihood method and Tamura-Nei model. The percentage of replicate trees in which the associated taxa clustered together in the bootstrap test (1000 replicates) are shown next to the branches. Initial tree(s) for the heuristic search were obtained automatically by applying Neighbor-Join and BioNJ algorithms to a matrix of pairwise distances estimated using the Tamura-Nei model, and then selecting the topology with superior log likelihood value. A discrete Gamma distribution was used to model evolutionary rate differences among sites (5 categories (+G, parameter = 0.2779)). The tree is drawn to scale, with branch lengths measured in the number of substitutions per site. The analysis involved 93 nucleotide sequences, of which 31 were used as marker sequences to determine the genotype of 62 sequences. All positions containing gaps and missing data were eliminated. There was a total of 755 positions in the final dataset. Evolutionary analyses were conducted in MEGA X.

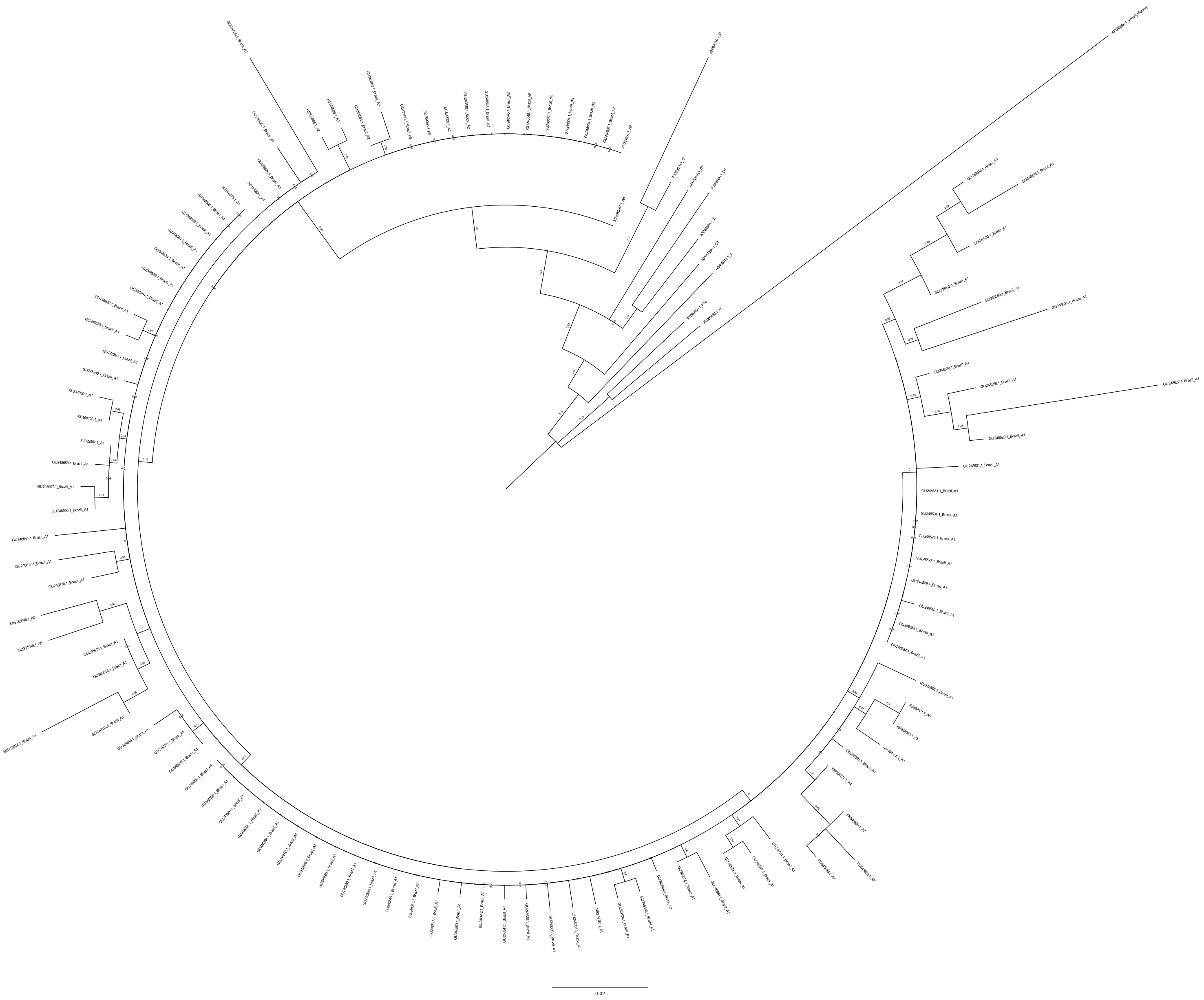

Tree 2. The evolutionary history was inferred by using the Maximum Likelihood method and Tamura-Nei model. The percentage of replicate trees in which the associated taxa clustered together in the bootstrap test (1000 replicates) are shown next to the branches. Initial tree(s) for the heuristic search were obtained automatically by applying Neighbor-Join and BioNJ algorithms to a matrix of pairwise distances estimated using the Tamura-Nei model, and then selecting the topology with superior log likelihood value. A discrete Gamma distribution was used to model evolutionary rate differences among sites (5 categories (+G, parameter = 0.3115)). The tree is drawn to scale, with branch lengths measured in the number of substitutions per site. The analysis involved 116 nucleotide sequences, of which 31 were used as marker sequences to determine the genotype of 85 sequences. All positions containing gaps and missing data were eliminated. There was a total of 380 positions in the final dataset. Evolutionary analyses were conducted in MEGA X.

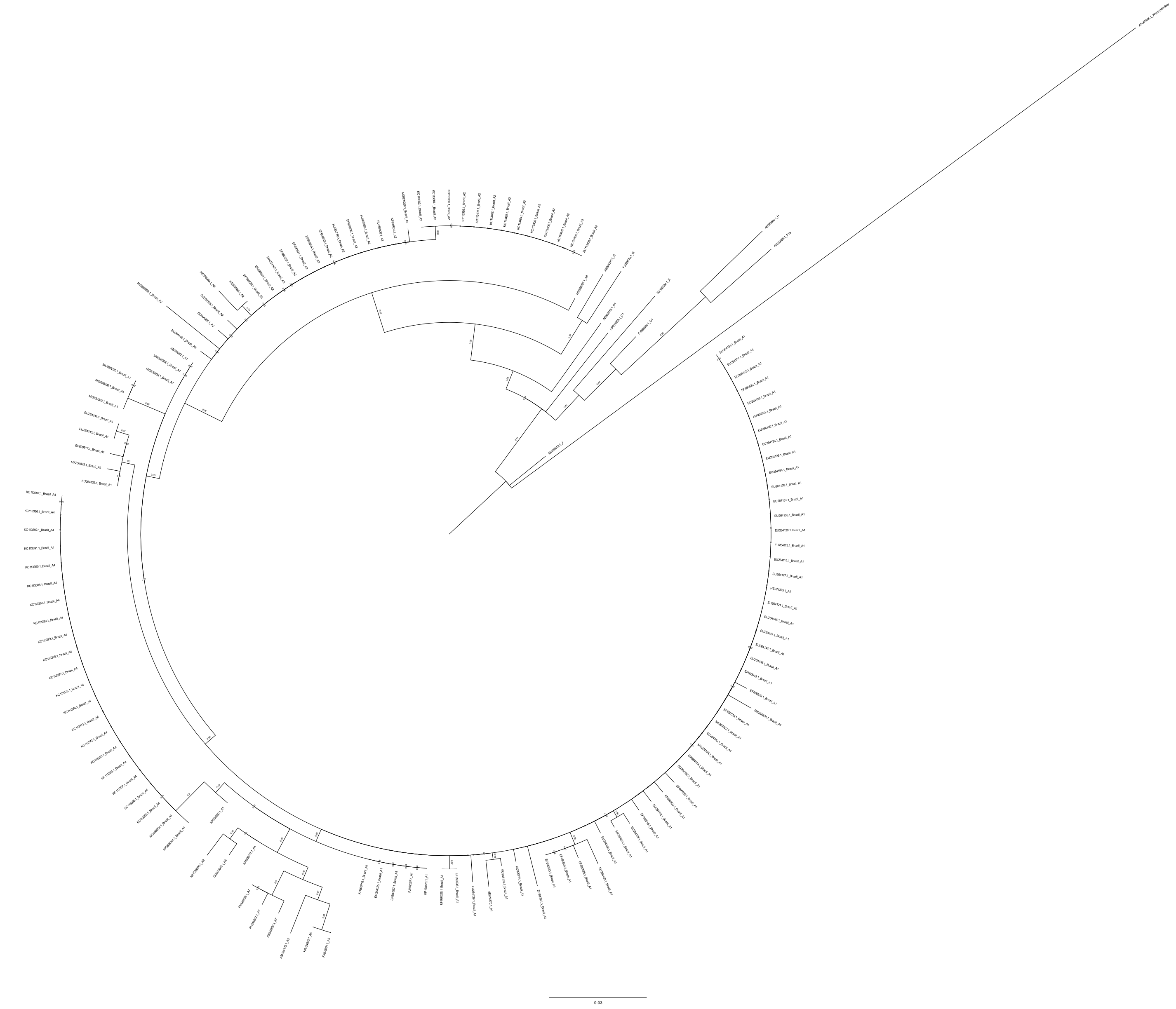

Tree 3. The evolutionary history was inferred by using the Maximum Likelihood method and Tamura-Nei model. The percentage of replicate trees in which the associated taxa clustered together in the bootstrap test (1000 replicates) are shown next to the branches. Initial tree(s) for the heuristic search were obtained automatically by applying Neighbor-Join and BioNJ algorithms to a matrix of pairwise distances estimated using the Tamura-Nei model, and then selecting the topology with superior log likelihood value. A discrete Gamma distribution was used to model evolutionary rate differences among sites (5 categories (+G, parameter = 0.1893)). The tree is drawn to scale, with branch lengths measured in the number of substitutions per site. The analysis involved 141 nucleotide sequences, of which 31 were used as marker sequences to determine the genotype of 110 sequences. All positions containing gaps and missing data were eliminated. There was a total of 306 positions in the final dataset. Evolutionary analyses were conducted in MEGA X.

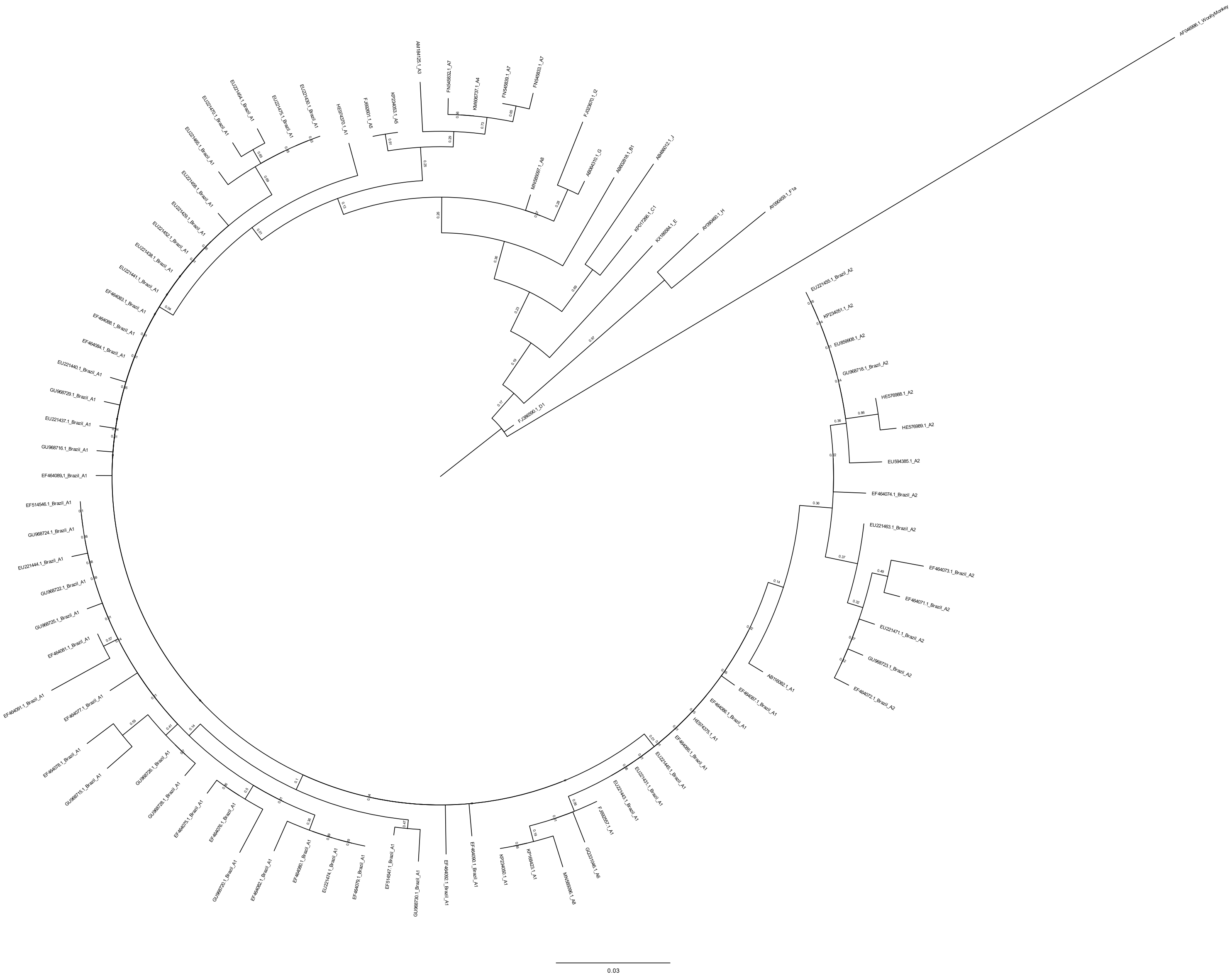

Tree 4. The evolutionary history was inferred by using the Maximum Likelihood method and Tamura-Nei model. The percentage of replicate trees in which the associated taxa clustered together in the bootstrap test (1000 replicates) are shown next to the branches. Initial tree(s) for the heuristic search were obtained automatically by applying Neighbor-Join and BioNJ algorithms to a matrix of pairwise distances estimated using the Tamura-Nei model, and then selecting the topology with superior log likelihood value. A discrete Gamma distribution was used to model evolutionary rate differences among sites (5 categories (+G, parameter = 0.2255)). The tree is drawn to scale, with branch lengths measured in the number of substitutions per site. The analysis involved 87 nucleotide sequences, of which 31 were used as marker sequences to determine the genotype of 56 sequences. All positions containing gaps and missing data were eliminated. There was a total of 266 positions in the final dataset. Evolutionary analyses were conducted in MEGA X.

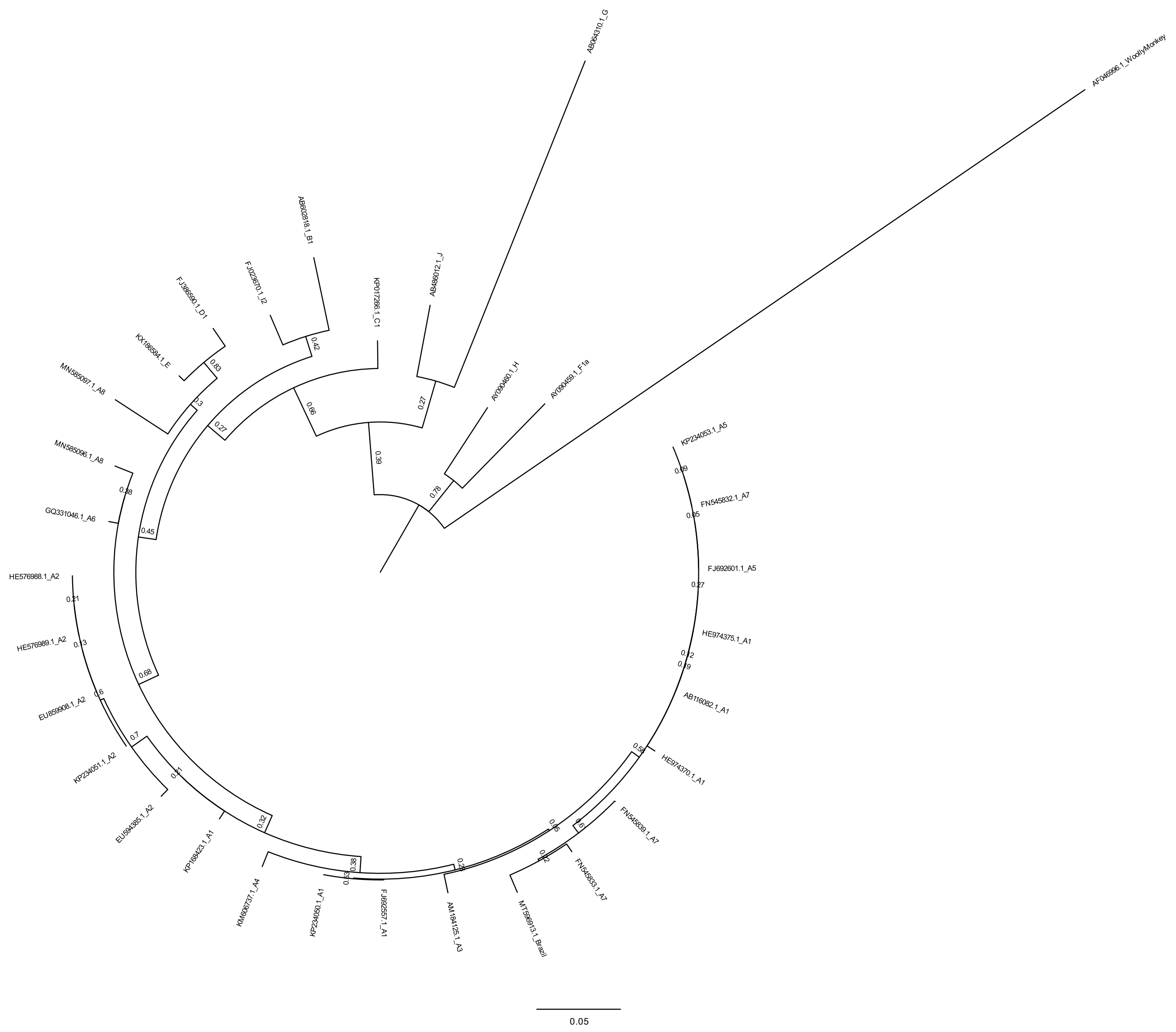

Tree 5. The evolutionary history was inferred by using the Maximum Likelihood method and Tamura-Nei model. The percentage of replicate trees in which the associated taxa clustered together in the bootstrap test (1000 replicates) are shown next to the branches. Initial tree(s) for the heuristic search were obtained automatically by applying Neighbor-Join and BioNJ algorithms to a matrix of pairwise distances estimated using the Tamura-Nei model, and then selecting the topology with superior log likelihood value. A discrete Gamma distribution was used to model evolutionary rate differences among sites (5 categories (+G, parameter = 0.2938)). The tree is drawn to scale, with branch lengths measured in the number of substitutions per site. The analysis involved 32 nucleotide sequences, of which 31 were used as marker sequences to determine the genotype of 1 sequence. All positions containing gaps and missing data were eliminated. There was a total of 189 positions in the final dataset. Evolutionary analyses were conducted in MEGA X.

ID	GENOTYPE	SUBTYPE	COUNTRY	TREE	ALIGNMENT[1]	BASE PAIRS
AY344098	A	A2	Brazil	1	1-3182	1207
AY344099	A	A1	Brazil	1	1-3182	1207
AY344100	A	A1	Brazil	1	1-3182	1207
AY344101	A	A1	Brazil	1	1-3182	1201

AY344102	A	A1	Brazil	1	1-3182	1148
AY344103	A	A2	Brazil	1	1-3182	1207
AY344104	A	A2	Brazil	1	1-3182	1207
AY344105	A	A1	Brazil	1	1-3182	1207
AY344106	A	A2	Brazil	1	1-3182	1207
AY344107	A	A1	Brazil	1	1-3182	1207
AY344109	A	A1	Brazil	1	1-3182	1207
AY344112	A	A1	Brazil	1	1-3182	1180
DQ131118	A	A2	Brazil	1	1-3182	1204
EF034149	A	A1	Brazil	1	1-3182	1181
EF034150	A	A1	Brazil	1	1-3182	1181
EF034151	A	A1	Brazil	1	1-3182	1181
JF784214	A	A1	Brazil	1	1-3182	1187
JF784215	A	A1	Brazil	1	1-3182	1187
JF784216	A	A1	Brazil	1	1-3182	1187
JF784217	A	A1	Brazil	1	1-3182	1187
JF784218	A	A1	Brazil	1	1-3182	1187
JF784219	A	A1	Brazil	1	1-3182	1187
JF784220	A	A1	Brazil	1	1-3182	1136
JF784221	A	A1	Brazil	1	1-3182	1139
JF784222	A	A2	Brazil	1	1-3182	1187
JF784223	A	A1	Brazil	1	1-3182	1187
JF784224	A	A1	Brazil	1	1-3182	1187
JF784225	A	A1	Brazil	1	1-3182	1187
JF784226	A	A1	Brazil	1	1-3182	1187
JF784227	A	A2	Brazil	1	1-3182	1187
JF784228	A	A1	Brazil	1	1-3182	1187
JF784229	A	A1	Brazil	1	1-3182	1187
JF784230	A	A1	Brazil	1	1-3182	1187
JF784231	A	A1	Brazil	1	1-3182	1187
JF784232	A	A1	Brazil	1	1-3182	1187
JF784233	A	A2	Brazil	1	1-3182	1187
JF784234	A	A1	Brazil	1	1-3182	1187
JF784235	A	A1	Brazil	1	1-3182	1157
JF784236	A	A2	Brazil	1	1-3182	1187
JF784237	A	A1	Brazil	1	1-3182	1187
JF784238	A	A2	Brazil	1	1-3182	1187
JF784239	A	A1	Brazil	1	1-3182	1187
JF784240	A	A1	Brazil	1	1-3182	1187
JF784241	A	A1	Brazil	1	1-3182	1187
JF784242	A	A1	Brazil	1	1-3182	1187
JF784243	A	A1	Brazil	1	46-3121	974
JF784244	A	A1	Brazil	1	1-3182	1187
JF784245	A	A2	Brazil	1	1-3182	1187
JF784246	A	A1	Brazil	1	1-3182	1187
JF784247	A	A1	Brazil	1	1-3182	1187

JF784248	A	A1	Brazil	1	1-3182	1187
JF784249	A	A1	Brazil	1	1-3182	1187
JF784250	A	A1	Brazil	1	1-3182	1187
JF784251	A	A2	Brazil	1	1-3182	1187
KF111245	A	A2	Brazil	1	1-3182	1184
MH268242	A	A1	Brazil	1	24-3181	1028
MH268243	A	A1	Brazil	1	1-3182	1052
MH268244	A	A1	Brazil	1	1-3182	1052
MH268245	A	A1	Brazil	1	1-3182	1052
U55220	A	A1	Brazil	1	1-3182	1182
U55221	A	A1	Brazil	1	1-3182	1182
U55222	A	A1	Brazil	1	1-3182	1182
DQ131121	A	A2	Brazil	2	1-3182	847
GU248531	A	A1	Brazil	2	1-3182	852
GU248534	A	A1	Brazil	2	1-3182	852
GU248535	A	A1	Brazil	2	1-3182	852
GU248537	A	A1	Brazil	2	1-3182	852
GU248538	A	A2	Brazil	2	1-3182	812
GU248540	A	A1	Brazil	2	1-3182	839
GU248541	A	A1	Brazil	2	1-3182	830
GU248543	A	A2	Brazil	2	1-3182	852
GU248544	A	A1	Brazil	2	1-3182	839
GU248545	A	A2	Brazil	2	1-3182	843
GU248546	A	A2	Brazil	2	1-3182	852
GU248547	A	A1	Brazil	2	1-3182	852
GU248548	A	A1	Brazil	2	1-3182	838
GU248549	A	A1	Brazil	2	1-3182	813
GU248550	A	A1	Brazil	2	46-3182	798
GU248552	A	A1	Brazil	2	1-3182	842
GU248553	A	A2	Brazil	2	1-3182	852
GU248554	A	A1	Brazil	2	1-3182	839
GU248555	A	A1	Brazil	2	1-3182	852
GU248556	A	A1	Brazil	2	1-3182	852
GU248558	A	A1	Brazil	2	1-3182	839
GU248559	A	A1	Brazil	2	1-3182	834
GU248560	A	A1	Brazil	2	1-3182	839
GU248561	A	A1	Brazil	2	1-3182	852
GU248564	A	A1	Brazil	2	1-3182	839
GU248566	A	A1	Brazil	2	1-3182	852
GU248568	A	A1	Brazil	2	1-3182	840
GU248569	A	A1	Brazil	2	1-3182	834
GU248570	A	A1	Brazil	2	1-3182	852
GU248572	A	A2	Brazil	2	1-3182	841
GU248573	A	A1	Brazil	2	1-3182	852
GU248574	A	A1	Brazil	2	1-3182	852
GU248575	A	A1	Brazil	2	1-3182	816

GU248576	A	A1	Brazil	2	1-3182	841
GU248577	A	A1	Brazil	2	1-3182	852
GU248578	A	A1	Brazil	2	1-3182	852
GU248579	A	A1	Brazil	2	1-3182	852
GU248581	A	A1	Brazil	2	1-3182	852
GU248582	A	A1	Brazil	2	1-3182	852
GU248584	A	A1	Brazil	2	1-3182	839
GU248586	A	A1	Brazil	2	1-3182	852
GU248587	A	A1	Brazil	2	43-3182	810
GU248588	A	A1	Brazil	2	1-3182	852
GU248590	A	A1	Brazil	2	1-3182	844
GU248591	A	A1	Brazil	2	1-3182	852
GU248592	A	A1	Brazil	2	1-3182	852
GU248593	A	A1	Brazil	2	1-3182	852
GU248594	A	A1	Brazil	2	1-3182	839
GU248595	A	A1	Brazil	2	1-3182	852
GU248596	A	A1	Brazil	2	1-3182	852
GU248597	A	A1	Brazil	2	1-3182	852
GU248598	A	A1	Brazil	2	1-3182	852
GU248599	A	A1	Brazil	2	1-3182	852
GU248601	A	A2	Brazil	2	1-3182	852
GU248602	A	A2	Brazil	2	1-3182	852
GU248604	A	A2	Brazil	2	1-3182	852
GU248605	A	A2	Brazil	2	1-3182	852
GU248606	A	A1	Brazil	2	1-3182	852
GU248607	A	A1	Brazil	2	1-3182	843
GU248608	A	A1	Brazil	2	1-3182	852
GU248610	A	A1	Brazil	2	1-3182	842
GU248611	A	A1	Brazil	2	1-3182	852
GU248612	A	A1	Brazil	2	1-3182	842
GU248613	A	A1	Brazil	2	1-3182	852
GU248614	A	A1	Brazil	2	1-3182	852
GU248616	A	A1	Brazil	2	1-3182	795
GU248617	A	A1	Brazil	2	1-3182	837
GU248618	A	A1	Brazil	2	1-3182	852
GU248619	A	A1	Brazil	2	1-3182	852
GU248620	A	A1	Brazil	2	1-3182	852
GU248621	A	A1	Brazil	2	1-3182	852
GU248623	A	A1	Brazil	2	1-3182	852
GU248624	A	A1	Brazil	2	1-3182	852
GU248626	A	A1	Brazil	2	1-3182	812
GU248627	A	A1	Brazil	2	1-3182	844
GU248628	A	A1	Brazil	2	1-3182	843
GU248629	A	A2	Brazil	2	1-3182	797
GU248630	A	A1	Brazil	2	1-3182	832
GU248631	A	A1	Brazil	2	1-3182	791

GU248632	A	A1	Brazil	2	1-3182	821
GU248633	A	A1	Brazil	2	1-3182	841
GU248634	A	A1	Brazil	2	1-3182	828
GU248635	A	A1	Brazil	2	1-3182	809
MK173814	A	A1	Brazil	2	19-3180	740
EF690515	A	A1	Brazil	3	157-837	681
EF690516	A	A1	Brazil	3	157-837	681
EF690517	A	A1	Brazil	3	157-837	681
EF690518	A	A1	Brazil	3	157-837	681
EF690519	A	A1	Brazil	3	157-837	681
EF690520	A	A1	Brazil	3	157-837	681
EF690521	A	A1	Brazil	3	157-837	681
EF690522	A	A1	Brazil	3	157-837	681
EF690523	A	A1	Brazil	3	157-837	681
EF690524	A	A1	Brazil	3	157-837	681
EF690525	A	A1	Brazil	3	157-837	681
EF690526	A	A1	Brazil	3	157-837	681
EF690527	A	A1	Brazil	3	157-837	681
EF690535	A	A1	Brazil	3	157-837	681
EF690536	A	A1	Brazil	3	157-837	681
EU264113	A	A1	Brazil	3	157-756	600
EU264115	A	A1	Brazil	3	162-756	595
EU264116	A	A1	Brazil	3	157-756	600
EU264118	A	A1	Brazil	3	163-746	584
EU264119	A	A1	Brazil	3	171-756	586
EU264120	A	A1	Brazil	3	197-756	560
EU264121	A	A1	Brazil	3	161-755	592
EU264122	A	A1	Brazil	3	189-756	568
EU264123	A	A1	Brazil	3	195-752	558
EU264125	A	A1	Brazil	3	166-746	581
EU264126	A	A1	Brazil	3	195-756	562
EU264127	A	A1	Brazil	3	163-742	580
EU264128	A	A1	Brazil	3	157-756	600
EU264129	A	A1	Brazil	3	194-756	563
EU264131	A	A1	Brazil	3	169-756	588
EU264133	A	A1	Brazil	3	200-756	557
EU264134	A	A1	Brazil	3	163-756	594
EU264135	A	A1	Brazil	3	197-755	559
EU264136	A	A1	Brazil	3	163-756	594
EU264138	A	A1	Brazil	3	161-744	584
EU264139	A	A1	Brazil	3	170-756	587
EU264140	A	A1	Brazil	3	178-752	575
EU264141	A	A1	Brazil	3	194-747	554
EU264143	A	A1	Brazil	3	165-756	592
EU264145	A	A1	Brazil	3	163-756	594
EU264147	A	A1	Brazil	3	197-756	560

EU264150	A	A1	Brazil	3	157-756	600
EU264151	A	A1	Brazil	3	183-756	574
EU264152	A	A1	Brazil	3	196-756	561
EU264154	A	A1	Brazil	3	162-756	595
EU264155	A	A1	Brazil	3	171-756	586
EU264156	A	A1	Brazil	3	183-756	574
KU900751	A	A1	Brazil	3	157-837	681
KU900753	A	A1	Brazil	3	157-837	681
KU900754	A	A1	Brazil	3	157-837	681
MG839201	A	A1	Brazil	3	157-837	681
MG839202	A	A1	Brazil	3	157-837	681
MG839203	A	A1	Brazil	3	157-837	681
MG839204	A	A1	Brazil	3	157-837	681
MG839205	A	A1	Brazil	3	157-837	681
MG839206	A	A1	Brazil	3	157-837	681
MG839207	A	A1	Brazil	3	157-837	681
MK854819	A	A1	Brazil	3	320-951	632
MK854821	A	A1	Brazil	3	320-951	632
MK854822	A	A1	Brazil	3	320-951	632
MK854823	A	A1	Brazil	3	320-950	631
MK854824	A	A1	Brazil	3	320-951	632
MN224164	A	A1	Brazil	3	410-938	528
DQ131125	A	A2	Brazil	3	157-838	682
EF690528	A	A2	Brazil	3	157-837	681
EF690529	A	A2	Brazil	3	157-837	681
EF690530	A	A2	Brazil	3	157-837	681
EF690531	A	A2	Brazil	3	157-837	681
EF690532	A	A2	Brazil	3	157-837	681
EF690533	A	A2	Brazil	3	157-837	681
EF690534	A	A2	Brazil	3	157-837	681
EU264149	A	A2	Brazil	3	163-756	594
KC113382	A	A2	Brazil	3	157-837	681
KC113384	A	A2	Brazil	3	157-837	681
KC113385	A	A2	Brazil	3	157-837	681
KC113398	A	A2	Brazil	3	157-837	681
KC113401	A	A2	Brazil	3	157-837	681
KC113402	A	A2	Brazil	3	157-837	681
KC113403	A	A2	Brazil	3	157-837	681
KC113404	A	A2	Brazil	3	157-837	681
KC113405	A	A2	Brazil	3	157-837	681
KC113406	A	A2	Brazil	3	157-837	681
KC113407	A	A2	Brazil	3	157-837	681
KC113408	A	A2	Brazil	3	157-837	681
KC113409	A	A2	Brazil	3	157-837	681
KU900750	A	A2	Brazil	3	157-837	681
KU900752	A	A2	Brazil	3	157-837	681

MG839208	A	A2	Brazil	3	157-837	681
MG839209	A	A2	Brazil	3	157-837	681
MN224163	A	A2	Brazil	3	410-938	529
KC113365	A	A1	Brazil	3	157-837	681
KC113366	A	A1	Brazil	3	157-837	681
KC113367	A	A1	Brazil	3	157-837	681
KC113369	A	A1	Brazil	3	157-837	681
KC113370	A	A1	Brazil	3	157-837	681
KC113372	A	A1	Brazil	3	157-837	681
KC113373	A	A1	Brazil	3	157-837	681
KC113374	A	A1	Brazil	3	157-837	681
KC113376	A	A1	Brazil	3	157-837	681
KC113377	A	A1	Brazil	3	157-837	681
KC113378	A	A1	Brazil	3	157-837	681
KC113379	A	A1	Brazil	3	157-837	681
KC113380	A	A1	Brazil	3	157-837	681
KC113387	A	A1	Brazil	3	157-837	681
KC113388	A	A1	Brazil	3	157-837	681
KC113390	A	A1	Brazil	3	157-837	681
KC113391	A	A1	Brazil	3	157-837	681
KC113392	A	A1	Brazil	3	157-837	681
KC113396	A	A1	Brazil	3	157-837	681
KC113397	A	A1	Brazil	3	157-837	681
EF464071	A	A2	Brazil	4	417-841	425
EF464072	A	A2	Brazil	4	417-841	425
EF464073	A	A2	Brazil	4	417-841	425
EF464074	A	A2	Brazil	4	417-841	425
EF464075	A	A1	Brazil	4	417-841	425
EF464076	A	A1	Brazil	4	417-841	425
EF464077	A	A1	Brazil	4	417-841	425
EF464078	A	A1	Brazil	4	417-841	425
EF464079	A	A1	Brazil	4	417-841	425
EF464080	A	A1	Brazil	4	417-841	425
EF464081	A	A1	Brazil	4	417-841	425
EF464082	A	A1	Brazil	4	417-841	425
EF464083	A	A1	Brazil	4	417-841	425
EF464084	A	A1	Brazil	4	417-841	425
EF464085	A	A1	Brazil	4	417-841	425
EF464086	A	A1	Brazil	4	417-841	425
EF464087	A	A1	Brazil	4	417-841	425
EF464088	A	A1	Brazil	4	417-841	425
EF464089	A	A1	Brazil	4	417-841	425
EF464090	A	A1	Brazil	4	417-841	425
EF464091	A	A1	Brazil	4	417-841	425
EF464092	A	A1	Brazil	4	417-841	425
EF514546	A	A1	Brazil	4	417-841	425

EF514547	A	A1	Brazil	4	417-841	425
EU221429	A	A1	Brazil	4	519-997	497
EU221430	A	A1	Brazil	4	519-997	497
EU221431	A	A1	Brazil	4	519-997	497
EU221437	A	A1	Brazil	4	519-997	497
EU221438	A	A1	Brazil	4	519-997	497
EU221440	A	A1	Brazil	4	519-997	497
EU221441	A	A1	Brazil	4	519-997	497
EU221443	A	A1	Brazil	4	519-997	497
EU221444	A	A1	Brazil	4	519-997	497
EU221445	A	A1	Brazil	4	519-997	497
EU221452	A	A1	Brazil	4	519-997	497
EU221454	A	A1	Brazil	4	519-997	497
EU221455	A	A2	Brazil	4	519-997	497
EU221458	A	A1	Brazil	4	519-997	497
EU221463	A	A2	Brazil	4	519-997	497
EU221465	A	A1	Brazil	4	519-997	497
EU221470	A	A1	Brazil	4	519-997	497
EU221471	A	A2	Brazil	4	519-997	497
EU221474	A	A1	Brazil	4	519-997	497
EU221475	A	A1	Brazil	4	519-997	497
GU968715	A	A1	Brazil	4	312-789	478
GU968716	A	A1	Brazil	4	312-789	478
GU968718	A	A2	Brazil	4	312-789	478
GU968720	A	A1	Brazil	4	312-789	478
GU968722	A	A1	Brazil	4	312-789	478
GU968723	A	A2	Brazil	4	312-789	478
GU968724	A	A1	Brazil	4	312-789	478
GU968725	A	A1	Brazil	4	312-789	478
GU968726	A	A1	Brazil	4	312-789	478
GU968728	A	A1	Brazil	4	312-789	478
GU968729	A	A1	Brazil	4	312-789	478
GU968730	A	A1	Brazil	4	312-789	478
MT596913	A	A7	Brazil	5	1649-1853	205

[1]Alignment to complete genome reference sequence VHB NC_003977.2

CANADA

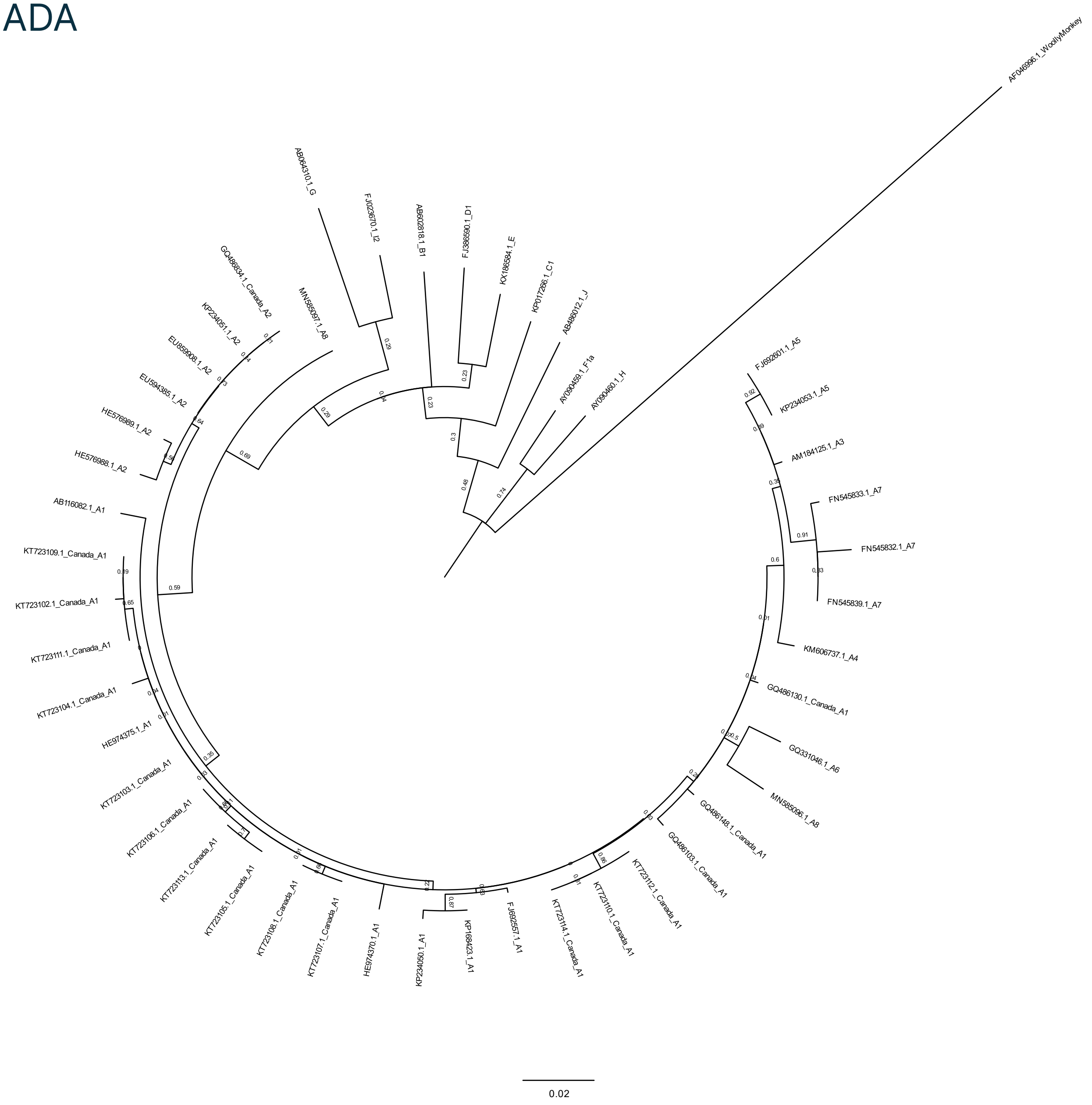

Tree 1. The evolutionary history was inferred by using the Maximum Likelihood method and Tamura-Nei model. The percentage of replicate trees in which the associated taxa clustered together in the bootstrap test (1000 replicates) are shown next to the branches. Initial tree(s) for the heuristic search were obtained automatically by applying Neighbor-Join and BioNJ algorithms to a matrix of pairwise distances estimated using the Tamura-Nei model, and then selecting the topology with superior log likelihood value. A discrete Gamma distribution was used to model evolutionary rate differences among sites (5 categories (+G, parameter = 0.1680)). The tree is drawn to scale, with branch lengths measured in the number of substitutions per site. The analysis involved 48 nucleotide sequences, of which 31 were used as marker sequences to determine the genotype of 17 sequences. All positions containing gaps and missing data were eliminated. There was a total of 444 positions in the final dataset. Evolutionary analyses were conducted in MEGA X.

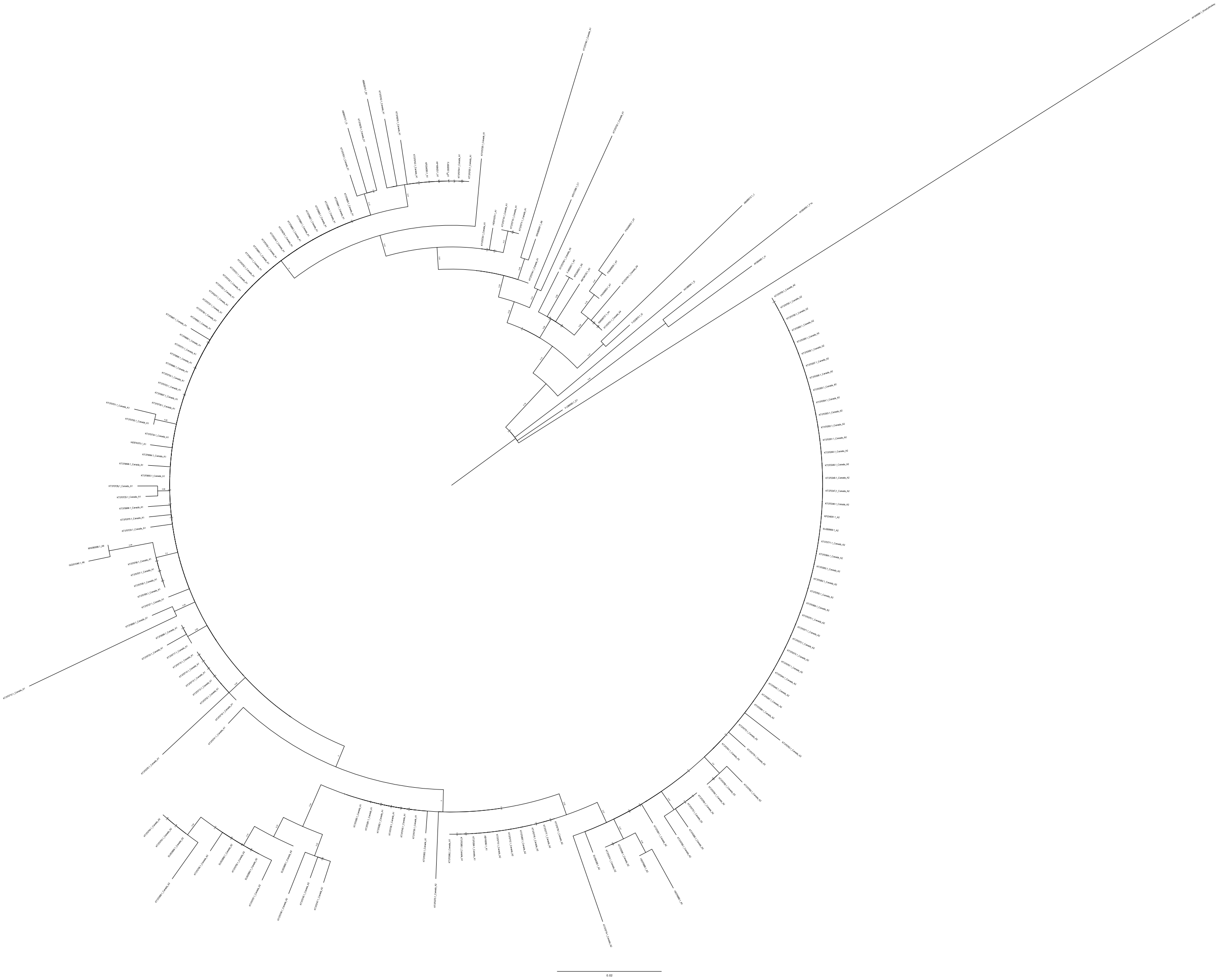

Tree 2. The evolutionary history was inferred by using the Maximum Likelihood method and Tamura-Nei model. The percentage of replicate trees in which the associated taxa clustered together in the bootstrap test (1000 replicates) are shown next to the branches. Initial tree(s) for the heuristic search were obtained automatically by applying Neighbor-Join and BioNJ algorithms to a matrix of pairwise distances estimated using the Tamura-Nei model, and then selecting the topology with superior log likelihood value. A discrete Gamma distribution was used to model evolutionary rate differences among sites (5 categories (+G, parameter = 0.2541)). The tree is drawn to scale, with branch lengths measured in the number of substitutions per site. The analysis involved 185 nucleotide sequences, of which 31 were used as marker sequences to determine the genotype of 154 sequences. All positions containing gaps and missing data were eliminated. There was a total of 287 positions in the final dataset. Evolutionary analyses were conducted in MEGA X.

ID	GENOTYPE	SUBTYPE	COUNTRY	TREE	ALIGNMENT[1]	BASE PAIRS
GQ486103	A	A1	Canada	1	132-1163	1032
GQ486130	A	A1	Canada	1	132-1163	1032

GQ486148	A	A1	Canada	1	132-1163	1032
GQ486834	A	A2	Canada	1	132-1163	1032
KT723103	A	A1	Canada	1	1-1166	964
KT723104	A	A1	Canada	1	1-1166	982
KT723105	A	A1	Canada	1	1-1166	982
KT723106	A	A1	Canada	1	1-1166	982
KT723107	A	A1	Canada	1	1-1166	964
KT723108	A	A1	Canada	1	1-1166	964
KT723109	A	A1	Canada	1	1-1166	982
KT723110	A	A1	Canada	1	1-1166	982
KT723111	A	A1	Canada	1	1-1166	982
KT723112	A	A1	Canada	1	1-1166	982
KT723113	A	A1	Canada	1	1-1166	982
KT723114	A	A1	Canada	1	1-1166	982
KT723102	A	A1	Canada	1	1-3182	982
EU833892	A	A2	Canada	2	417-827	411
EU833893	A	A2	Canada	2	417-827	411
EU833894	A	A2	Canada	2	417-827	411
EU833895	A	A2	Canada	2	417-827	411
KT370344	A	A2	Canada	2	313-831	519
KT370345	A	A2	Canada	2	313-831	519
KT370346	A	A2	Canada	2	313-831	519
KT370347	A	A2	Canada	2	313-831	519
KT370348	A	A2	Canada	2	313-831	519
KT370349	A	A2	Canada	2	313-831	519
KT370350	A	A2	Canada	2	313-831	519
KT370351	A	A2	Canada	2	313-831	519
KT370352	A	A2	Canada	2	313-831	519
KT370353	A	A2	Canada	2	313-831	519
KT370354	A	A2	Canada	2	313-831	519
KT370355	A	A2	Canada	2	313-831	519
KT370356	A	A2	Canada	2	313-831	519
KT370357	A	A2	Canada	2	313-831	519
KT370358	A	A2	Canada	2	313-831	519
KT370359	A	A2	Canada	2	313-831	519
KT370360	A	A2	Canada	2	313-831	519
KT370361	A	A2	Canada	2	313-831	519
KT370362	A	A2	Canada	2	313-831	519
KT370363	A	A2	Canada	2	313-831	519
KT370364	A	A2	Canada	2	313-831	519
KT370365	A	A2	Canada	2	313-831	519
KT370366	A	A2	Canada	2	313-831	519
KT370367	A	A2	Canada	2	313-831	519
KT370368	A	A2	Canada	2	313-831	519
KT370369	A	A2	Canada	2	313-831	519
KT370370	A	A2	Canada	2	313-831	519

KT370371	A	A2	Canada	2	313-831	519
KT370372	A	A2	Canada	2	313-831	519
KT370373	A	A2	Canada	2	313-831	519
KT370374	A	A2	Canada	2	313-831	519
KT370375	A	A2	Canada	2	313-831	519
KT370376	A	A1	Canada	2	313-831	519
KT370377	A	A1	Canada	2	313-831	519
KT370378	A	A1	Canada	2	313-831	519
KT370379	A	A1	Canada	2	313-831	519
KT370380	A	A1	Canada	2	313-831	519
KT370381	A	A1	Canada	2	313-831	519
KT370382	A	A1	Canada	2	313-831	519
KT370383	A	A1	Canada	2	327-831	505
KT370384	A	A1	Canada	2	313-831	519
KT370385	A	A1	Canada	2	323-831	509
KT370386	A	A1	Canada	2	313-831	519
KT370394	A	A2	Canada	2	456-794	339
KT370396	A	A2	Canada	2	415-822	408
KT370676	A	A1	Canada	2	313-831	519
KT370677	A	A1	Canada	2	313-831	519
KT370678	A	A1	Canada	2	313-831	519
KT370679	A	A1	Canada	2	329-831	503
KT370680	A	A1	Canada	2	313-831	519
KT370681	A	A1	Canada	2	313-831	519
KT370682	A	A1	Canada	2	313-831	519
KT370683	A	A1	Canada	2	313-831	519
KT370684	A	A1	Canada	2	313-831	519
KT370685	A	A1	Canada	2	313-831	519
KT370686	A	A1	Canada	2	313-831	519
KT370687	A	A1	Canada	2	313-831	519
KT370688	A	A1	Canada	2	313-831	519
KT370689	A	A1	Canada	2	313-831	519
KT370690	A	A1	Canada	2	313-831	519
KT370691	A	A1	Canada	2	313-831	519
KT370692	A	A1	Canada	2	313-831	519
KT370693	A	A1	Canada	2	313-831	519
KT370694	A	A1	Canada	2	313-831	519
KT370695	A	A1	Canada	2	313-831	519
KT370696	A	A1	Canada	2	313-831	519
KT370697	A	A1	Canada	2	313-831	519
KT370698	A	A1	Canada	2	313-831	519
KT370699	A	A1	Canada	2	313-831	519
KT370700	A	A1	Canada	2	313-831	519
KT370701	A	A1	Canada	2	313-831	519
KT370702	A	A1	Canada	2	313-831	519
KT370703	A	A1	Canada	2	313-831	519

KT370704	A	A1	Canada	2	313-831	519
KT370705	A	A1	Canada	2	313-831	519
KT370706	A	A1	Canada	2	313-831	519
KT370707	A	A1	Canada	2	313-831	519
KT370708	A	A1	Canada	2	313-831	519
KT370709	A	A1	Canada	2	313-831	519
KT370710	A	A1	Canada	2	313-831	519
KT370711	A	A1	Canada	2	313-831	519
KT370712	A	A1	Canada	2	313-831	519
KT370713	A	A1	Canada	2	313-831	519
KT370714	A	A1	Canada	2	313-831	519
KT370715	A	A1	Canada	2	313-831	519
KT370716	A	A1	Canada	2	313-831	519
KT370717	A	A1	Canada	2	313-831	519
KT370718	A	A1	Canada	2	313-831	519
KT370719	A	A1	Canada	2	313-831	519
KT370720	A	A1	Canada	2	313-831	519
KT370721	A	A1	Canada	2	313-831	519
KT370722	A	A1	Canada	2	313-831	519
KT370723	A	A1	Canada	2	313-831	519
KT370724	A	A1	Canada	2	313-831	519
KT370725	A	A1	Canada	2	313-831	519
KT370726	A	A1	Canada	2	313-831	519
KT370727	A	A1	Canada	2	313-831	519
KT370728	A	A1	Canada	2	313-831	519
KT370729	A	A1	Canada	2	313-831	519
KT370730	A	A1	Canada	2	313-831	519
KT370731	A	A1	Canada	2	313-831	519
KT370732	A	A1	Canada	2	313-831	519
KT370733	A	A1	Canada	2	313-831	519
KT370734	A	A1	Canada	2	313-831	519
KT370735	A	A1	Canada	2	313-831	519
KT370736	A	A1	Canada	2	313-831	519
KT370737	A	A1	Canada	2	313-831	519
KT370738	A	A1	Canada	2	313-831	519
KT370739	A	A1	Canada	2	313-831	519
KT370740	A	A1	Canada	2	313-831	519
KT370741	A	A1	Canada	2	313-831	519
KT370742	A	A1	Canada	2	313-831	519
KT370743	A	A1	Canada	2	313-831	519
KT370744	A	A1	Canada	2	313-831	519
KT370745	A	A2	Canada	2	313-831	519
KT370746	A	A2	Canada	2	313-831	519
KT370747	A	A2	Canada	2	313-831	519
KT370748	A	A1	Canada	2	313-831	519
KT370749	A	A5	Canada	2	313-831	519

KT370750	A	A4	Canada	2	313-831	519
KT370751	A	A4	Canada	2	313-831	519
KT370752	A	A1	Canada	2	313-831	519
KT370753	A	A2	Canada	2	313-831	519
KT370754	A	A2	Canada	2	313-831	519
KT370755	A	A2	Canada	2	313-831	519
KT370756	A	A2	Canada	2	313-831	519
KT370757	A	A2	Canada	2	313-831	519
KT370758	A	A2	Canada	2	313-831	519
KT370759	A	A2	Canada	2	313-831	519
KT370760	A	A2	Canada	2	313-831	519
KT370761	A	A2	Canada	2	313-831	519
KT370762	A	A2	Canada	2	313-831	519
KT370763	A	A2	Canada	2	313-831	519
KT370764	A	A2	Canada	2	313-831	519
KT370765	A	A2	Canada	2	313-831	519
KT370766	A	A2	Canada	2	313-831	519
KT370767	A	A2	Canada	2	313-831	519
KT370768	A	A2	Canada	2	313-831	519
KT370769	A	A2	Canada	2	313-831	519
KT370770	A	A2	Canada	2	313-831	519
KT370771	A	A2	Canada	2	313-831	519
KT370772	A	A2	Canada	2	313-831	519
KT370773	A	A2	Canada	2	313-831	519
KT370774	A	A2	Canada	2	313-831	519
KT370775	A	A2	Canada	2	313-831	519
KT370776	A	A2	Canada	2	313-831	519
KT370777	A	A2	Canada	2	313-831	519
KT370778	A	A2	Canada	2	313-831	519
KT370779	A	A2	Canada	2	313-831	519
KT370785	A	A1	Canada	2	313-831	519

[1]Alignment to complete genome reference sequence VHB NC_003977.2

COLOMBIA

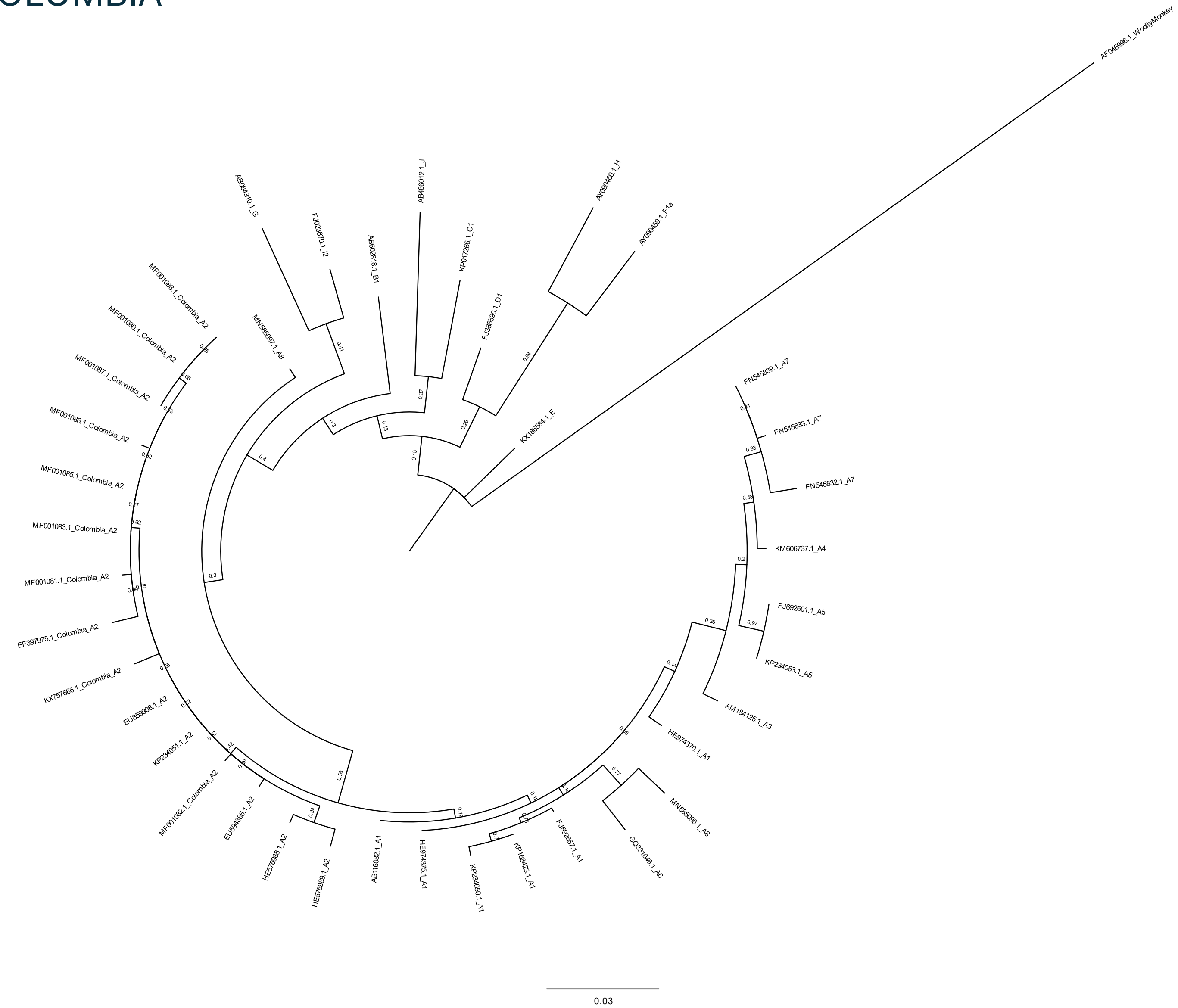

Tree 1. The evolutionary history was inferred by using the Maximum Likelihood method and Tamura-Nei model. The percentage of replicate trees in which the associated taxa clustered together in the bootstrap test (1000 replicates) are shown next to the branches. Initial tree(s) for the heuristic search were obtained automatically by applying Neighbor-Join and BioNJ algorithms to a matrix of pairwise distances estimated using the Tamura-Nei model, and then selecting the topology with superior log likelihood value. A discrete Gamma distribution was used to model evolutionary rate differences among sites (5 categories (+G, parameter = 0.1370)). The tree is drawn to scale, with branch lengths measured in the number of substitutions per site. The analysis involved 41 nucleotide sequences, of which 31 were used as marker sequences to determine the genotype of 10 sequences. All positions containing gaps and missing data were eliminated. There was a total of 507 positions in the final dataset. Evolutionary analyses were conducted in MEGA X.

ID	GENOTYPE	SUBTYPE	COUNTRY	TREE	ALIGNMENT[1]	BASE PAIRS
KX757666	A	A2	Colombia	1	107-1828	1721
MF001080	A	A2	Colombia	1	224-768	545
MF001081	A	A2	Colombia	1	224-768	545
MF001082	A	A2	Colombia	1	227-768	542

MF001083	A	A2	Colombia	1	224-768	545
MF001085	A	A2	Colombia	1	224-768	545
MF001086	A	A2	Colombia	1	224-768	545
MF001087	A	A2	Colombia	1	224-768	545
MF001088	A	A2	Colombia	1	224-768	545
EF397975	A	A2	Colombia	1	157-753	597

[1]Alignment to complete genome reference sequence VHB NC_003977.2

COSTA RICA

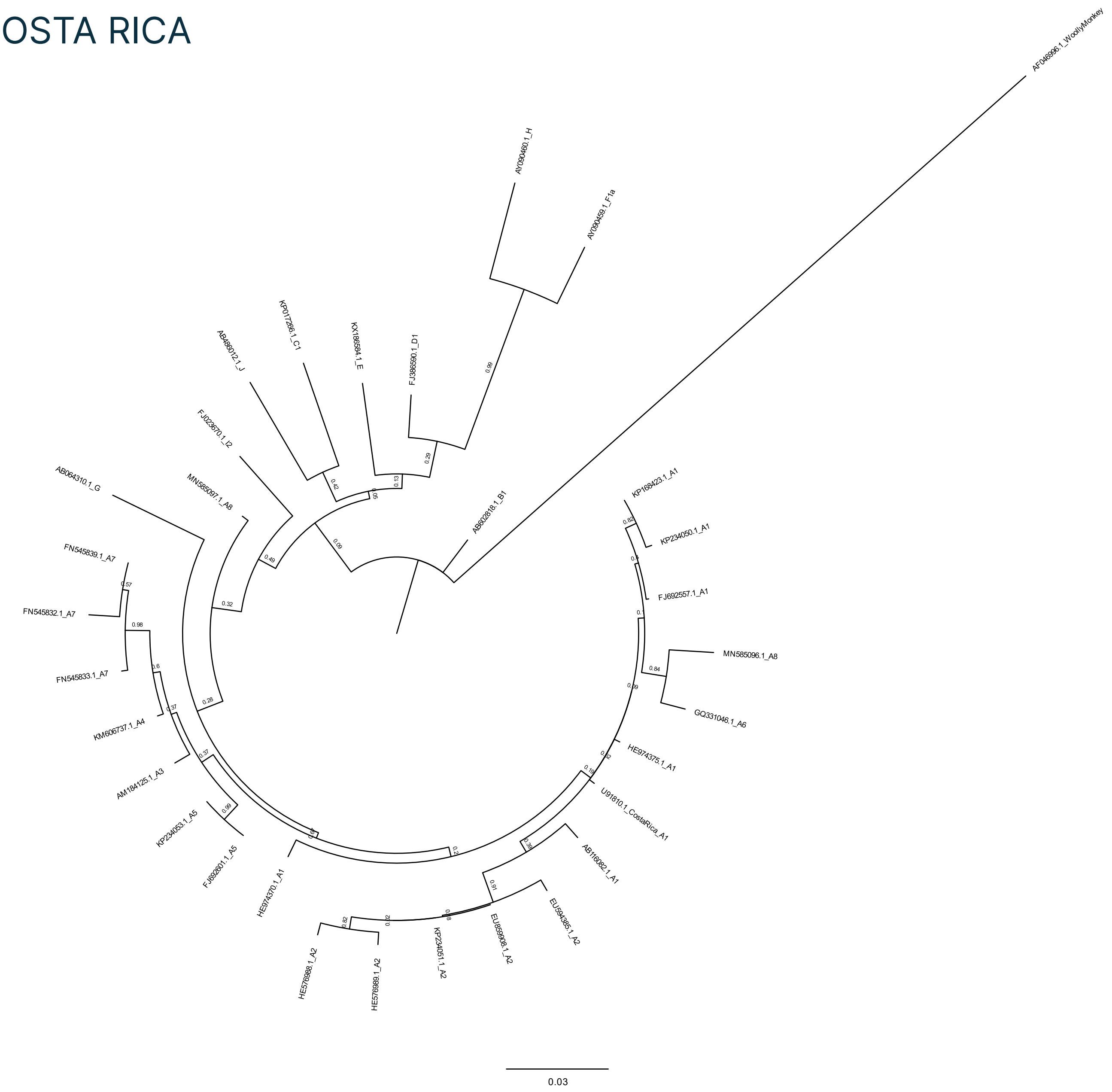

Tree 1. The evolutionary history was inferred by using the Maximum Likelihood method and Tamura-Nei model. The percentage of replicate trees in which the associated taxa clustered together in the bootstrap test (1000 replicates) are shown next to the branches. Initial tree(s) for the heuristic search were obtained automatically by applying Neighbor-Join and BioNJ algorithms to a matrix of pairwise distances estimated using the Tamura-Nei model, and then selecting the topology with superior log likelihood value. A discrete Gamma distribution was used to model evolutionary rate differences among sites (5 categories (+G, parameter = 0.1478)). The tree is drawn to scale, with branch lengths measured in the number of substitutions per site. The analysis involved 32 nucleotide sequences, of which 31 were used as marker sequences to determine the genotype of 1 sequence. All positions containing gaps and missing data were eliminated. There was a total of 668 positions in the final dataset. Evolutionary analyses were conducted in MEGA X.

ID	GENOTYPE	SUBTYPE	COUNTRY	TREE	ALIGNMENT[1]	BASE PAIRS
U91810	A	A1	Costa Rica	1	157-837	681

[1]Alignment to complete genome reference sequence VHB NC_003977.2

ECUADOR

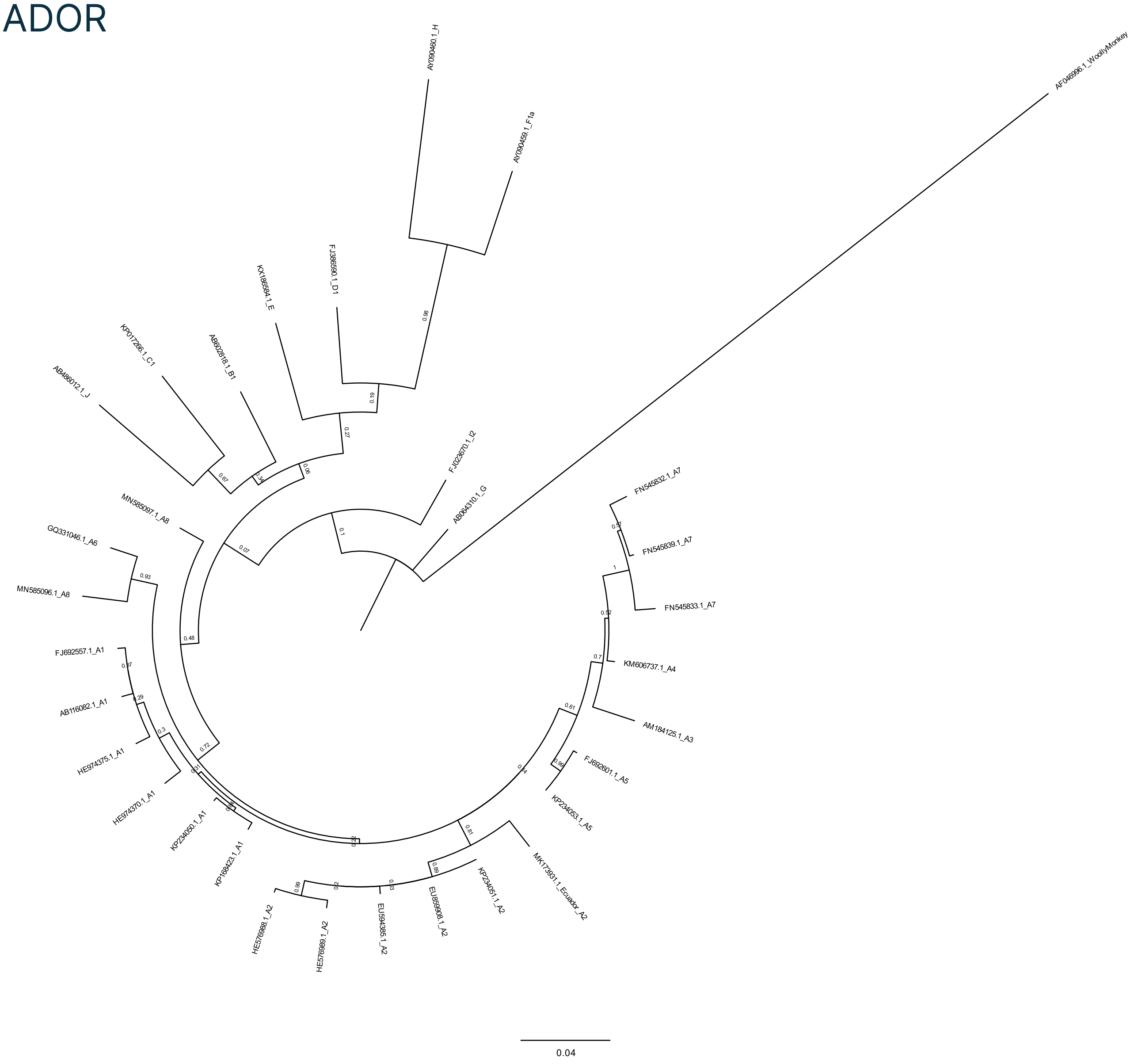

Tree 1. The evolutionary history was inferred by using the Maximum Likelihood method and Tamura-Nei model. The percentage of replicate trees in which the associated taxa clustered together in the bootstrap test (1000 replicates) are shown next to the branches. Initial tree(s) for the heuristic search were obtained automatically by applying Neighbor-Join and BioNJ algorithms to a matrix of pairwise distances estimated using the Tamura-Nei model, and then selecting the topology with superior log likelihood value. A discrete Gamma distribution was used to model evolutionary rate differences among sites (5 categories (+G, parameter = 0.1577)). The tree is drawn to scale, with branch lengths measured in the number of substitutions per site. The analysis involved 32 nucleotide sequences, of which 31 were used as marker sequences to determine the genotype of 1 sequence. All positions containing gaps and missing data were eliminated. There was a total of 726 positions in the final dataset. Evolutionary analyses were conducted in MEGA X.

ID	GENOTYPE	SUBTYPE	COUNTRY	TREE	ALIGNMENT[1]	BASE PAIRS
MK173931	A	A2	Ecuador	1	216-955	740

[1]Alignment to complete genome reference sequence VHB NC_003977.2

MEXICO

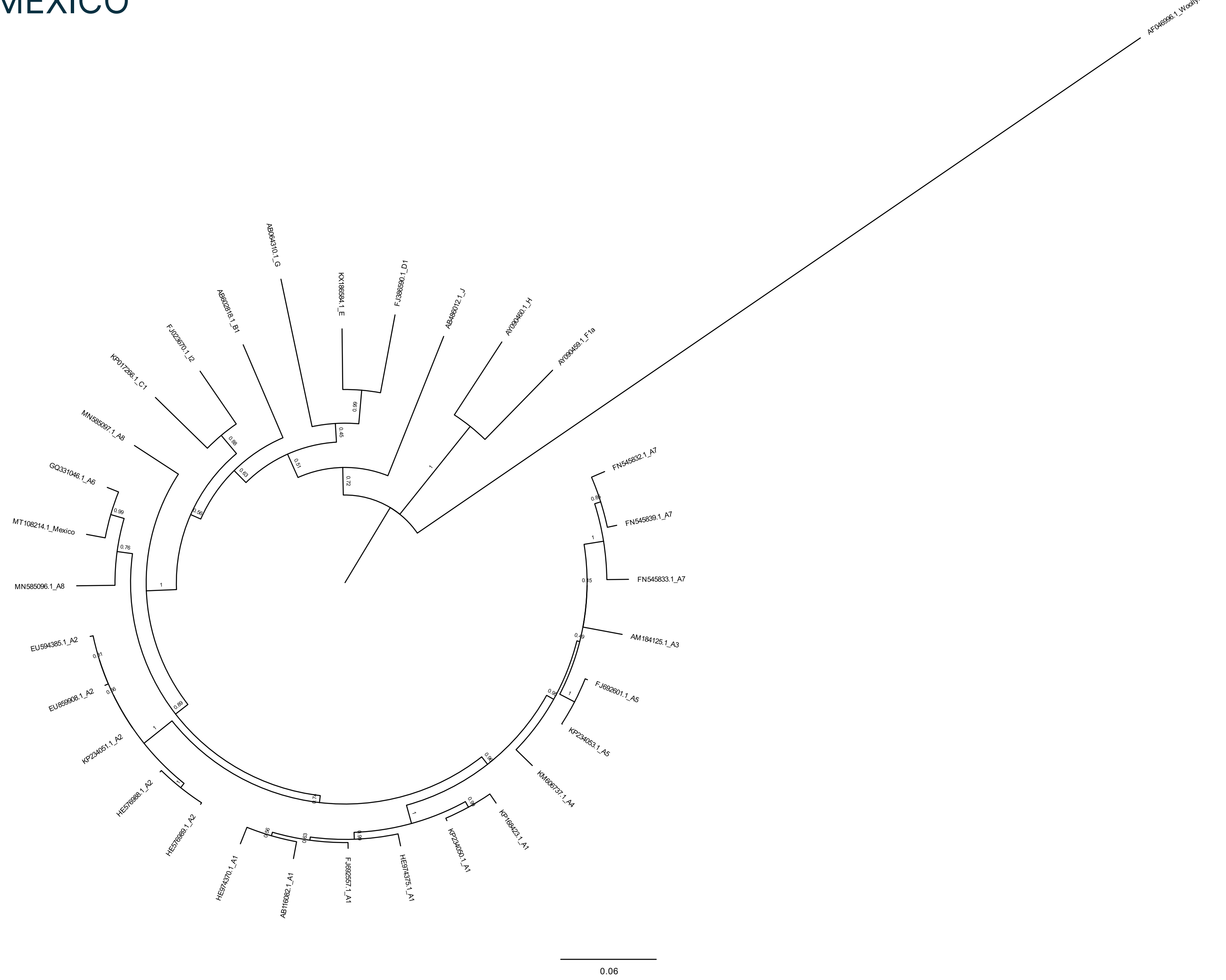

Tree 1. The evolutionary history was inferred by using the Maximum Likelihood method and Tamura-Nei model. The percentage of replicate trees in which the associated taxa clustered together in the bootstrap test (1000 replicates) are shown next to the branches. Initial tree(s) for the heuristic search were obtained automatically by applying Neighbor-Join and BioNJ algorithms to a matrix of pairwise distances estimated using the Tamura-Nei model, and then selecting the topology with superior log likelihood value. A discrete Gamma distribution was used to model evolutionary rate differences among sites (5 categories (+G, parameter = 0.2155)). The tree is drawn to scale, with branch lengths measured in the number of substitutions per site. The analysis involved 32 nucleotide sequences, of which 31 were used as marker sequences to determine the genotype of 1 sequence. All positions containing gaps and missing data were eliminated. There was a total of 2870 positions in the final dataset. Evolutionary analyses were conducted in MEGA X.

ID	GENOTYPE	SUBTYPE	COUNTRY	TREE	ALIGNMENT[1]	BASE PAIRS
MT108214	A	A6	Mexico	1	1-3182	3222

[1]Alignment to complete genome reference sequence VHB NC_003977.2

NICARAGUA

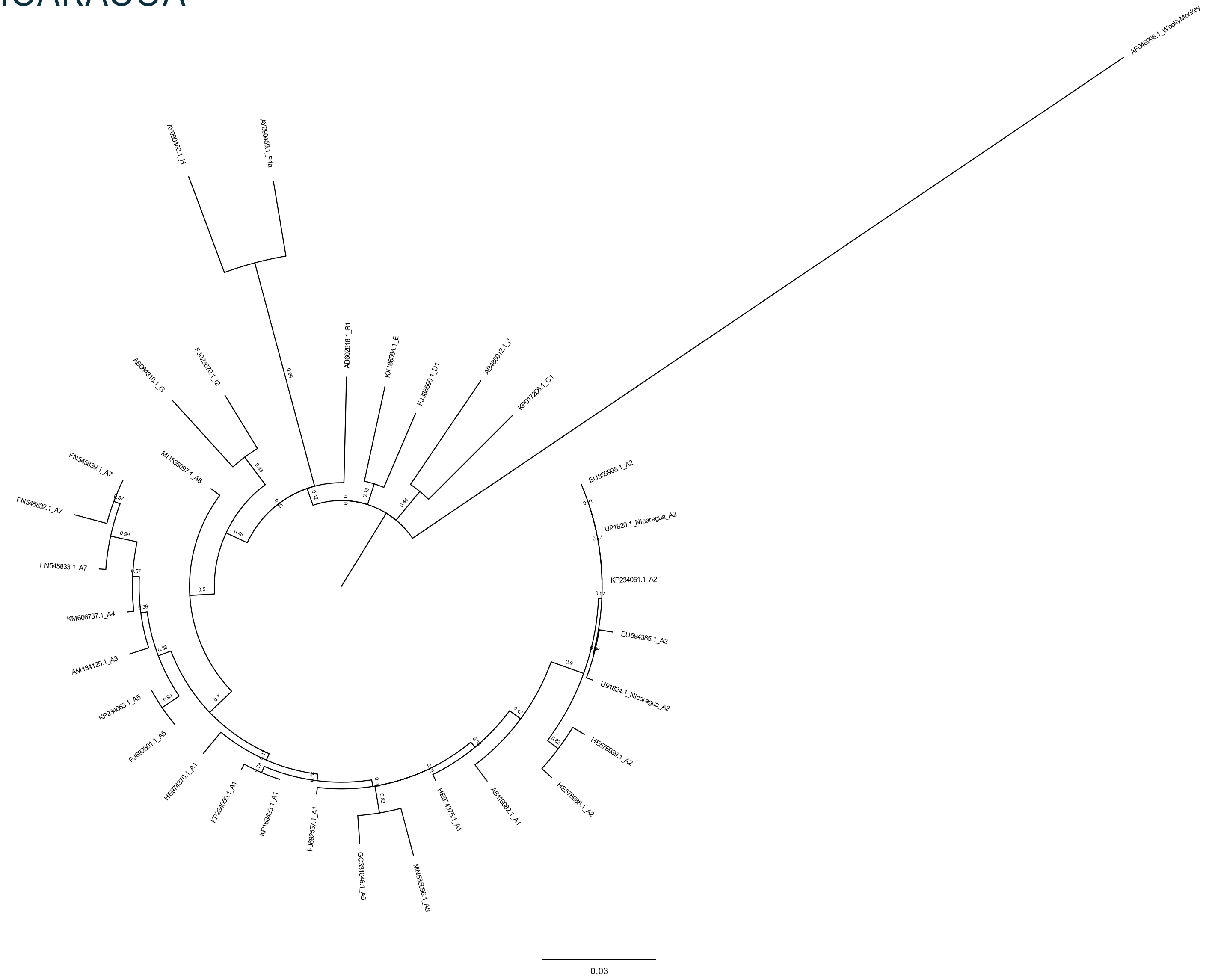

Tree 1. The evolutionary history was inferred by using the Maximum Likelihood method and Tamura-Nei model. The percentage of replicate trees in which the associated taxa clustered together in the bootstrap test (1000 replicates) are shown next to the branches. Initial tree(s) for the heuristic search were obtained automatically by applying Neighbor-Join and BioNJ algorithms to a matrix of pairwise distances estimated using the Tamura-Nei model, and then selecting the topology with superior log likelihood value. A discrete Gamma distribution was used to model evolutionary rate differences among sites (5 categories (+G, parameter = 0.1481)). The tree is drawn to scale, with branch lengths measured in the number of substitutions per site. The analysis involved 33 nucleotide sequences, of which 31 were used as marker sequences to determine the genotype of 2 sequences. All positions containing gaps and missing data were eliminated. There was a total of 668 positions in the final dataset. Evolutionary analyses were conducted in MEGA X.

ID	GENOTYPE	SUBTYPE	COUNTRY	TREE	ALIGNMENT[1]	BASE PAIRS
U91820	A	A2	Nicaragua	1	157-837	681
U91824	A	A2	Nicaragua	1	157-837	681

[1]Alignment to complete genome reference sequence VHB NC_003977.2

UNITED STATES OF AMERICA

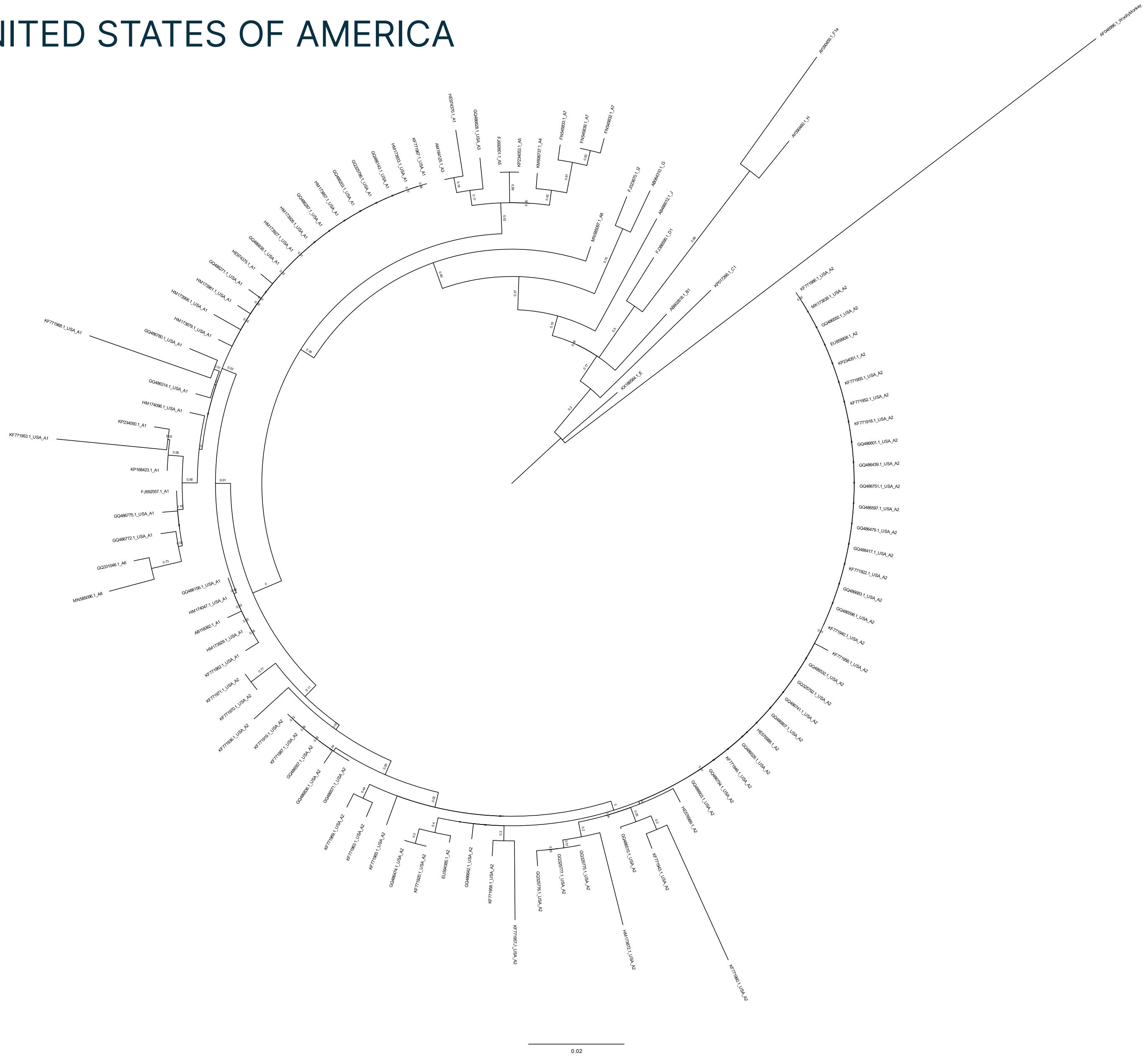

Tree 1. The evolutionary history was inferred by using the Maximum Likelihood method and Tamura-Nei model. The percentage of replicate trees in which the associated taxa clustered together in the bootstrap test (1000 replicates) are shown next to the branches. Initial tree(s) for the heuristic search were obtained automatically by applying Neighbor-Join and BioNJ algorithms to a matrix of pairwise distances estimated using the Tamura-Nei model, and then selecting the topology with superior log likelihood value. A discrete Gamma distribution was used to model evolutionary rate differences among sites (5 categories (+G, parameter = 0.2219)). The tree is drawn to scale, with branch lengths measured in the number of substitutions per site. The analysis involved 105 nucleotide sequences, of which 31 were used as marker sequences to determine the genotype of 74 sequences. All positions containing gaps and missing data were eliminated. There was a total of 358 positions in the final dataset. Evolutionary analyses were conducted in MEGA X.

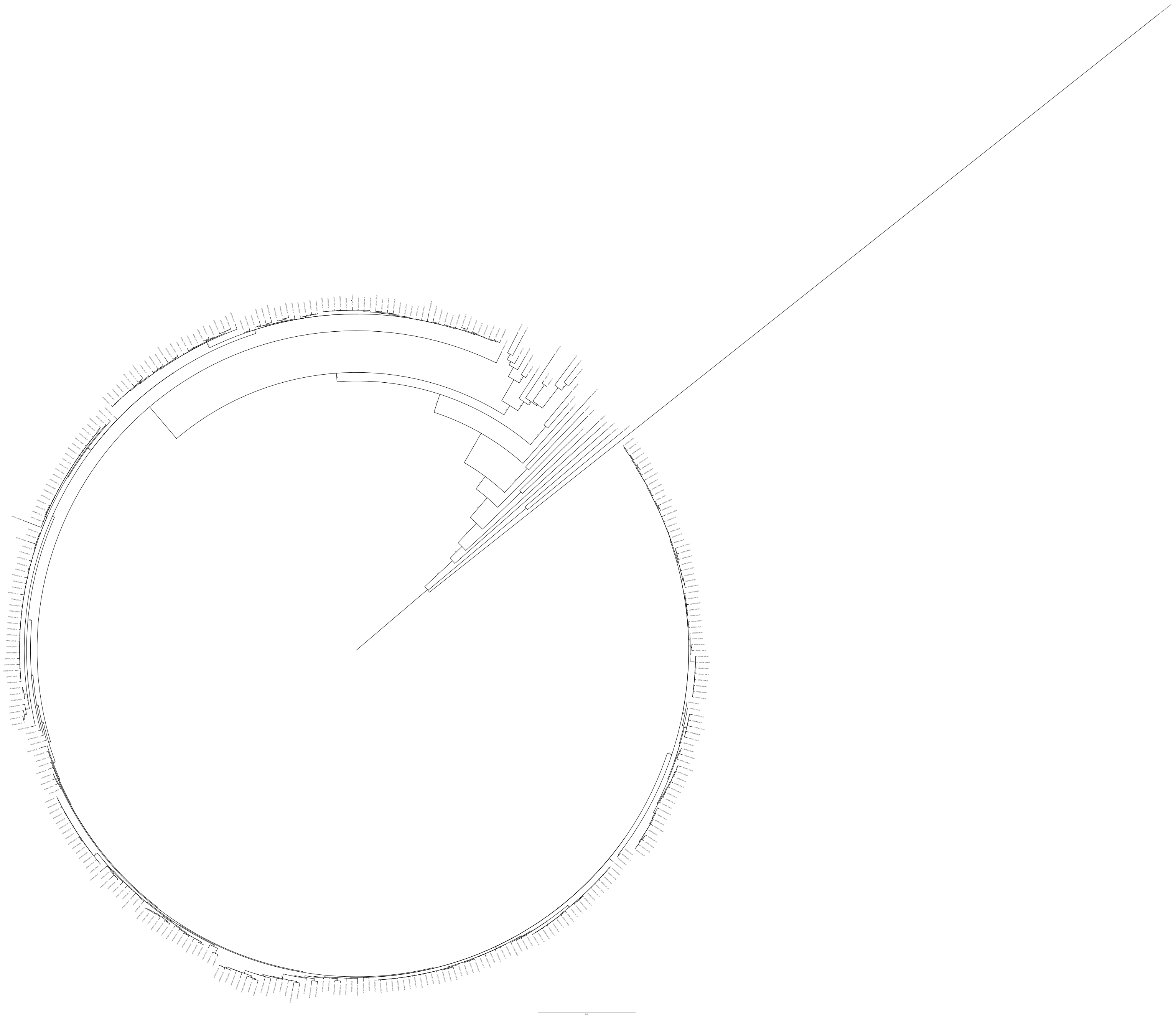

Tree 2. The evolutionary history was inferred by using the Maximum Likelihood method and Tamura-Nei model. The percentage of replicate trees in which the associated taxa clustered together in the bootstrap test (1000 replicates) are shown next to the branches. Initial tree(s) for the heuristic search were obtained automatically by applying Neighbor-Join and BioNJ algorithms to a matrix of pairwise distances estimated using the Tamura-Nei model, and then selecting the topology with superior log likelihood value. A discrete Gamma distribution was used to model evolutionary rate differences among sites (5 categories (+G, parameter = 0.2988)). The tree is drawn to scale, with branch lengths measured in the number of substitutions per site. The analysis involved 365 nucleotide sequences, of which 31 were used as marker sequences to determine the genotype of 334 sequences. All positions containing gaps and missing data were eliminated. There was a total of 2967 positions in the final dataset. Evolutionary analyses were conducted in MEGA X.

ID	GENOTYPE	SUBTYPE	COUNTRY	TREE	ALIGNMENT[1]	BASE PAIRS
GQ325775	A	A2	USA	1	132-1163	1032

GQ325776	A	A2	USA	1	132-1163	1032
GQ325777	A	A2	USA	1	132-1163	1032
GQ325786	A	A1	USA	1	132-1163	1032
GQ325792	A	A2	USA	1	132-1163	1032
GQ486143	A	A1	USA	1	132-1163	1032
GQ486156	A	A1	USA	1	132-1163	1032
GQ486203	A	A1	USA	1	132-1163	1032
GQ486271	A	A1	USA	1	132-1163	1032
GQ486287	A	A1	USA	1	132-1163	1032
GQ486314	A	A1	USA	1	132-1163	1032
GQ486417	A	A2	USA	1	132-1163	1032
GQ486439	A	A2	USA	1	132-1163	1032
GQ486474	A	A2	USA	1	132-1163	1032
GQ486479	A	A2	USA	1	132-1163	1032
GQ486510	A	A2	USA	1	132-1163	1032
GQ486529	A	A2	USA	1	132-1163	1032
GQ486532	A	A2	USA	1	132-1163	1032
GQ486550	A	A2	USA	1	132-1163	1032
GQ486557	A	A2	USA	1	132-1163	1032
GQ486571	A	A2	USA	1	132-1163	1032
GQ486597	A	A2	USA	1	132-1163	1032
GQ486598	A	A2	USA	1	132-1163	1032
GQ486601	A	A2	USA	1	132-1163	1032
GQ486603	A	A2	USA	1	132-1163	1032
GQ486642	A	A2	USA	1	132-1163	1032
GQ486683	A	A2	USA	1	132-1163	1032
GQ486741	A	A2	USA	1	132-1163	1032
GQ486751	A	A2	USA	1	132-1163	1032
GQ486754	A	A2	USA	1	132-1163	1032
GQ486772	A	A1	USA	1	132-1163	1032
GQ486775	A	A1	USA	1	132-1163	1032
GQ486780	A	A1	USA	1	132-1163	1032
GQ486807	A	A2	USA	1	132-1163	1032
GQ486828	A	A3	USA	1	132-1163	1032
GQ486836	A	A2	USA	1	132-1163	1032
GQ486838	A	A1	USA	1	132-1163	1032
HM173857	A	A1	USA	1	417-881	465
HM173872	A	A2	USA	1	417-881	465
HM173879	A	A1	USA	1	417-881	465
HM173906	A	A1	USA	1	417-881	465
HM173926	A	A1	USA	1	417-881	465
HM173927	A	A1	USA	1	417-881	465
HM173929	A	A1	USA	1	417-881	465
HM173933	A	A1	USA	1	417-881	465
HM173961	A	A1	USA	1	417-881	465
HM174047	A	A1	USA	1	417-881	465

HM174096	A	A1	USA	1	417-881	465
KF771918	A	A2	USA	1	157-837	681
KF771919	A	A2	USA	1	157-837	681
KF771920	A	A2	USA	1	157-837	681
KF771922	A	A2	USA	1	157-837	681
KF771936	A	A2	USA	1	157-837	681
KF771940	A	A2	USA	1	157-837	681
KF771943	A	A2	USA	1	157-837	681
KF771946	A	A2	USA	1	157-837	681
KF771952	A	A2	USA	1	157-837	681
KF771953	A	A1	USA	1	157-837	681
KF771955	A	A2	USA	1	157-837	681
KF771956	A	A2	USA	1	157-837	681
KF771957	A	A2	USA	1	157-837	681
KF771958	A	A2	USA	1	157-837	681
KF771960	A	A2	USA	1	157-837	681
KF771962	A	A1	USA	1	157-837	681
KF771963	A	A2	USA	1	157-837	681
KF771965	A	A2	USA	1	157-837	681
KF771967	A	A1	USA	1	157-837	681
KF771968	A	A1	USA	1	157-837	681
KF771969	A	A2	USA	1	157-837	681
KF771970	A	A2	USA	1	1-3182	1203
KF771971	A	A2	USA	1	1-3182	1020
KF771986	A	A2	USA	1	1-3182	1200
KF771987	A	A2	USA	1	1-3182	1200
MK173838	A	A2	USA	1	216-955	740
AB116076	A	A2	USA	2	Complete Genome	3221
AB116077	A	A2	USA	2	Complete Genome	3221
AB116078	A	A2	USA	2	Complete Genome	3221
AY902775	A	A2	USA	2	Complete Genome	3221
JQ707299	A	A2	USA	2	Complete Genome	3221
JQ707300	A	A2	USA	2	Complete Genome	3221
JQ707301	A	A2	USA	2	Complete Genome	3221
JQ707302	A	A2	USA	2	Complete Genome	3221
JQ707303	A	A2	USA	2	Complete Genome	3221
JQ707304	A	A2	USA	2	Complete Genome	3221
JQ707305	A	A2	USA	2	Complete Genome	3221
JQ707306	A	A2	USA	2	Complete Genome	3221
JQ707307	A	A2	USA	2	Complete Genome	3221
JQ707308	A	A2	USA	2	Complete Genome	3221
JQ707309	A	A2	USA	2	Complete Genome	3221
JQ707310	A	A2	USA	2	Complete Genome	3221
JQ707311	A	A2	USA	2	Complete Genome	3221
JQ707312	A	A2	USA	2	Complete Genome	3221
JQ707313	A	A2	USA	2	Complete Genome	3221

JQ707314	A	A2	USA	2	Complete Genome	3221
JQ707315	A	A2	USA	2	Complete Genome	3221
JQ707316	A	A2	USA	2	Complete Genome	3221
JQ707317	A	A2	USA	2	Complete Genome	3221
JQ707318	A	A2	USA	2	Complete Genome	3221
JQ707319	A	A2	USA	2	Complete Genome	3221
JQ707320	A	A2	USA	2	Complete Genome	3221
JQ707321	A	A2	USA	2	Complete Genome	3221
JQ707322	A	A2	USA	2	Complete Genome	3221
JQ707323	A	A2	USA	2	Complete Genome	3221
JQ707324	A	A2	USA	2	Complete Genome	3221
JQ707325	A	A2	USA	2	Complete Genome	3221
JQ707326	A	A2	USA	2	Complete Genome	3221
JQ707327	A	A2	USA	2	Complete Genome	3221
JQ707328	A	A2	USA	2	Complete Genome	3221
JQ707329	A	A2	USA	2	Complete Genome	3221
JQ707330	A	A2	USA	2	Complete Genome	3221
JQ707331	A	A2	USA	2	Complete Genome	3221
JQ707332	A	A2	USA	2	Complete Genome	3221
JQ707333	A	A2	USA	2	Complete Genome	3221
JQ707334	A	A2	USA	2	Complete Genome	3221
JQ707335	A	A2	USA	2	Complete Genome	3221
JQ707336	A	A2	USA	2	Complete Genome	3221
JQ707337	A	A2	USA	2	Complete Genome	3221
JQ707338	A	A2	USA	2	Complete Genome	3221
JQ707339	A	A2	USA	2	Complete Genome	3221
JQ707340	A	A2	USA	2	Complete Genome	3221
JQ707341	A	A2	USA	2	Complete Genome	3221
JQ707342	A	A2	USA	2	Complete Genome	3221
JQ707343	A	A2	USA	2	Complete Genome	3221
JQ707344	A	A2	USA	2	Complete Genome	3221
JQ707345	A	A2	USA	2	Complete Genome	3221
JQ707346	A	A2	USA	2	Complete Genome	3221
JQ707347	A	A2	USA	2	Complete Genome	3221
JQ707348	A	A2	USA	2	Complete Genome	3221
JQ707349	A	A2	USA	2	Complete Genome	3221
JQ707350	A	A2	USA	2	Complete Genome	3221
JQ707351	A	A2	USA	2	Complete Genome	3221
JQ707352	A	A2	USA	2	Complete Genome	3221
JQ707353	A	A2	USA	2	Complete Genome	3221
JQ707354	A	A2	USA	2	Complete Genome	3221
JQ707355	A	A2	USA	2	Complete Genome	3221
JQ707356	A	A2	USA	2	Complete Genome	3221
JQ707357	A	A2	USA	2	Complete Genome	3221
JQ707358	A	A2	USA	2	Complete Genome	3221
JQ707359	A	A2	USA	2	Complete Genome	3221

JQ707360	A	A2	USA	2	Complete Genome	3221
JQ707361	A	A2	USA	2	Complete Genome	3221
JQ707362	A	A2	USA	2	Complete Genome	3221
JQ707363	A	A2	USA	2	Complete Genome	3221
JQ707364	A	A2	USA	2	Complete Genome	3221
JQ707365	A	A2	USA	2	Complete Genome	3221
JQ707366	A	A2	USA	2	Complete Genome	3221
JQ707367	A	A2	USA	2	Complete Genome	3221
JQ707368	A	A2	USA	2	Complete Genome	3221
JQ707369	A	A2	USA	2	Complete Genome	3221
JQ707370	A	A2	USA	2	Complete Genome	3221
JQ707371	A	A2	USA	2	Complete Genome	3221
JQ707372	A	A2	USA	2	Complete Genome	3221
JQ707373	A	A2	USA	2	Complete Genome	3221
JQ707374	A	A2	USA	2	Complete Genome	3221
JQ707375	A	A2	USA	2	Complete Genome	3221
JQ707376	A	A2	USA	2	Complete Genome	3221
JQ707377	A	A2	USA	2	Complete Genome	3221
JQ707378	A	A2	USA	2	Complete Genome	3221
JQ707379	A	A2	USA	2	Complete Genome	3221
JQ707380	A	A2	USA	2	Complete Genome	3221
JQ707381	A	A2	USA	2	Complete Genome	3221
JQ707382	A	A2	USA	2	Complete Genome	3221
JQ707383	A	A2	USA	2	Complete Genome	3221
JQ707384	A	A2	USA	2	Complete Genome	3221
JQ707385	A	A2	USA	2	Complete Genome	3221
JQ707386	A	A2	USA	2	Complete Genome	3221
JQ707387	A	A2	USA	2	Complete Genome	3221
JQ707388	A	A2	USA	2	Complete Genome	3221
JQ707389	A	A2	USA	2	Complete Genome	3221
JQ707390	A	A2	USA	2	Complete Genome	3221
JQ707391	A	A2	USA	2	Complete Genome	3221
JQ707392	A	A2	USA	2	Complete Genome	3221
JQ707393	A	A2	USA	2	Complete Genome	3221
JQ707394	A	A2	USA	2	Complete Genome	3221
JQ707395	A	A2	USA	2	Complete Genome	3221
JQ707396	A	A2	USA	2	Complete Genome	3221
JQ707397	A	A2	USA	2	Complete Genome	3221
JQ707398	A	A2	USA	2	Complete Genome	3221
JQ707399	A	A2	USA	2	Complete Genome	3221
JQ707400	A	A2	USA	2	Complete Genome	3221
JQ707401	A	A2	USA	2	Complete Genome	3221
JQ707402	A	A2	USA	2	Complete Genome	3221
JQ707403	A	A2	USA	2	Complete Genome	3221
JQ707404	A	A2	USA	2	Complete Genome	3221
JQ707405	A	A2	USA	2	Complete Genome	3221

JQ707406	A	A2	USA	2	Complete Genome	3221
JQ707407	A	A2	USA	2	Complete Genome	3221
JQ707408	A	A2	USA	2	Complete Genome	3221
JQ707409	A	A2	USA	2	Complete Genome	3221
JQ707410	A	A2	USA	2	Complete Genome	3221
JQ707411	A	A2	USA	2	Complete Genome	3221
JQ707412	A	A2	USA	2	Complete Genome	3221
JQ707413	A	A2	USA	2	Complete Genome	3221
JQ707414	A	A2	USA	2	Complete Genome	3221
JQ707415	A	A2	USA	2	Complete Genome	3221
JQ707416	A	A2	USA	2	Complete Genome	3221
JQ707417	A	A2	USA	2	Complete Genome	3221
JQ707418	A	A2	USA	2	Complete Genome	3221
JQ707419	A	A2	USA	2	Complete Genome	3221
JQ707420	A	A2	USA	2	Complete Genome	3221
JQ707531	A	A2	USA	2	Complete Genome	3221
JQ707532	A	A2	USA	2	Complete Genome	3221
JQ707533	A	A2	USA	2	Complete Genome	3221
JQ707534	A	A2	USA	2	Complete Genome	3221
JQ707535	A	A2	USA	2	Complete Genome	3221
JQ707536	A	A2	USA	2	Complete Genome	3221
JQ707537	A	A2	USA	2	Complete Genome	3221
JQ707538	A	A2	USA	2	Complete Genome	3221
JQ707539	A	A2	USA	2	Complete Genome	3221
JQ707540	A	A2	USA	2	Complete Genome	3221
JQ707541	A	A2	USA	2	Complete Genome	3221
JQ707542	A	A2	USA	2	Complete Genome	3221
JQ707543	A	A2	USA	2	Complete Genome	3221
JQ707544	A	A2	USA	2	Complete Genome	3221
JQ707545	A	A2	USA	2	Complete Genome	3221
JQ707546	A	A2	USA	2	Complete Genome	3221
JQ707547	A	A2	USA	2	Complete Genome	3221
JQ707548	A	A2	USA	2	Complete Genome	3221
JQ707549	A	A2	USA	2	Complete Genome	3221
JQ707550	A	A2	USA	2	Complete Genome	3221
JQ707551	A	A2	USA	2	Complete Genome	3221
JQ707552	A	A2	USA	2	Complete Genome	3221
JQ707553	A	A2	USA	2	Complete Genome	3221
JQ707554	A	A2	USA	2	Complete Genome	3221
JQ707555	A	A2	USA	2	Complete Genome	3221
JQ707556	A	A2	USA	2	Complete Genome	3221
JQ707557	A	A2	USA	2	Complete Genome	3221
JQ707558	A	A2	USA	2	Complete Genome	3221
JQ707559	A	A2	USA	2	Complete Genome	3221
JQ707560	A	A2	USA	2	Complete Genome	3221
JQ707561	A	A2	USA	2	Complete Genome	3221

JQ707562	A	A2	USA	2	Complete Genome	3221
JQ707563	A	A2	USA	2	Complete Genome	3221
JQ707564	A	A2	USA	2	Complete Genome	3221
JQ707565	A	A2	USA	2	Complete Genome	3221
JQ707566	A	A2	USA	2	Complete Genome	3221
JQ707567	A	A2	USA	2	Complete Genome	3221
JQ707568	A	A2	USA	2	Complete Genome	3221
JQ707569	A	A2	USA	2	Complete Genome	3221
JQ707570	A	A2	USA	2	Complete Genome	3221
JQ707571	A	A2	USA	2	Complete Genome	3221
JQ707572	A	A2	USA	2	Complete Genome	3221
JQ707573	A	A2	USA	2	Complete Genome	3221
JQ707574	A	A2	USA	2	Complete Genome	3221
JQ707575	A	A2	USA	2	Complete Genome	3221
JQ707576	A	A2	USA	2	Complete Genome	3221
JQ707577	A	A2	USA	2	Complete Genome	3221
JQ707578	A	A2	USA	2	Complete Genome	3221
JQ707579	A	A2	USA	2	Complete Genome	3221
JQ707580	A	A2	USA	2	Complete Genome	3221
JQ707581	A	A2	USA	2	Complete Genome	3221
JQ707582	A	A2	USA	2	Complete Genome	3221
JQ707583	A	A2	USA	2	Complete Genome	3221
JQ707584	A	A2	USA	2	Complete Genome	3221
JQ707585	A	A2	USA	2	Complete Genome	3221
JQ707586	A	A2	USA	2	Complete Genome	3221
JQ707587	A	A2	USA	2	Complete Genome	3221
JQ707588	A	A2	USA	2	Complete Genome	3221
JQ707589	A	A2	USA	2	Complete Genome	3221
JQ707590	A	A2	USA	2	Complete Genome	3221
JQ707591	A	A2	USA	2	Complete Genome	3221
JQ707592	A	A2	USA	2	Complete Genome	3221
JQ707593	A	A2	USA	2	Complete Genome	3221
JQ707594	A	A2	USA	2	Complete Genome	3221
JQ707595	A	A2	USA	2	Complete Genome	3221
JQ707596	A	A2	USA	2	Complete Genome	3221
JQ707597	A	A2	USA	2	Complete Genome	3221
JQ707598	A	A2	USA	2	Complete Genome	3221
JQ707599	A	A2	USA	2	Complete Genome	3221
JQ707600	A	A2	USA	2	Complete Genome	3221
JQ707601	A	A2	USA	2	Complete Genome	3221
JQ707602	A	A2	USA	2	Complete Genome	3221
JQ707603	A	A2	USA	2	Complete Genome	3221
JQ707604	A	A2	USA	2	Complete Genome	3221
JQ707605	A	A2	USA	2	Complete Genome	3221
JQ707606	A	A2	USA	2	Complete Genome	3221
JQ707607	A	A2	USA	2	Complete Genome	3221

JQ707608	A	A2	USA	2	Complete Genome	3221
JQ707609	A	A2	USA	2	Complete Genome	3221
JQ707610	A	A2	USA	2	Complete Genome	3221
JQ707611	A	A2	USA	2	Complete Genome	3221
JQ707612	A	A2	USA	2	Complete Genome	3221
JQ707613	A	A2	USA	2	Complete Genome	3221
JQ707614	A	A2	USA	2	Complete Genome	3221
JQ707615	A	A2	USA	2	Complete Genome	3221
JQ707616	A	A2	USA	2	Complete Genome	3221
JQ707617	A	A2	USA	2	Complete Genome	3221
JQ707618	A	A2	USA	2	Complete Genome	3221
JQ707619	A	A2	USA	2	Complete Genome	3221
JQ707620	A	A2	USA	2	Complete Genome	3221
JQ707621	A	A2	USA	2	Complete Genome	3221
JQ707622	A	A2	USA	2	Complete Genome	3221
JQ707623	A	A2	USA	2	Complete Genome	3221
JQ707624	A	A2	USA	2	Complete Genome	3221
JQ707625	A	A2	USA	2	Complete Genome	3221
JQ707626	A	A2	USA	2	Complete Genome	3221
JQ707627	A	A2	USA	2	Complete Genome	3221
JQ707628	A	A2	USA	2	Complete Genome	3221
JQ707629	A	A2	USA	2	Complete Genome	3221
JQ707630	A	A2	USA	2	Complete Genome	3221
JQ707631	A	A2	USA	2	Complete Genome	3221
JQ707632	A	A2	USA	2	Complete Genome	3221
JQ707633	A	A2	USA	2	Complete Genome	3221
JQ707634	A	A2	USA	2	Complete Genome	3221
JQ707635	A	A2	USA	2	Complete Genome	3221
JQ707636	A	A2	USA	2	Complete Genome	3221
JQ707637	A	A2	USA	2	Complete Genome	3221
JQ707638	A	A2	USA	2	Complete Genome	3221
JQ707639	A	A2	USA	2	Complete Genome	3221
JQ707640	A	A2	USA	2	Complete Genome	3221
JQ707641	A	A2	USA	2	Complete Genome	3221
JQ707642	A	A2	USA	2	Complete Genome	3221
JQ707643	A	A2	USA	2	Complete Genome	3221
JQ707644	A	A2	USA	2	Complete Genome	3221
JQ707645	A	A2	USA	2	Complete Genome	3221
JQ707646	A	A2	USA	2	Complete Genome	3221
JQ707647	A	A2	USA	2	Complete Genome	3221
JQ707648	A	A2	USA	2	Complete Genome	3221
JQ707649	A	A2	USA	2	Complete Genome	3221
JQ707650	A	A2	USA	2	Complete Genome	3221
JQ707651	A	A2	USA	2	Complete Genome	3221
JQ707652	A	A2	USA	2	Complete Genome	3221
JQ707653	A	A2	USA	2	Complete Genome	3221

JQ707654	A	A2	USA	2	Complete Genome	3221
JQ707655	A	A2	USA	2	Complete Genome	3221
JQ707656	A	A2	USA	2	Complete Genome	3221
JQ707658	A	A2	USA	2	Complete Genome	3221
JQ707659	A	A2	USA	2	Complete Genome	3221
JQ707661	A	A2	USA	2	Complete Genome	3221
JQ707662	A	A2	USA	2	Complete Genome	3221
JQ707663	A	A2	USA	2	Complete Genome	3221
JQ707664	A	A2	USA	2	Complete Genome	3221
JQ707665	A	A2	USA	2	Complete Genome	3221
JQ707667	A	A2	USA	2	Complete Genome	3221
JQ707669	A	A2	USA	2	Complete Genome	3221
JQ707670	A	A2	USA	2	Complete Genome	3221
JQ707672	A	A2	USA	2	Complete Genome	3221
JQ707674	A	A2	USA	2	Complete Genome	3221
JQ707675	A	A2	USA	2	Complete Genome	3221
JQ707676	A	A2	USA	2	Complete Genome	3221
JQ707681	A	A2	USA	2	Complete Genome	3221
KF779227	A	A2	USA	2	Complete Genome	3165
KF779231	A	A2	USA	2	Complete Genome	3165
KF779232	A	A2	USA	2	Complete Genome	3165
KF779234	A	A2	USA	2	Complete Genome	3165
KF779238	A	A2	USA	2	Complete Genome	3165
KF779239	A	A2	USA	2	Complete Genome	3165
KF779240	A	A2	USA	2	Complete Genome	3165
KF779243	A	A2	USA	2	Complete Genome	3165
KF779244	A	A2	USA	2	Complete Genome	3165
KF779245	A	A2	USA	2	Complete Genome	3165
KF779246	A	A2	USA	2	Complete Genome	3165
KF779247	A	A2	USA	2	Complete Genome	3165
KF779248	A	A2	USA	2	Complete Genome	3165
KF779249	A	A2	USA	2	Complete Genome	3165
KF779269	A	A2	USA	2	Complete Genome	3165
KF779270	A	A2	USA	2	Complete Genome	3165
KF779271	A	A2	USA	2	Complete Genome	3165
KF779272	A	A2	USA	2	Complete Genome	3165
KF779273	A	A2	USA	2	Complete Genome	3165
KF779274	A	A2	USA	2	Complete Genome	3165
KF779275	A	A2	USA	2	Complete Genome	3165
KF779276	A	A2	USA	2	Complete Genome	3165
KF779277	A	A2	USA	2	Complete Genome	3165
KF779278	A	A2	USA	2	Complete Genome	3165
KF779279	A	A2	USA	2	Complete Genome	3165
KF779280	A	A2	USA	2	Complete Genome	3165
KF779281	A	A2	USA	2	Complete Genome	3165
KF779282	A	A2	USA	2	Complete Genome	3165

KF779283	A	A2	USA	2	Complete Genome	3165
KF779305	A	A2	USA	2	Complete Genome	3165
KF779306	A	A2	USA	2	Complete Genome	3165
KF779307	A	A2	USA	2	Complete Genome	3165
KF779308	A	A2	USA	2	Complete Genome	3165
KF779309	A	A2	USA	2	Complete Genome	3165
KF779344	A	A2	USA	2	Complete Genome	3165
KF779345	A	A2	USA	2	Complete Genome	3165
KF779346	A	A2	USA	2	Complete Genome	3165
KF779347	A	A2	USA	2	Complete Genome	3165
KF779348	A	A2	USA	2	Complete Genome	3165
KF779349	A	A2	USA	2	Complete Genome	3165
KF779350	A	A2	USA	2	Complete Genome	3165
KF779351	A	A2	USA	2	Complete Genome	3165
KF779352	A	A2	USA	2	Complete Genome	3165
KF779354	A	A2	USA	2	Complete Genome	3165
KF779355	A	A2	USA	2	Complete Genome	3165
KF779356	A	A2	USA	2	Complete Genome	3165
KF779358	A	A2	USA	2	Complete Genome	3165
KF779359	A	A2	USA	2	Complete Genome	3165
KF779360	A	A2	USA	2	Complete Genome	3165
KF779361	A	A2	USA	2	Complete Genome	3165
KF779362	A	A2	USA	2	Complete Genome	3165
KF779363	A	A2	USA	2	Complete Genome	3165
KF779364	A	A2	USA	2	Complete Genome	3165
KF779365	A	A2	USA	2	Complete Genome	3165
KF779366	A	A2	USA	2	Complete Genome	3165
KF779367	A	A2	USA	2	Complete Genome	3165
KF779368	A	A2	USA	2	Complete Genome	3165
KF779369	A	A2	USA	2	Complete Genome	3165
KF779370	A	A2	USA	2	Complete Genome	3165
KF779371	A	A2	USA	2	Complete Genome	3165
KF779372	A	A2	USA	2	Complete Genome	3165
KF779373	A	A2	USA	2	Complete Genome	3165
KF779374	A	A2	USA	2	Complete Genome	3165
KF779375	A	A2	USA	2	Complete Genome	3165
KF779381	A	A2	USA	2	Complete Genome	3165
KF779383	A	A2	USA	2	Complete Genome	3165
KF779310	A	A2	USA	2	Complete Genome	3165

[1]Alignment to complete genome reference sequence VHB NC_003977.2

VENEZUELA

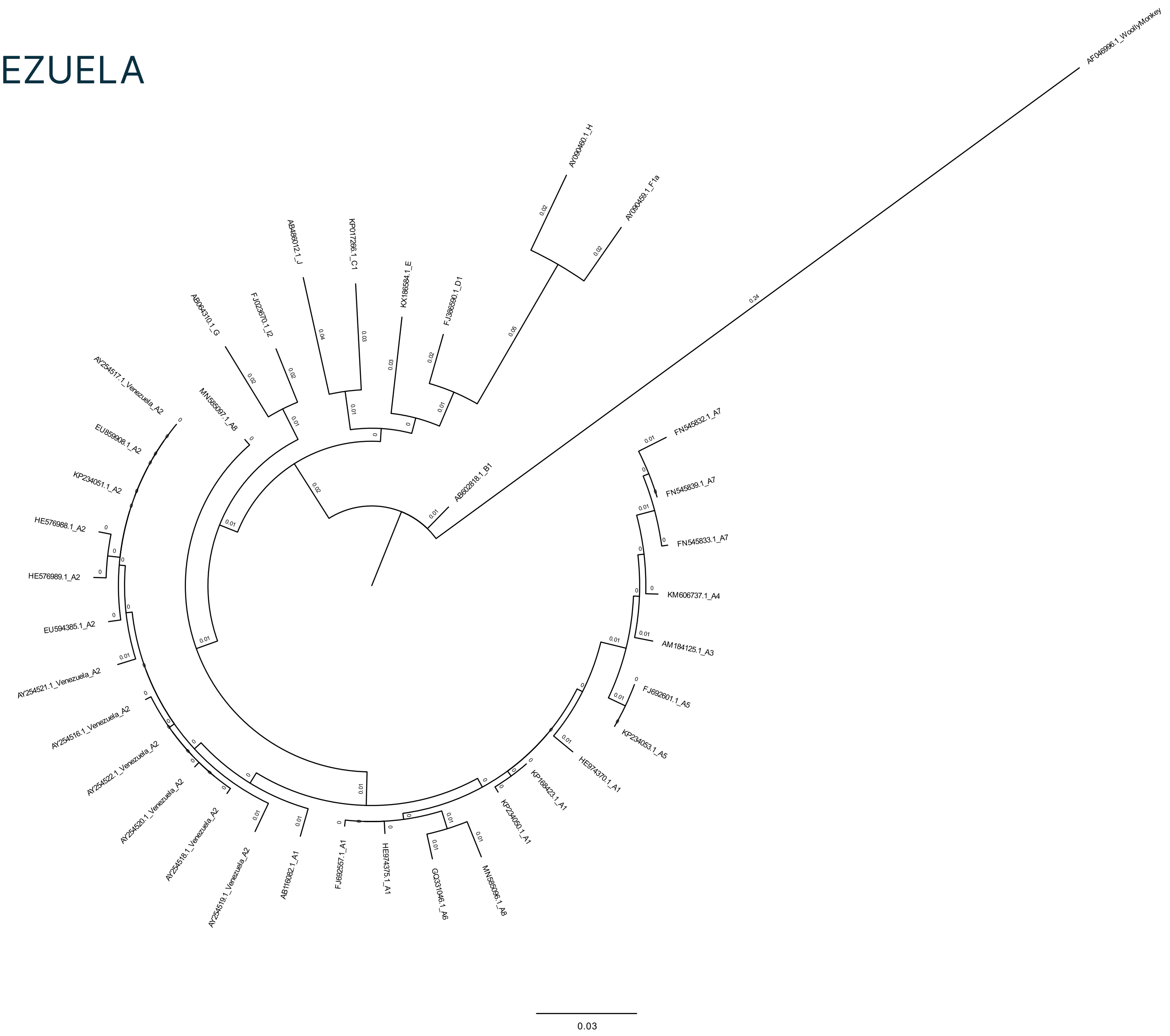

Tree 1. The evolutionary history was inferred by using the Maximum Likelihood method and Tamura-Nei model. The percentage of replicate trees in which the associated taxa clustered together in the bootstrap test (1000 replicates) are shown next to the branches. Initial tree(s) for the heuristic search were obtained automatically by applying Neighbor-Join and BioNJ algorithms to a matrix of pairwise distances estimated using the Tamura-Nei model, and then selecting the topology with superior log likelihood value. A discrete Gamma distribution was used to model evolutionary rate differences among sites (5 categories (+G, parameter = 0.1529)). The tree is drawn to scale, with branch lengths measured in the number of substitutions per site. The analysis involved 38 nucleotide sequences, of which 31 were used as marker sequences to determine the genotype of 7 sequences. All positions containing gaps and missing data were eliminated. There was a total of 659 positions in the final dataset. Evolutionary analyses were conducted in MEGA X.

ID	GENOTYPE	SUBTYPE	COUNTRY	TREE	ALIGNMENT[1]	BASE PAIRS
AY254516	A	A2	Venezuela	1	152-824	673
AY254517	A	A2	Venezuela	1	152-824	673
AY254518	A	A2	Venezuela	1	152-824	673
AY254519	A	A2	Venezuela	1	78-824	747
AY254520	A	A2	Venezuela	1	152-824	673
AY254521	A	A2	Venezuela	1	152-824	673
AY254522	A	A2	Venezuela	1	152-824	673

[1]Alignment to complete genome reference sequence VHB NC_003977.2

B – SUBGENOTYPE

ALASKA

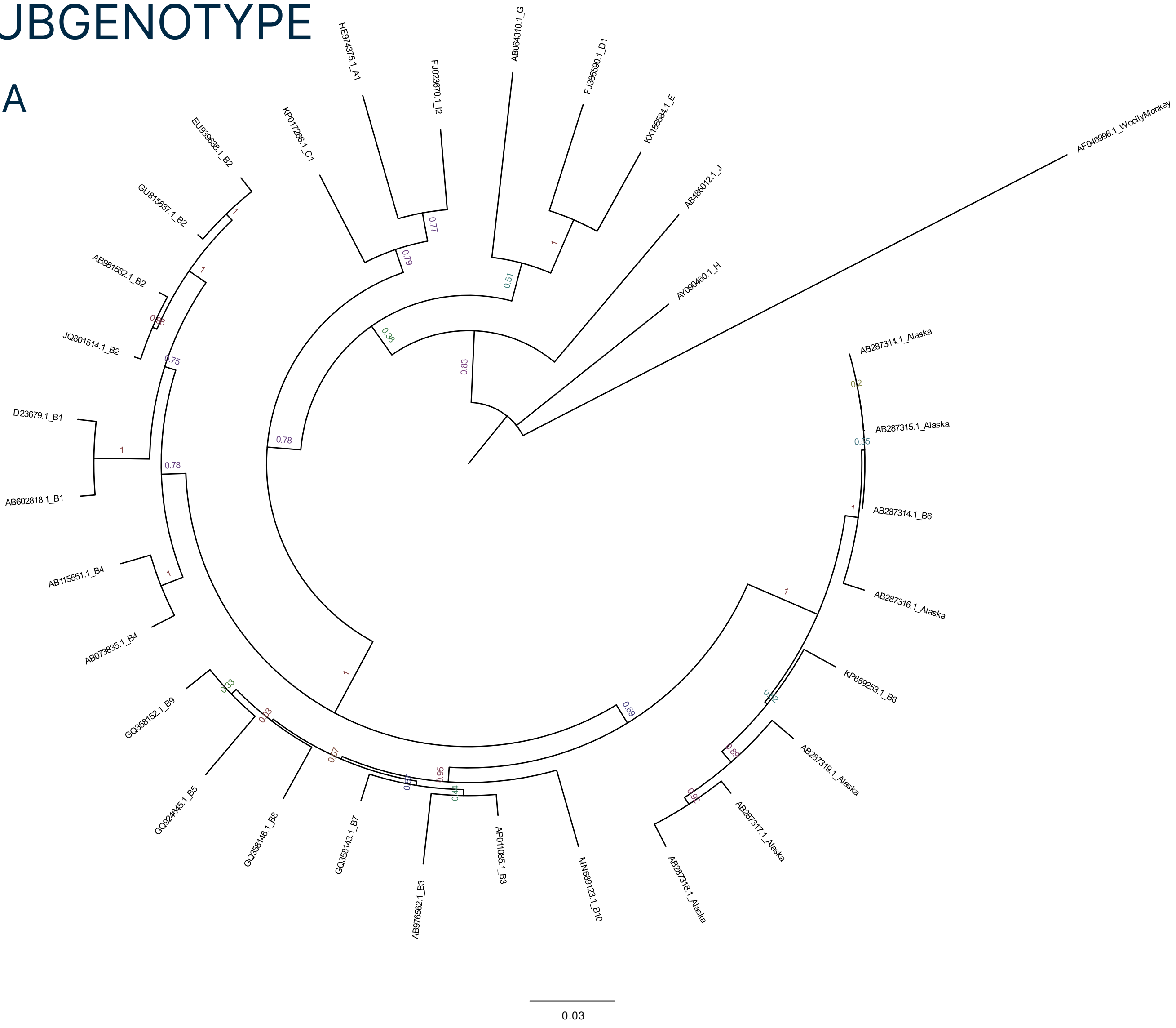

Tree 1. Molecular Phylogenetic analysis by Maximum Likelihood method conducted in MEGA7. The evolutionary history was inferred by using the Maximum Likelihood method based on the Tamura-Nei model with 1000 bootstraps. The tree with the highest log likelihood (-20540.40) is shown. The percentage of trees in which the associated taxa clustered together is shown next to the branches. The tree is drawn to scale, with branch lengths measured in the number of substitutions per site. The analysis involved 32 nucleotide sequences, of which 26 were used as marker sequences to determine the subgenotype of 6 sequences. All positions containing gaps and missing data were eliminated. There was a total of 3097 positions in the final dataset.

ID	GENOTYPE	SUBTYPE	COUNTRY	ALIGNMENT[1]	BASE PAIRS
AB287314	B	B6	Alaska	Complete Genome	3215
AB287315	B	B6	Alaska	Complete Genome	3215
AB287316	B	B6	Alaska	Complete Genome	3215
AB287317	B	B6	Alaska	Complete Genome	3215
AB287318	B	B6	Alaska	Complete Genome	3215
AB287319	B	B6	Alaska	Complete Genome	3215

ARGENTINA

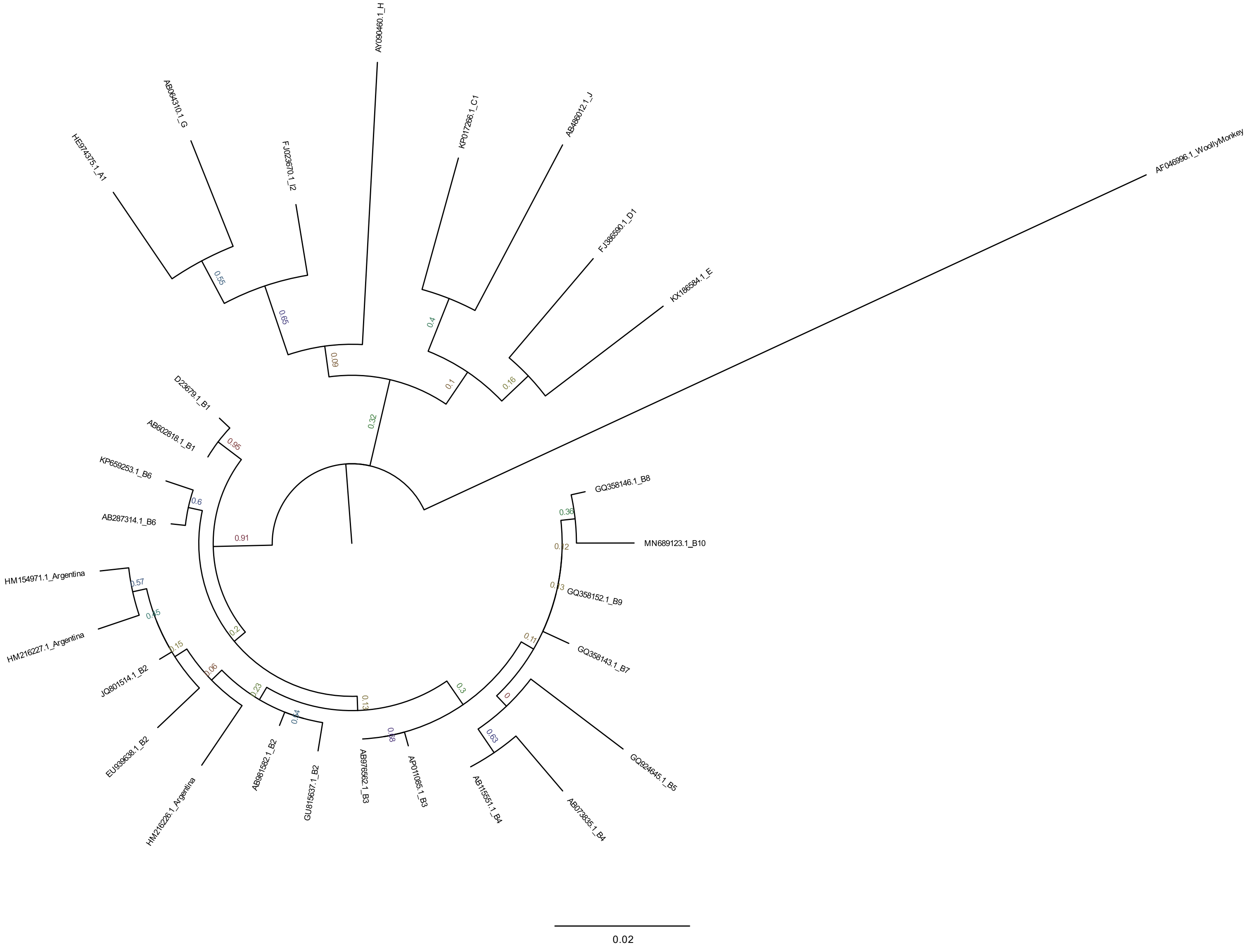

Tree 1. Molecular Phylogenetic analysis by Maximum Likelihood method conducted in MEGA7. The evolutionary history was inferred by using the Maximum Likelihood method based on the Tamura-Nei model with 1000 bootstraps. The tree with the highest log likelihood (-1896.56) is shown. The percentage of trees in which the associated taxa clustered together is shown next to the branches. The tree is drawn to scale, with branch lengths measured in the number of substitutions per site. The analysis involved 29 nucleotide sequences, of which 26 were used as marker sequences to determine the subgenotype of 3 sequences. All positions containing gaps and missing data were eliminated. There was a total of 471 positions in the final dataset.

ID	GENOTYPE	SUBTYPE	COUNTRY	ALIGNMENT[1]	BASE PAIRS
HM154971	B	B2	Argentina	244-753	504
HM216226	B	B2	Argentina	259-772	508
HM216227	B	B2	Argentina	262-772	505

BRAZIL

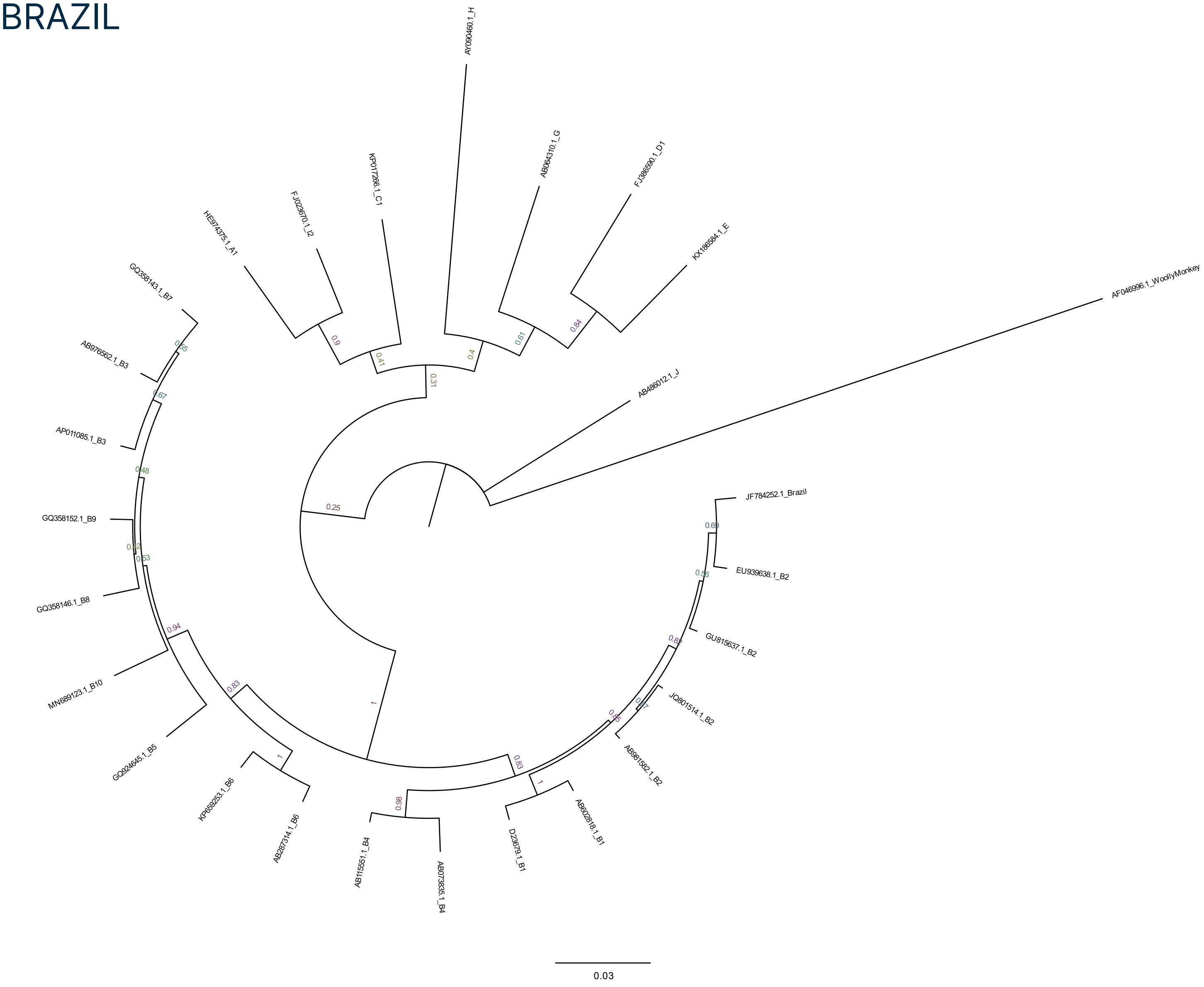

Tree 1. Molecular Phylogenetic analysis by Maximum Likelihood method conducted in MEGA7. The evolutionary history was inferred by using the Maximum Likelihood method based on the Tamura-Nei model with 1000 bootstraps. The tree with the highest log likelihood (-6281.18) is shown. The percentage of trees in which the associated taxa clustered together is shown next to the branches. The tree is drawn to scale, with branch lengths measured in the number of substitutions per site. The analysis involved 27 nucleotide sequences, of which 26 were used as marker sequences to determine the subgenotype of 1 sequence. All positions containing gaps and missing data were eliminated. There was a total of 1131 positions in the final dataset.

ID	GENOTYPE	SUBTYPE	COUNTRY	ALIGNMENT[1]	BASE PAIRS
JF784252	B	B2	Brazil	1-3182	1187

CANADA

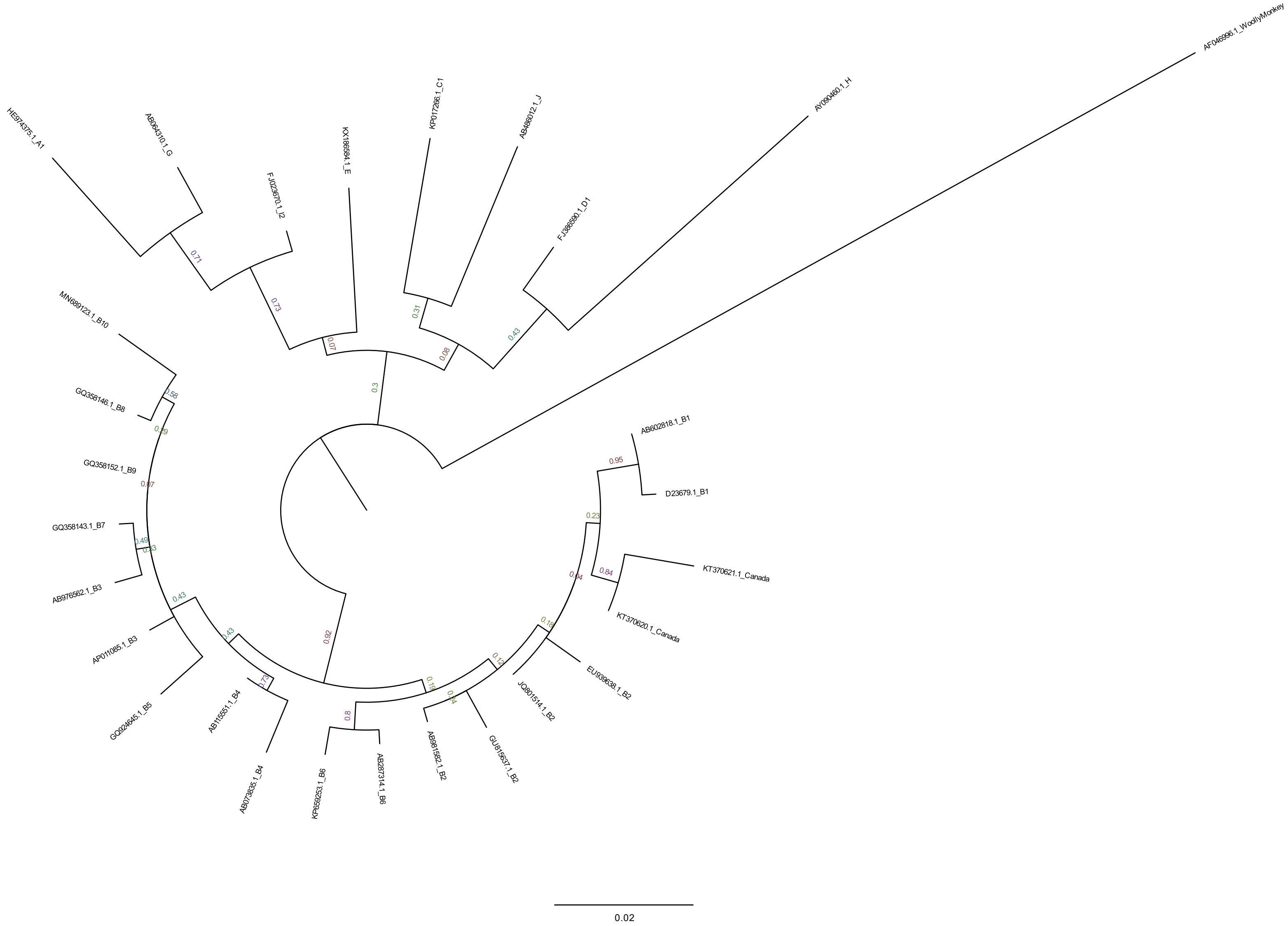

Tree 1. Molecular Phylogenetic analysis by Maximum Likelihood method conducted in MEGA7. The evolutionary history was inferred by using the Maximum Likelihood method based on the Tamura-Nei model with 1000 bootstraps. The tree with the highest log likelihood (-1928.74) is shown. The percentage of trees in which the associated taxa clustered together is shown next to the branches. The tree is drawn to scale, with branch lengths measured in the number of substitutions per site. The analysis involved 28 nucleotide sequences, of which 26 were used as marker sequences to determine the subgenotype of 2 sequences. All positions containing gaps and missing data were eliminated. There was a total of 498 positions in the final dataset.

ID	GENOTYPE	SUBTYPE	COUNTRY	ALIGNMENT[1]	BASE PAIRS
KT370620	B	B1	Canada	315-831	517
KT370621	B	B1	Canada	313-831	519

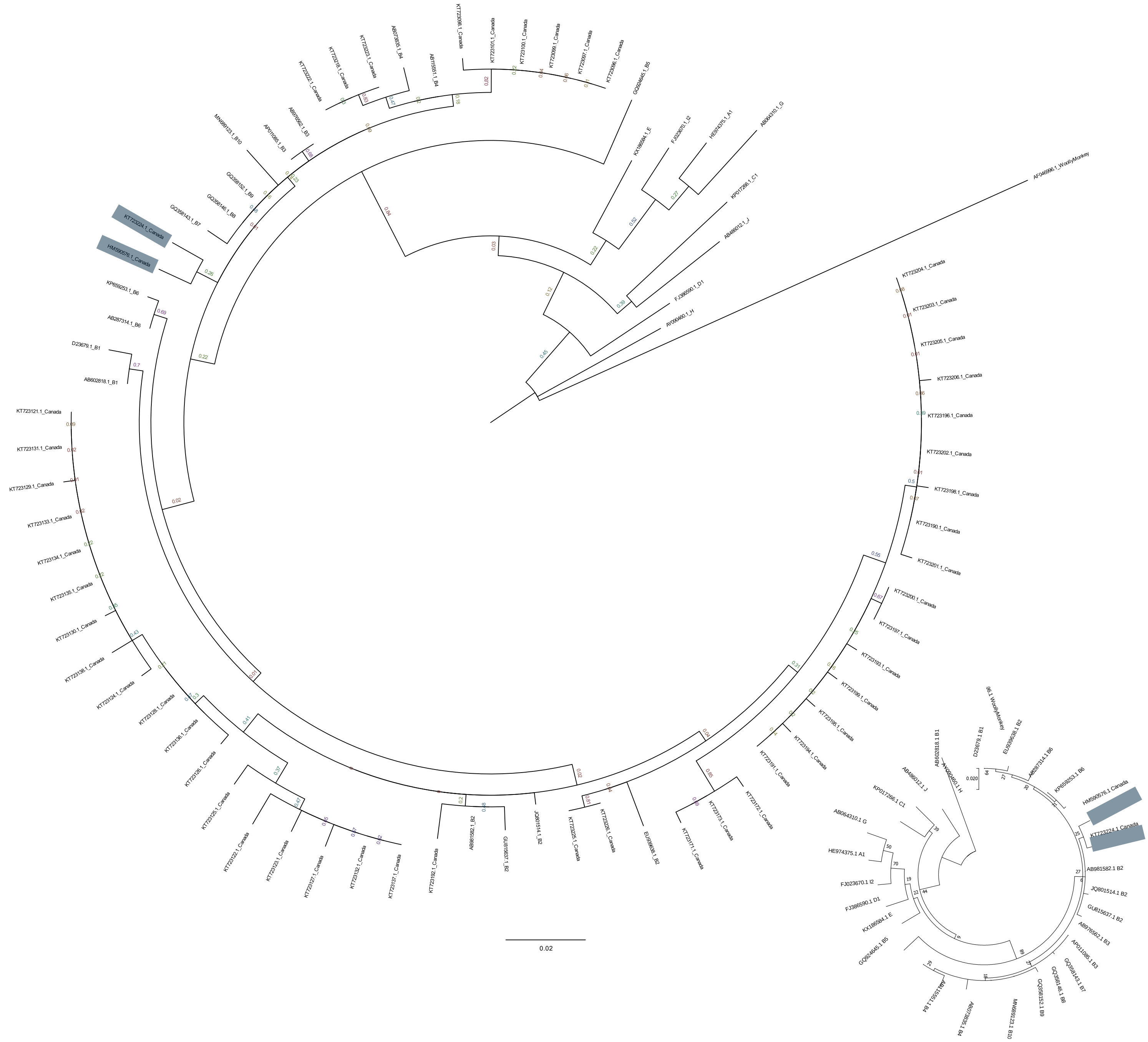

Tree 2. Molecular Phylogenetic analysis by Maximum Likelihood method conducted in MEGA7. The evolutionary history was inferred by using the Maximum Likelihood method based on the Tamura-Nei model with 1000 bootstraps. The tree with the highest log likelihood (-1817.92) is shown. The percentage of trees in which the associated taxa clustered together is shown next to the branches. The tree is drawn to scale, with branch lengths measured in the number of substitutions per site. The analysis involved 77 nucleotide sequences, of which 26 were used as marker sequences to determine the subgenotype of 51 sequences. All positions containing gaps and missing data were eliminated. There was a total of 342 positions in the final dataset. An additional tree was constructed to further narrow down the subtype of 2 sequences.

ID	GENOTYPE	SUBTYPE	COUNTRY	ALIGNMENT[1]	BASE PAIRS
HM590576	B	B2	Canada	1-3182	681
KT723121	B	B2	Canada	1-3182	981
KT723122	B	B2	Canada	1-3182	982
KT723123	B	B2	Canada	1-3182	981
KT723124	B	B2	Canada	1-3182	981
KT723125	B	B2	Canada	1-3182	982

KT723126	B	B2	Canada	1-3182	982
KT723127	B	B2	Canada	1-3182	982
KT723128	B	B2	Canada	1-3182	982
KT723129	B	B2	Canada	1-3182	981
KT723130	B	B2	Canada	1-3182	982
KT723131	B	B2	Canada	1-3182	982
KT723132	B	B2	Canada	1-3182	981
KT723133	B	B2	Canada	1-3182	982
KT723134	B	B2	Canada	1-3182	982
KT723135	B	B2	Canada	1-3182	982
KT723136	B	B2	Canada	1-3182	982
KT723137	B	B2	Canada	1-3182	981
KT723138	B	B2	Canada	1-3182	982
KT723171	B	B2	Canada	1-3182	982
KT723172	B	B2	Canada	1-3182	981
KT723173	B	B2	Canada	1-3182	982
KT723190	B	B2	Canada	1-3182	982
KT723191	B	B2	Canada	1-3182	982
KT723192	B	B2	Canada	1-3182	982
KT723193	B	B2	Canada	1-3182	982
KT723194	B	B2	Canada	1-3182	982
KT723195	B	B2	Canada	1-3182	982
KT723196	B	B2	Canada	1-3182	982
KT723197	B	B2	Canada	1-3182	982
KT723198	B	B2	Canada	1-3182	982
KT723199	B	B2	Canada	1-3182	982
KT723200	B	B2	Canada	1-3182	982
KT723201	B	B2	Canada	1-3182	982
KT723202	B	B2	Canada	1-3182	982
KT723203	B	B2	Canada	1-3182	982
KT723204	B	B2	Canada	1-3182	982
KT723205	B	B2	Canada	1-3182	982
KT723206	B	B2	Canada	1-3182	982
KT723224	B	B2	Canada	21-3109	889
KT723225	B	B2	Canada	1-3182	982
KT723226	B	B2	Canada	1-3182	982
KT723096	B	B4	Canada	1-3182	982
KT723097	B	B4	Canada	1-3182	982
KT723098	B	B4	Canada	1-3182	982
KT723099	B	B4	Canada	1-3182	982
KT723100	B	B4	Canada	1-3182	982
KT723101	B	B4	Canada	1-3182	982
KT723218	B	B4	Canada	1-3182	982
KT723222	B	B4	Canada	1-3182	982
KT723223	B	B4	Canada	1-3182	982

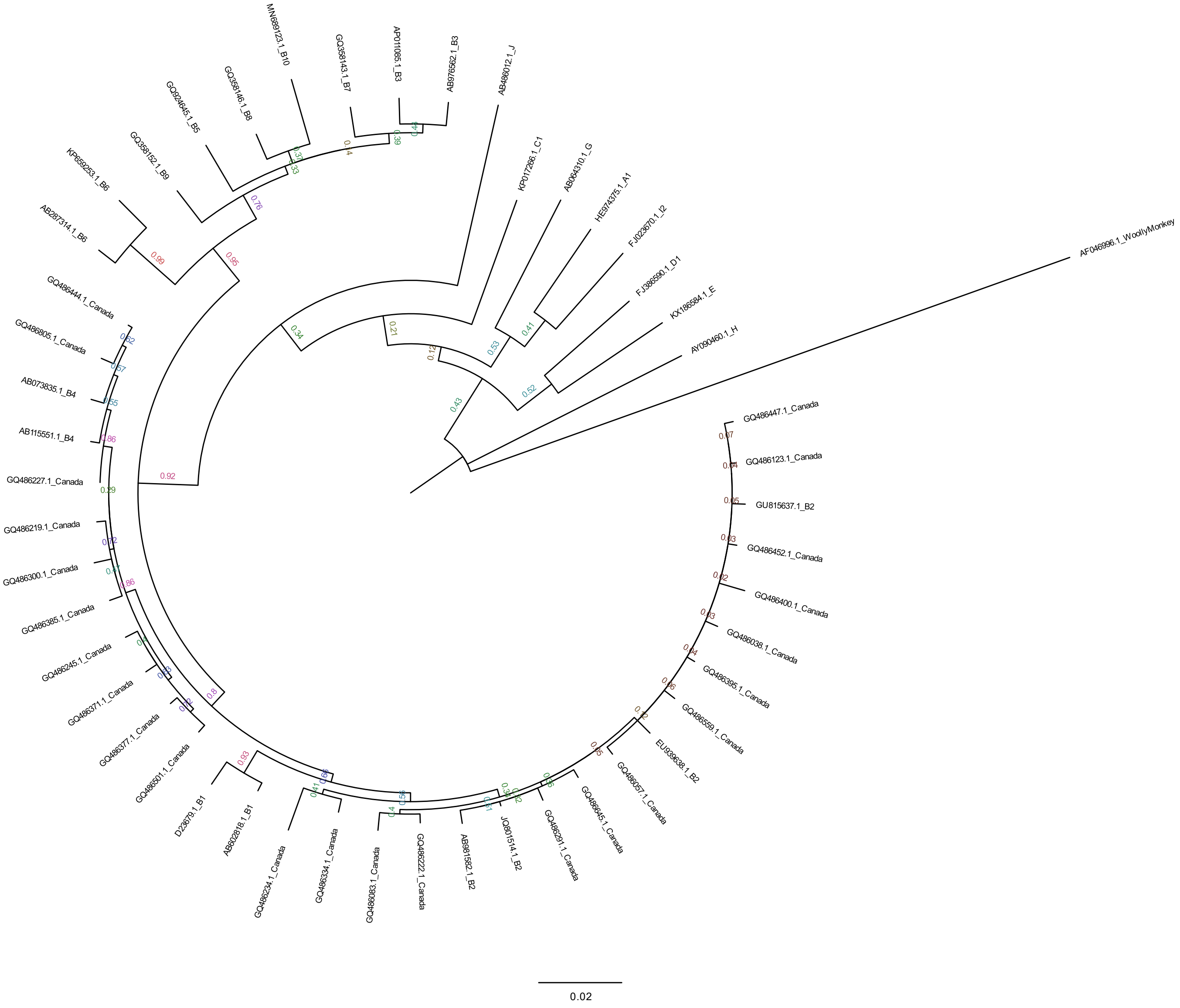

Tree 3. Molecular Phylogenetic analysis by Maximum Likelihood method conducted in MEGA7. The evolutionary history was inferred by using the Maximum Likelihood method based on the Tamura-Nei model with 1000 bootstraps. The tree with the highest log likelihood (-5203.05) is shown. The percentage of trees in which the associated taxa clustered together is shown next to the branches. The tree is drawn to scale, with branch lengths measured in the number of substitutions per site. The analysis involved 50 nucleotide sequences, of which 26 were used as marker sequences to determine the subgenotype of 24 sequences. All positions containing gaps and missing data were eliminated. There was a total of 938 positions in the final dataset.

ID	GENOTYPE	SUBTYPE	COUNTRY	ALIGNMENT[1]	BASE PAIRS
GQ486038	B	B2	Canada	132-1163	1032
GQ486057	B	B2	Canada	132-1163	1032
GQ486083	B	B2	Canada	132-1163	1032
GQ486123	B	B2	Canada	132-1163	1032
GQ486222	B	B2	Canada	132-1163	1032
GQ486234	B	B2	Canada	132-1163	1032
GQ486291	B	B2	Canada	132-1163	1032
GQ486334	B	B2	Canada	132-1163	1032

GQ486395	B	B2	Canada	132-1163	1032
GQ486400	B	B2	Canada	132-1163	1032
GQ486447	B	B2	Canada	132-1163	1032
GQ486452	B	B2	Canada	132-1163	1032
GQ486559	B	B2	Canada	132-1163	1032
GQ486645	B	B2	Canada	132-1163	1032
GQ486377	B	B4	Canada	132-1163	1032
GQ486219	B	B4	Canada	132-1163	1032
GQ486227	B	B4	Canada	132-1163	1032
GQ486245	B	B4	Canada	132-1163	1032
GQ486300	B	B4	Canada	132-1163	1032
GQ486371	B	B4	Canada	132-1163	1032
GQ486385	B	B4	Canada	132-1163	1032
GQ486444	B	B4	Canada	132-1163	1032
GQ486501	B	B4	Canada	132-1163	1032
GQ486805	B	B4	Canada	132-1163	1032

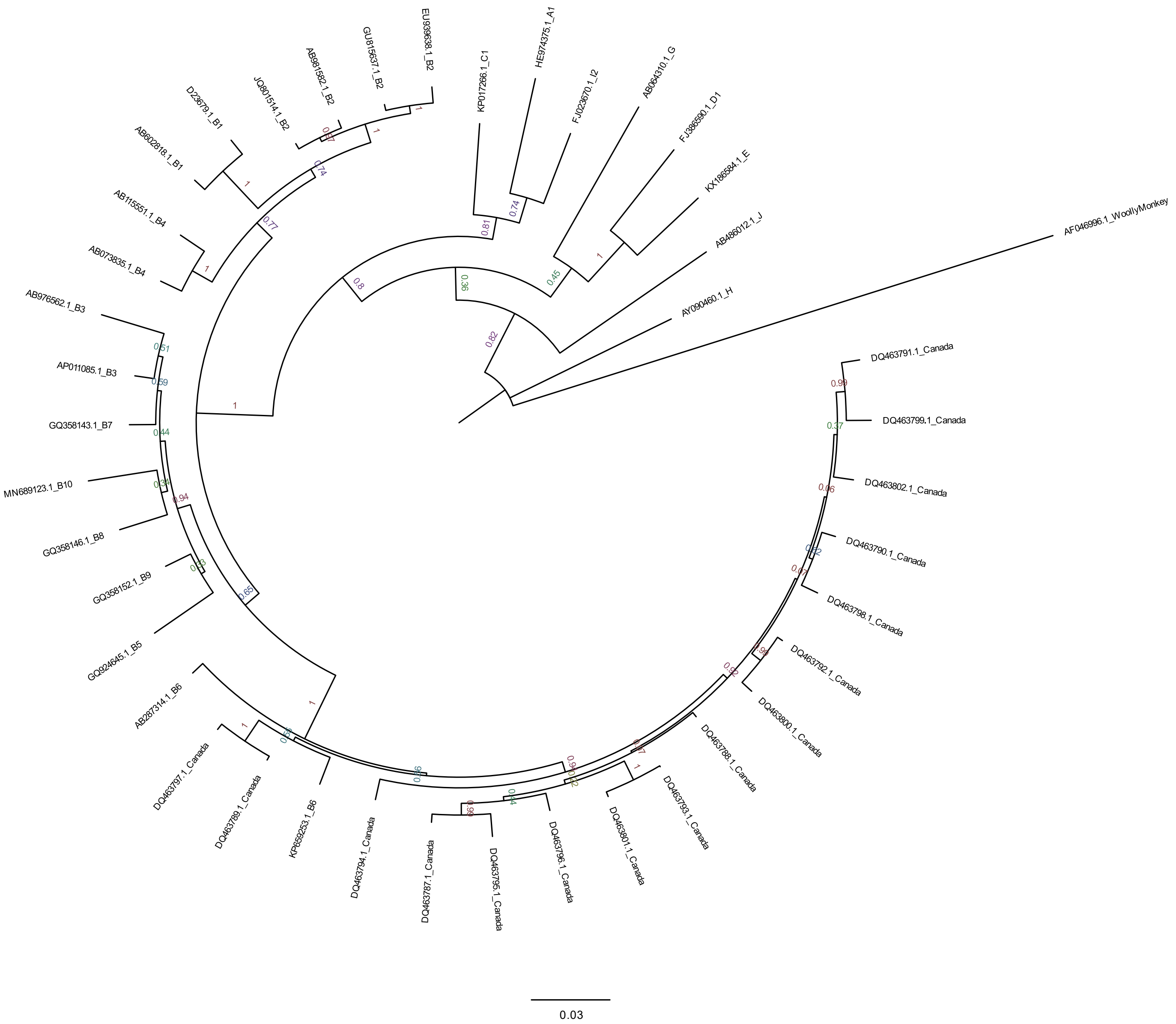

Tree 4. Molecular Phylogenetic analysis by Maximum Likelihood method conducted in MEGA7. The evolutionary history was inferred by using the Maximum Likelihood method based on the Tamura-Nei model with 1000 bootstraps. The tree with the highest log likelihood (-22404.07) is shown. The percentage of trees in which the associated taxa clustered together is shown next to the branches. The tree is drawn to scale, with branch lengths measured in the number of substitutions per site. The analysis involved 42 nucleotide sequences, of which 26 were used as marker sequences to determine the subgenotype of 16 sequences. All positions containing gaps and missing data were eliminated. There was a total of 3096 positions in the final dataset.

ID	GENOTYPE	SUBTYPE	COUNTRY	ALIGNMENT[1]	BASE PAIRS
DQ463787	B	B6	Canada	Complete Genome	3215
DQ463788	B	B6	Canada	Complete Genome	3215
DQ463789	B	B6	Canada	Complete Genome	3215
DQ463790	B	B6	Canada	Complete Genome	3215
DQ463791	B	B6	Canada	Complete Genome	3215
DQ463792	B	B6	Canada	Complete Genome	3215
DQ463793	B	B6	Canada	Complete Genome	3215
DQ463794	B	B6	Canada	Complete Genome	3215

DQ463795	B	B6	Canada	Complete Genome	3215
DQ463796	B	B6	Canada	Complete Genome	3215
DQ463797	B	B6	Canada	Complete Genome	3215
DQ463798	B	B6	Canada	Complete Genome	3215
DQ463799	B	B6	Canada	Complete Genome	3215
DQ463800	B	B6	Canada	Complete Genome	3215
DQ463801	B	B6	Canada	Complete Genome	3215
DQ463802	B	B6	Canada	Complete Genome	3215

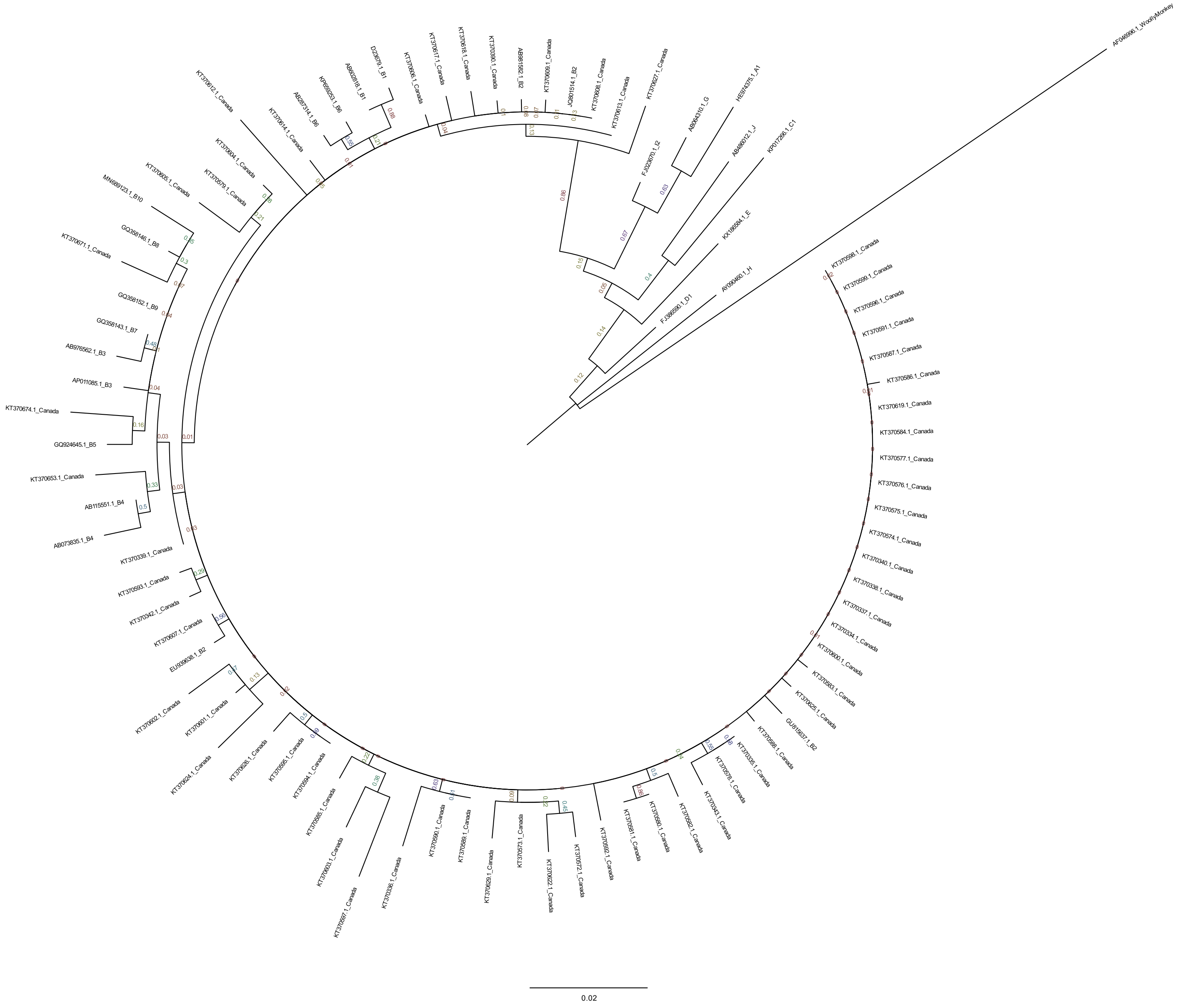

Tree 5. Molecular Phylogenetic analysis by Maximum Likelihood method conducted in MEGA7. The evolutionary history was inferred by using the Maximum Likelihood method based on the Tamura-Nei model with 1000 bootstraps. The tree with the highest log likelihood (-2419.82) is shown. The percentage of trees in which the associated taxa clustered together is shown next to the branches. The tree is drawn to scale, with branch lengths measured in the number of substitutions per site. The analysis involved 89 nucleotide sequences, of which 26 were used as marker sequences to determine the subgenotype of 63 sequences. All positions containing gaps and missing data were eliminated. There was a total of 473 positions in the final dataset.

ID	GENOTYPE	SUBTYPE	COUNTRY	ALIGNMENT[1]	BASE PAIRS
KT370334	B	B2	Canada	313-831	519
KT370335	B	B2	Canada	313-831	519
KT370336	B	B2	Canada	313-831	519
KT370337	B	B2	Canada	313-831	519
KT370338	B	B2	Canada	313-831	519
KT370339	B	B2	Canada	313-831	519
KT370340	B	B2	Canada	313-831	519
KT370342	B	B2	Canada	313-831	519

KT370343	B	B2	Canada	313-831	519
KT370390	B	B2	Canada	313-822	510
KT370572	B	B2	Canada	313-831	519
KT370573	B	B2	Canada	313-831	519
KT370574	B	B2	Canada	313-831	519
KT370575	B	B2	Canada	313-831	519
KT370576	B	B2	Canada	313-831	519
KT370577	B	B2	Canada	313-831	519
KT370578	B	B2	Canada	313-831	519
KT370579	B	B2	Canada	313-831	519
KT370580	B	B2	Canada	313-831	519
KT370581	B	B2	Canada	313-831	519
KT370582	B	B2	Canada	313-831	519
KT370583	B	B2	Canada	313-831	519
KT370584	B	B2	Canada	313-831	519
KT370585	B	B2	Canada	313-831	519
KT370586	B	B2	Canada	313-831	519
KT370587	B	B2	Canada	313-831	519
KT370588	B	B2	Canada	313-831	519
KT370589	B	B2	Canada	313-831	519
KT370590	B	B2	Canada	313-831	519
KT370591	B	B2	Canada	313-831	519
KT370592	B	B2	Canada	313-831	519
KT370593	B	B2	Canada	313-831	519
KT370594	B	B2	Canada	313-831	519
KT370595	B	B2	Canada	313-831	519
KT370596	B	B2	Canada	313-831	519
KT370597	B	B2	Canada	313-831	519
KT370598	B	B2	Canada	313-831	519
KT370599	B	B2	Canada	313-831	519
KT370600	B	B2	Canada	313-831	519
KT370601	B	B2	Canada	313-831	519
KT370602	B	B2	Canada	313-831	519
KT370603	B	B2	Canada	313-831	519
KT370604	B	B2	Canada	313-831	519
KT370605	B	B2	Canada	313-831	519
KT370606	B	B2	Canada	313-831	519
KT370607	B	B2	Canada	313-831	519
KT370608	B	B2	Canada	313-831	519
KT370609	B	B2	Canada	313-831	519
KT370612	B	B2	Canada	313-831	519
KT370613	B	B2	Canada	313-831	519
KT370614	B	B2	Canada	313-831	519
KT370617	B	B2	Canada	313-831	519
KT370618	B	B2	Canada	313-831	519
KT370619	B	B2	Canada	313-831	519

KT370622	B	B2	Canada	313-831	519
KT370624	B	B2	Canada	313-831	519
KT370625	B	B2	Canada	313-831	519
KT370626	B	B2	Canada	313-831	519
KT370627	B	B2	Canada	313-831	519
KT370629	B	B2	Canada	313-831	519
KT370653	B	B4	Canada	313-831	519
KT370671	B	B10	Canada	313-831	519
KT370674	B	B5	Canada	313-831	519

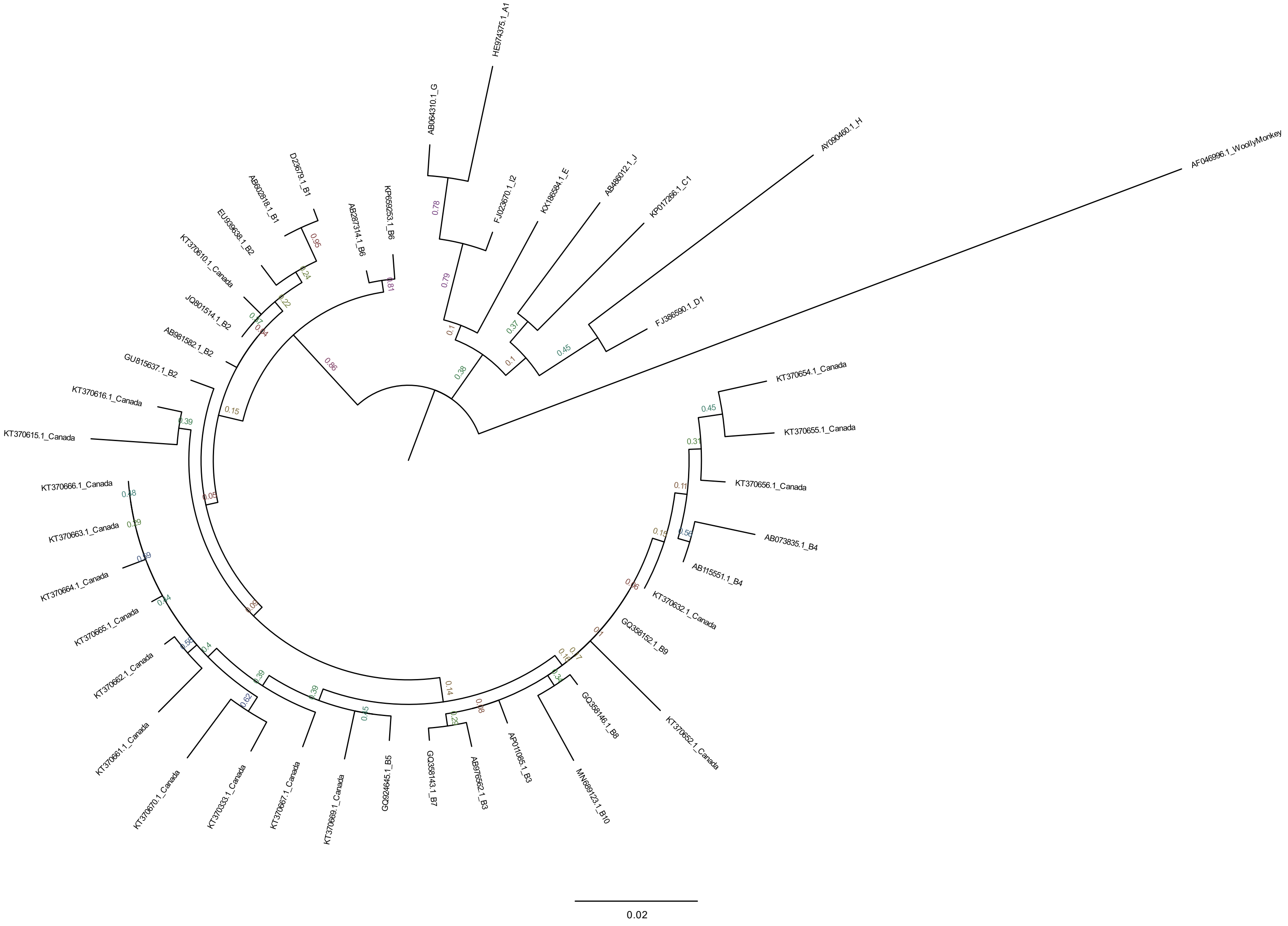

Tree 6. Molecular Phylogenetic analysis by Maximum Likelihood method conducted in MEGA7. The evolutionary history was inferred by using the Maximum Likelihood method based on the Tamura-Nei model with 1000 bootstraps. The tree with the highest log likelihood (-2329.66) is shown. The percentage of trees in which the associated taxa clustered together is shown next to the branches. The tree is drawn to scale, with branch lengths measured in the number of substitutions per site. The analysis involved 44 nucleotide sequences, of which 26 were used as marker sequences to determine the subgenotype of 18 sequences. All positions containing gaps and missing data were eliminated. There was a total of 501 positions in the final dataset.

ID	GENOTYPE	SUBTYPE	COUNTRY	ALIGNMENT[1]	BASE PAIRS
KT370333	B	B5	Canada	313-831	519
KT370610	B	B2	Canada	313-831	519
KT370615	B	B2	Canada	313-831	519
KT370616	B	B2	Canada	313-831	519
KT370632	B	B4	Canada	313-831	519
KT370652	B	B10	Canada	313-831	519
KT370654	B	B4	Canada	313-831	519
KT370655	B	B4	Canada	313-831	519
KT370656	B	B4	Canada	313-831	519
KT370661	B	B5	Canada	313-831	519

KT370662	B	B5	Canada	313-831	519
KT370663	B	B5	Canada	313-831	519
KT370664	B	B5	Canada	313-831	519
KT370665	B	B5	Canada	313-831	519
KT370666	B	B5	Canada	313-831	519
KT370667	B	B5	Canada	313-831	519
KT370669	B	B5	Canada	313-831	519
KT370670	B	B5	Canada	313-831	519

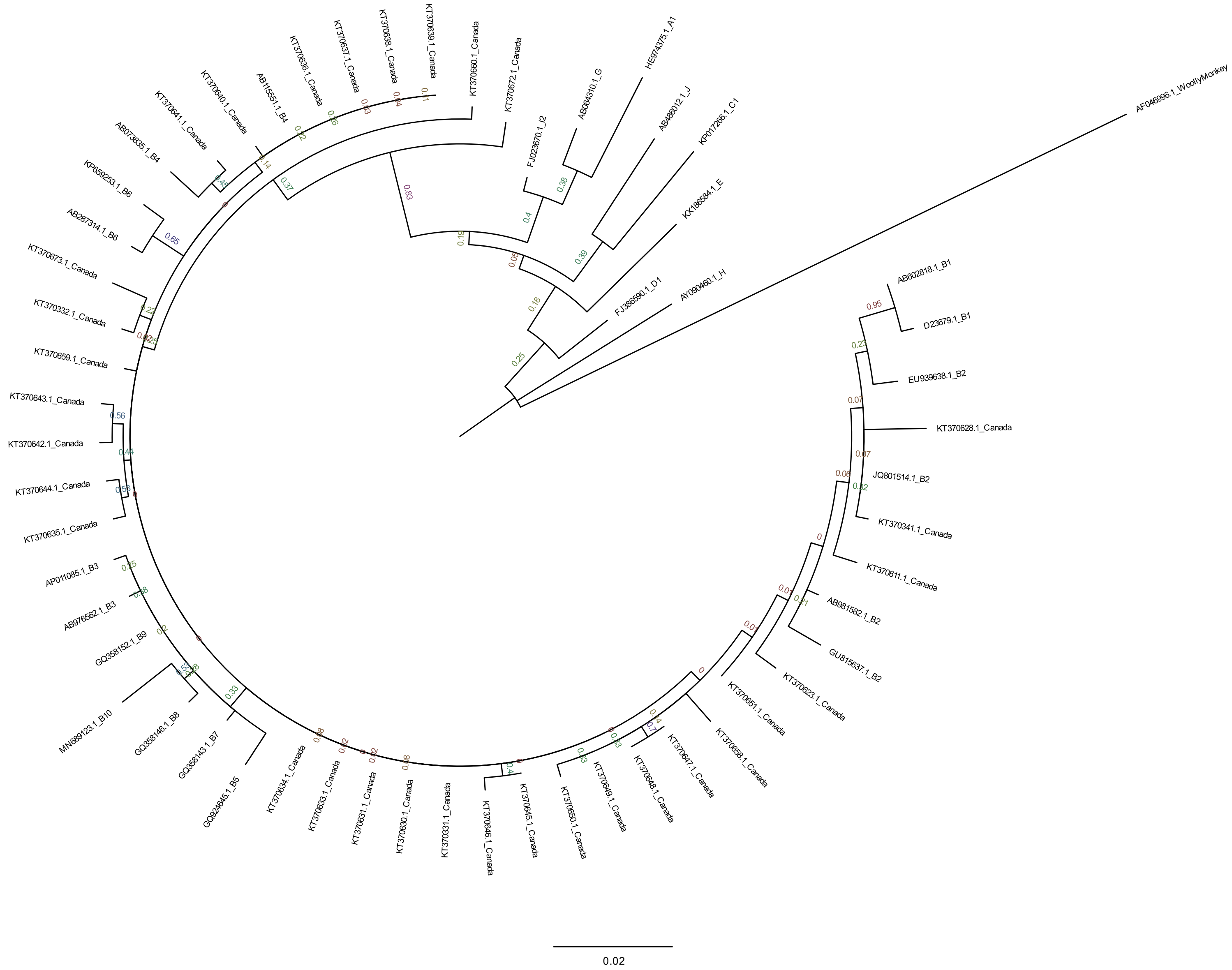

Tree 7. Molecular Phylogenetic analysis by Maximum Likelihood method conducted in MEGA7. The evolutionary history was inferred by using the Maximum Likelihood method based on the Tamura-Nei model with 1000 bootstraps. The tree with the highest log likelihood (-1997.97) is shown. The percentage of trees in which the associated taxa clustered together is shown next to the branches. The tree is drawn to scale, with branch lengths measured in the number of substitutions per site. The analysis involved 58 nucleotide sequences, of which 26 were used as marker sequences to determine the subgenotype of 32 sequences. All positions containing gaps and missing data were eliminated. There was a total of 480 positions in the final dataset.

ID	GENOTYPE	SUBTYPE	COUNTRY	ALIGNMENT[1]	BASE PAIRS
KT370331	B	B4	Canada	313-831	519
KT370332	B	B4	Canada	313-831	519
KT370341	B	B2	Canada	313-831	519
KT370611	B	B2	Canada	313-831	519
KT370623	B	B2	Canada	313-831	519
KT370628	B	B2	Canada	313-829	517
KT370630	B	B4	Canada	313-831	519
KT370631	B	B4	Canada	313-831	519

KT370633	B	B4	Canada	313-831	519
KT370634	B	B4	Canada	313-831	519
KT370635	B	B4	Canada	313-831	519
KT370636	B	B4	Canada	313-831	519
KT370637	B	B4	Canada	313-831	519
KT370638	B	B4	Canada	313-831	519
KT370639	B	B4	Canada	313-831	519
KT370640	B	B4	Canada	313-831	519
KT370641	B	B4	Canada	313-831	519
KT370642	B	B4	Canada	313-831	519
KT370643	B	B4	Canada	313-831	519
KT370644	B	B4	Canada	313-831	519
KT370645	B	B4	Canada	313-831	519
KT370646	B	B4	Canada	313-831	519
KT370647	B	B4	Canada	313-831	519
KT370648	B	B4	Canada	313-831	519
KT370649	B	B4	Canada	313-831	519
KT370650	B	B4	Canada	313-831	519
KT370651	B	B4	Canada	313-831	519
KT370658	B	B4	Canada	313-831	519
KT370659	B	B4	Canada	313-831	519
KT370660	B	B4	Canada	313-831	519
KT370672	B	B4	Canada	313-831	519
KT370673	B	B4	Canada	313-831	519

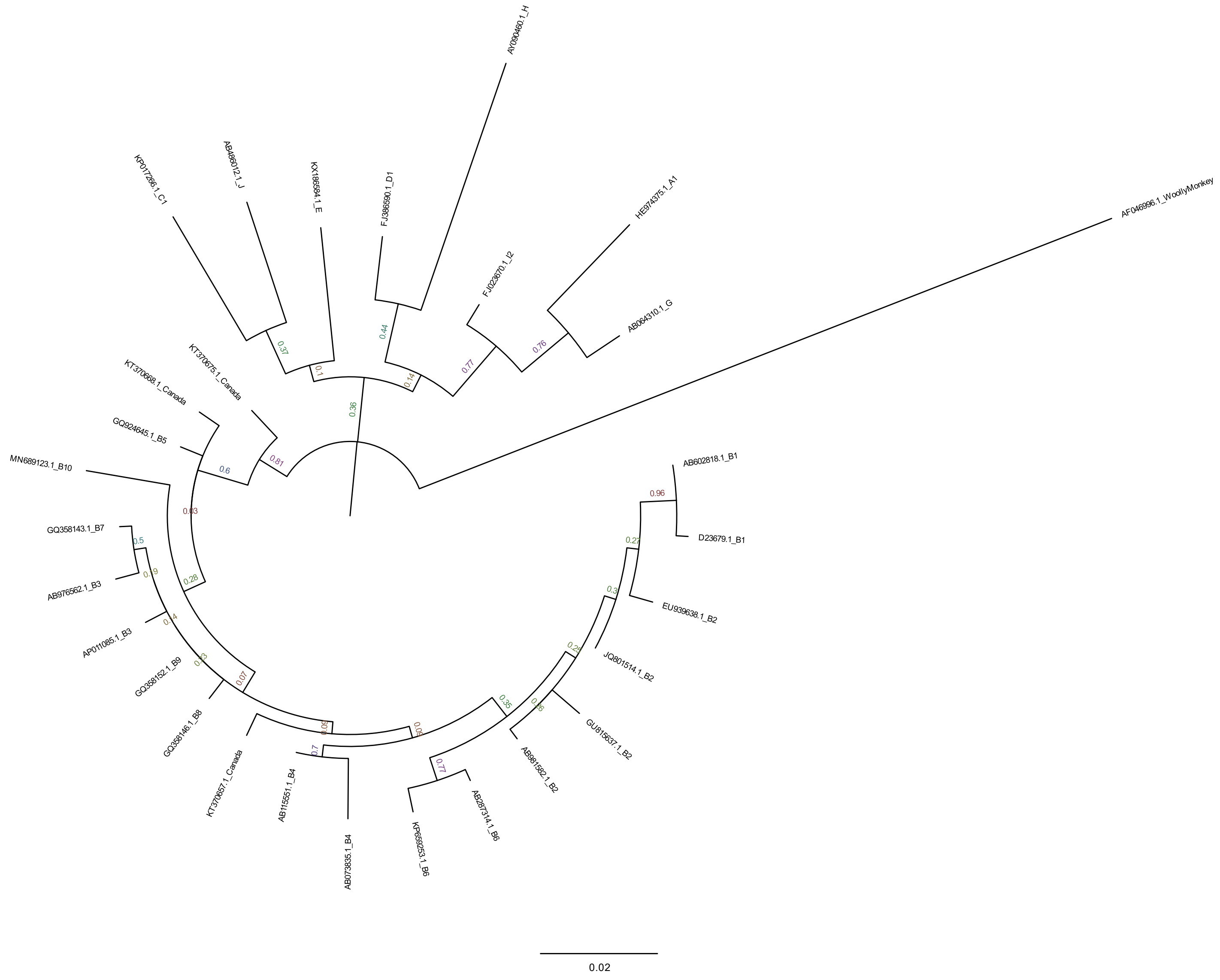

Tree 8. Molecular Phylogenetic analysis by Maximum Likelihood method conducted in MEGA7. The evolutionary history was inferred by using the Maximum Likelihood method based on the Tamura-Nei model with 1000 bootstraps. The tree with the highest log likelihood (-1997.97) is shown. The percentage of trees in which the associated taxa clustered together is shown next to the branches. The tree is drawn to scale, with branch lengths measured in the number of substitutions per site. The analysis involved 29 nucleotide sequences, of which 26 were used as marker sequences to determine the subgenotype of 3 sequences. All positions containing gaps and missing data were eliminated. There was a total of 480 positions in the final dataset.

ID	GENOTYPE	SUBTYPE	COUNTRY	ALIGNMENT[1]	BASE PAIRS
KT370668	B	B5	Canada	313-831	519
KT370657	B	B4	Canada	313-831	519
KT370675	B	B5	Canada	325-831	507

GREENLAND

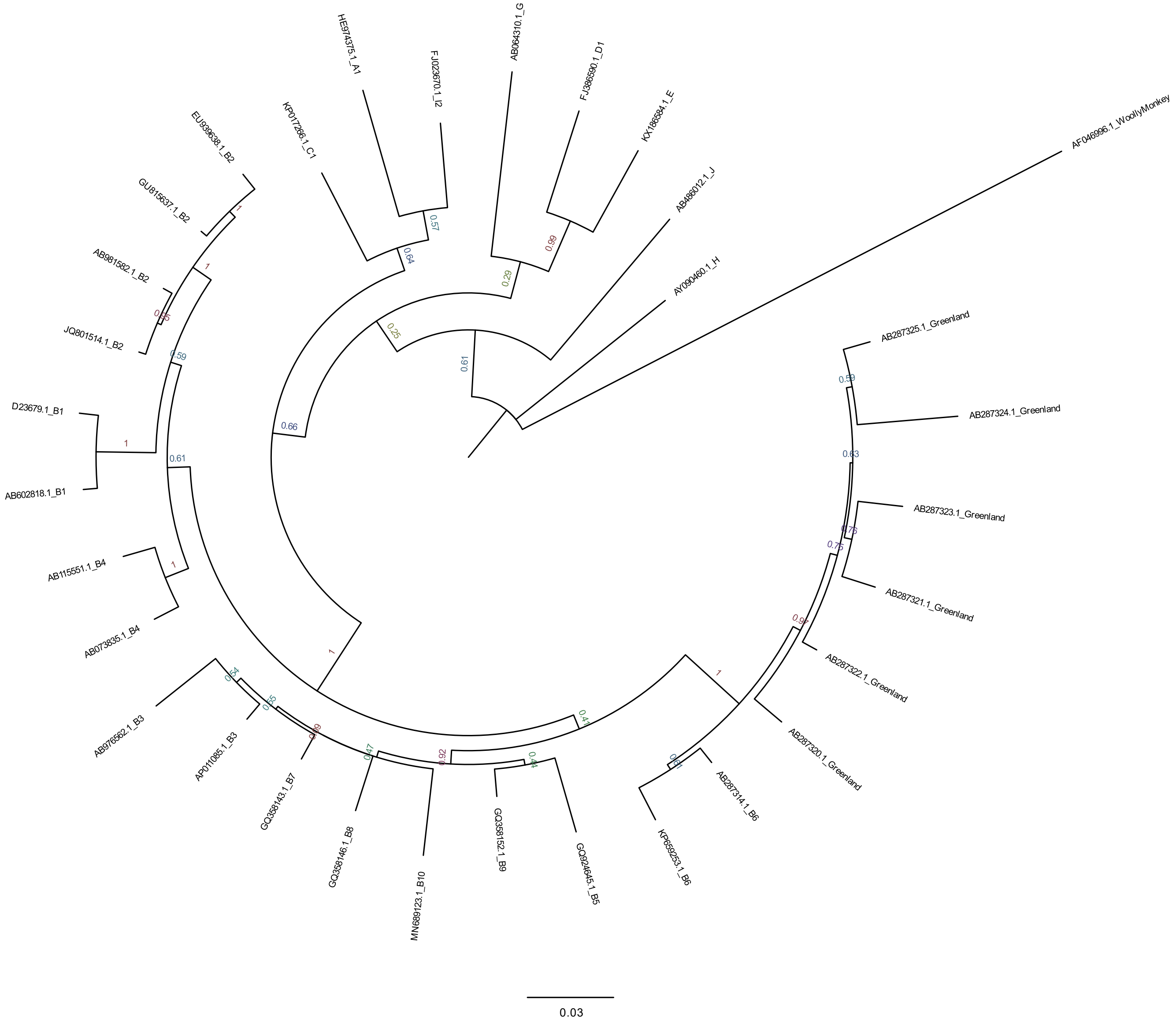

Tree 1. Molecular Phylogenetic analysis by Maximum Likelihood method conducted in MEGA7. The evolutionary history was inferred by using the Maximum Likelihood method based on the Tamura-Nei model with 1000 bootstraps. The tree with the highest log likelihood (-19967.55) is shown. The percentage of trees in which the associated taxa clustered together is shown next to the branches. The tree is drawn to scale, with branch lengths measured in the number of substitutions per site. The analysis involved 32 nucleotide sequences, of which 26 were used as marker sequences to determine the subgenotype of 6 sequences. All positions containing gaps and missing data were eliminated. There was a total of 2886 positions in the final dataset.

ID	GENOTYPE	SUBTYPE	COUNTRY	ALIGNMENT[1]	BASE PAIRS
AB287320	B	B6	Greenland	Complete Genome	3215
AB287321	B	B6	Greenland	Complete Genome	3215
AB287322	B	B6	Greenland	Complete Genome	3215
AB287323	B	B6	Greenland	Complete Genome	3215
AB287324	B	B6	Greenland	Complete Genome	2987
AB287325	B	B6	Greenland	Complete Genome	3215

USA

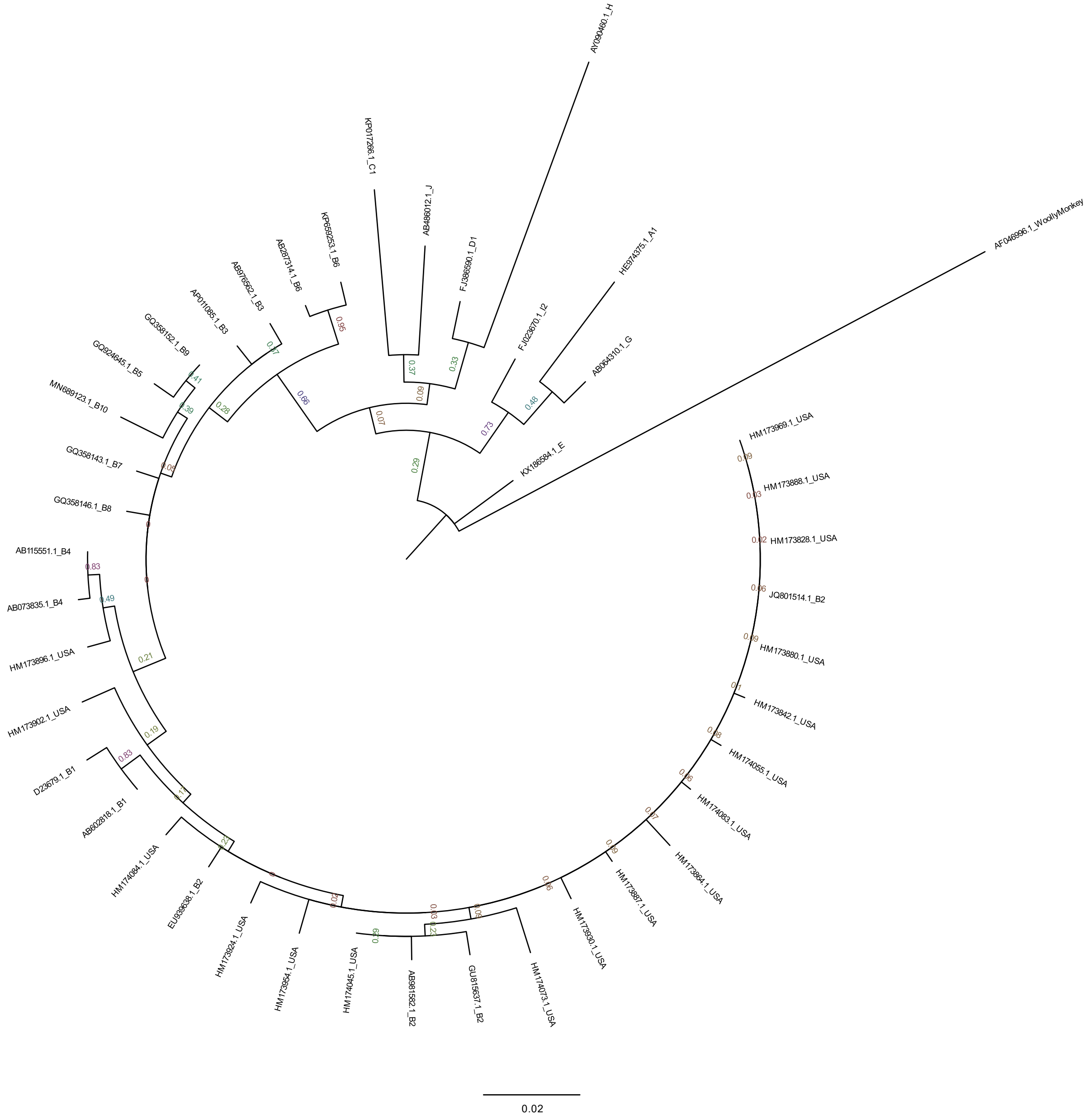

Tree 1. Molecular Phylogenetic analysis by Maximum Likelihood method conducted in MEGA7. The evolutionary history was inferred by using the Maximum Likelihood method based on the Tamura-Nei model with 1000 bootstraps. The tree with the highest log likelihood (-1869.81) is shown. The percentage of trees in which the associated taxa clustered together is shown next to the branches. The tree is drawn to scale, with branch lengths measured in the number of substitutions per site. The analysis involved 43 nucleotide sequences, of which 26 were used as marker sequences to determine the subgenotype of 17 sequences. All positions containing gaps and missing data were eliminated. There was a total of 413 positions in the final dataset.

ID	GENOTYPE	SUBTYPE	COUNTRY	ALIGNMENT[1]	BASE PAIRS
HM173828	B	B2	USA	417-881	465
HM173842	B	B2	USA	417-881	465
HM173864	B	B2	USA	417-881	465
HM173880	B	B2	USA	417-905	489
HM173887	B	B2	USA	417-881	465
HM173888	B	B2	USA	417-881	465
HM173896	B	B4	USA	417-881	465
HM173902	B	B4	USA	417-881	465

HM173924	B	B2	USA	417-881	465
HM173930	B	B2	USA	417-881	465
HM173954	B	B2	USA	417-881	465
HM173969	B	B2	USA	417-866	450
HM174045	B	B2	USA	417-881	465
HM174055	B	B2	USA	417-881	465
HM174073	B	B2	USA	417-881	465
HM174083	B	B2	USA	417-881	465
HM174084	B	B2	USA	417-881	465

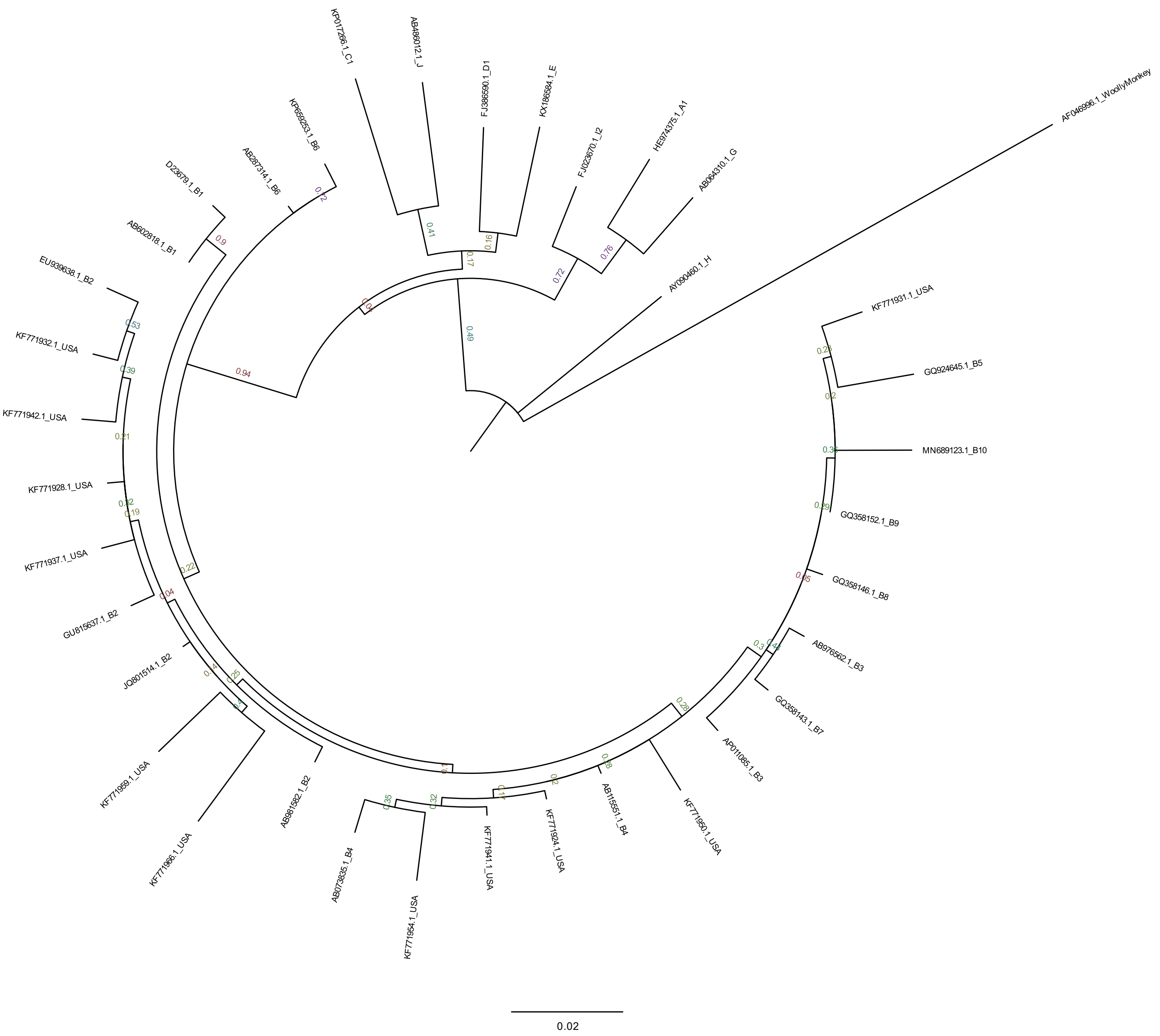

Tree 2. Molecular Phylogenetic analysis by Maximum Likelihood method conducted in MEGA7. The evolutionary history was inferred by using the Maximum Likelihood method based on the Tamura-Nei model with 1000 bootstraps. The tree with the highest log likelihood (-2947.58) is shown. The percentage of trees in which the associated taxa clustered together is shown next to the branches. The tree is drawn to scale, with branch lengths measured in the number of substitutions per site. The analysis involved 37 nucleotide sequences, of which 26 were used as marker sequences to determine the subgenotype of 11 sequences. All positions containing gaps and missing data were eliminated. There was a total of 659 positions in the final dataset.

ID	GENOTYPE	SUBTYPE	COUNTRY	ALIGNMENT[1]	BASE PAIRS
KF771928	B	B2	USA	157-837	681
KF771932	B	B2	USA	157-837	681
KF771937	B	B2	USA	157-837	681
KF771942	B	B2	USA	157-837	681
KF771959	B	B2	USA	157-837	681
KF771966	B	B2	USA	157-837	681
KF771924	B	B4	USA	157-837	681
KF771941	B	B4	USA	157-837	681
KF771950	B	B4	USA	157-837	681

KF771954	B	B4	USA	157-837	681
KF771931	B	B5	USA	157-837	681

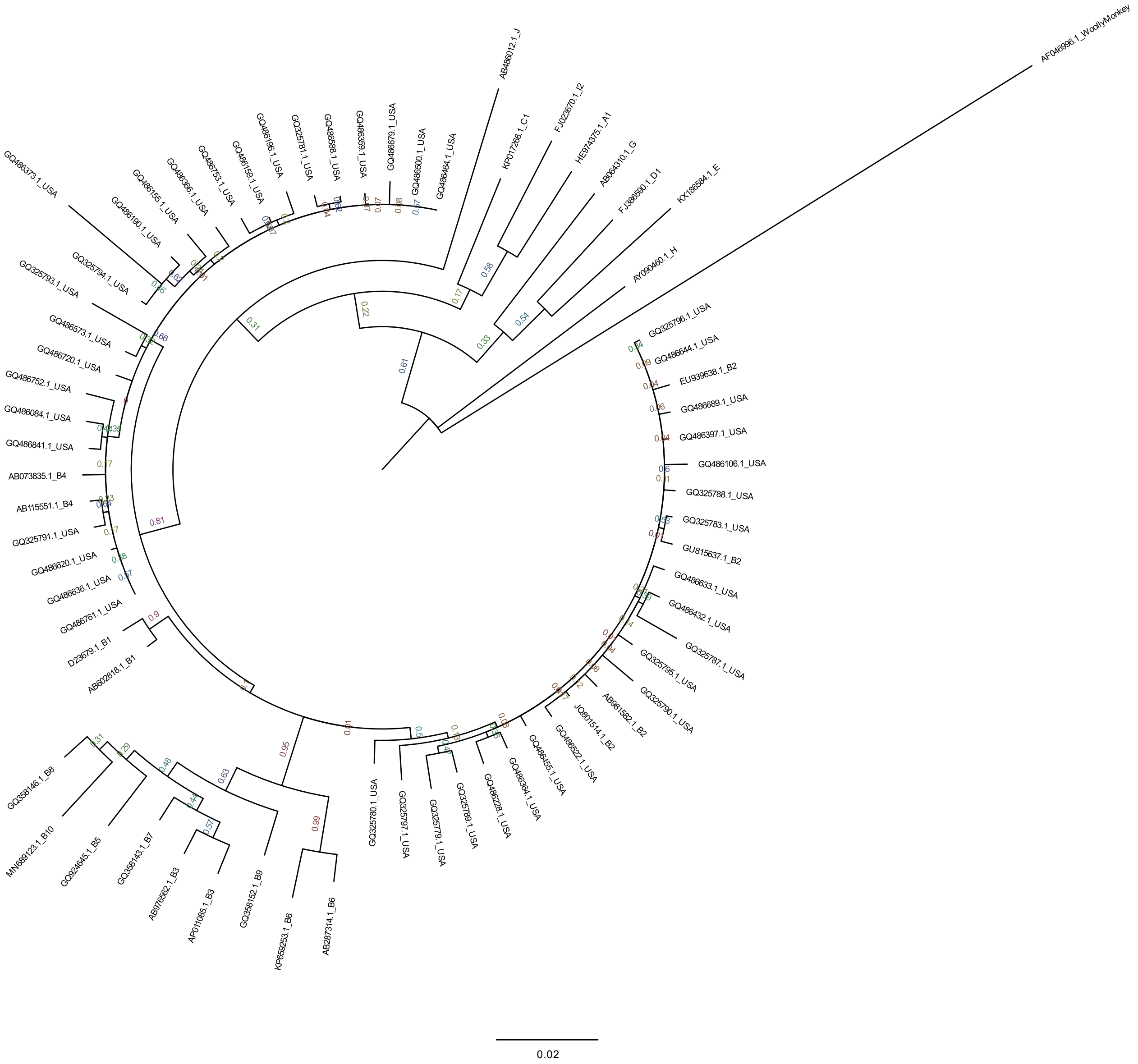

Tree 3. Molecular Phylogenetic analysis by Maximum Likelihood method conducted in MEGA7. The evolutionary history was inferred by using the Maximum Likelihood method based on the Tamura-Nei model with 1000 bootstraps. The tree with the highest log likelihood (-5255.34) is shown. The percentage of trees in which the associated taxa clustered together is shown next to the branches. The tree is drawn to scale, with branch lengths measured in the number of substitutions per site. The analysis involved 70 nucleotide sequences, of which 26 were used as marker sequences to determine the subgenotype of 44 sequences. All positions containing gaps and missing data were eliminated. There was a total of 891 positions in the final dataset.

ID	GENOTYPE	SUBTYPE	COUNTRY	ALIGNMENT[1]	BASE PAIRS
GQ325779	B	B2	USA	132-1163	1032
GQ325780	B	B2	USA	132-1163	1032
GQ325783	B	B2	USA	132-1163	1032
GQ325787	B	B2	USA	132-1163	1032
GQ325788	B	B2	USA	132-1163	1032
GQ325789	B	B2	USA	132-1163	1032

GQ325790	B	B2	USA	132-1163	1032
GQ325795	B	B2	USA	132-1163	1032
GQ325796	B	B2	USA	132-1163	1032
GQ325797	B	B2	USA	132-1163	1032
GQ486106	B	B2	USA	132-1163	1032
GQ486228	B	B2	USA	132-1163	1032
GQ486364	B	B2	USA	132-1163	1032
GQ486397	B	B2	USA	132-1163	1032
GQ486432	B	B2	USA	132-1163	1032
GQ486455	B	B2	USA	132-1163	1032
GQ486522	B	B2	USA	132-1163	1032
GQ486633	B	B2	USA	132-1163	1032
GQ486644	B	B2	USA	132-1163	1032
GQ486689	B	B2	USA	132-1163	1032
GQ325781	B	B4	USA	132-1163	1032
GQ325791	B	B4	USA	132-1163	1032
GQ325793	B	B4	USA	132-1163	1032
GQ325794	B	B4	USA	132-1163	1032
GQ486084	B	B4	USA	132-1163	1032
GQ486155	B	B4	USA	132-1163	1032
GQ486159	B	B4	USA	132-1163	1032
GQ486190	B	B4	USA	132-1163	1032
GQ486196	B	B4	USA	132-1163	1032
GQ486359	B	B4	USA	132-1163	1032
GQ486366	B	B4	USA	132-1163	1032
GQ486373	B	B4	USA	132-1163	1032
GQ486464	B	B4	USA	132-1163	1032
GQ486500	B	B4	USA	132-1163	1032
GQ486573	B	B4	USA	132-1163	1032
GQ486588	B	B4	USA	132-1163	1032
GQ486620	B	B4	USA	132-1163	1032
GQ486636	B	B4	USA	132-1163	1032
GQ486679	B	B4	USA	132-1163	1032
GQ486720	B	B4	USA	132-1163	1032
GQ486752	B	B4	USA	132-1163	1032
GQ486753	B	B4	USA	132-1163	1032
GQ486761	B	B4	USA	132-1163	1032
GQ486841	B	B4	USA	132-1163	1032

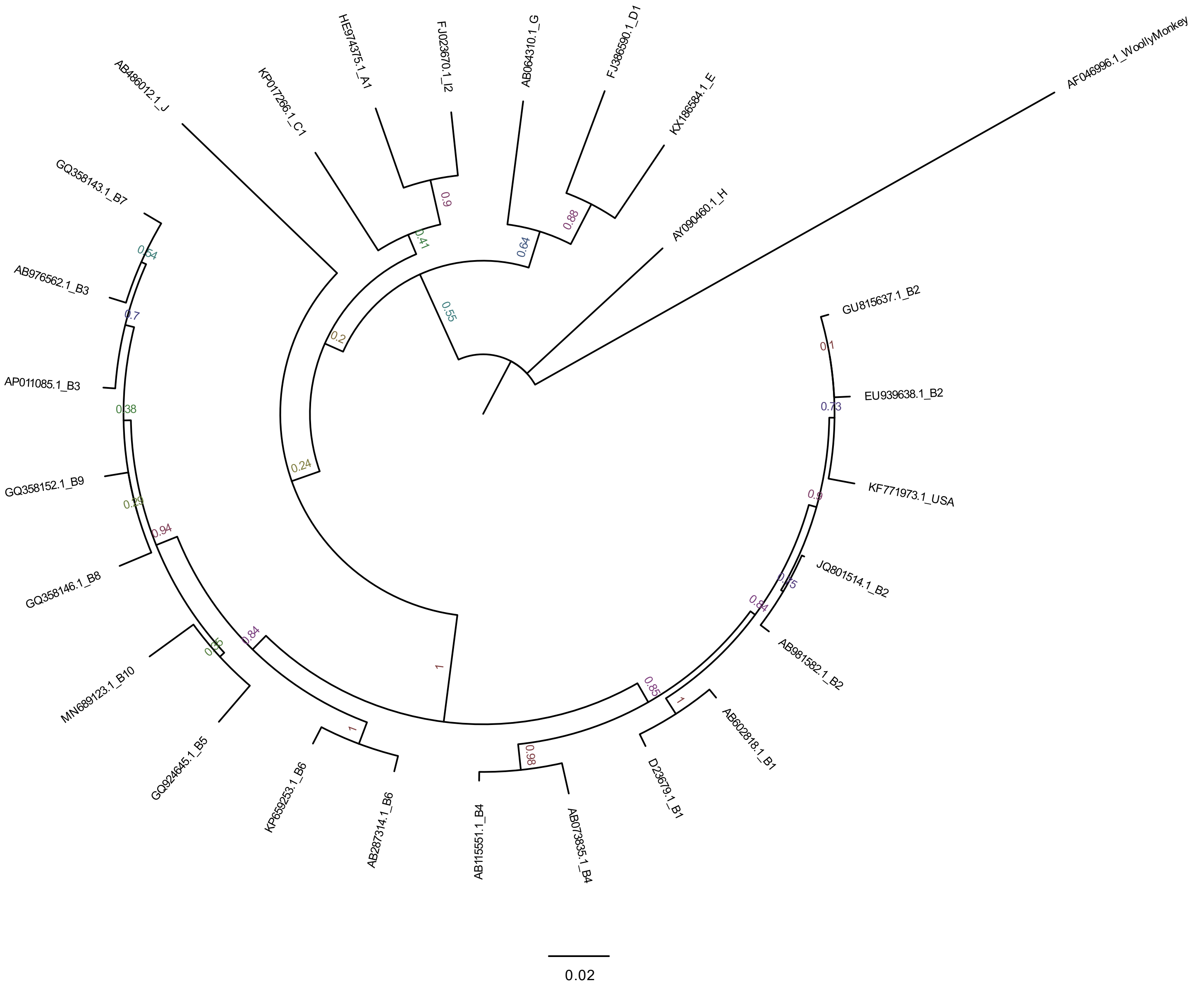

Tree 4. Molecular Phylogenetic analysis by Maximum Likelihood method conducted in MEGA7. The evolutionary history was inferred by using the Maximum Likelihood method based on the Tamura-Nei model with 1000 bootstraps. The tree with the highest log likelihood (-6364.61) is shown. The percentage of trees in which the associated taxa clustered together is shown next to the branches. The tree is drawn to scale, with branch lengths measured in the number of substitutions per site. The analysis involved 27 nucleotide sequences, of which 26 were used as marker sequences to determine the subgenotype of 1 sequence. All positions containing gaps and missing data were eliminated. There was a total of 1146 positions in the final dataset.

ID	GENOTYPE	SUBTYPE	COUNTRY	ALIGNMENT[1]	BASE PAIRS
KF771973	B	B2	USA	1-3182	1203

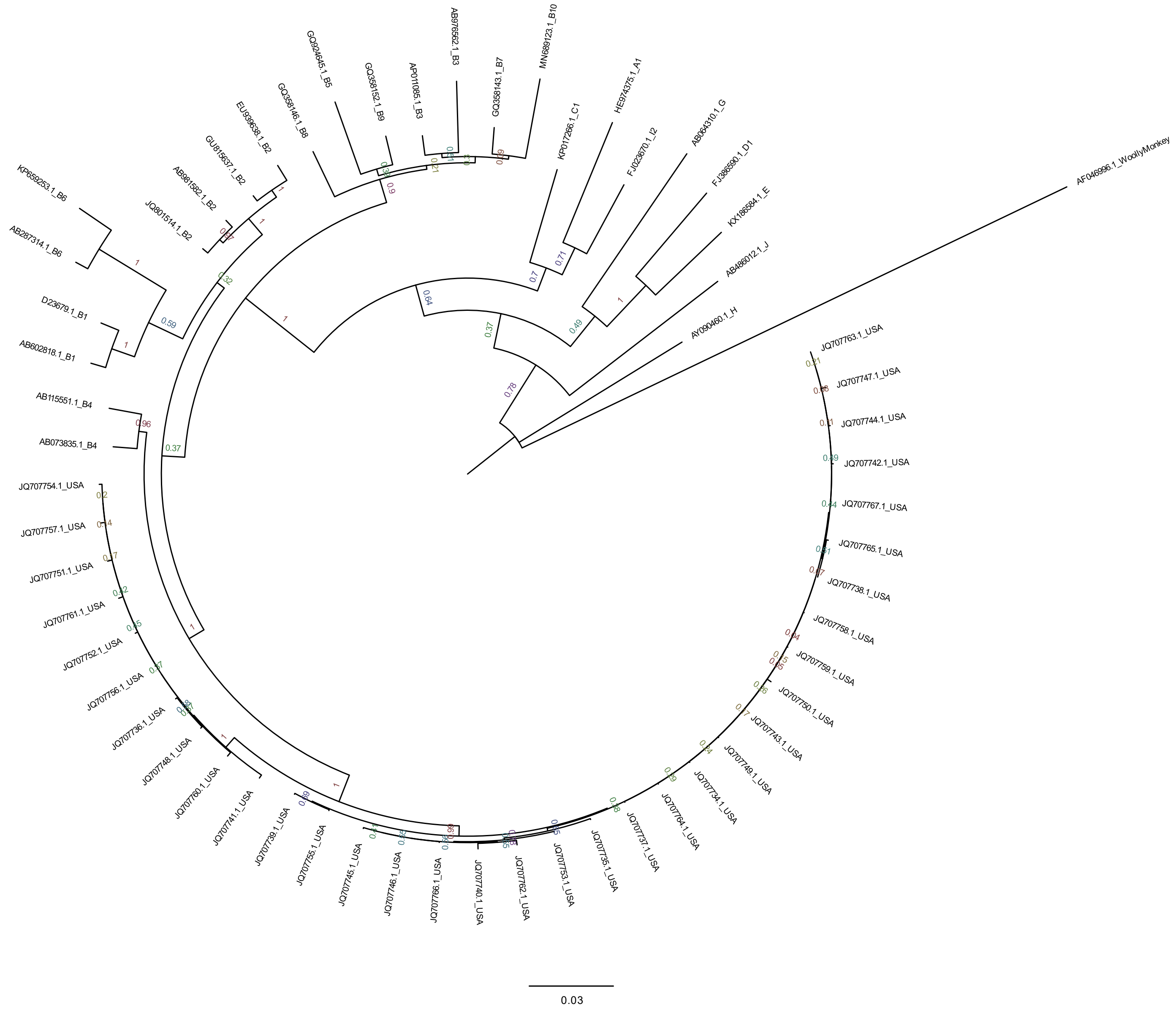

Tree 5. Molecular Phylogenetic analysis by Maximum Likelihood method conducted in MEGA7. The evolutionary history was inferred by using the Maximum Likelihood method based on the Tamura-Nei model with 1000 bootstraps. The tree with the highest log likelihood (-20595.98) is shown. The percentage of trees in which the associated taxa clustered together is shown next to the branches. The tree is drawn to scale, with branch lengths measured in the number of substitutions per site. The analysis involved 60 nucleotide sequences, of which 26 were used as marker sequences to determine the subgenotype of 34 sequences. All positions containing gaps and missing data were eliminated. There was a total of 3055 positions in the final dataset.

ID	GENOTYPE	SUBTYPE	COUNTRY	ALIGNMENT[1]	BASE PAIRS
JQ707734	B	B4	USA	Complete Genome	3215
JQ707735	B	B4	USA	Complete Genome	3215
JQ707736	B	B4	USA	Complete Genome	3173
JQ707737	B	B4	USA	Complete Genome	3215
JQ707738	B	B4	USA	Complete Genome	3215
JQ707739	B	B4	USA	Complete Genome	3215
JQ707740	B	B4	USA	Complete Genome	3215
JQ707741	B	B4	USA	Complete Genome	3173
JQ707742	B	B4	USA	Complete Genome	3215

JQ707743	B	B4	USA	Complete Genome	3215
JQ707744	B	B4	USA	Complete Genome	3215
JQ707745	B	B4	USA	Complete Genome	3215
JQ707746	B	B4	USA	Complete Genome	3215
JQ707747	B	B4	USA	Complete Genome	3215
JQ707748	B	B4	USA	Complete Genome	3173
JQ707749	B	B4	USA	Complete Genome	3215
JQ707750	B	B4	USA	Complete Genome	3215
JQ707751	B	B4	USA	Complete Genome	3173
JQ707752	B	B4	USA	Complete Genome	3173
JQ707753	B	B4	USA	Complete Genome	3215
JQ707754	B	B4	USA	Complete Genome	3173
JQ707755	B	B4	USA	Complete Genome	3215
JQ707756	B	B4	USA	Complete Genome	3173
JQ707757	B	B4	USA	Complete Genome	3173
JQ707758	B	B4	USA	Complete Genome	3215
JQ707759	B	B4	USA	Complete Genome	3215
JQ707760	B	B4	USA	Complete Genome	3173
JQ707761	B	B4	USA	Complete Genome	3173
JQ707762	B	B4	USA	Complete Genome	3215
JQ707763	B	B4	USA	Complete Genome	3215
JQ707764	B	B4	USA	Complete Genome	3215
JQ707765	B	B4	USA	Complete Genome	3215
JQ707766	B	B4	USA	Complete Genome	3215
JQ707767	B	B4	USA	Complete Genome	3215

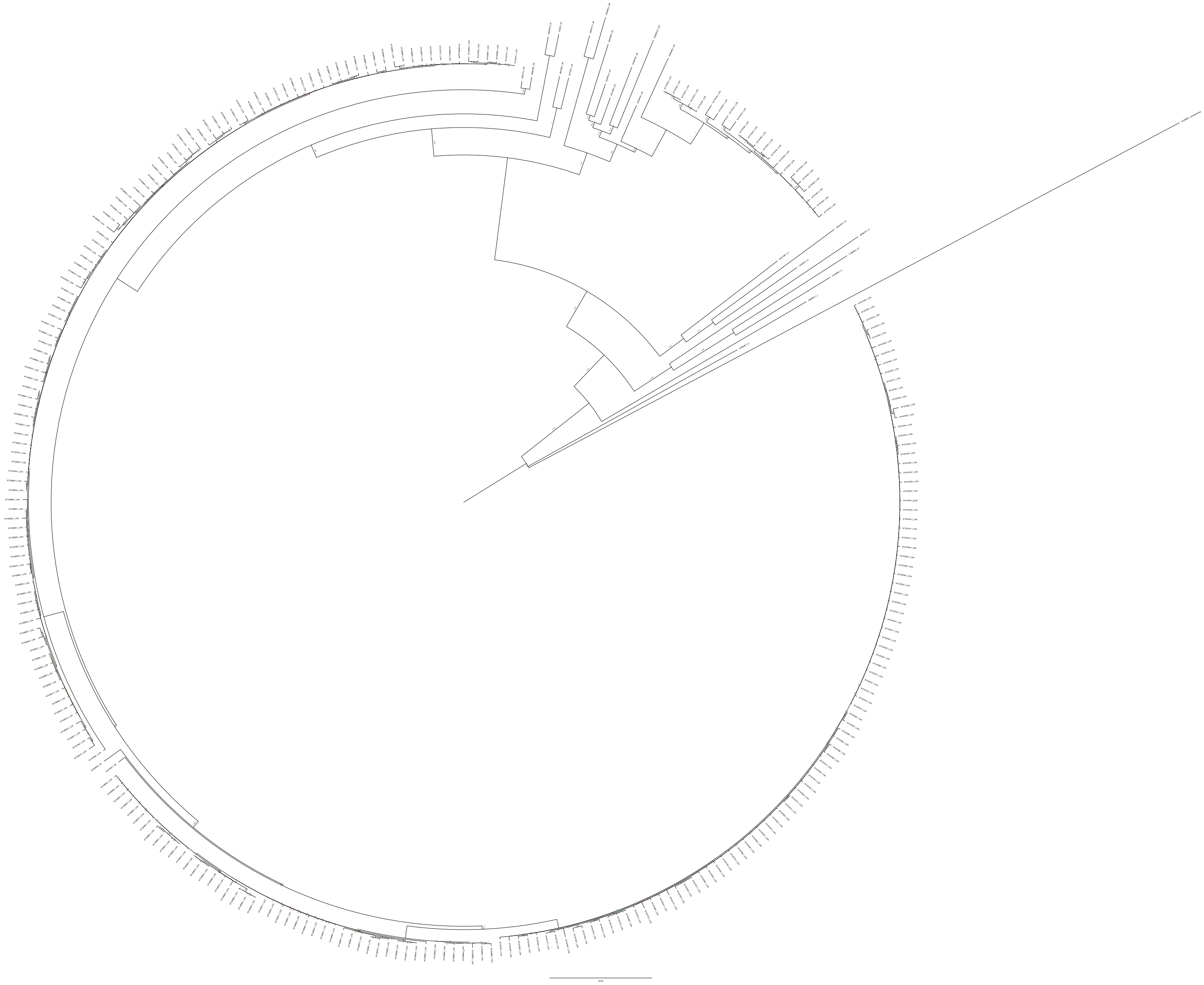

Tree 6. Molecular Phylogenetic analysis by Maximum Likelihood method conducted in MEGA7. The evolutionary history was inferred by using the Maximum Likelihood method based on the Tamura-Nei model with 1000 bootstraps. The tree with the highest log likelihood (-25254.65) is shown. The percentage of trees in which the associated taxa clustered together is shown next to the branches. The tree is drawn to scale, with branch lengths measured in the number of substitutions per site. The analysis involved 299 nucleotide sequences, of which 26 were used as marker sequences to determine the subgenotype of 273 sequences. All positions containing gaps and missing data were eliminated. There was a total of 3040 positions in the final dataset.

ID	GENOTYPE	SUBTYPE	COUNTRY	ALIGNMENT[1]	BASE PAIRS
MT426859	B	B2	USA	1-3182	3215
MT426860	B	B2	USA	1-3182	3215
MT426861	B	B2	USA	1-3182	3215
MT426862	B	B2	USA	1-3182	3215
MT426863	B	B2	USA	1-3182	3215
MT426864	B	B2	USA	1-3182	3215
MT426865	B	B2	USA	1-3182	3215
MT426866	B	B2	USA	1-3182	3215
MT426867	B	B2	USA	1-3182	3215

MT426868	B	B2	USA	1-3182	3215
MT426869	B	B2	USA	1-3182	3215
MT426870	B	B2	USA	1-3182	3215
MT426871	B	B2	USA	1-3182	3215
MT426872	B	B2	USA	1-3182	3215
MT426873	B	B2	USA	1-3182	3215
MT426874	B	B2	USA	1-3182	3215
MT426875	B	B2	USA	1-3182	3215
MT426876	B	B2	USA	1-3182	3215
MT426877	B	B2	USA	1-3182	3215
MT426878	B	B2	USA	1-3182	3215
MT426879	B	B2	USA	1-3182	3215
MT426880	B	B2	USA	1-3182	3215
MT426881	B	B2	USA	1-3182	3215
MT426882	B	B2	USA	1-3182	3215
MT426883	B	B2	USA	1-3182	3215
MT426884	B	B2	USA	1-3182	3215
MT426885	B	B2	USA	1-3182	3215
MT426886	B	B2	USA	1-3182	3215
MT426887	B	B2	USA	1-3182	3215
MT426888	B	B2	USA	1-3182	3215
MT426889	B	B2	USA	1-3182	3215
MT426890	B	B2	USA	1-3182	3215
MT426891	B	B2	USA	1-3182	3215
MT426892	B	B2	USA	1-3182	3215
MT426893	B	B2	USA	1-3182	3215
MT426894	B	B2	USA	1-3182	3215
MT426895	B	B2	USA	1-3182	3215
MT426896	B	B2	USA	1-3182	3215
MT426897	B	B2	USA	1-3182	3215
MT426898	B	B2	USA	1-3182	3215
MT426899	B	B2	USA	1-3182	3215
MT426900	B	B2	USA	1-3182	3215
MT426901	B	B2	USA	1-3182	3215
MT426902	B	B2	USA	1-3182	3215
MT426903	B	B2	USA	1-3182	3215
MT426904	B	B2	USA	1-3182	3215
MT426905	B	B2	USA	1-3182	3215
MT426906	B	B2	USA	1-3182	3215
MT426907	B	B2	USA	1-3182	3215
MT426908	B	B2	USA	1-3182	3215
MT426909	B	B2	USA	1-3182	3215
MT426910	B	B2	USA	1-3182	3215
MT426911	B	B2	USA	1-3182	3215
MT426912	B	B2	USA	1-3182	3215
MT426913	B	B2	USA	1-3182	3215

MT426914	B	B2	USA	1-3182	3215
MT426915	B	B2	USA	1-3182	3215
MT426916	B	B2	USA	1-3182	3215
MT426917	B	B2	USA	1-3182	3215
MT426918	B	B2	USA	1-3182	3215
MT426919	B	B2	USA	1-3182	3215
MT426920	B	B2	USA	1-3182	3215
MT426921	B	B2	USA	1-3182	3215
MT426922	B	B2	USA	1-3182	3215
MT426923	B	B2	USA	1-3182	3215
MT426924	B	B2	USA	1-3182	3215
MT426925	B	B2	USA	1-3182	3215
MT426926	B	B2	USA	1-3182	3215
MT426927	B	B2	USA	1-3182	3215
MT426928	B	B2	USA	1-3182	3215
MT426929	B	B2	USA	1-3182	3215
MT426930	B	B2	USA	1-3182	3215
MT426931	B	B2	USA	1-3182	3215
MT426932	B	B2	USA	1-3182	3215
MT426933	B	B2	USA	1-3182	3215
MT426934	B	B2	USA	1-3182	3215
MT426935	B	B2	USA	1-3182	3215
MT426936	B	B2	USA	1-3182	3215
MT426937	B	B2	USA	1-3182	3215
MT426938	B	B2	USA	1-3182	3215
MT426939	B	B2	USA	1-3182	3215
MT426940	B	B2	USA	1-3182	3215
MT426941	B	B2	USA	1-3182	3215
MT426942	B	B2	USA	1-3182	3215
MT426943	B	B2	USA	1-3182	3215
MT426944	B	B2	USA	1-3182	3215
MT426945	B	B2	USA	1-3182	3215
MT426946	B	B2	USA	1-3182	3215
MT426947	B	B2	USA	1-3182	3215
MT426948	B	B2	USA	1-3182	3215
MT426949	B	B2	USA	1-3182	3215
MT426950	B	B2	USA	1-3182	3215
MT426951	B	B2	USA	1-3182	3215
MT426952	B	B2	USA	1-3182	3215
MT426953	B	B2	USA	1-3182	3215
MT426954	B	B2	USA	1-3182	3215
MT426955	B	B2	USA	1-3182	3215
MT426956	B	B2	USA	1-3182	3215
MT426957	B	B2	USA	1-3182	3215
MT426958	B	B2	USA	1-3182	3215
MT426959	B	B2	USA	1-3182	3215

MT426960	B	B2	USA	1-3182	3215
MT426961	B	B2	USA	1-3182	3215
MT426962	B	B2	USA	1-3182	3215
MT426963	B	B2	USA	1-3182	3215
MT426964	B	B2	USA	1-3182	3215
MT426965	B	B2	USA	1-3182	3215
MT426966	B	B2	USA	1-3182	3215
MT426967	B	B2	USA	1-3182	3215
MT426968	B	B2	USA	1-3182	3215
MT426969	B	B2	USA	1-3182	3215
MT426970	B	B2	USA	1-3182	3215
MT426971	B	B2	USA	1-3182	3215
MT426972	B	B2	USA	1-3182	3215
MT426973	B	B2	USA	1-3182	3215
MT426974	B	B2	USA	1-3182	3215
MT426975	B	B2	USA	1-3182	3215
MT426976	B	B2	USA	1-3182	3215
MT426977	B	B2	USA	1-3182	3215
MT426978	B	B2	USA	1-3182	3215
MT426979	B	B2	USA	1-3182	3215
MT426980	B	B2	USA	1-3182	3215
MT426981	B	B2	USA	1-3182	3215
MT426982	B	B2	USA	1-3182	3215
MT426983	B	B2	USA	1-3182	3215
MT426984	B	B2	USA	1-3182	3215
MT426985	B	B2	USA	1-3182	3215
MT426986	B	B2	USA	1-3182	3215
MT426987	B	B2	USA	1-3182	3215
MT426988	B	B2	USA	1-3182	3215
MT426989	B	B2	USA	1-3182	3215
MT426990	B	B2	USA	1-3182	3215
MT426991	B	B2	USA	1-3182	3215
MT426992	B	B2	USA	1-3182	3215
MT426993	B	B2	USA	1-3182	3215
MT426994	B	B2	USA	1-3182	3215
MT426995	B	B2	USA	1-3182	3215
MT426996	B	B2	USA	1-3182	3215
MT426997	B	B2	USA	1-3182	3215
MT426998	B	B2	USA	1-3182	3215
MT426999	B	B2	USA	1-3182	3215
MT427000	B	B2	USA	1-3182	3215
MT427001	B	B2	USA	1-3182	3215
MT427002	B	B2	USA	1-3182	3215
MT427003	B	B2	USA	1-3182	3215
MT427004	B	B2	USA	1-3182	3215
MT427005	B	B2	USA	1-3182	3215

MT427006	B	B2	USA	1-3182	3215
MT427007	B	B2	USA	1-3182	3215
MT427008	B	B2	USA	1-3182	3215
MT427009	B	B2	USA	1-3182	3215
MT427010	B	B2	USA	1-3182	3215
MT427011	B	B2	USA	1-3182	3215
MT427012	B	B2	USA	1-3182	3215
MT427013	B	B2	USA	1-3182	3215
MT427014	B	B2	USA	1-3182	3215
MT427015	B	B2	USA	1-3182	3215
MT427016	B	B2	USA	1-3182	3215
MT427017	B	B2	USA	1-3182	3215
MT427018	B	B2	USA	1-3182	3215
MT427019	B	B2	USA	1-3182	3215
MT427020	B	B2	USA	1-3182	3215
MT427021	B	B2	USA	1-3182	3215
MT427022	B	B2	USA	1-3182	3215
MT427023	B	B2	USA	1-3182	3215
MT427024	B	B2	USA	1-3182	3215
MT427025	B	B2	USA	1-3182	3215
MT427026	B	B2	USA	1-3182	3215
MT427027	B	B2	USA	1-3182	3215
MT427028	B	B2	USA	1-3182	3215
MT427029	B	B2	USA	1-3182	3215
MT427030	B	B2	USA	1-3182	3215
MT427031	B	B2	USA	1-3182	3215
MT427032	B	B2	USA	1-3182	3215
MT427033	B	B2	USA	1-3182	3215
MT427034	B	B2	USA	1-3182	3215
MT427035	B	B2	USA	1-3182	3215
MT427036	B	B2	USA	1-3182	3215
MT427037	B	B2	USA	1-3182	3215
MT427038	B	B2	USA	1-3182	3215
MT427039	B	B2	USA	1-3182	3215
MT427040	B	B2	USA	1-3182	3215
MT427041	B	B2	USA	1-3182	3215
MT427042	B	B2	USA	1-3182	3215
MT427043	B	B2	USA	1-3182	3215
MT427044	B	B2	USA	1-3182	3215
MT427045	B	B2	USA	1-3182	3215
MT427046	B	B2	USA	1-3182	3215
MT427047	B	B2	USA	1-3182	3215
MT427048	B	B2	USA	1-3182	3215
MT427049	B	B2	USA	1-3182	3215
MT427050	B	B2	USA	1-3182	3215
MT427051	B	B2	USA	1-3182	3215

MT427052	B	B2	USA	1-3182	3215
MT427053	B	B2	USA	1-3182	3215
MT427054	B	B2	USA	1-3182	3215
MT427055	B	B2	USA	1-3182	3215
MT427056	B	B2	USA	1-3182	3215
MT427057	B	B2	USA	1-3182	3215
MT427058	B	B2	USA	1-3182	3215
MT427059	B	B2	USA	1-3182	3215
MT427060	B	B2	USA	1-3182	3215
MT427061	B	B2	USA	1-3182	3215
MT427062	B	B2	USA	1-3182	3215
MT427063	B	B2	USA	1-3182	3215
MT427064	B	B2	USA	1-3182	3215
MT427065	B	B2	USA	1-3182	3215
MT427066	B	B2	USA	1-3182	3215
MT427067	B	B2	USA	1-3182	3215
MT427068	B	B2	USA	1-3182	3215
MT427069	B	B2	USA	1-3182	3215
MT427070	B	B2	USA	1-3182	3215
MT427071	B	B2	USA	1-3182	3215
MT427072	B	B2	USA	1-3182	3215
MT427073	B	B2	USA	1-3182	3215
MT427074	B	B2	USA	1-3182	3215
MT427075	B	B2	USA	1-3182	3215
MT427076	B	B2	USA	1-3182	3215
MT427077	B	B2	USA	1-3182	3215
MT427078	B	B2	USA	1-3182	3215
MT427079	B	B2	USA	1-3182	3215
MT427080	B	B2	USA	1-3182	3215
MT427081	B	B2	USA	1-3182	3215
MT427082	B	B2	USA	1-3182	3215
MT427083	B	B2	USA	1-3182	3215
MT427084	B	B2	USA	1-3182	3215
MT427085	B	B2	USA	1-3182	3215
MT427086	B	B2	USA	1-3182	3215
MT427087	B	B2	USA	1-3182	3215
MT427088	B	B2	USA	1-3182	3215
MT427089	B	B2	USA	1-3182	3215
MT427090	B	B2	USA	1-3182	3215
MT427091	B	B2	USA	1-3182	3215
MT427092	B	B2	USA	1-3182	3215
MT427093	B	B2	USA	1-3182	3215
MT427094	B	B2	USA	1-3182	3215
MT427095	B	B2	USA	1-3182	3215
MT427096	B	B2	USA	1-3182	3215
MT427097	B	B2	USA	1-3182	3215

MT427098	B	B2	USA	1-3182	3215
MT427099	B	B2	USA	1-3182	3215
MT427100	B	B2	USA	1-3182	3215
MT427101	B	B2	USA	1-3182	3215
MT427102	B	B2	USA	1-3182	3215
MT427103	B	B2	USA	1-3182	3215
MT427104	B	B2	USA	1-3182	3215
MT427105	B	B2	USA	1-3182	3215
MT427106	B	B2	USA	1-3182	3215
MT427107	B	B2	USA	1-3182	3215
MT427108	B	B2	USA	1-3182	3215
MT427109	B	B2	USA	1-3182	3215
MT757436	B	B5	USA	1-3182	3215
MT757437	B	B5	USA	1-3182	3215
MT757438	B	B5	USA	1-3182	3215
MT757439	B	B5	USA	1-3182	3215
MT757440	B	B5	USA	1-3182	3215
MT757441	B	B5	USA	1-3182	3215
MT757442	B	B5	USA	1-3182	3215
MT757443	B	B5	USA	1-3182	3215
MT757444	B	B5	USA	1-3182	3215
MT757445	B	B5	USA	1-3182	3215
MT757446	B	B5	USA	1-3182	3215
MT757447	B	B5	USA	1-3182	3215
MT757448	B	B5	USA	1-3182	3215
MT757449	B	B5	USA	1-3182	3215
MT757450	B	B5	USA	1-3182	3215
MT757451	B	B5	USA	1-3182	3215
MT757452	B	B5	USA	1-3182	3215
MT757453	B	B5	USA	1-3182	3215
MT757454	B	B5	USA	1-3182	3215
MT757455	B	B5	USA	1-3182	3215
MT757456	B	B5	USA	1-3182	3215
MT757457	B	B5	USA	1-3182	3215

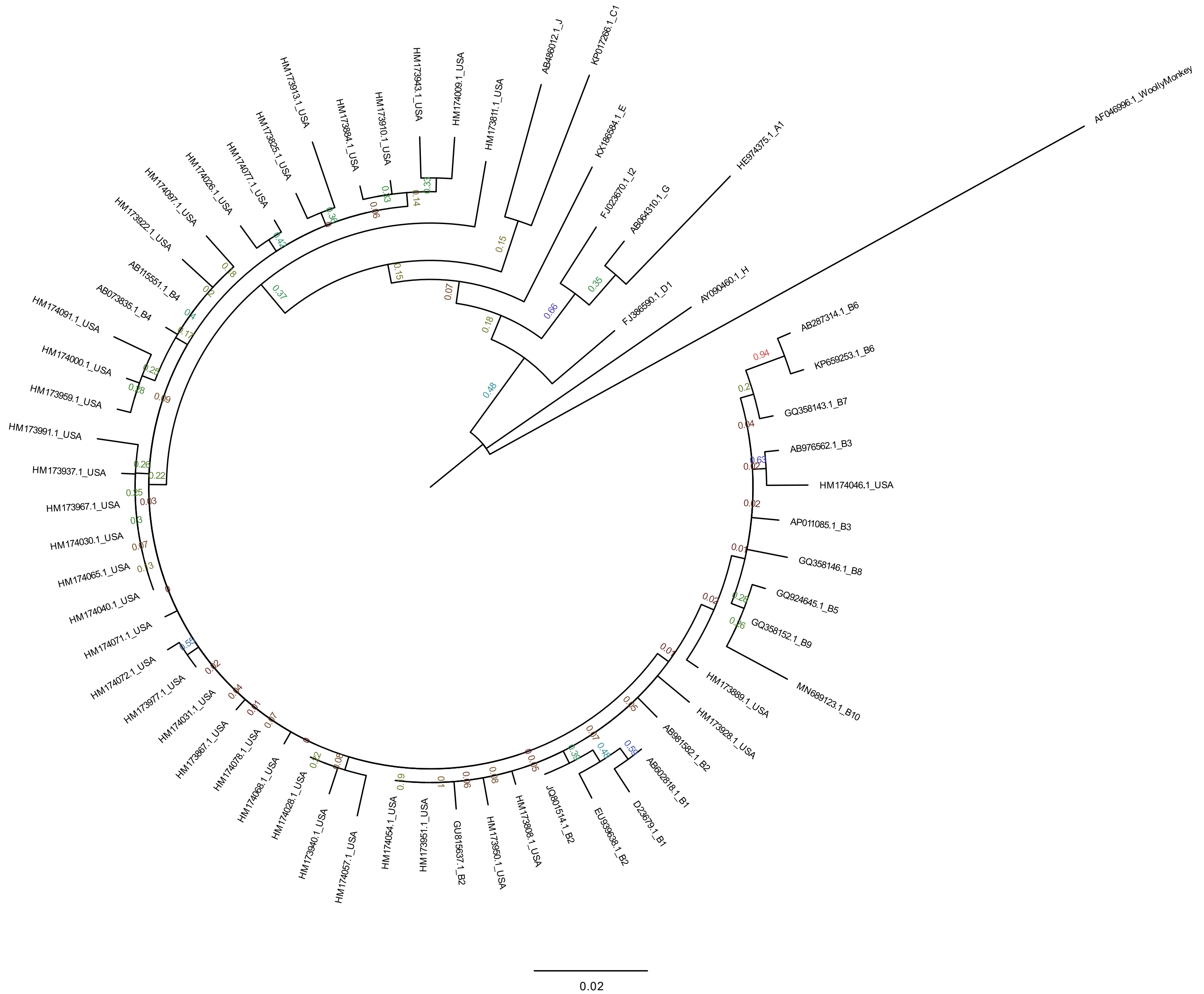

Tree 7. Molecular Phylogenetic analysis by Maximum Likelihood method conducted in MEGA7. The evolutionary history was inferred by using the Maximum Likelihood method based on the Tamura-Nei model with 1000 bootstraps. The tree with the highest log likelihood (-2066.48) is shown. The percentage of trees in which the associated taxa clustered together is shown next to the branches. The tree is drawn to scale, with branch lengths measured in the number of substitutions per site. The analysis involved 63 nucleotide sequences, of which 26 were used as marker sequences to determine the subgenotype of 37 sequences. All positions containing gaps and missing data were eliminated. There was a total of 414 positions in the final dataset.

ID	GENOTYPE	SUBTYPE	COUNTRY	ALIGNMENT[1]	BASE PAIRS
HM173808	B	B2	USA	417-881	465
HM173889	B	B2	USA	417-881	465
HM173928	B	B2	USA	417-881	465
HM173950	B	B2	USA	417-881	465
HM173951	B	B2	USA	417-881	465
HM174054	B	B2	USA	417-881	465
HM174046	B	B3	USA	417-881	465
HM173811	B	B4	USA	417-881	465
HM173825	B	B4	USA	417-881	465

HM173867	B	B4	USA	417-881	465
HM173884	B	B4	USA	417-881	465
HM173910	B	B4	USA	417-881	465
HM173913	B	B4	USA	417-881	465
HM173922	B	B4	USA	417-881	465
HM173937	B	B4	USA	417-881	465
HM173940	B	B4	USA	417-881	465
HM173943	B	B4	USA	417-881	465
HM173959	B	B4	USA	417-881	465
HM173967	B	B4	USA	417-881	465
HM173977	B	B4	USA	417-881	465
HM173991	B	B4	USA	417-881	465
HM174000	B	B4	USA	417-881	465
HM174009	B	B4	USA	417-881	465
HM174026	B	B4	USA	417-881	465
HM174028	B	B4	USA	417-881	465
HM174030	B	B4	USA	417-881	465
HM174031	B	B4	USA	417-881	465
HM174040	B	B4	USA	417-881	465
HM174057	B	B4	USA	417-881	465
HM174065	B	B4	USA	417-881	465
HM174068	B	B4	USA	417-881	465
HM174071	B	B4	USA	417-881	465
HM174072	B	B4	USA	417-881	465
HM174077	B	B4	USA	417-881	465
HM174078	B	B4	USA	417-881	465
HM174091	B	B4	USA	417-881	465
HM174097	B	B4	USA	417-881	465

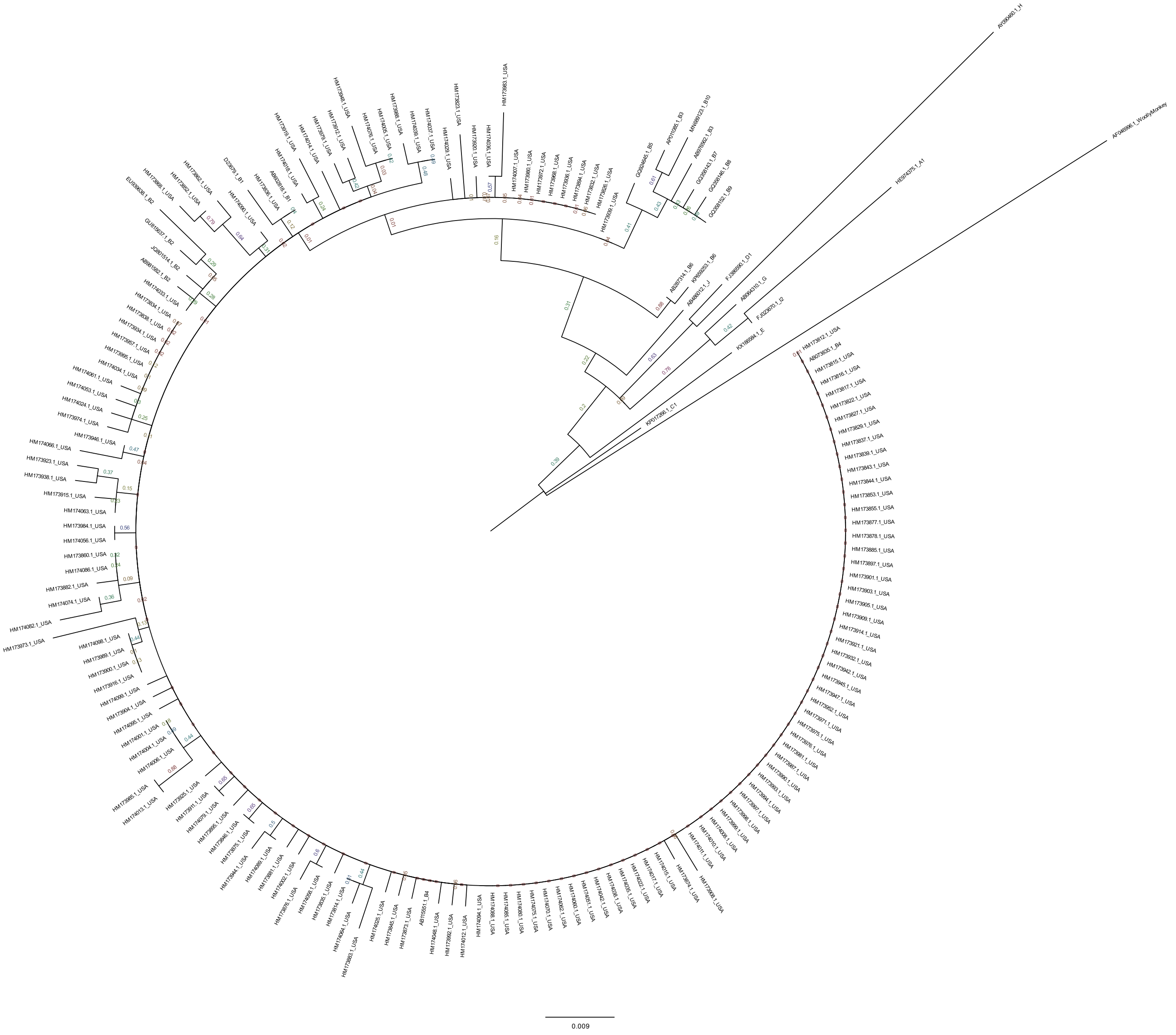

Tree 8. Molecular Phylogenetic analysis by Maximum Likelihood method conducted in MEGA7. The evolutionary history was inferred by using the Maximum Likelihood method based on the Tamura-Nei model with 1000 bootstraps. The tree with the highest log likelihood (-1878.24) is shown. The percentage of trees in which the associated taxa clustered together is shown next to the branches. The tree is drawn to scale, with branch lengths measured in the number of substitutions per site. The analysis involved 174 nucleotide sequences, of which 26 were used as marker sequences to determine the subgenotype of 148 sequences. All positions containing gaps and missing data were eliminated. There was a total of 361 positions in the final dataset.

ID	GENOTYPE	SUBTYPE	COUNTRY	ALIGNMENT[1]	BASE PAIRS
HM173812	B	B4	USA	417-881	465
HM173814	B	B4	USA	417-881	465
HM173815	B	B4	USA	417-881	465
HM173816	B	B4	USA	417-881	465
HM173817	B	B4	USA	417-881	465
HM173822	B	B4	USA	417-881	465

HM173823	B	B4	USA	417-881	465
HM173826	B	B4	USA	417-881	465
HM173827	B	B4	USA	417-881	465
HM173829	B	B4	USA	417-881	465
HM173832	B	B4	USA	417-866	450
HM173834	B	B4	USA	417-881	465
HM173835	B	B4	USA	417-881	465
HM173836	B	B1	USA	417-881	465
HM173837	B	B4	USA	417-881	465
HM173838	B	B4	USA	417-881	465
HM173839	B	B4	USA	417-881	465
HM173843	B	B4	USA	417-881	465
HM173844	B	B4	USA	417-881	465
HM173845	B	B4	USA	417-881	465
HM173846	B	B4	USA	420-881	462
HM173852	B	B4	USA	417-881	465
HM173853	B	B4	USA	417-881	465
HM173855	B	B4	USA	417-881	465
HM173860	B	B4	USA	417-881	465
HM173862	B	B4	USA	417-881	465
HM173868	B	B4	USA	417-881	465
HM173873	B	B4	USA	417-881	465
HM173874	B	B4	USA	417-881	465
HM173875	B	B4	USA	417-881	465
HM173876	B	B4	USA	417-881	465
HM173877	B	B4	USA	417-881	465
HM173878	B	B4	USA	417-881	465
HM173881	B	B4	USA	417-881	465
HM173882	B	B4	USA	417-881	465
HM173883	B	B4	USA	417-881	465
HM173885	B	B4	USA	417-881	465
HM173894	B	B4	USA	417-881	465
HM173895	B	B4	USA	417-881	465
HM173897	B	B4	USA	417-881	465
HM173900	B	B4	USA	417-881	465
HM173901	B	B4	USA	417-881	465
HM173903	B	B4	USA	417-881	465
HM173904	B	B4	USA	417-881	465
HM173905	B	B4	USA	417-881	465
HM173908	B	B4	USA	417-881	465
HM173909	B	B4	USA	417-881	465
HM173911	B	B4	USA	417-881	465
HM173912	B	B4	USA	417-881	465
HM173914	B	B4	USA	417-881	465
HM173915	B	B4	USA	417-881	465
HM173916	B	B4	USA	417-881	465

HM173919	B	B4	USA	417-881	465
HM173920	B	B4	USA	417-881	465
HM173921	B	B4	USA	417-881	465
HM173923	B	B4	USA	417-881	465
HM173925	B	B4	USA	417-881	465
HM173932	B	B4	USA	417-881	465
HM173934	B	B4	USA	417-881	465
HM173936	B	B4	USA	417-881	465
HM173938	B	B4	USA	417-881	465
HM173939	B	B5	USA	417-881	465
HM173942	B	B4	USA	417-881	465
HM173944	B	B4	USA	417-881	465
HM173945	B	B4	USA	417-881	465
HM173946	B	B4	USA	417-881	465
HM173947	B	B4	USA	417-881	465
HM173948	B	B4	USA	417-866	450
HM173952	B	B4	USA	417-881	465
HM173957	B	B4	USA	417-881	465
HM173968	B	B4	USA	417-881	465
HM173971	B	B4	USA	417-881	465
HM173972	B	B4	USA	417-881	465
HM173973	B	B4	USA	417-881	465
HM173974	B	B4	USA	417-881	465
HM173975	B	B4	USA	417-881	465
HM173976	B	B4	USA	417-881	465
HM173979	B	B4	USA	417-881	465
HM173980	B	B4	USA	417-881	465
HM173981	B	B4	USA	417-881	465
HM173983	B	B4	USA	417-881	465
HM173984	B	B4	USA	417-881	465
HM173985	B	B4	USA	417-881	465
HM173987	B	B4	USA	417-866	450
HM173988	B	B4	USA	417-881	465
HM173989	B	B4	USA	417-881	465
HM173990	B	B4	USA	417-881	465
HM173992	B	B4	USA	417-881	465
HM173993	B	B4	USA	417-881	465
HM173994	B	B4	USA	417-881	465
HM173995	B	B4	USA	417-881	465
HM173997	B	B4	USA	417-881	465
HM173998	B	B4	USA	417-881	465
HM173999	B	B4	USA	417-881	465
HM174001	B	B4	USA	417-881	465
HM174002	B	B4	USA	417-881	465
HM174004	B	B4	USA	417-881	465
HM174005	B	B4	USA	417-881	465

HM174006	B	B4	USA	417-881	465
HM174007	B	B4	USA	417-881	465
HM174008	B	B4	USA	417-881	465
HM174010	B	B4	USA	417-881	465
HM174011	B	B4	USA	417-881	465
HM174012	B	B4	USA	417-881	465
HM174013	B	B4	USA	417-881	465
HM174014	B	B4	USA	417-881	465
HM174015	B	B4	USA	417-881	465
HM174017	B	B4	USA	417-881	465
HM174018	B	B4	USA	417-881	465
HM174022	B	B4	USA	417-881	465
HM174024	B	B4	USA	417-881	465
HM174025	B	B4	USA	417-881	465
HM174029	B	B4	USA	417-866	450
HM174033	B	B2	USA	417-881	465
HM174034	B	B4	USA	417-866	450
HM174035	B	B4	USA	417-881	465
HM174036	B	B4	USA	417-881	465
HM174037	B	B4	USA	417-881	465
HM174038	B	B4	USA	417-881	465
HM174039	B	B4	USA	417-881	465
HM174042	B	B4	USA	417-881	465
HM174048	B	B4	USA	417-881	465
HM174051	B	B4	USA	417-881	465
HM174053	B	B4	USA	417-881	465
HM174056	B	B4	USA	417-881	465
HM174058	B	B4	USA	417-881	465
HM174060	B	B4	USA	417-881	465
HM174061	B	B4	USA	417-881	465
HM174062	B	B4	USA	417-881	465
HM174063	B	B4	USA	417-881	465
HM174064	B	B4	USA	417-881	465
HM174066	B	B4	USA	417-881	465
HM174070	B	B4	USA	417-881	465
HM174074	B	B4	USA	417-881	465
HM174075	B	B4	USA	417-881	465
HM174076	B	B4	USA	426-881	456
HM174079	B	B4	USA	417-881	465
HM174080	B	B4	USA	417-881	465
HM174082	B	B4	USA	417-881	465
HM174085	B	B4	USA	417-881	465
HM174086	B	B4	USA	417-881	465
HM174088	B	B4	USA	417-881	465
HM174089	B	B4	USA	417-881	465
HM174090	B	B4	USA	417-881	465

HM174094	B	B4	USA	417-881	465
HM174095	B	B4	USA	417-881	465
HM174098	B	B4	USA	417-881	465
HM174099	B	B4	USA	417-881	465

C – SUBGENOTYPE

ARGENTINA

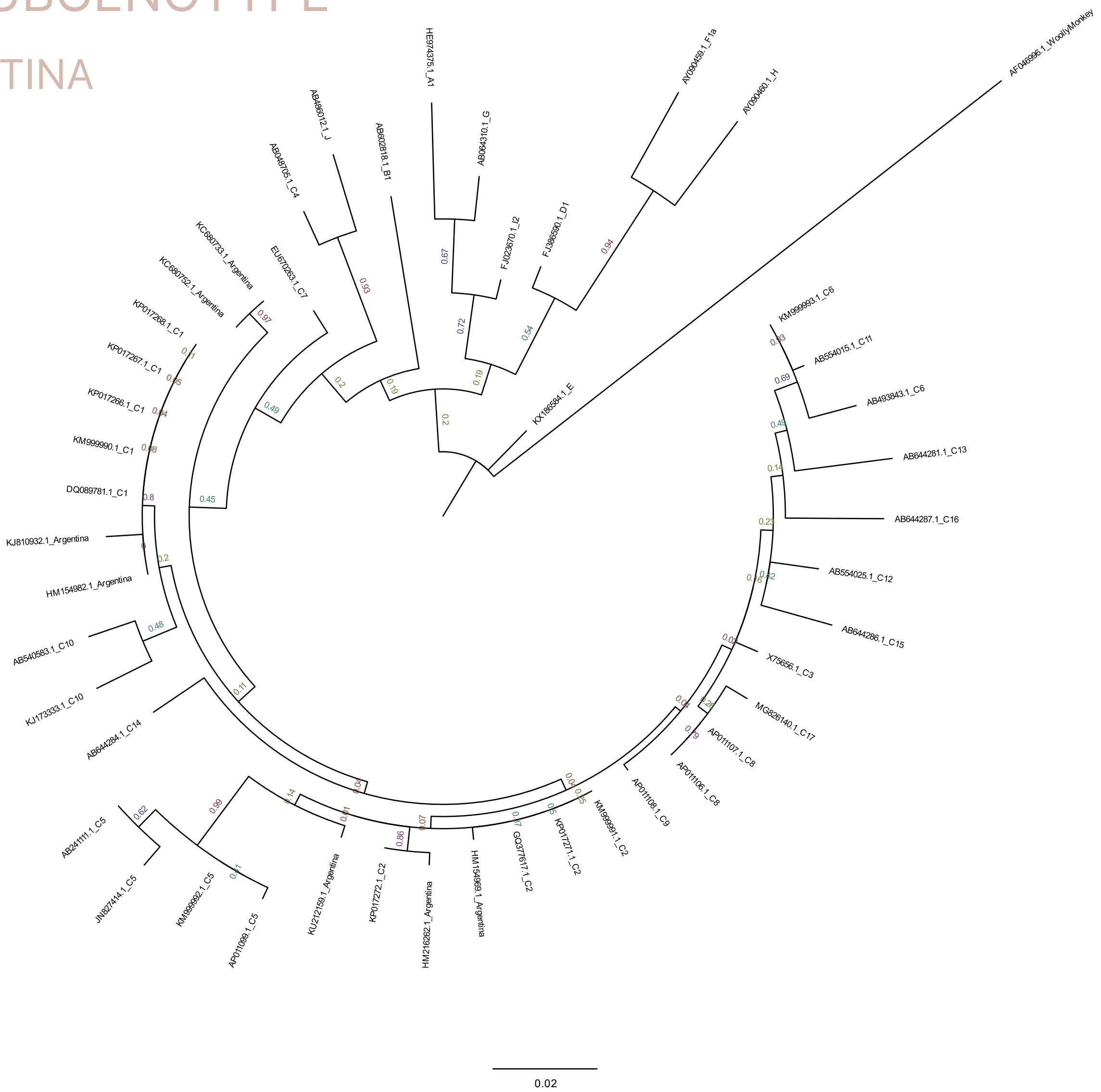

Tree 1. Molecular Phylogenetic analysis by Maximum Likelihood method conducted in MEGA7. The evolutionary history was inferred by using the Maximum Likelihood method based on the Tamura-Nei model with 1000 bootstraps. The tree with the highest log likelihood (-2108.27) is shown. The percentage of trees in which the associated taxa clustered together is shown next to the branches. The tree is drawn to scale, with branch lengths measured in the number of substitutions per site. The analysis involved 47 nucleotide sequences, of which 40 were used as marker sequences to determine the genotype of 7 sequences. All positions containing gaps and missing data were eliminated. There was a total of 430 positions in the final dataset.

ID	GENOTYPE	SUBTYPE	COUNTRY	ALIGNMENT[1]	BASE PAIRS
HM154969	C	C2	Argentina	244-747	504
HM154982	C	C1	Argentina	244-747	504
HM216262	C	C2	Argentina	259-766	508
KC680733	C	C10	Argentina	244-768	525
KC680752	C	C10	Argentina	244-768	525
KJ810932	C	C1	Argentina	313-805	493
KU212159	C	C5	Argentina	244-768	525

BRAZIL

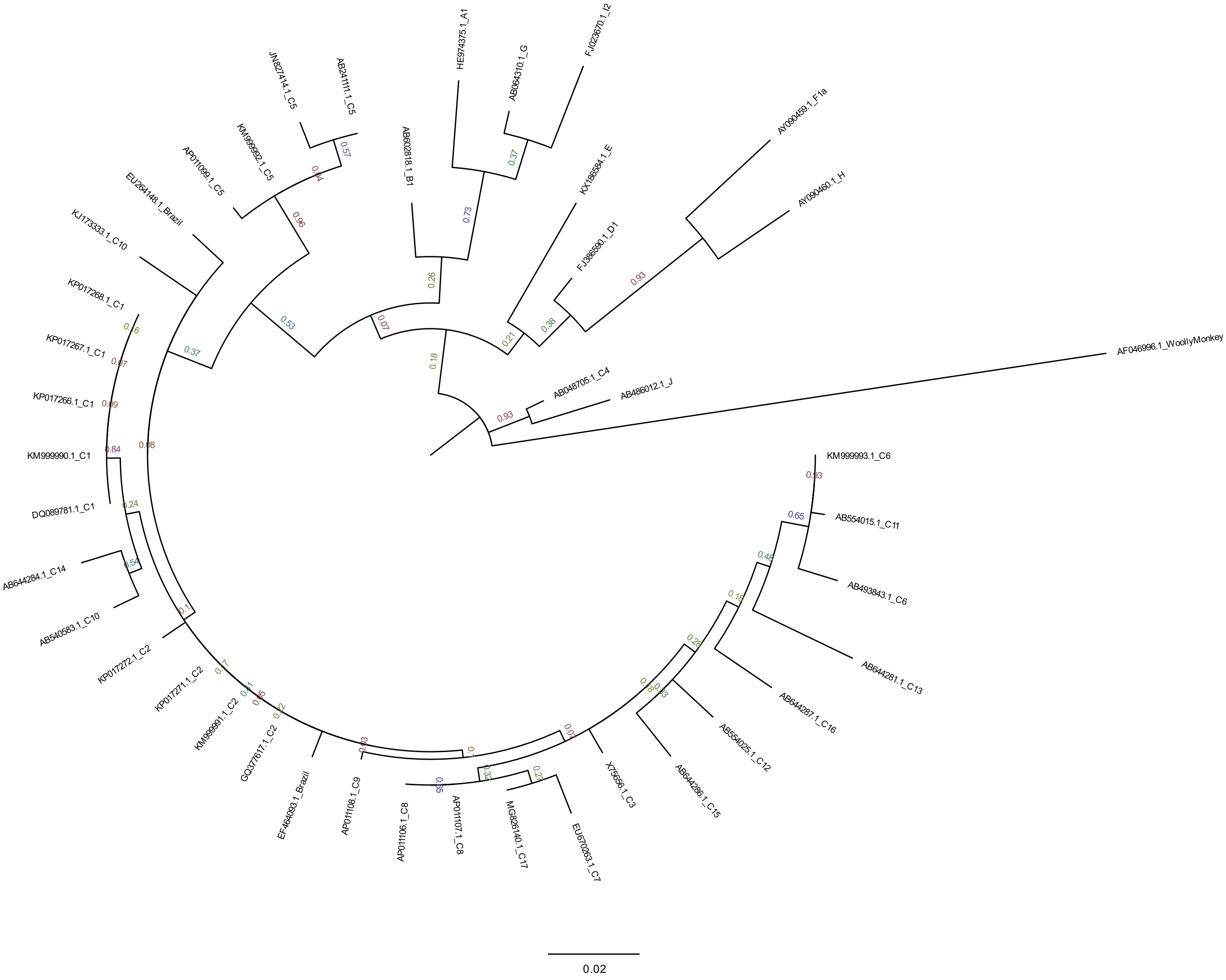

Tree 1. Molecular Phylogenetic analysis by Maximum Likelihood method conducted in MEGA7. The evolutionary history was inferred by using the Maximum Likelihood method based on the Tamura-Nei model with 1000 bootstraps. The tree with the highest log likelihood (-1677.17) is shown. The percentage of trees in which the associated taxa clustered together is shown next to the branches. The tree is drawn to scale, with branch lengths measured in the number of substitutions per site. The analysis involved 42 nucleotide sequences, of which 40 were used as marker sequences to determine the genotype of 2 sequences. All positions containing gaps and missing data were eliminated. There was a total of 333 positions in the final dataset.

ID	GENOTYPE	SUBTYPE	COUNTRY	ALIGNMENT[1]	BASE PAIRS
EF464093	C	C2	Brazil	417-841	425
EU264148	C	C5	Brazil	172-753	582

CANADA

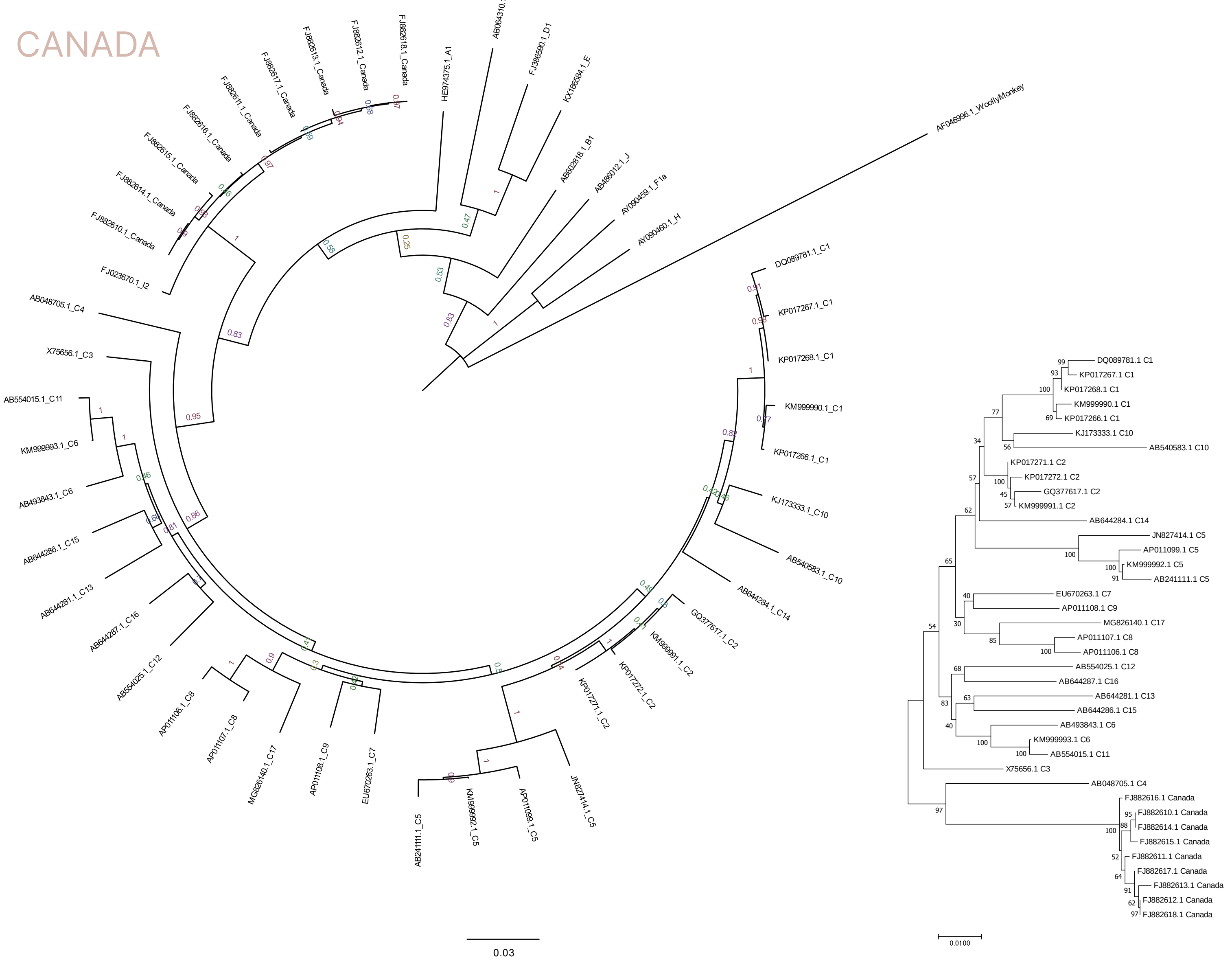

Tree 1. Molecular Phylogenetic analysis by Maximum Likelihood method conducted in MEGA7. The evolutionary history was inferred by using the Maximum Likelihood method based on the Tamura-Nei model with 1000 bootstraps. The tree with the highest log likelihood (-24430.26) is shown. The percentage of trees in which the associated taxa clustered together is shown next to the branches. The tree is drawn to scale, with branch lengths measured in the number of substitutions per site. The analysis involved 49 nucleotide sequences, of which 40 were used as marker sequences to determine the genotype of 9 sequences. All sequences analyzed were reclassified to genotype I. All positions containing gaps and missing data were eliminated. There was a total of 3054 positions in the final dataset. An additional tree was constructed to determine the subgenotype of each of the sequences.

ID	GENOTYPE	SUBTYPE	COUNTRY	ALIGNMENT[1]	BASE PAIRS
FJ882610	I	I2	Canada	Complete Genome	3215
FJ882611	I	I2	Canada	Complete Genome	3215
FJ882612	I	I2	Canada	Complete Genome	3215
FJ882613	I	I2	Canada	Complete Genome	3215
FJ882614	I	I2	Canada	Complete Genome	3215
FJ882615	I	I2	Canada	Complete Genome	3216
FJ882616	I	I2	Canada	Complete Genome	3168
FJ882617	I	I2	Canada	Complete Genome	3215
FJ882618	I	I2	Canada	Complete Genome	3215

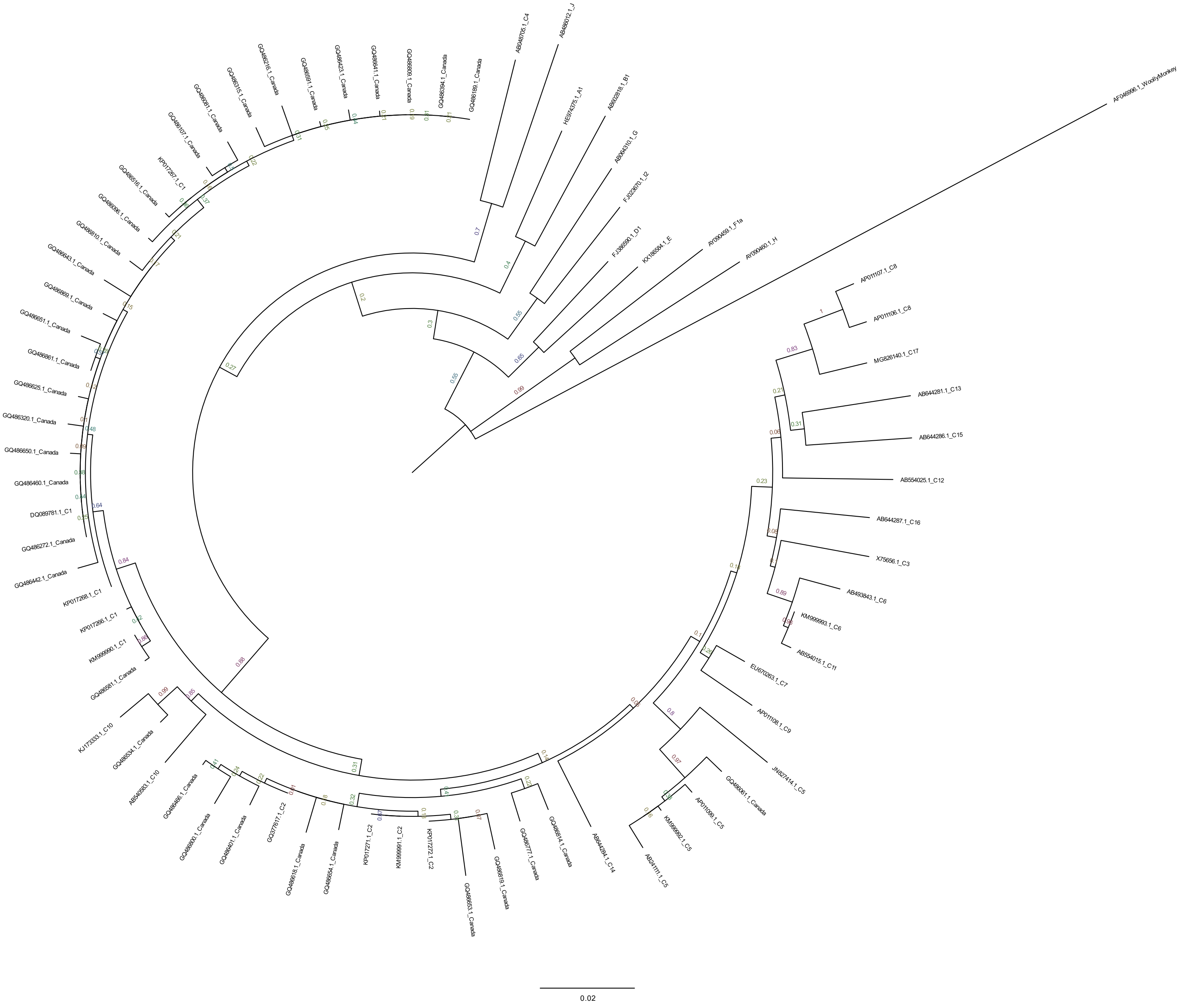

Tree 2. Molecular Phylogenetic analysis by Maximum Likelihood method conducted in MEGA7. The evolutionary history was inferred by using the Maximum Likelihood method based on the Tamura-Nei model with 1000 bootstraps. The tree with the highest log likelihood (-6569.41) is shown. The percentage of trees in which the associated taxa clustered together is shown next to the branches. The tree is drawn to scale, with branch lengths measured in the number of substitutions per site. The analysis involved 75 nucleotide sequences, of which 40 were used as marker sequences to determine the genotype of 35 sequences. All positions containing gaps and missing data were eliminated. There was a total of 910 positions in the final dataset. Additional trees were constructed to determine the subgenotype of each of the sequences.

ID	GENOTYPE	SUBTYPE	COUNTRY	ALIGNMENT[1]	BASE PAIRS
GQ486061	C	C5	Canada	132-1163	1032
GQ486081	C	C1	Canada	132-1163	1032
GQ486096	C	C1	Canada	132-1163	1032
GQ486107	C	C1	Canada	132-1163	1032
GQ486189	C	C1	Canada	132-1163	1032
GQ486216	C	C1	Canada	132-1163	1032

GQ486272	C	C1	Canada	132-1163	1032
GQ486315	C	C1	Canada	132-1163	1032
GQ486320	C	C1	Canada	132-1163	1032
GQ486394	C	C1	Canada	132-1163	1032
GQ486401	C	C2	Canada	132-1163	1032
GQ486423	C	C1	Canada	132-1163	1032
GQ486442	C	C1	Canada	132-1163	1032
GQ486460	C	C1	Canada	132-1163	1032
GQ486466	C	C2	Canada	132-1163	1032
GQ486516	C	C1	Canada	132-1163	1032
GQ486534	C	C10	Canada	132-1163	1032
GQ486581	C	C1	Canada	132-1163	1032
GQ486591	C	C1	Canada	132-1163	1032
GQ486618	C	C2	Canada	132-1163	1032
GQ486625	C	C1	Canada	132-1163	1032
GQ486641	C	C1	Canada	132-1163	1032
GQ486643	C	C1	Canada	132-1163	1032
GQ486650	C	C1	Canada	132-1163	1032
GQ486651	C	C1	Canada	132-1163	1032
GQ486653	C	C1	Canada	132-1163	1032
GQ486654	C	C2	Canada	132-1163	1032
GQ486777	C	C2	Canada	132-1163	1032
GQ486800	C	C2	Canada	132-1163	1032
GQ486809	C	C1	Canada	132-1163	1032
GQ486810	C	C1	Canada	132-1163	1032
GQ486814	C	C2	Canada	132-1163	1032
GQ486819	C	C2	Canada	132-1163	1032
GQ486861	C	C1	Canada	132-1163	1032
GQ486869	C	C1	Canada	132-1163	1032

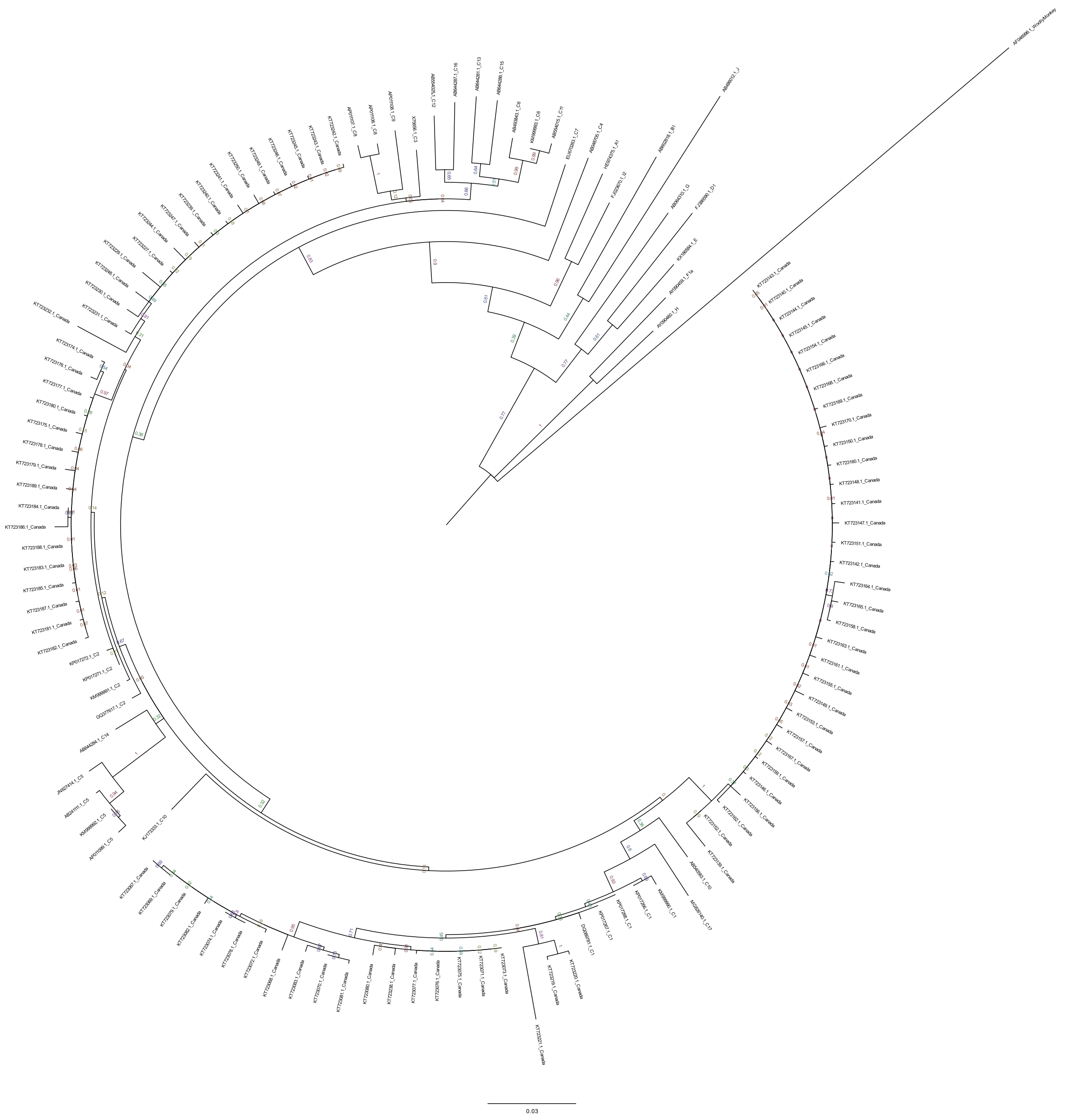

Tree 3. Molecular Phylogenetic analysis by Maximum Likelihood method conducted in MEGA7. The evolutionary history was inferred by using the Maximum Likelihood method based on the Tamura-Nei model with 1000 bootstraps. The tree with the highest log likelihood (-8194.60) is shown. The percentage of trees in which the associated taxa clustered together is shown next to the branches. The tree is drawn to scale, with branch lengths measured in the number of substitutions per site. The analysis involved 126 nucleotide sequences, of which 40 were used as marker sequences to determine the genotype of 86 sequences. All positions containing gaps and missing data were eliminated. There was a total of 896 positions in the final dataset. Additional trees were constructed to determine the subgenotype of each of the sequences.

ID	GENOTYPE	SUBTYPE	COUNTRY	ALIGNMENT[1]	BASE PAIRS
KT723067	C	C1	Canada	1-3182	982
KT723068	C	C1	Canada	1-3182	982
KT723069	C	C1	Canada	1-3182	982
KT723070	C	C1	Canada	1-3182	982
KT723071	C	C1	Canada	1-3182	982
KT723072	C	C1	Canada	1-3182	982
KT723073	C	C1	Canada	1-3182	982

KT723074	C	C1	Canada	1-3182	982
KT723075	C	C1	Canada	1-3182	982
KT723076	C	C1	Canada	1-3182	982
KT723077	C	C1	Canada	1-3182	982
KT723078	C	C1	Canada	1-3182	982
KT723079	C	C1	Canada	1-3182	982
KT723080	C	C1	Canada	1-3182	982
KT723081	C	C1	Canada	1-3182	952
KT723082	C	C1	Canada	1-3182	982
KT723083	C	C1	Canada	1-3182	982
KT723139	C	C1	Canada	1-3182	982
KT723140	C	C1	Canada	1-3182	982
KT723141	C	C1	Canada	1-3182	982
KT723142	C	C1	Canada	1-3182	982
KT723143	C	C1	Canada	1-3182	981
KT723144	C	C1	Canada	1-3182	982
KT723145	C	C1	Canada	1-3182	982
KT723146	C	C1	Canada	1-3182	982
KT723147	C	C1	Canada	1-3182	982
KT723148	C	C1	Canada	1-3182	982
KT723149	C	C1	Canada	1-3182	982
KT723150	C	C1	Canada	1-3182	982
KT723151	C	C1	Canada	1-3182	982
KT723152	C	C1	Canada	1-3182	982
KT723153	C	C1	Canada	1-3182	982
KT723154	C	C1	Canada	1-3182	982
KT723155	C	C1	Canada	1-3182	982
KT723156	C	C1	Canada	1-3182	982
KT723157	C	C1	Canada	1-3182	982
KT723158	C	C1	Canada	1-3182	982
KT723159	C	C1	Canada	1-3182	982
KT723160	C	C1	Canada	1-3182	982
KT723161	C	C1	Canada	1-3182	982
KT723162	C	C1	Canada	1-3182	982
KT723163	C	C1	Canada	1-3182	982
KT723164	C	C1	Canada	1-3182	982
KT723165	C	C1	Canada	1-3182	982
KT723166	C	C1	Canada	1-3182	982
KT723167	C	C1	Canada	1-3182	982
KT723168	C	C1	Canada	1-3182	982
KT723169	C	C1	Canada	1-3182	982
KT723170	C	C1	Canada	1-3182	982
KT723219	C	C1	Canada	1-3182	982
KT723220	C	C1	Canada	1-3182	982
KT723221	C	C1	Canada	1-3182	976
KT723238	C	C1	Canada	1-3182	982

KT723240	C	C2	Canada	1-3182	982
KT723241	C	C2	Canada	1-3182	982
KT723242	C	C2	Canada	1-3182	982
KT723243	C	C2	Canada	1-3182	982
KT723245	C	C2	Canada	1-3182	982
KT723246	C	C2	Canada	1-3182	981
KT723174	C	C2	Canada	1-3182	982
KT723175	C	C2	Canada	1-3182	982
KT723176	C	C2	Canada	1-3182	982
KT723177	C	C2	Canada	1-3182	982
KT723178	C	C2	Canada	1-3182	982
KT723179	C	C2	Canada	1-3182	982
KT723180	C	C2	Canada	1-3182	982
KT723181	C	C2	Canada	1-3182	982
KT723182	C	C2	Canada	1-3182	982
KT723183	C	C2	Canada	1-3182	982
KT723184	C	C2	Canada	1-3182	982
KT723185	C	C2	Canada	1-3182	982
KT723186	C	C2	Canada	1-3182	982
KT723187	C	C2	Canada	1-3182	980
KT723188	C	C2	Canada	1-3182	982
KT723189	C	C2	Canada	1-3182	982
KT723227	C	C2	Canada	1-3182	982
KT723229	C	C2	Canada	1-3182	982
KT723230	C	C2	Canada	1-3182	981
KT723231	C	C2	Canada	1-3182	982
KT723232	C	C2	Canada	1-3182	982
KT723239	C	C2	Canada	1-3182	982
KT723244	C	C2	Canada	1-3182	982
KT723247	C	C2	Canada	1-3182	982
KT723248	C	C2	Canada	1-3182	981
KT723249	C	C2	Canada	1-3182	982
KT723250	C	C2	Canada	1-3182	982

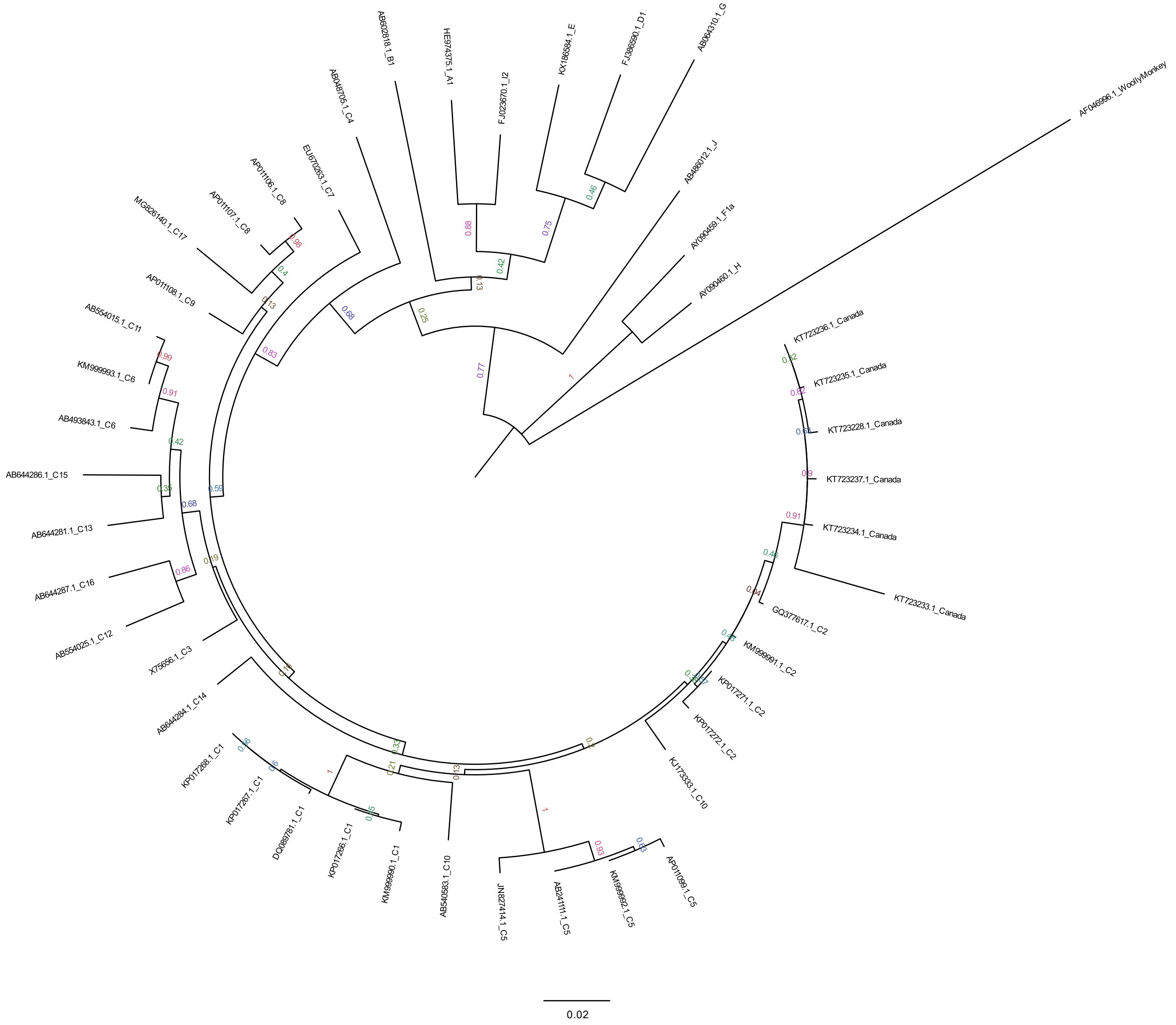

Tree 4. Molecular Phylogenetic analysis by Maximum Likelihood method conducted in MEGA7. The evolutionary history was inferred by using the Maximum Likelihood method based on the Tamura-Nei model with 1000 bootstraps. The tree with the highest log likelihood (-5345.55) is shown. The percentage of trees in which the associated taxa clustered together is shown next to the branches. The tree is drawn to scale, with branch lengths measured in the number of substitutions per site. The analysis involved 46 nucleotide sequences, of which 40 were used as marker sequences to determine the genotype of 6 sequences. All positions containing gaps and missing data were eliminated. There was a total of 754 positions in the final dataset. Additional trees were constructed to determine the subgenotype of each of the sequences.

ID	GENOTYPE	SUBTYPE	COUNTRY	ALIGNMENT[1]	BASE PAIRS
KT723228	C	C2	Canada	1-3182	799
KT723233	C	C2	Canada	1-3182	766
KT723234	C	C2	Canada	1-3182	799
KT723235	C	C2	Canada	1-3182	799
KT723236	C	C2	Canada	1-3182	799
KT723237	C	C2	Canada	1-3182	799

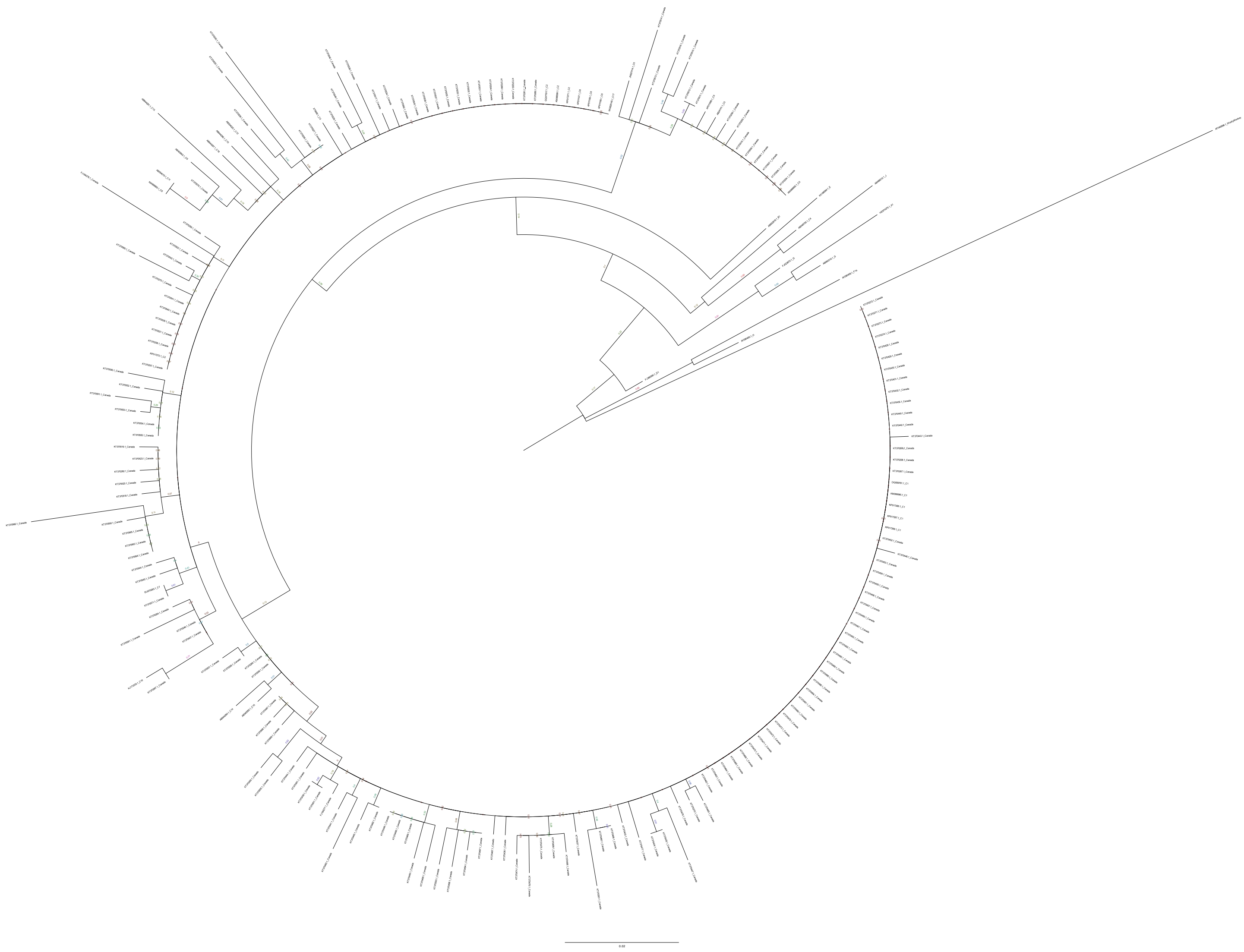

Tree 5. Molecular Phylogenetic analysis by Maximum Likelihood method conducted in MEGA7. The evolutionary history was inferred by using the Maximum Likelihood method based on the Tamura-Nei model with 1000 bootstraps. The tree with the highest log likelihood (-2298.34) is shown. The percentage of trees in which the associated taxa clustered together is shown next to the branches. The tree is drawn to scale, with branch lengths measured in the number of substitutions per site. The analysis involved 205 nucleotide sequences, of which 40 were used as marker sequences to determine the genotype of 165 sequences. All positions containing gaps and missing data were eliminated. There was a total of 306 positions in the final dataset. Additional trees were constructed to determine the subgenotype of each of the sequences.

ID	GENOTYPE	SUBTYPE	COUNTRY	ALIGNMENT[1]	BASE PAIRS
FJ184277	C	C1	Canada	210-788	579
FJ184278	C	C2	Canada	441-884	444
KT370254	C	C5	Canada	313-831	519
KT370255	C	C5	Canada	313-831	519
KT370256	C	C5	Canada	313-831	519
KT370257	C	C2	Canada	313-831	519
KT370258	C	C2	Canada	313-831	519
KT370259	C	C2	Canada	313-831	519
KT370260	C	C2	Canada	313-831	519
KT370261	C	C2	Canada	313-831	519

KT370262	C	C1	Canada	313-831	519
KT370263	C	C2	Canada	313-831	519
KT370264	C	C1	Canada	313-831	519
KT370265	C	C10	Canada	313-831	519
KT370266	C	C2	Canada	313-831	519
KT370267	C	C1	Canada	313-831	519
KT370268	C	C1	Canada	313-831	519
KT370269	C	C1	Canada	313-831	519
KT370270	C	C1	Canada	313-831	519
KT370271	C	C1	Canada	313-831	519
KT370272	C	C1	Canada	313-831	519
KT370273	C	C1	Canada	313-831	519
KT370274	C	C1	Canada	313-831	519
KT370275	C	C2	Canada	313-831	519
KT370428	C	C1	Canada	313-831	519
KT370429	C	C1	Canada	313-831	519
KT370430	C	C1	Canada	313-831	519
KT370431	C	C1	Canada	313-831	519
KT370432	C	C1	Canada	313-831	519
KT370433	C	C1	Canada	313-831	519
KT370434	C	C1	Canada	313-831	519
KT370435	C	C1	Canada	313-831	519
KT370436	C	C1	Canada	313-831	519
KT370437	C	C1	Canada	313-831	519
KT370438	C	C1	Canada	313-831	519
KT370439	C	C1	Canada	313-831	519
KT370440	C	C1	Canada	313-831	519
KT370441	C	C1	Canada	313-831	519
KT370442	C	C1	Canada	313-831	519
KT370443	C	C1	Canada	313-831	519
KT370444	C	C1	Canada	313-831	519
KT370445	C	C1	Canada	313-831	519
KT370446	C	C1	Canada	313-831	519
KT370447	C	C1	Canada	313-831	519
KT370448	C	C1	Canada	313-831	519
KT370449	C	C1	Canada	313-831	519
KT370450	C	C1	Canada	313-831	519
KT370451	C	C1	Canada	313-831	519
KT370452	C	C1	Canada	313-831	519
KT370453	C	C1	Canada	313-831	519
KT370454	C	C1	Canada	313-831	519
KT370455	C	C1	Canada	313-831	519
KT370456	C	C1	Canada	313-831	519
KT370457	C	C1	Canada	313-831	519
KT370458	C	C1	Canada	313-831	519
KT370459	C	C1	Canada	313-831	519

KT370460	C	C1	Canada	313-831	519
KT370461	C	C1	Canada	313-831	519
KT370462	C	C1	Canada	313-831	519
KT370463	C	C1	Canada	313-831	519
KT370464	C	C1	Canada	313-831	519
KT370465	C	C1	Canada	313-831	519
KT370466	C	C1	Canada	313-831	519
KT370467	C	C1	Canada	313-831	519
KT370468	C	C1	Canada	313-831	519
KT370469	C	C1	Canada	313-831	519
KT370470	C	C1	Canada	313-831	519
KT370471	C	C1	Canada	313-831	519
KT370472	C	C1	Canada	313-831	519
KT370473	C	C1	Canada	313-831	519
KT370474	C	C1	Canada	317-831	515
KT370475	C	C1	Canada	313-831	519
KT370476	C	C1	Canada	313-831	519
KT370477	C	C1	Canada	313-831	519
KT370478	C	C1	Canada	329-831	503
KT370479	C	C1	Canada	313-831	519
KT370480	C	C1	Canada	313-831	519
KT370481	C	C1	Canada	313-831	519
KT370482	C	C1	Canada	313-831	519
KT370483	C	C1	Canada	313-831	519
KT370484	C	C1	Canada	313-831	519
KT370485	C	C1	Canada	313-831	519
KT370486	C	C1	Canada	313-831	519
KT370487	C	C1	Canada	313-831	519
KT370488	C	C1	Canada	313-831	519
KT370489	C	C1	Canada	313-831	519
KT370490	C	C1	Canada	313-831	519
KT370491	C	C1	Canada	313-831	519
KT370492	C	C1	Canada	313-831	519
KT370493	C	C1	Canada	313-831	519
KT370494	C	C1	Canada	315-831	517
KT370495	C	C1	Canada	313-831	519
KT370496	C	C1	Canada	313-831	519
KT370497	C	C1	Canada	313-831	519
KT370498	C	C1	Canada	313-831	519
KT370499	C	C1	Canada	313-831	519
KT370501	C	C1	Canada	313-831	519
KT370503	C	C1	Canada	313-831	519
KT370505	C	C1	Canada	313-831	519
KT370506	C	C5	Canada	313-831	519
KT370507	C	C5	Canada	313-831	519
KT370508	C	C5	Canada	313-831	519

KT370509	C	C5	Canada	313-831	519
KT370510	C	C5	Canada	313-831	519
KT370511	C	C5	Canada	313-831	519
KT370512	C	C5	Canada	313-831	519
KT370513	C	C5	Canada	313-831	519
KT370514	C	C5	Canada	313-831	519
KT370515	C	C5	Canada	313-831	519
KT370516	C	C5	Canada	313-831	519
KT370517	C	C2	Canada	313-831	519
KT370518	C	C1	Canada	313-831	519
KT370519	C	C1	Canada	313-831	519
KT370520	C	C1	Canada	313-831	519
KT370521	C	C2	Canada	313-831	519
KT370522	C	C2	Canada	313-831	519
KT370523	C	C1	Canada	313-831	519
KT370524	C	C2	Canada	313-831	519
KT370525	C	C3	Canada	313-831	519
KT370526	C	C3	Canada	313-831	519
KT370527	C	C3	Canada	313-831	519
KT370528	C	C3	Canada	313-831	519
KT370529	C	C2	Canada	313-831	519
KT370530	C	C3	Canada	313-831	519
KT370531	C	C2	Canada	313-831	519
KT370532	C	C2	Canada	313-831	519
KT370533	C	C2	Canada	313-831	519
KT370534	C	C2	Canada	313-831	519
KT370535	C	C2	Canada	313-831	519
KT370536	C	C2	Canada	313-831	519
KT370537	C	C2	Canada	313-831	519
KT370538	C	C2	Canada	313-831	519
KT370539	C	C2	Canada	313-831	519
KT370540	C	C2	Canada	313-831	519
KT370541	C	C3	Canada	313-831	519
KT370542	C	C2	Canada	313-831	519
KT370543	C	C2	Canada	313-831	519
KT370544	C	C7	Canada	313-831	519
KT370545	C	C7	Canada	313-831	519
KT370546	C	C10	Canada	313-831	519
KT370547	C	C10	Canada	313-831	519
KT370548	C	C2	Canada	313-831	519
KT370549	C	C2	Canada	313-831	519
KT370550	C	C1	Canada	313-831	519
KT370551	C	C1	Canada	313-831	519
KT370552	C	C1	Canada	313-831	519
KT370553	C	C1	Canada	313-831	519
KT370554	C	C1	Canada	313-831	519

KT370555	C	C1	Canada	313-831	519
KT370556	C	C1	Canada	313-831	519
KT370557	C	C10	Canada	313-831	519
KT370558	C	C10	Canada	313-831	519
KT370559	C	C10	Canada	313-831	519
KT370560	C	C2	Canada	313-831	519
KT370561	C	C10	Canada	313-831	519
KT370562	C	C2	Canada	313-831	519
KT370563	C	C1	Canada	313-831	519
KT370564	C	C10	Canada	313-831	519
KT370565	C	C1	Canada	313-831	519
KT370566	C	C1	Canada	313-831	519
KT370567	C	C10	Canada	313-831	519
KT370568	C	C1	Canada	313-831	519
KT370569	C	C1	Canada	313-831	519
KT370570	C	C6	Canada	313-831	519
KT370571	C	C7	Canada	313-831	519

COLOMBIA

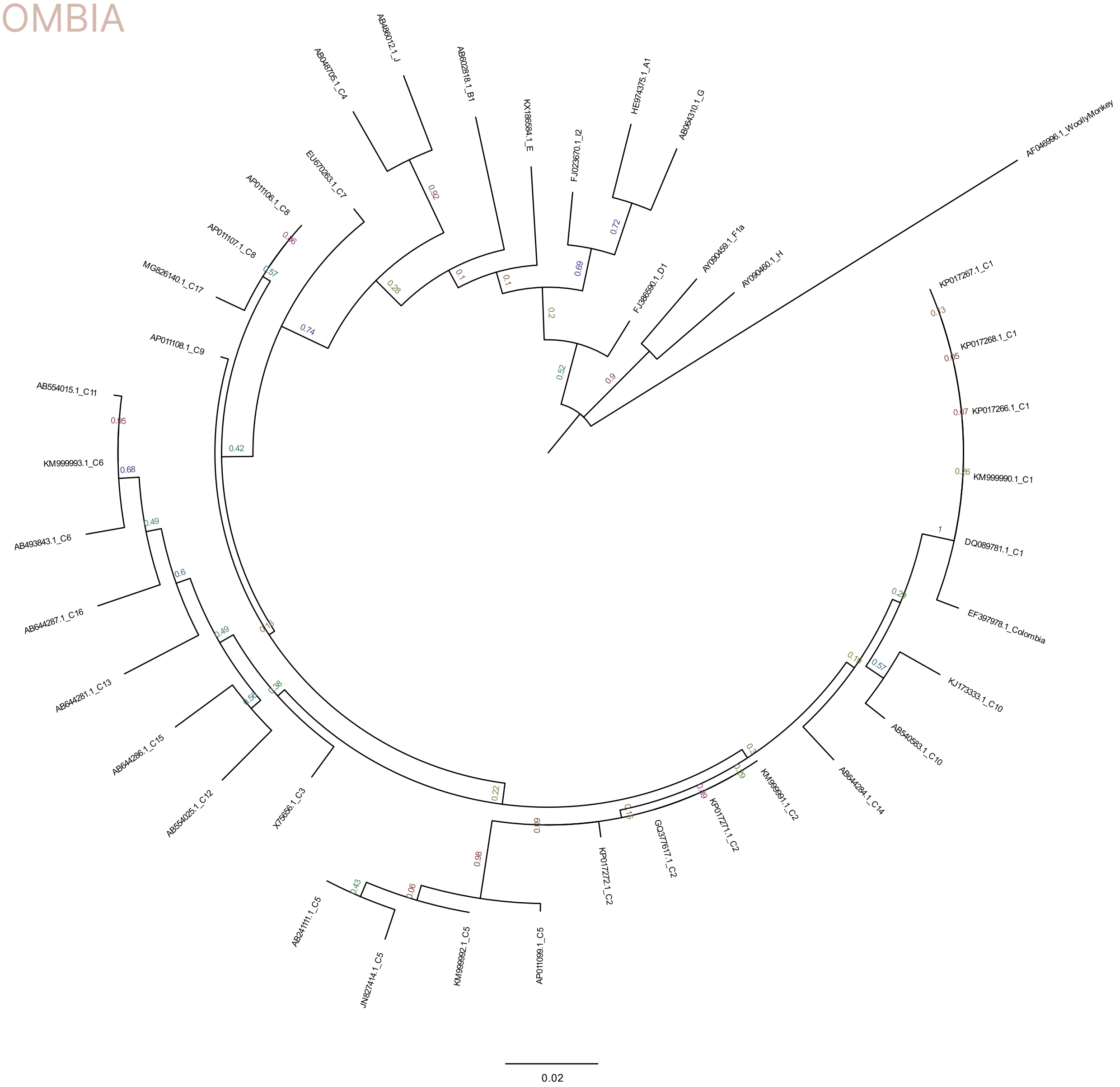

Tree 1. Molecular Phylogenetic analysis by Maximum Likelihood method conducted in MEGA7. The evolutionary history was inferred by using the Maximum Likelihood method based on the Tamura-Nei model with 1000 bootstraps. The tree with the highest log likelihood (-2903.26) is shown. The percentage of trees in which the associated taxa clustered together is shown next to the branches. The tree is drawn to scale, with branch lengths measured in the number of substitutions per site. The analysis involved 41 nucleotide sequences, of which 40 were used as marker sequences to determine the genotype of 1 sequence. All positions containing gaps and missing data were eliminated. There was a total of 594 positions in the final dataset. Additional trees were constructed to determine the subgenotype of each of the sequences.

ID	GENOTYPE	SUBTYPE	COUNTRY	ALIGNMENT[1]	BASE PAIRS
EF397978	C	C1	Colombia	157-753	597

USA

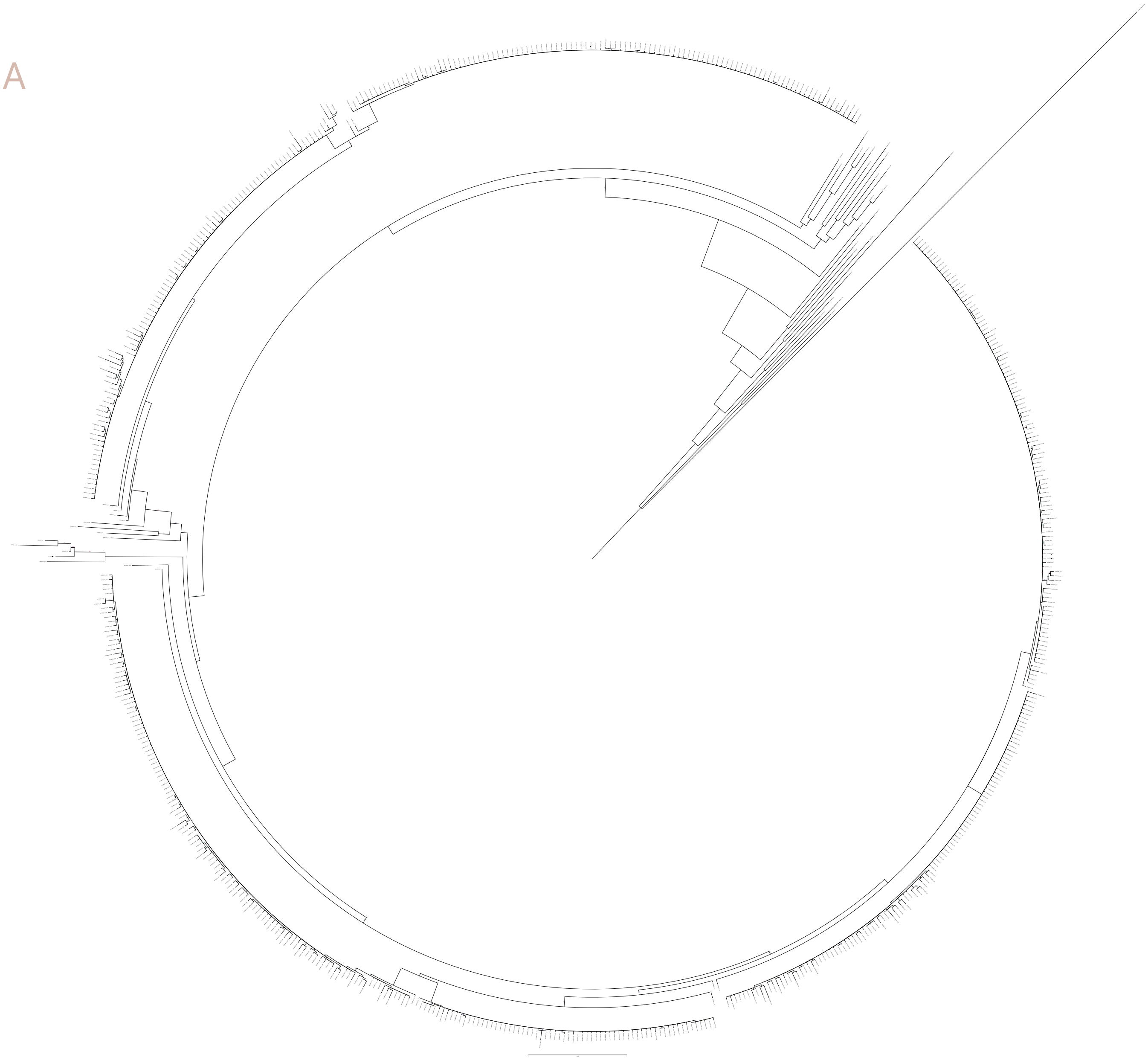

Tree 1. Molecular Phylogenetic analysis by Maximum Likelihood method conducted in MEGA7. The evolutionary history was inferred by using the Maximum Likelihood method based on the Tamura-Nei model with 1000 bootstraps. The tree with the highest log likelihood (-25678.07) is shown. The percentage of trees in which the associated taxa clustered together is shown next to the branches. The tree is drawn to scale, with branch lengths measured in the number of substitutions per site. The analysis involved 655 nucleotide sequences, of which 40 were used as marker sequences to determine the genotype of 615 sequences. All positions containing gaps and missing data were eliminated. There was a total of 2013 positions in the final dataset. Additional trees were constructed to determine the subgenotype of each of the sequences.

ID	GENOTYPE	SUBTYPE	COUNTRY	ALIGNMENT[1]	BASE PAIRS
AB105172	C	C6	USA	Complete Genome	3215
AB105173	C	C1	USA	Complete Genome	3215
AB105174	C	C1	USA	Complete Genome	3215
JN370955	C	C5	USA	1-3182	2053
KF779242	C	C2	USA	Complete Genome	3165
MT426248	C	C1	USA	1-3182	3215
MT426249	C	C1	USA	1-3182	3215
MT426250	C	C1	USA	1-3182	3215

MT426251	C	C1	USA	1-3182	3215
MT426252	C	C1	USA	1-3182	3215
MT426253	C	C1	USA	1-3182	3215
MT426254	C	C1	USA	1-3182	3215
MT426255	C	C1	USA	1-3182	3215
MT426256	C	C1	USA	1-3182	3215
MT426257	C	C1	USA	1-3182	3215
MT426258	C	C1	USA	1-3182	3215
MT426259	C	C1	USA	1-3182	3215
MT426260	C	C1	USA	1-3182	3215
MT426261	C	C1	USA	1-3182	3215
MT426262	C	C1	USA	1-3182	3215
MT426263	C	C1	USA	1-3182	3215
MT426264	C	C1	USA	1-3182	3215
MT426265	C	C1	USA	1-3182	3215
MT426266	C	C1	USA	1-3182	3215
MT426267	C	C1	USA	1-3182	3215
MT426268	C	C1	USA	1-3182	3215
MT426269	C	C1	USA	1-3182	3215
MT426270	C	C1	USA	1-3182	3215
MT426271	C	C1	USA	1-3182	3215
MT426272	C	C1	USA	1-3182	3215
MT426273	C	C1	USA	1-3182	3215
MT426274	C	C1	USA	1-3182	3215
MT426275	C	C1	USA	1-3182	3215
MT426276	C	C1	USA	1-3182	3215
MT426277	C	C1	USA	1-3182	3215
MT426278	C	C1	USA	1-3182	3215
MT426279	C	C1	USA	1-3182	3215
MT426280	C	C1	USA	1-3182	3215
MT426281	C	C1	USA	1-3182	3215
MT426282	C	C1	USA	1-3182	3215
MT426283	C	C1	USA	1-3182	3215
MT426284	C	C1	USA	1-3182	3215
MT426285	C	C1	USA	1-3182	3215
MT426286	C	C1	USA	1-3182	3215
MT426287	C	C1	USA	1-3182	3215
MT426288	C	C1	USA	1-3182	3215
MT426289	C	C1	USA	1-3182	3215
MT426290	C	C1	USA	1-3182	3215
MT426291	C	C1	USA	1-3182	3215
MT426292	C	C1	USA	1-3182	3215
MT426293	C	C1	USA	1-3182	3215
MT426294	C	C1	USA	1-3182	3215
MT426295	C	C1	USA	1-3182	3215
MT426296	C	C1	USA	1-3182	3215

MT426297	C	C1	USA	1-3182	3215
MT426298	C	C1	USA	1-3182	3215
MT426299	C	C1	USA	1-3182	3215
MT426300	C	C1	USA	1-3182	3215
MT426301	C	C1	USA	1-3182	3215
MT426302	C	C1	USA	1-3182	3215
MT426303	C	C1	USA	1-3182	3215
MT426304	C	C1	USA	1-3182	3215
MT426305	C	C1	USA	1-3182	3215
MT426306	C	C1	USA	1-3182	3215
MT426307	C	C1	USA	1-3182	3215
MT426308	C	C1	USA	1-3182	3215
MT426309	C	C1	USA	1-3182	3215
MT426310	C	C1	USA	1-3182	3215
MT426311	C	C1	USA	1-3182	3215
MT426312	C	C1	USA	1-3182	3215
MT426313	C	C1	USA	1-3182	3215
MT426314	C	C1	USA	1-3182	3215
MT426315	C	C1	USA	1-3182	3215
MT426316	C	C1	USA	1-3182	3215
MT426317	C	C1	USA	1-3182	3215
MT426318	C	C1	USA	1-3182	3215
MT426319	C	C1	USA	1-3182	3215
MT426320	C	C1	USA	1-3182	3215
MT426321	C	C1	USA	1-3182	3215
MT426322	C	C1	USA	1-3182	3215
MT426323	C	C1	USA	1-3182	3215
MT426324	C	C1	USA	1-3182	3215
MT426325	C	C1	USA	1-3182	3215
MT426326	C	C1	USA	1-3182	3215
MT426327	C	C1	USA	1-3182	3215
MT426328	C	C1	USA	1-3182	3215
MT426329	C	C1	USA	1-3182	3215
MT426330	C	C1	USA	1-3182	3215
MT426331	C	C1	USA	1-3182	3215
MT426332	C	C1	USA	1-3182	3215
MT426333	C	C1	USA	1-3182	3215
MT426334	C	C1	USA	1-3182	3215
MT426335	C	C1	USA	1-3182	3215
MT426336	C	C1	USA	1-3182	3215
MT426337	C	C1	USA	1-3182	3215
MT426338	C	C1	USA	1-3182	3215
MT426339	C	C1	USA	1-3182	3215
MT426340	C	C1	USA	1-3182	3215
MT426341	C	C1	USA	1-3182	3215
MT426342	C	C1	USA	1-3182	3215

MT426343	C	C1	USA	1-3182	3215
MT426344	C	C1	USA	1-3182	3215
MT426345	C	C1	USA	1-3182	3215
MT426346	C	C1	USA	1-3182	3215
MT426347	C	C1	USA	1-3182	3215
MT426348	C	C1	USA	1-3182	3215
MT426349	C	C1	USA	1-3182	3215
MT426350	C	C1	USA	1-3182	3215
MT426351	C	C1	USA	1-3182	3215
MT426352	C	C1	USA	1-3182	3215
MT426353	C	C1	USA	1-3182	3215
MT426354	C	C1	USA	1-3182	3215
MT426355	C	C1	USA	1-3182	3215
MT426356	C	C1	USA	1-3182	3215
MT426357	C	C1	USA	1-3182	3215
MT426358	C	C1	USA	1-3182	3215
MT426359	C	C1	USA	1-3182	3215
MT426360	C	C1	USA	1-3182	3215
MT426361	C	C1	USA	1-3182	3215
MT426362	C	C1	USA	1-3182	3215
MT426363	C	C1	USA	1-3182	3215
MT426364	C	C1	USA	1-3182	3215
MT426365	C	C1	USA	1-3182	3215
MT426366	C	C1	USA	1-3182	3215
MT426367	C	C1	USA	1-3182	3215
MT426368	C	C1	USA	1-3182	3215
MT426369	C	C1	USA	1-3182	3215
MT426370	C	C1	USA	1-3182	3215
MT426371	C	C1	USA	1-3182	3215
MT426372	C	C1	USA	1-3182	3215
MT426373	C	C1	USA	1-3182	3215
MT426374	C	C1	USA	1-3182	3215
MT426375	C	C1	USA	1-3182	3215
MT426376	C	C1	USA	1-3182	3215
MT426377	C	C1	USA	1-3182	3215
MT426378	C	C1	USA	1-3182	3215
MT426379	C	C1	USA	1-3182	3215
MT426380	C	C1	USA	1-3182	3215
MT426381	C	C1	USA	1-3182	3215
MT426382	C	C1	USA	1-3182	3215
MT426383	C	C1	USA	1-3182	3215
MT426384	C	C1	USA	1-3182	3215
MT426385	C	C1	USA	1-3182	3215
MT426386	C	C1	USA	1-3182	3215
MT426387	C	C1	USA	1-3182	3215
MT426388	C	C1	USA	1-3182	3215

MT426389	C	C1	USA	1-3182	3215
MT426390	C	C1	USA	1-3182	3215
MT426391	C	C1	USA	1-3182	3215
MT426392	C	C1	USA	1-3182	3215
MT426393	C	C1	USA	1-3182	3215
MT426394	C	C1	USA	1-3182	3215
MT426395	C	C1	USA	1-3182	3215
MT426396	C	C1	USA	1-3182	3215
MT426397	C	C1	USA	1-3182	3215
MT426398	C	C1	USA	1-3182	3215
MT426399	C	C1	USA	1-3182	3215
MT426400	C	C1	USA	1-3182	3215
MT426401	C	C1	USA	1-3182	3215
MT426402	C	C1	USA	1-3182	3215
MT426403	C	C1	USA	1-3182	3215
MT426404	C	C1	USA	1-3182	3215
MT426405	C	C1	USA	1-3182	3215
MT426406	C	C1	USA	1-3182	3215
MT426407	C	C1	USA	1-3182	3215
MT426408	C	C1	USA	1-3182	3215
MT426409	C	C1	USA	1-3182	3215
MT426410	C	C1	USA	1-3182	3215
MT426411	C	C1	USA	1-3182	3215
MT426412	C	C1	USA	1-3182	3215
MT426413	C	C1	USA	1-3182	3215
MT426414	C	C1	USA	1-3182	3215
MT426415	C	C1	USA	1-3182	3215
MT426416	C	C1	USA	1-3182	3215
MT426417	C	C1	USA	1-3182	3215
MT426418	C	C1	USA	1-3182	3215
MT426419	C	C1	USA	1-3182	3215
MT426420	C	C1	USA	1-3182	3215
MT426421	C	C1	USA	1-3182	3215
MT426422	C	C1	USA	1-3182	3215
MT426423	C	C1	USA	1-3182	3215
MT426424	C	C1	USA	1-3182	3215
MT426425	C	C1	USA	1-3182	3215
MT426426	C	C1	USA	1-3182	3215
MT426427	C	C1	USA	1-3182	3215
MT426428	C	C1	USA	1-3182	3215
MT426429	C	C1	USA	1-3182	3215
MT426430	C	C1	USA	1-3182	3215
MT426431	C	C1	USA	1-3182	3215
MT426432	C	C1	USA	1-3182	3215
MT426433	C	C1	USA	1-3182	3215
MT426434	C	C1	USA	1-3182	3215

MT426435	C	C1	USA	1-3182	3215
MT426436	C	C1	USA	1-3182	3215
MT426437	C	C1	USA	1-3182	3215
MT426438	C	C1	USA	1-3182	3215
MT426439	C	C1	USA	1-3182	3215
MT426440	C	C1	USA	1-3182	3215
MT426441	C	C1	USA	1-3182	3215
MT426442	C	C1	USA	1-3182	3215
MT426443	C	C1	USA	1-3182	3215
MT426444	C	C1	USA	1-3182	3215
MT426445	C	C1	USA	1-3182	3215
MT426446	C	C1	USA	1-3182	3215
MT426447	C	C1	USA	1-3182	3215
MT426448	C	C1	USA	1-3182	3215
MT426449	C	C1	USA	1-3182	3215
MT426450	C	C1	USA	1-3182	3215
MT426451	C	C1	USA	1-3182	3215
MT426452	C	C1	USA	1-3182	3215
MT426453	C	C1	USA	1-3182	3215
MT426454	C	C1	USA	1-3182	3215
MT426455	C	C2	USA	1-3182	3197
MT426456	C	C2	USA	1-3182	3197
MT426457	C	C2	USA	1-3182	3197
MT426458	C	C2	USA	1-3182	3197
MT426459	C	C2	USA	1-3182	3197
MT426460	C	C2	USA	1-3182	3197
MT426461	C	C2	USA	1-3182	3197
MT426462	C	C2	USA	1-3182	3197
MT426463	C	C2	USA	1-3182	3197
MT426464	C	C2	USA	1-3182	3197
MT426465	C	C2	USA	1-3182	3197
MT426466	C	C2	USA	1-3182	3197
MT426467	C	C2	USA	1-3182	3197
MT426468	C	C2	USA	1-3182	3197
MT426469	C	C2	USA	1-3182	3197
MT426470	C	C2	USA	1-3182	3197
MT426471	C	C2	USA	1-3182	3197
MT426472	C	C2	USA	1-3182	3197
MT426473	C	C2	USA	1-3182	3197
MT426474	C	C2	USA	1-3182	3197
MT426475	C	C2	USA	1-3182	3197
MT426476	C	C2	USA	1-3182	3197
MT426477	C	C2	USA	1-3182	3197
MT426478	C	C2	USA	1-3182	3197
MT426479	C	C2	USA	1-3182	3197
MT426480	C	C2	USA	1-3182	3197

MT426481	C	C2	USA	1-3182	3197
MT426482	C	C2	USA	1-3182	3197
MT426483	C	C2	USA	1-3182	3197
MT426484	C	C2	USA	1-3182	3197
MT426485	C	C2	USA	1-3182	3197
MT426486	C	C2	USA	1-3182	3197
MT426487	C	C2	USA	1-3182	3197
MT426488	C	C2	USA	1-3182	3197
MT426489	C	C2	USA	1-3182	3197
MT426490	C	C2	USA	1-3182	3197
MT426491	C	C2	USA	1-3182	3197
MT426492	C	C2	USA	1-3182	3197
MT426493	C	C2	USA	1-3182	3197
MT426494	C	C2	USA	1-3182	3197
MT426495	C	C2	USA	1-3182	3197
MT426496	C	C2	USA	1-3182	3197
MT426497	C	C2	USA	1-3182	3197
MT426498	C	C2	USA	1-3182	3197
MT426499	C	C2	USA	1-3182	3200
MT426500	C	C2	USA	1-3182	3200
MT426501	C	C2	USA	1-3182	3200
MT426502	C	C2	USA	1-3182	3200
MT426503	C	C2	USA	1-3182	3200
MT426504	C	C2	USA	1-3182	3200
MT426505	C	C2	USA	1-3182	3200
MT426506	C	C2	USA	1-3182	3200
MT426507	C	C2	USA	1-3182	3200
MT426508	C	C2	USA	1-3182	3200
MT426509	C	C2	USA	1-3182	3200
MT426510	C	C2	USA	1-3182	3200
MT426511	C	C2	USA	1-3182	3200
MT426512	C	C2	USA	1-3182	3200
MT426513	C	C2	USA	1-3182	3200
MT426515	C	C2	USA	1-3182	3200
MT426516	C	C2	USA	1-3182	3200
MT426517	C	C2	USA	1-3182	3200
MT426518	C	C2	USA	1-3182	3200
MT426519	C	C2	USA	1-3182	3200
MT426520	C	C2	USA	1-3182	3200
MT426521	C	C2	USA	1-3182	3200
MT426522	C	C2	USA	1-3182	3215
MT426523	C	C2	USA	1-3182	3215
MT426524	C	C2	USA	1-3182	3215
MT426525	C	C2	USA	1-3182	3215
MT426526	C	C2	USA	1-3182	3215
MT426527	C	C2	USA	1-3182	3215

MT426528	C	C2	USA	1-3182	3215
MT426529	C	C2	USA	1-3182	3215
MT426530	C	C2	USA	1-3182	3215
MT426531	C	C2	USA	1-3182	3215
MT426532	C	C2	USA	1-3182	3215
MT426533	C	C2	USA	1-3182	3215
MT426534	C	C2	USA	1-3182	3215
MT426535	C	C2	USA	1-3182	3215
MT426536	C	C2	USA	1-3182	3215
MT426537	C	C2	USA	1-3182	3215
MT426538	C	C2	USA	1-3182	3215
MT426539	C	C2	USA	1-3182	3215
MT426540	C	C2	USA	1-3182	3215
MT426541	C	C2	USA	1-3182	3215
MT426542	C	C2	USA	1-3182	3215
MT426543	C	C2	USA	1-3182	3215
MT426544	C	C2	USA	1-3182	3215
MT426545	C	C2	USA	1-3182	3215
MT426546	C	C2	USA	1-3182	3215
MT426547	C	C2	USA	1-3182	3215
MT426548	C	C2	USA	1-3182	3215
MT426549	C	C2	USA	1-3182	3215
MT426550	C	C2	USA	1-3182	3215
MT426551	C	C2	USA	1-3182	3215
MT426552	C	C2	USA	1-3182	3215
MT426553	C	C2	USA	1-3182	3215
MT426554	C	C2	USA	1-3182	3215
MT426555	C	C2	USA	1-3182	3215
MT426556	C	C2	USA	1-3182	3215
MT426557	C	C2	USA	1-3182	3215
MT426558	C	C2	USA	1-3182	3215
MT426559	C	C2	USA	1-3182	3215
MT426560	C	C2	USA	1-3182	3215
MT426561	C	C2	USA	1-3182	3215
MT426562	C	C2	USA	1-3182	3215
MT426563	C	C2	USA	1-3182	3215
MT426564	C	C2	USA	1-3182	3215
MT426565	C	C2	USA	1-3182	3215
MT426566	C	C2	USA	1-3182	3215
MT426567	C	C2	USA	1-3182	3215
MT426568	C	C2	USA	1-3182	3215
MT426569	C	C2	USA	1-3182	3215
MT426570	C	C2	USA	1-3182	3215
MT426571	C	C2	USA	1-3182	3215
MT426572	C	C2	USA	1-3182	3215
MT426573	C	C2	USA	1-3182	3215

MT426574	C	C2	USA	1-3182	3215
MT426575	C	C2	USA	1-3182	3215
MT426576	C	C2	USA	1-3182	3215
MT426577	C	C2	USA	1-3182	3215
MT426578	C	C2	USA	1-3182	3215
MT426579	C	C2	USA	1-3182	3215
MT426580	C	C2	USA	1-3182	3215
MT426581	C	C2	USA	1-3182	3215
MT426582	C	C2	USA	1-3182	3215
MT426583	C	C2	USA	1-3182	3215
MT426584	C	C2	USA	1-3182	3215
MT426585	C	C2	USA	1-3182	3215
MT426586	C	C2	USA	1-3182	3215
MT426587	C	C2	USA	1-3182	3215
MT426588	C	C2	USA	1-3182	3215
MT426589	C	C2	USA	1-3182	3215
MT426590	C	C2	USA	1-3182	3215
MT426591	C	C2	USA	1-3182	3215
MT426592	C	C2	USA	1-3182	3215
MT426593	C	C2	USA	1-3182	3215
MT426594	C	C2	USA	1-3182	3215
MT426595	C	C2	USA	1-3182	3215
MT426596	C	C2	USA	1-3182	3215
MT426597	C	C2	USA	1-3182	3215
MT426598	C	C2	USA	1-3182	3215
MT426599	C	C2	USA	1-3182	3215
MT426600	C	C2	USA	1-3182	3215
MT426601	C	C2	USA	1-3182	3215
MT426602	C	C2	USA	1-3182	3215
MT426603	C	C2	USA	1-3182	3215
MT426604	C	C2	USA	1-3182	3215
MT426605	C	C2	USA	1-3182	3215
MT426606	C	C2	USA	1-3182	3215
MT426607	C	C2	USA	1-3182	3215
MT426608	C	C2	USA	1-3182	3215
MT426609	C	C2	USA	1-3182	3215
MT426610	C	C2	USA	1-3182	3215
MT426611	C	C2	USA	1-3182	3215
MT426612	C	C2	USA	1-3182	3215
MT426613	C	C2	USA	1-3182	3215
MT426614	C	C2	USA	1-3182	3215
MT426615	C	C2	USA	1-3182	3215
MT426616	C	C2	USA	1-3182	3215
MT426617	C	C2	USA	1-3182	3215
MT426618	C	C2	USA	1-3182	3215
MT426619	C	C2	USA	1-3182	3215

MT426620	C	C2	USA	1-3182	3215
MT426621	C	C2	USA	1-3182	3215
MT426622	C	C2	USA	1-3182	3215
MT426623	C	C2	USA	1-3182	3215
MT426624	C	C2	USA	1-3182	3215
MT426625	C	C2	USA	1-3182	3215
MT426626	C	C2	USA	1-3182	3215
MT426627	C	C2	USA	1-3182	3215
MT426628	C	C2	USA	1-3182	3215
MT426629	C	C2	USA	1-3182	3215
MT426630	C	C2	USA	1-3182	3215
MT426631	C	C2	USA	1-3182	3215
MT426632	C	C2	USA	1-3182	3215
MT426633	C	C2	USA	1-3182	3215
MT426634	C	C2	USA	1-3182	3215
MT426635	C	C2	USA	1-3182	3215
MT426636	C	C2	USA	1-3182	3215
MT426637	C	C2	USA	1-3182	3215
MT426638	C	C2	USA	1-3182	3215
MT426639	C	C2	USA	1-3182	3215
MT426640	C	C2	USA	1-3182	3215
MT426641	C	C2	USA	1-3182	3215
MT426642	C	C2	USA	1-3182	3215
MT426643	C	C2	USA	1-3182	3215
MT426644	C	C2	USA	1-3182	3215
MT426645	C	C2	USA	1-3182	3215
MT426646	C	C2	USA	1-3182	3215
MT426647	C	C2	USA	1-3182	3215
MT426648	C	C2	USA	1-3182	3215
MT426649	C	C2	USA	1-3182	3215
MT426650	C	C2	USA	1-3182	3215
MT426651	C	C2	USA	1-3182	3215
MT426652	C	C2	USA	1-3182	3215
MT426653	C	C2	USA	1-3182	3215
MT426654	C	C2	USA	1-3182	3215
MT426655	C	C2	USA	1-3182	3215
MT426656	C	C2	USA	1-3182	3215
MT426657	C	C2	USA	1-3182	3215
MT426658	C	C2	USA	1-3182	3215
MT426659	C	C2	USA	1-3182	3215
MT426660	C	C2	USA	1-3182	3215
MT426661	C	C2	USA	1-3182	3215
MT426662	C	C2	USA	1-3182	3215
MT426663	C	C2	USA	1-3182	3215
MT426664	C	C2	USA	1-3182	3215
MT426665	C	C2	USA	1-3182	3215

MT426666	C	C2	USA	1-3182	3215
MT426667	C	C2	USA	1-3182	3215
MT426668	C	C2	USA	1-3182	3215
MT426669	C	C2	USA	1-3182	3215
MT426670	C	C2	USA	1-3182	3215
MT426671	C	C2	USA	1-3182	3215
MT426672	C	C2	USA	1-3182	3215
MT426673	C	C2	USA	1-3182	3215
MT426674	C	C2	USA	1-3182	3215
MT426675	C	C2	USA	1-3182	3215
MT426676	C	C2	USA	1-3182	3215
MT426677	C	C2	USA	1-3182	3215
MT426678	C	C2	USA	1-3182	3215
MT426679	C	C2	USA	1-3182	3215
MT426680	C	C2	USA	1-3182	3215
MT426681	C	C2	USA	1-3182	3215
MT426682	C	C2	USA	1-3182	3215
MT426683	C	C2	USA	1-3182	3215
MT426684	C	C2	USA	1-3182	3215
MT426685	C	C2	USA	1-3182	3215
MT426686	C	C2	USA	1-3182	3215
MT426687	C	C2	USA	1-3182	3215
MT426688	C	C2	USA	1-3182	3215
MT426689	C	C2	USA	1-3182	3215
MT426690	C	C2	USA	1-3182	3215
MT426691	C	C2	USA	1-3182	3215
MT426692	C	C2	USA	1-3182	3215
MT426693	C	C2	USA	1-3182	3215
MT426694	C	C2	USA	1-3182	3215
MT426695	C	C2	USA	1-3182	3215
MT426696	C	C2	USA	1-3182	3215
MT426697	C	C2	USA	1-3182	3215
MT426698	C	C2	USA	1-3182	3215
MT426699	C	C2	USA	1-3182	3215
MT426700	C	C2	USA	1-3182	3215
MT426701	C	C2	USA	1-3182	3215
MT426702	C	C2	USA	1-3182	3215
MT426703	C	C2	USA	1-3182	3215
MT426704	C	C2	USA	1-3182	3215
MT426705	C	C2	USA	1-3182	3215
MT426706	C	C2	USA	1-3182	3215
MT426707	C	C2	USA	1-3182	3215
MT426708	C	C2	USA	1-3182	3215
MT426709	C	C2	USA	1-3182	3215
MT426710	C	C2	USA	1-3182	3215
MT426711	C	C2	USA	1-3182	3215

MT426712	C	C2	USA	1-3182	3215
MT426713	C	C2	USA	1-3182	3215
MT426714	C	C2	USA	1-3182	3215
MT426715	C	C2	USA	1-3182	3215
MT426716	C	C2	USA	1-3182	3215
MT426717	C	C2	USA	1-3182	3215
MT426718	C	C2	USA	1-3182	3215
MT426719	C	C2	USA	1-3182	3215
MT426720	C	C2	USA	1-3182	3215
MT426721	C	C2	USA	1-3182	3215
MT426722	C	C2	USA	1-3182	3215
MT426723	C	C2	USA	1-3182	3215
MT426724	C	C2	USA	1-3182	3215
MT426725	C	C2	USA	1-3182	3215
MT426726	C	C2	USA	1-3182	3215
MT426727	C	C2	USA	1-3182	3215
MT426728	C	C2	USA	1-3182	3215
MT426729	C	C2	USA	1-3182	3215
MT426730	C	C2	USA	1-3182	3215
MT426731	C	C2	USA	1-3182	3215
MT426732	C	C2	USA	1-3182	3215
MT426733	C	C2	USA	1-3182	3215
MT426734	C	C2	USA	1-3182	3215
MT426735	C	C2	USA	1-3182	3215
MT426736	C	C2	USA	1-3182	3215
MT426737	C	C2	USA	1-3182	3215
MT426738	C	C2	USA	1-3182	3215
MT426739	C	C2	USA	1-3182	3215
MT426740	C	C2	USA	1-3182	3215
MT426741	C	C2	USA	1-3182	3215
MT426742	C	C2	USA	1-3182	3215
MT426743	C	C2	USA	1-3182	3215
MT426744	C	C2	USA	1-3182	3215
MT426745	C	C2	USA	1-3182	3215
MT426746	C	C2	USA	1-3182	3215
MT426747	C	C2	USA	1-3182	3215
MT426748	C	C2	USA	1-3182	3215
MT426749	C	C2	USA	1-3182	3215
MT426750	C	C2	USA	1-3182	3215
MT426751	C	C2	USA	1-3182	3215
MT426752	C	C2	USA	1-3182	3215
MT426753	C	C2	USA	1-3182	3215
MT426754	C	C2	USA	1-3182	3215
MT426755	C	C2	USA	1-3182	3215
MT426756	C	C2	USA	1-3182	3215
MT426757	C	C2	USA	1-3182	3215

MT426758	C	C2	USA	1-3182	3215
MT426759	C	C2	USA	1-3182	3215
MT426760	C	C2	USA	1-3182	3215
MT426761	C	C2	USA	1-3182	3215
MT426762	C	C2	USA	1-3182	3215
MT426763	C	C2	USA	1-3182	3215
MT426764	C	C2	USA	1-3182	3215
MT426765	C	C2	USA	1-3182	3215
MT426766	C	C2	USA	1-3182	3215
MT426767	C	C2	USA	1-3182	3215
MT426768	C	C2	USA	1-3182	3215
MT426769	C	C2	USA	1-3182	3215
MT426770	C	C2	USA	1-3182	3215
MT426771	C	C2	USA	1-3182	3215
MT426772	C	C2	USA	1-3182	3215
MT426773	C	C2	USA	1-3182	3215
MT426774	C	C2	USA	1-3182	3215
MT426775	C	C2	USA	1-3182	3215
MT426776	C	C2	USA	1-3182	3215
MT426777	C	C2	USA	1-3182	3215
MT426778	C	C2	USA	1-3182	3215
MT426779	C	C2	USA	1-3182	3215
MT426780	C	C2	USA	1-3182	3215
MT426781	C	C2	USA	1-3182	3215
MT426782	C	C2	USA	1-3182	3215
MT426783	C	C2	USA	1-3182	3215
MT426784	C	C2	USA	1-3182	3215
MT426785	C	C2	USA	1-3182	3215
MT426786	C	C2	USA	1-3182	3215
MT426787	C	C2	USA	1-3182	3215
MT426788	C	C2	USA	1-3182	3215
MT426789	C	C2	USA	1-3182	3215
MT426790	C	C2	USA	1-3182	3215
MT426791	C	C2	USA	1-3182	3215
MT426792	C	C2	USA	1-3182	3215
MT426793	C	C2	USA	1-3182	3215
MT426794	C	C2	USA	1-3182	3215
MT426795	C	C2	USA	1-3182	3215
MT426796	C	C2	USA	1-3182	3215
MT426797	C	C2	USA	1-3182	3215
MT426798	C	C2	USA	1-3182	3215
MT426799	C	C2	USA	1-3182	3215
MT426800	C	C2	USA	1-3182	3215
MT426801	C	C2	USA	1-3182	3215
MT426802	C	C2	USA	1-3182	3215
MT426803	C	C2	USA	1-3182	3215

MT426804	C	C2	USA	1-3182	3215
MT426805	C	C2	USA	1-3182	3215
MT426806	C	C2	USA	1-3182	3215
MT426807	C	C2	USA	1-3182	3215
MT426808	C	C2	USA	1-3182	3215
MT426809	C	C2	USA	1-3182	3215
MT426810	C	C2	USA	1-3182	3215
MT426811	C	C2	USA	1-3182	3215
MT426812	C	C2	USA	1-3182	3215
MT426813	C	C2	USA	1-3182	3215
MT426814	C	C2	USA	1-3182	3215
MT426815	C	C2	USA	1-3182	3215
MT426816	C	C2	USA	1-3182	3215
MT426817	C	C2	USA	1-3182	3215
MT426818	C	C2	USA	1-3182	3215
MT426819	C	C2	USA	1-3182	3215
MT426820	C	C2	USA	1-3182	3215
MT426821	C	C2	USA	1-3182	3215
MT426822	C	C2	USA	1-3182	3215
MT426823	C	C2	USA	1-3182	3215
MT426824	C	C2	USA	1-3182	3215
MT426825	C	C2	USA	1-3182	3215
MT426826	C	C2	USA	1-3182	3215
MT426827	C	C2	USA	1-3182	3215
MT426828	C	C2	USA	1-3182	3215
MT426829	C	C2	USA	1-3182	3215
MT426830	C	C2	USA	1-3182	3215
MT426831	C	C2	USA	1-3182	3215
MT426832	C	C2	USA	1-3182	3215
MT426833	C	C2	USA	1-3182	3215
MT426834	C	C2	USA	1-3182	3215
MT426835	C	C2	USA	1-3182	3215
MT426836	C	C2	USA	1-3182	3215
MT426837	C	C2	USA	1-3182	3215
MT426838	C	C2	USA	1-3182	3215
MT426839	C	C2	USA	1-3182	3215
MT426840	C	C2	USA	1-3182	3215
MT426841	C	C2	USA	1-3182	3215
MT426842	C	C2	USA	1-3182	3215
MT426843	C	C2	USA	1-3182	3215
MT426844	C	C2	USA	1-3182	3215
MT426845	C	C2	USA	1-3182	3215
MT426846	C	C2	USA	1-3182	3215
MT426847	C	C2	USA	1-3182	3215
MT426848	C	C2	USA	1-3182	3215
MT426849	C	C2	USA	1-3182	3215

MT426850	C	C2	USA	1-3182	3215
MT426851	C	C2	USA	1-3182	3215
MT426852	C	C2	USA	1-3182	3215
MT426853	C	C2	USA	1-3182	3215
MT426854	C	C2	USA	1-3182	3215
MT426855	C	C2	USA	1-3182	3215
MT426856	C	C2	USA	1-3182	3215
MT426857	C	C2	USA	1-3182	3215
MT426858	C	C2	USA	1-3182	3215

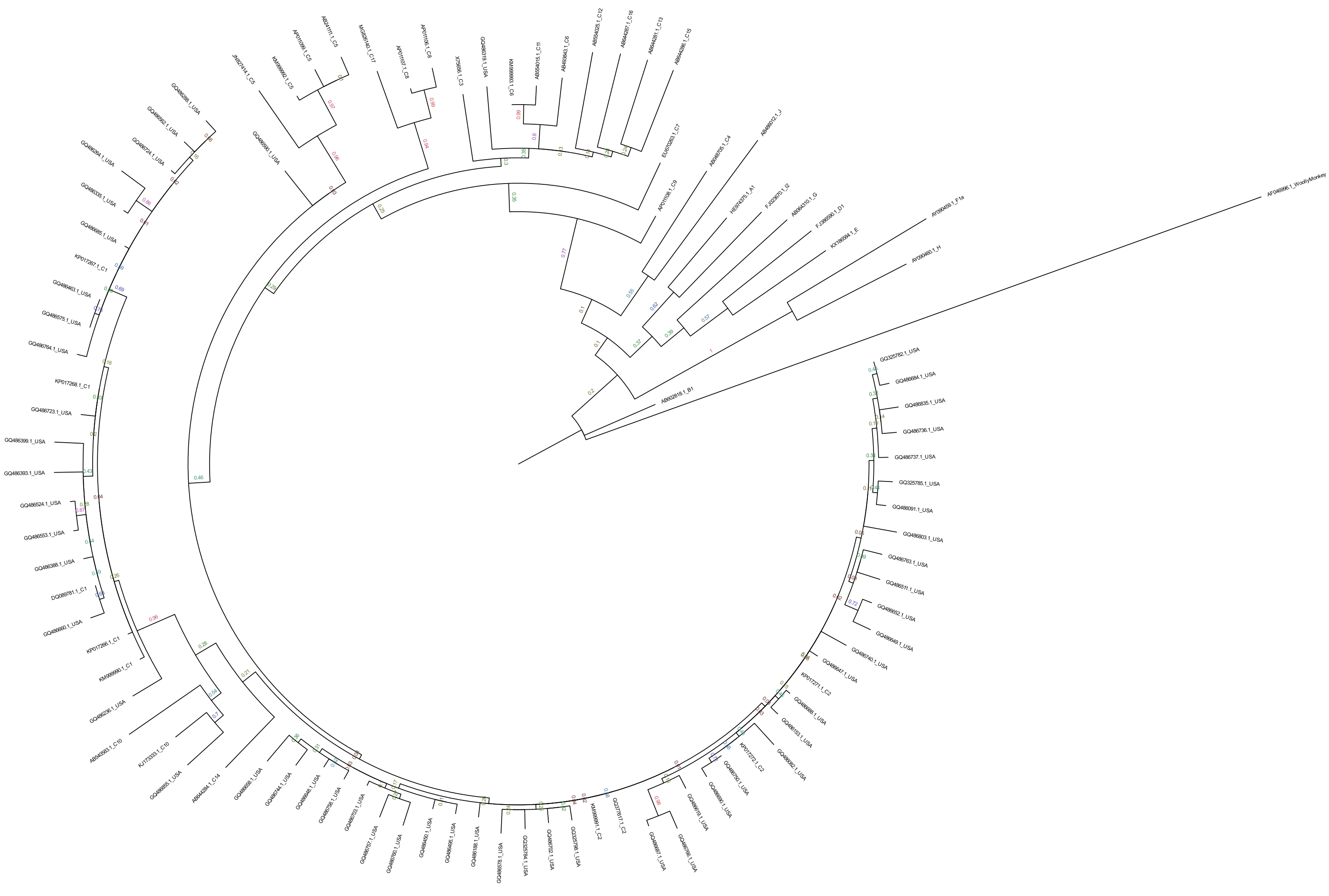

Tree 2. Molecular Phylogenetic analysis by Maximum Likelihood method conducted in MEGA7. The evolutionary history was inferred by using the Maximum Likelihood method based on the Tamura-Nei model with 1000 bootstraps. The tree with the highest log likelihood (-7509.64) is shown. The percentage of trees in which the associated taxa clustered together is shown next to the branches. The tree is drawn to scale, with branch lengths measured in the number of substitutions per site. The analysis involved 96 nucleotide sequences, of which 40 were used as marker sequences to determine the genotype of 56 sequences. All positions containing gaps and missing data were eliminated. There was a total of 930 positions in the final dataset. Additional trees were constructed to determine the subgenotype of each of the sequences.

ID	GENOTYPE	SUBTYPE	COUNTRY	ALIGNMENT[1]	BASE PAIRS
GQ325782	C	C2	USA	132-1163	1032
GQ325784	C	C2	USA	132-1163	1032
GQ325785	C	C2	USA	132-1163	1032
GQ325798	C	C2	USA	132-1163	1032
GQ486082	C	C2	USA	132-1163	1032
GQ486091	C	C2	USA	132-1163	1032
GQ486153	C	C2	USA	132-1163	1032
GQ486188	C	C2	USA	132-1163	1032
GQ486236	C	C1	USA	132-1163	1032
GQ486264	C	C1	USA	132-1163	1032

GQ486288	C	C1	USA	132-1163	1032
GQ486319	C	C3	USA	132-1163	1032
GQ486335	C	C1	USA	132-1163	1032
GQ486388	C	C1	USA	132-1163	1032
GQ486393	C	C1	USA	132-1163	1032
GQ486399	C	C1	USA	132-1163	1032
GQ486450	C	C1	USA	132-1163	1032
GQ486463	C	C1	USA	132-1163	1032
GQ486495	C	C2	USA	132-1163	1032
GQ486511	C	C2	USA	132-1163	1032
GQ486524	C	C1	USA	132-1163	1032
GQ486553	C	C1	USA	132-1163	1032
GQ486562	C	C1	USA	132-1163	1032
GQ486575	C	C1	USA	132-1163	1032
GQ486578	C	C2	USA	132-1163	1032
GQ486590	C	C5	USA	132-1163	1032
GQ486619	C	C2	USA	132-1163	1032
GQ486647	C	C2	USA	132-1163	1032
GQ486648	C	C2	USA	132-1163	1032
GQ486649	C	C2	USA	132-1163	1032
GQ486652	C	C2	USA	132-1163	1032
GQ486658	C	C2	USA	132-1163	1032
GQ486660	C	C1	USA	132-1163	1032
GQ486684	C	C2	USA	132-1163	1032
GQ486685	C	C1	USA	132-1163	1032
GQ486687	C	C2	USA	132-1163	1032
GQ486688	C	C2	USA	132-1163	1032
GQ486690	C	C2	USA	132-1163	1032
GQ486702	C	C2	USA	132-1163	1032
GQ486703	C	C2	USA	132-1163	1032
GQ486723	C	C1	USA	132-1163	1032
GQ486724	C	C1	USA	132-1163	1032
GQ486736	C	C2	USA	132-1163	1032
GQ486737	C	C2	USA	132-1163	1032
GQ486740	C	C2	USA	132-1163	1032
GQ486744	C	C2	USA	132-1163	1032
GQ486750	C	C2	USA	132-1163	1032
GQ486757	C	C2	USA	132-1163	1032
GQ486758	C	C2	USA	132-1163	1032
GQ486760	C	C2	USA	132-1163	1032
GQ486763	C	C2	USA	132-1163	1032
GQ486764	C	C1	USA	132-1163	1032
GQ486766	C	C2	USA	132-1163	1032
GQ486803	C	C2	USA	132-1163	1032
GQ486835	C	C2	USA	132-1163	1032
GQ486855	C	C10	USA	132-1163	1032

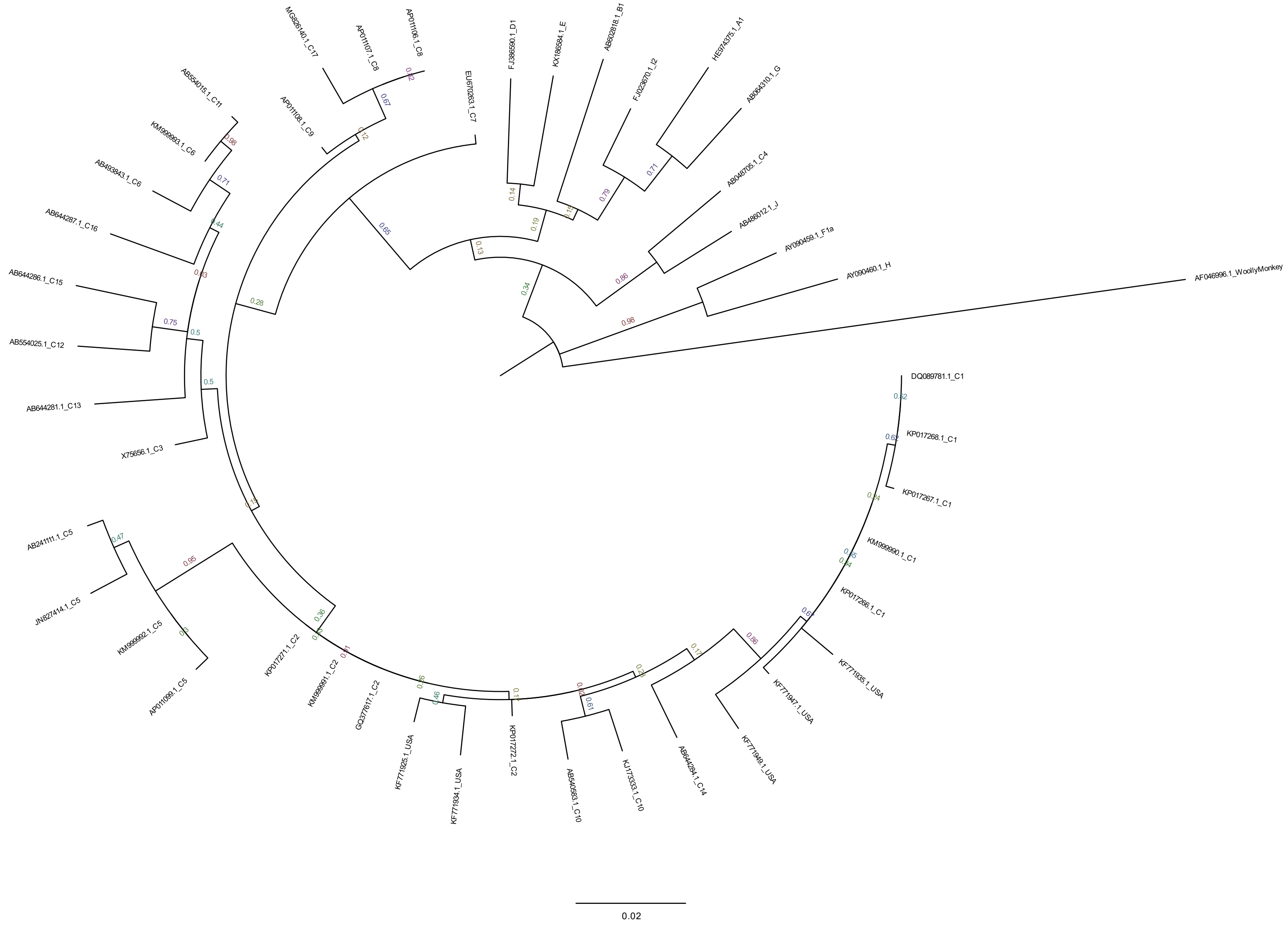

Tree 3. Molecular Phylogenetic analysis by Maximum Likelihood method conducted in MEGA7. The evolutionary history was inferred by using the Maximum Likelihood method based on the Tamura-Nei model with 1000 bootstraps. The tree with the highest log likelihood (-3449.42) is shown. The percentage of trees in which the associated taxa clustered together is shown next to the branches. The tree is drawn to scale, with branch lengths measured in the number of substitutions per site. The analysis involved 45 nucleotide sequences, of which 40 were used as marker sequences to determine the genotype of 5 sequences. All positions containing gaps and missing data were eliminated. There was a total of 673 positions in the final dataset. Additional trees were constructed to determine the subgenotype of each of the sequences.

ID	GENOTYPE	SUBTYPE	COUNTRY	ALIGNMENT[1]	BASE PAIRS
KF771925	C	C2	USA	157-837	681
KF771934	C	C2	USA	157-837	681
KF771935	C	C1	USA	157-837	681
KF771947	C	C1	USA	157-837	681
KF771949	C	C1	USA	157-837	681

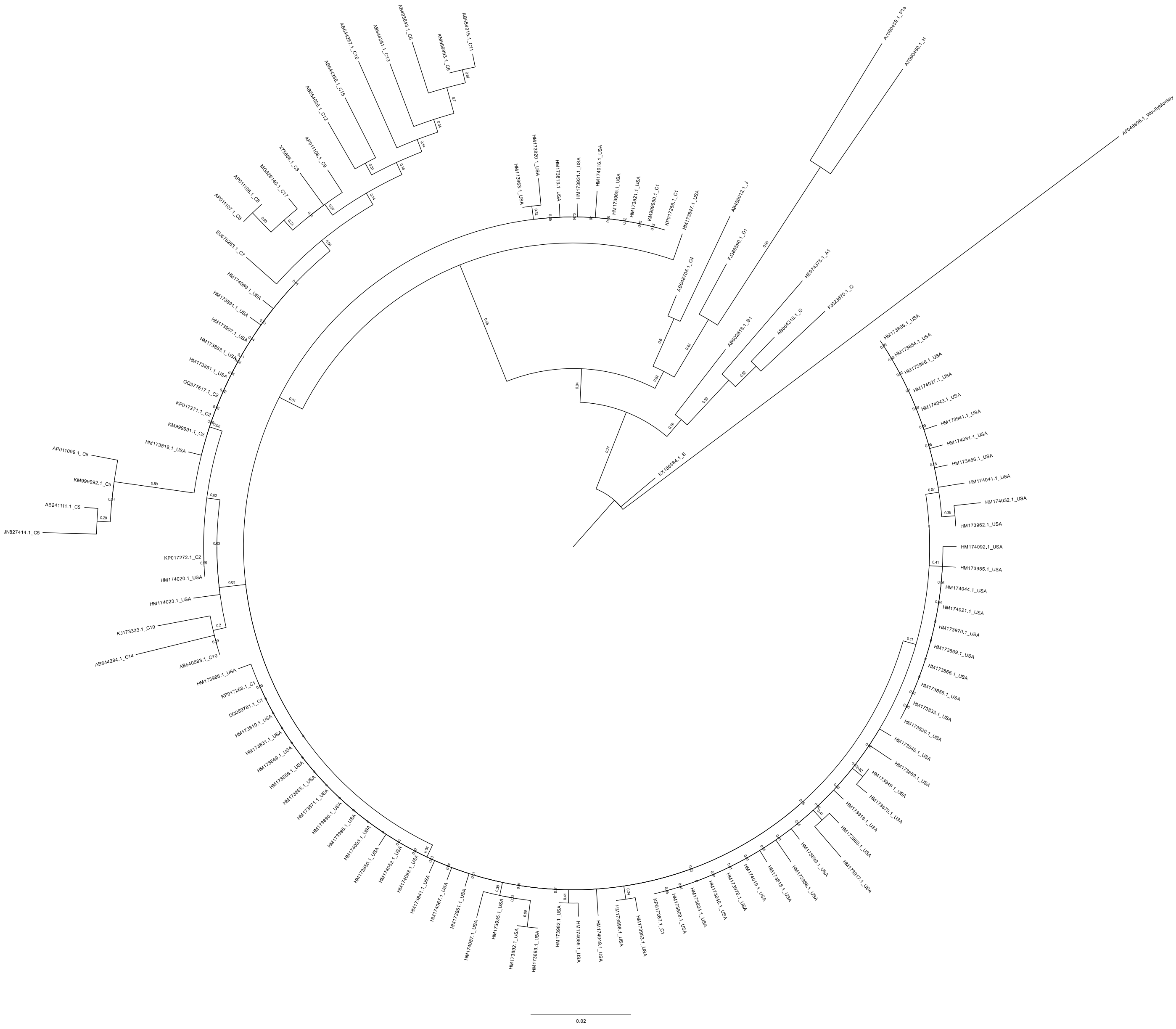

Tree 4. Molecular Phylogenetic analysis by Maximum Likelihood method conducted in MEGA7. The evolutionary history was inferred by using the Maximum Likelihood method based on the Tamura-Nei model with 1000 bootstraps. The tree with the highest log likelihood (-3449.42) is shown. The percentage of trees in which the associated taxa clustered together is shown next to the branches. The tree is drawn to scale, with branch lengths measured in the number of substitutions per site. The analysis involved 117 nucleotide sequences, of which 40 were used as marker sequences to determine the genotype of 77 sequences. All positions containing gaps and missing data were eliminated. There was a total of 377 positions in the final dataset. Additional trees were constructed to determine the subgenotype of each of the sequences.

ID	GENOTYPE	SUBTYPE	COUNTRY	ALIGNMENT[1]	BASE PAIRS
HM173810	C	C1	USA	417-881	465
HM173813	C	C1	USA	417-881	465
HM173818	C	C1	USA	417-881	465
HM173820	C	C1	USA	417-881	465
HM173821	C	C1	USA	417-881	465
HM173824	C	C1	USA	417-881	465

HM173830	C	C1	USA	417-881	465
HM173831	C	C1	USA	417-881	465
HM173833	C	C1	USA	417-881	465
HM173840	C	C1	USA	417-881	465
HM173841	C	C1	USA	417-881	465
HM173848	C	C1	USA	417-881	465
HM173849	C	C1	USA	417-881	465
HM173850	C	C1	USA	417-881	465
HM173854	C	C1	USA	417-881	465
HM173856	C	C1	USA	417-866	450
HM173858	C	C1	USA	417-881	465
HM173859	C	C1	USA	417-881	465
HM173861	C	C1	USA	417-866	450
HM173865	C	C1	USA	417-881	465
HM173866	C	C1	USA	417-881	465
HM173869	C	C1	USA	417-881	465
HM173870	C	C1	USA	417-881	465
HM173871	C	C1	USA	417-881	465
HM173886	C	C1	USA	417-881	465
HM173890	C	C1	USA	417-881	465
HM173892	C	C1	USA	417-881	465
HM173893	C	C1	USA	417-881	465
HM173898	C	C1	USA	417-881	465
HM173899	C	C1	USA	417-881	465
HM173917	C	C1	USA	417-881	465
HM173918	C	C1	USA	417-881	465
HM173931	C	C1	USA	417-881	465
HM173935	C	C1	USA	417-881	465
HM173941	C	C1	USA	417-881	465
HM173949	C	C1	USA	417-905	489
HM173953	C	C1	USA	417-881	465
HM173955	C	C1	USA	417-881	465
HM173956	C	C1	USA	417-881	465
HM173958	C	C1	USA	417-881	465
HM173960	C	C1	USA	417-881	465
HM173962	C	C1	USA	417-881	465
HM173963	C	C1	USA	417-881	465
HM173965	C	C1	USA	417-881	465
HM173966	C	C1	USA	417-881	465
HM173970	C	C1	USA	417-881	465
HM173978	C	C1	USA	417-881	465
HM173982	C	C1	USA	435-881	447
HM173986	C	C1	USA	417-866	450
HM173996	C	C1	USA	417-881	465
HM174003	C	C1	USA	417-881	465
HM174016	C	C1	USA	417-881	465

HM174019	C	C1	USA	417-881	465
HM174021	C	C1	USA	417-881	465
HM174027	C	C1	USA	417-881	465
HM174032	C	C1	USA	417-881	465
HM174041	C	C1	USA	417-881	465
HM174043	C	C1	USA	417-881	465
HM174044	C	C1	USA	417-881	465
HM174049	C	C1	USA	417-881	465
HM174052	C	C1	USA	417-881	465
HM174059	C	C1	USA	417-881	465
HM174067	C	C1	USA	417-881	465
HM174081	C	C1	USA	417-881	465
HM174087	C	C1	USA	417-881	465
HM174092	C	C1	USA	417-881	465
HM174093	C	C1	USA	417-881	465
HM173809	C	C1	USA	417-881	465
HM173819	C	C2	USA	417-881	465
HM173847	C	C1	USA	417-881	465
HM173851	C	C2	USA	417-881	465
HM173863	C	C2	USA	417-881	465
HM173891	C	C2	USA	417-881	465
HM173907	C	C2	USA	417-881	465
HM174020	C	C2	USA	417-881	465
HM174023	C	C2	USA	417-881	465
HM174069	C	C2	USA	417-881	465

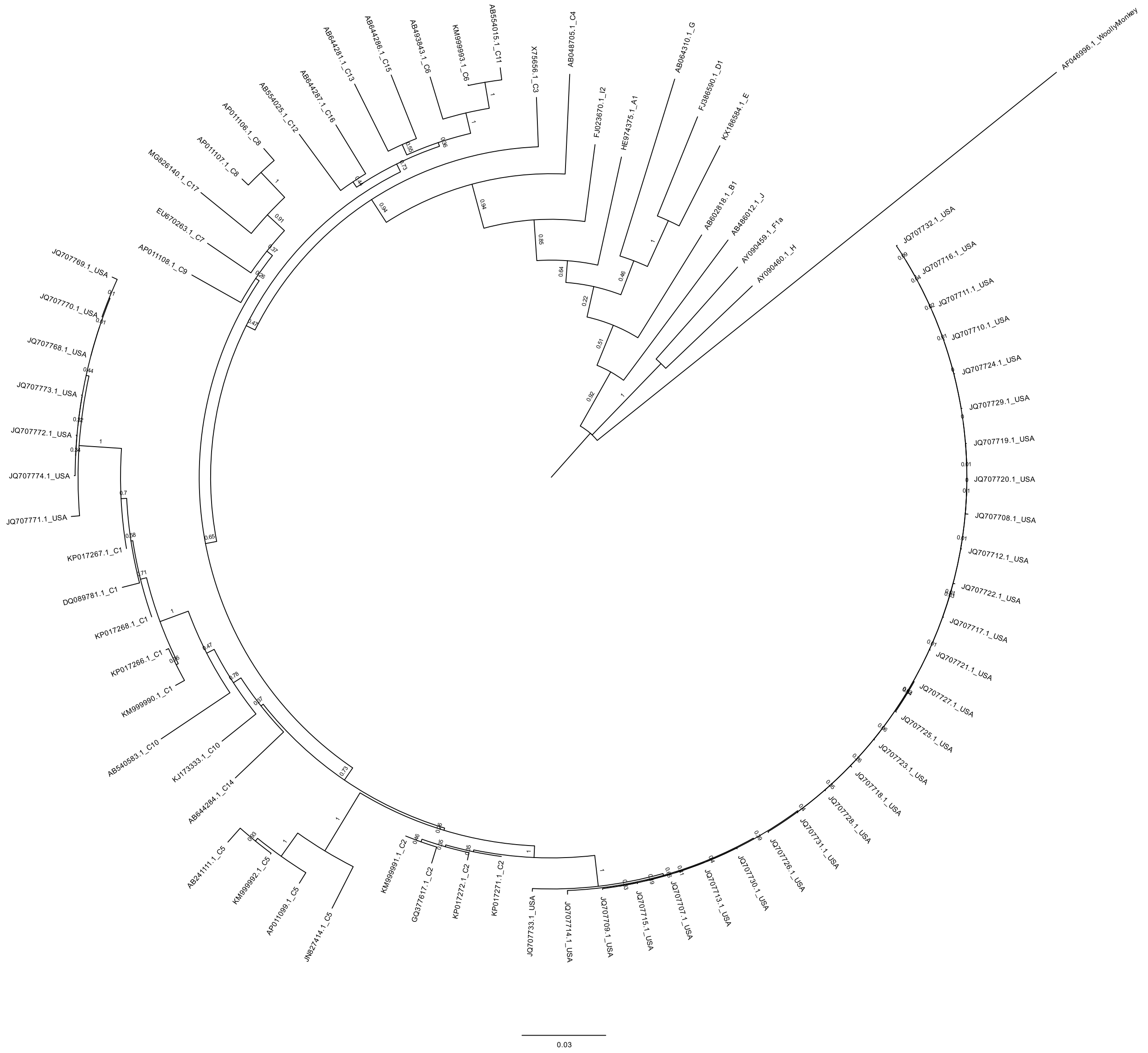

Tree 5. Molecular Phylogenetic analysis by Maximum Likelihood method conducted in MEGA7. The evolutionary history was inferred by using the Maximum Likelihood method based on the Tamura-Nei model with 1000 bootstraps. The tree with the highest log likelihood (-24431.62) is shown. The percentage of trees in which the associated taxa clustered together is shown next to the branches. The tree is drawn to scale, with branch lengths measured in the number of substitutions per site. The analysis involved 74 nucleotide sequences, of which 40 were used as marker sequences to determine the genotype of 34 sequences. All positions containing gaps and missing data were eliminated. There was a total of 2997 positions in the final dataset. Additional trees were constructed to determine the subgenotype of each of the sequences.

ID	GENOTYPE	SUBTYPE	COUNTRY	ALIGNMENT[1]	BASE PAIRS
JQ707707	C	C2	USA	Complete Genome	3161
JQ707708	C	C2	USA	Complete Genome	3161
JQ707709	C	C2	USA	Complete Genome	3161
JQ707710	C	C2	USA	Complete Genome	3161

JQ707711	C	C2	USA	Complete Genome	3161
JQ707712	C	C2	USA	Complete Genome	3161
JQ707713	C	C2	USA	Complete Genome	3161
JQ707714	C	C2	USA	Complete Genome	3161
JQ707715	C	C2	USA	Complete Genome	3161
JQ707716	C	C2	USA	Complete Genome	3161
JQ707717	C	C2	USA	Complete Genome	3161
JQ707718	C	C2	USA	Complete Genome	3161
JQ707719	C	C2	USA	Complete Genome	3161
JQ707720	C	C2	USA	Complete Genome	3161
JQ707721	C	C2	USA	Complete Genome	3161
JQ707722	C	C2	USA	Complete Genome	3161
JQ707723	C	C2	USA	Complete Genome	3161
JQ707724	C	C2	USA	Complete Genome	3161
JQ707725	C	C2	USA	Complete Genome	3161
JQ707726	C	C2	USA	Complete Genome	3161
JQ707727	C	C2	USA	Complete Genome	3161
JQ707728	C	C2	USA	Complete Genome	3161
JQ707729	C	C2	USA	Complete Genome	3161
JQ707730	C	C2	USA	Complete Genome	3161
JQ707731	C	C2	USA	Complete Genome	3161
JQ707732	C	C2	USA	Complete Genome	3161
JQ707733	C	C2	USA	Complete Genome	3161
JQ707768	C	C1	USA	Complete Genome	3191
JQ707769	C	C1	USA	Complete Genome	3191
JQ707770	C	C1	USA	Complete Genome	3191
JQ707771	C	C1	USA	Complete Genome	3104
JQ707772	C	C1	USA	Complete Genome	3191
JQ707773	C	C1	USA	Complete Genome	3191
JQ707774	C	C1	USA	Complete Genome	3191

D - SUBGENOTYPE

ARGENTINA

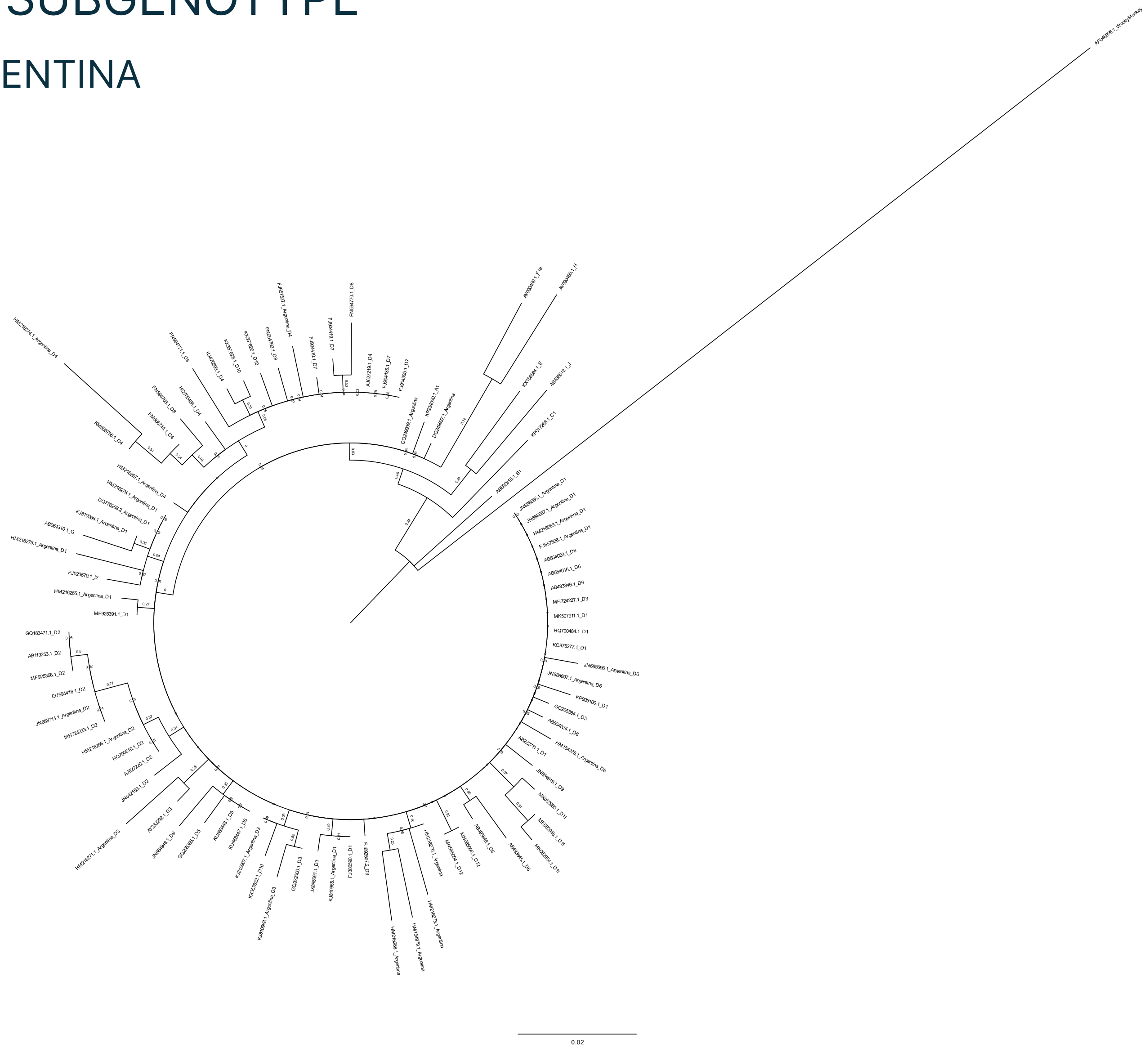

Tree 1. The evolutionary history was inferred by using the Maximum Likelihood method and Tamura-Nei model. The percentage of replicate trees in which the associated taxa clustered together in the bootstrap test (1000 replicates) are shown next to the branches. Initial tree(s) for the heuristic search were obtained automatically by applying Neighbor-Join and BioNJ algorithms to a matrix of pairwise distances estimated using the Tamura-Nei model, and then selecting the topology with superior log likelihood value. A discrete Gamma distribution was used to model evolutionary rate differences among sites (5 categories (+G, parameter = 0.2540)). The tree is drawn to scale, with branch lengths measured in the number of substitutions per site. The analysis involved 90 nucleotide sequences, of which 63 were used as marker sequences to determine the genotype of 27 sequences. All positions containing gaps and missing data were eliminated. There was a total of 400 positions in the final dataset. Evolutionary analyses were conducted in MEGA X.

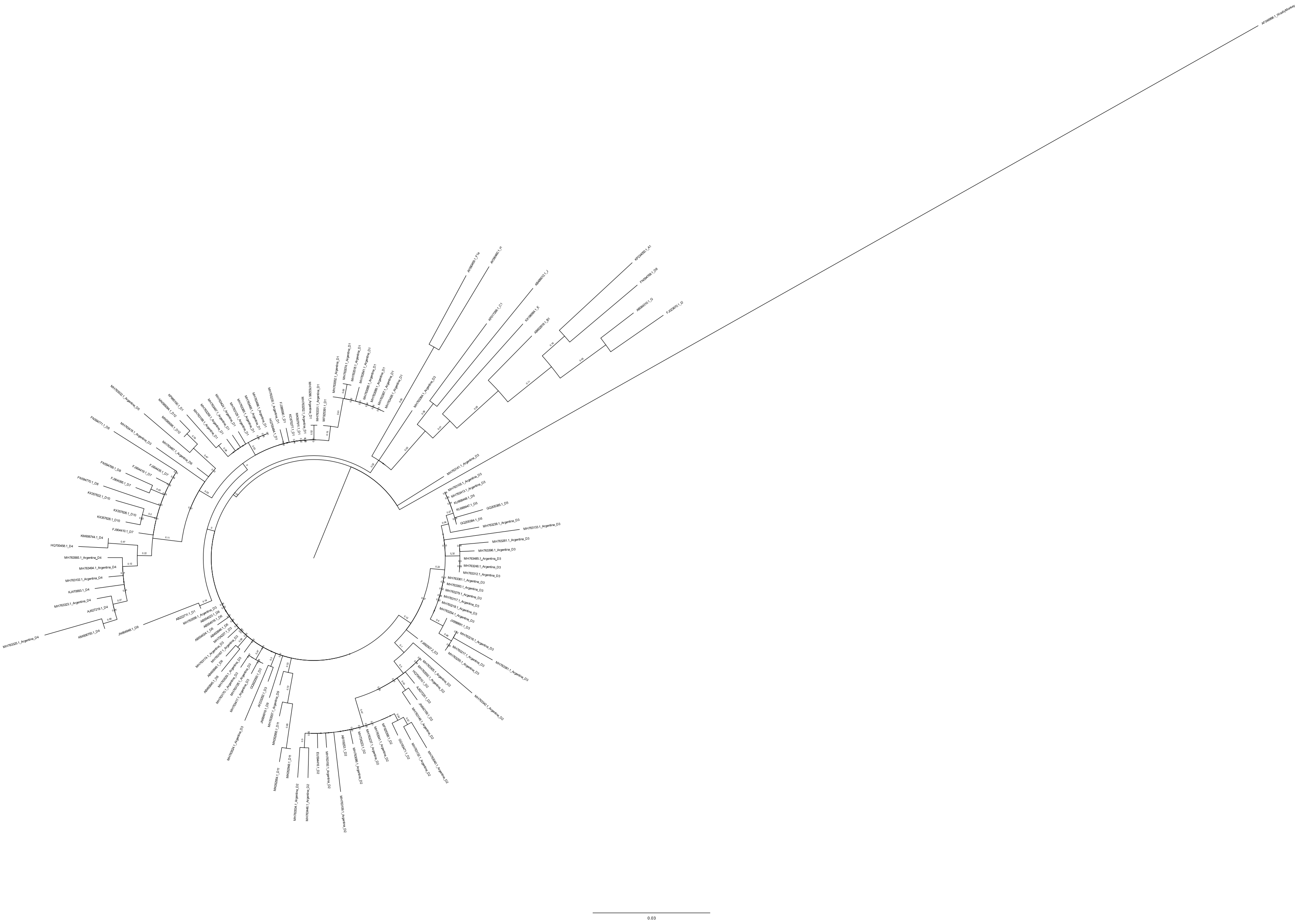

Tree 2. The evolutionary history was inferred by using the Maximum Likelihood method and Tamura-Nei model. The percentage of replicate trees in which the associated taxa clustered together in the bootstrap test (1000 replicates) are shown next to the branches. Initial tree(s) for the heuristic search were obtained automatically by applying Neighbor-Join and BioNJ algorithms to a matrix of pairwise distances estimated using the Tamura-Nei model, and then selecting the topology with superior log likelihood value. A discrete Gamma distribution was used to model evolutionary rate differences among sites (5 categories (+G, parameter = 0.2128)). The tree is drawn to scale, with branch lengths measured in the number of substitutions per site. The analysis involved 134 nucleotide sequences, of which 63 were used as marker sequences to determine the genotype of 71 sequences. All positions containing gaps and missing data were eliminated. There was a total of 362 positions in the final dataset. Evolutionary analyses were conducted in MEGA X.

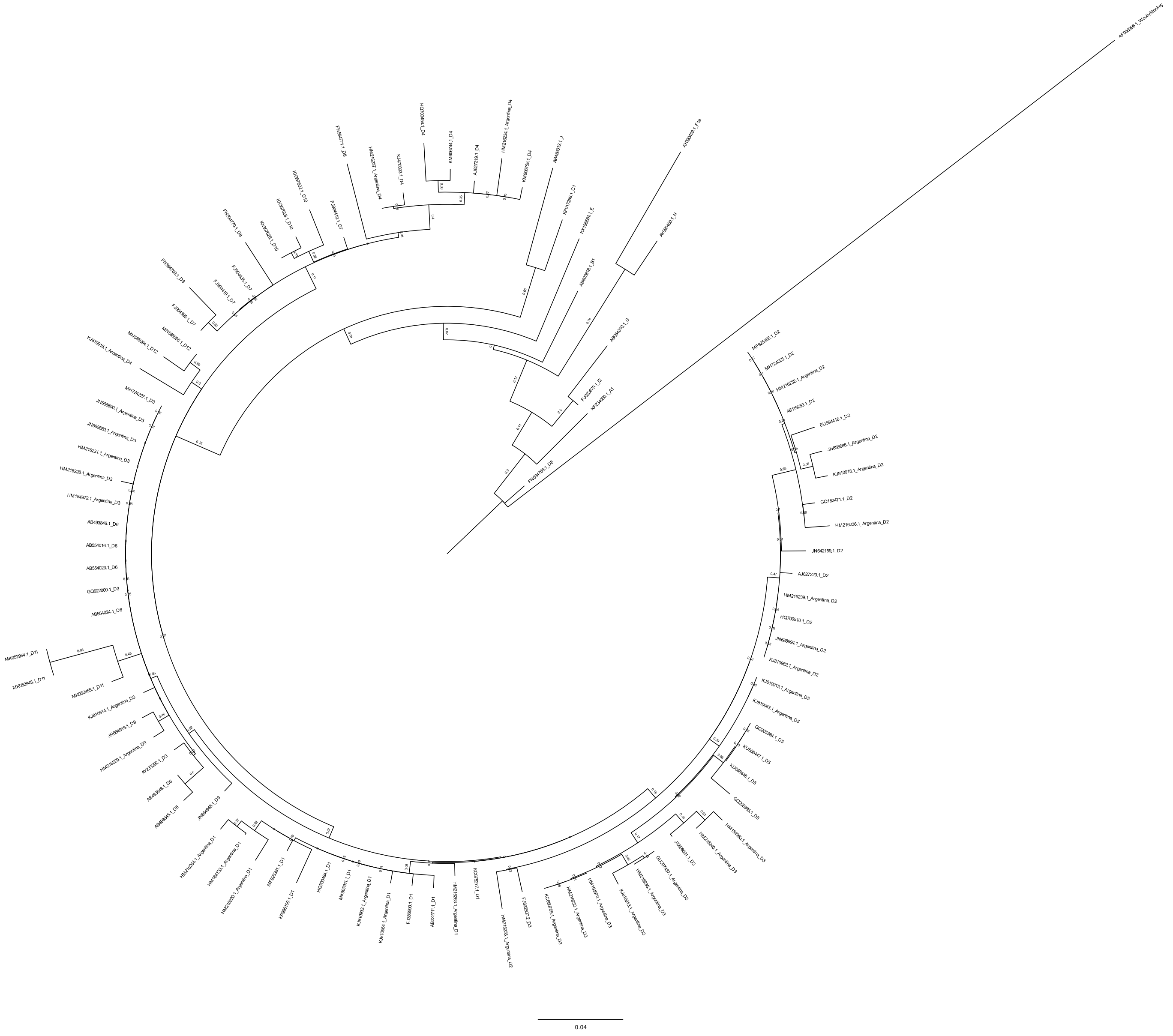

Tree 3. The evolutionary history was inferred by using the Maximum Likelihood method and Tamura-Nei model. The percentage of replicate trees in which the associated taxa clustered together in the bootstrap test (1000 replicates) are shown next to the branches. Initial tree(s) for the heuristic search were obtained automatically by applying Neighbor-Join and BioNJ algorithms to a matrix of pairwise distances estimated using the Tamura-Nei model, and then selecting the topology with superior log likelihood value. A discrete Gamma distribution was used to model evolutionary rate differences among sites (5 categories (+G, parameter = 0.2006)). The tree is drawn to scale, with branch lengths measured in the number of substitutions per site. The analysis involved 97 nucleotide sequences, of which 63 were used as marker sequences to determine the genotype of 34 sequences. All positions containing gaps and missing data were eliminated. There was a total of 250 positions in the final dataset. Evolutionary analyses were conducted in MEGA X.

ID	GENOTYPE	SUBTYPE	COUNTRY	TREE	ALIGNMENT[1]	BASE PAIRS
DQ249937	D	D2	Argentina	1	205-789	585
DQ249939	D	D2	Argentina	1	205-789	585
DQ776268	D	D1	Argentina	1	244-768	525
FJ657526	D	D3	Argentina	1	244-768	525

FJ657527	D	D4	Argentina	1	244-768	525
HM154975	D	D6	Argentina	1	244-747	504
HM154979	D	D12	Argentina	1	244-747	504
HM216265	D	D1	Argentina	1	259-766	508
HM216266	D	D2	Argentina	1	259-766	508
HM216267	D	D4	Argentina	1	259-766	508
HM216268	D	D8	Argentina	1	259-766	508
HM216269	D	D5	Argentina	1	259-766	508
HM216270	D	D1	Argentina	1	259-766	508
HM216271	D	D3	Argentina	1	259-759	501
HM216273	D	D12	Argentina	1	259-766	508
HM216274	D	D4	Argentina	1	259-766	508
HM216275	D	D2	Argentina	1	259-764	506
HM216276	D	D1	Argentina	1	259-766	508
JN688686	D	D3/D6	Argentina	1	217-771	555
JN688687	D	D3/D6	Argentina	1	217-771	555
JN688696	D	D3/D6	Argentina	1	217-771	555
JN688697	D	D3/D6	Argentina	1	217-771	555
JN688714	D	D2	Argentina	1	217-771	555
KJ810965	D	D3	Argentina	1	253-1002	750
KJ810966	D	D1	Argentina	1	311-1015	705
KJ810967	D	D3	Argentina	1	313-960	648
KJ810968	D	D3	Argentina	1	267-1007	741
MH763052	D	D1	Argentina	2	364-756	393
MH763074	D	D1	Argentina	2	364-756	393
MH763088	D	D1	Argentina	2	364-756	393
MH763089	D	D1	Argentina	2	364-756	393
MH763129	D	D1	Argentina	2	364-756	393
MH763188	D	D1	Argentina	2	364-756	393
MH763235	D	D1	Argentina	2	364-756	393
MH763252	D	D1	Argentina	2	364-756	393
MH763259	D	D1	Argentina	2	364-756	393
MH763296	D	D1	Argentina	2	364-756	393
MH763331	D	D1	Argentina	2	364-756	393
MH763385	D	D1	Argentina	2	364-756	393
MH763387	D	D1	Argentina	2	364-756	393
MH763424	D	D1	Argentina	2	364-756	393
MH763426	D	D1	Argentina	2	364-756	393
MH763495	D	D1	Argentina	2	364-756	393
MH763496	D	D1	Argentina	2	364-756	393
MH763497	D	D1	Argentina	2	364-756	393
MH763518	D	D1	Argentina	2	364-756	393
MH763541	D	D1	Argentina	2	364-756	393
MH763040	D	D2	Argentina	2	364-756	393
MH763041	D	D2	Argentina	2	364-756	393
MH763086	D	D2	Argentina	2	364-756	393

MH763109	D	D2	Argentina	2	364-756	393
MH763132	D	D2	Argentina	2	364-756	393
MH763148	D	D2	Argentina	2	364-756	393
MH763192	D	D2	Argentina	2	364-756	393
MH763237	D	D2	Argentina	2	364-756	393
MH763305	D	D2	Argentina	2	364-756	393
MH763342	D	D2	Argentina	2	364-756	393
MH763446	D	D2	Argentina	2	364-756	393
MH763534	D	D2	Argentina	2	364-756	393
MH763552	D	D2	Argentina	2	364-756	393
MH763105	D	D3	Argentina	2	364-756	393
MH763117	D	D3	Argentina	2	364-756	393
MH763126	D	D3	Argentina	2	364-756	393
MH763133	D	D3	Argentina	2	364-756	393
MH763141	D	D3	Argentina	2	364-756	393
MH763167	D	D3/D6	Argentina	2	364-756	393
MH763170	D	D3	Argentina	2	364-756	393
MH763174	D	D3/D6	Argentina	2	364-756	393
MH763216	D	D3	Argentina	2	364-756	393
MH763217	D	D3	Argentina	2	364-756	393
MH763218	D	D3	Argentina	2	364-756	393
MH763238	D	D3	Argentina	2	364-756	393
MH763248	D	D3	Argentina	2	364-756	393
MH763254	D	D3	Argentina	2	364-756	393
MH763255	D	D3	Argentina	2	364-756	393
MH763279	D	D3	Argentina	2	364-756	393
MH763281	D	D3	Argentina	2	364-756	393
MH763312	D	D3	Argentina	2	364-756	393
MH763361	D	D3	Argentina	2	364-756	393
MH763364	D	D3	Argentina	2	364-756	393
MH763381	D	D3	Argentina	2	364-756	393
MH763393	D	D3	Argentina	2	364-756	393
MH763396	D	D3	Argentina	2	364-756	393
MH763413	D	D3	Argentina	2	364-756	393
MH763417	D	D3	Argentina	2	364-756	393
MH763418	D	D3	Argentina	2	364-756	393
MH763485	D	D3	Argentina	2	364-756	393
MH763524	D	D3	Argentina	2	364-756	393
MH763529	D	D3/D6	Argentina	2	364-756	393
MH763556	D	D3/D6	Argentina	2	364-756	393
MH763102	D	D4	Argentina	2	364-756	393
MH763323	D	D4	Argentina	2	364-756	393
MH763325	D	D4	Argentina	2	364-756	393
MH763494	D	D4	Argentina	2	364-756	393
MH763565	D	D4	Argentina	2	364-756	393
MH763532	D	D12	Argentina	2	364-756	393

MH763487	D	NA	Argentina	2	364-756	393
MH763557	D	D3/D6	Argentina	2	364-756	393
GU207487	D	D3	Argentina	3	244-768	525
HM154963	D	D3	Argentina	3	244-747	504
HM154970	D	D3	Argentina	3	244-747	504
HM154972	D	D3	Argentina	3	244-747	504
HM164133	D	D1	Argentina	3	249-768	520
HM216228	D	D3	Argentina	3	259-766	508
HM216229	D	D9	Argentina	3	259-766	508
HM216230	D	D1	Argentina	3	259-766	508
HM216231	D	D3	Argentina	3	259-766	508
HM216232	D	D2	Argentina	3	259-766	508
HM216233	D	D3	Argentina	3	259-766	508
HM216234	D	D4	Argentina	3	259-766	508
HM216235	D	D3	Argentina	3	259-766	508
HM216236	D	D2	Argentina	3	259-766	508
HM216237	D	D4	Argentina	3	259-766	508
HM216238	D	D3	Argentina	3	259-766	508
HM216239	D	D2	Argentina	3	259-766	508
HM216240	D	D3	Argentina	3	259-766	508
HM216263	D	D1	Argentina	3	259-766	508
HM216264	D	D1	Argentina	3	259-766	508
JN688680	D	D3	Argentina	3	217-771	555
JN688688	D	D2	Argentina	3	217-771	555
JN688690	D	D3	Argentina	3	217-771	555
JN688694	D	D2	Argentina	3	217-771	555
KC680759	D	D3	Argentina	3	244-768	525
KJ810913	D	D3	Argentina	3	313-904	592
KJ810914	D	D9	Argentina	3	312-1005	694
KJ810915	D	D5	Argentina	3	313-1202	890
KJ810916	D	D12	Argentina	3	243-803	561
KJ810918	D	D2	Argentina	3	256-774	519
KJ810933	D	D1	Argentina	3	473-948	476
KJ810962	D	D2	Argentina	3	256-769	514
KJ810963	D	D5	Argentina	3	256-956	701
KJ810964	D	D1	Argentina	3	295-951	657

[1] Alignment to complete reference sequence: GenBank accession number NC_003977.2

BRAZIL

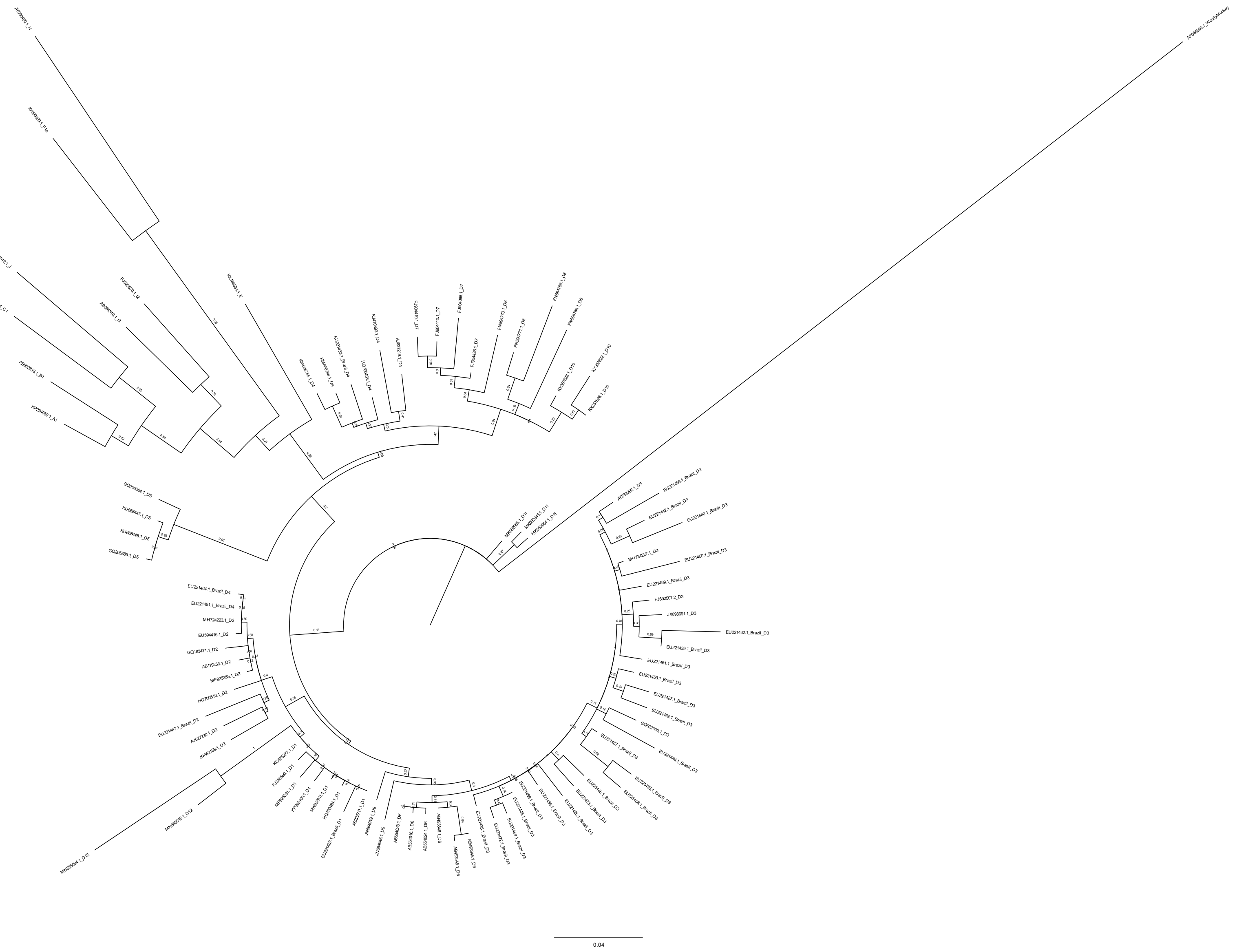

Tree 1. The evolutionary history was inferred by using the Maximum Likelihood method and Tamura-Nei model. The percentage of replicate trees in which the associated taxa clustered together in the bootstrap test (1000 replicates) are shown next to the branches. Initial tree(s) for the heuristic search were obtained automatically by applying Neighbor-Join and BioNJ algorithms to a matrix of pairwise distances estimated using the Tamura-Nei model, and then selecting the topology with superior log likelihood value. A discrete Gamma distribution was used to model evolutionary rate differences among sites (5 categories (+G, parameter = 0.2638)). The tree is drawn to scale, with branch lengths measured in the number of substitutions per site. The analysis involved 92 nucleotide sequences, of which 63 were used as marker sequences to determine the genotype of 29 sequences. All positions containing gaps and missing data were eliminated. There was a total of 476 positions in the final dataset. Evolutionary analyses were conducted in MEGA X.

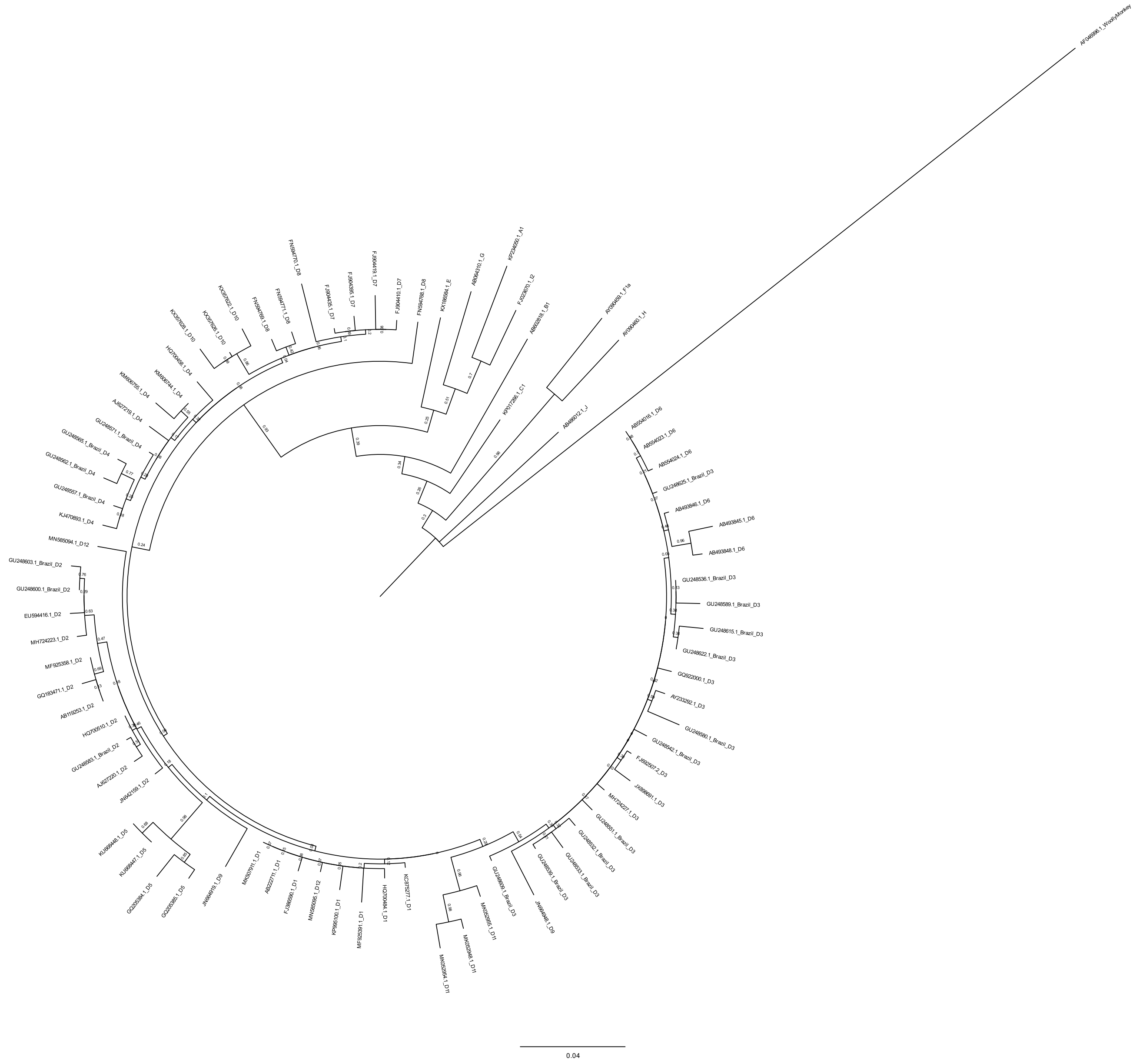

Tree 2. The evolutionary history was inferred by using the Maximum Likelihood method and Tamura-Nei model. The percentage of replicate trees in which the associated taxa clustered together in the bootstrap test (1000 replicates) are shown next to the branches. Initial tree(s) for the heuristic search were obtained automatically by applying Neighbor-Join and BioNJ algorithms to a matrix of pairwise distances estimated using the Tamura-Nei model, and then selecting the topology with superior log likelihood value. A discrete Gamma distribution was used to model evolutionary rate differences among sites (5 categories (+G, parameter = 0.2819)). The tree is drawn to scale, with branch lengths measured in the number of substitutions per site. The analysis involved 82 nucleotide sequences, of which 63 were used as marker sequences to determine the genotype of 19 sequences. All positions containing gaps and missing data were eliminated. There was a total of 635 positions in the final dataset. Evolutionary analyses were conducted in MEGA X.

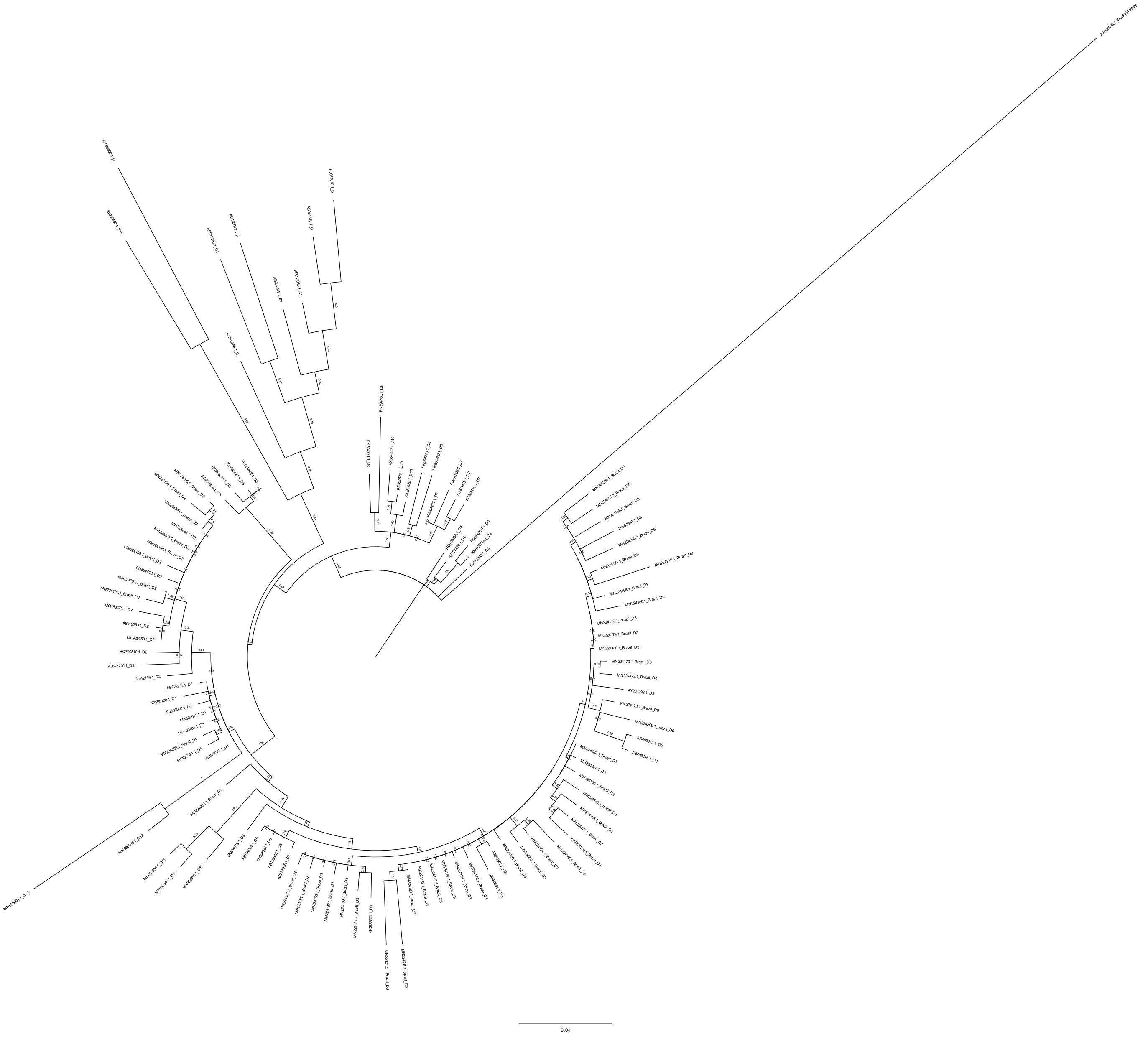

Tree 3. The evolutionary history was inferred by using the Maximum Likelihood method and Tamura-Nei model. The percentage of replicate trees in which the associated taxa clustered together in the bootstrap test (1000 replicates) are shown next to the branches. Initial tree(s) for the heuristic search were obtained automatically by applying Neighbor-Join and BioNJ algorithms to a matrix of pairwise distances estimated using the Tamura-Nei model, and then selecting the topology with superior log likelihood value. A discrete Gamma distribution was used to model evolutionary rate differences among sites (5 categories (+G, parameter = 0.2740)). The tree is drawn to scale, with branch lengths measured in the number of substitutions per site. The analysis involved 112 nucleotide sequences, of which 63 were used as marker sequences to determine the genotype of 49 sequences. All positions containing gaps and missing data were eliminated. There was a total of 464 positions in the final dataset. Evolutionary analyses were conducted in MEGA X.

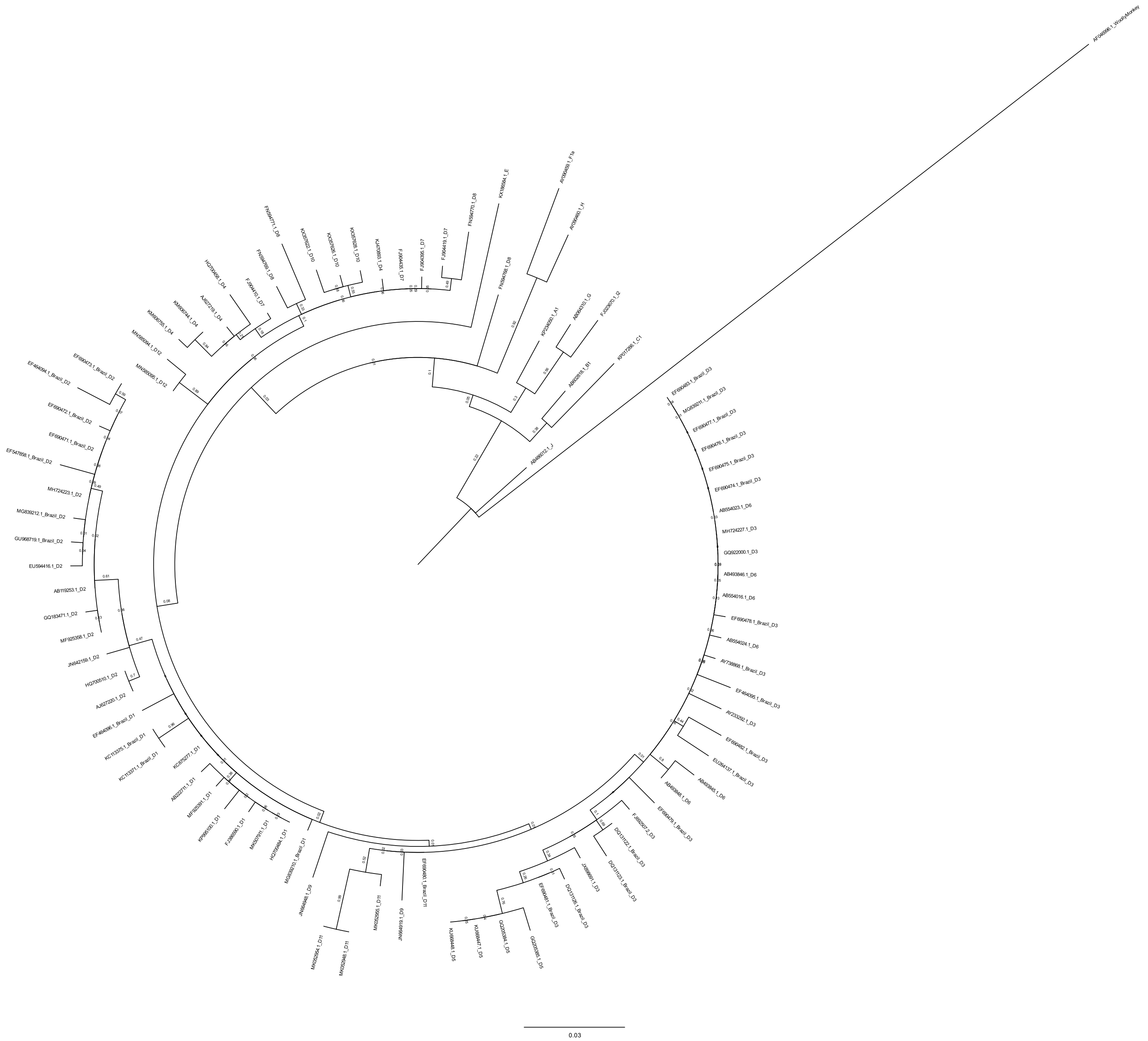

Tree 4. The evolutionary history was inferred by using the Maximum Likelihood method and Tamura-Nei model. The percentage of replicate trees in which the associated taxa clustered together in the bootstrap test (1000 replicates) are shown next to the branches. Initial tree(s) for the heuristic search were obtained automatically by applying Neighbor-Join and BioNJ algorithms to a matrix of pairwise distances estimated using the Tamura-Nei model, and then selecting the topology with superior log likelihood value. A discrete Gamma distribution was used to model evolutionary rate differences among sites (5 categories (+G, parameter = 0.2409)). The tree is drawn to scale, with branch lengths measured in the number of substitutions per site. The analysis involved 91 nucleotide sequences, of which 63 were used as marker sequences to determine the genotype of 28 sequences. All positions containing gaps and missing data were eliminated. There was a total of 331 positions in the final dataset. Evolutionary analyses were conducted in MEGA X.

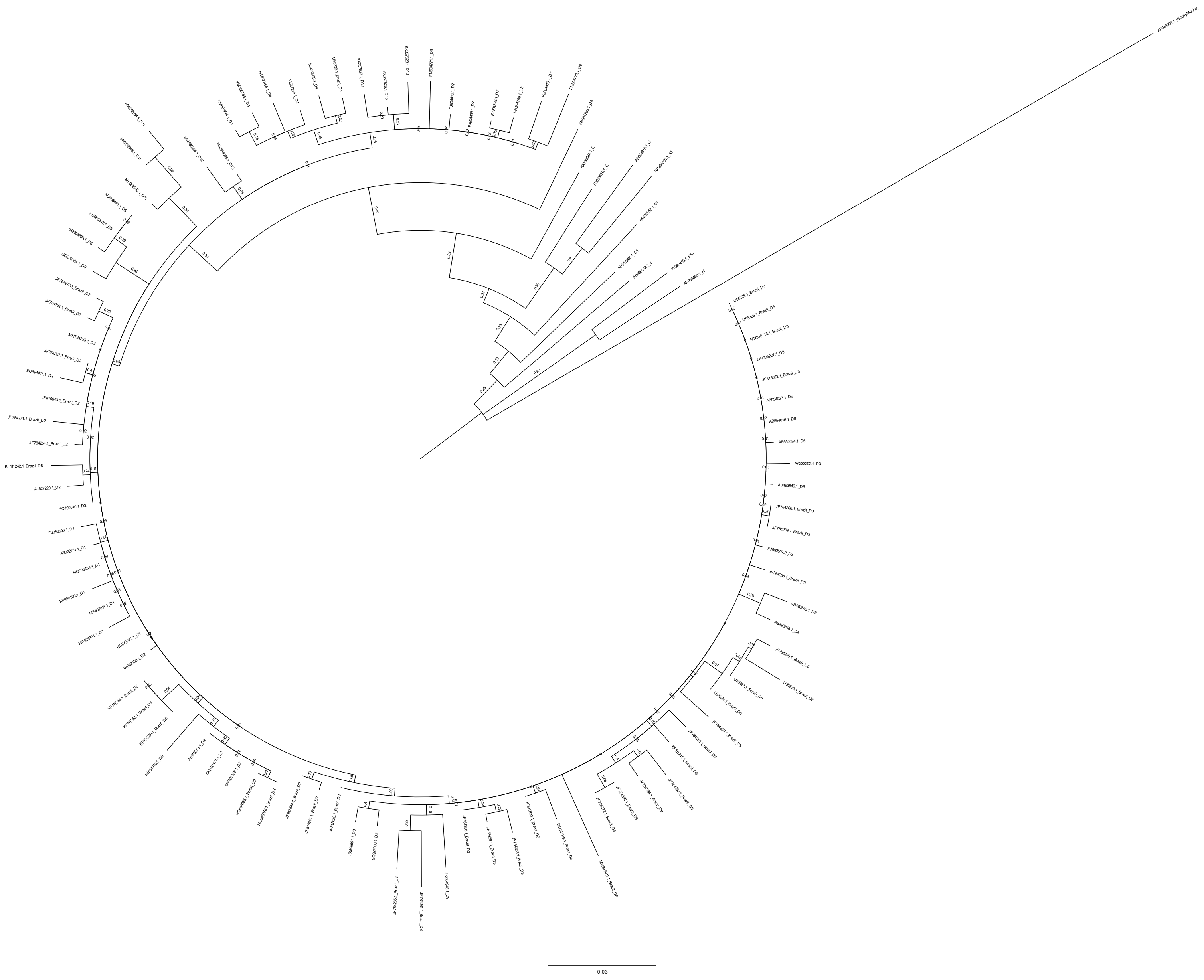

Tree 5. The evolutionary history was inferred by using the Maximum Likelihood method and Tamura-Nei model. The percentage of replicate trees in which the associated taxa clustered together in the bootstrap test (1000 replicates) are shown next to the branches. Initial tree(s) for the heuristic search were obtained automatically by applying Neighbor-Join and BioNJ algorithms to a matrix of pairwise distances estimated using the Tamura-Nei model, and then selecting the topology with superior log likelihood value. A discrete Gamma distribution was used to model evolutionary rate differences among sites (5 categories (+G, parameter = 0.2159)). The tree is drawn to scale, with branch lengths measured in the number of substitutions per site. The analysis involved 105 nucleotide sequences, of which 63 were used as marker sequences to determine the genotype of 42 sequences. All positions containing gaps and missing data were eliminated. There was a total of 581 positions in the final dataset. Evolutionary analyses were conducted in MEGA X.

ID	GENOTYPE	SUBTYPE	COUNTRY	TREE	ALIGNMENT[1]	BASE PAIRS
EU221426	D	D3	Brazil	1	519-997	479
EU221427	D	D3	Brazil	1	519-997	479
EU221428	D	D3	Brazil	1	519-997	479
EU221432	D	D3	Brazil	1	519-997	479

EU221433	D	D4	Brazil	1	519-997	479
EU221435	D	D3	Brazil	1	519-997	479
EU221436	D	D3	Brazil	1	519-997	479
EU221439	D	D3	Brazil	1	519-997	479
EU221442	D	D3	Brazil	1	519-997	479
EU221446	D	D3	Brazil	1	519-997	479
EU221447	D	D2	Brazil	1	519-997	479
EU221448	D	D3	Brazil	1	519-997	479
EU221449	D	D3	Brazil	1	519-997	479
EU221450	D	D3	Brazil	1	519-997	479
EU221451	D	D2	Brazil	1	519-997	479
EU221453	D	D3	Brazil	1	519-997	479
EU221456	D	D3	Brazil	1	519-997	479
EU221457	D	D1	Brazil	1	519-997	479
EU221459	D	D3	Brazil	1	519-997	479
EU221460	D	D3	Brazil	1	519-997	479
EU221461	D	D3	Brazil	1	519-997	479
EU221462	D	D3	Brazil	1	519-997	479
EU221464	D	D2	Brazil	1	519-997	479
EU221466	D	D3	Brazil	1	519-997	479
EU221467	D	D3	Brazil	1	519-997	479
EU221468	D	D3	Brazil	1	519-997	479
EU221469	D	D3	Brazil	1	519-997	479
EU221472	D	D3	Brazil	1	519-997	479
EU221473	D	D3	Brazil	1	519-997	479
GU248532	D	D3	Brazil	2	1-3182	830
GU248533	D	D3	Brazil	2	1-3182	852
GU248536	D	D3	Brazil	2	1-3182	852
GU248539	D	D3	Brazil	2	1-3182	832
GU248542	D	D3	Brazil	2	1-3182	774
GU248551	D	D3	Brazil	2	1-3182	806
GU248557	D	D4	Brazil	2	1-3182	842
GU248562	D	D4	Brazil	2	1-3182	833
GU248565	D	D4	Brazil	2	1-3095	746
GU248571	D	D4	Brazil	2	1-3182	842
GU248580	D	D3	Brazil	2	1-3182	844
GU248583	D	D2	Brazil	2	1-3182	852
GU248589	D	D3	Brazil	2	1-3182	852
GU248600	D	D2	Brazil	2	1-3182	852
GU248603	D	D2	Brazil	2	1-3182	852
GU248609	D	D3	Brazil	2	1-3182	852
GU248615	D	D3	Brazil	2	1-3182	852
GU248622	D	D3	Brazil	2	1-3182	852
GU248625	D	D6	Brazil	2	1-3182	842
MN224165	D	D3	Brazil	3	458-923	466
MN224166	D	D9	Brazil	3	458-923	466

MN224167	D	D3	Brazil	3	458-923	466
MN224168	D	D3	Brazil	3	458-923	466
MN224169	D	D9	Brazil	3	458-923	466
MN224170	D	D3	Brazil	3	458-923	466
MN224171	D	D9	Brazil	3	458-923	466
MN224172	D	D3	Brazil	3	458-923	466
MN224173	D	D6	Brazil	3	458-923	466
MN224174	D	D3	Brazil	3	458-923	466
MN224175	D	D3	Brazil	3	458-923	466
MN224176	D	D3	Brazil	3	458-923	466
MN224177	D	D3	Brazil	3	458-923	466
MN224178	D	D3	Brazil	3	458-923	466
MN224179	D	D3	Brazil	3	458-923	466
MN224180	D	D3	Brazil	3	458-923	466
MN224181	D	D3	Brazil	3	458-923	466
MN224182	D	D3	Brazil	3	458-923	466
MN224183	D	D3	Brazil	3	458-923	466
MN224184	D	D3	Brazil	3	458-923	466
MN224185	D	D3	Brazil	3	458-923	466
MN224186	D	D9	Brazil	3	458-923	466
MN224187	D	D3	Brazil	3	458-923	466
MN224188	D	D3	Brazil	3	458-923	466
MN224189	D	D3	Brazil	3	458-923	466
MN224190	D	D3	Brazil	3	458-923	466
MN224191	D	D3	Brazil	3	458-923	466
MN224192	D	D3	Brazil	3	458-923	466
MN224193	D	D3	Brazil	3	458-923	466
MN224194	D	D3	Brazil	3	458-923	466
MN224195	D	D2	Brazil	3	410-943	534
MN224196	D	D2	Brazil	3	410-943	534
MN224197	D	D2	Brazil	3	410-943	534
MN224198	D	D2	Brazil	3	410-943	534
MN224199	D	D2	Brazil	3	410-943	534
MN224200	D	D2	Brazil	3	410-943	534
MN224201	D	D2	Brazil	3	410-943	534
MN224202	D	D1	Brazil	3	425-933	509
MN224203	D	D1	Brazil	3	425-933	509
MN224204	D	D2	Brazil	3	410-943	534
MN224205	D	D9	Brazil	3	458-923	466
MN224206	D	D9	Brazil	3	458-923	466
MN224207	D	D9	Brazil	3	458-923	466
MN224208	D	D3	Brazil	3	458-923	466
MN224209	D	D6	Brazil	3	458-923	466
MN224210	D	D9	Brazil	3	458-923	466
MN224211	D	D3	Brazil	3	458-923	466
MN224212	D	D3	Brazil	3	458-923	466

MN224213	D	D3	Brazil	3	458-923	466
AY738868	D	D3/D6	Brazil	4	157-837	681
DQ131122	D	D3	Brazil	4	1-847	847
DQ131123	D	D3	Brazil	4	1-847	847
DQ131126	D	D3	Brazil	4	157-838	682
EF464094	D	D2	Brazil	4	417-841	425
EF464095	D	D3/D6	Brazil	4	417-841	425
EF464096	D	D1	Brazil	4	417-841	425
EF547858	D	D2	Brazil	4	157-837	681
EF690471	D	D2	Brazil	4	157-837	681
EF690472	D	D2	Brazil	4	157-837	681
EF690473	D	D2	Brazil	4	157-837	681
EF690474	D	D3/D6	Brazil	4	157-837	681
EF690475	D	D3/D6	Brazil	4	157-837	681
EF690476	D	D3/D6	Brazil	4	157-837	681
EF690477	D	D3/D6	Brazil	4	157-837	681
EF690478	D	D3/D6	Brazil	4	157-837	681
EF690479	D	D3	Brazil	4	157-837	681
EF690480	D	D11	Brazil	4	157-837	681
EF690481	D	D3	Brazil	4	157-837	681
EF690482	D	D3/D6	Brazil	4	157-837	681
EF690483	D	D3/D6	Brazil	4	157-837	681
EU264137	D	D3/D6	Brazil	4	161-756	596
GU968719	D	D2	Brazil	4	312-789	478
KC113371	D	D1	Brazil	4	157-837	681
KC113375	D	D1	Brazil	4	157-837	681
MG839210	D	D1	Brazil	4	157-837	681
MG839211	D	D3/D6	Brazil	4	157-837	681
MG839212	D	D2	Brazil	4	157-837	681
DQ131119	D	D3	Brazil	5	1-3182	1171
HQ646076	D	D2	Brazil	5	1-3182	1178
HQ646085	D	D2	Brazil	5	1-3182	1178
JF784253	D	D9	Brazil	5	1-3182	1154
JF784254	D	D2	Brazil	5	1-3182	1154
JF784255	D	D3	Brazil	5	1-3182	1154
JF784256	D	D9	Brazil	5	1-3182	1154
JF784257	D	D2	Brazil	5	1-3182	1154
JF784258	D	D9	Brazil	5	1-3182	1154
JF784259	D	D9	Brazil	5	1-3182	1154
JF784260	D	D3	Brazil	5	1-3182	1154
JF784261	D	D9	Brazil	5	1-3182	1154
JF784262	D	D2	Brazil	5	1-3182	1154
JF784263	D	D3	Brazil	5	1-3182	1154
JF784264	D	D9	Brazil	5	1-3182	1154
JF784265	D	D9	Brazil	5	1-3182	1154
JF784266	D	D9	Brazil	5	1-3182	1154

JF784267	D	D9	Brazil	5	1-3182	1154
JF784268	D	D3	Brazil	5	1-2849	1131
JF784269	D	D3	Brazil	5	1-3182	1154
JF784270	D	D2	Brazil	5	1-3182	1154
JF784271	D	D2	Brazil	5	1-3182	1070
JF784272	D	D9	Brazil	5	1-3182	1154
JF815622	D	D3	Brazil	5	1-3182	1411
JF815623	D	D3	Brazil	5	1-3182	1308
JF815638	D	D9	Brazil	5	1-3182	1053
JF815641	D	D2	Brazil	5	1-3182	1422
JF815643	D	D2	Brazil	5	133-3138	966
JF815644	D	D2	Brazil	5	1-3182	1425
KF111239	D	D2	Brazil	5	1-3182	1151
KF111240	D	D2	Brazil	5	1-3182	1130
KF111241	D	D9	Brazil	5	1-3182	1151
KF111242	D	D2	Brazil	5	1-3182	1151
KF111244	D	D2	Brazil	5	1-3182	1151
MN310715	D	D3	Brazil	5	1-3182	3182
MN845911	D	D3	Brazil	5	1-3182	1085
U55223	D	D4	Brazil	5	1-3182	1149
U55224	D	D9	Brazil	5	1-3182	1149
U55225	D	D3	Brazil	5	1-3182	1149
U55226	D	D9	Brazil	5	1-3182	1149
U55227	D	D9	Brazil	5	1-3182	1149
U55228	D	D9	Brazil	5	1-3182	1149

[1] Alignment to complete reference sequence: GenBank accession number NC_003977.2

CANADA

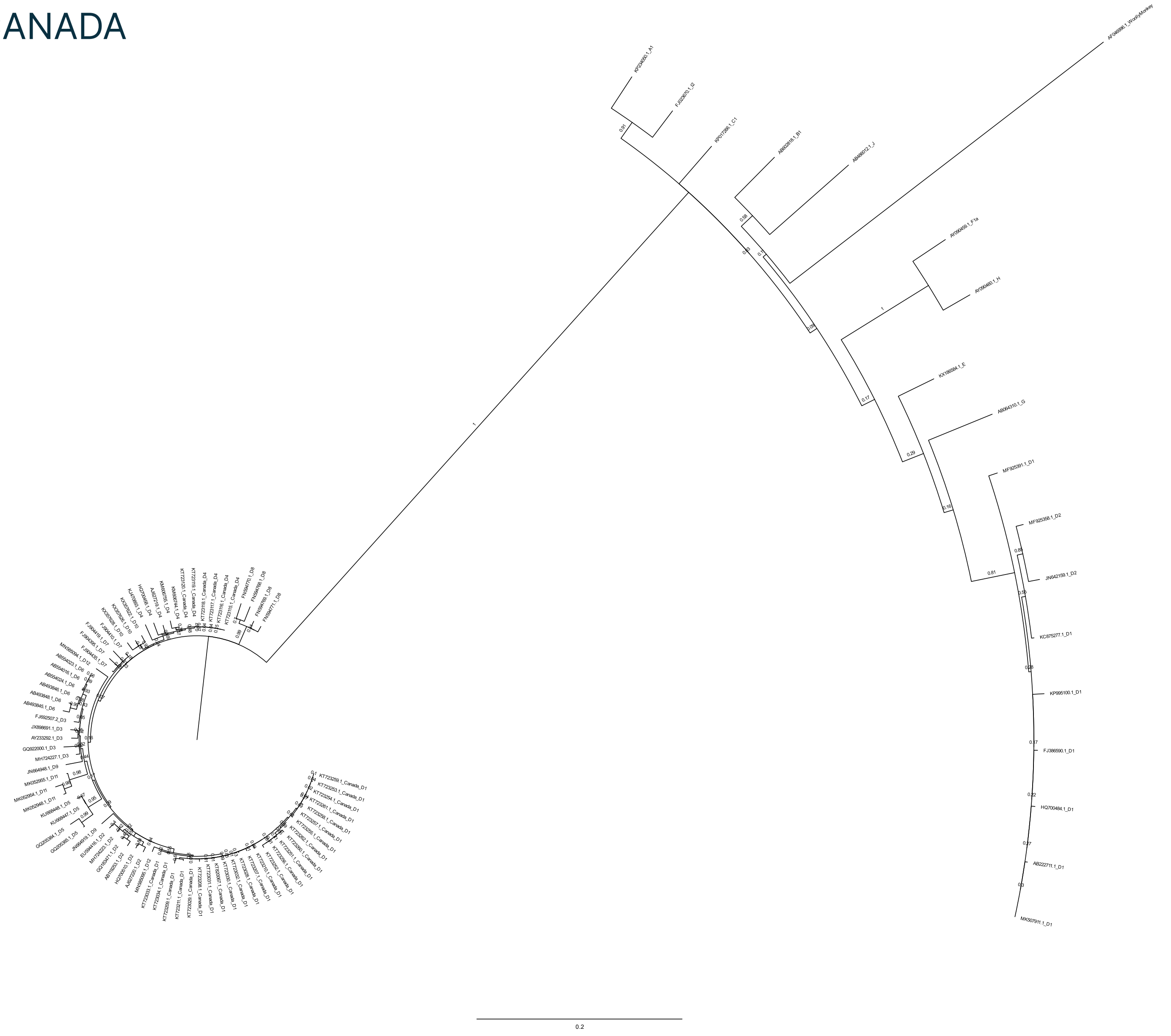

Tree 1. The evolutionary history was inferred by using the Maximum Likelihood method and Tamura-Nei model. The percentage of replicate trees in which the associated taxa clustered together in the bootstrap test (1000 replicates) are shown next to the branches. Initial tree(s) for the heuristic search were obtained automatically by applying Neighbor-Join and BioNJ algorithms to a matrix of pairwise distances estimated using the Tamura-Nei model, and then selecting the topology with superior log likelihood value. A discrete Gamma distribution was used to model evolutionary rate differences among sites (5 categories (+G, parameter = 0.4899)). The tree is drawn to scale, with branch lengths measured in the number of substitutions per site. The analysis involved 94 nucleotide sequences, of which 63 were used as marker sequences to determine the genotype of 31 sequences. All positions containing gaps and missing data were eliminated. There was a total of 872 positions in the final dataset. Evolutionary analyses were conducted in MEGA X.

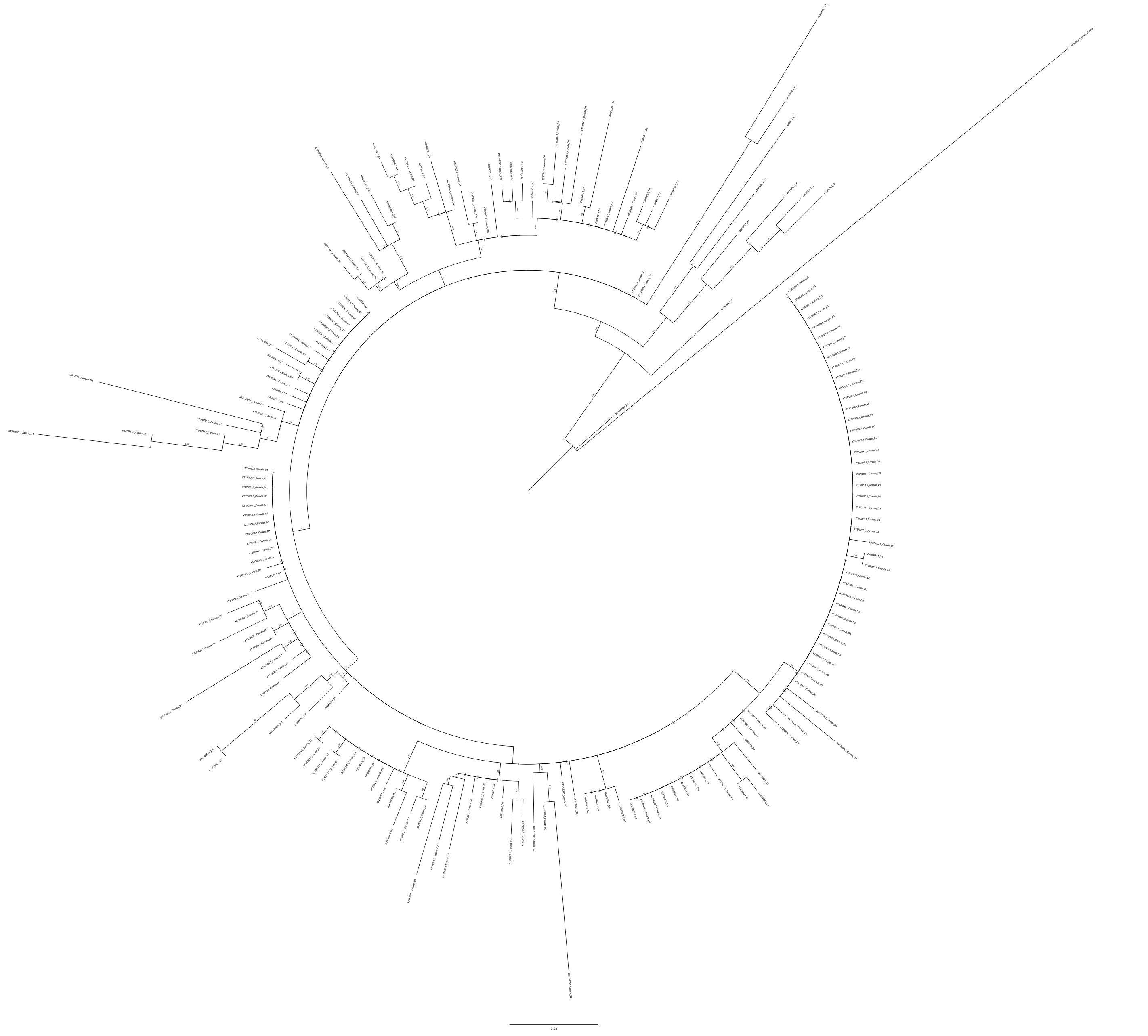

Tree 2. The evolutionary history was inferred by using the Maximum Likelihood method and Tamura-Nei model. The percentage of replicate trees in which the associated taxa clustered together in the bootstrap test (1000 replicates) are shown next to the branches. Initial tree(s) for the heuristic search were obtained automatically by applying Neighbor-Join and BioNJ algorithms to a matrix of pairwise distances estimated using the Tamura-Nei model, and then selecting the topology with superior log likelihood value. A discrete Gamma distribution was used to model evolutionary rate differences among sites (5 categories (+G, parameter = 0.2334)). The tree is drawn to scale, with branch lengths measured in the number of substitutions per site. The analysis involved 188 nucleotide sequences, of which 63 were used as marker sequences to determine the genotype of 125 sequences. All positions containing gaps and missing data were eliminated. There was a total of 269 positions in the final dataset. Evolutionary analyses were conducted in MEGA X.

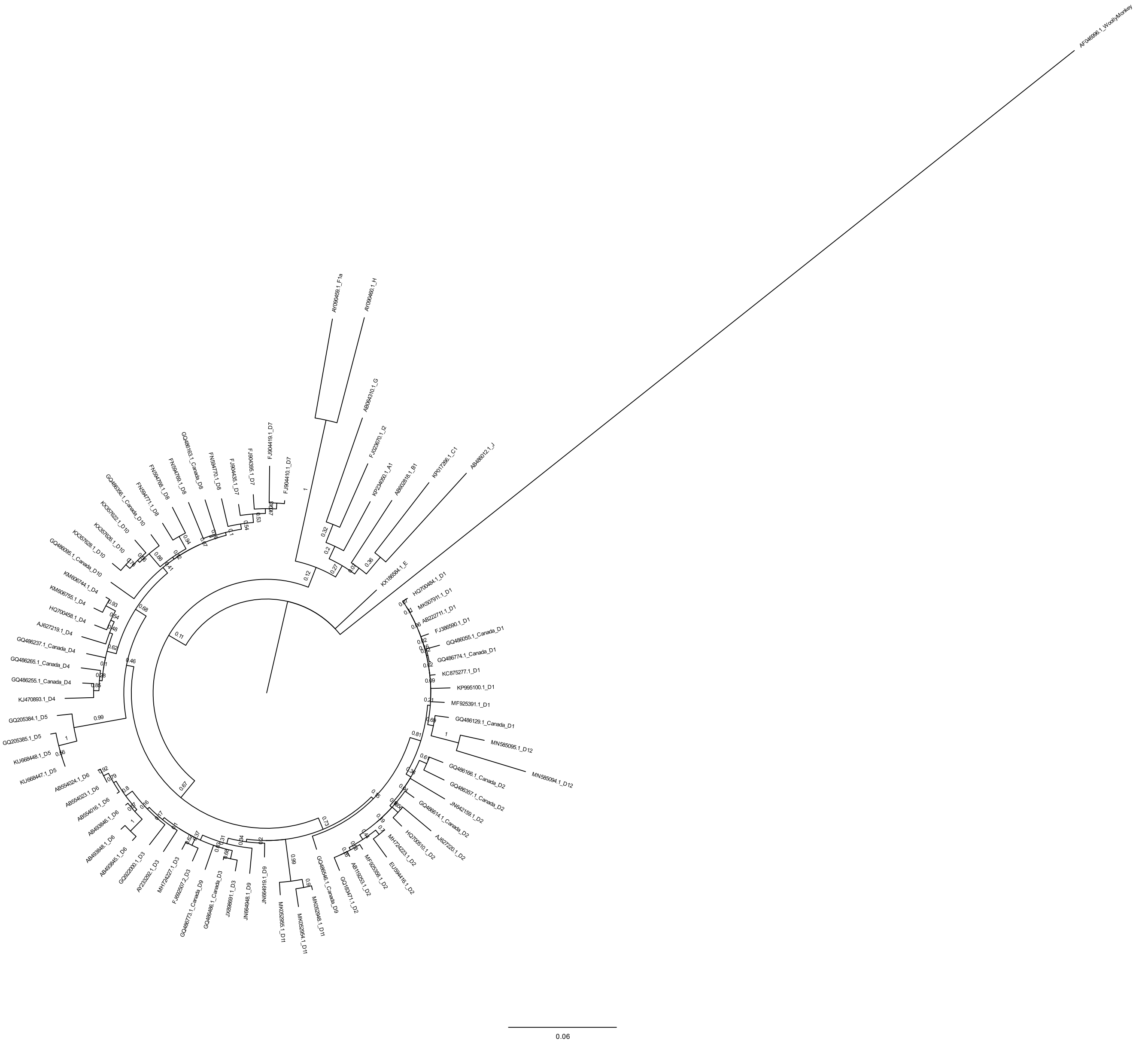

Tree 3. The evolutionary history was inferred by using the Maximum Likelihood method and Tamura-Nei model. The percentage of replicate trees in which the associated taxa clustered together in the bootstrap test (1000 replicates) are shown next to the branches. Initial tree(s) for the heuristic search were obtained automatically by applying Neighbor-Join and BioNJ algorithms to a matrix of pairwise distances estimated using the Tamura-Nei model, and then selecting the topology with superior log likelihood value. A discrete Gamma distribution was used to model evolutionary rate differences among sites (5 categories (+G, parameter = 0.2000)). The tree is drawn to scale, with branch lengths measured in the number of substitutions per site. The analysis involved 78 nucleotide sequences, of which 63 were used as marker sequences to determine the genotype of 15 sequences. All positions containing gaps and missing data were eliminated. There was a total of 958 positions in the final dataset. Evolutionary analyses were conducted in MEGA X.

ID	GENOTYPE	SUBTYPE	COUNTRY	TREE	ALIGNMENT[1]	BASE PAIRS
KT723028	D	D12	Canada	1	1-3182	949
KT723029	D	D12	Canada	1	1-3182	949
KT723030	D	D12	Canada	1	1-3182	949
KT723031	D	D12	Canada	1	1-3182	949

KT723032	D	D12	Canada	1	1-3182	949
KT723033	D	D12	Canada	1	1-3182	949
KT723034	D	D12	Canada	1	1-3182	949
KT723115	D	D4	Canada	1	1-3182	949
KT723116	D	D4	Canada	1	1-3182	949
KT723117	D	D4	Canada	1	1-3182	949
KT723118	D	D4	Canada	1	1-3182	949
KT723119	D	D4	Canada	1	1-3182	949
KT723120	D	D4	Canada	1	1-3182	949
KT723207	D	D12	Canada	1	1-3182	949
KT723208	D	D12	Canada	1	1-3182	949
KT723209	D	D12	Canada	1	1-3182	949
KT723210	D	D12	Canada	1	1-3182	949
KT723211	D	D12	Canada	1	1-3182	949
KT723251	D	D12	Canada	1	1-3182	949
KT723252	D	D12	Canada	1	1-3182	949
KT723253	D	D12	Canada	1	1-3182	949
KT723254	D	D12	Canada	1	1-3182	949
KT723255	D	D12	Canada	1	1-3182	949
KT723256	D	D12	Canada	1	1-3182	949
KT723257	D	D12	Canada	1	1-3182	949
KT723258	D	D12	Canada	1	1-3182	949
KT723259	D	D12	Canada	1	1-3182	949
KT723260	D	D12	Canada	1	1-3182	949
KT723261	D	D12	Canada	1	1-3182	949
KT723262	D	D12	Canada	1	1-3182	921
KT820067	D	D12	Canada	1	1-3182	949
KT370315	D	D1	Canada	2	313-831	519
KT370316	D	D1	Canada	2	313-831	519
KT370317	D	D1	Canada	2	313-831	519
KT370318	D	D1	Canada	2	313-831	519
KT370395	D	D1	Canada	2	357-759	403
KT370786	D	D1	Canada	2	313-831	519
KT370787	D	D1	Canada	2	313-831	519
KT370788	D	D1	Canada	2	313-831	519
KT370789	D	D1	Canada	2	313-831	519
KT370790	D	D1	Canada	2	313-831	519
KT370791	D	D1	Canada	2	313-831	519
KT370792	D	D1	Canada	2	313-831	519
KT370793	D	D1	Canada	2	313-831	519
KT370794	D	D1	Canada	2	313-810	498
KT370795	D	D1	Canada	2	313-831	519
KT370796	D	D1	Canada	2	313-831	519
KT370797	D	D1	Canada	2	313-831	519
KT370798	D	D1	Canada	2	313-831	519
KT370799	D	D1	Canada	2	313-831	519

KT370800	D	D1	Canada	2	313-831	519
KT370801	D	D1	Canada	2	313-831	519
KT370802	D	D1	Canada	2	313-831	519
KT370803	D	D1	Canada	2	313-831	519
KT370804	D	D1	Canada	2	313-831	519
KT370805	D	D12	Canada	2	313-831	519
KT370828	D	D1	Canada	2	313-831	519
KT370829	D	D1	Canada	2	313-831	519
KT370830	D	D1	Canada	2	313-831	519
KT370831	D	D1	Canada	2	313-831	519
KT370832	D	D1	Canada	2	313-831	519
KT370833	D	D1	Canada	2	313-831	519
KT370834	D	D1	Canada	2	313-831	519
KT370835	D	D1	Canada	2	313-831	519
KT370836	D	D1	Canada	2	313-831	519
KT370837	D	D1	Canada	2	313-831	519
KT370838	D	D1	Canada	2	313-831	519
KT370839	D	D1	Canada	2	315-831	517
KT370840	D	D1	Canada	2	313-831	519
KT370841	D	D1	Canada	2	313-831	519
KT370854	D	D1	Canada	2	313-831	519
KT370842	D	D4	Canada	2	313-831	519
KT370843	D	D10	Canada	2	313-831	519
KT370845	D	D10	Canada	2	313-831	519
KT370310	D	D2	Canada	2	313-831	519
KT370311	D	D2	Canada	2	313-831	519
KT370312	D	D2	Canada	2	313-831	519
KT370313	D	D2	Canada	2	313-831	519
KT370314	D	D2	Canada	2	313-831	519
KT370397	D	D2	Canada	2	415-822	408
KT370399	D	D2	Canada	2	466-943	478
KT370817	D	D2	Canada	2	313-831	519
KT370818	D	D2	Canada	2	313-831	519
KT370819	D	D2	Canada	2	313-831	519
KT370820	D	D1	Canada	2	313-831	519
KT370821	D	D2	Canada	2	313-831	519
KT370822	D	D2	Canada	2	313-831	519
KT370823	D	D2	Canada	2	313-831	519
KT370824	D	D2	Canada	2	313-831	519
KT370825	D	D2	Canada	2	313-831	519
KT370826	D	D2	Canada	2	313-831	519
KT370827	D	D2	Canada	2	313-831	519
KT370855	D	D2	Canada	2	313-831	519
KT370856	D	D2	Canada	2	313-831	519
KT370276	D	D3	Canada	2	313-831	519
KT370277	D	D3	Canada	2	313-831	519

KT370278	D	D3	Canada	2	313-831	519
KT370279	D	D3	Canada	2	313-831	519
KT370280	D	D3	Canada	2	313-831	519
KT370281	D	D3	Canada	2	313-831	519
KT370282	D	D3	Canada	2	313-831	519
KT370283	D	D3	Canada	2	313-831	519
KT370284	D	D3	Canada	2	313-831	519
KT370285	D	D3	Canada	2	313-831	519
KT370286	D	D3	Canada	2	313-831	519
KT370287	D	D3	Canada	2	313-831	519
KT370288	D	D3	Canada	2	313-831	519
KT370289	D	D3	Canada	2	313-831	519
KT370290	D	D3	Canada	2	313-831	519
KT370291	D	D3	Canada	2	313-831	519
KT370292	D	D3	Canada	2	313-831	519
KT370293	D	D3	Canada	2	313-831	519
KT370294	D	D3	Canada	2	313-831	519
KT370295	D	D3	Canada	2	313-831	519
KT370296	D	D3	Canada	2	313-831	519
KT370297	D	D3	Canada	2	313-831	519
KT370298	D	D3	Canada	2	313-831	519
KT370299	D	D3	Canada	2	313-831	519
KT370300	D	D3	Canada	2	313-831	519
KT370301	D	D3	Canada	2	313-831	519
KT370302	D	D3	Canada	2	313-831	519
KT370303	D	D3	Canada	2	313-831	519
KT370304	D	D3	Canada	2	313-831	519
KT370305	D	D3	Canada	2	313-831	519
KT370306	D	D3	Canada	2	313-831	519
KT370307	D	D3	Canada	2	313-831	519
KT370308	D	D3	Canada	2	313-831	519
KT370309	D	D3	Canada	2	313-831	519
KT370392	D	D3/D6	Canada	2	313-831	519
KT370398	D	D3	Canada	2	455-931	477
KT370806	D	D3	Canada	2	313-831	519
KT370807	D	D3	Canada	2	313-831	519
KT370808	D	D3	Canada	2	313-831	519
KT370809	D	D3	Canada	2	313-831	519
KT370810	D	D3	Canada	2	313-831	519
KT370811	D	D3	Canada	2	313-831	519
KT370812	D	D3	Canada	2	313-831	519
KT370813	D	D3	Canada	2	313-831	519
KT370814	D	D3	Canada	2	313-831	519
KT370815	D	D3/D6	Canada	2	313-831	519
KT370816	D	D3/D6	Canada	2	313-831	519
KT370319	D	D4	Canada	2	313-831	519

KT370320	D	D4	Canada	2	313-831	519
KT370321	D	D4	Canada	2	313-831	519
KT370322	D	D4	Canada	2	313-831	519
KT370846	D	D4	Canada	2	313-831	519
KT370847	D	D4	Canada	2	313-831	519
KT370848	D	D4	Canada	2	313-831	519
KT370849	D	D7	Canada	2	313-831	519
KT370850	D	D4	Canada	2	313-831	519
KT370851	D	D4	Canada	2	313-831	519
KT370852	D	D1	Canada	2	313-831	519
KT370853	D	D12	Canada	2	313-831	519
KT370323	D	D4	Canada	2	313-831	519
KT370324	D	D4	Canada	2	313-831	519
KT370844	D	D7	Canada	2	313-831	519
GQ486055	D	D1	Canada	3	132-1163	1032
GQ486095	D	D10	Canada	3	132-1163	1032
GQ486129	D	D12	Canada	3	132-1163	1032
GQ486163	D	D8	Canada	3	132-1163	1032
GQ486166	D	D2	Canada	3	132-1163	1032
GQ486237	D	D4	Canada	3	132-1163	1032
GQ486255	D	D4	Canada	3	132-1163	1032
GQ486265	D	D4	Canada	3	132-1163	1032
GQ486356	D	D10	Canada	3	132-1163	1032
GQ486357	D	D2	Canada	3	132-1163	1032
GQ486486	D	D3	Canada	3	132-1163	1032
GQ486546	D	D9	Canada	3	132-1163	1032
GQ486614	D	D2	Canada	3	132-1163	1032
GQ486773	D	D3	Canada	3	132-1163	1032
GQ486774	D	D1	Canada	3	132-1163	1032

[1] Alignment to complete reference sequence: GenBank accession number NC_003977.2

CHILE

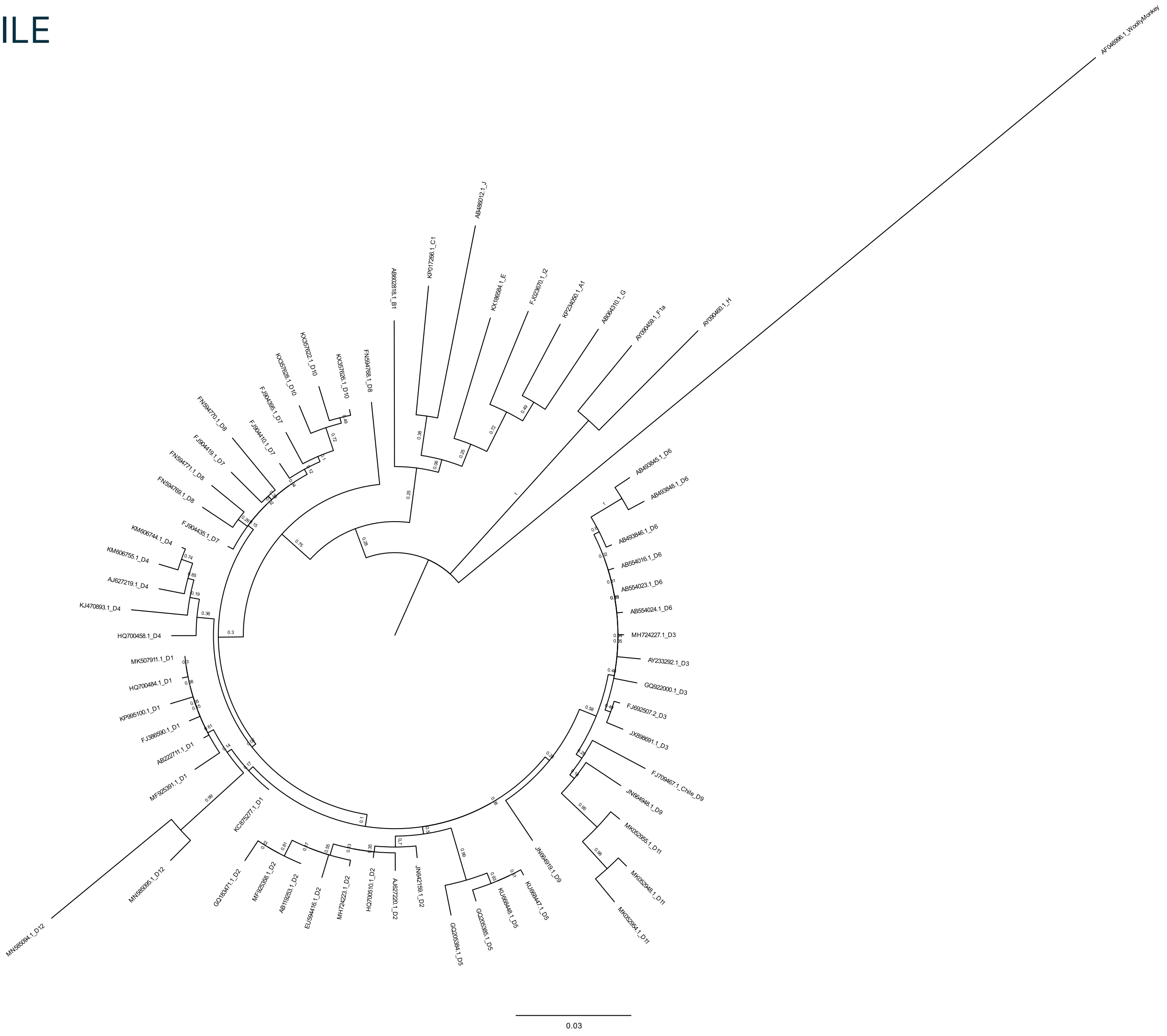

Tree 1. The evolutionary history was inferred by using the Maximum Likelihood method and Tamura-Nei model. The percentage of replicate trees in which the associated taxa clustered together in the bootstrap test (1000 replicates) are shown next to the branches. Initial tree(s) for the heuristic search were obtained automatically by applying Neighbor-Join and BioNJ algorithms to a matrix of pairwise distances estimated using the Tamura-Nei model, and then selecting the topology with superior log likelihood value. A discrete Gamma distribution was used to model evolutionary rate differences among sites (5 categories (+G, parameter = 0.2389)). The tree is drawn to scale, with branch lengths measured in the number of substitutions per site. The analysis involved 64 nucleotide sequences, of which 63 were used as marker sequences to determine the genotype of 1 sequence. All positions containing gaps and missing data were eliminated. There was a total of 749 positions in the final dataset. Evolutionary analyses were conducted in MEGA X.

ID	GENOTYPE	SUBTYPE	COUNTRY	TREE	ALIGNMENT[1]	BASE PAIRS
FJ709467	D	D9	Chile	1	106-858	753

[1] Alignment to complete reference sequence: GenBank accession number NC_003977.2

COLOMBIA

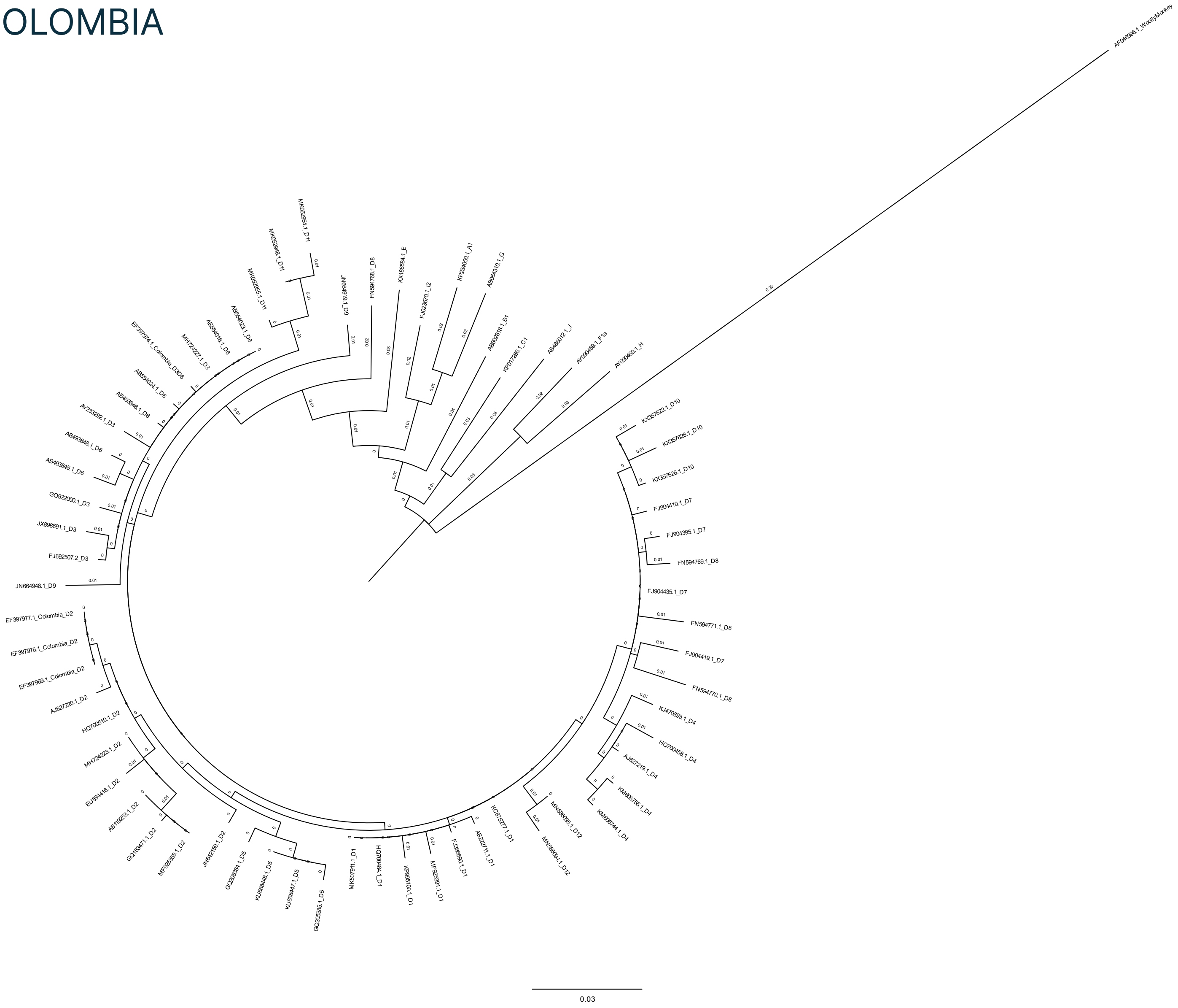

Tree 1. The evolutionary history was inferred by using the Maximum Likelihood method and Tamura-Nei model. The percentage of replicate trees in which the associated taxa clustered together in the bootstrap test (1000 replicates) are shown next to the branches. Initial tree(s) for the heuristic search were obtained automatically by applying Neighbor-Join and BioNJ algorithms to a matrix of pairwise distances estimated using the Tamura-Nei model, and then selecting the topology with superior log likelihood value. A discrete Gamma distribution was used to model evolutionary rate differences among sites (5 categories (+G, parameter = 0.1875)). The tree is drawn to scale, with branch lengths measured in the number of substitutions per site. The analysis involved 67 nucleotide sequences, of which 63 were used as marker sequences to determine the genotype of 4 sequences. All positions containing gaps and missing data were eliminated. There was a total of 595 positions in the final dataset. Evolutionary analyses were conducted in MEGA X.

ID	GENOTYPE	SUBTYPE	COUNTRY	TREE	ALIGNMENT[1]	BASE PAIRS
EF397969	D	D2	Colombia	1	157-753	597
EF397974	D	D3/D6	Colombia	1	157-753	597
EF397976	D	D2	Colombia	1	157-753	597
EF397977	D	D2	Colombia	1	157-753	597

[1] Alignment to complete reference sequence: GenBank accession number NC_003977.2

COSTA RICA

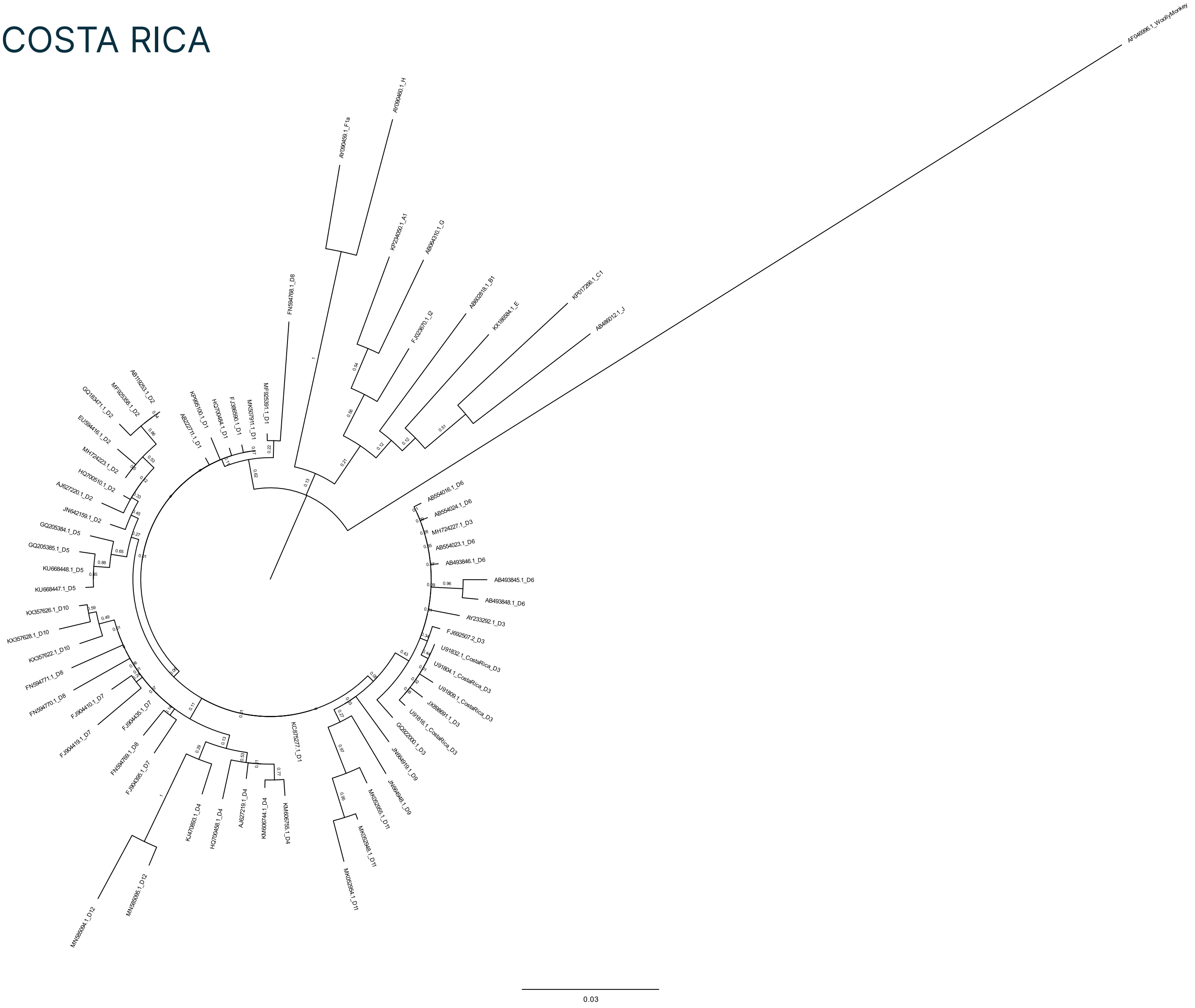

Tree 1. The evolutionary history was inferred by using the Maximum Likelihood method and Tamura-Nei model. The percentage of replicate trees in which the associated taxa clustered together in the bootstrap test (1000 replicates) are shown next to the branches. Initial tree(s) for the heuristic search were obtained automatically by applying Neighbor-Join and BioNJ algorithms to a matrix of pairwise distances estimated using the Tamura-Nei model, and then selecting the topology with superior log likelihood value. A discrete Gamma distribution was used to model evolutionary rate differences among sites (5 categories (+G, parameter = 0.2248)). The tree is drawn to scale, with branch lengths measured in the number of substitutions per site. The analysis involved 67 nucleotide sequences, of which 63 were used as marker sequences to determine the genotype of 4 sequences. All positions containing gaps and missing data were eliminated. There was a total of 678 positions in the final dataset. Evolutionary analyses were conducted in MEGA X.

ID	GENOTYPE	SUBTYPE	COUNTRY	TREE	ALIGNMENT[1]	BASE PAIRS
U91804	D	D3	Costa Rica	1	157-837	681
U91809	D	D3	Costa Rica	1	157-837	681
U91816	D	D3	Costa Rica	1	157-837	681
U91832	D	D3	Costa Rica	1	157-837	681

[1] Alignment to complete reference sequence: GenBank accession number NC_003977.2

CUBA

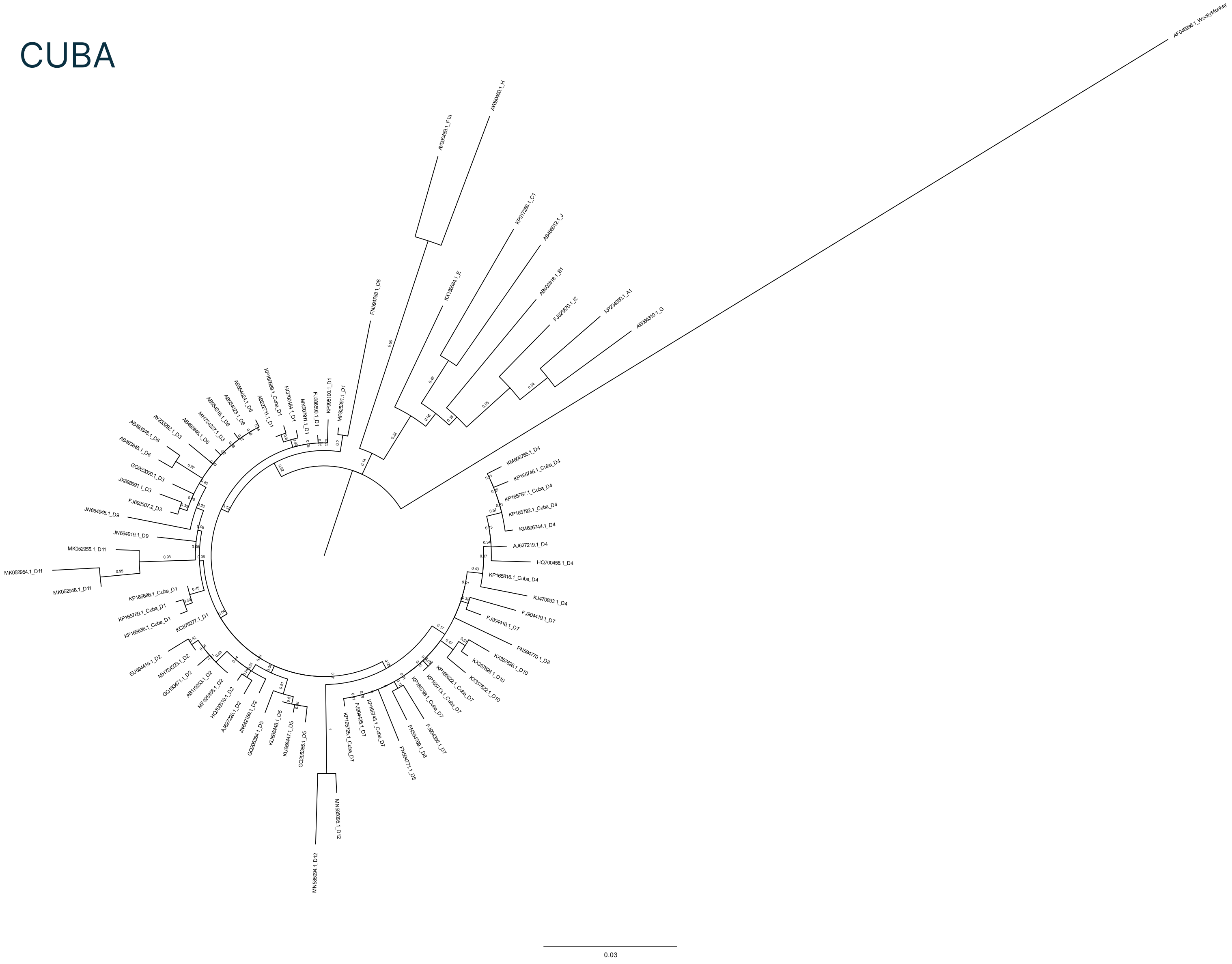

Tree 1. The evolutionary history was inferred by using the Maximum Likelihood method and Tamura-Nei model. The percentage of replicate trees in which the associated taxa clustered together in the bootstrap test (1000 replicates) are shown next to the branches. Initial tree(s) for the heuristic search were obtained automatically by applying Neighbor-Join and BioNJ algorithms to a matrix of pairwise distances estimated using the Tamura-Nei model, and then selecting the topology with superior log likelihood value. A discrete Gamma distribution was used to model evolutionary rate differences among sites (5 categories (+G, parameter = 0.2248)). The tree is drawn to scale, with branch lengths measured in the number of substitutions per site. The analysis involved 67 nucleotide sequences, of which 63 were used as marker sequences to determine the genotype of 4 sequences. All positions containing gaps and missing data were eliminated. There was a total of 678 positions in the final dataset. Evolutionary analyses were conducted in MEGA X.

ID	GENOTYPE	SUBTYPE	COUNTRY	TREE	ALIGNMENT[1]	BASE PAIRS
KP165622	D	D7	Cuba	1	157-837	681
KP165636	D	D9	Cuba	1	157-837	681
KP165686	D	D9	Cuba	1	157-837	681
KP165689	D	D1	Cuba	1	157-837	681
KP165713	D	D7	Cuba	1	157-837	681
KP165725	D	D7	Cuba	1	157-837	681
KP165743	D	D7	Cuba	1	157-837	681

KP165746	D	D4	Cuba	1	157-837	681
KP165769	D	D9	Cuba	1	157-837	681
KP165787	D	D4	Cuba	1	157-837	681
KP165792	D	D4	Cuba	1	157-837	681
KP165798	D	D7	Cuba	1	157-837	681
KP165816	D	D4	Cuba	1	157-837	681

[1] Alignment to complete reference sequence: GenBank accession number NC_003977.2

DOMINICAN REPUBLIC

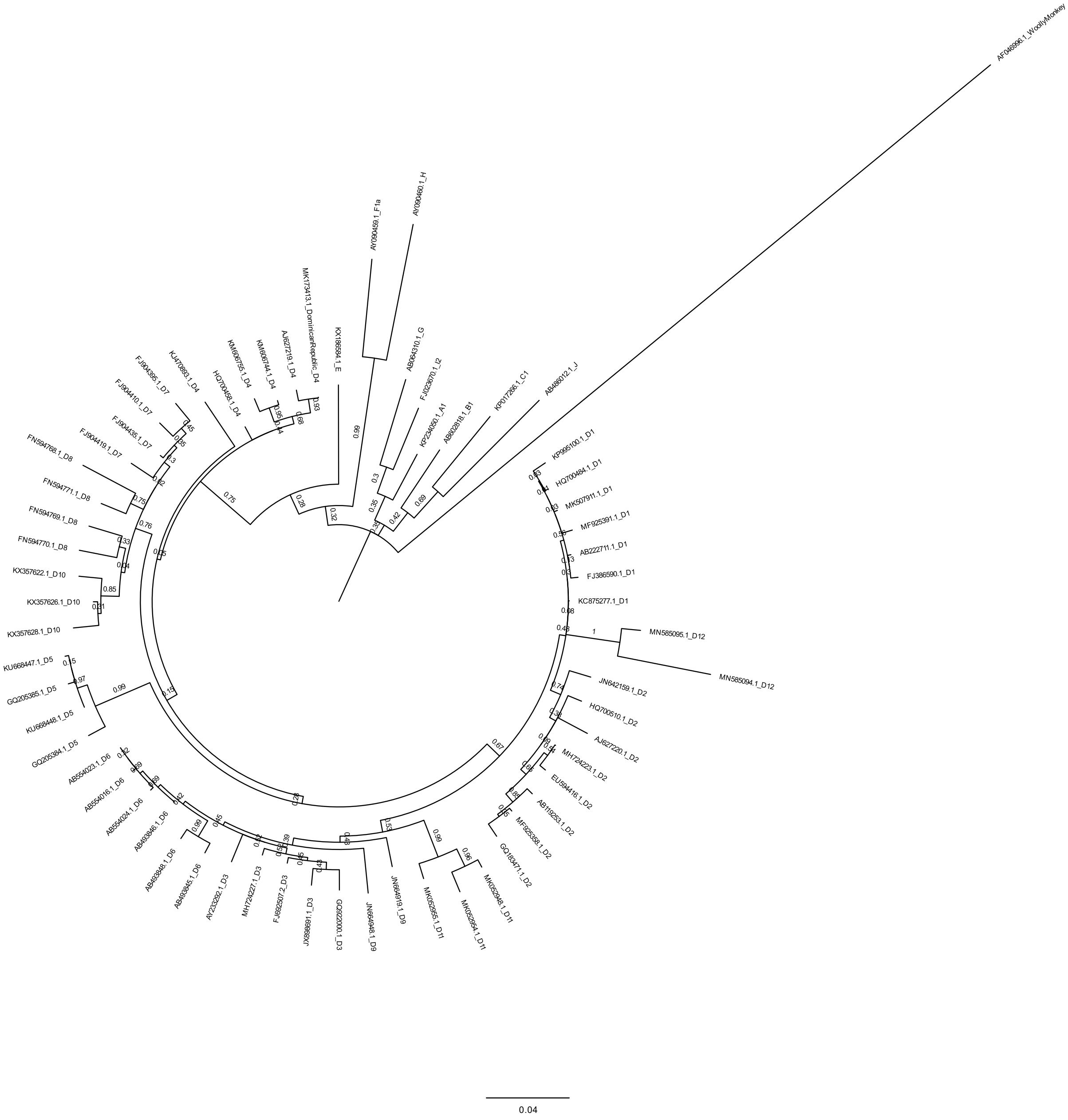

Tree 1. The evolutionary history was inferred by using the Maximum Likelihood method and Tamura-Nei model. The percentage of replicate trees in which the associated taxa clustered together in the bootstrap test (1000 replicates) are shown next to the branches. Initial tree(s) for the heuristic search were obtained automatically by applying Neighbor-Join and BioNJ algorithms to a matrix of pairwise distances estimated using the Tamura-Nei model, and then selecting the topology with superior log likelihood value. A discrete Gamma distribution was used to model evolutionary rate differences among sites (5 categories (+G, parameter = 0.2203)). The tree is drawn to scale, with branch lengths measured in the number of substitutions per site. The analysis involved 64 nucleotide sequences, of which 63 were used as marker sequences to determine the genotype of 1 sequence. All positions containing gaps and missing data were eliminated. There was a total of 737 positions in the final dataset. Evolutionary analyses were conducted in MEGA X.

ID	GENOTYPE	SUBTYPE	COUNTRY	TREE	ALIGNMENT[1]	BASE PAIRS
MK173413	D	D4	Dominican Republic	1	216-955	740

[1] Alignment to complete reference sequence: GenBank accession number NC_003977.2

GREENLAND

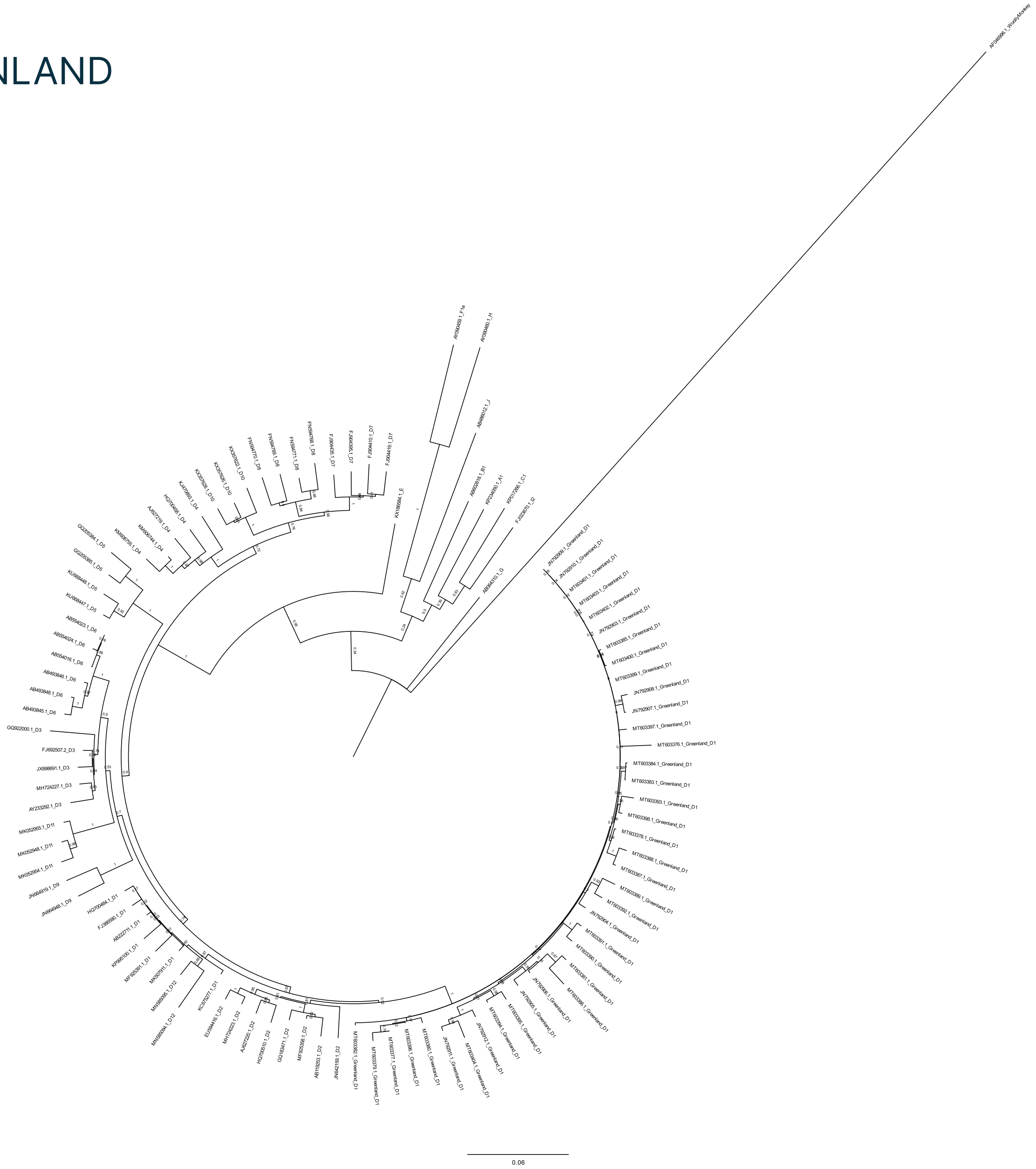

Tree 1. The evolutionary history was inferred by using the Maximum Likelihood method and Tamura-Nei model. The percentage of replicate trees in which the associated taxa clustered together in the bootstrap test (1000 replicates) are shown next to the branches. Initial tree(s) for the heuristic search were obtained automatically by applying Neighbor-Join and BioNJ algorithms to a matrix of pairwise distances estimated using the Tamura-Nei model, and then selecting the topology with superior log likelihood value. A discrete Gamma distribution was used to model evolutionary rate differences among sites (5 categories (+G, parameter = 0.2696)). The tree is drawn to scale, with branch lengths measured in the number of substitutions per site. The analysis involved 102 nucleotide sequences, of which 63 were used as marker sequences to determine the genotype of 39 sequences. All positions containing gaps and missing data were eliminated. There was a total of 2882 positions in the final dataset. Evolutionary analyses were conducted in MEGA X.

ID	GENOTYPE	SUBTYPE	COUNTRY	TREE	ALIGNMENT[1]	BASE PAIRS
JN792903	D	D2	Greenland	1	Complete Genome	3182
JN792904	D	D2	Greenland	1	Complete Genome	3182
JN792905	D	D2	Greenland	1	Complete Genome	3182

JN792906	D	D2	Greenland	1	Complete Genome	3182
JN792907	D	D2	Greenland	1	Complete Genome	3182
JN792908	D	D2	Greenland	1	Complete Genome	3185
JN792909	D	D2	Greenland	1	Complete Genome	3182
JN792910	D	D2	Greenland	1	Complete Genome	3182
JN792911	D	D2	Greenland	1	Complete Genome	3182
JN792912	D	D2	Greenland	1	Complete Genome	3182
MT603376	D	D2	Greenland	1	1-3182	3182
MT603377	D	D2	Greenland	1	1-3182	3182
MT603378	D	D2	Greenland	1	1-3182	3182
MT603379	D	D2	Greenland	1	1-3182	3182
MT603380	D	D2	Greenland	1	1-3182	3182
MT603381	D	D2	Greenland	1	1-3182	3182
MT603382	D	D2	Greenland	1	1-3182	3182
MT603383	D	D2	Greenland	1	1-3182	3182
MT603384	D	D2	Greenland	1	1-3182	3182
MT603385	D	D2	Greenland	1	1-3182	3182
MT603386	D	D2	Greenland	1	1-3182	3182
MT603387	D	D2	Greenland	1	Complete Genome	3182
MT603388	D	D2	Greenland	1	Complete Genome	3182
MT603389	D	D2	Greenland	1	Complete Genome	3182
MT603390	D	D2	Greenland	1	Complete Genome	3182
MT603391	D	D2	Greenland	1	Complete Genome	3182
MT603392	D	D2	Greenland	1	Complete Genome	3182
MT603393	D	D2	Greenland	1	Complete Genome	3182
MT603394	D	D2	Greenland	1	Complete Genome	3182
MT603395	D	D2	Greenland	1	Complete Genome	3182
MT603396	D	D2	Greenland	1	Complete Genome	3182
MT603397	D	D2	Greenland	1	Complete Genome	3182
MT603398	D	D2	Greenland	1	Complete Genome	3182
MT603399	D	D2	Greenland	1	Complete Genome	3182
MT603400	D	D2	Greenland	1	Complete Genome	3182
MT603401	D	D2	Greenland	1	Complete Genome	3182
MT603402	D	D2	Greenland	1	Complete Genome	3182
MT603403	D	D2	Greenland	1	Complete Genome	3182
MT603404	D	D2	Greenland	1	Complete Genome	3182

[1] Alignment to complete reference sequence: GenBank accession number NC_003977.2

UNITED STATES OF AMERICA

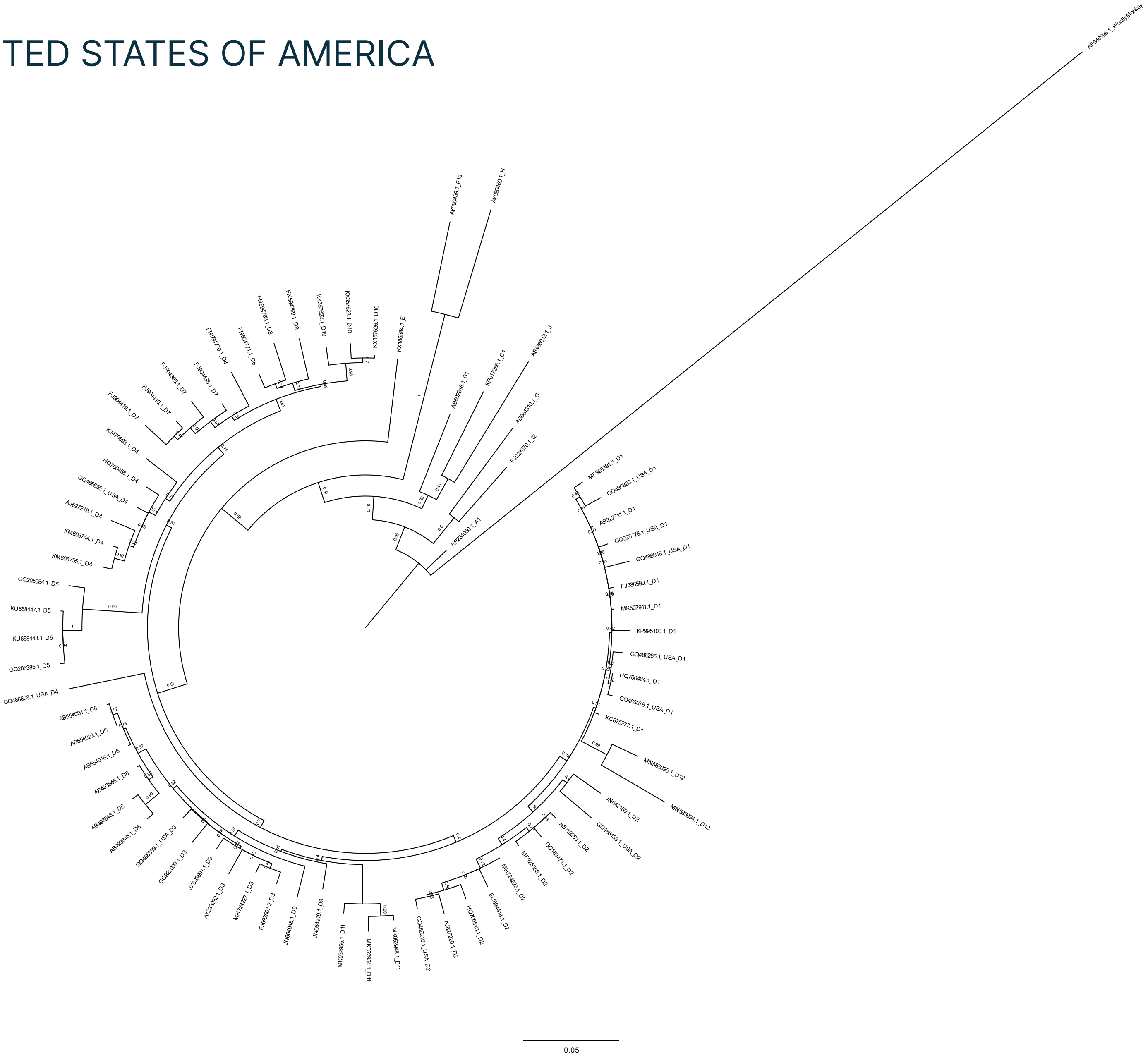

Tree 1. The evolutionary history was inferred by using the Maximum Likelihood method and Tamura-Nei model. The percentage of replicate trees in which the associated taxa clustered together in the bootstrap test (1000 replicates) are shown next to the branches. Initial tree(s) for the heuristic search were obtained automatically by applying Neighbor-Join and BioNJ algorithms to a matrix of pairwise distances estimated using the Tamura-Nei model, and then selecting the topology with superior log likelihood value. A discrete Gamma distribution was used to model evolutionary rate differences among sites (5 categories (+G, parameter = 0.2203)). The tree is drawn to scale, with branch lengths measured in the number of substitutions per site. The analysis involved 73 nucleotide sequences, of which 63 were used as marker sequences to determine the genotype of 10 sequences. All positions containing gaps and missing data were eliminated. There was a total of 994 positions in the final dataset. Evolutionary analyses were conducted in MEGA X.

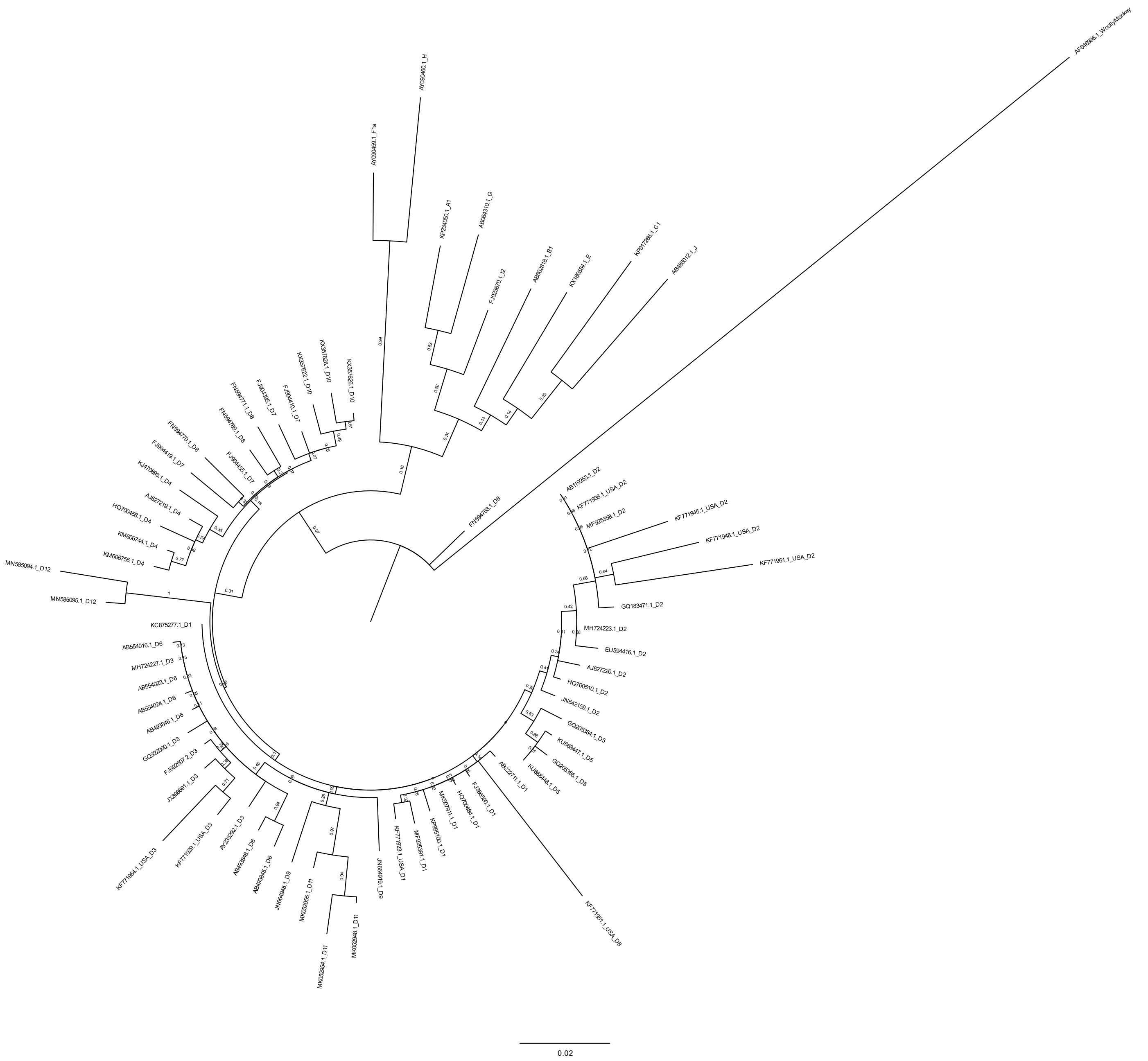

Tree 2. The evolutionary history was inferred by using the Maximum Likelihood method and Tamura-Nei model. The percentage of replicate trees in which the associated taxa clustered together in the bootstrap test (1000 replicates) are shown next to the branches. Initial tree(s) for the heuristic search were obtained automatically by applying Neighbor-Join and BioNJ algorithms to a matrix of pairwise distances estimated using the Tamura-Nei model, and then selecting the topology with superior log likelihood value. A discrete Gamma distribution was used to model evolutionary rate differences among sites (5 categories (+G, parameter = 0.2581)). The tree is drawn to scale, with branch lengths measured in the number of substitutions per site. The analysis involved 71 nucleotide sequences, of which 63 were used as marker sequences to determine the genotype of 8 sequences. All positions containing gaps and missing data were eliminated. There was a total of 676 positions in the final dataset. Evolutionary analyses were conducted in MEGA X.

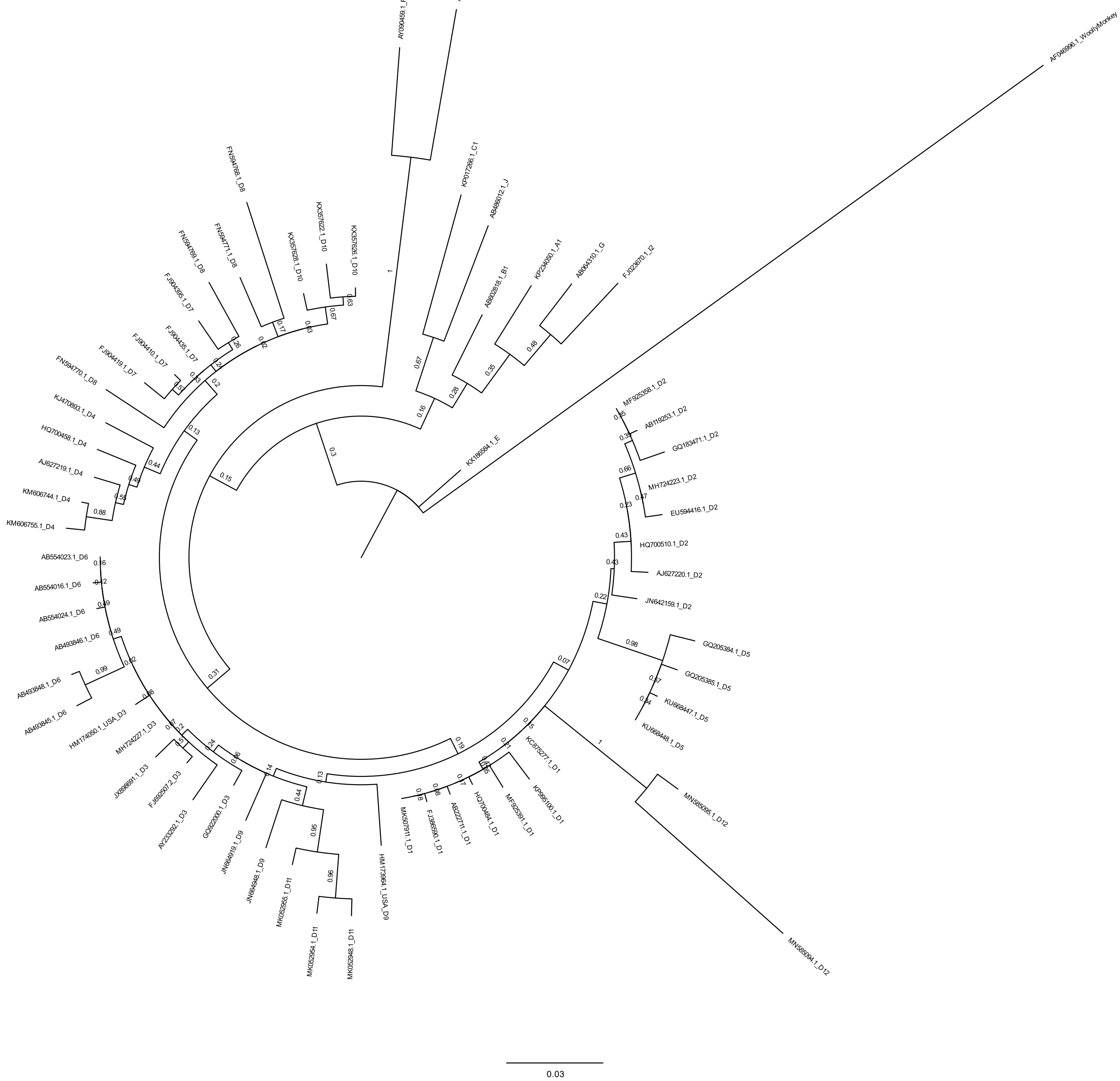

Tree 3. The evolutionary history was inferred by using the Maximum Likelihood method and Tamura-Nei model. The percentage of replicate trees in which the associated taxa clustered together in the bootstrap test (1000 replicates) are shown next to the branches. Initial tree(s) for the heuristic search were obtained automatically by applying Neighbor-Join and BioNJ algorithms to a matrix of pairwise distances estimated using the Tamura-Nei model, and then selecting the topology with superior log likelihood value. A discrete Gamma distribution was used to model evolutionary rate differences among sites (5 categories (+G, parameter = 0.2609)). The tree is drawn to scale, with branch lengths measured in the number of substitutions per site. The analysis involved 65 nucleotide sequences, of which 63 were used as marker sequences to determine the genotype of 2 sequences. All positions containing gaps and missing data were eliminated. There was a total of 458 positions in the final dataset. Evolutionary analyses were conducted in MEGA X.

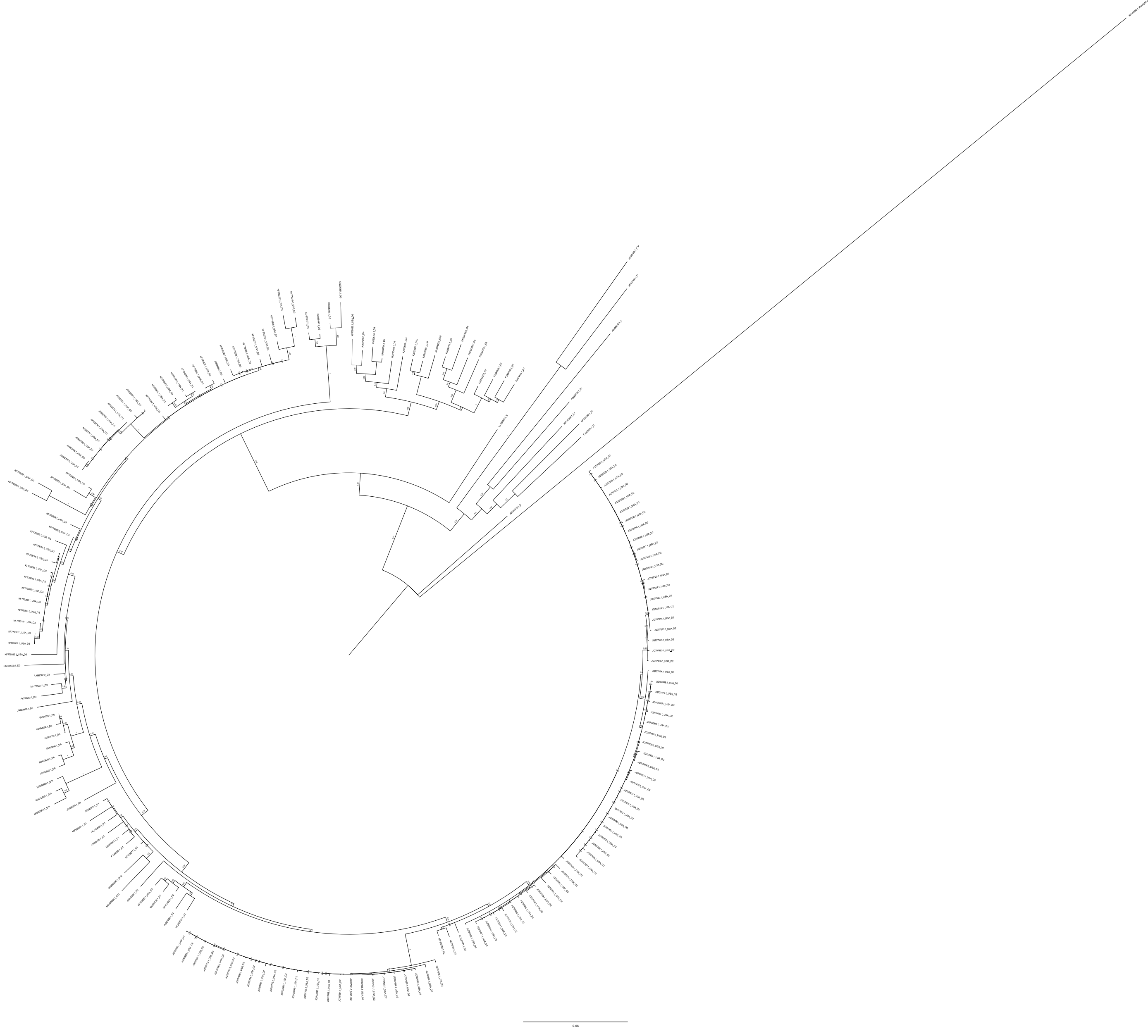

Tree 4. The evolutionary history was inferred by using the Maximum Likelihood method and Tamura-Nei model. The percentage of replicate trees in which the associated taxa clustered together in the bootstrap test (1000 replicates) are shown next to the branches. Initial tree(s) for the heuristic search were obtained automatically by applying Neighbor-Join and BioNJ algorithms to a matrix of pairwise distances estimated using the Tamura-Nei model, and then selecting the topology with superior log likelihood value. A discrete Gamma distribution was used to model evolutionary rate differences among sites (5 categories (+G, parameter = 0.2692)). The tree is drawn to scale, with branch lengths measured in the number of substitutions per site. The analysis involved 187 nucleotide sequences, of which 62 were used as marker sequences to determine the genotype of 125 sequences. All positions containing gaps and missing data were eliminated. There was a total of 2310 positions in the final dataset. Evolutionary analyses were conducted in MEGA X.

ID	GENOTYPE	SUBTYPE	COUNTRY	TREE	ALIGNMENT[1]	BASE PAIRS
GQ325778	D	D1	USA	1	132-1163	1032
GQ486078	D	D1	USA	1	132-1163	1032
GQ486133	D	D2	USA	1	132-1163	1032
GQ486210	D	D2	USA	1	132-1163	1032

GQ486285	D	D1	USA	1	132-1163	1032
GQ486339	D	D3	USA	1	132-1163	1032
GQ486655	D	D4	USA	1	132-1163	1032
GQ486808	D	D9	USA	1	132-1163	1032
GQ486820	D	D1	USA	1	132-1163	1032
GQ486848	D	D1	USA	1	132-1163	1032
KF771923	D	D1	USA	2	157-837	681
KF771929	D	D3	USA	2	157-837	681
KF771938	D	D2	USA	2	157-837	681
KF771945	D	D2	USA	2	157-837	681
KF771948	D	D2	USA	2	157-837	681
KF771951	D	D1	USA	2	157-837	681
KF771961	D	D2	USA	2	157-837	681
KF771964	D	D3	USA	2	157-837	681
HM173964	D	D9	USA	3	417-881	465
HM174050	D	D3	USA	3	417-881	465
AY902768	D	D3	USA	4	Complete Genome	3181
AY902769	D	D3	USA	4	Complete Genome	3181
AY902770	D	D3	USA	4	Complete Genome	3181
AY902771	D	D3	USA	4	Complete Genome	2637
AY902772	D	D3	USA	4	Complete Genome	3181
AY902773	D	D3	USA	4	Complete Genome	3181
AY902774	D	D3	USA	4	Complete Genome	3181
AY902776	D	D3	USA	4	Complete Genome	3181
AY902777	D	D3	USA	4	Complete Genome	3181
JQ707476	D	D2	USA	4	Complete Genome	3182
JQ707477	D	D2	USA	4	Complete Genome	2999
JQ707478	D	D2	USA	4	Complete Genome	3182
JQ707479	D	D2	USA	4	Complete Genome	3182
JQ707480	D	D2	USA	4	Complete Genome	3182
JQ707481	D	D2	USA	4	Complete Genome	3182
JQ707482	D	D2	USA	4	Complete Genome	3182
JQ707483	D	D2	USA	4	Complete Genome	3182
JQ707484	D	D2	USA	4	Complete Genome	2999
JQ707485	D	D2	USA	4	Complete Genome	3182
JQ707486	D	D2	USA	4	Complete Genome	3182
JQ707487	D	D2	USA	4	Complete Genome	3182
JQ707488	D	D2	USA	4	Complete Genome	3182
JQ707489	D	D2	USA	4	Complete Genome	3182
JQ707490	D	D2	USA	4	Complete Genome	3182
JQ707491	D	D2	USA	4	Complete Genome	3182
JQ707492	D	D2	USA	4	Complete Genome	2999
JQ707493	D	D2	USA	4	Complete Genome	3182
JQ707494	D	D2	USA	4	Complete Genome	3182
JQ707495	D	D2	USA	4	Complete Genome	2999
JQ707496	D	D2	USA	4	Complete Genome	3182

JQ707497	D	D2	USA	4	Complete Genome	3182
JQ707498	D	D2	USA	4	Complete Genome	2999
JQ707499	D	D2	USA	4	Complete Genome	3182
JQ707500	D	D2	USA	4	Complete Genome	3182
JQ707501	D	D2	USA	4	Complete Genome	2999
JQ707502	D	D2	USA	4	Complete Genome	3182
JQ707503	D	D2	USA	4	Complete Genome	3182
JQ707504	D	D2	USA	4	Complete Genome	2999
JQ707505	D	D2	USA	4	Complete Genome	3182
JQ707506	D	D2	USA	4	Complete Genome	2999
JQ707507	D	D2	USA	4	Complete Genome	3182
JQ707508	D	D2	USA	4	Complete Genome	3182
JQ707509	D	D2	USA	4	Complete Genome	3182
JQ707510	D	D2	USA	4	Complete Genome	3182
JQ707511	D	D2	USA	4	Complete Genome	3182
JQ707512	D	D2	USA	4	Complete Genome	3182
JQ707513	D	D2	USA	4	Complete Genome	3182
JQ707514	D	D2	USA	4	Complete Genome	3182
JQ707515	D	D2	USA	4	Complete Genome	3182
JQ707516	D	D2	USA	4	Complete Genome	3182
JQ707517	D	D2	USA	4	Complete Genome	3182
JQ707518	D	D2	USA	4	Complete Genome	3182
JQ707519	D	D2	USA	4	Complete Genome	3182
JQ707520	D	D2	USA	4	Complete Genome	3182
JQ707521	D	D2	USA	4	Complete Genome	3182
JQ707522	D	D2	USA	4	Complete Genome	3182
JQ707523	D	D2	USA	4	Complete Genome	3182
JQ707524	D	D2	USA	4	Complete Genome	3182
JQ707525	D	D2	USA	4	Complete Genome	3182
JQ707526	D	D2	USA	4	Complete Genome	3182
JQ707527	D	D2	USA	4	Complete Genome	3182
JQ707528	D	D2	USA	4	Complete Genome	3182
JQ707529	D	D2	USA	4	Complete Genome	3182
JQ707530	D	D2	USA	4	Complete Genome	3182
JQ707682	D	D2	USA	4	Complete Genome	3182
JQ707683	D	D2	USA	4	Complete Genome	3182
JQ707684	D	D2	USA	4	Complete Genome	3182
JQ707685	D	D2	USA	4	Complete Genome	3182
JQ707686	D	D2	USA	4	Complete Genome	3182
JQ707687	D	D2	USA	4	Complete Genome	3182
JQ707688	D	D2	USA	4	Complete Genome	3182
JQ707689	D	D2	USA	4	Complete Genome	3182
JQ707690	D	D2	USA	4	Complete Genome	3182
JQ707691	D	D2	USA	4	Complete Genome	3182
JQ707692	D	D2	USA	4	Complete Genome	3182
JQ707693	D	D2	USA	4	Complete Genome	3182

JQ707694	D	D2	USA	4	Complete Genome	3182
JQ707695	D	D2	USA	4	Complete Genome	3182
JQ707696	D	D2	USA	4	Complete Genome	3182
JQ707697	D	D2	USA	4	Complete Genome	3182
JQ707698	D	D2	USA	4	Complete Genome	3182
JQ707699	D	D2	USA	4	Complete Genome	3182
JQ707700	D	D2	USA	4	Complete Genome	3182
JQ707701	D	D2	USA	4	Complete Genome	3182
JQ707702	D	D2	USA	4	Complete Genome	3182
JQ707703	D	D2	USA	4	Complete Genome	3182
JQ707704	D	D2	USA	4	Complete Genome	3182
JQ707705	D	D2	USA	4	Complete Genome	3182
JQ707706	D	D2	USA	4	Complete Genome	3182
KF779209	D	D3	USA	4	Complete Genome	3126
KF779212	D	D3	USA	4	Complete Genome	3126
KF779214	D	D3	USA	4	Complete Genome	3126
KF779216	D	D3	USA	4	Complete Genome	3126
KF779217	D	D3	USA	4	Complete Genome	3126
KF779218	D	D3	USA	4	Complete Genome	3126
KF779219	D	D3	USA	4	Complete Genome	3126
KF779220	D	D2	USA	4	Complete Genome	3126
KF779222	D	D3	USA	4	Complete Genome	3126
KF779223	D	D3	USA	4	Complete Genome	3126
KF779224	D	D3	USA	4	Complete Genome	3126
KF779225	D	D3	USA	4	Complete Genome	3126
KF779226	D	D3	USA	4	Complete Genome	3127
KF779228	D	D3	USA	4	Complete Genome	3129
KF779229	D	D3	USA	4	Complete Genome	3129
KF779230	D	D3	USA	4	Complete Genome	3129
KF779237	D	D3	USA	4	Complete Genome	3159
KF779241	D	D3	USA	4	Complete Genome	3127
KF779250	D	D3	USA	4	Complete Genome	3159
KF779285	D	D3	USA	4	Complete Genome	3126
KF779288	D	D3	USA	4	Complete Genome	3126
KF779289	D	D3	USA	4	Complete Genome	3126
KF779292	D	D3	USA	4	Complete Genome	3126
KF779296	D	D3	USA	4	Complete Genome	3126
KF779301	D	D3	USA	4	Complete Genome	3126
KF779302	D	D3	USA	4	Complete Genome	3126
KF779303	D	D3	USA	4	Complete Genome	3126
KF779318	D	D3	USA	4	Complete Genome	3126
KF779340	D	D3	USA	4	Complete Genome	3126
KF779341	D	D3	USA	4	Complete Genome	3126
KF779343	D	D3	USA	4	Complete Genome	3127
KF779353	D	D4	USA	4	Complete Genome	3159
KF779376	D	D3	USA	4	Complete Genome	3126

KF779377	D	D3	USA	4	Complete Genome	3126
KF779380	D	D3	USA	4	Complete Genome	3128
KF779382	D	D3	USA	4	Complete Genome	3129

[1] Alignment to complete reference sequence: GenBank accession number NC_003977.2

VENEZUELA

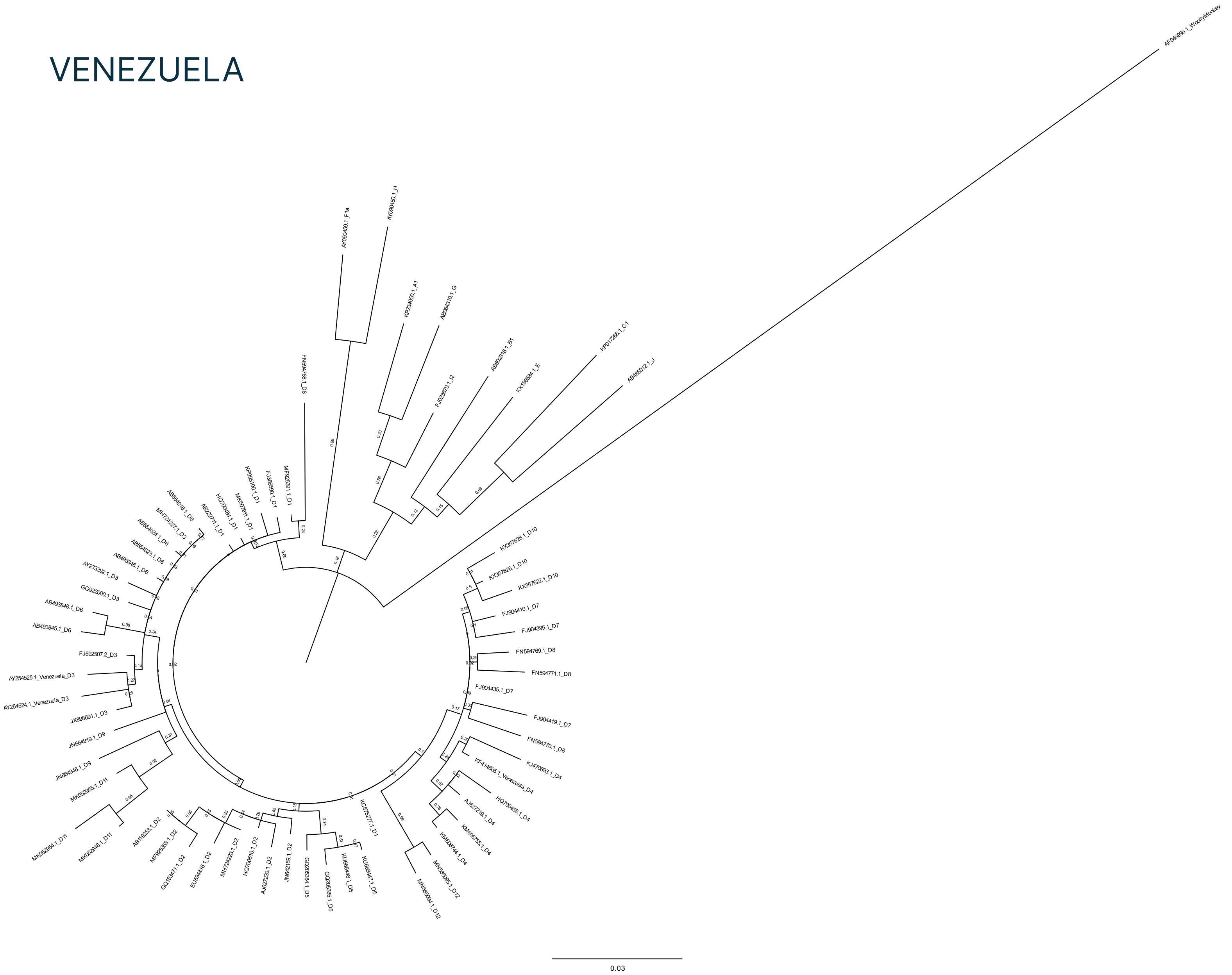

Tree 1. The evolutionary history was inferred by using the Maximum Likelihood method and Tamura-Nei model. The percentage of replicate trees in which the associated taxa clustered together in the bootstrap test (1000 replicates) are shown next to the branches. Initial tree(s) for the heuristic search were obtained automatically by applying Neighbor-Join and BioNJ algorithms to a matrix of pairwise distances estimated using the Tamura-Nei model, and then selecting the topology with superior log likelihood value. A discrete Gamma distribution was used to model evolutionary rate differences among sites (5 categories (+G, parameter = 0.2122)). The tree is drawn to scale, with branch lengths measured in the number of substitutions per site. The analysis involved 66 nucleotide sequences, of which 63 were used as marker sequences to determine the genotype of 3 sequences. All positions containing gaps and missing data were eliminated. There was a total of 671 positions in the final dataset. Evolutionary analyses were conducted in MEGA X.

ID	GENOTYPE	SUBTYPE	COUNTRY	TREE	ALIGNMENT[1]	BASE PAIRS
AY254524	D	D3	Venezuela	1	152-824	637
AY254525	D	D3	Venezuela	1	152-824	637
KF414665	D	D4	Venezuela	1	138-840	703

[1] Alignment to complete reference sequence: GenBank accession number NC_003977.

I – SUBGENOTYPE

CANADA

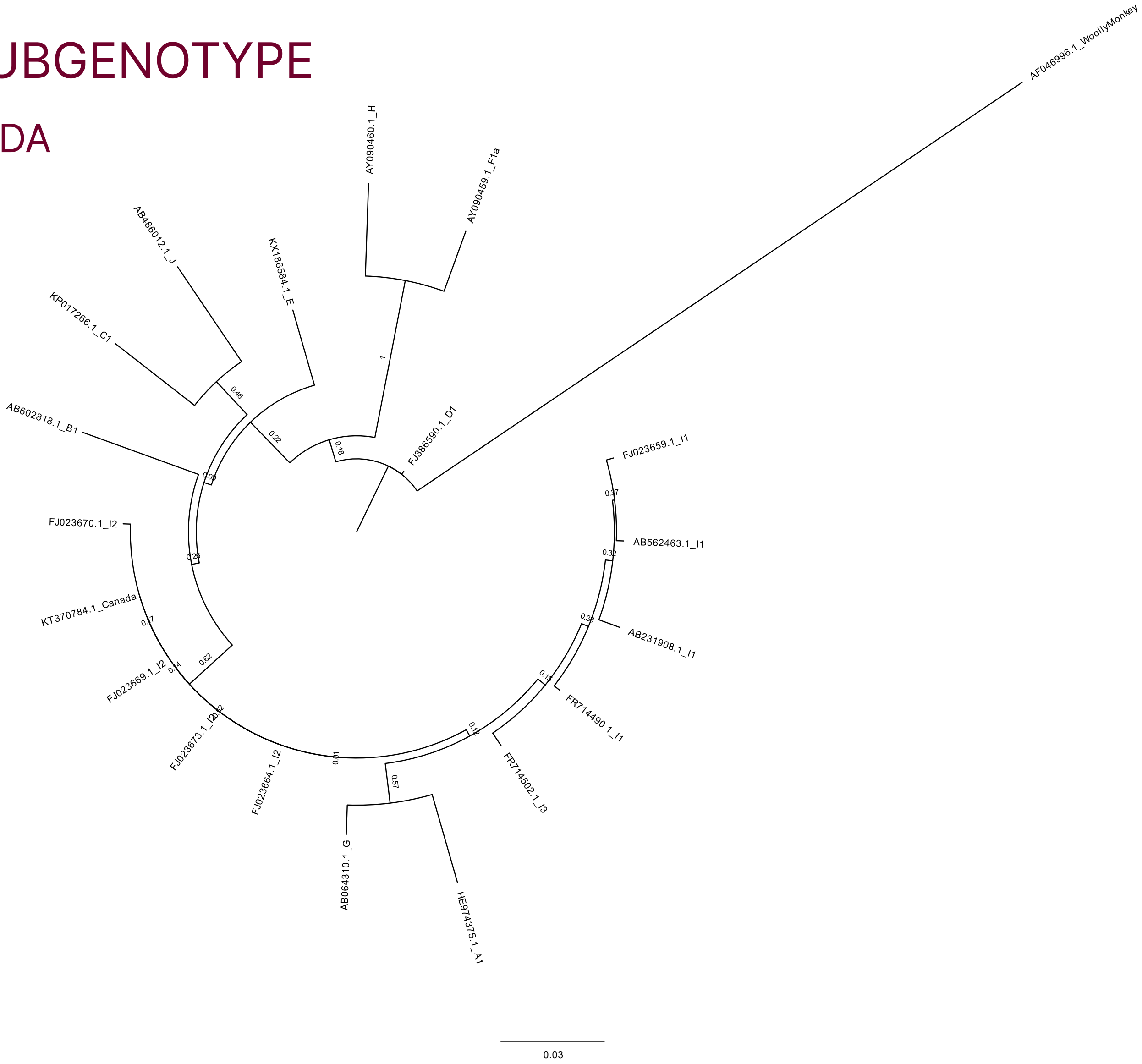

Tree 1. The evolutionary history was inferred by using the Maximum Likelihood method and Tamura-Nei model. The percentage of replicate trees in which the associated taxa clustered together in the bootstrap test (1000 replicates) are shown next to the branches. Initial tree(s) for the heuristic search were obtained automatically by applying Neighbor-Join and BioNJ algorithms to a matrix of pairwise distances estimated using the Tamura-Nei model, and then selecting the topology with superior log likelihood value. A discrete Gamma distribution was used to model evolutionary rate differences among sites (5 categories (+G, parameter = 0.1255)). The tree is drawn to scale, with branch lengths measured in the number of substitutions per site. The analysis involved 20 nucleotide sequences, of which 19 were used as marker sequences to determine the genotype of 1 sequence. All positions containing gaps and missing data were eliminated. There was a total of 511 positions in the final dataset. Evolutionary analyses were conducted in MEGA X.

ID	GENOTYPE	SUBTYPE	COUNTRY	ALIGNMENT[1]	BASE PAIRS
KT370784	I	I2	Canada	313-831	519

[1] Alignment to complete reference sequence: GenBank accession number NC_003977.2

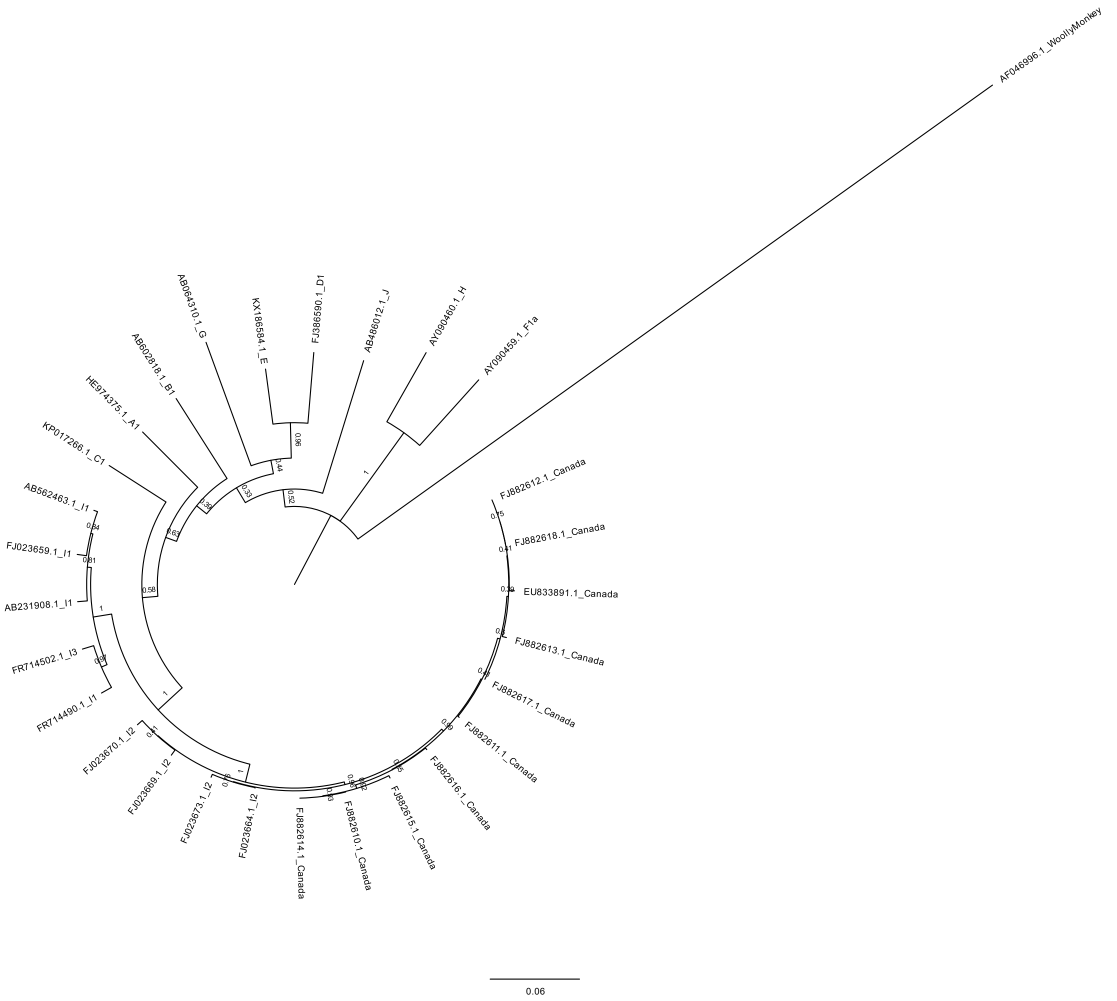

Tree 2. The evolutionary history was inferred by using the Maximum Likelihood method and Tamura-Nei model. The percentage of replicate trees in which the associated taxa clustered together in the bootstrap test (1000 replicates) are shown next to the branches. Initial tree(s) for the heuristic search were obtained automatically by applying Neighbor-Join and BioNJ algorithms to a matrix of pairwise distances estimated using the Tamura-Nei model, and then selecting the topology with superior log likelihood value. A discrete Gamma distribution was used to model evolutionary rate differences among sites (5 categories (+G, parameter = 0.1642)). The tree is drawn to scale, with branch lengths measured in the number of substitutions per site. The analysis involved 29 nucleotide sequences, of which 19 were used as marker sequences to determine the genotype of 10 sequences. All positions containing gaps and missing data were eliminated. There was a total of 898 positions in the final dataset. Evolutionary analyses were conducted in MEGA X.

ID	GENOTYPE	SUBTYPE	COUNTRY	ALIGNMENT[1]	BASE PAIRS
EU833891	I	I2	Canada	Complete Genome	3215
FJ882610	I	I2	Canada	Complete Genome	3215
FJ882611	I	I2	Canada	Complete Genome	3215
FJ882612	I	I2	Canada	Complete Genome	3215
FJ882613	I	I2	Canada	Complete Genome	3215
FJ882614	I	I2	Canada	Complete Genome	3215
FJ882615	I	I2	Canada	Complete Genome	3216
FJ882616	I	I2	Canada	Complete Genome	3168
FJ882617	I	I2	Canada	Complete Genome	3215
FJ882618	I	I2	Canada	Complete Genome	3215

[1] Alignment to complete reference sequence: GenBank accession number NC_003977.2

F – SUBGENOTYPE

ALASKA

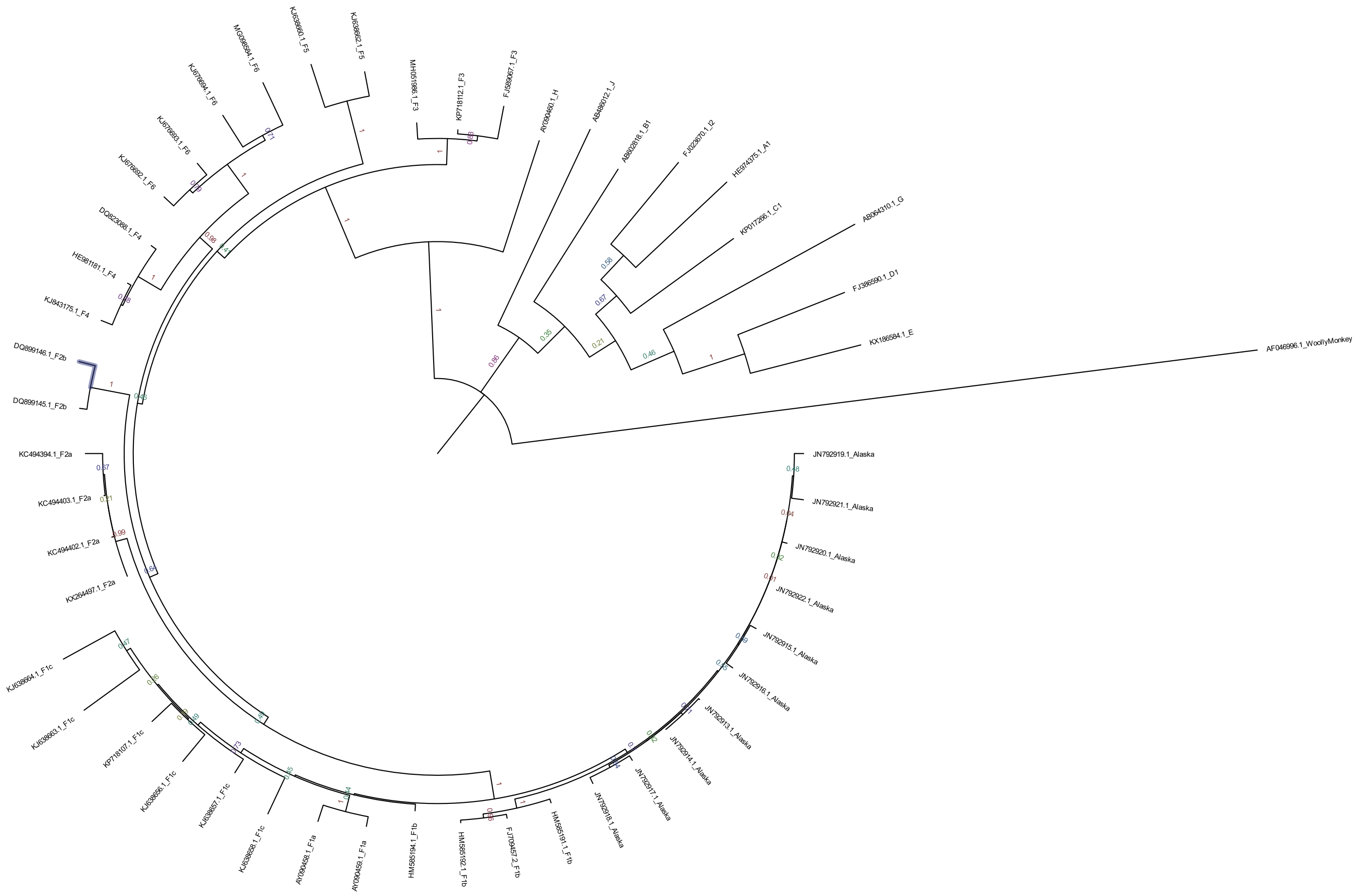

Tree 1. Molecular Phylogenetic analysis by Maximum Likelihood method conducted in MEGA7. The evolutionary history was inferred by using the Maximum Likelihood method based on the Tamura-Nei model with 1000 bootstraps. The tree with the highest log likelihood (-18219.19) is shown. The percentage of trees in which the associated taxa clustered together is shown next to the branches. The tree is drawn to scale, with branch lengths measured in the number of substitutions per site. The analysis involved 50 nucleotide sequences, of which 40 were used as marker sequences to determine the genotype of 10 sequences. All positions containing gaps and missing data were eliminated. There was a total of 2879 positions in the final dataset.

ID	GENOTYPE	SUBTYPE	COUNTRY	ALIGNMENT[1]	BASE PAIRS
JN792913	F	F1b	Alaska	Complete Genome	3207
JN792914	F	F1b	Alaska	Complete Genome	3209
JN792915	F	F1b	Alaska	Complete Genome	3207
JN792916	F	F1b	Alaska	Complete Genome	3206
JN792917	F	F1b	Alaska	Complete Genome	3215
JN792918	F	F1b	Alaska	Complete Genome	3215
JN792919	F	F1b	Alaska	Complete Genome	3215
JN792920	F	F1b	Alaska	Complete Genome	3215
JN792921	F	F1b	Alaska	Complete Genome	3215
JN792922	F	F1b	Alaska	Complete Genome	3215

ARGENTINA

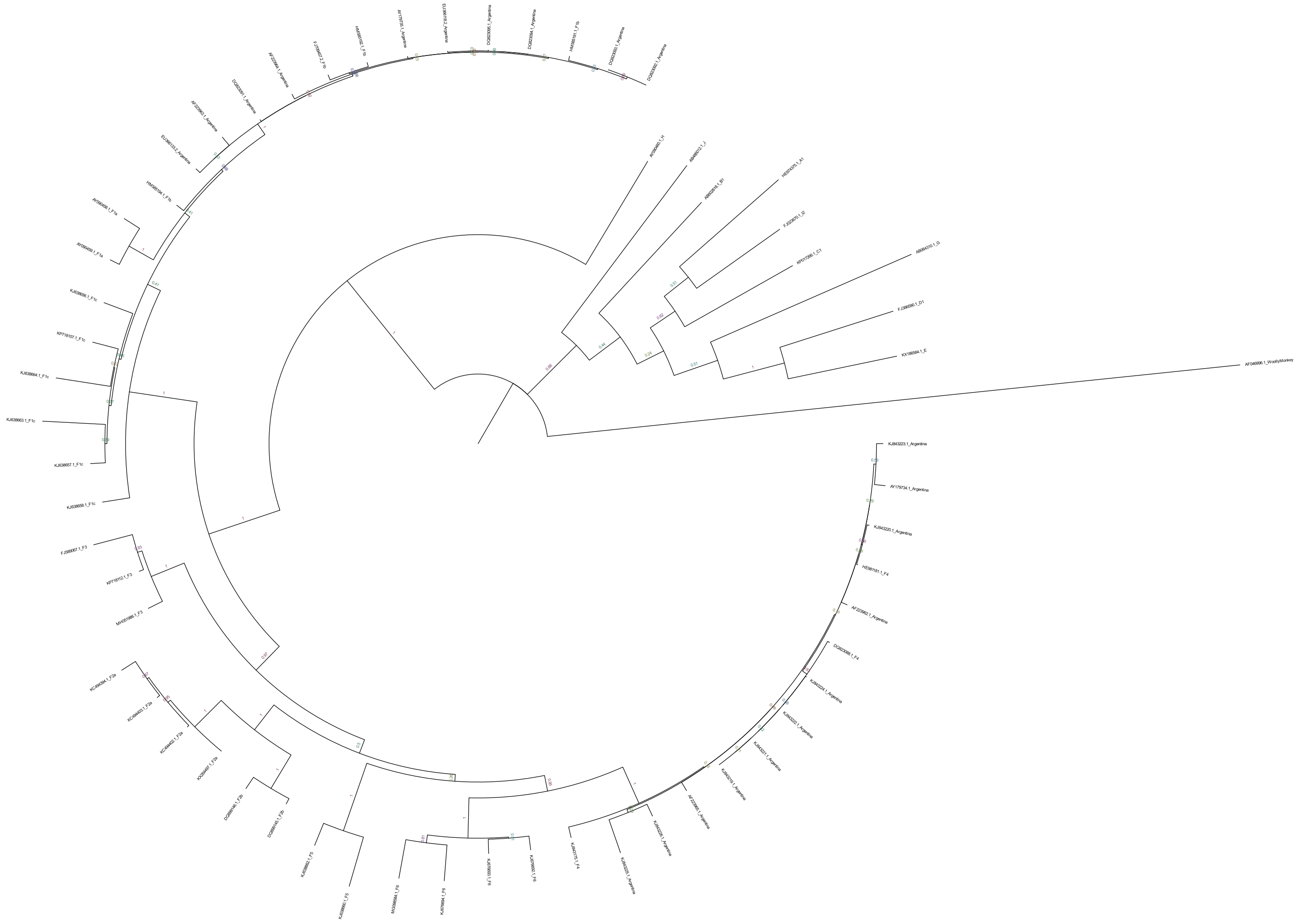

Tree 1. Molecular Phylogenetic analysis by Maximum Likelihood method conducted in MEGA7. The evolutionary history was inferred by using the Maximum Likelihood method based on the Tamura-Nei model with 1000 bootstraps. The tree with the highest log likelihood (-22097.71) is shown. The percentage of trees in which the associated taxa clustered together is shown next to the branches. The tree is drawn to scale, with branch lengths measured in the number of substitutions per site. The analysis involved 61 nucleotide sequences, of which 40 were used as marker sequences to determine the genotype of 21 sequences. All positions containing gaps and missing data were eliminated. There was a total of 3025 positions in the final dataset.

ID	GENOTYPE	SUBTYPE	COUNTRY	ALIGNMENT[1]	BASE PAIRS
DQ823091	F	F1b	Argentina	Complete Genome	3215
DQ823092	F	F1b	Argentina	Complete Genome	3215
DQ823093	F	F1b	Argentina	Complete Genome	3215
DQ823094	F	F1b	Argentina	Complete Genome	3215
DQ823095	F	F1b	Argentina	Complete Genome	3215
EU366118	F	F1b	Argentina	Complete Genome	3215
EU366133	F	F1b	Argentina	Complete Genome	3215
AF223963	F	F1b	Argentina	Complete Genome	3215
AF223964	F	F1b	Argentina	Complete Genome	3215
AY179735	F	F1b	Argentina	Complete Genome	3215
AF223962	F	F4	Argentina	Complete Genome	3215

AF223965	F	F4	Argentina	Complete Genome	3215
AY179734	F	F4	Argentina	Complete Genome	3215
KJ843219	F	F4	Argentina	Complete Genome	3215
KJ843220	F	F4	Argentina	Complete Genome	3215
KJ843221	F	F4	Argentina	Complete Genome	3215
KJ843222	F	F4	Argentina	Complete Genome	3215
KJ843223	F	F4	Argentina	Complete Genome	3215
KJ843224	F	F4	Argentina	Complete Genome	3215
KJ843225	F	F4	Argentina	Complete Genome	3215
KJ843226	F	F4	Argentina	Complete Genome	3215

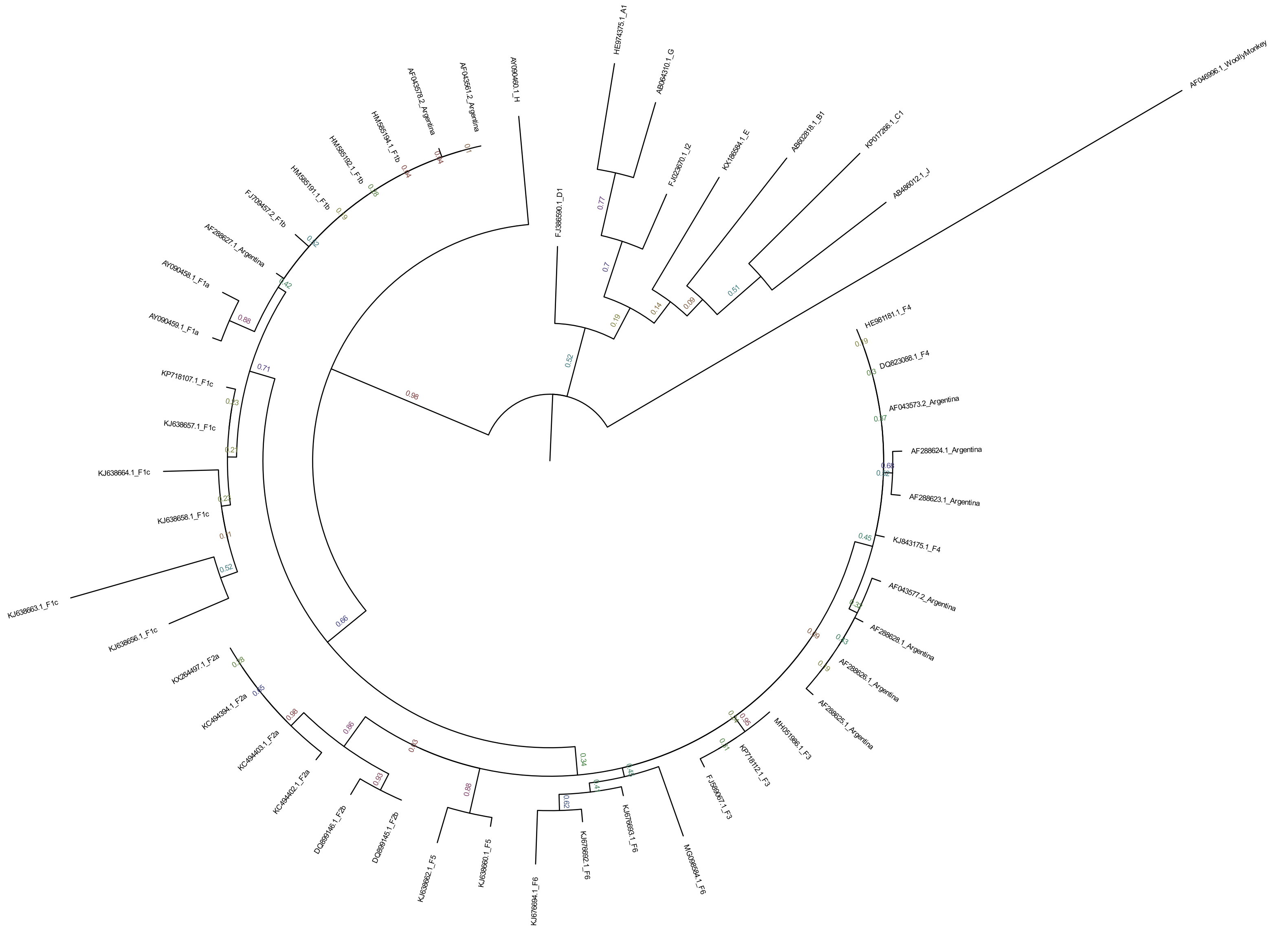

Tree 2. Molecular Phylogenetic analysis by Maximum Likelihood method conducted in MEGA7. The evolutionary history was inferred by using the Maximum Likelihood method based on the Tamura-Nei model with 1000 bootstraps. The tree with the highest log likelihood (-2939.86) is shown. The percentage of trees in which the associated taxa clustered together is shown next to the branches. The tree is drawn to scale, with branch lengths measured in the number of substitutions per site. The analysis involved 50 nucleotide sequences, of which 40 were used as marker sequences to determine the genotype of 10 sequences. All positions containing gaps and missing data were eliminated. There was a total of 655 positions in the final dataset.

ID	GENOTYPE	SUBTYPE	COUNTRY	ALIGNMENT[1]	BASE PAIRS
AF043561	F	F1b	Argentina	157-831	675
AF043578	F	F1b	Argentina	157-834	678
AF288627	F	F1b	Argentina	157-834	678
AF043573	F	F4	Argentina	157-834	678
AF043577	F	F4	Argentina	157-834	678
AF288623	F	F4	Argentina	157-834	678

AF288624	F	F4	Argentina	157-834	678
AF288625	F	F4	Argentina	157-834	678
AF288626	F	F4	Argentina	157-834	678
AF288628	F	F4	Argentina	157-831	675

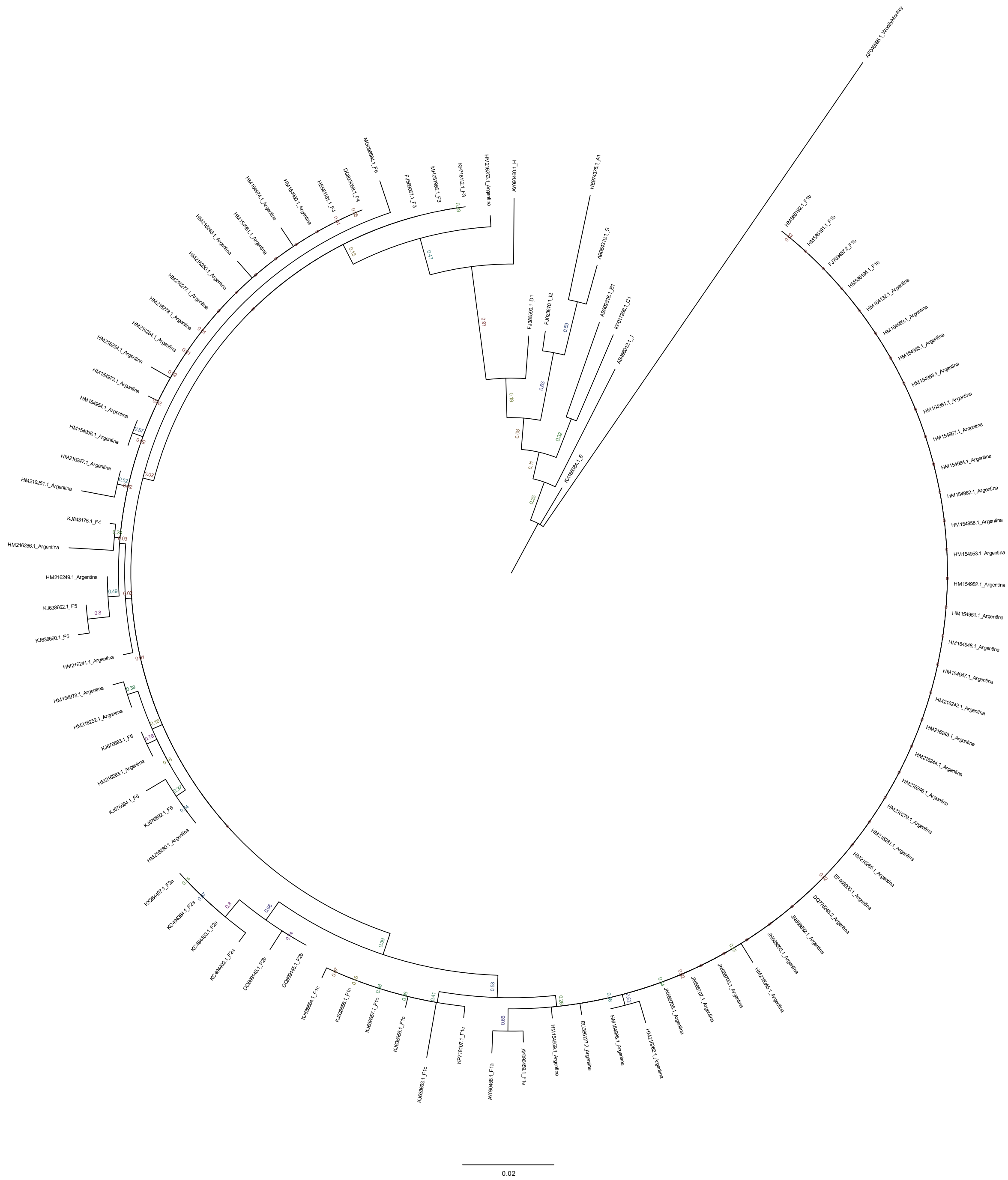

Tree 3. Molecular Phylogenetic analysis by Maximum Likelihood method conducted in MEGA7. The evolutionary history was inferred by using the Maximum Likelihood method based on the Tamura-Nei model with 1000 bootstraps. The tree with the highest log likelihood (-1846.60) is shown. The percentage of trees in which the associated taxa clustered together is shown next to the branches. The tree is drawn to scale, with branch lengths measured in the number of substitutions per site. The analysis involved 95 nucleotide sequences, of which 40 were used as marker sequences to determine the genotype of 55 sequences. All positions containing gaps and missing data were eliminated. There was a total of 412 positions in the final dataset.

ID	GENOTYPE	SUBTYPE	COUNTRY	ALIGNMENT[1]	BASE PAIRS
DQ776245	F	F1b	Argentina	244-768	525
EU366127	F	F1b	Argentina	244-768	525
EF468000	F	F1b	Argentina	282-793	512
HM216242	F	F1b	Argentina	259-766	508

HM216243	F	F1b	Argentina	259-766	508
HM216244	F	F1b	Argentina	259-766	508
HM216245	F	F1b	Argentina	259-766	508
HM216246	F	F1b	Argentina	259-766	508
HM216279	F	F1b	Argentina	259-766	508
HM216281	F	F1b	Argentina	259-766	508
HM216282	F	F1b	Argentina	259-766	508
HM216285	F	F1b	Argentina	259-766	508
HM216253	F	F3	Argentina	259-766	508
HM216247	F	F4	Argentina	259-766	508
HM216248	F	F4	Argentina	259-766	508
HM216250	F	F4	Argentina	259-766	508
HM216251	F	F4	Argentina	259-766	508
HM216254	F	F4	Argentina	259-766	508
HM216277	F	F4	Argentina	259-766	508
HM216278	F	F4	Argentina	259-766	508
HM216284	F	F4	Argentina	259-766	508
HM216241	F	F5	Argentina	259-766	508
HM216249	F	F5	Argentina	259-766	508
HM216252	F	F6	Argentina	259-766	508
HM216280	F	F6	Argentina	259-766	508
HM216283	F	F6	Argentina	259-766	508
HM154947	F	F1b	Argentina	244-747	504
HM154948	F	F1b	Argentina	244-747	504
HM154951	F	F1b	Argentina	244-750	507
HM154952	F	F1b	Argentina	244-747	504
HM154953	F	F1b	Argentina	244-744	501
HM154958	F	F1b	Argentina	244-750	507
HM154959	F	F1b	Argentina	244-747	504
HM154962	F	F1b	Argentina	244-747	504
HM154964	F	F1b	Argentina	244-747	504
HM154967	F	F1b	Argentina	244-750	507
HM154981	F	F1b	Argentina	316-744	429
HM154983	F	F1b	Argentina	244-747	504
HM154985	F	F1b	Argentina	244-747	504
HM154988	F	F1b	Argentina	244-750	507
HM154989	F	F1b	Argentina	244-747	504
HM164132	F	F1b	Argentina	246-768	523
JN688692	F	F1b	Argentina	217-771	555
JN688693	F	F1b	Argentina	217-771	555
JN688700	F	F1b	Argentina	217-771	555
JN688705	F	F1b	Argentina	217-771	555
JN688707	F	F1b	Argentina	217-771	555
HM154938	F	F4	Argentina	244-747	504
HM154954	F	F4	Argentina	244-747	504
HM154961	F	F4	Argentina	244-750	507

HM154973	F	F4	Argentina	244-747	504
HM154974	F	F4	Argentina	244-747	504
HM154980	F	F4	Argentina	244-747	504
HM216286	F	F4	Argentina	259-766	508
HM154978	F	F6	Argentina	244-747	504

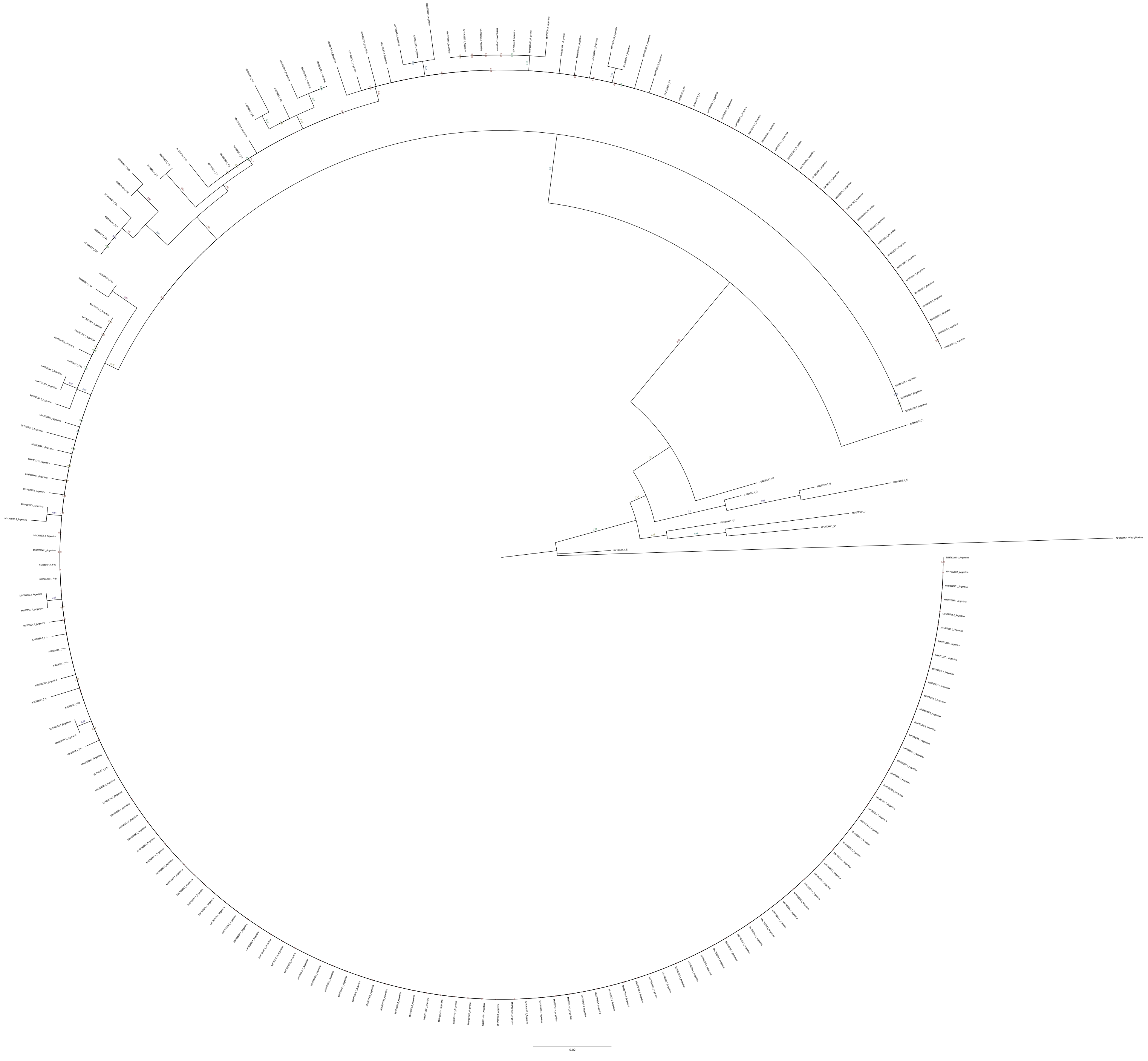

Tree 4. Molecular Phylogenetic analysis by Maximum Likelihood method conducted in MEGA7. The evolutionary history was inferred by using the Maximum Likelihood method based on the Tamura-Nei model with 1000 bootstraps. The tree with the highest log likelihood (-1438.87) is shown. The percentage of trees in which the associated taxa clustered together is shown next to the branches. The tree is drawn to scale, with branch lengths measured in the number of substitutions per site. The analysis involved 199 nucleotide sequences, of which 40 were used as marker sequences to determine the genotype of 159 sequences. All positions containing gaps and missing data were eliminated. There was a total of 262 positions in the final dataset.

ID	GENOTYPE	SUBTYPE	COUNTRY	ALIGNMENT[1]	BASE PAIRS
MH763046	F	F1b	Argentina	364-775	412
MH763059	F	F1b	Argentina	364-784	421
MH763098	F	F1b	Argentina	364-784	421
MH763114	F	F1b	Argentina	364-784	421

MH763127	F	F1b	Argentina	364-775	412
MH763138	F	F1b	Argentina	364-784	421
MH763155	F	F1b	Argentina	364-784	421
MH763158	F	F1b	Argentina	364-784	421
MH763169	F	F1b	Argentina	364-769	406
MH763171	F	F1b	Argentina	364-784	421
MH763175	F	F1b	Argentina	364-784	421
MH763197	F	F1b	Argentina	364-784	421
MH763244	F	F1b	Argentina	364-784	421
MH763285	F	F1b	Argentina	364-784	421
MH763289	F	F1b	Argentina	469-784	316
MH763294	F	F1b	Argentina	364-773	410
MH763298	F	F1b	Argentina	364-784	421
MH763038	F	F1c	Argentina	364-784	421
MH763044	F	F1c	Argentina	364-769	406
MH763050	F	F1c	Argentina	364-784	421
MH763053	F	F1c	Argentina	364-769	406
MH763055	F	F1c	Argentina	364-784	421
MH763056	F	F1c	Argentina	364-784	421
MH763058	F	F1c	Argentina	364-784	421
MH763061	F	F1c	Argentina	364-784	421
MH763063	F	F1c	Argentina	364-784	421
MH763067	F	F1c	Argentina	364-784	421
MH763069	F	F1c	Argentina	364-784	421
MH763073	F	F1c	Argentina	364-784	421
MH763075	F	F1c	Argentina	364-769	406
MH763079	F	F1c	Argentina	364-784	421
MH763083	F	F1c	Argentina	364-784	421
MH763084	F	F1c	Argentina	364-784	421
MH763085	F	F1c	Argentina	364-784	421
MH763090	F	F1c	Argentina	364-784	421
MH763097	F	F1c	Argentina	364-784	421
MH763101	F	F1c	Argentina	364-784	421
MH763103	F	F1c	Argentina	364-769	406
MH763106	F	F1c	Argentina	364-784	421
MH763110	F	F1c	Argentina	364-784	421
MH763111	F	F1c	Argentina	364-784	421
MH763112	F	F1c	Argentina	364-784	421
MH763115	F	F1c	Argentina	364-784	421
MH763116	F	F1c	Argentina	364-784	421
MH763119	F	F1c	Argentina	364-784	421
MH763125	F	F1c	Argentina	364-784	421
MH763128	F	F1c	Argentina	364-773	410
MH763134	F	F1c	Argentina	364-784	421
MH763135	F	F1c	Argentina	364-784	421
MH763137	F	F1c	Argentina	364-751	388

MH763143	F	F1c	Argentina	364-784	421
MH763146	F	F1c	Argentina	364-784	421
MH763150	F	F1c	Argentina	364-784	421
MH763151	F	F1c	Argentina	364-784	421
MH763153	F	F1c	Argentina	364-784	421
MH763160	F	F1c	Argentina	364-784	421
MH763162	F	F1c	Argentina	364-784	421
MH763163	F	F1c	Argentina	364-769	406
MH763166	F	F1c	Argentina	364-751	388
MH763168	F	F1c	Argentina	364-784	421
MH763177	F	F1c	Argentina	364-784	421
MH763178	F	F1c	Argentina	364-784	421
MH763184	F	F1c	Argentina	364-784	421
MH763185	F	F1c	Argentina	364-784	421
MH763190	F	F1c	Argentina	364-784	421
MH763193	F	F1c	Argentina	364-784	421
MH763194	F	F1c	Argentina	364-784	421
MH763195	F	F1c	Argentina	364-776	413
MH763199	F	F1c	Argentina	364-784	421
MH763202	F	F1c	Argentina	364-784	421
MH763203	F	F1c	Argentina	364-784	421
MH763204	F	F1c	Argentina	364-784	421
MH763205	F	F1c	Argentina	364-784	421
MH763206	F	F1c	Argentina	364-784	421
MH763207	F	F1c	Argentina	364-784	421
MH763208	F	F1c	Argentina	364-784	421
MH763209	F	F1c	Argentina	364-784	421
MH763213	F	F1c	Argentina	364-784	421
MH763214	F	F1c	Argentina	364-784	421
MH763221	F	F1c	Argentina	364-784	421
MH763224	F	F1c	Argentina	364-769	406
MH763228	F	F1c	Argentina	364-784	421
MH763231	F	F1c	Argentina	364-784	421
MH763232	F	F1c	Argentina	364-784	421
MH763233	F	F1c	Argentina	364-784	421
MH763234	F	F1c	Argentina	364-784	421
MH763239	F	F1c	Argentina	364-784	421
MH763240	F	F1c	Argentina	364-784	421
MH763242	F	F1c	Argentina	364-784	421
MH763243	F	F1c	Argentina	364-784	421
MH763251	F	F1c	Argentina	364-784	421
MH763253	F	F1c	Argentina	364-784	421
MH763256	F	F1c	Argentina	364-784	421
MH763260	F	F1c	Argentina	364-769	406
MH763261	F	F1c	Argentina	364-784	421
MH763262	F	F1c	Argentina	364-784	421

MH763264	F	F1c	Argentina	364-784	421
MH763265	F	F1c	Argentina	364-784	421
MH763266	F	F1c	Argentina	364-784	421
MH763268	F	F1c	Argentina	364-784	421
MH763271	F	F1c	Argentina	364-784	421
MH763276	F	F1c	Argentina	364-784	421
MH763277	F	F1c	Argentina	364-784	421
MH763280	F	F1c	Argentina	364-784	421
MH763282	F	F1c	Argentina	364-784	421
MH763284	F	F1c	Argentina	364-784	421
MH763286	F	F1c	Argentina	364-784	421
MH763287	F	F1c	Argentina	364-784	421
MH763291	F	F1c	Argentina	364-784	421
MH763293	F	F1c	Argentina	364-784	421
MH763299	F	F1c	Argentina	364-784	421
MH763283	F	F3	Argentina	364-756	393
MH763039	F	F4	Argentina	364-756	393
MH763045	F	F4	Argentina	364-756	393
MH763047	F	F4	Argentina	364-756	393
MH763048	F	F4	Argentina	364-756	393
MH763051	F	F4	Argentina	364-756	393
MH763060	F	F4	Argentina	364-756	393
MH763064	F	F4	Argentina	364-756	393
MH763066	F	F4	Argentina	364-756	393
MH763070	F	F4	Argentina	364-756	393
MH763071	F	F4	Argentina	364-756	393
MH763087	F	F4	Argentina	364-756	393
MH763091	F	F4	Argentina	364-756	393
MH763094	F	F4	Argentina	469-784	316
MH763095	F	F4	Argentina	364-756	393
MH763099	F	F4	Argentina	364-756	393
MH763104	F	F4	Argentina	364-756	393
MH763108	F	F4	Argentina	364-756	393
MH763113	F	F4	Argentina	364-756	393
MH763130	F	F4	Argentina	364-756	393
MH763136	F	F4	Argentina	364-756	393
MH763145	F	F4	Argentina	364-756	393
MH763149	F	F4	Argentina	364-756	393
MH763154	F	F4	Argentina	364-756	393
MH763172	F	F4	Argentina	364-756	393
MH763173	F	F4	Argentina	364-756	393
MH763176	F	F4	Argentina	364-756	393
MH763189	F	F4	Argentina	364-756	393
MH763200	F	F4	Argentina	364-756	393
MH763201	F	F4	Argentina	364-756	393
MH763211	F	F4	Argentina	364-756	393

MH763222	F	F4	Argentina	364-756	393
MH763227	F	F4	Argentina	364-756	393
MH763236	F	F4	Argentina	364-756	393
MH763241	F	F4	Argentina	364-756	393
MH763247	F	F4	Argentina	364-756	393
MH763257	F	F4	Argentina	364-756	393
MH763258	F	F4	Argentina	364-756	393
MH763267	F	F4	Argentina	364-756	393
MH763269	F	F4	Argentina	364-756	393
MH763270	F	F4	Argentina	364-756	393
MH763288	F	F4	Argentina	364-756	393
MH763295	F	F4	Argentina	364-756	393
MH763297	F	F4	Argentina	364-756	393
MH763300	F	F4	Argentina	364-756	393
MH763180	F	F6	Argentina	364-756	393
MH763223	F	F6	Argentina	364-756	393
MH763278	F	F6	Argentina	364-756	393

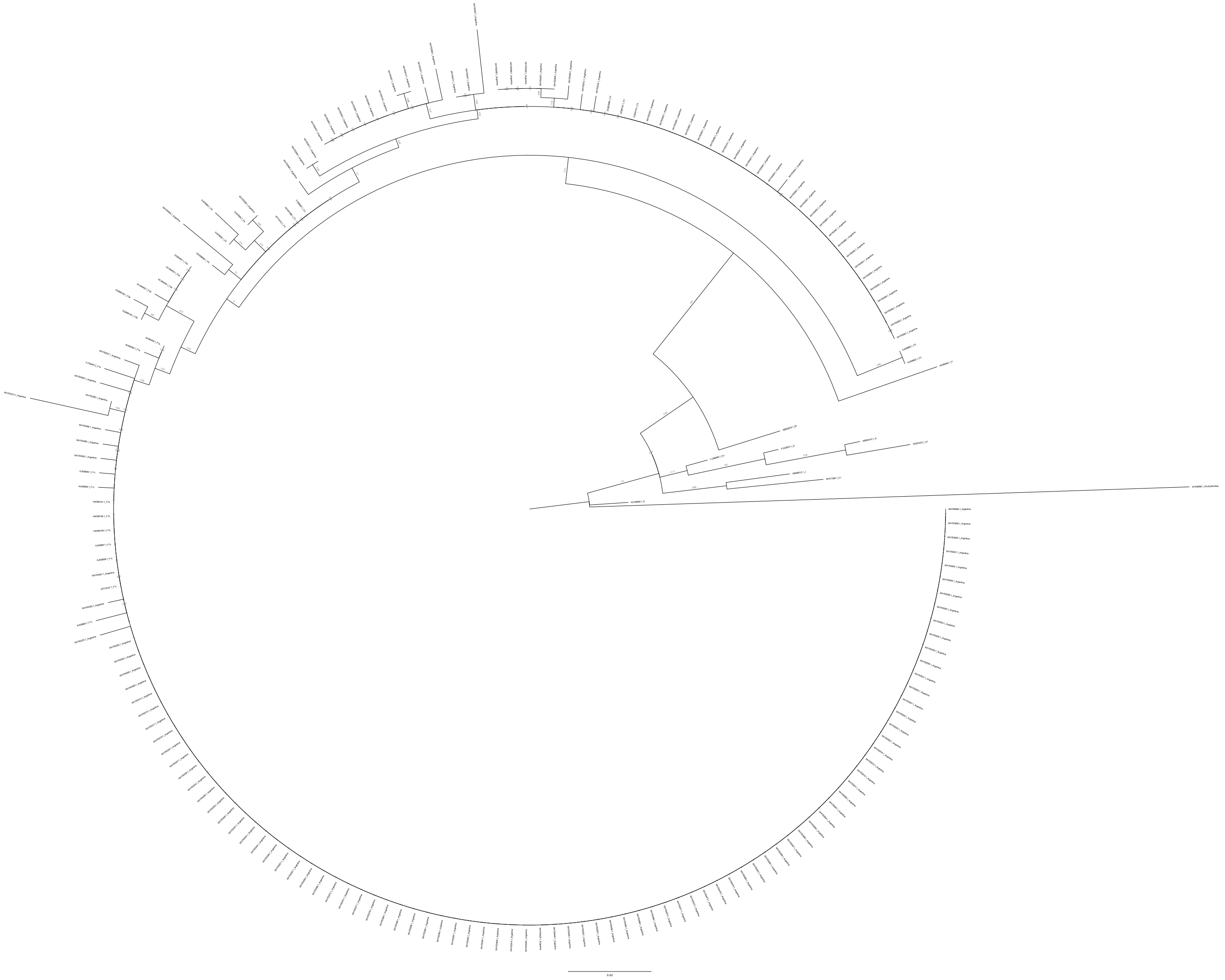

Tree 5. Molecular Phylogenetic analysis by Maximum Likelihood method conducted in MEGA7. The evolutionary history was inferred by using the Maximum Likelihood method based on the Tamura-Nei model with 1000 bootstraps. The tree with the highest log likelihood (-1337.24) is shown. The percentage of trees in which the associated taxa clustered together is shown next to the branches. The tree is drawn to scale, with branch lengths measured in the number of substitutions per site. The analysis involved 187 nucleotide sequences, of which 40 were used as marker sequences to determine the genotype of 147 sequences. All positions containing gaps and missing data were eliminated. There was a total of 264 positions in the final dataset.

ID	GENOTYPE	SUBTYPE	COUNTRY	ALIGNMENT[1]	BASE PAIRS
MH763313	F	F1b	Argentina	364-784	421
MH763350	F	F1b	Argentina	364-784	421
MH763382	F	F1b	Argentina	364-769	406
MH763383	F	F1b	Argentina	364-784	421
MH763386	F	F1b	Argentina	364-784	421
MH763398	F	F1b	Argentina	364-769	406
MH763537	F	F1b	Argentina	364-769	406
MH763301	F	F1c	Argentina	364-784	421
MH763302	F	F1c	Argentina	364-784	421

MH763303	F	F1c	Argentina	364-769	406
MH763308	F	F1c	Argentina	364-784	421
MH763309	F	F1c	Argentina	364-770	407
MH763314	F	F1c	Argentina	364-784	421
MH763315	F	F1c	Argentina	364-784	421
MH763317	F	F1c	Argentina	364-784	421
MH763319	F	F1c	Argentina	364-769	406
MH763324	F	F1c	Argentina	364-784	421
MH763327	F	F1c	Argentina	364-775	412
MH763329	F	F1c	Argentina	364-784	421
MH763333	F	F1c	Argentina	364-784	421
MH763336	F	F1c	Argentina	364-784	421
MH763339	F	F1c	Argentina	364-784	421
MH763340	F	F1c	Argentina	364-784	421
MH763341	F	F1c	Argentina	364-784	421
MH763343	F	F1c	Argentina	364-784	421
MH763344	F	F1c	Argentina	364-784	421
MH763349	F	F1c	Argentina	364-784	421
MH763351	F	F1c	Argentina	364-784	421
MH763352	F	F1c	Argentina	364-784	421
MH763357	F	F1c	Argentina	364-784	421
MH763358	F	F1c	Argentina	364-784	421
MH763366	F	F1c	Argentina	364-784	421
MH763372	F	F1c	Argentina	364-784	421
MH763373	F	F1c	Argentina	364-784	421
MH763374	F	F1c	Argentina	364-784	421
MH763377	F	F1c	Argentina	364-784	421
MH763378	F	F1c	Argentina	364-776	413
MH763380	F	F1c	Argentina	364-784	421
MH763384	F	F1c	Argentina	364-784	421
MH763389	F	F1c	Argentina	364-784	421
MH763394	F	F1c	Argentina	364-784	421
MH763395	F	F1c	Argentina	364-784	421
MH763397	F	F1c	Argentina	364-784	421
MH763400	F	F1c	Argentina	364-784	421
MH763401	F	F1c	Argentina	364-784	421
MH763409	F	F1c	Argentina	364-784	421
MH763410	F	F1c	Argentina	364-784	421
MH763428	F	F1c	Argentina	364-784	421
MH763430	F	F1c	Argentina	364-784	421
MH763440	F	F1c	Argentina	364-781	418
MH763450	F	F1c	Argentina	364-784	421
MH763452	F	F1c	Argentina	364-784	421
MH763453	F	F1c	Argentina	364-784	421
MH763458	F	F1c	Argentina	364-784	421
MH763462	F	F1c	Argentina	364-784	421

MH763466	F	F1c	Argentina	364-784	421
MH763468	F	F1c	Argentina	364-784	421
MH763470	F	F1c	Argentina	364-784	421
MH763471	F	F1c	Argentina	364-776	413
MH763472	F	F1c	Argentina	364-784	421
MH763473	F	F1c	Argentina	364-784	421
MH763476	F	F1c	Argentina	364-784	421
MH763479	F	F1c	Argentina	364-784	421
MH763480	F	F1c	Argentina	364-784	421
MH763483	F	F1c	Argentina	364-784	421
MH763484	F	F1c	Argentina	364-784	421
MH763489	F	F1c	Argentina	364-784	421
MH763491	F	F1c	Argentina	364-784	421
MH763498	F	F1c	Argentina	364-784	421
MH763499	F	F1c	Argentina	364-784	421
MH763504	F	F1c	Argentina	364-784	421
MH763507	F	F1c	Argentina	364-784	421
MH763508	F	F1c	Argentina	364-784	421
MH763511	F	F1c	Argentina	364-784	421
MH763514	F	F1c	Argentina	364-784	421
MH763515	F	F1c	Argentina	364-784	421
MH763516	F	F1c	Argentina	364-784	421
MH763520	F	F1c	Argentina	364-784	421
MH763523	F	F1c	Argentina	364-784	421
MH763525	F	F1c	Argentina	364-784	421
MH763527	F	F1c	Argentina	364-784	421
MH763528	F	F1c	Argentina	364-784	421
MH763531	F	F1c	Argentina	364-784	421
MH763536	F	F1c	Argentina	364-784	421
MH763539	F	F1c	Argentina	364-784	421
MH763540	F	F1c	Argentina	364-784	421
MH763543	F	F1c	Argentina	364-784	421
MH763545	F	F1c	Argentina	364-769	406
MH763548	F	F1c	Argentina	364-784	421
MH763549	F	F1c	Argentina	364-784	421
MH763550	F	F1c	Argentina	364-784	421
MH763551	F	F1c	Argentina	364-784	421
MH763559	F	F1c	Argentina	364-784	421
MH763562	F	F1c	Argentina	364-784	421
MH763566	F	F1c	Argentina	364-784	421
MH763316	F	F4	Argentina	364-756	393
MH763334	F	F4	Argentina	364-784	421
MH763335	F	F4	Argentina	364-756	393
MH763337	F	F4	Argentina	364-756	393
MH763345	F	F4	Argentina	364-756	393
MH763355	F	F4	Argentina	364-756	393

MH763356	F	F4	Argentina	364-756	393
MH763365	F	F4	Argentina	364-756	393
MH763390	F	F4	Argentina	364-756	393
MH763391	F	F4	Argentina	364-751	388
MH763402	F	F4	Argentina	364-756	393
MH763403	F	F4	Argentina	364-756	393
MH763405	F	F4	Argentina	364-756	393
MH763406	F	F4	Argentina	364-756	393
MH763411	F	F4	Argentina	364-756	393
MH763414	F	F4	Argentina	364-751	388
MH763419	F	F4	Argentina	364-756	393
MH763425	F	F4	Argentina	364-756	393
MH763434	F	F4	Argentina	364-756	393
MH763438	F	F4	Argentina	364-756	393
MH763443	F	F4	Argentina	364-756	393
MH763447	F	F4	Argentina	364-756	393
MH763448	F	F4	Argentina	364-756	393
MH763449	F	F4	Argentina	364-756	393
MH763454	F	F4	Argentina	364-756	393
MH763455	F	F4	Argentina	364-756	393
MH763456	F	F4	Argentina	364-756	393
MH763457	F	F4	Argentina	364-756	393
MH763459	F	F4	Argentina	364-756	393
MH763463	F	F4	Argentina	364-756	393
MH763464	F	F4	Argentina	364-756	393
MH763465	F	F4	Argentina	364-756	393
MH763469	F	F4	Argentina	364-756	393
MH763481	F	F4	Argentina	364-756	393
MH763486	F	F4	Argentina	364-756	393
MH763492	F	F4	Argentina	364-756	393
MH763500	F	F4	Argentina	364-756	393
MH763503	F	F4	Argentina	364-756	393
MH763506	F	F4	Argentina	364-756	393
MH763509	F	F4	Argentina	364-756	393
MH763512	F	F4	Argentina	364-756	393
MH763517	F	F4	Argentina	364-756	393
MH763535	F	F4	Argentina	364-756	393
MH763538	F	F4	Argentina	364-756	393
MH763542	F	F4	Argentina	364-756	393
MH763547	F	F4	Argentina	364-756	393
MH763558	F	F4	Argentina	364-756	393
MH763560	F	F4	Argentina	364-756	393
MH763561	F	F4	Argentina	466-756	291
MH763564	F	F4	Argentina	364-756	393
MH763399	F	F6	Argentina	364-770	407
MH763493	F	F6	Argentina	364-756	393

BRAZIL

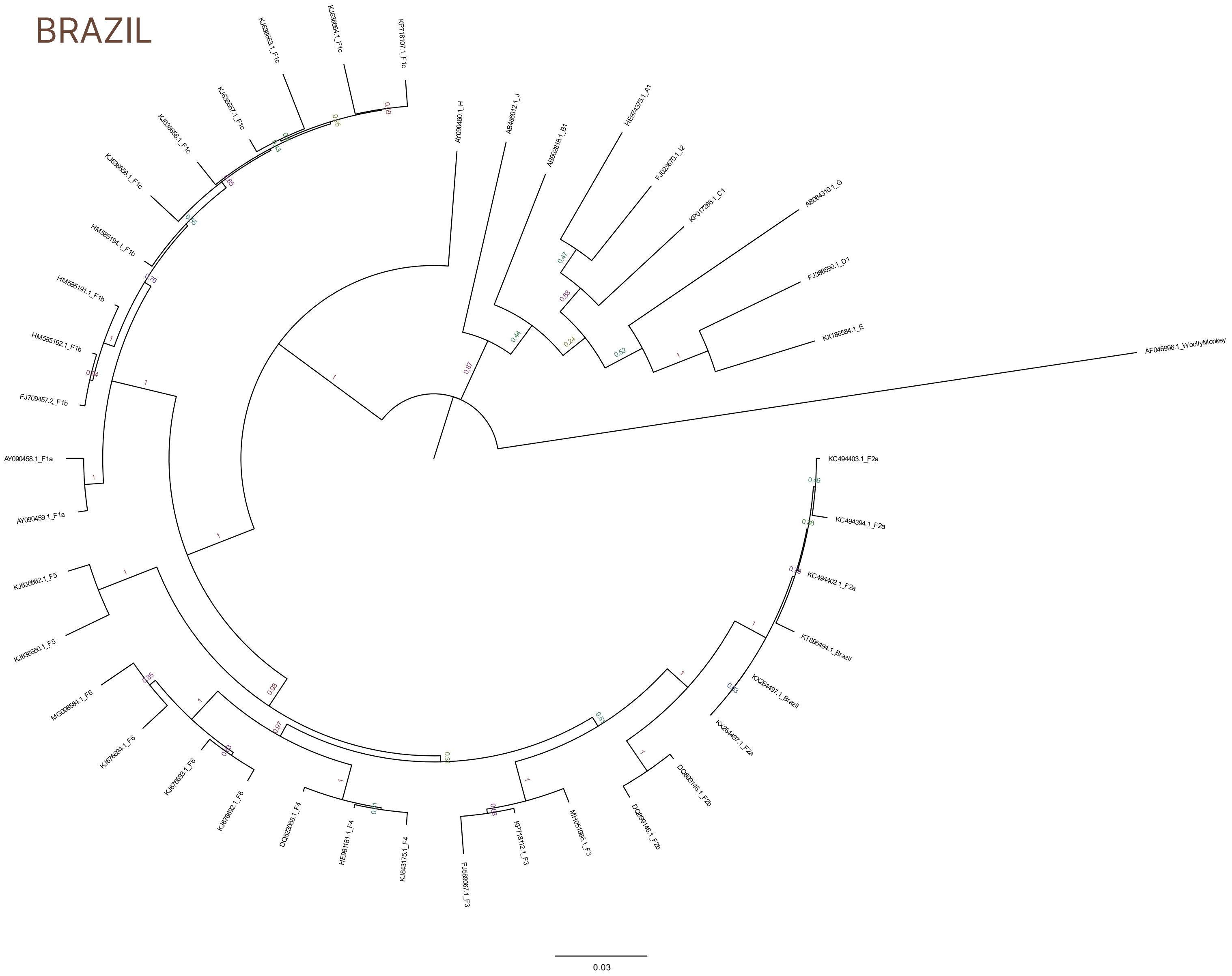

Tree 1. Molecular Phylogenetic analysis by Maximum Likelihood method conducted in MEGA7. The evolutionary history was inferred by using the Maximum Likelihood method based on the Tamura-Nei model with 1000 bootstraps. The tree with the highest log likelihood (-21462.45) is shown. The percentage of trees in which the associated taxa clustered together is shown next to the branches. The tree is drawn to scale, with branch lengths measured in the number of substitutions per site. The analysis involved 42 nucleotide sequences, of which 40 were used as marker sequences to determine the genotype of 2 sequences. All positions containing gaps and missing data were eliminated. There was a total of 3033 positions in the final dataset.

ID	GENOTYPE	SUBTYPE	COUNTRY	ALIGNMENT[1]	BASE PAIRS
KT896494	F	F2a	Brazil	Complete Genome	3215
KX264497	F	F2a	Brazil	Complete Genome	3215

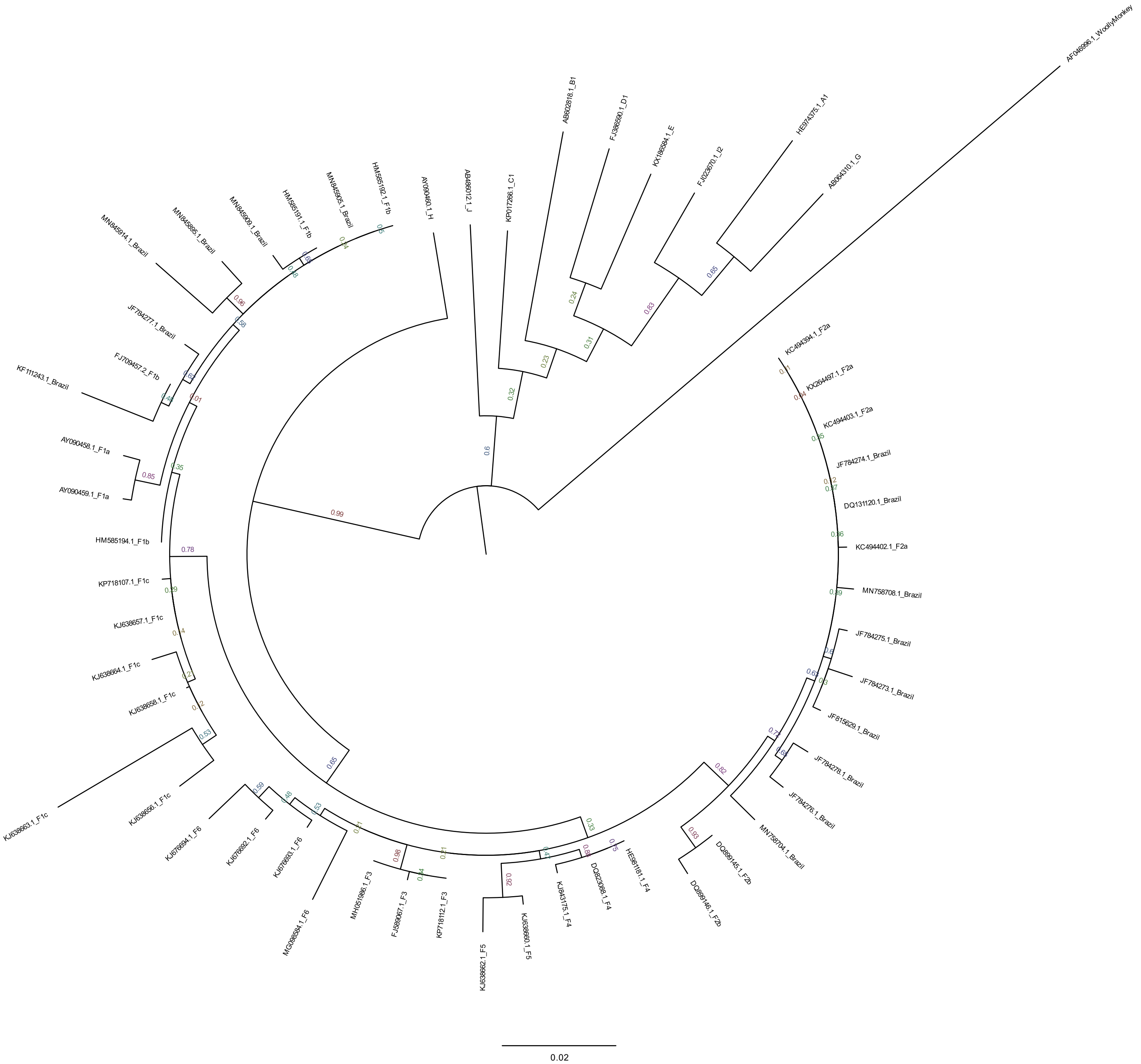

Tree 2. Molecular Phylogenetic analysis by Maximum Likelihood method conducted in MEGA7. The evolutionary history was inferred by using the Maximum Likelihood method based on the Tamura-Nei model with 1000 bootstraps. The tree with the highest log likelihood (-3416.43) is shown. The percentage of trees in which the associated taxa clustered together is shown next to the branches. The tree is drawn to scale, with branch lengths measured in the number of substitutions per site. The analysis involved 55 nucleotide sequences, of which 40 were used as marker sequences to determine the genotype of 15 sequences. All positions containing gaps and missing data were eliminated. There was a total of 673 positions in the final dataset.

ID	GENOTYPE	SUBTYPE	COUNTRY	ALIGNMENT[1]	BASE PAIRS
JF784273	F	F2a	Brazil	1-3182	1186
JF784274	F	F2a	Brazil	1-3182	1186
JF784275	F	F2a	Brazil	1-3182	1186
JF784276	F	F2a	Brazil	1-3182	1186
JF784278	F	F2a	Brazil	1-3182	1162
JF815629	F	F2a	Brazil	1-3182	1437

MN758704	F	F2a	Brazil	90-3181	1188
MN758708	F	F2a	Brazil	33-3181	1245
JF784277	F	F1b	Brazil	1-3182	1186
KF111243	F	F1b	Brazil	1-3182	1184
MN845895	F	F1b	Brazil	1-3182	1113
MN845905	F	F1b	Brazil	1-3182	1123
MN845909	F	F1b	Brazil	1-3182	1122
MN845914	F	F1b	Brazil	1-3182	1112
DQ131120	F	F2a	Brazil	1-3182	1204

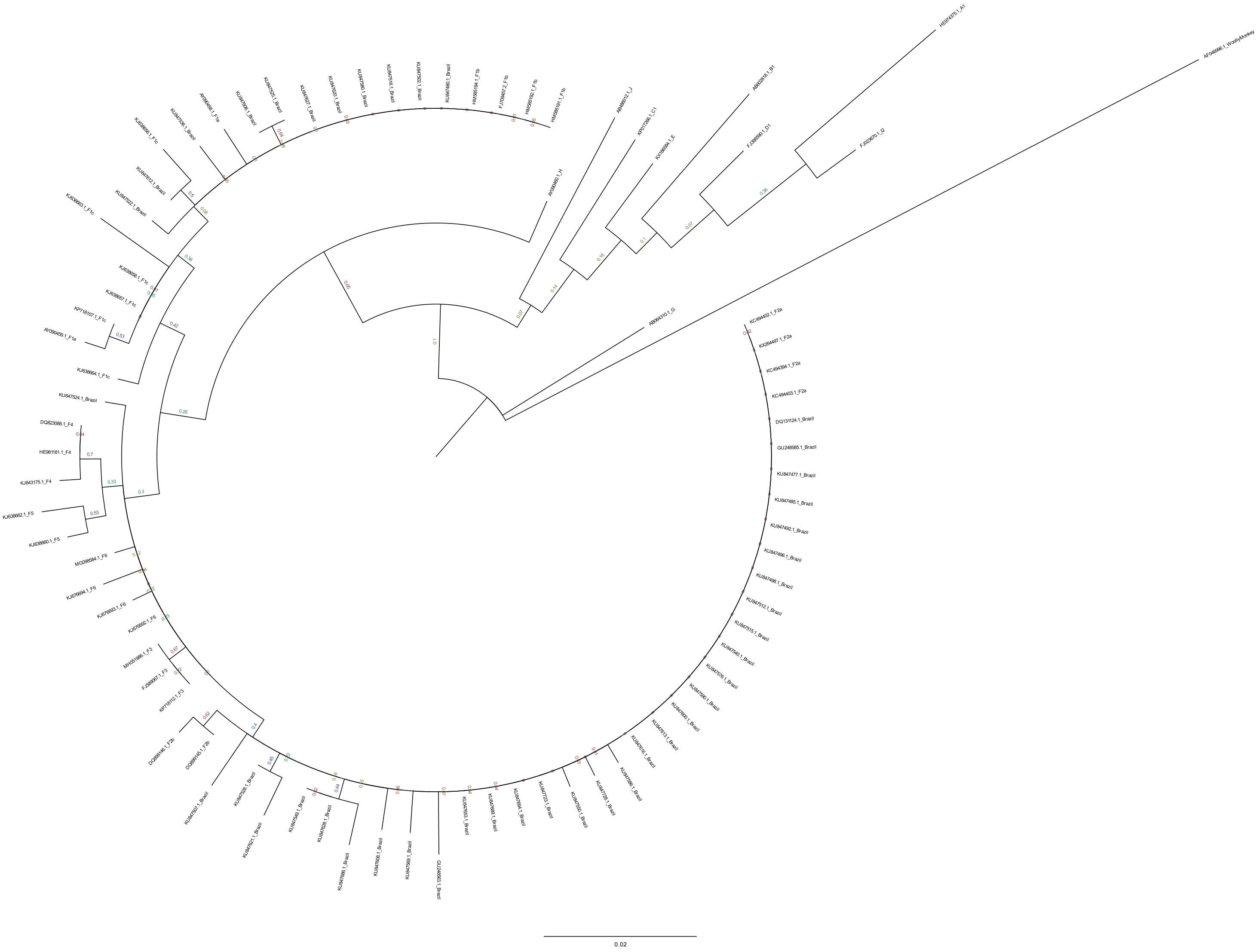

Tree 3. Molecular Phylogenetic analysis by Maximum Likelihood method conducted in MEGA7. The evolutionary history was inferred by using the Maximum Likelihood method based on the Tamura-Nei model with 1000 bootstraps. The tree with the highest log likelihood (-1540.93) is shown. The percentage of trees in which the associated taxa clustered together is shown next to the branches. The tree is drawn to scale, with branch lengths measured in the number of substitutions per site. The analysis involved 83 nucleotide sequences, of which 40 were used as marker sequences to determine the genotype of 43 sequences. All positions containing gaps and missing data were eliminated. There was a total of 367 positions in the final dataset.

ID	GENOTYPE	SUBTYPE	COUNTRY	ALIGNMENT[1]	BASE PAIRS
KU847480	F	F1b	Brazil	132-1163	1032
KU847502	F	F1b	Brazil	132-1163	1032
KU847516	F	F1b	Brazil	132-1163	1032
KU847522	F	F1c	Brazil	132-1163	1032
KU847525	F	F1b	Brazil	132-1163	1032
KU847536	F	F1b	Brazil	132-932	801
KU847580	F	F1b	Brazil	132-1163	1032
KU847606	F	F1b	Brazil	132-1163	1032
KU847620	F	F1b	Brazil	132-1163	1032
KU847627	F	F1b	Brazil	132-1163	1032

KU847612	F	F1c	Brazil	132-1163	1032
KU847477	F	F2a	Brazil	132-1163	1032
KU847485	F	F2a	Brazil	132-1163	1032
KU847492	F	F2a	Brazil	132-1163	1032
KU847496	F	F2a	Brazil	132-1163	1032
KU847498	F	F2a	Brazil	135-1163	1029
KU847507	F	F2a	Brazil	132-1163	1032
KU847512	F	F2a	Brazil	132-1163	1032
KU847515	F	F2a	Brazil	132-1163	1032
KU847524	F	F2a	Brazil	132-1163	1032
KU847528	F	F2a	Brazil	132-932	801
KU847540	F	F2a	Brazil	132-1163	1032
KU847549	F	F2a	Brazil	132-1163	1032
KU847550	F	F2a	Brazil	132-1163	1032
KU847569	F	F2a	Brazil	135-1163	1029
KU847576	F	F2a	Brazil	153-1163	1011
KU847586	F	F2a	Brazil	132-1163	1032
KU847590	F	F2a	Brazil	132-1163	1032
KU847600	F	F2a	Brazil	132-1163	1032
KU847608	F	F2a	Brazil	132-1163	1032
KU847613	F	F2a	Brazil	132-1163	1032
KU847616	F	F2a	Brazil	132-1163	1032
KU847621	F	F2a	Brazil	228-1163	936
KU847628	F	F2a	Brazil	132-1163	1032
KU847653	F	F2a	Brazil	132-929	798
KU847689	F	F2a	Brazil	132-836	705
KU847694	F	F2a	Brazil	132-1163	1032
KU847723	F	F2a	Brazil	132-1163	1032
KU847728	F	F2a	Brazil	132-1163	1029
GU248563	F	F2a	Brazil	3-1163	839
GU248585	F	F2a	Brazil	3-1163	824
KU847686	F	F2a	Brazil	132-1163	1032
DQ131124	F	F2a	Brazil	3-1163	847

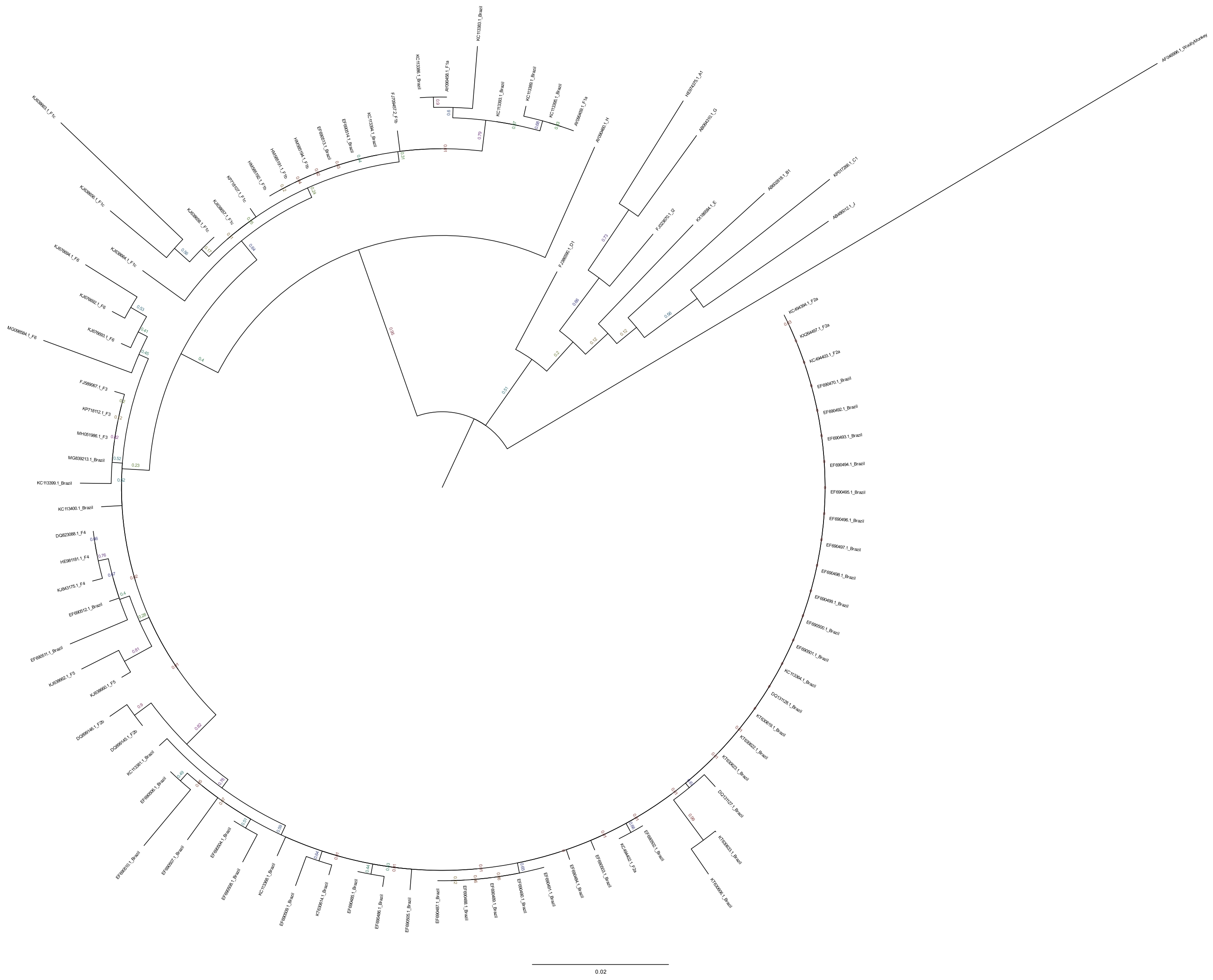

Tree 4. Molecular Phylogenetic analysis by Maximum Likelihood method conducted in MEGA7. The evolutionary history was inferred by using the Maximum Likelihood method based on the Tamura-Nei model with 1000 bootstraps. The tree with the highest log likelihood (-3298.02) is shown. The percentage of trees in which the associated taxa clustered together is shown next to the branches. The tree is drawn to scale, with branch lengths measured in the number of substitutions per site. The analysis involved 92 nucleotide sequences, of which 40 were used as marker sequences to determine the genotype of 52 sequences. All positions containing gaps and missing data were eliminated. There was a total of 661 positions in the final dataset.

ID	GENOTYPE	SUBTYPE	COUNTRY	ALIGNMENT[1]	BASE PAIRS
KT630606	F	F2a	Brazil	157-837	681
KT630614	F	F2a	Brazil	157-838	682
KT630619	F	F2a	Brazil	157-839	683
KT630622	F	F2a	Brazil	157-840	684
KT630623	F	F2a	Brazil	157-841	685
KT630633	F	F2a	Brazil	157-842	686
MG839213	F	F3	Brazil	157-842	686
KC113383	F	F1a	Brazil	157-837	681
KC113386	F	F1a	Brazil	157-837	681

KC113389	F	F1a	Brazil	157-837	681
KC113393	F	F1a	Brazil	157-837	681
KC113395	F	F1a	Brazil	157-837	681
EF690513	F	F1b	Brazil	157-837	681
EF690514	F	F1b	Brazil	157-837	681
KC113394	F	F1b	Brazil	157-837	681
DQ131127	F	F2a	Brazil	157-838	682
DQ131128	F	F2a	Brazil	157-838	682
EF690470	F	F2a	Brazil	157-837	681
EF690484	F	F2a	Brazil	157-837	681
EF690485	F	F2a	Brazil	157-837	681
EF690486	F	F2a	Brazil	157-837	681
EF690487	F	F2a	Brazil	157-837	681
EF690488	F	F2a	Brazil	157-837	681
EF690489	F	F2a	Brazil	157-837	681
EF690490	F	F2a	Brazil	157-837	681
EF690491	F	F2a	Brazil	157-837	681
EF690492	F	F2a	Brazil	157-837	681
EF690493	F	F2a	Brazil	157-837	681
EF690494	F	F2a	Brazil	157-837	681
EF690495	F	F2a	Brazil	157-837	681
EF690496	F	F2a	Brazil	157-837	681
EF690497	F	F2a	Brazil	157-837	681
EF690498	F	F2a	Brazil	157-837	681
EF690499	F	F2a	Brazil	157-837	681
EF690500	F	F2a	Brazil	157-837	681
EF690501	F	F2a	Brazil	157-837	681
EF690502	F	F2a	Brazil	157-837	681
EF690503	F	F2a	Brazil	157-837	681
EF690504	F	F2a	Brazil	157-837	681
EF690505	F	F2a	Brazil	157-837	681
EF690506	F	F2a	Brazil	157-837	681
EF690507	F	F2a	Brazil	157-837	681
EF690508	F	F2a	Brazil	157-837	681
EF690509	F	F2a	Brazil	157-837	681
EF690510	F	F2a	Brazil	157-837	681
KC113364	F	F2a	Brazil	157-837	681
KC113368	F	F2a	Brazil	157-837	681
KC113381	F	F2a	Brazil	157-837	681
KC113399	F	F3	Brazil	157-837	681
KC113400	F	F3	Brazil	157-837	681
EF690511	F	F4	Brazil	157-837	681
EF690512	F	F4	Brazil	157-837	681

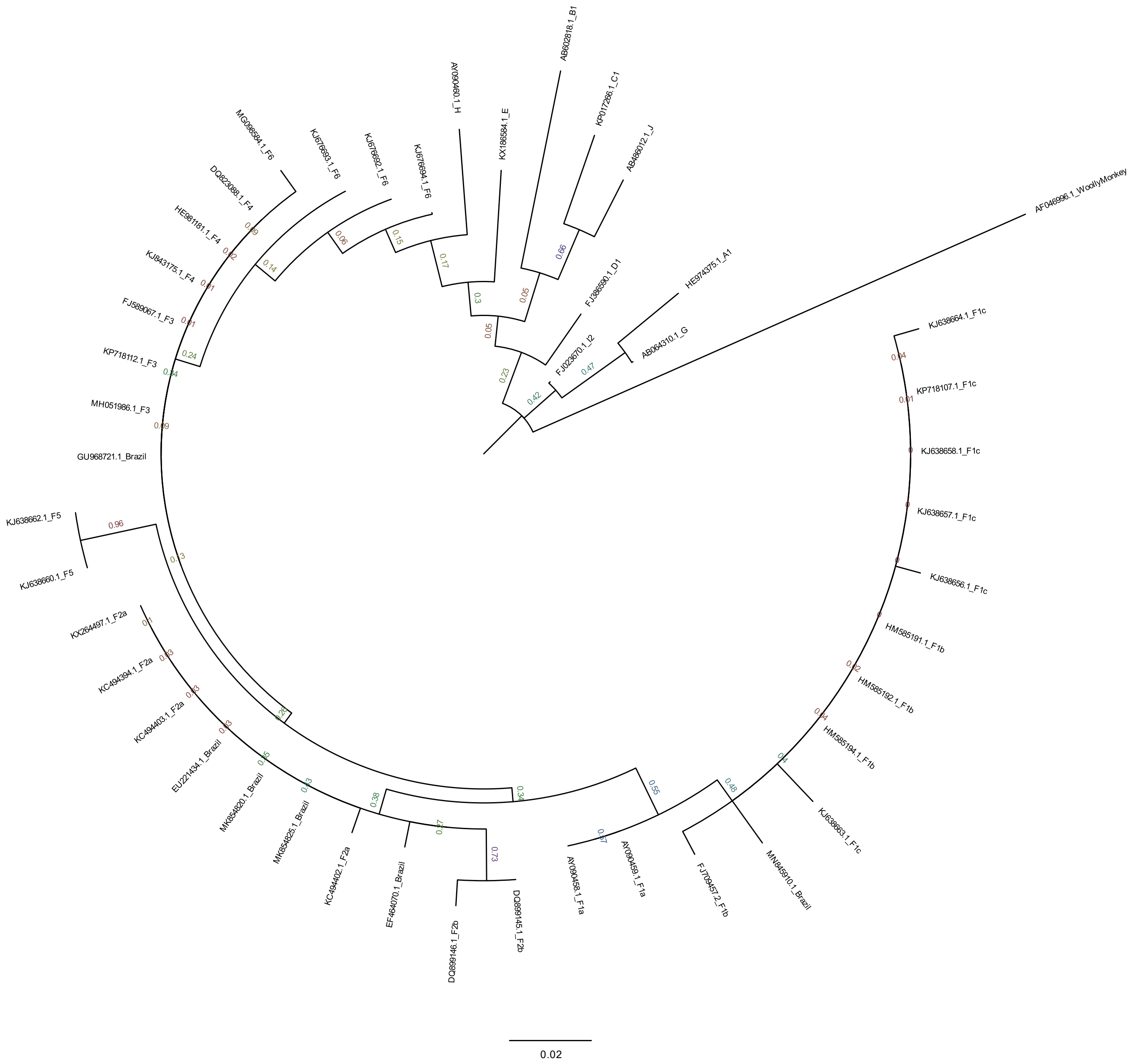

Tree 5. Molecular Phylogenetic analysis by Maximum Likelihood method conducted in MEGA7. The evolutionary history was inferred by using the Maximum Likelihood method based on the Tamura-Nei model with 1000 bootstraps. The tree with the highest log likelihood (-704.06) is shown. The percentage of trees in which the associated taxa clustered together is shown next to the branches. The tree is drawn to scale, with branch lengths measured in the number of substitutions per site. The analysis involved 46 nucleotide sequences, of which 40 were used as marker sequences to determine the genotype of 6 sequences. All positions containing gaps and missing data were eliminated. There was a total of 159 positions in the final dataset.

ID	GENOTYPE	SUBTYPE	COUNTRY	ALIGNMENT[1]	BASE PAIRS
EU221434	F	F2a	Brazil	519-997	479
MK854820	F	F2a	Brazil	320-951	632
MK854825	F	F2a	Brazil	320-951	632
EF464070	F	F2b	Brazil	417-841	425
MN845910	F	F1b	Brazil	101-682	582
GU968721	F	F3	Brazil	312-789	478

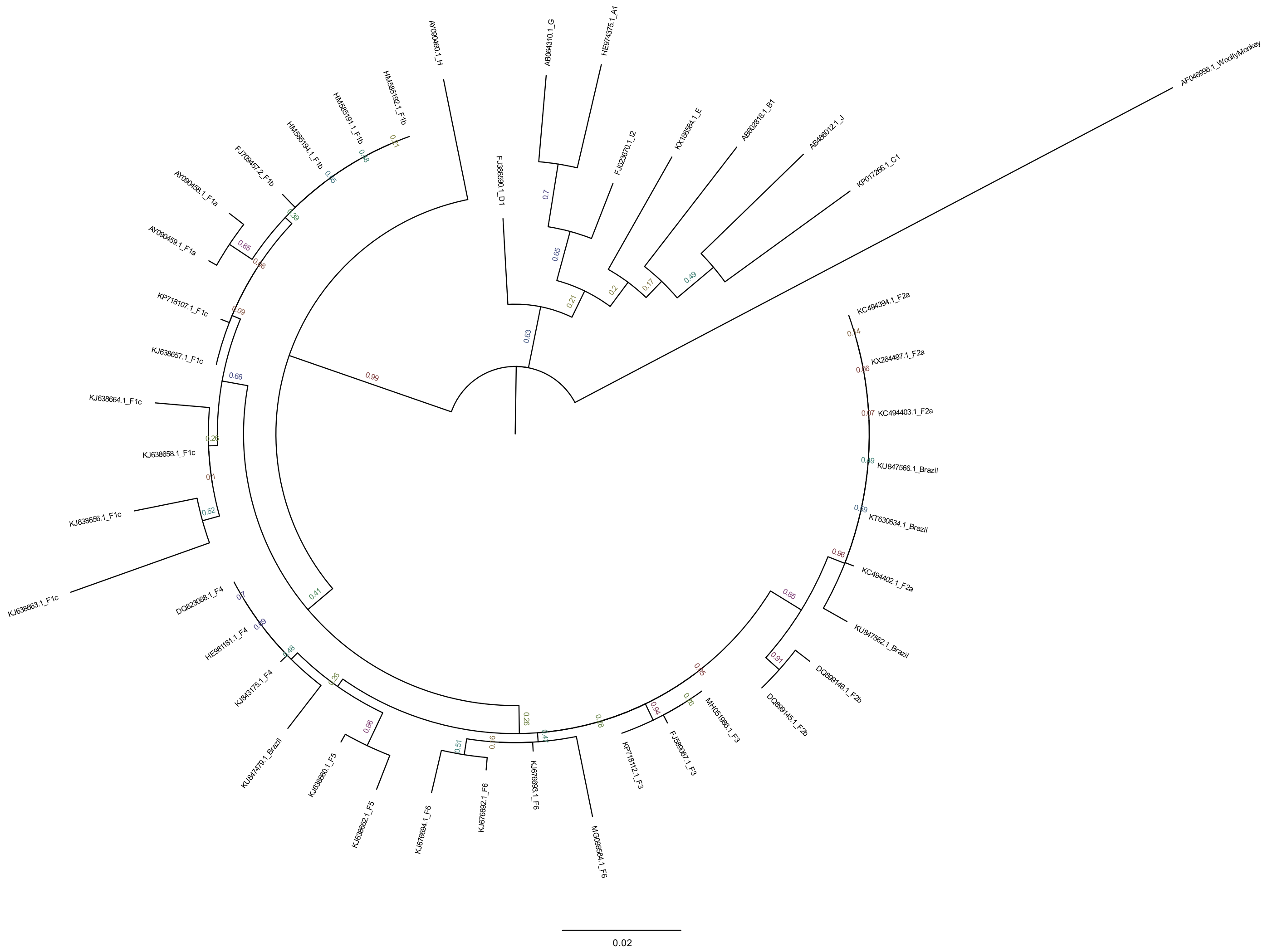

Tree 6. Molecular Phylogenetic analysis by Maximum Likelihood method conducted in MEGA7. The evolutionary history was inferred by using the Maximum Likelihood method based on the Tamura-Nei model with 1000 bootstraps. The tree with the highest log likelihood (-2909.26) is shown. The percentage of trees in which the associated taxa clustered together is shown next to the branches. The tree is drawn to scale, with branch lengths measured in the number of substitutions per site. The analysis involved 44 nucleotide sequences, of which 40 were used as marker sequences to determine the genotype of 4 sequences. All positions containing gaps and missing data were eliminated. There was a total of 658 positions in the final dataset.

ID	GENOTYPE	SUBTYPE	COUNTRY	ALIGNMENT[1]	BASE PAIRS
KT630634	F	F2a	Brazil	157-837	681
KU847479	F	F4	Brazil	135-1163	1029
KU847562	F	F2a	Brazil	132-1163	1032
KU847566	F	F2a	Brazil	135-1163	1029

CANADA

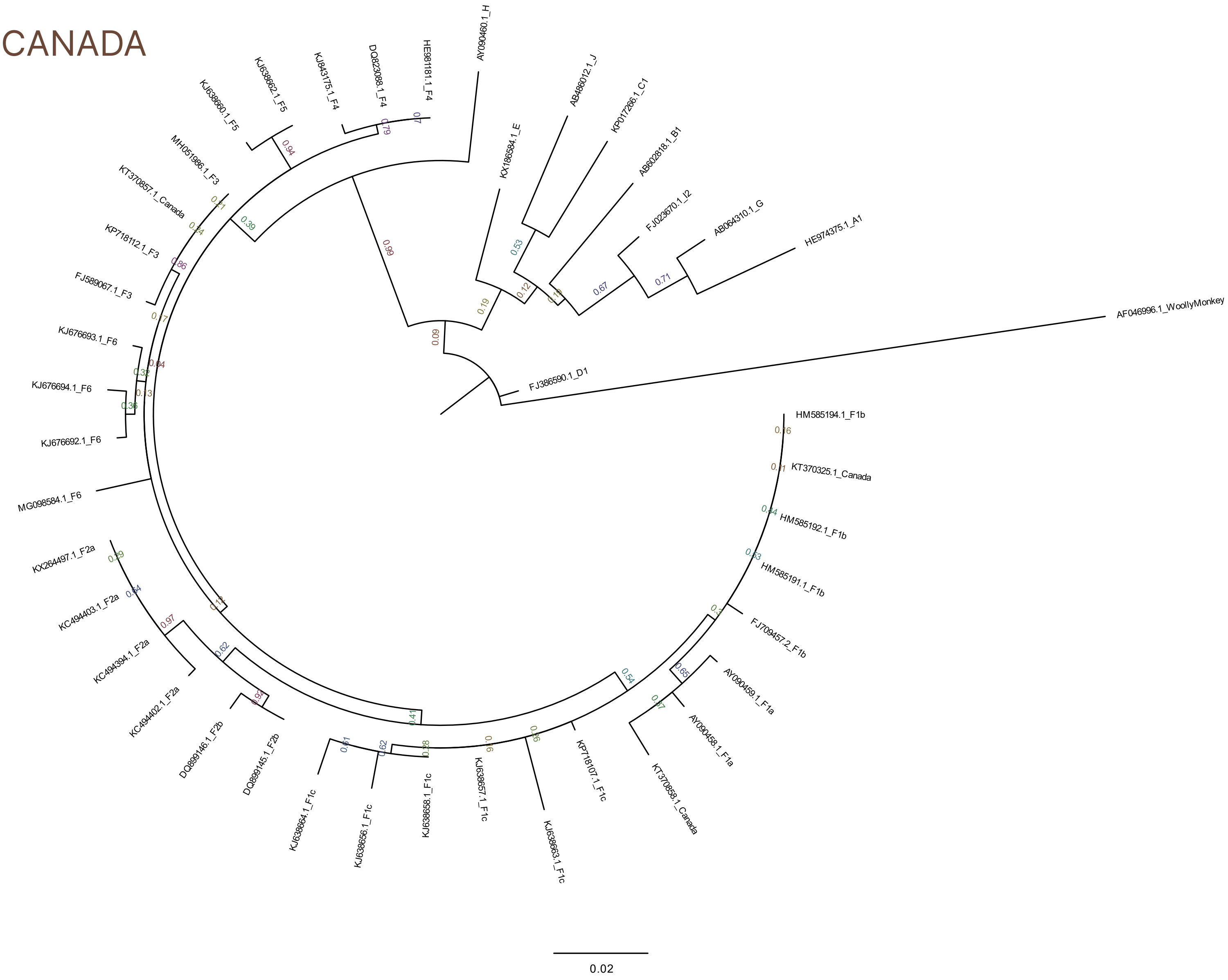

Tree 1. Molecular Phylogenetic analysis by Maximum Likelihood method conducted in MEGA7. The evolutionary history was inferred by using the Maximum Likelihood method based on the Tamura-Nei model with 1000 bootstraps. The tree with the highest log likelihood (-2154.22) is shown. The percentage of trees in which the associated taxa clustered together is shown next to the branches. The tree is drawn to scale, with branch lengths measured in the number of substitutions per site. The analysis involved 43 nucleotide sequences, of which 40 were used as marker sequences to determine the genotype of 3 sequences. All positions containing gaps and missing data were eliminated. There was a total of 507 positions in the final dataset.

ID	GENOTYPE	SUBTYPE	COUNTRY	ALIGNMENT[1]	BASE PAIRS
KT370858	F	F1a	Canada	313-831	519
KT370325	F	F1b	Canada	313-831	519
KT370857	F	F3	Canada	313-831	519

CHILE

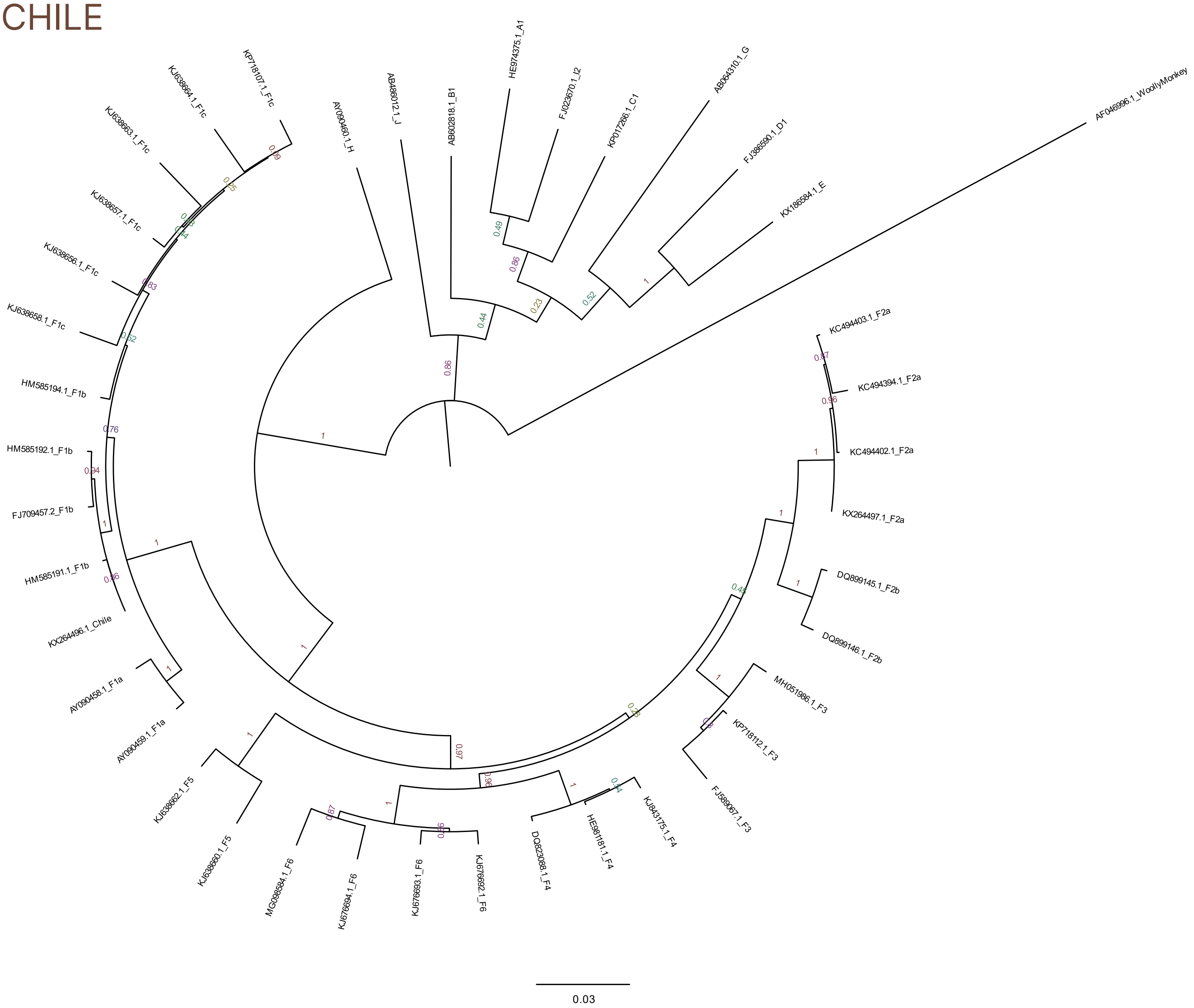

Tree 1. Molecular Phylogenetic analysis by Maximum Likelihood method conducted in MEGA7. The evolutionary history was inferred by using the Maximum Likelihood method based on the Tamura-Nei model with 1000 bootstraps. The tree with the highest log likelihood (-21347.45) is shown. The percentage of trees in which the associated taxa clustered together is shown next to the branches. The tree is drawn to scale, with branch lengths measured in the number of substitutions per site. The analysis involved 41 nucleotide sequences, of which 40 were used as marker sequences to determine the genotype of 1 sequence. All positions containing gaps and missing data were eliminated. There was a total of 3035 positions in the final dataset.

ID	GENOTYPE	SUBTYPE	COUNTRY	ALIGNMENT[1]	BASE PAIRS
KX264496	F	F1b	Chile	Complete Genome	3215

COLOMBIA

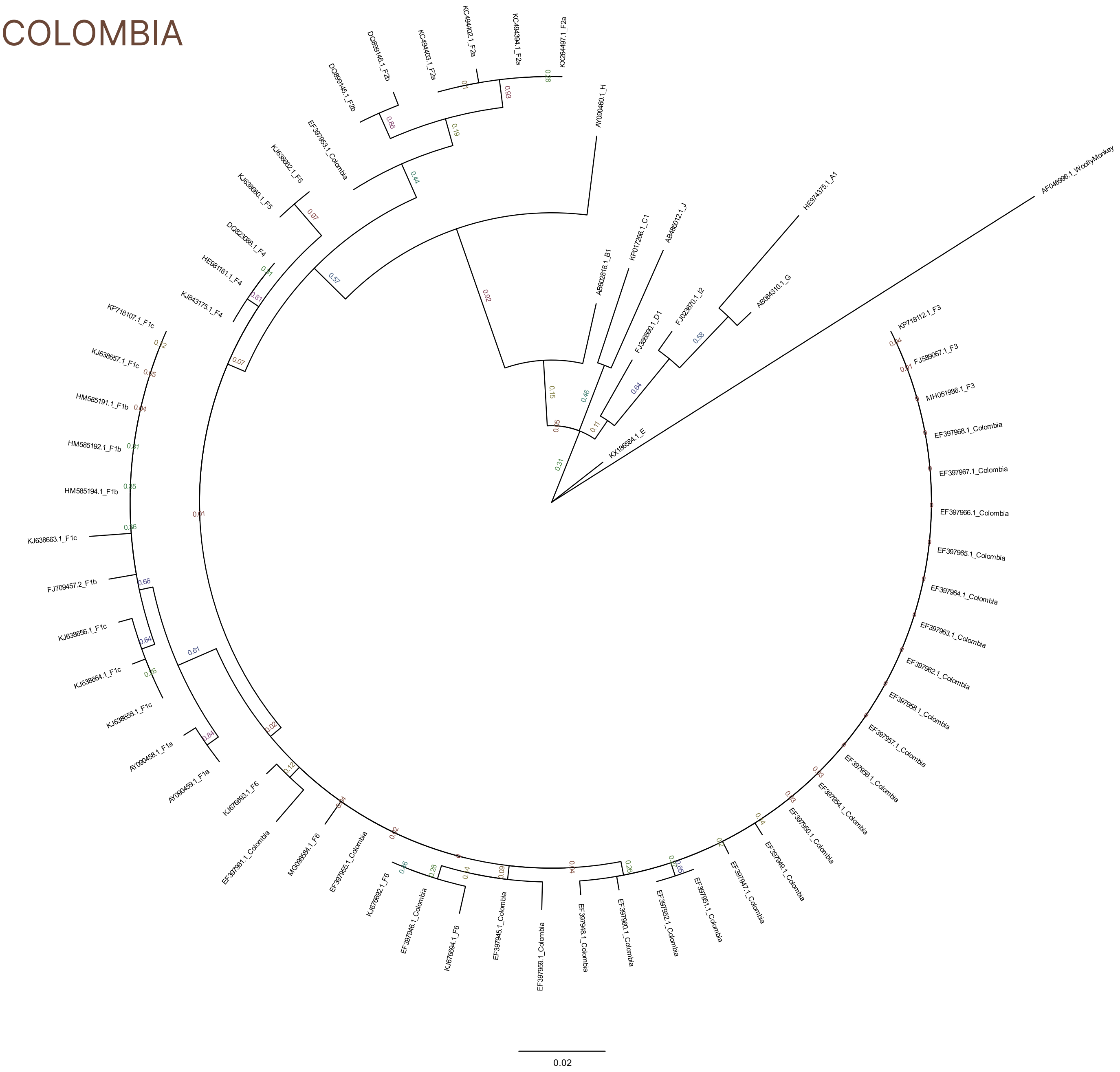

Tree 1. Molecular Phylogenetic analysis by Maximum Likelihood method conducted in MEGA7. The evolutionary history was inferred by using the Maximum Likelihood method based on the Tamura-Nei model with 1000 bootstraps. The tree with the highest log likelihood (-1457.63) is shown. The percentage of trees in which the associated taxa clustered together is shown next to the branches. The tree is drawn to scale, with branch lengths measured in the number of substitutions per site. The analysis involved 64 nucleotide sequences, of which 40 were used as marker sequences to determine the genotype of 24 sequences. All positions containing gaps and missing data were eliminated. There was a total of 313 positions in the final dataset.

ID	GENOTYPE	SUBTYPE	COUNTRY	ALIGNMENT[1]	BASE PAIRS
EF397953	F	F2b	Colombia	434-753	320
EF397947	F	F3	Colombia	434-753	320
EF397948	F	F3	Colombia	434-753	320
EF397949	F	F3	Colombia	434-753	320
EF397950	F	F3	Colombia	434-753	320
EF397951	F	F3	Colombia	434-753	320

EF397952	F	F3	Colombia	434-753	320
EF397954	F	F3	Colombia	434-753	320
EF397956	F	F3	Colombia	434-753	320
EF397957	F	F3	Colombia	434-753	320
EF397958	F	F3	Colombia	434-753	320
EF397960	F	F3	Colombia	434-753	320
EF397962	F	F3	Colombia	434-753	320
EF397963	F	F3	Colombia	434-753	320
EF397964	F	F3	Colombia	434-753	320
EF397965	F	F3	Colombia	434-753	320
EF397966	F	F3	Colombia	434-753	320
EF397967	F	F3	Colombia	434-753	320
EF397968	F	F3	Colombia	434-753	320
EF397945	F	F6	Colombia	434-753	320
EF397946	F	F6	Colombia	434-753	320
EF397955	F	F6	Colombia	434-753	320
EF397959	F	F6	Colombia	434-753	320
EF397961	F	F6	Colombia	434-753	320

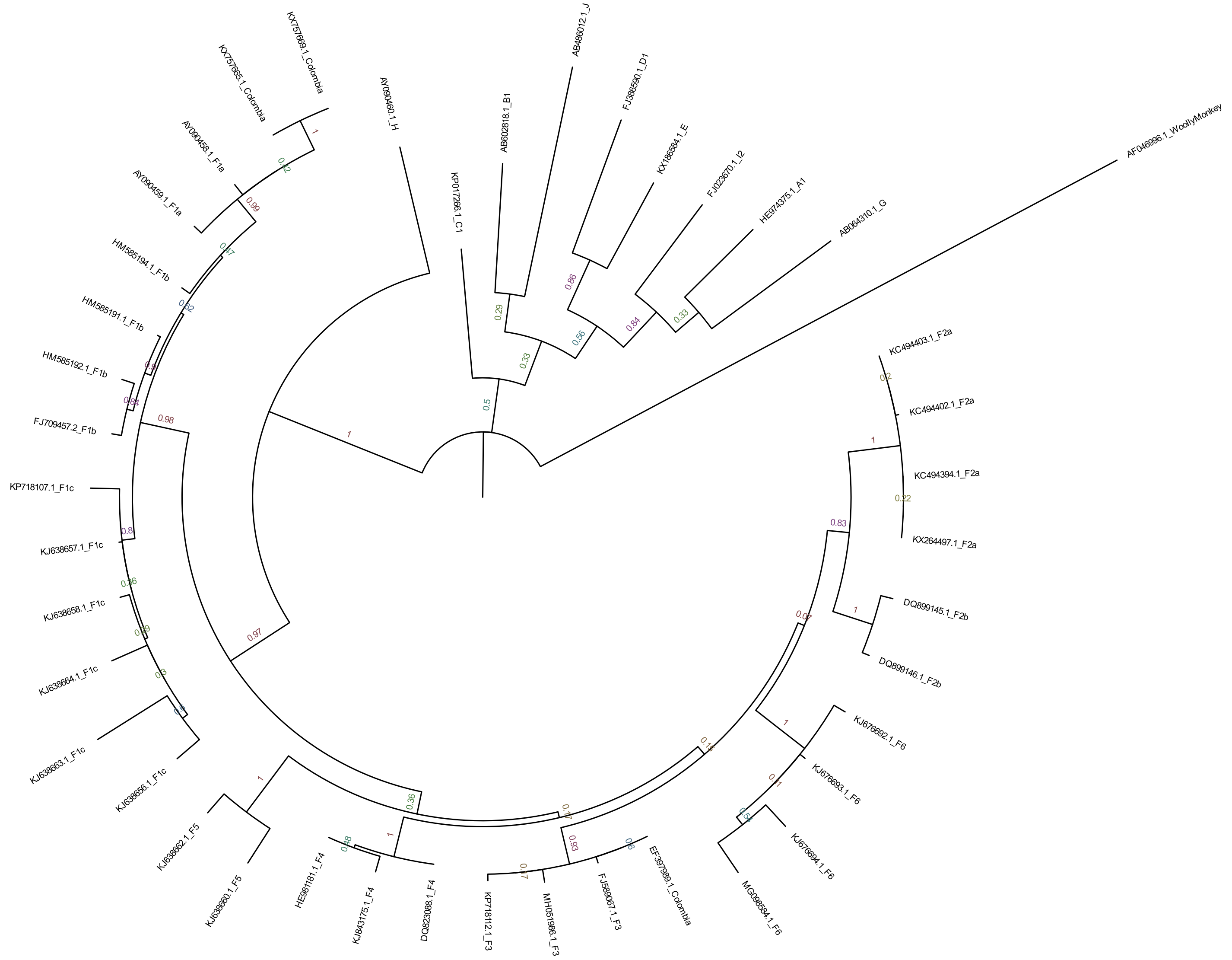

Tree 2. Molecular Phylogenetic analysis by Maximum Likelihood method conducted in MEGA7. The evolutionary history was inferred by using the Maximum Likelihood method based on the Tamura-Nei model with 1000 bootstraps. The tree with the highest log likelihood (-7401.01) is shown. The percentage of trees in which the associated taxa clustered together is shown next to the branches. The tree is drawn to scale, with branch lengths measured in the number of substitutions per site. The analysis involved 43 nucleotide sequences, of which 40 were used as marker sequences to determine the genotype of 3 sequences. All positions containing gaps and missing data were eliminated. There was a total of 1247 positions in the final dataset.

ID	GENOTYPE	SUBTYPE	COUNTRY	ALIGNMENT[1]	BASE PAIRS
KX757665	F	F1a	Colombia	1-3182	3134
KX757669	F	F1a	Colombia	105-2431	2327
EF397989	F	F3	Colombia	106-1398	1293

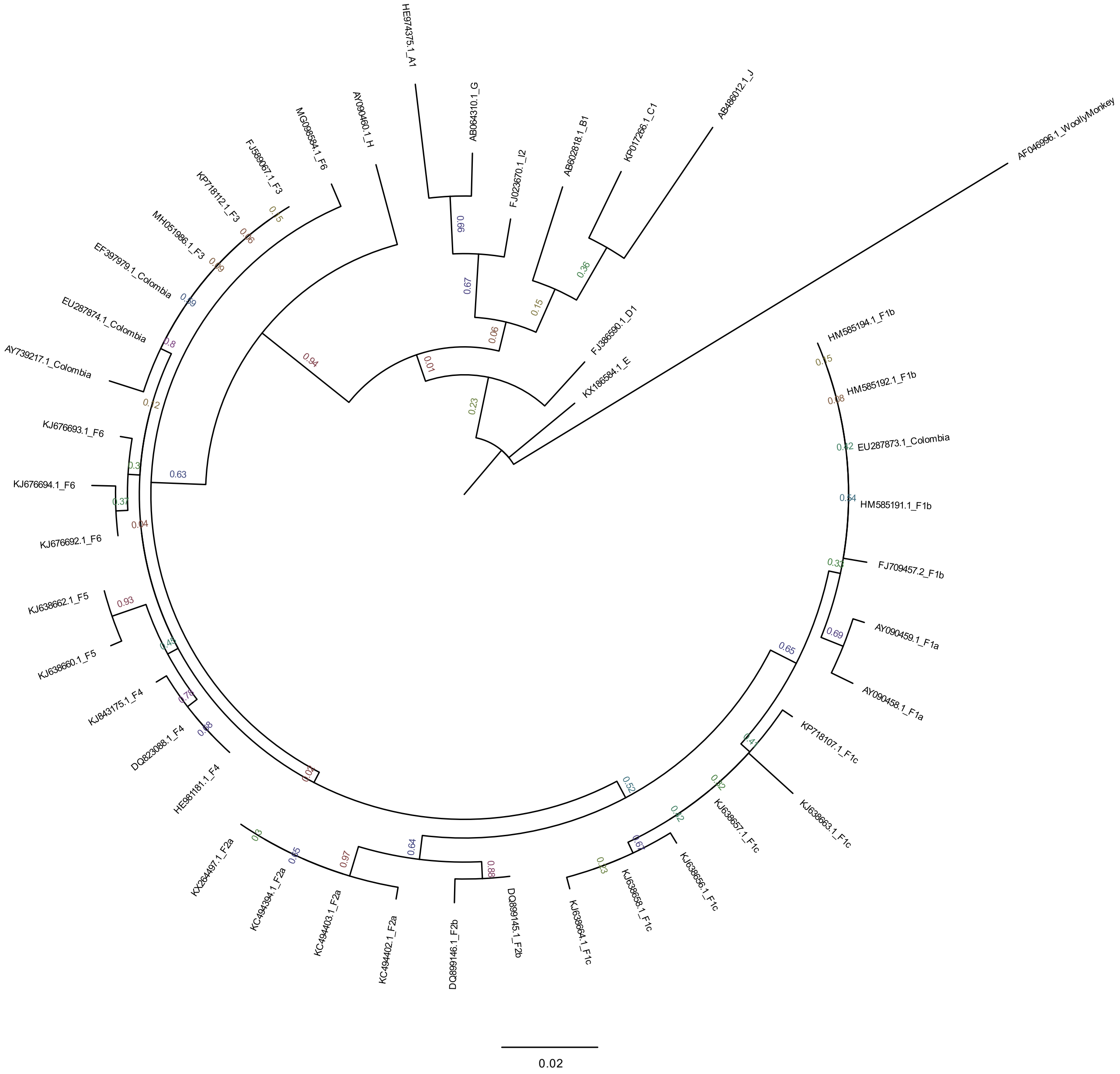

Tree 3. Molecular Phylogenetic analysis by Maximum Likelihood method conducted in MEGA7. The evolutionary history was inferred by using the Maximum Likelihood method based on the Tamura-Nei model with 1000 bootstraps. The tree with the highest log likelihood (-1715.85) is shown. The percentage of trees in which the associated taxa clustered together is shown next to the branches. The tree is drawn to scale, with branch lengths measured in the number of substitutions per site. The analysis involved 44 nucleotide sequences, of which 40 were used as marker sequences to determine the genotype of 4 sequences. All positions containing gaps and missing data were eliminated. There was a total of 404 positions in the final dataset.

ID	GENOTYPE	SUBTYPE	COUNTRY	ALIGNMENT[1]	BASE PAIRS
EU287873	F	F1b	Colombia	343-979	637
AY739217	F	F3	Colombia	157-837	681
EF397979	F	F3	Colombia	157-753	597
EU287874	F	F3	Colombia	343-979	637

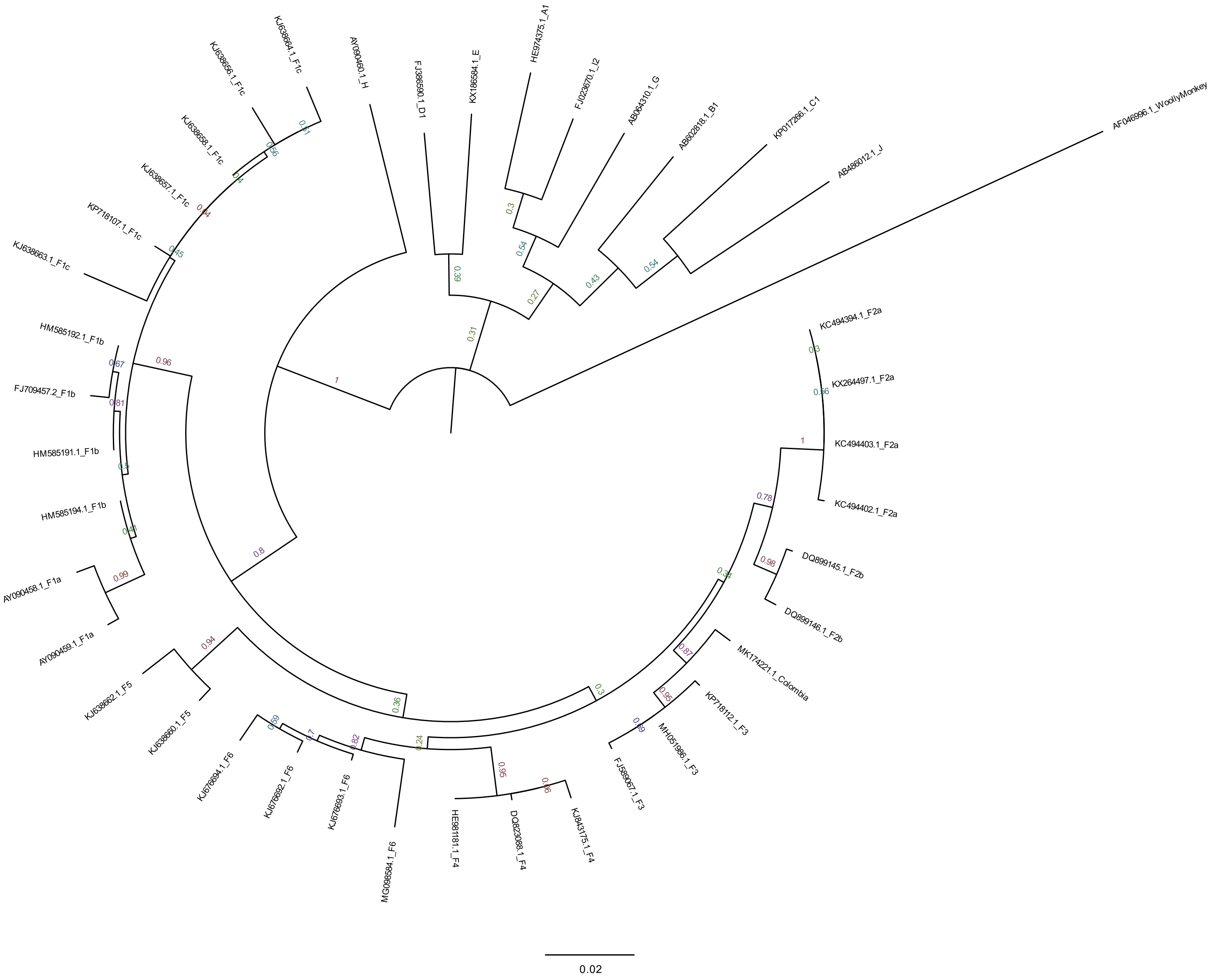

Tree 4. Molecular Phylogenetic analysis by Maximum Likelihood method conducted in MEGA7. The evolutionary history was inferred by using the Maximum Likelihood method based on the Tamura-Nei model with 1000 bootstraps. The tree with the highest log likelihood (-3931.09) is shown. The percentage of trees in which the associated taxa clustered together is shown next to the branches. The tree is drawn to scale, with branch lengths measured in the number of substitutions per site. The analysis involved 41 nucleotide sequences, of which 40 were used as marker sequences to determine the genotype of 1 sequence. All positions containing gaps and missing data were eliminated. There was a total of 722 positions in the final dataset.

ID	GENOTYPE	SUBTYPE	COUNTRY	ALIGNMENT[1]	BASE PAIRS
MK174221	F	F3	Colombia	216-955	740

COSTA RICA

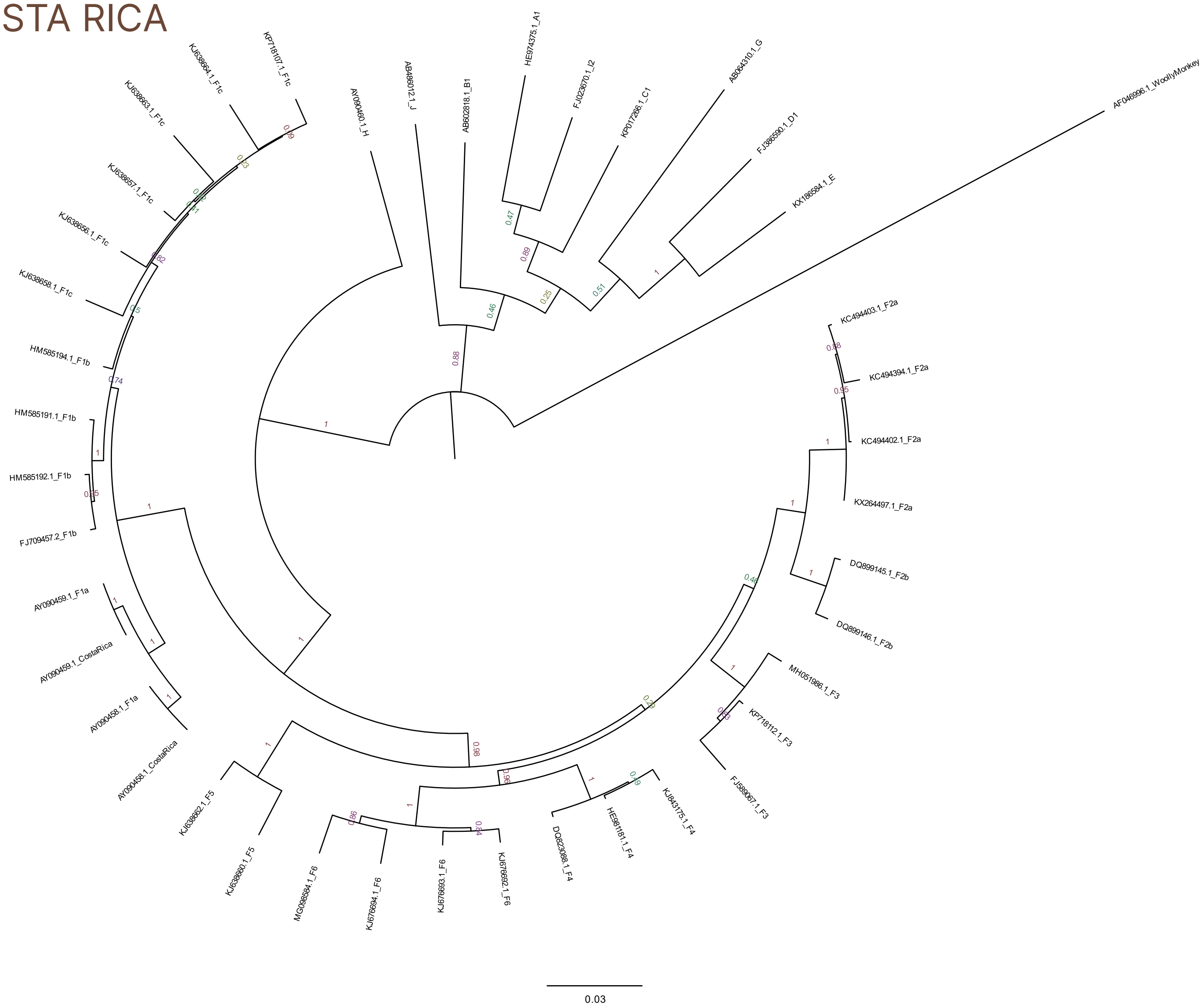

Tree 1. Molecular Phylogenetic analysis by Maximum Likelihood method conducted in MEGA7. The evolutionary history was inferred by using the Maximum Likelihood method based on the Tamura-Nei model with 1000 bootstraps. The tree with the highest log likelihood (-21347.40) is shown. The percentage of trees in which the associated taxa clustered together is shown next to the branches. The tree is drawn to scale, with branch lengths measured in the number of substitutions per site. The analysis involved 42 nucleotide sequences, of which 40 were used as marker sequences to determine the genotype of 2 sequences. All positions containing gaps and missing data were eliminated. There was a total of 3035 positions in the final dataset.

ID	GENOTYPE	SUBTYPE	COUNTRY	ALIGNMENT[1]	BASE PAIRS
AY090458	F	F1a	Costa Rica	Complete Genome	3215
AY090459	F	F1a	Costa Rica	Complete Genome	3215

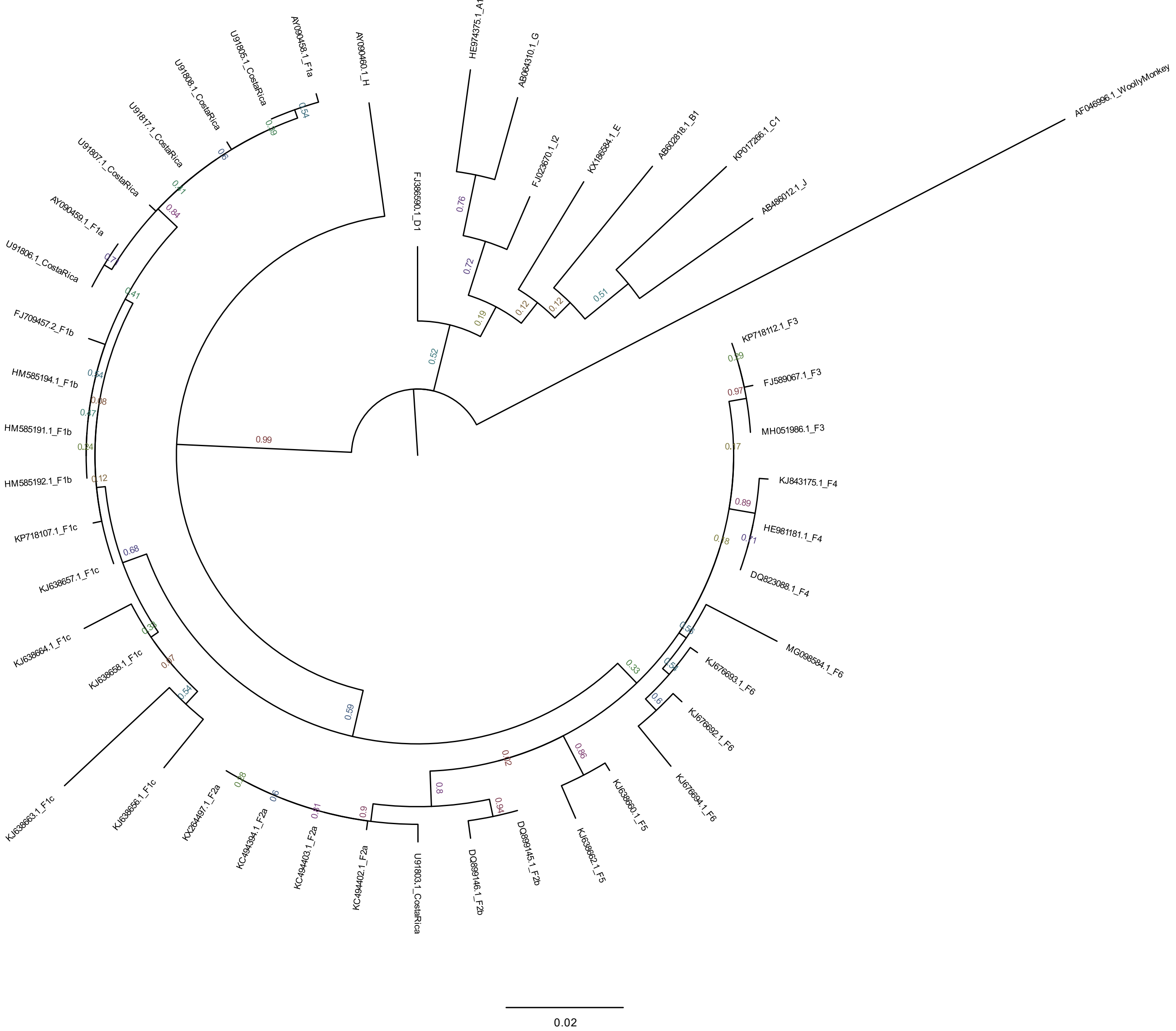

Tree 2. Molecular Phylogenetic analysis by Maximum Likelihood method conducted in MEGA7. The evolutionary history was inferred by using the Maximum Likelihood method based on the Tamura-Nei model with 1000 bootstraps. The tree with the highest log likelihood (-2948.64) is shown. The percentage of trees in which the associated taxa clustered together is shown next to the branches. The tree is drawn to scale, with branch lengths measured in the number of substitutions per site. The analysis involved 46 nucleotide sequences, of which 40 were used as marker sequences to determine the genotype of 6 sequences. All positions containing gaps and missing data were eliminated. There was a total of 667 positions in the final dataset.

ID	GENOTYPE	SUBTYPE	COUNTRY	ALIGNMENT[1]	BASE PAIRS
U91805	F	F1a	Costa Rica	157-837	681
U91806	F	F1a	Costa Rica	157-837	681
U91807	F	F1a	Costa Rica	157-837	681
U91808	F	F1a	Costa Rica	157-837	681
U91817	F	F1a	Costa Rica	157-837	681
U91803	F	F2a	Costa Rica	157-837	681

EL SALVADOR

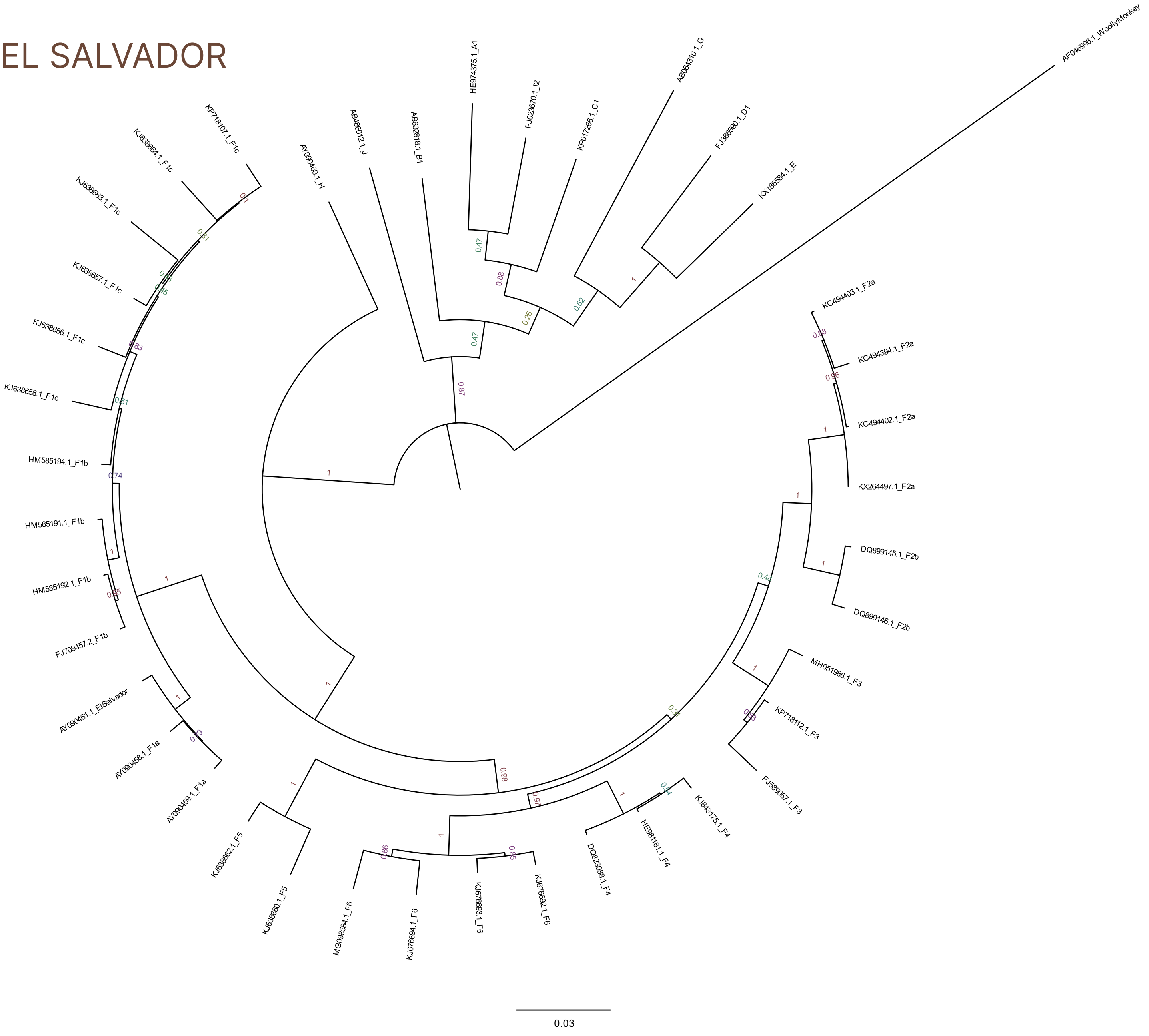

Tree 1. Molecular Phylogenetic analysis by Maximum Likelihood method conducted in MEGA7. The evolutionary history was inferred by using the Maximum Likelihood method based on the Tamura-Nei model with 1000 bootstraps. The tree with the highest log likelihood (-21437.24) is shown. The percentage of trees in which the associated taxa clustered together is shown next to the branches. The tree is drawn to scale, with branch lengths measured in the number of substitutions per site. The analysis involved 41 nucleotide sequences, of which 40 were used as marker sequences to determine the genotype of 1 sequence. All positions containing gaps and missing data were eliminated. There was a total of 3035 positions in the final dataset.

ID	GENOTYPE	SUBTYPE	COUNTRY	ALIGNMENT[1]	BASE PAIRS
AY090461	F	F1a	El Salvador	Complete Genome	3215

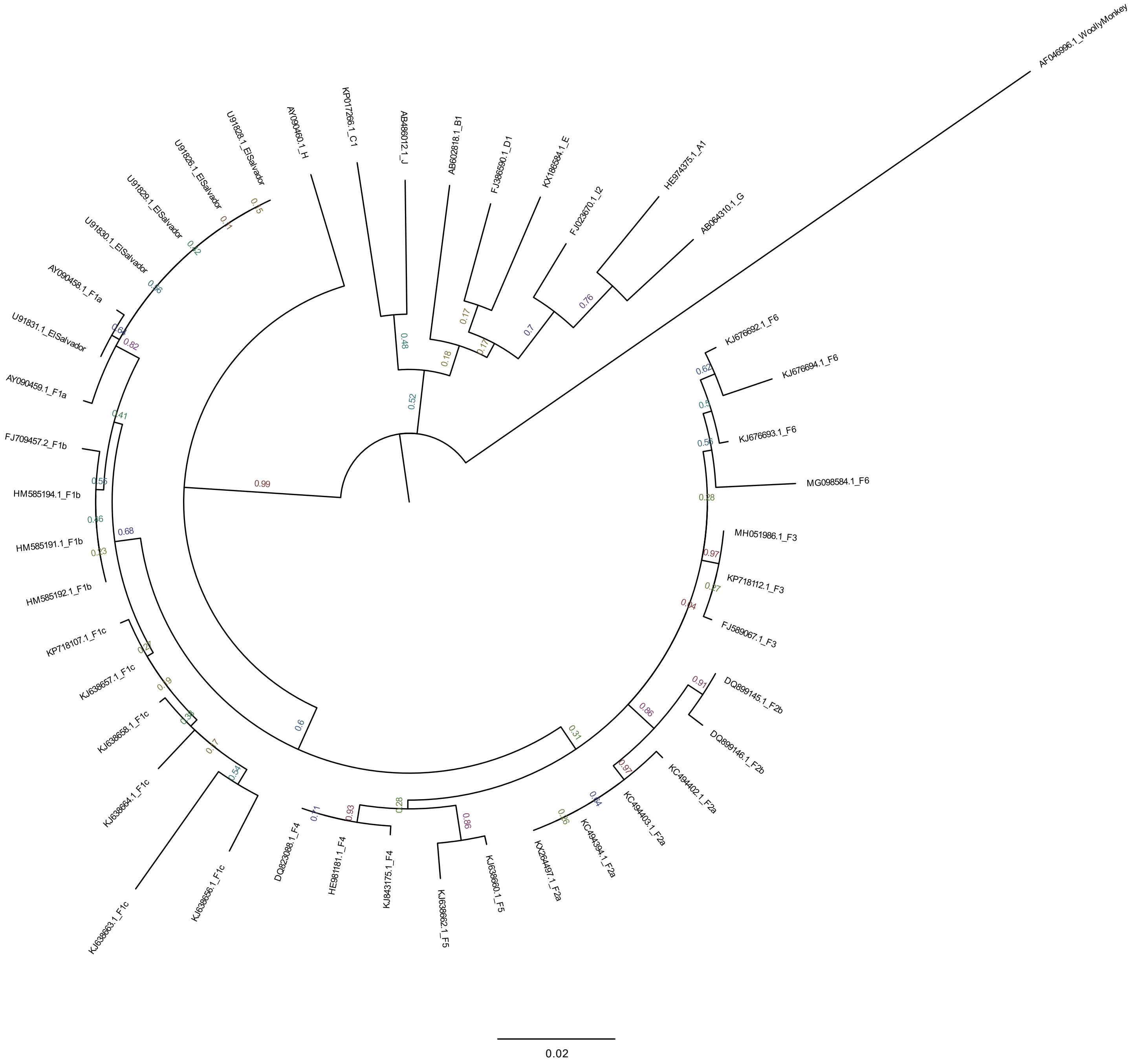

Tree 2. Molecular Phylogenetic analysis by Maximum Likelihood method conducted in MEGA7. The evolutionary history was inferred by using the Maximum Likelihood method based on the Tamura-Nei model with 1000 bootstraps. The tree with the highest log likelihood (-2918.02) is shown. The percentage of trees in which the associated taxa clustered together is shown next to the branches. The tree is drawn to scale, with branch lengths measured in the number of substitutions per site. The analysis involved 45 nucleotide sequences, of which 40 were used as marker sequences to determine the genotype of 5 sequences. All positions containing gaps and missing data were eliminated. There was a total of 667 positions in the final dataset.

ID	GENOTYPE	SUBTYPE	COUNTRY	ALIGNMENT[1]	BASE PAIRS
U91826	F	F1a	El Salvador	157-837	681
U91828	F	F1a	El Salvador	157-837	681
U91829	F	F1a	El Salvador	157-837	681
U91830	F	F1a	El Salvador	157-837	681
U91831	F	F1a	El Salvador	157-837	681

GUATEMALA

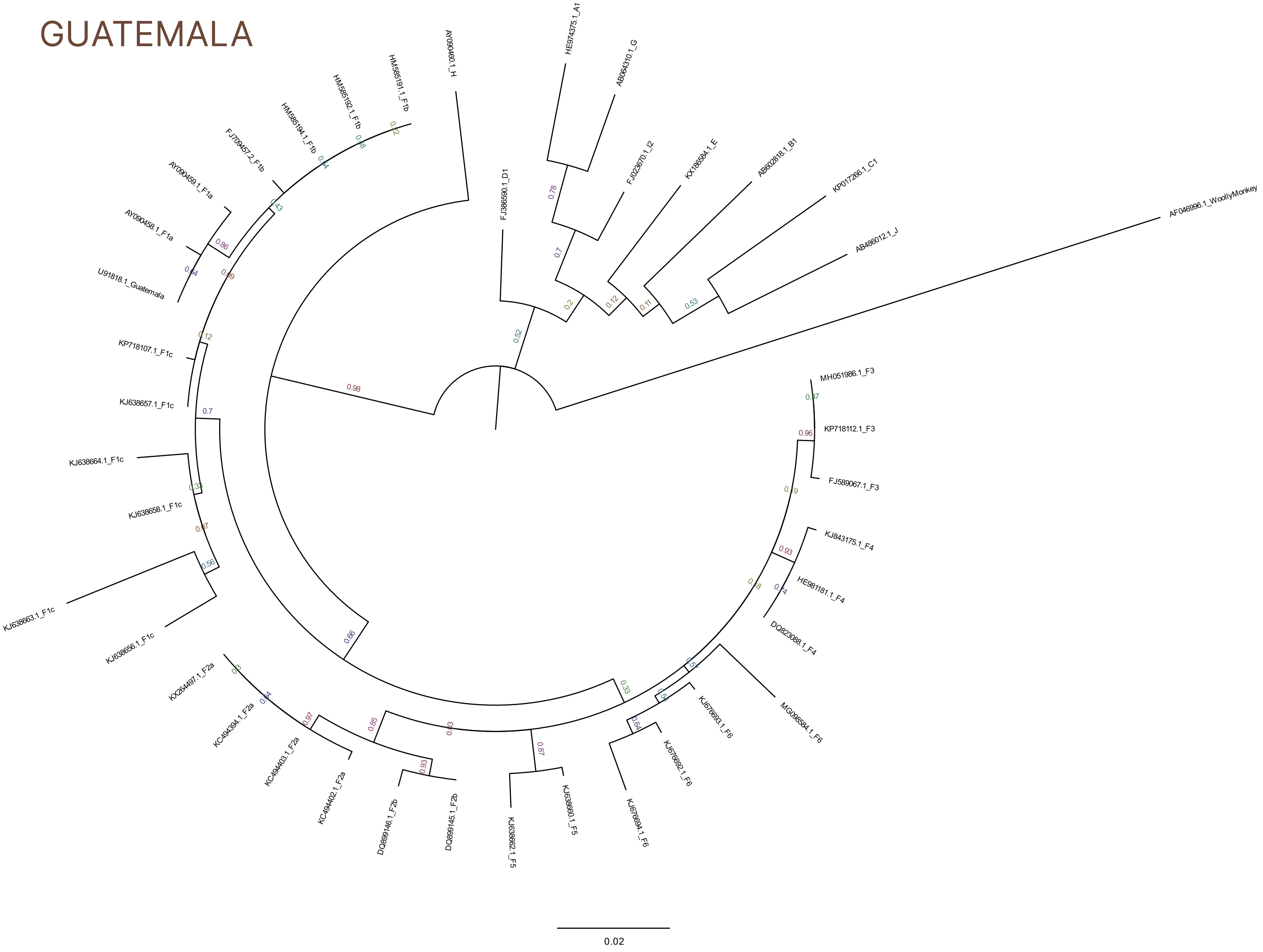

Tree 1. Molecular Phylogenetic analysis by Maximum Likelihood method conducted in MEGA7. The evolutionary history was inferred by using the Maximum Likelihood method based on the Tamura-Nei model with 1000 bootstraps. The tree with the highest log likelihood (-2915.43) is shown. The percentage of trees in which the associated taxa clustered together is shown next to the branches. The tree is drawn to scale, with branch lengths measured in the number of substitutions per site. The analysis involved 41 nucleotide sequences, of which 40 were used as marker sequences to determine the genotype of 1 sequence. All positions containing gaps and missing data were eliminated. There was a total of 667 positions in the final dataset.

ID	GENOTYPE	SUBTYPE	COUNTRY	ALIGNMENT[1]	BASE PAIRS
U91818	F	F1a	Guatemala	157-837	681

HONDURAS

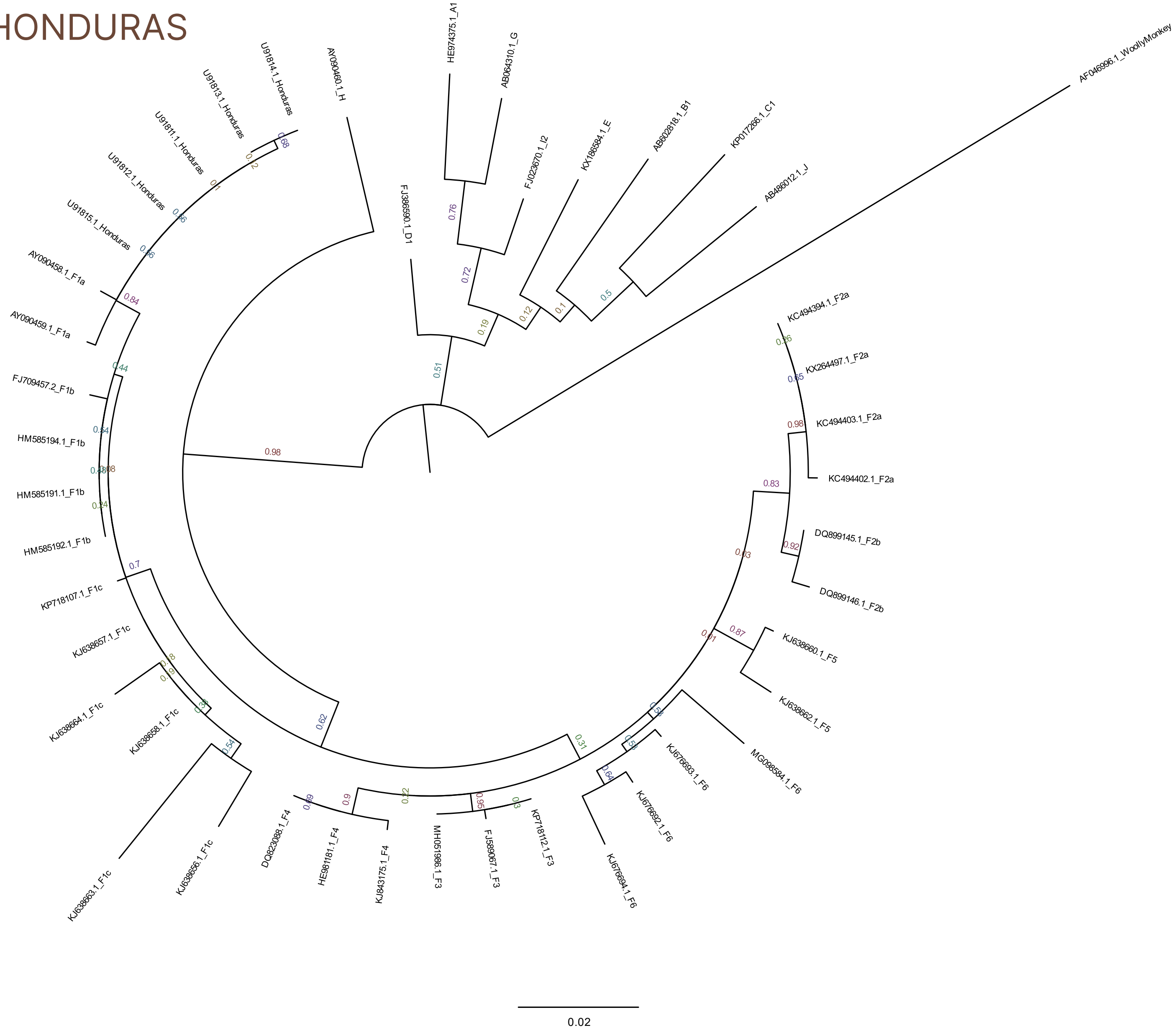

Tree 1. Molecular Phylogenetic analysis by Maximum Likelihood method conducted in MEGA7. The evolutionary history was inferred by using the Maximum Likelihood method based on the Tamura-Nei model with 1000 bootstraps. The tree with the highest log likelihood (-2922.39) is shown. The percentage of trees in which the associated taxa clustered together is shown next to the branches. The tree is drawn to scale, with branch lengths measured in the number of substitutions per site. The analysis involved 45 nucleotide sequences, of which 40 were used as marker sequences to determine the genotype of 5 sequences. All positions containing gaps and missing data were eliminated. There was a total of 667 positions in the final dataset.

ID	GENOTYPE	SUBTYPE	COUNTRY	ALIGNMENT[1]	BASE PAIRS
U91811	F	F1a	Honduras	157-837	681
U91812	F	F1a	Honduras	157-837	681
U91813	F	F1a	Honduras	157-837	681
U91814	F	F1a	Honduras	157-837	681
U91815	F	F1a	Honduras	157-837	681

MARTINIQUE

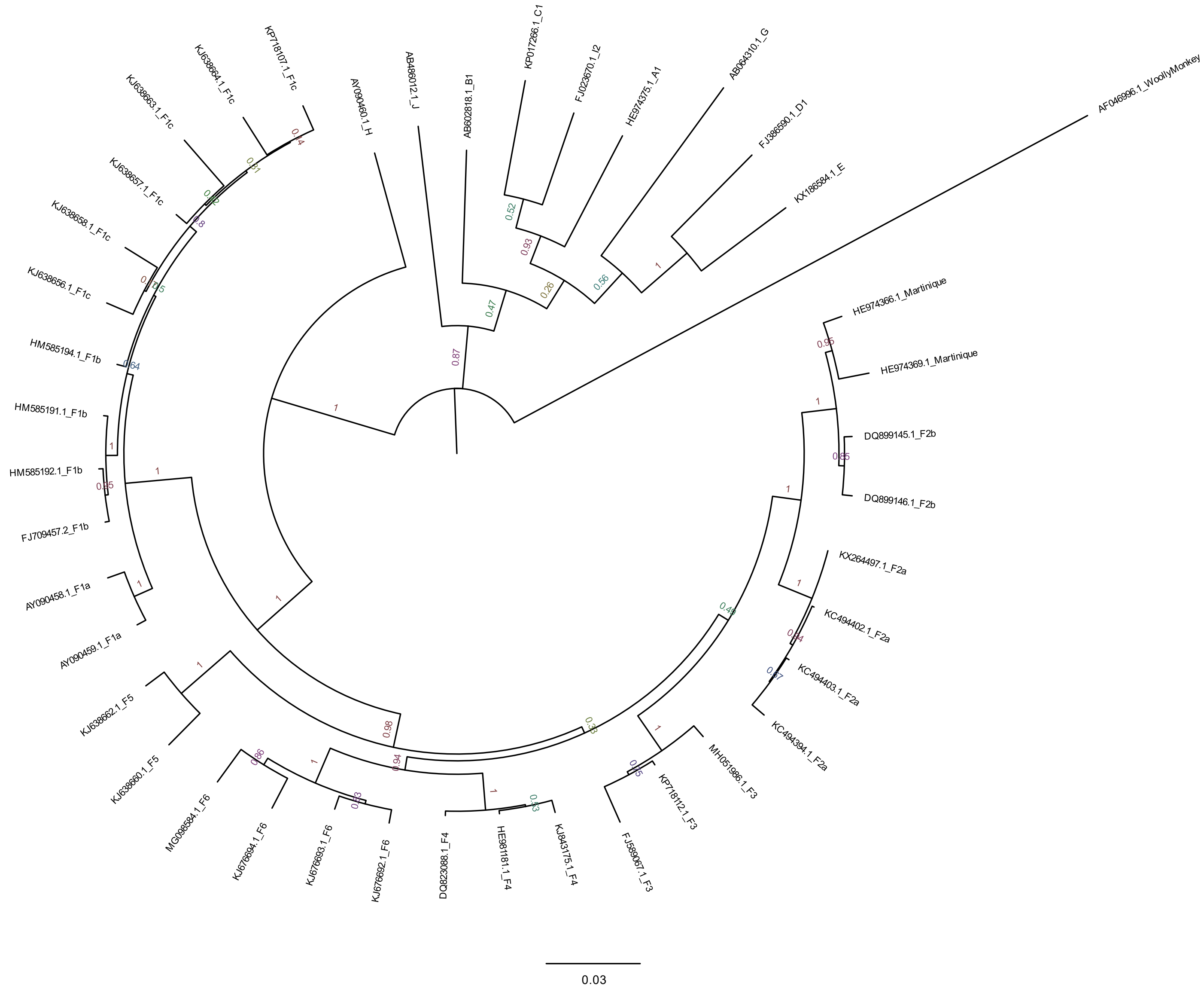

Tree 1. Molecular Phylogenetic analysis by Maximum Likelihood method conducted in MEGA7. The evolutionary history was inferred by using the Maximum Likelihood method based on the Tamura-Nei model with 1000 bootstraps. The tree with the highest log likelihood (-21319.50) is shown. The percentage of trees in which the associated taxa clustered together is shown next to the branches. The tree is drawn to scale, with branch lengths measured in the number of substitutions per site. The analysis involved 42 nucleotide sequences, of which 40 were used as marker sequences to determine the genotype of 2 sequences. All positions containing gaps and missing data were eliminated. There was a total of 3002 positions in the final dataset.

ID	GENOTYPE	SUBTYPE	COUNTRY	ALIGNMENT[1]	BASE PAIRS
HE974366	F	F2b	Martinique	Complete Genome	3215
HE974369	F	F2b	Martinique	Complete Genome	3182

MEXICO

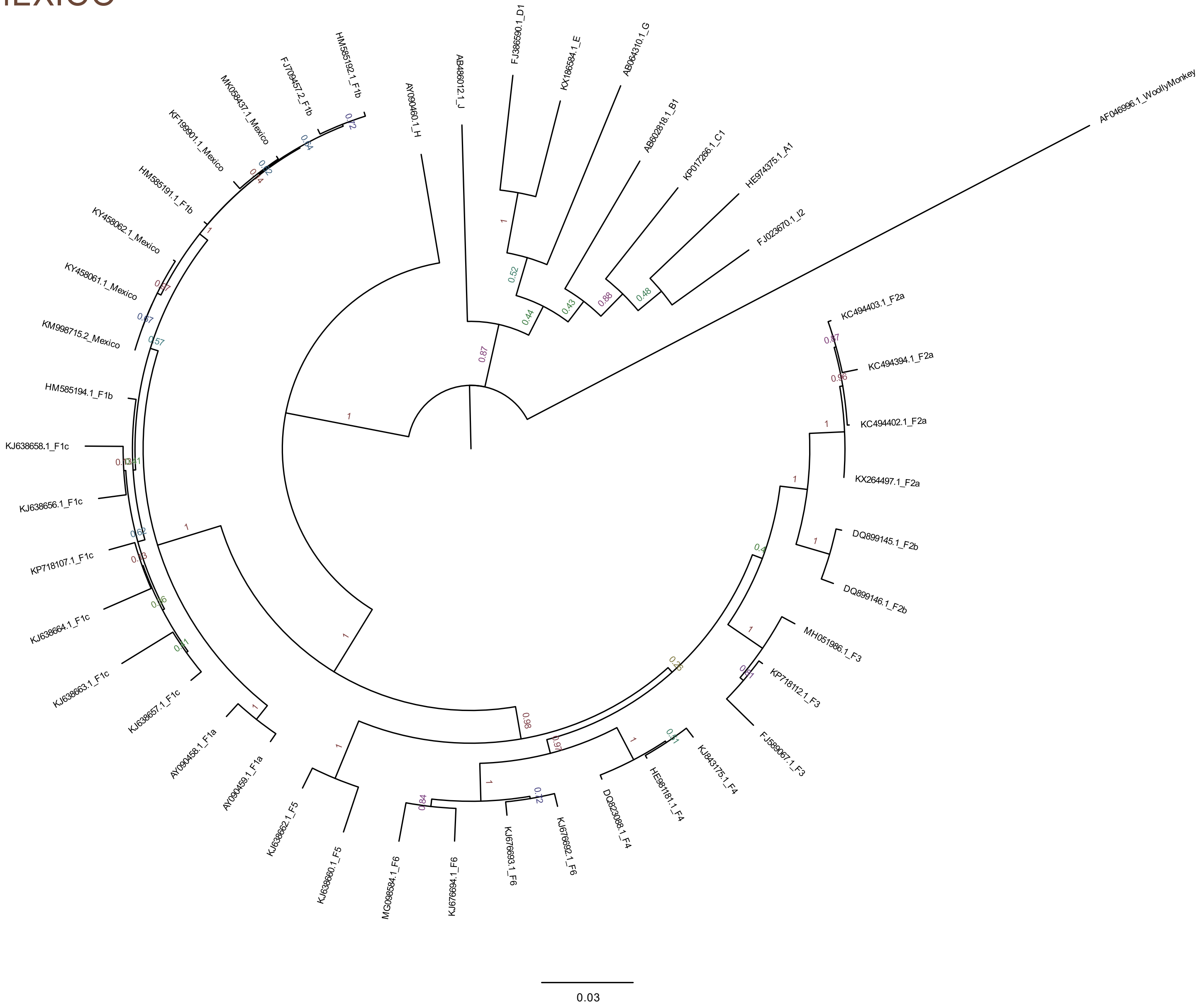

Tree 1. Molecular Phylogenetic analysis by Maximum Likelihood method conducted in MEGA7. The evolutionary history was inferred by using the Maximum Likelihood method based on the Tamura-Nei model with 1000 bootstraps. The tree with the highest log likelihood (-21163.72) is shown. The percentage of trees in which the associated taxa clustered together is shown next to the branches. The tree is drawn to scale, with branch lengths measured in the number of substitutions per site. The analysis involved 45 nucleotide sequences, of which 40 were used as marker sequences to determine the genotype of 5 sequences. All positions containing gaps and missing data were eliminated. There was a total of 3000 positions in the final dataset.

ID	GENOTYPE	SUBTYPE	COUNTRY	ALIGNMENT[1]	BASE PAIRS
KF199901	F	F1b	Mexico	Complete Genome	3215
KM998715	F	F1b	Mexico	Complete Genome	3215
KY458061	F	F1b	Mexico	Complete Genome	3215
KY458062	F	F1b	Mexico	Complete Genome	3215
MK058437	F	F1b	Mexico	Complete Genome	3215

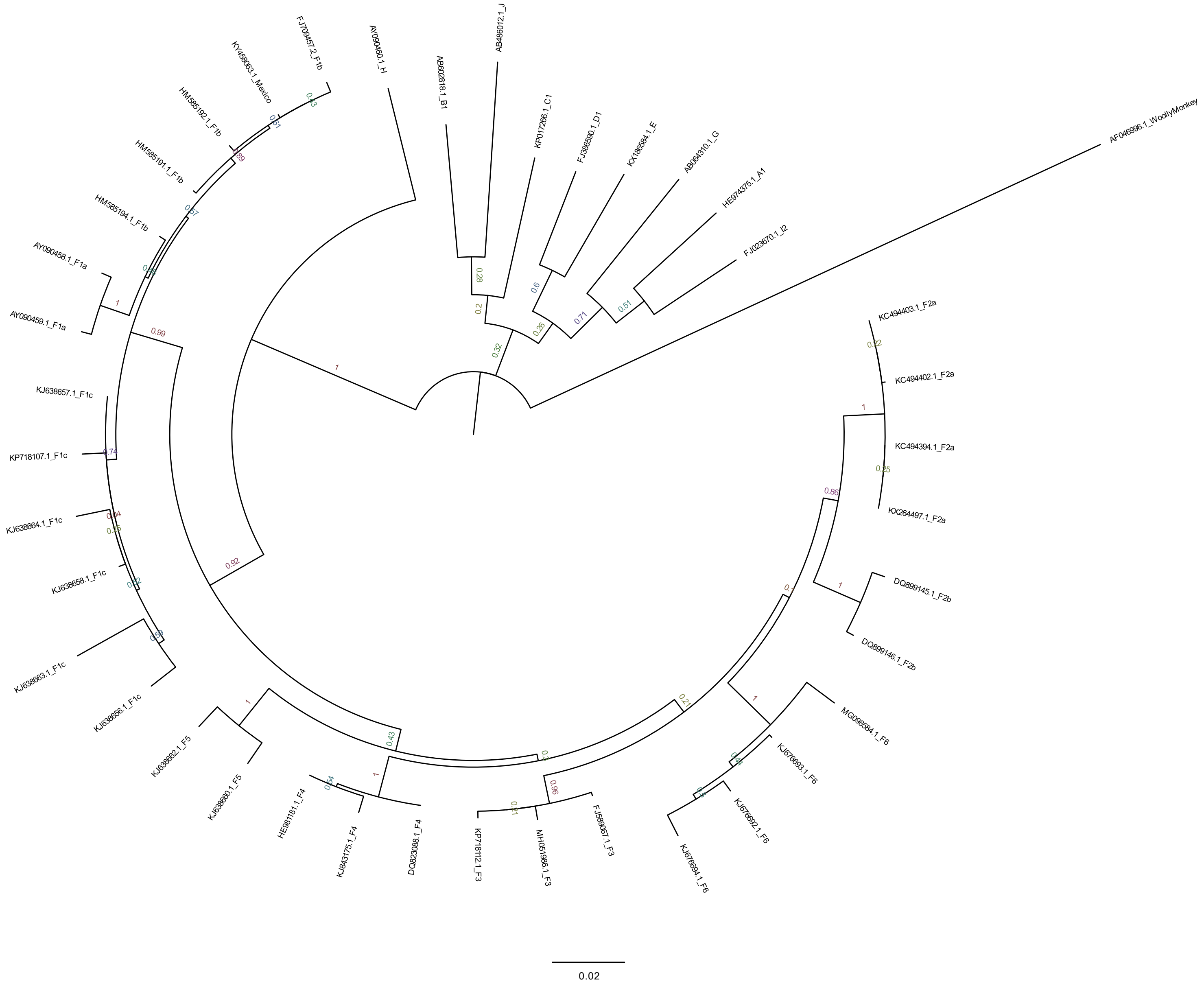

Tree 2. Molecular Phylogenetic analysis by Maximum Likelihood method conducted in MEGA7. The evolutionary history was inferred by using the Maximum Likelihood method based on the Tamura-Nei model with 1000 bootstraps. The tree with the highest log likelihood (-6415.56) is shown. The percentage of trees in which the associated taxa clustered together is shown next to the branches. The tree is drawn to scale, with branch lengths measured in the number of substitutions per site. The analysis involved 41 nucleotide sequences, of which 40 were used as marker sequences to determine the genotype of 1 sequence. All positions containing gaps and missing data were eliminated. There was a total of 1067 positions in the final dataset.

ID	GENOTYPE	SUBTYPE	COUNTRY	ALIGNMENT[1]	BASE PAIRS
KY458063	F	F1b	Mexico	78-1177	1100

NICARAGUA

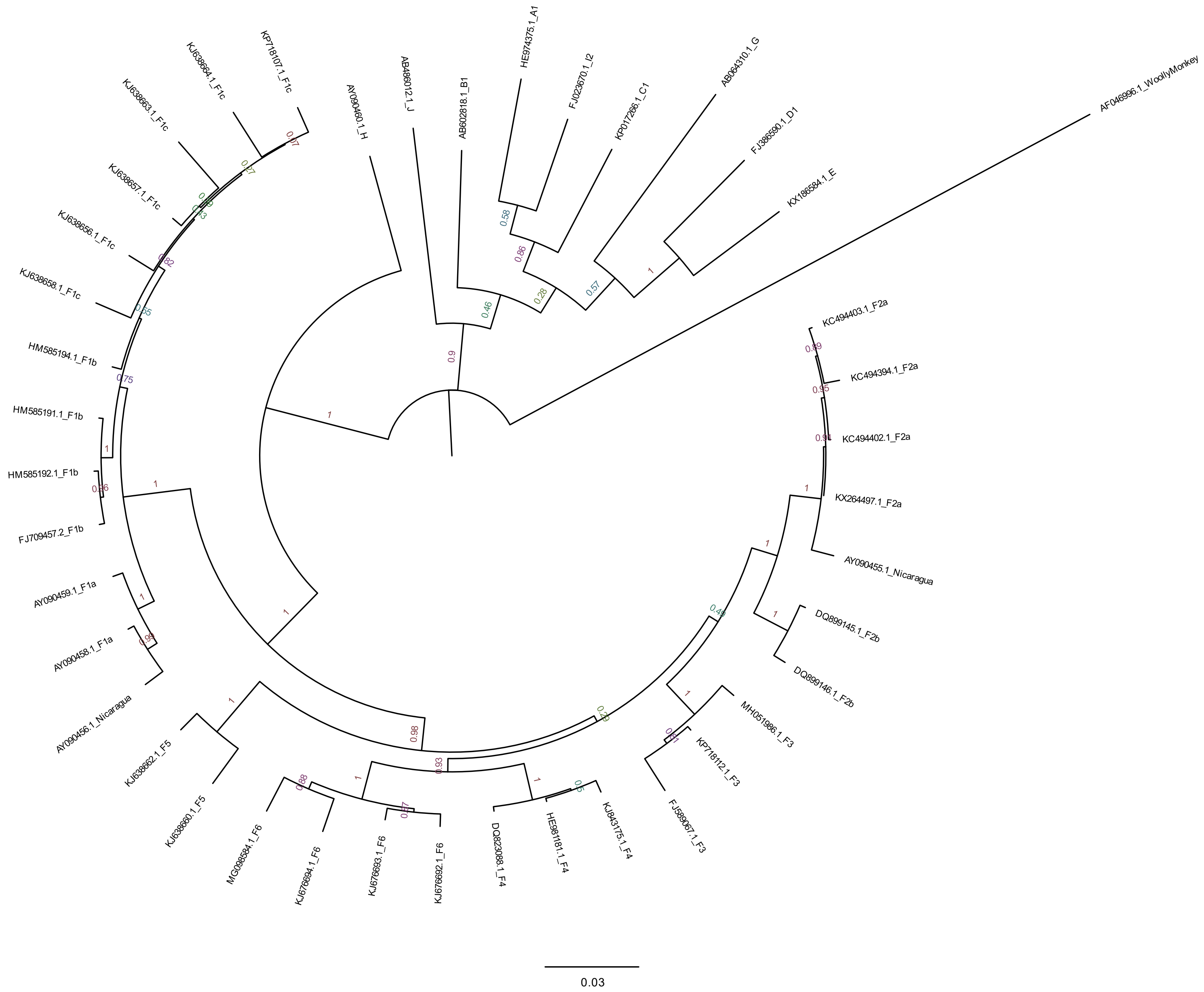

Tree 1. Molecular Phylogenetic analysis by Maximum Likelihood method conducted in MEGA7. The evolutionary history was inferred by using the Maximum Likelihood method based on the Tamura-Nei model with 1000 bootstraps. The tree with the highest log likelihood (-21173.14) is shown. The percentage of trees in which the associated taxa clustered together is shown next to the branches. The tree is drawn to scale, with branch lengths measured in the number of substitutions per site. The analysis involved 42 nucleotide sequences, of which 40 were used as marker sequences to determine the genotype of 2 sequences. All positions containing gaps and missing data were eliminated. There was a total of 2986 positions in the final dataset.

ID	GENOTYPE	SUBTYPE	COUNTRY	ALIGNMENT[1]	BASE PAIRS
AY090456	F	F1a	Nicaragua	Complete Genome	3215
AY090455	F	F2a	Nicaragua	Complete Genome	3215

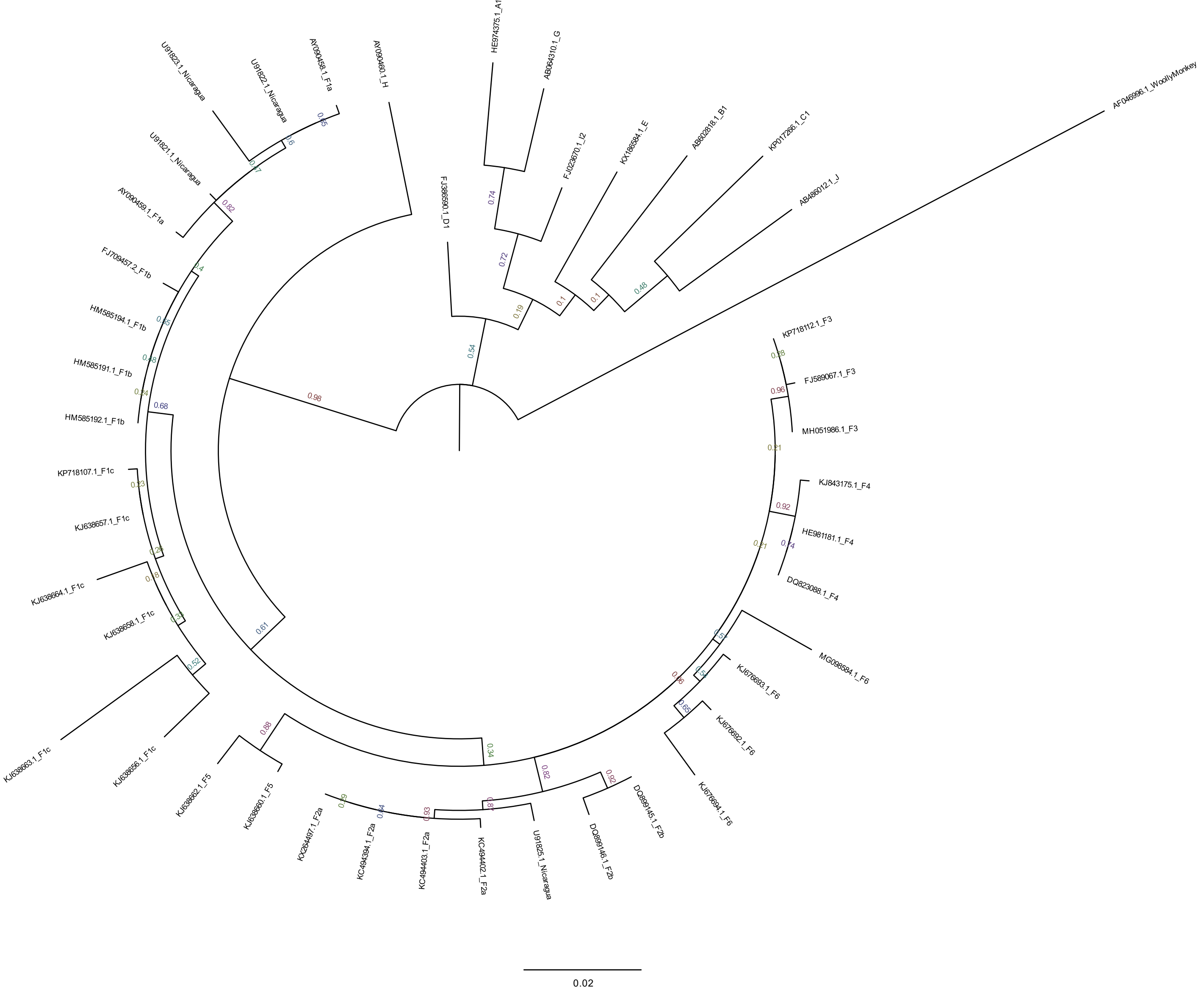

Tree 2. Molecular Phylogenetic analysis by Maximum Likelihood method conducted in MEGA7. The evolutionary history was inferred by using the Maximum Likelihood method based on the Tamura-Nei model with 1000 bootstraps. The tree with the highest log likelihood (-2990.98) is shown. The percentage of trees in which the associated taxa clustered together is shown next to the branches. The tree is drawn to scale, with branch lengths measured in the number of substitutions per site. The analysis involved 44 nucleotide sequences, of which 40 were used as marker sequences to determine the genotype of 4 sequences. All positions containing gaps and missing data were eliminated. There was a total of 667 positions in the final dataset.

ID	GENOTYPE	SUBTYPE	COUNTRY	ALIGNMENT[1]	BASE PAIRS
U91821	F	F1a	Nicaragua	157-837	681
U91822	F	F1a	Nicaragua	157-837	681
U91823	F	F1a	Nicaragua	157-837	681
U91825	F	F2a	Nicaragua	157-837	681

PANAMA

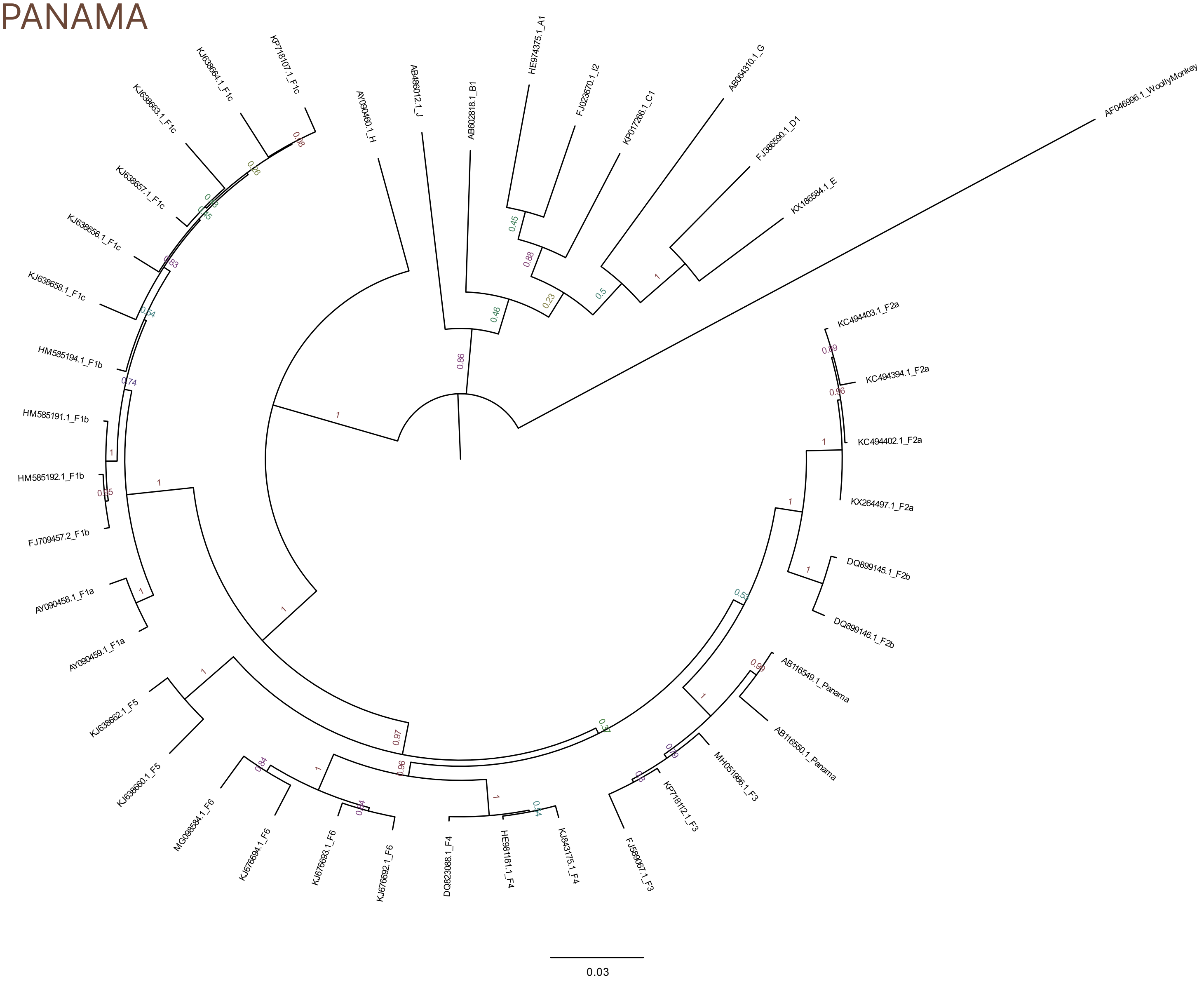

Tree 1. Molecular Phylogenetic analysis by Maximum Likelihood method conducted in MEGA7. The evolutionary history was inferred by using the Maximum Likelihood method based on the Tamura-Nei model with 1000 bootstraps. The tree with the highest log likelihood (-21681.31) is shown. The percentage of trees in which the associated taxa clustered together is shown next to the branches. The tree is drawn to scale, with branch lengths measured in the number of substitutions per site. The analysis involved 42 nucleotide sequences, of which 40 were used as marker sequences to determine the genotype of 2 sequences. All positions containing gaps and missing data were eliminated. There was a total of 3035 positions in the final dataset.

ID	GENOTYPE	SUBTYPE	COUNTRY	ALIGNMENT[1]	BASE PAIRS
AB116549	F	F3	Panama	Complete Genome	3215
AB116550	F	F3	Panama	Complete Genome	3215

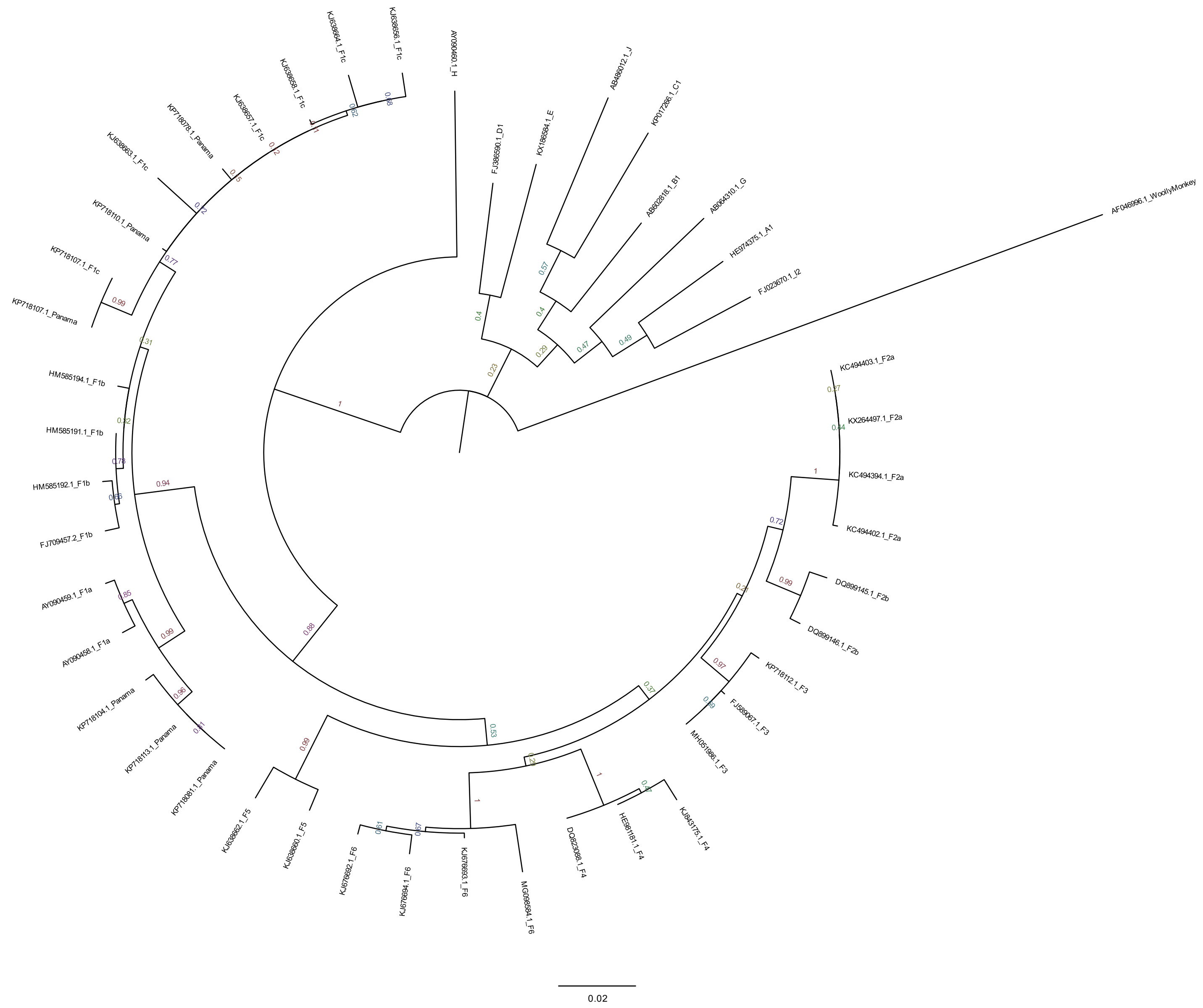

Tree 2. Molecular Phylogenetic analysis by Maximum Likelihood method conducted in MEGA7. The evolutionary history was inferred by using the Maximum Likelihood method based on the Tamura-Nei model with 1000 bootstraps. The tree with the highest log likelihood (-4900.05) is shown. The percentage of trees in which the associated taxa clustered together is shown next to the branches. The tree is drawn to scale, with branch lengths measured in the number of substitutions per site. The analysis involved 46 nucleotide sequences, of which 40 were used as marker sequences to determine the genotype of 6 sequences. All positions containing gaps and missing data were eliminated. There was a total of 816 positions in the final dataset.

ID	GENOTYPE	SUBTYPE	COUNTRY	ALIGNMENT[1]	BASE PAIRS
KP718078	F	F1c	Panama	249-1106	858
KP718081	F	F1a	Panama	261-1107	847
KP718104	F	F1a	Panama	Complete Genome	3215
KP718107	F	F1c	Panama	Complete Genome	3215
KP718110	F	F1c	Panama	Complete Genome	3215
KP718113	F	F1a	Panama	Complete Genome	3215

PERU

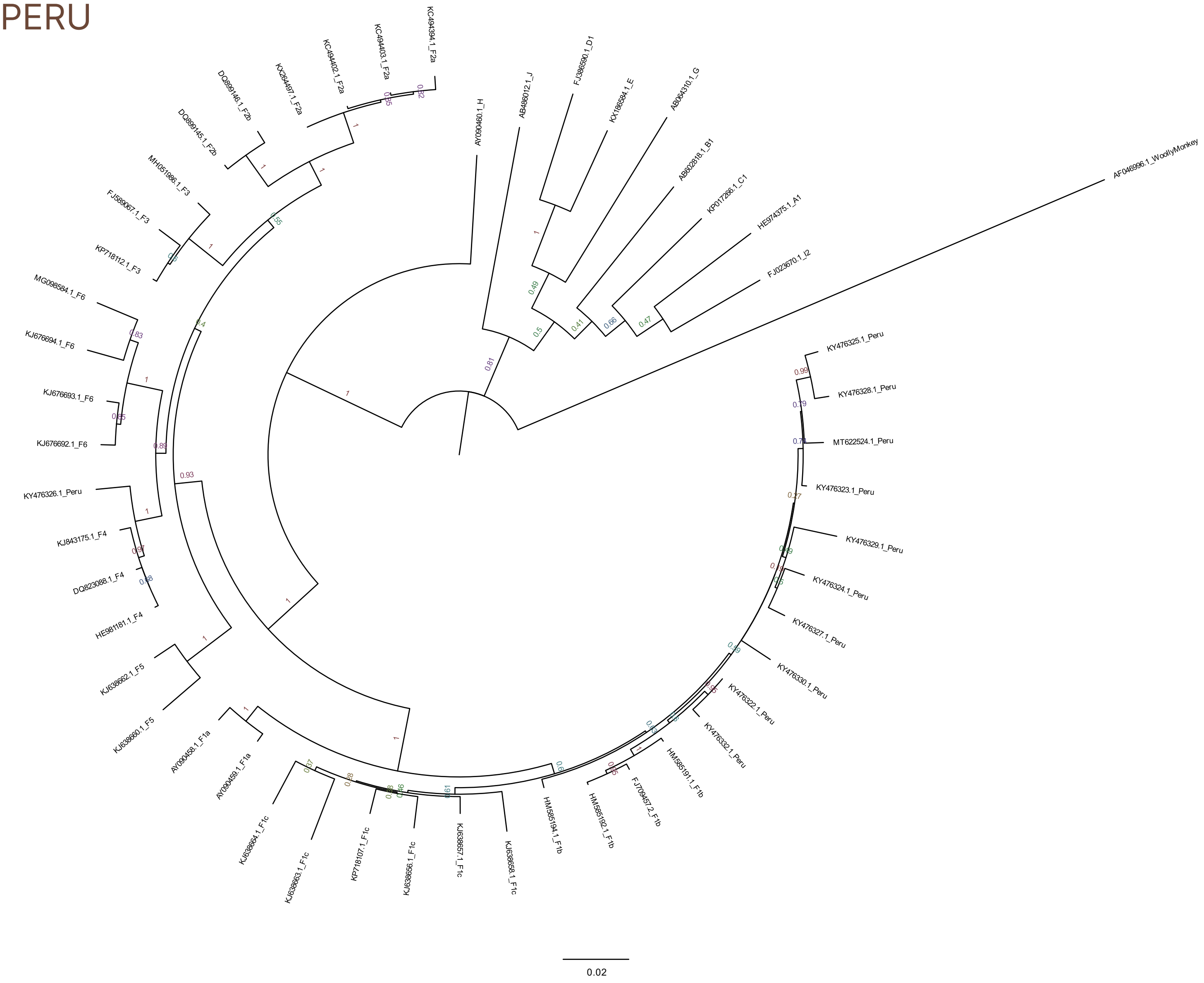

Tree 1. Molecular Phylogenetic analysis by Maximum Likelihood method conducted in MEGA7. The evolutionary history was inferred by using the Maximum Likelihood method based on the Tamura-Nei model with 1000 bootstraps. The tree with the highest log likelihood (-19759.00) is shown. The percentage of trees in which the associated taxa clustered together is shown next to the branches. The tree is drawn to scale, with branch lengths measured in the number of substitutions per site. The analysis involved 51 nucleotide sequences, of which 40 were used as marker sequences to determine the genotype of 11 sequences. All positions containing gaps and missing data were eliminated. There was a total of 2728 positions in the final dataset.

ID	GENOTYPE	SUBTYPE	COUNTRY	ALIGNMENT[1]	BASE PAIRS
MT622524	F	F1b	Peru	Complete Genome	3215
KY476322	F	F1b	Peru	Complete Genome	3215
KY476323	F	F1b	Peru	Complete Genome	3215
KY476324	F	F1b	Peru	Complete Genome	3215
KY476325	F	F1b	Peru	Complete Genome	3215
KY476327	F	F1b	Peru	Complete Genome	3215
KY476328	F	F1b	Peru	Complete Genome	3215
KY476329	F	F1b	Peru	Complete Genome	3215

KY476330	F	F1b	Peru	Complete Genome	3215
KY476332	F	F1b	Peru	Complete Genome	3215
KY476326	F	F4	Peru	Complete Genome	3215

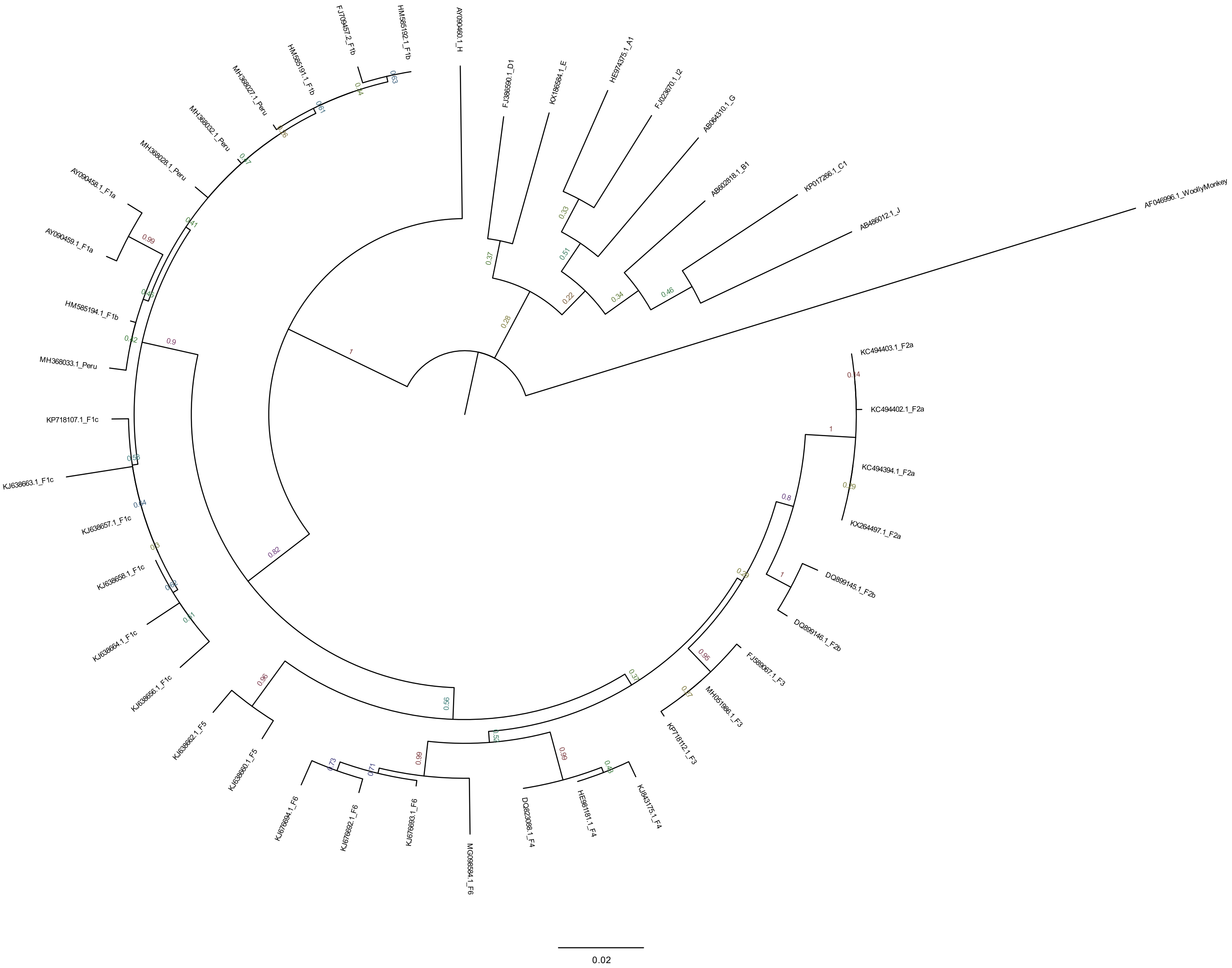

Tree 2. Molecular Phylogenetic analysis by Maximum Likelihood method conducted in MEGA7. The evolutionary history was inferred by using the Maximum Likelihood method based on the Tamura-Nei model with 1000 bootstraps. The tree with the highest log likelihood (-4328.85) is shown. The percentage of trees in which the associated taxa clustered together is shown next to the branches. The tree is drawn to scale, with branch lengths measured in the number of substitutions per site. The analysis involved 44 nucleotide sequences, of which 40 were used as marker sequences to determine the genotype of 4 sequences. All positions containing gaps and missing data were eliminated. There was a total of 770 positions in the final dataset.

ID	GENOTYPE	SUBTYPE	COUNTRY	ALIGNMENT[1]	BASE PAIRS
MH368027	F	F1b	Peru	207-1187	981
MH368028	F	F1b	Peru	207-1187	981
MH368032	F	F1b	Peru	207-1004	798
MH368033	F	F1b	Peru	207-1187	981

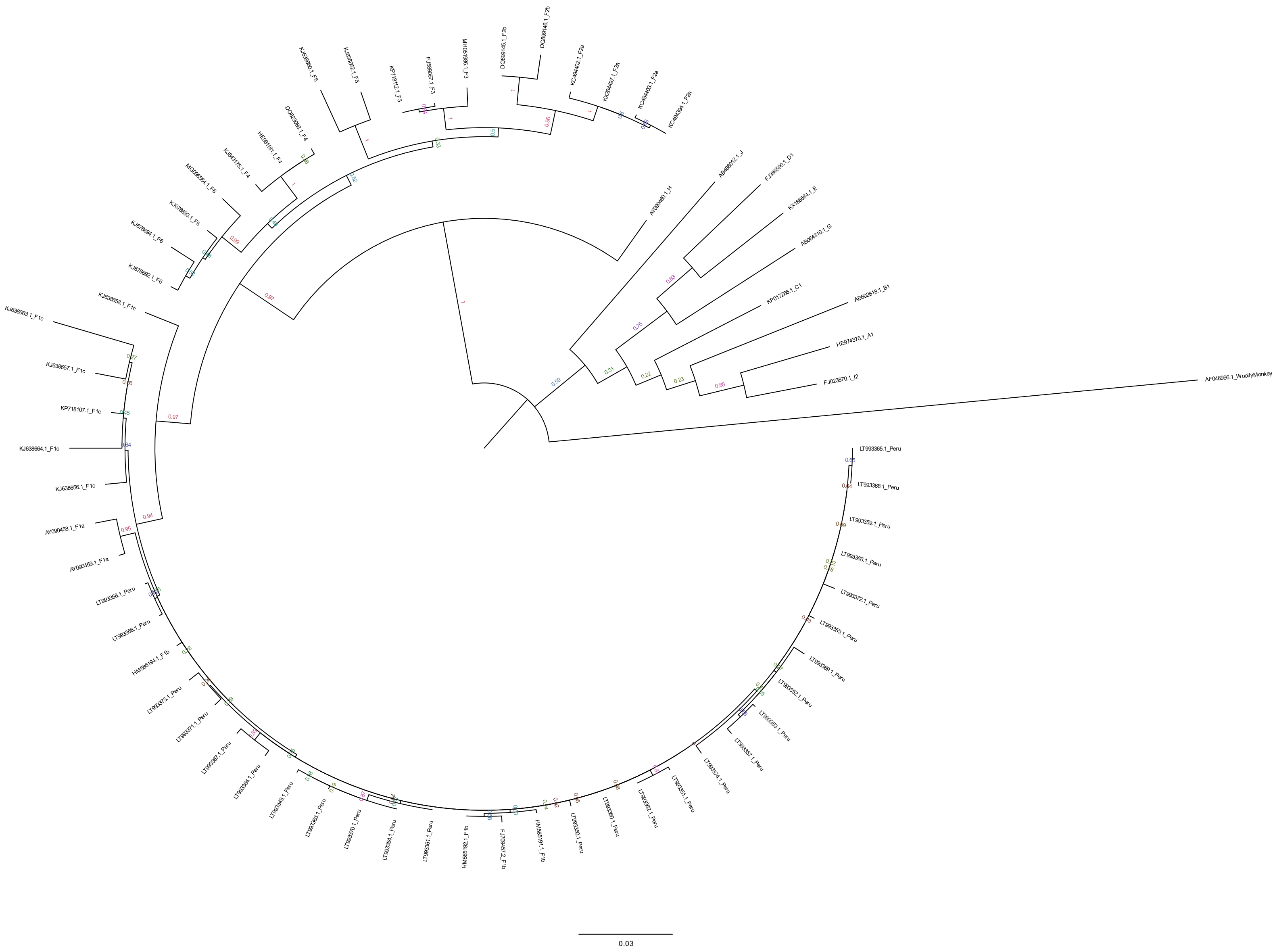

Tree 3. Molecular Phylogenetic analysis by Maximum Likelihood method conducted in MEGA7. The evolutionary history was inferred by using the Maximum Likelihood method based on the Tamura-Nei model with 1000 bootstraps. The tree with the highest log likelihood (-6985.97) is shown. The percentage of trees in which the associated taxa clustered together is shown next to the branches. The tree is drawn to scale, with branch lengths measured in the number of substitutions per site. The analysis involved 66 nucleotide sequences, of which 40 were used as marker sequences to determine the genotype of 26 sequences. All positions containing gaps and missing data were eliminated. There was a total of 1027 positions in the final dataset.

ID	GENOTYPE	SUBTYPE	COUNTRY	ALIGNMENT[1]	BASE PAIRS
LT993349	F	F1b	Peru	1-3182	1099
LT993350	F	F1b	Peru	1-3182	1099
LT993351	F	F1b	Peru	1-3182	1099
LT993352	F	F1b	Peru	1-3182	1099
LT993353	F	F1b	Peru	1-3182	1099

LT993354	F	F1b	Peru	1-3182	1147
LT993355	F	F1b	Peru	1-3182	1099
LT993356	F	F1b	Peru	1-3182	1099
LT993357	F	F1b	Peru	1-3182	1099
LT993358	F	F1b	Peru	1-3182	1099
LT993359	F	F1b	Peru	1-3182	1099
LT993360	F	F1b	Peru	1-3182	1099
LT993361	F	F1b	Peru	1-3182	1099
LT993362	F	F1b	Peru	1-3182	1099
LT993363	F	F1b	Peru	1-3182	1099
LT993364	F	F1b	Peru	1-3182	1099
LT993365	F	F1b	Peru	1-3182	1099
LT993366	F	F1b	Peru	1-3182	1099
LT993367	F	F1b	Peru	1-3182	1099
LT993368	F	F1b	Peru	1-3182	1099
LT993369	F	F1b	Peru	1-3182	1099
LT993370	F	F1b	Peru	1-3182	1099
LT993371	F	F1b	Peru	1-3182	1099
LT993372	F	F1b	Peru	1-3182	1099
LT993373	F	F1b	Peru	1-3182	1099
LT993374	F	F1b	Peru	1-3182	1099

USA

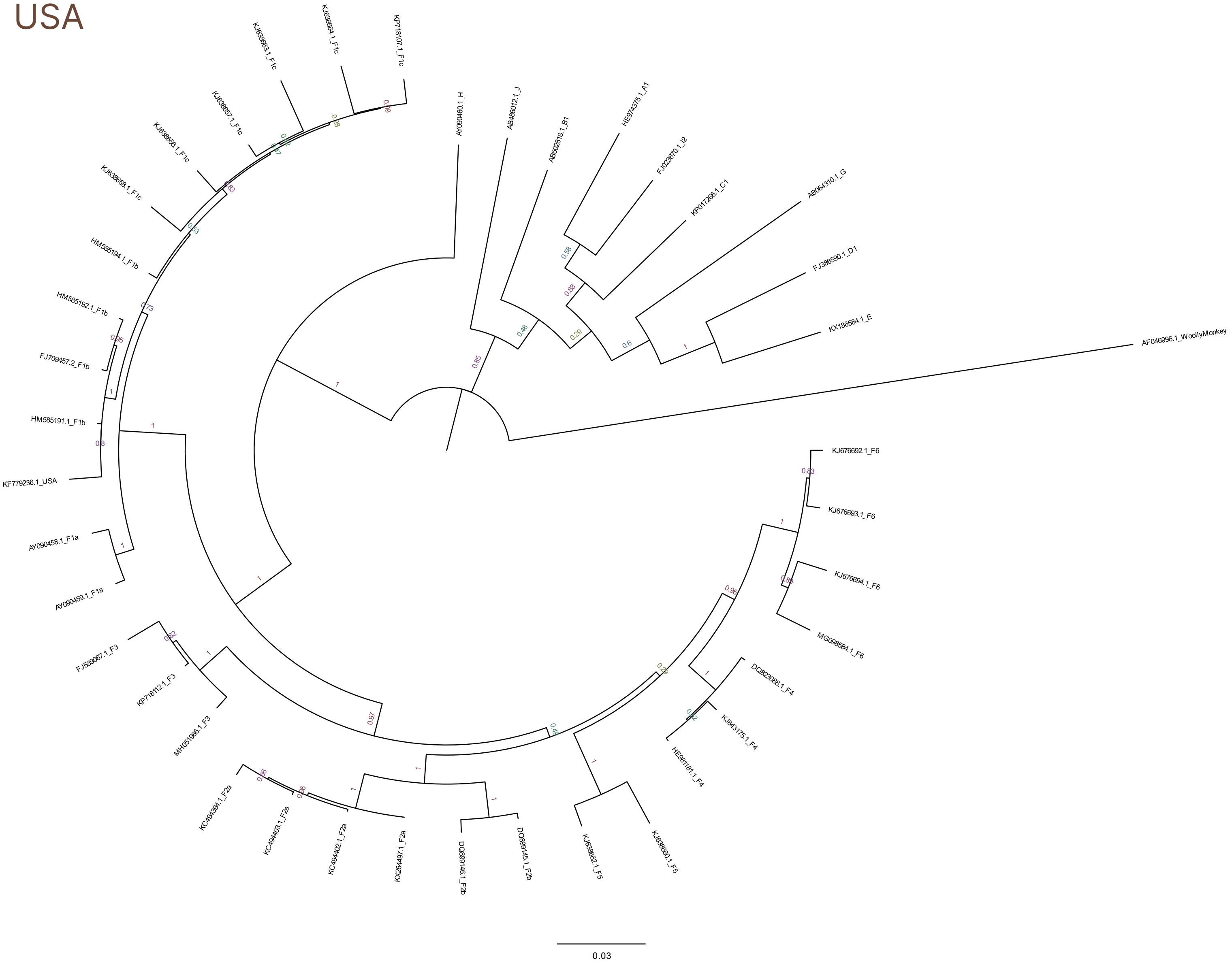

Tree 1. Molecular Phylogenetic analysis by Maximum Likelihood method conducted in MEGA7. The evolutionary history was inferred by using the Maximum Likelihood method based on the Tamura-Nei model with 1000 bootstraps. The tree with the highest log likelihood (-21190.28) is shown. The percentage of trees in which the associated taxa clustered together is shown next to the branches. The tree is drawn to scale, with branch lengths measured in the number of substitutions per site. The analysis involved 41 nucleotide sequences, of which 40 were used as marker sequences to determine the genotype of 1 sequence. All positions containing gaps and missing data were eliminated. There was a total of 2948 positions in the final dataset.

ID	GENOTYPE	SUBTYPE	COUNTRY	ALIGNMENT[1]	BASE PAIRS
KF779236	F	F1b	USA	Complete Genome	3165

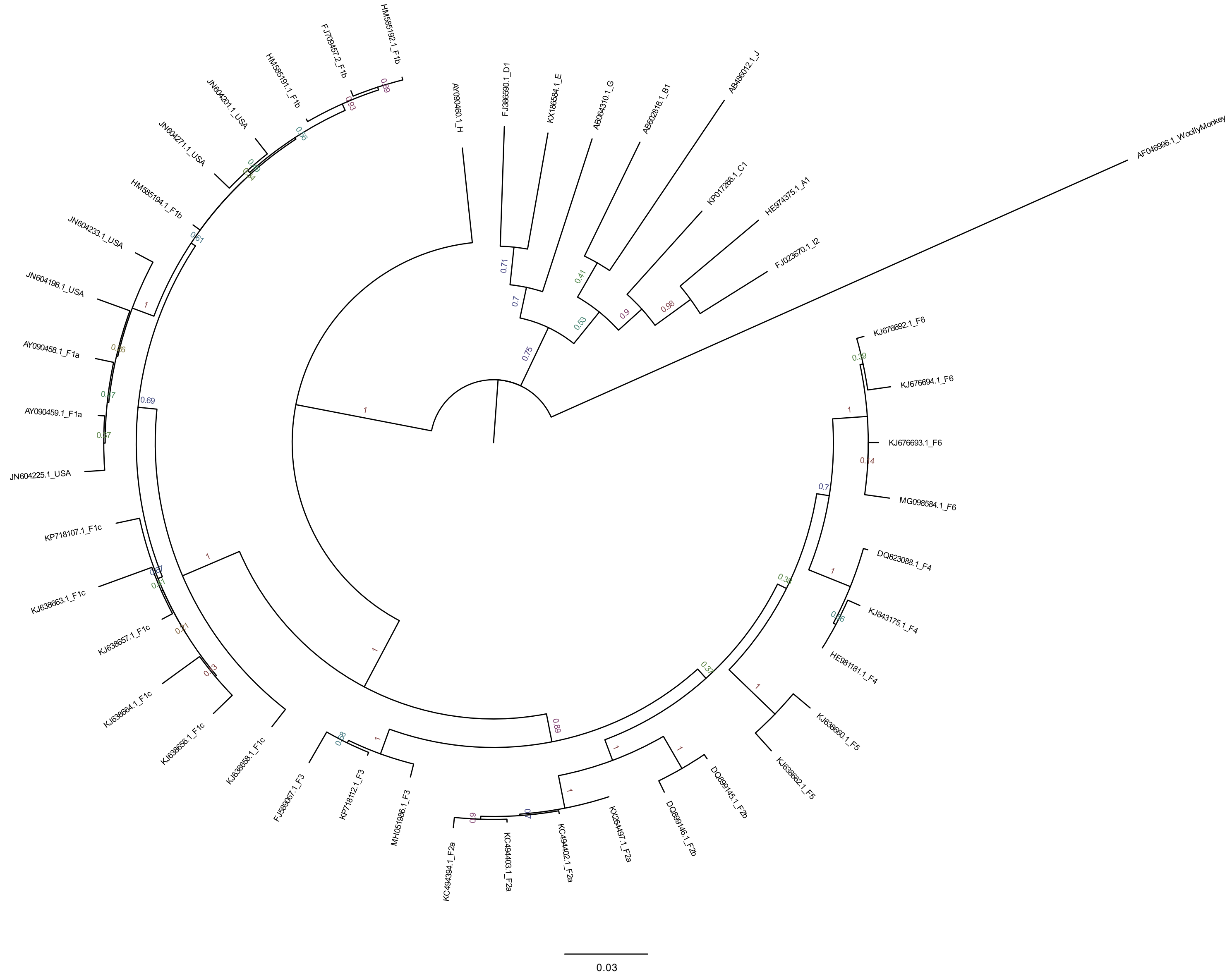

Tree 2. Molecular Phylogenetic analysis by Maximum Likelihood method conducted in MEGA7. The evolutionary history was inferred by using the Maximum Likelihood method based on the Tamura-Nei model with 1000 bootstraps. The tree with the highest log likelihood (-14037.95) is shown. The percentage of trees in which the associated taxa clustered together is shown next to the branches. The tree is drawn to scale, with branch lengths measured in the number of substitutions per site. The analysis involved 45 nucleotide sequences, of which 40 were used as marker sequences to determine the genotype of 5 sequences. All positions containing gaps and missing data were eliminated. There was a total of 1891 positions in the final dataset.

ID	GENOTYPE	SUBTYPE	COUNTRY	ALIGNMENT[1]	BASE PAIRS
JN604198	F	F1a	USA	1-3182	2015
JN604225	F	F1a	USA	1-3182	2015
JN604233	F	F1a	USA	1-3182	2015
JN604201	F	F1b	USA	1-3182	2015
JN604271	F	F1b	USA	1-3182	2015

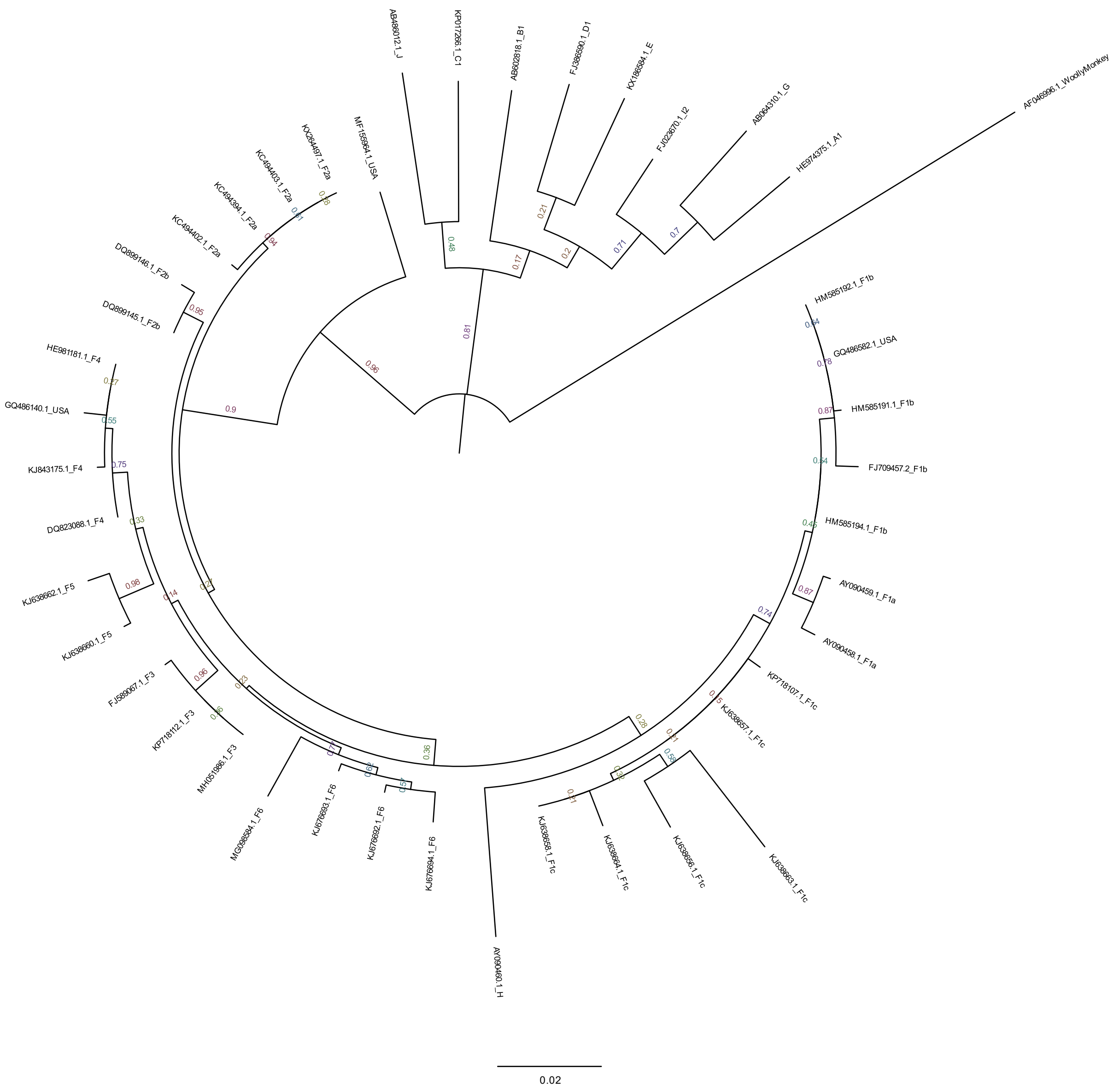

Tree 3. Molecular Phylogenetic analysis by Maximum Likelihood method conducted in MEGA7. The evolutionary history was inferred by using the Maximum Likelihood method based on the Tamura-Nei model with 1000 bootstraps. The tree with the highest log likelihood (-3326.33) is shown. The percentage of trees in which the associated taxa clustered together is shown next to the branches. The tree is drawn to scale, with branch lengths measured in the number of substitutions per site. The analysis involved 43 nucleotide sequences, of which 40 were used as marker sequences to determine the genotype of 3 sequences. All positions containing gaps and missing data were eliminated. There was a total of 695 positions in the final dataset.

ID	GENOTYPE	SUBTYPE	COUNTRY	ALIGNMENT[1]	BASE PAIRS
MF155964	F	NA	USA	3-1163	900
GQ486582	F	F1b	USA	132-1163	1032
GQ486140	F	F4	USA	132-1163	1032

VENEZUELA

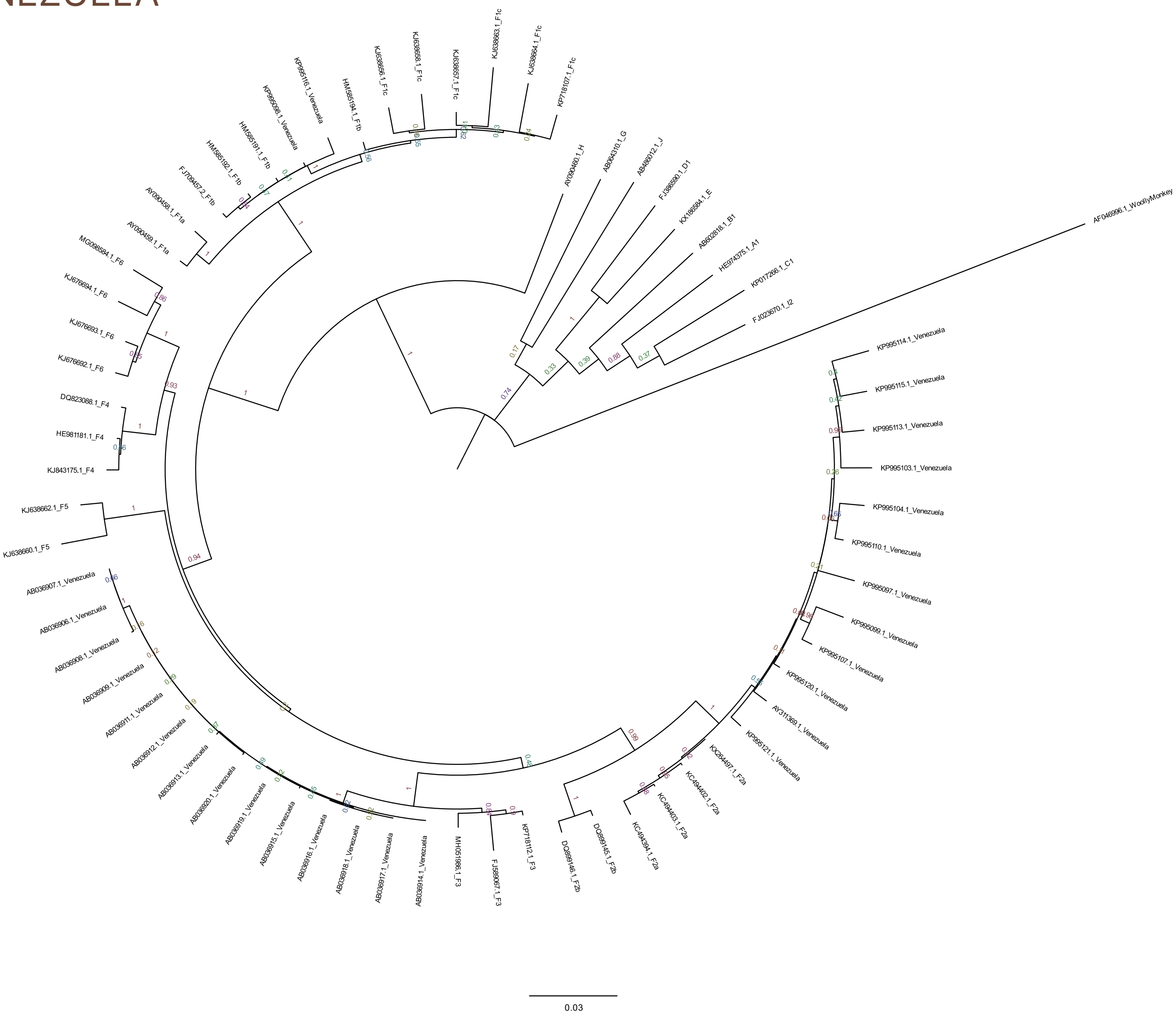

Tree 1. Molecular Phylogenetic analysis by Maximum Likelihood method conducted in MEGA7. The evolutionary history was inferred by using the Maximum Likelihood method based on the Tamura-Nei model with 1000 bootstraps. The tree with the highest log likelihood (-23100.33) is shown. The percentage of trees in which the associated taxa clustered together is shown next to the branches. The tree is drawn to scale, with branch lengths measured in the number of substitutions per site. The analysis involved 68 nucleotide sequences, of which 40 were used as marker sequences to determine the genotype of 28 sequences. All positions containing gaps and missing data were eliminated. There was a total of 2948 positions in the final dataset.

ID	GENOTYPE	SUBTYPE	COUNTRY	ALIGNMENT[1]	BASE PAIRS
KP995098	F	F1b	Venezuela	Complete Genome	3215
KP995116	F	F1b	Venezuela	Complete Genome	3215
AY311369	F	F2a	Venezuela	Complete Genome	3215
KP995097	F	F2a	Venezuela	Complete Genome	3215
KP995099	F	F2a	Venezuela	Complete Genome	3219
KP995103	F	F2a	Venezuela	Complete Genome	3214
KP995104	F	F2a	Venezuela	Complete Genome	3215
KP995107	F	F2a	Venezuela	Complete Genome	3215

KP995110	F	F2a	Venezuela	Complete Genome	3215
KP995113	F	F2a	Venezuela	Complete Genome	3215
KP995114	F	F2a	Venezuela	Complete Genome	3215
KP995115	F	F2a	Venezuela	Complete Genome	3215
KP995120	F	F2a	Venezuela	Complete Genome	3215
KP995121	F	F2a	Venezuela	Complete Genome	3214
AB036906	F	F3	Venezuela	Complete Genome	3206
AB036907	F	F3	Venezuela	Complete Genome	3206
AB036908	F	F3	Venezuela	Complete Genome	3206
AB036909	F	F3	Venezuela	Complete Genome	3215
AB036911	F	F3	Venezuela	Complete Genome	3215
AB036912	F	F3	Venezuela	Complete Genome	3215
AB036913	F	F3	Venezuela	Complete Genome	3215
AB036914	F	F3	Venezuela	Complete Genome	3215
AB036915	F	F3	Venezuela	Complete Genome	3215
AB036916	F	F3	Venezuela	Complete Genome	3215
AB036917	F	F3	Venezuela	Complete Genome	3215
AB036918	F	F3	Venezuela	Complete Genome	3129
AB036919	F	F3	Venezuela	Complete Genome	3215
AB036920	F	F3	Venezuela	Complete Genome	3215

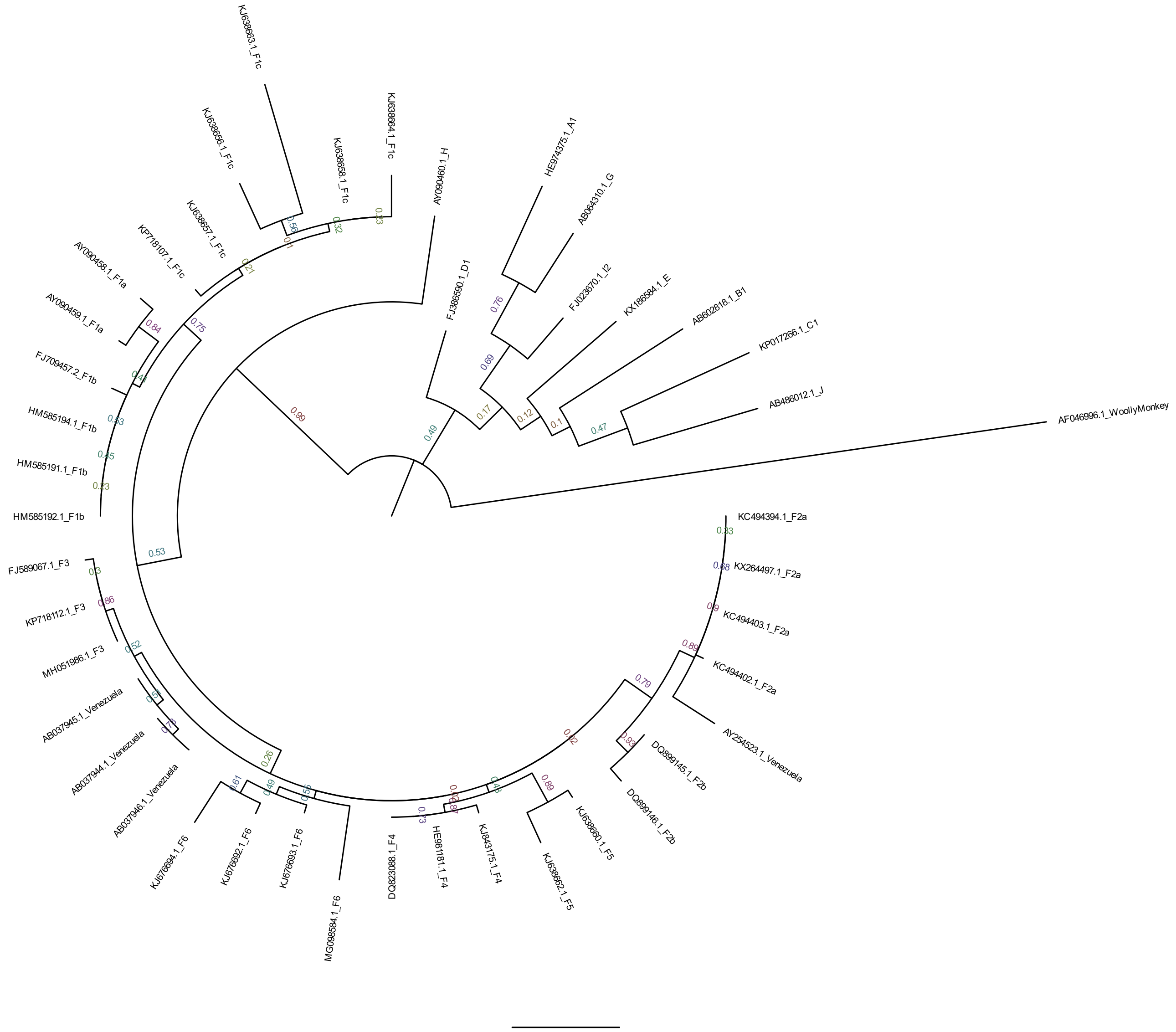

Tree 2. Molecular Phylogenetic analysis by Maximum Likelihood method conducted in MEGA7. The evolutionary history was inferred by using the Maximum Likelihood method based on the Tamura-Nei model with 1000 bootstraps. The tree with the highest log likelihood (-2902.35) is shown. The percentage of trees in which the associated taxa clustered together is shown next to the branches. The tree is drawn to scale, with branch lengths measured in the number of substitutions per site. The analysis involved 44 nucleotide sequences, of which 40 were used as marker sequences to determine the genotype of 4 sequences. All positions containing gaps and missing data were eliminated. There was a total of 654 positions in the final dataset.

ID	GENOTYPE	SUBTYPE	COUNTRY	ALIGNMENT[1]	BASE PAIRS
AB037944	F	F3	Venezuela	157-837	681
AB037945	F	F3	Venezuela	157-837	681
AB037946	F	F3	Venezuela	157-837	681
AY254523	F	F2a	Venezuela	152-824	673

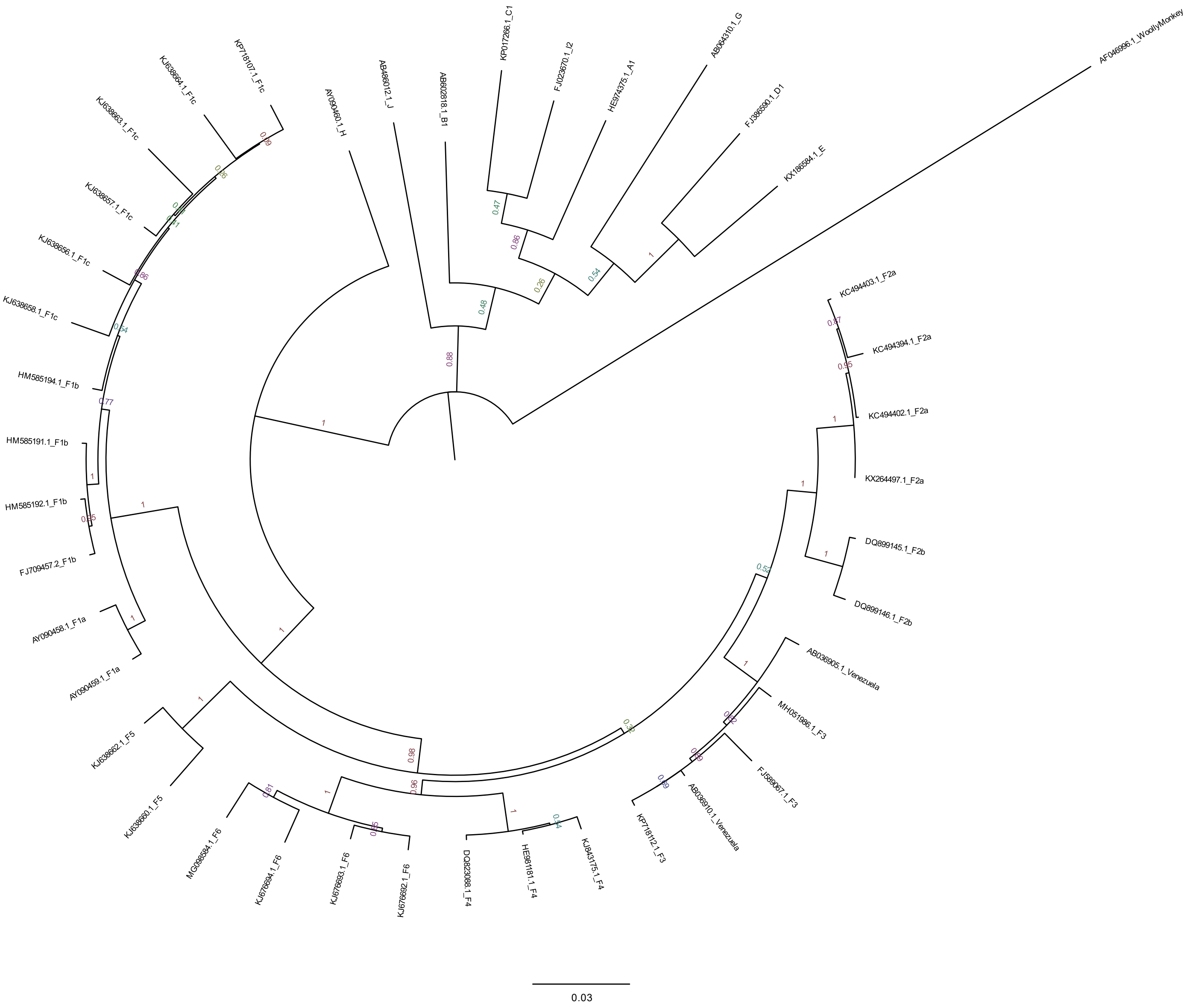

Tree 3. Molecular Phylogenetic analysis by Maximum Likelihood method conducted in MEGA7. The evolutionary history was inferred by using the Maximum Likelihood method based on the Tamura-Nei model with 1000 bootstraps. The tree with the highest log likelihood (-21457.94) is shown. The percentage of trees in which the associated taxa clustered together is shown next to the branches. The tree is drawn to scale, with branch lengths measured in the number of substitutions per site. The analysis involved 42 nucleotide sequences, of which 40 were used as marker sequences to determine the genotype of 2 sequences. All positions containing gaps and missing data were eliminated. There was a total of 3034 positions in the final dataset.

ID	GENOTYPE	SUBTYPE	COUNTRY	ALIGNMENT[1]	BASE PAIRS
AB036905	F	F3	Venezuela	Complete Genome	3215
AB036910	F	F3	Venezuela	Complete Genome	3215

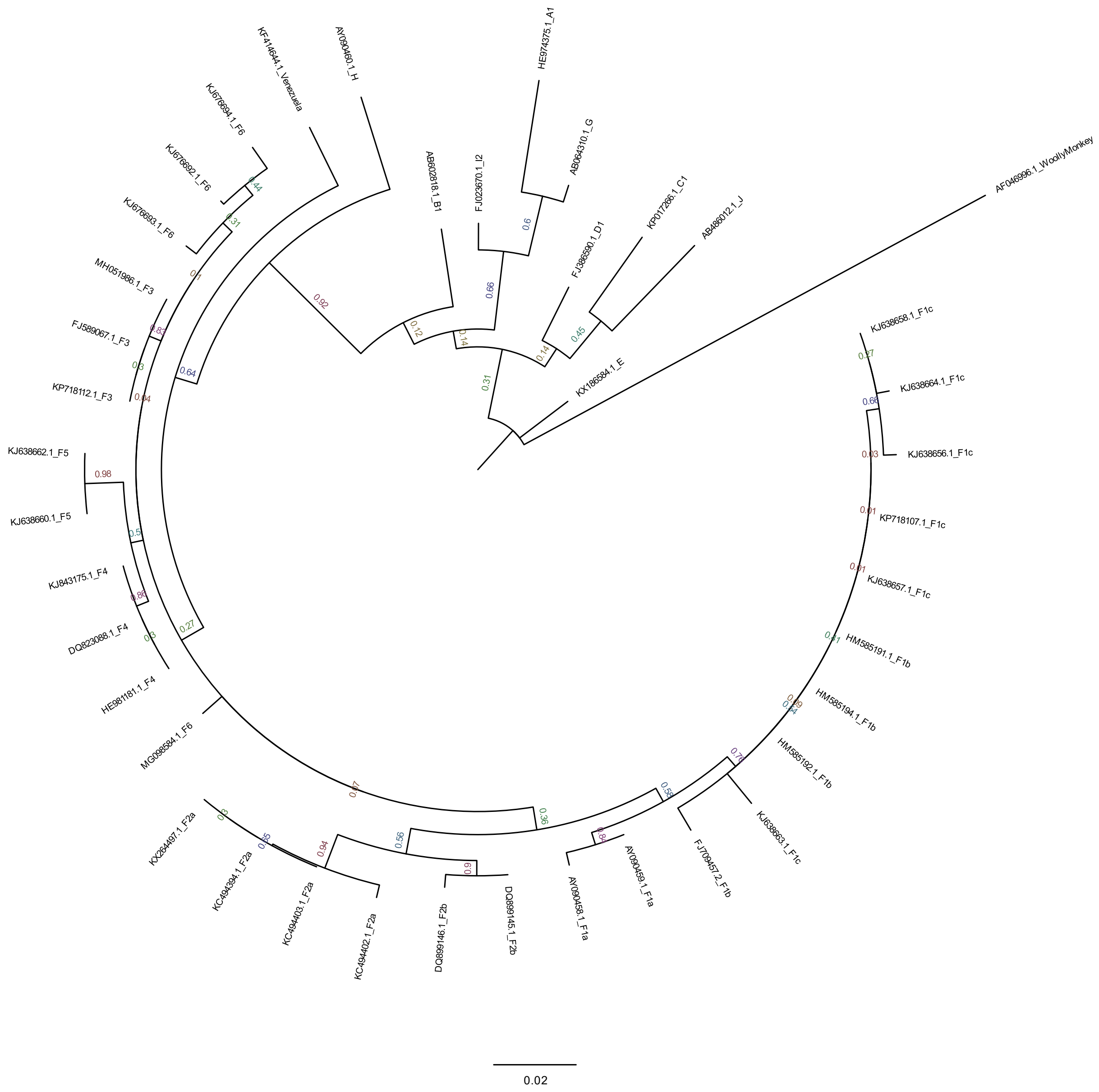

Tree 4. Molecular Phylogenetic analysis by Maximum Likelihood method conducted in MEGA7. The evolutionary history was inferred by using the Maximum Likelihood method based on the Tamura-Nei model with 1000 bootstraps. The tree with the highest log likelihood (-21457.94) is shown. The percentage of trees in which the associated taxa clustered together is shown next to the branches. The tree is drawn to scale, with branch lengths measured in the number of substitutions per site. The analysis involved 41 nucleotide sequences, of which 40 were used as marker sequences to determine the genotype of 1 sequence. All positions containing gaps and missing data were eliminated. There was a total of 3034 positions in the final dataset. Additional trees were constructed to narrow down the subtype of the sequence.

ID	GENOTYPE	SUBTYPE	COUNTRY	ALIGNMENT[1]	BASE PAIRS
KF414644	F	F6	Venezuela	424-753	330
