# Supplementary material for: The Molecular Epidemiology of Hepatitis B Virus and Its Resistance-Associated Mutations in the Polymerase Gene in the Americas
Source: Microorganisms. 2025 Aug 16;13(8):1913. doi: 10.3390/microorganisms13081913 (PMC12388563; doi:10.3390/microorganisms13081913)
Supplement: Supplementary file 1 [file microorganisms-13-01913-s001.zip › Figure S3 - Report Broad Genotyping.pdf]

# Phylogenetic Analysis of Sequences

*GENOTYPE NOT PUBLICLY REPORTED*

# ARGENTINA

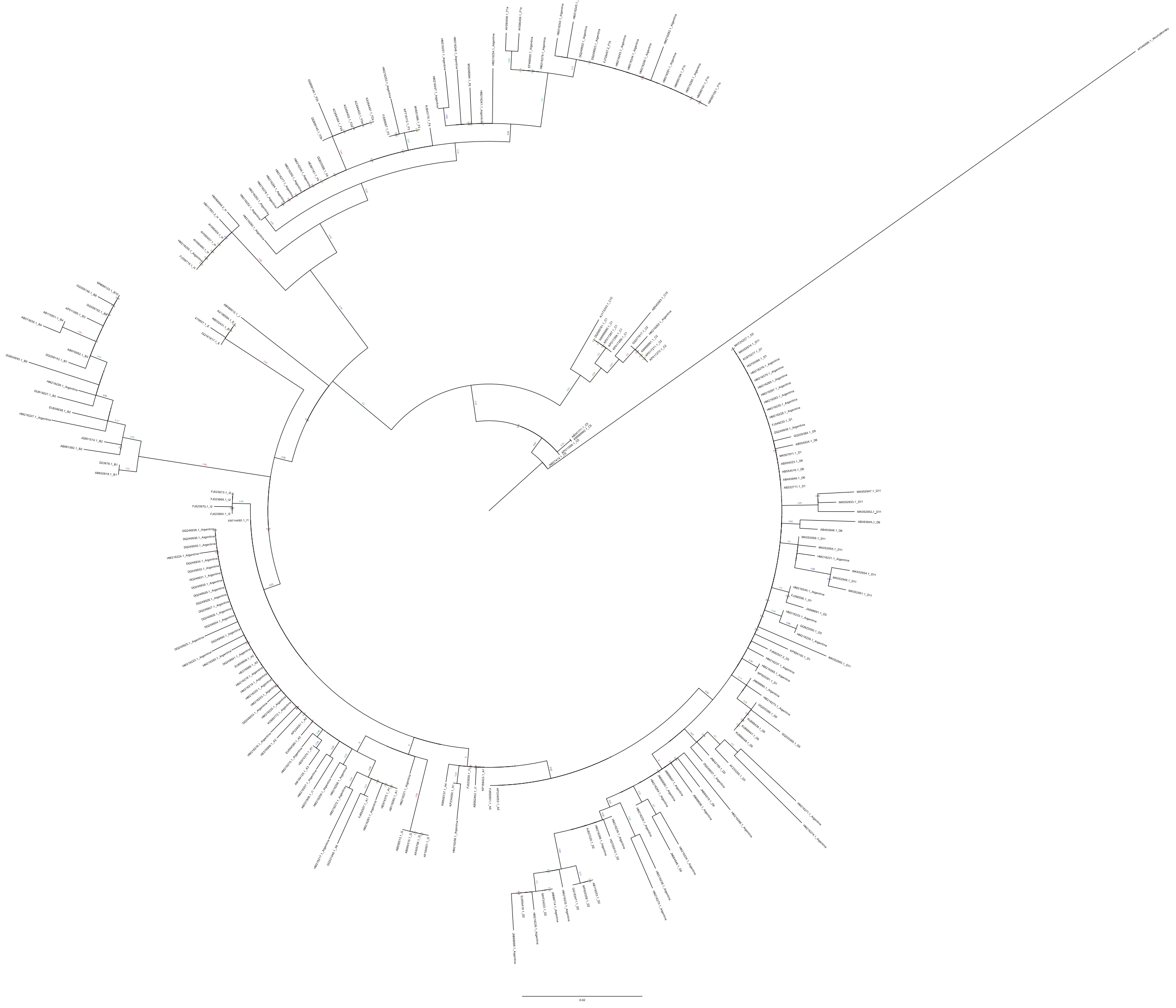

Tree 1. The evolutionary history was inferred by using the Maximum Likelihood method and Tamura-Nei model. The tree with the highest log likelihood (-2311.22) is shown. The percentage of replicate trees in which the associated taxa clustered together in the bootstrap test (1000 replicates) are shown next to the branches. Initial tree(s) for the heuristic search were obtained automatically by applying Neighbor-Join and BioNJ algorithms to a matrix of pairwise distances estimated using the Tamura-Nei model, and then selecting the topology with superior log likelihood value. A discrete Gamma distribution was used to model evolutionary rate differences among sites (5 categories (+G, parameter = 0.2702)). The tree is drawn to scale, with branch lengths measured in the number of substitutions per site. The analysis involved 233 nucleotide sequences, of which 134 were used as marker sequences to determine the genotype of 99 sequences. All positions containing gaps and missing data were eliminated (complete deletion option). There was a total of 406 positions in the final dataset. Evolutionary analyses were conducted in MEGA7.

# CANADA

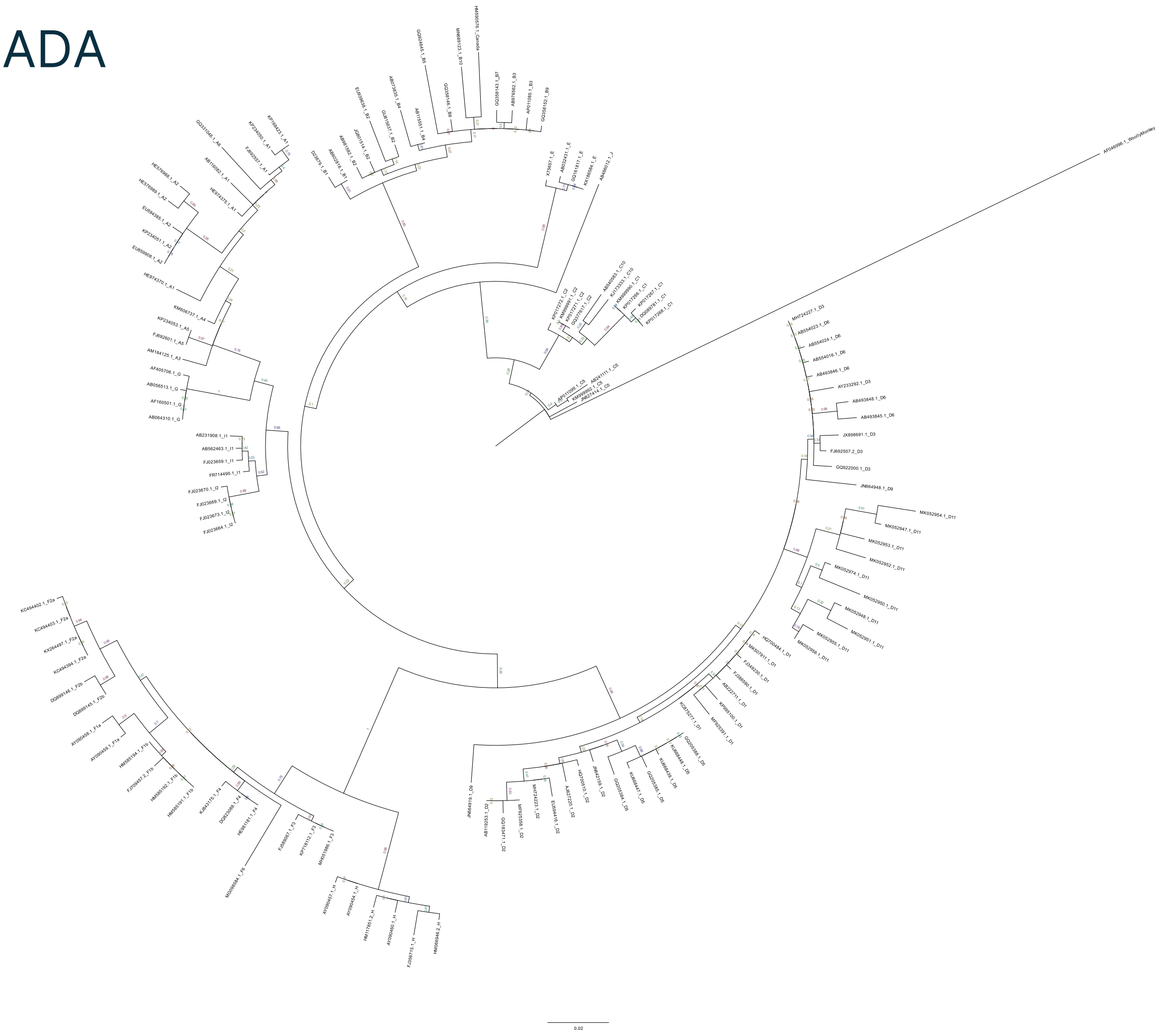

Tree 1. The evolutionary history was inferred by using the Maximum Likelihood method and Tamura-Nei model. The tree with the highest log likelihood (-4404.93) is shown. The percentage of replicate trees in which the associated taxa clustered together in the bootstrap test (1000 replicates) are shown next to the branches. Initial tree(s) for the heuristic search were obtained automatically by applying Neighbor-Join and BioNJ algorithms to a matrix of pairwise distances estimated using the Tamura-Nei model, and then selecting the topology with superior log likelihood value. A discrete Gamma distribution was used to model evolutionary rate differences among sites (5 categories (+G, parameter = 0.2204)). The tree is drawn to scale, with branch lengths measured in the number of substitutions per site. The analysis involved 135 nucleotide sequences, of which 134 were used as marker sequences to determine the genotype of 1 sequence. All positions containing gaps and missing data were eliminated. There was a total of 656 positions in the final dataset. Evolutionary analyses were conducted in MEGA7.

# COLOMBIA

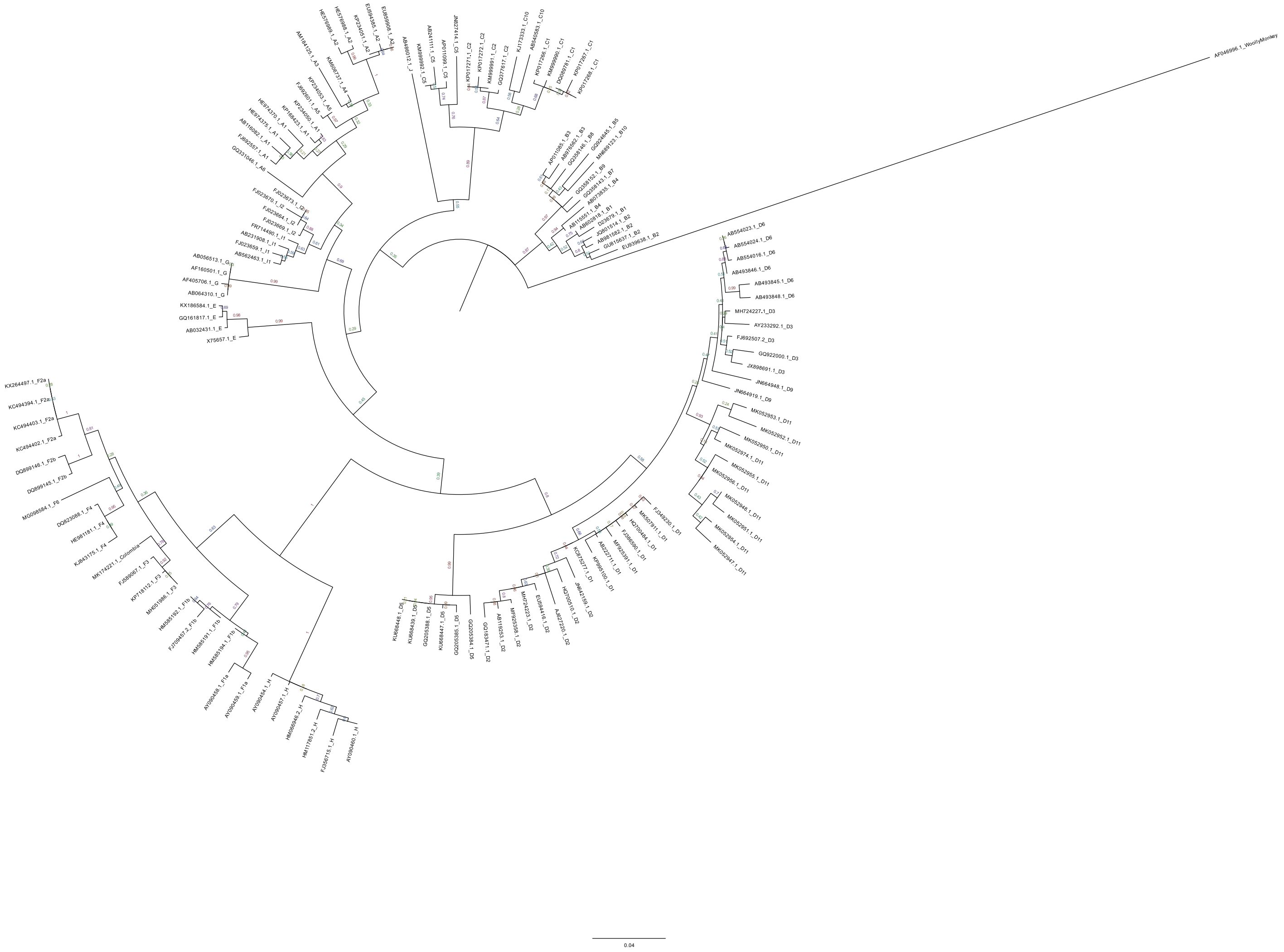

Tree 1. The evolutionary history was inferred by using the Maximum Likelihood method and Tamura-Nei model. The tree with the highest log likelihood (-6399.42) is shown. The percentage of replicate trees in which the associated taxa clustered together in the bootstrap test (1000 replicates) are shown next to the branches. Initial tree(s) for the heuristic search were obtained automatically by applying Neighbor-Join and BioNJ algorithms to a matrix of pairwise distances estimated using the Tamura-Nei model, and then selecting the topology with superior log likelihood value. A discrete Gamma distribution was used to model evolutionary rate differences among sites (5 categories (+G, parameter = 0.2081)). The tree is drawn to scale, with branch lengths measured in the number of substitutions per site. The analysis involved 135 nucleotide sequences, of which 134 were used as marker sequences to determine the genotype of 1 sequence. All positions containing gaps and missing data were eliminated. There was a total of 715 positions in the final dataset. Evolutionary analyses were conducted in MEGA7.

# DOMINICAN REPUBLIC

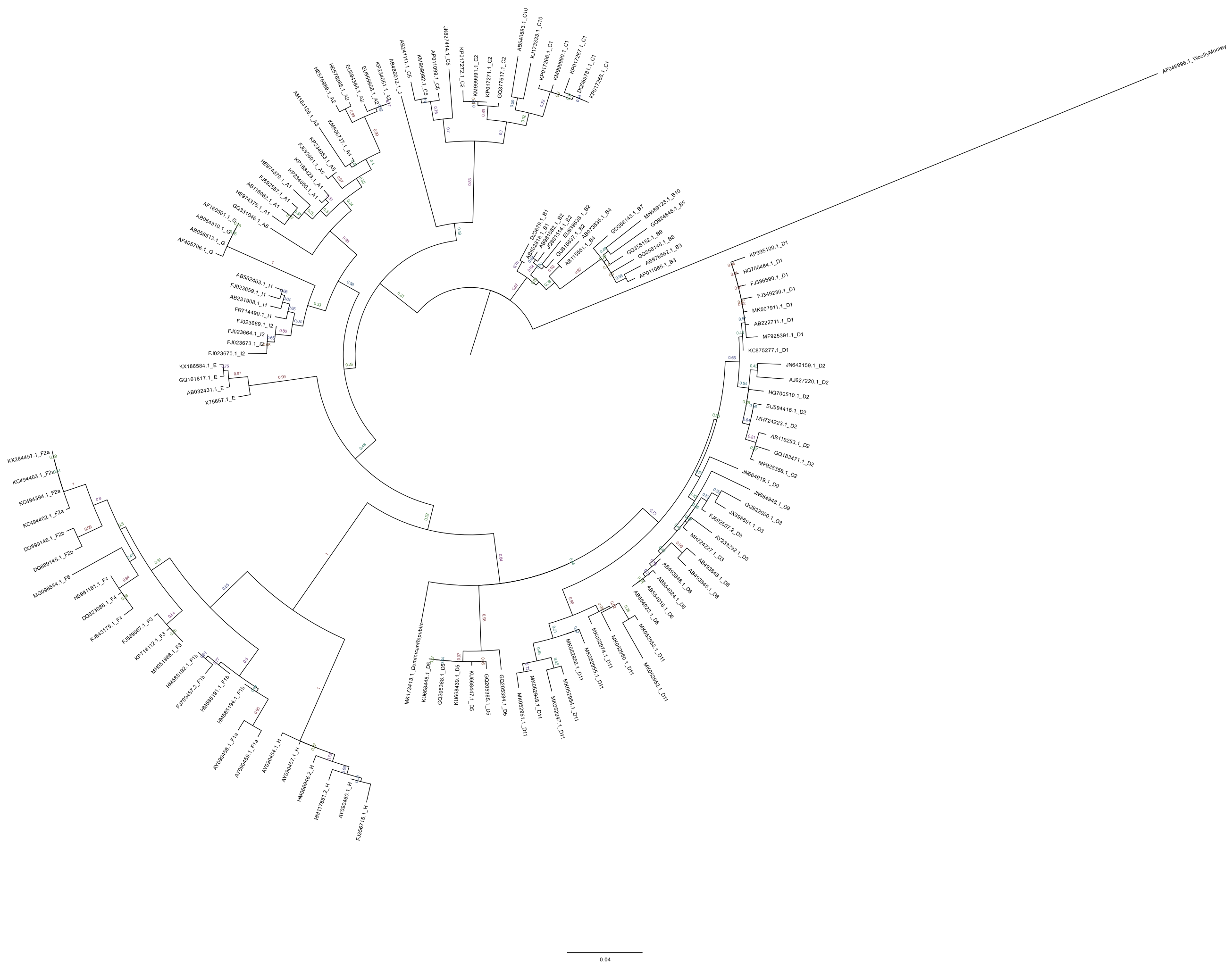

Tree 1. The evolutionary history was inferred by using the Maximum Likelihood method and Tamura-Nei model. The tree with the highest log likelihood (-6377.25) is shown. The percentage of replicate trees in which the associated taxa clustered together in the bootstrap test (1000 replicates) are shown next to the branches. Initial tree(s) for the heuristic search were obtained automatically by applying Neighbor-Join and BioNJ algorithms to a matrix of pairwise distances estimated using the Tamura-Nei model, and then selecting the topology with superior log likelihood value. A discrete Gamma distribution was used to model evolutionary rate differences among sites (5 categories (+G, parameter = 0.2050)). The tree is drawn to scale, with branch lengths measured in the number of substitutions per site. The analysis involved 135 nucleotide sequences, of which 134 were used as marker sequences to determine the genotype of 1 sequence. All positions containing gaps and missing data were eliminated. There was a total of 712 positions in the final dataset. Evolutionary analyses were conducted in MEGA7.

# VENEZUELA

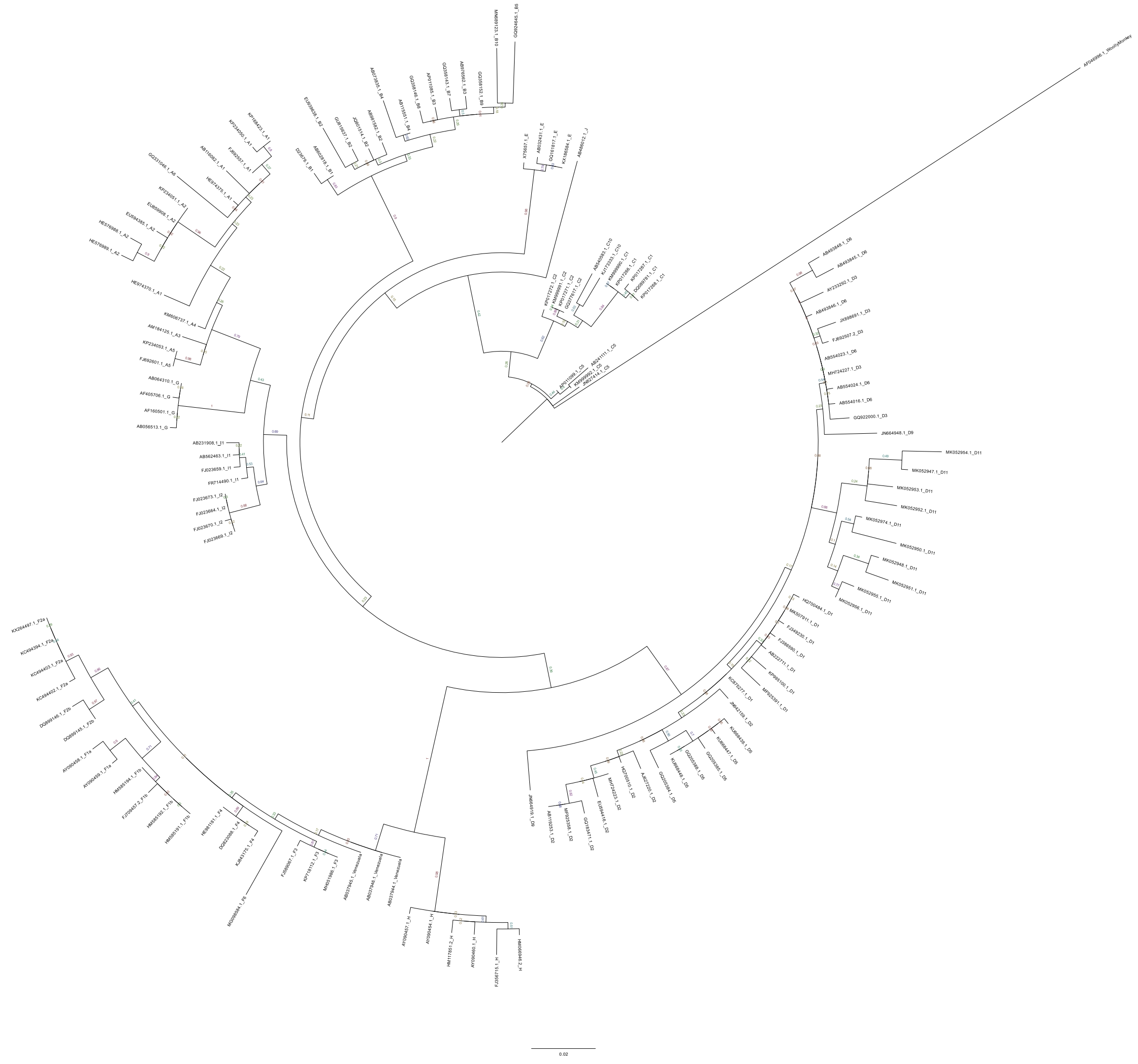

Tree 1. The evolutionary history was inferred by using the Maximum Likelihood method and Tamura-Nei model. The tree with the highest log likelihood (-4355.84) is shown. The percentage of replicate trees in which the associated taxa clustered together in the bootstrap test (1000 replicates) are shown next to the branches. Initial tree(s) for the heuristic search were obtained automatically by applying Neighbor-Join and BioNJ algorithms to a matrix of pairwise distances estimated using the Tamura-Nei model, and then selecting the topology with superior log likelihood value. A discrete Gamma distribution was used to model evolutionary rate differences among sites (5 categories (+G, parameter = 0.2229)). The tree is drawn to scale, with branch lengths measured in the number of substitutions per site. The analysis involved 137 nucleotide sequences, of which 134 were used as marker sequences to determine the genotype of 3 sequences. All positions containing gaps and missing data were eliminated. There was a total of 656 positions in the final dataset. Evolutionary analyses were conducted in MEGA7.

# USA

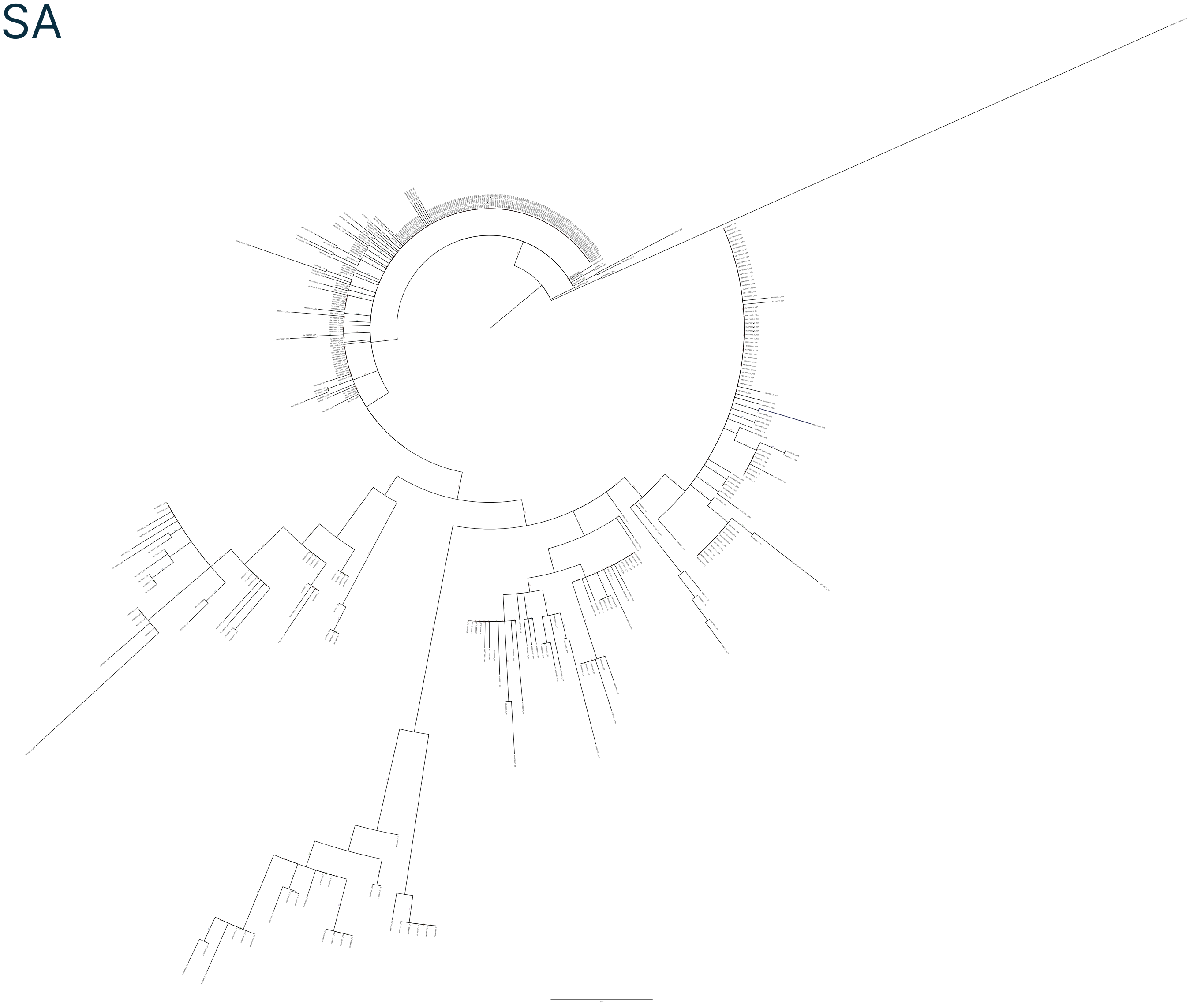

Tree 1. The evolutionary history was inferred by using the Maximum Likelihood method and Tamura-Nei model. The tree with the highest log likelihood (-2064.89) is shown. The percentage of replicate trees in which the associated taxa clustered together in the bootstrap test (1000 replicates) are shown next to the branches. Initial tree(s) for the heuristic search were obtained automatically by applying Neighbor-Join and BioNJ algorithms to a matrix of pairwise distances estimated using the Tamura-Nei model, and then selecting the topology with superior log likelihood value. A discrete Gamma distribution was used to model evolutionary rate differences among sites (5 categories (+G, parameter = 0.2400)). The tree is drawn to scale, with branch lengths measured in the number of substitutions per site. The analysis involved 426 nucleotide sequences, of which 134 were used as marker sequences to determine the genotype of 292 sequence. All positions containing gaps and missing data were eliminated. There was a total of 303 positions in the final dataset. Evolutionary analyses were conducted in MEGA7.

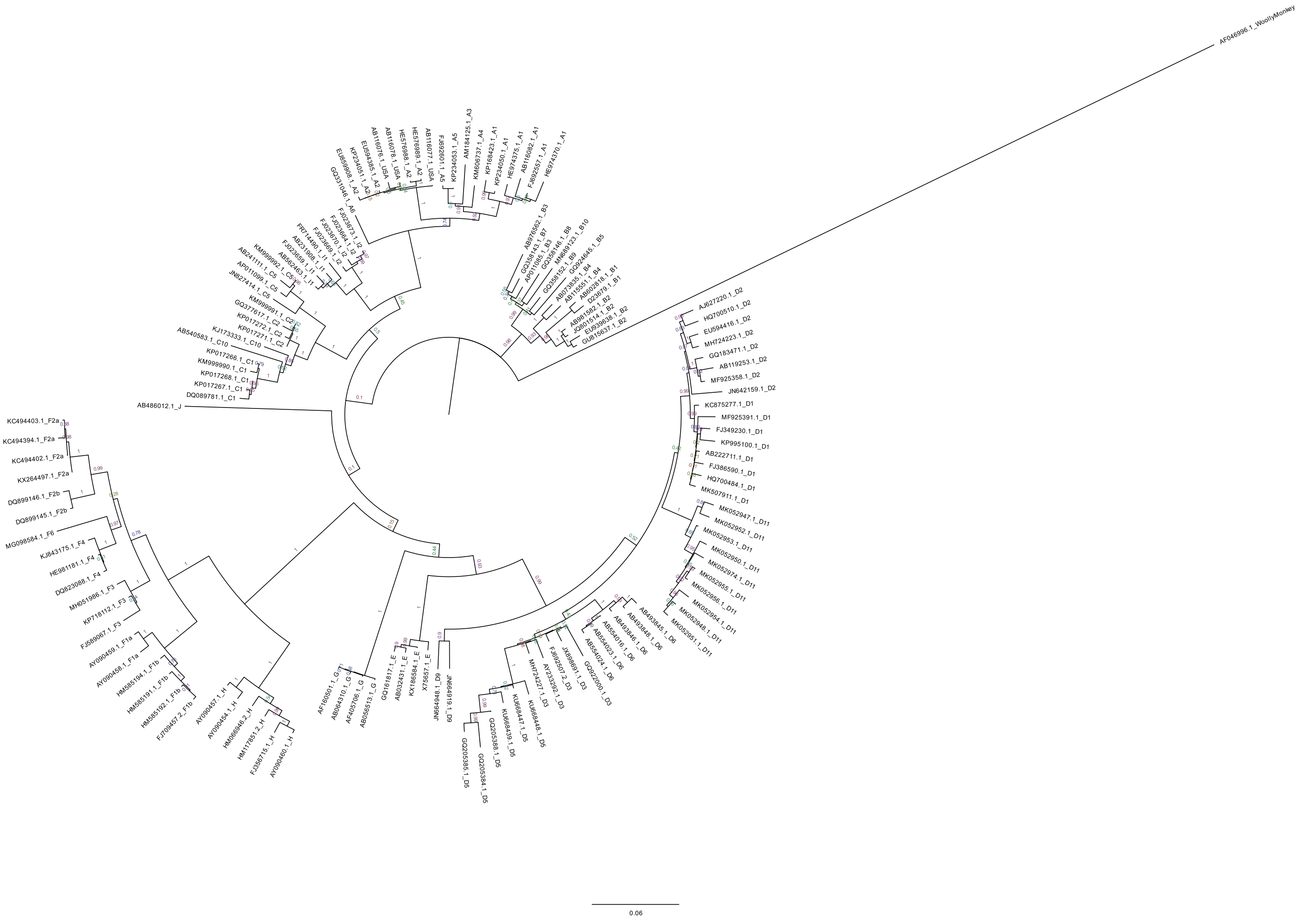

Tree 2. The evolutionary history was inferred by using the Maximum Likelihood method and Tamura-Nei model. The tree with the highest log likelihood (-35326.60) is shown. The percentage of replicate trees in which the associated taxa clustered together in the bootstrap test (1000 replicates) are shown next to the branches. Initial tree(s) for the heuristic search were obtained automatically by applying Neighbor-Join and BioNJ algorithms to a matrix of pairwise distances estimated using the Tamura-Nei model, and then selecting the topology with superior log likelihood value. A discrete Gamma distribution was used to model evolutionary rate differences among sites (5 categories (+G, parameter = 0.2773)). The tree is drawn to scale, with branch lengths measured in the number of substitutions per site. The analysis involved 137 nucleotide sequences, of which 134 were used as marker sequences to determine the genotype of 3 sequences. All positions containing gaps and missing data were eliminated. There was a total of 3001 positions in the final dataset. Evolutionary analyses were conducted in MEGA7.

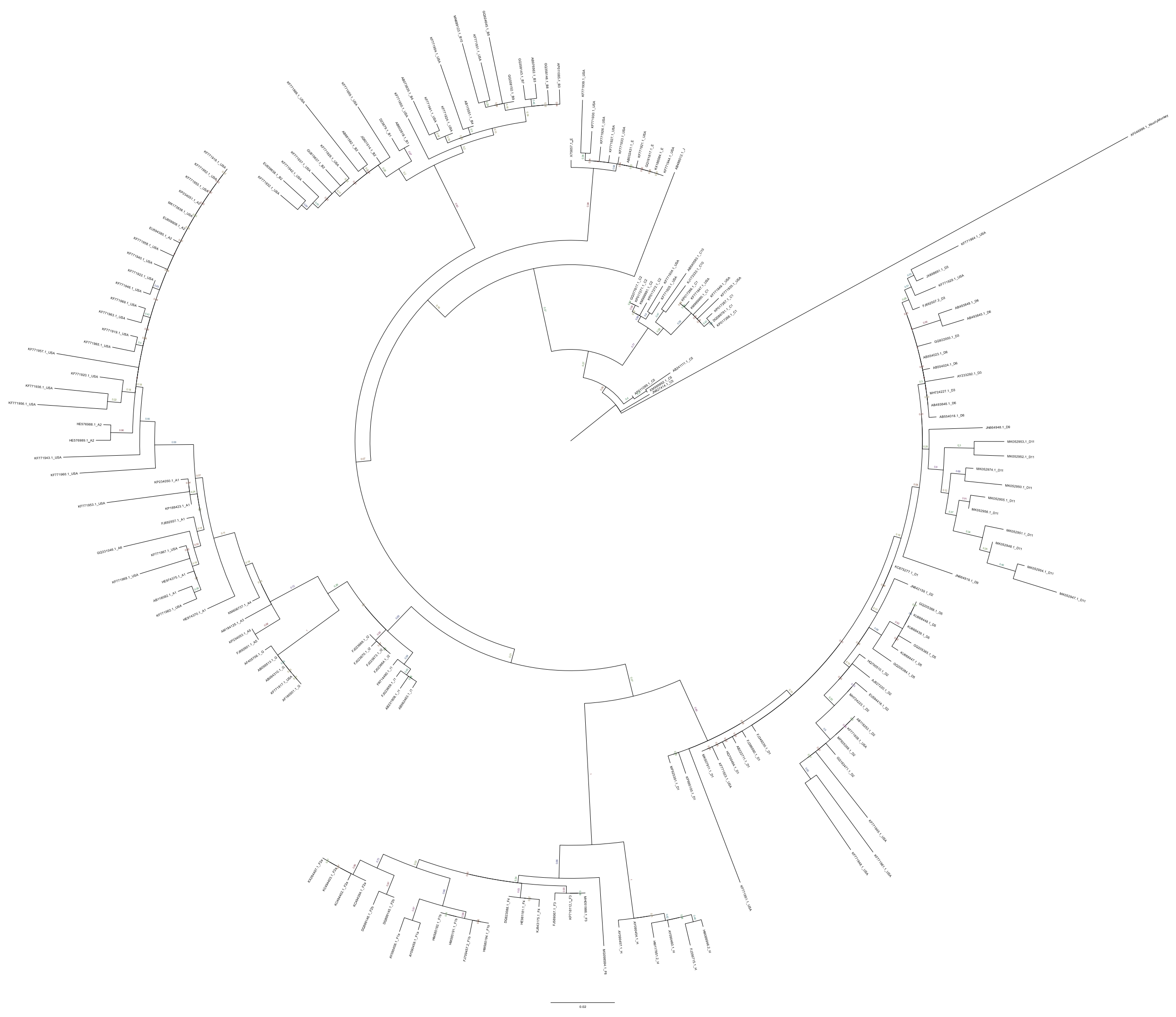

Tree 3. The evolutionary history was inferred by using the Maximum Likelihood method and Tamura-Nei model. The tree with the highest log likelihood (-5370.56) is shown. The percentage of replicate trees in which the associated taxa clustered together in the bootstrap test (1000 replicates) are shown next to the branches. Initial tree(s) for the heuristic search were obtained automatically by applying Neighbor-Join and BioNJ algorithms to a matrix of pairwise distances estimated using the Tamura-Nei model, and then selecting the topology with superior log likelihood value. A discrete Gamma distribution was used to model evolutionary rate differences among sites (5 categories (+G, parameter = 0.2660)). The tree is drawn to scale, with branch lengths measured in the number of substitutions per site. The analysis involved 188 nucleotide sequences, of which 134 were used as marker sequences to determine the genotype of 54 sequences. All positions containing gaps and missing data were eliminated. There was a total of 590 positions in the final dataset. Evolutionary analyses were conducted in MEGA7.

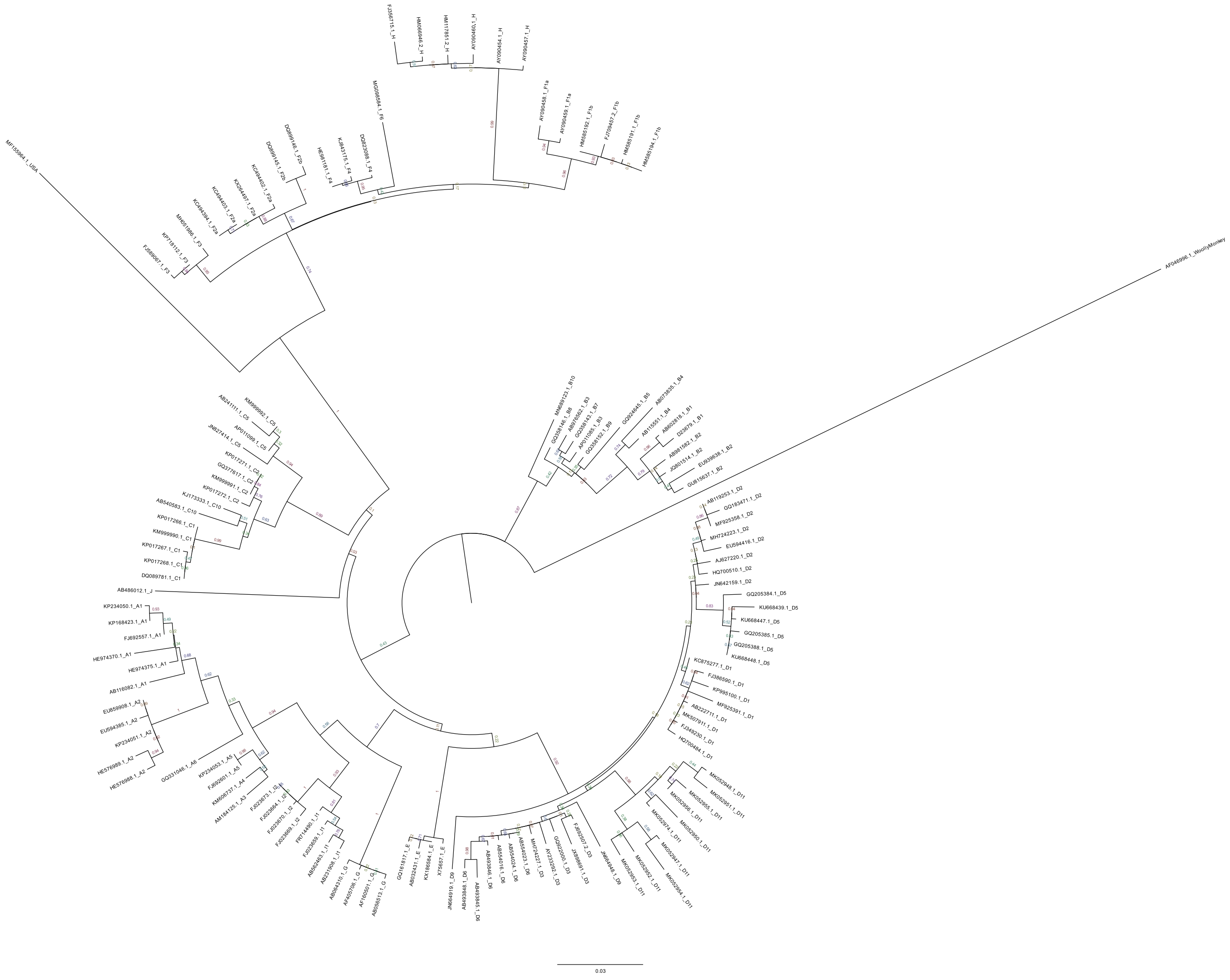

Tree 4. The evolutionary history was inferred by using the Maximum Likelihood method and Tamura-Nei model. The tree with the highest log likelihood (-6396.62) is shown. The percentage of replicate trees in which the associated taxa clustered together in the bootstrap test (1000 replicates) are shown next to the branches. Initial tree(s) for the heuristic search were obtained automatically by applying Neighbor-Join and BioNJ algorithms to a matrix of pairwise distances estimated using the Tamura-Nei model, and then selecting the topology with superior log likelihood value. A discrete Gamma distribution was used to model evolutionary rate differences among sites (5 categories (+G, parameter = 0.2483)). The tree is drawn to scale, with branch lengths measured in the number of substitutions per site. The analysis involved 135 nucleotide sequences, of which 134 were used as marker sequences to determine the genotype of 1 sequence. All positions containing gaps and missing data were eliminated. There was a total of 798 positions in the final dataset. Evolutionary analyses were conducted in MEGA7.

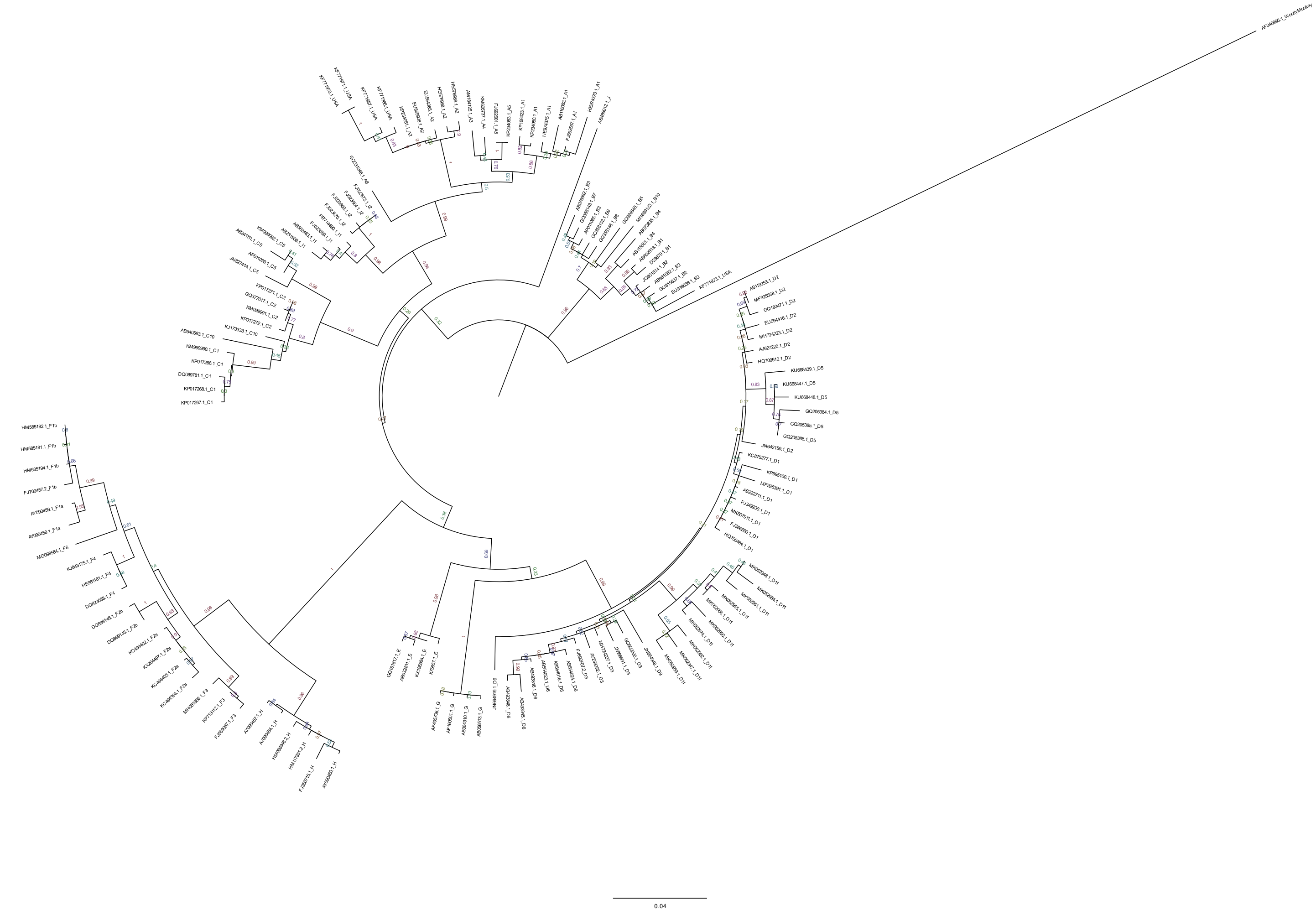

Tree 5. The evolutionary history was inferred by using the Maximum Likelihood method and Tamura-Nei model. The tree with the highest log likelihood (-8366.03) is shown. The percentage of replicate trees in which the associated taxa clustered together in the bootstrap test (1000 replicates) are shown next to the branches. Initial tree(s) for the heuristic search were obtained automatically by applying Neighbor-Join and BioNJ algorithms to a matrix of pairwise distances estimated using the Tamura-Nei model, and then selecting the topology with superior log likelihood value. A discrete Gamma distribution was used to model evolutionary rate differences among sites (5 categories (+G, parameter = 0.2691)). The tree is drawn to scale, with branch lengths measured in the number of substitutions per site. The analysis involved 139 nucleotide sequences, of which 134 were used as marker sequences to determine the genotype of 5 sequences. All positions containing gaps and missing data were eliminated. There was a total of 952 positions in the final dataset. Evolutionary analyses were conducted in MEGA7.

# BRAZIL

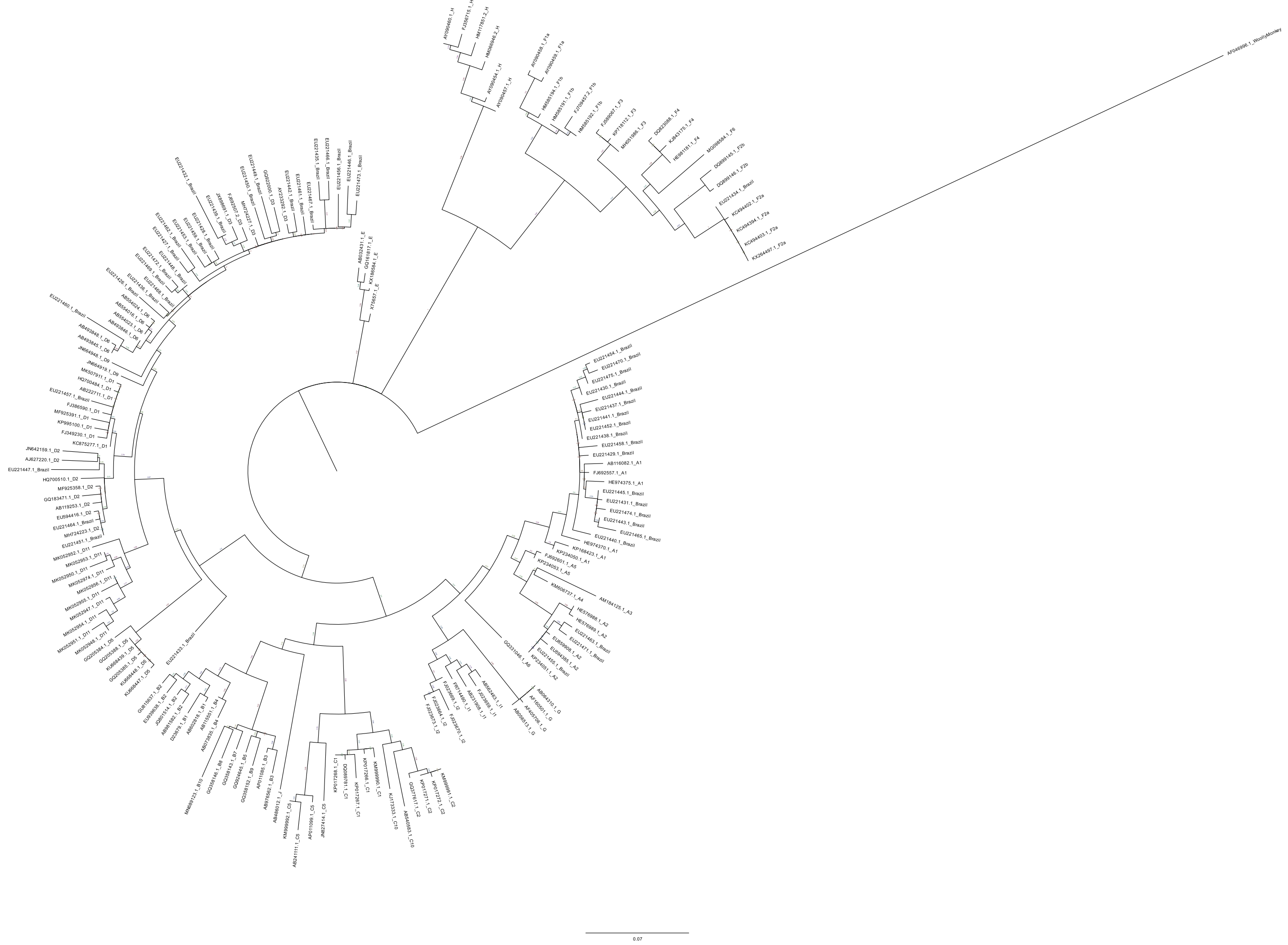

Tree 1. The evolutionary history was inferred by using the Maximum Likelihood method and Tamura-Nei model. The tree with the highest log likelihood (-6189.34) is shown. The percentage of replicate trees in which the associated taxa clustered together in the bootstrap test (1000 replicates) are shown next to the branches. Initial tree(s) for the heuristic search were obtained automatically by applying Neighbor-Join and BioNJ algorithms to a matrix of pairwise distances estimated using the Tamura-Nei model, and then selecting the topology with superior log likelihood value. A discrete Gamma distribution was used to model evolutionary rate differences among sites (5 categories (+G, parameter = 0.2601)). The tree is drawn to scale, with branch lengths measured in the number of substitutions per site. The analysis involved 184 nucleotide sequences, of which 134 were used as marker sequences to determine the genotype of 50 sequences. All positions containing gaps and missing data were eliminated. There was a total of 459 positions in the final dataset. Evolutionary analyses were conducted in MEGA7.

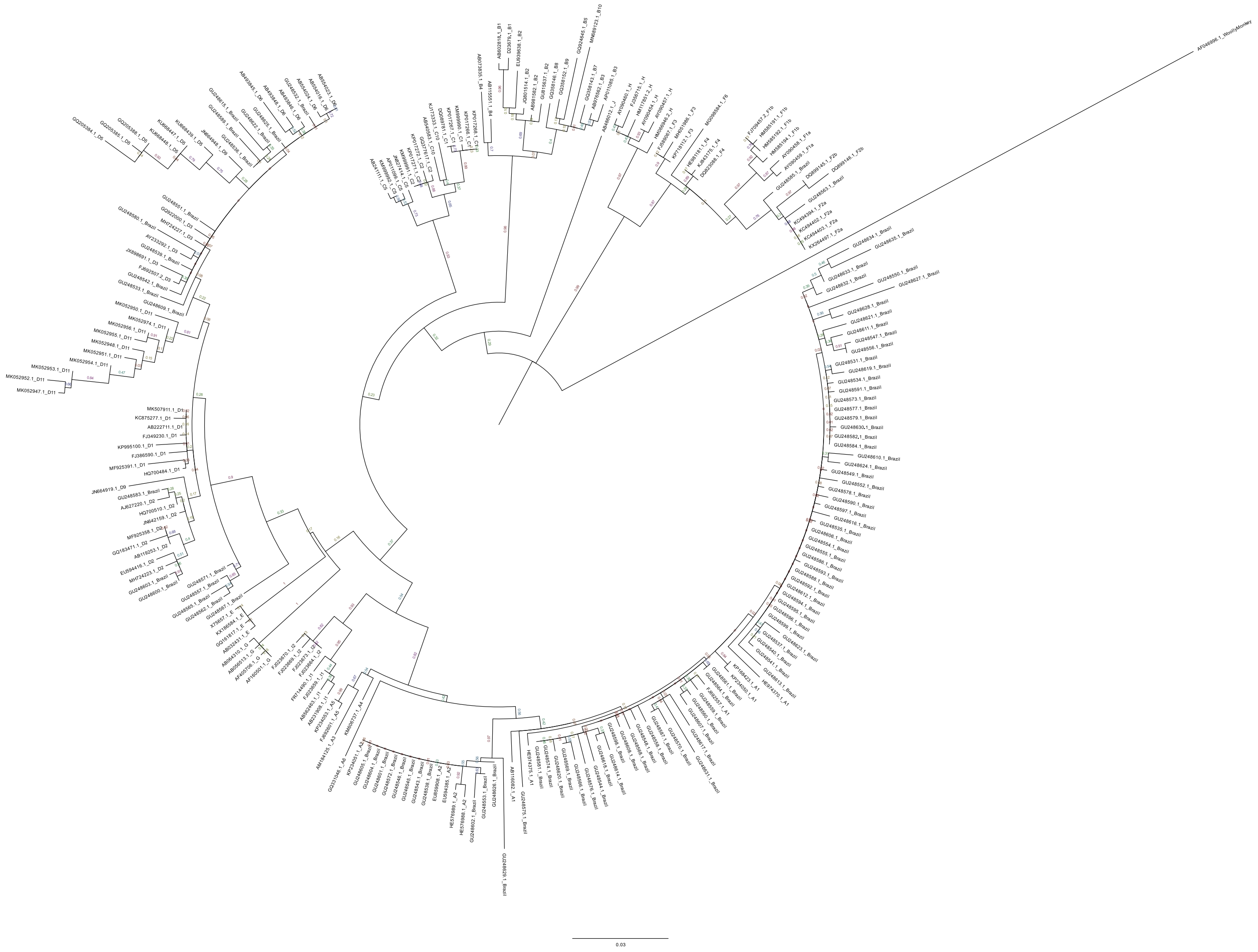

Tree 2. The evolutionary history was inferred by using the Maximum Likelihood method and Tamura-Nei model. The tree with the highest log likelihood (-6630.93) is shown. The percentage of replicate trees in which the associated taxa clustered together in the bootstrap test (1000 replicates) are shown next to the branches. Initial tree(s) for the heuristic search were obtained automatically by applying Neighbor-Join and BioNJ algorithms to a matrix of pairwise distances estimated using the Tamura-Nei model, and then selecting the topology with superior log likelihood value. A discrete Gamma distribution was used to model evolutionary rate differences among sites (5 categories (+G, parameter = 0.3496)). The tree is drawn to scale, with branch lengths measured in the number of substitutions per site. The analysis involved 239 nucleotide sequences, of which 134 were used as marker sequences to determine the genotype of 105 sequences. All positions containing gaps and missing data were eliminated. There was a total of 589 positions in the final dataset. Evolutionary analyses were conducted in MEGA7. After the creation of additional trees, the sequence with access number GU248567 could not be clearly genotyped and remained unclassified.

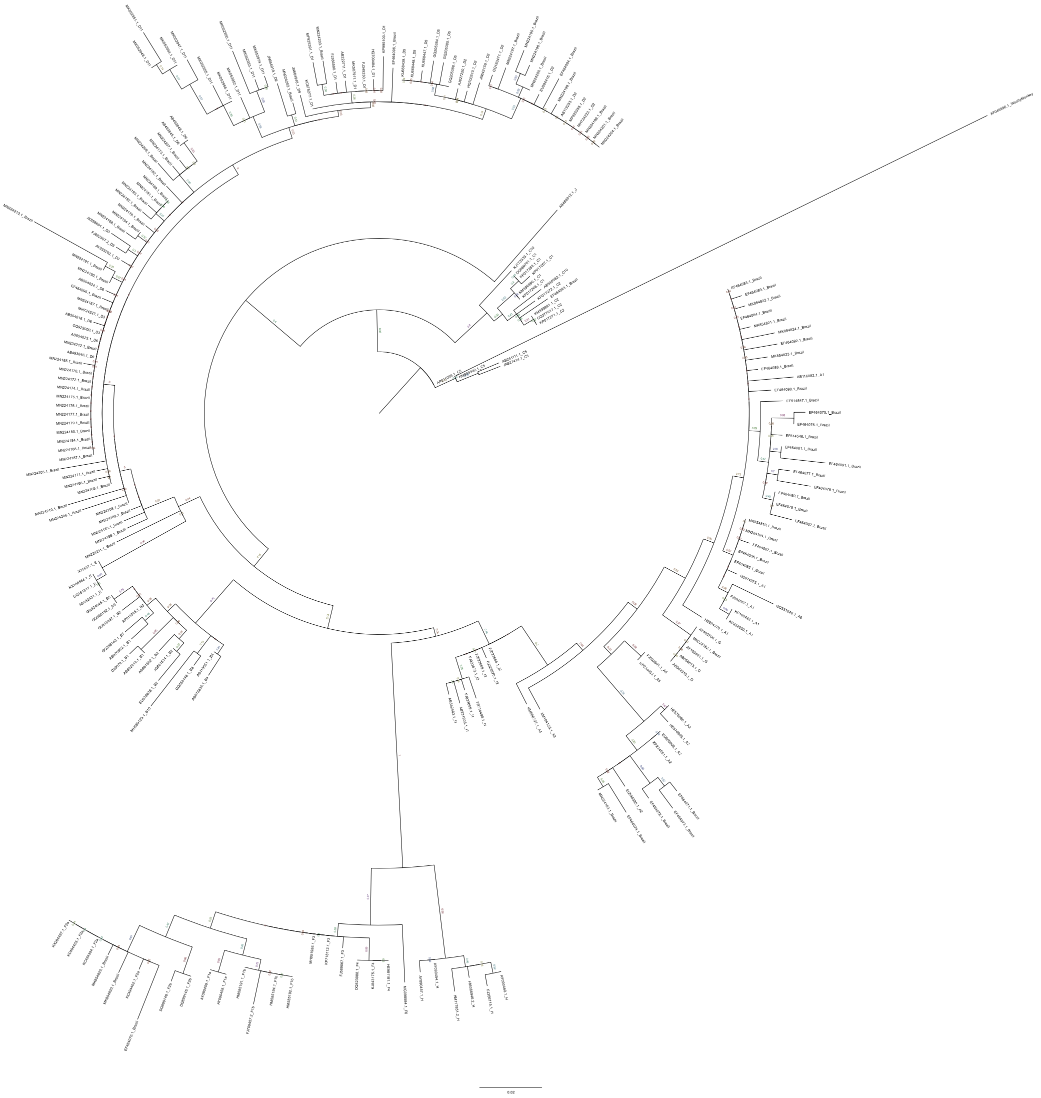

Tree 3. The evolutionary history was inferred by using the Maximum Likelihood method and Tamura-Nei model. The tree with the highest log likelihood (-3262.60) is shown. The percentage of replicate trees in which the associated taxa clustered together in the bootstrap test (1000 replicates) are shown next to the branches. Initial tree(s) for the heuristic search were obtained automatically by applying Neighbor-Join and BioNJ algorithms to a matrix of pairwise distances estimated using the Tamura-Nei model, and then selecting the topology with superior log likelihood value. A discrete Gamma distribution was used to model evolutionary rate differences among sites (5 categories (+G, parameter = 0.3101)). The tree is drawn to scale, with branch lengths measured in the number of substitutions per site. The analysis involved 222 nucleotide sequences, of which 134 were used as marker sequences to determine the genotype of 88 sequences. All positions containing gaps and missing data were eliminated. There was a total of 335 positions in the final dataset. Evolutionary analyses were conducted in MEGA7.

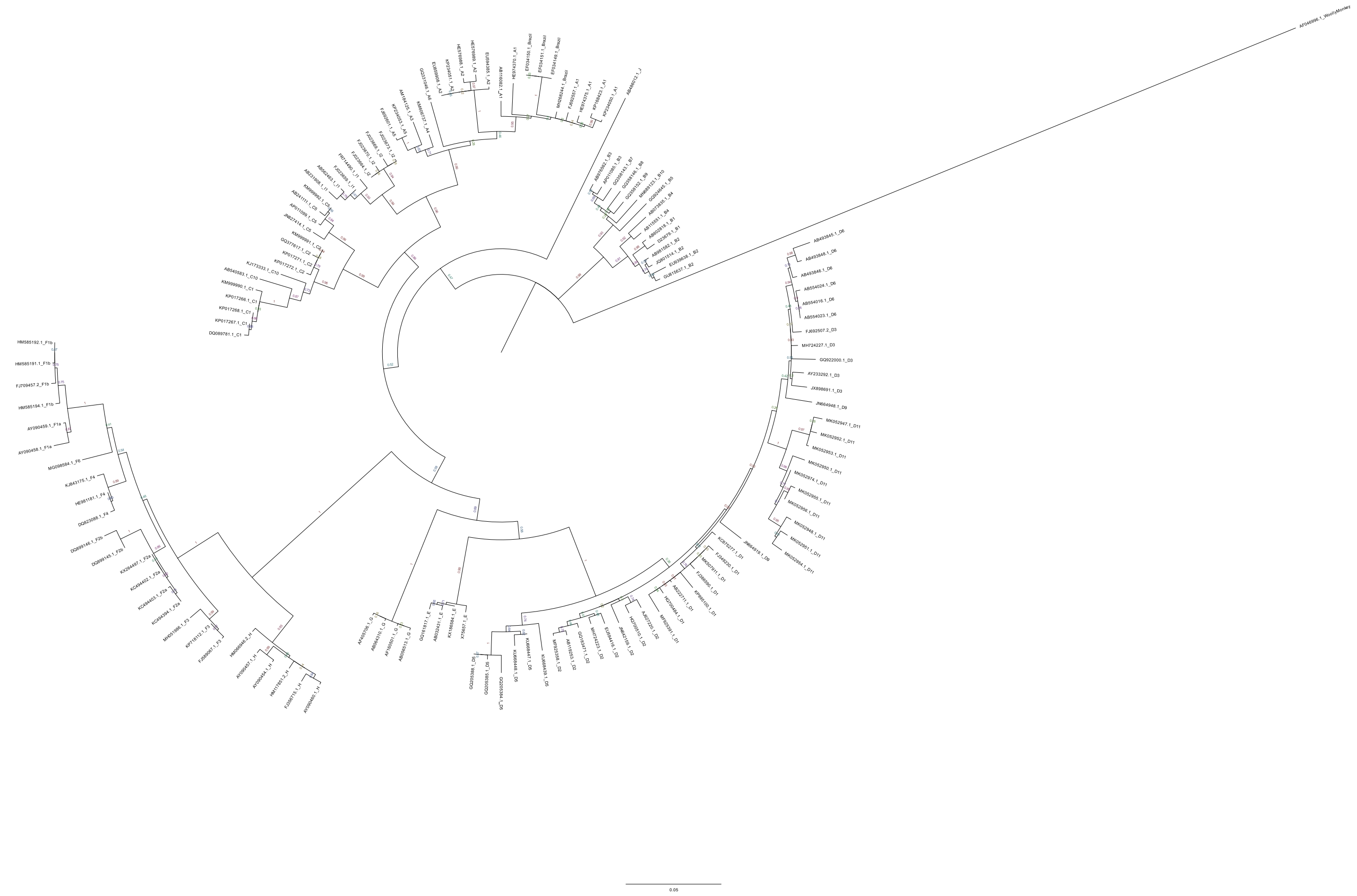

Tree 4. The evolutionary history was inferred by using the Maximum Likelihood method and Tamura-Nei model. The tree with the highest log likelihood (-9350.84) is shown. The percentage of replicate trees in which the associated taxa clustered together in the bootstrap test (1000 replicates) are shown next to the branches. Initial tree(s) for the heuristic search were obtained automatically by applying Neighbor-Join and BioNJ algorithms to a matrix of pairwise distances estimated using the Tamura-Nei model, and then selecting the topology with superior log likelihood value. A discrete Gamma distribution was used to model evolutionary rate differences among sites (5 categories (+G, parameter = 0.2941)). The tree is drawn to scale, with branch lengths measured in the number of substitutions per site. The analysis involved 138 nucleotide sequences, of which 134 were used as marker sequences to determine the genotype of 4 sequences. All positions containing gaps and missing data were eliminated. There was a total of 959 positions in the final dataset. Evolutionary analyses were conducted in MEGA7.

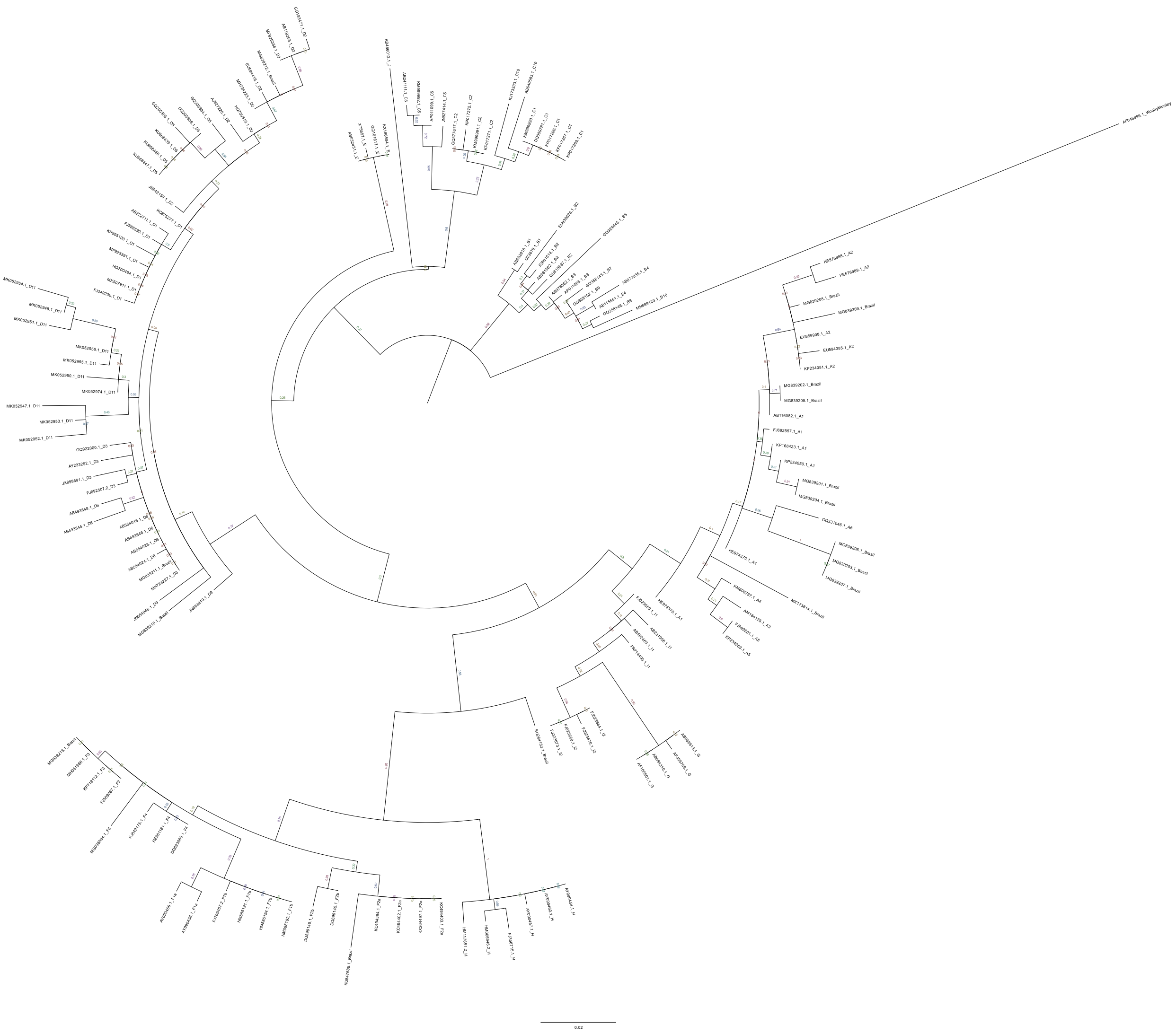

Tree 5. The evolutionary history was inferred by using the Maximum Likelihood method and Tamura-Nei model. The tree with the highest log likelihood (-3171.51) is shown. The percentage of replicate trees in which the associated taxa clustered together in the bootstrap test (1000 replicates) are shown next to the branches. Initial tree(s) for the heuristic search were obtained automatically by applying Neighbor-Join and BioNJ algorithms to a matrix of pairwise distances estimated using the Tamura-Nei model, and then selecting the topology with superior log likelihood value. A discrete Gamma distribution was used to model evolutionary rate differences among sites (5 categories (+G, parameter = 0.2091)). The tree is drawn to scale, with branch lengths measured in the number of substitutions per site. The analysis involved 150 nucleotide sequences, of which 134 were used as marker sequences to determine the genotype of 16 sequences. All positions containing gaps and missing data were eliminated. There was a total of 508 positions in the final dataset. Evolutionary analyses were conducted in MEGA7. After the creation of additional trees, the sequence with access number EU264153 could not be clearly genotyped and remained unclassified.
