# Supplementary material for: The Molecular Epidemiology of Hepatitis B Virus and Its Resistance-Associated Mutations in the Polymerase Gene in the Americas
Source: Microorganisms. 2025 Aug 16;13(8):1913. doi: 10.3390/microorganisms13081913 (PMC12388563; doi:10.3390/microorganisms13081913)
Supplement: Supplementary file 1 [file microorganisms-13-01913-s001.zip › Table S1 - Table of Reference sequences.pdf]

Country of Origin	Number of Sequences	Genbank Accession Numbers	Genotype	Source	Reference
Argentina	7	KJ843175	F4	Human	(1,2)
		DQ823088	F4	Human	(3)
		MG098584	F6	Human	(4)
		KJ676692	F6	Human	
		KJ676693	F6	Human	
		KJ676694	F6	Human	
		FJ356715	H	Human	(1)
Australia	2	AB048705	C4	Human	(2,5,6)
		AB048701	D4	Human	(7)
Bangladesh	3	AB116082	A1	Human	(5)
		MF925391	D1	Human	(7)
		MF925358	D2	Human	(1,7)
Belgium	8	EU859908	A2	Human	(5)
		KP234051	A2	Human	(8)
		GQ331046	A6	Human	(1)
		MN585097	A8	Human	(9)
		MN585096	A8	Human	
		FJ349230	D1	Human	(7)
		MN585095	D12	Human	(7,9)
		MN585094	D12	Human	
Brazil	8	MH724223	D2	Human	(7)
		MH724227	D3	Human	
		KJ470893	D4	Human	
		KC494403	F2a	Human	(10)
		KC494394	F2a	Human	
		KC494402	F2a	Human	(3,10)
		KX264497	F2a	Human	(8,10)
		HE981181	F4	Human	(3)
Cambodia	1	AB115551	B4	Human	(2)
Cameroon	3	FN545832	A7	Human	(5)
		FN545839	A7	Human	
		FN545833	A7	Human	
Canada	3	KP659253	B6	Human	(11)
		GQ922000	D3	Human	(5,7)
		GQ922005	D4	Human	
Chile	4	HM585194	F1b	Human	(1,2,7)
		FJ709457	F1b	Human	(10)
		HM585192	F1b	Human	(3)
		HM585191	F1b	Human	(3)
China	22	EU939638	B2	Human	(5)
		MN689123	B10	Human	(11)
		DQ089781	C1	Human	(1)
		KP017267	C1	Human	(12)
		KP017268	C1	Human	
		GQ377617	C2	Human	(1)
		KM999991	C2	Human	(8,12)
		KJ173333	C10	Human	(1)
		MG826140	C17	Human	(11)
		FJ386590	D1	Human	(7)
		MK052948	D11	Human	
		MK052954	D11	Human	
		MK052951	D11	Human	
		MK052955	D11	Human	
		MK052956	D11	Human	
		MK052950	D11	Human	
		MK052974	D11	Human	
		MK052953	D11	Human	
		MK052947	D11	Human	
		MK052952	D11	Human	
		FR714490	I1	Human	(13)

		FR714502	I3	Human	
Colombia	1	FJ589067	F3	Human	(3)
Costa Rica	2	AY090459	F1a	Human	(2,3)
		AY090458	F1a	Human	(3)
Cuba	3	KM606737	A4	Human	(1)
		KM606755	D4	Human	(3,7)
		KM606744	D4	Human	(7)
Estonia	1	EU594385	A2	Human	(1)
Ethiopia	9	KX357622	D10	Human	(7)
		KX357628	D10	Human	
		KX357627	D10	Human	
		KX357626	D10	Human	
		KX357623	D10	Human	
		KX357629	D10	Human	
		KX357625	D10	Human	
		KX357633	D10	Human	
		KX357624	D10	Human	
France	4	HE576989	A2	Human	(3,14)
		HE576988	A2	Human	(3)
		X75657	E	Human	(3,8,15,16)
		AF160501	G	Human	(6,8,16)
French Polynesia	1	X75656	C3	Human	(2,6)
Gabon	2	AM184126	A3	Human	(3)
		AM184125	A3	Human	
Germany	1	AF405706	G	Human	(16)
Guinea	2	GQ161817	E	Human	(1)
		KX186584	E	Human	(3,8)
Haiti	5	FJ692557	A1	Human	(1)
		FJ692601	A5	Human	(1,14)
		KP234053	A5	Human	(8,14,15)
		FJ692507	D3	Human	(1,7)
		FJ692533	D4	Human	(1,5,7,15)
India	13	KP017266	C1	Human	(12)
		KC875277	D1	Human	(1)
		MK507911	D1	Human	(7)
		GQ183471	D2	Human	
		KF192831	D4	Human	
		GQ205385	D5	Human	
		GQ205384	D5	Human	
		GQ205388	D5	Human	(3,7)
		KU668439	D5	Human	(7)
		KU668447	D5	Human	
		KU668448	D5	Human	
		JN664948	D9	Human	
		JN664919	D9	Human	
Indonesia	25	AP011085	B3	Human	(1,2)
		AB976562	B3	Human	(11)
		GQ358143	B7	Human	
		GQ358146	B8	Human	(15)
		GQ358152	B9	Human	(6)
		AP011099	C5	Human	(1,2)
		KM999993	C6	Human	(2,8,12,15)
		AB493843	C6	Human	(5,6)
		AP011107	C8	Human	(1,2,6,15)
		AP011106	C8	Human	
		AP011108	C9	Human	(1,2,5,6,15)
		AB540583	C10	Human	(2,6)
		AB554015	C11	Human	(1)
		AB554020	C11	Human	(2,6)
		AB554025	C12	Human	
		AB644281	C13	Human	(2,6,15)
		AB644284	C14	Human	(6)

		AB644286	C15	Human	(2,6,15)
		AB644287	C16	Human	
		AB493845	D6	Human	(2,7)
		AB493848	D6	Human	(5,7)
		AB493846	D6	Human	(2,7)
		AB554016	D6	Human	(7)
		AB554023	D6	Human	
		AB554024	D6	Human	
Japan	6	AB602818	B1	Human	(16)
		D23679	B1	Human	(1)
		AB981582	B2	Human	(11)
		AB073835	B4	Human	(1,2,6)
		AB119253	D2	Human	(7)
		AB486012	J	Human	(1,2,6,15)
Kenya	1	KP168423	A1	Human	(1)
Lebanon	1	JN642159	D2	Human	(7)
Laos	5	FJ023659	I1	Human	(6)
		FJ023669	I2	Human	(1)
		FJ023670	I2	Human	(3)
		FJ023664	I2	Human	(2,6)
		FJ023673	I2	Human	(3)
Liberia	1	AB032431	E	Chimpanzee	(6,16,17)
Malaysia	2	GQ924645	B5	Human	(11)
		KP017271	C2	Human	(12)
Martinique	2	HE974375	A1	Human	(3)
		HE974370	A1	Human	(3,14)
Mexico	2	HM117851	H	Human	(10)
		HM066946	H	Human	
New Zealand	2	HQ700484	D1	Human	(7)
		HQ700458	D4	Human	
New Caledonia	1	HQ700510	D2	Human	
Nicaragua	2	AY090454	H	Human	(8,15,16)
		AY090457	H	Human	(2,3,16)
Nigeria	4	FN594770	D8	Human	(7)
		FN594769	D8	Human	
		FN594771	D8	Human	
		FN594768	D8	Human	
Panama	9	KJ638656	F1c	Human	(4)
		KJ638657	F1c	Human	
		KJ638658	F1c	Human	
		KJ638663	F1c	Human	
		KJ638664	F1c	Human	
		KP718107	F1c	Human	
		KP718112	F3	Human	(3)
		KJ638660	F5	Human	(4)
		KJ638662	F5	Human	
Philippines	3	KM999992	C5	Human	(2,8,12,15)
		AB241111	C5	Human	(6)
		EU670263*	C7	Human	(1,2,6)
Russia	1	EU594416	D2	Human	(7)
South Africa	3	KP234050	A1	Human	(14,15)
		AY233292	D3	Human	(7)
		KF922432	D4	Human	
Spain	2	AJ627220	D2	Human	
		AJ627219	D4	Human	
Sweden	1	JX898691	D3	Human	
Taiwan	1	GU815637	B2	Human	(1)
Thailand	5	JQ801514	B2	Human	
		KM999990	C1	Human	(8,12,15)
		KP017272	C2	Human	(15)
		JN827414	C5	Human	(6)
		EU155829	J	Gibbon	(3)

Tunisia	6	FJ904435	D7	Human	(1,7)
		FJ904419	D7	Human	(7)
		FJ904410	D7	Human	
		FJ904395	D7	Human	
		FJ904438	D7	Human	
		FJ904439	D7	Human	(5,7)
USA	5	AB287314	B6	Human	(1,6)
		AB064310	G	Human	(2,3,16)
		AB056513	G	Human	(1,2)
		AY090460	H	Human	(3,6,16)
		AF046996	WMHBV (Outgroup)	Woolly Monkey	(3)
Uzbekistan	1	AB222711	D1	Human	(1)
Venezuela	4	KP995100	D1	Human	(7)
		DQ899145	F2b	Human	(3)
		DQ899146	F2b	Human	
		MH051986	F3	Human	(1,2,10)
Vietnam	2	AB562463	I1	Human	(1)
		AB231908	I1	Human	(18)

*Marked as C6 in GenBank, but used as a reference as no other C7 sequences have been proposed as a reference in past literature

REFERENCES

1. McNaughton AL, Revill PA, Littlejohn M, Matthews PC, Azim Ansari M. Analysis of genomic-length HBV sequences to determine genotype and subgenotype reference sequences. J Gen Virol [Internet]. 2020 [cited 2024 Mar 22];101(3):271–83. Available from: https://pubmed.ncbi.nlm.nih.gov/32134374/

2. Araujo NM, Teles SA, Spitz N. Comprehensive Analysis of Clinically Significant Hepatitis B Virus Mutations in Relation to Genotype, Subgenotype and Geographic Region. Front Microbiol [Internet]. 2020 Dec 14 [cited 2024 Mar 22];11. Available from: https://pubmed.ncbi.nlm.nih.gov/33381105/

3. Jose-Abrego A, Roman S, Rebello Pinho JR, Gomes-Gouvêa MS, Panduro A. High Frequency of Antiviral Resistance Mutations in HBV Genotypes A2 and H: Multidrug Resistance Strains in Mexico. J Clin Transl Hepatol [Internet]. 2023 Sep 1 [cited 2024 Mar 22];11(5):1023–34. Available from: https://pubmed.ncbi.nlm.nih.gov/37577226/

4. Mojsiejczuk L, Torres C, Flichman D, Campos RH. Long-term evolution of hepatitis B virus genotype F: Strong association between viral diversification and the prehistoric settlement of Central and South America. J Viral Hepat [Internet]. 2020 Jun 1 [cited 2024 Mar 22];27(6):620–30. Available from: https://pubmed.ncbi.nlm.nih.gov/32052519/

5. Yin Y, He K, Wu B, Xu M, Du L, Liu W, et al. A systematic genotype and subgenotype re-ranking of hepatitis B virus under a novel classification standard. Heliyon [Internet]. 2019 Oct 1 [cited 2024 Mar 22];5(10). Available from: https://pubmed.ncbi.nlm.nih.gov/31687483/

6. Wang XY, Harrison TJ, He X, Chen QY, Li GJ, Liu MH, et al. The prevalence of mutations in the major hydrophilic region of the surface antigen of hepatitis B virus varies with subgenotype. Epidemiol Infect [Internet]. 2015 Dec 1 [cited 2024 Mar 22];143(16):3572–82. Available from: https://pubmed.ncbi.nlm.nih.gov/25903946/

7. Sant'Anna TB, Araujo NM. Hepatitis B Virus Genotype D: An Overview of Molecular Epidemiology, Evolutionary History, and Clinical Characteristics. Microorganisms [Internet]. 2023 May 1 [cited 2024 Mar 22];11(5). Available from: https://pubmed.ncbi.nlm.nih.gov/37317074/

8. Chen L, Shi Y, Yang W, Zhang Y, Xie Q, Li Y, et al. Differences in Cpg Island Distribution Between Subgenotypes of the Hepatitis B Virus Genotype. Med Sci Monit [Internet]. 2018 Sep 25 [cited 2024 Mar 22];24:6781–94. Available from: https://pubmed.ncbi.nlm.nih.gov/30253420/

9. Thijssen M, Trovão NS, Mina T, Maes P, Pourkarim MR. Novel hepatitis B virus subgenotype A8 and quasi-subgenotype D12 in African–Belgian chronic carriers. International Journal of Infectious Diseases. 2020 Apr 1;93:98–101.

10. Gionda PO, Gomes-Gouvea M, Malta F de M, Sebe P, Salles APM, Francisco R dos S, et al. Analysis of the complete genome of HBV genotypes F and H found in Brazil and Mexico using the next generation sequencing method. Ann Hepatol [Internet]. 2022 Jan 1 [cited 2024 Mar 22];27 Suppl 1. Available from: https://pubmed.ncbi.nlm.nih.gov/34757035/

11. Chen J, Li L, Yin Q, Shen T. A review of epidemiology and clinical relevance of Hepatitis B virus genotypes and subgenotypes. Clin Res Hepatol Gastroenterol [Internet]. 2023 Aug 1 [cited 2024 Mar 22];47(7). Available from: https://pubmed.ncbi.nlm.nih.gov/37479136/

12. Zhu HL, Wang CT, Xia JB, Li X, Zhang ZH. Establishment of reference sequences of hepatitis B virus genotype C subgenotypes. Genet Mol Res [Internet]. 2015 Dec 9 [cited 2024 Mar 22];14(4):16521–34. Available from: https://pubmed.ncbi.nlm.nih.gov/26662451/

13. Jia HH, Chen QY, Jiang ZH, Wang XY, Zhang WJ, He X, et al. A novel subgenotype I3 of hepatitis B virus in Guangxi, China: a 15-year follow-up study. Virus Genes [Internet]. 2023 Jun 1 [cited 2024 Mar 22];59(3):359–69. Available from: https://pubmed.ncbi.nlm.nih.gov/36841897/

14. Cai Q, Zhu H, Zhang Y, Li X, Zhang Z. Hepatitis B virus genotype A: design of reference sequences for sub-genotypes. Virus Genes [Internet]. 2016 Jun 1 [cited 2024 Mar 22];52(3):325–33. Available from: https://pubmed.ncbi.nlm.nih.gov/27002608/

15. McNaughton AL, D'Arienzo V, Ansari MA, Lumley SF, Littlejohn M, Revill P, et al. Insights From Deep Sequencing of the HBV Genome-Unique, Tiny, and Misunderstood. Gastroenterology [Internet]. 2019 Jan 1 [cited 2024 Mar 22];156(2):384–99. Available from: https://pubmed.ncbi.nlm.nih.gov/30268787/

16. National Center for Biotechnology Information. NCBI. 2004 [cited 2024 Mar 22]. Genotyping. Available from: https://www.ncbi.nlm.nih.gov/projects/genotyping/formpage.cgi

17. Jose-Abrego A, Roman S, Rebello Pinho JR, de Castro VFD, Panduro A. Hepatitis B Virus (HBV) Genotype Mixtures, Viral Load, and Liver Damage in HBV Patients Co-infected With Human Immunodeficiency Virus. Front Microbiol [Internet]. 2021 Mar 3 [cited 2024 Mar 22];12. Available from: https://pubmed.ncbi.nlm.nih.gov/33746932/

18. Bell TG, Yousif M, Kramvis A. Bioinformatic curation and alignment of genotyped hepatitis B virus (HBV) sequence data from the GenBank public database. Springerplus [Internet]. 2016 Dec 1 [cited 2024 Mar 22];5(1). Available from: https://pubmed.ncbi.nlm.nih.gov/27843753/
