# Supplementary material for: The Molecular Epidemiology of Hepatitis B Virus and Its Resistance-Associated Mutations in the Polymerase Gene in the Americas
Source: Microorganisms. 2025 Aug 16;13(8):1913. doi: 10.3390/microorganisms13081913 (PMC12388563; doi:10.3390/microorganisms13081913)
Supplement: Supplementary file 1 [file microorganisms-13-01913-s001.zip › Table S2 - Sequence Classification according to genotype.pdf]

Supplementary Table 2. Classification of sequences according to genotype, country, and their alignment to the HBV complete genome reference sequence NC_003977.2.

Genebank accession number	Genotype	Country	Aligned region on reference HBV	Pair-base length
DQ249922	E	Argentina	205-789	585
DQ249923	E	Argentina	205-789	585
DQ249924	A	Argentina	205-789	585
DQ249925	A	Argentina	205-789	585
DQ249926	A	Argentina	205-789	585
DQ249927	A	Argentina	205-789	585
DQ249928	A	Argentina	205-789	585
DQ249929	A	Argentina	205-789	585
DQ249930	A	Argentina	205-789	585
DQ249931	A	Argentina	205-789	585
DQ249932	A	Argentina	205-789	585
DQ249933	A	Argentina	205-789	585
DQ249934	A	Argentina	205-789	585
DQ249935	A	Argentina	205-789	585
DQ249936	A	Argentina	205-789	585
DQ249937	D	Argentina	205-789	585
DQ249938	A	Argentina	205-789	585
DQ249939	D	Argentina	205-789	585
DQ249940	A	Argentina	205-789	585
DQ249941	A	Argentina	205-789	585
EF468000	F	Argentina	282-793	512
HM216215	A	Argentina	259-766	508
HM216216	A	Argentina	259-766	508
HM216217	A	Argentina	259-766	508
HM216218	A	Argentina	259-766	508
HM216219	A	Argentina	259-766	508
HM216220	A	Argentina	259-766	508
HM216221	A	Argentina	259-766	508
HM216222	A	Argentina	259-766	508
HM216223	A	Argentina	259-766	508
HM216224	A	Argentina	259-766	508
HM216225	A	Argentina	259-766	508
HM216226	B	Argentina	259-772	508
HM216227	B	Argentina	262-772	505
HM216228	D	Argentina	259-766	508
HM216229	D	Argentina	259-766	508
HM216230	D	Argentina	259-766	508
HM216231	D	Argentina	259-766	508
HM216232	D	Argentina	259-766	508
HM216233	D	Argentina	259-766	508
HM216234	D	Argentina	259-766	508
HM216235	D	Argentina	259-766	508
HM216236	D	Argentina	259-766	508
HM216237	D	Argentina	259-766	508
HM216238	D	Argentina	259-766	508
HM216239	D	Argentina	259-766	508
HM216240	D	Argentina	259-766	508
HM216241	F	Argentina	259-766	508
HM216242	F	Argentina	259-766	508

Genebank accession number	Genotype	Country	Aligned region on reference HBV	Pair-base length
HM590576	B	Canada	1-3182	681
MK174221	F	Colombia	216-955	740
MK173413	D	Rep. Dominican	216-955	740
AB116076	A	U.S.A	Complete Genome	3221
AB116077	A	U.S.A	Complete Genome	3221
AB116078	A	U.S.A	Complete Genome	3221
HM173808	B	U.S.A	417-881	465
HM173809	C	U.S.A	417-881	465
HM173810	C	U.S.A	417-881	465
HM173811	B	U.S.A	417-881	465
HM173812	B	U.S.A	417-881	465
HM173813	C	U.S.A	417-881	465
HM173814	B	U.S.A	417-881	465
HM173815	B	U.S.A	417-881	465
HM173816	B	U.S.A	417-881	465
HM173817	B	U.S.A	417-881	465
HM173818	C	U.S.A	417-881	465
HM173819	C	U.S.A	417-881	465
HM173820	C	U.S.A	417-881	465
HM173821	C	U.S.A	417-881	465
HM173822	B	U.S.A	417-881	465
HM173823	B	U.S.A	417-881	465
HM173824	C	U.S.A	417-881	465
HM173825	B	U.S.A	417-881	465
HM173826	B	U.S.A	417-881	465
HM173827	B	U.S.A	417-881	465
HM173828	B	U.S.A	417-881	465
HM173829	B	U.S.A	417-881	465
HM173830	C	U.S.A	417-881	465
HM173831	C	U.S.A	417-881	465
HM173832	B	U.S.A	417-866	450
HM173833	C	U.S.A	417-881	465
HM173834	B	U.S.A	417-881	465
HM173835	B	U.S.A	417-881	465
HM173836	B	U.S.A	417-881	465
HM173837	B	U.S.A	417-881	465
HM173838	B	U.S.A	417-881	465
HM173839	B	U.S.A	417-881	465
HM173840	C	U.S.A	417-881	465
HM173841	C	U.S.A	417-881	465
HM173842	B	U.S.A	417-881	465
HM173843	B	U.S.A	417-881	465
HM173844	B	U.S.A	417-881	465
HM173845	B	U.S.A	417-881	465
HM173846	B	U.S.A	420-881	462
HM173847	C	U.S.A	417-881	465
HM173848	C	U.S.A	417-881	465
HM173849	C	U.S.A	417-881	465
HM173850	C	U.S.A	417-881	465

HM216243	F	Argentina	259-766	508	HM173851	C	U.S.A	417-881	465
HM216244	F	Argentina	259-766	508	HM173852	B	U.S.A	417-881	465
HM216245	F	Argentina	259-766	508	HM173853	B	U.S.A	417-881	465
HM216246	F	Argentina	259-766	508	HM173854	C	U.S.A	417-881	465
HM216247	F	Argentina	259-766	508	HM173855	B	U.S.A	417-881	465
HM216248	F	Argentina	259-766	508	HM173856	C	U.S.A	417-866	450
HM216249	F	Argentina	259-766	508	HM173857	A	U.S.A	417-881	465
HM216250	F	Argentina	259-766	508	HM173858	C	U.S.A	417-881	465
HM216251	F	Argentina	259-766	508	HM173859	C	U.S.A	417-881	465
HM216252	F	Argentina	259-766	508	HM173860	B	U.S.A	417-881	465
HM216253	F	Argentina	259-766	508	HM173861	C	U.S.A	417-866	450
HM216254	F	Argentina	259-766	508	HM173862	B	U.S.A	417-881	465
HM216255	H	Argentina	259-766	508	HM173863	C	U.S.A	417-881	465
HM216256	A	Argentina	259-766	508	HM173864	B	U.S.A	417-881	465
HM216257	A	Argentina	259-766	508	HM173865	C	U.S.A	417-881	465
HM216258	A	Argentina	259-766	508	HM173866	C	U.S.A	417-881	465
HM216259	A	Argentina	259-766	508	HM173867	B	U.S.A	417-881	465
HM216260	A	Argentina	259-766	508	HM173868	B	U.S.A	417-881	465
HM216261	A	Argentina	259-766	508	HM173869	C	U.S.A	417-881	465
HM216262	C	Argentina	259-766	508	HM173870	C	U.S.A	417-881	465
HM216263	D	Argentina	259-766	508	HM173871	C	U.S.A	417-881	465
HM216264	D	Argentina	259-766	508	HM173872	A	U.S.A	417-881	465
HM216265	D	Argentina	259-766	508	HM173873	B	U.S.A	417-881	465
HM216266	D	Argentina	259-766	508	HM173874	B	U.S.A	417-881	465
HM216267	D	Argentina	259-766	508	HM173875	B	U.S.A	417-881	465
HM216268	D	Argentina	259-766	508	HM173876	B	U.S.A	417-881	465
HM216269	D	Argentina	259-766	508	HM173877	B	U.S.A	417-881	465
HM216270	D	Argentina	259-766	508	HM173878	B	U.S.A	417-881	465
HM216271	D	Argentina	259-759	501	HM173879	A	U.S.A	417-881	465
HM216272	A	Argentina	259-766	508	HM173880	B	U.S.A	417-905	489
HM216273	D	Argentina	259-766	508	HM173881	B	U.S.A	417-881	465
HM216274	D	Argentina	259-766	508	HM173882	B	U.S.A	417-881	465
HM216275	D	Argentina	259-764	506	HM173883	B	U.S.A	417-881	465
HM216276	D	Argentina	259-766	508	HM173884	B	U.S.A	417-881	465
HM216277	F	Argentina	259-766	508	HM173885	B	U.S.A	417-881	465
HM216278	F	Argentina	259-766	508	HM173886	C	U.S.A	417-881	465
HM216279	F	Argentina	259-766	508	HM173887	B	U.S.A	417-881	465
HM216280	F	Argentina	259-766	508	HM173888	B	U.S.A	417-881	465
HM216281	F	Argentina	259-766	508	HM173889	B	U.S.A	417-881	465
HM216282	F	Argentina	259-766	508	HM173890	C	U.S.A	417-881	465
HM216283	F	Argentina	259-766	508	HM173891	C	U.S.A	417-881	465
HM216284	F	Argentina	259-766	508	HM173892	C	U.S.A	417-881	465
HM216285	F	Argentina	259-766	508	HM173893	C	U.S.A	417-881	465
JN688680	D	Argentina	217-771	555	HM173894	B	U.S.A	417-881	465
JN688688	D	Argentina	217-771	555	HM173895	B	U.S.A	417-881	465
JN688690	D	Argentina	217-771	555	HM173896	B	U.S.A	417-881	465
JN688696	D	Argentina	217-771	555	HM173897	B	U.S.A	417-881	465
JN688697	D	Argentina	217-771	555	HM173898	C	U.S.A	417-881	465
JN688714	D	Argentina	217-771	555	HM173899	C	U.S.A	417-881	465
KC680772	A	Argentina	244-768	525	HM173900	B	U.S.A	417-881	465
EF034149	A	Brazil	1-3182	1181	HM173901	B	U.S.A	417-881	465
EF034150	A	Brazil	1-3182	1181	HM173902	B	U.S.A	417-881	465
EF034151	A	Brazil	1-3182	1181	HM173903	B	U.S.A	417-881	465
EF464070	F	Brazil	417-841	425	HM173904	B	U.S.A	417-881	465
EF464071	A	Brazil	417-841	425	HM173905	B	U.S.A	417-881	465
EF464072	A	Brazil	417-841	425	HM173906	A	U.S.A	417-881	465

EF464073	A	Brazil	417-841	425	HM173907	C	U.S.A	417-881	465
EF464074	A	Brazil	417-841	425	HM173908	B	U.S.A	417-881	465
EF464075	A	Brazil	417-841	425	HM173909	B	U.S.A	417-881	465
EF464076	A	Brazil	417-841	425	HM173910	B	U.S.A	417-881	465
EF464077	A	Brazil	417-841	425	HM173911	B	U.S.A	417-881	465
EF464078	A	Brazil	417-841	425	HM173912	B	U.S.A	417-881	465
EF464079	A	Brazil	417-841	425	HM173913	B	U.S.A	417-881	465
EF464080	A	Brazil	417-841	425	HM173914	B	U.S.A	417-881	465
EF464081	A	Brazil	417-841	425	HM173915	B	U.S.A	417-881	465
EF464082	A	Brazil	417-841	425	HM173916	B	U.S.A	417-881	465
EF464083	A	Brazil	417-841	425	HM173917	C	U.S.A	417-881	465
EF464084	A	Brazil	417-841	425	HM173918	C	U.S.A	417-881	465
EF464085	A	Brazil	417-841	425	HM173919	B	U.S.A	417-881	465
EF464086	A	Brazil	417-841	425	HM173920	B	U.S.A	417-881	465
EF464087	A	Brazil	417-841	425	HM173921	B	U.S.A	417-881	465
EF464088	A	Brazil	417-841	425	HM173922	B	U.S.A	417-881	465
EF464089	A	Brazil	417-841	425	HM173923	B	U.S.A	417-881	465
EF464090	A	Brazil	417-841	425	HM173924	B	U.S.A	417-881	465
EF464091	A	Brazil	417-841	425	HM173925	B	U.S.A	417-881	465
EF464092	A	Brazil	417-841	425	HM173926	A	U.S.A	417-881	465
EF464093	C	Brazil	417-841	425	HM173927	A	U.S.A	417-881	465
EF464094	D	Brazil	417-841	425	HM173928	B	U.S.A	417-881	465
EF464095	D	Brazil	417-841	425	HM173929	A	U.S.A	417-881	465
EF464096	D	Brazil	417-841	425	HM173930	B	U.S.A	417-881	465
EF514546	A	Brazil	417-841	425	HM173931	C	U.S.A	417-881	465
EF514547	A	Brazil	417-841	425	HM173932	B	U.S.A	417-881	465
EU221426	D	Brazil	519-997	479	HM173933	A	U.S.A	417-881	465
EU221427	D	Brazil	519-997	479	HM173934	B	U.S.A	417-881	465
EU221428	D	Brazil	519-997	479	HM173935	C	U.S.A	417-881	465
EU221429	A	Brazil	519-997	497	HM173936	B	U.S.A	417-881	465
EU221430	A	Brazil	519-997	497	HM173937	B	U.S.A	417-881	465
EU221431	A	Brazil	519-997	497	HM173938	B	U.S.A	417-881	465
EU221432	D	Brazil	519-997	479	HM173939	B	U.S.A	417-881	465
EU221433	D	Brazil	519-997	479	HM173940	B	U.S.A	417-881	465
EU221434	F	Brazil	519-997	479	HM173941	C	U.S.A	417-881	465
EU221435	D	Brazil	519-997	479	HM173942	B	U.S.A	417-881	465
EU221436	D	Brazil	519-997	479	HM173943	B	U.S.A	417-881	465
EU221437	A	Brazil	519-997	497	HM173944	B	U.S.A	417-881	465
EU221438	A	Brazil	519-997	497	HM173945	B	U.S.A	417-881	465
EU221439	D	Brazil	519-997	479	HM173946	B	U.S.A	417-881	465
EU221440	A	Brazil	519-997	497	HM173947	B	U.S.A	417-881	465
EU221441	A	Brazil	519-997	497	HM173948	B	U.S.A	417-866	450
EU221442	D	Brazil	519-997	479	HM173949	C	U.S.A	417-905	489
EU221443	A	Brazil	519-997	497	HM173950	B	U.S.A	417-881	465
EU221444	A	Brazil	519-997	497	HM173951	B	U.S.A	417-881	465
EU221445	A	Brazil	519-997	497	HM173952	B	U.S.A	417-881	465
EU221446	D	Brazil	519-997	479	HM173953	C	U.S.A	417-881	465
EU221447	D	Brazil	519-997	479	HM173954	B	U.S.A	417-881	465
EU221448	D	Brazil	519-997	479	HM173955	C	U.S.A	417-881	465
EU221449	D	Brazil	519-997	479	HM173956	C	U.S.A	417-881	465
EU221450	D	Brazil	519-997	479	HM173957	B	U.S.A	417-881	465
EU221451	D	Brazil	519-997	479	HM173958	C	U.S.A	417-881	465
EU221452	A	Brazil	519-997	497	HM173959	B	U.S.A	417-881	465
EU221453	D	Brazil	519-997	479	HM173960	C	U.S.A	417-881	465
EU221454	A	Brazil	519-997	497	HM173961	A	U.S.A	417-881	465
EU221455	A	Brazil	519-997	497	HM173962	C	U.S.A	417-881	465

EU221456	D	Brazil	519-997	479	HM173963	C	U.S.A	417-881	465
EU221457	D	Brazil	519-997	479	HM173964	D	U.S.A	417-881	465
EU221458	A	Brazil	519-997	497	HM173965	C	U.S.A	417-881	465
EU221459	D	Brazil	519-997	479	HM173966	C	U.S.A	417-881	465
EU221460	D	Brazil	519-997	479	HM173967	B	U.S.A	417-881	465
EU221461	D	Brazil	519-997	479	HM173968	B	U.S.A	417-881	465
EU221462	D	Brazil	519-997	479	HM173969	B	U.S.A	417-866	450
EU221463	A	Brazil	519-997	497	HM173970	C	U.S.A	417-881	465
EU221464	D	Brazil	519-997	479	HM173971	B	U.S.A	417-881	465
EU221465	A	Brazil	519-997	497	HM173972	B	U.S.A	417-881	465
EU221466	D	Brazil	519-997	479	HM173973	B	U.S.A	417-881	465
EU221467	D	Brazil	519-997	479	HM173974	B	U.S.A	417-881	465
EU221468	D	Brazil	519-997	479	HM173975	B	U.S.A	417-881	465
EU221469	D	Brazil	519-997	479	HM173976	B	U.S.A	417-881	465
EU221470	A	Brazil	519-997	497	HM173977	B	U.S.A	417-881	465
EU221471	A	Brazil	519-997	497	HM173978	C	U.S.A	417-881	465
EU221472	D	Brazil	519-997	479	HM173979	B	U.S.A	417-881	465
EU221473	D	Brazil	519-997	479	HM173980	B	U.S.A	417-881	465
EU221474	A	Brazil	519-997	497	HM173981	B	U.S.A	417-881	465
EU221475	A	Brazil	519-997	497	HM173982	C	U.S.A	435-881	447
EU264153	NC	Brazil	157-754	598	HM173983	B	U.S.A	417-881	465
GU248531	A	Brazil	1-3182	852	HM173984	B	U.S.A	417-881	465
GU248532	D	Brazil	1-3182	830	HM173985	B	U.S.A	417-881	465
GU248533	D	Brazil	1-3182	852	HM173986	C	U.S.A	417-866	450
GU248534	A	Brazil	1-3182	852	HM173987	B	U.S.A	417-866	450
GU248535	A	Brazil	1-3182	852	HM173988	B	U.S.A	417-881	465
GU248536	D	Brazil	1-3182	852	HM173989	B	U.S.A	417-881	465
GU248537	A	Brazil	1-3182	852	HM173990	B	U.S.A	417-881	465
GU248538	A	Brazil	1-3182	812	HM173991	B	U.S.A	417-881	465
GU248539	D	Brazil	1-3182	832	HM173992	B	U.S.A	417-881	465
GU248540	A	Brazil	1-3182	839	HM173993	B	U.S.A	417-881	465
GU248541	A	Brazil	1-3182	830	HM173994	B	U.S.A	417-881	465
GU248542	D	Brazil	1-3182	774	HM173995	B	U.S.A	417-881	465
GU248543	A	Brazil	1-3182	852	HM173996	C	U.S.A	417-881	465
GU248544	A	Brazil	1-3182	839	HM173997	B	U.S.A	417-881	465
GU248545	A	Brazil	1-3182	843	HM173998	B	U.S.A	417-881	465
GU248546	A	Brazil	1-3182	852	HM173999	B	U.S.A	417-881	465
GU248547	A	Brazil	1-3182	852	HM174000	B	U.S.A	417-881	465
GU248548	A	Brazil	1-3182	838	HM174001	B	U.S.A	417-881	465
GU248549	A	Brazil	1-3182	813	HM174002	B	U.S.A	417-881	465
GU248550	A	Brazil	46-3182	798	HM174003	C	U.S.A	417-881	465
GU248551	D	Brazil	1-3182	806	HM174004	B	U.S.A	417-881	465
GU248552	A	Brazil	1-3182	842	HM174005	B	U.S.A	417-881	465
GU248553	A	Brazil	1-3182	852	HM174006	B	U.S.A	417-881	465
GU248554	A	Brazil	1-3182	839	HM174007	B	U.S.A	417-881	465
GU248555	A	Brazil	1-3182	852	HM174008	B	U.S.A	417-881	465
GU248556	A	Brazil	1-3182	852	HM174009	B	U.S.A	417-881	465
GU248557	D	Brazil	1-3182	842	HM174010	B	U.S.A	417-881	465
GU248558	A	Brazil	1-3182	839	HM174011	B	U.S.A	417-881	465
GU248559	A	Brazil	1-3182	834	HM174012	B	U.S.A	417-881	465
GU248560	A	Brazil	1-3182	839	HM174013	B	U.S.A	417-881	465
GU248561	A	Brazil	1-3182	852	HM174014	B	U.S.A	417-881	465
GU248562	D	Brazil	1-3182	833	HM174015	B	U.S.A	417-881	465
GU248563	F	Brazil	3-1163	839	HM174016	C	U.S.A	417-881	465
GU248564	A	Brazil	1-3182	839	HM174017	B	U.S.A	417-881	465
GU248565	D	Brazil	1-3095	746	HM174018	B	U.S.A	417-881	465

GU248566	A	Brazil	1-3182	852	HM174019	C	U.S.A	417-881	465
GU248567	NC	Brazil	1-3182	842	HM174020	C	U.S.A	417-881	465
GU248568	A	Brazil	1-3182	840	HM174021	C	U.S.A	417-881	465
GU248569	A	Brazil	1-3182	834	HM174022	B	U.S.A	417-881	465
GU248570	A	Brazil	1-3182	852	HM174023	C	U.S.A	417-881	465
GU248571	D	Brazil	1-3182	842	HM174024	B	U.S.A	417-881	465
GU248572	A	Brazil	1-3182	841	HM174025	B	U.S.A	417-881	465
GU248573	A	Brazil	1-3182	852	HM174026	B	U.S.A	417-881	465
GU248574	A	Brazil	1-3182	852	HM174027	C	U.S.A	417-881	465
GU248575	A	Brazil	1-3182	816	HM174028	B	U.S.A	417-881	465
GU248576	A	Brazil	1-3182	841	HM174029	B	U.S.A	417-866	450
GU248577	A	Brazil	1-3182	852	HM174030	B	U.S.A	417-881	465
GU248578	A	Brazil	1-3182	852	HM174031	B	U.S.A	417-881	465
GU248579	A	Brazil	1-3182	852	HM174032	C	U.S.A	417-881	465
GU248580	D	Brazil	1-3182	844	HM174033	B	U.S.A	417-881	465
GU248581	A	Brazil	1-3182	852	HM174034	B	U.S.A	417-866	450
GU248582	A	Brazil	1-3182	852	HM174035	B	U.S.A	417-881	465
GU248583	D	Brazil	1-3182	852	HM174036	B	U.S.A	417-881	465
GU248584	A	Brazil	1-3182	839	HM174037	B	U.S.A	417-881	465
GU248585	F	Brazil	3-1163	824	HM174038	B	U.S.A	417-881	465
GU248586	A	Brazil	1-3182	852	HM174039	B	U.S.A	417-881	465
GU248587	A	Brazil	43-3182	810	HM174040	B	U.S.A	417-881	465
GU248588	A	Brazil	1-3182	852	HM174041	C	U.S.A	417-881	465
GU248589	D	Brazil	1-3182	852	HM174042	B	U.S.A	417-881	465
GU248590	A	Brazil	1-3182	844	HM174043	C	U.S.A	417-881	465
GU248591	A	Brazil	1-3182	852	HM174044	C	U.S.A	417-881	465
GU248592	A	Brazil	1-3182	852	HM174045	B	U.S.A	417-881	465
GU248593	A	Brazil	1-3182	852	HM174046	B	U.S.A	417-881	465
GU248594	A	Brazil	1-3182	839	HM174047	A	U.S.A	417-881	465
GU248595	A	Brazil	1-3182	852	HM174048	B	U.S.A	417-881	465
GU248596	A	Brazil	1-3182	852	HM174049	C	U.S.A	417-881	465
GU248597	A	Brazil	1-3182	852	HM174050	D	U.S.A	417-881	465
GU248598	A	Brazil	1-3182	852	HM174051	B	U.S.A	417-881	465
GU248599	A	Brazil	1-3182	852	HM174052	C	U.S.A	417-881	465
GU248600	D	Brazil	1-3182	852	HM174053	B	U.S.A	417-881	465
GU248601	A	Brazil	1-3182	852	HM174054	B	U.S.A	417-881	465
GU248602	A	Brazil	1-3182	852	HM174055	B	U.S.A	417-881	465
GU248603	D	Brazil	1-3182	852	HM174056	B	U.S.A	417-881	465
GU248604	A	Brazil	1-3182	852	HM174057	B	U.S.A	417-881	465
GU248605	A	Brazil	1-3182	852	HM174058	B	U.S.A	417-881	465
GU248606	A	Brazil	1-3182	852	HM174059	C	U.S.A	417-881	465
GU248607	A	Brazil	1-3182	843	HM174060	B	U.S.A	417-881	465
GU248608	A	Brazil	1-3182	852	HM174061	B	U.S.A	417-881	465
GU248609	D	Brazil	1-3182	852	HM174062	B	U.S.A	417-881	465
GU248610	A	Brazil	1-3182	842	HM174063	B	U.S.A	417-881	465
GU248611	A	Brazil	1-3182	852	HM174064	B	U.S.A	417-881	465
GU248612	A	Brazil	1-3182	842	HM174065	B	U.S.A	417-881	465
GU248613	A	Brazil	1-3182	852	HM174066	B	U.S.A	417-881	465
GU248614	A	Brazil	1-3182	852	HM174067	C	U.S.A	417-881	465
GU248615	D	Brazil	1-3182	852	HM174068	B	U.S.A	417-881	465
GU248616	A	Brazil	1-3182	795	HM174069	C	U.S.A	417-881	465
GU248617	A	Brazil	1-3182	837	HM174070	B	U.S.A	417-881	465
GU248618	A	Brazil	1-3182	852	HM174071	B	U.S.A	417-881	465
GU248619	A	Brazil	1-3182	852	HM174072	B	U.S.A	417-881	465
GU248620	A	Brazil	1-3182	852	HM174073	B	U.S.A	417-881	465
GU248621	A	Brazil	1-3182	852	HM174074	B	U.S.A	417-881	465

GU248622	D	Brazil	1-3182	852	HM174075	B	U.S.A	417-881	465
GU248623	A	Brazil	1-3182	852	HM174076	B	U.S.A	426-881	456
GU248624	A	Brazil	1-3182	852	HM174077	B	U.S.A	417-881	465
GU248625	D	Brazil	1-3182	842	HM174078	B	U.S.A	417-881	465
GU248626	A	Brazil	1-3182	812	HM174079	B	U.S.A	417-881	465
GU248627	A	Brazil	1-3182	844	HM174080	B	U.S.A	417-881	465
GU248628	A	Brazil	1-3182	843	HM174081	C	U.S.A	417-881	465
GU248629	A	Brazil	1-3182	797	HM174082	B	U.S.A	417-881	465
GU248630	A	Brazil	1-3182	832	HM174083	B	U.S.A	417-881	465
GU248631	A	Brazil	1-3182	791	HM174084	B	U.S.A	417-881	465
GU248632	A	Brazil	1-3182	821	HM174085	B	U.S.A	417-881	465
GU248633	A	Brazil	1-3182	841	HM174086	B	U.S.A	417-881	465
GU248634	A	Brazil	1-3182	828	HM174087	C	U.S.A	417-881	465
GU248635	A	Brazil	1-3182	809	HM174088	B	U.S.A	417-881	465
KU847686	F	Brazil	132-1163	1032	HM174089	B	U.S.A	417-881	465
MG839201	A	Brazil	157-837	681	HM174090	B	U.S.A	417-881	465
MG839202	A	Brazil	157-837	681	HM174091	B	U.S.A	417-881	465
MG839203	A	Brazil	157-837	681	HM174092	C	U.S.A	417-881	465
MG839204	A	Brazil	157-837	681	HM174093	C	U.S.A	417-881	465
MG839205	A	Brazil	157-837	681	HM174094	B	U.S.A	417-881	465
MG839206	A	Brazil	157-837	681	HM174095	B	U.S.A	417-881	465
MG839207	A	Brazil	157-837	681	HM174096	A	U.S.A	417-881	465
MG839208	A	Brazil	157-837	681	HM174097	B	U.S.A	417-881	465
MG839209	A	Brazil	157-837	681	HM174098	B	U.S.A	417-881	465
MG839210	D	Brazil	157-837	681	HM174099	B	U.S.A	417-881	465
MG839211	D	Brazil	157-837	681	KF771917	G	U.S.A	157-837	681
MG839212	D	Brazil	157-837	681	KF771918	A	U.S.A	157-837	681
MG839213	F	Brazil	157-842	686	KF771919	A	U.S.A	157-837	681
MH268244	A	Brazil	1-3182	1052	KF771920	A	U.S.A	157-837	681
MK173814	A	Brazil	19-3180	740	KF771921	E	U.S.A	157-837	681
MK854819	A	Brazil	320-951	632	KF771922	A	U.S.A	157-837	681
MK854820	F	Brazil	320-951	632	KF771923	D	U.S.A	157-837	681
MK854821	A	Brazil	320-951	632	KF771924	B	U.S.A	157-837	681
MK854822	A	Brazil	320-951	632	KF771925	C	U.S.A	157-837	681
MK854823	A	Brazil	320-950	631	KF771926	E	U.S.A	157-837	681
MK854824	A	Brazil	320-951	632	KF771927	E	U.S.A	157-837	681
MK854825	F	Brazil	320-951	632	KF771928	B	U.S.A	157-837	681
MN224162	G	Brazil	484-929	446	KF771929	D	U.S.A	157-837	681
MN224163	A	Brazil	410-938	529	KF771930	E	U.S.A	157-837	681
MN224164	A	Brazil	410-938	528	KF771931	B	U.S.A	157-837	681
MN224165	D	Brazil	458-923	466	KF771932	B	U.S.A	157-837	681
MN224166	D	Brazil	458-923	466	KF771933	E	U.S.A	157-837	681
MN224167	D	Brazil	458-923	466	KF771934	C	U.S.A	157-837	681
MN224168	D	Brazil	458-923	466	KF771935	C	U.S.A	157-837	681
MN224169	D	Brazil	458-923	466	KF771936	A	U.S.A	157-837	681
MN224170	D	Brazil	458-923	466	KF771937	B	U.S.A	157-837	681
MN224171	D	Brazil	458-923	466	KF771938	D	U.S.A	157-837	681
MN224172	D	Brazil	458-923	466	KF771939	E	U.S.A	157-837	681
MN224173	D	Brazil	458-923	466	KF771940	A	U.S.A	157-837	681
MN224174	D	Brazil	458-923	466	KF771941	B	U.S.A	157-837	681
MN224175	D	Brazil	458-923	466	KF771942	B	U.S.A	157-837	681
MN224176	D	Brazil	458-923	466	KF771943	A	U.S.A	157-837	681
MN224177	D	Brazil	458-923	466	KF771944	E	U.S.A	157-837	681
MN224178	D	Brazil	458-923	466	KF771945	D	U.S.A	157-837	681
MN224179	D	Brazil	458-923	466	KF771946	A	U.S.A	157-837	681
MN224180	D	Brazil	458-923	466	KF771947	C	U.S.A	157-837	681

MN224181	D	Brazil	458-923	466	KF771948	D	U.S.A	157-837	681
MN224182	D	Brazil	458-923	466	KF771949	C	U.S.A	157-837	681
MN224183	D	Brazil	458-923	466	KF771950	B	U.S.A	157-837	681
MN224184	D	Brazil	458-923	466	KF771951	D	U.S.A	157-837	681
MN224185	D	Brazil	458-923	466	KF771952	A	U.S.A	157-837	681
MN224186	D	Brazil	458-923	466	KF771953	A	U.S.A	157-837	681
MN224187	D	Brazil	458-923	466	KF771954	B	U.S.A	157-837	681
MN224188	D	Brazil	458-923	466	KF771955	A	U.S.A	157-837	681
MN224189	D	Brazil	458-923	466	KF771956	A	U.S.A	157-837	681
MN224190	D	Brazil	458-923	466	KF771957	A	U.S.A	157-837	681
MN224191	D	Brazil	458-923	466	KF771958	A	U.S.A	157-837	681
MN224192	D	Brazil	458-923	466	KF771959	B	U.S.A	157-837	681
MN224193	D	Brazil	458-923	466	KF771960	A	U.S.A	157-837	681
MN224194	D	Brazil	458-923	466	KF771961	D	U.S.A	157-837	681
MN224195	D	Brazil	410-943	534	KF771962	A	U.S.A	157-837	681
MN224196	D	Brazil	410-943	534	KF771963	A	U.S.A	157-837	681
MN224197	D	Brazil	410-943	534	KF771964	D	U.S.A	157-837	681
MN224198	D	Brazil	410-943	534	KF771965	A	U.S.A	157-837	681
MN224199	D	Brazil	410-943	534	KF771966	B	U.S.A	157-837	681
MN224200	D	Brazil	410-943	534	KF771967	A	U.S.A	157-837	681
MN224201	D	Brazil	410-943	534	KF771968	A	U.S.A	157-837	681
MN224202	D	Brazil	425-933	509	KF771969	A	U.S.A	157-837	681
MN224203	D	Brazil	425-933	509	KF771970	A	U.S.A	1-3182	1203
MN224204	D	Brazil	410-943	534	KF771971	A	U.S.A	1-3182	1020
MN224205	D	Brazil	458-923	466	KF771973	B	U.S.A	1-3182	1203
MN224206	D	Brazil	458-923	466	KF771986	A	U.S.A	1-3182	1200
MN224207	D	Brazil	458-923	466	KF771987	A	U.S.A	1-3182	1200
MN224208	D	Brazil	458-923	466	MF155964	F	U.S.A	3-1163	900
MN224209	D	Brazil	458-923	466	MK173838	A	U.S.A	216-955	740
MN224210	D	Brazil	458-923	466	AB037944	F	Venezuela	157-837	681
MN224211	D	Brazil	458-923	466	AB037945	F	Venezuela	157-837	681
MN224212	D	Brazil	458-923	466	AB037946	F	Venezuela	157-837	681
