# Supplementary figures and images for: The Molecular Epidemiology of Hepatitis B Virus and Its Resistance-Associated Mutations in the Polymerase Gene in the Americas
Source: Microorganisms. 2025 Aug 16;13(8):1913. doi: 10.3390/microorganisms13081913 (PMC12388563; doi:10.3390/microorganisms13081913)

# Dendrogram using Ward Linkage

Rescaled Distance Cluster Combine

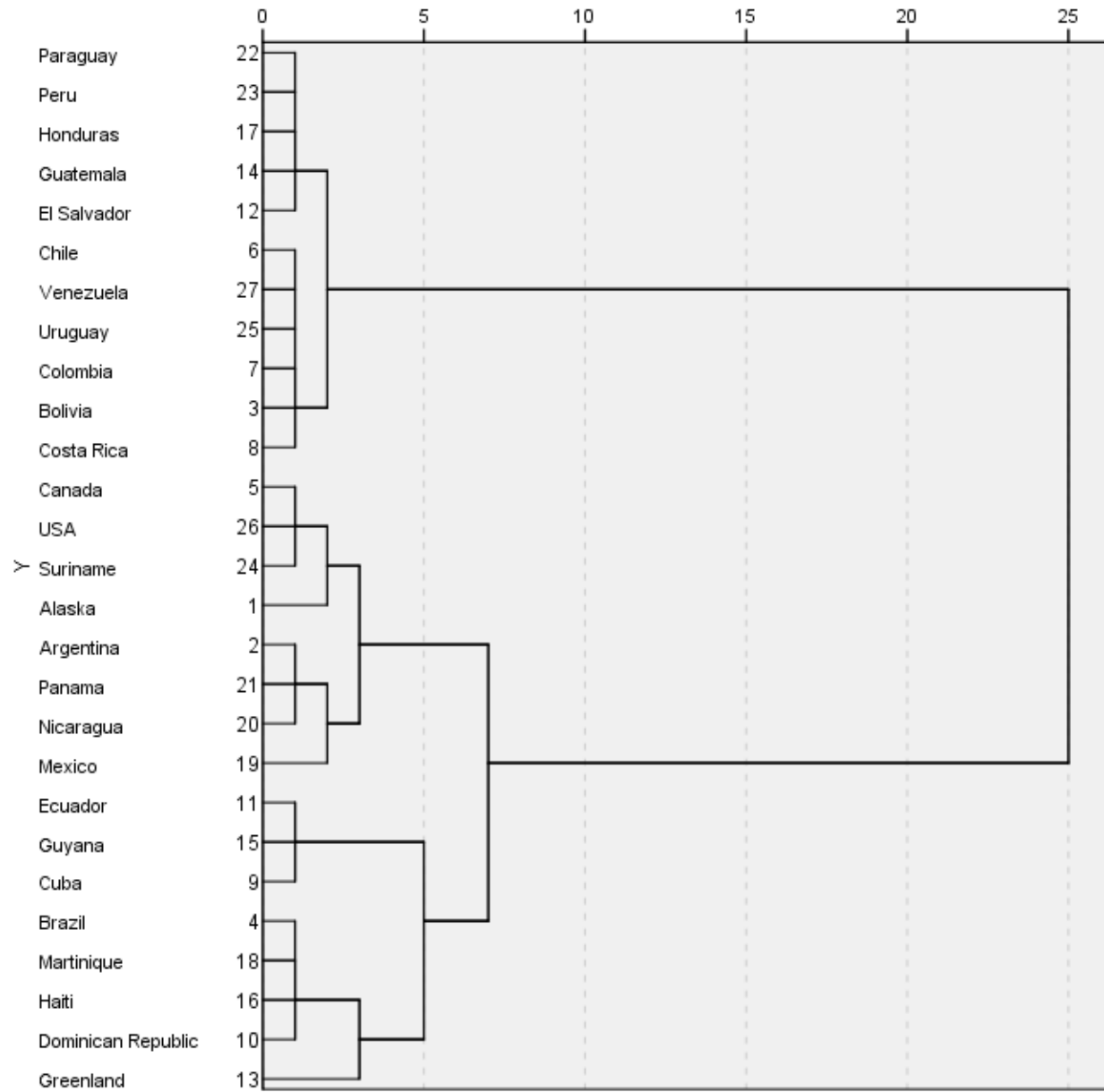

Supplement: Supplementary file 1 [file microorganisms-13-01913-s001.zip › Figure S4 - Dendogram.pdf]
